# Mechanical assessment of proprietary and improvised pelvic binders for use in the prehospital environment

**DOI:** 10.1136/military-2023-002398

**Published:** 2023-08-04

**Authors:** Thomas John Howe, H Claireaux, H Fox, G Morgan, L McMenemy, S D Masouros, A Ramasamy

**Affiliations:** 1 Department of Bioengineering, Imperial College London, London, UK; 2 Army Medical Service 16 Medical Regiment, Colchester, UK; 3 Army Medical Service, Camberley, Surrey, UK; 4 Academic Department of Military Surgery and Trauma, Royal Centre for Defence Medicine, Birmingham, UK

**Keywords:** trauma management, hip, orthopaedic & trauma surgery, trauma management, accident & emergency medicine

## Abstract

**Introduction:**

Pelvic fractures often result from high-energy trauma and are associated with a 10% mortality rate and significant morbidity. Pelvic binders are applied in suspected pelvic injury to stabilise fractured bone, decrease bleeding and potentiate tamponade. A binder must hold the pelvis with sufficient force for this effect to be achieved. This study aims to quantify the ability of proprietary and improvised pelvic binders to hold a target tensile force over time.

**Methods:**

The ability of three proprietary and three improvised binders to hold a binding force for 2 hours was tested. A uniaxial materials testing machine was used to tension each binder to 150 N and then hold the displacement for 2 hours; the drop in tension over time was recorded for each binder. The ability to hold tension above 130 N after 2 hours was set as the metric of binder performance.

**Results:**

The median tension at 2 hours was above 130 N for the SAM Pelvic Sling II and T-POD Pelvic Stabilisation Device and was below 130 N for the Prometheus Pelvic Splint, field-expedient pelvic splint (FES) and the Personal Clothing System-Multi-Terrain Pattern Combat Trousers binders. The tension in the improvised FES after 2 hours was approximately at the target 130 N; however, in 40% of the tests, it held above 130 N.

**Conclusions:**

Binders varied in their ability to maintain sufficient tension to treat a pelvic fracture over the 2-hour testing period. The FES performed well under our testing regime; with relatively low cost and weight, it represents a good alternative to proprietary binders for the austere environment.

**WHAT IS ALREADY KNOWN ON THIS TOPIC**

- Pelvic binding devices are effective in the early stabilisation of unstable pelvic fractures.
- Many improvised binders have been described; however, how they compare to their proprietary counterparts is unknown.

**WHAT THIS STUDY ADDS**

- This is the first study that quantifies the ability of different pelvic binders in maintaining a target tensile force for a set duration of time.

**HOW THIS STUDY MIGHT AFFECT RESEARCH, PRACTICE OR POLICY**

- The low cost, low weight and multipurpose use of the field-expedient pelvic splint represent a good alternative to proprietary binders when treating patients with pelvic injury in the prehospital environment.

##  Background

Pelvic injury commonly occurs after high-energy trauma from a road traffic collision, crush injury or fall from height, and is associated with an almost 10% mortality rate.^1^ In recent conflicts, lower limb blast casualties with associated pelvic fracture had a mortality of 60.8%, likely attributable to non-compressible haemorrhage.^2 3^

Bleeding in pelvic fracture can occur from injury to the arteries, veins and bone. The venous plexus around the sacroiliac joints is the most common source of significant bleeding.^4^ The volume of the pelvis is potentially increased after pelvic fracture. This may lead to significant bleeding as without bony containment the retroperitoneal space may expand, and so tamponade is unlikely to occur.^5^ Initial treatment of pelvic fracture aims to limit bleeding by restoring normal anatomy, compressing bleeding ends of bone and stabilising blood clots.^6^

Pelvic binding devices are effective in the early stabilisation of unstable pelvic fractures.^7^ Advanced Trauma Life Support and UK NICE guidelines state that these should be used.^8 9^ To achieve their effect in practice, pelvic binding devices need to be strong in construction, easy to apply and capable of tightening to adequate tension.^7^ The results of a prospective study of 13 patients suggested that a binder tensioned to 180±50 N was able to achieve significant reduction of externally rotated pelvic fractures in the emergency setting.^10^,^12^ North Atlantic Treaty Organization (NATO) and NATO military doctrine state that a patient should undergo damage-control surgery no later than 2 hours from the point of wounding.^13^

In scenarios where proprietary binders are not available, an improvised pelvic binder can be fabricated from items such as bedsheets, items of clothing such as trousers and belts or other items available to first responders.^14 15^ It is not known how improvised binders compare to their proprietary counterparts. This is the first study aimed to quantify the ability of proprietary and improvised pelvic binders to hold a target tensile force over time.

## Methods

### Mechanical testing

**Figure 1:**
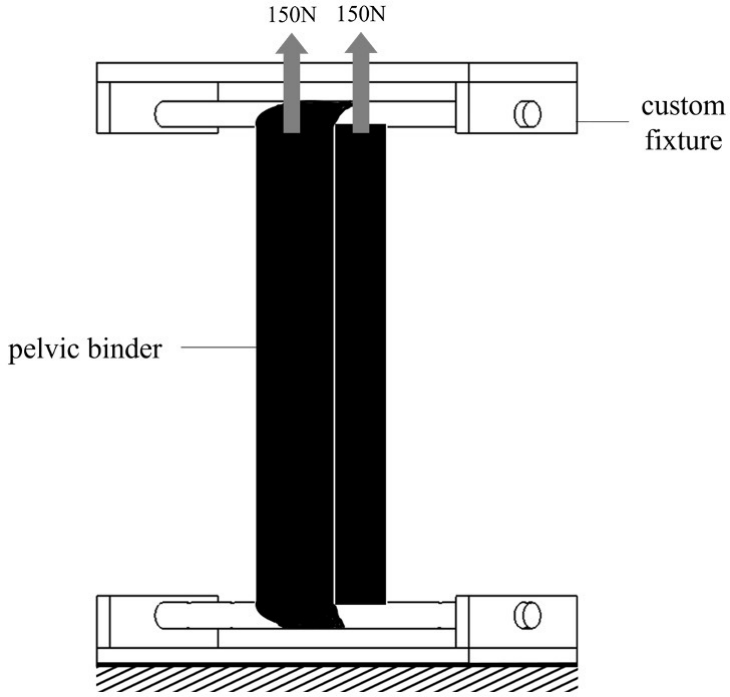
Diagram of the experimental set-up for testing the pelvic binders in tension. The binder is held at the bottom and a force of 300 N is applied at the top, creating a tension of 150 N through each strap of the binder.

Proprietary and improvised pelvic binders were investigated to determine their ability to hold a binding force for a period of 2 hours. Three proprietary binders were used: the SAM Pelvic Sling II (SAM; SAM Medical, Tualatin, Oregon, USA), the Prometheus Pelvic Splint (PROM; Prometheus Medical, Herefordshire, UK) and the T-POD Pelvic Stabilisation Device (TPOD; Teleflex, Wayne, Pennsylvania, USA). Improvised binders included the British Forces Personal Clothing System, Multi-Terrain Pattern (MTP) trousers and a ‘field-expedient pelvic splint’ (FES) constructed from the Combat Application Tourniquet (C-A-T Resources, LLC, Rock Hill, South Carolina, USA) and the SAM splint (SAM Medical).^14^ Relaxation tests were conducted using a uniaxial materials testing machine (5866; Instron, Canton, Massachusetts, USA) with a custom-designed fixture to hold the pelvic binder in uniaxial tension at a controlled displacement (Figure 1).

**Figure 2:**
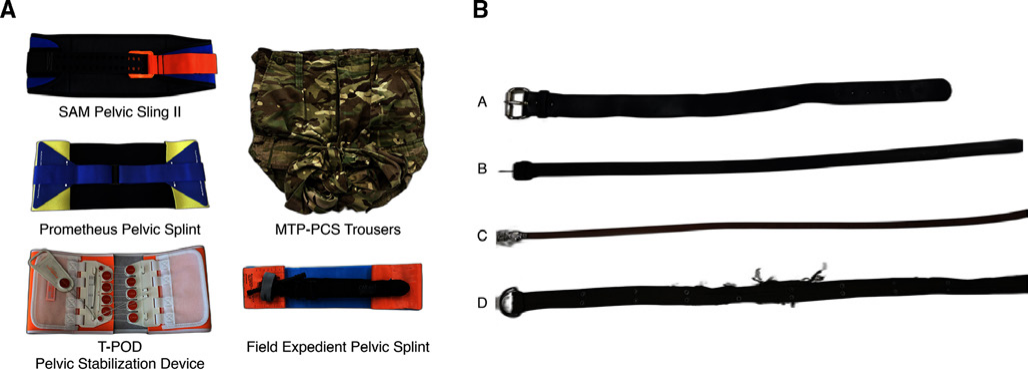
(A) Photographs of assembled proprietary and improvised pelvic binders used in this study. (B) The belts used as possible improvised pelvic binders in a civilian setting. Three used leather belts of different widths are shown: belt A, wide; belt B, medium; belt C, narrow; as well as belt D, used D-ring-type belt (figure created with photographs taken and owned by the authors). MTP, Multi-Terrain Pattern; PCS, Personal Clothing System.

Each proprietary binder was applied to the fixture according to the manufacturers’ instructions for use (Figure 2A) and subsequently tensioned to 150 N at a rate of 1 mm/s using the testing machine. The binder was then held at a constant displacement for 2 hours, with the force measurements recorded at a frequency of 1 Hz. Each binder was tested six times, with a minimum of 2 hours between subsequent tests. A strap tension of 150 N was chosen as the SAM AUTOSTOP buckle activates at this level to confirm correct application, and 150 N of strap tension falls within the range of effective binding force identified by cadaveric studies.^10^,^12^

Two improvised binders were assembled and then tested using the aforementioned protocol. The FES was assembled using the tourniquet as described by Savakus *et al*^14^ and the MTP trousers using the method described by Loftus *et al*,^15^ whereby trouser legs are cut anterolaterally and secured centrally with a knot before being tensioned (Figure 2A).

Four secondhand trouser belts were additionally tested to investigate their potential as improvised pelvic binders for use in armed conflict in a civilian setting. Three used leather belts of varying widths (belt A, belt B and belt C, corresponding to wide, medium and narrow, respectively) and a used D-ring-type belt (belt D) were sourced (Figure 2B) and tested using the aforementioned protocol. A total of eight tests were performed: belts A–C were each tested twice (n=2), and belt D was tested once with a standard D-ring fastening method, ‘knotless’ (n=1), and once with a knot additionally used to secure the free-end after fastening, ‘knotted’ (n=1).

### Statistical analysis

Binder tension at 2 hours was noted for each binder. Outliers were identified using Tukey’s method and were excluded from subsequent analysis. The data set of each binder was tested using a one-sample, one-tailed Student’s t-test against a null hypothesis value of 130 N, with a Bonferroni correction factor of 5.

### Finite element (FE) analysis

A static FE model was developed in MSC.Marc V.2021 (MSC.Software) to simulate the initial tension to 150 N of the experiment to investigate the influence of the geometry of the custom-designed fixture on the force-displacement results. The fixture was modelled as a rigid cylinder with a diameter of 20 mm, representing the fixture shown in Figure 1, or 200 mm, representing a hemipelvis fixture. The PROM was modelled with an idealised geometry of a rectangular cuboid measuring 220.0×175.0×4.5 mm, representing one-fourth of the binder. A mesh of quadratic tetrahedral elements with an average element size of 2.25 mm was used. The material was modelled as a five-term Mooney-Rivlin elastomer with material constants C_10_, C_01_, C_11_, C_20_ and C_30_ as 0.1037, 0, 0, 1.070 and 0.0864 MPa, respectively, which were obtained from a force–ramp experiment using the set-up shown in Figure 1. The contact between fixture and splint was modelled as frictionless. A 150 N force was applied to the fixture and the binder was held vertically at the centre of the distal end, allowing for sliding and rotation, and symmetry conditions were applied in the other two planes. The displacements at 150 N of the 20 mm and 200 mm diameter fixtures differed by less than 3% (33.05 and 32.18 mm, respectively), indicating that fixture diameter does not significantly affect test results.

## Results

**Figure 3:**
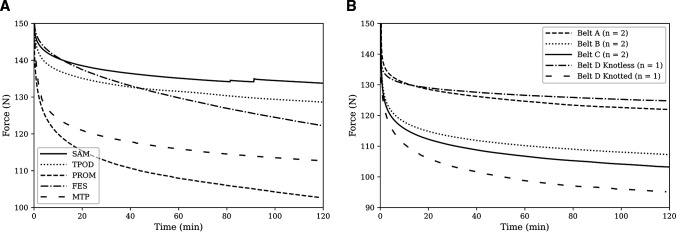
(A) Average force relaxation curves for SAM (by SAM Medical), TPOD (by Teleflex), PROM (by Prometheus Medical), FES and PCS-MTP Combat Trousers. The binders were tensioned to 150 N (where time=0) and kept at a constant displacement for 2 hours. (B) Average force–time curves of belts applied as improvised pelvic binders. Three widths of leather belts were each tested twice: belt A, wide; belt B, medium; belt C, narrow; A D-ring type, belt D, was tested once by securing it with the standard fastening method (belt D, knotless) and once with a knot securing the free end (belt D, knotted). FES, field-expedient pelvic splint; MTP, Multi-Terrain Pattern; PCS, Personal Clothing System; PROM, Prometheus Pelvic Splint; SAM, SAM Pelvic Sling II; TPOD, T-POD Pelvic Stabilisation Device.

The average force in each binder over the 2-hour test duration is shown in Figure 3A. Spikes in binder tension in the SAM occurred when the AUTOSTOP buckle reactivated after disengaging. The force–relaxation curves of the trouser-belt tests are shown in Figure 3B.

**Figure 4:**
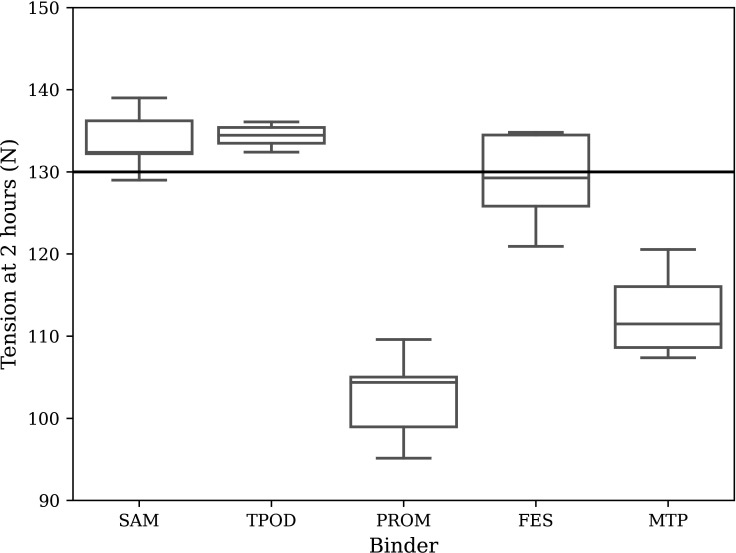
Box plots of binder tension at 2 hours: SAM (by SAM Medical), TPOD (by Teleflex), PROM (by Prometheus Medical), FES and PCS-MTP Combat Trousers. The threshold tension of 130 N against which the binders were compared is displayed as a thick horizontal line. FES, field-expedient pelvic splint; MTP, Multi-Terrain Pattern; PCS, Personal Clothing System; PROM, Prometheus Pelvic Splint; SAM, SAM Pelvic Sling II; TPOD, T-POD Pelvic Stabilisation Device.

Tension at 2 hours for each binder is shown in Figure 4, excluding outliers. The median tension at 2 hours was above 130 N for the SAM and TPOD and was below 130 N for the PROM, field-expedient pelvic splint (FES) and the PCS-MTP Combat Trousers binders. The proportion of tests which held above 130 N for each binder was 80%, 100%, 0%, 40% and 0% for the SAM, TPOD, PROM, FES and MTP binders, respectively.

Statistics on the sets of tension at 2 hours for each binder are given in Table 1, including sample size, mean, SD and p value of a one-sample, one-tailed Student’s t-test against a value of 130 N, including a Bonferroni correction.

**Table 1:** Results of a one-sample, one-tailed Student’s t-test on binder force at 2 hours against a value of 130 N (test for >130 N if mean is >130 N and vice versa)

Binder	Tests (n)	Mean force at 2 hours (N)	1 SD of the force at 2 hours (N)	P value (including Bonferroni)
SAM	5	133.8	3.9	0.2425
TPOD	5	134.4	1.5	0.0066
PROM	6	102.7	5.4	0.0002
FES	5	129.1	5.9	1.8507
MTP	6	112.7	5.2	0.0044

Six tests were performed on each binder, and the number of tests after excluding outliers is shown.

TPOD, T-POD Pelvic Stabilization Device by Teleflex; PROM, PrometheusPelvic Splint by Prometheus Medical.

FES, field-expedient pelvic splint; MTP, Multi-Terrain Pattern; PCS, Personal Clothing System; SAM, SAM Pelvic Sling II.

## Discussion

The TPOD was the only binder to hold a tension statistically significantly greater than the 130 N target for the duration of the 2-hour test. The SAM binder held tension of above 130 N for 2 hours 80% of the time, although this was not statistically significant. Both the PROM and improvised MTP binders held tensions significantly less than 130 N after 2 hours, with no tests on either binder holding above the target tension. The tension in the improvised FES binder after 2 hours was approximately at the target 130 N and 40% of the tests held above 130 N.

These results indicate that the PROM and the improvised MTP binders are highly unlikely to provide the intended tension to keep the unstable pelvic fracture reduced for a 2-hour period. In fact, this holds true also for a 30 min period (p<0.002 for mean less than target 130 N at 30 min). ‘Retightening’ of these binders at set intervals during evacuation could potentially help in achieving the tension required, while acknowledging that any attempt to retighten entails a risk of destabilising the formed blood clots. Further investigation is necessary to determine an appropriate retightening interval.

**Figure 5:**
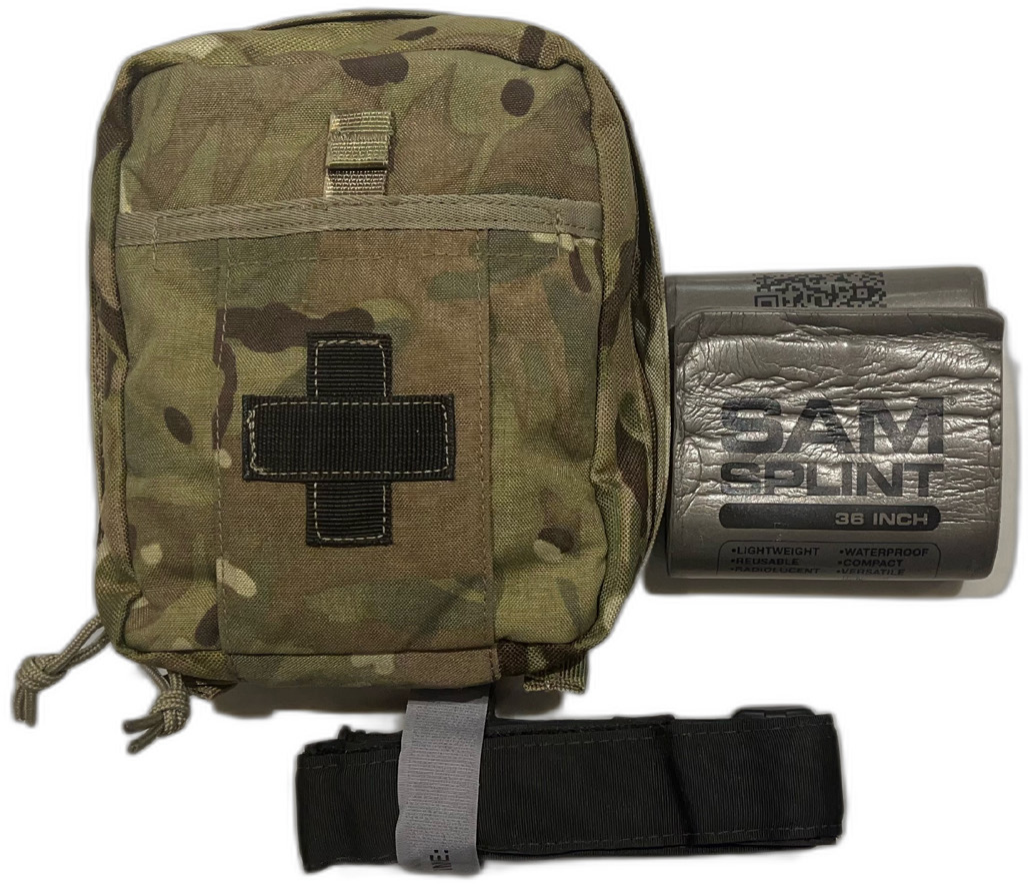
Photograph of a small military medical kit, highlighting the size and portability of the constituent parts of the field-expedient pelvic splint (figure created with a photograph taken and owned by the authors).

The FES binder yielded promising results, demonstrating an ability to maintain 130 N of tension for 2 hours 40% of the time and for 1 hour 60% of the time. The FES has several potential advantages over proprietary binders; the constituent parts are small and easily carried (Figure 5), and the SAM splint used in its construction is low in cost. If field testing of the FES shows positive results, then recommendations could be made for this to be used by military medical personnel. With appropriate training, this equipment could be pushed further forward to non-medical personnel, allowing rapid control of pelvic fractures soon after injury.

No used trouser belts were able to hold above the 130 N threshold for the 2-hour test period, but belt A held at approximately 122 N. The D-ring belt, belt D, with a knotless method, held a force of 124.8 N after the 2-hour test period. This result is highly dependent on the rivets in the belt; as the belt was tensioned, the belt slid with little resistance until a metal rivet was reached and held securely thereafter. Similarly, there is a limited flexibility in circumferences for leather belts due to the fastening holes. Trouser belts could be effective as improvised pelvic binders for implementation in austere environments or in conflicts such as the one currently in Ukraine, where civilians can be exposed to and are being trained to treat blast injuries, including pelvic fracture. While these results demonstrate that belts can be effective in holding a force, methods to tension the belt to the required force in a clinical application must be investigated.

Two hours was selected as the target time to hold adequate tension of 130 N as this fits with NATO medical planning timelines, which dictate that a patient with a pelvic fracture should arrive at damage-control surgery within 2 hours.^13^ Future military operations in austere environments may not match evacuation speeds achieved by coalition forces during recent conflicts in the Middle East. We have provided the raw experimental data as a supplementary file to enable further analyses against different target tensions and times.

A limitation is the simplified loading scenario adopted in our experimental protocol. In clinical use, pelvic binders are unlikely to be held at a constant displacement. This is especially true in a prehospital or austere environment involving patient transport on rugged terrain and on to vehicles and aircraft. There are no available data, however, to define the complex loading associated with transporting a patient. Additionally, the 130 N target tension used in the present study is not validated as the best stabilisation target for a pelvic fracture. It is possible that an optimal treatment targets a threshold displacement or strain, rather than a tensional force, in a scenario more representative of creep rather than stress relaxation.

## Conclusions

Our mechanical testing of proprietary binders has demonstrated that there is variability across models on their ability to maintain tension above a said value for a set duration of time. The FES performed well under our testing regime; with its relatively low cost, low weight and multipurpose use, it represents a good alternative to proprietary binders for treating patients with pelvic injury in the military, austere and prehospital environments.

Future work should involve field testing the pelvic binders used in the present study. Such field tests could potentially monitor the mechanical environment experienced by the binders and the pelvis through simulated treatment and evacuation; the time required to apply each binder could also be recorded.

## Supplementary material

10.1136/military-2023-002398 online supplemental file 1

## Data Availability

All data relevant to the study are included in the article or uploaded as supplementary information.
