# Supplementary material for: Mechanical assessment of proprietary and improvised pelvic binders for use in the prehospital environment
Source: BMJ Mil Health. 2023 Aug 4;171(2):e002398. doi: 10.1136/military-2023-002398 (PMC12015040; doi:10.1136/military-2023-002398)
Supplement: online supplemental file 1 [file military-171-2-s001.pdf]

SAM SLING	1		2		3		4		5		6	
	Time /mins	Strap Tensi	Time /mins	Strap Tensi	Time /mins	Strap Tensi	Time /mins	Strap Tensi	Time /mins	Strap Tensi	Time /mins	Strap Tension /N
	0	150.0335	0	150.0044	0	150.0038	0	150.0052	0	150.0852	0	150.0026
	0.038	149.9849	0.061017	149.6828	0.159733	149.503	0.06485	149.6818	0.040617	150.0771	0.2578	149.5023
	0.054667	149.9439	0.077683	149.4456	0.1764	149.0149	0.081517	149.4632	0.057283	150.0744	0.269683	149.0021
	0.071333	149.9123	0.09435	149.2575	0.193067	148.5309	0.098183	149.2946	0.07395	150.0741	0.28635	148.5543
	0.088	149.8822	0.111017	149.0919	0.209733	148.3141	0.11485	149.1586	0.090617	150.0748	0.303017	148.2258
	0.104667	149.8463	0.127683	148.9416	0.2264	148.1245	0.131517	149.0389	0.107283	150.0753	0.319683	147.9599
	0.121333	149.817	0.14435	148.8053	0.237983	147.6226	0.148183	148.9309	0.12395	150.0781	0.33635	147.7353
	0.138	149.7903	0.161017	148.6738	0.240117	147.1089	0.16485	148.8291	0.140617	150.0773	0.353017	147.5378
	0.154667	149.7615	0.177683	148.5505	0.240733	146.6061	0.181517	148.7346	0.157283	150.077	0.369683	147.3608
	0.171333	149.7348	0.19435	148.4414	0.2574	146.3179	0.198183	148.6488	0.17395	150.0785	0.38635	147.2021
	0.188	149.7044	0.211017	148.3273	0.274067	146.089	0.21485	148.5664	0.190617	150.0802	0.403017	146.9072
	0.204667	149.6781	0.227683	148.2181	0.290733	145.9427	0.231517	148.4858	0.207283	150.079	0.419683	146.7646
	0.221333	149.6533	0.24435	148.1083	0.3074	145.8286	0.248183	148.4137	0.22395	150.0783	0.43635	146.6372
	0.238	149.6263	0.261017	148.0057	0.324067	145.7373	0.26485	148.3479	0.240617	150.0806	0.453017	146.5241
	0.254667	149.6016	0.277683	147.9112	0.340733	145.6508	0.281517	148.2772	0.257283	150.0806	0.469683	146.4134
	0.271333	149.5765	0.29435	147.8159	0.3574	145.5752	0.298183	148.2135	0.27395	150.0822	0.48635	146.3108
	0.288	149.5533	0.311017	147.7249	0.374067	145.5014	0.31485	148.1553	0.290617	150.0793	0.503017	146.2174
	0.304667	149.5267	0.327683	147.636	0.390733	145.4328	0.331517	148.0964	0.307283	150.0831	0.519683	146.1238
	0.321333	149.5032	0.34435	147.5484	0.4074	145.3662	0.348183	148.0388	0.32395	150.0832	0.53635	146.0332
	0.338	149.4783	0.361017	147.4588	0.424067	145.3055	0.36485	147.9834	0.340617	150.0843	0.553017	145.9507
	0.354667	149.4547	0.377683	147.3773	0.440733	145.2449	0.381517	147.934	0.357283	150.0842	0.569683	145.8701
	0.371333	149.4275	0.39435	147.2955	0.4574	145.1857	0.398183	147.8824	0.37395	150.0847	0.58635	145.7904
	0.388	149.4073	0.411017	147.2104	0.474067	145.1285	0.41485	147.8348	0.390617	150.0868	0.603017	145.7131
	0.404667	149.3873	0.427683	147.0209	0.490733	145.0757	0.431517	147.7879	0.407283	150.0846	0.619683	145.6425
	0.421333	149.3645	0.44435	146.9406	0.5074	145.0228	0.448183	147.738	0.42395	150.0852	0.63635	145.568
	0.438	149.339	0.461017	146.862	0.524067	144.9745	0.46485	147.696	0.440617	150.0873	0.653017	145.5009
	0.454667	149.3227	0.477683	146.7872	0.540733	144.9279	0.481517	147.6516	0.457283	150.0872	0.669683	145.4326
	0.471333	149.2985	0.49435	146.7017	0.5574	144.8813	0.498183	147.6104	0.47395	150.0852	0.68635	145.3693
	0.488	149.2742	0.511017	146.6302	0.574067	144.8347	0.51485	147.569	0.490617	150.0857	0.703017	145.305
	0.504667	149.2529	0.527683	146.5588	0.590733	144.7899	0.531517	147.5243	0.507283	150.084	0.719683	145.2463
	0.521333	149.2321	0.54435	146.4947	0.6074	144.745	0.548183	147.4877	0.52395	150.0846	0.73635	145.1842
	0.538	149.2116	0.561017	146.4279	0.624067	144.7024	0.56485	147.4457	0.540617	150.0856	0.753017	145.1289
	0.554667	149.1896	0.577683	146.3621	0.640733	144.662	0.581517	147.4087	0.557283	150.0849	0.769683	145.072
	0.571333	149.1699	0.59435	146.2976	0.6574	144.6188	0.598183	147.3737	0.57395	150.0875	0.78635	145.0274
	0.588	149.1488	0.611017	146.2349	0.674067	144.5772	0.61485	147.3385	0.590617	150.0854	0.803017	144.9752
	0.604667	149.1274	0.627683	146.1771	0.690733	144.541	0.631517	147.2991	0.607283	150.0853	0.819683	144.9257
	0.621333	149.1063	0.64435	146.1123	0.7074	144.4996	0.648183	147.2638	0.62395	150.0886	0.83635	144.8777
	0.638	149.0867	0.661017	145.8536	0.724067	144.4627	0.66485	147.2329	0.640617	150.0853	0.853017	144.8346
	0.654667	149.0655	0.677683	145.7821	0.740733	144.4293	0.681517	147.2037	0.657283	150.085	0.869683	144.7883
	0.671333	149.0456	0.69435	145.7195	0.7574	144.3871	0.698183	147.169	0.67395	150.085	0.88635	144.7458
	0.688	149.0269	0.711017	145.6584	0.774067	144.3525	0.71485	147.1374	0.690617	150.0831	0.903017	144.6865
	0.704667	149.0052	0.727683	145.6022	0.790733	144.3187	0.731517	147.1064	0.707283	150.0845	0.919683	144.6436
	0.721333	148.9864	0.74435	145.5442	0.8074	144.2828	0.748183	147.0797	0.72395	150.0856	0.93635	144.6025
	0.738	148.9665	0.761017	145.4891	0.824067	144.2475	0.76485	147.0479	0.740617	150.0851	0.953017	144.5617
	0.754667	148.947	0.777683	145.4369	0.840733	144.2123	0.781517	147.0203	0.757283	150.085	0.969683	144.5202
	0.771333	148.9266	0.79435	145.3824	0.8574	144.1813	0.798183	146.9909	0.77395	150.0837	0.98635	144.4797
	0.788	148.9077	0.811017	145.3316	0.874067	144.1479	0.81485	146.9616	0.790617	150.0826	1.003017	144.4409
	0.804667	148.8916	0.827683	145.2827	0.890733	144.1149	0.831517	146.9345	0.807283	150.0819	1.019683	144.4
	0.821333	148.8676	0.84435	145.2334	0.9074	144.0829	0.848183	146.9059	0.82395	150.0816	1.03635	144.3632
	0.838	148.8536	0.861017	145.184	0.924067	144.0483	0.86485	146.8812	0.840617	150.0802	1.053017	144.325
	0.854667	148.8355	0.877683	145.1357	0.940733	144.0091	0.881517	146.8553	0.857283	150.0831	1.069683	144.2878
	0.871333	148.8146	0.89435	145.088	0.9574	143.9746	0.898183	146.8275	0.87395	150.082	1.08635	144.2531
	0.888	148.7985	0.911017	145.0395	0.974067	143.9352	0.91485	146.803	0.890617	150.0788	1.103017	144.2168
	0.904667	148.7787	0.927683	144.9951	0.990733	143.9041	0.931517	146.778	0.907283	150.083	1.119683	144.178
	0.921333	148.7608	0.94435	144.9494	1.0074	143.8715	0.948183	146.7517	0.92395	150.0783	1.13635	144.1466
	0.938	148.7426	0.961017	144.9024	1.024067	143.837	0.96485	146.7275	0.940617	150.0823	1.153017	144.1171
	0.954667	148.7255	0.977683	144.8588	1.040733	143.8092	0.981517	146.7048	0.957283	150.0799	1.169683	144.081
	0.971333	148.6981	0.99435	144.8156	1.0574	143.7786	0.998183	146.6824	0.97395	150.0789	1.18635	144.0501
	0.988	148.6268	1.011017	144.7706	1.074067	143.7517	1.01485	146.6585	0.990617	150.0783	1.203017	144.0168
	1.004667	148.6113	1.027683	144.7282	1.090733	143.7233	1.031517	146.6341	1.007283	150.0785	1.219683	143.9906
	1.021333	148.5939	1.04435	144.6853	1.1074	143.6962	1.048183	146.6108	1.02395	150.0791	1.23635	143.9579
	1.038	148.5745	1.061017	144.6431	1.124067	143.6708	1.06485	146.5898	1.040617	150.0766	1.253017	143.9289
	1.054667	148.554	1.077683	144.6004	1.140733	143.6451	1.081517	146.5639	1.057283	150.0801	1.269683	143.9002
	1.071333	148.5404	1.09435	144.562	1.1574	143.6188	1.098183	146.5413	1.07395	150.0765	1.28635	143.8708
	1.088	148.5125	1.111017	144.5179	1.174067	143.5916	1.11485	146.5224	1.090617	150.0767	1.303017	143.8435
	1.104667	148.4923	1.127683	144.4797	1.190733	143.5661	1.131517	146.5038	1.107283	150.077	1.319683	143.8129
	1.121333	148.471	1.14435	144.4387	1.2074	143.5408	1.148183	146.4828	1.12395	150.0773	1.33635	143.7867

1.138	148.4506	1.161017	144.3978	1.224067	143.5174	1.16485	146.4625	1.140617	150.0733	1.353017	143.7592
1.154667	148.4383	1.177683	144.3584	1.240733	143.4945	1.181517	146.4432	1.157283	150.0765	1.369683	143.7311
1.171333	148.4255	1.19435	144.3184	1.2574	143.4721	1.198183	146.4217	1.17395	150.0757	1.38635	143.7028
1.188	148.4013	1.211017	144.2828	1.274067	143.4452	1.21485	146.4033	1.190617	150.0758	1.403017	143.6752
1.204667	148.3865	1.227683	144.2443	1.290733	143.4225	1.231517	146.3855	1.207283	150.0731	1.419683	143.6534
1.221333	148.3717	1.24435	144.2043	1.3074	143.3979	1.248183	146.3669	1.22395	150.0746	1.43635	143.6242
1.238	148.3502	1.261017	144.1666	1.324067	143.3752	1.26485	146.345	1.240617	150.0737	1.453017	143.6001
1.254667	148.3335	1.277683	144.1302	1.340733	143.3521	1.281517	146.3253	1.257283	150.0722	1.469683	143.5733
1.271333	148.3255	1.29435	144.092	1.3574	143.3297	1.298183	146.3082	1.27395	150.0709	1.48635	143.5512
1.288	148.3036	1.311017	144.0556	1.374067	143.3026	1.31485	146.2907	1.290617	150.0709	1.503017	143.5228
1.304667	148.2857	1.327683	144.022	1.390733	143.2811	1.331517	146.2732	1.307283	150.0701	1.519683	143.4995
1.321333	148.2681	1.34435	143.9849	1.4074	143.26	1.348183	146.2559	1.32395	150.0682	1.53635	143.4783
1.338	148.2506	1.361017	143.9505	1.424067	143.2385	1.36485	146.2386	1.340617	150.0721	1.553017	143.4521
1.354667	148.2346	1.377683	143.9147	1.440733	143.214	1.381517	146.2214	1.357283	150.0684	1.569683	143.4273
1.371333	148.2201	1.39435	143.8773	1.4574	143.1918	1.398183	146.2026	1.37395	150.0696	1.58635	143.4053
1.388	148.2034	1.411017	143.8432	1.474067	143.171	1.41485	146.1889	1.390617	150.0657	1.603017	143.3831
1.404667	148.1899	1.427683	143.811	1.490733	143.1532	1.431517	146.1697	1.407283	150.0689	1.619683	143.3616
1.421333	148.1741	1.44435	143.777	1.5074	143.1295	1.448183	146.1572	1.42395	150.0651	1.63635	143.3397
1.438	148.1624	1.461017	143.7428	1.524067	143.109	1.46485	146.1387	1.440617	150.0653	1.653017	143.3174
1.454667	148.1507	1.477683	143.7111	1.540733	143.088	1.481517	146.1244	1.457283	150.0647	1.669683	143.2981
1.471333	148.1318	1.49435	143.6756	1.5574	143.0683	1.498183	146.1085	1.47395	150.065	1.68635	143.2769
1.488	148.1193	1.511017	143.6421	1.574067	143.0493	1.51485	146.092	1.490617	150.0638	1.703017	143.2517
1.504667	148.106	1.527683	143.61	1.590733	143.0282	1.531517	146.0763	1.507283	150.0617	1.719683	143.2313
1.521333	148.0868	1.54435	143.5782	1.6074	143.0076	1.548183	146.0611	1.52395	150.0634	1.73635	143.2131
1.538	148.0702	1.561017	143.5459	1.624067	142.9891	1.56485	146.046	1.540617	150.0607	1.753017	143.1908
1.554667	148.058	1.577683	143.5135	1.640733	142.9684	1.581517	146.031	1.557283	150.0602	1.769683	143.17
1.571333	148.0405	1.59435	143.4855	1.6574	142.9501	1.598183	146.0145	1.57395	150.0628	1.78635	143.1521
1.588	148.0219	1.611017	143.4544	1.674067	142.9323	1.61485	146.0009	1.590617	150.0594	1.803017	143.1334
1.604667	148.0095	1.627683	143.4249	1.690733	142.9113	1.631517	145.9886	1.607283	150.0587	1.819683	143.1136
1.621333	147.9921	1.64435	143.3904	1.7074	142.8924	1.648183	145.9735	1.62395	150.0601	1.83635	143.0931
1.638	147.9762	1.661017	143.361	1.724067	142.8756	1.66485	145.9573	1.640617	150.0587	1.853017	143.0763
1.654667	147.9625	1.677683	143.3343	1.740733	142.8553	1.681517	145.9424	1.657283	150.0585	1.869683	143.0557
1.671333	147.9455	1.69435	143.3012	1.7574	142.8365	1.698183	145.9279	1.67395	150.0578	1.88635	143.0401
1.688	147.93	1.711017	143.2736	1.774067	142.8176	1.71485	145.9189	1.690617	150.0556	1.903017	143.0225
1.704667	147.9178	1.727683	143.2479	1.790733	142.7993	1.731517	145.9043	1.707283	150.053	1.919683	143.0064
1.721333	147.8973	1.74435	143.2174	1.8074	142.7819	1.748183	145.8907	1.72395	150.0543	1.93635	142.9866
1.738	147.8879	1.761017	143.1847	1.824067	142.7627	1.76485	145.8739	1.740617	150.054	1.953017	142.9721
1.754667	147.8728	1.777683	143.1607	1.840733	142.7425	1.781517	145.8602	1.757283	150.0539	1.969683	142.9484
1.771333	147.8586	1.79435	143.1328	1.8574	142.7295	1.798183	145.8475	1.77395	150.0521	1.98635	142.9257
1.788	147.8451	1.811017	143.1004	1.874067	142.7066	1.81485	145.8349	1.790617	150.0534	2.003017	142.9004
1.804667	147.8308	1.827683	143.073	1.890733	142.6931	1.831517	145.8225	1.807283	150.0516	2.019683	142.8815
1.821333	147.8168	1.84435	143.045	1.9074	142.6746	1.848183	145.8077	1.82395	150.0512	2.03635	142.8633
1.838	147.8028	1.861017	143.0173	1.924067	142.6571	1.86485	145.7942	1.840617	150.0488	2.053017	142.8398
1.854667	147.7882	1.877683	142.994	1.940733	142.6385	1.881517	145.7821	1.857283	150.0499	2.069683	142.8272
1.871333	147.7753	1.89435	142.9669	1.9574	142.6233	1.898183	145.7712	1.87395	150.0508	2.08635	142.8084
1.888	147.763	1.911017	142.9374	1.974067	142.6073	1.91485	145.7591	1.890617	150.049	2.103017	142.7917
1.904667	147.7474	1.927683	142.912	1.990733	142.5915	1.931517	145.7444	1.907283	150.0464	2.119683	142.7736
1.921333	147.7346	1.94435	142.8837	2.0074	142.5758	1.948183	145.7333	1.92395	150.0481	2.13635	142.7562
1.938	147.7196	1.961017	142.8574	2.024067	142.5568	1.96485	145.7219	1.940617	150.0453	2.153017	142.7368
1.954667	147.7097	1.977683	142.8299	2.040733	142.5417	1.981517	145.7065	1.957283	150.0464	2.169683	142.7213
1.971333	147.6962	1.99435	142.8046	2.0574	142.523	1.998183	145.6987	1.97395	150.0436	2.18635	142.7053
1.988	147.6851	2.011017	142.779	2.074067	142.5054	2.01485	145.6878	1.990617	150.0451	2.203017	142.6847
2.004667	147.6724	2.027683	142.7532	2.090733	142.4914	2.031517	145.6753	2.007283	150.0437	2.219683	142.673
2.021333	147.6572	2.04435	142.7264	2.1074	142.4759	2.048183	145.6613	2.02395	150.0441	2.23635	142.6545
2.038	147.6469	2.061017	142.7005	2.124067	142.4625	2.06485	145.6507	2.040617	150.0414	2.253017	142.6398
2.054667	147.6354	2.077683	142.6774	2.140733	142.4466	2.081517	145.6392	2.057283	150.0423	2.269683	142.6217
2.071333	147.6221	2.09435	142.6513	2.1574	142.4282	2.098183	145.6309	2.07395	150.0415	2.28635	142.6079
2.088	147.6075	2.111017	142.6286	2.174067	142.4062	2.11485	145.6174	2.090617	150.0393	2.303017	142.5915
2.104667	147.5964	2.127683	142.6031	2.190733	142.3943	2.131517	145.605	2.107283	150.0416	2.319683	142.5764
2.121333	147.5823	2.14435	142.5782	2.2074	142.3804	2.148183	145.5935	2.12395	150.0379	2.33635	142.5585
2.138	147.5683	2.161017	142.5531	2.224067	142.3663	2.16485	145.5796	2.140617	150.0369	2.353017	142.5455
2.154667	147.5548	2.177683	142.5291	2.240733	142.3493	2.181517	145.5731	2.157283	150.0364	2.369683	142.5255
2.171333	147.5429	2.19435	142.5061	2.2574	142.3326	2.198183	145.5601	2.17395	150.0328	2.38635	142.5136
2.188	147.5282	2.211017	142.4822	2.274067	142.3181	2.21485	145.5509	2.190617	150.0352	2.403017	142.4975
2.204667	147.5155	2.227683	142.4574	2.290733	142.3054	2.231517	145.5397	2.207283	150.0343	2.419683	142.4814
2.221333	147.5017	2.24435	142.434	2.3074	142.2876	2.248183	145.5278	2.22395	150.0361	2.43635	142.4631
2.238	147.4876	2.261017	142.4127	2.324067	142.272	2.26485	145.5229	2.240617	150.0333	2.453017	142.4495
2.254667	147.4732	2.277683	142.3901	2.340733	142.2582	2.281517	145.5095	2.257283	150.0314	2.469683	142.4307
2.271333	147.4662	2.29435	142.3656	2.3574	142.2445	2.298183	145.4992	2.27395	150.0291	2.48635	142.4186

2.288	147.4511	2.311017	142.3428	2.374067	142.2271	2.31485	145.4907	2.290617	150.0309	2.503017	142.4004
2.304667	147.4412	2.327683	142.3226	2.390733	142.2146	2.331517	145.4795	2.307283	150.0302	2.519683	142.3854
2.321333	147.4297	2.34435	142.3006	2.4074	142.1979	2.348183	145.472	2.32395	150.0293	2.53635	142.3694
2.338	147.4186	2.361017	142.2789	2.424067	142.1863	2.36485	145.4583	2.340617	150.0295	2.553017	142.3567
2.354667	147.4082	2.377683	142.2559	2.440733	142.1672	2.381517	145.451	2.357283	150.0272	2.569683	142.3365
2.371333	147.3921	2.39435	142.2348	2.4574	142.1558	2.398183	145.4403	2.37395	150.0269	2.58635	142.3232
2.388	147.3733	2.411017	142.2089	2.474067	142.1408	2.41485	145.4299	2.390617	150.0281	2.603017	142.3105
2.404667	147.3614	2.427683	142.1911	2.490733	142.1264	2.431517	145.4189	2.407283	150.0249	2.619683	142.296
2.421333	147.3545	2.44435	142.165	2.5074	142.1112	2.448183	145.4131	2.42395	150.0236	2.63635	142.2792
2.438	147.3388	2.461017	142.1434	2.524067	142.0992	2.46485	145.3997	2.440617	150.0214	2.653017	142.2668
2.454667	147.3276	2.477683	142.1216	2.540733	142.0867	2.481517	145.3888	2.457283	150.0211	2.669683	142.2512
2.471333	147.3136	2.49435	142.102	2.5574	142.0696	2.498183	145.3805	2.47395	150.0227	2.68635	142.2369
2.488	147.3015	2.511017	142.0808	2.574067	142.0559	2.51485	145.372	2.490617	150.0181	2.703017	142.2226
2.504667	147.2902	2.527683	142.0616	2.590733	142.0427	2.531517	145.3624	2.507283	150.0207	2.719683	142.2067
2.521333	147.2757	2.54435	142.04	2.6074	142.026	2.548183	145.352	2.52395	150.0209	2.73635	142.1938
2.538	147.2647	2.561017	142.0196	2.624067	142.0151	2.56485	145.3413	2.540617	150.0185	2.753017	142.18
2.554667	147.252	2.577683	141.9981	2.640733	142.0003	2.581517	145.3349	2.557283	150.0176	2.769683	142.1665
2.571333	147.2394	2.59435	141.9787	2.6574	141.9872	2.598183	145.3237	2.57395	150.0167	2.78635	142.1538
2.588	147.231	2.611017	141.9555	2.674067	141.9727	2.61485	145.3169	2.590617	150.0146	2.803017	142.1428
2.604667	147.2184	2.627683	141.9372	2.690733	141.9546	2.631517	145.3064	2.607283	150.0147	2.819683	142.1322
2.621333	147.206	2.64435	141.9115	2.7074	141.9431	2.648183	145.2968	2.62395	150.0118	2.83635	142.1167
2.638	147.1948	2.661017	141.8929	2.724067	141.9307	2.66485	145.289	2.640617	150.0111	2.853017	142.1048
2.654667	147.181	2.677683	141.8735	2.740733	141.9148	2.681517	145.2797	2.657283	150.0111	2.869683	142.0944
2.671333	147.1658	2.69435	141.8547	2.7574	141.9008	2.698183	145.2693	2.67395	150.012	2.88635	142.0792
2.688	147.1614	2.711017	141.8359	2.774067	141.8866	2.71485	145.263	2.690617	150.0089	2.903017	142.0684
2.704667	147.1483	2.727683	141.8177	2.790733	141.8745	2.731517	145.2526	2.707283	150.0055	2.919683	142.059
2.721333	147.1355	2.74435	141.7989	2.8074	141.8628	2.748183	145.2445	2.72395	150.0047	2.93635	142.0444
2.738	147.1263	2.761017	141.7776	2.824067	141.8505	2.76485	145.235	2.740617	150.0106	2.953017	142.0342
2.754667	147.1105	2.777683	141.7601	2.840733	141.8354	2.781517	145.2272	2.757283	150.0065	2.969683	142.019
2.771333	147.0989	2.79435	141.7401	2.8574	141.8232	2.798183	145.2195	2.77395	150.0041	2.98635	142.006
2.788	147.0884	2.811017	141.7217	2.874067	141.8107	2.81485	145.2118	2.790617	150.0034	3.003017	141.997
2.804667	147.0721	2.827683	141.7002	2.890733	141.7983	2.831517	145.2033	2.807283	150.0028	3.019683	141.9795
2.821333	147.0636	2.84435	141.6788	2.9074	141.7854	2.848183	145.1951	2.82395	150.0029	3.03635	141.9663
2.838	147.0518	2.861017	141.6587	2.924067	141.7714	2.86485	145.1857	2.840617	149.9998	3.053017	141.955
2.854667	147.0388	2.877683	141.6426	2.940733	141.7624	2.881517	145.1787	2.857283	150.0017	3.069683	141.9444
2.871333	147.0243	2.89435	141.6261	2.9574	141.7475	2.898183	145.1709	2.87395	150.0006	3.08635	141.93
2.888	147.0164	2.911017	141.6059	2.974067	141.7352	2.91485	145.1619	2.890617	149.9995	3.103017	141.9167
2.904667	147.005	2.927683	141.5884	2.990733	141.7253	2.931517	145.1531	2.907283	149.997	3.119683	141.9052
2.921333	146.9909	2.94435	141.5687	3.0074	141.7118	2.948183	145.1464	2.92395	149.9922	3.13635	141.8972
2.938	146.9827	2.961017	141.5498	3.024067	141.6999	2.96485	145.1361	2.940617	149.9929	3.153017	141.8836
2.954667	146.9686	2.977683	141.533	3.040733	141.6872	2.981517	145.1306	2.957283	149.9948	3.169683	141.8713
2.971333	146.9599	2.99435	141.5145	3.0574	141.6723	2.998183	145.1195	2.97395	149.9895	3.18635	141.8601
2.988	146.9479	3.011017	141.4951	3.074067	141.6623	3.01485	145.1147	2.990617	149.9871	3.203017	141.854
3.004667	146.9412	3.027683	141.475	3.090733	141.6505	3.031517	145.1067	3.007283	149.9912	3.219683	141.837
3.021333	146.9253	3.04435	141.4601	3.1074	141.636	3.048183	145.0982	3.02395	149.9888	3.23635	141.8272
3.038	146.9206	3.061017	141.441	3.124067	141.6257	3.06485	145.089	3.040617	149.9846	3.253017	141.8153
3.054667	146.9059	3.077683	141.4252	3.140733	141.6128	3.081517	145.081	3.057283	149.9925	3.269683	141.8022
3.071333	146.8967	3.09435	141.4097	3.1574	141.6013	3.098183	145.0741	3.07395	149.9866	3.28635	141.7914
3.088	146.8884	3.111017	141.3895	3.174067	141.5908	3.11485	145.0683	3.090617	149.9864	3.303017	141.7786
3.104667	146.8768	3.127683	141.3739	3.190733	141.5772	3.131517	145.0558	3.107283	149.9847	3.319683	141.7651
3.121333	146.8632	3.14435	141.3544	3.2074	141.5679	3.148183	145.0485	3.12395	149.9846	3.33635	141.7552
3.138	146.8535	3.161017	141.3363	3.224067	141.5548	3.16485	145.0444	3.140617	149.9831	3.353017	141.7435
3.154667	146.8441	3.177683	141.3197	3.240733	141.546	3.181517	145.0302	3.157283	149.985	3.369683	141.7342
3.171333	146.8405	3.19435	141.3047	3.2574	141.5348	3.198183	145.0277	3.17395	149.9819	3.38635	141.7223
3.188	146.8241	3.211017	141.2854	3.274067	141.5211	3.21485	145.0183	3.190617	149.9826	3.403017	141.7124
3.204667	146.8164	3.227683	141.2705	3.290733	141.5098	3.231517	145.012	3.207283	149.9807	3.419683	141.7004
3.221333	146.8017	3.24435	141.2517	3.3074	141.4991	3.248183	145.0033	3.22395	149.9842	3.43635	141.6898
3.238	146.7953	3.261017	141.2358	3.324067	141.4861	3.26485	144.9963	3.240617	149.9768	3.453017	141.6753
3.254667	146.7836	3.277683	141.2139	3.340733	141.4754	3.281517	144.9907	3.257283	149.9777	3.469683	141.6644
3.271333	146.7723	3.29435	141.2008	3.3574	141.466	3.298183	144.9813	3.27395	149.9802	3.48635	141.6508
3.288	146.7613	3.311017	141.1858	3.374067	141.4519	3.31485	144.9765	3.290617	149.9725	3.503017	141.6447
3.304667	146.7505	3.327683	141.1721	3.390733	141.4388	3.331517	144.9696	3.307283	149.9782	3.519683	141.6318
3.321333	146.7435	3.34435	141.1533	3.4074	141.4267	3.348183	144.9597	3.32395	149.972	3.53635	141.6183
3.338	146.7322	3.361017	141.136	3.424067	141.4165	3.36485	144.9552	3.340617	149.9749	3.553017	141.6112
3.354667	146.7221	3.377683	141.1178	3.440733	141.4024	3.381517	144.9485	3.357283	149.9705	3.569683	141.6002
3.371333	146.7122	3.39435	141.1031	3.4574	141.3892	3.398183	144.9403	3.37395	149.9669	3.58635	141.5921
3.388	146.7036	3.411017	141.0844	3.474067	141.3758	3.41485	144.9358	3.390617	149.9682	3.603017	141.582
3.404667	146.6907	3.427683	141.0658	3.490733	141.3615	3.431517	144.9269	3.407283	149.9689	3.619683	141.5728
3.421333	146.6797	3.44435	141.0516	3.5074	141.3532	3.448183	144.9203	3.42395	149.9663	3.63635	141.5635

3.438	146.672	3.461017	141.0346	3.524067	141.3441	3.46485	144.9133	3.440617	149.9648	3.653017	141.5534
3.454667	146.6608	3.477683	141.022	3.540733	141.3305	3.481517	144.9067	3.457283	149.9636	3.669683	141.5443
3.471333	146.6525	3.49435	141.0034	3.5574	141.3205	3.498183	144.9008	3.47395	149.9616	3.68635	141.5354
3.488	146.6407	3.511017	140.9896	3.574067	141.3061	3.51485	144.8933	3.490617	149.9636	3.703017	141.5237
3.504667	146.6337	3.527683	140.9707	3.590733	141.2986	3.531517	144.8882	3.507283	149.9646	3.719683	141.5139
3.521333	146.6239	3.54435	140.9549	3.6074	141.2882	3.548183	144.8797	3.52395	149.9622	3.73635	141.5055
3.538	146.6139	3.561017	140.9399	3.624067	141.2761	3.56485	144.8717	3.540617	149.9595	3.753017	141.4968
3.554667	146.6053	3.577683	140.924	3.640733	141.2651	3.581517	144.8661	3.557283	149.9609	3.769683	141.4869
3.571333	146.5928	3.59435	140.9103	3.6574	141.2552	3.598183	144.8554	3.57395	149.9573	3.78635	141.4757
3.588	146.5867	3.611017	140.8935	3.674067	141.2446	3.61485	144.8525	3.590617	149.9584	3.803017	141.4662
3.604667	146.5758	3.627683	140.881	3.690733	141.2361	3.631517	144.8435	3.607283	149.9544	3.819683	141.4576
3.621333	146.569	3.64435	140.8679	3.7074	141.2236	3.648183	144.8385	3.62395	149.955	3.83635	141.4484
3.638	146.5593	3.661017	140.8502	3.724067	141.213	3.66485	144.8285	3.640617	149.952	3.853017	141.4396
3.654667	146.5475	3.677683	140.8357	3.740733	141.2024	3.681517	144.8241	3.657283	149.9545	3.869683	141.4274
3.671333	146.5412	3.69435	140.8187	3.7574	141.1906	3.698183	144.8202	3.67395	149.9518	3.88635	141.4192
3.688	146.5294	3.711017	140.8061	3.774067	141.1794	3.71485	144.811	3.690617	149.9494	3.903017	141.4092
3.704667	146.5219	3.727683	140.7915	3.790733	141.1709	3.731517	144.8067	3.707283	149.9506	3.919683	141.4005
3.721333	146.5121	3.74435	140.7764	3.8074	141.1597	3.748183	144.7992	3.72395	149.9528	3.93635	141.3917
3.738	146.5032	3.761017	140.7587	3.824067	141.1499	3.76485	144.7926	3.740617	149.9486	3.953017	141.3843
3.754667	146.4919	3.777683	140.7484	3.840733	141.1394	3.781517	144.7883	3.757283	149.9483	3.969683	141.3724
3.771333	146.4826	3.79435	140.7323	3.8574	141.1275	3.798183	144.7812	3.77395	149.9464	3.98635	141.3664
3.788	146.4722	3.811017	140.7186	3.874067	141.1176	3.81485	144.7758	3.790617	149.9488	4.003017	141.3543
3.804667	146.4645	3.827683	140.7013	3.890733	141.1094	3.831517	144.7676	3.807283	149.9423	4.019683	141.3473
3.821333	146.4543	3.84435	140.6891	3.9074	141.0977	3.848183	144.7634	3.82395	149.9453	4.03635	141.3367
3.838	146.4405	3.861017	140.6732	3.924067	141.0877	3.86485	144.7593	3.840617	149.942	4.053017	141.33
3.854667	146.4343	3.877683	140.6569	3.940733	141.0763	3.881517	144.7515	3.857283	149.9421	4.069683	141.3158
3.871333	146.4288	3.89435	140.6437	3.9574	141.0675	3.898183	144.7433	3.87395	149.9368	4.08635	141.3105
3.888	146.4134	3.911017	140.6339	3.974067	141.0583	3.91485	144.7424	3.890617	149.9397	4.103017	141.3
3.904667	146.4061	3.927683	140.6123	3.990733	141.048	3.931517	144.7335	3.907283	149.9358	4.119683	141.2931
3.921333	146.3996	3.94435	140.603	4.0074	141.0354	3.948183	144.7284	3.92395	149.9377	4.13635	141.2815
3.938	146.3904	3.961017	140.5886	4.024067	141.0267	3.96485	144.722	3.940617	149.9307	4.153017	141.2713
3.954667	146.3806	3.977683	140.5729	4.040733	141.0147	3.981517	144.7164	3.957283	149.9351	4.169683	141.2656
3.971333	146.3681	3.99435	140.5589	4.0574	141.0051	3.998183	144.7104	3.97395	149.93	4.18635	141.2598
3.988	146.3596	4.011017	140.5447	4.074067	140.9976	4.01485	144.7051	3.990617	149.9305	4.203017	141.2425
4.004667	146.3491	4.027683	140.5324	4.090733	140.9898	4.031517	144.6986	4.007283	149.9316	4.219683	141.2418
4.021333	146.3415	4.04435	140.521	4.1074	140.9751	4.048183	144.6924	4.02395	149.9273	4.23635	141.2287
4.038	146.3337	4.061017	140.5054	4.124067	140.9639	4.06485	144.686	4.040617	149.9319	4.253017	141.222
4.054667	146.3236	4.077683	140.4911	4.140733	140.9535	4.081517	144.6813	4.057283	149.9266	4.269683	141.2117
4.071333	146.3131	4.09435	140.4756	4.1574	140.9447	4.098183	144.6698	4.07395	149.9262	4.28635	141.2021
4.088	146.3027	4.111017	140.461	4.174067	140.9347	4.11485	144.6669	4.090617	149.928	4.303017	141.1921
4.104667	146.2963	4.127683	140.4475	4.190733	140.9256	4.131517	144.6613	4.107283	149.9259	4.319683	141.1879
4.121333	146.291	4.14435	140.4309	4.2074	140.913	4.148183	144.6535	4.12395	149.9237	4.33635	141.1762
4.138	146.2796	4.161017	140.4215	4.224067	140.9052	4.16485	144.65	4.140617	149.9261	4.353017	141.1718
4.154667	146.2682	4.177683	140.4064	4.240733	140.8947	4.181517	144.6435	4.157283	149.9192	4.369683	141.1631
4.171333	146.2597	4.19435	140.3929	4.2574	140.886	4.198183	144.6369	4.17395	149.9208	4.38635	141.1551
4.188	146.2488	4.211017	140.3795	4.274067	140.8729	4.21485	144.6337	4.190617	149.9198	4.403017	141.1459
4.204667	146.2439	4.227683	140.3684	4.290733	140.8672	4.231517	144.6231	4.207283	149.9186	4.419683	141.1374
4.221333	146.2335	4.24435	140.3542	4.3074	140.8574	4.248183	144.6195	4.22395	149.9182	4.43635	141.1295
4.238	146.2246	4.261017	140.3392	4.324067	140.8452	4.26485	144.6137	4.240617	149.9144	4.453017	141.1233
4.254667	146.2143	4.277683	140.3277	4.340733	140.8418	4.281517	144.6099	4.257283	149.9181	4.469683	141.115
4.271333	146.2064	4.29435	140.3156	4.3574	140.8285	4.298183	144.6036	4.27395	149.9145	4.48635	141.1072
4.288	146.1978	4.311017	140.3026	4.374067	140.8163	4.31485	144.5978	4.290617	149.9124	4.503017	141.0989
4.304667	146.188	4.327683	140.2861	4.390733	140.8091	4.331517	144.5907	4.307283	149.9116	4.519683	141.0947
4.321333	146.1815	4.34435	140.2736	4.4074	140.8004	4.348183	144.5854	4.32395	149.9137	4.53635	141.0834
4.338	146.1744	4.361017	140.2556	4.424067	140.7928	4.36485	144.5821	4.340617	149.9108	4.553017	141.0766
4.354667	146.1653	4.377683	140.2468	4.440733	140.7831	4.381517	144.5784	4.357283	149.9107	4.569683	141.0677
4.371333	146.1536	4.39435	140.2358	4.4574	140.7722	4.398183	144.57	4.37395	149.9101	4.58635	141.0612
4.388	146.147	4.411017	140.2201	4.474067	140.7651	4.41485	144.5659	4.390617	149.9079	4.603017	141.0527
4.404667	146.1378	4.427683	140.2057	4.490733	140.7537	4.431517	144.5613	4.407283	149.9071	4.619683	141.0463
4.421333	146.1278	4.44435	140.1948	4.5074	140.7474	4.448183	144.5588	4.42395	149.9055	4.63635	141.0376
4.438	146.1233	4.461017	140.1769	4.524067	140.7389	4.46485	144.5532	4.440617	149.9042	4.653017	141.0298
4.454667	146.1122	4.477683	140.161	4.540733	140.7281	4.481517	144.547	4.457283	149.9025	4.669683	141.0236
4.471333	146.1062	4.49435	140.1495	4.5574	140.7175	4.498183	144.541	4.47395	149.9031	4.68635	141.0156
4.488	146.0955	4.511017	140.1403	4.574067	140.71	4.51485	144.5397	4.490617	149.9031	4.703017	141.0047
4.504667	146.0873	4.527683	140.1276	4.590733	140.7004	4.531517	144.5344	4.507283	149.8992	4.719683	140.997
4.521333	146.0753	4.54435	140.1134	4.6074	140.6914	4.548183	144.5289	4.52395	149.8991	4.73635	140.9909
4.538	146.07	4.561017	140.1003	4.624067	140.6812	4.56485	144.5242	4.540617	149.8963	4.753017	140.9843
4.554667	146.0616	4.577683	140.0876	4.640733	140.6738	4.581517	144.518	4.557283	149.8962	4.769683	140.9736
4.571333	146.0518	4.59435	140.0781	4.6574	140.6671	4.598183	144.515	4.57395	149.8931	4.78635	140.9692

4.588	146.0404	4.611017	140.0645	4.674067	140.6532	4.61485	144.5118	4.590617	149.8959	4.803017	140.9613
4.604667	146.0322	4.627683	140.0494	4.690733	140.6428	4.631517	144.5019	4.607283	149.8922	4.819683	140.954
4.621333	146.022	4.64435	140.0415	4.7074	140.6371	4.648183	144.4974	4.62395	149.8903	4.83635	140.9459
4.638	146.0136	4.661017	140.0291	4.724067	140.6252	4.66485	144.4939	4.640617	149.892	4.853017	140.9399
4.654667	146.0078	4.677683	140.0152	4.740733	140.6142	4.681517	144.4876	4.657283	149.8896	4.869683	140.9306
4.671333	145.9998	4.69435	140.0053	4.7574	140.6087	4.698183	144.4826	4.67395	149.8879	4.88635	140.9251
4.688	145.989	4.711017	139.9921	4.774067	140.6013	4.71485	144.4795	4.690617	149.8872	4.903017	140.9175
4.704667	145.9841	4.727683	139.9796	4.790733	140.5908	4.731517	144.473	4.707283	149.8854	4.919683	140.9101
4.721333	145.9731	4.74435	139.9683	4.8074	140.5818	4.748183	144.4663	4.72395	149.8828	4.93635	140.9018
4.738	145.9665	4.761017	139.9531	4.824067	140.57	4.76485	144.4633	4.740617	149.8831	4.953017	140.8968
4.754667	145.9547	4.777683	139.9422	4.840733	140.563	4.781517	144.4578	4.757283	149.8851	4.969683	140.8848
4.771333	145.95	4.79435	139.9292	4.8574	140.5543	4.798183	144.4518	4.77395	149.8829	4.98635	140.8765
4.788	145.9389	4.811017	139.9192	4.874067	140.5457	4.81485	144.4502	4.790617	149.8786	5.003017	140.8728
4.804667	145.9323	4.827683	139.9063	4.890733	140.5393	4.831517	144.4436	4.807283	149.8794	5.019683	140.8651
4.821333	145.9235	4.84435	139.897	4.9074	140.5283	4.848183	144.4371	4.82395	149.8784	5.03635	140.8568
4.838	145.9085	4.861017	139.8837	4.924067	140.5179	4.86485	144.4338	4.840617	149.8756	5.053017	140.8518
4.854667	145.9032	4.877683	139.8745	4.940733	140.5101	4.881517	144.4275	4.857283	149.8747	5.069683	140.8428
4.871333	145.8964	4.89435	139.86	4.9574	140.5003	4.898183	144.4226	4.87395	149.8744	5.08635	140.836
4.888	145.8862	4.911017	139.8508	4.974067	140.4898	4.91485	144.4185	4.890617	149.8723	5.103017	140.8295
4.904667	145.875	4.927683	139.8371	4.990733	140.4848	4.931517	144.4148	4.907283	149.8724	5.119683	140.8183
4.921333	145.8717	4.94435	139.8253	5.0074	140.4767	4.948183	144.4103	4.92395	149.868	5.13635	140.8139
4.938	145.8633	4.961017	139.8168	5.024067	140.4682	4.96485	144.4053	4.940617	149.8715	5.153017	140.8063
4.954667	145.8536	4.977683	139.8028	5.040733	140.4586	4.981517	144.4012	4.957283	149.8669	5.169683	140.8001
4.971333	145.8457	4.99435	139.7929	5.0574	140.45	4.998183	144.3918	4.97395	149.8655	5.18635	140.7928
4.988	145.833	5.011017	139.7823	5.074067	140.4386	5.01485	144.3915	4.990617	149.8673	5.203017	140.7871
5.004667	145.8304	5.027683	139.7698	5.090733	140.429	5.031517	144.3839	5.007283	149.8652	5.219683	140.7818
5.021333	145.8193	5.04435	139.757	5.1074	140.4225	5.048183	144.3799	5.02395	149.8616	5.23635	140.7715
5.038	145.812	5.061017	139.7472	5.124067	140.4111	5.06485	144.3737	5.040617	149.8648	5.253017	140.7661
5.054667	145.8047	5.077683	139.7359	5.140733	140.4033	5.081517	144.369	5.057283	149.8616	5.269683	140.7588
5.071333	145.7983	5.09435	139.7221	5.1574	140.3934	5.098183	144.3631	5.07395	149.8612	5.28635	140.7541
5.088	145.7894	5.111017	139.7115	5.174067	140.3879	5.11485	144.3589	5.090617	149.8589	5.303017	140.7453
5.104667	145.7821	5.127683	139.7011	5.190733	140.3781	5.131517	144.3547	5.107283	149.8588	5.319683	140.7391
5.121333	145.7723	5.14435	139.6889	5.2074	140.3697	5.148183	144.3505	5.12395	149.8591	5.33635	140.7308
5.138	145.7638	5.161017	139.6769	5.224067	140.3599	5.16485	144.3435	5.140617	149.8543	5.353017	140.7264
5.154667	145.757	5.177683	139.6694	5.240733	140.3529	5.181517	144.3412	5.157283	149.8512	5.369683	140.7166
5.171333	145.7479	5.19435	139.6537	5.2574	140.3441	5.198183	144.3315	5.17395	149.8532	5.38635	140.7084
5.188	145.7409	5.211017	139.6441	5.274067	140.34	5.21485	144.33	5.190617	149.853	5.403017	140.701
5.204667	145.7357	5.227683	139.635	5.290733	140.3279	5.231517	144.3265	5.207283	149.8503	5.419683	140.6962
5.221333	145.7244	5.24435	139.6196	5.3074	140.3218	5.248183	144.3214	5.22395	149.8466	5.43635	140.69
5.238	145.7215	5.261017	139.6141	5.324067	140.3117	5.26485	144.3156	5.240617	149.8493	5.453017	140.6798
5.254667	145.7128	5.277683	139.6042	5.340733	140.3027	5.281517	144.3139	5.257283	149.8489	5.469683	140.6755
5.271333	145.7046	5.29435	139.5918	5.3574	140.2983	5.298183	144.3053	5.27395	149.8438	5.48635	140.6675
5.288	145.6925	5.311017	139.5827	5.374067	140.2878	5.31485	144.3045	5.290617	149.8459	5.503017	140.662
5.304667	145.6898	5.327683	139.5707	5.390733	140.2795	5.331517	144.2979	5.307283	149.8446	5.519683	140.6552
5.321333	145.6821	5.34435	139.5605	5.4074	140.2741	5.348183	144.2961	5.32395	149.8437	5.53635	140.6483
5.338	145.6742	5.361017	139.5508	5.424067	140.268	5.36485	144.293	5.340617	149.8419	5.553017	140.6446
5.354667	145.6669	5.377683	139.5425	5.440733	140.2572	5.381517	144.2878	5.357283	149.8406	5.569683	140.6372
5.371333	145.6559	5.39435	139.5303	5.4574	140.2482	5.398183	144.2837	5.37395	149.8382	5.58635	140.6318
5.388	145.6514	5.411017	139.5167	5.474067	140.2385	5.41485	144.2789	5.390617	149.841	5.603017	140.6251
5.404667	145.6443	5.427683	139.508	5.490733	140.2312	5.431517	144.2762	5.407283	149.838	5.619683	140.6178
5.421333	145.6356	5.44435	139.4929	5.5074	140.2258	5.448183	144.2724	5.42395	149.836	5.63635	140.611
5.438	145.629	5.461017	139.4876	5.524067	140.2174	5.46485	144.2663	5.440617	149.8339	5.653017	140.6043
5.454667	145.6262	5.477683	139.4764	5.540733	140.2072	5.481517	144.2633	5.457283	149.8346	5.669683	140.6005
5.471333	145.6147	5.49435	139.46	5.5574	140.2	5.498183	144.2582	5.47395	149.8314	5.68635	140.592
5.488	145.6067	5.511017	139.4504	5.574067	140.192	5.51485	144.2547	5.490617	149.8308	5.703017	140.5849
5.504667	145.5981	5.527683	139.4406	5.590733	140.1834	5.531517	144.2515	5.507283	149.8306	5.719683	140.5775
5.521333	145.5899	5.54435	139.4306	5.6074	140.1784	5.548183	144.2478	5.52395	149.8309	5.73635	140.5675
5.538	145.5848	5.561017	139.4214	5.624067	140.1669	5.56485	144.2452	5.540617	149.8245	5.753017	140.5662
5.554667	145.5788	5.577683	139.4129	5.640733	140.1597	5.581517	144.2412	5.557283	149.8267	5.769683	140.5556
5.571333	145.5695	5.59435	139.4015	5.6574	140.1531	5.598183	144.234	5.57395	149.8248	5.78635	140.5499
5.588	145.5588	5.611017	139.3875	5.674067	140.1445	5.61485	144.2302	5.590617	149.8245	5.803017	140.544
5.604667	145.5525	5.627683	139.3826	5.690733	140.1359	5.631517	144.2262	5.607283	149.8213	5.819683	140.535
5.621333	145.5463	5.64435	139.3709	5.7074	140.128	5.648183	144.2249	5.62395	149.8217	5.83635	140.5285
5.638	145.537	5.661017	139.3562	5.724067	140.1202	5.66485	144.2177	5.640617	149.8184	5.853017	140.5212
5.654667	145.5279	5.677683	139.347	5.740733	140.1115	5.681517	144.2113	5.657283	149.8181	5.869683	140.5178
5.671333	145.5252	5.69435	139.3385	5.7574	140.1042	5.698183	144.2102	5.67395	149.8158	5.88635	140.5093
5.688	145.5173	5.711017	139.3324	5.774067	140.0959	5.71485	144.2048	5.690617	149.8181	5.903017	140.5039
5.704667	145.5117	5.727683	139.3183	5.790733	140.0878	5.731517	144.1977	5.707283	149.815	5.919683	140.4981
5.721333	145.5058	5.74435	139.3092	5.8074	140.0805	5.748183	144.1943	5.72395	149.8146	5.93635	140.4894

5.738	145.4973	5.761017	139.2965	5.824067	140.0727	5.76485	144.1905	5.740617	149.8118	5.953017	140.4866
5.754667	145.4896	5.777683	139.2882	5.840733	140.0648	5.781517	144.1884	5.757283	149.8128	5.969683	140.4781
5.771333	145.4819	5.79435	139.2769	5.8574	140.0556	5.798183	144.1852	5.77395	149.8096	5.98635	140.4728
5.788	145.4721	5.811017	139.2654	5.874067	140.0487	5.81485	144.1776	5.790617	149.8096	6.003017	140.4662
5.804667	145.4665	5.827683	139.2568	5.890733	140.0428	5.831517	144.1756	5.807283	149.8086	6.019683	140.4599
5.821333	145.4605	5.84435	139.2498	5.9074	140.0337	5.848183	144.1725	5.82395	149.8075	6.03635	140.4544
5.838	145.4542	5.861017	139.2371	5.924067	140.0277	5.86485	144.1664	5.840617	149.8052	6.053017	140.447
5.854667	145.4485	5.877683	139.2256	5.940733	140.0173	5.881517	144.1625	5.857283	149.8058	6.069683	140.4418
5.871333	145.4396	5.89435	139.2188	5.9574	140.0118	5.898183	144.1578	5.87395	149.8018	6.08635	140.4377
5.888	145.432	5.911017	139.2075	5.974067	140.0007	5.91485	144.1539	5.890617	149.8003	6.103017	140.4297
5.904667	145.4287	5.927683	139.1956	5.990733	139.9963	5.931517	144.1484	5.907283	149.8042	6.119683	140.4225
5.921333	145.4201	5.94435	139.1871	6.0074	139.9895	5.948183	144.1449	5.92395	149.8	6.13635	140.4182
5.938	145.4111	5.961017	139.1779	6.024067	139.9808	5.96485	144.1417	5.940617	149.7969	6.153017	140.4104
5.954667	145.4058	5.977683	139.1689	6.040733	139.9749	5.981517	144.1365	5.957283	149.7973	6.169683	140.4067
5.971333	145.3975	5.99435	139.1582	6.0574	139.9657	5.998183	144.1286	5.97395	149.7994	6.18635	140.3997
5.988	145.3908	6.011017	139.149	6.074067	139.961	6.01485	144.1268	5.990617	149.7967	6.203017	140.3909
6.004667	145.3833	6.027683	139.1403	6.090733	139.9504	6.031517	144.1232	6.007283	149.7943	6.219683	140.3861
6.021333	145.3786	6.04435	139.13	6.1074	139.9428	6.048183	144.1168	6.02395	149.7959	6.23635	140.3781
6.038	145.3709	6.061017	139.1215	6.124067	139.9371	6.06485	144.1147	6.040617	149.7946	6.253017	140.3746
6.054667	145.3629	6.077683	139.1123	6.140733	139.9282	6.081517	144.1112	6.057283	149.7911	6.269683	140.3676
6.071333	145.356	6.09435	139.101	6.1574	139.9225	6.098183	144.1076	6.07395	149.7916	6.28635	140.3621
6.088	145.3493	6.111017	139.094	6.174067	139.9153	6.11485	144.1037	6.090617	149.7887	6.303017	140.357
6.104667	145.3449	6.127683	139.0817	6.190733	139.9061	6.131517	144.0988	6.107283	149.7905	6.319683	140.3518
6.121333	145.3329	6.14435	139.0715	6.2074	139.9006	6.148183	144.0986	6.12395	149.7876	6.33635	140.3419
6.138	145.3295	6.161017	139.0634	6.224067	139.8934	6.16485	144.0904	6.140617	149.7848	6.353017	140.3404
6.154667	145.3217	6.177683	139.0543	6.240733	139.8881	6.181517	144.0891	6.157283	149.7853	6.369683	140.3342
6.171333	145.3136	6.19435	139.0444	6.2574	139.8796	6.198183	144.0863	6.17395	149.7902	6.38635	140.3264
6.188	145.303	6.211017	139.0378	6.274067	139.874	6.21485	144.0822	6.190617	149.7857	6.403017	140.322
6.204667	145.2926	6.227683	139.0251	6.290733	139.8673	6.231517	144.0752	6.207283	149.7799	6.419683	140.3113
6.221333	145.2925	6.24435	139.0182	6.3074	139.8562	6.248183	144.072	6.22395	149.7818	6.43635	140.3092
6.238	145.2798	6.261017	139.0064	6.324067	139.8507	6.26485	144.0709	6.240617	149.7778	6.453017	140.3032
6.254667	145.2744	6.277683	138.9981	6.340733	139.8473	6.281517	144.0641	6.257283	149.7805	6.469683	140.2973
6.271333	145.2702	6.29435	138.9889	6.3574	139.8389	6.298183	144.0603	6.27395	149.7763	6.48635	140.2885
6.288	145.2613	6.311017	138.9781	6.374067	139.8332	6.31485	144.0558	6.290617	149.7749	6.503017	140.2784
6.304667	145.2567	6.327683	138.9708	6.390733	139.8243	6.331517	144.0544	6.307283	149.7744	6.519683	140.2755
6.321333	145.2473	6.34435	138.9624	6.4074	139.8168	6.348183	144.0474	6.32395	149.7734	6.53635	140.2673
6.338	145.2381	6.361017	138.9521	6.424067	139.8081	6.36485	144.0437	6.340617	149.7726	6.553017	140.261
6.354667	145.2323	6.377683	138.9417	6.440733	139.807	6.381517	144.042	6.357283	149.7707	6.569683	140.2535
6.371333	145.2266	6.39435	138.9331	6.4574	139.8021	6.398183	144.0367	6.37395	149.7658	6.58635	140.249
6.388	145.2181	6.411017	138.926	6.474067	139.7906	6.41485	144.0321	6.390617	149.7684	6.603017	140.2416
6.404667	145.2144	6.427683	138.9139	6.490733	139.786	6.431517	144.0303	6.407283	149.7661	6.619683	140.2363
6.421333	145.211	6.44435	138.9055	6.5074	139.778	6.448183	144.0244	6.42395	149.7663	6.63635	140.2288
6.438	145.2038	6.461017	138.8959	6.524067	139.7721	6.46485	144.0204	6.440617	149.7633	6.653017	140.2239
6.454667	145.1963	6.477683	138.8866	6.540733	139.7636	6.481517	144.0181	6.457283	149.7639	6.669683	140.2191
6.471333	145.1901	6.49435	138.8786	6.5574	139.7572	6.498183	144.016	6.47395	149.764	6.68635	140.2094
6.488	145.1822	6.511017	138.8705	6.574067	139.7472	6.51485	144.0135	6.490617	149.7609	6.703017	140.2039
6.504667	145.1751	6.527683	138.8601	6.590733	139.7444	6.531517	144.0074	6.507283	149.7559	6.719683	140.1998
6.521333	145.1727	6.54435	138.8524	6.6074	139.7372	6.548183	144.0038	6.52395	149.7582	6.73635	140.1955
6.538	145.1638	6.561017	138.8453	6.624067	139.7333	6.56485	143.9955	6.540617	149.7598	6.753017	140.1893
6.554667	145.1534	6.577683	138.8369	6.640733	139.7228	6.581517	143.9934	6.557283	149.7547	6.769683	140.1825
6.571333	145.1517	6.59435	138.8251	6.6574	139.7193	6.598183	143.9925	6.57395	149.754	6.78635	140.1805
6.588	145.1435	6.611017	138.815	6.674067	139.7124	6.61485	143.9882	6.590617	149.753	6.803017	140.1747
6.604667	145.1358	6.627683	138.8061	6.690733	139.7059	6.631517	143.9842	6.607283	149.7525	6.819683	140.1698
6.621333	145.1322	6.64435	138.7978	6.7074	139.6987	6.648183	143.9819	6.62395	149.7519	6.83635	140.1622
6.638	145.1249	6.661017	138.7924	6.724067	139.6909	6.66485	143.9774	6.640617	149.7513	6.853017	140.1574
6.654667	145.1187	6.677683	138.7804	6.740733	139.6869	6.681517	143.9726	6.657283	149.7483	6.869683	140.1519
6.671333	145.1105	6.69435	138.778	6.7574	139.6793	6.698183	143.9694	6.67395	149.7486	6.88635	140.1452
6.688	145.1054	6.711017	138.7667	6.774067	139.6722	6.71485	143.9663	6.690617	149.747	6.903017	140.1394
6.704667	145.0972	6.727683	138.7566	6.790733	139.6661	6.731517	143.9637	6.707283	149.7484	6.919683	140.1365
6.721333	145.0951	6.74435	138.7438	6.8074	139.6593	6.748183	143.9606	6.72395	149.7438	6.93635	140.1289
6.738	145.086	6.761017	138.737	6.824067	139.6533	6.76485	143.9546	6.740617	149.743	6.953017	140.124
6.754667	145.0821	6.777683	138.7328	6.840733	139.6435	6.781517	143.9499	6.757283	149.7466	6.969683	140.1175
6.771333	145.0736	6.79435	138.7222	6.8574	139.6392	6.798183	143.9477	6.77395	149.7416	6.98635	140.1126
6.788	145.0667	6.811017	138.7119	6.874067	139.6316	6.81485	143.9434	6.790617	149.7421	7.003017	140.1053
6.804667	145.0627	6.827683	138.707	6.890733	139.6251	6.831517	143.9408	6.807283	149.7388	7.019683	140.1024
6.821333	145.0517	6.84435	138.6963	6.9074	139.6202	6.848183	143.9398	6.82395	149.7425	7.03635	140.0969
6.838	145.0459	6.861017	138.6871	6.924067	139.6109	6.86485	143.9362	6.840617	149.7367	7.053017	140.0912
6.854667	145.0383	6.877683	138.6777	6.940733	139.6038	6.881517	143.9316	6.857283	149.7371	7.069683	140.0833
6.871333	145.0356	6.89435	138.6725	6.9574	139.601	6.898183	143.9282	6.87395	149.7374	7.08635	140.0789

6.888	145.0298	6.911017	138.6652	6.974067	139.589	6.91485	143.927	6.890617	149.7391	7.103017	140.073
6.904667	145.021	6.927683	138.6573	6.990733	139.5843	6.931517	143.9248	6.907283	149.7342	7.119683	140.0678
6.921333	145.0143	6.94435	138.6471	7.0074	139.5794	6.948183	143.9207	6.92395	149.7326	7.13635	140.0631
6.938	145.0089	6.961017	138.6397	7.024067	139.5705	6.96485	143.914	6.940617	149.7323	7.153017	140.0579
6.954667	145.0039	6.977683	138.6301	7.040733	139.5632	6.981517	143.9142	6.957283	149.7305	7.169683	140.0501
6.971333	144.9914	6.99435	138.6186	7.0574	139.5585	6.998183	143.9093	6.97395	149.7302	7.18635	140.0442
6.988	144.9871	7.011017	138.6112	7.074067	139.5504	7.01485	143.9051	6.990617	149.7319	7.203017	140.0394
7.004667	144.9796	7.027683	138.6035	7.090733	139.5472	7.031517	143.9023	7.007283	149.7287	7.219683	140.0328
7.021333	144.9729	7.04435	138.5965	7.1074	139.5384	7.048183	143.9007	7.02395	149.7278	7.23635	140.0268
7.038	144.9638	7.061017	138.5871	7.124067	139.5299	7.06485	143.8975	7.040617	149.7263	7.253017	140.0227
7.054667	144.9595	7.077683	138.5795	7.140733	139.5262	7.081517	143.8914	7.057283	149.7253	7.269683	140.0167
7.071333	144.9509	7.09435	138.5729	7.1574	139.5176	7.098183	143.891	7.07395	149.7237	7.28635	140.009
7.088	144.9445	7.111017	138.5642	7.174067	139.5106	7.11485	143.8843	7.090617	149.7241	7.303017	140.0068
7.104667	144.9381	7.127683	138.5571	7.190733	139.5077	7.131517	143.8804	7.107283	149.7233	7.319683	139.9979
7.121333	144.9321	7.14435	138.5471	7.2074	139.4991	7.148183	143.8786	7.12395	149.7187	7.33635	139.996
7.138	144.9258	7.161017	138.5375	7.224067	139.4888	7.16485	143.8749	7.140617	149.7223	7.353017	139.9905
7.154667	144.9196	7.177683	138.5319	7.240733	139.4843	7.181517	143.8679	7.157283	149.7182	7.369683	139.9817
7.171333	144.9114	7.19435	138.5206	7.2574	139.4769	7.198183	143.8673	7.17395	149.7181	7.38635	139.9762
7.188	144.9064	7.211017	138.5135	7.274067	139.4686	7.21485	143.8645	7.190617	149.7169	7.403017	139.9719
7.204667	144.8957	7.227683	138.5053	7.290733	139.4599	7.231517	143.8588	7.207283	149.714	7.419683	139.967
7.221333	144.8922	7.24435	138.4999	7.3074	139.4628	7.248183	143.8546	7.22395	149.7126	7.43635	139.9603
7.238	144.8822	7.261017	138.4884	7.324067	139.4546	7.26485	143.8529	7.240617	149.7133	7.453017	139.9557
7.254667	144.8816	7.277683	138.4811	7.340733	139.4461	7.281517	143.8485	7.257283	149.7101	7.469683	139.9516
7.271333	144.8735	7.29435	138.4707	7.3574	139.4458	7.298183	143.8462	7.27395	149.7097	7.48635	139.9442
7.288	144.8629	7.311017	138.4635	7.374067	139.4327	7.31485	143.8445	7.290617	149.7094	7.503017	139.9414
7.304667	144.8605	7.327683	138.4549	7.390733	139.4247	7.331517	143.8388	7.307283	149.7045	7.519683	139.9332
7.321333	144.8548	7.34435	138.4468	7.4074	139.4212	7.348183	143.8372	7.32395	149.7069	7.53635	139.9333
7.338	144.8442	7.361017	138.4389	7.424067	139.4079	7.36485	143.8341	7.340617	149.704	7.553017	139.923
7.354667	144.8343	7.377683	138.4329	7.440733	139.405	7.381517	143.829	7.357283	149.7045	7.569683	139.9178
7.371333	144.8319	7.39435	138.4244	7.4574	139.4009	7.398183	143.829	7.37395	149.7022	7.58635	139.916
7.388	144.8241	7.411017	138.4146	7.474067	139.396	7.41485	143.8238	7.390617	149.6994	7.603017	139.9091
7.404667	144.8202	7.427683	138.4083	7.490733	139.3867	7.431517	143.8199	7.407283	149.6985	7.619683	139.9026
7.421333	144.8114	7.44435	138.402	7.5074	139.3793	7.448183	143.8169	7.42395	149.6986	7.63635	139.8982
7.438	144.8038	7.461017	138.3928	7.524067	139.375	7.46485	143.8153	7.440617	149.6978	7.653017	139.8955
7.454667	144.8013	7.477683	138.3844	7.540733	139.3661	7.481517	143.8099	7.457283	149.6943	7.669683	139.8903
7.471333	144.7941	7.49435	138.378	7.5574	139.3601	7.498183	143.807	7.47395	149.6956	7.68635	139.8857
7.488	144.7883	7.511017	138.3692	7.574067	139.356	7.51485	143.8045	7.490617	149.6948	7.703017	139.8807
7.504667	144.7818	7.527683	138.3628	7.590733	139.352	7.531517	143.8036	7.507283	149.6907	7.719683	139.874
7.521333	144.7747	7.54435	138.3524	7.6074	139.3434	7.548183	143.7978	7.52395	149.6911	7.73635	139.8712
7.538	144.7668	7.561017	138.3457	7.624067	139.3396	7.56485	143.7944	7.540617	149.6889	7.753017	139.8668
7.554667	144.7625	7.577683	138.3374	7.640733	139.3323	7.581517	143.7905	7.557283	149.6893	7.769683	139.862
7.571333	144.7582	7.59435	138.3296	7.6574	139.3254	7.598183	143.787	7.57395	149.6885	7.78635	139.8553
7.588	144.7537	7.611017	138.3231	7.674067	139.3196	7.61485	143.7842	7.590617	149.6854	7.803017	139.851
7.604667	144.745	7.627683	138.3151	7.690733	139.3109	7.631517	143.7811	7.607283	149.6837	7.819683	139.8474
7.621333	144.7424	7.64435	138.3084	7.7074	139.3121	7.648183	143.7772	7.62395	149.6847	7.83635	139.8421
7.638	144.7333	7.661017	138.2991	7.724067	139.3052	7.66485	143.7764	7.640617	149.684	7.853017	139.8368
7.654667	144.7254	7.677683	138.2912	7.740733	139.2962	7.681517	143.7715	7.657283	149.6818	7.869683	139.832
7.671333	144.7241	7.69435	138.2834	7.7574	139.2926	7.698183	143.7688	7.67395	149.6789	7.88635	139.8283
7.688	144.7141	7.711017	138.2767	7.774067	139.2874	7.71485	143.7676	7.690617	149.6799	7.903017	139.8236
7.704667	144.7102	7.727683	138.2677	7.790733	139.2789	7.731517	143.7676	7.707283	149.6803	7.919683	139.8173
7.721333	144.7038	7.74435	138.2597	7.8074	139.2769	7.748183	143.7622	7.72395	149.6768	7.93635	139.8131
7.738	144.6994	7.761017	138.2541	7.824067	139.2707	7.76485	143.7592	7.740617	149.6761	7.953017	139.8117
7.754667	144.6925	7.777683	138.2465	7.840733	139.2646	7.781517	143.756	7.757283	149.6753	7.969683	139.8031
7.771333	144.6887	7.79435	138.2363	7.8574	139.2567	7.798183	143.7542	7.77395	149.6751	7.98635	139.7971
7.788	144.6798	7.811017	138.2299	7.874067	139.2498	7.81485	143.7524	7.790617	149.6745	8.003017	139.793
7.804667	144.6723	7.827683	138.2231	7.890733	139.2435	7.831517	143.7492	7.807283	149.6727	8.019683	139.7897
7.821333	144.6673	7.84435	138.2195	7.9074	139.2399	7.848183	143.7439	7.82395	149.6693	8.03635	139.7835
7.838	144.6616	7.861017	138.21	7.924067	139.2326	7.86485	143.7442	7.840617	149.6717	8.053017	139.7787
7.854667	144.6533	7.877683	138.2022	7.940733	139.2286	7.881517	143.741	7.857283	149.6707	8.069683	139.7714
7.871333	144.649	7.89435	138.1911	7.9574	139.2217	7.898183	143.7391	7.87395	149.6664	8.08635	139.7661
7.888	144.6421	7.911017	138.1884	7.974067	139.2167	7.91485	143.7355	7.890617	149.6652	8.103017	139.7623
7.904667	144.6383	7.927683	138.1783	7.990733	139.2096	7.931517	143.7324	7.907283	149.663	8.119683	139.7561
7.921333	144.6293	7.94435	138.171	8.0074	139.2039	7.948183	143.7278	7.92395	149.6642	8.13635	139.7515
7.938	144.6259	7.961017	138.1637	8.024067	139.1967	7.96485	143.7264	7.940617	149.6621	8.153017	139.7447
7.954667	144.618	7.977683	138.1552	8.040733	139.1935	7.981517	143.7241	7.957283	149.6608	8.169683	139.7418
7.971333	144.6156	7.99435	138.1506	8.0574	139.1872	7.998183	143.7215	7.97395	149.6604	8.18635	139.737
7.988	144.6052	8.011017	138.1445	8.074067	139.1807	8.01485	143.7167	7.990617	149.6609	8.203017	139.7313
8.004667	144.6022	8.027683	138.1379	8.090733	139.1757	8.031517	143.7168	8.007283	149.6597	8.219683	139.7244
8.021333	144.598	8.04435	138.1279	8.1074	139.1694	8.048183	143.7138	8.02395	149.6564	8.23635	139.7226

8.038	144.5879	8.061017	138.1179	8.124067	139.1663	8.06485	143.7064	8.040617	149.6563	8.253017	139.7174
8.054667	144.5871	8.077683	138.1126	8.140733	139.1609	8.081517	143.7068	8.057283	149.6549	8.269683	139.7121
8.071333	144.5793	8.09435	138.1064	8.1574	139.1508	8.098183	143.7046	8.07395	149.6578	8.28635	139.7066
8.088	144.5684	8.111017	138.0992	8.174067	139.1483	8.11485	143.6984	8.090617	149.6539	8.303017	139.7012
8.104667	144.5641	8.127683	138.0909	8.190733	139.1434	8.131517	143.6954	8.107283	149.6525	8.319683	139.6976
8.121333	144.5606	8.14435	138.0854	8.2074	139.1383	8.148183	143.6931	8.12395	149.6528	8.33635	139.6914
8.138	144.5535	8.161017	138.075	8.224067	139.1326	8.16485	143.6881	8.140617	149.6506	8.353017	139.6878
8.154667	144.5442	8.177683	138.0678	8.240733	139.1296	8.181517	143.6851	8.157283	149.6491	8.369683	139.6806
8.171333	144.5435	8.19435	138.0632	8.2574	139.1202	8.198183	143.6834	8.17395	149.6466	8.38635	139.6761
8.188	144.5411	8.211017	138.0557	8.274067	139.1163	8.21485	143.6804	8.190617	149.6458	8.403017	139.673
8.204667	144.5306	8.227683	138.0498	8.290733	139.113	8.231517	143.6782	8.207283	149.6465	8.419683	139.6661
8.221333	144.5249	8.24435	138.041	8.3074	139.1065	8.248183	143.6737	8.22395	149.6446	8.43635	139.6616
8.238	144.5191	8.261017	138.0312	8.324067	139.1007	8.26485	143.6691	8.240617	149.6424	8.453017	139.6563
8.254667	144.5174	8.277683	138.0234	8.340733	139.0978	8.281517	143.6677	8.257283	149.6455	8.469683	139.6525
8.271333	144.5059	8.29435	138.0148	8.3574	139.0897	8.298183	143.6659	8.27395	149.6401	8.48635	139.6477
8.288	144.5053	8.311017	138.0086	8.374067	139.0825	8.31485	143.6625	8.290617	149.6403	8.503017	139.6451
8.304667	144.4963	8.327683	138	8.390733	139.0802	8.331517	143.6571	8.307283	149.6388	8.519683	139.6374
8.321333	144.4902	8.34435	137.9906	8.4074	139.0717	8.348183	143.6555	8.32395	149.6379	8.53635	139.6335
8.338	144.4877	8.361017	137.9868	8.424067	139.0658	8.36485	143.6521	8.340617	149.6374	8.553017	139.6301
8.354667	144.4787	8.377683	137.9815	8.440733	139.0619	8.381517	143.6488	8.357283	149.6373	8.569683	139.6239
8.371333	144.4752	8.39435	137.9746	8.4574	139.0536	8.398183	143.6456	8.37395	149.6359	8.58635	139.6201
8.388	144.4678	8.411017	137.9662	8.474067	139.0495	8.41485	143.6435	8.390617	149.6358	8.603017	139.615
8.404667	144.4647	8.427683	137.9597	8.490733	139.0444	8.431517	143.6396	8.407283	149.6332	8.619683	139.6092
8.421333	144.4587	8.44435	137.9537	8.5074	139.04	8.448183	143.6384	8.42395	149.6329	8.63635	139.609
8.438	144.4528	8.461017	137.9435	8.524067	139.0322	8.46485	143.6328	8.440617	149.6307	8.653017	139.6028
8.454667	144.4485	8.477683	137.9365	8.540733	139.0297	8.481517	143.6305	8.457283	149.6298	8.669683	139.602
8.471333	144.4388	8.49435	137.9321	8.5574	139.0226	8.498183	143.627	8.47395	149.6274	8.68635	139.5894
8.488	144.4373	8.511017	137.9253	8.574067	139.0185	8.51485	143.6236	8.490617	149.6263	8.703017	139.5888
8.504667	144.4264	8.527683	137.9179	8.590733	139.0126	8.531517	143.6199	8.507283	149.6252	8.719683	139.5846
8.521333	144.4229	8.54435	137.9104	8.6074	139.0091	8.548183	143.6162	8.52395	149.6268	8.73635	139.5834
8.538	144.4171	8.561017	137.9042	8.624067	139.001	8.56485	143.6147	8.540617	149.6255	8.753017	139.5767
8.554667	144.4116	8.577683	137.8946	8.640733	138.9954	8.581517	143.6103	8.557283	149.6226	8.769683	139.5707
8.571333	144.4049	8.59435	137.8915	8.6574	138.9866	8.598183	143.6063	8.57395	149.6208	8.78635	139.5675
8.588	144.4016	8.611017	137.8839	8.674067	138.9844	8.61485	143.6035	8.590617	149.6213	8.803017	139.5638
8.604667	144.3953	8.627683	137.8761	8.690733	138.9793	8.631517	143.6003	8.607283	149.6219	8.819683	139.5609
8.621333	144.3885	8.64435	137.8688	8.7074	138.9743	8.648183	143.5986	8.62395	149.6186	8.83635	139.5578
8.638	144.3826	8.661017	137.8628	8.724067	138.9641	8.66485	143.5956	8.640617	149.6192	8.853017	139.5528
8.654667	144.3766	8.677683	137.8521	8.740733	138.9589	8.681517	143.5912	8.657283	149.6165	8.869683	139.5478
8.671333	144.3718	8.69435	137.8476	8.7574	138.9526	8.698183	143.5874	8.67395	149.6178	8.88635	139.5469
8.688	144.3646	8.711017	137.8404	8.774067	138.9495	8.71485	143.5855	8.690617	149.616	8.903017	139.5385
8.704667	144.3589	8.727683	137.8311	8.790733	138.9442	8.731517	143.5826	8.707283	149.6122	8.919683	139.5355
8.721333	144.3558	8.74435	137.827	8.8074	138.9358	8.748183	143.5797	8.72395	149.6152	8.93635	139.5306
8.738	144.3473	8.761017	137.8184	8.824067	138.9309	8.76485	143.5762	8.740617	149.6102	8.953017	139.5261
8.754667	144.3432	8.777683	137.8125	8.840733	138.9271	8.781517	143.5732	8.757283	149.6082	8.969683	139.5238
8.771333	144.3366	8.79435	137.8073	8.8574	138.9214	8.798183	143.5688	8.77395	149.6072	8.98635	139.5166
8.788	144.3293	8.811017	137.7991	8.874067	138.9163	8.81485	143.5673	8.790617	149.6086	9.003017	139.5137
8.804667	144.3253	8.827683	137.7929	8.890733	138.9106	8.831517	143.565	8.807283	149.6081	9.019683	139.5087
8.821333	144.3189	8.84435	137.7853	8.9074	138.9022	8.848183	143.5594	8.82395	149.6058	9.03635	139.5036
8.838	144.3142	8.861017	137.777	8.924067	138.8979	8.86485	143.5592	8.840617	149.6043	9.053017	139.5009
8.854667	144.3099	8.877683	137.7713	8.940733	138.8929	8.881517	143.5546	8.857283	149.6021	9.069683	139.4939
8.871333	144.3047	8.89435	137.7632	8.9574	138.8884	8.898183	143.5523	8.87395	149.604	9.08635	139.4885
8.888	144.2971	8.911017	137.7574	8.974067	138.8838	8.91485	143.5493	8.890617	149.6017	9.103017	139.4861
8.904667	144.291	8.927683	137.7484	8.990733	138.88	8.931517	143.5484	8.907283	149.6002	9.119683	139.4839
8.921333	144.2877	8.94435	137.742	9.0074	138.8741	8.948183	143.5428	8.92395	149.5995	9.13635	139.4772
8.938	144.2784	8.961017	137.7351	9.024067	138.8698	8.96485	143.5395	8.940617	149.5995	9.153017	139.472
8.954667	144.2767	8.977683	137.7295	9.040733	138.863	8.981517	143.5391	8.957283	149.5989	9.169683	139.4687
8.971333	144.267	8.99435	137.7214	9.0574	138.8622	8.998183	143.5304	8.97395	149.5981	9.18635	139.4602
8.988	144.2638	9.011017	137.7164	9.074067	138.8518	9.01485	143.5301	8.990617	149.5957	9.203017	139.4589
9.004667	144.2584	9.027683	137.7085	9.090733	138.846	9.031517	143.529	9.007283	149.5969	9.219683	139.4524
9.021333	144.2502	9.04435	137.7005	9.1074	138.8449	9.048183	143.5278	9.02395	149.5944	9.23635	139.449
9.038	144.245	9.061017	137.6937	9.124067	138.8358	9.06485	143.522	9.040617	149.594	9.253017	139.4432
9.054667	144.2427	9.077683	137.6893	9.140733	138.8327	9.081517	143.5171	9.057283	149.5892	9.269683	139.4372
9.071333	144.2375	9.09435	137.6818	9.1574	138.8297	9.098183	143.5159	9.07395	149.5918	9.28635	139.4328
9.088	144.2281	9.111017	137.6778	9.174067	138.8228	9.11485	143.5155	9.090617	149.5928	9.303017	139.4256
9.104667	144.2243	9.127683	137.671	9.190733	138.8148	9.131517	143.5106	9.107283	149.5883	9.319683	139.4245
9.121333	144.2197	9.14435	137.6652	9.2074	138.8138	9.148183	143.5107	9.12395	149.5888	9.33635	139.4174
9.138	144.218	9.161017	137.6589	9.224067	138.806	9.16485	143.5047	9.140617	149.5855	9.353017	139.4139
9.154667	144.2073	9.177683	137.6518	9.240733	138.802	9.181517	143.5018	9.157283	149.584	9.369683	139.4062
9.171333	144.2055	9.19435	137.6447	9.2574	138.7975	9.198183	143.5021	9.17395	149.5851	9.38635	139.4028

9.188	144.1979	9.211017	137.6396	9.274067	138.7928	9.21485	143.4966	9.190617	149.5849	9.403017	139.3982
9.204667	144.193	9.227683	137.6354	9.290733	138.7876	9.231517	143.4934	9.207283	149.5818	9.419683	139.3937
9.221333	144.188	9.24435	137.6278	9.3074	138.7801	9.248183	143.4931	9.22395	149.582	9.43635	139.3883
9.238	144.1799	9.261017	137.6178	9.324067	138.7775	9.26485	143.4893	9.240617	149.5808	9.453017	139.3846
9.254667	144.1786	9.277683	137.6148	9.340733	138.7695	9.281517	143.4872	9.257283	149.5811	9.469683	139.384
9.271333	144.1718	9.29435	137.6033	9.3574	138.7652	9.298183	143.4852	9.27395	149.5778	9.48635	139.3791
9.288	144.1644	9.311017	137.5995	9.374067	138.7625	9.31485	143.4834	9.290617	149.576	9.503017	139.373
9.304667	144.16	9.327683	137.5954	9.390733	138.7545	9.331517	143.4815	9.307283	149.5764	9.519683	139.3694
9.321333	144.1567	9.34435	137.5851	9.4074	138.7526	9.348183	143.4796	9.32395	149.575	9.53635	139.3664
9.338	144.1499	9.361017	137.5771	9.424067	138.7421	9.36485	143.4767	9.340617	149.5727	9.553017	139.3596
9.354667	144.1447	9.377683	137.5734	9.440733	138.7389	9.381517	143.4732	9.357283	149.5725	9.569683	139.3566
9.371333	144.1394	9.39435	137.569	9.4574	138.7326	9.398183	143.4715	9.37395	149.5702	9.58635	139.3527
9.388	144.1317	9.411017	137.5631	9.474067	138.731	9.41485	143.4682	9.390617	149.5697	9.603017	139.3477
9.404667	144.1286	9.427683	137.5575	9.490733	138.7249	9.431517	143.4667	9.407283	149.5689	9.619683	139.3442
9.421333	144.1244	9.44435	137.5496	9.5074	138.719	9.448183	143.4615	9.42395	149.5697	9.63635	139.339
9.438	144.1168	9.461017	137.5432	9.524067	138.7139	9.46485	143.4617	9.440617	149.5679	9.653017	139.3356
9.454667	144.1138	9.477683	137.5371	9.540733	138.7083	9.481517	143.4574	9.457283	149.5672	9.669683	139.3279
9.471333	144.1046	9.49435	137.5292	9.5574	138.6995	9.498183	143.4544	9.47395	149.5671	9.68635	139.3279
9.488	144.1002	9.511017	137.5249	9.574067	138.7004	9.51485	143.4507	9.490617	149.5663	9.703017	139.3215
9.504667	144.0945	9.527683	137.5154	9.590733	138.6934	9.531517	143.4486	9.507283	149.5653	9.719683	139.3187
9.521333	144.0894	9.54435	137.5103	9.6074	138.6875	9.548183	143.4458	9.52395	149.5614	9.73635	139.3144
9.538	144.0832	9.561017	137.5026	9.624067	138.6851	9.56485	143.443	9.540617	149.5606	9.753017	139.3122
9.554667	144.0789	9.577683	137.4995	9.640733	138.6812	9.581517	143.4398	9.557283	149.5599	9.769683	139.3061
9.571333	144.072	9.59435	137.491	9.6574	138.6768	9.598183	143.4386	9.57395	149.5593	9.78635	139.3038
9.588	144.0677	9.611017	137.4845	9.674067	138.6659	9.61485	143.4336	9.590617	149.5561	9.803017	139.2982
9.604667	144.0615	9.627683	137.4821	9.690733	138.6624	9.631517	143.4331	9.607283	149.5572	9.819683	139.2898
9.621333	144.0554	9.64435	137.4735	9.7074	138.6583	9.648183	143.4292	9.62395	149.5582	9.83635	139.288
9.638	144.0485	9.661017	137.4638	9.724067	138.6508	9.66485	143.4277	9.640617	149.5567	9.853017	139.2888
9.654667	144.0473	9.677683	137.4612	9.740733	138.6486	9.681517	143.4255	9.657283	149.5564	9.869683	139.2833
9.671333	144.0408	9.69435	137.4555	9.7574	138.6434	9.698183	143.4194	9.67395	149.5538	9.88635	139.2795
9.688	144.0346	9.711017	137.4492	9.774067	138.6368	9.71485	143.4171	9.690617	149.5541	9.903017	139.2736
9.704667	144.0343	9.727683	137.441	9.790733	138.6335	9.731517	143.4166	9.707283	149.551	9.919683	139.2727
9.721333	144.0233	9.74435	137.4342	9.8074	138.6285	9.748183	143.4125	9.72395	149.5534	9.93635	139.2666
9.738	144.0201	9.761017	137.4289	9.824067	138.6241	9.76485	143.4123	9.740617	149.5512	9.953017	139.2623
9.754667	144.0184	9.777683	137.4242	9.840733	138.6191	9.781517	143.407	9.757283	149.5494	9.969683	139.2555
9.771333	144.0085	9.79435	137.4176	9.8574	138.6157	9.798183	143.4047	9.77395	149.5497	9.98635	139.2523
9.788	144.0053	9.811017	137.4091	9.874067	138.6085	9.81485	143.4001	9.790617	149.5464	10.00302	139.2531
9.804667	143.9995	9.827683	137.4035	9.890733	138.5993	9.831517	143.4018	9.807283	149.5469	10.01968	139.2443
9.821333	143.9954	9.84435	137.4016	9.9074	138.5989	9.848183	143.3974	9.82395	149.5451	10.03635	139.2415
9.838	143.9911	9.861017	137.3928	9.924067	138.5912	9.86485	143.3927	9.840617	149.5427	10.05302	139.2353
9.854667	143.9805	9.877683	137.3877	9.940733	138.5856	9.881517	143.3886	9.857283	149.5434	10.06968	139.2295
9.871333	143.979	9.89435	137.3815	9.9574	138.5849	9.898183	143.389	9.87395	149.5404	10.08635	139.2285
9.888	143.9774	9.911017	137.3734	9.974067	138.5767	9.91485	143.3856	9.890617	149.539	10.10302	139.2295
9.904667	143.9728	9.927683	137.3688	9.990733	138.5685	9.931517	143.384	9.907283	149.5393	10.11968	139.2158
9.921333	143.9643	9.94435	137.3619	10.0074	138.5646	9.948183	143.3794	9.92395	149.5374	10.13635	139.2191
9.938	143.957	9.961017	137.3579	10.02407	138.5606	9.96485	143.3775	9.940617	149.5348	10.15302	139.2125
9.954667	143.9581	9.977683	137.3512	10.04073	138.5603	9.981517	143.376	9.957283	149.5361	10.16968	139.2091
9.971333	143.9512	9.99435	137.3439	10.0574	138.5512	9.998183	143.3768	9.97395	149.5344	10.18635	139.2054
9.988	143.9388	10.01102	137.3387	10.07407	138.5471	10.01485	143.3713	9.990617	149.5326	10.20302	139.2037
10.00467	143.9397	10.02768	137.3316	10.09073	138.5448	10.03152	143.3698	10.00728	149.5333	10.21968	139.1959
10.02133	143.9347	10.04435	137.326	10.1074	138.5397	10.04818	143.3672	10.02395	149.532	10.23635	139.1952
10.038	143.9314	10.06102	137.3223	10.12407	138.5317	10.06485	143.3618	10.04062	149.5321	10.25302	139.1906
10.05467	143.9259	10.07768	137.3142	10.14073	138.5277	10.08152	143.3599	10.05728	149.529	10.26968	139.1882
10.07133	143.9202	10.09435	137.3083	10.1574	138.5225	10.09818	143.3589	10.07395	149.5286	10.28635	139.1846
10.088	143.9145	10.11102	137.3042	10.17407	138.519	10.11485	143.3558	10.09062	149.5287	10.30302	139.1777
10.10467	143.9123	10.12768	137.2957	10.19073	138.5135	10.13152	143.3554	10.10728	149.5314	10.31968	139.1766
10.12133	143.9066	10.14435	137.2932	10.2074	138.5056	10.14818	143.3523	10.12395	149.5256	10.33635	139.1734
10.138	143.9012	10.16102	137.2863	10.22407	138.5022	10.16485	143.3495	10.14062	149.5268	10.35302	139.1681
10.15467	143.8958	10.17768	137.2806	10.24073	138.4991	10.18152	143.348	10.15728	149.5255	10.36968	139.1686
10.17133	143.8927	10.19435	137.2759	10.2574	138.4934	10.19818	143.347	10.17395	149.5247	10.38635	139.1633
10.188	143.8874	10.21102	137.2679	10.27407	138.4869	10.21485	143.3431	10.19062	149.5217	10.40302	139.1596
10.20467	143.8827	10.22768	137.263	10.29073	138.4828	10.23152	143.3434	10.20728	149.5209	10.41968	139.1545
10.22133	143.8769	10.24435	137.2557	10.3074	138.4779	10.24818	143.3371	10.22395	149.5229	10.43635	139.1521
10.238	143.8722	10.26102	137.2494	10.32407	138.4736	10.26485	143.3363	10.24062	149.5234	10.45302	139.148
10.25467	143.8651	10.27768	137.2457	10.34073	138.4679	10.28152	143.3345	10.25728	149.5185	10.46968	139.1403
10.27133	143.8612	10.29435	137.24	10.3574	138.4633	10.29818	143.3332	10.27395	149.5175	10.48635	139.1384
10.288	143.8544	10.31102	137.2339	10.37407	138.4605	10.31485	143.3318	10.29062	149.5222	10.50302	139.1361
10.30467	143.8529	10.32768	137.2252	10.39073	138.4535	10.33152	143.3293	10.30728	149.5184	10.51968	139.1336
10.32133	143.8451	10.34435	137.2226	10.4074	138.4498	10.34818	143.3247	10.32395	149.5122	10.53635	139.1298

10.338	143.8391	10.36102	137.2145	10.42407	138.4424	10.36485	143.3249	10.34062	149.5166	10.55302	139.1228
10.35467	143.8352	10.37768	137.2081	10.44073	138.4398	10.38152	143.3222	10.35728	149.5179	10.56968	139.1213
10.37133	143.8302	10.39435	137.2014	10.4574	138.4357	10.39818	143.32	10.37395	149.5159	10.58635	139.1163
10.388	143.8241	10.41102	137.1966	10.47407	138.4296	10.41485	143.3178	10.39062	149.5151	10.60302	139.1113
10.40467	143.819	10.42768	137.1921	10.49073	138.4259	10.43152	143.3122	10.40728	149.5091	10.61968	139.106
10.42133	143.8153	10.44435	137.187	10.5074	138.4222	10.44818	143.3126	10.42395	149.5101	10.63635	139.1016
10.438	143.8087	10.46102	137.1812	10.52407	138.4175	10.46485	143.3117	10.44062	149.511	10.65302	139.0962
10.45467	143.8051	10.47768	137.1767	10.54073	138.4156	10.48152	143.3057	10.45728	149.5086	10.66968	139.0931
10.47133	143.7999	10.49435	137.1683	10.5574	138.4087	10.49818	143.3071	10.47395	149.5084	10.68635	139.0909
10.488	143.7934	10.51102	137.16	10.57407	138.4072	10.51485	143.3021	10.49062	149.5074	10.70302	139.0845
10.50467	143.7891	10.52768	137.1566	10.59073	138.4003	10.53152	143.3007	10.50728	149.5012	10.71968	139.0805
10.52133	143.7827	10.54435	137.1496	10.6074	138.3926	10.54818	143.2983	10.52395	149.5033	10.73635	139.0771
10.538	143.7777	10.56102	137.1434	10.62407	138.3901	10.56485	143.2964	10.54062	149.5033	10.75302	139.0724
10.55467	143.7718	10.57768	137.1385	10.64073	138.3858	10.58152	143.2928	10.55728	149.5005	10.76968	139.0668
10.57133	143.7732	10.59435	137.1337	10.6574	138.3808	10.59818	143.2918	10.57395	149.4998	10.78635	139.0654
10.588	143.7635	10.61102	137.1277	10.67407	138.3775	10.61485	143.289	10.59062	149.4954	10.80302	139.0578
10.60467	143.7585	10.62768	137.1228	10.69073	138.3714	10.63152	143.2874	10.60728	149.4996	10.81968	139.0521
10.62133	143.7546	10.64435	137.1158	10.7074	138.3651	10.64818	143.285	10.62395	149.4991	10.83635	139.0524
10.638	143.7473	10.66102	137.109	10.72407	138.3598	10.66485	143.2803	10.64062	149.4945	10.85302	139.0479
10.65467	143.744	10.67768	137.1053	10.74073	138.3584	10.68152	143.2786	10.65728	149.4948	10.86968	139.0443
10.67133	143.7382	10.69435	137.0997	10.7574	138.3534	10.69818	143.2763	10.67395	149.4954	10.88635	139.0389
10.688	143.7349	10.71102	137.095	10.77407	138.3482	10.71485	143.2753	10.69062	149.4884	10.90302	139.0376
10.70467	143.7288	10.72768	137.0858	10.79073	138.3429	10.73152	143.2707	10.70728	149.4895	10.91968	139.0307
10.72133	143.7229	10.74435	137.0827	10.8074	138.3387	10.74818	143.2691	10.72395	149.4892	10.93635	139.0301
10.738	143.7174	10.76102	137.0788	10.82407	138.334	10.76485	143.2672	10.74062	149.4867	10.95302	139.0245
10.75467	143.713	10.77768	137.0729	10.84073	138.3267	10.78152	143.2649	10.75728	149.4873	10.96968	139.02
10.77133	143.7082	10.79435	137.0656	10.8574	138.3238	10.79818	143.2629	10.77395	149.4853	10.98635	139.0158
10.788	143.702	10.81102	137.0586	10.87407	138.3184	10.81485	143.2605	10.79062	149.4862	11.00302	139.0128
10.80467	143.6983	10.82768	137.0536	10.89073	138.3168	10.83152	143.2581	10.80728	149.4827	11.01968	139.0066
10.82133	143.6938	10.84435	137.0489	10.9074	138.3107	10.84818	143.2562	10.82395	149.4826	11.03635	139.0036
10.838	143.6913	10.86102	137.042	10.92407	138.305	10.86485	143.2545	10.84062	149.4794	11.05302	138.9976
10.85467	143.6858	10.87768	137.0369	10.94073	138.2988	10.88152	143.2501	10.85728	149.4799	11.06968	138.9936
10.87133	143.6786	10.89435	137.0329	10.9574	138.2972	10.89818	143.2481	10.87395	149.4766	11.08635	138.9902
10.888	143.6755	10.91102	137.0287	10.97407	138.2922	10.91485	143.2448	10.89062	149.4783	11.10302	138.9878
10.90467	143.6692	10.92768	137.0182	10.99073	138.2884	10.93152	143.2439	10.90728	149.4736	11.11968	138.9837
10.92133	143.6662	10.94435	137.0162	11.0074	138.2848	10.94818	143.2406	10.92395	149.474	11.13635	138.9784
10.938	143.6621	10.96102	137.0084	11.02407	138.2835	10.96485	143.2397	10.94062	149.4748	11.15302	138.9756
10.95467	143.6586	10.97768	137.004	11.04073	138.2751	10.98152	143.2381	10.95728	149.4724	11.16968	138.9732
10.97133	143.6528	10.99435	136.9981	11.0574	138.2703	10.99818	143.2352	10.97395	149.474	11.18635	138.9705
10.988	143.6477	11.01102	136.9925	11.07407	138.2662	11.01485	143.2299	10.99062	149.4733	11.20302	138.9657
11.00467	143.6413	11.02768	136.9877	11.09073	138.2602	11.03152	143.2284	11.00728	149.4681	11.21968	138.9597
11.02133	143.6366	11.04435	136.9829	11.1074	138.2581	11.04818	143.2279	11.02395	149.4687	11.23635	138.9583
11.038	143.6295	11.06102	136.9745	11.12407	138.2523	11.06485	143.2252	11.04062	149.4681	11.25302	138.9549
11.05467	143.625	11.07768	136.9701	11.14073	138.2504	11.08152	143.2219	11.05728	149.4658	11.26968	138.9506
11.07133	143.6212	11.09435	136.9644	11.1574	138.2438	11.09818	143.2206	11.07395	149.467	11.28635	138.9451
11.088	143.616	11.11102	136.9618	11.17407	138.2373	11.11485	143.215	11.09062	149.4666	11.30302	138.9432
11.10467	143.6084	11.12768	136.9552	11.19073	138.2359	11.13152	143.2159	11.10728	149.4638	11.31968	138.9394
11.12133	143.6058	11.14435	136.9465	11.2074	138.2287	11.14818	143.2166	11.12395	149.4629	11.33635	138.938
11.138	143.6	11.16102	136.9443	11.22407	138.226	11.16485	143.2127	11.14062	149.4631	11.35302	138.932
11.15467	143.5946	11.17768	136.9404	11.24073	138.2214	11.18152	143.2102	11.15728	149.4611	11.36968	138.927
11.17133	143.5887	11.19435	136.9315	11.2574	138.2147	11.19818	143.2091	11.17395	149.46	11.38635	138.9232
11.188	143.5852	11.21102	136.928	11.27407	138.2095	11.21485	143.2071	11.19062	149.4612	11.40302	138.9197
11.20467	143.5786	11.22768	136.9221	11.29073	138.2073	11.23152	143.2062	11.20728	149.4561	11.41968	138.9187
11.22133	143.5795	11.24435	136.918	11.3074	138.2006	11.24818	143.2038	11.22395	149.4536	11.43635	138.9154
11.238	143.5713	11.26102	136.9144	11.32407	138.1984	11.26485	143.2009	11.24062	149.4563	11.45302	138.9098
11.25467	143.5666	11.27768	136.9053	11.34073	138.1911	11.28152	143.1995	11.25728	149.4561	11.46968	138.9092
11.27133	143.5642	11.29435	136.9013	11.3574	138.1896	11.29818	143.1971	11.27395	149.455	11.48635	138.9029
11.288	143.5588	11.31102	136.8977	11.37407	138.1866	11.31485	143.1977	11.29062	149.4507	11.50302	138.8995
11.30467	143.5507	11.32768	136.8927	11.39073	138.18	11.33152	143.193	11.30728	149.4523	11.51968	138.8942
11.32133	143.5476	11.34435	136.8858	11.4074	138.1738	11.34818	143.1926	11.32395	149.4504	11.53635	138.892
11.338	143.5434	11.36102	136.8805	11.42407	138.1714	11.36485	143.1892	11.34062	149.4478	11.55302	138.8867
11.35467	143.5392	11.37768	136.8741	11.44073	138.1681	11.38152	143.189	11.35728	149.4494	11.56968	138.8818
11.37133	143.5372	11.39435	136.8703	11.4574	138.1633	11.39818	143.1843	11.37395	149.4475	11.58635	138.876
11.388	143.5315	11.41102	136.8668	11.47407	138.1573	11.41485	143.1824	11.39062	149.4469	11.60302	138.8745
11.40467	143.5252	11.42768	136.8597	11.49073	138.1593	11.43152	143.1809	11.40728	149.4466	11.61968	138.8688
11.42133	143.5189	11.44435	136.8547	11.5074	138.1518	11.44818	143.1788	11.42395	149.4452	11.63635	138.8715
11.438	143.5163	11.46102	136.8491	11.52407	138.146	11.46485	143.1793	11.44062	149.4419	11.65302	138.8661
11.45467	143.51	11.47768	136.843	11.54073	138.1439	11.48152	143.1732	11.45728	149.4442	11.66968	138.8652
11.47133	143.5066	11.49435	136.835	11.5574	138.1382	11.49818	143.1719	11.47395	149.4435	11.68635	138.8601

11.488	143.4979	11.51102	136.8304	11.57407	138.1336	11.51485	143.1687	11.49062	149.4388	11.70302	138.8575
11.50467	143.4934	11.52768	136.8273	11.59073	138.1294	11.53152	143.169	11.50728	149.4405	11.71968	138.8538
11.52133	143.4878	11.54435	136.8228	11.6074	138.1291	11.54818	143.1643	11.52395	149.4391	11.73635	138.8515
11.538	143.4802	11.56102	136.8171	11.62407	138.1223	11.56485	143.1614	11.54062	149.4379	11.75302	138.8454
11.55467	143.4799	11.57768	136.8151	11.64073	138.12	11.58152	143.1626	11.55728	149.439	11.76968	138.8432
11.57133	143.475	11.59435	136.8065	11.6574	138.1161	11.59818	143.1587	11.57395	149.4374	11.78635	138.8394
11.588	143.4685	11.61102	136.7999	11.67407	138.1142	11.61485	143.1569	11.59062	149.4336	11.80302	138.8336
11.60467	143.4638	11.62768	136.795	11.69073	138.1041	11.63152	143.1544	11.60728	149.4334	11.81968	138.8316
11.62133	143.4589	11.64435	136.7923	11.7074	138.1045	11.64818	143.1523	11.62395	149.4341	11.83635	138.8289
11.638	143.4544	11.66102	136.7854	11.72407	138.0994	11.66485	143.1502	11.64062	149.4314	11.85302	138.8249
11.65467	143.4521	11.67768	136.7809	11.74073	138.0934	11.68152	143.1473	11.65728	149.4309	11.86968	138.8237
11.67133	143.4479	11.69435	136.7745	11.7574	138.0935	11.69818	143.1415	11.67395	149.43	11.88635	138.8172
11.688	143.4414	11.71102	136.7677	11.77407	138.0862	11.71485	143.1411	11.69062	149.4277	11.90302	138.814
11.70467	143.4373	11.72768	136.7628	11.79073	138.083	11.73152	143.1382	11.70728	149.427	11.91968	138.8162
11.72133	143.4338	11.74435	136.7581	11.8074	138.0777	11.74818	143.1358	11.72395	149.4313	11.93635	138.8089
11.738	143.43	11.76102	136.7498	11.82407	138.076	11.76485	143.1339	11.74062	149.4247	11.95302	138.809
11.75467	143.4274	11.77768	136.7436	11.84073	138.0718	11.78152	143.1317	11.75728	149.4245	11.96968	138.8055
11.77133	143.4207	11.79435	136.7394	11.8574	138.0676	11.79818	143.1304	11.77395	149.424	11.98635	138.8008
11.788	143.4156	11.81102	136.7366	11.87407	138.0628	11.81485	143.1285	11.79062	149.4246	12.00302	138.7986
11.80467	143.4092	11.82768	136.7315	11.89073	138.0562	11.83152	143.1256	11.80728	149.4256	12.01968	138.796
11.82133	143.4067	11.84435	136.727	11.9074	138.0509	11.84818	143.1252	11.82395	149.4232	12.03635	138.7928
11.838	143.3983	11.86102	136.717	11.92407	138.0479	11.86485	143.1232	11.84062	149.4206	12.05302	138.7875
11.85467	143.3964	11.87768	136.7127	11.94073	138.0452	11.88152	143.1173	11.85728	149.4207	12.06968	138.7862
11.87133	143.3904	11.89435	136.7095	11.9574	138.0404	11.89818	143.1158	11.87395	149.4197	12.08635	138.7807
11.888	143.3855	11.91102	136.7055	11.97407	138.0359	11.91485	143.1168	11.89062	149.4192	12.10302	138.7812
11.90467	143.3837	11.92768	136.6961	11.99073	138.0311	11.93152	143.1136	11.90728	149.4208	12.11968	138.7759
11.92133	143.3767	11.94435	136.6943	12.0074	138.0238	11.94818	143.1094	11.92395	149.418	12.13635	138.7669
11.938	143.3715	11.96102	136.6891	12.02407	138.0221	11.96485	143.1101	11.94062	149.417	12.15302	138.7693
11.95467	143.3701	11.97768	136.681	12.04073	138.018	11.98152	143.1034	11.95728	149.4192	12.16968	138.7659
11.97133	143.3631	11.99435	136.6823	12.0574	138.0129	11.99818	143.102	11.97395	149.4153	12.18635	138.7579
11.988	143.359	12.01102	136.6747	12.07407	138.0092	12.01485	143.1026	11.99062	149.4157	12.20302	138.757
12.00467	143.3543	12.02768	136.667	12.09073	138.0058	12.03152	143.0995	12.00728	149.4178	12.21968	138.7572
12.02133	143.3502	12.04435	136.6658	12.1074	137.9989	12.04818	143.0959	12.02395	149.4125	12.23635	138.7553
12.038	143.3485	12.06102	136.6581	12.12407	137.9989	12.06485	143.0946	12.04062	149.4131	12.25302	138.7508
12.05467	143.3438	12.07768	136.651	12.14073	137.9922	12.08152	143.0911	12.05728	149.4118	12.26968	138.7493
12.07133	143.3359	12.09435	136.6453	12.1574	137.9879	12.09818	143.0884	12.07395	149.4132	12.28635	138.7484
12.088	143.3307	12.11102	136.6452	12.17407	137.9851	12.11485	143.0854	12.09062	149.4127	12.30302	138.743
12.10467	143.327	12.12768	136.6379	12.19073	137.978	12.13152	143.0875	12.10728	149.4118	12.31968	138.7426
12.12133	143.3251	12.14435	136.6337	12.2074	137.9765	12.14818	143.0861	12.12395	149.4108	12.33635	138.7364
12.138	143.3172	12.16102	136.6294	12.22407	137.9714	12.16485	143.0849	12.14062	149.4107	12.35302	138.7335
12.15467	143.3128	12.17768	136.6236	12.24073	137.9679	12.18152	143.082	12.15728	149.4092	12.36968	138.7316
12.17133	143.3064	12.19435	136.619	12.2574	137.9644	12.19818	143.077	12.17395	149.4092	12.38635	138.7263
12.188	143.3054	12.21102	136.614	12.27407	137.9601	12.21485	143.075	12.19062	149.4051	12.40302	138.725
12.20467	143.2992	12.22768	136.61	12.29073	137.9545	12.23152	143.0745	12.20728	149.4066	12.41968	138.7235
12.22133	143.2954	12.24435	136.603	12.3074	137.9535	12.24818	143.0722	12.22395	149.4052	12.43635	138.7186
12.238	143.2898	12.26102	136.599	12.32407	137.9507	12.26485	143.0705	12.24062	149.4066	12.45302	138.7169
12.25467	143.287	12.27768	136.5956	12.34073	137.9434	12.28152	143.0691	12.25728	149.4027	12.46968	138.7143
12.27133	143.2797	12.29435	136.5893	12.3574	137.9402	12.29818	143.0652	12.27395	149.3999	12.48635	138.7086
12.288	143.2784	12.31102	136.5853	12.37407	137.9378	12.31485	143.0642	12.29062	149.4035	12.50302	138.7058
12.30467	143.272	12.32768	136.5792	12.39073	137.9315	12.33152	143.0592	12.30728	149.3988	12.51968	138.7013
12.32133	143.2662	12.34435	136.5761	12.4074	137.9289	12.34818	143.0589	12.32395	149.4015	12.53635	138.702
12.338	143.2618	12.36102	136.5702	12.42407	137.9252	12.36485	143.0577	12.34062	149.3982	12.55302	138.6985
12.35467	143.2594	12.37768	136.5659	12.44073	137.9214	12.38152	143.0546	12.35728	149.397	12.56968	138.6954
12.37133	143.2545	12.39435	136.5609	12.4574	137.9175	12.39818	143.0527	12.37395	149.3999	12.58635	138.69
12.388	143.2486	12.41102	136.5583	12.47407	137.9133	12.41485	143.0506	12.39062	149.3955	12.60302	138.6885
12.40467	143.2468	12.42768	136.5515	12.49073	137.9099	12.43152	143.0498	12.40728	149.3945	12.61968	138.6847
12.42133	143.2404	12.44435	136.5434	12.5074	137.9066	12.44818	143.0454	12.42395	149.3932	12.63635	138.6813
12.438	143.234	12.46102	136.5379	12.52407	137.9014	12.46485	143.0427	12.44062	149.3927	12.65302	138.6781
12.45467	143.2305	12.47768	136.5379	12.54073	137.8953	12.48152	143.045	12.45728	149.3912	12.66968	138.675
12.47133	143.2275	12.49435	136.5309	12.5574	137.8921	12.49818	143.0394	12.47395	149.3921	12.68635	138.6735
12.488	143.2218	12.51102	136.522	12.57407	137.8888	12.51485	143.0367	12.49062	149.3917	12.70302	138.6692
12.50467	143.2161	12.52768	136.5213	12.59073	137.883	12.53152	143.0358	12.50728	149.3874	12.71968	138.6671
12.52133	143.2116	12.54435	136.5107	12.6074	137.8797	12.54818	143.0348	12.52395	149.3855	12.73635	138.6621
12.538	143.206	12.56102	136.511	12.62407	137.8757	12.56485	143.0303	12.54062	149.3872	12.75302	138.6612
12.55467	143.2038	12.57768	136.5081	12.64073	137.8705	12.58152	143.0302	12.55728	149.3843	12.76968	138.6576
12.57133	143.1988	12.59435	136.5028	12.6574	137.8679	12.59818	143.0294	12.57395	149.3855	12.78635	138.6534
12.588	143.1945	12.61102	136.4964	12.67407	137.8642	12.61485	143.0224	12.59062	149.3842	12.80302	138.6491
12.60467	143.1906	12.62768	136.4934	12.69073	137.8627	12.63152	143.0211	12.60728	149.3815	12.81968	138.6469
12.62133	143.1888	12.64435	136.4856	12.7074	137.8591	12.64818	143.0222	12.62395	149.3813	12.83635	138.6473

12.638	143.184	12.66102	136.4828	12.72407	137.854	12.66485	143.0172	12.64062	149.3807	12.85302	138.6403
12.65467	143.1787	12.67768	136.4764	12.74073	137.8486	12.68152	143.021	12.65728	149.3768	12.86968	138.636
12.67133	143.1743	12.69435	136.4718	12.7574	137.8471	12.69818	143.0166	12.67395	149.3805	12.88635	138.6342
12.688	143.1699	12.71102	136.4667	12.77407	137.8424	12.71485	143.013	12.69062	149.3772	12.90302	138.6325
12.70467	143.1683	12.72768	136.4604	12.79073	137.8365	12.73152	143.0137	12.70728	149.3741	12.91968	138.6289
12.72133	143.1586	12.74435	136.4534	12.8074	137.8333	12.74818	143.0094	12.72395	149.3757	12.93635	138.6258
12.738	143.1583	12.76102	136.4496	12.82407	137.8301	12.76485	143.0073	12.74062	149.3767	12.95302	138.6216
12.75467	143.1524	12.77768	136.4485	12.84073	137.8246	12.78152	143.0058	12.75728	149.3723	12.96968	138.6152
12.77133	143.1452	12.79435	136.4412	12.8574	137.8227	12.79818	143.0023	12.77395	149.374	12.98635	138.6156
12.788	143.1435	12.81102	136.4367	12.87407	137.8163	12.81485	143.0012	12.79062	149.3702	13.00302	138.6115
12.80467	143.1385	12.82768	136.4338	12.89073	137.8151	12.83152	142.998	12.80728	149.3681	13.01968	138.6086
12.82133	143.1362	12.84435	136.4263	12.9074	137.8081	12.84818	142.9961	12.82395	149.3707	13.03635	138.6041
12.838	143.1307	12.86102	136.4246	12.92407	137.8043	12.86485	142.9947	12.84062	149.3688	13.05302	138.6018
12.85467	143.1279	12.87768	136.4211	12.94073	137.804	12.88152	142.9909	12.85728	149.3655	13.06968	138.6013
12.87133	143.1221	12.89435	136.4174	12.9574	137.7996	12.89818	142.9929	12.87395	149.3663	13.08635	138.5963
12.888	143.1211	12.91102	136.4108	12.97407	137.7983	12.91485	142.9897	12.89062	149.3651	13.10302	138.5961
12.90467	143.1124	12.92768	136.4072	12.99073	137.7859	12.93152	142.9862	12.90728	149.3638	13.11968	138.5905
12.92133	143.1097	12.94435	136.4037	13.0074	137.7826	12.94818	142.9839	12.92395	149.3627	13.13635	138.5907
12.938	143.1039	12.96102	136.3978	13.02407	137.7795	12.96485	142.9838	12.94062	149.3602	13.15302	138.5858
12.95467	143.098	12.97768	136.3924	13.04073	137.7775	12.98152	142.982	12.95728	149.3598	13.16968	138.5823
12.97133	143.0915	12.99435	136.3857	13.0574	137.7693	12.99818	142.9793	12.97395	149.3596	13.18635	138.58
12.988	143.0884	13.01102	136.379	13.07407	137.7665	13.01485	142.9794	12.99062	149.3596	13.20302	138.5786
13.00467	143.0847	13.02768	136.374	13.09073	137.7686	13.03152	142.9796	13.00728	149.356	13.21968	138.5734
13.02133	143.0821	13.04435	136.3672	13.1074	137.7606	13.04818	142.9781	13.02395	149.3539	13.23635	138.5709
13.038	143.0768	13.06102	136.363	13.12407	137.7558	13.06485	142.9747	13.04062	149.3516	13.25302	138.5714
13.05467	143.0721	13.07768	136.358	13.14073	137.7491	13.08152	142.9733	13.05728	149.3531	13.26968	138.5619
13.07133	143.0674	13.09435	136.3551	13.1574	137.7516	13.09818	142.9707	13.07395	149.3518	13.28635	138.5611
13.088	143.064	13.11102	136.35	13.17407	137.7469	13.11485	142.9707	13.09062	149.3514	13.30302	138.5568
13.10467	143.0594	13.12768	136.3464	13.19073	137.7421	13.13152	142.9669	13.10728	149.3503	13.31968	138.554
13.12133	143.0528	13.14435	136.3403	13.2074	137.7343	13.14818	142.9658	13.12395	149.3489	13.33635	138.5517
13.138	143.0524	13.16102	136.337	13.22407	137.7338	13.16485	142.9663	13.14062	149.3452	13.35302	138.549
13.15467	143.0459	13.17768	136.3341	13.24073	137.7341	13.18152	142.9624	13.15728	149.3473	13.36968	138.547
13.17133	143.0411	13.19435	136.3261	13.2574	137.7277	13.19818	142.9623	13.17395	149.3429	13.38635	138.5437
13.188	143.0383	13.21102	136.3234	13.27407	137.7194	13.21485	142.9573	13.19062	149.3452	13.40302	138.5402
13.20467	143.0374	13.22768	136.3202	13.29073	137.7234	13.23152	142.9575	13.20728	149.3436	13.41968	138.5386
13.22133	143.0297	13.24435	136.3158	13.3074	137.7124	13.24818	142.9558	13.22395	149.338	13.43635	138.535
13.238	143.0212	13.26102	136.3113	13.32407	137.7111	13.26485	142.9545	13.24062	149.342	13.45302	138.5312
13.25467	143.0214	13.27768	136.3038	13.34073	137.7072	13.28152	142.9533	13.25728	149.3392	13.46968	138.5287
13.27133	143.0106	13.29435	136.301	13.3574	137.7006	13.29818	142.9504	13.27395	149.339	13.48635	138.525
13.288	143.0124	13.31102	136.2944	13.37407	137.697	13.31485	142.9475	13.29062	149.337	13.50302	138.5212
13.30467	143.0089	13.32768	136.2935	13.39073	137.6959	13.33152	142.9461	13.30728	149.3342	13.51968	138.5176
13.32133	143.0039	13.34435	136.2866	13.4074	137.6921	13.34818	142.9435	13.32395	149.3332	13.53635	138.514
13.338	143.0013	13.36102	136.2824	13.42407	137.6893	13.36485	142.9409	13.34062	149.3348	13.55302	138.5123
13.35467	142.9935	13.37768	136.2787	13.44073	137.684	13.38152	142.9392	13.35728	149.333	13.56968	138.5104
13.37133	142.9894	13.39435	136.2724	13.4574	137.6795	13.39818	142.9384	13.37395	149.3317	13.58635	138.5048
13.388	142.987	13.41102	136.267	13.47407	137.6792	13.41485	142.9365	13.39062	149.3327	13.60302	138.5056
13.40467	142.9815	13.42768	136.2662	13.49073	137.671	13.43152	142.9327	13.40728	149.3298	13.61968	138.5017
13.42133	142.9794	13.44435	136.2592	13.5074	137.67	13.44818	142.9287	13.42395	149.3288	13.63635	138.4993
13.438	142.9746	13.46102	136.257	13.52407	137.6653	13.46485	142.9276	13.44062	149.3272	13.65302	138.4955
13.45467	142.9694	13.47768	136.2529	13.54073	137.6625	13.48152	142.9259	13.45728	149.3262	13.66968	138.4951
13.47133	142.9647	13.49435	136.2448	13.5574	137.6574	13.49818	142.9213	13.47395	149.3252	13.68635	138.4904
13.488	142.9615	13.51102	136.2404	13.57407	137.6541	13.51485	142.9176	13.49062	149.3241	13.70302	138.4872
13.50467	142.9576	13.52768	136.2364	13.59073	137.6474	13.53152	142.9177	13.50728	149.3227	13.71968	138.4852
13.52133	142.9513	13.54435	136.2315	13.6074	137.6446	13.54818	142.9146	13.52395	149.3243	13.73635	138.4804
13.538	142.9481	13.56102	136.2261	13.62407	137.6429	13.56485	142.9121	13.54062	149.3208	13.75302	138.4781
13.55467	142.9449	13.57768	136.2225	13.64073	137.6382	13.58152	142.9096	13.55728	149.3199	13.76968	138.4748
13.57133	142.939	13.59435	136.2186	13.6574	137.6339	13.59818	142.9087	13.57395	149.3191	13.78635	138.4757
13.588	142.9361	13.61102	136.2116	13.67407	137.6344	13.61485	142.9068	13.59062	149.319	13.80302	138.4718
13.60467	142.9329	13.62768	136.2078	13.69073	137.6264	13.63152	142.9042	13.60728	149.3207	13.81968	138.4678
13.62133	142.9271	13.64435	136.2064	13.7074	137.6229	13.64818	142.9043	13.62395	149.317	13.83635	138.4656
13.638	142.9222	13.66102	136.2007	13.72407	137.62	13.66485	142.9003	13.64062	149.3153	13.85302	138.4632
13.65467	142.9172	13.67768	136.1968	13.74073	137.6157	13.68152	142.8986	13.65728	149.3139	13.86968	138.4601
13.67133	142.9133	13.69435	136.1945	13.7574	137.6132	13.69818	142.8998	13.67395	149.3132	13.88635	138.4582
13.688	142.9148	13.71102	136.1893	13.77407	137.6081	13.71485	142.8975	13.69062	149.3124	13.90302	138.4541
13.70467	142.904	13.72768	136.1842	13.79073	137.605	13.73152	142.8948	13.70728	149.311	13.91968	138.4504
13.72133	142.9008	13.74435	136.1784	13.8074	137.6004	13.74818	142.8949	13.72395	149.3113	13.93635	138.4485
13.738	142.8971	13.76102	136.1758	13.82407	137.5964	13.76485	142.8939	13.74062	149.3094	13.95302	138.4451
13.75467	142.8968	13.77768	136.1715	13.84073	137.594	13.78152	142.8892	13.75728	149.3105	13.96968	138.4431
13.77133	142.886	13.79435	136.1668	13.8574	137.59	13.79818	142.8873	13.77395	149.3073	13.98635	138.4394

13.788	142.8858	13.81102	136.1612	13.87407	137.5853	13.81485	142.8885	13.79062	149.3057	14.00302	138.4353
13.80467	142.8809	13.82768	136.1575	13.89073	137.5825	13.83152	142.8831	13.80728	149.3092	14.01968	138.434
13.82133	142.8772	13.84435	136.1503	13.9074	137.5782	13.84818	142.8828	13.82395	149.305	14.03635	138.4304
13.838	142.8749	13.86102	136.1485	13.92407	137.5737	13.86485	142.8827	13.84062	149.303	14.05302	138.4274
13.85467	142.8699	13.87768	136.1426	13.94073	137.5712	13.88152	142.8788	13.85728	149.3	14.06968	138.4234
13.87133	142.8647	13.89435	136.1423	13.9574	137.5616	13.89818	142.8744	13.87395	149.3022	14.08635	138.4218
13.888	142.8601	13.91102	136.1373	13.97407	137.5643	13.91485	142.8769	13.89062	149.3001	14.10302	138.4194
13.90467	142.8571	13.92768	136.1353	13.99073	137.5568	13.93152	142.8735	13.90728	149.3018	14.11968	138.4146
13.92133	142.8536	13.94435	136.1277	14.0074	137.5525	13.94818	142.8694	13.92395	149.3017	14.13635	138.4119
13.938	142.848	13.96102	136.1219	14.02407	137.549	13.96485	142.8658	13.94062	149.2981	14.15302	138.4102
13.95467	142.8428	13.97768	136.1187	14.04073	137.5478	13.98152	142.8647	13.95728	149.301	14.16968	138.4052
13.97133	142.8386	13.99435	136.1173	14.0574	137.543	13.99818	142.8619	13.97395	149.3002	14.18635	138.404
13.988	142.8343	14.01102	136.1119	14.07407	137.5388	14.01485	142.8607	13.99062	149.2972	14.20302	138.3973
14.00467	142.8321	14.02768	136.1053	14.09073	137.5339	14.03152	142.8603	14.00728	149.2996	14.21968	138.3951
14.02133	142.8233	14.04435	136.1004	14.1074	137.5307	14.04818	142.8589	14.02395	149.2999	14.23635	138.3937
14.038	142.8171	14.06102	136.0965	14.12407	137.5264	14.06485	142.8564	14.04062	149.2977	14.25302	138.3893
14.05467	142.8195	14.07768	136.0943	14.14073	137.5215	14.08152	142.8564	14.05728	149.292	14.26968	138.3862
14.07133	142.8128	14.09435	136.0898	14.1574	137.5179	14.09818	142.8544	14.07395	149.2965	14.28635	138.3844
14.088	142.8091	14.11102	136.0867	14.17407	137.5165	14.11485	142.8522	14.09062	149.2979	14.30302	138.3838
14.10467	142.8045	14.12768	136.083	14.19073	137.5129	14.13152	142.8507	14.10728	149.2923	14.31968	138.3776
14.12133	142.7991	14.14435	136.0735	14.2074	137.509	14.14818	142.8491	14.12395	149.2956	14.33635	138.3768
14.138	142.7953	14.16102	136.0766	14.22407	137.5046	14.16485	142.8463	14.14062	149.2928	14.35302	138.3739
14.15467	142.7909	14.17768	136.0666	14.24073	137.5021	14.18152	142.8453	14.15728	149.2893	14.36968	138.3715
14.17133	142.7907	14.19435	136.0624	14.2574	137.497	14.19818	142.8453	14.17395	149.29	14.38635	138.3685
14.188	142.7834	14.21102	136.0606	14.27407	137.4952	14.21485	142.8407	14.19062	149.2902	14.40302	138.3649
14.20467	142.7775	14.22768	136.0566	14.29073	137.4865	14.23152	142.8394	14.20728	149.2871	14.41968	138.3595
14.22133	142.7806	14.24435	136.0504	14.3074	137.4849	14.24818	142.8381	14.22395	149.2872	14.43635	138.3605
14.238	142.7701	14.26102	136.0469	14.32407	137.4826	14.26485	142.8365	14.24062	149.2864	14.45302	138.3561
14.25467	142.7658	14.27768	136.039	14.34073	137.4803	14.28152	142.8315	14.25728	149.286	14.46968	138.3543
14.27133	142.7634	14.29435	136.036	14.3574	137.4751	14.29818	142.8312	14.27395	149.2845	14.48635	138.3526
14.288	142.7559	14.31102	136.0343	14.37407	137.4701	14.31485	142.8274	14.29062	149.2842	14.50302	138.3504
14.30467	142.7515	14.32768	136.0266	14.39073	137.4661	14.33152	142.8244	14.30728	149.2842	14.51968	138.3509
14.32133	142.7512	14.34435	136.0236	14.4074	137.4615	14.34818	142.8262	14.32395	149.2851	14.53635	138.343
14.338	142.746	14.36102	136.0189	14.42407	137.4596	14.36485	142.8207	14.34062	149.2833	14.55302	138.3446
14.35467	142.7432	14.37768	136.0151	14.44073	137.4583	14.38152	142.8204	14.35728	149.279	14.56968	138.3402
14.37133	142.7369	14.39435	136.0115	14.4574	137.4543	14.39818	142.8182	14.37395	149.2765	14.58635	138.337
14.388	142.7327	14.41102	136.0068	14.47407	137.4483	14.41485	142.816	14.39062	149.279	14.60302	138.3348
14.40467	142.7274	14.42768	136.0035	14.49073	137.4437	14.43152	142.8186	14.40728	149.278	14.61968	138.3311
14.42133	142.7265	14.44435	135.9989	14.5074	137.4415	14.44818	142.8148	14.42395	149.2783	14.63635	138.3281
14.438	142.7189	14.46102	135.9959	14.52407	137.437	14.46485	142.8132	14.44062	149.2776	14.65302	138.3256
14.45467	142.7196	14.47768	135.9893	14.54073	137.4333	14.48152	142.8131	14.45728	149.2779	14.66968	138.3224
14.47133	142.7092	14.49435	135.9872	14.5574	137.4296	14.49818	142.8086	14.47395	149.2726	14.68635	138.3201
14.488	142.7083	14.51102	135.9818	14.57407	137.431	14.51485	142.8081	14.49062	149.2716	14.70302	138.3179
14.50467	142.7058	14.52768	135.9767	14.59073	137.4226	14.53152	142.8119	14.50728	149.2728	14.71968	138.3146
14.52133	142.6986	14.54435	135.9759	14.6074	137.4183	14.54818	142.8096	14.52395	149.2715	14.73635	138.3125
14.538	142.6967	14.56102	135.9703	14.62407	137.4177	14.56485	142.8071	14.54062	149.2704	14.75302	138.3078
14.55467	142.6919	14.57768	135.9642	14.64073	137.4133	14.58152	142.8041	14.55728	149.2691	14.76968	138.3085
14.57133	142.6871	14.59435	135.9606	14.6574	137.4077	14.59818	142.8036	14.57395	149.2684	14.78635	138.3034
14.588	142.685	14.61102	135.9568	14.67407	137.4079	14.61485	142.8018	14.59062	149.2677	14.80302	138.3002
14.60467	142.6817	14.62768	135.9551	14.69073	137.4044	14.63152	142.8002	14.60728	149.265	14.81968	138.2998
14.62133	142.6796	14.64435	135.9497	14.7074	137.3967	14.64818	142.7965	14.62395	149.2599	14.83635	138.2971
14.638	142.6749	14.66102	135.9452	14.72407	137.3945	14.66485	142.7949	14.64062	149.2588	14.85302	138.294
14.65467	142.6727	14.67768	135.9407	14.74073	137.3927	14.68152	142.794	14.65728	149.2614	14.86968	138.2904
14.67133	142.6663	14.69435	135.9391	14.7574	137.3883	14.69818	142.7931	14.67395	149.2609	14.88635	138.2899
14.688	142.6641	14.71102	135.932	14.77407	137.3847	14.71485	142.7877	14.69062	149.2618	14.90302	138.2846
14.70467	142.6594	14.72768	135.9288	14.79073	137.3836	14.73152	142.7891	14.70728	149.2595	14.91968	138.2855
14.72133	142.6552	14.74435	135.9231	14.8074	137.3786	14.74818	142.7866	14.72395	149.258	14.93635	138.2799
14.738	142.653	14.76102	135.9221	14.82407	137.3753	14.76485	142.7842	14.74062	149.2577	14.95302	138.2769
14.75467	142.6486	14.77768	135.9153	14.84073	137.3715	14.78152	142.7832	14.75728	149.2573	14.96968	138.2775
14.77133	142.6431	14.79435	135.9135	14.8574	137.3675	14.79818	142.7804	14.77395	149.2565	14.98635	138.273
14.788	142.6377	14.81102	135.9095	14.87407	137.3646	14.81485	142.7786	14.79062	149.2547	15.00302	138.2706
14.80467	142.637	14.82768	135.9052	14.89073	137.362	14.83152	142.7753	14.80728	149.2529	15.01968	138.2675
14.82133	142.6317	14.84435	135.9016	14.9074	137.3566	14.84818	142.7745	14.82395	149.2531	15.03635	138.2655
14.838	142.6284	14.86102	135.8974	14.92407	137.3521	14.86485	142.7732	14.84062	149.251	15.05302	138.2608
14.85467	142.6266	14.87768	135.8901	14.94073	137.3474	14.88152	142.7736	14.85728	149.2532	15.06968	138.2569
14.87133	142.6213	14.89435	135.8891	14.9574	137.3459	14.89818	142.7723	14.87395	149.2519	15.08635	138.2577
14.888	142.6165	14.91102	135.886	14.97407	137.3417	14.91485	142.769	14.89062	149.2476	15.10302	138.2546
14.90467	142.6141	14.92768	135.8799	14.99073	137.3384	14.93152	142.7664	14.90728	149.2464	15.11968	138.2513
14.92133	142.6104	14.94435	135.8713	15.0074	137.3332	14.94818	142.7655	14.92395	149.2468	15.13635	138.2458

14.938	142.6063	14.96102	135.8706	15.02407	137.3299	14.96485	142.7615	14.94062	149.2466	15.15302	138.246
14.95467	142.6033	14.97768	135.8664	15.04073	137.3249	14.98152	142.7614	14.95728	149.2453	15.16968	138.2439
14.97133	142.5968	14.99435	135.8638	15.0574	137.3231	14.99818	142.759	14.97395	149.2456	15.18635	138.24
14.988	142.5984	15.01102	135.8585	15.07407	137.3189	15.01485	142.7578	14.99062	149.2454	15.20302	138.2366
15.00467	142.5918	15.02768	135.8538	15.09073	137.3144	15.03152	142.7572	15.00728	149.2409	15.21968	138.2362
15.02133	142.5867	15.04435	135.8508	15.1074	137.312	15.04818	142.7555	15.02395	149.2418	15.23635	138.2292
15.038	142.5856	15.06102	135.8434	15.12407	137.3056	15.06485	142.7556	15.04062	149.2418	15.25302	138.2251
15.05467	142.5778	15.07768	135.8388	15.14073	137.3052	15.08152	142.7533	15.05728	149.2399	15.26968	138.2227
15.07133	142.577	15.09435	135.8341	15.1574	137.3009	15.09818	142.7501	15.07395	149.2403	15.28635	138.2236
15.088	142.5725	15.11102	135.8319	15.17407	137.2991	15.11485	142.7494	15.09062	149.2403	15.30302	138.2179
15.10467	142.5697	15.12768	135.8268	15.19073	137.2971	15.13152	142.7485	15.10728	149.2385	15.31968	138.2167
15.12133	142.5641	15.14435	135.8233	15.2074	137.2932	15.14818	142.745	15.12395	149.2373	15.33635	138.2157
15.138	142.5579	15.16102	135.8183	15.22407	137.2883	15.16485	142.7457	15.14062	149.2391	15.35302	138.2106
15.15467	142.5574	15.17768	135.8143	15.24073	137.2876	15.18152	142.743	15.15728	149.2329	15.36968	138.2058
15.17133	142.5542	15.19435	135.8117	15.2574	137.2853	15.19818	142.7423	15.17395	149.2337	15.38635	138.2059
15.188	142.5494	15.21102	135.8082	15.27407	137.2786	15.21485	142.7393	15.19062	149.23	15.40302	138.2024
15.20467	142.5436	15.22768	135.805	15.29073	137.2762	15.23152	142.7391	15.20728	149.2343	15.41968	138.1985
15.22133	142.5412	15.24435	135.8014	15.3074	137.2732	15.24818	142.7386	15.22395	149.2311	15.43635	138.2021
15.238	142.5396	15.26102	135.7949	15.32407	137.2673	15.26485	142.7356	15.24062	149.2291	15.45302	138.1921
15.25467	142.5333	15.27768	135.7908	15.34073	137.2641	15.28152	142.7347	15.25728	149.2327	15.46968	138.1884
15.27133	142.5311	15.29435	135.7867	15.3574	137.2615	15.29818	142.7355	15.27395	149.2275	15.48635	138.1882
15.288	142.527	15.31102	135.7799	15.37407	137.2579	15.31485	142.7318	15.29062	149.2314	15.50302	138.1831
15.30467	142.5226	15.32768	135.7792	15.39073	137.2531	15.33152	142.7314	15.30728	149.2276	15.51968	138.1797
15.32133	142.5194	15.34435	135.7727	15.4074	137.2505	15.34818	142.7288	15.32395	149.2277	15.53635	138.1776
15.338	142.5149	15.36102	135.7699	15.42407	137.2468	15.36485	142.7246	15.34062	149.2289	15.55302	138.1744
15.35467	142.5092	15.37768	135.7684	15.44073	137.241	15.38152	142.723	15.35728	149.2228	15.56968	138.1726
15.37133	142.5058	15.39435	135.7638	15.4574	137.2391	15.39818	142.7221	15.37395	149.2261	15.58635	138.1667
15.388	142.5043	15.41102	135.7604	15.47407	137.2347	15.41485	142.7217	15.39062	149.223	15.60302	138.167
15.40467	142.4983	15.42768	135.7564	15.49073	137.2294	15.43152	142.7173	15.40728	149.2249	15.61968	138.161
15.42133	142.4957	15.44435	135.7506	15.5074	137.2267	15.44818	142.7165	15.42395	149.2219	15.63635	138.1608
15.438	142.491	15.46102	135.7488	15.52407	137.2218	15.46485	142.7132	15.44062	149.2208	15.65302	138.1549
15.45467	142.4883	15.47768	135.7416	15.54073	137.2169	15.48152	142.7084	15.45728	149.2196	15.66968	138.1521
15.47133	142.4867	15.49435	135.7398	15.5574	137.2186	15.49818	142.7125	15.47395	149.2188	15.68635	138.1506
15.488	142.4819	15.51102	135.7318	15.57407	137.2116	15.51485	142.7093	15.49062	149.2196	15.70302	138.1448
15.50467	142.4797	15.52768	135.731	15.59073	137.208	15.53152	142.7096	15.50728	149.2181	15.71968	138.1445
15.52133	142.4749	15.54435	135.7251	15.6074	137.2031	15.54818	142.7037	15.52395	149.2156	15.73635	138.139
15.538	142.4707	15.56102	135.7221	15.62407	137.2027	15.56485	142.7035	15.54062	149.2149	15.75302	138.1378
15.55467	142.4665	15.57768	135.7192	15.64073	137.1965	15.58152	142.7032	15.55728	149.2144	15.76968	138.1349
15.57133	142.4626	15.59435	135.7138	15.6574	137.1953	15.59818	142.7018	15.57395	149.2139	15.78635	138.1314
15.588	142.4591	15.61102	135.7096	15.67407	137.1932	15.61485	142.6974	15.59062	149.2118	15.80302	138.1293
15.60467	142.4569	15.62768	135.7057	15.69073	137.1904	15.63152	142.6968	15.60728	149.2105	15.81968	138.1259
15.62133	142.4517	15.64435	135.7024	15.7074	137.1855	15.64818	142.6922	15.62395	149.2103	15.83635	138.1232
15.638	142.4502	15.66102	135.6992	15.72407	137.1814	15.66485	142.6927	15.64062	149.2078	15.85302	138.1234
15.65467	142.4447	15.67768	135.6921	15.74073	137.1783	15.68152	142.6918	15.65728	149.2077	15.86968	138.1173
15.67133	142.4414	15.69435	135.6903	15.7574	137.1738	15.69818	142.6882	15.67395	149.2052	15.88635	138.1163
15.688	142.439	15.71102	135.6867	15.77407	137.1697	15.71485	142.6865	15.69062	149.2058	15.90302	138.112
15.70467	142.4358	15.72768	135.6833	15.79073	137.1688	15.73152	142.6836	15.70728	149.205	15.91968	138.1112
15.72133	142.4301	15.74435	135.6786	15.8074	137.1632	15.74818	142.6849	15.72395	149.203	15.93635	138.1082
15.738	142.4286	15.76102	135.6749	15.82407	137.1606	15.76485	142.6827	15.74062	149.1993	15.95302	138.1056
15.75467	142.4196	15.77768	135.6703	15.84073	137.1571	15.78152	142.6805	15.75728	149.2031	15.96968	138.1027
15.77133	142.4196	15.79435	135.6681	15.8574	137.1542	15.79818	142.6802	15.77395	149.2	15.98635	138.1021
15.788	142.4151	15.81102	135.6629	15.87407	137.1497	15.81485	142.6756	15.79062	149.1977	16.00302	138.0986
15.80467	142.411	15.82768	135.6578	15.89073	137.1467	15.83152	142.6767	15.80728	149.1976	16.01968	138.0978
15.82133	142.4093	15.84435	135.6527	15.9074	137.1417	15.84818	142.6753	15.82395	149.1977	16.03635	138.0938
15.838	142.405	15.86102	135.6527	15.92407	137.1394	15.86485	142.6696	15.84062	149.1952	16.05302	138.0935
15.85467	142.3992	15.87768	135.6452	15.94073	137.1362	15.88152	142.6703	15.85728	149.1961	16.06968	138.0908
15.87133	142.399	15.89435	135.642	15.9574	137.1332	15.89818	142.6667	15.87395	149.1981	16.08635	138.0897
15.888	142.3934	15.91102	135.6364	15.97407	137.1296	15.91485	142.6672	15.89062	149.1942	16.10302	138.0861
15.90467	142.3926	15.92768	135.6342	15.99073	137.1273	15.93152	142.665	15.90728	149.194	16.11968	138.0833
15.92133	142.3832	15.94435	135.6284	16.0074	137.124	15.94818	142.6636	15.92395	149.1941	16.13635	138.0813
15.938	142.3828	15.96102	135.628	16.02407	137.1206	15.96485	142.6586	15.94062	149.191	16.15302	138.078
15.95467	142.3786	15.97768	135.6237	16.04073	137.116	15.98152	142.6563	15.95728	149.1904	16.16968	138.0763
15.97133	142.3762	15.99435	135.6168	16.0574	137.1149	15.99818	142.6584	15.97395	149.1909	16.18635	138.073
15.988	142.3735	16.01102	135.6151	16.07407	137.1101	16.01485	142.6569	15.99062	149.1893	16.20302	138.0695
16.00467	142.3678	16.02768	135.6105	16.09073	137.1072	16.03152	142.653	16.00728	149.1876	16.21968	138.0689
16.02133	142.3619	16.04435	135.6059	16.1074	137.1025	16.04818	142.6503	16.02395	149.1878	16.23635	138.0656
16.038	142.3597	16.06102	135.6018	16.12407	137.1029	16.06485	142.6496	16.04062	149.1902	16.25302	138.0632
16.05467	142.355	16.07768	135.598	16.14073	137.0951	16.08152	142.6463	16.05728	149.1864	16.26968	138.0584
16.07133	142.3505	16.09435	135.5957	16.1574	137.0938	16.09818	142.6476	16.07395	149.1846	16.28635	138.0567

16.088	142.346	16.11102	135.5911	16.17407	137.0881	16.11485	142.648	16.09062	149.1836	16.30302	138.0537
16.10467	142.3438	16.12768	135.5856	16.19073	137.0866	16.13152	142.6421	16.10728	149.1852	16.31968	138.0502
16.12133	142.3398	16.14435	135.5831	16.2074	137.0798	16.14818	142.6428	16.12395	149.183	16.33635	138.0494
16.138	142.335	16.16102	135.5804	16.22407	137.0775	16.16485	142.6393	16.14062	149.1785	16.35302	138.0453
16.15467	142.3315	16.17768	135.576	16.24073	137.0741	16.18152	142.6376	16.15728	149.1807	16.36968	138.0453
16.17133	142.3279	16.19435	135.5721	16.2574	137.0702	16.19818	142.6382	16.17395	149.1777	16.38635	138.0418
16.188	142.326	16.21102	135.5683	16.27407	137.0666	16.21485	142.6361	16.19062	149.178	16.40302	138.0383
16.20467	142.317	16.22768	135.5639	16.29073	137.066	16.23152	142.6313	16.20728	149.1754	16.41968	138.0336
16.22133	142.3146	16.24435	135.5621	16.3074	137.0589	16.24818	142.6331	16.22395	149.1741	16.43635	138.035
16.238	142.3125	16.26102	135.5579	16.32407	137.0564	16.26485	142.6305	16.24062	149.1754	16.45302	138.0338
16.25467	142.3077	16.27768	135.5548	16.34073	137.0535	16.28152	142.6282	16.25728	149.1759	16.46968	138.0298
16.27133	142.3013	16.29435	135.5485	16.3574	137.0508	16.29818	142.6273	16.27395	149.1752	16.48635	138.0277
16.288	142.2989	16.31102	135.5429	16.37407	137.0477	16.31485	142.6232	16.29062	149.1722	16.50302	138.0234
16.30467	142.2949	16.32768	135.5406	16.39073	137.0458	16.33152	142.6233	16.30728	149.1728	16.51968	138.026
16.32133	142.2926	16.34435	135.5391	16.4074	137.0392	16.34818	142.6232	16.32395	149.1746	16.53635	138.0235
16.338	142.2888	16.36102	135.535	16.42407	137.0371	16.36485	142.6192	16.34062	149.1721	16.55302	138.0211
16.35467	142.2854	16.37768	135.5308	16.44073	137.0339	16.38152	142.6175	16.35728	149.1747	16.56968	138.0157
16.37133	142.2836	16.39435	135.5259	16.4574	137.0316	16.39818	142.6161	16.37395	149.1716	16.58635	138.0145
16.388	142.2771	16.41102	135.5243	16.47407	137.0274	16.41485	142.6168	16.39062	149.1706	16.60302	138.0123
16.40467	142.2742	16.42768	135.5168	16.49073	137.0229	16.43152	142.6136	16.40728	149.167	16.61968	138.0103
16.42133	142.2711	16.44435	135.5124	16.5074	137.0198	16.44818	142.6102	16.42395	149.1654	16.63635	138.0065
16.438	142.2681	16.46102	135.5154	16.52407	137.0169	16.46485	142.6083	16.44062	149.164	16.65302	138.0046
16.45467	142.2631	16.47768	135.5086	16.54073	137.0153	16.48152	142.6089	16.45728	149.1715	16.66968	138.0041
16.47133	142.2606	16.49435	135.5051	16.5574	137.0123	16.49818	142.6063	16.47395	149.1654	16.68635	138.0017
16.488	142.2551	16.51102	135.5016	16.57407	137.006	16.51485	142.6045	16.49062	149.1626	16.70302	138.0021
16.50467	142.2508	16.52768	135.4949	16.59073	137.0063	16.53152	142.6008	16.50728	149.1654	16.71968	137.9947
16.52133	142.2494	16.54435	135.4938	16.6074	137.0015	16.54818	142.6	16.52395	149.1596	16.73635	137.9942
16.538	142.2449	16.56102	135.4902	16.62407	136.9987	16.56485	142.5993	16.54062	149.165	16.75302	137.9942
16.55467	142.2425	16.57768	135.4857	16.64073	136.996	16.58152	142.5987	16.55728	149.1626	16.76968	137.9899
16.57133	142.2372	16.59435	135.4836	16.6574	136.9943	16.59818	142.5972	16.57395	149.1594	16.78635	137.9847
16.588	142.2303	16.61102	135.4807	16.67407	136.9879	16.61485	142.5968	16.59062	149.159	16.80302	137.9862
16.60467	142.2268	16.62768	135.4724	16.69073	136.9842	16.63152	142.593	16.60728	149.1622	16.81968	137.9843
16.62133	142.2227	16.64435	135.4705	16.7074	136.9813	16.64818	142.5913	16.62395	149.1599	16.83635	137.9792
16.638	142.2198	16.66102	135.4665	16.72407	136.9776	16.66485	142.591	16.64062	149.1584	16.85302	137.9771
16.65467	142.2174	16.67768	135.4647	16.74073	136.9766	16.68152	142.5899	16.65728	149.1563	16.86968	137.9722
16.67133	142.2122	16.69435	135.4585	16.7574	136.9718	16.69818	142.5888	16.67395	149.1545	16.88635	137.9725
16.688	142.2072	16.71102	135.4565	16.77407	136.9673	16.71485	142.5873	16.69062	149.1537	16.90302	137.9672
16.70467	142.2031	16.72768	135.4528	16.79073	136.9652	16.73152	142.5833	16.70728	149.1541	16.91968	137.9654
16.72133	142.1976	16.74435	135.4463	16.8074	136.9621	16.74818	142.5819	16.72395	149.1532	16.93635	137.9592
16.738	142.1947	16.76102	135.4484	16.82407	136.9579	16.76485	142.5815	16.74062	149.1519	16.95302	137.9596
16.75467	142.1924	16.77768	135.4399	16.84073	136.955	16.78152	142.5815	16.75728	149.1526	16.96968	137.9555
16.77133	142.1882	16.79435	135.4418	16.8574	136.9497	16.79818	142.5769	16.77395	149.15	16.98635	137.9526
16.788	142.1856	16.81102	135.4305	16.87407	136.9472	16.81485	142.5762	16.79062	149.148	17.00302	137.9504
16.80467	142.1826	16.82768	135.4285	16.89073	136.9453	16.83152	142.575	16.80728	149.1513	17.01968	137.949
16.82133	142.1784	16.84435	135.4264	16.9074	136.9402	16.84818	142.5733	16.82395	149.1469	17.03635	137.9462
16.838	142.1718	16.86102	135.4231	16.92407	136.9373	16.86485	142.571	16.84062	149.1493	17.05302	137.9432
16.85467	142.1695	16.87768	135.4195	16.94073	136.9323	16.88152	142.5696	16.85728	149.1473	17.06968	137.9394
16.87133	142.1689	16.89435	135.4159	16.9574	136.9305	16.89818	142.5665	16.87395	149.1449	17.08635	137.9391
16.888	142.1638	16.91102	135.4155	16.97407	136.9259	16.91485	142.5651	16.89062	149.1436	17.10302	137.934
16.90467	142.1607	16.92768	135.4065	16.99073	136.9251	16.93152	142.5624	16.90728	149.1442	17.11968	137.9343
16.92133	142.1563	16.94435	135.4011	17.0074	136.9228	16.94818	142.5634	16.92395	149.1434	17.13635	137.9277
16.938	142.1531	16.96102	135.3981	17.02407	136.9208	16.96485	142.5607	16.94062	149.141	17.15302	137.9265
16.95467	142.1487	16.97768	135.3957	17.04073	136.9137	16.98152	142.5578	16.95728	149.138	17.16968	137.925
16.97133	142.1453	16.99435	135.3938	17.0574	136.9127	16.99818	142.5544	16.97395	149.1399	17.18635	137.9225
16.988	142.1425	17.01102	135.3888	17.07407	136.9106	17.01485	142.5544	16.99062	149.1395	17.20302	137.9206
17.00467	142.1418	17.02768	135.3831	17.09073	136.9084	17.03152	142.5517	17.00728	149.1379	17.21968	137.9183
17.02133	142.1372	17.04435	135.3834	17.1074	136.9018	17.04818	142.5502	17.02395	149.1375	17.23635	137.914
17.038	142.133	17.06102	135.3782	17.12407	136.9	17.06485	142.5495	17.04062	149.1375	17.25302	137.9126
17.05467	142.1314	17.07768	135.3765	17.14073	136.8966	17.08152	142.5493	17.05728	149.1366	17.26968	137.9111
17.07133	142.1249	17.09435	135.3735	17.1574	136.8938	17.09818	142.5465	17.07395	149.1342	17.28635	137.9078
17.088	142.123	17.11102	135.3675	17.17407	136.8901	17.11485	142.5456	17.09062	149.1342	17.30302	137.9043
17.10467	142.1194	17.12768	135.3661	17.19073	136.8877	17.13152	142.5446	17.10728	149.1352	17.31968	137.9033
17.12133	142.1161	17.14435	135.3607	17.2074	136.8816	17.14818	142.5427	17.12395	149.1332	17.33635	137.8983
17.138	142.1143	17.16102	135.3597	17.22407	136.8838	17.16485	142.5424	17.14062	149.1308	17.35302	137.8954
17.15467	142.1105	17.17768	135.3549	17.24073	136.8772	17.18152	142.5388	17.15728	149.1313	17.36968	137.8925
17.17133	142.1064	17.19435	135.3492	17.2574	136.8732	17.19818	142.5391	17.17395	149.1305	17.38635	137.8891
17.188	142.1004	17.21102	135.3502	17.27407	136.8717	17.21485	142.5376	17.19062	149.1283	17.40302	137.8872
17.20467	142.1011	17.22768	135.3456	17.29073	136.8662	17.23152	142.5342	17.20728	149.1286	17.41968	137.8849
17.22133	142.094	17.24435	135.3387	17.3074	136.8649	17.24818	142.5347	17.22395	149.1268	17.43635	137.8785

17.238	142.0917	17.26102	135.3338	17.32407	136.8621	17.26485	142.5328	17.24062	149.1277	17.45302	137.8789
17.25467	142.088	17.27768	135.3285	17.34073	136.8589	17.28152	142.5336	17.25728	149.1247	17.46968	137.875
17.27133	142.0856	17.29435	135.3276	17.3574	136.8559	17.29818	142.5301	17.27395	149.1253	17.48635	137.8713
17.288	142.0796	17.31102	135.3255	17.37407	136.8537	17.31485	142.5288	17.29062	149.1239	17.50302	137.8706
17.30467	142.0792	17.32768	135.3229	17.39073	136.8494	17.33152	142.5271	17.30728	149.1233	17.51968	137.8705
17.32133	142.0713	17.34435	135.3178	17.4074	136.8465	17.34818	142.5263	17.32395	149.124	17.53635	137.8691
17.338	142.0675	17.36102	135.3146	17.42407	136.8453	17.36485	142.5265	17.34062	149.1197	17.55302	137.8672
17.35467	142.0654	17.37768	135.3105	17.44073	136.8405	17.38152	142.5232	17.35728	149.121	17.56968	137.8603
17.37133	142.0608	17.39435	135.3049	17.4574	136.8396	17.39818	142.5216	17.37395	149.1203	17.58635	137.8589
17.388	142.0571	17.41102	135.3036	17.47407	136.8335	17.41485	142.519	17.39062	149.1183	17.60302	137.8573
17.40467	142.0523	17.42768	135.2996	17.49073	136.832	17.43152	142.5181	17.40728	149.119	17.61968	137.8554
17.42133	142.0486	17.44435	135.2952	17.5074	136.8293	17.44818	142.5166	17.42395	149.1186	17.63635	137.853
17.438	142.048	17.46102	135.2918	17.52407	136.8255	17.46485	142.5148	17.44062	149.1175	17.65302	137.8516
17.45467	142.0433	17.47768	135.2888	17.54073	136.8242	17.48152	142.5143	17.45728	149.1161	17.66968	137.847
17.47133	142.0423	17.49435	135.2842	17.5574	136.8229	17.49818	142.5106	17.47395	149.1154	17.68635	137.8464
17.488	142.0338	17.51102	135.2822	17.57407	136.8161	17.51485	142.5107	17.49062	149.1144	17.70302	137.8465
17.50467	142.0342	17.52768	135.2796	17.59073	136.812	17.53152	142.5084	17.50728	149.1123	17.71968	137.8421
17.52133	142.0312	17.54435	135.2761	17.6074	136.8128	17.54818	142.5063	17.52395	149.1128	17.73635	137.8417
17.538	142.0294	17.56102	135.2703	17.62407	136.8117	17.56485	142.5039	17.54062	149.1105	17.75302	137.8355
17.55467	142.0226	17.57768	135.2668	17.64073	136.8058	17.58152	142.5031	17.55728	149.1099	17.76968	137.8309
17.57133	142.0231	17.59435	135.2636	17.6574	136.8032	17.59818	142.5012	17.57395	149.1095	17.78635	137.8379
17.588	142.0179	17.61102	135.2597	17.67407	136.7974	17.61485	142.5	17.59062	149.1101	17.80302	137.83
17.60467	142.0156	17.62768	135.2563	17.69073	136.7953	17.63152	142.4985	17.60728	149.1079	17.81968	137.8263
17.62133	142.0113	17.64435	135.2495	17.7074	136.7918	17.64818	142.4978	17.62395	149.1082	17.83635	137.8255
17.638	142.0101	17.66102	135.2481	17.72407	136.7888	17.66485	142.4964	17.64062	149.1088	17.85302	137.8229
17.65467	142.0062	17.67768	135.2456	17.74073	136.7825	17.68152	142.4945	17.65728	149.1056	17.86968	137.8212
17.67133	142.0048	17.69435	135.2425	17.7574	136.7837	17.69818	142.4937	17.67395	149.1054	17.88635	137.8187
17.688	142.001	17.71102	135.2361	17.77407	136.7786	17.71485	142.493	17.69062	149.1038	17.90302	137.8154
17.70467	141.9973	17.72768	135.2354	17.79073	136.7775	17.73152	142.4926	17.70728	149.1052	17.91968	137.8148
17.72133	141.9953	17.74435	135.2309	17.8074	136.7744	17.74818	142.4877	17.72395	149.1034	17.93635	137.8104
17.738	141.9898	17.76102	135.2282	17.82407	136.7701	17.76485	142.4863	17.74062	149.1006	17.95302	137.81
17.75467	141.9876	17.77768	135.2259	17.84073	136.7689	17.78152	142.485	17.75728	149.1049	17.96968	137.8046
17.77133	141.9844	17.79435	135.223	17.8574	136.7638	17.79818	142.4857	17.77395	149.0964	17.98635	137.8046
17.788	141.9818	17.81102	135.2174	17.87407	136.7624	17.81485	142.4845	17.79062	149.0998	18.00302	137.8003
17.80467	141.9787	17.82768	135.2128	17.89073	136.7609	17.83152	142.4808	17.80728	149.0982	18.01968	137.7998
17.82133	141.976	17.84435	135.2111	17.9074	136.7551	17.84818	142.479	17.82395	149.0962	18.03635	137.797
17.838	141.973	17.86102	135.2072	17.92407	136.7527	17.86485	142.4773	17.84062	149.0947	18.05302	137.7944
17.85467	141.9676	17.87768	135.2021	17.94073	136.7514	17.88152	142.4775	17.85728	149.0921	18.06968	137.7909
17.87133	141.9646	17.89435	135.2006	17.9574	136.747	17.89818	142.4741	17.87395	149.0961	18.08635	137.7896
17.888	141.9645	17.91102	135.1954	17.97407	136.7437	17.91485	142.4737	17.89062	149.095	18.10302	137.7873
17.90467	141.9591	17.92768	135.1938	17.99073	136.7427	17.93152	142.4684	17.90728	149.0879	18.11968	137.7856
17.92133	141.9524	17.94435	135.1881	18.0074	136.7364	17.94818	142.4675	17.92395	149.0934	18.13635	137.7823
17.938	141.9503	17.96102	135.1843	18.02407	136.7344	17.96485	142.4681	17.94062	149.087	18.15302	137.7811
17.95467	141.9492	17.97768	135.1796	18.04073	136.729	17.98152	142.465	17.95728	149.0931	18.16968	137.7801
17.97133	141.9463	17.99435	135.175	18.0574	136.728	17.99818	142.4626	17.97395	149.0909	18.18635	137.7781
17.988	141.9457	18.01102	135.1731	18.07407	136.7257	18.01485	142.4616	17.99062	149.0833	18.20302	137.7753
18.00467	141.9387	18.02768	135.1697	18.09073	136.72	18.03152	142.4597	18.00728	149.0885	18.21968	137.772
18.02133	141.9373	18.04435	135.1617	18.1074	136.7176	18.04818	142.4617	18.02395	149.0872	18.23635	137.7695
18.038	141.9336	18.06102	135.1636	18.12407	136.718	18.06485	142.4598	18.04062	149.0848	18.25302	137.7685
18.05467	141.9299	18.07768	135.1587	18.14073	136.7124	18.08152	142.4549	18.05728	149.0831	18.26968	137.7628
18.07133	141.9257	18.09435	135.1535	18.1574	136.7107	18.09818	142.4545	18.07395	149.0843	18.28635	137.761
18.088	141.9244	18.11102	135.1532	18.17407	136.7062	18.11485	142.4525	18.09062	149.0826	18.30302	137.7559
18.10467	141.9197	18.12768	135.1482	18.19073	136.702	18.13152	142.4551	18.10728	149.0799	18.31968	137.7546
18.12133	141.9188	18.14435	135.1444	18.2074	136.7007	18.14818	142.4535	18.12395	149.0786	18.33635	137.7512
18.138	141.9158	18.16102	135.1438	18.22407	136.6999	18.16485	142.4493	18.14062	149.0766	18.35302	137.7501
18.15467	141.9123	18.17768	135.1402	18.24073	136.6941	18.18152	142.4484	18.15728	149.077	18.36968	137.7487
18.17133	141.9109	18.19435	135.1353	18.2574	136.6903	18.19818	142.447	18.17395	149.0772	18.38635	137.7456
18.188	141.9053	18.21102	135.1316	18.27407	136.6876	18.21485	142.4428	18.19062	149.0793	18.40302	137.7417
18.20467	141.8994	18.22768	135.1287	18.29073	136.6843	18.23152	142.4417	18.20728	149.0749	18.41968	137.7386
18.22133	141.8985	18.24435	135.1218	18.3074	136.6809	18.24818	142.4405	18.22395	149.0763	18.43635	137.7381
18.238	141.8944	18.26102	135.1207	18.32407	136.6781	18.26485	142.4343	18.24062	149.0745	18.45302	137.737
18.25467	141.8925	18.27768	135.1176	18.34073	136.674	18.28152	142.4375	18.25728	149.0718	18.46968	137.7328
18.27133	141.8897	18.29435	135.1136	18.3574	136.6733	18.29818	142.4383	18.27395	149.0699	18.48635	137.7333
18.288	141.8854	18.31102	135.1111	18.37407	136.6724	18.31485	142.4346	18.29062	149.0718	18.50302	137.7279
18.30467	141.8862	18.32768	135.111	18.39073	136.6668	18.33152	142.431	18.30728	149.0705	18.51968	137.7288
18.32133	141.8812	18.34435	135.1029	18.4074	136.6646	18.34818	142.4282	18.32395	149.0665	18.53635	137.7268
18.338	141.8785	18.36102	135.0998	18.42407	136.66	18.36485	142.4269	18.34062	149.0662	18.55302	137.723
18.35467	141.8746	18.37768	135.0973	18.44073	136.6565	18.38152	142.4272	18.35728	149.0661	18.56968	137.7218
18.37133	141.8714	18.39435	135.0913	18.4574	136.6555	18.39818	142.4228	18.37395	149.062	18.58635	137.7217

18.388	141.8671	18.41102	135.092	18.47407	136.6518	18.41485	142.4237	18.39062	149.065	18.60302	137.7186
18.40467	141.8641	18.42768	135.0888	18.49073	136.6504	18.43152	142.4207	18.40728	149.0632	18.61968	137.7172
18.42133	141.8613	18.44435	135.0839	18.5074	136.6454	18.44818	142.4203	18.42395	149.0649	18.63635	137.7167
18.438	141.8597	18.46102	135.0825	18.52407	136.6414	18.46485	142.4166	18.44062	149.0615	18.65302	137.7133
18.45467	141.854	18.47768	135.0817	18.54073	136.6408	18.48152	142.4167	18.45728	149.0609	18.66968	137.7127
18.47133	141.8545	18.49435	135.0755	18.5574	136.6387	18.49818	142.4154	18.47395	149.0616	18.68635	137.7089
18.488	141.8505	18.51102	135.0726	18.57407	136.6336	18.51485	142.4136	18.49062	149.0602	18.70302	137.7062
18.50467	141.8465	18.52768	135.0676	18.59073	136.6317	18.53152	142.4112	18.50728	149.0587	18.71968	137.7073
18.52133	141.8436	18.54435	135.0649	18.6074	136.6285	18.54818	142.4102	18.52395	149.0574	18.73635	137.707
18.538	141.8391	18.56102	135.0616	18.62407	136.6261	18.56485	142.4075	18.54062	149.0584	18.75302	137.7044
18.55467	141.8389	18.57768	135.057	18.64073	136.6224	18.58152	142.4062	18.55728	149.0567	18.76968	137.7023
18.57133	141.8316	18.59435	135.0557	18.6574	136.6177	18.59818	142.4077	18.57395	149.0568	18.78635	137.6997
18.588	141.8309	18.61102	135.05	18.67407	136.6166	18.61485	142.4059	18.59062	149.0551	18.80302	137.6993
18.60467	141.8266	18.62768	135.0473	18.69073	136.6119	18.63152	142.4015	18.60728	149.0531	18.81968	137.6955
18.62133	141.8245	18.64435	135.0448	18.7074	136.6089	18.64818	142.4016	18.62395	149.0549	18.83635	137.6939
18.638	141.8212	18.66102	135.0399	18.72407	136.6074	18.66485	142.4009	18.64062	149.0513	18.85302	137.6907
18.65467	141.82	18.67768	135.036	18.74073	136.6034	18.68152	142.399	18.65728	149.0528	18.86968	137.6891
18.67133	141.815	18.69435	135.0339	18.7574	136.6019	18.69818	142.3967	18.67395	149.0514	18.88635	137.689
18.688	141.8111	18.71102	135.0314	18.77407	136.5978	18.71485	142.3988	18.69062	149.0531	18.90302	137.687
18.70467	141.8072	18.72768	135.0291	18.79073	136.5969	18.73152	142.3957	18.70728	149.0489	18.91968	137.6825
18.72133	141.804	18.74435	135.0256	18.8074	136.5953	18.74818	142.3935	18.72395	149.0508	18.93635	137.6822
18.738	141.8024	18.76102	135.023	18.82407	136.5899	18.76485	142.3936	18.74062	149.0492	18.95302	137.6815
18.75467	141.7985	18.77768	135.018	18.84073	136.586	18.78152	142.3919	18.75728	149.0492	18.96968	137.678
18.77133	141.7959	18.79435	135.0176	18.8574	136.5829	18.79818	142.3867	18.77395	149.0469	18.98635	137.6765
18.788	141.7931	18.81102	135.0167	18.87407	136.5821	18.81485	142.3877	18.79062	149.0482	19.00302	137.6725
18.80467	141.7861	18.82768	135.0089	18.89073	136.5816	18.83152	142.3856	18.80728	149.0486	19.01968	137.6734
18.82133	141.7864	18.84435	135.0059	18.9074	136.5775	18.84818	142.3839	18.82395	149.0471	19.03635	137.6716
18.838	141.7817	18.86102	135.004	18.92407	136.5706	18.86485	142.3825	18.84062	149.0424	19.05302	137.6705
18.85467	141.781	18.87768	135.001	18.94073	136.5726	18.88152	142.383	18.85728	149.0441	19.06968	137.6673
18.87133	141.7783	18.89435	134.9976	18.9574	136.5668	18.89818	142.3801	18.87395	149.043	19.08635	137.6655
18.888	141.7712	18.91102	134.9922	18.97407	136.5651	18.91485	142.3823	18.89062	149.0438	19.10302	137.6652
18.90467	141.7686	18.92768	134.9912	18.99073	136.5651	18.93152	142.3751	18.90728	149.0423	19.11968	137.6633
18.92133	141.7658	18.94435	134.9907	19.0074	136.5602	18.94818	142.3729	18.92395	149.041	19.13635	137.6601
18.938	141.7622	18.96102	134.9818	19.02407	136.5572	18.96485	142.3754	18.94062	149.0416	19.15302	137.6571
18.95467	141.7546	18.97768	134.9858	19.04073	136.5568	18.98152	142.3731	18.95728	149.041	19.16968	137.6588
18.97133	141.7561	18.99435	134.9792	19.0574	136.5504	18.99818	142.3712	18.97395	149.0398	19.18635	137.6543
18.988	141.7519	19.01102	134.9734	19.07407	136.5445	19.01485	142.3692	18.99062	149.0395	19.20302	137.6505
19.00467	141.7478	19.02768	134.9691	19.09073	136.5463	19.03152	142.3655	19.00728	149.038	19.21968	137.6493
19.02133	141.7461	19.04435	134.9693	19.1074	136.5408	19.04818	142.366	19.02395	149.0373	19.23635	137.6486
19.038	141.7448	19.06102	134.9667	19.12407	136.5423	19.06485	142.3646	19.04062	149.0385	19.25302	137.6481
19.05467	141.7327	19.07768	134.9631	19.14073	136.5389	19.08152	142.3644	19.05728	149.0357	19.26968	137.6416
19.07133	141.7359	19.09435	134.9587	19.1574	136.5346	19.09818	142.3599	19.07395	149.0357	19.28635	137.6403
19.088	141.7299	19.11102	134.9572	19.17407	136.5315	19.11485	142.3626	19.09062	149.0353	19.30302	137.6392
19.10467	141.73	19.12768	134.9514	19.19073	136.5287	19.13152	142.36	19.10728	149.0329	19.31968	137.6392
19.12133	141.7291	19.14435	134.9472	19.2074	136.5221	19.14818	142.3557	19.12395	149.036	19.33635	137.6334
19.138	141.7246	19.16102	134.9454	19.22407	136.5218	19.16485	142.3537	19.14062	149.0316	19.35302	137.6321
19.15467	141.7209	19.17768	134.9427	19.24073	136.5196	19.18152	142.3525	19.15728	149.0298	19.36968	137.6291
19.17133	141.718	19.19435	134.9396	19.2574	136.5137	19.19818	142.3539	19.17395	149.0327	19.38635	137.6269
19.188	141.7153	19.21102	134.9356	19.27407	136.5139	19.21485	142.3509	19.19062	149.034	19.40302	137.6275
19.20467	141.7091	19.22768	134.9311	19.29073	136.5105	19.23152	142.3511	19.20728	149.0303	19.41968	137.6242
19.22133	141.71	19.24435	134.9305	19.3074	136.5048	19.24818	142.3457	19.22395	149.0292	19.43635	137.6219
19.238	141.7032	19.26102	134.9262	19.32407	136.502	19.26485	142.3444	19.24062	149.0249	19.45302	137.6201
19.25467	141.7026	19.27768	134.9217	19.34073	136.5025	19.28152	142.3456	19.25728	149.0235	19.46968	137.6167
19.27133	141.6989	19.29435	134.9197	19.3574	136.4963	19.29818	142.3446	19.27395	149.0265	19.48635	137.6125
19.288	141.6958	19.31102	134.919	19.37407	136.4943	19.31485	142.3423	19.29062	149.0244	19.50302	137.6116
19.30467	141.6914	19.32768	134.913	19.39073	136.4905	19.33152	142.3398	19.30728	149.0214	19.51968	137.6093
19.32133	141.6877	19.34435	134.9098	19.4074	136.4887	19.34818	142.3384	19.32395	149.0219	19.53635	137.6067
19.338	141.6854	19.36102	134.9042	19.42407	136.4867	19.36485	142.3369	19.34062	149.0225	19.55302	137.6084
19.35467	141.6818	19.37768	134.9076	19.44073	136.4802	19.38152	142.3349	19.35728	149.0234	19.56968	137.604
19.37133	141.6778	19.39435	134.9009	19.4574	136.4823	19.39818	142.3356	19.37395	149.0222	19.58635	137.6042
19.388	141.6787	19.41102	134.8986	19.47407	136.4678	19.41485	142.3334	19.39062	149.0217	19.60302	137.6001
19.40467	141.6739	19.42768	134.8945	19.49073	136.4729	19.43152	142.3305	19.40728	149.0211	19.61968	137.5962
19.42133	141.6729	19.44435	134.8924	19.5074	136.4736	19.44818	142.3315	19.42395	149.0193	19.63635	137.5963
19.438	141.6679	19.46102	134.8894	19.52407	136.4693	19.46485	142.3271	19.44062	149.0176	19.65302	137.5926
19.45467	141.6652	19.47768	134.8874	19.54073	136.4647	19.48152	142.3241	19.45728	149.0166	19.66968	137.5913
19.47133	141.6634	19.49435	134.8855	19.5574	136.4629	19.49818	142.3229	19.47395	149.0165	19.68635	137.5882
19.488	141.6626	19.51102	134.8785	19.57407	136.4626	19.51485	142.3222	19.49062	149.0146	19.70302	137.5859
19.50467	141.6566	19.52768	134.8742	19.59073	136.4584	19.53152	142.3228	19.50728	149.0152	19.71968	137.5846
19.52133	141.6532	19.54435	134.873	19.6074	136.4539	19.54818	142.32	19.52395	149.0151	19.73635	137.5804

19.538	141.6512	19.56102	134.8719	19.62407	136.4486	19.56485	142.3185	19.54062	149.0104	19.75302	137.5803
19.55467	141.6468	19.57768	134.8636	19.64073	136.4476	19.58152	142.3174	19.55728	149.0108	19.76968	137.5758
19.57133	141.6449	19.59435	134.8667	19.6574	136.4466	19.59818	142.3164	19.57395	149.01	19.78635	137.5748
19.588	141.6408	19.61102	134.8631	19.67407	136.4433	19.61485	142.3151	19.59062	149.0101	19.80302	137.5744
19.60467	141.6376	19.62768	134.8618	19.69073	136.4402	19.63152	142.3122	19.60728	149.0086	19.81968	137.5731
19.62133	141.6333	19.64435	134.8565	19.7074	136.4381	19.64818	142.3098	19.62395	149.0099	19.83635	137.5677
19.638	141.6328	19.66102	134.8537	19.72407	136.4316	19.66485	142.3098	19.64062	149.0063	19.85302	137.5677
19.65467	141.6273	19.67768	134.8525	19.74073	136.4297	19.68152	142.3086	19.65728	149.007	19.86968	137.567
19.67133	141.6249	19.69435	134.8454	19.7574	136.4298	19.69818	142.3076	19.67395	149.0059	19.88635	137.5643
19.688	141.6192	19.71102	134.842	19.77407	136.424	19.71485	142.3073	19.69062	149.0047	19.90302	137.5634
19.70467	141.6179	19.72768	134.8431	19.79073	136.4232	19.73152	142.3037	19.70728	149.0034	19.91968	137.5582
19.72133	141.6117	19.74435	134.835	19.8074	136.4194	19.74818	142.3048	19.72395	149.0037	19.93635	137.5575
19.738	141.6078	19.76102	134.835	19.82407	136.4164	19.76485	142.3015	19.74062	149.0035	19.95302	137.5569
19.75467	141.6075	19.77768	134.8321	19.84073	136.411	19.78152	142.3025	19.75728	149.0038	19.96968	137.5536
19.77133	141.6044	19.79435	134.8278	19.8574	136.4103	19.79818	142.2999	19.77395	149.0012	19.98635	137.5524
19.788	141.6002	19.81102	134.8239	19.87407	136.4093	19.81485	142.3	19.79062	148.9987	20.00302	137.5524
19.80467	141.5989	19.82768	134.8206	19.89073	136.406	19.83152	142.2966	19.80728	148.9966	20.01968	137.5485
19.82133	141.5946	19.84435	134.8188	19.9074	136.4021	19.84818	142.295	19.82395	148.998	20.03635	137.5495
19.838	141.5928	19.86102	134.8172	19.92407	136.4012	19.86485	142.2935	19.84062	148.998	20.05302	137.5483
19.85467	141.5885	19.87768	134.81	19.94073	136.3977	19.88152	142.2929	19.85728	148.9956	20.06968	137.5464
19.87133	141.5826	19.89435	134.8081	19.9574	136.3961	19.89818	142.293	19.87395	148.9948	20.08635	137.5442
19.888	141.5837	19.91102	134.8068	19.97407	136.3948	19.91485	142.292	19.89062	148.994	20.10302	137.5375
19.90467	141.5767	19.92768	134.7984	19.99073	136.389	19.93152	142.2871	19.90728	148.9946	20.11968	137.5379
19.92133	141.5756	19.94435	134.8009	20.0074	136.3846	19.94818	142.288	19.92395	148.9949	20.13635	137.5364
19.938	141.5736	19.96102	134.7959	20.02407	136.3847	19.96485	142.2845	19.94062	148.9919	20.15302	137.5328
19.95467	141.5675	19.97768	134.7888	20.04073	136.3807	19.98152	142.2867	19.95728	148.9905	20.16968	137.5291
19.97133	141.5662	19.99435	134.7857	20.0574	136.3769	19.99818	142.2838	19.97395	148.9905	20.18635	137.5288
19.988	141.5609	20.01102	134.7818	20.07407	136.3768	20.01485	142.281	19.99062	148.9912	20.20302	137.5279
20.00467	141.5598	20.02768	134.7811	20.09073	136.373	20.03152	142.2808	20.00728	148.9895	20.21968	137.5265
20.02133	141.5552	20.04435	134.7798	20.1074	136.3692	20.04818	142.2799	20.02395	148.9871	20.23635	137.5255
20.038	141.554	20.06102	134.7768	20.12407	136.3688	20.06485	142.2766	20.04062	148.9867	20.25302	137.5201
20.05467	141.5514	20.07768	134.7677	20.14073	136.3622	20.08152	142.2754	20.05728	148.9884	20.26968	137.52
20.07133	141.5485	20.09435	134.7667	20.1574	136.3608	20.09818	142.2751	20.07395	148.9872	20.28635	137.5183
20.088	141.5439	20.11102	134.7625	20.17407	136.3616	20.11485	142.2726	20.09062	148.9837	20.30302	137.5175
20.10467	141.5407	20.12768	134.76	20.19073	136.3548	20.13152	142.2711	20.10728	148.9835	20.31968	137.5133
20.12133	141.5376	20.14435	134.7587	20.2074	136.3517	20.14818	142.2689	20.12395	148.9818	20.33635	137.5103
20.138	141.5336	20.16102	134.7546	20.22407	136.3501	20.16485	142.2669	20.14062	148.9836	20.35302	137.5114
20.15467	141.5278	20.17768	134.7529	20.24073	136.3453	20.18152	142.2684	20.15728	148.9817	20.36968	137.5063
20.17133	141.5289	20.19435	134.7498	20.2574	136.3451	20.19818	142.2669	20.17395	148.982	20.38635	137.5068
20.188	141.5273	20.21102	134.7472	20.27407	136.3454	20.21485	142.2642	20.19062	148.9792	20.40302	137.5027
20.20467	141.5232	20.22768	134.7436	20.29073	136.34	20.23152	142.2622	20.20728	148.9799	20.41968	137.5038
20.22133	141.5189	20.24435	134.7386	20.3074	136.3352	20.24818	142.2636	20.22395	148.9813	20.43635	137.501
20.238	141.5186	20.26102	134.7367	20.32407	136.3341	20.26485	142.2619	20.24062	148.9776	20.45302	137.4979
20.25467	141.5158	20.27768	134.7333	20.34073	136.3304	20.28152	142.2622	20.25728	148.9783	20.46968	137.4963
20.27133	141.5098	20.29435	134.7319	20.3574	136.3267	20.29818	142.2586	20.27395	148.9753	20.48635	137.4947
20.288	141.5081	20.31102	134.7296	20.37407	136.3247	20.31485	142.2571	20.29062	148.9751	20.50302	137.4938
20.30467	141.5076	20.32768	134.7251	20.39073	136.3241	20.33152	142.2564	20.30728	148.9732	20.51968	137.4933
20.32133	141.503	20.34435	134.7212	20.4074	136.3195	20.34818	142.2561	20.32395	148.9753	20.53635	137.4864
20.338	141.5007	20.36102	134.7184	20.42407	136.3183	20.36485	142.2528	20.34062	148.974	20.55302	137.4878
20.35467	141.4962	20.37768	134.7162	20.44073	136.3153	20.38152	142.2507	20.35728	148.9704	20.56968	137.4846
20.37133	141.4932	20.39435	134.7111	20.4574	136.3119	20.39818	142.2518	20.37395	148.9715	20.58635	137.4832
20.388	141.4919	20.41102	134.7105	20.47407	136.3108	20.41485	142.2493	20.39062	148.9717	20.60302	137.4801
20.40467	141.4887	20.42768	134.7041	20.49073	136.3059	20.43152	142.2465	20.40728	148.9709	20.61968	137.4786
20.42133	141.484	20.44435	134.7046	20.5074	136.3037	20.44818	142.246	20.42395	148.9686	20.63635	137.4792
20.438	141.4821	20.46102	134.6991	20.52407	136.3021	20.46485	142.246	20.44062	148.9674	20.65302	137.4784
20.45467	141.4787	20.47768	134.6959	20.54073	136.2996	20.48152	142.2452	20.45728	148.9699	20.66968	137.4742
20.47133	141.4777	20.49435	134.6963	20.5574	136.2941	20.49818	142.2433	20.47395	148.966	20.68635	137.4714
20.488	141.4716	20.51102	134.6911	20.57407	136.2915	20.51485	142.2414	20.49062	148.9647	20.70302	137.4712
20.50467	141.4694	20.52768	134.6875	20.59073	136.2885	20.53152	142.2429	20.50728	148.9658	20.71968	137.4726
20.52133	141.4669	20.54435	134.684	20.6074	136.2899	20.54818	142.2414	20.52395	148.9625	20.73635	137.4643
20.538	141.4667	20.56102	134.6843	20.62407	136.2851	20.56485	142.2371	20.54062	148.9597	20.75302	137.4644
20.55467	141.4594	20.57768	134.6762	20.64073	136.2818	20.58152	142.2366	20.55728	148.9594	20.76968	137.4644
20.57133	141.4516	20.59435	134.6764	20.6574	136.2788	20.59818	142.236	20.57395	148.9592	20.78635	137.4619
20.588	141.4528	20.61102	134.6741	20.67407	136.2744	20.61485	142.2352	20.59062	148.9582	20.80302	137.4589
20.60467	141.4518	20.62768	134.6667	20.69073	136.275	20.63152	142.235	20.60728	148.9586	20.81968	137.4573
20.62133	141.45	20.64435	134.6641	20.7074	136.2717	20.64818	142.2309	20.62395	148.9561	20.83635	137.4533
20.638	141.4472	20.66102	134.661	20.72407	136.2674	20.66485	142.2321	20.64062	148.9567	20.85302	137.4524
20.65467	141.4439	20.67768	134.6565	20.74073	136.263	20.68152	142.2273	20.65728	148.9574	20.86968	137.4489
20.67133	141.4395	20.69435	134.6537	20.7574	136.2619	20.69818	142.2286	20.67395	148.9546	20.88635	137.4475

20.688	141.4334	20.71102	134.6504	20.77407	136.2569	20.71485	142.2276	20.69062	148.9534	20.90302	137.4464
20.70467	141.4326	20.72768	134.6486	20.79073	136.2555	20.73152	142.2227	20.70728	148.9531	20.91968	137.4467
20.72133	141.4308	20.74435	134.6479	20.8074	136.2555	20.74818	142.2238	20.72395	148.9517	20.93635	137.4438
20.738	141.4269	20.76102	134.6423	20.82407	136.2529	20.76485	142.2211	20.74062	148.9521	20.95302	137.4408
20.75467	141.4244	20.77768	134.6381	20.84073	136.25	20.78152	142.2191	20.75728	148.9511	20.96968	137.4406
20.77133	141.4191	20.79435	134.6376	20.8574	136.2463	20.79818	142.2184	20.77395	148.9519	20.98635	137.4396
20.788	141.4177	20.81102	134.6322	20.87407	136.2417	20.81485	142.2161	20.79062	148.9517	21.00302	137.4354
20.80467	141.4168	20.82768	134.6313	20.89073	136.2379	20.83152	142.2142	20.80728	148.9505	21.01968	137.4361
20.82133	141.4128	20.84435	134.6275	20.9074	136.2392	20.84818	142.2141	20.82395	148.9465	21.03635	137.4332
20.838	141.4079	20.86102	134.6269	20.92407	136.2332	20.86485	142.2147	20.84062	148.9482	21.05302	137.4284
20.85467	141.4064	20.87768	134.6234	20.94073	136.2295	20.88152	142.2111	20.85728	148.9455	21.06968	137.4293
20.87133	141.4037	20.89435	134.6204	20.9574	136.2274	20.89818	142.2101	20.87395	148.9456	21.08635	137.4247
20.888	141.399	20.91102	134.6202	20.97407	136.2253	20.91485	142.211	20.89062	148.9462	21.10302	137.4232
20.90467	141.3932	20.92768	134.6131	20.99073	136.2178	20.93152	142.2107	20.90728	148.9452	21.11968	137.4212
20.92133	141.3913	20.94435	134.6101	21.0074	136.2185	20.94818	142.2078	20.92395	148.9454	21.13635	137.4224
20.938	141.3906	20.96102	134.6067	21.02407	136.2145	20.96485	142.2077	20.94062	148.9399	21.15302	137.4182
20.95467	141.3894	20.97768	134.6047	21.04073	136.212	20.98152	142.2068	20.95728	148.9431	21.16968	137.4161
20.97133	141.3833	20.99435	134.5975	21.0574	136.2079	20.99818	142.2046	20.97395	148.9429	21.18635	137.4144
20.988	141.3813	21.01102	134.5967	21.07407	136.2072	21.01485	142.2025	20.99062	148.9425	21.20302	137.412
21.00467	141.376	21.02768	134.5915	21.09073	136.2025	21.03152	142.202	21.00728	148.9415	21.21968	137.4119
21.02133	141.3734	21.04435	134.591	21.1074	136.1991	21.04818	142.2007	21.02395	148.9389	21.23635	137.4089
21.038	141.3716	21.06102	134.5858	21.12407	136.2003	21.06485	142.2006	21.04062	148.9378	21.25302	137.4074
21.05467	141.3704	21.07768	134.5847	21.14073	136.195	21.08152	142.2002	21.05728	148.9397	21.26968	137.4073
21.07133	141.3698	21.09435	134.5815	21.1574	136.1904	21.09818	142.1988	21.07395	148.9371	21.28635	137.4026
21.088	141.3632	21.11102	134.5781	21.17407	136.1869	21.11485	142.1957	21.09062	148.939	21.30302	137.404
21.10467	141.3605	21.12768	134.5774	21.19073	136.1855	21.13152	142.1958	21.10728	148.9336	21.31968	137.4022
21.12133	141.3589	21.14435	134.5717	21.2074	136.1829	21.14818	142.1913	21.12395	148.9346	21.33635	137.4008
21.138	141.354	21.16102	134.5693	21.22407	136.1794	21.16485	142.1906	21.14062	148.9359	21.35302	137.3964
21.15467	141.3567	21.17768	134.5642	21.24073	136.1779	21.18152	142.1917	21.15728	148.9347	21.36968	137.3942
21.17133	141.3516	21.19435	134.5651	21.2574	136.1746	21.19818	142.1881	21.17395	148.9353	21.38635	137.3952
21.188	141.3457	21.21102	134.5614	21.27407	136.1723	21.21485	142.1896	21.19062	148.933	21.40302	137.3947
21.20467	141.3447	21.22768	134.5591	21.29073	136.169	21.23152	142.1883	21.20728	148.9323	21.41968	137.392
21.22133	141.3409	21.24435	134.5574	21.3074	136.1667	21.24818	142.1856	21.22395	148.933	21.43635	137.3857
21.238	141.3405	21.26102	134.5511	21.32407	136.1642	21.26485	142.1862	21.24062	148.9326	21.45302	137.3865
21.25467	141.3368	21.27768	134.5477	21.34073	136.1632	21.28152	142.1827	21.25728	148.9327	21.46968	137.3849
21.27133	141.3342	21.29435	134.5473	21.3574	136.1594	21.29818	142.1795	21.27395	148.9303	21.48635	137.3808
21.288	141.331	21.31102	134.5393	21.37407	136.1575	21.31485	142.1802	21.29062	148.9289	21.50302	137.3794
21.30467	141.3249	21.32768	134.5413	21.39073	136.1542	21.33152	142.1793	21.30728	148.9274	21.51968	137.3747
21.32133	141.3226	21.34435	134.5383	21.4074	136.1523	21.34818	142.1795	21.32395	148.9273	21.53635	137.3744
21.338	141.3222	21.36102	134.5316	21.42407	136.1476	21.36485	142.1759	21.34062	148.9252	21.55302	137.372
21.35467	141.3194	21.37768	134.5315	21.44073	136.145	21.38152	142.1733	21.35728	148.9271	21.56968	137.3684
21.37133	141.3158	21.39435	134.5302	21.4574	136.1423	21.39818	142.1719	21.37395	148.9244	21.58635	137.3673
21.388	141.31	21.41102	134.527	21.47407	136.1401	21.41485	142.1731	21.39062	148.9241	21.60302	137.3653
21.40467	141.3098	21.42768	134.5197	21.49073	136.1394	21.43152	142.1707	21.40728	148.9249	21.61968	137.3622
21.42133	141.3066	21.44435	134.5254	21.5074	136.1318	21.44818	142.1696	21.42395	148.9236	21.63635	137.3625
21.438	141.3039	21.46102	134.5153	21.52407	136.1313	21.46485	142.165	21.44062	148.9235	21.65302	137.3591
21.45467	141.2992	21.47768	134.515	21.54073	136.1291	21.48152	142.1668	21.45728	148.9213	21.66968	137.3559
21.47133	141.2944	21.49435	134.513	21.5574	136.1253	21.49818	142.1683	21.47395	148.92	21.68635	137.3542
21.488	141.2909	21.51102	134.5101	21.57407	136.1217	21.51485	142.1658	21.49062	148.918	21.70302	137.3535
21.50467	141.2896	21.52768	134.5071	21.59073	136.1206	21.53152	142.1625	21.50728	148.9205	21.71968	137.3494
21.52133	141.286	21.54435	134.5057	21.6074	136.1164	21.54818	142.1596	21.52395	148.9205	21.73635	137.3493
21.538	141.2858	21.56102	134.5067	21.62407	136.1137	21.56485	142.1588	21.54062	148.919	21.75302	137.3462
21.55467	141.2823	21.57768	134.5009	21.64073	136.1111	21.58152	142.1577	21.55728	148.9186	21.76968	137.3437
21.57133	141.2778	21.59435	134.4979	21.6574	136.106	21.59818	142.1569	21.57395	148.9169	21.78635	137.3432
21.588	141.2788	21.61102	134.4931	21.67407	136.1048	21.61485	142.1549	21.59062	148.9176	21.80302	137.3393
21.60467	141.2714	21.62768	134.4885	21.69073	136.0986	21.63152	142.1531	21.60728	148.9165	21.81968	137.339
21.62133	141.2687	21.64435	134.4879	21.7074	136.0962	21.64818	142.1509	21.62395	148.914	21.83635	137.3381
21.638	141.2668	21.66102	134.4866	21.72407	136.0959	21.66485	142.1494	21.64062	148.9134	21.85302	137.3358
21.65467	141.2645	21.67768	134.4875	21.74073	136.0931	21.68152	142.1519	21.65728	148.9138	21.86968	137.3343
21.67133	141.2573	21.69435	134.4841	21.7574	136.0895	21.69818	142.1479	21.67395	148.9162	21.88635	137.3311
21.688	141.2579	21.71102	134.4732	21.77407	136.0868	21.71485	142.1469	21.69062	148.9128	21.90302	137.3297
21.70467	141.2528	21.72768	134.4737	21.79073	136.0811	21.73152	142.1456	21.70728	148.9126	21.91968	137.3285
21.72133	141.2559	21.74435	134.4648	21.8074	136.0856	21.74818	142.1426	21.72395	148.9131	21.93635	137.3267
21.738	141.2493	21.76102	134.4619	21.82407	136.0795	21.76485	142.1457	21.74062	148.9108	21.95302	137.3237
21.75467	141.2463	21.77768	134.4609	21.84073	136.0819	21.78152	142.1409	21.75728	148.9078	21.96968	137.3209
21.77133	141.2424	21.79435	134.4563	21.8574	136.0735	21.79818	142.1383	21.77395	148.909	21.98635	137.3224
21.788	141.2404	21.81102	134.4537	21.87407	136.072	21.81485	142.1383	21.79062	148.9061	22.00302	137.3184
21.80467	141.2377	21.82768	134.4523	21.89073	136.072	21.83152	142.1394	21.80728	148.9037	22.01968	137.3182
21.82133	141.2348	21.84435	134.4483	21.9074	136.0688	21.84818	142.1368	21.82395	148.9047	22.03635	137.3148

21.838	141.2311	21.86102	134.4463	21.92407	136.0681	21.86485	142.1348	21.84062	148.9033	22.05302	137.3139
21.85467	141.2296	21.87768	134.4456	21.94073	136.0666	21.88152	142.1382	21.85728	148.901	22.06968	137.311
21.87133	141.2263	21.89435	134.4406	21.9574	136.0604	21.89818	142.1322	21.87395	148.8998	22.08635	137.3106
21.888	141.2221	21.91102	134.4409	21.97407	136.0603	21.91485	142.1329	21.89062	148.8988	22.10302	137.3076
21.90467	141.2233	21.92768	134.4389	21.99073	136.055	21.93152	142.1303	21.90728	148.8994	22.11968	137.3075
21.92133	141.2209	21.94435	134.4326	22.0074	136.0528	21.94818	142.1299	21.92395	148.897	22.13635	137.3056
21.938	141.218	21.96102	134.4298	22.02407	136.0515	21.96485	142.1294	21.94062	148.8979	22.15302	137.2997
21.95467	141.2151	21.97768	134.4263	22.04073	136.0512	21.98152	142.1301	21.95728	148.8965	22.16968	137.3009
21.97133	141.2099	21.99435	134.4237	22.0574	136.048	21.99818	142.1289	21.97395	148.8943	22.18635	137.297
21.988	141.2078	22.01102	134.4208	22.07407	136.0439	22.01485	142.1275	21.99062	148.8959	22.20302	137.2974
22.00467	141.2055	22.02768	134.4159	22.09073	136.0413	22.03152	142.1259	22.00728	148.8959	22.21968	137.296
22.02133	141.2025	22.04435	134.4185	22.1074	136.0412	22.04818	142.1234	22.02395	148.8925	22.23635	137.2944
22.038	141.1973	22.06102	134.4121	22.12407	136.0339	22.06485	142.1229	22.04062	148.8925	22.25302	137.2914
22.05467	141.1994	22.07768	134.4105	22.14073	136.0337	22.08152	142.1223	22.05728	148.8922	22.26968	137.29
22.07133	141.1916	22.09435	134.4065	22.1574	136.0318	22.09818	142.1208	22.07395	148.8926	22.28635	137.2881
22.088	141.1925	22.11102	134.4011	22.17407	136.0293	22.11485	142.1188	22.09062	148.893	22.30302	137.2859
22.10467	141.1883	22.12768	134.4051	22.19073	136.0255	22.13152	142.1185	22.10728	148.8906	22.31968	137.2832
22.12133	141.185	22.14435	134.3983	22.2074	136.0234	22.14818	142.117	22.12395	148.8908	22.33635	137.2817
22.138	141.1793	22.16102	134.395	22.22407	136.0228	22.16485	142.1142	22.14062	148.8888	22.35302	137.2813
22.15467	141.1776	22.17768	134.3951	22.24073	136.0188	22.18152	142.114	22.15728	148.8876	22.36968	137.2764
22.17133	141.1768	22.19435	134.3909	22.2574	136.0159	22.19818	142.1101	22.17395	148.8887	22.38635	137.2778
22.188	141.1723	22.21102	134.3913	22.27407	136.012	22.21485	142.1113	22.19062	148.8848	22.40302	137.2734
22.20467	141.1688	22.22768	134.3872	22.29073	136.0131	22.23152	142.1091	22.20728	148.8866	22.41968	137.2712
22.22133	141.1653	22.24435	134.3847	22.3074	136.0124	22.24818	142.1094	22.22395	148.8859	22.43635	137.27
22.238	141.1617	22.26102	134.3783	22.32407	136.0059	22.26485	142.1087	22.24062	148.8848	22.45302	137.2679
22.25467	141.1617	22.27768	134.3792	22.34073	136.0025	22.28152	142.1069	22.25728	148.8865	22.46968	137.2671
22.27133	141.1547	22.29435	134.3742	22.3574	136.0017	22.29818	142.1046	22.27395	148.8837	22.48635	137.2659
22.288	141.1514	22.31102	134.371	22.37407	135.9994	22.31485	142.1007	22.29062	148.883	22.50302	137.2624
22.30467	141.1531	22.32768	134.3697	22.39073	135.9972	22.33152	142.1016	22.30728	148.8818	22.51968	137.2591
22.32133	141.1465	22.34435	134.3633	22.4074	135.999	22.34818	142.1014	22.32395	148.8805	22.53635	137.2603
22.338	141.147	22.36102	134.3593	22.42407	135.9951	22.36485	142.101	22.34062	148.8805	22.55302	137.26
22.35467	141.1428	22.37768	134.3585	22.44073	135.9927	22.38152	142.098	22.35728	148.8801	22.56968	137.254
22.37133	141.1379	22.39435	134.3567	22.4574	135.9884	22.39818	142.0999	22.37395	148.8816	22.58635	137.2537
22.388	141.1355	22.41102	134.3524	22.47407	135.9868	22.41485	142.094	22.39062	148.8791	22.60302	137.2515
22.40467	141.1329	22.42768	134.3491	22.49073	135.9845	22.43152	142.094	22.40728	148.8787	22.61968	137.2505
22.42133	141.1331	22.44435	134.3439	22.5074	135.9819	22.44818	142.0923	22.42395	148.8785	22.63635	137.2472
22.438	141.1262	22.46102	134.3451	22.52407	135.9792	22.46485	142.0925	22.44062	148.8757	22.65302	137.2463
22.45467	141.1241	22.47768	134.3404	22.54073	135.9779	22.48152	142.0919	22.45728	148.8761	22.66968	137.2426
22.47133	141.1243	22.49435	134.3353	22.5574	135.974	22.49818	142.0869	22.47395	148.8771	22.68635	137.2401
22.488	141.117	22.51102	134.3336	22.57407	135.9703	22.51485	142.0858	22.49062	148.8753	22.70302	137.2407
22.50467	141.118	22.52768	134.3329	22.59073	135.9701	22.53152	142.0868	22.50728	148.873	22.71968	137.2378
22.52133	141.1137	22.54435	134.3256	22.6074	135.968	22.54818	142.0856	22.52395	148.8736	22.73635	137.2357
22.538	141.1089	22.56102	134.3245	22.62407	135.9627	22.56485	142.0844	22.54062	148.872	22.75302	137.2326
22.55467	141.1071	22.57768	134.325	22.64073	135.9629	22.58152	142.0827	22.55728	148.8709	22.76968	137.2324
22.57133	141.1074	22.59435	134.3217	22.6574	135.9588	22.59818	142.0805	22.57395	148.8689	22.78635	137.2287
22.588	141.1004	22.61102	134.3168	22.67407	135.9549	22.61485	142.0788	22.59062	148.8663	22.80302	137.2279
22.60467	141.1	22.62768	134.3157	22.69073	135.9539	22.63152	142.0808	22.60728	148.867	22.81968	137.2248
22.62133	141.0979	22.64435	134.3115	22.7074	135.9523	22.64818	142.0793	22.62395	148.8661	22.83635	137.2234
22.638	141.0901	22.66102	134.3102	22.72407	135.9475	22.66485	142.078	22.64062	148.8654	22.85302	137.2225
22.65467	141.0888	22.67768	134.3111	22.74073	135.9458	22.68152	142.0744	22.65728	148.865	22.86968	137.2205
22.67133	141.0871	22.69435	134.3013	22.7574	135.9414	22.69818	142.077	22.67395	148.8641	22.88635	137.2175
22.688	141.0849	22.71102	134.2989	22.77407	135.9421	22.71485	142.0732	22.69062	148.8625	22.90302	137.2155
22.70467	141.079	22.72768	134.2972	22.79073	135.94	22.73152	142.0725	22.70728	148.8618	22.91968	137.2142
22.72133	141.0765	22.74435	134.295	22.8074	135.9367	22.74818	142.0737	22.72395	148.8599	22.93635	137.212
22.738	141.0736	22.76102	134.2905	22.82407	135.9342	22.76485	142.0726	22.74062	148.8604	22.95302	137.208
22.75467	141.0719	22.77768	134.2896	22.84073	135.9322	22.78152	142.0685	22.75728	148.8599	22.96968	137.2088
22.77133	141.0693	22.79435	134.2854	22.8574	135.9294	22.79818	142.068	22.77395	148.8595	22.98635	137.2076
22.788	141.0648	22.81102	134.2849	22.87407	135.9266	22.81485	142.0661	22.79062	148.8602	23.00302	137.2041
22.80467	141.064	22.82768	134.2806	22.89073	135.925	22.83152	142.0672	22.80728	148.8581	23.01968	137.2045
22.82133	141.0651	22.84435	134.28	22.9074	135.9241	22.84818	142.0641	22.82395	148.8575	23.03635	137.2022
22.838	141.0607	22.86102	134.2758	22.92407	135.9205	22.86485	142.0621	22.84062	148.8554	23.05302	137.2001
22.85467	141.059	22.87768	134.2704	22.94073	135.9174	22.88152	142.0609	22.85728	148.861	23.06968	137.1986
22.87133	141.0564	22.89435	134.2679	22.9574	135.9155	22.89818	142.0608	22.87395	148.8578	23.08635	137.1948
22.888	141.0523	22.91102	134.2666	22.97407	135.913	22.91485	142.0573	22.89062	148.8572	23.10302	137.1929
22.90467	141.051	22.92768	134.2615	22.99073	135.9095	22.93152	142.0574	22.90728	148.8563	23.11968	137.1897
22.92133	141.0479	22.94435	134.2628	23.0074	135.9054	22.94818	142.057	22.92395	148.8551	23.13635	137.1877
22.938	141.0429	22.96102	134.2577	23.02407	135.9031	22.96485	142.0574	22.94062	148.8566	23.15302	137.1851
22.95467	141.0411	22.97768	134.2565	23.04073	135.9004	22.98152	142.0534	22.95728	148.8558	23.16968	137.1861
22.97133	141.038	22.99435	134.2503	23.0574	135.9016	22.99818	142.0522	22.97395	148.8535	23.18635	137.1823

22.988	141.0353	23.01102	134.2512	23.07407	135.897	23.01485	142.0515	22.99062	148.8535	23.20302	137.1803
23.00467	141.033	23.02768	134.2461	23.09073	135.892	23.03152	142.0505	23.00728	148.8517	23.21968	137.1774
23.02133	141.0298	23.04435	134.2418	23.1074	135.8925	23.04818	142.0445	23.02395	148.8522	23.23635	137.1762
23.038	141.025	23.06102	134.2405	23.12407	135.8886	23.06485	142.0401	23.04062	148.8511	23.25302	137.1751
23.05467	141.0225	23.07768	134.2391	23.14073	135.8852	23.08152	142.0401	23.05728	148.8487	23.26968	137.1757
23.07133	141.0195	23.09435	134.2351	23.1574	135.8831	23.09818	142.0408	23.07395	148.851	23.28635	137.1715
23.088	141.0186	23.11102	134.2304	23.17407	135.8818	23.11485	142.0374	23.09062	148.8484	23.30302	137.1701
23.10467	141.0129	23.12768	134.2312	23.19073	135.8829	23.13152	142.0363	23.10728	148.8471	23.31968	137.1695
23.12133	141.0101	23.14435	134.2296	23.2074	135.8792	23.14818	142.0352	23.12395	148.8434	23.33635	137.1673
23.138	141.01	23.16102	134.2233	23.22407	135.8719	23.16485	142.0331	23.14062	148.8421	23.35302	137.1641
23.15467	141.0064	23.17768	134.222	23.24073	135.8729	23.18152	142.0316	23.15728	148.8436	23.36968	137.163
23.17133	141.0052	23.19435	134.2169	23.2574	135.8698	23.19818	142.032	23.17395	148.8438	23.38635	137.1619
23.188	141.001	23.21102	134.2151	23.27407	135.8689	23.21485	142.0292	23.19062	148.8407	23.40302	137.1609
23.20467	140.9996	23.22768	134.2131	23.29073	135.8666	23.23152	142.0286	23.20728	148.8388	23.41968	137.1598
23.22133	140.9968	23.24435	134.2095	23.3074	135.8674	23.24818	142.0269	23.22395	148.8374	23.43635	137.1584
23.238	140.9931	23.26102	134.2061	23.32407	135.8635	23.26485	142.0293	23.24062	148.8365	23.45302	137.1549
23.25467	140.9907	23.27768	134.203	23.34073	135.8599	23.28152	142.0238	23.25728	148.8373	23.46968	137.1541
23.27133	140.9917	23.29435	134.2043	23.3574	135.8566	23.29818	142.0229	23.27395	148.836	23.48635	137.1527
23.288	140.9884	23.31102	134.2002	23.37407	135.8569	23.31485	142.0244	23.29062	148.8332	23.50302	137.1535
23.30467	140.9862	23.32768	134.1987	23.39073	135.8517	23.33152	142.0211	23.30728	148.8339	23.51968	137.1484
23.32133	140.9826	23.34435	134.1964	23.4074	135.854	23.34818	142.0207	23.32395	148.8321	23.53635	137.1477
23.338	140.9802	23.36102	134.1921	23.42407	135.849	23.36485	142.0207	23.34062	148.8324	23.55302	137.1446
23.35467	140.977	23.37768	134.1874	23.44073	135.8445	23.38152	142.0158	23.35728	148.8317	23.56968	137.1451
23.37133	140.9745	23.39435	134.1867	23.4574	135.8444	23.39818	142.0173	23.37395	148.8326	23.58635	137.1421
23.388	140.9734	23.41102	134.1844	23.47407	135.8435	23.41485	142.0127	23.39062	148.83	23.60302	137.1407
23.40467	140.9677	23.42768	134.1799	23.49073	135.8367	23.43152	142.0137	23.40728	148.8312	23.61968	137.1404
23.42133	140.9655	23.44435	134.1761	23.5074	135.834	23.44818	142.013	23.42395	148.8286	23.63635	137.1387
23.438	140.9603	23.46102	134.1751	23.52407	135.8343	23.46485	142.0101	23.44062	148.8314	23.65302	137.1319
23.45467	140.9615	23.47768	134.173	23.54073	135.8309	23.48152	142.0083	23.45728	148.8297	23.66968	137.1351
23.47133	140.9578	23.49435	134.1724	23.5574	135.8263	23.49818	142.0097	23.47395	148.829	23.68635	137.133
23.488	140.9531	23.51102	134.167	23.57407	135.8259	23.51485	142.0082	23.49062	148.8283	23.70302	137.1303
23.50467	140.9483	23.52768	134.1646	23.59073	135.8234	23.53152	142.007	23.50728	148.8283	23.71968	137.1276
23.52133	140.9485	23.54435	134.1618	23.6074	135.8195	23.54818	142.0052	23.52395	148.8279	23.73635	137.1257
23.538	140.9455	23.56102	134.1591	23.62407	135.82	23.56485	142.0037	23.54062	148.8262	23.75302	137.125
23.55467	140.9426	23.57768	134.1566	23.64073	135.8171	23.58152	142.0035	23.55728	148.8222	23.76968	137.1231
23.57133	140.9404	23.59435	134.1549	23.6574	135.8141	23.59818	142.0041	23.57395	148.8258	23.78635	137.1194
23.588	140.938	23.61102	134.151	23.67407	135.8136	23.61485	142.0023	23.59062	148.8234	23.80302	137.1193
23.60467	140.9372	23.62768	134.1473	23.69073	135.8084	23.63152	142.0008	23.60728	148.8247	23.81968	137.1181
23.62133	140.9327	23.64435	134.1447	23.7074	135.8068	23.64818	141.9984	23.62395	148.8228	23.83635	137.1162
23.638	140.9314	23.66102	134.1431	23.72407	135.8034	23.66485	141.9977	23.64062	148.8207	23.85302	137.1115
23.65467	140.9318	23.67768	134.1432	23.74073	135.8035	23.68152	141.9982	23.65728	148.8227	23.86968	137.1124
23.67133	140.9289	23.69435	134.1381	23.7574	135.7981	23.69818	141.9966	23.67395	148.8217	23.88635	137.1096
23.688	140.9261	23.71102	134.1337	23.77407	135.7961	23.71485	141.9954	23.69062	148.8202	23.90302	137.1074
23.70467	140.9199	23.72768	134.1302	23.79073	135.7938	23.73152	141.9948	23.70728	148.8182	23.91968	137.1036
23.72133	140.9195	23.74435	134.1305	23.8074	135.7914	23.74818	141.9908	23.72395	148.8195	23.93635	137.1037
23.738	140.9143	23.76102	134.1276	23.82407	135.789	23.76485	141.9914	23.74062	148.8197	23.95302	137.0997
23.75467	140.9157	23.77768	134.1258	23.84073	135.787	23.78152	141.9911	23.75728	148.8175	23.96968	137.0985
23.77133	140.9108	23.79435	134.1197	23.8574	135.7848	23.79818	141.9924	23.77395	148.818	23.98635	137.0958
23.788	140.9036	23.81102	134.1192	23.87407	135.7824	23.81485	141.9901	23.79062	148.8176	24.00302	137.0937
23.80467	140.9065	23.82768	134.1158	23.89073	135.7796	23.83152	141.9883	23.80728	148.8145	24.01968	137.0898
23.82133	140.9027	23.84435	134.1143	23.9074	135.776	23.84818	141.9863	23.82395	148.8153	24.03635	137.0904
23.838	140.8987	23.86102	134.1121	23.92407	135.7736	23.86485	141.9836	23.84062	148.8138	24.05302	137.0874
23.85467	140.896	23.87768	134.1088	23.94073	135.7691	23.88152	141.9827	23.85728	148.8137	24.06968	137.0865
23.87133	140.8952	23.89435	134.1072	23.9574	135.7689	23.89818	141.9834	23.87395	148.8116	24.08635	137.085
23.888	140.8905	23.91102	134.1049	23.97407	135.7668	23.91485	141.9823	23.89062	148.8107	24.10302	137.0829
23.90467	140.8854	23.92768	134.0971	23.99073	135.7631	23.93152	141.981	23.90728	148.8119	24.11968	137.0802
23.92133	140.8849	23.94435	134.0971	24.0074	135.7631	23.94818	141.978	23.92395	148.8127	24.13635	137.0768
23.938	140.8809	23.96102	134.0976	24.02407	135.7614	23.96485	141.9798	23.94062	148.8082	24.15302	137.0724
23.95467	140.8779	23.97768	134.0901	24.04073	135.7559	23.98152	141.9779	23.95728	148.8084	24.16968	137.0723
23.97133	140.8733	23.99435	134.0877	24.0574	135.7526	23.99818	141.9762	23.97395	148.8082	24.18635	137.0714
23.988	140.8728	24.01102	134.0852	24.07407	135.7522	24.01485	141.9756	23.99062	148.8081	24.20302	137.0687
24.00467	140.8701	24.02768	134.0826	24.09073	135.7499	24.03152	141.9733	24.00728	148.8052	24.21968	137.066
24.02133	140.8656	24.04435	134.0799	24.1074	135.7474	24.04818	141.9729	24.02395	148.8038	24.23635	137.0664
24.038	140.8641	24.06102	134.0791	24.12407	135.7449	24.06485	141.9734	24.04062	148.8058	24.25302	137.0638
24.05467	140.8608	24.07768	134.0769	24.14073	135.7448	24.08152	141.9703	24.05728	148.8052	24.26968	137.06
24.07133	140.8588	24.09435	134.0744	24.1574	135.7415	24.09818	141.9687	24.07395	148.8066	24.28635	137.0568
24.088	140.859	24.11102	134.0689	24.17407	135.7393	24.11485	141.969	24.09062	148.8047	24.30302	137.0561
24.10467	140.8524	24.12768	134.066	24.19073	135.7376	24.13152	141.9654	24.10728	148.8019	24.31968	137.0523
24.12133	140.852	24.14435	134.0675	24.2074	135.7355	24.14818	141.9672	24.12395	148.8022	24.33635	137.0501

24.138	140.8516	24.16102	134.0633	24.22407	135.7295	24.16485	141.9657	24.14062	148.8029	24.35302	137.0477
24.15467	140.8485	24.17768	134.0625	24.24073	135.7293	24.18152	141.9624	24.15728	148.7992	24.36968	137.0466
24.17133	140.8416	24.19435	134.0574	24.2574	135.7275	24.19818	141.9615	24.17395	148.803	24.38635	137.0451
24.188	140.8413	24.21102	134.0591	24.27407	135.7257	24.21485	141.9614	24.19062	148.8019	24.40302	137.0411
24.20467	140.8392	24.22768	134.0518	24.29073	135.7284	24.23152	141.958	24.20728	148.7986	24.41968	137.0389
24.22133	140.8353	24.24435	134.0481	24.3074	135.7223	24.24818	141.9577	24.22395	148.7994	24.43635	137.0393
24.238	140.8321	24.26102	134.0469	24.32407	135.7214	24.26485	141.9574	24.24062	148.7997	24.45302	137.0355
24.25467	140.8318	24.27768	134.0441	24.34073	135.7178	24.28152	141.9551	24.25728	148.7985	24.46968	137.0337
24.27133	140.8248	24.29435	134.0419	24.3574	135.7148	24.29818	141.955	24.27395	148.7989	24.48635	137.0332
24.288	140.8256	24.31102	134.0407	24.37407	135.7108	24.31485	141.9542	24.29062	148.7958	24.50302	137.0301
24.30467	140.8224	24.32768	134.036	24.39073	135.7103	24.33152	141.9556	24.30728	148.7972	24.51968	137.0287
24.32133	140.817	24.34435	134.0362	24.4074	135.7091	24.34818	141.953	24.32395	148.7962	24.53635	137.0283
24.338	140.8157	24.36102	134.0315	24.42407	135.7062	24.36485	141.952	24.34062	148.7935	24.55302	137.0262
24.35467	140.8138	24.37768	134.0297	24.44073	135.7038	24.38152	141.9492	24.35728	148.7937	24.56968	137.0234
24.37133	140.8101	24.39435	134.0294	24.4574	135.7025	24.39818	141.9475	24.37395	148.7944	24.58635	137.0235
24.388	140.8089	24.41102	134.0299	24.47407	135.7012	24.41485	141.947	24.39062	148.7918	24.60302	137.0207
24.40467	140.8041	24.42768	134.0264	24.49073	135.7006	24.43152	141.9439	24.40728	148.7916	24.61968	137.0205
24.42133	140.804	24.44435	134.0221	24.5074	135.6934	24.44818	141.9454	24.42395	148.79	24.63635	137.0205
24.438	140.8018	24.46102	134.0204	24.52407	135.6901	24.46485	141.9446	24.44062	148.7908	24.65302	137.0187
24.45467	140.7978	24.47768	134.017	24.54073	135.6913	24.48152	141.9425	24.45728	148.7901	24.66968	137.015
24.47133	140.7944	24.49435	134.0137	24.5574	135.6882	24.49818	141.9427	24.47395	148.7901	24.68635	137.015
24.488	140.7927	24.51102	134.0123	24.57407	135.6841	24.51485	141.9413	24.49062	148.7891	24.70302	137.0123
24.50467	140.7917	24.52768	134.0077	24.59073	135.6806	24.53152	141.9402	24.50728	148.7893	24.71968	137.0105
24.52133	140.7891	24.54435	134.0062	24.6074	135.6798	24.54818	141.9375	24.52395	148.7858	24.73635	137.0096
24.538	140.7842	24.56102	134.0047	24.62407	135.6775	24.56485	141.9366	24.54062	148.788	24.75302	137.0067
24.55467	140.7796	24.57768	134.0009	24.64073	135.6741	24.58152	141.9382	24.55728	148.7872	24.76968	137.0057
24.57133	140.7775	24.59435	133.998	24.6574	135.6729	24.59818	141.9363	24.57395	148.7864	24.78635	137.0037
24.588	140.7758	24.61102	133.9974	24.67407	135.6706	24.61485	141.9327	24.59062	148.7838	24.80302	137.0046
24.60467	140.7743	24.62768	133.9935	24.69073	135.6665	24.63152	141.9357	24.60728	148.7853	24.81968	137.001
24.62133	140.7694	24.64435	133.9933	24.7074	135.6664	24.64818	141.9306	24.62395	148.7829	24.83635	137.0005
24.638	140.7694	24.66102	133.9882	24.72407	135.6662	24.66485	141.9285	24.64062	148.7827	24.85302	136.9967
24.65467	140.7674	24.67768	133.9851	24.74073	135.6624	24.68152	141.93	24.65728	148.7833	24.86968	136.9972
24.67133	140.7607	24.69435	133.9829	24.7574	135.6598	24.69818	141.929	24.67395	148.7803	24.88635	136.994
24.688	140.7584	24.71102	133.9851	24.77407	135.6575	24.71485	141.9274	24.69062	148.7815	24.90302	136.9907
24.70467	140.7544	24.72768	133.9786	24.79073	135.6558	24.73152	141.9246	24.70728	148.7794	24.91968	136.9911
24.72133	140.7574	24.74435	133.9783	24.8074	135.6528	24.74818	141.9235	24.72395	148.7805	24.93635	136.9884
24.738	140.753	24.76102	133.9755	24.82407	135.6506	24.76485	141.9235	24.74062	148.7769	24.95302	136.9864
24.75467	140.7486	24.77768	133.9732	24.84073	135.6471	24.78152	141.9227	24.75728	148.7754	24.96968	136.9828
24.77133	140.7501	24.79435	133.968	24.8574	135.645	24.79818	141.9217	24.77395	148.7758	24.98635	136.9848
24.788	140.7453	24.81102	133.9658	24.87407	135.6451	24.81485	141.9169	24.79062	148.7754	25.00302	136.9824
24.80467	140.7412	24.82768	133.9661	24.89073	135.6413	24.83152	141.9151	24.80728	148.774	25.01968	136.9795
24.82133	140.7415	24.84435	133.961	24.9074	135.6389	24.84818	141.9153	24.82395	148.7721	25.03635	136.9756
24.838	140.7345	24.86102	133.9599	24.92407	135.6391	24.86485	141.9149	24.84062	148.7713	25.05302	136.9761
24.85467	140.732	24.87768	133.9555	24.94073	135.6345	24.88152	141.9116	24.85728	148.7696	25.06968	136.9732
24.87133	140.7322	24.89435	133.9554	24.9574	135.632	24.89818	141.9116	24.87395	148.771	25.08635	136.9697
24.888	140.727	24.91102	133.95	24.97407	135.63	24.91485	141.9045	24.89062	148.7703	25.10302	136.9682
24.90467	140.724	24.92768	133.9486	24.99073	135.6271	24.93152	141.9061	24.90728	148.7681	25.11968	136.9683
24.92133	140.7243	24.94435	133.9464	25.0074	135.6268	24.94818	141.9067	24.92395	148.7688	25.13635	136.9643
24.938	140.7237	24.96102	133.9449	25.02407	135.6239	24.96485	141.9041	24.94062	148.7675	25.15302	136.9637
24.95467	140.7183	24.97768	133.9419	25.04073	135.6228	24.98152	141.902	24.95728	148.7675	25.16968	136.9604
24.97133	140.7163	24.99435	133.9379	25.0574	135.6207	24.99818	141.9031	24.97395	148.7666	25.18635	136.9612
24.988	140.7138	25.01102	133.9359	25.07407	135.6195	25.01485	141.8979	24.99062	148.7655	25.20302	136.9593
25.00467	140.7096	25.02768	133.9338	25.09073	135.6145	25.03152	141.8971	25.00728	148.7641	25.21968	136.956
25.02133	140.7073	25.04435	133.9306	25.1074	135.611	25.04818	141.8979	25.02395	148.7644	25.23635	136.9564
25.038	140.7037	25.06102	133.9268	25.12407	135.6117	25.06485	141.8961	25.04062	148.7647	25.25302	136.9548
25.05467	140.7054	25.07768	133.9259	25.14073	135.6102	25.08152	141.8948	25.05728	148.7604	25.26968	136.9543
25.07133	140.7029	25.09435	133.9221	25.1574	135.6065	25.09818	141.8931	25.07395	148.7613	25.28635	136.9526
25.088	140.6972	25.11102	133.9208	25.17407	135.6079	25.11485	141.8908	25.09062	148.7628	25.30302	136.9491
25.10467	140.6933	25.12768	133.9168	25.19073	135.6104	25.13152	141.8909	25.10728	148.7606	25.31968	136.9487
25.12133	140.6921	25.14435	133.9176	25.2074	135.6024	25.14818	141.889	25.12395	148.759	25.33635	136.9458
25.138	140.6903	25.16102	133.912	25.22407	135.5983	25.16485	141.8899	25.14062	148.756	25.35302	136.9452
25.15467	140.6887	25.17768	133.9057	25.24073	135.5979	25.18152	141.8876	25.15728	148.7583	25.36968	136.941
25.17133	140.6813	25.19435	133.9037	25.2574	135.5939	25.19818	141.8872	25.17395	148.7587	25.38635	136.9402
25.188	140.6804	25.21102	133.9052	25.27407	135.5931	25.21485	141.8853	25.19062	148.7555	25.40302	136.9387
25.20467	140.6801	25.22768	133.9025	25.29073	135.5913	25.23152	141.8866	25.20728	148.7558	25.41968	136.9374
25.22133	140.6776	25.24435	133.903	25.3074	135.5872	25.24818	141.8863	25.22395	148.7564	25.43635	136.9353
25.238	140.6737	25.26102	133.9059	25.32407	135.584	25.26485	141.8836	25.24062	148.7503	25.45302	136.934
25.25467	140.6751	25.27768	133.8923	25.34073	135.5845	25.28152	141.8811	25.25728	148.7535	25.46968	136.9314
25.27133	140.6697	25.29435	133.8949	25.3574	135.5847	25.29818	141.8833	25.27395	148.7545	25.48635	136.9291

25.288	140.6693	25.31102	133.8919	25.37407	135.5819	25.31485	141.8781	25.29062	148.7553	25.50302	136.9299
25.30467	140.6625	25.32768	133.8895	25.39073	135.5805	25.33152	141.8784	25.30728	148.7537	25.51968	136.9279
25.32133	140.6645	25.34435	133.8889	25.4074	135.5774	25.34818	141.8769	25.32395	148.7515	25.53635	136.9265
25.338	140.6599	25.36102	133.8813	25.42407	135.577	25.36485	141.8748	25.34062	148.7528	25.55302	136.923
25.35467	140.6558	25.37768	133.8842	25.44073	135.5734	25.38152	141.8737	25.35728	148.7519	25.56968	136.9217
25.37133	140.656	25.39435	133.8818	25.4574	135.5709	25.39818	141.8722	25.37395	148.7533	25.58635	136.9186
25.388	140.6501	25.41102	133.8775	25.47407	135.5703	25.41485	141.872	25.39062	148.7508	25.60302	136.9163
25.40467	140.649	25.42768	133.8781	25.49073	135.5662	25.43152	141.8711	25.40728	148.7504	25.61968	136.9148
25.42133	140.6439	25.44435	133.8735	25.5074	135.5663	25.44818	141.8698	25.42395	148.7513	25.63635	136.9135
25.438	140.6421	25.46102	133.8696	25.52407	135.5644	25.46485	141.8716	25.44062	148.7482	25.65302	136.9134
25.45467	140.6433	25.47768	133.8668	25.54073	135.563	25.48152	141.8668	25.45728	148.7491	25.66968	136.9117
25.47133	140.64	25.49435	133.8645	25.5574	135.5619	25.49818	141.8685	25.47395	148.7471	25.68635	136.9103
25.488	140.6351	25.51102	133.862	25.57407	135.5551	25.51485	141.8689	25.49062	148.746	25.70302	136.9075
25.50467	140.634	25.52768	133.8593	25.59073	135.5568	25.53152	141.8659	25.50728	148.7442	25.71968	136.9041
25.52133	140.6331	25.54435	133.8595	25.6074	135.5547	25.54818	141.8618	25.52395	148.7441	25.73635	136.9043
25.538	140.6322	25.56102	133.8597	25.62407	135.5489	25.56485	141.8619	25.54062	148.7439	25.75302	136.9012
25.55467	140.6311	25.57768	133.8559	25.64073	135.5475	25.58152	141.8621	25.55728	148.7432	25.76968	136.9011
25.57133	140.6267	25.59435	133.8507	25.6574	135.5455	25.59818	141.8617	25.57395	148.7441	25.78635	136.8974
25.588	140.6248	25.61102	133.8508	25.67407	135.5459	25.61485	141.8592	25.59062	148.7438	25.80302	136.8973
25.60467	140.6226	25.62768	133.8452	25.69073	135.5401	25.63152	141.8625	25.60728	148.7425	25.81968	136.8945
25.62133	140.6158	25.64435	133.8435	25.7074	135.5359	25.64818	141.8588	25.62395	148.74	25.83635	136.892
25.638	140.6143	25.66102	133.8426	25.72407	135.5367	25.66485	141.8572	25.64062	148.7414	25.85302	136.8916
25.65467	140.612	25.67768	133.8393	25.74073	135.5318	25.68152	141.8558	25.65728	148.7416	25.86968	136.8914
25.67133	140.6074	25.69435	133.8375	25.7574	135.5302	25.69818	141.8552	25.67395	148.7375	25.88635	136.8899
25.688	140.6082	25.71102	133.8382	25.77407	135.5266	25.71485	141.8584	25.69062	148.7403	25.90302	136.8892
25.70467	140.6029	25.72768	133.8341	25.79073	135.5274	25.73152	141.8543	25.70728	148.7342	25.91968	136.8852
25.72133	140.6053	25.74435	133.8306	25.8074	135.5258	25.74818	141.8517	25.72395	148.7353	25.93635	136.8843
25.738	140.5984	25.76102	133.829	25.82407	135.5216	25.76485	141.8513	25.74062	148.7376	25.95302	136.8827
25.75467	140.597	25.77768	133.8264	25.84073	135.5187	25.78152	141.8529	25.75728	148.7368	25.96968	136.8838
25.77133	140.5944	25.79435	133.8226	25.8574	135.5174	25.79818	141.8493	25.77395	148.7374	25.98635	136.8788
25.788	140.5932	25.81102	133.8217	25.87407	135.515	25.81485	141.8512	25.79062	148.7312	26.00302	136.8784
25.80467	140.5867	25.82768	133.8189	25.89073	135.5138	25.83152	141.8472	25.80728	148.7346	26.01968	136.8759
25.82133	140.5844	25.84435	133.8163	25.9074	135.5103	25.84818	141.8477	25.82395	148.7339	26.03635	136.8743
25.838	140.5839	25.86102	133.8148	25.92407	135.5091	25.86485	141.8477	25.84062	148.7325	26.05302	136.8723
25.85467	140.5806	25.87768	133.8124	25.94073	135.5073	25.88152	141.8472	25.85728	148.732	26.06968	136.8713
25.87133	140.5795	25.89435	133.8083	25.9574	135.5057	25.89818	141.8453	25.87395	148.7328	26.08635	136.8672
25.888	140.5755	25.91102	133.8061	25.97407	135.5031	25.91485	141.8425	25.89062	148.7314	26.10302	136.866
25.90467	140.572	25.92768	133.804	25.99073	135.4988	25.93152	141.843	25.90728	148.7282	26.11968	136.8641
25.92133	140.5685	25.94435	133.802	26.0074	135.4989	25.94818	141.8407	25.92395	148.729	26.13635	136.8617
25.938	140.5666	25.96102	133.7987	26.02407	135.4964	25.96485	141.8383	25.94062	148.7307	26.15302	136.8582
25.95467	140.5664	25.97768	133.7968	26.04073	135.4949	25.98152	141.8381	25.95728	148.7293	26.16968	136.8582
25.97133	140.5623	25.99435	133.7934	26.0574	135.4884	25.99818	141.839	25.97395	148.7298	26.18635	136.8553
25.988	140.5599	26.01102	133.7936	26.07407	135.4894	26.01485	141.8369	25.99062	148.7305	26.20302	136.854
26.00467	140.558	26.02768	133.7904	26.09073	135.4871	26.03152	141.8341	26.00728	148.7243	26.21968	136.8508
26.02133	140.5526	26.04435	133.787	26.1074	135.4842	26.04818	141.8324	26.02395	148.7249	26.23635	136.8524
26.038	140.5529	26.06102	133.7852	26.12407	135.4815	26.06485	141.8325	26.04062	148.7267	26.25302	136.8476
26.05467	140.5489	26.07768	133.7813	26.14073	135.48	26.08152	141.8332	26.05728	148.7274	26.26968	136.8477
26.07133	140.5443	26.09435	133.7787	26.1574	135.4775	26.09818	141.8313	26.07395	148.7226	26.28635	136.8445
26.088	140.5452	26.11102	133.7746	26.17407	135.4754	26.11485	141.8299	26.09062	148.7265	26.30302	136.8429
26.10467	140.5428	26.12768	133.7718	26.19073	135.4741	26.13152	141.8316	26.10728	148.7244	26.31968	136.8428
26.12133	140.5386	26.14435	133.7698	26.2074	135.4723	26.14818	141.826	26.12395	148.7265	26.33635	136.8407
26.138	140.5333	26.16102	133.7678	26.22407	135.4669	26.16485	141.8266	26.14062	148.7206	26.35302	136.8405
26.15467	140.5341	26.17768	133.7649	26.24073	135.4658	26.18152	141.8269	26.15728	148.7189	26.36968	136.8378
26.17133	140.5323	26.19435	133.7629	26.2574	135.4654	26.19818	141.8253	26.17395	148.7226	26.38635	136.8375
26.188	140.5306	26.21102	133.7613	26.27407	135.4617	26.21485	141.826	26.19062	148.7224	26.40302	136.8339
26.20467	140.5247	26.22768	133.7572	26.29073	135.4586	26.23152	141.825	26.20728	148.7199	26.41968	136.835
26.22133	140.5235	26.24435	133.7552	26.3074	135.4586	26.24818	141.8216	26.22395	148.7217	26.43635	136.8342
26.238	140.5223	26.26102	133.7529	26.32407	135.456	26.26485	141.8202	26.24062	148.7203	26.45302	136.8306
26.25467	140.5186	26.27768	133.7498	26.34073	135.4507	26.28152	141.8208	26.25728	148.7172	26.46968	136.8289
26.27133	140.5162	26.29435	133.7476	26.3574	135.4478	26.29818	141.8191	26.27395	148.7167	26.48635	136.8323
26.288	140.5108	26.31102	133.7418	26.37407	135.4493	26.31485	141.8171	26.29062	148.7179	26.50302	136.8217
26.30467	140.5104	26.32768	133.7427	26.39073	135.4476	26.33152	141.8174	26.30728	148.7146	26.51968	136.8223
26.32133	140.5091	26.34435	133.7417	26.4074	135.4457	26.34818	141.8161	26.32395	148.7181	26.53635	136.8232
26.338	140.5062	26.36102	133.7382	26.42407	135.4432	26.36485	141.8141	26.34062	148.7181	26.55302	136.8231
26.35467	140.5041	26.37768	133.7355	26.44073	135.4399	26.38152	141.8146	26.35728	148.7129	26.56968	136.8176
26.37133	140.5012	26.39435	133.7314	26.4574	135.4384	26.39818	141.811	26.37395	148.7195	26.58635	136.8177
26.388	140.4969	26.41102	133.7305	26.47407	135.4396	26.41485	141.8126	26.39062	148.7081	26.60302	136.8174
26.40467	140.4888	26.42768	133.7262	26.49073	135.4346	26.43152	141.8116	26.40728	148.7115	26.61968	136.8156
26.42133	140.4926	26.44435	133.7219	26.5074	135.4351	26.44818	141.807	26.42395	148.7137	26.63635	136.8126

26.438	140.4903	26.46102	133.7176	26.52407	135.4306	26.46485	141.8087	26.44062	148.7146	26.65302	136.811
26.45467	140.4876	26.47768	133.716	26.54073	135.4295	26.48152	141.8085	26.45728	148.712	26.66968	136.8094
26.47133	140.484	26.49435	133.7165	26.5574	135.4252	26.49818	141.8066	26.47395	148.7131	26.68635	136.8104
26.488	140.4822	26.51102	133.711	26.57407	135.4255	26.51485	141.8066	26.49062	148.7112	26.70302	136.8062
26.50467	140.4779	26.52768	133.7098	26.59073	135.4214	26.53152	141.8052	26.50728	148.7121	26.71968	136.8073
26.52133	140.4786	26.54435	133.7111	26.6074	135.418	26.54818	141.801	26.52395	148.7098	26.73635	136.8
26.538	140.4748	26.56102	133.7087	26.62407	135.4161	26.56485	141.7976	26.54062	148.7092	26.75302	136.8023
26.55467	140.4702	26.57768	133.7044	26.64073	135.4144	26.58152	141.8015	26.55728	148.7059	26.76968	136.7997
26.57133	140.4697	26.59435	133.7015	26.6574	135.4138	26.59818	141.7968	26.57395	148.708	26.78635	136.7986
26.588	140.4699	26.61102	133.7004	26.67407	135.4121	26.61485	141.8002	26.59062	148.7093	26.80302	136.7998
26.60467	140.4608	26.62768	133.6953	26.69073	135.4144	26.63152	141.7971	26.60728	148.7086	26.81968	136.7955
26.62133	140.4606	26.64435	133.6922	26.7074	135.4084	26.64818	141.794	26.62395	148.7056	26.83635	136.7921
26.638	140.4598	26.66102	133.6905	26.72407	135.4068	26.66485	141.7974	26.64062	148.7066	26.85302	136.7903
26.65467	140.4569	26.67768	133.6912	26.74073	135.4063	26.68152	141.7932	26.65728	148.7056	26.86968	136.7919
26.67133	140.4532	26.69435	133.6863	26.7574	135.4038	26.69818	141.7927	26.67395	148.7053	26.88635	136.7879
26.688	140.4527	26.71102	133.6827	26.77407	135.4015	26.71485	141.7999	26.69062	148.7046	26.90302	136.787
26.70467	140.4498	26.72768	133.6827	26.79073	135.3997	26.73152	141.7879	26.70728	148.7047	26.91968	136.7832
26.72133	140.4429	26.74435	133.6774	26.8074	135.4014	26.74818	141.7877	26.72395	148.7034	26.93635	136.7824
26.738	140.4465	26.76102	133.6773	26.82407	135.3993	26.76485	141.7888	26.74062	148.703	26.95302	136.7759
26.75467	140.4387	26.77768	133.6746	26.84073	135.3981	26.78152	141.7898	26.75728	148.7023	26.96968	136.7754
26.77133	140.437	26.79435	133.6723	26.8574	135.3929	26.79818	141.7889	26.77395	148.7028	26.98635	136.7739
26.788	140.4336	26.81102	133.6679	26.87407	135.3943	26.81485	141.7869	26.79062	148.7002	27.00302	136.7725
26.80467	140.4357	26.82768	133.6676	26.89073	135.3881	26.83152	141.7846	26.80728	148.7012	27.01968	136.7692
26.82133	140.428	26.84435	133.6664	26.9074	135.3855	26.84818	141.7824	26.82395	148.7019	27.03635	136.77
26.838	140.4278	26.86102	133.6644	26.92407	135.3833	26.86485	141.7848	26.84062	148.7	27.05302	136.7654
26.85467	140.4284	26.87768	133.6616	26.94073	135.3834	26.88152	141.7823	26.85728	148.6992	27.06968	136.7642
26.87133	140.423	26.89435	133.6618	26.9574	135.3797	26.89818	141.7796	26.87395	148.6994	27.08635	136.7613
26.888	140.4205	26.91102	133.6556	26.97407	135.3822	26.91485	141.78	26.89062	148.6981	27.10302	136.7621
26.90467	140.4175	26.92768	133.6527	26.99073	135.3747	26.93152	141.7776	26.90728	148.6999	27.11968	136.7588
26.92133	140.4185	26.94435	133.6509	27.0074	135.3707	26.94818	141.7776	26.92395	148.6972	27.13635	136.7573
26.938	140.4143	26.96102	133.6483	27.02407	135.3731	26.96485	141.7772	26.94062	148.699	27.15302	136.7522
26.95467	140.4133	26.97768	133.6472	27.04073	135.3692	26.98152	141.7776	26.95728	148.696	27.16968	136.7543
26.97133	140.4077	26.99435	133.6462	27.0574	135.3692	26.99818	141.7755	26.97395	148.6979	27.18635	136.749
26.988	140.406	27.01102	133.6424	27.07407	135.3673	27.01485	141.7757	26.99062	148.6958	27.20302	136.7487
27.00467	140.4054	27.02768	133.6409	27.09073	135.3647	27.03152	141.7715	27.00728	148.6952	27.21968	136.7466
27.02133	140.4013	27.04435	133.641	27.1074	135.3607	27.04818	141.7711	27.02395	148.6963	27.23635	136.7441
27.038	140.3984	27.06102	133.6381	27.12407	135.361	27.06485	141.7712	27.04062	148.696	27.25302	136.7421
27.05467	140.3954	27.07768	133.6353	27.14073	135.3592	27.08152	141.7703	27.05728	148.6953	27.26968	136.7397
27.07133	140.3936	27.09435	133.6361	27.1574	135.356	27.09818	141.7709	27.07395	148.6949	27.28635	136.7373
27.088	140.392	27.11102	133.6347	27.17407	135.3543	27.11485	141.7684	27.09062	148.6933	27.30302	136.736
27.10467	140.3878	27.12768	133.6245	27.19073	135.3529	27.13152	141.7667	27.10728	148.6924	27.31968	136.737
27.12133	140.3885	27.14435	133.6286	27.2074	135.3551	27.14818	141.7639	27.12395	148.6895	27.33635	136.7317
27.138	140.3842	27.16102	133.6256	27.22407	135.3521	27.16485	141.7668	27.14062	148.6925	27.35302	136.7291
27.15467	140.3767	27.17768	133.6212	27.24073	135.3479	27.18152	141.7633	27.15728	148.6901	27.36968	136.7281
27.17133	140.3789	27.19435	133.6237	27.2574	135.3467	27.19818	141.7641	27.17395	148.6902	27.38635	136.7273
27.188	140.3759	27.21102	133.6166	27.27407	135.3441	27.21485	141.7619	27.19062	148.6902	27.40302	136.7235
27.20467	140.3741	27.22768	133.6127	27.29073	135.3457	27.23152	141.7591	27.20728	148.6894	27.41968	136.7238
27.22133	140.3722	27.24435	133.6123	27.3074	135.3422	27.24818	141.7589	27.22395	148.6885	27.43635	136.7209
27.238	140.3679	27.26102	133.6065	27.32407	135.3368	27.26485	141.7588	27.24062	148.6875	27.45302	136.7213
27.25467	140.3649	27.27768	133.6072	27.34073	135.3388	27.28152	141.7589	27.25728	148.6864	27.46968	136.7166
27.27133	140.3653	27.29435	133.6071	27.3574	135.3376	27.29818	141.7594	27.27395	148.6822	27.48635	136.7153
27.288	140.3607	27.31102	133.6024	27.37407	135.336	27.31485	141.7568	27.29062	148.683	27.50302	136.7154
27.30467	140.3553	27.32768	133.5969	27.39073	135.3343	27.33152	141.7557	27.30728	148.685	27.51968	136.7119
27.32133	140.3548	27.34435	133.5971	27.4074	135.33	27.34818	141.7533	27.32395	148.6827	27.53635	136.7127
27.338	140.3531	27.36102	133.5961	27.42407	135.3282	27.36485	141.7541	27.34062	148.6822	27.55302	136.708
27.35467	140.3547	27.37768	133.5911	27.44073	135.3267	27.38152	141.7511	27.35728	148.68	27.56968	136.708
27.37133	140.3454	27.39435	133.5877	27.4574	135.3275	27.39818	141.7496	27.37395	148.6824	27.58635	136.7068
27.388	140.345	27.41102	133.586	27.47407	135.325	27.41485	141.7529	27.39062	148.6813	27.60302	136.7038
27.40467	140.3474	27.42768	133.5819	27.49073	135.3224	27.43152	141.7467	27.40728	148.6814	27.61968	136.7022
27.42133	140.3439	27.44435	133.581	27.5074	135.3224	27.44818	141.7486	27.42395	148.6776	27.63635	136.7001
27.438	140.3386	27.46102	133.5814	27.52407	135.3162	27.46485	141.7476	27.44062	148.6768	27.65302	136.6983
27.45467	140.3368	27.47768	133.5785	27.54073	135.3164	27.48152	141.7468	27.45728	148.6778	27.66968	136.6953
27.47133	140.3323	27.49435	133.5778	27.5574	135.3163	27.49818	141.7461	27.47395	148.6767	27.68635	136.6934
27.488	140.3301	27.51102	133.5715	27.57407	135.3142	27.51485	141.7455	27.49062	148.6784	27.70302	136.6912
27.50467	140.3308	27.52768	133.5715	27.59073	135.3145	27.53152	141.7447	27.50728	148.6769	27.71968	136.6866
27.52133	140.3288	27.54435	133.5671	27.6074	135.3098	27.54818	141.7425	27.52395	148.6771	27.73635	136.6885
27.538	140.3249	27.56102	133.5657	27.62407	135.305	27.56485	141.7412	27.54062	148.6757	27.75302	136.6883
27.55467	140.3248	27.57768	133.56	27.64073	135.3041	27.58152	141.7404	27.55728	148.6728	27.76968	136.6865
27.57133	140.3215	27.59435	133.5581	27.6574	135.3037	27.59818	141.7382	27.57395	148.6711	27.78635	136.686

27.588	140.3204	27.61102	133.5576	27.67407	135.302	27.61485	141.7373	27.59062	148.6738	27.80302	136.6837
27.60467	140.3158	27.62768	133.5552	27.69073	135.3008	27.63152	141.7377	27.60728	148.6727	27.81968	136.6812
27.62133	140.3152	27.64435	133.5548	27.7074	135.2988	27.64818	141.7375	27.62395	148.6746	27.83635	136.6819
27.638	140.3118	27.66102	133.5525	27.72407	135.2941	27.66485	141.7345	27.64062	148.6741	27.85302	136.6786
27.65467	140.3077	27.67768	133.5488	27.74073	135.2926	27.68152	141.7326	27.65728	148.6719	27.86968	136.6803
27.67133	140.3057	27.69435	133.5478	27.7574	135.2911	27.69818	141.734	27.67395	148.6733	27.88635	136.6763
27.688	140.3053	27.71102	133.5422	27.77407	135.2871	27.71485	141.7295	27.69062	148.6712	27.90302	136.675
27.70467	140.3013	27.72768	133.5416	27.79073	135.2867	27.73152	141.7292	27.70728	148.6699	27.91968	136.676
27.72133	140.2985	27.74435	133.5391	27.8074	135.2868	27.74818	141.729	27.72395	148.667	27.93635	136.6716
27.738	140.2962	27.76102	133.5346	27.82407	135.2807	27.76485	141.7249	27.74062	148.6707	27.95302	136.6719
27.75467	140.2931	27.77768	133.5353	27.84073	135.281	27.78152	141.729	27.75728	148.6686	27.96968	136.6681
27.77133	140.293	27.79435	133.533	27.8574	135.2796	27.79818	141.7245	27.77395	148.6693	27.98635	136.6696
27.788	140.2918	27.81102	133.5317	27.87407	135.2784	27.81485	141.7234	27.79062	148.667	28.00302	136.6707
27.80467	140.2848	27.82768	133.5271	27.89073	135.2771	27.83152	141.7236	27.80728	148.6687	28.01968	136.6656
27.82133	140.2845	27.84435	133.526	27.9074	135.2757	27.84818	141.7202	27.82395	148.6662	28.03635	136.6654
27.838	140.2791	27.86102	133.5203	27.92407	135.2705	27.86485	141.7212	27.84062	148.6646	28.05302	136.6641
27.85467	140.2787	27.87768	133.5176	27.94073	135.272	27.88152	141.7192	27.85728	148.664	28.06968	136.661
27.87133	140.2763	27.89435	133.5191	27.9574	135.2707	27.89818	141.7187	27.87395	148.6651	28.08635	136.6589
27.888	140.2741	27.91102	133.5148	27.97407	135.2694	27.91485	141.7163	27.89062	148.666	28.10302	136.6558
27.90467	140.2722	27.92768	133.5122	27.99073	135.2641	27.93152	141.7142	27.90728	148.6636	28.11968	136.6545
27.92133	140.2689	27.94435	133.5101	28.0074	135.2647	27.94818	141.7138	27.92395	148.658	28.13635	136.6542
27.938	140.2702	27.96102	133.5113	28.02407	135.2643	27.96485	141.7134	27.94062	148.6573	28.15302	136.6523
27.95467	140.2663	27.97768	133.5049	28.04073	135.2595	27.98152	141.7135	27.95728	148.6622	28.16968	136.652
27.97133	140.2602	27.99435	133.5019	28.0574	135.2596	27.99818	141.7119	27.97395	148.6634	28.18635	136.6513
27.988	140.2625	28.01102	133.4998	28.07407	135.2585	28.01485	141.7086	27.99062	148.6615	28.20302	136.6504
28.00467	140.2592	28.02768	133.4983	28.09073	135.2558	28.03152	141.7075	28.00728	148.6631	28.21968	136.6474
28.02133	140.257	28.04435	133.4965	28.1074	135.2571	28.04818	141.7091	28.02395	148.6601	28.23635	136.6463
28.038	140.2566	28.06102	133.4955	28.12407	135.2537	28.06485	141.7089	28.04062	148.657	28.25302	136.6447
28.05467	140.255	28.07768	133.4923	28.14073	135.2519	28.08152	141.7046	28.05728	148.6576	28.26968	136.6434
28.07133	140.2524	28.09435	133.4897	28.1574	135.2516	28.09818	141.7052	28.07395	148.6605	28.28635	136.6403
28.088	140.2494	28.11102	133.4881	28.17407	135.2494	28.11485	141.7029	28.09062	148.6573	28.30302	136.6413
28.10467	140.2452	28.12768	133.4863	28.19073	135.2451	28.13152	141.7013	28.10728	148.6598	28.31968	136.6401
28.12133	140.2449	28.14435	133.4823	28.2074	135.2467	28.14818	141.7012	28.12395	148.6588	28.33635	136.6388
28.138	140.2428	28.16102	133.4806	28.22407	135.2422	28.16485	141.7007	28.14062	148.6583	28.35302	136.6367
28.15467	140.2366	28.17768	133.4772	28.24073	135.2438	28.18152	141.6977	28.15728	148.6572	28.36968	136.6374
28.17133	140.2382	28.19435	133.4756	28.2574	135.2388	28.19818	141.7003	28.17395	148.6586	28.38635	136.6338
28.188	140.2331	28.21102	133.4729	28.27407	135.2378	28.21485	141.696	28.19062	148.6571	28.40302	136.6359
28.20467	140.2324	28.22768	133.4726	28.29073	135.2366	28.23152	141.6958	28.20728	148.6586	28.41968	136.6337
28.22133	140.2299	28.24435	133.4677	28.3074	135.2313	28.24818	141.6945	28.22395	148.6566	28.43635	136.6327
28.238	140.2279	28.26102	133.4695	28.32407	135.2356	28.26485	141.6959	28.24062	148.6546	28.45302	136.6316
28.25467	140.2269	28.27768	133.4659	28.34073	135.2297	28.28152	141.6941	28.25728	148.6549	28.46968	136.6293
28.27133	140.2234	28.29435	133.4626	28.3574	135.2281	28.29818	141.6934	28.27395	148.6548	28.48635	136.6282
28.288	140.2228	28.31102	133.4622	28.37407	135.2277	28.31485	141.6939	28.29062	148.6517	28.50302	136.6268
28.30467	140.2199	28.32768	133.4594	28.39073	135.2241	28.33152	141.6915	28.30728	148.6538	28.51968	136.625
28.32133	140.212	28.34435	133.4575	28.4074	135.2228	28.34818	141.6903	28.32395	148.6534	28.53635	136.6227
28.338	140.2127	28.36102	133.4559	28.42407	135.2248	28.36485	141.6894	28.34062	148.652	28.55302	136.6215
28.35467	140.2109	28.37768	133.451	28.44073	135.2199	28.38152	141.6867	28.35728	148.6525	28.56968	136.6188
28.37133	140.2089	28.39435	133.4503	28.4574	135.218	28.39818	141.6878	28.37395	148.6536	28.58635	136.6179
28.388	140.2052	28.41102	133.4479	28.47407	135.2173	28.41485	141.6897	28.39062	148.6511	28.60302	136.6152
28.40467	140.2039	28.42768	133.4461	28.49073	135.2182	28.43152	141.6858	28.40728	148.6494	28.61968	136.6146
28.42133	140.2003	28.44435	133.4432	28.5074	135.2137	28.44818	141.6847	28.42395	148.6502	28.63635	136.6148
28.438	140.2016	28.46102	133.4423	28.52407	135.2135	28.46485	141.6848	28.44062	148.65	28.65302	136.6112
28.45467	140.1981	28.47768	133.44	28.54073	135.2128	28.48152	141.6835	28.45728	148.6488	28.66968	136.6108
28.47133	140.1944	28.49435	133.4381	28.5574	135.2062	28.49818	141.682	28.47395	148.6489	28.68635	136.6072
28.488	140.1921	28.51102	133.4367	28.57407	135.2036	28.51485	141.6831	28.49062	148.6477	28.70302	136.607
28.50467	140.1894	28.52768	133.4348	28.59073	135.2033	28.53152	141.6808	28.50728	148.6481	28.71968	136.6085
28.52133	140.1871	28.54435	133.4312	28.6074	135.2049	28.54818	141.6777	28.52395	148.648	28.73635	136.6054
28.538	140.1886	28.56102	133.4323	28.62407	135.1988	28.56485	141.6796	28.54062	148.6467	28.75302	136.6017
28.55467	140.1824	28.57768	133.4312	28.64073	135.1975	28.58152	141.6766	28.55728	148.6471	28.76968	136.6016
28.57133	140.1797	28.59435	133.4265	28.6574	135.1968	28.59818	141.6752	28.57395	148.6434	28.78635	136.6023
28.588	140.1759	28.61102	133.4242	28.67407	135.1955	28.61485	141.675	28.59062	148.6421	28.80302	136.6004
28.60467	140.1756	28.62768	133.4203	28.69073	135.1989	28.63152	141.6749	28.60728	148.6454	28.81968	136.5986
28.62133	140.1736	28.64435	133.4177	28.7074	135.1938	28.64818	141.6723	28.62395	148.6442	28.83635	136.5964
28.638	140.1699	28.66102	133.4167	28.72407	135.1918	28.66485	141.6704	28.64062	148.6443	28.85302	136.5963
28.65467	140.1686	28.67768	133.4163	28.74073	135.19	28.68152	141.6724	28.65728	148.6457	28.86968	136.5952
28.67133	140.1637	28.69435	133.4129	28.7574	135.1897	28.69818	141.6744	28.67395	148.6426	28.88635	136.5932
28.688	140.1631	28.71102	133.4118	28.77407	135.1887	28.71485	141.6691	28.69062	148.6434	28.90302	136.5908
28.70467	140.1617	28.72768	133.4072	28.79073	135.1874	28.73152	141.6699	28.70728	148.6403	28.91968	136.5886
28.72133	140.1569	28.74435	133.4069	28.8074	135.1838	28.74818	141.6699	28.72395	148.6415	28.93635	136.5882

28.738	140.1552	28.76102	133.4043	28.82407	135.1818	28.76485	141.6641	28.74062	148.6427	28.95302	136.588
28.75467	140.1546	28.77768	133.4017	28.84073	135.1811	28.78152	141.6649	28.75728	148.6394	28.96968	136.5854
28.77133	140.1508	28.79435	133.4014	28.8574	135.1766	28.79818	141.6635	28.77395	148.6388	28.98635	136.5821
28.788	140.1495	28.81102	133.3955	28.87407	135.178	28.81485	141.6608	28.79062	148.6375	29.00302	136.5828
28.80467	140.1469	28.82768	133.3943	28.89073	135.1781	28.83152	141.6652	28.80728	148.637	29.01968	136.5812
28.82133	140.1425	28.84435	133.3928	28.9074	135.1754	28.84818	141.6637	28.82395	148.641	29.03635	136.578
28.838	140.1425	28.86102	133.3901	28.92407	135.1722	28.86485	141.6611	28.84062	148.6384	29.05302	136.5763
28.85467	140.1412	28.87768	133.3882	28.94073	135.1702	28.88152	141.6595	28.85728	148.6366	29.06968	136.5736
28.87133	140.1357	28.89435	133.387	28.9574	135.1683	28.89818	141.6603	28.87395	148.6378	29.08635	136.5735
28.888	140.1326	28.91102	133.3857	28.97407	135.1652	28.91485	141.6586	28.89062	148.6365	29.10302	136.5714
28.90467	140.1301	28.92768	133.3825	28.99073	135.165	28.93152	141.6571	28.90728	148.6324	29.11968	136.5688
28.92133	140.1297	28.94435	133.3787	29.0074	135.1623	28.94818	141.6577	28.92395	148.6343	29.13635	136.5682
28.938	140.1269	28.96102	133.3786	29.02407	135.1621	28.96485	141.6558	28.94062	148.6352	29.15302	136.5668
28.95467	140.1251	28.97768	133.3759	29.04073	135.1589	28.98152	141.657	28.95728	148.6341	29.16968	136.5655
28.97133	140.1216	28.99435	133.3728	29.0574	135.1601	28.99818	141.655	28.97395	148.6326	29.18635	136.5618
28.988	140.1205	29.01102	133.3701	29.07407	135.1559	29.01485	141.6536	28.99062	148.6326	29.20302	136.564
29.00467	140.1185	29.02768	133.3684	29.09073	135.1561	29.03152	141.6528	29.00728	148.6302	29.21968	136.5594
29.02133	140.1161	29.04435	133.3669	29.1074	135.1525	29.04818	141.6524	29.02395	148.6302	29.23635	136.5593
29.038	140.1122	29.06102	133.3649	29.12407	135.1491	29.06485	141.6514	29.04062	148.632	29.25302	136.5563
29.05467	140.1126	29.07768	133.3599	29.14073	135.1473	29.08152	141.6509	29.05728	148.6296	29.26968	136.5594
29.07133	140.1084	29.09435	133.3587	29.1574	135.1462	29.09818	141.6512	29.07395	148.6274	29.28635	136.5558
29.088	140.1087	29.11102	133.3571	29.17407	135.1443	29.11485	141.6483	29.09062	148.6309	29.30302	136.5525
29.10467	140.1062	29.12768	133.3584	29.19073	135.1411	29.13152	141.6486	29.10728	148.6277	29.31968	136.5514
29.12133	140.1025	29.14435	133.353	29.2074	135.1405	29.14818	141.6472	29.12395	148.6275	29.33635	136.5464
29.138	140.1018	29.16102	133.3523	29.22407	135.1389	29.16485	141.6485	29.14062	148.6271	29.35302	136.5491
29.15467	140.0977	29.17768	133.3508	29.24073	135.1361	29.18152	141.6459	29.15728	148.6258	29.36968	136.5458
29.17133	140.0959	29.19435	133.3466	29.2574	135.135	29.19818	141.6458	29.17395	148.6265	29.38635	136.5462
29.188	140.0921	29.21102	133.3451	29.27407	135.1323	29.21485	141.6461	29.19062	148.6252	29.40302	136.5426
29.20467	140.09	29.22768	133.3411	29.29073	135.1329	29.23152	141.6441	29.20728	148.6265	29.41968	136.5443
29.22133	140.0889	29.24435	133.3389	29.3074	135.1301	29.24818	141.6447	29.22395	148.6254	29.43635	136.542
29.238	140.0856	29.26102	133.3393	29.32407	135.1296	29.26485	141.643	29.24062	148.6234	29.45302	136.5402
29.25467	140.0856	29.27768	133.333	29.34073	135.1281	29.28152	141.6421	29.25728	148.6227	29.46968	136.5378
29.27133	140.0839	29.29435	133.3331	29.3574	135.1275	29.29818	141.6418	29.27395	148.6224	29.48635	136.5384
29.288	140.0782	29.31102	133.3333	29.37407	135.127	29.31485	141.64	29.29062	148.6234	29.50302	136.5365
29.30467	140.0788	29.32768	133.325	29.39073	135.1256	29.33152	141.6382	29.30728	148.6213	29.51968	136.5342
29.32133	140.0746	29.34435	133.3244	29.4074	135.1243	29.34818	141.6398	29.32395	148.618	29.53635	136.5337
29.338	140.071	29.36102	133.3245	29.42407	135.1224	29.36485	141.6395	29.34062	148.6216	29.55302	136.5302
29.35467	140.0688	29.37768	133.3193	29.44073	135.1222	29.38152	141.6376	29.35728	148.6183	29.56968	136.5303
29.37133	140.0668	29.39435	133.3168	29.4574	135.1188	29.39818	141.6374	29.37395	148.6204	29.58635	136.529
29.388	140.065	29.41102	133.317	29.47407	135.1195	29.41485	141.6355	29.39062	148.618	29.60302	136.527
29.40467	140.0626	29.42768	133.316	29.49073	135.1194	29.43152	141.6341	29.40728	148.6184	29.61968	136.5254
29.42133	140.0601	29.44435	133.3114	29.5074	135.1152	29.44818	141.634	29.42395	148.6194	29.63635	136.5248
29.438	140.0567	29.46102	133.3111	29.52407	135.1164	29.46485	141.6329	29.44062	148.6173	29.65302	136.5237
29.45467	140.0538	29.47768	133.3074	29.54073	135.1123	29.48152	141.6327	29.45728	148.6185	29.66968	136.5217
29.47133	140.0514	29.49435	133.3065	29.5574	135.1151	29.49818	141.6302	29.47395	148.6165	29.68635	136.5233
29.488	140.0536	29.51102	133.3044	29.57407	135.1115	29.51485	141.6319	29.49062	148.6184	29.70302	136.5211
29.50467	140.0481	29.52768	133.2992	29.59073	135.1126	29.53152	141.6285	29.50728	148.6177	29.71968	136.5205
29.52133	140.0459	29.54435	133.298	29.6074	135.1067	29.54818	141.6269	29.52395	148.6149	29.73635	136.5171
29.538	140.0435	29.56102	133.2952	29.62407	135.1052	29.56485	141.6289	29.54062	148.6157	29.75302	136.5179
29.55467	140.0411	29.57768	133.2937	29.64073	135.1046	29.58152	141.6286	29.55728	148.615	29.76968	136.5154
29.57133	140.0395	29.59435	133.2894	29.6574	135.1056	29.59818	141.6247	29.57395	148.6154	29.78635	136.5166
29.588	140.0375	29.61102	133.2924	29.67407	135.103	29.61485	141.6231	29.59062	148.6147	29.80302	136.515
29.60467	140.0381	29.62768	133.2838	29.69073	135.0995	29.63152	141.6238	29.60728	148.614	29.81968	136.5118
29.62133	140.03	29.64435	133.2818	29.7074	135.1001	29.64818	141.6219	29.62395	148.6144	29.83635	136.5127
29.638	140.0324	29.66102	133.2851	29.72407	135.0982	29.66485	141.6226	29.64062	148.6143	29.85302	136.5114
29.65467	140.0275	29.67768	133.2807	29.74073	135.0965	29.68152	141.6216	29.65728	148.613	29.86968	136.5101
29.67133	140.0272	29.69435	133.2767	29.7574	135.0937	29.69818	141.6176	29.67395	148.6107	29.88635	136.5084
29.688	140.0223	29.71102	133.276	29.77407	135.0921	29.71485	141.6186	29.69062	148.6114	29.90302	136.508
29.70467	140.0217	29.72768	133.2729	29.79073	135.092	29.73152	141.6169	29.70728	148.6126	29.91968	136.5068
29.72133	140.0181	29.74435	133.27	29.8074	135.0902	29.74818	141.6158	29.72395	148.6099	29.93635	136.5046
29.738	140.017	29.76102	133.2693	29.82407	135.0869	29.76485	141.6165	29.74062	148.6097	29.95302	136.5023
29.75467	140.0148	29.77768	133.2652	29.84073	135.0821	29.78152	141.6155	29.75728	148.6079	29.96968	136.5022
29.77133	140.0151	29.79435	133.2633	29.8574	135.0824	29.79818	141.6133	29.77395	148.6069	29.98635	136.5011
29.788	140.0132	29.81102	133.2634	29.87407	135.0809	29.81485	141.6125	29.79062	148.6089	30.00302	136.5017
29.80467	140.0054	29.82768	133.2586	29.89073	135.0802	29.83152	141.6108	29.80728	148.6067	30.01968	136.4963
29.82133	140.0044	29.84435	133.2568	29.9074	135.0758	29.84818	141.6111	29.82395	148.6075	30.03635	136.4941
29.838	140.0052	29.86102	133.2562	29.92407	135.0754	29.86485	141.6081	29.84062	148.6111	30.05302	136.495
29.85467	139.9994	29.87768	133.2522	29.94073	135.0744	29.88152	141.6081	29.85728	148.606	30.06968	136.4932
29.87133	139.9968	29.89435	133.2492	29.9574	135.0721	29.89818	141.6085	29.87395	148.6041	30.08635	136.4913

29.888	139.9968	29.91102	133.2497	29.97407	135.071	29.91485	141.6053	29.89062	148.6136	30.10302	136.49
29.90467	139.9968	29.92768	133.2427	29.99073	135.07	29.93152	141.6052	29.90728	148.6003	30.11968	136.4901
29.92133	139.9911	29.94435	133.244	30.0074	135.0665	29.94818	141.6051	29.92395	148.6038	30.13635	136.4883
29.938	139.9894	29.96102	133.2437	30.02407	135.0671	29.96485	141.6048	29.94062	148.6039	30.15302	136.4866
29.95467	139.9902	29.97768	133.2408	30.04073	135.0614	29.98152	141.6021	29.95728	148.5992	30.16968	136.4843
29.97133	139.9888	29.99435	133.2376	30.0574	135.0616	29.99818	141.6051	29.97395	148.6024	30.18635	136.4842
29.988	139.9849	30.01102	133.2351	30.07407	135.0617	30.01485	141.6007	29.99062	148.6015	30.20302	136.483
30.00467	139.981	30.02768	133.2336	30.09073	135.0615	30.03152	141.5991	30.00728	148.6043	30.21968	136.4829
30.02133	139.9809	30.04435	133.2302	30.1074	135.0567	30.04818	141.5992	30.02395	148.6025	30.23635	136.4803
30.038	139.9767	30.06102	133.2299	30.12407	135.0553	30.06485	141.5999	30.04062	148.5987	30.25302	136.4787
30.05467	139.9781	30.07768	133.2303	30.14073	135.0537	30.08152	141.5992	30.05728	148.6013	30.26968	136.48
30.07133	139.9752	30.09435	133.2268	30.1574	135.0549	30.09818	141.5974	30.07395	148.5995	30.28635	136.478
30.088	139.9719	30.11102	133.2239	30.17407	135.0514	30.11485	141.5965	30.09062	148.5993	30.30302	136.4745
30.10467	139.9681	30.12768	133.2214	30.19073	135.0492	30.13152	141.5948	30.10728	148.5973	30.31968	136.4751
30.12133	139.9691	30.14435	133.2163	30.2074	135.0488	30.14818	141.596	30.12395	148.5963	30.33635	136.4755
30.138	139.961	30.16102	133.217	30.22407	135.0456	30.16485	141.594	30.14062	148.5997	30.35302	136.4728
30.15467	139.9628	30.17768	133.2134	30.24073	135.0436	30.18152	141.5945	30.15728	148.5985	30.36968	136.4712
30.17133	139.9612	30.19435	133.2082	30.2574	135.0455	30.19818	141.5906	30.17395	148.5968	30.38635	136.4678
30.188	139.9572	30.21102	133.2083	30.27407	135.0443	30.21485	141.5905	30.19062	148.5969	30.40302	136.4658
30.20467	139.9531	30.22768	133.2098	30.29073	135.0422	30.23152	141.5904	30.20728	148.5936	30.41968	136.4679
30.22133	139.9507	30.24435	133.2064	30.3074	135.0361	30.24818	141.5909	30.22395	148.5951	30.43635	136.464
30.238	139.9503	30.26102	133.2051	30.32407	135.0362	30.26485	141.5886	30.24062	148.599	30.45302	136.4612
30.25467	139.9456	30.27768	133.2024	30.34073	135.0346	30.28152	141.5883	30.25728	148.5939	30.46968	136.461
30.27133	139.9449	30.29435	133.1989	30.3574	135.0332	30.29818	141.5862	30.27395	148.5955	30.48635	136.4598
30.288	139.9397	30.31102	133.1985	30.37407	135.0319	30.31485	141.5848	30.29062	148.5912	30.50302	136.458
30.30467	139.9427	30.32768	133.1972	30.39073	135.033	30.33152	141.5826	30.30728	148.5951	30.51968	136.4577
30.32133	139.9376	30.34435	133.1944	30.4074	135.0306	30.34818	141.5853	30.32395	148.5928	30.53635	136.4542
30.338	139.9336	30.36102	133.1923	30.42407	135.0274	30.36485	141.5821	30.34062	148.5931	30.55302	136.4522
30.35467	139.9361	30.37768	133.1891	30.44073	135.0309	30.38152	141.5833	30.35728	148.5907	30.56968	136.4505
30.37133	139.9308	30.39435	133.1902	30.4574	135.0234	30.39818	141.5819	30.37395	148.5919	30.58635	136.4495
30.388	139.9298	30.41102	133.1864	30.47407	135.0242	30.41485	141.5805	30.39062	148.5908	30.60302	136.4445
30.40467	139.9283	30.42768	133.1839	30.49073	135.0248	30.43152	141.579	30.40728	148.5911	30.61968	136.4457
30.42133	139.924	30.44435	133.1805	30.5074	135.0208	30.44818	141.5793	30.42395	148.5898	30.63635	136.4436
30.438	139.9201	30.46102	133.1802	30.52407	135.0194	30.46485	141.5796	30.44062	148.5882	30.65302	136.4432
30.45467	139.921	30.47768	133.1774	30.54073	135.018	30.48152	141.5777	30.45728	148.5902	30.66968	136.4401
30.47133	139.9183	30.49435	133.1749	30.5574	135.0157	30.49818	141.5755	30.47395	148.5838	30.68635	136.4413
30.488	139.9129	30.51102	133.1752	30.57407	135.0137	30.51485	141.5759	30.49062	148.5883	30.70302	136.4388
30.50467	139.9111	30.52768	133.1728	30.59073	135.012	30.53152	141.5702	30.50728	148.5845	30.71968	136.4366
30.52133	139.9112	30.54435	133.1722	30.6074	135.0093	30.54818	141.5734	30.52395	148.587	30.73635	136.4347
30.538	139.907	30.56102	133.1703	30.62407	135.0084	30.56485	141.5715	30.54062	148.5882	30.75302	136.4346
30.55467	139.9049	30.57768	133.1662	30.64073	135.0075	30.58152	141.572	30.55728	148.5825	30.76968	136.4306
30.57133	139.908	30.59435	133.1636	30.6574	135.0101	30.59818	141.5704	30.57395	148.5808	30.78635	136.4294
30.588	139.9026	30.61102	133.1616	30.67407	135.0035	30.61485	141.5695	30.59062	148.5823	30.80302	136.4274
30.60467	139.8976	30.62768	133.161	30.69073	135.0015	30.63152	141.5668	30.60728	148.5834	30.81968	136.4265
30.62133	139.8912	30.64435	133.1562	30.7074	135.0025	30.64818	141.5678	30.62395	148.5819	30.83635	136.4257
30.638	139.8937	30.66102	133.155	30.72407	135.0025	30.66485	141.5676	30.64062	148.5814	30.85302	136.4256
30.65467	139.8947	30.67768	133.1527	30.74073	134.9999	30.68152	141.5654	30.65728	148.5825	30.86968	136.4202
30.67133	139.8909	30.69435	133.1498	30.7574	134.9972	30.69818	141.5668	30.67395	148.5807	30.88635	136.4194
30.688	139.8903	30.71102	133.1473	30.77407	134.9967	30.71485	141.5654	30.69062	148.5799	30.90302	136.4185
30.70467	139.8839	30.72768	133.1439	30.79073	134.9938	30.73152	141.5668	30.70728	148.5793	30.91968	136.4179
30.72133	139.882	30.74435	133.1398	30.8074	134.994	30.74818	141.5635	30.72395	148.5784	30.93635	136.4155
30.738	139.8849	30.76102	133.1406	30.82407	134.9922	30.76485	141.5635	30.74062	148.5808	30.95302	136.4152
30.75467	139.8814	30.77768	133.139	30.84073	134.9914	30.78152	141.5617	30.75728	148.5787	30.96968	136.4135
30.77133	139.8754	30.79435	133.1354	30.8574	134.9906	30.79818	141.5582	30.77395	148.5751	30.98635	136.4106
30.788	139.8778	30.81102	133.1285	30.87407	134.9891	30.81485	141.5588	30.79062	148.5797	31.00302	136.4096
30.80467	139.8735	30.82768	133.1366	30.89073	134.986	30.83152	141.5586	30.80728	148.5768	31.01968	136.4059
30.82133	139.869	30.84435	133.1236	30.9074	134.984	30.84818	141.5575	30.82395	148.5777	31.03635	136.4064
30.838	139.8672	30.86102	133.1261	30.92407	134.9829	30.86485	141.557	30.84062	148.577	31.05302	136.4069
30.85467	139.8682	30.87768	133.1244	30.94073	134.9829	30.88152	141.5538	30.85728	148.5763	31.06968	136.4046
30.87133	139.8635	30.89435	133.1215	30.9574	134.9801	30.89818	141.5551	30.87395	148.574	31.08635	136.4028
30.888	139.8621	30.91102	133.1224	30.97407	134.9786	30.91485	141.5516	30.89062	148.5722	31.10302	136.4029
30.90467	139.8618	30.92768	133.116	30.99073	134.9755	30.93152	141.5506	30.90728	148.576	31.11968	136.4017
30.92133	139.8554	30.94435	133.1143	31.0074	134.9738	30.94818	141.5524	30.92395	148.5725	31.13635	136.4004
30.938	139.8562	30.96102	133.1128	31.02407	134.9736	30.96485	141.5509	30.94062	148.5714	31.15302	136.3961
30.95467	139.8524	30.97768	133.1105	31.04073	134.9744	30.98152	141.5495	30.95728	148.5751	31.16968	136.3962
30.97133	139.8475	30.99435	133.1095	31.0574	134.9696	30.99818	141.5512	30.97395	148.5741	31.18635	136.3941
30.988	139.848	31.01102	133.1055	31.07407	134.969	31.01485	141.5491	30.99062	148.5704	31.20302	136.395
31.00467	139.8467	31.02768	133.1033	31.09073	134.9667	31.03152	141.5467	31.00728	148.5735	31.21968	136.3915
31.02133	139.8463	31.04435	133.1046	31.1074	134.9655	31.04818	141.5483	31.02395	148.5713	31.23635	136.3932

31.038	139.8446	31.06102	133.1017	31.12407	134.9651	31.06485	141.5469	31.04062	148.5741	31.25302	136.3922
31.05467	139.8395	31.07768	133.0962	31.14073	134.9627	31.08152	141.5456	31.05728	148.5736	31.26968	136.3908
31.07133	139.8377	31.09435	133.0986	31.1574	134.9619	31.09818	141.543	31.07395	148.5742	31.28635	136.389
31.088	139.8363	31.11102	133.0932	31.17407	134.9588	31.11485	141.545	31.09062	148.5706	31.30302	136.3875
31.10467	139.8378	31.12768	133.0951	31.19073	134.9573	31.13152	141.5452	31.10728	148.5725	31.31968	136.3883
31.12133	139.8332	31.14435	133.0908	31.2074	134.956	31.14818	141.5418	31.12395	148.5714	31.33635	136.3864
31.138	139.8314	31.16102	133.0898	31.22407	134.9553	31.16485	141.5418	31.14062	148.5714	31.35302	136.3853
31.15467	139.8291	31.17768	133.0887	31.24073	134.9515	31.18152	141.5415	31.15728	148.5669	31.36968	136.3851
31.17133	139.8251	31.19435	133.0877	31.2574	134.9522	31.19818	141.54	31.17395	148.567	31.38635	136.382
31.188	139.8229	31.21102	133.0839	31.27407	134.9519	31.21485	141.5404	31.19062	148.5689	31.40302	136.3828
31.20467	139.8187	31.22768	133.0836	31.29073	134.949	31.23152	141.539	31.20728	148.5684	31.41968	136.3795
31.22133	139.8205	31.24435	133.08	31.3074	134.9454	31.24818	141.5377	31.22395	148.5687	31.43635	136.377
31.238	139.8142	31.26102	133.0767	31.32407	134.9432	31.26485	141.5327	31.24062	148.5686	31.45302	136.3789
31.25467	139.8127	31.27768	133.0807	31.34073	134.9461	31.28152	141.5376	31.25728	148.5689	31.46968	136.3762
31.27133	139.8116	31.29435	133.0764	31.3574	134.9397	31.29818	141.5394	31.27395	148.5663	31.48635	136.3756
31.288	139.813	31.31102	133.0724	31.37407	134.9412	31.31485	141.5324	31.29062	148.5671	31.50302	136.3735
31.30467	139.8093	31.32768	133.0714	31.39073	134.9398	31.33152	141.5361	31.30728	148.5654	31.51968	136.3766
31.32133	139.8003	31.34435	133.0684	31.4074	134.9316	31.34818	141.5345	31.32395	148.5646	31.53635	136.3719
31.338	139.7996	31.36102	133.0628	31.42407	134.9358	31.36485	141.5342	31.34062	148.5667	31.55302	136.3708
31.35467	139.7952	31.37768	133.0631	31.44073	134.9335	31.38152	141.5329	31.35728	148.5643	31.56968	136.3684
31.37133	139.8028	31.39435	133.0604	31.4574	134.9321	31.39818	141.5303	31.37395	148.563	31.58635	136.3688
31.388	139.7968	31.41102	133.0591	31.47407	134.9266	31.41485	141.5295	31.39062	148.5645	31.60302	136.3689
31.40467	139.796	31.42768	133.0591	31.49073	134.9271	31.43152	141.5311	31.40728	148.5621	31.61968	136.3644
31.42133	139.7902	31.44435	133.0545	31.5074	134.9248	31.44818	141.531	31.42395	148.5626	31.63635	136.363
31.438	139.7921	31.46102	133.0529	31.52407	134.9207	31.46485	141.5284	31.44062	148.5623	31.65302	136.3628
31.45467	139.7879	31.47768	133.0493	31.54073	134.9258	31.48152	141.5266	31.45728	148.5608	31.66968	136.3637
31.47133	139.7881	31.49435	133.0481	31.5574	134.9179	31.49818	141.5276	31.47395	148.5606	31.68635	136.3594
31.488	139.7858	31.51102	133.0476	31.57407	134.92	31.51485	141.5276	31.49062	148.5602	31.70302	136.359
31.50467	139.7848	31.52768	133.0436	31.59073	134.9146	31.53152	141.5265	31.50728	148.5616	31.71968	136.3575
31.52133	139.7779	31.54435	133.0427	31.6074	134.915	31.54818	141.5258	31.52395	148.5588	31.73635	136.3561
31.538	139.7807	31.56102	133.043	31.62407	134.9122	31.56485	141.527	31.54062	148.5566	31.75302	136.3551
31.55467	139.7741	31.57768	133.0391	31.64073	134.9131	31.58152	141.5266	31.55728	148.5575	31.76968	136.3533
31.57133	139.7749	31.59435	133.0367	31.6574	134.9138	31.59818	141.5248	31.57395	148.5584	31.78635	136.354
31.588	139.7695	31.61102	133.0348	31.67407	134.9104	31.61485	141.5242	31.59062	148.5592	31.80302	136.3508
31.60467	139.7678	31.62768	133.0338	31.69073	134.9058	31.63152	141.5237	31.60728	148.5611	31.81968	136.3525
31.62133	139.7655	31.64435	133.0317	31.7074	134.9064	31.64818	141.522	31.62395	148.5586	31.83635	136.3487
31.638	139.7639	31.66102	133.0273	31.72407	134.9043	31.66485	141.5216	31.64062	148.5567	31.85302	136.349
31.65467	139.7605	31.67768	133.0279	31.74073	134.9041	31.68152	141.5213	31.65728	148.5574	31.86968	136.3458
31.67133	139.7574	31.69435	133.0255	31.7574	134.9027	31.69818	141.5206	31.67395	148.5574	31.88635	136.3457
31.688	139.7549	31.71102	133.0234	31.77407	134.8999	31.71485	141.5215	31.69062	148.5585	31.90302	136.3428
31.70467	139.7511	31.72768	133.0182	31.79073	134.9055	31.73152	141.5199	31.70728	148.5543	31.91968	136.3434
31.72133	139.7506	31.74435	133.0155	31.8074	134.8998	31.74818	141.5185	31.72395	148.5549	31.93635	136.3416
31.738	139.7526	31.76102	133.0152	31.82407	134.897	31.76485	141.5167	31.74062	148.5539	31.95302	136.3398
31.75467	139.7413	31.77768	133.0123	31.84073	134.8923	31.78152	141.5153	31.75728	148.5539	31.96968	136.3401
31.77133	139.742	31.79435	133.0099	31.8574	134.8926	31.79818	141.5182	31.77395	148.554	31.98635	136.3383
31.788	139.7411	31.81102	133.0081	31.87407	134.8927	31.81485	141.5167	31.79062	148.5533	32.00302	136.3389
31.80467	139.7399	31.82768	133.0083	31.89073	134.8898	31.83152	141.514	31.80728	148.5509	32.01968	136.3358
31.82133	139.7378	31.84435	133.0042	31.9074	134.8881	31.84818	141.5154	31.82395	148.55	32.03635	136.3345
31.838	139.7358	31.86102	133	31.92407	134.8891	31.86485	141.5123	31.84062	148.5495	32.05302	136.3332
31.85467	139.7326	31.87768	133.001	31.94073	134.8847	31.88152	141.5124	31.85728	148.5502	32.06968	136.3327
31.87133	139.7285	31.89435	133.0005	31.9574	134.8861	31.89818	141.5105	31.87395	148.5499	32.08635	136.3307
31.888	139.7272	31.91102	132.9964	31.97407	134.8825	31.91485	141.512	31.89062	148.5502	32.10302	136.3281
31.90467	139.7239	31.92768	132.9945	31.99073	134.8833	31.93152	141.5119	31.90728	148.5497	32.11968	136.3278
31.92133	139.722	31.94435	132.9954	32.0074	134.882	31.94818	141.5095	31.92395	148.5476	32.13635	136.3256
31.938	139.7215	31.96102	132.9927	32.02407	134.8813	31.96485	141.5111	31.94062	148.5493	32.15302	136.3251
31.95467	139.7212	31.97768	132.9919	32.04073	134.8796	31.98152	141.505	31.95728	148.5478	32.16968	136.3222
31.97133	139.7164	31.99435	132.9845	32.0574	134.88	31.99818	141.5063	31.97395	148.5487	32.18635	136.3213
31.988	139.712	32.01102	132.9865	32.07407	134.8767	32.01485	141.5063	31.99062	148.5481	32.20302	136.3196
32.00467	139.7133	32.02768	132.9841	32.09073	134.8759	32.03152	141.5028	32.00728	148.5464	32.21968	136.3193
32.02133	139.7081	32.04435	132.9781	32.1074	134.8767	32.04818	141.5039	32.02395	148.5457	32.23635	136.3177
32.038	139.7051	32.06102	132.9779	32.12407	134.8728	32.06485	141.5031	32.04062	148.5463	32.25302	136.3158
32.05467	139.7056	32.07768	132.9794	32.14073	134.8717	32.08152	141.5033	32.05728	148.5465	32.26968	136.3123
32.07133	139.7026	32.09435	132.9746	32.1574	134.8703	32.09818	141.5017	32.07395	148.544	32.28635	136.313
32.088	139.7031	32.11102	132.9726	32.17407	134.8711	32.11485	141.5015	32.09062	148.5439	32.30302	136.3093
32.10467	139.6987	32.12768	132.9682	32.19073	134.8691	32.13152	141.4999	32.10728	148.5428	32.31968	136.307
32.12133	139.6955	32.14435	132.9677	32.2074	134.8671	32.14818	141.5001	32.12395	148.5433	32.33635	136.3074
32.138	139.6932	32.16102	132.9661	32.22407	134.8661	32.16485	141.4954	32.14062	148.5441	32.35302	136.3087
32.15467	139.6907	32.17768	132.9637	32.24073	134.8639	32.18152	141.5003	32.15728	148.543	32.36968	136.3066
32.17133	139.6907	32.19435	132.9622	32.2574	134.8627	32.19818	141.4959	32.17395	148.5419	32.38635	136.3032

32.188	139.686	32.21102	132.9582	32.27407	134.8621	32.21485	141.4949	32.19062	148.542	32.40302	136.3049
32.20467	139.6849	32.22768	132.9581	32.29073	134.8599	32.23152	141.4912	32.20728	148.5423	32.41968	136.3022
32.22133	139.6836	32.24435	132.953	32.3074	134.8567	32.24818	141.4938	32.22395	148.5411	32.43635	136.3015
32.238	139.6818	32.26102	132.9536	32.32407	134.8578	32.26485	141.4924	32.24062	148.5416	32.45302	136.3015
32.25467	139.6773	32.27768	132.9506	32.34073	134.8566	32.28152	141.492	32.25728	148.5431	32.46968	136.2997
32.27133	139.6776	32.29435	132.9498	32.3574	134.8521	32.29818	141.4896	32.27395	148.5399	32.48635	136.2989
32.288	139.6757	32.31102	132.9464	32.37407	134.8521	32.31485	141.4929	32.29062	148.5428	32.50302	136.297
32.30467	139.6716	32.32768	132.9438	32.39073	134.8477	32.33152	141.4906	32.30728	148.5419	32.51968	136.2949
32.32133	139.6717	32.34435	132.9435	32.4074	134.8454	32.34818	141.4882	32.32395	148.5425	32.53635	136.2954
32.338	139.6686	32.36102	132.9431	32.42407	134.8468	32.36485	141.4876	32.34062	148.5418	32.55302	136.2924
32.35467	139.6663	32.37768	132.939	32.44073	134.8444	32.38152	141.4875	32.35728	148.5401	32.56968	136.2908
32.37133	139.6647	32.39435	132.9368	32.4574	134.8418	32.39818	141.4873	32.37395	148.5403	32.58635	136.2891
32.388	139.661	32.41102	132.9368	32.47407	134.8422	32.41485	141.4845	32.39062	148.5399	32.60302	136.2884
32.40467	139.659	32.42768	132.9326	32.49073	134.8401	32.43152	141.483	32.40728	148.5379	32.61968	136.2864
32.42133	139.6582	32.44435	132.9298	32.5074	134.8384	32.44818	141.4846	32.42395	148.5401	32.63635	136.2832
32.438	139.6544	32.46102	132.9312	32.52407	134.8383	32.46485	141.4816	32.44062	148.5383	32.65302	136.2814
32.45467	139.6529	32.47768	132.9276	32.54073	134.8356	32.48152	141.4818	32.45728	148.5377	32.66968	136.2818
32.47133	139.6495	32.49435	132.9262	32.5574	134.8331	32.49818	141.4797	32.47395	148.5377	32.68635	136.2808
32.488	139.6507	32.51102	132.9249	32.57407	134.8323	32.51485	141.4801	32.49062	148.5389	32.70302	136.2785
32.50467	139.6463	32.52768	132.9229	32.59073	134.8328	32.53152	141.4778	32.50728	148.5377	32.71968	136.2762
32.52133	139.6415	32.54435	132.9195	32.6074	134.826	32.54818	141.4776	32.52395	148.5389	32.73635	136.2782
32.538	139.6418	32.56102	132.9173	32.62407	134.8267	32.56485	141.4781	32.54062	148.5346	32.75302	136.2746
32.55467	139.6433	32.57768	132.9151	32.64073	134.8234	32.58152	141.4759	32.55728	148.5364	32.76968	136.2723
32.57133	139.6392	32.59435	132.9121	32.6574	134.8244	32.59818	141.475	32.57395	148.536	32.78635	136.2691
32.588	139.6381	32.61102	132.9133	32.67407	134.8224	32.61485	141.4747	32.59062	148.5341	32.80302	136.2682
32.60467	139.6357	32.62768	132.9151	32.69073	134.8209	32.63152	141.4739	32.60728	148.5331	32.81968	136.2646
32.62133	139.632	32.64435	132.9089	32.7074	134.8212	32.64818	141.4745	32.62395	148.5312	32.83635	136.2644
32.638	139.631	32.66102	132.9092	32.72407	134.816	32.66485	141.4754	32.64062	148.5338	32.85302	136.2604
32.65467	139.6269	32.67768	132.9031	32.74073	134.814	32.68152	141.4735	32.65728	148.536	32.86968	136.2611
32.67133	139.625	32.69435	132.9012	32.7574	134.8163	32.69818	141.4724	32.67395	148.5319	32.88635	136.2602
32.688	139.6219	32.71102	132.8996	32.77407	134.8137	32.71485	141.4723	32.69062	148.5304	32.90302	136.2574
32.70467	139.6219	32.72768	132.8981	32.79073	134.8081	32.73152	141.4701	32.70728	148.5339	32.91968	136.2565
32.72133	139.6203	32.74435	132.898	32.8074	134.8103	32.74818	141.4697	32.72395	148.5335	32.93635	136.2567
32.738	139.6187	32.76102	132.8952	32.82407	134.8064	32.76485	141.4689	32.74062	148.5316	32.95302	136.255
32.75467	139.618	32.77768	132.8905	32.84073	134.806	32.78152	141.4691	32.75728	148.5332	32.96968	136.2523
32.77133	139.6159	32.79435	132.8897	32.8574	134.8038	32.79818	141.4696	32.77395	148.531	32.98635	136.2539
32.788	139.6111	32.81102	132.8876	32.87407	134.8043	32.81485	141.4712	32.79062	148.5308	33.00302	136.2491
32.80467	139.6103	32.82768	132.8866	32.89073	134.8015	32.83152	141.4668	32.80728	148.5279	33.01968	136.2483
32.82133	139.6079	32.84435	132.884	32.9074	134.7983	32.84818	141.4654	32.82395	148.5295	33.03635	136.2478
32.838	139.6051	32.86102	132.8829	32.92407	134.7997	32.86485	141.4666	32.84062	148.5255	33.05302	136.2465
32.85467	139.6039	32.87768	132.8801	32.94073	134.7963	32.88152	141.4642	32.85728	148.5281	33.06968	136.2451
32.87133	139.602	32.89435	132.8777	32.9574	134.7943	32.89818	141.4668	32.87395	148.527	33.08635	136.2433
32.888	139.6006	32.91102	132.875	32.97407	134.7945	32.91485	141.4661	32.89062	148.5276	33.10302	136.242
32.90467	139.5986	32.92768	132.8696	32.99073	134.795	32.93152	141.4642	32.90728	148.5262	33.11968	136.2407
32.92133	139.5976	32.94435	132.8691	33.0074	134.7911	32.94818	141.4658	32.92395	148.5291	33.13635	136.242
32.938	139.5922	32.96102	132.8648	33.02407	134.7901	32.96485	141.4607	32.94062	148.5256	33.15302	136.2413
32.95467	139.5916	32.97768	132.8643	33.04073	134.7906	32.98152	141.4615	32.95728	148.5253	33.16968	136.2364
32.97133	139.5903	32.99435	132.8636	33.0574	134.788	32.99818	141.4598	32.97395	148.5236	33.18635	136.2367
32.988	139.5889	33.01102	132.8613	33.07407	134.7896	33.01485	141.4593	32.99062	148.5245	33.20302	136.2356
33.00467	139.5863	33.02768	132.8593	33.09073	134.7871	33.03152	141.4575	33.00728	148.5253	33.21968	136.2333
33.02133	139.5842	33.04435	132.8574	33.1074	134.7871	33.04818	141.4577	33.02395	148.5225	33.23635	136.2336
33.038	139.5807	33.06102	132.8547	33.12407	134.7837	33.06485	141.4576	33.04062	148.5211	33.25302	136.2319
33.05467	139.5823	33.07768	132.8527	33.14073	134.7817	33.08152	141.4571	33.05728	148.521	33.26968	136.2305
33.07133	139.5798	33.09435	132.8511	33.1574	134.7819	33.09818	141.455	33.07395	148.5185	33.28635	136.2298
33.088	139.577	33.11102	132.8489	33.17407	134.7819	33.11485	141.4548	33.09062	148.5186	33.30302	136.2285
33.10467	139.5749	33.12768	132.8472	33.19073	134.7793	33.13152	141.4523	33.10728	148.5196	33.31968	136.228
33.12133	139.5698	33.14435	132.8417	33.2074	134.7761	33.14818	141.4537	33.12395	148.5194	33.33635	136.2283
33.138	139.572	33.16102	132.8392	33.22407	134.7779	33.16485	141.4499	33.14062	148.518	33.35302	136.2285
33.15467	139.5703	33.17768	132.8398	33.24073	134.7744	33.18152	141.4512	33.15728	148.5183	33.36968	136.2235
33.17133	139.5683	33.19435	132.8379	33.2574	134.7721	33.19818	141.4487	33.17395	148.516	33.38635	136.2222
33.188	139.5645	33.21102	132.8334	33.27407	134.7708	33.21485	141.4475	33.19062	148.5154	33.40302	136.2232
33.20467	139.563	33.22768	132.8331	33.29073	134.7703	33.23152	141.4441	33.20728	148.5151	33.41968	136.2226
33.22133	139.5612	33.24435	132.831	33.3074	134.7703	33.24818	141.443	33.22395	148.5141	33.43635	136.2222
33.238	139.5593	33.26102	132.8329	33.32407	134.7687	33.26485	141.4433	33.24062	148.5138	33.45302	136.2218
33.25467	139.5545	33.27768	132.8274	33.34073	134.7669	33.28152	141.4412	33.25728	148.5107	33.46968	136.2193
33.27133	139.5507	33.29435	132.8251	33.3574	134.7623	33.29818	141.4405	33.27395	148.5088	33.48635	136.2192
33.288	139.5481	33.31102	132.821	33.37407	134.7646	33.31485	141.4396	33.29062	148.5154	33.50302	136.2192
33.30467	139.5496	33.32768	132.8196	33.39073	134.7646	33.33152	141.439	33.30728	148.5097	33.51968	136.2178
33.32133	139.5457	33.34435	132.8216	33.4074	134.7645	33.34818	141.4373	33.32395	148.5101	33.53635	136.2173

33.338	139.543	33.36102	132.8192	33.42407	134.7604	33.36485	141.4367	33.34062	148.5139	33.55302	136.215
33.35467	139.5383	33.37768	132.8148	33.44073	134.7594	33.38152	141.4379	33.35728	148.5114	33.56968	136.2154
33.37133	139.5383	33.39435	132.8159	33.4574	134.7595	33.39818	141.4389	33.37395	148.5119	33.58635	136.2117
33.388	139.5386	33.41102	132.8135	33.47407	134.7544	33.41485	141.4363	33.39062	148.5122	33.60302	136.2085
33.40467	139.5269	33.42768	132.8082	33.49073	134.7563	33.43152	141.4362	33.40728	148.5116	33.61968	136.2094
33.42133	139.5311	33.44435	132.8057	33.5074	134.7545	33.44818	141.4356	33.42395	148.5097	33.63635	136.2045
33.438	139.5289	33.46102	132.8064	33.52407	134.7517	33.46485	141.4341	33.44062	148.5089	33.65302	136.205
33.45467	139.527	33.47768	132.8032	33.54073	134.7498	33.48152	141.4327	33.45728	148.5091	33.66968	136.2026
33.47133	139.524	33.49435	132.8045	33.5574	134.7526	33.49818	141.4351	33.47395	148.5104	33.68635	136.2001
33.488	139.5247	33.51102	132.8005	33.57407	134.7471	33.51485	141.4342	33.49062	148.5083	33.70302	136.1987
33.50467	139.5176	33.52768	132.7976	33.59073	134.7458	33.53152	141.4324	33.50728	148.5075	33.71968	136.1965
33.52133	139.5225	33.54435	132.7942	33.6074	134.7438	33.54818	141.4283	33.52395	148.5075	33.73635	136.1972
33.538	139.5167	33.56102	132.7922	33.62407	134.7413	33.56485	141.4283	33.54062	148.5092	33.75302	136.1936
33.55467	139.5111	33.57768	132.7921	33.64073	134.7411	33.58152	141.4284	33.55728	148.511	33.76968	136.1914
33.57133	139.5123	33.59435	132.7858	33.6574	134.7398	33.59818	141.4257	33.57395	148.5096	33.78635	136.1888
33.588	139.5078	33.61102	132.786	33.67407	134.7396	33.61485	141.4244	33.59062	148.505	33.80302	136.1885
33.60467	139.5077	33.62768	132.783	33.69073	134.7379	33.63152	141.4269	33.60728	148.5075	33.81968	136.1881
33.62133	139.5054	33.64435	132.7813	33.7074	134.7353	33.64818	141.4264	33.62395	148.5062	33.83635	136.1857
33.638	139.5062	33.66102	132.7839	33.72407	134.7344	33.66485	141.4228	33.64062	148.5088	33.85302	136.1845
33.65467	139.4982	33.67768	132.7781	33.74073	134.7344	33.68152	141.423	33.65728	148.5068	33.86968	136.1826
33.67133	139.4966	33.69435	132.7752	33.7574	134.7305	33.69818	141.422	33.67395	148.5046	33.88635	136.1818
33.688	139.4958	33.71102	132.7735	33.77407	134.7296	33.71485	141.4226	33.69062	148.506	33.90302	136.181
33.70467	139.494	33.72768	132.7709	33.79073	134.7308	33.73152	141.4209	33.70728	148.506	33.91968	136.1799
33.72133	139.496	33.74435	132.7696	33.8074	134.7275	33.74818	141.4169	33.72395	148.5066	33.93635	136.1778
33.738	139.4907	33.76102	132.7643	33.82407	134.7272	33.76485	141.4192	33.74062	148.5028	33.95302	136.1792
33.75467	139.4875	33.77768	132.7628	33.84073	134.7251	33.78152	141.4201	33.75728	148.5057	33.96968	136.1764
33.77133	139.4837	33.79435	132.7625	33.8574	134.7236	33.79818	141.4194	33.77395	148.5049	33.98635	136.1742
33.788	139.4835	33.81102	132.7599	33.87407	134.7232	33.81485	141.4203	33.79062	148.5027	34.00302	136.1717
33.80467	139.4788	33.82768	132.7597	33.89073	134.7213	33.83152	141.4176	33.80728	148.5003	34.01968	136.1723
33.82133	139.4811	33.84435	132.7578	33.9074	134.7196	33.84818	141.4163	33.82395	148.5027	34.03635	136.1726
33.838	139.4781	33.86102	132.7551	33.92407	134.7157	33.86485	141.4161	33.84062	148.5007	34.05302	136.1707
33.85467	139.4746	33.87768	132.7531	33.94073	134.7198	33.88152	141.4153	33.85728	148.5018	34.06968	136.1697
33.87133	139.472	33.89435	132.753	33.9574	134.7171	33.89818	141.4145	33.87395	148.5011	34.08635	136.1692
33.888	139.4701	33.91102	132.7469	33.97407	134.7138	33.91485	141.415	33.89062	148.5023	34.10302	136.1659
33.90467	139.4692	33.92768	132.7447	33.99073	134.7167	33.93152	141.4143	33.90728	148.5009	34.11968	136.1679
33.92133	139.4651	33.94435	132.7464	34.0074	134.7106	33.94818	141.4142	33.92395	148.5005	34.13635	136.1622
33.938	139.4634	33.96102	132.7429	34.02407	134.7087	33.96485	141.414	33.94062	148.5002	34.15302	136.1628
33.95467	139.4598	33.97768	132.7422	34.04073	134.7117	33.98152	141.4124	33.95728	148.4994	34.16968	136.1594
33.97133	139.4593	33.99435	132.7392	34.0574	134.7091	33.99818	141.4115	33.97395	148.501	34.18635	136.1607
33.988	139.4555	34.01102	132.7428	34.07407	134.7076	34.01485	141.4082	33.99062	148.4983	34.20302	136.1577
34.00467	139.4571	34.02768	132.7349	34.09073	134.7089	34.03152	141.408	34.00728	148.4975	34.21968	136.1553
34.02133	139.453	34.04435	132.7349	34.1074	134.706	34.04818	141.408	34.02395	148.4943	34.23635	136.1558
34.038	139.4508	34.06102	132.7315	34.12407	134.7035	34.06485	141.4061	34.04062	148.4973	34.25302	136.1541
34.05467	139.4491	34.07768	132.7322	34.14073	134.7031	34.08152	141.4091	34.05728	148.4979	34.26968	136.1522
34.07133	139.4467	34.09435	132.7293	34.1574	134.7034	34.09818	141.406	34.07395	148.4957	34.28635	136.1503
34.088	139.4439	34.11102	132.7288	34.17407	134.7022	34.11485	141.404	34.09062	148.495	34.30302	136.1514
34.10467	139.4436	34.12768	132.7256	34.19073	134.7006	34.13152	141.4049	34.10728	148.4966	34.31968	136.1484
34.12133	139.442	34.14435	132.7235	34.2074	134.6989	34.14818	141.4033	34.12395	148.4966	34.33635	136.1482
34.138	139.4388	34.16102	132.7223	34.22407	134.6956	34.16485	141.402	34.14062	148.494	34.35302	136.1481
34.15467	139.4379	34.17768	132.7203	34.24073	134.6919	34.18152	141.4038	34.15728	148.494	34.36968	136.1448
34.17133	139.4341	34.19435	132.7211	34.2574	134.6934	34.19818	141.4011	34.17395	148.4925	34.38635	136.1455
34.188	139.4327	34.21102	132.7152	34.27407	134.6927	34.21485	141.4017	34.19062	148.4928	34.40302	136.1429
34.20467	139.4312	34.22768	132.7132	34.29073	134.6888	34.23152	141.4005	34.20728	148.492	34.41968	136.1434
34.22133	139.4313	34.24435	132.7139	34.3074	134.6879	34.24818	141.3996	34.22395	148.488	34.43635	136.1411
34.238	139.4265	34.26102	132.7079	34.32407	134.6858	34.26485	141.3984	34.24062	148.4927	34.45302	136.1414
34.25467	139.425	34.27768	132.7076	34.34073	134.6813	34.28152	141.3988	34.25728	148.4925	34.46968	136.1386
34.27133	139.4248	34.29435	132.7086	34.3574	134.6834	34.29818	141.3975	34.27395	148.4923	34.48635	136.1374
34.288	139.4226	34.31102	132.7051	34.37407	134.681	34.31485	141.3971	34.29062	148.4892	34.50302	136.1377
34.30467	139.4209	34.32768	132.7002	34.39073	134.6791	34.33152	141.3962	34.30728	148.4894	34.51968	136.1359
34.32133	139.4172	34.34435	132.6974	34.4074	134.6773	34.34818	141.3946	34.32395	148.4901	34.53635	136.1375
34.338	139.4146	34.36102	132.6982	34.42407	134.6769	34.36485	141.3949	34.34062	148.4888	34.55302	136.1325
34.35467	139.413	34.37768	132.696	34.44073	134.673	34.38152	141.3932	34.35728	148.4896	34.56968	136.1338
34.37133	139.4085	34.39435	132.691	34.4574	134.6718	34.39818	141.3936	34.37395	148.4907	34.58635	136.1301
34.388	139.4096	34.41102	132.6899	34.47407	134.6741	34.41485	141.3903	34.39062	148.4878	34.60302	136.1325
34.40467	139.4042	34.42768	132.689	34.49073	134.671	34.43152	141.3887	34.40728	148.4886	34.61968	136.13
34.42133	139.402	34.44435	132.6853	34.5074	134.6696	34.44818	141.3897	34.42395	148.4864	34.63635	136.1265
34.438	139.4041	34.46102	132.6841	34.52407	134.6667	34.46485	141.3892	34.44062	148.4887	34.65302	136.1297
34.45467	139.3981	34.47768	132.6839	34.54073	134.6634	34.48152	141.3903	34.45728	148.4879	34.66968	136.1275
34.47133	139.3978	34.49435	132.6804	34.5574	134.6637	34.49818	141.389	34.47395	148.4878	34.68635	136.1254

34.488	139.3955	34.51102	132.6787	34.57407	134.6603	34.51485	141.3873	34.49062	148.4856	34.70302	136.1229
34.50467	139.3956	34.52768	132.6757	34.59073	134.6613	34.53152	141.3849	34.50728	148.4849	34.71968	136.1223
34.52133	139.3905	34.54435	132.6743	34.6074	134.6574	34.54818	141.3845	34.52395	148.4873	34.73635	136.1238
34.538	139.3924	34.56102	132.6735	34.62407	134.6538	34.56485	141.384	34.54062	148.486	34.75302	136.1228
34.55467	139.3866	34.57768	132.6728	34.64073	134.653	34.58152	141.3829	34.55728	148.4836	34.76968	136.119
34.57133	139.3833	34.59435	132.6685	34.6574	134.6514	34.59818	141.383	34.57395	148.4876	34.78635	136.1165
34.588	139.3864	34.61102	132.6652	34.67407	134.6515	34.61485	141.3836	34.59062	148.4901	34.80302	136.1186
34.60467	139.3837	34.62768	132.6646	34.69073	134.6484	34.63152	141.3804	34.60728	148.4844	34.81968	136.1178
34.62133	139.3845	34.64435	132.6661	34.7074	134.6468	34.64818	141.3784	34.62395	148.4815	34.83635	136.1141
34.638	139.3771	34.66102	132.6637	34.72407	134.6463	34.66485	141.3781	34.64062	148.4864	34.85302	136.1142
34.65467	139.3733	34.67768	132.6632	34.74073	134.6422	34.68152	141.3801	34.65728	148.4839	34.86968	136.1115
34.67133	139.378	34.69435	132.6578	34.7574	134.6423	34.69818	141.3812	34.67395	148.4835	34.88635	136.1136
34.688	139.3722	34.71102	132.6551	34.77407	134.6394	34.71485	141.3784	34.69062	148.4825	34.90302	136.1104
34.70467	139.376	34.72768	132.6527	34.79073	134.6354	34.73152	141.3779	34.70728	148.4843	34.91968	136.1105
34.72133	139.3674	34.74435	132.6522	34.8074	134.6352	34.74818	141.3744	34.72395	148.4818	34.93635	136.1088
34.738	139.3657	34.76102	132.6507	34.82407	134.6354	34.76485	141.3734	34.74062	148.4806	34.95302	136.1072
34.75467	139.3612	34.77768	132.6478	34.84073	134.6316	34.78152	141.3738	34.75728	148.4821	34.96968	136.1062
34.77133	139.3625	34.79435	132.6464	34.8574	134.6299	34.79818	141.3734	34.77395	148.4854	34.98635	136.1051
34.788	139.3599	34.81102	132.6445	34.87407	134.6303	34.81485	141.3707	34.79062	148.4796	35.00302	136.1024
34.80467	139.3575	34.82768	132.6405	34.89073	134.626	34.83152	141.3704	34.80728	148.4788	35.01968	136.1022
34.82133	139.3535	34.84435	132.6365	34.9074	134.6277	34.84818	141.3695	34.82395	148.4823	35.03635	136.102
34.838	139.3518	34.86102	132.6377	34.92407	134.6254	34.86485	141.3678	34.84062	148.4807	35.05302	136.1011
34.85467	139.3521	34.87768	132.6351	34.94073	134.6204	34.88152	141.369	34.85728	148.4803	35.06968	136.0999
34.87133	139.3499	34.89435	132.634	34.9574	134.6218	34.89818	141.3695	34.87395	148.4793	35.08635	136.098
34.888	139.3474	34.91102	132.6322	34.97407	134.6203	34.91485	141.368	34.89062	148.4781	35.10302	136.0986
34.90467	139.3473	34.92768	132.6323	34.99073	134.6192	34.93152	141.3675	34.90728	148.4782	35.11968	136.1014
34.92133	139.3451	34.94435	132.6279	35.0074	134.6167	34.94818	141.3657	34.92395	148.4794	35.13635	136.0974
34.938	139.3416	34.96102	132.6277	35.02407	134.6168	34.96485	141.3638	34.94062	148.4756	35.15302	136.0946
34.95467	139.3419	34.97768	132.6244	35.04073	134.6125	34.98152	141.364	34.95728	148.4766	35.16968	136.0934
34.97133	139.3395	34.99435	132.6199	35.0574	134.6106	34.99818	141.366	34.97395	148.477	35.18635	136.0957
34.988	139.3362	35.01102	132.6189	35.07407	134.6112	35.01485	141.362	34.99062	148.4775	35.20302	136.093
35.00467	139.3334	35.02768	132.622	35.09073	134.6103	35.03152	141.3607	35.00728	148.478	35.21968	136.0913
35.02133	139.3293	35.04435	132.6161	35.1074	134.6079	35.04818	141.3629	35.02395	148.4743	35.23635	136.0895
35.038	139.3314	35.06102	132.6133	35.12407	134.6047	35.06485	141.3615	35.04062	148.477	35.25302	136.0904
35.05467	139.3278	35.07768	132.6113	35.14073	134.6063	35.08152	141.3626	35.05728	148.475	35.26968	136.0865
35.07133	139.325	35.09435	132.6135	35.1574	134.5994	35.09818	141.3615	35.07395	148.4732	35.28635	136.0877
35.088	139.3203	35.11102	132.6093	35.17407	134.5995	35.11485	141.3598	35.09062	148.4737	35.30302	136.0872
35.10467	139.3181	35.12768	132.6107	35.19073	134.6018	35.13152	141.3578	35.10728	148.4735	35.31968	136.0848
35.12133	139.3163	35.14435	132.608	35.2074	134.5931	35.14818	141.3588	35.12395	148.4748	35.33635	136.084
35.138	139.316	35.16102	132.6059	35.22407	134.5884	35.16485	141.3565	35.14062	148.4727	35.35302	136.0821
35.15467	139.3141	35.17768	132.6056	35.24073	134.5925	35.18152	141.3543	35.15728	148.4723	35.36968	136.0816
35.17133	139.313	35.19435	132.601	35.2574	134.5886	35.19818	141.3557	35.17395	148.4705	35.38635	136.0816
35.188	139.3061	35.21102	132.6053	35.27407	134.5925	35.21485	141.3538	35.19062	148.4689	35.40302	136.0803
35.20467	139.3103	35.22768	132.6022	35.29073	134.5886	35.23152	141.3544	35.20728	148.4732	35.41968	136.0782
35.22133	139.3036	35.24435	132.599	35.3074	134.5871	35.24818	141.3518	35.22395	148.4702	35.43635	136.0771
35.238	139.3052	35.26102	132.5976	35.32407	134.5856	35.26485	141.3521	35.24062	148.4686	35.45302	136.0763
35.25467	139.3012	35.27768	132.5936	35.34073	134.5838	35.28152	141.352	35.25728	148.4683	35.46968	136.0745
35.27133	139.2981	35.29435	132.5942	35.3574	134.5792	35.29818	141.3504	35.27395	148.4673	35.48635	136.0753
35.288	139.2946	35.31102	132.5908	35.37407	134.5801	35.31485	141.3478	35.29062	148.4706	35.50302	136.0744
35.30467	139.2953	35.32768	132.5902	35.39073	134.5808	35.33152	141.352	35.30728	148.4643	35.51968	136.0723
35.32133	139.2924	35.34435	132.5878	35.4074	134.5748	35.34818	141.347	35.32395	148.47	35.53635	136.0717
35.338	139.2878	35.36102	132.5834	35.42407	134.576	35.36485	141.3471	35.34062	148.468	35.55302	136.0707
35.35467	139.2883	35.37768	132.5827	35.44073	134.5715	35.38152	141.3464	35.35728	148.4666	35.56968	136.0668
35.37133	139.287	35.39435	132.5813	35.4574	134.569	35.39818	141.3468	35.37395	148.4645	35.58635	136.0655
35.388	139.2865	35.41102	132.5789	35.47407	134.5689	35.41485	141.3486	35.39062	148.4662	35.60302	136.0661
35.40467	139.2834	35.42768	132.5775	35.49073	134.5685	35.43152	141.3448	35.40728	148.4644	35.61968	136.0655
35.42133	139.2795	35.44435	132.5767	35.5074	134.5659	35.44818	141.3464	35.42395	148.4655	35.63635	136.0649
35.438	139.2814	35.46102	132.5732	35.52407	134.5662	35.46485	141.3455	35.44062	148.4644	35.65302	136.0638
35.45467	139.2787	35.47768	132.5711	35.54073	134.5628	35.48152	141.3473	35.45728	148.4643	35.66968	136.0625
35.47133	139.2745	35.49435	132.5683	35.5574	134.5599	35.49818	141.3459	35.47395	148.4629	35.68635	136.0608
35.488	139.2703	35.51102	132.5677	35.57407	134.5598	35.51485	141.344	35.49062	148.4625	35.70302	136.058
35.50467	139.2684	35.52768	132.566	35.59073	134.5597	35.53152	141.3456	35.50728	148.4644	35.71968	136.0599
35.52133	139.2676	35.54435	132.5655	35.6074	134.5553	35.54818	141.346	35.52395	148.463	35.73635	136.0525
35.538	139.2648	35.56102	132.5593	35.62407	134.5547	35.56485	141.3414	35.54062	148.4626	35.75302	136.0581
35.55467	139.2612	35.57768	132.562	35.64073	134.5556	35.58152	141.3434	35.55728	148.4617	35.76968	136.0525
35.57133	139.2639	35.59435	132.557	35.6574	134.5531	35.59818	141.3419	35.57395	148.4601	35.78635	136.0556
35.588	139.2627	35.61102	132.5561	35.67407	134.553	35.61485	141.3401	35.59062	148.4611	35.80302	136.0508
35.60467	139.2599	35.62768	132.5519	35.69073	134.5504	35.63152	141.3368	35.60728	148.4579	35.81968	136.0516
35.62133	139.2582	35.64435	132.5509	35.7074	134.5481	35.64818	141.3362	35.62395	148.4586	35.83635	136.0505

35.638	139.2542	35.66102	132.5509	35.72407	134.5467	35.66485	141.3346	35.64062	148.4577	35.85302	136.0493
35.65467	139.2511	35.67768	132.5488	35.74073	134.546	35.68152	141.3345	35.65728	148.4602	35.86968	136.0458
35.67133	139.2484	35.69435	132.5444	35.7574	134.5442	35.69818	141.3342	35.67395	148.4587	35.88635	136.0427
35.688	139.245	35.71102	132.5446	35.77407	134.5438	35.71485	141.334	35.69062	148.4566	35.90302	136.0461
35.70467	139.2462	35.72768	132.5384	35.79073	134.5418	35.73152	141.3321	35.70728	148.453	35.91968	136.0424
35.72133	139.2463	35.74435	132.5377	35.8074	134.5417	35.74818	141.3318	35.72395	148.4549	35.93635	136.0434
35.738	139.2438	35.76102	132.5359	35.82407	134.541	35.76485	141.3318	35.74062	148.4526	35.95302	136.0402
35.75467	139.2403	35.77768	132.5317	35.84073	134.542	35.78152	141.3305	35.75728	148.4529	35.96968	136.0374
35.77133	139.2395	35.79435	132.5327	35.8574	134.5394	35.79818	141.3333	35.77395	148.4522	35.98635	136.0391
35.788	139.2383	35.81102	132.5291	35.87407	134.5362	35.81485	141.3289	35.79062	148.454	36.00302	136.0341
35.80467	139.2335	35.82768	132.5278	35.89073	134.5372	35.83152	141.3291	35.80728	148.4512	36.01968	136.0334
35.82133	139.2309	35.84435	132.525	35.9074	134.5362	35.84818	141.3301	35.82395	148.4508	36.03635	136.0322
35.838	139.2299	35.86102	132.5245	35.92407	134.5337	35.86485	141.3277	35.84062	148.4509	36.05302	136.0323
35.85467	139.2303	35.87768	132.5216	35.94073	134.5324	35.88152	141.3275	35.85728	148.4531	36.06968	136.0315
35.87133	139.2269	35.89435	132.5191	35.9574	134.5333	35.89818	141.3261	35.87395	148.4539	36.08635	136.0296
35.888	139.2245	35.91102	132.517	35.97407	134.5314	35.91485	141.3249	35.89062	148.4535	36.10302	136.0299
35.90467	139.2227	35.92768	132.5173	35.99073	134.529	35.93152	141.3253	35.90728	148.4514	36.11968	136.0274
35.92133	139.2196	35.94435	132.5165	36.0074	134.5311	35.94818	141.3252	35.92395	148.4535	36.13635	136.0305
35.938	139.2167	35.96102	132.5134	36.02407	134.5291	35.96485	141.3249	35.94062	148.4532	36.15302	136.0261
35.95467	139.2154	35.97768	132.5117	36.04073	134.5289	35.98152	141.3226	35.95728	148.4531	36.16968	136.024
35.97133	139.215	35.99435	132.5114	36.0574	134.527	35.99818	141.3221	35.97395	148.4515	36.18635	136.027
35.988	139.2117	36.01102	132.508	36.07407	134.5257	36.01485	141.3217	35.99062	148.453	36.20302	136.0237
36.00467	139.2096	36.02768	132.5063	36.09073	134.5254	36.03152	141.3207	36.00728	148.4506	36.21968	136.0261
36.02133	139.2094	36.04435	132.5066	36.1074	134.5232	36.04818	141.318	36.02395	148.4501	36.23635	136.0225
36.038	139.2048	36.06102	132.5026	36.12407	134.5224	36.06485	141.3176	36.04062	148.4485	36.25302	136.0212
36.05467	139.2044	36.07768	132.4996	36.14073	134.5196	36.08152	141.317	36.05728	148.452	36.26968	136.0195
36.07133	139.2025	36.09435	132.4987	36.1574	134.5177	36.09818	141.3177	36.07395	148.4491	36.28635	136.0205
36.088	139.2011	36.11102	132.494	36.17407	134.5172	36.11485	141.3156	36.09062	148.4477	36.30302	136.0182
36.10467	139.197	36.12768	132.4962	36.19073	134.516	36.13152	141.3147	36.10728	148.4491	36.31968	136.0146
36.12133	139.1954	36.14435	132.4928	36.2074	134.5186	36.14818	141.3131	36.12395	148.4489	36.33635	136.0171
36.138	139.1929	36.16102	132.4904	36.22407	134.5168	36.16485	141.3126	36.14062	148.4513	36.35302	136.0178
36.15467	139.1904	36.17768	132.4912	36.24073	134.5151	36.18152	141.3115	36.15728	148.4488	36.36968	136.0146
36.17133	139.1895	36.19435	132.4873	36.2574	134.5118	36.19818	141.3111	36.17395	148.4448	36.38635	136.0128
36.188	139.1879	36.21102	132.4863	36.27407	134.5137	36.21485	141.3097	36.19062	148.4479	36.40302	136.0135
36.20467	139.186	36.22768	132.4806	36.29073	134.5109	36.23152	141.3085	36.20728	148.4452	36.41968	136.0105
36.22133	139.1808	36.24435	132.4828	36.3074	134.5118	36.24818	141.3083	36.22395	148.446	36.43635	136.0132
36.238	139.1801	36.26102	132.4788	36.32407	134.5115	36.26485	141.3067	36.24062	148.4493	36.45302	136.0107
36.25467	139.1784	36.27768	132.4774	36.34073	134.508	36.28152	141.3079	36.25728	148.4447	36.46968	136.0098
36.27133	139.1764	36.29435	132.4763	36.3574	134.506	36.29818	141.3074	36.27395	148.4473	36.48635	136.0073
36.288	139.1738	36.31102	132.4713	36.37407	134.5089	36.31485	141.3039	36.29062	148.449	36.50302	136.0065
36.30467	139.1712	36.32768	132.4729	36.39073	134.5056	36.33152	141.3022	36.30728	148.444	36.51968	136.0055
36.32133	139.1707	36.34435	132.4693	36.4074	134.5043	36.34818	141.3043	36.32395	148.4457	36.53635	136.0046
36.338	139.1656	36.36102	132.4678	36.42407	134.5038	36.36485	141.3034	36.34062	148.4458	36.55302	136.0027
36.35467	139.1654	36.37768	132.4683	36.44073	134.4986	36.38152	141.3028	36.35728	148.4458	36.56968	136
36.37133	139.1661	36.39435	132.4625	36.4574	134.4957	36.39818	141.3041	36.37395	148.4446	36.58635	136
36.388	139.1641	36.41102	132.4625	36.47407	134.5008	36.41485	141.3005	36.39062	148.4466	36.60302	136.0014
36.40467	139.1605	36.42768	132.4615	36.49073	134.498	36.43152	141.3016	36.40728	148.4423	36.61968	135.9999
36.42133	139.1608	36.44435	132.4593	36.5074	134.4989	36.44818	141.3021	36.42395	148.4413	36.63635	135.9957
36.438	139.158	36.46102	132.4541	36.52407	134.4966	36.46485	141.3009	36.44062	148.4438	36.65302	135.9954
36.45467	139.1563	36.47768	132.451	36.54073	134.4927	36.48152	141.3015	36.45728	148.4392	36.66968	135.9965
36.47133	139.1533	36.49435	132.4513	36.5574	134.4933	36.49818	141.2996	36.47395	148.4403	36.68635	135.9959
36.488	139.1525	36.51102	132.4492	36.57407	134.4927	36.51485	141.2989	36.49062	148.4415	36.70302	135.9949
36.50467	139.1512	36.52768	132.4477	36.59073	134.4907	36.53152	141.2955	36.50728	148.438	36.71968	135.9915
36.52133	139.15	36.54435	132.4448	36.6074	134.4923	36.54818	141.2965	36.52395	148.4429	36.73635	135.9943
36.538	139.1465	36.56102	132.4439	36.62407	134.4873	36.56485	141.2961	36.54062	148.4392	36.75302	135.9936
36.55467	139.1443	36.57768	132.4402	36.64073	134.4864	36.58152	141.2947	36.55728	148.4383	36.76968	135.9931
36.57133	139.1403	36.59435	132.4393	36.6574	134.4854	36.59818	141.2913	36.57395	148.4398	36.78635	135.9875
36.588	139.1398	36.61102	132.4412	36.67407	134.4872	36.61485	141.2915	36.59062	148.4373	36.80302	135.9889
36.60467	139.1379	36.62768	132.4361	36.69073	134.4882	36.63152	141.2914	36.60728	148.4374	36.81968	135.9903
36.62133	139.1368	36.64435	132.4373	36.7074	134.4847	36.64818	141.2926	36.62395	148.4382	36.83635	135.9892
36.638	139.1352	36.66102	132.433	36.72407	134.4835	36.66485	141.2924	36.64062	148.4381	36.85302	135.983
36.65467	139.1304	36.67768	132.4311	36.74073	134.4821	36.68152	141.2916	36.65728	148.4383	36.86968	135.9854
36.67133	139.1294	36.69435	132.4274	36.7574	134.4791	36.69818	141.291	36.67395	148.4389	36.88635	135.9858
36.688	139.1295	36.71102	132.4275	36.77407	134.4813	36.71485	141.2892	36.69062	148.4381	36.90302	135.9855
36.70467	139.1275	36.72768	132.4262	36.79073	134.4767	36.73152	141.2887	36.70728	148.4367	36.91968	135.9818
36.72133	139.1196	36.74435	132.4239	36.8074	134.4775	36.74818	141.2885	36.72395	148.4398	36.93635	135.9811
36.738	139.1252	36.76102	132.4205	36.82407	134.4759	36.76485	141.2885	36.74062	148.4379	36.95302	135.9819
36.75467	139.1219	36.77768	132.4183	36.84073	134.4748	36.78152	141.287	36.75728	148.4363	36.96968	135.9808
36.77133	139.119	36.79435	132.4187	36.8574	134.4756	36.79818	141.2885	36.77395	148.4383	36.98635	135.9779

36.788	139.1167	36.81102	132.4157	36.87407	134.4713	36.81485	141.2864	36.79062	148.437	37.00302	135.9782
36.80467	139.1137	36.82768	132.4118	36.89073	134.4689	36.83152	141.2859	36.80728	148.438	37.01968	135.9778
36.82133	139.1096	36.84435	132.4109	36.9074	134.4691	36.84818	141.283	36.82395	148.4355	37.03635	135.9767
36.838	139.1101	36.86102	132.4106	36.92407	134.4658	36.86485	141.2851	36.84062	148.431	37.05302	135.9747
36.85467	139.1087	36.87768	132.4111	36.94073	134.4667	36.88152	141.2814	36.85728	148.4315	37.06968	135.973
36.87133	139.1031	36.89435	132.4077	36.9574	134.4644	36.89818	141.2858	36.87395	148.4351	37.08635	135.9738
36.888	139.1033	36.91102	132.4048	36.97407	134.4665	36.91485	141.2836	36.89062	148.4342	37.10302	135.9701
36.90467	139.1013	36.92768	132.3994	36.99073	134.4627	36.93152	141.2831	36.90728	148.4332	37.11968	135.9686
36.92133	139.0966	36.94435	132.3984	37.0074	134.461	36.94818	141.2784	36.92395	148.4336	37.13635	135.9664
36.938	139.096	36.96102	132.3955	37.02407	134.4581	36.96485	141.2767	36.94062	148.4336	37.15302	135.9672
36.95467	139.0966	36.97768	132.3949	37.04073	134.4563	36.98152	141.28	36.95728	148.4291	37.16968	135.9648
36.97133	139.0936	36.99435	132.3937	37.0574	134.4529	36.99818	141.2787	36.97395	148.4288	37.18635	135.9645
36.988	139.0889	37.01102	132.39	37.07407	134.4528	37.01485	141.2781	36.99062	148.4302	37.20302	135.9627
37.00467	139.0925	37.02768	132.3903	37.09073	134.4515	37.03152	141.2789	37.00728	148.4293	37.21968	135.9611
37.02133	139.0868	37.04435	132.3881	37.1074	134.4482	37.04818	141.2781	37.02395	148.4302	37.23635	135.9604
37.038	139.0871	37.06102	132.385	37.12407	134.4481	37.06485	141.2766	37.04062	148.4289	37.25302	135.9563
37.05467	139.0847	37.07768	132.3828	37.14073	134.4459	37.08152	141.2744	37.05728	148.4291	37.26968	135.9546
37.07133	139.0823	37.09435	132.3844	37.1574	134.4427	37.09818	141.2759	37.07395	148.428	37.28635	135.9574
37.088	139.0819	37.11102	132.3847	37.17407	134.4456	37.11485	141.2768	37.09062	148.4279	37.30302	135.9548
37.10467	139.0799	37.12768	132.3813	37.19073	134.4404	37.13152	141.2754	37.10728	148.4262	37.31968	135.952
37.12133	139.0795	37.14435	132.3803	37.2074	134.4406	37.14818	141.2752	37.12395	148.4257	37.33635	135.9533
37.138	139.0774	37.16102	132.3781	37.22407	134.4397	37.16485	141.2745	37.14062	148.4267	37.35302	135.9559
37.15467	139.0758	37.17768	132.3738	37.24073	134.4374	37.18152	141.2716	37.15728	148.4262	37.36968	135.9499
37.17133	139.0755	37.19435	132.3729	37.2574	134.4365	37.19818	141.2733	37.17395	148.4253	37.38635	135.9492
37.188	139.0721	37.21102	132.3726	37.27407	134.4371	37.21485	141.2756	37.19062	148.4263	37.40302	135.9486
37.20467	139.0712	37.22768	132.3719	37.29073	134.4345	37.23152	141.273	37.20728	148.424	37.41968	135.9504
37.22133	139.0631	37.24435	132.3665	37.3074	134.4335	37.24818	141.2729	37.22395	148.4222	37.43635	135.9473
37.238	139.0663	37.26102	132.3685	37.32407	134.4333	37.26485	141.2712	37.24062	148.4212	37.45302	135.9444
37.25467	139.0662	37.27768	132.3634	37.34073	134.4309	37.28152	141.273	37.25728	148.4256	37.46968	135.9445
37.27133	139.0628	37.29435	132.3614	37.3574	134.4279	37.29818	141.2715	37.27395	148.4261	37.48635	135.9424
37.288	139.0642	37.31102	132.3621	37.37407	134.4277	37.31485	141.272	37.29062	148.4248	37.50302	135.9415
37.30467	139.0562	37.32768	132.3626	37.39073	134.4274	37.33152	141.2699	37.30728	148.4216	37.51968	135.9405
37.32133	139.055	37.34435	132.3587	37.4074	134.4234	37.34818	141.2697	37.32395	148.4261	37.53635	135.9399
37.338	139.0583	37.36102	132.3589	37.42407	134.4251	37.36485	141.2692	37.34062	148.4233	37.55302	135.9392
37.35467	139.0542	37.37768	132.3544	37.44073	134.4232	37.38152	141.2693	37.35728	148.4191	37.56968	135.9352
37.37133	139.0509	37.39435	132.3521	37.4574	134.4203	37.39818	141.2683	37.37395	148.4202	37.58635	135.9351
37.388	139.0501	37.41102	132.352	37.47407	134.4176	37.41485	141.2679	37.39062	148.4234	37.60302	135.9337
37.40467	139.0497	37.42768	132.3486	37.49073	134.4163	37.43152	141.2664	37.40728	148.4239	37.61968	135.9335
37.42133	139.0479	37.44435	132.3504	37.5074	134.4154	37.44818	141.2665	37.42395	148.4215	37.63635	135.932
37.438	139.0407	37.46102	132.3481	37.52407	134.4138	37.46485	141.2643	37.44062	148.4207	37.65302	135.9307
37.45467	139.0435	37.47768	132.3436	37.54073	134.4113	37.48152	141.2638	37.45728	148.4202	37.66968	135.9289
37.47133	139.0415	37.49435	132.3423	37.5574	134.4065	37.49818	141.264	37.47395	148.4168	37.68635	135.9269
37.488	139.0379	37.51102	132.3417	37.57407	134.4126	37.51485	141.2641	37.49062	148.4194	37.70302	135.9271
37.50467	139.0393	37.52768	132.3398	37.59073	134.4082	37.53152	141.2601	37.50728	148.4192	37.71968	135.9284
37.52133	139.0388	37.54435	132.3392	37.6074	134.4049	37.54818	141.2601	37.52395	148.4201	37.73635	135.9255
37.538	139.0342	37.56102	132.336	37.62407	134.4055	37.56485	141.2599	37.54062	148.4154	37.75302	135.9235
37.55467	139.036	37.57768	132.3311	37.64073	134.4054	37.58152	141.2594	37.55728	148.4174	37.76968	135.9253
37.57133	139.0296	37.59435	132.3315	37.6574	134.4055	37.59818	141.2583	37.57395	148.4179	37.78635	135.9235
37.588	139.0269	37.61102	132.3319	37.67407	134.399	37.61485	141.2579	37.59062	148.4163	37.80302	135.9232
37.60467	139.0286	37.62768	132.3284	37.69073	134.4009	37.63152	141.2578	37.60728	148.4152	37.81968	135.9215
37.62133	139.0246	37.64435	132.3273	37.7074	134.3988	37.64818	141.2562	37.62395	148.4191	37.83635	135.9179
37.638	139.0224	37.66102	132.3258	37.72407	134.397	37.66485	141.2542	37.64062	148.4181	37.85302	135.9183
37.65467	139.0195	37.67768	132.3249	37.74073	134.3999	37.68152	141.257	37.65728	148.4141	37.86968	135.9182
37.67133	139.0178	37.69435	132.3256	37.7574	134.394	37.69818	141.2549	37.67395	148.4155	37.88635	135.9146
37.688	139.0175	37.71102	132.325	37.77407	134.3917	37.71485	141.255	37.69062	148.4156	37.90302	135.9137
37.70467	139.0147	37.72768	132.3158	37.79073	134.3962	37.73152	141.2539	37.70728	148.4145	37.91968	135.9124
37.72133	139.0115	37.74435	132.3205	37.8074	134.3926	37.74818	141.2569	37.72395	148.4154	37.93635	135.9107
37.738	139.0076	37.76102	132.3163	37.82407	134.3898	37.76485	141.255	37.74062	148.4162	37.95302	135.9086
37.75467	139.0075	37.77768	132.3118	37.84073	134.3894	37.78152	141.2534	37.75728	148.4118	37.96968	135.9099
37.77133	139.0069	37.79435	132.3162	37.8574	134.3916	37.79818	141.2534	37.77395	148.4126	37.98635	135.9085
37.788	139.0035	37.81102	132.3107	37.87407	134.3858	37.81485	141.2521	37.79062	148.4124	38.00302	135.9063
37.80467	139.0021	37.82768	132.31	37.89073	134.384	37.83152	141.2516	37.80728	148.4099	38.01968	135.9051
37.82133	138.9999	37.84435	132.3094	37.9074	134.3834	37.84818	141.2532	37.82395	148.413	38.03635	135.9034
37.838	139.0001	37.86102	132.3072	37.92407	134.381	37.86485	141.2514	37.84062	148.4112	38.05302	135.9003
37.85467	138.998	37.87768	132.3037	37.94073	134.3798	37.88152	141.2504	37.85728	148.4109	38.06968	135.8988
37.87133	138.9937	37.89435	132.3068	37.9574	134.3834	37.89818	141.2522	37.87395	148.4113	38.08635	135.9015
37.888	138.9934	37.91102	132.306	37.97407	134.3813	37.91485	141.2486	37.89062	148.4101	38.10302	135.8972
37.90467	138.9904	37.92768	132.2992	37.99073	134.3777	37.93152	141.2483	37.90728	148.4119	38.11968	135.8978
37.92133	138.9884	37.94435	132.2991	38.0074	134.3773	37.94818	141.2499	37.92395	148.4114	38.13635	135.8942

37.938	138.986	37.96102	132.2976	38.02407	134.3751	37.96485	141.2486	37.94062	148.4096	38.15302	135.8961
37.95467	138.9843	37.97768	132.2952	38.04073	134.374	37.98152	141.2495	37.95728	148.4101	38.16968	135.8933
37.97133	138.9832	37.99435	132.2944	38.0574	134.3729	37.99818	141.2454	37.97395	148.4093	38.18635	135.8916
37.988	138.9809	38.01102	132.2922	38.07407	134.3728	38.01485	141.2455	37.99062	148.4077	38.20302	135.8901
38.00467	138.9781	38.02768	132.2912	38.09073	134.3703	38.03152	141.2445	38.00728	148.4095	38.21968	135.8896
38.02133	138.9745	38.04435	132.2891	38.1074	134.3708	38.04818	141.2443	38.02395	148.4077	38.23635	135.8896
38.038	138.9766	38.06102	132.2867	38.12407	134.3678	38.06485	141.2454	38.04062	148.4093	38.25302	135.8871
38.05467	138.9739	38.07768	132.2857	38.14073	134.3682	38.08152	141.2423	38.05728	148.4084	38.26968	135.8884
38.07133	138.9702	38.09435	132.2834	38.1574	134.3662	38.09818	141.2413	38.07395	148.4112	38.28635	135.8864
38.088	138.9698	38.11102	132.2829	38.17407	134.3643	38.11485	141.2381	38.09062	148.4097	38.30302	135.8858
38.10467	138.9681	38.12768	132.2839	38.19073	134.3652	38.13152	141.24	38.10728	148.4087	38.31968	135.8855
38.12133	138.9666	38.14435	132.2793	38.2074	134.3651	38.14818	141.238	38.12395	148.4057	38.33635	135.8836
38.138	138.9631	38.16102	132.277	38.22407	134.3623	38.16485	141.2389	38.14062	148.4046	38.35302	135.8826
38.15467	138.9624	38.17768	132.2735	38.24073	134.3596	38.18152	141.237	38.15728	148.4044	38.36968	135.8812
38.17133	138.9577	38.19435	132.2743	38.2574	134.3612	38.19818	141.2356	38.17395	148.4047	38.38635	135.8799
38.188	138.958	38.21102	132.273	38.27407	134.3594	38.21485	141.2353	38.19062	148.4043	38.40302	135.8803
38.20467	138.9545	38.22768	132.2699	38.29073	134.3586	38.23152	141.2353	38.20728	148.4054	38.41968	135.8789
38.22133	138.9534	38.24435	132.2684	38.3074	134.3606	38.24818	141.2306	38.22395	148.4032	38.43635	135.8796
38.238	138.9489	38.26102	132.2664	38.32407	134.3592	38.26485	141.2323	38.24062	148.4051	38.45302	135.8781
38.25467	138.9503	38.27768	132.2643	38.34073	134.3575	38.28152	141.2283	38.25728	148.4041	38.46968	135.8768
38.27133	138.9484	38.29435	132.2616	38.3574	134.3544	38.29818	141.2308	38.27395	148.4043	38.48635	135.8778
38.288	138.9472	38.31102	132.2619	38.37407	134.3551	38.31485	141.229	38.29062	148.4045	38.50302	135.8759
38.30467	138.9422	38.32768	132.2585	38.39073	134.3519	38.33152	141.2279	38.30728	148.4031	38.51968	135.8748
38.32133	138.9421	38.34435	132.2559	38.4074	134.3537	38.34818	141.2238	38.32395	148.4014	38.53635	135.876
38.338	138.9415	38.36102	132.2541	38.42407	134.3483	38.36485	141.225	38.34062	148.4021	38.55302	135.8769
38.35467	138.9399	38.37768	132.2499	38.44073	134.3511	38.38152	141.2262	38.35728	148.4022	38.56968	135.8724
38.37133	138.9369	38.39435	132.2488	38.4574	134.3485	38.39818	141.225	38.37395	148.4025	38.58635	135.8724
38.388	138.9365	38.41102	132.247	38.47407	134.349	38.41485	141.2254	38.39062	148.4047	38.60302	135.8706
38.40467	138.9325	38.42768	132.2502	38.49073	134.3466	38.43152	141.2238	38.40728	148.4029	38.61968	135.8707
38.42133	138.9353	38.44435	132.2478	38.5074	134.347	38.44818	141.2234	38.42395	148.4022	38.63635	135.8722
38.438	138.9288	38.46102	132.2445	38.52407	134.3451	38.46485	141.2256	38.44062	148.3981	38.65302	135.8691
38.45467	138.9277	38.47768	132.2423	38.54073	134.3436	38.48152	141.2217	38.45728	148.398	38.66968	135.8687
38.47133	138.9242	38.49435	132.2429	38.5574	134.3412	38.49818	141.2219	38.47395	148.401	38.68635	135.8676
38.488	138.923	38.51102	132.2404	38.57407	134.3403	38.51485	141.2221	38.49062	148.4016	38.70302	135.8648
38.50467	138.9231	38.52768	132.2406	38.59073	134.3418	38.53152	141.2217	38.50728	148.3998	38.71968	135.8628
38.52133	138.9173	38.54435	132.2337	38.6074	134.3412	38.54818	141.224	38.52395	148.3974	38.73635	135.8651
38.538	138.9175	38.56102	132.2348	38.62407	134.3384	38.56485	141.2219	38.54062	148.3965	38.75302	135.8614
38.55467	138.9157	38.57768	132.2329	38.64073	134.3404	38.58152	141.2209	38.55728	148.3957	38.76968	135.8611
38.57133	138.9122	38.59435	132.227	38.6574	134.3377	38.59818	141.2209	38.57395	148.3957	38.78635	135.8587
38.588	138.9099	38.61102	132.229	38.67407	134.3366	38.61485	141.2194	38.59062	148.3971	38.80302	135.8601
38.60467	138.9058	38.62768	132.2283	38.69073	134.3353	38.63152	141.2187	38.60728	148.3955	38.81968	135.8564
38.62133	138.9052	38.64435	132.2241	38.7074	134.3366	38.64818	141.221	38.62395	148.3954	38.83635	135.8565
38.638	138.904	38.66102	132.2218	38.72407	134.3297	38.66485	141.2187	38.64062	148.3947	38.85302	135.8551
38.65467	138.9051	38.67768	132.2189	38.74073	134.3313	38.68152	141.2204	38.65728	148.3944	38.86968	135.8557
38.67133	138.9015	38.69435	132.2205	38.7574	134.3329	38.69818	141.2196	38.67395	148.3964	38.88635	135.8511
38.688	138.8993	38.71102	132.2174	38.77407	134.3277	38.71485	141.2157	38.69062	148.3951	38.90302	135.8523
38.70467	138.8959	38.72768	132.2156	38.79073	134.3307	38.73152	141.2161	38.70728	148.394	38.91968	135.8487
38.72133	138.8959	38.74435	132.2142	38.8074	134.3293	38.74818	141.2154	38.72395	148.3938	38.93635	135.848
38.738	138.8961	38.76102	132.2108	38.82407	134.3264	38.76485	141.2138	38.74062	148.3898	38.95302	135.8442
38.75467	138.892	38.77768	132.2116	38.84073	134.328	38.78152	141.2151	38.75728	148.3925	38.96968	135.8466
38.77133	138.8912	38.79435	132.209	38.8574	134.3254	38.79818	141.2156	38.77395	148.3921	38.98635	135.845
38.788	138.8905	38.81102	132.2081	38.87407	134.325	38.81485	141.2155	38.79062	148.3972	39.00302	135.8453
38.80467	138.8871	38.82768	132.2067	38.89073	134.3242	38.83152	141.2142	38.80728	148.3908	39.01968	135.8453
38.82133	138.8829	38.84435	132.2032	38.9074	134.3238	38.84818	141.2127	38.82395	148.3912	39.03635	135.8427
38.838	138.882	38.86102	132.2057	38.92407	134.3197	38.86485	141.214	38.84062	148.3905	39.05302	135.8411
38.85467	138.8804	38.87768	132.1999	38.94073	134.3169	38.88152	141.2145	38.85728	148.3895	39.06968	135.8407
38.87133	138.88	38.89435	132.2041	38.9574	134.3176	38.89818	141.212	38.87395	148.3922	39.08635	135.8396
38.888	138.8747	38.91102	132.201	38.97407	134.3194	38.91485	141.2136	38.89062	148.3909	39.10302	135.8366
38.90467	138.8755	38.92768	132.1932	38.99073	134.315	38.93152	141.2123	38.90728	148.3889	39.11968	135.8395
38.92133	138.8719	38.94435	132.1942	39.0074	134.3158	38.94818	141.2105	38.92395	148.3904	39.13635	135.8368
38.938	138.8704	38.96102	132.1971	39.02407	134.3121	38.96485	141.2133	38.94062	148.3878	39.15302	135.8337
38.95467	138.8693	38.97768	132.1923	39.04073	134.3119	38.98152	141.2117	38.95728	148.3884	39.16968	135.8327
38.97133	138.8666	38.99435	132.1884	39.0574	134.3082	38.99818	141.2123	38.97395	148.3865	39.18635	135.8314
38.988	138.8636	39.01102	132.1905	39.07407	134.3087	39.01485	141.2092	38.99062	148.3849	39.20302	135.8305
39.00467	138.8615	39.02768	132.1854	39.09073	134.3074	39.03152	141.2112	39.00728	148.3857	39.21968	135.8289
39.02133	138.8634	39.04435	132.1863	39.1074	134.3062	39.04818	141.2105	39.02395	148.385	39.23635	135.8261
39.038	138.8595	39.06102	132.1841	39.12407	134.3049	39.06485	141.2089	39.04062	148.3865	39.25302	135.8271
39.05467	138.8572	39.07768	132.1816	39.14073	134.2995	39.08152	141.2085	39.05728	148.3831	39.26968	135.826
39.07133	138.8555	39.09435	132.1751	39.1574	134.3002	39.09818	141.21	39.07395	148.3842	39.28635	135.8249

39.088	138.8495	39.11102	132.1736	39.17407	134.2984	39.11485	141.2081	39.09062	148.3851	39.30302	135.8226
39.10467	138.8517	39.12768	132.1765	39.19073	134.2947	39.13152	141.2047	39.10728	148.3834	39.31968	135.8227
39.12133	138.851	39.14435	132.1761	39.2074	134.2969	39.14818	141.2058	39.12395	148.385	39.33635	135.8245
39.138	138.849	39.16102	132.1713	39.22407	134.2942	39.16485	141.2058	39.14062	148.3825	39.35302	135.8214
39.15467	138.8441	39.17768	132.171	39.24073	134.2941	39.18152	141.2049	39.15728	148.3826	39.36968	135.8215
39.17133	138.8454	39.19435	132.165	39.2574	134.29	39.19818	141.2052	39.17395	148.3782	39.38635	135.8184
39.188	138.8443	39.21102	132.1645	39.27407	134.2912	39.21485	141.2021	39.19062	148.3781	39.40302	135.82
39.20467	138.8405	39.22768	132.1645	39.29073	134.29	39.23152	141.2014	39.20728	148.3842	39.41968	135.8163
39.22133	138.8404	39.24435	132.1643	39.3074	134.29	39.24818	141.2	39.22395	148.3798	39.43635	135.8168
39.238	138.8361	39.26102	132.1636	39.32407	134.2896	39.26485	141.2031	39.24062	148.3769	39.45302	135.8138
39.25467	138.8377	39.27768	132.1614	39.34073	134.2877	39.28152	141.2014	39.25728	148.3794	39.46968	135.8138
39.27133	138.8323	39.29435	132.1594	39.3574	134.285	39.29818	141.202	39.27395	148.3783	39.48635	135.8139
39.288	138.8305	39.31102	132.1567	39.37407	134.286	39.31485	141.1974	39.29062	148.3788	39.50302	135.8111
39.30467	138.8294	39.32768	132.1543	39.39073	134.2831	39.33152	141.1994	39.30728	148.379	39.51968	135.8099
39.32133	138.8282	39.34435	132.1522	39.4074	134.2807	39.34818	141.1952	39.32395	148.3773	39.53635	135.8101
39.338	138.8265	39.36102	132.1535	39.42407	134.284	39.36485	141.1956	39.34062	148.3751	39.55302	135.8086
39.35467	138.8229	39.37768	132.1443	39.44073	134.2816	39.38152	141.1973	39.35728	148.3772	39.56968	135.8086
39.37133	138.8228	39.39435	132.1504	39.4574	134.2791	39.39818	141.1973	39.37395	148.3794	39.58635	135.8071
39.388	138.82	39.41102	132.148	39.47407	134.2757	39.41485	141.1925	39.39062	148.3737	39.60302	135.8068
39.40467	138.8183	39.42768	132.1452	39.49073	134.2763	39.43152	141.1927	39.40728	148.3742	39.61968	135.804
39.42133	138.8132	39.44435	132.1418	39.5074	134.2779	39.44818	141.1921	39.42395	148.3782	39.63635	135.8056
39.438	138.8139	39.46102	132.1419	39.52407	134.2754	39.46485	141.1921	39.44062	148.3758	39.65302	135.8041
39.45467	138.8122	39.47768	132.1434	39.54073	134.2713	39.48152	141.1944	39.45728	148.3771	39.66968	135.8036
39.47133	138.8093	39.49435	132.1436	39.5574	134.2718	39.49818	141.1957	39.47395	148.3768	39.68635	135.8006
39.488	138.8059	39.51102	132.135	39.57407	134.2705	39.51485	141.1912	39.49062	148.3728	39.70302	135.8024
39.50467	138.8046	39.52768	132.1381	39.59073	134.2682	39.53152	141.1916	39.50728	148.374	39.71968	135.8015
39.52133	138.8068	39.54435	132.1374	39.6074	134.2684	39.54818	141.1891	39.52395	148.3751	39.73635	135.8007
39.538	138.8008	39.56102	132.1354	39.62407	134.2669	39.56485	141.1903	39.54062	148.373	39.75302	135.7974
39.55467	138.7987	39.57768	132.1334	39.64073	134.2643	39.58152	141.1865	39.55728	148.3754	39.76968	135.7974
39.57133	138.7961	39.59435	132.1293	39.6574	134.2621	39.59818	141.1891	39.57395	148.3732	39.78635	135.7963
39.588	138.794	39.61102	132.1319	39.67407	134.2615	39.61485	141.1864	39.59062	148.3706	39.80302	135.7967
39.60467	138.7957	39.62768	132.1271	39.69073	134.2593	39.63152	141.1867	39.60728	148.3707	39.81968	135.7947
39.62133	138.7925	39.64435	132.1256	39.7074	134.2579	39.64818	141.1857	39.62395	148.3719	39.83635	135.7923
39.638	138.7883	39.66102	132.1222	39.72407	134.2574	39.66485	141.1854	39.64062	148.3725	39.85302	135.7921
39.65467	138.7869	39.67768	132.1204	39.74073	134.2576	39.68152	141.1845	39.65728	148.37	39.86968	135.7902
39.67133	138.7879	39.69435	132.119	39.7574	134.2537	39.69818	141.1855	39.67395	148.3698	39.88635	135.7877
39.688	138.7844	39.71102	132.1181	39.77407	134.2525	39.71485	141.1852	39.69062	148.3692	39.90302	135.7878
39.70467	138.7845	39.72768	132.1196	39.79073	134.2509	39.73152	141.184	39.70728	148.3686	39.91968	135.7872
39.72133	138.7816	39.74435	132.1189	39.8074	134.25	39.74818	141.1844	39.72395	148.3665	39.93635	135.7853
39.738	138.7786	39.76102	132.1184	39.82407	134.2486	39.76485	141.1822	39.74062	148.3677	39.95302	135.7839
39.75467	138.7788	39.77768	132.1152	39.84073	134.2503	39.78152	141.1812	39.75728	148.3676	39.96968	135.784
39.77133	138.7737	39.79435	132.1119	39.8574	134.2481	39.79818	141.183	39.77395	148.3687	39.98635	135.7808
39.788	138.7746	39.81102	132.1062	39.87407	134.2467	39.81485	141.181	39.79062	148.3712	40.00302	135.7809
39.80467	138.7727	39.82768	132.1097	39.89073	134.2449	39.83152	141.1804	39.80728	148.3695	40.01968	135.7809
39.82133	138.7712	39.84435	132.1079	39.9074	134.2471	39.84818	141.1796	39.82395	148.3678	40.03635	135.7802
39.838	138.7691	39.86102	132.1039	39.92407	134.2458	39.86485	141.1787	39.84062	148.3674	40.05302	135.7765
39.85467	138.7665	39.87768	132.1037	39.94073	134.2418	39.88152	141.1787	39.85728	148.3684	40.06968	135.7778
39.87133	138.7645	39.89435	132.1002	39.9574	134.2438	39.89818	141.1791	39.87395	148.3687	40.08635	135.7796
39.888	138.7625	39.91102	132.0999	39.97407	134.2409	39.91485	141.1755	39.89062	148.366	40.10302	135.7784
39.90467	138.7636	39.92768	132.1002	39.99073	134.2365	39.93152	141.1764	39.90728	148.3685	40.11968	135.7741
39.92133	138.76	39.94435	132.0936	40.0074	134.2402	39.94818	141.1732	39.92395	148.3683	40.13635	135.777
39.938	138.7564	39.96102	132.0941	40.02407	134.2399	39.96485	141.1742	39.94062	148.3669	40.15302	135.7754
39.95467	138.7577	39.97768	132.094	40.04073	134.2389	39.98152	141.1747	39.95728	148.3661	40.16968	135.7737
39.97133	138.756	39.99435	132.0891	40.0574	134.2359	39.99818	141.1724	39.97395	148.3665	40.18635	135.7716
39.988	138.7514	40.01102	132.0883	40.07407	134.2356	40.01485	141.1728	39.99062	148.3639	40.20302	135.7728
40.00467	138.7519	40.02768	132.0873	40.09073	134.2323	40.03152	141.1721	40.00728	148.3691	40.21968	135.7685
40.02133	138.7517	40.04435	132.0836	40.1074	134.234	40.04818	141.1706	40.02395	148.3674	40.23635	135.769
40.038	138.7437	40.06102	132.0831	40.12407	134.2333	40.06485	141.1696	40.04062	148.3666	40.25302	135.7701
40.05467	138.744	40.07768	132.0837	40.14073	134.2354	40.08152	141.1698	40.05728	148.3649	40.26968	135.767
40.07133	138.7413	40.09435	132.0793	40.1574	134.2308	40.09818	141.1665	40.07395	148.3658	40.28635	135.7635
40.088	138.7395	40.11102	132.0775	40.17407	134.2302	40.11485	141.168	40.09062	148.3664	40.30302	135.7664
40.10467	138.7389	40.12768	132.0778	40.19073	134.2291	40.13152	141.1684	40.10728	148.3657	40.31968	135.7627
40.12133	138.7378	40.14435	132.0742	40.2074	134.2275	40.14818	141.1661	40.12395	148.3669	40.33635	135.7641
40.138	138.7357	40.16102	132.0745	40.22407	134.2269	40.16485	141.1661	40.14062	148.3668	40.35302	135.7636
40.15467	138.7332	40.17768	132.0733	40.24073	134.2271	40.18152	141.1651	40.15728	148.3661	40.36968	135.7607
40.17133	138.7303	40.19435	132.0723	40.2574	134.2244	40.19818	141.1645	40.17395	148.3666	40.38635	135.7599
40.188	138.7319	40.21102	132.0684	40.27407	134.2216	40.21485	141.1647	40.19062	148.3667	40.40302	135.7584
40.20467	138.7309	40.22768	132.0663	40.29073	134.222	40.23152	141.1645	40.20728	148.3675	40.41968	135.7549
40.22133	138.7266	40.24435	132.0675	40.3074	134.2223	40.24818	141.1611	40.22395	148.3654	40.43635	135.7551

40.238	138.7241	40.26102	132.065	40.32407	134.2179	40.26485	141.1613	40.24062	148.3669	40.45302	135.7555
40.25467	138.7223	40.27768	132.0618	40.34073	134.2185	40.28152	141.1567	40.25728	148.3627	40.46968	135.7544
40.27133	138.7187	40.29435	132.061	40.3574	134.2161	40.29818	141.1642	40.27395	148.367	40.48635	135.7519
40.288	138.7203	40.31102	132.0614	40.37407	134.2175	40.31485	141.1605	40.29062	148.3661	40.50302	135.754
40.30467	138.7166	40.32768	132.0593	40.39073	134.2144	40.33152	141.1592	40.30728	148.3632	40.51968	135.7495
40.32133	138.7157	40.34435	132.0582	40.4074	134.2111	40.34818	141.1597	40.32395	148.3607	40.53635	135.7488
40.338	138.7125	40.36102	132.0553	40.42407	134.2104	40.36485	141.1559	40.34062	148.3561	40.55302	135.7498
40.35467	138.7108	40.37768	132.055	40.44073	134.2094	40.38152	141.1571	40.35728	148.3629	40.56968	135.747
40.37133	138.7137	40.39435	132.051	40.4574	134.2092	40.39818	141.1565	40.37395	148.3614	40.58635	135.7403
40.388	138.7118	40.41102	132.0489	40.47407	134.2052	40.41485	141.1547	40.39062	148.3599	40.60302	135.7406
40.40467	138.7079	40.42768	132.0504	40.49073	134.2043	40.43152	141.1547	40.40728	148.3597	40.61968	135.7406
40.42133	138.7057	40.44435	132.0493	40.5074	134.2063	40.44818	141.1523	40.42395	148.3596	40.63635	135.7384
40.438	138.7047	40.46102	132.049	40.52407	134.2026	40.46485	141.156	40.44062	148.3573	40.65302	135.7395
40.45467	138.7026	40.47768	132.0471	40.54073	134.2029	40.48152	141.1521	40.45728	148.3567	40.66968	135.7354
40.47133	138.6988	40.49435	132.0433	40.5574	134.202	40.49818	141.1526	40.47395	148.3565	40.68635	135.734
40.488	138.6998	40.51102	132.0396	40.57407	134.2017	40.51485	141.1497	40.49062	148.3549	40.70302	135.7351
40.50467	138.695	40.52768	132.039	40.59073	134.201	40.53152	141.1507	40.50728	148.3563	40.71968	135.7304
40.52133	138.6958	40.54435	132.036	40.6074	134.1992	40.54818	141.1508	40.52395	148.3553	40.73635	135.7305
40.538	138.692	40.56102	132.0388	40.62407	134.1976	40.56485	141.1494	40.54062	148.3565	40.75302	135.7268
40.55467	138.6901	40.57768	132.0355	40.64073	134.1961	40.58152	141.1483	40.55728	148.3536	40.76968	135.7293
40.57133	138.6862	40.59435	132.035	40.6574	134.1943	40.59818	141.1487	40.57395	148.3562	40.78635	135.7269
40.588	138.6846	40.61102	132.03	40.67407	134.1905	40.61485	141.1484	40.59062	148.3552	40.80302	135.7267
40.60467	138.6853	40.62768	132.0345	40.69073	134.1919	40.63152	141.149	40.60728	148.3533	40.81968	135.7244
40.62133	138.6839	40.64435	132.0268	40.7074	134.1912	40.64818	141.147	40.62395	148.3532	40.83635	135.7232
40.638	138.6807	40.66102	132.0277	40.72407	134.1911	40.66485	141.1491	40.64062	148.3497	40.85302	135.7216
40.65467	138.6779	40.67768	132.0251	40.74073	134.1901	40.68152	141.1461	40.65728	148.3481	40.86968	135.7206
40.67133	138.6759	40.69435	132.0251	40.7574	134.1874	40.69818	141.1474	40.67395	148.3495	40.88635	135.7187
40.688	138.6751	40.71102	132.0226	40.77407	134.1881	40.71485	141.146	40.69062	148.3484	40.90302	135.717
40.70467	138.6729	40.72768	132.0218	40.79073	134.1855	40.73152	141.1466	40.70728	148.3479	40.91968	135.7166
40.72133	138.6704	40.74435	132.0213	40.8074	134.1844	40.74818	141.1454	40.72395	148.3456	40.93635	135.7148
40.738	138.6691	40.76102	132.0201	40.82407	134.1854	40.76485	141.1465	40.74062	148.3452	40.95302	135.7138
40.75467	138.6668	40.77768	132.0153	40.84073	134.1832	40.78152	141.1443	40.75728	148.3464	40.96968	135.7138
40.77133	138.6647	40.79435	132.0125	40.8574	134.182	40.79818	141.1444	40.77395	148.346	40.98635	135.7101
40.788	138.6649	40.81102	132.0104	40.87407	134.1821	40.81485	141.1446	40.79062	148.3487	41.00302	135.712
40.80467	138.6596	40.82768	132.0133	40.89073	134.1789	40.83152	141.1429	40.80728	148.3486	41.01968	135.7099
40.82133	138.6585	40.84435	132.0102	40.9074	134.1782	40.84818	141.1414	40.82395	148.3453	41.03635	135.7097
40.838	138.6566	40.86102	132.0077	40.92407	134.1767	40.86485	141.1416	40.84062	148.3449	41.05302	135.7086
40.85467	138.6573	40.87768	132.0083	40.94073	134.1737	40.88152	141.1414	40.85728	148.3478	41.06968	135.7073
40.87133	138.6548	40.89435	132.0071	40.9574	134.1731	40.89818	141.138	40.87395	148.3475	41.08635	135.7049
40.888	138.652	40.91102	132.0079	40.97407	134.1714	40.91485	141.1399	40.89062	148.3439	41.10302	135.7068
40.90467	138.6512	40.92768	132.0023	40.99073	134.1701	40.93152	141.1398	40.90728	148.3468	41.11968	135.7025
40.92133	138.6509	40.94435	132.0028	41.0074	134.1678	40.94818	141.1371	40.92395	148.3442	41.13635	135.7021
40.938	138.6497	40.96102	131.9996	41.02407	134.1666	40.96485	141.1368	40.94062	148.3437	41.15302	135.6993
40.95467	138.6473	40.97768	131.9985	41.04073	134.1653	40.98152	141.1365	40.95728	148.345	41.16968	135.7011
40.97133	138.646	40.99435	131.9952	41.0574	134.1607	40.99818	141.1359	40.97395	148.3437	41.18635	135.7004
40.988	138.645	41.01102	131.9948	41.07407	134.161	41.01485	141.1371	40.99062	148.3421	41.20302	135.6962
41.00467	138.6439	41.02768	131.9953	41.09073	134.1617	41.03152	141.1337	41.00728	148.3459	41.21968	135.6988
41.02133	138.6453	41.04435	131.9934	41.1074	134.1606	41.04818	141.1332	41.02395	148.3411	41.23635	135.6951
41.038	138.6387	41.06102	131.9933	41.12407	134.1594	41.06485	141.1344	41.04062	148.3442	41.25302	135.6938
41.05467	138.6429	41.07768	131.9885	41.14073	134.1589	41.08152	141.1329	41.05728	148.3419	41.26968	135.6936
41.07133	138.6363	41.09435	131.9886	41.1574	134.156	41.09818	141.132	41.07395	148.3456	41.28635	135.6918
41.088	138.6346	41.11102	131.9876	41.17407	134.1527	41.11485	141.133	41.09062	148.3417	41.30302	135.6915
41.10467	138.6332	41.12768	131.9863	41.19073	134.1517	41.13152	141.1321	41.10728	148.3422	41.31968	135.6908
41.12133	138.6304	41.14435	131.9825	41.2074	134.1521	41.14818	141.1296	41.12395	148.3418	41.33635	135.6887
41.138	138.6297	41.16102	131.9816	41.22407	134.1517	41.16485	141.1282	41.14062	148.345	41.35302	135.6868
41.15467	138.6299	41.17768	131.9828	41.24073	134.1505	41.18152	141.1291	41.15728	148.3434	41.36968	135.6849
41.17133	138.6278	41.19435	131.9795	41.2574	134.1492	41.19818	141.1289	41.17395	148.343	41.38635	135.6874
41.188	138.6241	41.21102	131.977	41.27407	134.1482	41.21485	141.1312	41.19062	148.3431	41.40302	135.6838
41.20467	138.6234	41.22768	131.9769	41.29073	134.1461	41.23152	141.1266	41.20728	148.3438	41.41968	135.682
41.22133	138.6214	41.24435	131.9749	41.3074	134.1455	41.24818	141.1272	41.22395	148.3467	41.43635	135.6843
41.238	138.6196	41.26102	131.9754	41.32407	134.1432	41.26485	141.1277	41.24062	148.3444	41.45302	135.6806
41.25467	138.6185	41.27768	131.9735	41.34073	134.1461	41.28152	141.1257	41.25728	148.3418	41.46968	135.6797
41.27133	138.615	41.29435	131.9699	41.3574	134.1416	41.29818	141.1229	41.27395	148.3451	41.48635	135.6785
41.288	138.6152	41.31102	131.9695	41.37407	134.1397	41.31485	141.1237	41.29062	148.3432	41.50302	135.6795
41.30467	138.6112	41.32768	131.9678	41.39073	134.1384	41.33152	141.1239	41.30728	148.3441	41.51968	135.6787
41.32133	138.6118	41.34435	131.9666	41.4074	134.1378	41.34818	141.1218	41.32395	148.3435	41.53635	135.6782
41.338	138.6079	41.36102	131.9633	41.42407	134.1355	41.36485	141.1184	41.34062	148.3444	41.55302	135.6751
41.35467	138.6022	41.37768	131.9622	41.44073	134.138	41.38152	141.1209	41.35728	148.343	41.56968	135.6746
41.37133	138.6046	41.39435	131.9595	41.4574	134.1352	41.39818	141.1171	41.37395	148.3439	41.58635	135.6737

41.388	138.6012	41.41102	131.9591	41.47407	134.1347	41.41485	141.1122	41.39062	148.3432	41.60302	135.6732
41.40467	138.5987	41.42768	131.9548	41.49073	134.1313	41.43152	141.1162	41.40728	148.3412	41.61968	135.6698
41.42133	138.5978	41.44435	131.9559	41.5074	134.13	41.44818	141.1143	41.42395	148.3426	41.63635	135.6676
41.438	138.5966	41.46102	131.9511	41.52407	134.1311	41.46485	141.1152	41.44062	148.3412	41.65302	135.6719
41.45467	138.5933	41.47768	131.9518	41.54073	134.128	41.48152	141.1163	41.45728	148.3448	41.66968	135.6683
41.47133	138.5893	41.49435	131.9519	41.5574	134.1281	41.49818	141.1141	41.47395	148.343	41.68635	135.667
41.488	138.5903	41.51102	131.9481	41.57407	134.1278	41.51485	141.112	41.49062	148.3416	41.70302	135.6635
41.50467	138.5846	41.52768	131.9474	41.59073	134.1276	41.53152	141.1087	41.50728	148.3385	41.71968	135.6638
41.52133	138.5843	41.54435	131.9454	41.6074	134.1265	41.54818	141.1103	41.52395	148.3408	41.73635	135.6626
41.538	138.5858	41.56102	131.9412	41.62407	134.124	41.56485	141.1112	41.54062	148.3389	41.75302	135.6604
41.55467	138.5817	41.57768	131.9422	41.64073	134.1244	41.58152	141.1085	41.55728	148.3419	41.76968	135.6584
41.57133	138.5758	41.59435	131.9387	41.6574	134.1223	41.59818	141.1075	41.57395	148.3426	41.78635	135.6596
41.588	138.5761	41.61102	131.9418	41.67407	134.1212	41.61485	141.1081	41.59062	148.3385	41.80302	135.6582
41.60467	138.5736	41.62768	131.9371	41.69073	134.1207	41.63152	141.1068	41.60728	148.3412	41.81968	135.6577
41.62133	138.5698	41.64435	131.9265	41.7074	134.1206	41.64818	141.1059	41.62395	148.3397	41.83635	135.6547
41.638	138.5711	41.66102	131.9326	41.72407	134.1176	41.66485	141.1059	41.64062	148.3397	41.85302	135.6562
41.65467	138.5688	41.67768	131.9332	41.74073	134.1147	41.68152	141.1058	41.65728	148.3377	41.86968	135.6543
41.67133	138.5667	41.69435	131.9262	41.7574	134.115	41.69818	141.1044	41.67395	148.3418	41.88635	135.6524
41.688	138.5663	41.71102	131.932	41.77407	134.112	41.71485	141.1019	41.69062	148.3377	41.90302	135.6507
41.70467	138.5617	41.72768	131.9306	41.79073	134.1137	41.73152	141.1003	41.70728	148.3386	41.91968	135.6501
41.72133	138.5604	41.74435	131.9262	41.8074	134.109	41.74818	141.0999	41.72395	148.3395	41.93635	135.6496
41.738	138.5576	41.76102	131.9226	41.82407	134.109	41.76485	141.0973	41.74062	148.3363	41.95302	135.6496
41.75467	138.5561	41.77768	131.9234	41.84073	134.1076	41.78152	141.1009	41.75728	148.3357	41.96968	135.6478
41.77133	138.556	41.79435	131.9186	41.8574	134.1076	41.79818	141.1015	41.77395	148.3366	41.98635	135.6458
41.788	138.5513	41.81102	131.9175	41.87407	134.1066	41.81485	141.0981	41.79062	148.335	42.00302	135.6431
41.80467	138.5548	41.82768	131.9156	41.89073	134.1036	41.83152	141.0977	41.80728	148.3352	42.01968	135.6419
41.82133	138.5507	41.84435	131.9201	41.9074	134.1044	41.84818	141.0963	41.82395	148.3332	42.03635	135.6402
41.838	138.5492	41.86102	131.9216	41.92407	134.1013	41.86485	141.096	41.84062	148.3363	42.05302	135.6394
41.85467	138.5498	41.87768	131.9097	41.94073	134.1013	41.88152	141.093	41.85728	148.3355	42.06968	135.6372
41.87133	138.5443	41.89435	131.9158	41.9574	134.1009	41.89818	141.0958	41.87395	148.3359	42.08635	135.6371
41.888	138.5401	41.91102	131.911	41.97407	134.1009	41.91485	141.092	41.89062	148.3352	42.10302	135.6387
41.90467	138.5429	41.92768	131.9089	41.99073	134.0991	41.93152	141.0903	41.90728	148.3361	42.11968	135.6355
41.92133	138.5438	41.94435	131.9067	42.0074	134.097	41.94818	141.0905	41.92395	148.3343	42.13635	135.6362
41.938	138.5379	41.96102	131.9054	42.02407	134.0925	41.96485	141.0902	41.94062	148.3338	42.15302	135.6326
41.95467	138.5354	41.97768	131.9049	42.04073	134.0982	41.98152	141.0874	41.95728	148.334	42.16968	135.6348
41.97133	138.5359	41.99435	131.9041	42.0574	134.0951	41.99818	141.0898	41.97395	148.3361	42.18635	135.6332
41.988	138.5338	42.01102	131.9045	42.07407	134.0922	42.01485	141.0896	41.99062	148.3342	42.20302	135.6347
42.00467	138.5323	42.02768	131.9047	42.09073	134.0907	42.03152	141.0845	42.00728	148.3304	42.21968	135.6298
42.02133	138.5311	42.04435	131.8981	42.1074	134.0919	42.04818	141.0841	42.02395	148.3304	42.23635	135.6303
42.038	138.5296	42.06102	131.8974	42.12407	134.0854	42.06485	141.0865	42.04062	148.3334	42.25302	135.6258
42.05467	138.5286	42.07768	131.8981	42.14073	134.088	42.08152	141.0865	42.05728	148.3321	42.26968	135.627
42.07133	138.5228	42.09435	131.8948	42.1574	134.0866	42.09818	141.085	42.07395	148.3344	42.28635	135.6244
42.088	138.5251	42.11102	131.8952	42.17407	134.0875	42.11485	141.0851	42.09062	148.3333	42.30302	135.6235
42.10467	138.5221	42.12768	131.8892	42.19073	134.0873	42.13152	141.0829	42.10728	148.3294	42.31968	135.6223
42.12133	138.523	42.14435	131.8913	42.2074	134.0855	42.14818	141.082	42.12395	148.328	42.33635	135.6236
42.138	138.5201	42.16102	131.888	42.22407	134.0814	42.16485	141.0817	42.14062	148.3329	42.35302	135.6204
42.15467	138.5158	42.17768	131.8884	42.24073	134.0825	42.18152	141.0817	42.15728	148.332	42.36968	135.6202
42.17133	138.5142	42.19435	131.8876	42.2574	134.0824	42.19818	141.0817	42.17395	148.3298	42.38635	135.6166
42.188	138.517	42.21102	131.8859	42.27407	134.0809	42.21485	141.0786	42.19062	148.3301	42.40302	135.6146
42.20467	138.5116	42.22768	131.883	42.29073	134.0808	42.23152	141.0799	42.20728	148.328	42.41968	135.6163
42.22133	138.5119	42.24435	131.8809	42.3074	134.0769	42.24818	141.079	42.22395	148.3322	42.43635	135.6126
42.238	138.5073	42.26102	131.8807	42.32407	134.0743	42.26485	141.0792	42.24062	148.3309	42.45302	135.6113
42.25467	138.5076	42.27768	131.8787	42.34073	134.071	42.28152	141.0788	42.25728	148.3275	42.46968	135.6129
42.27133	138.5036	42.29435	131.8761	42.3574	134.0725	42.29818	141.0782	42.27395	148.3287	42.48635	135.6103
42.288	138.505	42.31102	131.8755	42.37407	134.0724	42.31485	141.0771	42.29062	148.3299	42.50302	135.6085
42.30467	138.5023	42.32768	131.8738	42.39073	134.0701	42.33152	141.0759	42.30728	148.3247	42.51968	135.6084
42.32133	138.501	42.34435	131.872	42.4074	134.0679	42.34818	141.0728	42.32395	148.3289	42.53635	135.6072
42.338	138.4995	42.36102	131.8693	42.42407	134.0666	42.36485	141.0746	42.34062	148.3294	42.55302	135.6048
42.35467	138.4983	42.37768	131.8706	42.44073	134.0671	42.38152	141.0751	42.35728	148.3305	42.56968	135.6023
42.37133	138.4964	42.39435	131.8661	42.4574	134.064	42.39818	141.0743	42.37395	148.3287	42.58635	135.6046
42.388	138.4932	42.41102	131.866	42.47407	134.0646	42.41485	141.0733	42.39062	148.3262	42.60302	135.6012
42.40467	138.4921	42.42768	131.8634	42.49073	134.0615	42.43152	141.0709	42.40728	148.3282	42.61968	135.6019
42.42133	138.4893	42.44435	131.8601	42.5074	134.0618	42.44818	141.0717	42.42395	148.3292	42.63635	135.6021
42.438	138.4897	42.46102	131.8574	42.52407	134.0609	42.46485	141.0737	42.44062	148.3292	42.65302	135.5997
42.45467	138.4864	42.47768	131.8579	42.54073	134.0594	42.48152	141.0697	42.45728	148.3305	42.66968	135.6004
42.47133	138.4835	42.49435	131.858	42.5574	134.0575	42.49818	141.0712	42.47395	148.3289	42.68635	135.5998
42.488	138.4817	42.51102	131.8547	42.57407	134.0585	42.51485	141.07	42.49062	148.3272	42.70302	135.5968
42.50467	138.4795	42.52768	131.8519	42.59073	134.0554	42.53152	141.0678	42.50728	148.3272	42.71968	135.5942
42.52133	138.4775	42.54435	131.8507	42.6074	134.0542	42.54818	141.0669	42.52395	148.3266	42.73635	135.5989

42.538	138.4741	42.56102	131.8482	42.62407	134.0532	42.56485	141.0689	42.54062	148.3293	42.75302	135.5936
42.55467	138.4735	42.57768	131.8469	42.64073	134.049	42.58152	141.0676	42.55728	148.328	42.76968	135.593
42.57133	138.4715	42.59435	131.8461	42.6574	134.0506	42.59818	141.0667	42.57395	148.3271	42.78635	135.5938
42.588	138.4707	42.61102	131.843	42.67407	134.0488	42.61485	141.0663	42.59062	148.328	42.80302	135.5944
42.60467	138.4677	42.62768	131.8406	42.69073	134.0487	42.63152	141.0631	42.60728	148.3273	42.81968	135.5918
42.62133	138.4662	42.64435	131.8405	42.7074	134.0424	42.64818	141.0625	42.62395	148.3285	42.83635	135.5904
42.638	138.4658	42.66102	131.8374	42.72407	134.0449	42.66485	141.0641	42.64062	148.33	42.85302	135.5863
42.65467	138.4629	42.67768	131.836	42.74073	134.0437	42.68152	141.0644	42.65728	148.3289	42.86968	135.5912
42.67133	138.4614	42.69435	131.8353	42.7574	134.0427	42.69818	141.0612	42.67395	148.3277	42.88635	135.589
42.688	138.4599	42.71102	131.8337	42.77407	134.0431	42.71485	141.061	42.69062	148.3267	42.90302	135.5855
42.70467	138.461	42.72768	131.8306	42.79073	134.0413	42.73152	141.0613	42.70728	148.3272	42.91968	135.5852
42.72133	138.4568	42.74435	131.8315	42.8074	134.0381	42.74818	141.0589	42.72395	148.3269	42.93635	135.5862
42.738	138.4546	42.76102	131.8253	42.82407	134.0406	42.76485	141.0586	42.74062	148.3276	42.95302	135.5852
42.75467	138.4534	42.77768	131.8253	42.84073	134.0376	42.78152	141.0607	42.75728	148.3289	42.96968	135.583
42.77133	138.452	42.79435	131.8233	42.8574	134.0384	42.79818	141.0604	42.77395	148.3259	42.98635	135.581
42.788	138.4514	42.81102	131.8216	42.87407	134.0355	42.81485	141.0569	42.79062	148.3254	43.00302	135.579
42.80467	138.4479	42.82768	131.8219	42.89073	134.0344	42.83152	141.059	42.80728	148.326	43.01968	135.5801
42.82133	138.4449	42.84435	131.822	42.9074	134.0329	42.84818	141.0559	42.82395	148.3269	43.03635	135.5772
42.838	138.4459	42.86102	131.8188	42.92407	134.0336	42.86485	141.0559	42.84062	148.3249	43.05302	135.5748
42.85467	138.4471	42.87768	131.8191	42.94073	134.0311	42.88152	141.0538	42.85728	148.3251	43.06968	135.5716
42.87133	138.4408	42.89435	131.8175	42.9574	134.0339	42.89818	141.0548	42.87395	148.324	43.08635	135.5759
42.888	138.4404	42.91102	131.8162	42.97407	134.0311	42.91485	141.0547	42.89062	148.3256	43.10302	135.5746
42.90467	138.4388	42.92768	131.8147	42.99073	134.0288	42.93152	141.0544	42.90728	148.3218	43.11968	135.5725
42.92133	138.4353	42.94435	131.8091	43.0074	134.0293	42.94818	141.0521	42.92395	148.3254	43.13635	135.5708
42.938	138.4357	42.96102	131.8128	43.02407	134.0282	42.96485	141.0547	42.94062	148.322	43.15302	135.5698
42.95467	138.4326	42.97768	131.8089	43.04073	134.0281	42.98152	141.0544	42.95728	148.3215	43.16968	135.5704
42.97133	138.4328	42.99435	131.8093	43.0574	134.0269	42.99818	141.0521	42.97395	148.3225	43.18635	135.5689
42.988	138.4296	43.01102	131.8077	43.07407	134.0287	43.01485	141.0527	42.99062	148.3224	43.20302	135.5692
43.00467	138.4308	43.02768	131.8078	43.09073	134.0242	43.03152	141.052	43.00728	148.3234	43.21968	135.5673
43.02133	138.429	43.04435	131.803	43.1074	134.0232	43.04818	141.0525	43.02395	148.3215	43.23635	135.5675
43.038	138.4264	43.06102	131.8026	43.12407	134.0242	43.06485	141.0518	43.04062	148.3229	43.25302	135.5655
43.05467	138.424	43.07768	131.7983	43.14073	134.0245	43.08152	141.0492	43.05728	148.3239	43.26968	135.565
43.07133	138.4249	43.09435	131.7999	43.1574	134.0215	43.09818	141.049	43.07395	148.3218	43.28635	135.5639
43.088	138.4219	43.11102	131.7987	43.17407	134.0232	43.11485	141.0476	43.09062	148.3196	43.30302	135.563
43.10467	138.4192	43.12768	131.7995	43.19073	134.0208	43.13152	141.0483	43.10728	148.319	43.31968	135.5608
43.12133	138.4172	43.14435	131.795	43.2074	134.0182	43.14818	141.047	43.12395	148.3212	43.33635	135.5607
43.138	138.4136	43.16102	131.7943	43.22407	134.0154	43.16485	141.046	43.14062	148.3207	43.35302	135.559
43.15467	138.4139	43.17768	131.7924	43.24073	134.018	43.18152	141.0432	43.15728	148.321	43.36968	135.5592
43.17133	138.4104	43.19435	131.7891	43.2574	134.0163	43.19818	141.0422	43.17395	148.3212	43.38635	135.5565
43.188	138.405	43.21102	131.787	43.27407	134.0132	43.21485	141.0431	43.19062	148.3207	43.40302	135.5542
43.20467	138.4061	43.22768	131.786	43.29073	134.0147	43.23152	141.0401	43.20728	148.3215	43.41968	135.5538
43.22133	138.403	43.24435	131.7862	43.3074	134.0131	43.24818	141.04	43.22395	148.3209	43.43635	135.553
43.238	138.4054	43.26102	131.7835	43.32407	134.0097	43.26485	141.0395	43.24062	148.3215	43.45302	135.5515
43.25467	138.3982	43.27768	131.7819	43.34073	134.0106	43.28152	141.041	43.25728	148.3214	43.46968	135.5498
43.27133	138.397	43.29435	131.7787	43.3574	134.0126	43.29818	141.0406	43.27395	148.3215	43.48635	135.5471
43.288	138.3976	43.31102	131.7788	43.37407	134.0075	43.31485	141.0372	43.29062	148.3189	43.50302	135.5466
43.30467	138.3952	43.32768	131.7765	43.39073	134.0066	43.33152	141.0395	43.30728	148.3215	43.51968	135.5461
43.32133	138.3963	43.34435	131.7747	43.4074	134.0068	43.34818	141.039	43.32395	148.3215	43.53635	135.5448
43.338	138.3906	43.36102	131.7752	43.42407	134.0037	43.36485	141.0396	43.34062	148.3216	43.55302	135.5444
43.35467	138.3898	43.37768	131.7747	43.44073	134.0028	43.38152	141.0375	43.35728	148.3201	43.56968	135.5446
43.37133	138.3882	43.39435	131.7735	43.4574	134.0025	43.39818	141.0367	43.37395	148.32	43.58635	135.5405
43.388	138.3886	43.41102	131.7716	43.47407	134.0018	43.41485	141.0358	43.39062	148.3207	43.60302	135.5417
43.40467	138.387	43.42768	131.7716	43.49073	133.9996	43.43152	141.035	43.40728	148.3197	43.61968	135.54
43.42133	138.3854	43.44435	131.7689	43.5074	133.9987	43.44818	141.0325	43.42395	148.3173	43.63635	135.539
43.438	138.3848	43.46102	131.7655	43.52407	133.9951	43.46485	141.0341	43.44062	148.3185	43.65302	135.5414
43.45467	138.3814	43.47768	131.765	43.54073	133.9909	43.48152	141.032	43.45728	148.3171	43.66968	135.5366
43.47133	138.3809	43.49435	131.7638	43.5574	133.9943	43.49818	141.0327	43.47395	148.3164	43.68635	135.5385
43.488	138.3766	43.51102	131.7604	43.57407	133.9951	43.51485	141.0306	43.49062	148.3172	43.70302	135.5369
43.50467	138.3759	43.52768	131.7637	43.59073	133.9925	43.53152	141.0312	43.50728	148.3177	43.71968	135.5372
43.52133	138.3744	43.54435	131.7602	43.6074	133.9928	43.54818	141.0315	43.52395	148.3186	43.73635	135.535
43.538	138.3728	43.56102	131.7559	43.62407	133.987	43.56485	141.0297	43.54062	148.3153	43.75302	135.5346
43.55467	138.3715	43.57768	131.7576	43.64073	133.9925	43.58152	141.0291	43.55728	148.323	43.76968	135.5335
43.57133	138.3705	43.59435	131.7525	43.6574	133.9886	43.59818	141.0295	43.57395	148.3161	43.78635	135.5326
43.588	138.3686	43.61102	131.7541	43.67407	133.9853	43.61485	141.0306	43.59062	148.3153	43.80302	135.5336
43.60467	138.3644	43.62768	131.7522	43.69073	133.9862	43.63152	141.0278	43.60728	148.3182	43.81968	135.5303
43.62133	138.3668	43.64435	131.7536	43.7074	133.9856	43.64818	141.0248	43.62395	148.3182	43.83635	135.5328
43.638	138.3634	43.66102	131.7483	43.72407	133.9816	43.66485	141.0291	43.64062	148.3126	43.85302	135.5301
43.65467	138.3577	43.67768	131.7498	43.74073	133.9804	43.68152	141.0307	43.65728	148.3158	43.86968	135.5289
43.67133	138.363	43.69435	131.744	43.7574	133.9776	43.69818	141.0295	43.67395	148.3105	43.88635	135.5278

43.688	138.3571	43.71102	131.7432	43.77407	133.9804	43.71485	141.0265	43.69062	148.3211	43.90302	135.527
43.70467	138.3574	43.72768	131.7451	43.79073	133.9774	43.73152	141.0263	43.70728	148.3148	43.91968	135.528
43.72133	138.3563	43.74435	131.7402	43.8074	133.9741	43.74818	141.0246	43.72395	148.3169	43.93635	135.5251
43.738	138.3502	43.76102	131.7409	43.82407	133.9778	43.76485	141.0242	43.74062	148.317	43.95302	135.5261
43.75467	138.3535	43.77768	131.7361	43.84073	133.974	43.78152	141.0249	43.75728	148.317	43.96968	135.5233
43.77133	138.3516	43.79435	131.7346	43.8574	133.9734	43.79818	141.0235	43.77395	148.3173	43.98635	135.5223
43.788	138.3473	43.81102	131.7344	43.87407	133.9714	43.81485	141.0212	43.79062	148.3158	44.00302	135.5227
43.80467	138.3489	43.82768	131.7326	43.89073	133.9714	43.83152	141.0234	43.80728	148.3133	44.01968	135.5224
43.82133	138.346	43.84435	131.7319	43.9074	133.9714	43.84818	141.0206	43.82395	148.3136	44.03635	135.5215
43.838	138.3419	43.86102	131.7299	43.92407	133.9707	43.86485	141.0205	43.84062	148.3143	44.05302	135.5204
43.85467	138.3407	43.87768	131.728	43.94073	133.971	43.88152	141.022	43.85728	148.3119	44.06968	135.5203
43.87133	138.34	43.89435	131.7237	43.9574	133.9698	43.89818	141.0207	43.87395	148.3146	44.08635	135.5198
43.888	138.339	43.91102	131.7257	43.97407	133.9666	43.91485	141.0196	43.89062	148.3119	44.10302	135.5169
43.90467	138.3332	43.92768	131.7222	43.99073	133.9659	43.93152	141.0186	43.90728	148.3131	44.11968	135.5147
43.92133	138.3352	43.94435	131.7214	44.0074	133.9645	43.94818	141.0186	43.92395	148.3138	44.13635	135.5147
43.938	138.3297	43.96102	131.7184	44.02407	133.9641	43.96485	141.0174	43.94062	148.3119	44.15302	135.5145
43.95467	138.3292	43.97768	131.7144	44.04073	133.966	43.98152	141.0164	43.95728	148.3128	44.16968	135.5128
43.97133	138.3289	43.99435	131.7151	44.0574	133.9629	43.99818	141.0187	43.97395	148.3114	44.18635	135.514
43.988	138.3279	44.01102	131.7126	44.07407	133.9615	44.01485	141.0164	43.99062	148.3097	44.20302	135.5118
44.00467	138.3267	44.02768	131.7145	44.09073	133.9643	44.03152	141.014	44.00728	148.3146	44.21968	135.5099
44.02133	138.3236	44.04435	131.7118	44.1074	133.9593	44.04818	141.014	44.02395	148.3109	44.23635	135.5093
44.038	138.3251	44.06102	131.7106	44.12407	133.9566	44.06485	141.0133	44.04062	148.3115	44.25302	135.5086
44.05467	138.3231	44.07768	131.7091	44.14073	133.959	44.08152	141.0134	44.05728	148.3107	44.26968	135.5077
44.07133	138.3204	44.09435	131.7091	44.1574	133.9579	44.09818	141.0122	44.07395	148.313	44.28635	135.5065
44.088	138.3178	44.11102	131.7072	44.17407	133.9556	44.11485	141.0124	44.09062	148.3066	44.30302	135.5035
44.10467	138.3166	44.12768	131.7042	44.19073	133.9561	44.13152	141.0102	44.10728	148.3106	44.31968	135.5034
44.12133	138.3156	44.14435	131.7049	44.2074	133.9548	44.14818	141.007	44.12395	148.3093	44.33635	135.5062
44.138	138.3103	44.16102	131.7002	44.22407	133.9492	44.16485	141.0093	44.14062	148.3075	44.35302	135.504
44.15467	138.311	44.17768	131.7007	44.24073	133.952	44.18152	141.0098	44.15728	148.3098	44.36968	135.5008
44.17133	138.3099	44.19435	131.7014	44.2574	133.9513	44.19818	141.0071	44.17395	148.3097	44.38635	135.5009
44.188	138.3065	44.21102	131.6986	44.27407	133.948	44.21485	141.0061	44.19062	148.3063	44.40302	135.4998
44.20467	138.3055	44.22768	131.6984	44.29073	133.9484	44.23152	141.0055	44.20728	148.3083	44.41968	135.4976
44.22133	138.3039	44.24435	131.6966	44.3074	133.949	44.24818	141.0062	44.22395	148.3066	44.43635	135.4981
44.238	138.3024	44.26102	131.6952	44.32407	133.9472	44.26485	141.0046	44.24062	148.3066	44.45302	135.4953
44.25467	138.3	44.27768	131.6922	44.34073	133.9453	44.28152	141.0057	44.25728	148.3059	44.46968	135.496
44.27133	138.2964	44.29435	131.6901	44.3574	133.9448	44.29818	141.0012	44.27395	148.3078	44.48635	135.4968
44.288	138.2934	44.31102	131.6887	44.37407	133.9453	44.31485	141.0039	44.29062	148.3051	44.50302	135.4968
44.30467	138.2951	44.32768	131.6862	44.39073	133.9463	44.33152	141.0028	44.30728	148.3081	44.51968	135.4948
44.32133	138.2911	44.34435	131.6861	44.4074	133.9405	44.34818	141.0002	44.32395	148.3059	44.53635	135.4943
44.338	138.2892	44.36102	131.6847	44.42407	133.9432	44.36485	140.998	44.34062	148.3057	44.55302	135.4933
44.35467	138.2887	44.37768	131.6825	44.44073	133.9406	44.38152	140.998	44.35728	148.3051	44.56968	135.4921
44.37133	138.2879	44.39435	131.6793	44.4574	133.9396	44.39818	140.9964	44.37395	148.3052	44.58635	135.4899
44.388	138.2855	44.41102	131.6795	44.47407	133.9392	44.41485	140.9958	44.39062	148.3055	44.60302	135.4921
44.40467	138.2834	44.42768	131.6752	44.49073	133.9362	44.43152	140.9943	44.40728	148.3033	44.61968	135.4907
44.42133	138.2797	44.44435	131.6729	44.5074	133.9369	44.44818	140.9944	44.42395	148.3038	44.63635	135.49
44.438	138.281	44.46102	131.6706	44.52407	133.935	44.46485	140.9928	44.44062	148.3047	44.65302	135.4864
44.45467	138.2784	44.47768	131.6707	44.54073	133.9321	44.48152	140.9947	44.45728	148.3023	44.66968	135.4867
44.47133	138.2799	44.49435	131.669	44.5574	133.9326	44.49818	140.9946	44.47395	148.3049	44.68635	135.4857
44.488	138.2758	44.51102	131.6664	44.57407	133.9306	44.51485	140.993	44.49062	148.2999	44.70302	135.4862
44.50467	138.2738	44.52768	131.6653	44.59073	133.9307	44.53152	140.9889	44.50728	148.3009	44.71968	135.4842
44.52133	138.2717	44.54435	131.6613	44.6074	133.9289	44.54818	140.9901	44.52395	148.301	44.73635	135.4806
44.538	138.271	44.56102	131.6638	44.62407	133.9283	44.56485	140.9883	44.54062	148.3029	44.75302	135.481
44.55467	138.2681	44.57768	131.6593	44.64073	133.9272	44.58152	140.9874	44.55728	148.3027	44.76968	135.4806
44.57133	138.2661	44.59435	131.6573	44.6574	133.9258	44.59818	140.9867	44.57395	148.3021	44.78635	135.4827
44.588	138.2635	44.61102	131.6551	44.67407	133.9246	44.61485	140.9879	44.59062	148.2976	44.80302	135.479
44.60467	138.262	44.62768	131.6568	44.69073	133.9217	44.63152	140.9857	44.60728	148.3017	44.81968	135.4772
44.62133	138.2613	44.64435	131.6537	44.7074	133.9219	44.64818	140.9844	44.62395	148.3024	44.83635	135.4763
44.638	138.2634	44.66102	131.6505	44.72407	133.9224	44.66485	140.9827	44.64062	148.298	44.85302	135.4759
44.65467	138.2559	44.67768	131.6488	44.74073	133.92	44.68152	140.9831	44.65728	148.3017	44.86968	135.473
44.67133	138.259	44.69435	131.6476	44.7574	133.9203	44.69818	140.9826	44.67395	148.2966	44.88635	135.4744
44.688	138.2533	44.71102	131.644	44.77407	133.9202	44.71485	140.9823	44.69062	148.3009	44.90302	135.4714
44.70467	138.254	44.72768	131.6452	44.79073	133.92	44.73152	140.9813	44.70728	148.303	44.91968	135.4711
44.72133	138.2533	44.74435	131.6464	44.8074	133.9182	44.74818	140.9783	44.72395	148.3017	44.93635	135.4679
44.738	138.2552	44.76102	131.6426	44.82407	133.9206	44.76485	140.9793	44.74062	148.3014	44.95302	135.4688
44.75467	138.2503	44.77768	131.6398	44.84073	133.9198	44.78152	140.9787	44.75728	148.2997	44.96968	135.468
44.77133	138.2472	44.79435	131.6399	44.8574	133.918	44.79818	140.98	44.77395	148.2995	44.98635	135.468
44.788	138.2478	44.81102	131.6378	44.87407	133.92	44.81485	140.9808	44.79062	148.2993	45.00302	135.4659
44.80467	138.2431	44.82768	131.636	44.89073	133.9194	44.83152	140.9791	44.80728	148.2974	45.01968	135.4662
44.82133	138.2474	44.84435	131.6343	44.9074	133.9197	44.84818	140.9783	44.82395	148.3019	45.03635	135.4649

44.838	138.2403	44.86102	131.6318	44.92407	133.9211	44.86485	140.9778	44.84062	148.3007	45.05302	135.4625
44.85467	138.2368	44.87768	131.6309	44.94073	133.9193	44.88152	140.9755	44.85728	148.2986	45.06968	135.4612
44.87133	138.238	44.89435	131.6321	44.9574	133.9199	44.89818	140.9758	44.87395	148.3022	45.08635	135.4632
44.888	138.2371	44.91102	131.6288	44.97407	133.9183	44.91485	140.9768	44.89062	148.3006	45.10302	135.4586
44.90467	138.2351	44.92768	131.6277	44.99073	133.9203	44.93152	140.9764	44.90728	148.2986	45.11968	135.4579
44.92133	138.2351	44.94435	131.6262	45.0074	133.9165	44.94818	140.9751	44.92395	148.2995	45.13635	135.4574
44.938	138.2321	44.96102	131.6272	45.02407	133.9163	44.96485	140.9747	44.94062	148.3	45.15302	135.4591
44.95467	138.2296	44.97768	131.624	45.04073	133.9177	44.98152	140.974	44.95728	148.2986	45.16968	135.4546
44.97133	138.2287	44.99435	131.6242	45.0574	133.9176	44.99818	140.9692	44.97395	148.297	45.18635	135.4549
44.988	138.2293	45.01102	131.622	45.07407	133.9156	45.01485	140.9732	44.99062	148.2988	45.20302	135.4536
45.00467	138.2241	45.02768	131.6177	45.09073	133.9147	45.03152	140.9713	45.00728	148.2968	45.21968	135.4557
45.02133	138.2246	45.04435	131.6192	45.1074	133.9157	45.04818	140.97	45.02395	148.2973	45.23635	135.4534
45.038	138.2198	45.06102	131.6163	45.12407	133.9153	45.06485	140.9682	45.04062	148.2969	45.25302	135.4498
45.05467	138.2178	45.07768	131.6137	45.14073	133.9107	45.08152	140.9659	45.05728	148.2946	45.26968	135.4495
45.07133	138.216	45.09435	131.6121	45.1574	133.9108	45.09818	140.9637	45.07395	148.2939	45.28635	135.451
45.088	138.2133	45.11102	131.6107	45.17407	133.9096	45.11485	140.9643	45.09062	148.2961	45.30302	135.4481
45.10467	138.2111	45.12768	131.6076	45.19073	133.9104	45.13152	140.9637	45.10728	148.2948	45.31968	135.4449
45.12133	138.209	45.14435	131.606	45.2074	133.91	45.14818	140.962	45.12395	148.2938	45.33635	135.4479
45.138	138.208	45.16102	131.6083	45.22407	133.9112	45.16485	140.9581	45.14062	148.2975	45.35302	135.4481
45.15467	138.2071	45.17768	131.6052	45.24073	133.9067	45.18152	140.96	45.15728	148.2938	45.36968	135.4426
45.17133	138.2067	45.19435	131.6038	45.2574	133.9052	45.19818	140.9577	45.17395	148.2947	45.38635	135.4462
45.188	138.2031	45.21102	131.6006	45.27407	133.903	45.21485	140.9566	45.19062	148.2926	45.40302	135.442
45.20467	138.1981	45.22768	131.5982	45.29073	133.9034	45.23152	140.9571	45.20728	148.2929	45.41968	135.4421
45.22133	138.1977	45.24435	131.5988	45.3074	133.903	45.24818	140.9552	45.22395	148.2935	45.43635	135.4406
45.238	138.2004	45.26102	131.5952	45.32407	133.9013	45.26485	140.9524	45.24062	148.295	45.45302	135.4393
45.25467	138.197	45.27768	131.5923	45.34073	133.9006	45.28152	140.9527	45.25728	148.2944	45.46968	135.4395
45.27133	138.1948	45.29435	131.5907	45.3574	133.9009	45.29818	140.95	45.27395	148.2928	45.48635	135.438
45.288	138.1946	45.31102	131.5914	45.37407	133.9001	45.31485	140.9499	45.29062	148.2917	45.50302	135.4385
45.30467	138.1921	45.32768	131.5915	45.39073	133.8982	45.33152	140.9509	45.30728	148.2912	45.51968	135.4372
45.32133	138.1917	45.34435	131.5897	45.4074	133.8965	45.34818	140.95	45.32395	148.2931	45.53635	135.4358
45.338	138.1905	45.36102	131.5875	45.42407	133.8947	45.36485	140.9485	45.34062	148.2929	45.55302	135.4329
45.35467	138.1909	45.37768	131.5868	45.44073	133.8979	45.38152	140.9471	45.35728	148.2907	45.56968	135.4311
45.37133	138.1934	45.39435	131.5832	45.4574	133.8959	45.39818	140.9462	45.37395	148.2926	45.58635	135.4335
45.388	138.184	45.41102	131.5816	45.47407	133.8935	45.41485	140.9473	45.39062	148.2925	45.60302	135.429
45.40467	138.1867	45.42768	131.5809	45.49073	133.8949	45.43152	140.9465	45.40728	148.2883	45.61968	135.4306
45.42133	138.1812	45.44435	131.5782	45.5074	133.8922	45.44818	140.9436	45.42395	148.2894	45.63635	135.4278
45.438	138.1827	45.46102	131.5755	45.52407	133.8896	45.46485	140.9427	45.44062	148.2908	45.65302	135.4281
45.45467	138.1739	45.47768	131.5776	45.54073	133.892	45.48152	140.9434	45.45728	148.293	45.66968	135.4243
45.47133	138.1764	45.49435	131.5778	45.5574	133.8925	45.49818	140.9412	45.47395	148.2911	45.68635	135.426
45.488	138.1754	45.51102	131.5748	45.57407	133.8901	45.51485	140.9423	45.49062	148.2906	45.70302	135.4233
45.50467	138.175	45.52768	131.5725	45.59073	133.8883	45.53152	140.941	45.50728	148.29	45.71968	135.4234
45.52133	138.1708	45.54435	131.568	45.6074	133.8868	45.54818	140.939	45.52395	148.2887	45.73635	135.422
45.538	138.1673	45.56102	131.5668	45.62407	133.8846	45.56485	140.9413	45.54062	148.2893	45.75302	135.4206
45.55467	138.169	45.57768	131.5676	45.64073	133.8818	45.58152	140.9415	45.55728	148.2902	45.76968	135.4189
45.57133	138.1651	45.59435	131.5658	45.6574	133.8829	45.59818	140.9387	45.57395	148.2876	45.78635	135.4179
45.588	138.1632	45.61102	131.5632	45.67407	133.8803	45.61485	140.9385	45.59062	148.2884	45.80302	135.4183
45.60467	138.1634	45.62768	131.5651	45.69073	133.8827	45.63152	140.9385	45.60728	148.2884	45.81968	135.4154
45.62133	138.1608	45.64435	131.5637	45.7074	133.8788	45.64818	140.9377	45.62395	148.2873	45.83635	135.4152
45.638	138.1603	45.66102	131.5588	45.72407	133.8789	45.66485	140.9365	45.64062	148.2891	45.85302	135.4142
45.65467	138.1552	45.67768	131.5579	45.74073	133.8773	45.68152	140.9376	45.65728	148.287	45.86968	135.4134
45.67133	138.1566	45.69435	131.5601	45.7574	133.8771	45.69818	140.935	45.67395	148.2869	45.88635	135.4136
45.688	138.1555	45.71102	131.5556	45.77407	133.8744	45.71485	140.9347	45.69062	148.2887	45.90302	135.4111
45.70467	138.1532	45.72768	131.5603	45.79073	133.8748	45.73152	140.9344	45.70728	148.2863	45.91968	135.4097
45.72133	138.1515	45.74435	131.5499	45.8074	133.8762	45.74818	140.9337	45.72395	148.2867	45.93635	135.407
45.738	138.1513	45.76102	131.5527	45.82407	133.8728	45.76485	140.9343	45.74062	148.287	45.95302	135.4088
45.75467	138.1499	45.77768	131.5507	45.84073	133.8696	45.78152	140.9338	45.75728	148.2853	45.96968	135.4058
45.77133	138.1456	45.79435	131.5532	45.8574	133.8725	45.79818	140.9321	45.77395	148.2835	45.98635	135.4056
45.788	138.1429	45.81102	131.5524	45.87407	133.8692	45.81485	140.9329	45.79062	148.2825	46.00302	135.4051
45.80467	138.145	45.82768	131.5489	45.89073	133.8682	45.83152	140.9318	45.80728	148.2842	46.01968	135.4042
45.82133	138.1376	45.84435	131.5417	45.9074	133.8652	45.84818	140.9307	45.82395	148.2821	46.03635	135.4014
45.838	138.1405	45.86102	131.5446	45.92407	133.8677	45.86485	140.9302	45.84062	148.2832	46.05302	135.4022
45.85467	138.1392	45.87768	131.5391	45.94073	133.8643	45.88152	140.9331	45.85728	148.2827	46.06968	135.4036
45.87133	138.1366	45.89435	131.5395	45.9574	133.8635	45.89818	140.9302	45.87395	148.2834	46.08635	135.4013
45.888	138.1353	45.91102	131.5383	45.97407	133.8619	45.91485	140.9254	45.89062	148.2815	46.10302	135.3986
45.90467	138.1351	45.92768	131.5384	45.99073	133.8627	45.93152	140.9289	45.90728	148.2849	46.11968	135.3982
45.92133	138.1341	45.94435	131.5393	46.0074	133.859	45.94818	140.9277	45.92395	148.2844	46.13635	135.3981
45.938	138.1307	45.96102	131.5305	46.02407	133.8592	45.96485	140.9261	45.94062	148.283	46.15302	135.396
45.95467	138.1274	45.97768	131.5318	46.04073	133.8588	45.98152	140.9284	45.95728	148.281	46.16968	135.3957
45.97133	138.1259	45.99435	131.5346	46.0574	133.8587	45.99818	140.9236	45.97395	148.2799	46.18635	135.3939

45.988	138.1231	46.01102	131.5292	46.07407	133.856	46.01485	140.9263	45.99062	148.2801	46.20302	135.3927
46.00467	138.1243	46.02768	131.5299	46.09073	133.8534	46.03152	140.9248	46.00728	148.2802	46.21968	135.3924
46.02133	138.1242	46.04435	131.5263	46.1074	133.8535	46.04818	140.9248	46.02395	148.2808	46.23635	135.3902
46.038	138.1203	46.06102	131.5235	46.12407	133.8562	46.06485	140.9245	46.04062	148.2806	46.25302	135.3906
46.05467	138.1177	46.07768	131.5242	46.14073	133.8518	46.08152	140.9212	46.05728	148.2793	46.26968	135.3886
46.07133	138.1148	46.09435	131.5198	46.1574	133.8521	46.09818	140.9227	46.07395	148.2814	46.28635	135.3865
46.088	138.1174	46.11102	131.5197	46.17407	133.8513	46.11485	140.9218	46.09062	148.2801	46.30302	135.387
46.10467	138.1126	46.12768	131.5196	46.19073	133.8513	46.13152	140.9211	46.10728	148.2818	46.31968	135.3854
46.12133	138.1091	46.14435	131.5185	46.2074	133.8489	46.14818	140.9176	46.12395	148.2799	46.33635	135.3834
46.138	138.1104	46.16102	131.5195	46.22407	133.8509	46.16485	140.9164	46.14062	148.2799	46.35302	135.3845
46.15467	138.1065	46.17768	131.5154	46.24073	133.8508	46.18152	140.9146	46.15728	148.2797	46.36968	135.3827
46.17133	138.1053	46.19435	131.5135	46.2574	133.8482	46.19818	140.9152	46.17395	148.2809	46.38635	135.382
46.188	138.1045	46.21102	131.5121	46.27407	133.8459	46.21485	140.9165	46.19062	148.2781	46.40302	135.3782
46.20467	138.1062	46.22768	131.5136	46.29073	133.8464	46.23152	140.9132	46.20728	148.2792	46.41968	135.3815
46.22133	138.0984	46.24435	131.508	46.3074	133.8424	46.24818	140.9152	46.22395	148.278	46.43635	135.3764
46.238	138.1035	46.26102	131.5058	46.32407	133.8465	46.26485	140.9123	46.24062	148.2796	46.45302	135.3772
46.25467	138.0995	46.27768	131.505	46.34073	133.8436	46.28152	140.9125	46.25728	148.2783	46.46968	135.3759
46.27133	138.0985	46.29435	131.5032	46.3574	133.8417	46.29818	140.9114	46.27395	148.276	46.48635	135.3772
46.288	138.098	46.31102	131.5019	46.37407	133.8419	46.31485	140.9132	46.29062	148.2754	46.50302	135.3763
46.30467	138.0976	46.32768	131.5018	46.39073	133.8416	46.33152	140.9087	46.30728	148.2761	46.51968	135.3743
46.32133	138.0921	46.34435	131.4992	46.4074	133.8386	46.34818	140.9091	46.32395	148.2771	46.53635	135.375
46.338	138.0942	46.36102	131.497	46.42407	133.8395	46.36485	140.9098	46.34062	148.2755	46.55302	135.3742
46.35467	138.0905	46.37768	131.4951	46.44073	133.8359	46.38152	140.906	46.35728	148.2746	46.56968	135.3729
46.37133	138.0882	46.39435	131.4968	46.4574	133.8381	46.39818	140.9054	46.37395	148.2753	46.58635	135.3713
46.388	138.0886	46.41102	131.4894	46.47407	133.8365	46.41485	140.9065	46.39062	148.2743	46.60302	135.3699
46.40467	138.0869	46.42768	131.4923	46.49073	133.8361	46.43152	140.903	46.40728	148.271	46.61968	135.3708
46.42133	138.0857	46.44435	131.4915	46.5074	133.8345	46.44818	140.9035	46.42395	148.2734	46.63635	135.3679
46.438	138.0816	46.46102	131.4891	46.52407	133.8313	46.46485	140.9031	46.44062	148.2721	46.65302	135.3662
46.45467	138.0825	46.47768	131.4836	46.54073	133.8308	46.48152	140.9028	46.45728	148.2745	46.66968	135.3657
46.47133	138.0808	46.49435	131.4843	46.5574	133.8287	46.49818	140.8993	46.47395	148.274	46.68635	135.3672
46.488	138.078	46.51102	131.4819	46.57407	133.8308	46.51485	140.9014	46.49062	148.2711	46.70302	135.3649
46.50467	138.0741	46.52768	131.484	46.59073	133.8302	46.53152	140.8986	46.50728	148.2733	46.71968	135.3642
46.52133	138.0748	46.54435	131.48	46.6074	133.8281	46.54818	140.899	46.52395	148.2731	46.73635	135.3652
46.538	138.0748	46.56102	131.4772	46.62407	133.8236	46.56485	140.8991	46.54062	148.2695	46.75302	135.3641
46.55467	138.0728	46.57768	131.4768	46.64073	133.8256	46.58152	140.8981	46.55728	148.274	46.76968	135.3616
46.57133	138.072	46.59435	131.476	46.6574	133.8237	46.59818	140.8965	46.57395	148.2751	46.78635	135.3608
46.588	138.0687	46.61102	131.4759	46.67407	133.8232	46.61485	140.8944	46.59062	148.2743	46.80302	135.3578
46.60467	138.0681	46.62768	131.4741	46.69073	133.8233	46.63152	140.8952	46.60728	148.2732	46.81968	135.3592
46.62133	138.0642	46.64435	131.4764	46.7074	133.8214	46.64818	140.8949	46.62395	148.2744	46.83635	135.3558
46.638	138.0636	46.66102	131.4691	46.72407	133.8214	46.66485	140.8937	46.64062	148.2739	46.85302	135.3551
46.65467	138.0601	46.67768	131.4691	46.74073	133.8174	46.68152	140.8934	46.65728	148.2745	46.86968	135.3579
46.67133	138.06	46.69435	131.4691	46.7574	133.8187	46.69818	140.8926	46.67395	148.2758	46.88635	135.3566
46.688	138.0594	46.71102	131.4679	46.77407	133.8205	46.71485	140.8912	46.69062	148.2756	46.90302	135.3546
46.70467	138.0573	46.72768	131.4651	46.79073	133.82	46.73152	140.8905	46.70728	148.2686	46.91968	135.3541
46.72133	138.0556	46.74435	131.4633	46.8074	133.8199	46.74818	140.8905	46.72395	148.2708	46.93635	135.3544
46.738	138.0545	46.76102	131.4648	46.82407	133.8158	46.76485	140.8897	46.74062	148.2726	46.95302	135.353
46.75467	138.0516	46.77768	131.4584	46.84073	133.8149	46.78152	140.8901	46.75728	148.2719	46.96968	135.3525
46.77133	138.0516	46.79435	131.4594	46.8574	133.8143	46.79818	140.8868	46.77395	148.2704	46.98635	135.3528
46.788	138.049	46.81102	131.4595	46.87407	133.8191	46.81485	140.8883	46.79062	148.2724	47.00302	135.3492
46.80467	138.0492	46.82768	131.4558	46.89073	133.8177	46.83152	140.8869	46.80728	148.2729	47.01968	135.347
46.82133	138.0472	46.84435	131.4565	46.9074	133.8166	46.84818	140.8846	46.82395	148.2692	47.03635	135.348
46.838	138.0461	46.86102	131.4521	46.92407	133.8166	46.86485	140.8858	46.84062	148.2713	47.05302	135.3476
46.85467	138.0435	46.87768	131.448	46.94073	133.8133	46.88152	140.8864	46.85728	148.269	47.06968	135.3446
46.87133	138.0433	46.89435	131.4506	46.9574	133.8133	46.89818	140.8811	46.87395	148.2684	47.08635	135.3452
46.888	138.0405	46.91102	131.4478	46.97407	133.8118	46.91485	140.8809	46.89062	148.2695	47.10302	135.3418
46.90467	138.0384	46.92768	131.4496	46.99073	133.8118	46.93152	140.8822	46.90728	148.268	47.11968	135.3436
46.92133	138.0357	46.94435	131.4455	47.0074	133.8102	46.94818	140.8792	46.92395	148.2663	47.13635	135.3406
46.938	138.0351	46.96102	131.4456	47.02407	133.8106	46.96485	140.881	46.94062	148.2705	47.15302	135.3401
46.95467	138.0352	46.97768	131.4447	47.04073	133.8078	46.98152	140.8795	46.95728	148.2683	47.16968	135.34
46.97133	138.032	46.99435	131.4409	47.0574	133.8105	46.99818	140.8783	46.97395	148.2691	47.18635	135.3388
46.988	138.032	47.01102	131.4406	47.07407	133.809	47.01485	140.8782	46.99062	148.2677	47.20302	135.3367
47.00467	138.0283	47.02768	131.4401	47.09073	133.8084	47.03152	140.8785	47.00728	148.2695	47.21968	135.3365
47.02133	138.0286	47.04435	131.4385	47.1074	133.8063	47.04818	140.8755	47.02395	148.2696	47.23635	135.3328
47.038	138.0265	47.06102	131.4369	47.12407	133.8071	47.06485	140.8751	47.04062	148.2639	47.25302	135.3365
47.05467	138.0242	47.07768	131.4338	47.14073	133.8034	47.08152	140.8756	47.05728	148.2718	47.26968	135.3307
47.07133	138.0227	47.09435	131.431	47.1574	133.8088	47.09818	140.8718	47.07395	148.2696	47.28635	135.3319
47.088	138.019	47.11102	131.4324	47.17407	133.8026	47.11485	140.871	47.09062	148.2694	47.30302	135.3315
47.10467	138.0192	47.12768	131.4276	47.19073	133.8035	47.13152	140.8688	47.10728	148.2675	47.31968	135.3299
47.12133	138.0165	47.14435	131.4281	47.2074	133.8026	47.14818	140.8707	47.12395	148.268	47.33635	135.328

47.138	138.0161	47.16102	131.4273	47.22407	133.8026	47.16485	140.8706	47.14062	148.2703	47.35302	135.3312
47.15467	138.0149	47.17768	131.4221	47.24073	133.7974	47.18152	140.8695	47.15728	148.269	47.36968	135.3269
47.17133	138.0124	47.19435	131.4234	47.2574	133.7984	47.19818	140.8694	47.17395	148.2672	47.38635	135.3219
47.188	138.0092	47.21102	131.4236	47.27407	133.7995	47.21485	140.8655	47.19062	148.2666	47.40302	135.3244
47.20467	138.0118	47.22768	131.4206	47.29073	133.7977	47.23152	140.8639	47.20728	148.2675	47.41968	135.3234
47.22133	138.007	47.24435	131.4175	47.3074	133.792	47.24818	140.8647	47.22395	148.2655	47.43635	135.3212
47.238	138.0069	47.26102	131.4159	47.32407	133.7936	47.26485	140.8655	47.24062	148.2698	47.45302	135.3186
47.25467	138.0056	47.27768	131.417	47.34073	133.791	47.28152	140.8639	47.25728	148.2655	47.46968	135.3188
47.27133	138.0033	47.29435	131.4137	47.3574	133.7919	47.29818	140.8638	47.27395	148.2653	47.48635	135.3146
47.288	137.9996	47.31102	131.4118	47.37407	133.7904	47.31485	140.8638	47.29062	148.2672	47.50302	135.3136
47.30467	137.9999	47.32768	131.4058	47.39073	133.7913	47.33152	140.8636	47.30728	148.267	47.51968	135.3146
47.32133	137.9975	47.34435	131.4071	47.4074	133.7892	47.34818	140.8619	47.32395	148.2676	47.53635	135.3138
47.338	137.9967	47.36102	131.4092	47.42407	133.7883	47.36485	140.8605	47.34062	148.2646	47.55302	135.3116
47.35467	137.9936	47.37768	131.4063	47.44073	133.7862	47.38152	140.8621	47.35728	148.2656	47.56968	135.3102
47.37133	137.9938	47.39435	131.4061	47.4574	133.787	47.39818	140.863	47.37395	148.2655	47.58635	135.3093
47.388	137.9921	47.41102	131.3988	47.47407	133.7867	47.41485	140.8642	47.39062	148.2645	47.60302	135.3064
47.40467	137.9886	47.42768	131.3995	47.49073	133.7845	47.43152	140.861	47.40728	148.2661	47.61968	135.3083
47.42133	137.9859	47.44435	131.4022	47.5074	133.7855	47.44818	140.8604	47.42395	148.2636	47.63635	135.3038
47.438	137.9872	47.46102	131.3997	47.52407	133.7813	47.46485	140.8597	47.44062	148.2655	47.65302	135.3057
47.45467	137.9831	47.47768	131.3942	47.54073	133.7799	47.48152	140.8595	47.45728	148.2639	47.66968	135.3041
47.47133	137.9826	47.49435	131.3912	47.5574	133.781	47.49818	140.8612	47.47395	148.2632	47.68635	135.305
47.488	137.9813	47.51102	131.3935	47.57407	133.7796	47.51485	140.8602	47.49062	148.2638	47.70302	135.3015
47.50467	137.9776	47.52768	131.3912	47.59073	133.7791	47.53152	140.8568	47.50728	148.2656	47.71968	135.3011
47.52133	137.9771	47.54435	131.3919	47.6074	133.7788	47.54818	140.8573	47.52395	148.2619	47.73635	135.3006
47.538	137.9779	47.56102	131.3898	47.62407	133.7765	47.56485	140.8565	47.54062	148.264	47.75302	135.3007
47.55467	137.9756	47.57768	131.3825	47.64073	133.774	47.58152	140.8533	47.55728	148.2658	47.76968	135.2996
47.57133	137.9696	47.59435	131.3827	47.6574	133.7727	47.59818	140.8515	47.57395	148.2625	47.78635	135.2983
47.588	137.9689	47.61102	131.3793	47.67407	133.772	47.61485	140.8557	47.59062	148.2638	47.80302	135.2958
47.60467	137.9714	47.62768	131.3816	47.69073	133.7716	47.63152	140.8533	47.60728	148.2636	47.81968	135.2962
47.62133	137.9662	47.64435	131.3731	47.7074	133.7716	47.64818	140.85	47.62395	148.2633	47.83635	135.2938
47.638	137.9673	47.66102	131.3755	47.72407	133.7706	47.66485	140.8489	47.64062	148.2609	47.85302	135.2944
47.65467	137.9667	47.67768	131.3738	47.74073	133.7704	47.68152	140.8474	47.65728	148.2631	47.86968	135.2913
47.67133	137.9631	47.69435	131.3737	47.7574	133.767	47.69818	140.8454	47.67395	148.2617	47.88635	135.2936
47.688	137.9658	47.71102	131.3728	47.77407	133.7658	47.71485	140.8463	47.69062	148.2629	47.90302	135.2896
47.70467	137.9596	47.72768	131.3715	47.79073	133.7672	47.73152	140.8471	47.70728	148.2631	47.91968	135.2912
47.72133	137.9584	47.74435	131.3666	47.8074	133.7673	47.74818	140.8463	47.72395	148.2613	47.93635	135.2901
47.738	137.9554	47.76102	131.3672	47.82407	133.7653	47.76485	140.8451	47.74062	148.2613	47.95302	135.2894
47.75467	137.9565	47.77768	131.3641	47.84073	133.7638	47.78152	140.8469	47.75728	148.2608	47.96968	135.287
47.77133	137.9563	47.79435	131.361	47.8574	133.7639	47.79818	140.8469	47.77395	148.2614	47.98635	135.2877
47.788	137.9525	47.81102	131.3613	47.87407	133.7625	47.81485	140.8456	47.79062	148.2622	48.00302	135.2857
47.80467	137.9536	47.82768	131.3609	47.89073	133.7628	47.83152	140.8447	47.80728	148.2624	48.01968	135.2859
47.82133	137.9485	47.84435	131.3623	47.9074	133.7628	47.84818	140.8454	47.82395	148.2611	48.03635	135.2839
47.838	137.944	47.86102	131.3594	47.92407	133.7643	47.86485	140.8431	47.84062	148.2622	48.05302	135.2852
47.85467	137.9455	47.87768	131.3553	47.94073	133.7596	47.88152	140.8419	47.85728	148.2617	48.06968	135.2806
47.87133	137.943	47.89435	131.3548	47.9574	133.7598	47.89818	140.8414	47.87395	148.2627	48.08635	135.2811
47.888	137.9444	47.91102	131.3559	47.97407	133.7591	47.91485	140.8439	47.89062	148.2625	48.10302	135.2806
47.90467	137.9431	47.92768	131.3535	47.99073	133.7599	47.93152	140.8394	47.90728	148.2636	48.11968	135.2805
47.92133	137.9362	47.94435	131.35	48.0074	133.7571	47.94818	140.84	47.92395	148.2643	48.13635	135.2807
47.938	137.9377	47.96102	131.3512	48.02407	133.756	47.96485	140.8393	47.94062	148.2597	48.15302	135.278
47.95467	137.9376	47.97768	131.3511	48.04073	133.7558	47.98152	140.8399	47.95728	148.2612	48.16968	135.2781
47.97133	137.9346	47.99435	131.3469	48.0574	133.7543	47.99818	140.8376	47.97395	148.2622	48.18635	135.2773
47.988	137.9304	48.01102	131.3458	48.07407	133.7554	48.01485	140.8383	47.99062	148.2628	48.20302	135.2768
48.00467	137.9293	48.02768	131.3431	48.09073	133.7543	48.03152	140.8389	48.00728	148.2622	48.21968	135.2765
48.02133	137.9273	48.04435	131.3397	48.1074	133.7529	48.04818	140.8365	48.02395	148.2611	48.23635	135.2736
48.038	137.9278	48.06102	131.3443	48.12407	133.751	48.06485	140.8327	48.04062	148.2626	48.25302	135.2755
48.05467	137.9251	48.07768	131.3427	48.14073	133.7494	48.08152	140.8341	48.05728	148.2588	48.26968	135.2749
48.07133	137.925	48.09435	131.3387	48.1574	133.7486	48.09818	140.8343	48.07395	148.2524	48.28635	135.2736
48.088	137.9215	48.11102	131.337	48.17407	133.7507	48.11485	140.8331	48.09062	148.2604	48.30302	135.2712
48.10467	137.9207	48.12768	131.3366	48.19073	133.7487	48.13152	140.8305	48.10728	148.2593	48.31968	135.2729
48.12133	137.9172	48.14435	131.3339	48.2074	133.7478	48.14818	140.8318	48.12395	148.2603	48.33635	135.2719
48.138	137.918	48.16102	131.3338	48.22407	133.745	48.16485	140.8323	48.14062	148.2582	48.35302	135.2699
48.15467	137.9157	48.17768	131.3316	48.24073	133.7469	48.18152	140.83	48.15728	148.2607	48.36968	135.2695
48.17133	137.9143	48.19435	131.3293	48.2574	133.745	48.19818	140.8282	48.17395	148.2569	48.38635	135.2702
48.188	137.9135	48.21102	131.3251	48.27407	133.7436	48.21485	140.8274	48.19062	148.2554	48.40302	135.2668
48.20467	137.9117	48.22768	131.3246	48.29073	133.7454	48.23152	140.8253	48.20728	148.2566	48.41968	135.2661
48.22133	137.91	48.24435	131.3245	48.3074	133.7435	48.24818	140.8276	48.22395	148.2575	48.43635	135.2646
48.238	137.9107	48.26102	131.3227	48.32407	133.7416	48.26485	140.8269	48.24062	148.2548	48.45302	135.2668
48.25467	137.9085	48.27768	131.3194	48.34073	133.741	48.28152	140.8257	48.25728	148.2584	48.46968	135.266
48.27133	137.9038	48.29435	131.3188	48.3574	133.7421	48.29818	140.8267	48.27395	148.2562	48.48635	135.2648

48.288	137.906	48.31102	131.3166	48.37407	133.7383	48.31485	140.8254	48.29062	148.2551	48.50302	135.2608
48.30467	137.9044	48.32768	131.3143	48.39073	133.7361	48.33152	140.8234	48.30728	148.2531	48.51968	135.2599
48.32133	137.9007	48.34435	131.3132	48.4074	133.7377	48.34818	140.8224	48.32395	148.2552	48.53635	135.2622
48.338	137.8997	48.36102	131.3145	48.42407	133.7378	48.36485	140.8212	48.34062	148.2563	48.55302	135.2574
48.35467	137.8978	48.37768	131.3097	48.44073	133.7348	48.38152	140.8238	48.35728	148.2574	48.56968	135.2567
48.37133	137.8971	48.39435	131.307	48.4574	133.7354	48.39818	140.8234	48.37395	148.2573	48.58635	135.257
48.388	137.8943	48.41102	131.3084	48.47407	133.7333	48.41485	140.8214	48.39062	148.2546	48.60302	135.2532
48.40467	137.8933	48.42768	131.3076	48.49073	133.7317	48.43152	140.8233	48.40728	148.2544	48.61968	135.2549
48.42133	137.8897	48.44435	131.3088	48.5074	133.7293	48.44818	140.8207	48.42395	148.2543	48.63635	135.2509
48.438	137.8884	48.46102	131.3025	48.52407	133.7293	48.46485	140.8188	48.44062	148.256	48.65302	135.253
48.45467	137.8902	48.47768	131.3024	48.54073	133.7288	48.48152	140.8207	48.45728	148.2553	48.66968	135.253
48.47133	137.8886	48.49435	131.3031	48.5574	133.7305	48.49818	140.8183	48.47395	148.2527	48.68635	135.252
48.488	137.8842	48.51102	131.2995	48.57407	133.7302	48.51485	140.8214	48.49062	148.2562	48.70302	135.2486
48.50467	137.8816	48.52768	131.2974	48.59073	133.7279	48.53152	140.8199	48.50728	148.2541	48.71968	135.2486
48.52133	137.88	48.54435	131.2967	48.6074	133.7258	48.54818	140.8175	48.52395	148.2544	48.73635	135.2486
48.538	137.8791	48.56102	131.297	48.62407	133.7257	48.56485	140.8161	48.54062	148.2532	48.75302	135.2455
48.55467	137.8808	48.57768	131.2963	48.64073	133.7275	48.58152	140.8172	48.55728	148.2541	48.76968	135.2447
48.57133	137.881	48.59435	131.297	48.6574	133.7253	48.59818	140.8165	48.57395	148.257	48.78635	135.2429
48.588	137.8775	48.61102	131.2918	48.67407	133.7255	48.61485	140.8171	48.59062	148.2531	48.80302	135.2447
48.60467	137.8746	48.62768	131.2931	48.69073	133.7239	48.63152	140.8175	48.60728	148.2523	48.81968	135.2441
48.62133	137.8725	48.64435	131.2897	48.7074	133.7228	48.64818	140.8171	48.62395	148.2535	48.83635	135.2422
48.638	137.874	48.66102	131.2871	48.72407	133.7249	48.66485	140.8137	48.64062	148.2537	48.85302	135.2429
48.65467	137.8697	48.67768	131.2849	48.74073	133.7246	48.68152	140.8145	48.65728	148.252	48.86968	135.2427
48.67133	137.8678	48.69435	131.2852	48.7574	133.7216	48.69818	140.8135	48.67395	148.2533	48.88635	135.2433
48.688	137.8665	48.71102	131.2836	48.77407	133.7212	48.71485	140.8142	48.69062	148.2525	48.90302	135.2391
48.70467	137.8676	48.72768	131.2825	48.79073	133.7191	48.73152	140.8143	48.70728	148.2531	48.91968	135.2393
48.72133	137.8651	48.74435	131.2772	48.8074	133.7194	48.74818	140.8141	48.72395	148.2535	48.93635	135.239
48.738	137.8626	48.76102	131.279	48.82407	133.7191	48.76485	140.8116	48.74062	148.255	48.95302	135.2381
48.75467	137.8606	48.77768	131.2752	48.84073	133.7156	48.78152	140.8135	48.75728	148.2517	48.96968	135.2363
48.77133	137.8629	48.79435	131.2748	48.8574	133.7184	48.79818	140.8119	48.77395	148.2533	48.98635	135.2377
48.788	137.8608	48.81102	131.2717	48.87407	133.7169	48.81485	140.8136	48.79062	148.2543	49.00302	135.2345
48.80467	137.8591	48.82768	131.2752	48.89073	133.7147	48.83152	140.8121	48.80728	148.2501	49.01968	135.2352
48.82133	137.8613	48.84435	131.2716	48.9074	133.7156	48.84818	140.8138	48.82395	148.2538	49.03635	135.2321
48.838	137.8566	48.86102	131.2724	48.92407	133.7149	48.86485	140.8102	48.84062	148.2503	49.05302	135.2346
48.85467	137.8525	48.87768	131.2695	48.94073	133.7154	48.88152	140.8103	48.85728	148.2507	49.06968	135.2328
48.87133	137.8522	48.89435	131.2702	48.9574	133.7125	48.89818	140.8109	48.87395	148.2509	49.08635	135.2338
48.888	137.8496	48.91102	131.2651	48.97407	133.712	48.91485	140.8095	48.89062	148.2516	49.10302	135.2311
48.90467	137.8498	48.92768	131.2642	48.99073	133.7135	48.93152	140.8073	48.90728	148.2541	49.11968	135.2293
48.92133	137.8491	48.94435	131.2639	49.0074	133.7128	48.94818	140.8055	48.92395	148.2524	49.13635	135.2289
48.938	137.8492	48.96102	131.2633	49.02407	133.7102	48.96485	140.8049	48.94062	148.2508	49.15302	135.2292
48.95467	137.845	48.97768	131.2582	49.04073	133.7086	48.98152	140.802	48.95728	148.2517	49.16968	135.23
48.97133	137.8456	48.99435	131.2607	49.0574	133.708	48.99818	140.8045	48.97395	148.2517	49.18635	135.2304
48.988	137.8421	49.01102	131.2555	49.07407	133.7052	49.01485	140.8004	48.99062	148.2524	49.20302	135.228
49.00467	137.8385	49.02768	131.2552	49.09073	133.7084	49.03152	140.7996	49.00728	148.2498	49.21968	135.2288
49.02133	137.8384	49.04435	131.2581	49.1074	133.7054	49.04818	140.7997	49.02395	148.2521	49.23635	135.2255
49.038	137.8388	49.06102	131.2521	49.12407	133.698	49.06485	140.7995	49.04062	148.254	49.25302	135.2266
49.05467	137.8379	49.07768	131.2494	49.14073	133.7068	49.08152	140.7971	49.05728	148.2505	49.26968	135.2215
49.07133	137.8343	49.09435	131.2517	49.1574	133.7048	49.09818	140.8003	49.07395	148.249	49.28635	135.2227
49.088	137.8348	49.11102	131.2501	49.17407	133.7048	49.11485	140.7974	49.09062	148.2528	49.30302	135.2215
49.10467	137.8297	49.12768	131.2495	49.19073	133.6992	49.13152	140.7939	49.10728	148.2507	49.31968	135.2197
49.12133	137.8318	49.14435	131.2425	49.2074	133.703	49.14818	140.7951	49.12395	148.2484	49.33635	135.2182
49.138	137.8306	49.16102	131.2454	49.22407	133.7021	49.16485	140.7941	49.14062	148.25	49.35302	135.219
49.15467	137.829	49.17768	131.2421	49.24073	133.6995	49.18152	140.794	49.15728	148.2522	49.36968	135.2166
49.17133	137.828	49.19435	131.2413	49.2574	133.6987	49.19818	140.7941	49.17395	148.2504	49.38635	135.2148
49.188	137.8224	49.21102	131.2399	49.27407	133.6997	49.21485	140.7945	49.19062	148.2487	49.40302	135.2161
49.20467	137.8224	49.22768	131.2369	49.29073	133.6997	49.23152	140.7913	49.20728	148.2478	49.41968	135.2159
49.22133	137.8203	49.24435	131.2379	49.3074	133.6997	49.24818	140.79	49.22395	148.2478	49.43635	135.2146
49.238	137.8181	49.26102	131.2365	49.32407	133.6956	49.26485	140.7925	49.24062	148.2465	49.45302	135.2148
49.25467	137.818	49.27768	131.2357	49.34073	133.6978	49.28152	140.7876	49.25728	148.2498	49.46968	135.2136
49.27133	137.8174	49.29435	131.2337	49.3574	133.6978	49.29818	140.7854	49.27395	148.2496	49.48635	135.2123
49.288	137.817	49.31102	131.2329	49.37407	133.6953	49.31485	140.7849	49.29062	148.2484	49.50302	135.2112
49.30467	137.8132	49.32768	131.231	49.39073	133.6937	49.33152	140.7825	49.30728	148.2486	49.51968	135.2131
49.32133	137.8133	49.34435	131.2283	49.4074	133.6931	49.34818	140.7843	49.32395	148.2477	49.53635	135.2075
49.338	137.8113	49.36102	131.228	49.42407	133.6925	49.36485	140.7817	49.34062	148.2467	49.55302	135.2081
49.35467	137.8088	49.37768	131.2252	49.44073	133.6912	49.38152	140.7803	49.35728	148.2469	49.56968	135.2057
49.37133	137.8072	49.39435	131.2257	49.4574	133.6927	49.39818	140.7796	49.37395	148.2471	49.58635	135.2088
49.388	137.8056	49.41102	131.2176	49.47407	133.6915	49.41485	140.7792	49.39062	148.2476	49.60302	135.203
49.40467	137.8025	49.42768	131.2228	49.49073	133.6892	49.43152	140.7787	49.40728	148.2479	49.61968	135.2039
49.42133	137.8016	49.44435	131.221	49.5074	133.6914	49.44818	140.7759	49.42395	148.2462	49.63635	135.2028

49.438	137.8001	49.46102	131.2152	49.52407	133.6909	49.46485	140.776	49.44062	148.2443	49.65302	135.2038
49.45467	137.7968	49.47768	131.2167	49.54073	133.6897	49.48152	140.7769	49.45728	148.2457	49.66968	135.2006
49.47133	137.7974	49.49435	131.2187	49.5574	133.6901	49.49818	140.7777	49.47395	148.2445	49.68635	135.1972
49.488	137.7928	49.51102	131.2117	49.57407	133.6903	49.51485	140.7767	49.49062	148.2451	49.70302	135.196
49.50467	137.7912	49.52768	131.2038	49.59073	133.6896	49.53152	140.7747	49.50728	148.2425	49.71968	135.1975
49.52133	137.7909	49.54435	131.2198	49.6074	133.6877	49.54818	140.7729	49.52395	148.2431	49.73635	135.1958
49.538	137.7885	49.56102	131.1997	49.62407	133.6864	49.56485	140.7748	49.54062	148.2434	49.75302	135.1922
49.55467	137.7891	49.57768	131.2055	49.64073	133.6887	49.58152	140.776	49.55728	148.2435	49.76968	135.1928
49.57133	137.7858	49.59435	131.2037	49.6574	133.6852	49.59818	140.7736	49.57395	148.2426	49.78635	135.1974
49.588	137.7854	49.61102	131.2033	49.67407	133.6841	49.61485	140.7732	49.59062	148.2398	49.80302	135.1921
49.60467	137.7827	49.62768	131.1999	49.69073	133.6847	49.63152	140.7723	49.60728	148.2423	49.81968	135.1896
49.62133	137.7809	49.64435	131.1964	49.7074	133.6849	49.64818	140.7711	49.62395	148.2438	49.83635	135.1886
49.638	137.7832	49.66102	131.1969	49.72407	133.6821	49.66485	140.7732	49.64062	148.2412	49.85302	135.193
49.65467	137.7785	49.67768	131.1949	49.74073	133.6823	49.68152	140.7734	49.65728	148.2412	49.86968	135.1889
49.67133	137.7764	49.69435	131.194	49.7574	133.6822	49.69818	140.7703	49.67395	148.2432	49.88635	135.1863
49.688	137.7753	49.71102	131.1902	49.77407	133.6822	49.71485	140.77	49.69062	148.2453	49.90302	135.186
49.70467	137.7743	49.72768	131.2019	49.79073	133.6829	49.73152	140.7695	49.70728	148.2442	49.91968	135.1878
49.72133	137.7722	49.74435	131.1913	49.8074	133.6797	49.74818	140.7698	49.72395	148.2428	49.93635	135.1856
49.738	137.7732	49.76102	131.191	49.82407	133.6805	49.76485	140.7692	49.74062	148.2427	49.95302	135.1856
49.75467	137.7686	49.77768	131.1864	49.84073	133.6794	49.78152	140.7698	49.75728	148.2425	49.96968	135.1817
49.77133	137.7673	49.79435	131.1876	49.8574	133.6768	49.79818	140.7688	49.77395	148.2445	49.98635	135.1847
49.788	137.7674	49.81102	131.1869	49.87407	133.6774	49.81485	140.7686	49.79062	148.2439	50.00302	135.1833
49.80467	137.7636	49.82768	131.1885	49.89073	133.6787	49.83152	140.767	49.80728	148.2409	50.01968	135.181
49.82133	137.7618	49.84435	131.1818	49.9074	133.6789	49.84818	140.7662	49.82395	148.2442	50.03635	135.1795
49.838	137.7617	49.86102	131.1789	49.92407	133.6771	49.86485	140.7658	49.84062	148.2419	50.05302	135.1809
49.85467	137.7623	49.87768	131.1834	49.94073	133.6762	49.88152	140.766	49.85728	148.2414	50.06968	135.1787
49.87133	137.7576	49.89435	131.1784	49.9574	133.679	49.89818	140.7646	49.87395	148.2395	50.08635	135.1784
49.888	137.7584	49.91102	131.1774	49.97407	133.6746	49.91485	140.7642	49.89062	148.2371	50.10302	135.1787
49.90467	137.7579	49.92768	131.1762	49.99073	133.6717	49.93152	140.7646	49.90728	148.2406	50.11968	135.1767
49.92133	137.7539	49.94435	131.1715	50.0074	133.6762	49.94818	140.762	49.92395	148.2398	50.13635	135.1754
49.938	137.7507	49.96102	131.1724	50.02407	133.675	49.96485	140.7633	49.94062	148.2389	50.15302	135.1753
49.95467	137.7494	49.97768	131.1706	50.04073	133.6747	49.98152	140.7618	49.95728	148.2399	50.16968	135.1712
49.97133	137.7479	49.99435	131.1705	50.0574	133.6703	49.99818	140.7623	49.97395	148.2384	50.18635	135.1707
49.988	137.7441	50.01102	131.167	50.07407	133.6736	50.01485	140.7606	49.99062	148.2372	50.20302	135.1712
50.00467	137.7464	50.02768	131.1655	50.09073	133.6716	50.03152	140.7602	50.00728	148.2373	50.21968	135.1697
50.02133	137.7444	50.04435	131.1673	50.1074	133.6682	50.04818	140.7611	50.02395	148.2373	50.23635	135.1684
50.038	137.7424	50.06102	131.1656	50.12407	133.6695	50.06485	140.7564	50.04062	148.2377	50.25302	135.1702
50.05467	137.7435	50.07768	131.1651	50.14073	133.667	50.08152	140.7573	50.05728	148.2384	50.26968	135.1683
50.07133	137.7444	50.09435	131.162	50.1574	133.6665	50.09818	140.7574	50.07395	148.2395	50.28635	135.1661
50.088	137.7388	50.11102	131.1625	50.17407	133.666	50.11485	140.7547	50.09062	148.2371	50.30302	135.1662
50.10467	137.7348	50.12768	131.1602	50.19073	133.6652	50.13152	140.7548	50.10728	148.2411	50.31968	135.1651
50.12133	137.7348	50.14435	131.1602	50.2074	133.6646	50.14818	140.7545	50.12395	148.2381	50.33635	135.1633
50.138	137.7352	50.16102	131.1582	50.22407	133.6662	50.16485	140.751	50.14062	148.237	50.35302	135.161
50.15467	137.7351	50.17768	131.1576	50.24073	133.6625	50.18152	140.7534	50.15728	148.2356	50.36968	135.1643
50.17133	137.7332	50.19435	131.1566	50.2574	133.6598	50.19818	140.7513	50.17395	148.238	50.38635	135.1605
50.188	137.7306	50.21102	131.1534	50.27407	133.6595	50.21485	140.7501	50.19062	148.2383	50.40302	135.1597
50.20467	137.7279	50.22768	131.1519	50.29073	133.6616	50.23152	140.7486	50.20728	148.2373	50.41968	135.1585
50.22133	137.7263	50.24435	131.1499	50.3074	133.655	50.24818	140.751	50.22395	148.2407	50.43635	135.1575
50.238	137.7252	50.26102	131.1488	50.32407	133.6546	50.26485	140.7485	50.24062	148.236	50.45302	135.156
50.25467	137.7256	50.27768	131.1485	50.34073	133.6551	50.28152	140.7488	50.25728	148.2373	50.46968	135.1584
50.27133	137.7221	50.29435	131.1461	50.3574	133.6556	50.29818	140.7489	50.27395	148.2375	50.48635	135.1554
50.288	137.7217	50.31102	131.1435	50.37407	133.6529	50.31485	140.7483	50.29062	148.2375	50.50302	135.1535
50.30467	137.7193	50.32768	131.1415	50.39073	133.6552	50.33152	140.7486	50.30728	148.2351	50.51968	135.1549
50.32133	137.7183	50.34435	131.1421	50.4074	133.65	50.34818	140.7482	50.32395	148.2352	50.53635	135.1515
50.338	137.7169	50.36102	131.1402	50.42407	133.651	50.36485	140.7468	50.34062	148.2341	50.55302	135.1513
50.35467	137.7129	50.37768	131.1386	50.44073	133.6531	50.38152	140.7461	50.35728	148.2344	50.56968	135.15
50.37133	137.7154	50.39435	131.1363	50.4574	133.6532	50.39818	140.7467	50.37395	148.237	50.58635	135.1504
50.388	137.7145	50.41102	131.1356	50.47407	133.6527	50.41485	140.7442	50.39062	148.2329	50.60302	135.1462
50.40467	137.7104	50.42768	131.1365	50.49073	133.6517	50.43152	140.7448	50.40728	148.2347	50.61968	135.1484
50.42133	137.7104	50.44435	131.1333	50.5074	133.6515	50.44818	140.7422	50.42395	148.235	50.63635	135.147
50.438	137.7057	50.46102	131.1304	50.52407	133.6489	50.46485	140.7433	50.44062	148.2355	50.65302	135.1452
50.45467	137.7033	50.47768	131.1285	50.54073	133.6502	50.48152	140.7417	50.45728	148.2358	50.66968	135.1435
50.47133	137.7034	50.49435	131.1269	50.5574	133.6471	50.49818	140.744	50.47395	148.2333	50.68635	135.1424
50.488	137.7019	50.51102	131.1269	50.57407	133.6474	50.51485	140.7418	50.49062	148.2358	50.70302	135.1423
50.50467	137.701	50.52768	131.1246	50.59073	133.6471	50.53152	140.7387	50.50728	148.235	50.71968	135.1441
50.52133	137.6961	50.54435	131.1221	50.6074	133.6455	50.54818	140.7411	50.52395	148.2349	50.73635	135.1401
50.538	137.6951	50.56102	131.1205	50.62407	133.6459	50.56485	140.7401	50.54062	148.232	50.75302	135.1387
50.55467	137.6962	50.57768	131.1203	50.64073	133.6432	50.58152	140.7382	50.55728	148.2339	50.76968	135.1402
50.57133	137.6918	50.59435	131.1192	50.6574	133.6447	50.59818	140.7402	50.57395	148.231	50.78635	135.141

50.588	137.6948	50.61102	131.1207	50.67407	133.6415	50.61485	140.7351	50.59062	148.2354	50.80302	135.1387
50.60467	137.6893	50.62768	131.1149	50.69073	133.6443	50.63152	140.734	50.60728	148.2334	50.81968	135.1376
50.62133	137.6898	50.64435	131.1147	50.7074	133.6423	50.64818	140.7382	50.62395	148.2359	50.83635	135.1358
50.638	137.6888	50.66102	131.1127	50.72407	133.6417	50.66485	140.7335	50.64062	148.2328	50.85302	135.1348
50.65467	137.6868	50.67768	131.1112	50.74073	133.6435	50.68152	140.7365	50.65728	148.2313	50.86968	135.1325
50.67133	137.6835	50.69435	131.1089	50.7574	133.6408	50.69818	140.7357	50.67395	148.2279	50.88635	135.1345
50.688	137.6829	50.71102	131.1074	50.77407	133.6378	50.71485	140.7317	50.69062	148.2312	50.90302	135.1341
50.70467	137.6826	50.72768	131.1028	50.79073	133.6421	50.73152	140.7332	50.70728	148.2338	50.91968	135.1315
50.72133	137.6808	50.74435	131.1065	50.8074	133.641	50.74818	140.7311	50.72395	148.229	50.93635	135.1285
50.738	137.6751	50.76102	131.1045	50.82407	133.637	50.76485	140.7295	50.74062	148.2271	50.95302	135.1302
50.75467	137.6762	50.77768	131.101	50.84073	133.6379	50.78152	140.7284	50.75728	148.2339	50.96968	135.1291
50.77133	137.6729	50.79435	131.1017	50.8574	133.6366	50.79818	140.727	50.77395	148.2293	50.98635	135.129
50.788	137.6724	50.81102	131.0979	50.87407	133.6367	50.81485	140.7267	50.79062	148.2294	51.00302	135.1278
50.80467	137.6688	50.82768	131.0997	50.89073	133.635	50.83152	140.728	50.80728	148.2314	51.01968	135.1292
50.82133	137.6686	50.84435	131.0937	50.9074	133.6351	50.84818	140.7257	50.82395	148.2315	51.03635	135.1259
50.838	137.6679	50.86102	131.0935	50.92407	133.636	50.86485	140.7219	50.84062	148.231	51.05302	135.1265
50.85467	137.6652	50.87768	131.0955	50.94073	133.6331	50.88152	140.723	50.85728	148.2293	51.06968	135.1229
50.87133	137.6647	50.89435	131.0921	50.9574	133.6321	50.89818	140.7204	50.87395	148.2296	51.08635	135.1274
50.888	137.6599	50.91102	131.0863	50.97407	133.6305	50.91485	140.7222	50.89062	148.2287	51.10302	135.1245
50.90467	137.6627	50.92768	131.0885	50.99073	133.632	50.93152	140.7206	50.90728	148.2279	51.11968	135.1226
50.92133	137.6563	50.94435	131.0901	51.0074	133.6287	50.94818	140.7207	50.92395	148.2319	51.13635	135.1201
50.938	137.6592	50.96102	131.0879	51.02407	133.6287	50.96485	140.7198	50.94062	148.234	51.15302	135.1219
50.95467	137.6597	50.97768	131.0835	51.04073	133.626	50.98152	140.7201	50.95728	148.2331	51.16968	135.1202
50.97133	137.6576	50.99435	131.0822	51.0574	133.6284	50.99818	140.7209	50.97395	148.2292	51.18635	135.1205
50.988	137.655	51.01102	131.0777	51.07407	133.6295	51.01485	140.719	50.99062	148.2305	51.20302	135.1197
51.00467	137.6522	51.02768	131.0805	51.09073	133.6266	51.03152	140.7207	51.00728	148.2306	51.21968	135.1191
51.02133	137.6508	51.04435	131.0777	51.1074	133.629	51.04818	140.7159	51.02395	148.2286	51.23635	135.1183
51.038	137.65	51.06102	131.0779	51.12407	133.6255	51.06485	140.7174	51.04062	148.2313	51.25302	135.1172
51.05467	137.6488	51.07768	131.0763	51.14073	133.6243	51.08152	140.7184	51.05728	148.229	51.26968	135.1164
51.07133	137.6491	51.09435	131.074	51.1574	133.6244	51.09818	140.7175	51.07395	148.2278	51.28635	135.116
51.088	137.6454	51.11102	131.074	51.17407	133.6227	51.11485	140.7151	51.09062	148.2276	51.30302	135.1152
51.10467	137.6433	51.12768	131.0676	51.19073	133.6207	51.13152	140.7135	51.10728	148.229	51.31968	135.1151
51.12133	137.6432	51.14435	131.0711	51.2074	133.6205	51.14818	140.714	51.12395	148.2278	51.33635	135.1135
51.138	137.6409	51.16102	131.0654	51.22407	133.6193	51.16485	140.7125	51.14062	148.2279	51.35302	135.113
51.15467	137.6416	51.17768	131.0649	51.24073	133.6196	51.18152	140.7135	51.15728	148.2266	51.36968	135.1114
51.17133	137.6378	51.19435	131.0645	51.2574	133.6179	51.19818	140.713	51.17395	148.2266	51.38635	135.1098
51.188	137.6391	51.21102	131.063	51.27407	133.6224	51.21485	140.7093	51.19062	148.2284	51.40302	135.1118
51.20467	137.6355	51.22768	131.062	51.29073	133.6207	51.23152	140.7115	51.20728	148.2282	51.41968	135.1116
51.22133	137.6349	51.24435	131.0587	51.3074	133.6163	51.24818	140.7099	51.22395	148.2272	51.43635	135.1089
51.238	137.6332	51.26102	131.0573	51.32407	133.6168	51.26485	140.7106	51.24062	148.2265	51.45302	135.1079
51.25467	137.6308	51.27768	131.054	51.34073	133.6157	51.28152	140.7106	51.25728	148.2285	51.46968	135.1065
51.27133	137.6296	51.29435	131.0565	51.3574	133.6133	51.29818	140.7101	51.27395	148.2273	51.48635	135.1059
51.288	137.6274	51.31102	131.0558	51.37407	133.6158	51.31485	140.7097	51.29062	148.2284	51.50302	135.1038
51.30467	137.6257	51.32768	131.0504	51.39073	133.6144	51.33152	140.7078	51.30728	148.2296	51.51968	135.1051
51.32133	137.6246	51.34435	131.0512	51.4074	133.6093	51.34818	140.7083	51.32395	148.2259	51.53635	135.1045
51.338	137.6202	51.36102	131.0479	51.42407	133.6151	51.36485	140.7075	51.34062	148.2277	51.55302	135.1007
51.35467	137.6203	51.37768	131.0506	51.44073	133.6139	51.38152	140.7069	51.35728	148.2267	51.56968	135.1024
51.37133	137.621	51.39435	131.0459	51.4574	133.6108	51.39818	140.707	51.37395	148.2285	51.58635	135.1001
51.388	137.6159	51.41102	131.0454	51.47407	133.6105	51.41485	140.7055	51.39062	148.2277	51.60302	135.1019
51.40467	137.6176	51.42768	131.0415	51.49073	133.6084	51.43152	140.7036	51.40728	148.2278	51.61968	135.1003
51.42133	137.614	51.44435	131.0396	51.5074	133.6093	51.44818	140.705	51.42395	148.2269	51.63635	135.0984
51.438	137.6154	51.46102	131.0388	51.52407	133.6064	51.46485	140.7019	51.44062	148.2257	51.65302	135.099
51.45467	137.6123	51.47768	131.0365	51.54073	133.6066	51.48152	140.702	51.45728	148.2253	51.66968	135.0986
51.47133	137.609	51.49435	131.0373	51.5574	133.603	51.49818	140.7009	51.47395	148.2235	51.68635	135.0963
51.488	137.604	51.51102	131.0357	51.57407	133.6057	51.51485	140.6978	51.49062	148.2287	51.70302	135.0943
51.50467	137.6049	51.52768	131.0283	51.59073	133.605	51.53152	140.6968	51.50728	148.2234	51.71968	135.0946
51.52133	137.6078	51.54435	131.0327	51.6074	133.604	51.54818	140.6973	51.52395	148.2304	51.73635	135.0939
51.538	137.6035	51.56102	131.0311	51.62407	133.6002	51.56485	140.6947	51.54062	148.2244	51.75302	135.0934
51.55467	137.6014	51.57768	131.0253	51.64073	133.5993	51.58152	140.6956	51.55728	148.227	51.76968	135.0941
51.57133	137.6006	51.59435	131.0247	51.6574	133.5999	51.59818	140.6952	51.57395	148.2283	51.78635	135.0916
51.588	137.5955	51.61102	131.0243	51.67407	133.6008	51.61485	140.6919	51.59062	148.2263	51.80302	135.0906
51.60467	137.5973	51.62768	131.0213	51.69073	133.5977	51.63152	140.6915	51.60728	148.2284	51.81968	135.0888
51.62133	137.5941	51.64435	131.0183	51.7074	133.5973	51.64818	140.691	51.62395	148.2249	51.83635	135.089
51.638	137.5932	51.66102	131.02	51.72407	133.5953	51.66485	140.691	51.64062	148.2313	51.85302	135.0879
51.65467	137.5933	51.67768	131.0208	51.74073	133.5959	51.68152	140.6899	51.65728	148.224	51.86968	135.0858
51.67133	137.5888	51.69435	131.0168	51.7574	133.5975	51.69818	140.6875	51.67395	148.2275	51.88635	135.0875
51.688	137.589	51.71102	131.0163	51.77407	133.595	51.71485	140.687	51.69062	148.2275	51.90302	135.0877
51.70467	137.5852	51.72768	131.0202	51.79073	133.595	51.73152	140.6891	51.70728	148.2247	51.91968	135.0832
51.72133	137.5856	51.74435	131.0138	51.8074	133.594	51.74818	140.6884	51.72395	148.2255	51.93635	135.0851

51.738	137.5835	51.76102	131.0088	51.82407	133.5949	51.76485	140.6864	51.74062	148.2279	51.95302	135.0841
51.75467	137.5825	51.77768	131.0129	51.84073	133.594	51.78152	140.6883	51.75728	148.2275	51.96968	135.0832
51.77133	137.5788	51.79435	131.0111	51.8574	133.5904	51.79818	140.6831	51.77395	148.2285	51.98635	135.0818
51.788	137.576	51.81102	131.0103	51.87407	133.5897	51.81485	140.6809	51.79062	148.2262	52.00302	135.0805
51.80467	137.578	51.82768	131.0084	51.89073	133.5925	51.83152	140.6846	51.80728	148.2273	52.01968	135.0798
51.82133	137.5758	51.84435	131.0062	51.9074	133.5923	51.84818	140.6822	51.82395	148.225	52.03635	135.0775
51.838	137.5746	51.86102	131.0066	51.92407	133.593	51.86485	140.6822	51.84062	148.2254	52.05302	135.0772
51.85467	137.5725	51.87768	131.0026	51.94073	133.5871	51.88152	140.6807	51.85728	148.2287	52.06968	135.0731
51.87133	137.5736	51.89435	131.0006	51.9574	133.5922	51.89818	140.6798	51.87395	148.2272	52.08635	135.0736
51.888	137.5688	51.91102	130.9975	51.97407	133.5908	51.91485	140.6789	51.89062	148.2297	52.10302	135.0708
51.90467	137.5683	51.92768	130.9985	51.99073	133.5883	51.93152	140.6773	51.90728	148.2286	52.11968	135.0678
51.92133	137.5682	51.94435	130.9972	52.0074	133.5868	51.94818	140.6761	51.92395	148.2255	52.13635	135.068
51.938	137.5665	51.96102	130.9979	52.02407	133.5897	51.96485	140.6756	51.94062	148.2247	52.15302	135.067
51.95467	137.5639	51.97768	130.993	52.04073	133.5883	51.98152	140.6757	51.95728	148.2264	52.16968	135.0656
51.97133	137.5621	51.99435	130.9929	52.0574	133.5914	51.99818	140.6753	51.97395	148.2262	52.18635	135.066
51.988	137.5618	52.01102	130.9884	52.07407	133.5899	52.01485	140.6726	51.99062	148.2261	52.20302	135.0646
52.00467	137.5602	52.02768	130.9882	52.09073	133.5886	52.03152	140.6741	52.00728	148.2296	52.21968	135.0608
52.02133	137.5579	52.04435	130.9862	52.1074	133.586	52.04818	140.6722	52.02395	148.2215	52.23635	135.0615
52.038	137.5583	52.06102	130.986	52.12407	133.5894	52.06485	140.6746	52.04062	148.223	52.25302	135.0615
52.05467	137.5558	52.07768	130.985	52.14073	133.5843	52.08152	140.6719	52.05728	148.224	52.26968	135.0578
52.07133	137.5529	52.09435	130.9823	52.1574	133.5884	52.09818	140.6701	52.07395	148.2241	52.28635	135.0585
52.088	137.5527	52.11102	130.9803	52.17407	133.5908	52.11485	140.6675	52.09062	148.2261	52.30302	135.0592
52.10467	137.5515	52.12768	130.9778	52.19073	133.5856	52.13152	140.6683	52.10728	148.224	52.31968	135.0553
52.12133	137.5505	52.14435	130.9772	52.2074	133.5836	52.14818	140.6671	52.12395	148.2241	52.33635	135.0543
52.138	137.5505	52.16102	130.976	52.22407	133.5828	52.16485	140.668	52.14062	148.2259	52.35302	135.0534
52.15467	137.5482	52.17768	130.9745	52.24073	133.5871	52.18152	140.6681	52.15728	148.2223	52.36968	135.0537
52.17133	137.5487	52.19435	130.9752	52.2574	133.5813	52.19818	140.6688	52.17395	148.2222	52.38635	135.0507
52.188	137.5461	52.21102	130.971	52.27407	133.5851	52.21485	140.6676	52.19062	148.224	52.40302	135.0485
52.20467	137.5443	52.22768	130.9703	52.29073	133.5834	52.23152	140.6662	52.20728	148.2252	52.41968	135.0491
52.22133	137.5437	52.24435	130.9729	52.3074	133.5821	52.24818	140.666	52.22395	148.2239	52.43635	135.0472
52.238	137.5396	52.26102	130.9669	52.32407	133.5763	52.26485	140.6659	52.24062	148.223	52.45302	135.0443
52.25467	137.5416	52.27768	130.9671	52.34073	133.5786	52.28152	140.6637	52.25728	148.2257	52.46968	135.044
52.27133	137.5392	52.29435	130.9639	52.3574	133.5818	52.29818	140.6657	52.27395	148.2221	52.48635	135.0447
52.288	137.5347	52.31102	130.9633	52.37407	133.5797	52.31485	140.6636	52.29062	148.2246	52.50302	135.0422
52.30467	137.5339	52.32768	130.9644	52.39073	133.5749	52.33152	140.6619	52.30728	148.2269	52.51968	135.0419
52.32133	137.5357	52.34435	130.9621	52.4074	133.5787	52.34818	140.6636	52.32395	148.2243	52.53635	135.0418
52.338	137.532	52.36102	130.9603	52.42407	133.5747	52.36485	140.6621	52.34062	148.2241	52.55302	135.0424
52.35467	137.5299	52.37768	130.9573	52.44073	133.5763	52.38152	140.6587	52.35728	148.2251	52.56968	135.0381
52.37133	137.5295	52.39435	130.9536	52.4574	133.5754	52.39818	140.6596	52.37395	148.225	52.58635	135.0391
52.388	137.5249	52.41102	130.9539	52.47407	133.577	52.41485	140.6616	52.39062	148.2242	52.60302	135.0378
52.40467	137.5241	52.42768	130.9516	52.49073	133.5733	52.43152	140.6592	52.40728	148.2242	52.61968	135.0361
52.42133	137.5244	52.44435	130.9536	52.5074	133.5742	52.44818	140.6587	52.42395	148.228	52.63635	135.0364
52.438	137.5215	52.46102	130.9503	52.52407	133.5758	52.46485	140.6575	52.44062	148.2263	52.65302	135.0361
52.45467	137.52	52.47768	130.9457	52.54073	133.5748	52.48152	140.6561	52.45728	148.2257	52.66968	135.0343
52.47133	137.5219	52.49435	130.9491	52.5574	133.5724	52.49818	140.6539	52.47395	148.2268	52.68635	135.0331
52.488	137.5194	52.51102	130.9476	52.57407	133.5749	52.51485	140.6549	52.49062	148.2246	52.70302	135.0365
52.50467	137.5165	52.52768	130.9448	52.59073	133.5757	52.53152	140.6548	52.50728	148.2245	52.71968	135.0355
52.52133	137.5171	52.54435	130.9442	52.6074	133.5703	52.54818	140.652	52.52395	148.2247	52.73635	135.0346
52.538	137.5134	52.56102	130.9445	52.62407	133.5706	52.56485	140.6515	52.54062	148.2257	52.75302	135.0312
52.55467	137.511	52.57768	130.9412	52.64073	133.5722	52.58152	140.6519	52.55728	148.2271	52.76968	135.032
52.57133	137.5106	52.59435	130.9389	52.6574	133.5713	52.59818	140.6511	52.57395	148.2231	52.78635	135.0311
52.588	137.5097	52.61102	130.9402	52.67407	133.5695	52.61485	140.6507	52.59062	148.2236	52.80302	135.0294
52.60467	137.5059	52.62768	130.9364	52.69073	133.57	52.63152	140.6493	52.60728	148.2234	52.81968	135.0311
52.62133	137.5078	52.64435	130.9346	52.7074	133.5711	52.64818	140.648	52.62395	148.2214	52.83635	135.0294
52.638	137.503	52.66102	130.935	52.72407	133.5678	52.66485	140.6475	52.64062	148.2239	52.85302	135.0287
52.65467	137.5033	52.67768	130.934	52.74073	133.5683	52.68152	140.6469	52.65728	148.2249	52.86968	135.0271
52.67133	137.5012	52.69435	130.9301	52.7574	133.5682	52.69818	140.6457	52.67395	148.2216	52.88635	135.0255
52.688	137.5006	52.71102	130.9327	52.77407	133.5696	52.71485	140.647	52.69062	148.2243	52.90302	135.0254
52.70467	137.4966	52.72768	130.9287	52.79073	133.5696	52.73152	140.6453	52.70728	148.2231	52.91968	135.025
52.72133	137.4991	52.74435	130.9308	52.8074	133.5673	52.74818	140.6428	52.72395	148.222	52.93635	135.0247
52.738	137.4954	52.76102	130.9274	52.82407	133.5661	52.76485	140.6428	52.74062	148.2214	52.95302	135.0244
52.75467	137.4918	52.77768	130.9259	52.84073	133.5668	52.78152	140.644	52.75728	148.223	52.96968	135.0231
52.77133	137.4941	52.79435	130.9237	52.8574	133.5653	52.79818	140.6428	52.77395	148.2201	52.98635	135.024
52.788	137.4955	52.81102	130.9238	52.87407	133.5674	52.81485	140.6419	52.79062	148.2223	53.00302	135.0221
52.80467	137.4902	52.82768	130.9217	52.89073	133.566	52.83152	140.6413	52.80728	148.2234	53.01968	135.0217
52.82133	137.4907	52.84435	130.9222	52.9074	133.5642	52.84818	140.6391	52.82395	148.22	53.03635	135.0203
52.838	137.4885	52.86102	130.9194	52.92407	133.5622	52.86485	140.6405	52.84062	148.2202	53.05302	135.0218
52.85467	137.4846	52.87768	130.916	52.94073	133.564	52.88152	140.6377	52.85728	148.2216	53.06968	135.0196
52.87133	137.4881	52.89435	130.9158	52.9574	133.5634	52.89818	140.6378	52.87395	148.2197	53.08635	135.0187

52.888	137.4839	52.91102	130.9155	52.97407	133.5667	52.91485	140.6358	52.89062	148.2219	53.10302	135.0184
52.90467	137.482	52.92768	130.9156	52.99073	133.565	52.93152	140.6327	52.90728	148.2228	53.11968	135.017
52.92133	137.4806	52.94435	130.9145	53.0074	133.5621	52.94818	140.6348	52.92395	148.2204	53.13635	135.0167
52.938	137.481	52.96102	130.9157	53.02407	133.5601	52.96485	140.634	52.94062	148.2173	53.15302	135.0145
52.95467	137.4761	52.97768	130.9087	53.04073	133.5617	52.98152	140.6301	52.95728	148.2225	53.16968	135.0146
52.97133	137.4789	52.99435	130.9045	53.0574	133.5593	52.99818	140.6312	52.97395	148.2187	53.18635	135.0133
52.988	137.4753	53.01102	130.9053	53.07407	133.5612	53.01485	140.6318	52.99062	148.2199	53.20302	135.0135
53.00467	137.4735	53.02768	130.9038	53.09073	133.5613	53.03152	140.6298	53.00728	148.2202	53.21968	135.0097
53.02133	137.4746	53.04435	130.9031	53.1074	133.559	53.04818	140.6318	53.02395	148.2188	53.23635	135.0118
53.038	137.4721	53.06102	130.9033	53.12407	133.5597	53.06485	140.6309	53.04062	148.2213	53.25302	135.007
53.05467	137.4723	53.07768	130.8979	53.14073	133.5576	53.08152	140.6297	53.05728	148.2207	53.26968	135.007
53.07133	137.4709	53.09435	130.8999	53.1574	133.5597	53.09818	140.6291	53.07395	148.2166	53.28635	135.0033
53.088	137.4652	53.11102	130.8936	53.17407	133.5591	53.11485	140.6258	53.09062	148.2183	53.30302	135.0055
53.10467	137.4626	53.12768	130.8957	53.19073	133.5593	53.13152	140.6276	53.10728	148.2195	53.31968	135.0044
53.12133	137.4645	53.14435	130.8945	53.2074	133.555	53.14818	140.626	53.12395	148.2202	53.33635	135.0029
53.138	137.4614	53.16102	130.8948	53.22407	133.5552	53.16485	140.6253	53.14062	148.2179	53.35302	135.0033
53.15467	137.4591	53.17768	130.8932	53.24073	133.5571	53.18152	140.6237	53.15728	148.219	53.36968	135.0017
53.17133	137.4611	53.19435	130.8851	53.2574	133.5557	53.19818	140.6228	53.17395	148.2165	53.38635	135.0008
53.188	137.4578	53.21102	130.8862	53.27407	133.5549	53.21485	140.6228	53.19062	148.2181	53.40302	134.9986
53.20467	137.4556	53.22768	130.8843	53.29073	133.5536	53.23152	140.624	53.20728	148.2149	53.41968	134.9986
53.22133	137.4566	53.24435	130.881	53.3074	133.5564	53.24818	140.6199	53.22395	148.2158	53.43635	134.9985
53.238	137.4525	53.26102	130.8803	53.32407	133.5539	53.26485	140.6184	53.24062	148.2156	53.45302	134.9974
53.25467	137.4492	53.27768	130.8754	53.34073	133.5535	53.28152	140.6169	53.25728	148.2181	53.46968	134.9961
53.27133	137.4522	53.29435	130.876	53.3574	133.5512	53.29818	140.6179	53.27395	148.2163	53.48635	134.9929
53.288	137.4526	53.31102	130.874	53.37407	133.5514	53.31485	140.6177	53.29062	148.2169	53.50302	134.9932
53.30467	137.4482	53.32768	130.8742	53.39073	133.5511	53.33152	140.6148	53.30728	148.215	53.51968	134.993
53.32133	137.4479	53.34435	130.8736	53.4074	133.5506	53.34818	140.6164	53.32395	148.2124	53.53635	134.9918
53.338	137.4482	53.36102	130.8737	53.42407	133.549	53.36485	140.6135	53.34062	148.2136	53.55302	134.9899
53.35467	137.4449	53.37768	130.8712	53.44073	133.5475	53.38152	140.6168	53.35728	148.2187	53.56968	134.991
53.37133	137.4445	53.39435	130.8694	53.4574	133.5454	53.39818	140.6142	53.37395	148.2153	53.58635	134.9875
53.388	137.4421	53.41102	130.8665	53.47407	133.5459	53.41485	140.6125	53.39062	148.214	53.60302	134.9868
53.40467	137.4404	53.42768	130.8666	53.49073	133.5469	53.43152	140.6135	53.40728	148.2143	53.61968	134.9886
53.42133	137.4367	53.44435	130.8627	53.5074	133.5465	53.44818	140.6114	53.42395	148.2164	53.63635	134.9867
53.438	137.4382	53.46102	130.8628	53.52407	133.5446	53.46485	140.6126	53.44062	148.2137	53.65302	134.9859
53.45467	137.4358	53.47768	130.8632	53.54073	133.5461	53.48152	140.6088	53.45728	148.2145	53.66968	134.9843
53.47133	137.4352	53.49435	130.8621	53.5574	133.5443	53.49818	140.6117	53.47395	148.2141	53.68635	134.9869
53.488	137.4338	53.51102	130.8588	53.57407	133.5451	53.51485	140.6101	53.49062	148.2129	53.70302	134.9834
53.50467	137.4314	53.52768	130.8526	53.59073	133.5437	53.53152	140.6105	53.50728	148.2138	53.71968	134.9833
53.52133	137.4286	53.54435	130.8536	53.6074	133.5421	53.54818	140.6096	53.52395	148.2129	53.73635	134.9837
53.538	137.4289	53.56102	130.8513	53.62407	133.54	53.56485	140.6073	53.54062	148.2129	53.75302	134.9813
53.55467	137.4228	53.57768	130.8508	53.64073	133.5425	53.58152	140.6082	53.55728	148.2163	53.76968	134.9791
53.57133	137.4246	53.59435	130.8509	53.6574	133.5415	53.59818	140.6074	53.57395	148.2135	53.78635	134.9791
53.588	137.4227	53.61102	130.849	53.67407	133.5402	53.61485	140.6051	53.59062	148.211	53.80302	134.9801
53.60467	137.4195	53.62768	130.8442	53.69073	133.5389	53.63152	140.6046	53.60728	148.2099	53.81968	134.9803
53.62133	137.4187	53.64435	130.8504	53.7074	133.5387	53.64818	140.6026	53.62395	148.2109	53.83635	134.9765
53.638	137.4201	53.66102	130.8426	53.72407	133.5391	53.66485	140.6047	53.64062	148.2106	53.85302	134.976
53.65467	137.414	53.67768	130.8412	53.74073	133.5397	53.68152	140.6023	53.65728	148.2122	53.86968	134.9765
53.67133	137.4125	53.69435	130.8419	53.7574	133.5376	53.69818	140.6035	53.67395	148.2111	53.88635	134.9741
53.688	137.4118	53.71102	130.8403	53.77407	133.5363	53.71485	140.6002	53.69062	148.2108	53.90302	134.9748
53.70467	137.408	53.72768	130.8394	53.79073	133.5358	53.73152	140.601	53.70728	148.2159	53.91968	134.9738
53.72133	137.4116	53.74435	130.8363	53.8074	133.536	53.74818	140.5993	53.72395	148.2103	53.93635	134.9713
53.738	137.4067	53.76102	130.8351	53.82407	133.537	53.76485	140.6004	53.74062	148.2137	53.95302	134.9733
53.75467	137.4048	53.77768	130.8339	53.84073	133.5326	53.78152	140.5992	53.75728	148.2128	53.96968	134.9711
53.77133	137.4042	53.79435	130.8303	53.8574	133.5334	53.79818	140.5979	53.77395	148.208	53.98635	134.9712
53.788	137.4002	53.81102	130.832	53.87407	133.5335	53.81485	140.5962	53.79062	148.2107	54.00302	134.97
53.80467	137.405	53.82768	130.8266	53.89073	133.5336	53.83152	140.5972	53.80728	148.2109	54.01968	134.9677
53.82133	137.4004	53.84435	130.8269	53.9074	133.5323	53.84818	140.5964	53.82395	148.2122	54.03635	134.9688
53.838	137.4008	53.86102	130.8251	53.92407	133.5296	53.86485	140.5935	53.84062	148.2112	54.05302	134.9697
53.85467	137.3982	53.87768	130.824	53.94073	133.5304	53.88152	140.5924	53.85728	148.2104	54.06968	134.9672
53.87133	137.3933	53.89435	130.8234	53.9574	133.5314	53.89818	140.5914	53.87395	148.2103	54.08635	134.9663
53.888	137.4012	53.91102	130.8179	53.97407	133.5306	53.91485	140.5901	53.89062	148.2111	54.10302	134.9656
53.90467	137.3955	53.92768	130.8184	53.99073	133.5296	53.93152	140.5899	53.90728	148.2111	54.11968	134.9642
53.92133	137.3964	53.94435	130.8167	54.0074	133.5304	53.94818	140.5891	53.92395	148.2119	54.13635	134.9632
53.938	137.3927	53.96102	130.813	54.02407	133.5274	53.96485	140.5857	53.94062	148.2102	54.15302	134.9634
53.95467	137.3908	53.97768	130.8113	54.04073	133.5321	53.98152	140.5853	53.95728	148.2107	54.16968	134.9624
53.97133	137.3879	53.99435	130.8144	54.0574	133.5256	53.99818	140.5859	53.97395	148.2096	54.18635	134.9599
53.988	137.3847	54.01102	130.8111	54.07407	133.5291	54.01485	140.584	53.99062	148.209	54.20302	134.9608
54.00467	137.3789	54.02768	130.8105	54.09073	133.5249	54.03152	140.5848	54.00728	148.2101	54.21968	134.9591
54.02133	137.3855	54.04435	130.8094	54.1074	133.5284	54.04818	140.5807	54.02395	148.2087	54.23635	134.9587

54.038	137.3812	54.06102	130.8068	54.12407	133.5272	54.06485	140.5807	54.04062	148.2094	54.25302	134.9583
54.05467	137.3782	54.07768	130.8069	54.14073	133.5257	54.08152	140.5814	54.05728	148.2087	54.26968	134.9576
54.07133	137.3785	54.09435	130.8044	54.1574	133.5255	54.09818	140.578	54.07395	148.2089	54.28635	134.9571
54.088	137.378	54.11102	130.8037	54.17407	133.5244	54.11485	140.5802	54.09062	148.209	54.30302	134.9574
54.10467	137.377	54.12768	130.8037	54.19073	133.5271	54.13152	140.5765	54.10728	148.2111	54.31968	134.956
54.12133	137.3733	54.14435	130.7995	54.2074	133.5254	54.14818	140.5782	54.12395	148.209	54.33635	134.9559
54.138	137.3699	54.16102	130.7987	54.22407	133.526	54.16485	140.5751	54.14062	148.2105	54.35302	134.9558
54.15467	137.3701	54.17768	130.7946	54.24073	133.5254	54.18152	140.5731	54.15728	148.2083	54.36968	134.9535
54.17133	137.3673	54.19435	130.7977	54.2574	133.5224	54.19818	140.5767	54.17395	148.2085	54.38635	134.9509
54.188	137.367	54.21102	130.7923	54.27407	133.5217	54.21485	140.5764	54.19062	148.2094	54.40302	134.9521
54.20467	137.3672	54.22768	130.7905	54.29073	133.5226	54.23152	140.5726	54.20728	148.2087	54.41968	134.9525
54.22133	137.3659	54.24435	130.7904	54.3074	133.521	54.24818	140.5768	54.22395	148.2078	54.43635	134.9518
54.238	137.3622	54.26102	130.7895	54.32407	133.5225	54.26485	140.5741	54.24062	148.2061	54.45302	134.9483
54.25467	137.363	54.27768	130.7879	54.34073	133.5214	54.28152	140.5731	54.25728	148.2085	54.46968	134.9483
54.27133	137.361	54.29435	130.7844	54.3574	133.5187	54.29818	140.5727	54.27395	148.2067	54.48635	134.9479
54.288	137.3595	54.31102	130.7858	54.37407	133.5213	54.31485	140.5707	54.29062	148.2073	54.50302	134.9486
54.30467	137.3579	54.32768	130.7845	54.39073	133.5193	54.33152	140.5732	54.30728	148.2073	54.51968	134.9465
54.32133	137.3565	54.34435	130.7835	54.4074	133.519	54.34818	140.5707	54.32395	148.2063	54.53635	134.9438
54.338	137.3556	54.36102	130.7812	54.42407	133.5183	54.36485	140.5701	54.34062	148.2089	54.55302	134.9403
54.35467	137.3539	54.37768	130.7811	54.44073	133.5184	54.38152	140.5687	54.35728	148.2079	54.56968	134.9437
54.37133	137.35	54.39435	130.7777	54.4574	133.5157	54.39818	140.5699	54.37395	148.2054	54.58635	134.94
54.388	137.3502	54.41102	130.7775	54.47407	133.5205	54.41485	140.568	54.39062	148.2041	54.60302	134.9394
54.40467	137.3486	54.42768	130.7793	54.49073	133.5188	54.43152	140.5676	54.40728	148.2066	54.61968	134.9364
54.42133	137.3484	54.44435	130.7736	54.5074	133.5156	54.44818	140.5657	54.42395	148.207	54.63635	134.9358
54.438	137.3456	54.46102	130.77	54.52407	133.5149	54.46485	140.5671	54.44062	148.205	54.65302	134.9349
54.45467	137.3426	54.47768	130.7676	54.54073	133.5135	54.48152	140.5657	54.45728	148.2051	54.66968	134.936
54.47133	137.3446	54.49435	130.7662	54.5574	133.5143	54.49818	140.5634	54.47395	148.2066	54.68635	134.9326
54.488	137.3425	54.51102	130.7664	54.57407	133.5167	54.51485	140.5643	54.49062	148.2075	54.70302	134.9312
54.50467	137.3406	54.52768	130.7659	54.59073	133.514	54.53152	140.5649	54.50728	148.2059	54.71968	134.9286
54.52133	137.3381	54.54435	130.761	54.6074	133.5135	54.54818	140.5625	54.52395	148.2053	54.73635	134.9312
54.538	137.3396	54.56102	130.7606	54.62407	133.5146	54.56485	140.5643	54.54062	148.2057	54.75302	134.9294
54.55467	137.3385	54.57768	130.7591	54.64073	133.5133	54.58152	140.5609	54.55728	148.2041	54.76968	134.9294
54.57133	137.3345	54.59435	130.7603	54.6574	133.513	54.59818	140.5603	54.57395	148.2058	54.78635	134.9261
54.588	137.3335	54.61102	130.757	54.67407	133.5134	54.61485	140.5598	54.59062	148.2079	54.80302	134.9265
54.60467	137.3354	54.62768	130.7592	54.69073	133.5124	54.63152	140.561	54.60728	148.2048	54.81968	134.9255
54.62133	137.3325	54.64435	130.7584	54.7074	133.5106	54.64818	140.5584	54.62395	148.2056	54.83635	134.9235
54.638	137.3302	54.66102	130.7555	54.72407	133.5107	54.66485	140.5581	54.64062	148.2071	54.85302	134.9217
54.65467	137.3322	54.67768	130.7548	54.74073	133.5109	54.68152	140.5584	54.65728	148.2053	54.86968	134.9208
54.67133	137.3243	54.69435	130.7557	54.7574	133.5111	54.69818	140.5567	54.67395	148.2075	54.88635	134.9193
54.688	137.3278	54.71102	130.7521	54.77407	133.5103	54.71485	140.558	54.69062	148.2084	54.90302	134.9208
54.70467	137.3255	54.72768	130.7529	54.79073	133.5078	54.73152	140.5552	54.70728	148.2071	54.91968	134.9173
54.72133	137.322	54.74435	130.7478	54.8074	133.5079	54.74818	140.556	54.72395	148.2049	54.93635	134.9188
54.738	137.322	54.76102	130.7488	54.82407	133.5066	54.76485	140.5544	54.74062	148.2048	54.95302	134.9159
54.75467	137.3199	54.77768	130.7477	54.84073	133.5072	54.78152	140.5537	54.75728	148.2037	54.96968	134.916
54.77133	137.3169	54.79435	130.7439	54.8574	133.5061	54.79818	140.5562	54.77395	148.2049	54.98635	134.9151
54.788	137.3163	54.81102	130.7421	54.87407	133.5016	54.81485	140.5526	54.79062	148.2065	55.00302	134.9139
54.80467	137.3154	54.82768	130.7433	54.89073	133.501	54.83152	140.5531	54.80728	148.2061	55.01968	134.9112
54.82133	137.314	54.84435	130.7416	54.9074	133.5038	54.84818	140.5512	54.82395	148.2042	55.03635	134.9121
54.838	137.3135	54.86102	130.7374	54.92407	133.5026	54.86485	140.5514	54.84062	148.2038	55.05302	134.9082
54.85467	137.3103	54.87768	130.7401	54.94073	133.5018	54.88152	140.5515	54.85728	148.2033	55.06968	134.9105
54.87133	137.3094	54.89435	130.7373	54.9574	133.5018	54.89818	140.5511	54.87395	148.2077	55.08635	134.9055
54.888	137.3076	54.91102	130.739	54.97407	133.5008	54.91485	140.5509	54.89062	148.2046	55.10302	134.9075
54.90467	137.3065	54.92768	130.7348	54.99073	133.5009	54.93152	140.5498	54.90728	148.2037	55.11968	134.9072
54.92133	137.3072	54.94435	130.7339	55.0074	133.5008	54.94818	140.5477	54.92395	148.2041	55.13635	134.9053
54.938	137.3039	54.96102	130.7314	55.02407	133.5003	54.96485	140.5489	54.94062	148.2041	55.15302	134.9044
54.95467	137.3043	54.97768	130.7336	55.04073	133.4976	54.98152	140.5468	54.95728	148.2017	55.16968	134.9045
54.97133	137.3005	54.99435	130.7302	55.0574	133.4994	54.99818	140.5469	54.97395	148.2056	55.18635	134.9035
54.988	137.3018	55.01102	130.7269	55.07407	133.4993	55.01485	140.5453	54.99062	148.2017	55.20302	134.9031
55.00467	137.3009	55.02768	130.7272	55.09073	133.4994	55.03152	140.5445	55.00728	148.2057	55.21968	134.901
55.02133	137.2966	55.04435	130.7261	55.1074	133.497	55.04818	140.5467	55.02395	148.2035	55.23635	134.8992
55.038	137.2963	55.06102	130.7219	55.12407	133.4983	55.06485	140.5456	55.04062	148.2061	55.25302	134.8991
55.05467	137.2991	55.07768	130.7223	55.14073	133.4956	55.08152	140.5442	55.05728	148.2071	55.26968	134.8992
55.07133	137.2949	55.09435	130.7202	55.1574	133.4948	55.09818	140.5442	55.07395	148.2046	55.28635	134.8961
55.088	137.296	55.11102	130.7197	55.17407	133.4952	55.11485	140.5429	55.09062	148.206	55.30302	134.8991
55.10467	137.2941	55.12768	130.7179	55.19073	133.4927	55.13152	140.5428	55.10728	148.2049	55.31968	134.8928
55.12133	137.2904	55.14435	130.7147	55.2074	133.4942	55.14818	140.5424	55.12395	148.2053	55.33635	134.8941
55.138	137.2907	55.16102	130.7151	55.22407	133.492	55.16485	140.5428	55.14062	148.2056	55.35302	134.8927
55.15467	137.2873	55.17768	130.7141	55.24073	133.4921	55.18152	140.538	55.15728	148.2052	55.36968	134.8933
55.17133	137.2864	55.19435	130.7127	55.2574	133.4912	55.19818	140.5432	55.17395	148.2054	55.38635	134.8923

55.188	137.2868	55.21102	130.7093	55.27407	133.4897	55.21485	140.5411	55.19062	148.2073	55.40302	134.8904
55.20467	137.283	55.22768	130.7112	55.29073	133.4889	55.23152	140.5405	55.20728	148.2076	55.41968	134.8904
55.22133	137.2823	55.24435	130.7068	55.3074	133.488	55.24818	140.5389	55.22395	148.2051	55.43635	134.8891
55.238	137.2807	55.26102	130.706	55.32407	133.4881	55.26485	140.5374	55.24062	148.2076	55.45302	134.8875
55.25467	137.2799	55.27768	130.7062	55.34073	133.4888	55.28152	140.5399	55.25728	148.2068	55.46968	134.8865
55.27133	137.2785	55.29435	130.7031	55.3574	133.4883	55.29818	140.5394	55.27395	148.2093	55.48635	134.8863
55.288	137.276	55.31102	130.7013	55.37407	133.4856	55.31485	140.5359	55.29062	148.208	55.50302	134.8855
55.30467	137.276	55.32768	130.6987	55.39073	133.4858	55.33152	140.5335	55.30728	148.2074	55.51968	134.8869
55.32133	137.2731	55.34435	130.6993	55.4074	133.4858	55.34818	140.5342	55.32395	148.2045	55.53635	134.8817
55.338	137.2706	55.36102	130.699	55.42407	133.4868	55.36485	140.5367	55.34062	148.2057	55.55302	134.8826
55.35467	137.2701	55.37768	130.6974	55.44073	133.4858	55.38152	140.5332	55.35728	148.2059	55.56968	134.8842
55.37133	137.2665	55.39435	130.6991	55.4574	133.4835	55.39818	140.5321	55.37395	148.2072	55.58635	134.8822
55.388	137.2667	55.41102	130.6974	55.47407	133.4833	55.41485	140.5312	55.39062	148.2073	55.60302	134.879
55.40467	137.2632	55.42768	130.6917	55.49073	133.4816	55.43152	140.531	55.40728	148.2057	55.61968	134.8793
55.42133	137.2637	55.44435	130.6968	55.5074	133.4826	55.44818	140.5318	55.42395	148.206	55.63635	134.8791
55.438	137.2645	55.46102	130.6903	55.52407	133.481	55.46485	140.5303	55.44062	148.2033	55.65302	134.8768
55.45467	137.2651	55.47768	130.6881	55.54073	133.4818	55.48152	140.5293	55.45728	148.2068	55.66968	134.8775
55.47133	137.2581	55.49435	130.6875	55.5574	133.481	55.49818	140.5291	55.47395	148.2079	55.68635	134.8723
55.488	137.261	55.51102	130.6858	55.57407	133.478	55.51485	140.5309	55.49062	148.2046	55.70302	134.8746
55.50467	137.2616	55.52768	130.6828	55.59073	133.479	55.53152	140.5288	55.50728	148.2042	55.71968	134.8724
55.52133	137.2551	55.54435	130.6835	55.6074	133.4795	55.54818	140.5293	55.52395	148.2049	55.73635	134.8749
55.538	137.2541	55.56102	130.6779	55.62407	133.4794	55.56485	140.5284	55.54062	148.2056	55.75302	134.8715
55.55467	137.2521	55.57768	130.6781	55.64073	133.4782	55.58152	140.5288	55.55728	148.2056	55.76968	134.8694
55.57133	137.2534	55.59435	130.6782	55.6574	133.4761	55.59818	140.5285	55.57395	148.2038	55.78635	134.8725
55.588	137.2504	55.61102	130.6751	55.67407	133.4741	55.61485	140.5259	55.59062	148.2035	55.80302	134.8712
55.60467	137.2507	55.62768	130.6759	55.69073	133.4732	55.63152	140.527	55.60728	148.2054	55.81968	134.869
55.62133	137.2478	55.64435	130.6712	55.7074	133.4738	55.64818	140.5261	55.62395	148.2039	55.83635	134.8698
55.638	137.2428	55.66102	130.6777	55.72407	133.4729	55.66485	140.5231	55.64062	148.2047	55.85302	134.8691
55.65467	137.2458	55.67768	130.6687	55.74073	133.4702	55.68152	140.5266	55.65728	148.2058	55.86968	134.8699
55.67133	137.2418	55.69435	130.6671	55.7574	133.4698	55.69818	140.5239	55.67395	148.2045	55.88635	134.8667
55.688	137.2432	55.71102	130.6713	55.77407	133.4711	55.71485	140.522	55.69062	148.2046	55.90302	134.8675
55.70467	137.2413	55.72768	130.6632	55.79073	133.4703	55.73152	140.5209	55.70728	148.2049	55.91968	134.8649
55.72133	137.2408	55.74435	130.6702	55.8074	133.4692	55.74818	140.5194	55.72395	148.2068	55.93635	134.8639
55.738	137.2389	55.76102	130.6625	55.82407	133.4665	55.76485	140.5226	55.74062	148.2066	55.95302	134.8643
55.75467	137.2368	55.77768	130.6649	55.84073	133.4681	55.78152	140.5196	55.75728	148.2019	55.96968	134.8631
55.77133	137.2338	55.79435	130.6581	55.8574	133.4677	55.79818	140.5193	55.77395	148.2048	55.98635	134.8623
55.788	137.2328	55.81102	130.6577	55.87407	133.4661	55.81485	140.5161	55.79062	148.2052	56.00302	134.8615
55.80467	137.2324	55.82768	130.6581	55.89073	133.465	55.83152	140.5205	55.80728	148.2063	56.01968	134.8633
55.82133	137.2317	55.84435	130.6551	55.9074	133.466	55.84818	140.518	55.82395	148.2046	56.03635	134.8618
55.838	137.2298	55.86102	130.6516	55.92407	133.4645	55.86485	140.5182	55.84062	148.2049	56.05302	134.8578
55.85467	137.2245	55.87768	130.6556	55.94073	133.4641	55.88152	140.5151	55.85728	148.2057	56.06968	134.8592
55.87133	137.2233	55.89435	130.6537	55.9574	133.4633	55.89818	140.5145	55.87395	148.207	56.08635	134.8596
55.888	137.2227	55.91102	130.6553	55.97407	133.4633	55.91485	140.5162	55.89062	148.2067	56.10302	134.8607
55.90467	137.2227	55.92768	130.6493	55.99073	133.4634	55.93152	140.5158	55.90728	148.2055	56.11968	134.856
55.92133	137.2205	55.94435	130.6494	56.0074	133.4617	55.94818	140.5135	55.92395	148.2046	56.13635	134.8569
55.938	137.2163	55.96102	130.6489	56.02407	133.4613	55.96485	140.5129	55.94062	148.2057	56.15302	134.8568
55.95467	137.2193	55.97768	130.6441	56.04073	133.4597	55.98152	140.5115	55.95728	148.2028	56.16968	134.8541
55.97133	137.2159	55.99435	130.6442	56.0574	133.4613	55.99818	140.512	55.97395	148.2056	56.18635	134.8519
55.988	137.2166	56.01102	130.6428	56.07407	133.4593	56.01485	140.5094	55.99062	148.2055	56.20302	134.8511
56.00467	137.2133	56.02768	130.6437	56.09073	133.4606	56.03152	140.5085	56.00728	148.2033	56.21968	134.8543
56.02133	137.2123	56.04435	130.6409	56.1074	133.4588	56.04818	140.5079	56.02395	148.203	56.23635	134.8504
56.038	137.2106	56.06102	130.6358	56.12407	133.4587	56.06485	140.5107	56.04062	148.2049	56.25302	134.8527
56.05467	137.2087	56.07768	130.6375	56.14073	133.4568	56.08152	140.5076	56.05728	148.2064	56.26968	134.85
56.07133	137.2109	56.09435	130.6354	56.1574	133.4558	56.09818	140.5088	56.07395	148.202	56.28635	134.8474
56.088	137.2119	56.11102	130.6361	56.17407	133.4566	56.11485	140.5059	56.09062	148.2048	56.30302	134.8498
56.10467	137.2025	56.12768	130.6339	56.19073	133.4533	56.13152	140.506	56.10728	148.2047	56.31968	134.8498
56.12133	137.2076	56.14435	130.6329	56.2074	133.4541	56.14818	140.5051	56.12395	148.2023	56.33635	134.848
56.138	137.2037	56.16102	130.6308	56.22407	133.4546	56.16485	140.5044	56.14062	148.2022	56.35302	134.845
56.15467	137.1985	56.17768	130.6313	56.24073	133.455	56.18152	140.5038	56.15728	148.2049	56.36968	134.8466
56.17133	137.1983	56.19435	130.627	56.2574	133.4538	56.19818	140.5028	56.17395	148.2039	56.38635	134.846
56.188	137.1968	56.21102	130.6281	56.27407	133.4527	56.21485	140.5026	56.19062	148.2011	56.40302	134.846
56.20467	137.1967	56.22768	130.6281	56.29073	133.4499	56.23152	140.5021	56.20728	148.203	56.41968	134.845
56.22133	137.1937	56.24435	130.6244	56.3074	133.4525	56.24818	140.5012	56.22395	148.2026	56.43635	134.8424
56.238	137.1906	56.26102	130.6223	56.32407	133.4535	56.26485	140.503	56.24062	148.2022	56.45302	134.8427
56.25467	137.192	56.27768	130.6232	56.34073	133.4511	56.28152	140.4987	56.25728	148.2041	56.46968	134.843
56.27133	137.19	56.29435	130.6212	56.3574	133.4518	56.29818	140.4983	56.27395	148.2061	56.48635	134.8411
56.288	137.1929	56.31102	130.6205	56.37407	133.4522	56.31485	140.5001	56.29062	148.2008	56.50302	134.8387
56.30467	137.1881	56.32768	130.6131	56.39073	133.4513	56.33152	140.4976	56.30728	148.2031	56.51968	134.8361
56.32133	137.1859	56.34435	130.6161	56.4074	133.452	56.34818	140.4973	56.32395	148.2039	56.53635	134.8384

56.338	137.1841	56.36102	130.6115	56.42407	133.4527	56.36485	140.4978	56.34062	148.2048	56.55302	134.8376
56.35467	137.1813	56.37768	130.6131	56.44073	133.4522	56.38152	140.4959	56.35728	148.2042	56.56968	134.8377
56.37133	137.1816	56.39435	130.6106	56.4574	133.45	56.39818	140.4956	56.37395	148.2032	56.58635	134.8345
56.388	137.1815	56.41102	130.6079	56.47407	133.4518	56.41485	140.4951	56.39062	148.2019	56.60302	134.8339
56.40467	137.1778	56.42768	130.6066	56.49073	133.4539	56.43152	140.4961	56.40728	148.2023	56.61968	134.8318
56.42133	137.1792	56.44435	130.6053	56.5074	133.4533	56.44818	140.494	56.42395	148.2045	56.63635	134.8326
56.438	137.1729	56.46102	130.604	56.52407	133.4526	56.46485	140.4952	56.44062	148.2035	56.65302	134.8316
56.45467	137.1741	56.47768	130.6009	56.54073	133.4537	56.48152	140.4927	56.45728	148.2009	56.66968	134.8307
56.47133	137.1733	56.49435	130.6012	56.5574	133.4542	56.49818	140.4928	56.47395	148.2045	56.68635	134.829
56.488	137.1715	56.51102	130.5996	56.57407	133.4548	56.51485	140.4922	56.49062	148.2032	56.70302	134.8285
56.50467	137.1694	56.52768	130.5998	56.59073	133.454	56.53152	140.4929	56.50728	148.2034	56.71968	134.8264
56.52133	137.1733	56.54435	130.5989	56.6074	133.4552	56.54818	140.489	56.52395	148.201	56.73635	134.8272
56.538	137.168	56.56102	130.5948	56.62407	133.4542	56.56485	140.4899	56.54062	148.2029	56.75302	134.8254
56.55467	137.1707	56.57768	130.594	56.64073	133.4546	56.58152	140.4914	56.55728	148.2032	56.76968	134.8252
56.57133	137.1687	56.59435	130.5945	56.6574	133.4523	56.59818	140.4888	56.57395	148.204	56.78635	134.8233
56.588	137.1655	56.61102	130.5927	56.67407	133.4521	56.61485	140.4883	56.59062	148.2009	56.80302	134.824
56.60467	137.1626	56.62768	130.5912	56.69073	133.4504	56.63152	140.4886	56.60728	148.2024	56.81968	134.8212
56.62133	137.1645	56.64435	130.5923	56.7074	133.4521	56.64818	140.4876	56.62395	148.2035	56.83635	134.8198
56.638	137.1596	56.66102	130.5857	56.72407	133.45	56.66485	140.4886	56.64062	148.2036	56.85302	134.8201
56.65467	137.1622	56.67768	130.5896	56.74073	133.4496	56.68152	140.4874	56.65728	148.2028	56.86968	134.8187
56.67133	137.1576	56.69435	130.5844	56.7574	133.451	56.69818	140.4874	56.67395	148.2021	56.88635	134.8165
56.688	137.1579	56.71102	130.5843	56.77407	133.4488	56.71485	140.4864	56.69062	148.2022	56.90302	134.8149
56.70467	137.1552	56.72768	130.5817	56.79073	133.4477	56.73152	140.4853	56.70728	148.2049	56.91968	134.8158
56.72133	137.1527	56.74435	130.583	56.8074	133.4482	56.74818	140.4835	56.72395	148.2019	56.93635	134.8159
56.738	137.1495	56.76102	130.5818	56.82407	133.4465	56.76485	140.4847	56.74062	148.2043	56.95302	134.815
56.75467	137.1506	56.77768	130.5778	56.84073	133.4484	56.78152	140.4864	56.75728	148.2038	56.96968	134.8124
56.77133	137.1503	56.79435	130.5789	56.8574	133.4465	56.79818	140.4821	56.77395	148.2024	56.98635	134.809
56.788	137.1483	56.81102	130.5773	56.87407	133.4477	56.81485	140.4809	56.79062	148.2038	57.00302	134.8117
56.80467	137.1453	56.82768	130.5732	56.89073	133.446	56.83152	140.4804	56.80728	148.2033	57.01968	134.8109
56.82133	137.1462	56.84435	130.5738	56.9074	133.4448	56.84818	140.4793	56.82395	148.2042	57.03635	134.8091
56.838	137.1438	56.86102	130.5734	56.92407	133.4421	56.86485	140.4822	56.84062	148.204	57.05302	134.8047
56.85467	137.1447	56.87768	130.5736	56.94073	133.4431	56.88152	140.4801	56.85728	148.2016	57.06968	134.8058
56.87133	137.1424	56.89435	130.5716	56.9574	133.442	56.89818	140.4762	56.87395	148.2058	57.08635	134.8052
56.888	137.1428	56.91102	130.569	56.97407	133.4434	56.91485	140.4781	56.89062	148.202	57.10302	134.8035
56.90467	137.1382	56.92768	130.5666	56.99073	133.441	56.93152	140.4778	56.90728	148.2034	57.11968	134.8038
56.92133	137.1367	56.94435	130.5671	57.0074	133.4434	56.94818	140.4765	56.92395	148.2017	57.13635	134.8022
56.938	137.1361	56.96102	130.5674	57.02407	133.4413	56.96485	140.4769	56.94062	148.2064	57.15302	134.8003
56.95467	137.1366	56.97768	130.5625	57.04073	133.4402	56.98152	140.4738	56.95728	148.2041	57.16968	134.802
56.97133	137.1346	56.99435	130.5633	57.0574	133.4406	56.99818	140.4766	56.97395	148.2047	57.18635	134.7982
56.988	137.1309	57.01102	130.5642	57.07407	133.4423	57.01485	140.4728	56.99062	148.2021	57.20302	134.7994
57.00467	137.1307	57.02768	130.5582	57.09073	133.4403	57.03152	140.4761	57.00728	148.2053	57.21968	134.799
57.02133	137.1327	57.04435	130.5572	57.1074	133.4414	57.04818	140.4728	57.02395	148.2042	57.23635	134.797
57.038	137.1315	57.06102	130.5582	57.12407	133.442	57.06485	140.4723	57.04062	148.2075	57.25302	134.7938
57.05467	137.1323	57.07768	130.5581	57.14073	133.4387	57.08152	140.4711	57.05728	148.2048	57.26968	134.795
57.07133	137.1282	57.09435	130.5541	57.1574	133.441	57.09818	140.4728	57.07395	148.2042	57.28635	134.7914
57.088	137.1235	57.11102	130.5506	57.17407	133.4385	57.11485	140.472	57.09062	148.2034	57.30302	134.7919
57.10467	137.1254	57.12768	130.5538	57.19073	133.4403	57.13152	140.4704	57.10728	148.2027	57.31968	134.79
57.12133	137.1251	57.14435	130.5496	57.2074	133.4369	57.14818	140.4693	57.12395	148.2038	57.33635	134.792
57.138	137.1262	57.16102	130.5465	57.22407	133.4344	57.16485	140.4718	57.14062	148.2084	57.35302	134.7882
57.15467	137.1261	57.17768	130.5522	57.24073	133.4357	57.18152	140.4676	57.15728	148.2054	57.36968	134.7921
57.17133	137.1202	57.19435	130.546	57.2574	133.4358	57.19818	140.468	57.17395	148.2052	57.38635	134.7883
57.188	137.1185	57.21102	130.5468	57.27407	133.4353	57.21485	140.467	57.19062	148.201	57.40302	134.7882
57.20467	137.1169	57.22768	130.5475	57.29073	133.4338	57.23152	140.4666	57.20728	148.2039	57.41968	134.7891
57.22133	137.1144	57.24435	130.5439	57.3074	133.4329	57.24818	140.4665	57.22395	148.2043	57.43635	134.7883
57.238	137.1153	57.26102	130.5388	57.32407	133.4333	57.26485	140.4656	57.24062	148.2056	57.45302	134.7839
57.25467	137.1129	57.27768	130.5409	57.34073	133.4327	57.28152	140.4665	57.25728	148.2041	57.46968	134.7865
57.27133	137.1094	57.29435	130.5393	57.3574	133.4343	57.29818	140.4632	57.27395	148.2035	57.48635	134.7857
57.288	137.1042	57.31102	130.5402	57.37407	133.4313	57.31485	140.463	57.29062	148.2035	57.50302	134.7842
57.30467	137.1072	57.32768	130.5361	57.39073	133.4311	57.33152	140.4619	57.30728	148.2043	57.51968	134.7825
57.32133	137.1074	57.34435	130.5364	57.4074	133.4315	57.34818	140.463	57.32395	148.2053	57.53635	134.782
57.338	137.102	57.36102	130.5314	57.42407	133.4294	57.36485	140.462	57.34062	148.2065	57.55302	134.7834
57.35467	137.1043	57.37768	130.5324	57.44073	133.433	57.38152	140.4606	57.35728	148.2068	57.56968	134.7836
57.37133	137.1013	57.39435	130.5296	57.4574	133.432	57.39818	140.4609	57.37395	148.2073	57.58635	134.7829
57.388	137.0996	57.41102	130.5299	57.47407	133.43	57.41485	140.4606	57.39062	148.2064	57.60302	134.781
57.40467	137.0995	57.42768	130.5256	57.49073	133.4288	57.43152	140.4584	57.40728	148.2064	57.61968	134.7788
57.42133	137.095	57.44435	130.5273	57.5074	133.4325	57.44818	140.4596	57.42395	148.208	57.63635	134.7788
57.438	137.0972	57.46102	130.5249	57.52407	133.4301	57.46485	140.458	57.44062	148.2064	57.65302	134.7777
57.45467	137.0949	57.47768	130.5255	57.54073	133.4299	57.48152	140.4579	57.45728	148.207	57.66968	134.7782
57.47133	137.0946	57.49435	130.5245	57.5574	133.4279	57.49818	140.4546	57.47395	148.2083	57.68635	134.7765

57.488	137.0923	57.51102	130.5245	57.57407	133.4274	57.51485	140.4558	57.49062	148.2092	57.70302	134.7724
57.50467	137.0928	57.52768	130.5185	57.59073	133.4308	57.53152	140.4568	57.50728	148.2095	57.71968	134.7735
57.52133	137.0904	57.54435	130.5201	57.6074	133.4266	57.54818	140.4534	57.52395	148.208	57.73635	134.7701
57.538	137.0882	57.56102	130.5214	57.62407	133.4266	57.56485	140.4537	57.54062	148.209	57.75302	134.7722
57.55467	137.0863	57.57768	130.516	57.64073	133.4262	57.58152	140.4499	57.55728	148.2104	57.76968	134.7707
57.57133	137.0858	57.59435	130.5159	57.6574	133.4273	57.59818	140.4518	57.57395	148.2098	57.78635	134.7685
57.588	137.0796	57.61102	130.5145	57.67407	133.4265	57.61485	140.4529	57.59062	148.209	57.80302	134.7672
57.60467	137.0834	57.62768	130.513	57.69073	133.4228	57.63152	140.4499	57.60728	148.2078	57.81968	134.7659
57.62133	137.081	57.64435	130.5114	57.7074	133.427	57.64818	140.4484	57.62395	148.2121	57.83635	134.7646
57.638	137.0811	57.66102	130.5104	57.72407	133.4245	57.66485	140.4484	57.64062	148.2103	57.85302	134.7654
57.65467	137.0815	57.67768	130.5098	57.74073	133.4239	57.68152	140.4475	57.65728	148.2121	57.86968	134.762
57.67133	137.0742	57.69435	130.5083	57.7574	133.4236	57.69818	140.4459	57.67395	148.2141	57.88635	134.7612
57.688	137.0764	57.71102	130.5065	57.77407	133.4244	57.71485	140.4437	57.69062	148.21	57.90302	134.7612
57.70467	137.074	57.72768	130.5037	57.79073	133.4228	57.73152	140.445	57.70728	148.2131	57.91968	134.7602
57.72133	137.0726	57.74435	130.5064	57.8074	133.4239	57.74818	140.4432	57.72395	148.2139	57.93635	134.758
57.738	137.0677	57.76102	130.5028	57.82407	133.4224	57.76485	140.4452	57.74062	148.2162	57.95302	134.7586
57.75467	137.07	57.77768	130.5027	57.84073	133.4185	57.78152	140.4446	57.75728	148.2107	57.96968	134.7587
57.77133	137.0682	57.79435	130.5001	57.8574	133.4215	57.79818	140.4433	57.77395	148.2121	57.98635	134.7582
57.788	137.0661	57.81102	130.4987	57.87407	133.4213	57.81485	140.4422	57.79062	148.2132	58.00302	134.7565
57.80467	137.0647	57.82768	130.4942	57.89073	133.4198	57.83152	140.4411	57.80728	148.2097	58.01968	134.7561
57.82133	137.0615	57.84435	130.4948	57.9074	133.4203	57.84818	140.4415	57.82395	148.2086	58.03635	134.7561
57.838	137.061	57.86102	130.4942	57.92407	133.4232	57.86485	140.4413	57.84062	148.2108	58.05302	134.7557
57.85467	137.0631	57.87768	130.4908	57.94073	133.4197	57.88152	140.4425	57.85728	148.2157	58.06968	134.7528
57.87133	137.0598	57.89435	130.4931	57.9574	133.4182	57.89818	140.4385	57.87395	148.2134	58.08635	134.7523
57.888	137.0656	57.91102	130.4923	57.97407	133.4192	57.91485	140.4416	57.89062	148.2167	58.10302	134.7533
57.90467	137.0646	57.92768	130.4901	57.99073	133.4194	57.93152	140.4414	57.90728	148.2137	58.11968	134.7525
57.92133	137.0623	57.94435	130.4893	58.0074	133.4185	57.94818	140.4369	57.92395	148.2136	58.13635	134.7504
57.938	137.0557	57.96102	130.4889	58.02407	133.42	57.96485	140.438	57.94062	148.2136	58.15302	134.7498
57.95467	137.0595	57.97768	130.4877	58.04073	133.4201	57.98152	140.4364	57.95728	148.2126	58.16968	134.75
57.97133	137.0586	57.99435	130.4856	58.0574	133.4207	57.99818	140.4369	57.97395	148.212	58.18635	134.7488
57.988	137.0551	58.01102	130.4847	58.07407	133.417	58.01485	140.4378	57.99062	148.2133	58.20302	134.7484
58.00467	137.054	58.02768	130.4825	58.09073	133.4154	58.03152	140.4356	58.00728	148.2099	58.21968	134.7464
58.02133	137.0504	58.04435	130.4827	58.1074	133.418	58.04818	140.4344	58.02395	148.2111	58.23635	134.7489
58.038	137.0508	58.06102	130.482	58.12407	133.4154	58.06485	140.4354	58.04062	148.2163	58.25302	134.7479
58.05467	137.0507	58.07768	130.48	58.14073	133.4179	58.08152	140.4338	58.05728	148.2145	58.26968	134.7474
58.07133	137.0486	58.09435	130.4794	58.1574	133.4183	58.09818	140.4329	58.07395	148.2136	58.28635	134.7436
58.088	137.0483	58.11102	130.4754	58.17407	133.4176	58.11485	140.4345	58.09062	148.2121	58.30302	134.7442
58.10467	137.0442	58.12768	130.4786	58.19073	133.416	58.13152	140.4303	58.10728	148.2153	58.31968	134.7451
58.12133	137.0433	58.14435	130.4749	58.2074	133.4153	58.14818	140.4299	58.12395	148.2137	58.33635	134.7409
58.138	137.0436	58.16102	130.4718	58.22407	133.4158	58.16485	140.4306	58.14062	148.2123	58.35302	134.7421
58.15467	137.0421	58.17768	130.4701	58.24073	133.4173	58.18152	140.4286	58.15728	148.2148	58.36968	134.7422
58.17133	137.039	58.19435	130.4718	58.2574	133.4158	58.19818	140.4304	58.17395	148.2104	58.38635	134.7399
58.188	137.0376	58.21102	130.4751	58.27407	133.411	58.21485	140.4287	58.19062	148.2122	58.40302	134.7378
58.20467	137.0372	58.22768	130.4655	58.29073	133.413	58.23152	140.4248	58.20728	148.2142	58.41968	134.7373
58.22133	137.0343	58.24435	130.4684	58.3074	133.4145	58.24818	140.4271	58.22395	148.2135	58.43635	134.7345
58.238	137.0319	58.26102	130.4667	58.32407	133.4128	58.26485	140.4246	58.24062	148.2133	58.45302	134.7367
58.25467	137.0318	58.27768	130.4665	58.34073	133.411	58.28152	140.4259	58.25728	148.2134	58.46968	134.7338
58.27133	137.0292	58.29435	130.4702	58.3574	133.4139	58.29818	140.4257	58.27395	148.2124	58.48635	134.7324
58.288	137.0285	58.31102	130.462	58.37407	133.4129	58.31485	140.4228	58.29062	148.2128	58.50302	134.733
58.30467	137.0302	58.32768	130.4617	58.39073	133.4158	58.33152	140.4259	58.30728	148.21	58.51968	134.7329
58.32133	137.0262	58.34435	130.4651	58.4074	133.4149	58.34818	140.4237	58.32395	148.2138	58.53635	134.7297
58.338	137.0233	58.36102	130.4597	58.42407	133.4117	58.36485	140.421	58.34062	148.2139	58.55302	134.729
58.35467	137.024	58.37768	130.4546	58.44073	133.4134	58.38152	140.4251	58.35728	148.2141	58.56968	134.7272
58.37133	137.0227	58.39435	130.46	58.4574	133.4166	58.39818	140.4219	58.37395	148.2164	58.58635	134.7266
58.388	137.0196	58.41102	130.4529	58.47407	133.4128	58.41485	140.4224	58.39062	148.2123	58.60302	134.7262
58.40467	137.0172	58.42768	130.4548	58.49073	133.4089	58.43152	140.421	58.40728	148.2113	58.61968	134.7234
58.42133	137.0185	58.44435	130.457	58.5074	133.4114	58.44818	140.4196	58.42395	148.2131	58.63635	134.7248
58.438	137.0165	58.46102	130.4527	58.52407	133.4103	58.46485	140.4208	58.44062	148.2142	58.65302	134.7233
58.45467	137.0151	58.47768	130.4504	58.54073	133.4097	58.48152	140.4185	58.45728	148.2163	58.66968	134.7231
58.47133	137.0144	58.49435	130.4503	58.5574	133.4121	58.49818	140.419	58.47395	148.2148	58.68635	134.7186
58.488	137.0105	58.51102	130.4479	58.57407	133.4091	58.51485	140.4162	58.49062	148.2141	58.70302	134.7192
58.50467	137.0103	58.52768	130.4463	58.59073	133.4086	58.53152	140.4181	58.50728	148.2152	58.71968	134.7176
58.52133	137.0086	58.54435	130.4481	58.6074	133.4094	58.54818	140.4188	58.52395	148.2129	58.73635	134.7166
58.538	137.0099	58.56102	130.4439	58.62407	133.4098	58.56485	140.4181	58.54062	148.214	58.75302	134.7145
58.55467	137.0063	58.57768	130.443	58.64073	133.4098	58.58152	140.4167	58.55728	148.2154	58.76968	134.7135
58.57133	137.0072	58.59435	130.4458	58.6574	133.4104	58.59818	140.4135	58.57395	148.2154	58.78635	134.7136
58.588	137.002	58.61102	130.4439	58.67407	133.4094	58.61485	140.414	58.59062	148.2142	58.80302	134.711
58.60467	137.0021	58.62768	130.4391	58.69073	133.4105	58.63152	140.4131	58.60728	148.214	58.81968	134.7105
58.62133	137.0013	58.64435	130.4409	58.7074	133.4085	58.64818	140.4139	58.62395	148.2139	58.83635	134.7074

58.638	137.0007	58.66102	130.4391	58.72407	133.4097	58.66485	140.4101	58.64062	148.213	58.85302	134.7073
58.65467	136.9973	58.67768	130.435	58.74073	133.4091	58.68152	140.4096	58.65728	148.2142	58.86968	134.7076
58.67133	136.9984	58.69435	130.4364	58.7574	133.4096	58.69818	140.4106	58.67395	148.2138	58.88635	134.7063
58.688	136.9903	58.71102	130.4363	58.77407	133.4075	58.71485	140.4114	58.69062	148.2143	58.90302	134.7049
58.70467	136.9932	58.72768	130.4315	58.79073	133.4096	58.73152	140.4082	58.70728	148.2134	58.91968	134.7043
58.72133	136.9913	58.74435	130.4355	58.8074	133.4087	58.74818	140.4075	58.72395	148.2128	58.93635	134.7055
58.738	136.9932	58.76102	130.4294	58.82407	133.4064	58.76485	140.4085	58.74062	148.2126	58.95302	134.707
58.75467	136.989	58.77768	130.4306	58.84073	133.4067	58.78152	140.4067	58.75728	148.215	58.96968	134.7041
58.77133	136.9874	58.79435	130.4316	58.8574	133.4045	58.79818	140.4076	58.77395	148.217	58.98635	134.7033
58.788	136.9855	58.81102	130.4236	58.87407	133.4043	58.81485	140.4094	58.79062	148.2143	59.00302	134.702
58.80467	136.9853	58.82768	130.4239	58.89073	133.4032	58.83152	140.4052	58.80728	148.2123	59.01968	134.7024
58.82133	136.9813	58.84435	130.4251	58.9074	133.4057	58.84818	140.4041	58.82395	148.2129	59.03635	134.6997
58.838	136.981	58.86102	130.4242	58.92407	133.4046	58.86485	140.4052	58.84062	148.2145	59.05302	134.6991
58.85467	136.9799	58.87768	130.4214	58.94073	133.4043	58.88152	140.4039	58.85728	148.2137	59.06968	134.7008
58.87133	136.9778	58.89435	130.4225	58.9574	133.403	58.89818	140.4039	58.87395	148.2151	59.08635	134.7025
58.888	136.9769	58.91102	130.4179	58.97407	133.4046	58.91485	140.4043	58.89062	148.2126	59.10302	134.6995
58.90467	136.974	58.92768	130.4194	58.99073	133.4006	58.93152	140.4017	58.90728	148.2143	59.11968	134.7002
58.92133	136.9732	58.94435	130.4151	59.0074	133.4	58.94818	140.4039	58.92395	148.2145	59.13635	134.697
58.938	136.9725	58.96102	130.4169	59.02407	133.4012	58.96485	140.4015	58.94062	148.2133	59.15302	134.698
58.95467	136.9719	58.97768	130.4133	59.04073	133.4053	58.98152	140.3982	58.95728	148.2142	59.16968	134.6955
58.97133	136.9684	58.99435	130.4105	59.0574	133.4016	58.99818	140.3983	58.97395	148.2166	59.18635	134.6968
58.988	136.9702	59.01102	130.4157	59.07407	133.4016	59.01485	140.3991	58.99062	148.2155	59.20302	134.6931
59.00467	136.9685	59.02768	130.4102	59.09073	133.4035	59.03152	140.3995	59.00728	148.2172	59.21968	134.6958
59.02133	136.9677	59.04435	130.4087	59.1074	133.4016	59.04818	140.3968	59.02395	148.2173	59.23635	134.6951
59.038	136.9661	59.06102	130.4053	59.12407	133.4015	59.06485	140.3934	59.04062	148.2174	59.25302	134.6925
59.05467	136.9663	59.07768	130.405	59.14073	133.4025	59.08152	140.3935	59.05728	148.2144	59.26968	134.6913
59.07133	136.9641	59.09435	130.4048	59.1574	133.3993	59.09818	140.3942	59.07395	148.2146	59.28635	134.6933
59.088	136.9624	59.11102	130.4026	59.17407	133.4018	59.11485	140.3892	59.09062	148.2166	59.30302	134.6937
59.10467	136.9578	59.12768	130.4019	59.19073	133.401	59.13152	140.3908	59.10728	148.2164	59.31968	134.6915
59.12133	136.9607	59.14435	130.4039	59.2074	133.4002	59.14818	140.3899	59.12395	148.2163	59.33635	134.6895
59.138	136.9581	59.16102	130.3988	59.22407	133.399	59.16485	140.3897	59.14062	148.2175	59.35302	134.6893
59.15467	136.956	59.17768	130.3944	59.24073	133.3998	59.18152	140.3902	59.15728	148.2152	59.36968	134.6903
59.17133	136.9573	59.19435	130.3943	59.2574	133.3993	59.19818	140.3881	59.17395	148.2163	59.38635	134.6894
59.188	136.9555	59.21102	130.3957	59.27407	133.3967	59.21485	140.3885	59.19062	148.2166	59.40302	134.6886
59.20467	136.9531	59.22768	130.3934	59.29073	133.3983	59.23152	140.3902	59.20728	148.2166	59.41968	134.686
59.22133	136.9556	59.24435	130.3946	59.3074	133.3998	59.24818	140.389	59.22395	148.216	59.43635	134.6864
59.238	136.952	59.26102	130.3932	59.32407	133.3988	59.26485	140.3901	59.24062	148.2187	59.45302	134.6845
59.25467	136.951	59.27768	130.3913	59.34073	133.3959	59.28152	140.3869	59.25728	148.2182	59.46968	134.6849
59.27133	136.9517	59.29435	130.3908	59.3574	133.3953	59.29818	140.3892	59.27395	148.2163	59.48635	134.6827
59.288	136.9474	59.31102	130.3872	59.37407	133.3941	59.31485	140.3875	59.29062	148.2182	59.50302	134.6854
59.30467	136.9473	59.32768	130.3856	59.39073	133.3974	59.33152	140.3865	59.30728	148.2175	59.51968	134.6809
59.32133	136.9468	59.34435	130.3841	59.4074	133.3949	59.34818	140.3868	59.32395	148.2163	59.53635	134.6805
59.338	136.944	59.36102	130.384	59.42407	133.3976	59.36485	140.3849	59.34062	148.2164	59.55302	134.6793
59.35467	136.9424	59.37768	130.3819	59.44073	133.3975	59.38152	140.385	59.35728	148.2191	59.56968	134.6795
59.37133	136.9441	59.39435	130.3829	59.4574	133.397	59.39818	140.3858	59.37395	148.2152	59.58635	134.6801
59.388	136.9409	59.41102	130.3813	59.47407	133.3968	59.41485	140.3839	59.39062	148.217	59.60302	134.6769
59.40467	136.9406	59.42768	130.3797	59.49073	133.3944	59.43152	140.3847	59.40728	148.2153	59.61968	134.6763
59.42133	136.9398	59.44435	130.3787	59.5074	133.3954	59.44818	140.3847	59.42395	148.2172	59.63635	134.6725
59.438	136.9396	59.46102	130.3755	59.52407	133.3914	59.46485	140.3818	59.44062	148.2176	59.65302	134.6763
59.45467	136.9383	59.47768	130.3726	59.54073	133.3914	59.48152	140.3833	59.45728	148.217	59.66968	134.6733
59.47133	136.9381	59.49435	130.3746	59.5574	133.3928	59.49818	140.3832	59.47395	148.2185	59.68635	134.673
59.488	136.9347	59.51102	130.3688	59.57407	133.393	59.51485	140.3827	59.49062	148.2167	59.70302	134.6698
59.50467	136.9315	59.52768	130.371	59.59073	133.3918	59.53152	140.3817	59.50728	148.2183	59.71968	134.6728
59.52133	136.9341	59.54435	130.369	59.6074	133.3914	59.54818	140.3811	59.52395	148.2155	59.73635	134.6711
59.538	136.9308	59.56102	130.3668	59.62407	133.3941	59.56485	140.3793	59.54062	148.2176	59.75302	134.6696
59.55467	136.93	59.57768	130.3652	59.64073	133.3898	59.58152	140.3783	59.55728	148.2165	59.76968	134.668
59.57133	136.9244	59.59435	130.3663	59.6574	133.3907	59.59818	140.3786	59.57395	148.2161	59.78635	134.6695
59.588	136.9293	59.61102	130.3614	59.67407	133.39	59.61485	140.379	59.59062	148.2156	59.80302	134.6666
59.60467	136.9252	59.62768	130.3646	59.69073	133.3932	59.63152	140.378	59.60728	148.2185	59.81968	134.6645
59.62133	136.9226	59.64435	130.361	59.7074	133.3913	59.64818	140.3816	59.62395	148.2144	59.83635	134.6677
59.638	136.9207	59.66102	130.3598	59.72407	133.391	59.66485	140.3762	59.64062	148.2159	59.85302	134.6656
59.65467	136.9198	59.67768	130.3579	59.74073	133.3912	59.68152	140.3745	59.65728	148.2151	59.86968	134.6642
59.67133	136.9204	59.69435	130.3591	59.7574	133.3905	59.69818	140.3782	59.67395	148.2165	59.88635	134.6633
59.688	136.9178	59.71102	130.3574	59.77407	133.3907	59.71485	140.378	59.69062	148.215	59.90302	134.6611
59.70467	136.9145	59.72768	130.3542	59.79073	133.3908	59.73152	140.377	59.70728	148.2143	59.91968	134.6614
59.72133	136.9134	59.74435	130.3524	59.8074	133.3919	59.74818	140.3748	59.72395	148.2178	59.93635	134.6575
59.738	136.9124	59.76102	130.3498	59.82407	133.3906	59.76485	140.3765	59.74062	148.2136	59.95302	134.6589
59.75467	136.9095	59.77768	130.3504	59.84073	133.3908	59.78152	140.3741	59.75728	148.2144	59.96968	134.6571
59.77133	136.9104	59.79435	130.3505	59.8574	133.3887	59.79818	140.376	59.77395	148.2166	59.98635	134.6582

59.788	136.9064	59.81102	130.3476	59.87407	133.3877	59.81485	140.3755	59.79062	148.2153	60.00302	134.6556
59.80467	136.9041	59.82768	130.3446	59.89073	133.3887	59.83152	140.3739	59.80728	148.2138	60.01968	134.6565
59.82133	136.9051	59.84435	130.3442	59.9074	133.3889	59.84818	140.3751	59.82395	148.2153	60.03635	134.6539
59.838	136.9063	59.86102	130.3417	59.92407	133.3895	59.86485	140.3748	59.84062	148.2186	60.05302	134.6553
59.85467	136.9033	59.87768	130.3432	59.94073	133.3844	59.88152	140.3744	59.85728	148.2169	60.06968	134.6526
59.87133	136.901	59.89435	130.3418	59.9574	133.3849	59.89818	140.373	59.87395	148.2174	60.08635	134.6544
59.888	136.8981	59.91102	130.3401	59.97407	133.3857	59.91485	140.372	59.89062	148.2186	60.10302	134.6505
59.90467	136.8959	59.92768	130.3387	59.99073	133.3863	59.93152	140.3724	59.90728	148.2189	60.11968	134.6516
59.92133	136.8996	59.94435	130.3366	60.0074	133.3822	59.94818	140.3706	59.92395	148.2193	60.13635	134.6479
59.938	136.8954	59.96102	130.335	60.02407	133.386	59.96485	140.3715	59.94062	148.2189	60.15302	134.6481
59.95467	136.8925	59.97768	130.3325	60.04073	133.3867	59.98152	140.3673	59.95728	148.218	60.16968	134.6476
59.97133	136.8913	59.99435	130.3347	60.0574	133.3849	59.99818	140.3707	59.97395	148.2153	60.18635	134.6452
59.988	136.8924	60.01102	130.3337	60.07407	133.3857	60.01485	140.3703	59.99062	148.2219	60.20302	134.6453
60.00467	136.8898	60.02768	130.3314	60.09073	133.3841	60.03152	140.3684	60.00728	148.2189	60.21968	134.6438
60.02133	136.8904	60.04435	130.3283	60.1074	133.3823	60.04818	140.3691	60.02395	148.2212	60.23635	134.6436
60.038	136.8869	60.06102	130.3275	60.12407	133.3845	60.06485	140.3683	60.04062	148.2206	60.25302	134.6428
60.05467	136.885	60.07768	130.3234	60.14073	133.3827	60.08152	140.3666	60.05728	148.221	60.26968	134.6418
60.07133	136.8858	60.09435	130.3254	60.1574	133.3835	60.09818	140.3657	60.07395	148.2233	60.28635	134.6401
60.088	136.8819	60.11102	130.3224	60.17407	133.3795	60.11485	140.3647	60.09062	148.2193	60.30302	134.6415
60.10467	136.8842	60.12768	130.323	60.19073	133.3798	60.13152	140.3647	60.10728	148.2216	60.31968	134.6368
60.12133	136.8803	60.14435	130.3209	60.2074	133.3793	60.14818	140.3617	60.12395	148.218	60.33635	134.6372
60.138	136.8785	60.16102	130.3202	60.22407	133.3799	60.16485	140.3628	60.14062	148.2235	60.35302	134.638
60.15467	136.8811	60.17768	130.3196	60.24073	133.3827	60.18152	140.3636	60.15728	148.2211	60.36968	134.635
60.17133	136.8773	60.19435	130.318	60.2574	133.3791	60.19818	140.3633	60.17395	148.2217	60.38635	134.6339
60.188	136.8757	60.21102	130.3155	60.27407	133.3811	60.21485	140.3614	60.19062	148.2232	60.40302	134.6346
60.20467	136.8724	60.22768	130.3139	60.29073	133.3811	60.23152	140.3604	60.20728	148.2213	60.41968	134.6336
60.22133	136.8738	60.24435	130.312	60.3074	133.3768	60.24818	140.361	60.22395	148.2225	60.43635	134.6326
60.238	136.8729	60.26102	130.3127	60.32407	133.3798	60.26485	140.3617	60.24062	148.224	60.45302	134.6299
60.25467	136.8721	60.27768	130.3141	60.34073	133.3797	60.28152	140.3614	60.25728	148.2214	60.46968	134.6281
60.27133	136.8717	60.29435	130.3093	60.3574	133.3747	60.29818	140.3593	60.27395	148.2214	60.48635	134.6298
60.288	136.8676	60.31102	130.3092	60.37407	133.3758	60.31485	140.3577	60.29062	148.2272	60.50302	134.627
60.30467	136.8633	60.32768	130.3069	60.39073	133.3755	60.33152	140.3601	60.30728	148.2231	60.51968	134.6286
60.32133	136.8657	60.34435	130.3062	60.4074	133.3784	60.34818	140.3586	60.32395	148.2219	60.53635	134.6241
60.338	136.8652	60.36102	130.307	60.42407	133.3796	60.36485	140.3597	60.34062	148.224	60.55302	134.6245
60.35467	136.8549	60.37768	130.3028	60.44073	133.3776	60.38152	140.3569	60.35728	148.2238	60.56968	134.6254
60.37133	136.8642	60.39435	130.3028	60.4574	133.3789	60.39818	140.3565	60.37395	148.2254	60.58635	134.6247
60.388	136.8616	60.41102	130.2994	60.47407	133.3758	60.41485	140.3581	60.39062	148.2216	60.60302	134.6231
60.40467	136.8603	60.42768	130.3003	60.49073	133.3742	60.43152	140.3574	60.40728	148.2247	60.61968	134.6221
60.42133	136.8587	60.44435	130.299	60.5074	133.3749	60.44818	140.3562	60.42395	148.2228	60.63635	134.6206
60.438	136.8609	60.46102	130.2974	60.52407	133.3786	60.46485	140.3552	60.44062	148.2194	60.65302	134.6188
60.45467	136.8505	60.47768	130.2936	60.54073	133.3754	60.48152	140.3556	60.45728	148.2242	60.66968	134.6175
60.47133	136.8549	60.49435	130.2938	60.5574	133.3748	60.49818	140.3549	60.47395	148.2201	60.68635	134.6183
60.488	136.8539	60.51102	130.2905	60.57407	133.3765	60.51485	140.3542	60.49062	148.2226	60.70302	134.6172
60.50467	136.8524	60.52768	130.2922	60.59073	133.3755	60.53152	140.3524	60.50728	148.219	60.71968	134.6175
60.52133	136.8501	60.54435	130.2897	60.6074	133.3734	60.54818	140.3542	60.52395	148.224	60.73635	134.6162
60.538	136.8473	60.56102	130.287	60.62407	133.3754	60.56485	140.3519	60.54062	148.2243	60.75302	134.6171
60.55467	136.8437	60.57768	130.2886	60.64073	133.3763	60.58152	140.3533	60.55728	148.2229	60.76968	134.614
60.57133	136.8402	60.59435	130.285	60.6574	133.3747	60.59818	140.352	60.57395	148.2253	60.78635	134.6132
60.588	136.8436	60.61102	130.2859	60.67407	133.3727	60.61485	140.3512	60.59062	148.2247	60.80302	134.6133
60.60467	136.8402	60.62768	130.2875	60.69073	133.3722	60.63152	140.3509	60.60728	148.2227	60.81968	134.6118
60.62133	136.837	60.64435	130.2852	60.7074	133.3719	60.64818	140.3511	60.62395	148.222	60.83635	134.6099
60.638	136.8345	60.66102	130.2813	60.72407	133.3724	60.66485	140.3505	60.64062	148.2265	60.85302	134.6099
60.65467	136.8364	60.67768	130.2811	60.74073	133.3702	60.68152	140.35	60.65728	148.2226	60.86968	134.6093
60.67133	136.8346	60.69435	130.2771	60.7574	133.3696	60.69818	140.3479	60.67395	148.2249	60.88635	134.6081
60.688	136.836	60.71102	130.275	60.77407	133.3689	60.71485	140.3463	60.69062	148.2258	60.90302	134.6064
60.70467	136.835	60.72768	130.274	60.79073	133.3676	60.73152	140.3466	60.70728	148.2266	60.91968	134.6068
60.72133	136.8313	60.74435	130.2735	60.8074	133.3689	60.74818	140.3475	60.72395	148.2228	60.93635	134.607
60.738	136.8322	60.76102	130.2744	60.82407	133.3687	60.76485	140.3478	60.74062	148.2258	60.95302	134.6051
60.75467	136.8323	60.77768	130.2717	60.84073	133.3679	60.78152	140.3447	60.75728	148.2235	60.96968	134.6026
60.77133	136.8302	60.79435	130.2697	60.8574	133.3672	60.79818	140.3444	60.77395	148.2244	60.98635	134.6008
60.788	136.828	60.81102	130.2691	60.87407	133.3683	60.81485	140.3422	60.79062	148.2228	61.00302	134.6016
60.80467	136.8267	60.82768	130.2687	60.89073	133.3655	60.83152	140.3428	60.80728	148.2261	61.01968	134.6008
60.82133	136.8222	60.84435	130.2659	60.9074	133.3663	60.84818	140.3439	60.82395	148.2244	61.03635	134.601
60.838	136.8234	60.86102	130.2642	60.92407	133.364	60.86485	140.3392	60.84062	148.2231	61.05302	134.6008
60.85467	136.8226	60.87768	130.2634	60.94073	133.3652	60.88152	140.3424	60.85728	148.2242	61.06968	134.5999
60.87133	136.8208	60.89435	130.264	60.9574	133.367	60.89818	140.3425	60.87395	148.2246	61.08635	134.5958
60.888	136.8206	60.91102	130.2581	60.97407	133.3654	60.91485	140.3422	60.89062	148.2243	61.10302	134.5986
60.90467	136.8186	60.92768	130.2613	60.99073	133.3648	60.93152	140.3397	60.90728	148.2248	61.11968	134.5962
60.92133	136.8176	60.94435	130.2575	61.0074	133.3624	60.94818	140.3375	60.92395	148.2236	61.13635	134.5966

60.938	136.817	60.96102	130.2567	61.02407	133.3668	60.96485	140.3412	60.94062	148.2252	61.15302	134.5936
60.95467	136.8121	60.97768	130.2543	61.04073	133.3662	60.98152	140.341	60.95728	148.2248	61.16968	134.595
60.97133	136.8127	60.99435	130.2552	61.0574	133.3637	60.99818	140.3362	60.97395	148.2283	61.18635	134.595
60.988	136.8121	61.01102	130.2526	61.07407	133.364	61.01485	140.3388	60.99062	148.228	61.20302	134.5936
61.00467	136.8103	61.02768	130.251	61.09073	133.3647	61.03152	140.3356	61.00728	148.2268	61.21968	134.5915
61.02133	136.8124	61.04435	130.249	61.1074	133.3622	61.04818	140.3384	61.02395	148.2278	61.23635	134.5895
61.038	136.8096	61.06102	130.2477	61.12407	133.3679	61.06485	140.3361	61.04062	148.2275	61.25302	134.5896
61.05467	136.8057	61.07768	130.2481	61.14073	133.3626	61.08152	140.3359	61.05728	148.2284	61.26968	134.5877
61.07133	136.8048	61.09435	130.2455	61.1574	133.3642	61.09818	140.3373	61.07395	148.2286	61.28635	134.5876
61.088	136.8056	61.11102	130.2444	61.17407	133.3628	61.11485	140.3323	61.09062	148.2279	61.30302	134.5847
61.10467	136.8012	61.12768	130.2401	61.19073	133.3607	61.13152	140.3335	61.10728	148.2258	61.31968	134.5865
61.12133	136.8013	61.14435	130.2417	61.2074	133.3604	61.14818	140.3339	61.12395	148.2286	61.33635	134.5861
61.138	136.7996	61.16102	130.2393	61.22407	133.3624	61.16485	140.333	61.14062	148.2282	61.35302	134.5843
61.15467	136.796	61.17768	130.2371	61.24073	133.3615	61.18152	140.3323	61.15728	148.2256	61.36968	134.5825
61.17133	136.7957	61.19435	130.2364	61.2574	133.3608	61.19818	140.3322	61.17395	148.2273	61.38635	134.5815
61.188	136.795	61.21102	130.2369	61.27407	133.3593	61.21485	140.3287	61.19062	148.2273	61.40302	134.5805
61.20467	136.7943	61.22768	130.2346	61.29073	133.3608	61.23152	140.3284	61.20728	148.2278	61.41968	134.5805
61.22133	136.7915	61.24435	130.2313	61.3074	133.3607	61.24818	140.3302	61.22395	148.2291	61.43635	134.5791
61.238	136.793	61.26102	130.2322	61.32407	133.3594	61.26485	140.328	61.24062	148.2296	61.45302	134.5798
61.25467	136.7892	61.27768	130.2284	61.34073	133.3608	61.28152	140.3292	61.25728	148.2282	61.46968	134.577
61.27133	136.7885	61.29435	130.2295	61.3574	133.3626	61.29818	140.3265	61.27395	148.2314	61.48635	134.5772
61.288	136.7868	61.31102	130.2289	61.37407	133.3605	61.31485	140.3265	61.29062	148.2296	61.50302	134.5763
61.30467	136.7877	61.32768	130.2275	61.39073	133.3607	61.33152	140.3286	61.30728	148.23	61.51968	134.5751
61.32133	136.7852	61.34435	130.2265	61.4074	133.3616	61.34818	140.3281	61.32395	148.2312	61.53635	134.5746
61.338	136.7817	61.36102	130.2237	61.42407	133.3604	61.36485	140.324	61.34062	148.2316	61.55302	134.5721
61.35467	136.7812	61.37768	130.221	61.44073	133.361	61.38152	140.3248	61.35728	148.2303	61.56968	134.5722
61.37133	136.7801	61.39435	130.2244	61.4574	133.3659	61.39818	140.3251	61.37395	148.2322	61.58635	134.5725
61.388	136.781	61.41102	130.2219	61.47407	133.3609	61.41485	140.3244	61.39062	148.2319	61.60302	134.5695
61.40467	136.7792	61.42768	130.2164	61.49073	133.3622	61.43152	140.3263	61.40728	148.2339	61.61968	134.5689
61.42133	136.7746	61.44435	130.2203	61.5074	133.3642	61.44818	140.323	61.42395	148.2344	61.63635	134.5689
61.438	136.7777	61.46102	130.22	61.52407	133.3606	61.46485	140.3215	61.44062	148.2337	61.65302	134.5694
61.45467	136.7764	61.47768	130.2135	61.54073	133.3604	61.48152	140.3221	61.45728	148.2322	61.66968	134.5662
61.47133	136.7746	61.49435	130.2167	61.5574	133.3621	61.49818	140.3219	61.47395	148.2342	61.68635	134.5663
61.488	136.7745	61.51102	130.21	61.57407	133.3617	61.51485	140.3211	61.49062	148.2312	61.70302	134.5657
61.50467	136.7743	61.52768	130.2121	61.59073	133.3662	61.53152	140.3195	61.50728	148.2301	61.71968	134.5648
61.52133	136.769	61.54435	130.2102	61.6074	133.3635	61.54818	140.3201	61.52395	148.2316	61.73635	134.5624
61.538	136.7691	61.56102	130.2093	61.62407	133.3628	61.56485	140.3187	61.54062	148.2296	61.75302	134.5641
61.55467	136.768	61.57768	130.2059	61.64073	133.3643	61.58152	140.3194	61.55728	148.2324	61.76968	134.562
61.57133	136.7704	61.59435	130.2049	61.6574	133.3621	61.59818	140.3187	61.57395	148.2316	61.78635	134.5601
61.588	136.766	61.61102	130.2016	61.67407	133.3616	61.61485	140.317	61.59062	148.2324	61.80302	134.5622
61.60467	136.7666	61.62768	130.2024	61.69073	133.3642	61.63152	140.3176	61.60728	148.2335	61.81968	134.5614
61.62133	136.7635	61.64435	130.2003	61.7074	133.3615	61.64818	140.3189	61.62395	148.2319	61.83635	134.5586
61.638	136.7644	61.66102	130.2012	61.72407	133.365	61.66485	140.3171	61.64062	148.2316	61.85302	134.5589
61.65467	136.7615	61.67768	130.2009	61.74073	133.3652	61.68152	140.3185	61.65728	148.2322	61.86968	134.5565
61.67133	136.7586	61.69435	130.1976	61.7574	133.3665	61.69818	140.3156	61.67395	148.2321	61.88635	134.5568
61.688	136.7549	61.71102	130.1953	61.77407	133.3672	61.71485	140.3187	61.69062	148.2332	61.90302	134.5555
61.70467	136.7577	61.72768	130.1995	61.79073	133.3639	61.73152	140.3143	61.70728	148.2322	61.91968	134.5546
61.72133	136.7548	61.74435	130.1935	61.8074	133.3668	61.74818	140.3161	61.72395	148.2332	61.93635	134.5549
61.738	136.7524	61.76102	130.1941	61.82407	133.3667	61.76485	140.3165	61.74062	148.2317	61.95302	134.5532
61.75467	136.7541	61.77768	130.1888	61.84073	133.3679	61.78152	140.3156	61.75728	148.2323	61.96968	134.5494
61.77133	136.7526	61.79435	130.1883	61.8574	133.3673	61.79818	140.3117	61.77395	148.2316	61.98635	134.5509
61.788	136.7478	61.81102	130.1896	61.87407	133.367	61.81485	140.3154	61.79062	148.2314	62.00302	134.5516
61.80467	136.7472	61.82768	130.1881	61.89073	133.3696	61.83152	140.3131	61.80728	148.2326	62.01968	134.55
61.82133	136.7483	61.84435	130.1848	61.9074	133.3673	61.84818	140.3117	61.82395	148.2339	62.03635	134.5489
61.838	136.7486	61.86102	130.1854	61.92407	133.37	61.86485	140.3113	61.84062	148.2306	62.05302	134.5496
61.85467	136.7439	61.87768	130.1826	61.94073	133.3686	61.88152	140.3125	61.85728	148.2329	62.06968	134.5476
61.87133	136.7455	61.89435	130.181	61.9574	133.3672	61.89818	140.3101	61.87395	148.2325	62.08635	134.5463
61.888	136.7423	61.91102	130.1796	61.97407	133.3701	61.91485	140.31	61.89062	148.2317	62.10302	134.5442
61.90467	136.7414	61.92768	130.1765	61.99073	133.3699	61.93152	140.3077	61.90728	148.2342	62.11968	134.5468
61.92133	136.7397	61.94435	130.1788	62.0074	133.366	61.94818	140.3081	61.92395	148.2351	62.13635	134.5437
61.938	136.7387	61.96102	130.177	62.02407	133.369	61.96485	140.3055	61.94062	148.2339	62.15302	134.5433
61.95467	136.7375	61.97768	130.1744	62.04073	133.3697	61.98152	140.3081	61.95728	148.236	62.16968	134.5404
61.97133	136.735	61.99435	130.1723	62.0574	133.3704	61.99818	140.3086	61.97395	148.2343	62.18635	134.5433
61.988	136.7367	62.01102	130.1689	62.07407	133.3696	62.01485	140.3043	61.99062	148.2348	62.20302	134.5369
62.00467	136.7343	62.02768	130.1699	62.09073	133.3718	62.03152	140.3034	62.00728	148.235	62.21968	134.5388
62.02133	136.7319	62.04435	130.168	62.1074	133.374	62.04818	140.3076	62.02395	148.2367	62.23635	134.5371
62.038	136.733	62.06102	130.1674	62.12407	133.3706	62.06485	140.3029	62.04062	148.2357	62.25302	134.5394
62.05467	136.7325	62.07768	130.1651	62.14073	133.3674	62.08152	140.3066	62.05728	148.2346	62.26968	134.5367
62.07133	136.728	62.09435	130.1654	62.1574	133.3723	62.09818	140.3048	62.07395	148.233	62.28635	134.535

62.088	136.726	62.11102	130.1639	62.17407	133.3704	62.11485	140.3024	62.09062	148.238	62.30302	134.5321
62.10467	136.7266	62.12768	130.1596	62.19073	133.3719	62.13152	140.3016	62.10728	148.2362	62.31968	134.5325
62.12133	136.7261	62.14435	130.1591	62.2074	133.3669	62.14818	140.3032	62.12395	148.238	62.33635	134.5328
62.138	136.7275	62.16102	130.1571	62.22407	133.3702	62.16485	140.3001	62.14062	148.2388	62.35302	134.5308
62.15467	136.7202	62.17768	130.1585	62.24073	133.3658	62.18152	140.3013	62.15728	148.24	62.36968	134.5304
62.17133	136.7227	62.19435	130.1551	62.2574	133.3696	62.19818	140.299	62.17395	148.2385	62.38635	134.5298
62.188	136.7198	62.21102	130.1539	62.27407	133.3683	62.21485	140.3015	62.19062	148.2384	62.40302	134.5285
62.20467	136.7199	62.22768	130.1556	62.29073	133.3694	62.23152	140.301	62.20728	148.2394	62.41968	134.5277
62.22133	136.7198	62.24435	130.1511	62.3074	133.3664	62.24818	140.2998	62.22395	148.2389	62.43635	134.5279
62.238	136.7196	62.26102	130.1545	62.32407	133.3681	62.26485	140.2995	62.24062	148.2404	62.45302	134.5247
62.25467	136.7129	62.27768	130.1504	62.34073	133.3681	62.28152	140.2958	62.25728	148.2422	62.46968	134.5259
62.27133	136.7128	62.29435	130.1457	62.3574	133.3667	62.29818	140.2959	62.27395	148.2407	62.48635	134.5265
62.288	136.7143	62.31102	130.1495	62.37407	133.3688	62.31485	140.3003	62.29062	148.2414	62.50302	134.5238
62.30467	136.711	62.32768	130.146	62.39073	133.3653	62.33152	140.2943	62.30728	148.2416	62.51968	134.522
62.32133	136.71	62.34435	130.1421	62.4074	133.3666	62.34818	140.2961	62.32395	148.2432	62.53635	134.5196
62.338	136.7117	62.36102	130.1419	62.42407	133.3666	62.36485	140.2962	62.34062	148.2398	62.55302	134.5215
62.35467	136.708	62.37768	130.1399	62.44073	133.3661	62.38152	140.2937	62.35728	148.2423	62.56968	134.5204
62.37133	136.7091	62.39435	130.1382	62.4574	133.3628	62.39818	140.2956	62.37395	148.2431	62.58635	134.5203
62.388	136.7045	62.41102	130.1363	62.47407	133.3619	62.41485	140.2954	62.39062	148.2422	62.60302	134.519
62.40467	136.7078	62.42768	130.1387	62.49073	133.3624	62.43152	140.2915	62.40728	148.2427	62.61968	134.5202
62.42133	136.7044	62.44435	130.1386	62.5074	133.3631	62.44818	140.2942	62.42395	148.243	62.63635	134.5187
62.438	136.7029	62.46102	130.1361	62.52407	133.3636	62.46485	140.2895	62.44062	148.24	62.65302	134.5176
62.45467	136.6995	62.47768	130.1331	62.54073	133.3612	62.48152	140.2929	62.45728	148.2415	62.66968	134.5154
62.47133	136.699	62.49435	130.1297	62.5574	133.363	62.49818	140.292	62.47395	148.2412	62.68635	134.5166
62.488	136.6998	62.51102	130.1307	62.57407	133.3666	62.51485	140.2901	62.49062	148.2413	62.70302	134.5152
62.50467	136.6999	62.52768	130.1267	62.59073	133.3648	62.53152	140.2886	62.50728	148.2413	62.71968	134.514
62.52133	136.6951	62.54435	130.127	62.6074	133.3632	62.54818	140.2907	62.52395	148.2421	62.73635	134.5123
62.538	136.6954	62.56102	130.1275	62.62407	133.3639	62.56485	140.2892	62.54062	148.2459	62.75302	134.5121
62.55467	136.6927	62.57768	130.1233	62.64073	133.3658	62.58152	140.2889	62.55728	148.2471	62.76968	134.5111
62.57133	136.6963	62.59435	130.1234	62.6574	133.3661	62.59818	140.288	62.57395	148.2453	62.78635	134.5099
62.588	136.6906	62.61102	130.1231	62.67407	133.3647	62.61485	140.2875	62.59062	148.2465	62.80302	134.5104
62.60467	136.693	62.62768	130.1213	62.69073	133.3616	62.63152	140.2864	62.60728	148.2456	62.81968	134.5098
62.62133	136.6892	62.64435	130.1163	62.7074	133.3643	62.64818	140.2874	62.62395	148.2439	62.83635	134.5082
62.638	136.6864	62.66102	130.1175	62.72407	133.367	62.66485	140.2886	62.64062	148.2459	62.85302	134.5054
62.65467	136.6878	62.67768	130.1151	62.74073	133.361	62.68152	140.2883	62.65728	148.2463	62.86968	134.5067
62.67133	136.686	62.69435	130.1149	62.7574	133.3654	62.69818	140.2852	62.67395	148.2462	62.88635	134.5062
62.688	136.683	62.71102	130.115	62.77407	133.3688	62.71485	140.2859	62.69062	148.2435	62.90302	134.502
62.70467	136.6826	62.72768	130.1121	62.79073	133.3664	62.73152	140.2863	62.70728	148.2459	62.91968	134.5028
62.72133	136.6854	62.74435	130.1111	62.8074	133.364	62.74818	140.2852	62.72395	148.2445	62.93635	134.501
62.738	136.6834	62.76102	130.1106	62.82407	133.3649	62.76485	140.2853	62.74062	148.247	62.95302	134.5026
62.75467	136.6821	62.77768	130.1069	62.84073	133.3647	62.78152	140.2846	62.75728	148.2492	62.96968	134.4993
62.77133	136.6802	62.79435	130.1039	62.8574	133.364	62.79818	140.2848	62.77395	148.2446	62.98635	134.4998
62.788	136.6798	62.81102	130.1063	62.87407	133.365	62.81485	140.2846	62.79062	148.2484	63.00302	134.4989
62.80467	136.6774	62.82768	130.1031	62.89073	133.3614	62.83152	140.2828	62.80728	148.2465	63.01968	134.4964
62.82133	136.6765	62.84435	130.1037	62.9074	133.36	62.84818	140.2823	62.82395	148.246	63.03635	134.4976
62.838	136.6741	62.86102	130.0993	62.92407	133.3598	62.86485	140.2833	62.84062	148.248	63.05302	134.4975
62.85467	136.6723	62.87768	130.0999	62.94073	133.3609	62.88152	140.2812	62.85728	148.2508	63.06968	134.4941
62.87133	136.6705	62.89435	130.0964	62.9574	133.3632	62.89818	140.2806	62.87395	148.2488	63.08635	134.493
62.888	136.6689	62.91102	130.0942	62.97407	133.3574	62.91485	140.2808	62.89062	148.2524	63.10302	134.4953
62.90467	136.6694	62.92768	130.0939	62.99073	133.3621	62.93152	140.2806	62.90728	148.2507	63.11968	134.4945
62.92133	136.666	62.94435	130.0928	63.0074	133.3588	62.94818	140.2797	62.92395	148.2513	63.13635	134.4917
62.938	136.6639	62.96102	130.0917	63.02407	133.3589	62.96485	140.2782	62.94062	148.2502	63.15302	134.4915
62.95467	136.6609	62.97768	130.093	63.04073	133.3584	62.98152	140.2765	62.95728	148.2517	63.16968	134.4911
62.97133	136.6663	62.99435	130.0911	63.0574	133.3576	62.99818	140.2757	62.97395	148.2523	63.18635	134.49
62.988	136.6611	63.01102	130.0881	63.07407	133.3571	63.01485	140.2773	62.99062	148.2521	63.20302	134.4875
63.00467	136.6607	63.02768	130.0899	63.09073	133.3583	63.03152	140.2779	63.00728	148.2527	63.21968	134.4904
63.02133	136.659	63.04435	130.0873	63.1074	133.357	63.04818	140.2752	63.02395	148.2547	63.23635	134.4874
63.038	136.6546	63.06102	130.0854	63.12407	133.3567	63.06485	140.275	63.04062	148.2533	63.25302	134.4865
63.05467	136.6577	63.07768	130.0831	63.14073	133.3557	63.08152	140.2751	63.05728	148.2552	63.26968	134.4839
63.07133	136.6545	63.09435	130.0788	63.1574	133.357	63.09818	140.2747	63.07395	148.2559	63.28635	134.4857
63.088	136.6532	63.11102	130.0781	63.17407	133.3542	63.11485	140.274	63.09062	148.2564	63.30302	134.4806
63.10467	136.654	63.12768	130.0785	63.19073	133.3538	63.13152	140.2758	63.10728	148.2536	63.31968	134.4815
63.12133	136.653	63.14435	130.0789	63.2074	133.3536	63.14818	140.272	63.12395	148.2541	63.33635	134.4793
63.138	136.6521	63.16102	130.0764	63.22407	133.351	63.16485	140.2745	63.14062	148.2565	63.35302	134.4803
63.15467	136.6493	63.17768	130.0752	63.24073	133.3511	63.18152	140.2738	63.15728	148.2559	63.36968	134.4774
63.17133	136.6497	63.19435	130.0733	63.2574	133.3493	63.19818	140.2725	63.17395	148.255	63.38635	134.4766
63.188	136.6476	63.21102	130.072	63.27407	133.3479	63.21485	140.2733	63.19062	148.2547	63.40302	134.4778
63.20467	136.6461	63.22768	130.0678	63.29073	133.3497	63.23152	140.2722	63.20728	148.2554	63.41968	134.478
63.22133	136.6502	63.24435	130.0679	63.3074	133.3488	63.24818	140.2694	63.22395	148.2575	63.43635	134.4751

63.238	136.645	63.26102	130.0679	63.32407	133.3479	63.26485	140.2699	63.24062	148.2565	63.45302	134.4755
63.25467	136.6464	63.27768	130.065	63.34073	133.3477	63.28152	140.2704	63.25728	148.2546	63.46968	134.472
63.27133	136.6435	63.29435	130.0645	63.3574	133.3504	63.29818	140.2668	63.27395	148.2572	63.48635	134.4746
63.288	136.6406	63.31102	130.0628	63.37407	133.3482	63.31485	140.27	63.29062	148.2523	63.50302	134.4703
63.30467	136.6414	63.32768	130.0635	63.39073	133.3477	63.33152	140.2666	63.30728	148.2542	63.51968	134.4723
63.32133	136.6407	63.34435	130.0632	63.4074	133.3482	63.34818	140.2687	63.32395	148.2549	63.53635	134.4716
63.338	136.637	63.36102	130.0607	63.42407	133.3492	63.36485	140.2649	63.34062	148.2537	63.55302	134.4693
63.35467	136.6381	63.37768	130.0583	63.44073	133.3466	63.38152	140.2676	63.35728	148.2519	63.56968	134.4684
63.37133	136.6373	63.39435	130.0582	63.4574	133.3498	63.39818	140.2671	63.37395	148.255	63.58635	134.4678
63.388	136.6357	63.41102	130.0572	63.47407	133.3488	63.41485	140.2673	63.39062	148.2573	63.60302	134.4662
63.40467	136.6361	63.42768	130.0552	63.49073	133.3479	63.43152	140.2653	63.40728	148.2572	63.61968	134.4645
63.42133	136.6339	63.44435	130.0538	63.5074	133.3465	63.44818	140.2655	63.42395	148.2602	63.63635	134.4646
63.438	136.6317	63.46102	130.0522	63.52407	133.3483	63.46485	140.2667	63.44062	148.2572	63.65302	134.4625
63.45467	136.6284	63.47768	130.0502	63.54073	133.3485	63.48152	140.267	63.45728	148.2569	63.66968	134.4624
63.47133	136.6315	63.49435	130.0523	63.5574	133.348	63.49818	140.2653	63.47395	148.2536	63.68635	134.4616
63.488	136.6271	63.51102	130.0518	63.57407	133.347	63.51485	140.2641	63.49062	148.2588	63.70302	134.4571
63.50467	136.6276	63.52768	130.0491	63.59073	133.3457	63.53152	140.2644	63.50728	148.2554	63.71968	134.4595
63.52133	136.6253	63.54435	130.0457	63.6074	133.3467	63.54818	140.2637	63.52395	148.259	63.73635	134.4593
63.538	136.6253	63.56102	130.046	63.62407	133.3441	63.56485	140.2618	63.54062	148.2595	63.75302	134.4591
63.55467	136.622	63.57768	130.042	63.64073	133.3454	63.58152	140.2614	63.55728	148.2562	63.76968	134.4574
63.57133	136.6205	63.59435	130.0434	63.6574	133.3462	63.59818	140.2603	63.57395	148.26	63.78635	134.4556
63.588	136.6204	63.61102	130.0395	63.67407	133.3433	63.61485	140.2591	63.59062	148.259	63.80302	134.456
63.60467	136.6181	63.62768	130.0413	63.69073	133.3451	63.63152	140.2604	63.60728	148.2573	63.81968	134.4542
63.62133	136.6178	63.64435	130.0394	63.7074	133.3445	63.64818	140.2573	63.62395	148.2575	63.83635	134.4529
63.638	136.6159	63.66102	130.0402	63.72407	133.3411	63.66485	140.2591	63.64062	148.2566	63.85302	134.4493
63.65467	136.6104	63.67768	130.0319	63.74073	133.3411	63.68152	140.2586	63.65728	148.2579	63.86968	134.4505
63.67133	136.6112	63.69435	130.0364	63.7574	133.3448	63.69818	140.2576	63.67395	148.2585	63.88635	134.4498
63.688	136.6097	63.71102	130.0309	63.77407	133.3399	63.71485	140.258	63.69062	148.2609	63.90302	134.4476
63.70467	136.6117	63.72768	130.0319	63.79073	133.3417	63.73152	140.2581	63.70728	148.2586	63.91968	134.4474
63.72133	136.6091	63.74435	130.0299	63.8074	133.3413	63.74818	140.2555	63.72395	148.2577	63.93635	134.4434
63.738	136.6097	63.76102	130.03	63.82407	133.3407	63.76485	140.2531	63.74062	148.2606	63.95302	134.4429
63.75467	136.6069	63.77768	130.0275	63.84073	133.337	63.78152	140.2556	63.75728	148.2569	63.96968	134.4421
63.77133	136.6083	63.79435	130.0248	63.8574	133.3361	63.79818	140.2558	63.77395	148.2622	63.98635	134.4404
63.788	136.6082	63.81102	130.0258	63.87407	133.3372	63.81485	140.2542	63.79062	148.2586	64.00302	134.4393
63.80467	136.6039	63.82768	130.0258	63.89073	133.337	63.83152	140.254	63.80728	148.2596	64.01968	134.4413
63.82133	136.6008	63.84435	130.0234	63.9074	133.3379	63.84818	140.2536	63.82395	148.2606	64.03635	134.4387
63.838	136.6029	63.86102	130.0224	63.92407	133.3384	63.86485	140.2522	63.84062	148.2668	64.05302	134.4385
63.85467	136.6012	63.87768	130.0208	63.94073	133.3375	63.88152	140.2508	63.85728	148.262	64.06968	134.4383
63.87133	136.601	63.89435	130.0195	63.9574	133.337	63.89818	140.2522	63.87395	148.261	64.08635	134.4357
63.888	136.5973	63.91102	130.0182	63.97407	133.3348	63.91485	140.2523	63.89062	148.263	64.10302	134.4377
63.90467	136.5985	63.92768	130.0167	63.99073	133.3356	63.93152	140.2483	63.90728	148.2622	64.11968	134.4352
63.92133	136.5953	63.94435	130.0151	64.0074	133.3348	63.94818	140.2513	63.92395	148.2621	64.13635	134.4319
63.938	136.5929	63.96102	130.0154	64.02407	133.3343	63.96485	140.2485	63.94062	148.2643	64.15302	134.4317
63.95467	136.5936	63.97768	130.0125	64.04073	133.3346	63.98152	140.2516	63.95728	148.2655	64.16968	134.4311
63.97133	136.5914	63.99435	130.0131	64.0574	133.333	63.99818	140.2482	63.97395	148.2657	64.18635	134.4316
63.988	136.5905	64.01102	130.008	64.07407	133.3308	64.01485	140.2476	63.99062	148.2667	64.20302	134.4314
64.00467	136.5882	64.02768	130.0092	64.09073	133.3312	64.03152	140.2491	64.00728	148.2646	64.21968	134.4314
64.02133	136.5906	64.04435	130.0115	64.1074	133.3303	64.04818	140.2463	64.02395	148.2681	64.23635	134.4299
64.038	136.5865	64.06102	130.0054	64.12407	133.3281	64.06485	140.246	64.04062	148.2666	64.25302	134.4291
64.05467	136.5856	64.07768	130.0051	64.14073	133.3316	64.08152	140.2457	64.05728	148.2651	64.26968	134.427
64.07133	136.5849	64.09435	130.0017	64.1574	133.3281	64.09818	140.2451	64.07395	148.2637	64.28635	134.4267
64.088	136.5817	64.11102	130.0003	64.17407	133.3249	64.11485	140.2434	64.09062	148.2673	64.30302	134.4255
64.10467	136.5813	64.12768	130.0003	64.19073	133.3259	64.13152	140.2446	64.10728	148.2684	64.31968	134.4258
64.12133	136.5801	64.14435	129.9983	64.2074	133.331	64.14818	140.2423	64.12395	148.2662	64.33635	134.423
64.138	136.579	64.16102	129.9983	64.22407	133.3289	64.16485	140.2447	64.14062	148.266	64.35302	134.4241
64.15467	136.5779	64.17768	129.9944	64.24073	133.325	64.18152	140.2428	64.15728	148.2685	64.36968	134.4212
64.17133	136.578	64.19435	129.9947	64.2574	133.3273	64.19818	140.2418	64.17395	148.2681	64.38635	134.4229
64.188	136.5736	64.21102	129.9938	64.27407	133.3263	64.21485	140.2432	64.19062	148.2706	64.40302	134.4197
64.20467	136.5767	64.22768	129.9942	64.29073	133.3269	64.23152	140.2415	64.20728	148.2669	64.41968	134.4197
64.22133	136.5751	64.24435	129.9858	64.3074	133.3262	64.24818	140.2438	64.22395	148.2663	64.43635	134.4184
64.238	136.5742	64.26102	129.992	64.32407	133.324	64.26485	140.2414	64.24062	148.2687	64.45302	134.4173
64.25467	136.5723	64.27768	129.985	64.34073	133.3275	64.28152	140.2405	64.25728	148.2668	64.46968	134.4167
64.27133	136.5719	64.29435	129.9848	64.3574	133.3252	64.29818	140.2397	64.27395	148.269	64.48635	134.4164
64.288	136.5704	64.31102	129.9867	64.37407	133.3255	64.31485	140.2408	64.29062	148.2713	64.50302	134.4152
64.30467	136.5667	64.32768	129.9818	64.39073	133.3267	64.33152	140.2386	64.30728	148.2687	64.51968	134.4143
64.32133	136.5661	64.34435	129.9818	64.4074	133.3268	64.34818	140.2391	64.32395	148.2664	64.53635	134.4154
64.338	136.5664	64.36102	129.9791	64.42407	133.3256	64.36485	140.2356	64.34062	148.2695	64.55302	134.412
64.35467	136.5644	64.37768	129.9774	64.44073	133.3242	64.38152	140.2353	64.35728	148.2688	64.56968	134.4102
64.37133	136.5631	64.39435	129.9765	64.4574	133.3256	64.39818	140.2358	64.37395	148.2675	64.58635	134.4109

64.388	136.5626	64.41102	129.978	64.47407	133.3238	64.41485	140.2364	64.39062	148.2725	64.60302	134.4097
64.40467	136.5588	64.42768	129.9768	64.49073	133.3244	64.43152	140.2371	64.40728	148.2712	64.61968	134.4112
64.42133	136.561	64.44435	129.9753	64.5074	133.3236	64.44818	140.235	64.42395	148.272	64.63635	134.4069
64.438	136.5578	64.46102	129.974	64.52407	133.3241	64.46485	140.2329	64.44062	148.274	64.65302	134.4084
64.45467	136.5566	64.47768	129.9695	64.54073	133.3256	64.48152	140.2341	64.45728	148.2737	64.66968	134.4083
64.47133	136.5562	64.49435	129.9705	64.5574	133.3229	64.49818	140.2344	64.47395	148.2704	64.68635	134.4071
64.488	136.558	64.51102	129.97	64.57407	133.3211	64.51485	140.234	64.49062	148.2758	64.70302	134.4052
64.50467	136.5505	64.52768	129.967	64.59073	133.3222	64.53152	140.2339	64.50728	148.2752	64.71968	134.4054
64.52133	136.5515	64.54435	129.9648	64.6074	133.3192	64.54818	140.2317	64.52395	148.2756	64.73635	134.4068
64.538	136.5527	64.56102	129.9602	64.62407	133.3205	64.56485	140.2322	64.54062	148.276	64.75302	134.4035
64.55467	136.5485	64.57768	129.9582	64.64073	133.3232	64.58152	140.2296	64.55728	148.2746	64.76968	134.4037
64.57133	136.5486	64.59435	129.9581	64.6574	133.3211	64.59818	140.2304	64.57395	148.2775	64.78635	134.4029
64.588	136.5503	64.61102	129.9566	64.67407	133.321	64.61485	140.2308	64.59062	148.2768	64.80302	134.4014
64.60467	136.5467	64.62768	129.9544	64.69073	133.3193	64.63152	140.2285	64.60728	148.2779	64.81968	134.4016
64.62133	136.5456	64.64435	129.9567	64.7074	133.3204	64.64818	140.2302	64.62395	148.2768	64.83635	134.3972
64.638	136.5425	64.66102	129.9531	64.72407	133.3194	64.66485	140.2278	64.64062	148.2839	64.85302	134.3993
64.65467	136.5412	64.67768	129.9538	64.74073	133.3193	64.68152	140.2284	64.65728	148.2804	64.86968	134.3985
64.67133	136.5388	64.69435	129.949	64.7574	133.3212	64.69818	140.2288	64.67395	148.2776	64.88635	134.3946
64.688	136.5376	64.71102	129.9485	64.77407	133.3191	64.71485	140.2311	64.69062	148.2773	64.90302	134.3973
64.70467	136.5366	64.72768	129.9479	64.79073	133.3221	64.73152	140.2262	64.70728	148.2774	64.91968	134.3952
64.72133	136.5364	64.74435	129.9444	64.8074	133.3188	64.74818	140.2277	64.72395	148.2824	64.93635	134.3933
64.738	136.533	64.76102	129.9421	64.82407	133.3193	64.76485	140.2278	64.74062	148.2811	64.95302	134.3937
64.75467	136.533	64.77768	129.9441	64.84073	133.3191	64.78152	140.226	64.75728	148.2794	64.96968	134.3929
64.77133	136.5308	64.79435	129.9433	64.8574	133.322	64.79818	140.2264	64.77395	148.2808	64.98635	134.3899
64.788	136.5308	64.81102	129.9423	64.87407	133.3226	64.81485	140.2238	64.79062	148.2804	65.00302	134.3895
64.80467	136.5295	64.82768	129.9395	64.89073	133.3213	64.83152	140.2228	64.80728	148.2802	65.01968	134.3871
64.82133	136.5284	64.84435	129.9349	64.9074	133.3223	64.84818	140.2241	64.82395	148.2841	65.03635	134.3856
64.838	136.5261	64.86102	129.9357	64.92407	133.3226	64.86485	140.2238	64.84062	148.2831	65.05302	134.387
64.85467	136.5284	64.87768	129.9356	64.94073	133.3209	64.88152	140.224	64.85728	148.2819	65.06968	134.383
64.87133	136.5277	64.89435	129.9334	64.9574	133.3249	64.89818	140.2233	64.87395	148.2817	65.08635	134.3826
64.888	136.5244	64.91102	129.9322	64.97407	133.3269	64.91485	140.2219	64.89062	148.2798	65.10302	134.3825
64.90467	136.5236	64.92768	129.9308	64.99073	133.3251	64.93152	140.2206	64.90728	148.2828	65.11968	134.3817
64.92133	136.5243	64.94435	129.9322	65.0074	133.3257	64.94818	140.2237	64.92395	148.2806	65.13635	134.3806
64.938	136.521	64.96102	129.9315	65.02407	133.3243	64.96485	140.223	64.94062	148.2778	65.15302	134.3792
64.95467	136.5219	64.97768	129.9289	65.04073	133.3285	64.98152	140.222	64.95728	148.2797	65.16968	134.3798
64.97133	136.5198	64.99435	129.9274	65.0574	133.3225	64.99818	140.2196	64.97395	148.2798	65.18635	134.379
64.988	136.5205	65.01102	129.9249	65.07407	133.3284	65.01485	140.2199	64.99062	148.2808	65.20302	134.3776
65.00467	136.5212	65.02768	129.9232	65.09073	133.3263	65.03152	140.2179	65.00728	148.2806	65.21968	134.3763
65.02133	136.5147	65.04435	129.9213	65.1074	133.3256	65.04818	140.2219	65.02395	148.2824	65.23635	134.3737
65.038	136.5168	65.06102	129.9216	65.12407	133.3303	65.06485	140.2193	65.04062	148.28	65.25302	134.3757
65.05467	136.5127	65.07768	129.9209	65.14073	133.3269	65.08152	140.2171	65.05728	148.2798	65.26968	134.377
65.07133	136.5143	65.09435	129.9189	65.1574	133.3294	65.09818	140.2153	65.07395	148.2832	65.28635	134.372
65.088	136.5123	65.11102	129.9176	65.17407	133.3258	65.11485	140.2168	65.09062	148.2841	65.30302	134.3694
65.10467	136.5117	65.12768	129.918	65.19073	133.3267	65.13152	140.2131	65.10728	148.2849	65.31968	134.3713
65.12133	136.5088	65.14435	129.9139	65.2074	133.3283	65.14818	140.215	65.12395	148.2814	65.33635	134.3711
65.138	136.5107	65.16102	129.9123	65.22407	133.3257	65.16485	140.214	65.14062	148.2834	65.35302	134.3683
65.15467	136.5106	65.17768	129.9115	65.24073	133.3241	65.18152	140.2136	65.15728	148.2814	65.36968	134.3632
65.17133	136.5086	65.19435	129.9098	65.2574	133.3267	65.19818	140.2124	65.17395	148.2858	65.38635	134.3653
65.188	136.5081	65.21102	129.9069	65.27407	133.3274	65.21485	140.2114	65.19062	148.2821	65.40302	134.3624
65.20467	136.5059	65.22768	129.9078	65.29073	133.326	65.23152	140.2115	65.20728	148.2819	65.41968	134.3611
65.22133	136.507	65.24435	129.9035	65.3074	133.3251	65.24818	140.2114	65.22395	148.2839	65.43635	134.3616
65.238	136.5046	65.26102	129.9019	65.32407	133.3265	65.26485	140.2083	65.24062	148.2835	65.45302	134.3603
65.25467	136.5042	65.27768	129.9037	65.34073	133.3267	65.28152	140.2077	65.25728	148.2839	65.46968	134.3572
65.27133	136.4977	65.29435	129.901	65.3574	133.3212	65.29818	140.2088	65.27395	148.2902	65.48635	134.3592
65.288	136.4976	65.31102	129.8991	65.37407	133.3264	65.31485	140.2087	65.29062	148.2873	65.50302	134.3597
65.30467	136.4994	65.32768	129.8993	65.39073	133.3275	65.33152	140.2055	65.30728	148.2846	65.51968	134.3537
65.32133	136.4975	65.34435	129.8988	65.4074	133.3267	65.34818	140.2075	65.32395	148.292	65.53635	134.3538
65.338	136.4935	65.36102	129.8947	65.42407	133.3261	65.36485	140.2051	65.34062	148.2871	65.55302	134.3497
65.35467	136.4971	65.37768	129.8946	65.44073	133.3247	65.38152	140.2067	65.35728	148.2906	65.56968	134.353
65.37133	136.4919	65.39435	129.8934	65.4574	133.3262	65.39818	140.2055	65.37395	148.2878	65.58635	134.3492
65.388	136.4894	65.41102	129.8912	65.47407	133.3237	65.41485	140.2059	65.39062	148.2907	65.60302	134.3462
65.40467	136.4913	65.42768	129.8848	65.49073	133.3219	65.43152	140.2058	65.40728	148.2837	65.61968	134.3457
65.42133	136.4923	65.44435	129.8862	65.5074	133.3244	65.44818	140.2026	65.42395	148.2891	65.63635	134.3461
65.438	136.4902	65.46102	129.884	65.52407	133.3233	65.46485	140.2015	65.44062	148.2915	65.65302	134.3453
65.45467	136.4889	65.47768	129.8858	65.54073	133.3235	65.48152	140.2058	65.45728	148.2891	65.66968	134.3433
65.47133	136.484	65.49435	129.8845	65.5574	133.3252	65.49818	140.202	65.47395	148.2897	65.68635	134.3407
65.488	136.4851	65.51102	129.8809	65.57407	133.3254	65.51485	140.2021	65.49062	148.2906	65.70302	134.3403
65.50467	136.4869	65.52768	129.8797	65.59073	133.3238	65.53152	140.2013	65.50728	148.29	65.71968	134.3422
65.52133	136.4862	65.54435	129.8798	65.6074	133.321	65.54818	140.2019	65.52395	148.2855	65.73635	134.341

65.538	136.4851	65.56102	129.8755	65.62407	133.3233	65.56485	140.2013	65.54062	148.2875	65.75302	134.3416
65.55467	136.4826	65.57768	129.8748	65.64073	133.3258	65.58152	140.2024	65.55728	148.2889	65.76968	134.3394
65.57133	136.481	65.59435	129.8733	65.6574	133.3253	65.59818	140.1999	65.57395	148.2904	65.78635	134.339
65.588	136.4792	65.61102	129.8735	65.67407	133.3241	65.61485	140.1988	65.59062	148.2886	65.80302	134.3367
65.60467	136.4785	65.62768	129.8712	65.69073	133.3248	65.63152	140.1975	65.60728	148.2942	65.81968	134.3365
65.62133	136.4757	65.64435	129.8721	65.7074	133.322	65.64818	140.1978	65.62395	148.2891	65.83635	134.3363
65.638	136.4751	65.66102	129.8679	65.72407	133.3215	65.66485	140.1964	65.64062	148.2902	65.85302	134.3367
65.65467	136.4739	65.67768	129.8648	65.74073	133.3216	65.68152	140.1974	65.65728	148.2891	65.86968	134.3352
65.67133	136.4727	65.69435	129.8657	65.7574	133.3219	65.69818	140.1966	65.67395	148.2926	65.88635	134.3352
65.688	136.4725	65.71102	129.8632	65.77407	133.324	65.71485	140.1965	65.69062	148.2911	65.90302	134.335
65.70467	136.4696	65.72768	129.8643	65.79073	133.3211	65.73152	140.1953	65.70728	148.2899	65.91968	134.3326
65.72133	136.4707	65.74435	129.862	65.8074	133.32	65.74818	140.1961	65.72395	148.293	65.93635	134.3322
65.738	136.4676	65.76102	129.8612	65.82407	133.3217	65.76485	140.1951	65.74062	148.2891	65.95302	134.3326
65.75467	136.4671	65.77768	129.8555	65.84073	133.3205	65.78152	140.1959	65.75728	148.2893	65.96968	134.331
65.77133	136.4645	65.79435	129.8572	65.8574	133.3218	65.79818	140.1929	65.77395	148.2881	65.98635	134.3299
65.788	136.4635	65.81102	129.8542	65.87407	133.3227	65.81485	140.1946	65.79062	148.29	66.00302	134.3312
65.80467	136.4612	65.82768	129.8527	65.89073	133.3224	65.83152	140.1965	65.80728	148.2859	66.01968	134.3285
65.82133	136.4624	65.84435	129.8499	65.9074	133.3202	65.84818	140.1943	65.82395	148.289	66.03635	134.3285
65.838	136.4576	65.86102	129.848	65.92407	133.32	65.86485	140.1933	65.84062	148.2901	66.05302	134.3255
65.85467	136.4589	65.87768	129.8472	65.94073	133.3185	65.88152	140.1941	65.85728	148.2907	66.06968	134.3246
65.87133	136.4572	65.89435	129.8459	65.9574	133.3197	65.89818	140.1921	65.87395	148.2902	66.08635	134.3247
65.888	136.4565	65.91102	129.8447	65.97407	133.3203	65.91485	140.1917	65.89062	148.292	66.10302	134.3249
65.90467	136.4561	65.92768	129.8429	65.99073	133.3167	65.93152	140.1942	65.90728	148.2897	66.11968	134.3242
65.92133	136.4565	65.94435	129.8422	66.0074	133.3174	65.94818	140.1927	65.92395	148.2899	66.13635	134.3231
65.938	136.4525	65.96102	129.8393	66.02407	133.3169	65.96485	140.1905	65.94062	148.2907	66.15302	134.3226
65.95467	136.4546	65.97768	129.8362	66.04073	133.3186	65.98152	140.1898	65.95728	148.2931	66.16968	134.3232
65.97133	136.4528	65.99435	129.8349	66.0574	133.3151	65.99818	140.1892	65.97395	148.2901	66.18635	134.3236
65.988	136.4519	66.01102	129.8334	66.07407	133.3151	66.01485	140.1933	65.99062	148.2907	66.20302	134.3214
66.00467	136.4471	66.02768	129.8331	66.09073	133.3191	66.03152	140.188	66.00728	148.2897	66.21968	134.3199
66.02133	136.4485	66.04435	129.8294	66.1074	133.3171	66.04818	140.1879	66.02395	148.2928	66.23635	134.32
66.038	136.4472	66.06102	129.8302	66.12407	133.3125	66.06485	140.1888	66.04062	148.2916	66.25302	134.3196
66.05467	136.4463	66.07768	129.8291	66.14073	133.317	66.08152	140.186	66.05728	148.2942	66.26968	134.3176
66.07133	136.4476	66.09435	129.8267	66.1574	133.3169	66.09818	140.1881	66.07395	148.2931	66.28635	134.3169
66.088	136.4403	66.11102	129.8259	66.17407	133.3168	66.11485	140.1882	66.09062	148.2952	66.30302	134.3181
66.10467	136.4408	66.12768	129.8264	66.19073	133.3138	66.13152	140.1875	66.10728	148.2931	66.31968	134.3172
66.12133	136.4395	66.14435	129.8225	66.2074	133.3163	66.14818	140.1894	66.12395	148.2949	66.33635	134.315
66.138	136.4397	66.16102	129.8202	66.22407	133.312	66.16485	140.1847	66.14062	148.2927	66.35302	134.3166
66.15467	136.4375	66.17768	129.8181	66.24073	133.3141	66.18152	140.1846	66.15728	148.2938	66.36968	134.3146
66.17133	136.4375	66.19435	129.8221	66.2574	133.3097	66.19818	140.1866	66.17395	148.2943	66.38635	134.3135
66.188	136.4355	66.21102	129.8162	66.27407	133.3126	66.21485	140.185	66.19062	148.2938	66.40302	134.3122
66.20467	136.432	66.22768	129.8152	66.29073	133.3117	66.23152	140.1853	66.20728	148.2947	66.41968	134.3123
66.22133	136.4325	66.24435	129.8148	66.3074	133.3149	66.24818	140.1859	66.22395	148.2969	66.43635	134.3118
66.238	136.4312	66.26102	129.8131	66.32407	133.3118	66.26485	140.1823	66.24062	148.2963	66.45302	134.3102
66.25467	136.4302	66.27768	129.812	66.34073	133.3114	66.28152	140.1851	66.25728	148.2952	66.46968	134.3095
66.27133	136.4306	66.29435	129.8084	66.3574	133.3093	66.29818	140.1834	66.27395	148.2972	66.48635	134.3101
66.288	136.4253	66.31102	129.8091	66.37407	133.3083	66.31485	140.1819	66.29062	148.2961	66.50302	134.3099
66.30467	136.4255	66.32768	129.8099	66.39073	133.3113	66.33152	140.1848	66.30728	148.2959	66.51968	134.3063
66.32133	136.4249	66.34435	129.8085	66.4074	133.3117	66.34818	140.1824	66.32395	148.298	66.53635	134.3056
66.338	136.4234	66.36102	129.8042	66.42407	133.3099	66.36485	140.1846	66.34062	148.2954	66.55302	134.3063
66.35467	136.4237	66.37768	129.8036	66.44073	133.3115	66.38152	140.1834	66.35728	148.2991	66.56968	134.3051
66.37133	136.4219	66.39435	129.8021	66.4574	133.31	66.39818	140.182	66.37395	148.3005	66.58635	134.3037
66.388	136.4182	66.41102	129.7971	66.47407	133.3087	66.41485	140.1815	66.39062	148.3001	66.60302	134.3038
66.40467	136.4183	66.42768	129.7972	66.49073	133.3092	66.43152	140.1829	66.40728	148.3	66.61968	134.303
66.42133	136.42	66.44435	129.7976	66.5074	133.3115	66.44818	140.1805	66.42395	148.3002	66.63635	134.3019
66.438	136.4187	66.46102	129.7935	66.52407	133.3125	66.46485	140.181	66.44062	148.3011	66.65302	134.2999
66.45467	136.4182	66.47768	129.7894	66.54073	133.3098	66.48152	140.1798	66.45728	148.2984	66.66968	134.3029
66.47133	136.414	66.49435	129.7905	66.5574	133.3106	66.49818	140.1804	66.47395	148.2991	66.68635	134.3032
66.488	136.4145	66.51102	129.7887	66.57407	133.31	66.51485	140.1805	66.49062	148.303	66.70302	134.2998
66.50467	136.4156	66.52768	129.7889	66.59073	133.3115	66.53152	140.179	66.50728	148.2988	66.71968	134.2965
66.52133	136.4126	66.54435	129.7858	66.6074	133.3088	66.54818	140.1772	66.52395	148.2999	66.73635	134.3007
66.538	136.4125	66.56102	129.785	66.62407	133.3114	66.56485	140.177	66.54062	148.3009	66.75302	134.2975
66.55467	136.4104	66.57768	129.7846	66.64073	133.3125	66.58152	140.1776	66.55728	148.302	66.76968	134.2975
66.57133	136.4092	66.59435	129.782	66.6574	133.313	66.59818	140.1772	66.57395	148.3003	66.78635	134.2961
66.588	136.4077	66.61102	129.7797	66.67407	133.3121	66.61485	140.1752	66.59062	148.3033	66.80302	134.2953
66.60467	136.4065	66.62768	129.7785	66.69073	133.3107	66.63152	140.175	66.60728	148.3006	66.81968	134.295
66.62133	136.4093	66.64435	129.778	66.7074	133.31	66.64818	140.1747	66.62395	148.3	66.83635	134.2938
66.638	136.4053	66.66102	129.7762	66.72407	133.309	66.66485	140.1739	66.64062	148.3023	66.85302	134.2932
66.65467	136.4038	66.67768	129.7766	66.74073	133.3108	66.68152	140.1739	66.65728	148.302	66.86968	134.2913
66.67133	136.4025	66.69435	129.7767	66.7574	133.3114	66.69818	140.1728	66.67395	148.2988	66.88635	134.2889

66.688	136.4018	66.71102	129.7736	66.77407	133.3106	66.71485	140.1721	66.69062	148.3018	66.90302	134.2884
66.70467	136.4031	66.72768	129.7719	66.79073	133.3082	66.73152	140.1711	66.70728	148.3023	66.91968	134.2891
66.72133	136.399	66.74435	129.7705	66.8074	133.309	66.74818	140.1714	66.72395	148.302	66.93635	134.2885
66.738	136.3979	66.76102	129.7681	66.82407	133.3106	66.76485	140.1701	66.74062	148.2992	66.95302	134.2885
66.75467	136.3954	66.77768	129.7675	66.84073	133.3085	66.78152	140.1732	66.75728	148.3038	66.96968	134.2861
66.77133	136.3974	66.79435	129.7649	66.8574	133.3089	66.79818	140.1728	66.77395	148.3008	66.98635	134.2852
66.788	136.397	66.81102	129.7701	66.87407	133.3102	66.81485	140.1724	66.79062	148.3018	67.00302	134.285
66.80467	136.3963	66.82768	129.7654	66.89073	133.3092	66.83152	140.1713	66.80728	148.3	67.01968	134.2839
66.82133	136.3911	66.84435	129.7634	66.9074	133.3086	66.84818	140.17	66.82395	148.3041	67.03635	134.2857
66.838	136.3914	66.86102	129.761	66.92407	133.3075	66.86485	140.1718	66.84062	148.3002	67.05302	134.2826
66.85467	136.3892	66.87768	129.7578	66.94073	133.3114	66.88152	140.171	66.85728	148.3018	67.06968	134.283
66.87133	136.3884	66.89435	129.7545	66.9574	133.3094	66.89818	140.1703	66.87395	148.3059	67.08635	134.2808
66.888	136.3875	66.91102	129.7551	66.97407	133.3084	66.91485	140.1727	66.89062	148.3031	67.10302	134.282
66.90467	136.3847	66.92768	129.7548	66.99073	133.308	66.93152	140.1666	66.90728	148.3002	67.11968	134.2788
66.92133	136.3831	66.94435	129.7539	67.0074	133.3078	66.94818	140.1658	66.92395	148.3019	67.13635	134.2769
66.938	136.3823	66.96102	129.7554	67.02407	133.3081	66.96485	140.1677	66.94062	148.3002	67.15302	134.2758
66.95467	136.3817	66.97768	129.7503	67.04073	133.3057	66.98152	140.1673	66.95728	148.3024	67.16968	134.2809
66.97133	136.382	66.99435	129.7515	67.0574	133.3069	66.99818	140.1658	66.97395	148.3048	67.18635	134.2746
66.988	136.3793	67.01102	129.7467	67.07407	133.309	67.01485	140.167	66.99062	148.3033	67.20302	134.2778
67.00467	136.3775	67.02768	129.7462	67.09073	133.3049	67.03152	140.1648	67.00728	148.3028	67.21968	134.2752
67.02133	136.3765	67.04435	129.745	67.1074	133.3059	67.04818	140.1643	67.02395	148.3032	67.23635	134.2744
67.038	136.3756	67.06102	129.7429	67.12407	133.3058	67.06485	140.1625	67.04062	148.3018	67.25302	134.2735
67.05467	136.3743	67.07768	129.7412	67.14073	133.3047	67.08152	140.1645	67.05728	148.3005	67.26968	134.2736
67.07133	136.3764	67.09435	129.7409	67.1574	133.3042	67.09818	140.1656	67.07395	148.3044	67.28635	134.2721
67.088	136.3731	67.11102	129.7407	67.17407	133.3028	67.11485	140.1627	67.09062	148.3036	67.30302	134.2713
67.10467	136.3694	67.12768	129.7396	67.19073	133.3025	67.13152	140.1625	67.10728	148.3074	67.31968	134.2709
67.12133	136.3702	67.14435	129.7386	67.2074	133.3003	67.14818	140.1604	67.12395	148.3058	67.33635	134.272
67.138	136.3693	67.16102	129.74	67.22407	133.3015	67.16485	140.1628	67.14062	148.3075	67.35302	134.2688
67.15467	136.3688	67.17768	129.7367	67.24073	133.3006	67.18152	140.1623	67.15728	148.3098	67.36968	134.2681
67.17133	136.3653	67.19435	129.7343	67.2574	133.3019	67.19818	140.1626	67.17395	148.305	67.38635	134.2689
67.188	136.3661	67.21102	129.7337	67.27407	133.3021	67.21485	140.1601	67.19062	148.3083	67.40302	134.2668
67.20467	136.3641	67.22768	129.734	67.29073	133.3043	67.23152	140.1591	67.20728	148.3059	67.41968	134.2651
67.22133	136.3635	67.24435	129.7306	67.3074	133.2988	67.24818	140.1583	67.22395	148.3085	67.43635	134.2652
67.238	136.362	67.26102	129.7281	67.32407	133.2985	67.26485	140.1598	67.24062	148.3077	67.45302	134.2637
67.25467	136.3598	67.27768	129.7292	67.34073	133.2994	67.28152	140.1596	67.25728	148.3077	67.46968	134.2623
67.27133	136.3569	67.29435	129.7246	67.3574	133.2979	67.29818	140.1599	67.27395	148.3098	67.48635	134.2628
67.288	136.3603	67.31102	129.7256	67.37407	133.2971	67.31485	140.1568	67.29062	148.3072	67.50302	134.2617
67.30467	136.3581	67.32768	129.7246	67.39073	133.2971	67.33152	140.1572	67.30728	148.3114	67.51968	134.2596
67.32133	136.3554	67.34435	129.721	67.4074	133.2975	67.34818	140.1586	67.32395	148.3097	67.53635	134.2581
67.338	136.357	67.36102	129.7183	67.42407	133.2949	67.36485	140.1612	67.34062	148.3087	67.55302	134.2591
67.35467	136.352	67.37768	129.7193	67.44073	133.296	67.38152	140.1559	67.35728	148.3088	67.56968	134.2574
67.37133	136.3544	67.39435	129.7183	67.4574	133.2958	67.39818	140.1538	67.37395	148.3077	67.58635	134.258
67.388	136.3539	67.41102	129.7173	67.47407	133.2944	67.41485	140.1532	67.39062	148.3107	67.60302	134.2581
67.40467	136.3469	67.42768	129.7142	67.49073	133.2956	67.43152	140.1545	67.40728	148.3078	67.61968	134.2571
67.42133	136.351	67.44435	129.7144	67.5074	133.2931	67.44818	140.1541	67.42395	148.3084	67.63635	134.2555
67.438	136.3477	67.46102	129.7137	67.52407	133.2913	67.46485	140.155	67.44062	148.3092	67.65302	134.2537
67.45467	136.3481	67.47768	129.7122	67.54073	133.289	67.48152	140.1533	67.45728	148.3097	67.66968	134.2542
67.47133	136.3445	67.49435	129.709	67.5574	133.2911	67.49818	140.1514	67.47395	148.3104	67.68635	134.254
67.488	136.3453	67.51102	129.7079	67.57407	133.2896	67.51485	140.1526	67.49062	148.3123	67.70302	134.2542
67.50467	136.3439	67.52768	129.7045	67.59073	133.2879	67.53152	140.1534	67.50728	148.3126	67.71968	134.2507
67.52133	136.339	67.54435	129.7091	67.6074	133.2868	67.54818	140.1508	67.52395	148.312	67.73635	134.2505
67.538	136.3407	67.56102	129.705	67.62407	133.2873	67.56485	140.1488	67.54062	148.3126	67.75302	134.2499
67.55467	136.3423	67.57768	129.7025	67.64073	133.2851	67.58152	140.1489	67.55728	148.3112	67.76968	134.2515
67.57133	136.3406	67.59435	129.703	67.6574	133.2852	67.59818	140.148	67.57395	148.3106	67.78635	134.2472
67.588	136.3394	67.61102	129.6997	67.67407	133.2894	67.61485	140.1468	67.59062	148.3113	67.80302	134.2488
67.60467	136.3403	67.62768	129.6977	67.69073	133.2864	67.63152	140.1492	67.60728	148.312	67.81968	134.2466
67.62133	136.3371	67.64435	129.6978	67.7074	133.2848	67.64818	140.1444	67.62395	148.3117	67.83635	134.2455
67.638	136.3361	67.66102	129.6967	67.72407	133.2827	67.66485	140.1464	67.64062	148.311	67.85302	134.2428
67.65467	136.3336	67.67768	129.6931	67.74073	133.2815	67.68152	140.147	67.65728	148.3119	67.86968	134.2427
67.67133	136.3339	67.69435	129.6904	67.7574	133.2843	67.69818	140.1436	67.67395	148.3106	67.88635	134.2421
67.688	136.337	67.71102	129.6905	67.77407	133.2856	67.71485	140.1433	67.69062	148.3126	67.90302	134.2405
67.70467	136.3331	67.72768	129.689	67.79073	133.2827	67.73152	140.1439	67.70728	148.312	67.91968	134.2378
67.72133	136.3273	67.74435	129.6886	67.8074	133.2802	67.74818	140.1434	67.72395	148.3137	67.93635	134.2393
67.738	136.3288	67.76102	129.6847	67.82407	133.2798	67.76485	140.1439	67.74062	148.3113	67.95302	134.2372
67.75467	136.328	67.77768	129.6845	67.84073	133.2807	67.78152	140.1406	67.75728	148.3144	67.96968	134.236
67.77133	136.326	67.79435	129.6836	67.8574	133.279	67.79818	140.1435	67.77395	148.315	67.98635	134.2349
67.788	136.3271	67.81102	129.6817	67.87407	133.2795	67.81485	140.1433	67.79062	148.3099	68.00302	134.2373
67.80467	136.3208	67.82768	129.6805	67.89073	133.2765	67.83152	140.1416	67.80728	148.314	68.01968	134.2321
67.82133	136.3236	67.84435	129.6805	67.9074	133.2762	67.84818	140.1424	67.82395	148.3117	68.03635	134.2326

67.838	136.3211	67.86102	129.6784	67.92407	133.275	67.86485	140.1402	67.84062	148.3138	68.05302	134.2299
67.85467	136.3248	67.87768	129.6787	67.94073	133.2735	67.88152	140.1391	67.85728	148.3151	68.06968	134.23
67.87133	136.3202	67.89435	129.6791	67.9574	133.274	67.89818	140.1421	67.87395	148.3114	68.08635	134.2303
67.888	136.3218	67.91102	129.6766	67.97407	133.2753	67.91485	140.1396	67.89062	148.3128	68.10302	134.2274
67.90467	136.3205	67.92768	129.6737	67.99073	133.2721	67.93152	140.1401	67.90728	148.3139	68.11968	134.2283
67.92133	136.3199	67.94435	129.6725	68.0074	133.273	67.94818	140.1418	67.92395	148.318	68.13635	134.2254
67.938	136.3191	67.96102	129.6717	68.02407	133.2713	67.96485	140.1391	67.94062	148.3152	68.15302	134.2236
67.95467	136.3155	67.97768	129.6727	68.04073	133.2702	67.98152	140.1374	67.95728	148.318	68.16968	134.2228
67.97133	136.3153	67.99435	129.6691	68.0574	133.2695	67.99818	140.1401	67.97395	148.3148	68.18635	134.2241
67.988	136.3156	68.01102	129.6692	68.07407	133.2672	68.01485	140.1416	67.99062	148.3146	68.20302	134.2219
68.00467	136.3146	68.02768	129.6666	68.09073	133.2676	68.03152	140.1399	68.00728	148.3154	68.21968	134.2238
68.02133	136.3105	68.04435	129.6663	68.1074	133.2689	68.04818	140.1396	68.02395	148.3149	68.23635	134.2213
68.038	136.3104	68.06102	129.6657	68.12407	133.2662	68.06485	140.1368	68.04062	148.3145	68.25302	134.2203
68.05467	136.3109	68.07768	129.663	68.14073	133.2658	68.08152	140.1384	68.05728	148.3148	68.26968	134.2209
68.07133	136.3118	68.09435	129.6619	68.1574	133.2671	68.09818	140.1399	68.07395	148.3162	68.28635	134.2212
68.088	136.3084	68.11102	129.6627	68.17407	133.2631	68.11485	140.137	68.09062	148.3142	68.30302	134.2196
68.10467	136.3106	68.12768	129.6604	68.19073	133.264	68.13152	140.1389	68.10728	148.3144	68.31968	134.2172
68.12133	136.3101	68.14435	129.6595	68.2074	133.2655	68.14818	140.1375	68.12395	148.3145	68.33635	134.2177
68.138	136.3048	68.16102	129.6595	68.22407	133.266	68.16485	140.1367	68.14062	148.3154	68.35302	134.2151
68.15467	136.3071	68.17768	129.6569	68.24073	133.2629	68.18152	140.1375	68.15728	148.3161	68.36968	134.2168
68.17133	136.3029	68.19435	129.6564	68.2574	133.2639	68.19818	140.1365	68.17395	148.3164	68.38635	134.2138
68.188	136.3024	68.21102	129.6582	68.27407	133.2648	68.21485	140.1373	68.19062	148.3192	68.40302	134.2121
68.20467	136.3016	68.22768	129.6538	68.29073	133.2645	68.23152	140.1358	68.20728	148.3159	68.41968	134.2146
68.22133	136.2999	68.24435	129.6535	68.3074	133.2654	68.24818	140.1343	68.22395	148.318	68.43635	134.2137
68.238	136.3023	68.26102	129.6514	68.32407	133.2636	68.26485	140.1373	68.24062	148.3194	68.45302	134.2112
68.25467	136.2992	68.27768	129.6502	68.34073	133.261	68.28152	140.1342	68.25728	148.319	68.46968	134.2119
68.27133	136.297	68.29435	129.6477	68.3574	133.2604	68.29818	140.1354	68.27395	148.3194	68.48635	134.21
68.288	136.2975	68.31102	129.6486	68.37407	133.2638	68.31485	140.1335	68.29062	148.3187	68.50302	134.212
68.30467	136.2967	68.32768	129.6447	68.39073	133.2626	68.33152	140.1312	68.30728	148.3188	68.51968	134.2115
68.32133	136.2974	68.34435	129.6459	68.4074	133.2621	68.34818	140.1316	68.32395	148.3209	68.53635	134.2104
68.338	136.2918	68.36102	129.6434	68.42407	133.2626	68.36485	140.1321	68.34062	148.3188	68.55302	134.2091
68.35467	136.2955	68.37768	129.6439	68.44073	133.2614	68.38152	140.1331	68.35728	148.3193	68.56968	134.2107
68.37133	136.2919	68.39435	129.643	68.4574	133.2611	68.39818	140.1326	68.37395	148.3194	68.58635	134.2087
68.388	136.2931	68.41102	129.642	68.47407	133.2627	68.41485	140.1311	68.39062	148.3186	68.60302	134.2062
68.40467	136.2914	68.42768	129.6392	68.49073	133.258	68.43152	140.1315	68.40728	148.3193	68.61968	134.2064
68.42133	136.2877	68.44435	129.6381	68.5074	133.2628	68.44818	140.1309	68.42395	148.3215	68.63635	134.2072
68.438	136.2885	68.46102	129.6354	68.52407	133.2626	68.46485	140.1321	68.44062	148.3209	68.65302	134.2039
68.45467	136.2894	68.47768	129.6333	68.54073	133.263	68.48152	140.1301	68.45728	148.3191	68.66968	134.2044
68.47133	136.2871	68.49435	129.637	68.5574	133.2624	68.49818	140.1308	68.47395	148.3203	68.68635	134.2053
68.488	136.2863	68.51102	129.6329	68.57407	133.2625	68.51485	140.1274	68.49062	148.3189	68.70302	134.2042
68.50467	136.2866	68.52768	129.631	68.59073	133.2614	68.53152	140.13	68.50728	148.3206	68.71968	134.2042
68.52133	136.2859	68.54435	129.6302	68.6074	133.2638	68.54818	140.1296	68.52395	148.3184	68.73635	134.2024
68.538	136.2838	68.56102	129.6269	68.62407	133.2617	68.56485	140.1276	68.54062	148.3191	68.75302	134.2003
68.55467	136.2835	68.57768	129.6272	68.64073	133.2621	68.58152	140.1253	68.55728	148.3196	68.76968	134.2004
68.57133	136.2809	68.59435	129.6244	68.6574	133.2602	68.59818	140.1242	68.57395	148.3179	68.78635	134.1998
68.588	136.2792	68.61102	129.6234	68.67407	133.2613	68.61485	140.1249	68.59062	148.3219	68.80302	134.1995
68.60467	136.2769	68.62768	129.6222	68.69073	133.2609	68.63152	140.1279	68.60728	148.3169	68.81968	134.1984
68.62133	136.2775	68.64435	129.6241	68.7074	133.2601	68.64818	140.1237	68.62395	148.3192	68.83635	134.1941
68.638	136.2766	68.66102	129.6187	68.72407	133.2607	68.66485	140.1224	68.64062	148.3221	68.85302	134.1964
68.65467	136.275	68.67768	129.6196	68.74073	133.2609	68.68152	140.1224	68.65728	148.3138	68.86968	134.1974
68.67133	136.2734	68.69435	129.6203	68.7574	133.2608	68.69818	140.124	68.67395	148.3218	68.88635	134.1943
68.688	136.2731	68.71102	129.6162	68.77407	133.2582	68.71485	140.1212	68.69062	148.322	68.90302	134.1942
68.70467	136.2719	68.72768	129.6142	68.79073	133.2584	68.73152	140.1231	68.70728	148.3171	68.91968	134.1914
68.72133	136.2724	68.74435	129.6163	68.8074	133.2603	68.74818	140.1217	68.72395	148.3231	68.93635	134.193
68.738	136.2696	68.76102	129.6117	68.82407	133.2596	68.76485	140.1202	68.74062	148.3179	68.95302	134.191
68.75467	136.2698	68.77768	129.6105	68.84073	133.2566	68.78152	140.1228	68.75728	148.3227	68.96968	134.19
68.77133	136.2677	68.79435	129.6117	68.8574	133.2565	68.79818	140.1202	68.77395	148.3214	68.98635	134.1874
68.788	136.2649	68.81102	129.6091	68.87407	133.2559	68.81485	140.1187	68.79062	148.3213	69.00302	134.186
68.80467	136.2666	68.82768	129.6064	68.89073	133.2553	68.83152	140.1201	68.80728	148.3206	69.01968	134.1867
68.82133	136.2618	68.84435	129.6059	68.9074	133.256	68.84818	140.1195	68.82395	148.3215	69.03635	134.1867
68.838	136.2641	68.86102	129.6035	68.92407	133.2522	68.86485	140.1184	68.84062	148.3204	69.05302	134.1841
68.85467	136.2573	68.87768	129.6039	68.94073	133.2554	68.88152	140.1192	68.85728	148.3215	69.06968	134.1801
68.87133	136.2563	68.89435	129.6033	68.9574	133.2536	68.89818	140.1168	68.87395	148.3207	69.08635	134.182
68.888	136.2592	68.91102	129.6004	68.97407	133.2536	68.91485	140.1182	68.89062	148.3209	69.10302	134.1807
68.90467	136.2565	68.92768	129.5997	68.99073	133.2534	68.93152	140.1162	68.90728	148.3212	69.11968	134.1798
68.92133	136.2546	68.94435	129.5988	69.0074	133.2538	68.94818	140.117	68.92395	148.3205	69.13635	134.1817
68.938	136.254	68.96102	129.5984	69.02407	133.254	68.96485	140.1178	68.94062	148.3199	69.15302	134.179
68.95467	136.2552	68.97768	129.597	69.04073	133.2509	68.98152	140.1153	68.95728	148.3202	69.16968	134.179
68.97133	136.2486	68.99435	129.5951	69.0574	133.253	68.99818	140.1158	68.97395	148.3201	69.18635	134.1811

68.988	136.2482	69.01102	129.5957	69.07407	133.2528	69.01485	140.1143	68.99062	148.319	69.20302	134.1779
69.00467	136.2478	69.02768	129.5948	69.09073	133.2508	69.03152	140.1144	69.00728	148.3228	69.21968	134.1723
69.02133	136.2491	69.04435	129.5949	69.1074	133.2512	69.04818	140.1124	69.02395	148.3203	69.23635	134.1768
69.038	136.248	69.06102	129.5936	69.12407	133.2475	69.06485	140.1117	69.04062	148.3253	69.25302	134.1778
69.05467	136.2447	69.07768	129.5913	69.14073	133.2481	69.08152	140.1118	69.05728	148.3209	69.26968	134.1774
69.07133	136.2413	69.09435	129.5879	69.1574	133.248	69.09818	140.1123	69.07395	148.326	69.28635	134.175
69.088	136.2427	69.11102	129.5896	69.17407	133.2465	69.11485	140.1092	69.09062	148.3224	69.30302	134.1734
69.10467	136.2413	69.12768	129.5876	69.19073	133.2469	69.13152	140.1109	69.10728	148.3202	69.31968	134.1755
69.12133	136.2405	69.14435	129.586	69.2074	133.2471	69.14818	140.1108	69.12395	148.3226	69.33635	134.1738
69.138	136.238	69.16102	129.5859	69.22407	133.2476	69.16485	140.1106	69.14062	148.3212	69.35302	134.1715
69.15467	136.2309	69.17768	129.5823	69.24073	133.2467	69.18152	140.1093	69.15728	148.321	69.36968	134.1702
69.17133	136.2363	69.19435	129.5815	69.2574	133.2443	69.19818	140.1101	69.17395	148.322	69.38635	134.1727
69.188	136.2325	69.21102	129.5787	69.27407	133.2452	69.21485	140.1072	69.19062	148.3212	69.40302	134.172
69.20467	136.233	69.22768	129.5773	69.29073	133.2443	69.23152	140.1065	69.20728	148.3241	69.41968	134.1667
69.22133	136.232	69.24435	129.5748	69.3074	133.2412	69.24818	140.1067	69.22395	148.3193	69.43635	134.1673
69.238	136.2302	69.26102	129.5764	69.32407	133.2414	69.26485	140.1069	69.24062	148.3215	69.45302	134.166
69.25467	136.2283	69.27768	129.5775	69.34073	133.2408	69.28152	140.1071	69.25728	148.3213	69.46968	134.1676
69.27133	136.2289	69.29435	129.5728	69.3574	133.2408	69.29818	140.1057	69.27395	148.3207	69.48635	134.1643
69.288	136.2289	69.31102	129.5718	69.37407	133.2414	69.31485	140.1027	69.29062	148.3215	69.50302	134.1609
69.30467	136.224	69.32768	129.5724	69.39073	133.2379	69.33152	140.1053	69.30728	148.3213	69.51968	134.1592
69.32133	136.2277	69.34435	129.5711	69.4074	133.2403	69.34818	140.1021	69.32395	148.3229	69.53635	134.1624
69.338	136.2236	69.36102	129.5692	69.42407	133.2386	69.36485	140.102	69.34062	148.3196	69.55302	134.1592
69.35467	136.2206	69.37768	129.5684	69.44073	133.2394	69.38152	140.1007	69.35728	148.3202	69.56968	134.1568
69.37133	136.2195	69.39435	129.565	69.4574	133.2382	69.39818	140.0999	69.37395	148.3237	69.58635	134.156
69.388	136.2185	69.41102	129.5658	69.47407	133.2374	69.41485	140.0992	69.39062	148.325	69.60302	134.1533
69.40467	136.2166	69.42768	129.5644	69.49073	133.2368	69.43152	140.1001	69.40728	148.3204	69.61968	134.1536
69.42133	136.2141	69.44435	129.5621	69.5074	133.2369	69.44818	140.0996	69.42395	148.3256	69.63635	134.1546
69.438	136.2129	69.46102	129.5614	69.52407	133.2327	69.46485	140.1015	69.44062	148.3249	69.65302	134.1537
69.45467	136.212	69.47768	129.5612	69.54073	133.2345	69.48152	140.1009	69.45728	148.3244	69.66968	134.1508
69.47133	136.2108	69.49435	129.5568	69.5574	133.2335	69.49818	140.0993	69.47395	148.3242	69.68635	134.1516
69.488	136.2112	69.51102	129.5609	69.57407	133.2325	69.51485	140.099	69.49062	148.3273	69.70302	134.15
69.50467	136.21	69.52768	129.5549	69.59073	133.2333	69.53152	140.1005	69.50728	148.3258	69.71968	134.1508
69.52133	136.2082	69.54435	129.5545	69.6074	133.2332	69.54818	140.1008	69.52395	148.3273	69.73635	134.1483
69.538	136.2066	69.56102	129.5543	69.62407	133.2284	69.56485	140.1011	69.54062	148.3251	69.75302	134.147
69.55467	136.2035	69.57768	129.5518	69.64073	133.2294	69.58152	140.1002	69.55728	148.3275	69.76968	134.1491
69.57133	136.2038	69.59435	129.5506	69.6574	133.2317	69.59818	140.0977	69.57395	148.3265	69.78635	134.1483
69.588	136.2018	69.61102	129.5488	69.67407	133.2286	69.61485	140.0986	69.59062	148.3274	69.80302	134.1471
69.60467	136.2001	69.62768	129.5496	69.69073	133.2268	69.63152	140.0993	69.60728	148.3263	69.81968	134.1434
69.62133	136.2013	69.64435	129.5487	69.7074	133.2269	69.64818	140.0971	69.62395	148.3294	69.83635	134.1463
69.638	136.2013	69.66102	129.5462	69.72407	133.2257	69.66485	140.0965	69.64062	148.3285	69.85302	134.1442
69.65467	136.2012	69.67768	129.545	69.74073	133.228	69.68152	140.0974	69.65728	148.3254	69.86968	134.1439
69.67133	136.1962	69.69435	129.5418	69.7574	133.2236	69.69818	140.0981	69.67395	148.327	69.88635	134.1432
69.688	136.1988	69.71102	129.5399	69.77407	133.2272	69.71485	140.0991	69.69062	148.3267	69.90302	134.1427
69.70467	136.1966	69.72768	129.5399	69.79073	133.2247	69.73152	140.0985	69.70728	148.3248	69.91968	134.1405
69.72133	136.1946	69.74435	129.5391	69.8074	133.2257	69.74818	140.0973	69.72395	148.3281	69.93635	134.142
69.738	136.1912	69.76102	129.5357	69.82407	133.2232	69.76485	140.0975	69.74062	148.3254	69.95302	134.1411
69.75467	136.1942	69.77768	129.5355	69.84073	133.2255	69.78152	140.0957	69.75728	148.3256	69.96968	134.1404
69.77133	136.1915	69.79435	129.5325	69.8574	133.2251	69.79818	140.0966	69.77395	148.3247	69.98635	134.1402
69.788	136.1921	69.81102	129.5346	69.87407	133.2256	69.81485	140.0967	69.79062	148.3288	70.00302	134.1359
69.80467	136.1872	69.82768	129.5308	69.89073	133.2252	69.83152	140.0971	69.80728	148.3283	70.01968	134.1386
69.82133	136.1894	69.84435	129.5298	69.9074	133.2262	69.84818	140.0935	69.82395	148.3289	70.03635	134.1366
69.838	136.1876	69.86102	129.5301	69.92407	133.2249	69.86485	140.0939	69.84062	148.3288	70.05302	134.1335
69.85467	136.1901	69.87768	129.527	69.94073	133.2243	69.88152	140.0949	69.85728	148.3285	70.06968	134.134
69.87133	136.1842	69.89435	129.527	69.9574	133.2255	69.89818	140.0957	69.87395	148.329	70.08635	134.1344
69.888	136.1832	69.91102	129.5242	69.97407	133.2251	69.91485	140.0948	69.89062	148.3274	70.10302	134.1343
69.90467	136.1808	69.92768	129.5217	69.99073	133.2242	69.93152	140.0966	69.90728	148.3294	70.11968	134.1309
69.92133	136.181	69.94435	129.5206	70.0074	133.2232	69.94818	140.0958	69.92395	148.3251	70.13635	134.1331
69.938	136.181	69.96102	129.5215	70.02407	133.2233	69.96485	140.0933	69.94062	148.33	70.15302	134.1296
69.95467	136.1793	69.97768	129.5212	70.04073	133.2237	69.98152	140.0947	69.95728	148.3286	70.16968	134.1287
69.97133	136.1767	69.99435	129.5209	70.0574	133.2233	69.99818	140.0957	69.97395	148.326	70.18635	134.1277
69.988	136.1769	70.01102	129.5205	70.07407	133.2222	70.01485	140.0933	69.99062	148.3309	70.20302	134.1287
70.00467	136.1742	70.02768	129.5176	70.09073	133.221	70.03152	140.0929	70.00728	148.3244	70.21968	134.1282
70.02133	136.1712	70.04435	129.518	70.1074	133.2231	70.04818	140.0944	70.02395	148.3311	70.23635	134.1262
70.038	136.1728	70.06102	129.5156	70.12407	133.2211	70.06485	140.0953	70.04062	148.3235	70.25302	134.1239
70.05467	136.1713	70.07768	129.5137	70.14073	133.2224	70.08152	140.0937	70.05728	148.3263	70.26968	134.1251
70.07133	136.1701	70.09435	129.5146	70.1574	133.2202	70.09818	140.0929	70.07395	148.3302	70.28635	134.1239
70.088	136.1712	70.11102	129.5154	70.17407	133.2207	70.11485	140.0917	70.09062	148.323	70.30302	134.123
70.10467	136.1689	70.12768	129.5108	70.19073	133.2216	70.13152	140.0937	70.10728	148.33	70.31968	134.1226
70.12133	136.1659	70.14435	129.5119	70.2074	133.219	70.14818	140.0908	70.12395	148.3274	70.33635	134.1229

70.138	136.166	70.16102	129.5106	70.22407	133.2174	70.16485	140.0905	70.14062	148.3271	70.35302	134.1223
70.15467	136.1658	70.17768	129.5106	70.24073	133.2153	70.18152	140.0904	70.15728	148.328	70.36968	134.1198
70.17133	136.1632	70.19435	129.5046	70.2574	133.2144	70.19818	140.09	70.17395	148.3291	70.38635	134.1204
70.188	136.163	70.21102	129.5059	70.27407	133.2167	70.21485	140.0887	70.19062	148.3253	70.40302	134.1204
70.20467	136.162	70.22768	129.5058	70.29073	133.2135	70.23152	140.0889	70.20728	148.326	70.41968	134.1177
70.22133	136.1596	70.24435	129.5062	70.3074	133.2128	70.24818	140.0878	70.22395	148.3287	70.43635	134.1189
70.238	136.1588	70.26102	129.5027	70.32407	133.2133	70.26485	140.0884	70.24062	148.3278	70.45302	134.1182
70.25467	136.1562	70.27768	129.5048	70.34073	133.2114	70.28152	140.089	70.25728	148.3275	70.46968	134.1202
70.27133	136.1583	70.29435	129.5001	70.3574	133.2105	70.29818	140.0874	70.27395	148.3284	70.48635	134.1184
70.288	136.1565	70.31102	129.4991	70.37407	133.2134	70.31485	140.0871	70.29062	148.3264	70.50302	134.1198
70.30467	136.1537	70.32768	129.4966	70.39073	133.2106	70.33152	140.0852	70.30728	148.3287	70.51968	134.1172
70.32133	136.1515	70.34435	129.494	70.4074	133.21	70.34818	140.0842	70.32395	148.3257	70.53635	134.1152
70.338	136.1509	70.36102	129.4924	70.42407	133.209	70.36485	140.0855	70.34062	148.3248	70.55302	134.1141
70.35467	136.1477	70.37768	129.4939	70.44073	133.2073	70.38152	140.08	70.35728	148.3257	70.56968	134.1175
70.37133	136.1471	70.39435	129.496	70.4574	133.2096	70.39818	140.0818	70.37395	148.3272	70.58635	134.1157
70.388	136.1474	70.41102	129.4962	70.47407	133.2084	70.41485	140.0816	70.39062	148.3265	70.60302	134.1141
70.40467	136.1471	70.42768	129.4934	70.49073	133.204	70.43152	140.0823	70.40728	148.3258	70.61968	134.1147
70.42133	136.143	70.44435	129.4938	70.5074	133.2064	70.44818	140.0796	70.42395	148.3262	70.63635	134.1143
70.438	136.1412	70.46102	129.4886	70.52407	133.2055	70.46485	140.0799	70.44062	148.3274	70.65302	134.1119
70.45467	136.1437	70.47768	129.4847	70.54073	133.2063	70.48152	140.0802	70.45728	148.3274	70.66968	134.1118
70.47133	136.1411	70.49435	129.4828	70.5574	133.2063	70.49818	140.0772	70.47395	148.3272	70.68635	134.1099
70.488	136.1417	70.51102	129.4823	70.57407	133.2057	70.51485	140.0766	70.49062	148.3264	70.70302	134.1108
70.50467	136.1375	70.52768	129.4862	70.59073	133.2027	70.53152	140.0768	70.50728	148.328	70.71968	134.1081
70.52133	136.1366	70.54435	129.4832	70.6074	133.2017	70.54818	140.0769	70.52395	148.3283	70.73635	134.1098
70.538	136.1365	70.56102	129.4825	70.62407	133.2034	70.56485	140.0773	70.54062	148.3294	70.75302	134.1059
70.55467	136.1382	70.57768	129.4784	70.64073	133.2026	70.58152	140.0772	70.55728	148.3259	70.76968	134.1072
70.57133	136.1326	70.59435	129.4766	70.6574	133.2028	70.59818	140.0771	70.57395	148.3305	70.78635	134.1073
70.588	136.1309	70.61102	129.4779	70.67407	133.2014	70.61485	140.0757	70.59062	148.3281	70.80302	134.1095
70.60467	136.1294	70.62768	129.4747	70.69073	133.2021	70.63152	140.0751	70.60728	148.327	70.81968	134.1047
70.62133	136.1316	70.64435	129.4738	70.7074	133.2016	70.64818	140.0763	70.62395	148.3273	70.83635	134.1066
70.638	136.1261	70.66102	129.4772	70.72407	133.2005	70.66485	140.0754	70.64062	148.327	70.85302	134.1075
70.65467	136.1272	70.67768	129.4755	70.74073	133.2011	70.68152	140.0765	70.65728	148.3281	70.86968	134.1052
70.67133	136.1238	70.69435	129.4717	70.7574	133.2015	70.69818	140.0754	70.67395	148.3289	70.88635	134.1059
70.688	136.1242	70.71102	129.4736	70.77407	133.2015	70.71485	140.075	70.69062	148.3285	70.90302	134.1073
70.70467	136.1226	70.72768	129.4686	70.79073	133.2001	70.73152	140.0736	70.70728	148.3285	70.91968	134.1032
70.72133	136.1226	70.74435	129.4691	70.8074	133.1991	70.74818	140.0717	70.72395	148.3283	70.93635	134.1008
70.738	136.1223	70.76102	129.4694	70.82407	133.1963	70.76485	140.0731	70.74062	148.3297	70.95302	134.104
70.75467	136.1224	70.77768	129.4659	70.84073	133.1997	70.78152	140.0737	70.75728	148.3278	70.96968	134.1038
70.77133	136.1221	70.79435	129.4657	70.8574	133.1962	70.79818	140.0742	70.77395	148.3289	70.98635	134.1012
70.788	136.1183	70.81102	129.4634	70.87407	133.2006	70.81485	140.071	70.79062	148.3307	71.00302	134.1036
70.80467	136.1151	70.82768	129.4621	70.89073	133.1929	70.83152	140.073	70.80728	148.3283	71.01968	134.098
70.82133	136.1174	70.84435	129.4614	70.9074	133.1966	70.84818	140.071	70.82395	148.3284	71.03635	134.0995
70.838	136.1153	70.86102	129.4601	70.92407	133.1935	70.86485	140.0691	70.84062	148.3269	71.05302	134.0983
70.85467	136.1141	70.87768	129.458	70.94073	133.1992	70.88152	140.0672	70.85728	148.3269	71.06968	134.0985
70.87133	136.1102	70.89435	129.457	70.9574	133.1972	70.89818	140.0695	70.87395	148.3281	71.08635	134.0978
70.888	136.1119	70.91102	129.4573	70.97407	133.1967	70.91485	140.07	70.89062	148.3262	71.10302	134.0975
70.90467	136.1144	70.92768	129.4549	70.99073	133.1969	70.93152	140.0697	70.90728	148.3279	71.11968	134.0952
70.92133	136.1106	70.94435	129.4514	71.0074	133.1936	70.94818	140.0691	70.92395	148.3258	71.13635	134.0959
70.938	136.1069	70.96102	129.4519	71.02407	133.1951	70.96485	140.0694	70.94062	148.3275	71.15302	134.0933
70.95467	136.1078	70.97768	129.4508	71.04073	133.1957	70.98152	140.0662	70.95728	148.3268	71.16968	134.0947
70.97133	136.1041	70.99435	129.4499	71.0574	133.1955	70.99818	140.0673	70.97395	148.3248	71.18635	134.0917
70.988	136.1061	71.01102	129.4487	71.07407	133.196	71.01485	140.0667	70.99062	148.3268	71.20302	134.0939
71.00467	136.0996	71.02768	129.4486	71.09073	133.1931	71.03152	140.0683	71.00728	148.3277	71.21968	134.0909
71.02133	136.1026	71.04435	129.4479	71.1074	133.1939	71.04818	140.0638	71.02395	148.3268	71.23635	134.0912
71.038	136.1007	71.06102	129.446	71.12407	133.1952	71.06485	140.0659	71.04062	148.327	71.25302	134.0908
71.05467	136.0986	71.07768	129.4428	71.14073	133.1926	71.08152	140.0634	71.05728	148.3277	71.26968	134.0914
71.07133	136.0969	71.09435	129.4412	71.1574	133.192	71.09818	140.0648	71.07395	148.3269	71.28635	134.0892
71.088	136.099	71.11102	129.4387	71.17407	133.195	71.11485	140.0628	71.09062	148.3257	71.30302	134.0894
71.10467	136.0946	71.12768	129.4432	71.19073	133.1919	71.13152	140.0633	71.10728	148.3265	71.31968	134.0884
71.12133	136.0948	71.14435	129.4387	71.2074	133.189	71.14818	140.0657	71.12395	148.3268	71.33635	134.0902
71.138	136.0931	71.16102	129.4363	71.22407	133.1891	71.16485	140.065	71.14062	148.329	71.35302	134.0874
71.15467	136.0939	71.17768	129.4384	71.24073	133.1878	71.18152	140.0623	71.15728	148.3291	71.36968	134.0899
71.17133	136.0925	71.19435	129.4338	71.2574	133.1886	71.19818	140.0602	71.17395	148.3259	71.38635	134.0877
71.188	136.0913	71.21102	129.4337	71.27407	133.1871	71.21485	140.0589	71.19062	148.3253	71.40302	134.0885
71.20467	136.0877	71.22768	129.4306	71.29073	133.1901	71.23152	140.058	71.20728	148.3268	71.41968	134.0876
71.22133	136.0892	71.24435	129.4303	71.3074	133.1861	71.24818	140.0581	71.22395	148.3291	71.43635	134.0856
71.238	136.0893	71.26102	129.4314	71.32407	133.186	71.26485	140.0588	71.24062	148.3264	71.45302	134.0853
71.25467	136.0859	71.27768	129.4262	71.34073	133.1892	71.28152	140.0591	71.25728	148.3263	71.46968	134.0844
71.27133	136.0846	71.29435	129.4276	71.3574	133.1862	71.29818	140.0587	71.27395	148.3269	71.48635	134.0855

71.288	136.0841	71.31102	129.4227	71.37407	133.1867	71.31485	140.0579	71.29062	148.3249	71.50302	134.0824
71.30467	136.0845	71.32768	129.4217	71.39073	133.187	71.33152	140.0585	71.30728	148.3309	71.51968	134.083
71.32133	136.0842	71.34435	129.4212	71.4074	133.188	71.34818	140.0591	71.32395	148.3288	71.53635	134.0845
71.338	136.0793	71.36102	129.4204	71.42407	133.188	71.36485	140.0565	71.34062	148.3271	71.55302	134.0799
71.35467	136.0795	71.37768	129.4204	71.44073	133.1854	71.38152	140.057	71.35728	148.3277	71.56968	134.083
71.37133	136.0796	71.39435	129.4186	71.4574	133.1845	71.39818	140.0556	71.37395	148.3301	71.58635	134.0832
71.388	136.0799	71.41102	129.4188	71.47407	133.1883	71.41485	140.055	71.39062	148.3279	71.60302	134.0801
71.40467	136.0735	71.42768	129.414	71.49073	133.1835	71.43152	140.0557	71.40728	148.3284	71.61968	134.0795
71.42133	136.0717	71.44435	129.4136	71.5074	133.1842	71.44818	140.0546	71.42395	148.3317	71.63635	134.0806
71.438	136.0698	71.46102	129.4115	71.52407	133.1823	71.46485	140.0553	71.44062	148.3293	71.65302	134.0784
71.45467	136.0735	71.47768	129.4111	71.54073	133.183	71.48152	140.052	71.45728	148.3292	71.66968	134.0766
71.47133	136.0709	71.49435	129.4133	71.5574	133.1826	71.49818	140.0543	71.47395	148.3307	71.68635	134.0771
71.488	136.0691	71.51102	129.4096	71.57407	133.1843	71.51485	140.0527	71.49062	148.3285	71.70302	134.0783
71.50467	136.0671	71.52768	129.4079	71.59073	133.1845	71.53152	140.054	71.50728	148.3328	71.71968	134.0731
71.52133	136.0659	71.54435	129.4072	71.6074	133.182	71.54818	140.0531	71.52395	148.332	71.73635	134.0764
71.538	136.0692	71.56102	129.406	71.62407	133.1824	71.56485	140.0519	71.54062	148.3301	71.75302	134.0766
71.55467	136.068	71.57768	129.4051	71.64073	133.1847	71.58152	140.0509	71.55728	148.3322	71.76968	134.0748
71.57133	136.0621	71.59435	129.4058	71.6574	133.1818	71.59818	140.05	71.57395	148.3298	71.78635	134.0732
71.588	136.0604	71.61102	129.404	71.67407	133.1812	71.61485	140.0526	71.59062	148.3291	71.80302	134.0738
71.60467	136.0593	71.62768	129.4	71.69073	133.1815	71.63152	140.051	71.60728	148.3304	71.81968	134.0711
71.62133	136.0626	71.64435	129.4017	71.7074	133.1811	71.64818	140.0498	71.62395	148.3338	71.83635	134.0717
71.638	136.0611	71.66102	129.3971	71.72407	133.1812	71.66485	140.0524	71.64062	148.3333	71.85302	134.0717
71.65467	136.0584	71.67768	129.3964	71.74073	133.1791	71.68152	140.0533	71.65728	148.3311	71.86968	134.072
71.67133	136.0609	71.69435	129.394	71.7574	133.1812	71.69818	140.0512	71.67395	148.3304	71.88635	134.0699
71.688	136.0584	71.71102	129.3945	71.77407	133.1787	71.71485	140.0536	71.69062	148.3336	71.90302	134.0717
71.70467	136.0518	71.72768	129.3933	71.79073	133.1784	71.73152	140.0544	71.70728	148.3327	71.91968	134.0692
71.72133	136.0522	71.74435	129.3899	71.8074	133.178	71.74818	140.0536	71.72395	148.3291	71.93635	134.0674
71.738	136.0442	71.76102	129.3885	71.82407	133.1758	71.76485	140.05	71.74062	148.3295	71.95302	134.0662
71.75467	136.052	71.77768	129.3897	71.84073	133.1795	71.78152	140.0511	71.75728	148.3298	71.96968	134.0672
71.77133	136.0527	71.79435	129.3884	71.8574	133.1759	71.79818	140.0501	71.77395	148.3312	71.98635	134.0665
71.788	136.0467	71.81102	129.388	71.87407	133.1759	71.81485	140.0491	71.79062	148.3314	72.00302	134.0643
71.80467	136.0474	71.82768	129.3813	71.89073	133.1737	71.83152	140.0518	71.80728	148.3314	72.01968	134.0652
71.82133	136.0471	71.84435	129.3838	71.9074	133.176	71.84818	140.0501	71.82395	148.3304	72.03635	134.0659
71.838	136.048	71.86102	129.3815	71.92407	133.1724	71.86485	140.0506	71.84062	148.3359	72.05302	134.0632
71.85467	136.0467	71.87768	129.381	71.94073	133.172	71.88152	140.0497	71.85728	148.3319	72.06968	134.0609
71.87133	136.0479	71.89435	129.3778	71.9574	133.1737	71.89818	140.0483	71.87395	148.3294	72.08635	134.062
71.888	136.0385	71.91102	129.3759	71.97407	133.1731	71.91485	140.0473	71.89062	148.3301	72.10302	134.0598
71.90467	136.0374	71.92768	129.3774	71.99073	133.1705	71.93152	140.0502	71.90728	148.3312	72.11968	134.0577
71.92133	136.0384	71.94435	129.3772	72.0074	133.1701	71.94818	140.0492	71.92395	148.3293	72.13635	134.061
71.938	136.0364	71.96102	129.3739	72.02407	133.1698	71.96485	140.0497	71.94062	148.3288	72.15302	134.0574
71.95467	136.0403	71.97768	129.3772	72.04073	133.1701	71.98152	140.046	71.95728	148.3314	72.16968	134.0588
71.97133	136.0358	71.99435	129.3758	72.0574	133.1687	71.99818	140.0445	71.97395	148.3307	72.18635	134.0571
71.988	136.0349	72.01102	129.3707	72.07407	133.1686	72.01485	140.0429	71.99062	148.3297	72.20302	134.0577
72.00467	136.0328	72.02768	129.3698	72.09073	133.1686	72.03152	140.0477	72.00728	148.33	72.21968	134.0528
72.02133	136.0297	72.04435	129.3675	72.1074	133.1685	72.04818	140.0465	72.02395	148.326	72.23635	134.0547
72.038	136.0307	72.06102	129.3679	72.12407	133.1685	72.06485	140.0451	72.04062	148.3306	72.25302	134.0522
72.05467	136.0287	72.07768	129.3642	72.14073	133.1653	72.08152	140.048	72.05728	148.329	72.26968	134.0536
72.07133	136.0274	72.09435	129.3669	72.1574	133.1658	72.09818	140.044	72.07395	148.3338	72.28635	134.0509
72.088	136.021	72.11102	129.3635	72.17407	133.1671	72.11485	140.0474	72.09062	148.3295	72.30302	134.0517
72.10467	136.0253	72.12768	129.3612	72.19073	133.1645	72.13152	140.0476	72.10728	148.3317	72.31968	134.05
72.12133	136.0229	72.14435	129.3583	72.2074	133.1653	72.14818	140.0491	72.12395	148.3303	72.33635	134.0504
72.138	136.0187	72.16102	129.358	72.22407	133.1644	72.16485	140.0459	72.14062	148.3297	72.35302	134.0486
72.15467	136.021	72.17768	129.3577	72.24073	133.1644	72.18152	140.046	72.15728	148.3304	72.36968	134.0481
72.17133	136.0195	72.19435	129.3565	72.2574	133.1647	72.19818	140.0437	72.17395	148.3289	72.38635	134.0469
72.188	136.0183	72.21102	129.3528	72.27407	133.1652	72.21485	140.0466	72.19062	148.3281	72.40302	134.0464
72.20467	136.0192	72.22768	129.3519	72.29073	133.1624	72.23152	140.0449	72.20728	148.3317	72.41968	134.0447
72.22133	136.0121	72.24435	129.3517	72.3074	133.1641	72.24818	140.045	72.22395	148.3294	72.43635	134.0465
72.238	136.0154	72.26102	129.3494	72.32407	133.1638	72.26485	140.045	72.24062	148.3315	72.45302	134.0447
72.25467	136.0168	72.27768	129.3474	72.34073	133.1622	72.28152	140.0433	72.25728	148.3313	72.46968	134.0438
72.27133	136.0115	72.29435	129.3493	72.3574	133.1623	72.29818	140.0454	72.27395	148.3324	72.48635	134.042
72.288	136.0118	72.31102	129.3438	72.37407	133.1625	72.31485	140.0442	72.29062	148.3315	72.50302	134.0424
72.30467	136.0069	72.32768	129.3455	72.39073	133.1625	72.33152	140.0433	72.30728	148.3322	72.51968	134.0439
72.32133	136.0082	72.34435	129.3434	72.4074	133.1601	72.34818	140.0429	72.32395	148.3329	72.53635	134.0421
72.338	136.0085	72.36102	129.3409	72.42407	133.1603	72.36485	140.0445	72.34062	148.3334	72.55302	134.0423
72.35467	136.0055	72.37768	129.3422	72.44073	133.1597	72.38152	140.0418	72.35728	148.3317	72.56968	134.0398
72.37133	136.0067	72.39435	129.3384	72.4574	133.1581	72.39818	140.042	72.37395	148.3343	72.58635	134.0389
72.388	136.0039	72.41102	129.3363	72.47407	133.1607	72.41485	140.0419	72.39062	148.3302	72.60302	134.0386
72.40467	136.0001	72.42768	129.3366	72.49073	133.1583	72.43152	140.0419	72.40728	148.3338	72.61968	134.0383
72.42133	136.0031	72.44435	129.3358	72.5074	133.1589	72.44818	140.0425	72.42395	148.3346	72.63635	134.0383

72.438	136.0032	72.46102	129.3333	72.52407	133.1578	72.46485	140.0436	72.44062	148.3318	72.65302	134.0386
72.45467	135.9959	72.47768	129.3337	72.54073	133.1591	72.48152	140.0421	72.45728	148.3348	72.66968	134.0363
72.47133	136.0009	72.49435	129.3313	72.5574	133.1585	72.49818	140.0432	72.47395	148.3345	72.68635	134.0337
72.488	135.9952	72.51102	129.3331	72.57407	133.1574	72.51485	140.0424	72.49062	148.3318	72.70302	134.0346
72.50467	135.9927	72.52768	129.3305	72.59073	133.1555	72.53152	140.0432	72.50728	148.3323	72.71968	134.0344
72.52133	135.9952	72.54435	129.3284	72.6074	133.1562	72.54818	140.0424	72.52395	148.3329	72.73635	134.0339
72.538	135.9978	72.56102	129.327	72.62407	133.1541	72.56485	140.042	72.54062	148.3316	72.75302	134.0314
72.55467	135.997	72.57768	129.3261	72.64073	133.1532	72.58152	140.0427	72.55728	148.3298	72.76968	134.0319
72.57133	135.9906	72.59435	129.3277	72.6574	133.1549	72.59818	140.0424	72.57395	148.3323	72.78635	134.0319
72.588	135.9915	72.61102	129.3233	72.67407	133.1549	72.61485	140.0423	72.59062	148.3325	72.80302	134.0297
72.60467	135.9888	72.62768	129.3239	72.69073	133.1533	72.63152	140.0427	72.60728	148.3304	72.81968	134.0283
72.62133	135.9879	72.64435	129.3245	72.7074	133.1504	72.64818	140.0435	72.62395	148.3333	72.83635	134.0307
72.638	135.9872	72.66102	129.3236	72.72407	133.1515	72.66485	140.0432	72.64062	148.3329	72.85302	134.0296
72.65467	135.9891	72.67768	129.3235	72.74073	133.1527	72.68152	140.0428	72.65728	148.3317	72.86968	134.0278
72.67133	135.983	72.69435	129.3237	72.7574	133.1525	72.69818	140.0412	72.67395	148.3308	72.88635	134.0246
72.688	135.9867	72.71102	129.3186	72.77407	133.1531	72.71485	140.0431	72.69062	148.3285	72.90302	134.0267
72.70467	135.9811	72.72768	129.3178	72.79073	133.1537	72.73152	140.043	72.70728	148.3317	72.91968	134.0243
72.72133	135.9826	72.74435	129.3177	72.8074	133.1516	72.74818	140.0428	72.72395	148.3272	72.93635	134.0234
72.738	135.9819	72.76102	129.3186	72.82407	133.1493	72.76485	140.0436	72.74062	148.329	72.95302	134.0236
72.75467	135.9838	72.77768	129.3173	72.84073	133.1492	72.78152	140.0448	72.75728	148.3285	72.96968	134.0233
72.77133	135.9796	72.79435	129.3145	72.8574	133.1493	72.79818	140.0424	72.77395	148.327	72.98635	134.0239
72.788	135.9796	72.81102	129.3152	72.87407	133.15	72.81485	140.0402	72.79062	148.329	73.00302	134.0236
72.80467	135.9808	72.82768	129.3135	72.89073	133.147	72.83152	140.0444	72.80728	148.3284	73.01968	134.0221
72.82133	135.9786	72.84435	129.3108	72.9074	133.1485	72.84818	140.0428	72.82395	148.3269	73.03635	134.0225
72.838	135.9756	72.86102	129.3106	72.92407	133.1487	72.86485	140.0429	72.84062	148.3298	73.05302	134.0214
72.85467	135.9776	72.87768	129.3092	72.94073	133.1457	72.88152	140.042	72.85728	148.3281	73.06968	134.0214
72.87133	135.9738	72.89435	129.3081	72.9574	133.1454	72.89818	140.0426	72.87395	148.3276	73.08635	134.02
72.888	135.9751	72.91102	129.3086	72.97407	133.1434	72.91485	140.0377	72.89062	148.3261	73.10302	134.0193
72.90467	135.9768	72.92768	129.3062	72.99073	133.1443	72.93152	140.0375	72.90728	148.3293	73.11968	134.018
72.92133	135.9722	72.94435	129.3054	73.0074	133.1457	72.94818	140.0388	72.92395	148.3281	73.13635	134.0196
72.938	135.9709	72.96102	129.3036	73.02407	133.1431	72.96485	140.0387	72.94062	148.3278	73.15302	134.0182
72.95467	135.9695	72.97768	129.3028	73.04073	133.1441	72.98152	140.0386	72.95728	148.3272	73.16968	134.0172
72.97133	135.97	72.99435	129.3016	73.0574	133.1437	72.99818	140.0387	72.97395	148.3274	73.18635	134.016
72.988	135.9692	73.01102	129.3021	73.07407	133.1407	73.01485	140.0377	72.99062	148.326	73.20302	134.0157
73.00467	135.972	73.02768	129.3004	73.09073	133.1408	73.03152	140.0373	73.00728	148.3245	73.21968	134.0156
73.02133	135.9662	73.04435	129.2997	73.1074	133.1426	73.04818	140.0372	73.02395	148.3268	73.23635	134.0134
73.038	135.967	73.06102	129.3008	73.12407	133.1398	73.06485	140.0375	73.04062	148.326	73.25302	134.015
73.05467	135.9645	73.07768	129.2991	73.14073	133.138	73.08152	140.0358	73.05728	148.3235	73.26968	134.0129
73.07133	135.9641	73.09435	129.2972	73.1574	133.1368	73.09818	140.037	73.07395	148.3266	73.28635	134.0143
73.088	135.962	73.11102	129.2955	73.17407	133.1394	73.11485	140.0364	73.09062	148.3254	73.30302	134.0147
73.10467	135.963	73.12768	129.296	73.19073	133.1379	73.13152	140.0359	73.10728	148.324	73.31968	134.0108
73.12133	135.9591	73.14435	129.2957	73.2074	133.1366	73.14818	140.0352	73.12395	148.3244	73.33635	134.0101
73.138	135.9606	73.16102	129.2974	73.22407	133.1367	73.16485	140.0374	73.14062	148.3266	73.35302	134.0098
73.15467	135.9595	73.17768	129.2935	73.24073	133.136	73.18152	140.0366	73.15728	148.3261	73.36968	134.01
73.17133	135.9608	73.19435	129.2914	73.2574	133.1345	73.19818	140.0387	73.17395	148.3257	73.38635	134.0103
73.188	135.957	73.21102	129.2889	73.27407	133.1314	73.21485	140.0374	73.19062	148.3235	73.40302	134.0045
73.20467	135.9576	73.22768	129.2908	73.29073	133.1339	73.23152	140.0361	73.20728	148.3263	73.41968	134.004
73.22133	135.9588	73.24435	129.2865	73.3074	133.1335	73.24818	140.0362	73.22395	148.3256	73.43635	134.0063
73.238	135.9505	73.26102	129.2897	73.32407	133.1343	73.26485	140.0353	73.24062	148.3263	73.45302	134.005
73.25467	135.9513	73.27768	129.2854	73.34073	133.1325	73.28152	140.0382	73.25728	148.3257	73.46968	134.0056
73.27133	135.9503	73.29435	129.2853	73.3574	133.1336	73.29818	140.0383	73.27395	148.3229	73.48635	134.0069
73.288	135.9488	73.31102	129.2845	73.37407	133.1321	73.31485	140.0359	73.29062	148.3248	73.50302	134.0006
73.30467	135.9517	73.32768	129.2843	73.39073	133.1324	73.33152	140.0372	73.30728	148.3242	73.51968	134.002
73.32133	135.9499	73.34435	129.2823	73.4074	133.1297	73.34818	140.0341	73.32395	148.3187	73.53635	134.0013
73.338	135.9473	73.36102	129.2801	73.42407	133.131	73.36485	140.0352	73.34062	148.3262	73.55302	134.0026
73.35467	135.9449	73.37768	129.2792	73.44073	133.1303	73.38152	140.0357	73.35728	148.3249	73.56968	134.0004
73.37133	135.9449	73.39435	129.2781	73.4574	133.1334	73.39818	140.037	73.37395	148.3262	73.58635	134.0001
73.388	135.9457	73.41102	129.2756	73.47407	133.1291	73.41485	140.0357	73.39062	148.3236	73.60302	133.9984
73.40467	135.9411	73.42768	129.2747	73.49073	133.1292	73.43152	140.0355	73.40728	148.3242	73.61968	133.9967
73.42133	135.9403	73.44435	129.2741	73.5074	133.1301	73.44818	140.0345	73.42395	148.3237	73.63635	133.999
73.438	135.9393	73.46102	129.2789	73.52407	133.1288	73.46485	140.0348	73.44062	148.3267	73.65302	133.9997
73.45467	135.9383	73.47768	129.2684	73.54073	133.1292	73.48152	140.0333	73.45728	148.3252	73.66968	133.9969
73.47133	135.9388	73.49435	129.2681	73.5574	133.1272	73.49818	140.033	73.47395	148.3271	73.68635	133.9976
73.488	135.9324	73.51102	129.2718	73.57407	133.1253	73.51485	140.0325	73.49062	148.3255	73.70302	133.9983
73.50467	135.9324	73.52768	129.2716	73.59073	133.1263	73.53152	140.0328	73.50728	148.3255	73.71968	133.9949
73.52133	135.9318	73.54435	129.268	73.6074	133.1205	73.54818	140.0324	73.52395	148.3256	73.73635	133.9966
73.538	135.931	73.56102	129.266	73.62407	133.1232	73.56485	140.0331	73.54062	148.3268	73.75302	133.9951
73.55467	135.9314	73.57768	129.267	73.64073	133.1222	73.58152	140.0318	73.55728	148.3288	73.76968	133.9941
73.57133	135.9314	73.59435	129.2663	73.6574	133.1201	73.59818	140.0315	73.57395	148.3271	73.78635	133.9939

73.588	135.9315	73.61102	129.2622	73.67407	133.1184	73.61485	140.0322	73.59062	148.3301	73.80302	133.995
73.60467	135.9285	73.62768	129.265	73.69073	133.116	73.63152	140.0339	73.60728	148.323	73.81968	133.9934
73.62133	135.9288	73.64435	129.2611	73.7074	133.118	73.64818	140.0309	73.62395	148.3271	73.83635	133.9931
73.638	135.9266	73.66102	129.265	73.72407	133.114	73.66485	140.0319	73.64062	148.3247	73.85302	133.9936
73.65467	135.9251	73.67768	129.26	73.74073	133.1147	73.68152	140.0323	73.65728	148.3204	73.86968	133.9895
73.67133	135.9206	73.69435	129.2576	73.7574	133.1126	73.69818	140.0304	73.67395	148.3218	73.88635	133.988
73.688	135.9191	73.71102	129.2582	73.77407	133.1119	73.71485	140.0304	73.69062	148.3209	73.90302	133.9876
73.70467	135.9174	73.72768	129.2556	73.79073	133.1132	73.73152	140.0307	73.70728	148.3229	73.91968	133.9863
73.72133	135.9178	73.74435	129.258	73.8074	133.1127	73.74818	140.0295	73.72395	148.3228	73.93635	133.9849
73.738	135.9172	73.76102	129.2558	73.82407	133.112	73.76485	140.0307	73.74062	148.3238	73.95302	133.9838
73.75467	135.9177	73.77768	129.2552	73.84073	133.1091	73.78152	140.0277	73.75728	148.3226	73.96968	133.982
73.77133	135.9139	73.79435	129.2527	73.8574	133.1104	73.79818	140.029	73.77395	148.3205	73.98635	133.9852
73.788	135.9146	73.81102	129.2503	73.87407	133.1122	73.81485	140.029	73.79062	148.3229	74.00302	133.984
73.80467	135.9121	73.82768	129.2487	73.89073	133.1097	73.83152	140.0291	73.80728	148.3225	74.01968	133.9802
73.82133	135.9094	73.84435	129.2514	73.9074	133.1097	73.84818	140.0272	73.82395	148.3226	74.03635	133.9805
73.838	135.9098	73.86102	129.2522	73.92407	133.1103	73.86485	140.0263	73.84062	148.3203	74.05302	133.983
73.85467	135.9096	73.87768	129.2492	73.94073	133.1103	73.88152	140.0263	73.85728	148.3219	74.06968	133.9813
73.87133	135.9118	73.89435	129.2455	73.9574	133.1083	73.89818	140.0274	73.87395	148.3235	74.08635	133.9781
73.888	135.9097	73.91102	129.2448	73.97407	133.108	73.91485	140.0271	73.89062	148.3233	74.10302	133.9793
73.90467	135.9028	73.92768	129.2445	73.99073	133.108	73.93152	140.0271	73.90728	148.3222	74.11968	133.9804
73.92133	135.9053	73.94435	129.2428	74.0074	133.1114	73.94818	140.0257	73.92395	148.3232	74.13635	133.9789
73.938	135.902	73.96102	129.2447	74.02407	133.109	73.96485	140.0263	73.94062	148.3228	74.15302	133.9795
73.95467	135.9022	73.97768	129.2435	74.04073	133.1076	73.98152	140.0267	73.95728	148.3226	74.16968	133.9774
73.97133	135.9009	73.99435	129.2431	74.0574	133.1067	73.99818	140.0274	73.97395	148.322	74.18635	133.9772
73.988	135.8984	74.01102	129.243	74.07407	133.1069	74.01485	140.0265	73.99062	148.32	74.20302	133.9773
74.00467	135.8991	74.02768	129.2409	74.09073	133.1048	74.03152	140.0262	74.00728	148.3218	74.21968	133.9739
74.02133	135.8973	74.04435	129.2396	74.1074	133.1045	74.04818	140.0259	74.02395	148.3211	74.23635	133.9721
74.038	135.8948	74.06102	129.2365	74.12407	133.1066	74.06485	140.0241	74.04062	148.3213	74.25302	133.9744
74.05467	135.8949	74.07768	129.2343	74.14073	133.1053	74.08152	140.0244	74.05728	148.3206	74.26968	133.9735
74.07133	135.8966	74.09435	129.2363	74.1574	133.1037	74.09818	140.0259	74.07395	148.32	74.28635	133.9735
74.088	135.8946	74.11102	129.2317	74.17407	133.1039	74.11485	140.0239	74.09062	148.321	74.30302	133.9708
74.10467	135.8895	74.12768	129.2331	74.19073	133.1049	74.13152	140.0215	74.10728	148.3203	74.31968	133.9719
74.12133	135.8857	74.14435	129.2308	74.2074	133.0991	74.14818	140.0222	74.12395	148.3235	74.33635	133.9718
74.138	135.8916	74.16102	129.2327	74.22407	133.1	74.16485	140.0223	74.14062	148.3231	74.35302	133.9701
74.15467	135.8886	74.17768	129.2321	74.24073	133.1024	74.18152	140.0226	74.15728	148.3221	74.36968	133.9692
74.17133	135.8842	74.19435	129.2289	74.2574	133.0995	74.19818	140.0213	74.17395	148.3224	74.38635	133.9672
74.188	135.886	74.21102	129.2285	74.27407	133.1012	74.21485	140.0206	74.19062	148.3194	74.40302	133.9676
74.20467	135.8793	74.22768	129.225	74.29073	133.0959	74.23152	140.0197	74.20728	148.3178	74.41968	133.9697
74.22133	135.8821	74.24435	129.2269	74.3074	133.0973	74.24818	140.0207	74.22395	148.3194	74.43635	133.9663
74.238	135.8784	74.26102	129.2238	74.32407	133.0972	74.26485	140.0227	74.24062	148.3213	74.45302	133.9659
74.25467	135.8794	74.27768	129.2219	74.34073	133.0941	74.28152	140.0206	74.25728	148.3203	74.46968	133.9672
74.27133	135.8806	74.29435	129.2236	74.3574	133.0944	74.29818	140.0188	74.27395	148.3189	74.48635	133.9646
74.288	135.8823	74.31102	129.223	74.37407	133.0961	74.31485	140.0171	74.29062	148.3191	74.50302	133.964
74.30467	135.8763	74.32768	129.2232	74.39073	133.0954	74.33152	140.0184	74.30728	148.3168	74.51968	133.9665
74.32133	135.8755	74.34435	129.2188	74.4074	133.097	74.34818	140.0185	74.32395	148.3178	74.53635	133.9601
74.338	135.8748	74.36102	129.2179	74.42407	133.0953	74.36485	140.0177	74.34062	148.3196	74.55302	133.9619
74.35467	135.8725	74.37768	129.2186	74.44073	133.0924	74.38152	140.0175	74.35728	148.3201	74.56968	133.9636
74.37133	135.8731	74.39435	129.216	74.4574	133.0941	74.39818	140.0184	74.37395	148.318	74.58635	133.9625
74.388	135.8687	74.41102	129.2117	74.47407	133.0969	74.41485	140.0151	74.39062	148.3188	74.60302	133.9593
74.40467	135.8704	74.42768	129.2122	74.49073	133.0938	74.43152	140.0152	74.40728	148.3168	74.61968	133.9614
74.42133	135.8677	74.44435	129.2117	74.5074	133.0924	74.44818	140.0148	74.42395	148.3161	74.63635	133.9581
74.438	135.8675	74.46102	129.2103	74.52407	133.0945	74.46485	140.0143	74.44062	148.3184	74.65302	133.9583
74.45467	135.8664	74.47768	129.2082	74.54073	133.0928	74.48152	140.0144	74.45728	148.3175	74.66968	133.961
74.47133	135.8675	74.49435	129.208	74.5574	133.0932	74.49818	140.0107	74.47395	148.3197	74.68635	133.9578
74.488	135.8695	74.51102	129.2064	74.57407	133.0905	74.51485	140.014	74.49062	148.3194	74.70302	133.9577
74.50467	135.8664	74.52768	129.2043	74.59073	133.0911	74.53152	140.0137	74.50728	148.3142	74.71968	133.9571
74.52133	135.864	74.54435	129.2049	74.6074	133.0913	74.54818	140.0123	74.52395	148.316	74.73635	133.956
74.538	135.8601	74.56102	129.2024	74.62407	133.089	74.56485	140.0121	74.54062	148.3131	74.75302	133.9585
74.55467	135.8643	74.57768	129.2021	74.64073	133.0903	74.58152	140.0126	74.55728	148.3208	74.76968	133.9553
74.57133	135.8632	74.59435	129.2019	74.6574	133.0891	74.59818	140.0137	74.57395	148.3169	74.78635	133.9552
74.588	135.8596	74.61102	129.2004	74.67407	133.0888	74.61485	140.0151	74.59062	148.3203	74.80302	133.9518
74.60467	135.8638	74.62768	129.1995	74.69073	133.0894	74.63152	140.0134	74.60728	148.3181	74.81968	133.9545
74.62133	135.8588	74.64435	129.198	74.7074	133.0875	74.64818	140.0118	74.62395	148.3191	74.83635	133.9548
74.638	135.8585	74.66102	129.1986	74.72407	133.0903	74.66485	140.0127	74.64062	148.318	74.85302	133.9538
74.65467	135.8566	74.67768	129.195	74.74073	133.0902	74.68152	140.0142	74.65728	148.3189	74.86968	133.9518
74.67133	135.8531	74.69435	129.1934	74.7574	133.0911	74.69818	140.0127	74.67395	148.3196	74.88635	133.9533
74.688	135.8525	74.71102	129.1954	74.77407	133.0896	74.71485	140.0126	74.69062	148.3163	74.90302	133.9546
74.70467	135.8526	74.72768	129.1928	74.79073	133.0893	74.73152	140.0144	74.70728	148.3207	74.91968	133.9519
74.72133	135.8463	74.74435	129.1935	74.8074	133.0872	74.74818	140.012	74.72395	148.3175	74.93635	133.9519

74.738	135.8513	74.76102	129.1925	74.82407	133.0906	74.76485	140.0134	74.74062	148.3184	74.95302	133.9526
74.75467	135.8438	74.77768	129.1895	74.84073	133.0865	74.78152	140.0138	74.75728	148.3165	74.96968	133.9506
74.77133	135.8459	74.79435	129.1884	74.8574	133.0889	74.79818	140.011	74.77395	148.3163	74.98635	133.9509
74.788	135.8462	74.81102	129.1878	74.87407	133.0848	74.81485	140.0108	74.79062	148.3178	75.00302	133.9493
74.80467	135.8446	74.82768	129.1889	74.89073	133.0869	74.83152	140.0107	74.80728	148.3168	75.01968	133.9479
74.82133	135.8415	74.84435	129.189	74.9074	133.0875	74.84818	140.0126	74.82395	148.3159	75.03635	133.9483
74.838	135.841	74.86102	129.1857	74.92407	133.0877	74.86485	140.0107	74.84062	148.3164	75.05302	133.9469
74.85467	135.8417	74.87768	129.1851	74.94073	133.0865	74.88152	140.0108	74.85728	148.3142	75.06968	133.9466
74.87133	135.841	74.89435	129.1856	74.9574	133.085	74.89818	140.0079	74.87395	148.3182	75.08635	133.9469
74.888	135.8389	74.91102	129.1832	74.97407	133.0845	74.91485	140.0089	74.89062	148.3184	75.10302	133.9445
74.90467	135.8369	74.92768	129.1808	74.99073	133.0874	74.93152	140.0088	74.90728	148.3163	75.11968	133.9444
74.92133	135.8362	74.94435	129.1802	75.0074	133.0849	74.94818	140.008	74.92395	148.3138	75.13635	133.9429
74.938	135.8371	74.96102	129.1787	75.02407	133.0856	74.96485	140.0069	74.94062	148.316	75.15302	133.9415
74.95467	135.835	74.97768	129.1754	75.04073	133.0825	74.98152	140.0086	74.95728	148.3143	75.16968	133.9396
74.97133	135.8336	74.99435	129.1764	75.0574	133.086	74.99818	140.0072	74.97395	148.3174	75.18635	133.94
74.988	135.8326	75.01102	129.1772	75.07407	133.0845	75.01485	140.0072	74.99062	148.3164	75.20302	133.9396
75.00467	135.8294	75.02768	129.1741	75.09073	133.0859	75.03152	140.0073	75.00728	148.3184	75.21968	133.9412
75.02133	135.8291	75.04435	129.1743	75.1074	133.0827	75.04818	140.0054	75.02395	148.3178	75.23635	133.9391
75.038	135.8277	75.06102	129.175	75.12407	133.0809	75.06485	140.0045	75.04062	148.3169	75.25302	133.9391
75.05467	135.8263	75.07768	129.1713	75.14073	133.0824	75.08152	140.0064	75.05728	148.3168	75.26968	133.9366
75.07133	135.8285	75.09435	129.1712	75.1574	133.0819	75.09818	140.0062	75.07395	148.3159	75.28635	133.9386
75.088	135.8251	75.11102	129.169	75.17407	133.079	75.11485	140.0039	75.09062	148.3164	75.30302	133.9388
75.10467	135.8258	75.12768	129.1676	75.19073	133.0781	75.13152	140.0062	75.10728	148.3175	75.31968	133.9367
75.12133	135.8228	75.14435	129.1685	75.2074	133.0791	75.14818	140.0058	75.12395	148.3163	75.33635	133.9361
75.138	135.8216	75.16102	129.1672	75.22407	133.0783	75.16485	140.0055	75.14062	148.3159	75.35302	133.9354
75.15467	135.8214	75.17768	129.1681	75.24073	133.0776	75.18152	140.0047	75.15728	148.3149	75.36968	133.9332
75.17133	135.8196	75.19435	129.1649	75.2574	133.0779	75.19818	140.0047	75.17395	148.3164	75.38635	133.9366
75.188	135.8177	75.21102	129.1632	75.27407	133.0758	75.21485	140.005	75.19062	148.3134	75.40302	133.9344
75.20467	135.8173	75.22768	129.1653	75.29073	133.0764	75.23152	140.0044	75.20728	148.3125	75.41968	133.9344
75.22133	135.8174	75.24435	129.1629	75.3074	133.0776	75.24818	140.0035	75.22395	148.3113	75.43635	133.9353
75.238	135.8158	75.26102	129.1634	75.32407	133.0761	75.26485	140.0031	75.24062	148.3155	75.45302	133.9315
75.25467	135.8152	75.27768	129.1605	75.34073	133.078	75.28152	140.0053	75.25728	148.3121	75.46968	133.9282
75.27133	135.8162	75.29435	129.16	75.3574	133.0773	75.29818	140.0024	75.27395	148.3145	75.48635	133.9304
75.288	135.8119	75.31102	129.1573	75.37407	133.0767	75.31485	140.0037	75.29062	148.3141	75.50302	133.9284
75.30467	135.8134	75.32768	129.1564	75.39073	133.0757	75.33152	140.003	75.30728	148.3144	75.51968	133.9271
75.32133	135.8089	75.34435	129.1553	75.4074	133.0736	75.34818	140.0036	75.32395	148.3142	75.53635	133.927
75.338	135.8074	75.36102	129.1565	75.42407	133.0766	75.36485	140.0009	75.34062	148.3142	75.55302	133.926
75.35467	135.8115	75.37768	129.1551	75.44073	133.0737	75.38152	139.9998	75.35728	148.3146	75.56968	133.9266
75.37133	135.8058	75.39435	129.1531	75.4574	133.0749	75.39818	140.0009	75.37395	148.3134	75.58635	133.9259
75.388	135.8068	75.41102	129.1537	75.47407	133.0718	75.41485	140.0034	75.39062	148.3126	75.60302	133.9278
75.40467	135.8061	75.42768	129.1499	75.49073	133.0749	75.43152	140.0007	75.40728	148.3127	75.61968	133.9259
75.42133	135.8023	75.44435	129.1512	75.5074	133.0742	75.44818	140.0001	75.42395	148.3135	75.63635	133.9252
75.438	135.8022	75.46102	129.1497	75.52407	133.0725	75.46485	139.9986	75.44062	148.3116	75.65302	133.9271
75.45467	135.8014	75.47768	129.1473	75.54073	133.0737	75.48152	140.0001	75.45728	148.3109	75.66968	133.9253
75.47133	135.7999	75.49435	129.1454	75.5574	133.0749	75.49818	140.0002	75.47395	148.3115	75.68635	133.9259
75.488	135.7985	75.51102	129.147	75.57407	133.0707	75.51485	139.9983	75.49062	148.3098	75.70302	133.9232
75.50467	135.7978	75.52768	129.1473	75.59073	133.0729	75.53152	139.9983	75.50728	148.3108	75.71968	133.9243
75.52133	135.7979	75.54435	129.1425	75.6074	133.0712	75.54818	139.9971	75.52395	148.311	75.73635	133.9222
75.538	135.7962	75.56102	129.1435	75.62407	133.0689	75.56485	139.997	75.54062	148.3114	75.75302	133.9232
75.55467	135.7952	75.57768	129.1403	75.64073	133.0698	75.58152	139.9985	75.55728	148.3097	75.76968	133.9222
75.57133	135.7953	75.59435	129.1414	75.6574	133.0717	75.59818	139.9944	75.57395	148.3151	75.78635	133.9215
75.588	135.7905	75.61102	129.1398	75.67407	133.0676	75.61485	139.9935	75.59062	148.3076	75.80302	133.9194
75.60467	135.7917	75.62768	129.137	75.69073	133.0709	75.63152	139.9931	75.60728	148.3101	75.81968	133.9184
75.62133	135.7877	75.64435	129.1353	75.7074	133.0698	75.64818	139.9936	75.62395	148.3109	75.83635	133.9169
75.638	135.789	75.66102	129.1349	75.72407	133.071	75.66485	139.9957	75.64062	148.3112	75.85302	133.9187
75.65467	135.7881	75.67768	129.1364	75.74073	133.0676	75.68152	139.995	75.65728	148.3115	75.86968	133.917
75.67133	135.7875	75.69435	129.1354	75.7574	133.0676	75.69818	139.9928	75.67395	148.3119	75.88635	133.9154
75.688	135.7856	75.71102	129.136	75.77407	133.0658	75.71485	139.9924	75.69062	148.3112	75.90302	133.9171
75.70467	135.7874	75.72768	129.1329	75.79073	133.0663	75.73152	139.9904	75.70728	148.3086	75.91968	133.9163
75.72133	135.784	75.74435	129.1319	75.8074	133.0654	75.74818	139.9918	75.72395	148.3113	75.93635	133.9147
75.738	135.7815	75.76102	129.1337	75.82407	133.0635	75.76485	139.9936	75.74062	148.308	75.95302	133.9161
75.75467	135.7821	75.77768	129.1313	75.84073	133.063	75.78152	139.9927	75.75728	148.3084	75.96968	133.913
75.77133	135.7813	75.79435	129.1315	75.8574	133.0648	75.79818	139.99	75.77395	148.3069	75.98635	133.9163
75.788	135.7785	75.81102	129.1298	75.87407	133.0596	75.81485	139.9905	75.79062	148.3109	76.00302	133.9131
75.80467	135.7822	75.82768	129.1297	75.89073	133.062	75.83152	139.9925	75.80728	148.3153	76.01968	133.9118
75.82133	135.772	75.84435	129.1297	75.9074	133.0614	75.84818	139.991	75.82395	148.3035	76.03635	133.9122
75.838	135.7776	75.86102	129.1285	75.92407	133.0609	75.86485	139.9896	75.84062	148.3049	76.05302	133.9114
75.85467	135.7797	75.87768	129.1278	75.94073	133.0598	75.88152	139.9901	75.85728	148.3097	76.06968	133.9119
75.87133	135.7733	75.89435	129.125	75.9574	133.057	75.89818	139.9879	75.87395	148.307	76.08635	133.9111

75.888	135.7724	75.91102	129.1261	75.97407	133.0585	75.91485	139.9908	75.89062	148.3078	76.10302	133.909
75.90467	135.7717	75.92768	129.1237	75.99073	133.0586	75.93152	139.9905	75.90728	148.3058	76.11968	133.9094
75.92133	135.7672	75.94435	129.1251	76.0074	133.0596	75.94818	139.9887	75.92395	148.3075	76.13635	133.908
75.938	135.7653	75.96102	129.1217	76.02407	133.0578	75.96485	139.987	75.94062	148.3063	76.15302	133.9076
75.95467	135.7664	75.97768	129.1191	76.04073	133.055	75.98152	139.9881	75.95728	148.3045	76.16968	133.907
75.97133	135.7644	75.99435	129.1196	76.0574	133.059	75.99818	139.9881	75.97395	148.3068	76.18635	133.9062
75.988	135.7627	76.01102	129.1194	76.07407	133.0579	76.01485	139.9868	75.99062	148.3045	76.20302	133.9072
76.00467	135.7596	76.02768	129.117	76.09073	133.0579	76.03152	139.9867	76.00728	148.3075	76.21968	133.9062
76.02133	135.7602	76.04435	129.1132	76.1074	133.0548	76.04818	139.9864	76.02395	148.3076	76.23635	133.9061
76.038	135.7596	76.06102	129.1167	76.12407	133.055	76.06485	139.9849	76.04062	148.3094	76.25302	133.9053
76.05467	135.7559	76.07768	129.1132	76.14073	133.0572	76.08152	139.9864	76.05728	148.3077	76.26968	133.9039
76.07133	135.7548	76.09435	129.1133	76.1574	133.0561	76.09818	139.9838	76.07395	148.3081	76.28635	133.9058
76.088	135.7567	76.11102	129.1118	76.17407	133.0522	76.11485	139.985	76.09062	148.3048	76.30302	133.9045
76.10467	135.7563	76.12768	129.1127	76.19073	133.0545	76.13152	139.9833	76.10728	148.3069	76.31968	133.9031
76.12133	135.753	76.14435	129.1116	76.2074	133.0556	76.14818	139.9839	76.12395	148.3065	76.33635	133.9005
76.138	135.7529	76.16102	129.1101	76.22407	133.0525	76.16485	139.9804	76.14062	148.3072	76.35302	133.9026
76.15467	135.7528	76.17768	129.1076	76.24073	133.0489	76.18152	139.9823	76.15728	148.3065	76.36968	133.9007
76.17133	135.7493	76.19435	129.1061	76.2574	133.0505	76.19818	139.987	76.17395	148.3091	76.38635	133.8997
76.188	135.7508	76.21102	129.1039	76.27407	133.0529	76.21485	139.9859	76.19062	148.3064	76.40302	133.899
76.20467	135.7481	76.22768	129.1047	76.29073	133.05	76.23152	139.9803	76.20728	148.3054	76.41968	133.8972
76.22133	135.7455	76.24435	129.1025	76.3074	133.0486	76.24818	139.9813	76.22395	148.3057	76.43635	133.8963
76.238	135.7463	76.26102	129.1018	76.32407	133.0497	76.26485	139.9823	76.24062	148.3082	76.45302	133.899
76.25467	135.7445	76.27768	129.1021	76.34073	133.0493	76.28152	139.9808	76.25728	148.3066	76.46968	133.8949
76.27133	135.7444	76.29435	129.1001	76.3574	133.0469	76.29818	139.9813	76.27395	148.3068	76.48635	133.8943
76.288	135.7419	76.31102	129.0979	76.37407	133.046	76.31485	139.9787	76.29062	148.305	76.50302	133.895
76.30467	135.7402	76.32768	129.0973	76.39073	133.0494	76.33152	139.9786	76.30728	148.31	76.51968	133.8954
76.32133	135.7412	76.34435	129.0996	76.4074	133.0489	76.34818	139.9783	76.32395	148.306	76.53635	133.8941
76.338	135.7412	76.36102	129.0971	76.42407	133.0485	76.36485	139.979	76.34062	148.3085	76.55302	133.8952
76.35467	135.74	76.37768	129.0944	76.44073	133.0462	76.38152	139.9797	76.35728	148.3084	76.56968	133.8928
76.37133	135.7387	76.39435	129.0944	76.4574	133.0456	76.39818	139.9758	76.37395	148.307	76.58635	133.893
76.388	135.7357	76.41102	129.0918	76.47407	133.0446	76.41485	139.9761	76.39062	148.3062	76.60302	133.8925
76.40467	135.735	76.42768	129.0919	76.49073	133.0459	76.43152	139.9734	76.40728	148.3052	76.61968	133.8919
76.42133	135.735	76.44435	129.0934	76.5074	133.0446	76.44818	139.9747	76.42395	148.3086	76.63635	133.8915
76.438	135.7319	76.46102	129.0902	76.52407	133.0479	76.46485	139.9741	76.44062	148.3086	76.65302	133.888
76.45467	135.7301	76.47768	129.0907	76.54073	133.0425	76.48152	139.973	76.45728	148.3083	76.66968	133.8914
76.47133	135.7306	76.49435	129.0924	76.5574	133.0449	76.49818	139.9713	76.47395	148.3074	76.68635	133.8904
76.488	135.7297	76.51102	129.0922	76.57407	133.042	76.51485	139.9737	76.49062	148.3076	76.70302	133.888
76.50467	135.7303	76.52768	129.0912	76.59073	133.0442	76.53152	139.9717	76.50728	148.3087	76.71968	133.8902
76.52133	135.7278	76.54435	129.0888	76.6074	133.0438	76.54818	139.9711	76.52395	148.3088	76.73635	133.8855
76.538	135.7299	76.56102	129.0882	76.62407	133.0427	76.56485	139.9676	76.54062	148.308	76.75302	133.8872
76.55467	135.7284	76.57768	129.0834	76.64073	133.0425	76.58152	139.9694	76.55728	148.3074	76.76968	133.887
76.57133	135.7283	76.59435	129.0825	76.6574	133.0427	76.59818	139.9701	76.57395	148.3074	76.78635	133.8855
76.588	135.7254	76.61102	129.0832	76.67407	133.0433	76.61485	139.9683	76.59062	148.3076	76.80302	133.8826
76.60467	135.727	76.62768	129.0807	76.69073	133.043	76.63152	139.9649	76.60728	148.308	76.81968	133.8857
76.62133	135.7212	76.64435	129.0806	76.7074	133.0406	76.64818	139.9669	76.62395	148.303	76.83635	133.8875
76.638	135.7212	76.66102	129.0804	76.72407	133.041	76.66485	139.9677	76.64062	148.3047	76.85302	133.8823
76.65467	135.7178	76.67768	129.081	76.74073	133.0412	76.68152	139.9668	76.65728	148.3037	76.86968	133.8854
76.67133	135.7185	76.69435	129.0804	76.7574	133.0405	76.69818	139.9627	76.67395	148.3036	76.88635	133.8869
76.688	135.7167	76.71102	129.0797	76.77407	133.0392	76.71485	139.9633	76.69062	148.3027	76.90302	133.8826
76.70467	135.7164	76.72768	129.0782	76.79073	133.0425	76.73152	139.964	76.70728	148.3039	76.91968	133.8806
76.72133	135.7145	76.74435	129.0729	76.8074	133.0391	76.74818	139.9636	76.72395	148.3061	76.93635	133.8815
76.738	135.713	76.76102	129.0778	76.82407	133.0416	76.76485	139.9622	76.74062	148.3038	76.95302	133.8835
76.75467	135.7138	76.77768	129.0754	76.84073	133.0372	76.78152	139.9633	76.75728	148.3006	76.96968	133.8791
76.77133	135.7131	76.79435	129.0743	76.8574	133.039	76.79818	139.9615	76.77395	148.3036	76.98635	133.8811
76.788	135.7099	76.81102	129.0728	76.87407	133.0398	76.81485	139.9624	76.79062	148.3024	77.00302	133.8774
76.80467	135.7083	76.82768	129.0719	76.89073	133.0372	76.83152	139.9635	76.80728	148.3013	77.01968	133.8782
76.82133	135.7109	76.84435	129.0732	76.9074	133.036	76.84818	139.9609	76.82395	148.3025	77.03635	133.8795
76.838	135.7079	76.86102	129.0699	76.92407	133.0357	76.86485	139.9621	76.84062	148.3009	77.05302	133.8806
76.85467	135.7048	76.87768	129.0685	76.94073	133.0366	76.88152	139.9622	76.85728	148.2987	77.06968	133.8762
76.87133	135.7046	76.89435	129.0685	76.9574	133.0383	76.89818	139.9611	76.87395	148.2995	77.08635	133.8775
76.888	135.7039	76.91102	129.0677	76.97407	133.0351	76.91485	139.963	76.89062	148.3018	77.10302	133.8784
76.90467	135.7031	76.92768	129.0658	76.99073	133.037	76.93152	139.9635	76.90728	148.2999	77.11968	133.8771
76.92133	135.7042	76.94435	129.0634	77.0074	133.0359	76.94818	139.9589	76.92395	148.2969	77.13635	133.8756
76.938	135.7034	76.96102	129.0644	77.02407	133.0366	76.96485	139.9593	76.94062	148.3011	77.15302	133.8769
76.95467	135.7021	76.97768	129.0639	77.04073	133.0377	76.98152	139.9605	76.95728	148.3028	77.16968	133.876
76.97133	135.699	76.99435	129.0614	77.0574	133.0386	76.99818	139.9573	76.97395	148.2979	77.18635	133.8735
76.988	135.697	77.01102	129.0614	77.07407	133.0368	77.01485	139.9597	76.99062	148.3007	77.20302	133.8715
77.00467	135.6981	77.02768	129.063	77.09073	133.0349	77.03152	139.9595	77.00728	148.3032	77.21968	133.8754
77.02133	135.6952	77.04435	129.0572	77.1074	133.0332	77.04818	139.9599	77.02395	148.2994	77.23635	133.8719

77.038	135.6934	77.06102	129.0564	77.12407	133.0377	77.06485	139.9565	77.04062	148.2999	77.25302	133.8728
77.05467	135.6954	77.07768	129.0565	77.14073	133.037	77.08152	139.9584	77.05728	148.2999	77.26968	133.8706
77.07133	135.6909	77.09435	129.0563	77.1574	133.0356	77.09818	139.9569	77.07395	148.2987	77.28635	133.8754
77.088	135.6928	77.11102	129.0534	77.17407	133.0344	77.11485	139.9546	77.09062	148.2992	77.30302	133.8697
77.10467	135.6878	77.12768	129.0541	77.19073	133.0346	77.13152	139.9564	77.10728	148.2996	77.31968	133.8722
77.12133	135.6857	77.14435	129.0504	77.2074	133.0327	77.14818	139.9549	77.12395	148.301	77.33635	133.8715
77.138	135.6909	77.16102	129.0494	77.22407	133.0349	77.16485	139.9551	77.14062	148.3028	77.35302	133.8696
77.15467	135.6844	77.17768	129.0529	77.24073	133.0343	77.18152	139.9533	77.15728	148.2974	77.36968	133.8711
77.17133	135.6893	77.19435	129.0484	77.2574	133.0367	77.19818	139.9522	77.17395	148.2978	77.38635	133.8699
77.188	135.6845	77.21102	129.0479	77.27407	133.0292	77.21485	139.9527	77.19062	148.2986	77.40302	133.8664
77.20467	135.6865	77.22768	129.0486	77.29073	133.0329	77.23152	139.9543	77.20728	148.2987	77.41968	133.867
77.22133	135.6841	77.24435	129.0472	77.3074	133.0336	77.24818	139.9536	77.22395	148.2995	77.43635	133.8701
77.238	135.6816	77.26102	129.0481	77.32407	133.0336	77.26485	139.9521	77.24062	148.3011	77.45302	133.8673
77.25467	135.6805	77.27768	129.0438	77.34073	133.0341	77.28152	139.9532	77.25728	148.2992	77.46968	133.8663
77.27133	135.6778	77.29435	129.0471	77.3574	133.033	77.29818	139.9504	77.27395	148.3011	77.48635	133.8685
77.288	135.6779	77.31102	129.0451	77.37407	133.0278	77.31485	139.952	77.29062	148.2999	77.50302	133.8672
77.30467	135.6793	77.32768	129.045	77.39073	133.0313	77.33152	139.9513	77.30728	148.3021	77.51968	133.8659
77.32133	135.6789	77.34435	129.0411	77.4074	133.0313	77.34818	139.9509	77.32395	148.2998	77.53635	133.8648
77.338	135.6753	77.36102	129.0413	77.42407	133.0311	77.36485	139.9512	77.34062	148.3008	77.55302	133.8666
77.35467	135.6734	77.37768	129.0425	77.44073	133.0298	77.38152	139.9493	77.35728	148.3026	77.56968	133.8648
77.37133	135.6724	77.39435	129.0381	77.4574	133.0285	77.39818	139.9484	77.37395	148.2994	77.58635	133.8626
77.388	135.671	77.41102	129.0357	77.47407	133.0294	77.41485	139.9495	77.39062	148.3025	77.60302	133.8619
77.40467	135.6731	77.42768	129.0389	77.49073	133.0283	77.43152	139.9474	77.40728	148.3051	77.61968	133.8645
77.42133	135.67	77.44435	129.0353	77.5074	133.027	77.44818	139.9488	77.42395	148.2978	77.63635	133.8636
77.438	135.6697	77.46102	129.0314	77.52407	133.0294	77.46485	139.9456	77.44062	148.2969	77.65302	133.8616
77.45467	135.6684	77.47768	129.0326	77.54073	133.0283	77.48152	139.9461	77.45728	148.3068	77.66968	133.8602
77.47133	135.6666	77.49435	129.0316	77.5574	133.0277	77.49818	139.9427	77.47395	148.3059	77.68635	133.8616
77.488	135.6699	77.51102	129.0311	77.57407	133.0249	77.51485	139.9442	77.49062	148.3032	77.70302	133.8586
77.50467	135.6638	77.52768	129.0358	77.59073	133.0244	77.53152	139.9428	77.50728	148.304	77.71968	133.8569
77.52133	135.6646	77.54435	129.0305	77.6074	133.0259	77.54818	139.9415	77.52395	148.3027	77.73635	133.8561
77.538	135.6692	77.56102	129.0299	77.62407	133.0254	77.56485	139.942	77.54062	148.3049	77.75302	133.858
77.55467	135.6611	77.57768	129.0274	77.64073	133.0279	77.58152	139.9405	77.55728	148.3069	77.76968	133.8549
77.57133	135.6644	77.59435	129.0249	77.6574	133.0263	77.59818	139.9401	77.57395	148.3055	77.78635	133.8547
77.588	135.6627	77.61102	129.0284	77.67407	133.026	77.61485	139.9407	77.59062	148.3061	77.80302	133.8528
77.60467	135.6584	77.62768	129.0258	77.69073	133.028	77.63152	139.9399	77.60728	148.3065	77.81968	133.8542
77.62133	135.6591	77.64435	129.0231	77.7074	133.0251	77.64818	139.9381	77.62395	148.3079	77.83635	133.8504
77.638	135.6561	77.66102	129.0265	77.72407	133.0275	77.66485	139.939	77.64062	148.3106	77.85302	133.851
77.65467	135.6581	77.67768	129.0239	77.74073	133.029	77.68152	139.9393	77.65728	148.3078	77.86968	133.8502
77.67133	135.6572	77.69435	129.0211	77.7574	133.0264	77.69818	139.9375	77.67395	148.3078	77.88635	133.8513
77.688	135.6549	77.71102	129.0235	77.77407	133.0257	77.71485	139.9377	77.69062	148.3074	77.90302	133.8497
77.70467	135.6552	77.72768	129.0215	77.79073	133.0271	77.73152	139.9381	77.70728	148.307	77.91968	133.8495
77.72133	135.6519	77.74435	129.0213	77.8074	133.0263	77.74818	139.937	77.72395	148.3057	77.93635	133.8485
77.738	135.655	77.76102	129.0205	77.82407	133.0247	77.76485	139.9343	77.74062	148.3071	77.95302	133.8499
77.75467	135.6513	77.77768	129.0171	77.84073	133.0257	77.78152	139.935	77.75728	148.3062	77.96968	133.8486
77.77133	135.6507	77.79435	129.0215	77.8574	133.0244	77.79818	139.9348	77.77395	148.3043	77.98635	133.8482
77.788	135.6503	77.81102	129.0156	77.87407	133.0244	77.81485	139.934	77.79062	148.3074	78.00302	133.8451
77.80467	135.6488	77.82768	129.0205	77.89073	133.0256	77.83152	139.9358	77.80728	148.3053	78.01968	133.8486
77.82133	135.6458	77.84435	129.0166	77.9074	133.0252	77.84818	139.9322	77.82395	148.3088	78.03635	133.847
77.838	135.6454	77.86102	129.0132	77.92407	133.0252	77.86485	139.9322	77.84062	148.3071	78.05302	133.8479
77.85467	135.6486	77.87768	129.0165	77.94073	133.0243	77.88152	139.9317	77.85728	148.3078	78.06968	133.8464
77.87133	135.6403	77.89435	129.0103	77.9574	133.0249	77.89818	139.9314	77.87395	148.3089	78.08635	133.845
77.888	135.6407	77.91102	129.0136	77.97407	133.0221	77.91485	139.9299	77.89062	148.3085	78.10302	133.8439
77.90467	135.6425	77.92768	129.0135	77.99073	133.0249	77.93152	139.9292	77.90728	148.3106	78.11968	133.8449
77.92133	135.6443	77.94435	129.0129	78.0074	133.0243	77.94818	139.9301	77.92395	148.31	78.13635	133.8435
77.938	135.6384	77.96102	129.014	78.02407	133.0256	77.96485	139.9305	77.94062	148.3083	78.15302	133.8455
77.95467	135.6385	77.97768	129.0068	78.04073	133.0237	77.98152	139.9277	77.95728	148.3073	78.16968	133.8436
77.97133	135.6378	77.99435	129.0117	78.0574	133.0227	77.99818	139.926	77.97395	148.3097	78.18635	133.8403
77.988	135.6373	78.01102	129.0081	78.07407	133.023	78.01485	139.9277	77.99062	148.3117	78.20302	133.8415
78.00467	135.6338	78.02768	129.0085	78.09073	133.023	78.03152	139.9304	78.00728	148.3108	78.21968	133.8421
78.02133	135.6337	78.04435	129.0065	78.1074	133.023	78.04818	139.9296	78.02395	148.3089	78.23635	133.8393
78.038	135.6306	78.06102	129.0044	78.12407	133.0239	78.06485	139.9276	78.04062	148.3133	78.25302	133.8392
78.05467	135.6318	78.07768	129.0075	78.14073	133.0193	78.08152	139.9253	78.05728	148.3114	78.26968	133.8399
78.07133	135.6291	78.09435	129.006	78.1574	133.0211	78.09818	139.9283	78.07395	148.3114	78.28635	133.8395
78.088	135.6322	78.11102	129.005	78.17407	133.0207	78.11485	139.9261	78.09062	148.3114	78.30302	133.8365
78.10467	135.6275	78.12768	129.0001	78.19073	133.0202	78.13152	139.925	78.10728	148.3105	78.31968	133.8382
78.12133	135.6264	78.14435	129.0031	78.2074	133.0222	78.14818	139.9258	78.12395	148.3127	78.33635	133.8366
78.138	135.6266	78.16102	129.0035	78.22407	133.0199	78.16485	139.9243	78.14062	148.3105	78.35302	133.8369
78.15467	135.6247	78.17768	129.0025	78.24073	133.0156	78.18152	139.9247	78.15728	148.3131	78.36968	133.8347
78.17133	135.6215	78.19435	129.0024	78.2574	133.0178	78.19818	139.9248	78.17395	148.3104	78.38635	133.8338

78.188	135.6248	78.21102	129.0005	78.27407	133.0188	78.21485	139.9215	78.19062	148.3132	78.40302	133.8347
78.20467	135.619	78.22768	129.0026	78.29073	133.0155	78.23152	139.9215	78.20728	148.3127	78.41968	133.8358
78.22133	135.6196	78.24435	128.9978	78.3074	133.0152	78.24818	139.923	78.22395	148.3104	78.43635	133.8339
78.238	135.6182	78.26102	128.9989	78.32407	133.0166	78.26485	139.9227	78.24062	148.3133	78.45302	133.8338
78.25467	135.6182	78.27768	129.0008	78.34073	133.0151	78.28152	139.9211	78.25728	148.312	78.46968	133.8337
78.27133	135.6156	78.29435	128.997	78.3574	133.0149	78.29818	139.9215	78.27395	148.3126	78.48635	133.8328
78.288	135.6159	78.31102	128.9946	78.37407	133.0143	78.31485	139.9208	78.29062	148.3136	78.50302	133.8323
78.30467	135.6142	78.32768	128.9933	78.39073	133.0129	78.33152	139.9193	78.30728	148.3123	78.51968	133.8331
78.32133	135.6117	78.34435	128.9931	78.4074	133.0119	78.34818	139.9226	78.32395	148.3139	78.53635	133.8287
78.338	135.6128	78.36102	128.9927	78.42407	133.0136	78.36485	139.9177	78.34062	148.3128	78.55302	133.8283
78.35467	135.6129	78.37768	128.9911	78.44073	133.0123	78.38152	139.9197	78.35728	148.3128	78.56968	133.8283
78.37133	135.6119	78.39435	128.9917	78.4574	133.0139	78.39818	139.9194	78.37395	148.311	78.58635	133.8272
78.388	135.6085	78.41102	128.9884	78.47407	133.0121	78.41485	139.9194	78.39062	148.3117	78.60302	133.8267
78.40467	135.614	78.42768	128.9873	78.49073	133.0104	78.43152	139.9181	78.40728	148.3123	78.61968	133.826
78.42133	135.6062	78.44435	128.9882	78.5074	133.0084	78.44818	139.9197	78.42395	148.3114	78.63635	133.8253
78.438	135.6064	78.46102	128.9874	78.52407	133.011	78.46485	139.9183	78.44062	148.3135	78.65302	133.8259
78.45467	135.6046	78.47768	128.9847	78.54073	133.0108	78.48152	139.9194	78.45728	148.3131	78.66968	133.8243
78.47133	135.6081	78.49435	128.9862	78.5574	133.0104	78.49818	139.9168	78.47395	148.3136	78.68635	133.8271
78.488	135.6062	78.51102	128.9857	78.57407	133.0091	78.51485	139.9174	78.49062	148.3144	78.70302	133.8224
78.50467	135.6044	78.52768	128.9859	78.59073	133.0086	78.53152	139.916	78.50728	148.3129	78.71968	133.8235
78.52133	135.6004	78.54435	128.984	78.6074	133.0072	78.54818	139.9158	78.52395	148.3139	78.73635	133.8217
78.538	135.6007	78.56102	128.9832	78.62407	133.0106	78.56485	139.9158	78.54062	148.3133	78.75302	133.8219
78.55467	135.5971	78.57768	128.9821	78.64073	133.0075	78.58152	139.9146	78.55728	148.3133	78.76968	133.8214
78.57133	135.6001	78.59435	128.9823	78.6574	133.0058	78.59818	139.9152	78.57395	148.3141	78.78635	133.8235
78.588	135.6001	78.61102	128.9789	78.67407	133.005	78.61485	139.9138	78.59062	148.315	78.80302	133.8189
78.60467	135.5966	78.62768	128.9781	78.69073	133.0068	78.63152	139.9136	78.60728	148.3177	78.81968	133.8209
78.62133	135.5949	78.64435	128.9775	78.7074	133.0064	78.64818	139.9139	78.62395	148.3162	78.83635	133.8212
78.638	135.5978	78.66102	128.9766	78.72407	133.006	78.66485	139.9121	78.64062	148.3155	78.85302	133.8205
78.65467	135.5945	78.67768	128.9753	78.74073	133.004	78.68152	139.911	78.65728	148.3142	78.86968	133.8183
78.67133	135.5884	78.69435	128.9741	78.7574	133.0034	78.69818	139.9119	78.67395	148.3149	78.88635	133.8204
78.688	135.5899	78.71102	128.9742	78.77407	133.0015	78.71485	139.9108	78.69062	148.3161	78.90302	133.816
78.70467	135.5857	78.72768	128.9733	78.79073	133.0036	78.73152	139.9113	78.70728	148.315	78.91968	133.8177
78.72133	135.5876	78.74435	128.9719	78.8074	133.0041	78.74818	139.9087	78.72395	148.316	78.93635	133.8142
78.738	135.5879	78.76102	128.9691	78.82407	133.0008	78.76485	139.9089	78.74062	148.3208	78.95302	133.8173
78.75467	135.5861	78.77768	128.9724	78.84073	133.0007	78.78152	139.9101	78.75728	148.318	78.96968	133.8152
78.77133	135.5836	78.79435	128.9701	78.8574	133.0003	78.79818	139.9107	78.77395	148.3192	78.98635	133.8139
78.788	135.5834	78.81102	128.9683	78.87407	132.9999	78.81485	139.9091	78.79062	148.3209	79.00302	133.8138
78.80467	135.586	78.82768	128.9677	78.89073	133.0009	78.83152	139.9085	78.80728	148.3189	79.01968	133.8139
78.82133	135.5821	78.84435	128.965	78.9074	132.9984	78.84818	139.91	78.82395	148.3204	79.03635	133.8126
78.838	135.5812	78.86102	128.9628	78.92407	132.9988	78.86485	139.9085	78.84062	148.3198	79.05302	133.8144
78.85467	135.5796	78.87768	128.9648	78.94073	132.9984	78.88152	139.9064	78.85728	148.3206	79.06968	133.8103
78.87133	135.5807	78.89435	128.9625	78.9574	132.9969	78.89818	139.9075	78.87395	148.3219	79.08635	133.8125
78.888	135.5819	78.91102	128.9624	78.97407	132.9974	78.91485	139.9066	78.89062	148.3215	79.10302	133.8106
78.90467	135.5759	78.92768	128.9622	78.99073	132.9979	78.93152	139.9062	78.90728	148.3248	79.11968	133.8094
78.92133	135.5763	78.94435	128.9594	79.0074	132.9953	78.94818	139.9051	78.92395	148.325	79.13635	133.8083
78.938	135.5728	78.96102	128.9614	79.02407	132.9953	78.96485	139.9065	78.94062	148.3227	79.15302	133.8115
78.95467	135.5731	78.97768	128.9588	79.04073	132.9949	78.98152	139.9052	78.95728	148.328	79.16968	133.8092
78.97133	135.5745	78.99435	128.958	79.0574	132.9951	78.99818	139.9051	78.97395	148.3242	79.18635	133.8093
78.988	135.5767	79.01102	128.9578	79.07407	132.9934	79.01485	139.9015	78.99062	148.3268	79.20302	133.8076
79.00467	135.5658	79.02768	128.9593	79.09073	132.9926	79.03152	139.905	79.00728	148.3217	79.21968	133.8066
79.02133	135.5686	79.04435	128.953	79.1074	132.9898	79.04818	139.9035	79.02395	148.3248	79.23635	133.807
79.038	135.5648	79.06102	128.9525	79.12407	132.9941	79.06485	139.9044	79.04062	148.324	79.25302	133.8086
79.05467	135.5673	79.07768	128.9577	79.14073	132.9893	79.08152	139.8994	79.05728	148.3277	79.26968	133.8081
79.07133	135.5662	79.09435	128.9508	79.1574	132.9866	79.09818	139.9032	79.07395	148.3267	79.28635	133.8059
79.088	135.5641	79.11102	128.9526	79.17407	132.9907	79.11485	139.9023	79.09062	148.3261	79.30302	133.807
79.10467	135.5634	79.12768	128.9541	79.19073	132.9875	79.13152	139.901	79.10728	148.3252	79.31968	133.8078
79.12133	135.5639	79.14435	128.9515	79.2074	132.9881	79.14818	139.8998	79.12395	148.3281	79.33635	133.8091
79.138	135.5608	79.16102	128.9514	79.22407	132.9862	79.16485	139.9008	79.14062	148.3274	79.35302	133.806
79.15467	135.5635	79.17768	128.951	79.24073	132.9885	79.18152	139.8998	79.15728	148.3296	79.36968	133.8047
79.17133	135.5598	79.19435	128.9515	79.2574	132.9883	79.19818	139.8986	79.17395	148.327	79.38635	133.806
79.188	135.5599	79.21102	128.9513	79.27407	132.9873	79.21485	139.8988	79.19062	148.3279	79.40302	133.8034
79.20467	135.5592	79.22768	128.9498	79.29073	132.9867	79.23152	139.8972	79.20728	148.3233	79.41968	133.8045
79.22133	135.559	79.24435	128.9488	79.3074	132.9849	79.24818	139.8964	79.22395	148.3317	79.43635	133.801
79.238	135.5568	79.26102	128.9464	79.32407	132.985	79.26485	139.8936	79.24062	148.3254	79.45302	133.8034
79.25467	135.554	79.27768	128.9465	79.34073	132.9857	79.28152	139.8921	79.25728	148.3236	79.46968	133.8007
79.27133	135.5492	79.29435	128.9453	79.3574	132.9848	79.29818	139.8917	79.27395	148.3254	79.48635	133.8028
79.288	135.5514	79.31102	128.9437	79.37407	132.9856	79.31485	139.8934	79.29062	148.3239	79.50302	133.7992
79.30467	135.5509	79.32768	128.942	79.39073	132.9831	79.33152	139.8913	79.30728	148.3238	79.51968	133.8007
79.32133	135.5495	79.34435	128.9433	79.4074	132.983	79.34818	139.892	79.32395	148.3242	79.53635	133.7991

79.338	135.5488	79.36102	128.9397	79.42407	132.9817	79.36485	139.8892	79.34062	148.3237	79.55302	133.7977
79.35467	135.5502	79.37768	128.94	79.44073	132.9816	79.38152	139.8915	79.35728	148.3246	79.56968	133.7976
79.37133	135.545	79.39435	128.9416	79.4574	132.981	79.39818	139.8889	79.37395	148.3234	79.58635	133.7964
79.388	135.544	79.41102	128.9399	79.47407	132.9805	79.41485	139.89	79.39062	148.3235	79.60302	133.796
79.40467	135.5437	79.42768	128.9405	79.49073	132.9787	79.43152	139.8883	79.40728	148.3238	79.61968	133.7945
79.42133	135.5411	79.44435	128.9381	79.5074	132.9766	79.44818	139.8879	79.42395	148.3197	79.63635	133.7946
79.438	135.5418	79.46102	128.9371	79.52407	132.979	79.46485	139.8909	79.44062	148.3219	79.65302	133.7926
79.45467	135.5394	79.47768	128.9366	79.54073	132.9792	79.48152	139.8866	79.45728	148.3217	79.66968	133.7953
79.47133	135.5423	79.49435	128.9342	79.5574	132.9797	79.49818	139.8867	79.47395	148.3205	79.68635	133.7927
79.488	135.5383	79.51102	128.9357	79.57407	132.9764	79.51485	139.8872	79.49062	148.3234	79.70302	133.7918
79.50467	135.5338	79.52768	128.9341	79.59073	132.9769	79.53152	139.8884	79.50728	148.3204	79.71968	133.7929
79.52133	135.5355	79.54435	128.9348	79.6074	132.9762	79.54818	139.8879	79.52395	148.3229	79.73635	133.7934
79.538	135.5327	79.56102	128.9314	79.62407	132.9751	79.56485	139.8863	79.54062	148.3219	79.75302	133.7928
79.55467	135.5292	79.57768	128.9289	79.64073	132.9754	79.58152	139.8851	79.55728	148.3232	79.76968	133.7915
79.57133	135.5315	79.59435	128.9291	79.6574	132.9719	79.59818	139.8829	79.57395	148.3247	79.78635	133.7902
79.588	135.5289	79.61102	128.9315	79.67407	132.9723	79.61485	139.8843	79.59062	148.3227	79.80302	133.7899
79.60467	135.533	79.62768	128.932	79.69073	132.9728	79.63152	139.8835	79.60728	148.326	79.81968	133.7903
79.62133	135.5283	79.64435	128.9284	79.7074	132.9728	79.64818	139.8837	79.62395	148.3261	79.83635	133.7886
79.638	135.529	79.66102	128.9276	79.72407	132.9713	79.66485	139.8833	79.64062	148.3243	79.85302	133.79
79.65467	135.5254	79.67768	128.9286	79.74073	132.9707	79.68152	139.8836	79.65728	148.3261	79.86968	133.7869
79.67133	135.5236	79.69435	128.9271	79.7574	132.9717	79.69818	139.8814	79.67395	148.3241	79.88635	133.7869
79.688	135.5236	79.71102	128.9247	79.77407	132.9705	79.71485	139.8826	79.69062	148.3257	79.90302	133.7883
79.70467	135.5229	79.72768	128.9241	79.79073	132.9678	79.73152	139.8806	79.70728	148.3249	79.91968	133.7836
79.72133	135.5197	79.74435	128.9259	79.8074	132.9685	79.74818	139.881	79.72395	148.3246	79.93635	133.7859
79.738	135.5211	79.76102	128.9227	79.82407	132.9713	79.76485	139.8796	79.74062	148.324	79.95302	133.7862
79.75467	135.5186	79.77768	128.9259	79.84073	132.9701	79.78152	139.8799	79.75728	148.3222	79.96968	133.7845
79.77133	135.5206	79.79435	128.924	79.8574	132.9674	79.79818	139.8771	79.77395	148.3251	79.98635	133.7801
79.788	135.5175	79.81102	128.9213	79.87407	132.9645	79.81485	139.8793	79.79062	148.3239	80.00302	133.7854
79.80467	135.5196	79.82768	128.9217	79.89073	132.9689	79.83152	139.8785	79.80728	148.3264	80.01968	133.784
79.82133	135.5177	79.84435	128.919	79.9074	132.9676	79.84818	139.876	79.82395	148.3269	80.03635	133.7804
79.838	135.517	79.86102	128.919	79.92407	132.9678	79.86485	139.8767	79.84062	148.3271	80.05302	133.7785
79.85467	135.5122	79.87768	128.9178	79.94073	132.9682	79.88152	139.8755	79.85728	148.325	80.06968	133.781
79.87133	135.5113	79.89435	128.9183	79.9574	132.9683	79.89818	139.8754	79.87395	148.3272	80.08635	133.7774
79.888	135.5117	79.91102	128.9182	79.97407	132.9649	79.91485	139.8734	79.89062	148.3267	80.10302	133.7767
79.90467	135.5108	79.92768	128.9157	79.99073	132.9651	79.93152	139.8746	79.90728	148.3242	80.11968	133.7757
79.92133	135.5076	79.94435	128.9152	80.0074	132.9641	79.94818	139.8723	79.92395	148.3241	80.13635	133.7771
79.938	135.5099	79.96102	128.9138	80.02407	132.9641	79.96485	139.8732	79.94062	148.3232	80.15302	133.7756
79.95467	135.5051	79.97768	128.9121	80.04073	132.9647	79.98152	139.8751	79.95728	148.327	80.16968	133.7745
79.97133	135.5058	79.99435	128.9132	80.0574	132.9644	79.99818	139.87	79.97395	148.3235	80.18635	133.7766
79.988	135.5043	80.01102	128.9135	80.07407	132.9647	80.01485	139.8719	79.99062	148.3258	80.20302	133.7737
80.00467	135.5002	80.02768	128.9132	80.09073	132.9644	80.03152	139.8719	80.00728	148.3263	80.21968	133.7729
80.02133	135.5036	80.04435	128.911	80.1074	132.9615	80.04818	139.8726	80.02395	148.3267	80.23635	133.7731
80.038	135.5001	80.06102	128.9076	80.12407	132.9612	80.06485	139.8722	80.04062	148.3265	80.25302	133.7707
80.05467	135.5005	80.07768	128.9104	80.14073	132.9601	80.08152	139.8719	80.05728	148.3246	80.26968	133.7673
80.07133	135.4998	80.09435	128.9072	80.1574	132.9586	80.09818	139.8715	80.07395	148.3258	80.28635	133.7674
80.088	135.4983	80.11102	128.9064	80.17407	132.9551	80.11485	139.8718	80.09062	148.3228	80.30302	133.7682
80.10467	135.4981	80.12768	128.9078	80.19073	132.958	80.13152	139.8702	80.10728	148.3241	80.31968	133.766
80.12133	135.4943	80.14435	128.9063	80.2074	132.9543	80.14818	139.8722	80.12395	148.3238	80.33635	133.7658
80.138	135.4938	80.16102	128.9042	80.22407	132.9573	80.16485	139.8705	80.14062	148.3256	80.35302	133.7671
80.15467	135.4912	80.17768	128.9062	80.24073	132.9559	80.18152	139.8702	80.15728	148.3257	80.36968	133.7656
80.17133	135.4898	80.19435	128.9056	80.2574	132.9556	80.19818	139.8703	80.17395	148.3255	80.38635	133.7654
80.188	135.4907	80.21102	128.9037	80.27407	132.9571	80.21485	139.8698	80.19062	148.326	80.40302	133.7651
80.20467	135.4889	80.22768	128.9027	80.29073	132.9515	80.23152	139.8699	80.20728	148.3257	80.41968	133.7646
80.22133	135.4859	80.24435	128.9038	80.3074	132.9532	80.24818	139.8697	80.22395	148.3241	80.43635	133.7619
80.238	135.488	80.26102	128.9018	80.32407	132.9549	80.26485	139.8675	80.24062	148.3235	80.45302	133.7619
80.25467	135.4833	80.27768	128.9023	80.34073	132.9555	80.28152	139.8672	80.25728	148.3246	80.46968	133.7653
80.27133	135.4816	80.29435	128.8992	80.3574	132.954	80.29818	139.8675	80.27395	148.3243	80.48635	133.7622
80.288	135.4824	80.31102	128.8978	80.37407	132.9519	80.31485	139.8664	80.29062	148.3253	80.50302	133.7635
80.30467	135.481	80.32768	128.899	80.39073	132.9501	80.33152	139.8643	80.30728	148.3251	80.51968	133.7616
80.32133	135.4788	80.34435	128.8977	80.4074	132.953	80.34818	139.8663	80.32395	148.3235	80.53635	133.7628
80.338	135.4783	80.36102	128.8988	80.42407	132.953	80.36485	139.866	80.34062	148.3258	80.55302	133.7624
80.35467	135.4797	80.37768	128.8956	80.44073	132.9509	80.38152	139.8643	80.35728	148.3249	80.56968	133.7614
80.37133	135.4762	80.39435	128.8949	80.4574	132.9504	80.39818	139.8635	80.37395	148.3254	80.58635	133.7611
80.388	135.4738	80.41102	128.8942	80.47407	132.9511	80.41485	139.8629	80.39062	148.3257	80.60302	133.7598
80.40467	135.4752	80.42768	128.8916	80.49073	132.9489	80.43152	139.8628	80.40728	148.3255	80.61968	133.7601
80.42133	135.4724	80.44435	128.8931	80.5074	132.9482	80.44818	139.8651	80.42395	148.3229	80.63635	133.7573
80.438	135.4724	80.46102	128.8915	80.52407	132.9487	80.46485	139.8623	80.44062	148.326	80.65302	133.7608
80.45467	135.472	80.47768	128.8915	80.54073	132.9458	80.48152	139.8643	80.45728	148.326	80.66968	133.7582
80.47133	135.4701	80.49435	128.8921	80.5574	132.9476	80.49818	139.8613	80.47395	148.3247	80.68635	133.7558

80.488	135.4687	80.51102	128.8935	80.57407	132.9476	80.51485	139.8629	80.49062	148.3284	80.70302	133.7593
80.50467	135.4675	80.52768	128.8898	80.59073	132.9462	80.53152	139.8602	80.50728	148.3275	80.71968	133.7575
80.52133	135.4652	80.54435	128.8912	80.6074	132.9466	80.54818	139.8618	80.52395	148.3288	80.73635	133.7579
80.538	135.4652	80.56102	128.8879	80.62407	132.9481	80.56485	139.8586	80.54062	148.3279	80.75302	133.7571
80.55467	135.4603	80.57768	128.8879	80.64073	132.9478	80.58152	139.8589	80.55728	148.327	80.76968	133.7556
80.57133	135.4629	80.59435	128.8864	80.6574	132.9454	80.59818	139.8601	80.57395	148.3278	80.78635	133.7569
80.588	135.4611	80.61102	128.8883	80.67407	132.9459	80.61485	139.8556	80.59062	148.3281	80.80302	133.7552
80.60467	135.4601	80.62768	128.8867	80.69073	132.9435	80.63152	139.8585	80.60728	148.3264	80.81968	133.7554
80.62133	135.4598	80.64435	128.8839	80.7074	132.9454	80.64818	139.8559	80.62395	148.326	80.83635	133.7547
80.638	135.4605	80.66102	128.8833	80.72407	132.9434	80.66485	139.8575	80.64062	148.3255	80.85302	133.7526
80.65467	135.4586	80.67768	128.8846	80.74073	132.9441	80.68152	139.8563	80.65728	148.3237	80.86968	133.7528
80.67133	135.4595	80.69435	128.8836	80.7574	132.945	80.69818	139.8532	80.67395	148.3231	80.88635	133.7535
80.688	135.4587	80.71102	128.8832	80.77407	132.9438	80.71485	139.8529	80.69062	148.3273	80.90302	133.7519
80.70467	135.4552	80.72768	128.8835	80.79073	132.9419	80.73152	139.8517	80.70728	148.3262	80.91968	133.7531
80.72133	135.4552	80.74435	128.8833	80.8074	132.9387	80.74818	139.8529	80.72395	148.3215	80.93635	133.7501
80.738	135.454	80.76102	128.8801	80.82407	132.9455	80.76485	139.8506	80.74062	148.3225	80.95302	133.7505
80.75467	135.4516	80.77768	128.8768	80.84073	132.9395	80.78152	139.8517	80.75728	148.3243	80.96968	133.7525
80.77133	135.4512	80.79435	128.88	80.8574	132.9407	80.79818	139.8505	80.77395	148.3242	80.98635	133.7485
80.788	135.448	80.81102	128.8819	80.87407	132.9399	80.81485	139.8492	80.79062	148.3247	81.00302	133.7493
80.80467	135.447	80.82768	128.8777	80.89073	132.9388	80.83152	139.8514	80.80728	148.3259	81.01968	133.746
80.82133	135.4478	80.84435	128.878	80.9074	132.9419	80.84818	139.8502	80.82395	148.3249	81.03635	133.7465
80.838	135.4477	80.86102	128.8775	80.92407	132.9401	80.86485	139.8509	80.84062	148.3266	81.05302	133.7433
80.85467	135.4472	80.87768	128.8767	80.94073	132.9372	80.88152	139.8511	80.85728	148.3225	81.06968	133.7463
80.87133	135.4472	80.89435	128.8781	80.9574	132.937	80.89818	139.8491	80.87395	148.3267	81.08635	133.7469
80.888	135.4456	80.91102	128.8767	80.97407	132.9347	80.91485	139.8525	80.89062	148.3256	81.10302	133.7441
80.90467	135.4429	80.92768	128.8708	80.99073	132.9338	80.93152	139.85	80.90728	148.3231	81.11968	133.7407
80.92133	135.4403	80.94435	128.8793	81.0074	132.9359	80.94818	139.8503	80.92395	148.3267	81.13635	133.7416
80.938	135.4423	80.96102	128.8736	81.02407	132.9347	80.96485	139.8517	80.94062	148.3272	81.15302	133.7426
80.95467	135.439	80.97768	128.8682	81.04073	132.933	80.98152	139.8487	80.95728	148.3279	81.16968	133.7411
80.97133	135.4423	80.99435	128.8718	81.0574	132.9313	80.99818	139.8502	80.97395	148.3265	81.18635	133.7387
80.988	135.435	81.01102	128.8737	81.07407	132.9312	81.01485	139.8482	80.99062	148.3272	81.20302	133.7404
81.00467	135.4374	81.02768	128.8674	81.09073	132.9311	81.03152	139.8506	81.00728	148.3267	81.21968	133.7387
81.02133	135.4392	81.04435	128.8704	81.1074	132.9332	81.04818	139.8483	81.02395	148.3267	81.23635	133.7392
81.038	135.4339	81.06102	128.8713	81.12407	132.9333	81.06485	139.8484	81.04062	148.3227	81.25302	133.7384
81.05467	135.4314	81.07768	128.8676	81.14073	132.9325	81.08152	139.8497	81.05728	148.331	81.26968	133.7397
81.07133	135.4349	81.09435	128.8696	81.1574	132.9327	81.09818	139.8466	81.07395	148.328	81.28635	133.7374
81.088	135.4319	81.11102	128.8671	81.17407	132.9334	81.11485	139.8485	81.09062	148.3297	81.30302	133.7338
81.10467	135.4295	81.12768	128.8678	81.19073	132.9319	81.13152	139.8493	81.10728	148.326	81.31968	133.7364
81.12133	135.4305	81.14435	128.8683	81.2074	132.932	81.14818	139.8474	81.12395	148.3273	81.33635	133.7347
81.138	135.4271	81.16102	128.8655	81.22407	132.9325	81.16485	139.8493	81.14062	148.3296	81.35302	133.7377
81.15467	135.4269	81.17768	128.8653	81.24073	132.9313	81.18152	139.8468	81.15728	148.3288	81.36968	133.7344
81.17133	135.4241	81.19435	128.8634	81.2574	132.9306	81.19818	139.8487	81.17395	148.3298	81.38635	133.734
81.188	135.4243	81.21102	128.8647	81.27407	132.9314	81.21485	139.8456	81.19062	148.3298	81.40302	133.7336
81.20467	135.4225	81.22768	128.8644	81.29073	132.932	81.23152	139.8475	81.20728	148.3283	81.41968	133.7321
81.22133	135.4242	81.24435	128.8666	81.3074	132.9328	81.24818	139.8476	81.22395	148.3329	81.43635	133.7315
81.238	135.4217	81.26102	128.864	81.32407	132.9314	81.26485	139.8466	81.24062	148.3285	81.45302	133.7298
81.25467	135.4213	81.27768	128.865	81.34073	132.9321	81.28152	139.8465	81.25728	148.3296	81.46968	133.7289
81.27133	135.4179	81.29435	128.8639	81.3574	132.9319	81.29818	139.8455	81.27395	148.3292	81.48635	133.7311
81.288	135.4183	81.31102	128.8659	81.37407	132.9329	81.31485	139.8437	81.29062	148.3289	81.50302	133.7282
81.30467	135.4178	81.32768	128.8688	81.39073	132.9321	81.33152	139.8431	81.30728	148.33	81.51968	133.7255
81.32133	135.4167	81.34435	128.8735	81.4074	132.9309	81.34818	139.8445	81.32395	148.3311	81.53635	133.7254
81.338	135.4129	81.36102	129.2853	81.42407	132.9348	81.36485	139.8433	81.34062	148.3305	81.55302	133.7233
81.35467	135.4127	81.36508	129.7991	81.44073	132.9345	81.38152	139.8405	81.35728	148.3323	81.56968	133.7234
81.37133	135.4111	81.36527	130.3091	81.4574	132.9308	81.39818	139.8422	81.37395	148.3332	81.58635	133.7223
81.388	135.4096	81.36553	130.8254	81.47407	132.9326	81.41485	139.8409	81.39062	148.3328	81.60302	133.7212
81.40467	135.4115	81.3822	130.8261	81.49073	132.9334	81.43152	139.8402	81.40728	148.3299	81.61968	133.7193
81.42133	135.4102	81.39887	130.7926	81.5074	132.933	81.44818	139.839	81.42395	148.3333	81.63635	133.7224
81.438	135.4084	81.41553	130.7742	81.52407	132.9304	81.46485	139.8394	81.44062	148.3319	81.65302	133.7196
81.45467	135.4064	81.4322	130.7548	81.54073	132.9314	81.48152	139.8368	81.45728	148.3335	81.66968	133.7229
81.47133	135.4066	81.44887	130.7457	81.5574	132.9295	81.49818	139.8386	81.47395	148.3313	81.68635	133.7195
81.488	135.4018	81.46553	130.736	81.57407	132.9316	81.51485	139.8356	81.49062	148.3343	81.70302	133.7206
81.50467	135.4064	81.4822	130.7263	81.59073	132.9259	81.53152	139.835	81.50728	148.3337	81.71968	133.7194
81.52133	135.4039	81.49887	130.7181	81.6074	132.9294	81.54818	139.8365	81.52395	148.331	81.73635	133.7195
81.538	135.3971	81.51553	130.7136	81.62407	132.9273	81.56485	139.8331	81.54062	148.3322	81.75302	133.7201
81.55467	135.4021	81.5322	130.705	81.64073	132.9272	81.58152	139.8361	81.55728	148.3311	81.76968	133.718
81.57133	135.3962	81.54887	130.7019	81.6574	132.9274	81.59818	139.8363	81.57395	148.3302	81.78635	133.7196
81.588	135.3976	81.56553	130.697	81.67407	132.9255	81.61485	139.8349	81.59062	148.331	81.80302	133.7188
81.60467	135.3966	81.5822	130.6904	81.69073	132.9248	81.63152	139.8316	81.60728	148.3296	81.81968	133.7195
81.62133	135.3925	81.59887	130.6847	81.7074	132.9269	81.64818	139.8328	81.62395	148.3312	81.83635	133.7179

81.638	135.3959	81.61553	130.6798	81.72407	132.9245	81.66485	139.8358	81.64062	148.3297	81.85302	133.717
81.65467	135.3919	81.6322	130.6756	81.74073	132.9242	81.68152	139.8356	81.65728	148.3295	81.86968	133.719
81.67133	135.3877	81.64887	130.6707	81.7574	132.9228	81.69818	139.8347	81.67395	148.33	81.88635	133.7201
81.688	135.3901	81.66553	130.6679	81.77407	132.922	81.71485	139.8316	81.69062	148.3301	81.90302	133.7173
81.70467	135.3888	81.6822	130.6633	81.79073	132.9203	81.73152	139.8328	81.70728	148.3304	81.91968	133.7149
81.72133	135.3899	81.69887	130.6597	81.8074	132.9224	81.74818	139.8325	81.72395	148.3282	81.93635	133.7154
81.738	135.3882	81.71553	130.6548	81.82407	132.9216	81.76485	139.8311	81.74062	148.3317	81.95302	133.7149
81.75467	135.3851	81.7322	130.6529	81.84073	132.922	81.78152	139.8319	81.75728	148.3296	81.96968	133.7142
81.77133	135.3851	81.74887	130.65	81.8574	132.9209	81.79818	139.8293	81.77395	148.3293	81.98635	133.7136
81.788	135.3832	81.76553	130.646	81.87407	132.9188	81.81485	139.8286	81.79062	148.3279	82.00302	133.7122
81.80467	135.3845	81.7822	130.6403	81.89073	132.9222	81.83152	139.8308	81.80728	148.3271	82.01968	133.712
81.82133	135.3806	81.79887	130.6394	81.9074	132.9158	81.84818	139.8292	81.82395	148.3298	82.03635	133.7096
81.838	135.3822	81.81553	130.6344	81.92407	132.9196	81.86485	139.8281	81.84062	148.3275	82.05302	133.7109
81.85467	135.3801	81.8322	130.631	81.94073	132.917	81.88152	139.8285	81.85728	148.3298	82.06968	133.7074
81.87133	135.3764	81.84887	130.6284	81.9574	132.9194	81.89818	139.827	81.87395	148.3298	82.08635	133.7107
81.888	135.3765	81.86553	130.623	81.97407	132.9172	81.91485	139.8253	81.89062	148.3306	82.10302	133.7095
81.90467	135.3742	81.8822	130.6219	81.99073	132.9168	81.93152	139.8244	81.90728	148.332	82.11968	133.7088
81.92133	135.3765	81.89887	130.6175	82.0074	132.917	81.94818	139.8242	81.92395	148.3316	82.13635	133.7075
81.938	135.3707	81.91553	130.6159	82.02407	132.9148	81.96485	139.8253	81.94062	148.3298	82.15302	133.7077
81.95467	135.374	81.9322	130.6135	82.04073	132.9175	81.98152	139.8235	81.95728	148.3316	82.16968	133.7079
81.97133	135.3709	81.94887	130.6115	82.0574	132.9169	81.99818	139.8245	81.97395	148.3323	82.18635	133.7068
81.988	135.3633	81.96553	130.6105	82.07407	132.9188	82.01485	139.8257	81.99062	148.3319	82.20302	133.7049
82.00467	135.3677	81.9822	130.6046	82.09073	132.9161	82.03152	139.8241	82.00728	148.3301	82.21968	133.7057
82.02133	135.3704	81.99887	130.6051	82.1074	132.9165	82.04818	139.8249	82.02395	148.3319	82.23635	133.708
82.038	135.3651	82.01553	130.6005	82.12407	132.9154	82.06485	139.826	82.04062	148.3304	82.25302	133.7055
82.05467	135.3631	82.0322	130.5978	82.14073	132.9154	82.08152	139.8232	82.05728	148.3316	82.26968	133.7049
82.07133	135.3617	82.04887	130.5961	82.1574	132.9127	82.09818	139.8246	82.07395	148.3319	82.28635	133.7047
82.088	135.3631	82.06553	130.5958	82.17407	132.9171	82.11485	139.8247	82.09062	148.3332	82.30302	133.705
82.10467	135.3665	82.0822	130.5921	82.19073	132.9152	82.13152	139.8243	82.10728	148.3325	82.31968	133.7022
82.12133	135.3594	82.09887	130.5933	82.2074	132.9134	82.14818	139.8249	82.12395	148.3351	82.33635	133.7055
82.138	135.358	82.11553	130.5892	82.22407	132.9131	82.16485	139.8239	82.14062	148.3317	82.35302	133.7011
82.15467	135.3579	82.1322	130.585	82.24073	132.9142	82.18152	139.8236	82.15728	148.3313	82.36968	133.7055
82.17133	135.3545	82.14887	130.5821	82.2574	132.9129	82.19818	139.8249	82.17395	148.3319	82.38635	133.704
82.188	135.356	82.16553	130.5806	82.27407	132.9126	82.21485	139.824	82.19062	148.3311	82.40302	133.702
82.20467	135.3554	82.1822	130.5801	82.29073	132.9138	82.23152	139.8245	82.20728	148.3326	82.41968	133.7008
82.22133	135.3545	82.19887	130.58	82.3074	132.9154	82.24818	139.8208	82.22395	148.334	82.43635	133.7033
82.238	135.3528	82.21553	130.5765	82.32407	132.9146	82.26485	139.8239	82.24062	148.3308	82.45302	133.7026
82.25467	135.35	82.2322	130.5743	82.34073	132.9143	82.28152	139.8237	82.25728	148.3353	82.46968	133.6997
82.27133	135.3499	82.24887	130.5728	82.3574	132.9146	82.29818	139.8224	82.27395	148.3324	82.48635	133.6999
82.288	135.3482	82.26553	130.5705	82.37407	132.9132	82.31485	139.8219	82.29062	148.3351	82.50302	133.6988
82.30467	135.3442	82.2822	130.5673	82.39073	132.9135	82.33152	139.8225	82.30728	148.3334	82.51968	133.7019
82.32133	135.3465	82.29887	130.567	82.4074	132.911	82.34818	139.8214	82.32395	148.3325	82.53635	133.7001
82.338	135.3459	82.31553	130.5638	82.42407	132.9112	82.36485	139.8211	82.34062	148.335	82.55302	133.6987
82.35467	135.3434	82.3322	130.5633	82.44073	132.9124	82.38152	139.8214	82.35728	148.3346	82.56968	133.696
82.37133	135.3416	82.34887	130.5598	82.4574	132.9126	82.39818	139.8189	82.37395	148.3333	82.58635	133.7001
82.388	135.3397	82.36553	130.5583	82.47407	132.9074	82.41485	139.8215	82.39062	148.3342	82.60302	133.6969
82.40467	135.3399	82.3822	130.5584	82.49073	132.9106	82.43152	139.8197	82.40728	148.3328	82.61968	133.696
82.42133	135.3382	82.39887	130.5564	82.5074	132.911	82.44818	139.8189	82.42395	148.3322	82.63635	133.6943
82.438	135.3361	82.41553	130.5533	82.52407	132.9071	82.46485	139.8188	82.44062	148.3363	82.65302	133.6936
82.45467	135.3346	82.4322	130.5528	82.54073	132.9095	82.48152	139.8173	82.45728	148.3341	82.66968	133.6957
82.47133	135.3353	82.44887	130.5503	82.5574	132.9106	82.49818	139.8157	82.47395	148.3336	82.68635	133.695
82.488	135.3309	82.46553	130.5474	82.57407	132.9116	82.51485	139.8163	82.49062	148.3332	82.70302	133.696
82.50467	135.3305	82.4822	130.5483	82.59073	132.908	82.53152	139.8182	82.50728	148.3338	82.71968	133.6961
82.52133	135.3295	82.49887	130.5444	82.6074	132.9067	82.54818	139.816	82.52395	148.3322	82.73635	133.6933
82.538	135.3289	82.51553	130.5406	82.62407	132.9105	82.56485	139.8153	82.54062	148.3354	82.75302	133.6945
82.55467	135.3277	82.5322	130.5404	82.64073	132.9093	82.58152	139.8156	82.55728	148.3361	82.76968	133.6906
82.57133	135.3275	82.54887	130.5405	82.6574	132.9058	82.59818	139.8144	82.57395	148.335	82.78635	133.6929
82.588	135.3245	82.56553	130.5353	82.67407	132.9079	82.61485	139.8141	82.59062	148.3338	82.80302	133.6924
82.60467	135.3216	82.5822	130.5371	82.69073	132.9074	82.63152	139.8149	82.60728	148.3323	82.81968	133.6924
82.62133	135.3216	82.59887	130.5363	82.7074	132.909	82.64818	139.8122	82.62395	148.3357	82.83635	133.6887
82.638	135.3209	82.61553	130.5324	82.72407	132.9037	82.66485	139.8132	82.64062	148.3357	82.85302	133.6902
82.65467	135.3189	82.6322	130.53	82.74073	132.9052	82.68152	139.8136	82.65728	148.3318	82.86968	133.6885
82.67133	135.3175	82.64887	130.5284	82.7574	132.9059	82.69818	139.8119	82.67395	148.3349	82.88635	133.6902
82.688	135.3164	82.66553	130.5292	82.77407	132.9048	82.71485	139.8108	82.69062	148.3355	82.90302	133.6874
82.70467	135.3155	82.6822	130.5238	82.79073	132.9015	82.73152	139.8105	82.70728	148.3351	82.91968	133.6885
82.72133	135.3171	82.69887	130.5268	82.8074	132.9077	82.74818	139.8093	82.72395	148.3339	82.93635	133.6874
82.738	135.3144	82.71553	130.5255	82.82407	132.9034	82.76485	139.8107	82.74062	148.3357	82.95302	133.6891
82.75467	135.3129	82.7322	130.5258	82.84073	132.9059	82.78152	139.8083	82.75728	148.3357	82.96968	133.6872
82.77133	135.3124	82.74887	130.5207	82.8574	132.906	82.79818	139.8097	82.77395	148.3342	82.98635	133.6861

82.788	135.3122	82.76553	130.5169	82.87407	132.9056	82.81485	139.8064	82.79062	148.3363	83.00302	133.6877
82.80467	135.3098	82.7822	130.5201	82.89073	132.9008	82.83152	139.8052	82.80728	148.3393	83.01968	133.6881
82.82133	135.307	82.79887	130.519	82.9074	132.9045	82.84818	139.8049	82.82395	148.3373	83.03635	133.688
82.838	135.3068	82.81553	130.5122	82.92407	132.9055	82.86485	139.8045	82.84062	148.3394	83.05302	133.6852
82.85467	135.3061	82.8322	130.5152	82.94073	132.9007	82.88152	139.8021	82.85728	148.3351	83.06968	133.6874
82.87133	135.3038	82.84887	130.5138	82.9574	132.901	82.89818	139.8032	82.87395	148.338	83.08635	133.6855
82.888	135.3045	82.86553	130.5109	82.97407	132.9022	82.91485	139.8012	82.89062	148.3369	83.10302	133.6882
82.90467	135.3034	82.8822	130.5086	82.99073	132.8997	82.93152	139.8031	82.90728	148.3373	83.11968	133.6813
82.92133	135.302	82.89887	130.5098	83.0074	132.8978	82.94818	139.8024	82.92395	148.3369	83.13635	133.6861
82.938	135.3022	82.91553	130.5085	83.02407	132.9009	82.96485	139.7995	82.94062	148.3382	83.15302	133.6846
82.95467	135.2982	82.9322	130.5065	83.04073	132.9022	82.98152	139.7979	82.95728	148.3377	83.16968	133.685
82.97133	135.2972	82.94887	130.504	83.0574	132.8995	82.99818	139.7994	82.97395	148.3405	83.18635	133.6814
82.988	135.2962	82.96553	130.505	83.07407	132.8982	83.01485	139.7962	82.99062	148.3425	83.20302	133.6828
83.00467	135.2959	82.9822	130.5006	83.09073	132.8994	83.03152	139.8	83.00728	148.3406	83.21968	133.6826
83.02133	135.2958	82.99887	130.5002	83.1074	132.9001	83.04818	139.7998	83.02395	148.3397	83.23635	133.6822
83.038	135.2935	83.01553	130.4968	83.12407	132.8972	83.06485	139.7977	83.04062	148.3422	83.25302	133.6829
83.05467	135.2907	83.0322	130.4986	83.14073	132.9031	83.08152	139.7989	83.05728	148.341	83.26968	133.6816
83.07133	135.2882	83.04887	130.497	83.1574	132.8974	83.09818	139.7978	83.07395	148.34	83.28635	133.681
83.088	135.2871	83.06553	130.4934	83.17407	132.9011	83.11485	139.7961	83.09062	148.3412	83.30302	133.68
83.10467	135.2886	83.0822	130.4913	83.19073	132.8976	83.13152	139.7967	83.10728	148.3366	83.31968	133.6805
83.12133	135.2871	83.09887	130.4899	83.2074	132.8981	83.14818	139.7961	83.12395	148.3391	83.33635	133.679
83.138	135.2872	83.11553	130.4892	83.22407	132.8969	83.16485	139.7957	83.14062	148.3393	83.35302	133.6788
83.15467	135.2866	83.1322	130.4894	83.24073	132.8933	83.18152	139.795	83.15728	148.3394	83.36968	133.6787
83.17133	135.2849	83.14887	130.4875	83.2574	132.8966	83.19818	139.7978	83.17395	148.3368	83.38635	133.6742
83.188	135.2794	83.16553	130.4859	83.27407	132.8963	83.21485	139.7961	83.19062	148.3399	83.40302	133.6769
83.20467	135.2797	83.1822	130.4867	83.29073	132.8956	83.23152	139.7967	83.20728	148.3368	83.41968	133.6755
83.22133	135.2806	83.19887	130.4836	83.3074	132.8949	83.24818	139.7967	83.22395	148.3357	83.43635	133.6737
83.238	135.2808	83.21553	130.4789	83.32407	132.8929	83.26485	139.7958	83.24062	148.3367	83.45302	133.6735
83.25467	135.2792	83.2322	130.4774	83.34073	132.8955	83.28152	139.7963	83.25728	148.3401	83.46968	133.6755
83.27133	135.2788	83.24887	130.4784	83.3574	132.8936	83.29818	139.7959	83.27395	148.3401	83.48635	133.6751
83.288	135.2757	83.26553	130.4777	83.37407	132.8937	83.31485	139.795	83.29062	148.3376	83.50302	133.6742
83.30467	135.2746	83.2822	130.4729	83.39073	132.891	83.33152	139.7931	83.30728	148.339	83.51968	133.6737
83.32133	135.2713	83.29887	130.4753	83.4074	132.8915	83.34818	139.7934	83.32395	148.3375	83.53635	133.6751
83.338	135.2703	83.31553	130.4731	83.42407	132.893	83.36485	139.7925	83.34062	148.342	83.55302	133.6726
83.35467	135.2703	83.3322	130.4682	83.44073	132.8906	83.38152	139.7931	83.35728	148.3387	83.56968	133.6738
83.37133	135.2681	83.34887	130.4705	83.4574	132.8885	83.39818	139.7928	83.37395	148.3399	83.58635	133.6719
83.388	135.2667	83.36553	130.4675	83.47407	132.8907	83.41485	139.7887	83.39062	148.3415	83.60302	133.6736
83.40467	135.267	83.3822	130.4674	83.49073	132.8854	83.43152	139.7892	83.40728	148.3398	83.61968	133.6729
83.42133	135.264	83.39887	130.468	83.5074	132.8894	83.44818	139.7905	83.42395	148.3379	83.63635	133.6735
83.438	135.261	83.41553	130.4616	83.52407	132.8849	83.46485	139.789	83.44062	148.3381	83.65302	133.6707
83.45467	135.2601	83.4322	130.4623	83.54073	132.8914	83.48152	139.7885	83.45728	148.34	83.66968	133.6718
83.47133	135.2648	83.44887	130.4624	83.5574	132.8881	83.49818	139.7887	83.47395	148.3388	83.68635	133.6688
83.488	135.2621	83.46553	130.4587	83.57407	132.8882	83.51485	139.7861	83.49062	148.3398	83.70302	133.6699
83.50467	135.2595	83.4822	130.458	83.59073	132.887	83.53152	139.786	83.50728	148.3409	83.71968	133.6692
83.52133	135.2584	83.49887	130.455	83.6074	132.8843	83.54818	139.7852	83.52395	148.3407	83.73635	133.6697
83.538	135.2572	83.51553	130.456	83.62407	132.8866	83.56485	139.7865	83.54062	148.3412	83.75302	133.6672
83.55467	135.2537	83.5322	130.4559	83.64073	132.8866	83.58152	139.7855	83.55728	148.3407	83.76968	133.6694
83.57133	135.2527	83.54887	130.4533	83.6574	132.8864	83.59818	139.7856	83.57395	148.3451	83.78635	133.6676
83.588	135.2543	83.56553	130.449	83.67407	132.8842	83.61485	139.7835	83.59062	148.3419	83.80302	133.6681
83.60467	135.2494	83.5822	130.4512	83.69073	132.8842	83.63152	139.7841	83.60728	148.3427	83.81968	133.6682
83.62133	135.2488	83.59887	130.4483	83.7074	132.8838	83.64818	139.7817	83.62395	148.3431	83.83635	133.6683
83.638	135.2518	83.61553	130.4491	83.72407	132.8815	83.66485	139.781	83.64062	148.3455	83.85302	133.669
83.65467	135.248	83.6322	130.4457	83.74073	132.8849	83.68152	139.7819	83.65728	148.3421	83.86968	133.6674
83.67133	135.2434	83.64887	130.4458	83.7574	132.8818	83.69818	139.7796	83.67395	148.3459	83.88635	133.6669
83.688	135.2467	83.66553	130.4457	83.77407	132.8822	83.71485	139.7805	83.69062	148.3433	83.90302	133.6674
83.70467	135.2409	83.6822	130.4463	83.79073	132.8796	83.73152	139.7805	83.70728	148.3422	83.91968	133.6664
83.72133	135.2397	83.69887	130.4424	83.8074	132.8788	83.74818	139.7786	83.72395	148.3425	83.93635	133.6641
83.738	135.2387	83.71553	130.4414	83.82407	132.8797	83.76485	139.7789	83.74062	148.3424	83.95302	133.6656
83.75467	135.2381	83.7322	130.4398	83.84073	132.8784	83.78152	139.7796	83.75728	148.3421	83.96968	133.6651
83.77133	135.2371	83.74887	130.4393	83.8574	132.8798	83.79818	139.7803	83.77395	148.3425	83.98635	133.666
83.788	135.2367	83.76553	130.44	83.87407	132.8788	83.81485	139.7776	83.79062	148.3463	84.00302	133.6651
83.80467	135.2363	83.7822	130.4364	83.89073	132.8775	83.83152	139.7774	83.80728	148.3414	84.01968	133.6661
83.82133	135.2349	83.79887	130.4389	83.9074	132.8776	83.84818	139.7772	83.82395	148.3427	84.03635	133.6654
83.838	135.2353	83.81553	130.4334	83.92407	132.8756	83.86485	139.7754	83.84062	148.3437	84.05302	133.6631
83.85467	135.2355	83.8322	130.4334	83.94073	132.8771	83.88152	139.7746	83.85728	148.3418	84.06968	133.6636
83.87133	135.2305	83.84887	130.4328	83.9574	132.8771	83.89818	139.776	83.87395	148.3441	84.08635	133.6648
83.888	135.2285	83.86553	130.4334	83.97407	132.8757	83.91485	139.7743	83.89062	148.3447	84.10302	133.6596
83.90467	135.2301	83.8822	130.4273	83.99073	132.8734	83.93152	139.7746	83.90728	148.3466	84.11968	133.6625
83.92133	135.2283	83.89887	130.4274	84.0074	132.8733	83.94818	139.774	83.92395	148.3443	84.13635	133.6618

83.938	135.2251	83.91553	130.4266	84.02407	132.8676	83.96485	139.7713	83.94062	148.3449	84.15302	133.6634
83.95467	135.2267	83.9322	130.4245	84.04073	132.873	83.98152	139.7727	83.95728	148.3447	84.16968	133.661
83.97133	135.2271	83.94887	130.4231	84.0574	132.8712	83.99818	139.7739	83.97395	148.3439	84.18635	133.6595
83.988	135.2254	83.96553	130.4251	84.07407	132.8693	84.01485	139.7718	83.99062	148.3428	84.20302	133.6623
84.00467	135.2229	83.9822	130.423	84.09073	132.8724	84.03152	139.7706	84.00728	148.3408	84.21968	133.662
84.02133	135.2243	83.99887	130.4198	84.1074	132.8688	84.04818	139.7684	84.02395	148.3397	84.23635	133.6591
84.038	135.2203	84.01553	130.4198	84.12407	132.8704	84.06485	139.771	84.04062	148.3478	84.25302	133.6592
84.05467	135.2204	84.0322	130.4163	84.14073	132.8714	84.08152	139.7692	84.05728	148.3474	84.26968	133.6615
84.07133	135.2199	84.04887	130.4171	84.1574	132.8693	84.09818	139.7698	84.07395	148.3441	84.28635	133.6584
84.088	135.2162	84.06553	130.4152	84.17407	132.8682	84.11485	139.7677	84.09062	148.3469	84.30302	133.6582
84.10467	135.2159	84.0822	130.4149	84.19073	132.8663	84.13152	139.7681	84.10728	148.3468	84.31968	133.657
84.12133	135.2161	84.09887	130.4137	84.2074	132.8681	84.14818	139.7669	84.12395	148.3454	84.33635	133.6589
84.138	135.2117	84.11553	130.4116	84.22407	132.8689	84.16485	139.7678	84.14062	148.3454	84.35302	133.6552
84.15467	135.2133	84.1322	130.4121	84.24073	132.8697	84.18152	139.7664	84.15728	148.3479	84.36968	133.6562
84.17133	135.2078	84.14887	130.4089	84.2574	132.8656	84.19818	139.767	84.17395	148.3472	84.38635	133.6543
84.188	135.2096	84.16553	130.4087	84.27407	132.8686	84.21485	139.7664	84.19062	148.3497	84.40302	133.6542
84.20467	135.2101	84.1822	130.4088	84.29073	132.8643	84.23152	139.7653	84.20728	148.3463	84.41968	133.6527
84.22133	135.2084	84.19887	130.4093	84.3074	132.8656	84.24818	139.7645	84.22395	148.3487	84.43635	133.6544
84.238	135.2059	84.21553	130.4041	84.32407	132.8676	84.26485	139.764	84.24062	148.3452	84.45302	133.6533
84.25467	135.2064	84.2322	130.4046	84.34073	132.867	84.28152	139.7638	84.25728	148.3475	84.46968	133.6516
84.27133	135.2035	84.24887	130.4042	84.3574	132.8683	84.29818	139.7644	84.27395	148.3476	84.48635	133.6512
84.288	135.203	84.26553	130.4037	84.37407	132.867	84.31485	139.7652	84.29062	148.3491	84.50302	133.6513
84.30467	135.2016	84.2822	130.4005	84.39073	132.8653	84.33152	139.7645	84.30728	148.3484	84.51968	133.6485
84.32133	135.2004	84.29887	130.4021	84.4074	132.8641	84.34818	139.7612	84.32395	148.3466	84.53635	133.6508
84.338	135.2012	84.31553	130.4006	84.42407	132.8615	84.36485	139.764	84.34062	148.3472	84.55302	133.649
84.35467	135.1989	84.3322	130.3987	84.44073	132.8654	84.38152	139.7621	84.35728	148.3484	84.56968	133.6506
84.37133	135.1987	84.34887	130.3965	84.4574	132.8639	84.39818	139.7614	84.37395	148.3481	84.58635	133.6483
84.388	135.1956	84.36553	130.3967	84.47407	132.8632	84.41485	139.7616	84.39062	148.3476	84.60302	133.6466
84.40467	135.1964	84.3822	130.3962	84.49073	132.8621	84.43152	139.7607	84.40728	148.3481	84.61968	133.6465
84.42133	135.1933	84.39887	130.3927	84.5074	132.862	84.44818	139.7622	84.42395	148.3474	84.63635	133.6452
84.438	135.1938	84.41553	130.3953	84.52407	132.8631	84.46485	139.7614	84.44062	148.3469	84.65302	133.6466
84.45467	135.1954	84.4322	130.3938	84.54073	132.8619	84.48152	139.7596	84.45728	148.3489	84.66968	133.6449
84.47133	135.1909	84.44887	130.3893	84.5574	132.8621	84.49818	139.7607	84.47395	148.3491	84.68635	133.645
84.488	135.1872	84.46553	130.3896	84.57407	132.8612	84.51485	139.7592	84.49062	148.3478	84.70302	133.6455
84.50467	135.187	84.4822	130.3888	84.59073	132.862	84.53152	139.76	84.50728	148.3469	84.71968	133.6435
84.52133	135.1859	84.49887	130.3863	84.6074	132.8612	84.54818	139.7601	84.52395	148.3477	84.73635	133.642
84.538	135.1886	84.51553	130.3808	84.62407	132.8595	84.56485	139.7601	84.54062	148.3472	84.75302	133.6432
84.55467	135.1876	84.5322	130.3827	84.64073	132.8625	84.58152	139.7598	84.55728	148.3466	84.76968	133.6442
84.57133	135.1867	84.54887	130.3852	84.6574	132.8609	84.59818	139.7565	84.57395	148.3497	84.78635	133.6387
84.588	135.1849	84.56553	130.3824	84.67407	132.859	84.61485	139.7581	84.59062	148.3515	84.80302	133.6414
84.60467	135.181	84.5822	130.3816	84.69073	132.8567	84.63152	139.7604	84.60728	148.3513	84.81968	133.6392
84.62133	135.1776	84.59887	130.3814	84.7074	132.8577	84.64818	139.7572	84.62395	148.3506	84.83635	133.6383
84.638	135.1784	84.61553	130.3775	84.72407	132.8585	84.66485	139.7592	84.64062	148.3495	84.85302	133.6379
84.65467	135.1803	84.6322	130.3798	84.74073	132.8588	84.68152	139.7557	84.65728	148.3507	84.86968	133.638
84.67133	135.1793	84.64887	130.3735	84.7574	132.8593	84.69818	139.7574	84.67395	148.3497	84.88635	133.6374
84.688	135.1789	84.66553	130.3747	84.77407	132.8558	84.71485	139.7557	84.69062	148.3493	84.90302	133.637
84.70467	135.1775	84.6822	130.3772	84.79073	132.856	84.73152	139.7548	84.70728	148.348	84.91968	133.6348
84.72133	135.1731	84.69887	130.3733	84.8074	132.855	84.74818	139.7555	84.72395	148.35	84.93635	133.6364
84.738	135.1747	84.71553	130.3723	84.82407	132.8553	84.76485	139.7536	84.74062	148.3537	84.95302	133.6336
84.75467	135.1737	84.7322	130.3725	84.84073	132.8551	84.78152	139.7473	84.75728	148.3495	84.96968	133.6343
84.77133	135.1735	84.74887	130.3706	84.8574	132.8564	84.79818	139.752	84.77395	148.3507	84.98635	133.635
84.788	135.1708	84.76553	130.3694	84.87407	132.8556	84.81485	139.7523	84.79062	148.3511	85.00302	133.6328
84.80467	135.17	84.7822	130.3696	84.89073	132.8537	84.83152	139.7517	84.80728	148.3515	85.01968	133.6318
84.82133	135.169	84.79887	130.3685	84.9074	132.854	84.84818	139.7489	84.82395	148.3506	85.03635	133.6325
84.838	135.1684	84.81553	130.3679	84.92407	132.8529	84.86485	139.7496	84.84062	148.3537	85.05302	133.6312
84.85467	135.1685	84.8322	130.3648	84.94073	132.8533	84.88152	139.7458	84.85728	148.3504	85.06968	133.6311
84.87133	135.1672	84.84887	130.3652	84.9574	132.8516	84.89818	139.7483	84.87395	148.3507	85.08635	133.6293
84.888	135.1647	84.86553	130.3632	84.97407	132.8528	84.91485	139.7461	84.89062	148.3528	85.10302	133.6306
84.90467	135.1622	84.8822	130.3638	84.99073	132.8509	84.93152	139.7467	84.90728	148.3529	85.11968	133.6264
84.92133	135.1652	84.89887	130.3613	85.0074	132.8506	84.94818	139.7451	84.92395	148.3517	85.13635	133.6286
84.938	135.1619	84.91553	130.3609	85.02407	132.8503	84.96485	139.7452	84.94062	148.3536	85.15302	133.626
84.95467	135.1643	84.9322	130.3604	85.04073	132.8519	84.98152	139.7444	84.95728	148.3531	85.16968	133.6264
84.97133	135.1606	84.94887	130.36	85.0574	132.8501	84.99818	139.7448	84.97395	148.3494	85.18635	133.6259
84.988	135.1594	84.96553	130.3604	85.07407	132.8499	85.01485	139.7445	84.99062	148.3523	85.20302	133.6263
85.00467	135.1578	84.9822	130.3577	85.09073	132.8493	85.03152	139.744	85.00728	148.3535	85.21968	133.625
85.02133	135.1573	84.99887	130.3578	85.1074	132.8495	85.04818	139.7444	85.02395	148.3538	85.23635	133.6245
85.038	135.1573	85.01553	130.3541	85.12407	132.8461	85.06485	139.7428	85.04062	148.353	85.25302	133.6244
85.05467	135.1557	85.0322	130.3549	85.14073	132.8486	85.08152	139.7432	85.05728	148.3539	85.26968	133.6228
85.07133	135.1556	85.04887	130.3534	85.1574	132.8471	85.09818	139.7428	85.07395	148.3503	85.28635	133.6215

85.088	135.1544	85.06553	130.3526	85.17407	132.8505	85.11485	139.7408	85.09062	148.3567	85.30302	133.6243
85.10467	135.1524	85.0822	130.35	85.19073	132.8459	85.13152	139.7454	85.10728	148.3547	85.31968	133.6201
85.12133	135.152	85.09887	130.3496	85.2074	132.845	85.14818	139.7407	85.12395	148.353	85.33635	133.6203
85.138	135.1491	85.11553	130.3489	85.22407	132.8469	85.16485	139.7424	85.14062	148.3515	85.35302	133.6206
85.15467	135.147	85.1322	130.351	85.24073	132.8451	85.18152	139.7412	85.15728	148.3575	85.36968	133.6193
85.17133	135.1454	85.14887	130.3469	85.2574	132.8456	85.19818	139.7443	85.17395	148.3561	85.38635	133.6187
85.188	135.146	85.16553	130.3475	85.27407	132.8439	85.21485	139.7412	85.19062	148.3537	85.40302	133.6183
85.20467	135.1457	85.1822	130.345	85.29073	132.8468	85.23152	139.7408	85.20728	148.3592	85.41968	133.6166
85.22133	135.1427	85.19887	130.344	85.3074	132.8409	85.24818	139.7423	85.22395	148.3588	85.43635	133.6179
85.238	135.1428	85.21553	130.3446	85.32407	132.8434	85.26485	139.7395	85.24062	148.357	85.45302	133.6166
85.25467	135.1417	85.2322	130.3433	85.34073	132.844	85.28152	139.7409	85.25728	148.3554	85.46968	133.6153
85.27133	135.1389	85.24887	130.3397	85.3574	132.8417	85.29818	139.7407	85.27395	148.3536	85.48635	133.6161
85.288	135.1393	85.26553	130.3375	85.37407	132.842	85.31485	139.7393	85.29062	148.3597	85.50302	133.6139
85.30467	135.1366	85.2822	130.3384	85.39073	132.8433	85.33152	139.741	85.30728	148.3558	85.51968	133.6118
85.32133	135.1401	85.29887	130.3361	85.4074	132.8417	85.34818	139.7381	85.32395	148.3552	85.53635	133.6145
85.338	135.1367	85.31553	130.3373	85.42407	132.8406	85.36485	139.7412	85.34062	148.3556	85.55302	133.6124
85.35467	135.1341	85.3322	130.3379	85.44073	132.8397	85.38152	139.7382	85.35728	148.3578	85.56968	133.6113
85.37133	135.1356	85.34887	130.3342	85.4574	132.8402	85.39818	139.7368	85.37395	148.3557	85.58635	133.6078
85.388	135.1356	85.36553	130.3336	85.47407	132.8404	85.41485	139.7376	85.39062	148.3547	85.60302	133.612
85.40467	135.1323	85.3822	130.3289	85.49073	132.8385	85.43152	139.737	85.40728	148.3623	85.61968	133.6109
85.42133	135.1298	85.39887	130.3314	85.5074	132.8401	85.44818	139.7356	85.42395	148.3571	85.63635	133.6105
85.438	135.129	85.41553	130.3318	85.52407	132.8349	85.46485	139.7334	85.44062	148.3594	85.65302	133.6074
85.45467	135.1294	85.4322	130.3295	85.54073	132.837	85.48152	139.7356	85.45728	148.3638	85.66968	133.609
85.47133	135.13	85.44887	130.329	85.5574	132.8388	85.49818	139.7354	85.47395	148.3565	85.68635	133.6086
85.488	135.1262	85.46553	130.3264	85.57407	132.8389	85.51485	139.7313	85.49062	148.3621	85.70302	133.6085
85.50467	135.1258	85.4822	130.3235	85.59073	132.8383	85.53152	139.7324	85.50728	148.3567	85.71968	133.6071
85.52133	135.1263	85.49887	130.3223	85.6074	132.8374	85.54818	139.7308	85.52395	148.3598	85.73635	133.6063
85.538	135.123	85.51553	130.323	85.62407	132.836	85.56485	139.7295	85.54062	148.3602	85.75302	133.6069
85.55467	135.1214	85.5322	130.3233	85.64073	132.8378	85.58152	139.731	85.55728	148.3619	85.76968	133.6061
85.57133	135.1216	85.54887	130.3193	85.6574	132.8382	85.59818	139.728	85.57395	148.3604	85.78635	133.6063
85.588	135.1205	85.56553	130.3206	85.67407	132.8361	85.61485	139.7272	85.59062	148.3592	85.80302	133.6049
85.60467	135.1204	85.5822	130.3182	85.69073	132.8364	85.63152	139.727	85.60728	148.3604	85.81968	133.6044
85.62133	135.1193	85.59887	130.318	85.7074	132.8326	85.64818	139.727	85.62395	148.3575	85.83635	133.6016
85.638	135.1179	85.61553	130.3152	85.72407	132.8318	85.66485	139.7297	85.64062	148.3617	85.85302	133.6037
85.65467	135.1176	85.6322	130.3137	85.74073	132.8311	85.68152	139.7255	85.65728	148.3594	85.86968	133.6022
85.67133	135.117	85.64887	130.3151	85.7574	132.8338	85.69818	139.7273	85.67395	148.3601	85.88635	133.601
85.688	135.1175	85.66553	130.312	85.77407	132.8322	85.71485	139.7243	85.69062	148.3612	85.90302	133.6001
85.70467	135.1148	85.6822	130.3134	85.79073	132.8303	85.73152	139.7241	85.70728	148.3591	85.91968	133.6009
85.72133	135.1138	85.69887	130.3138	85.8074	132.8312	85.74818	139.7249	85.72395	148.3577	85.93635	133.5989
85.738	135.1115	85.71553	130.3101	85.82407	132.8295	85.76485	139.7217	85.74062	148.3581	85.95302	133.5972
85.75467	135.1099	85.7322	130.3079	85.84073	132.8294	85.78152	139.723	85.75728	148.3589	85.96968	133.5989
85.77133	135.1087	85.74887	130.3094	85.8574	132.8312	85.79818	139.7252	85.77395	148.3584	85.98635	133.5978
85.788	135.1113	85.76553	130.3084	85.87407	132.8311	85.81485	139.7223	85.79062	148.358	86.00302	133.5968
85.80467	135.1101	85.7822	130.3054	85.89073	132.8315	85.83152	139.7226	85.80728	148.3586	86.01968	133.5965
85.82133	135.1071	85.79887	130.3068	85.9074	132.8315	85.84818	139.7219	85.82395	148.359	86.03635	133.5968
85.838	135.1061	85.81553	130.3004	85.92407	132.829	85.86485	139.7216	85.84062	148.3597	86.05302	133.5946
85.85467	135.1045	85.8322	130.3032	85.94073	132.8312	85.88152	139.723	85.85728	148.3609	86.06968	133.5965
85.87133	135.1018	85.84887	130.3025	85.9574	132.8298	85.89818	139.7212	85.87395	148.3606	86.08635	133.5935
85.888	135.1014	85.86553	130.3005	85.97407	132.8278	85.91485	139.722	85.89062	148.3607	86.10302	133.5953
85.90467	135.0992	85.8822	130.2982	85.99073	132.8302	85.93152	139.7208	85.90728	148.359	86.11968	133.5943
85.92133	135.0986	85.89887	130.3	86.0074	132.831	85.94818	139.7193	85.92395	148.3591	86.13635	133.5939
85.938	135.1013	85.91553	130.2986	86.02407	132.8285	85.96485	139.7204	85.94062	148.3597	86.15302	133.593
85.95467	135.0964	85.9322	130.297	86.04073	132.83	85.98152	139.7165	85.95728	148.3611	86.16968	133.5942
85.97133	135.0981	85.94887	130.2996	86.0574	132.8278	85.99818	139.7209	85.97395	148.3583	86.18635	133.5906
85.988	135.0966	85.96553	130.2974	86.07407	132.8255	86.01485	139.7191	85.99062	148.3606	86.20302	133.5918
86.00467	135.0956	85.9822	130.2937	86.09073	132.8297	86.03152	139.7184	86.00728	148.3612	86.21968	133.5924
86.02133	135.0932	85.99887	130.2957	86.1074	132.8292	86.04818	139.7164	86.02395	148.3613	86.23635	133.5911
86.038	135.0921	86.01553	130.2928	86.12407	132.8248	86.06485	139.7175	86.04062	148.3626	86.25302	133.5911
86.05467	135.0929	86.0322	130.2928	86.14073	132.8272	86.08152	139.7163	86.05728	148.3614	86.26968	133.5908
86.07133	135.0877	86.04887	130.2924	86.1574	132.8261	86.09818	139.716	86.07395	148.3639	86.28635	133.5904
86.088	135.0911	86.06553	130.2923	86.17407	132.827	86.11485	139.7151	86.09062	148.3622	86.30302	133.5895
86.10467	135.0906	86.0822	130.2913	86.19073	132.8253	86.13152	139.7167	86.10728	148.3643	86.31968	133.5888
86.12133	135.0836	86.09887	130.2888	86.2074	132.8263	86.14818	139.7177	86.12395	148.3633	86.33635	133.5861
86.138	135.0893	86.11553	130.289	86.22407	132.8224	86.16485	139.7161	86.14062	148.3631	86.35302	133.5883
86.15467	135.0842	86.1322	130.2872	86.24073	132.8255	86.18152	139.7129	86.15728	148.3622	86.36968	133.5869
86.17133	135.083	86.14887	130.2866	86.2574	132.8238	86.19818	139.7156	86.17395	148.3612	86.38635	133.5833
86.188	135.0811	86.16553	130.2851	86.27407	132.8232	86.21485	139.7165	86.19062	148.3625	86.40302	133.5873
86.20467	135.0781	86.1822	130.2869	86.29073	132.8237	86.23152	139.7149	86.20728	148.3627	86.41968	133.5883
86.22133	135.0791	86.19887	130.2842	86.3074	132.8214	86.24818	139.7125	86.22395	148.3613	86.43635	133.5886

86.238	135.0778	86.21553	130.2845	86.32407	132.8213	86.26485	139.7131	86.24062	148.36	86.45302	133.5885
86.25467	135.0752	86.2322	130.2849	86.34073	132.821	86.28152	139.7127	86.25728	148.3617	86.46968	133.5878
86.27133	135.0775	86.24887	130.2797	86.3574	132.8184	86.29818	139.7149	86.27395	148.3616	86.48635	133.5881
86.288	135.0748	86.26553	130.2817	86.37407	132.8213	86.31485	139.7111	86.29062	148.3614	86.50302	133.5874
86.30467	135.074	86.2822	130.2807	86.39073	132.8186	86.33152	139.7121	86.30728	148.3645	86.51968	133.5857
86.32133	135.0714	86.29887	130.279	86.4074	132.8188	86.34818	139.7108	86.32395	148.3621	86.53635	133.5861
86.338	135.0703	86.31553	130.2766	86.42407	132.8151	86.36485	139.7123	86.34062	148.3621	86.55302	133.5869
86.35467	135.0723	86.3322	130.2779	86.44073	132.8182	86.38152	139.7142	86.35728	148.3616	86.56968	133.5886
86.37133	135.0688	86.34887	130.2767	86.4574	132.8167	86.39818	139.7096	86.37395	148.3624	86.58635	133.5847
86.388	135.0696	86.36553	130.2737	86.47407	132.8174	86.41485	139.7097	86.39062	148.3603	86.60302	133.5844
86.40467	135.0675	86.3822	130.2735	86.49073	132.8168	86.43152	139.7091	86.40728	148.3596	86.61968	133.5856
86.42133	135.0663	86.39887	130.2728	86.5074	132.8181	86.44818	139.7114	86.42395	148.3588	86.63635	133.5852
86.438	135.0649	86.41553	130.2746	86.52407	132.8156	86.46485	139.7097	86.44062	148.3602	86.65302	133.5832
86.45467	135.0641	86.4322	130.2747	86.54073	132.8148	86.48152	139.7103	86.45728	148.3599	86.66968	133.5845
86.47133	135.0635	86.44887	130.2711	86.5574	132.815	86.49818	139.7098	86.47395	148.3615	86.68635	133.5837
86.488	135.0633	86.46553	130.27	86.57407	132.8139	86.51485	139.7079	86.49062	148.3616	86.70302	133.5815
86.50467	135.0653	86.4822	130.2684	86.59073	132.8125	86.53152	139.711	86.50728	148.36	86.71968	133.58
86.52133	135.0635	86.49887	130.27	86.6074	132.8135	86.54818	139.707	86.52395	148.3598	86.73635	133.5796
86.538	135.0596	86.51553	130.267	86.62407	132.8112	86.56485	139.71	86.54062	148.3592	86.75302	133.5781
86.55467	135.0602	86.5322	130.2626	86.64073	132.8109	86.58152	139.7074	86.55728	148.3568	86.76968	133.5765
86.57133	135.0593	86.54887	130.2665	86.6574	132.8125	86.59818	139.7094	86.57395	148.3606	86.78635	133.5786
86.588	135.055	86.56553	130.2674	86.67407	132.8105	86.61485	139.7057	86.59062	148.3588	86.80302	133.5799
86.60467	135.0548	86.5822	130.2628	86.69073	132.8105	86.63152	139.7059	86.60728	148.3597	86.81968	133.578
86.62133	135.0547	86.59887	130.2629	86.7074	132.8085	86.64818	139.7027	86.62395	148.3567	86.83635	133.5794
86.638	135.0533	86.61553	130.2623	86.72407	132.8064	86.66485	139.7041	86.64062	148.3591	86.85302	133.5787
86.65467	135.0536	86.6322	130.2613	86.74073	132.8088	86.68152	139.7031	86.65728	148.3585	86.86968	133.5789
86.67133	135.0518	86.64887	130.2566	86.7574	132.8091	86.69818	139.7035	86.67395	148.357	86.88635	133.5762
86.688	135.0502	86.66553	130.2583	86.77407	132.8087	86.71485	139.7014	86.69062	148.3561	86.90302	133.5785
86.70467	135.0462	86.6822	130.2567	86.79073	132.8076	86.73152	139.6994	86.70728	148.3558	86.91968	133.5773
86.72133	135.0475	86.69887	130.2559	86.8074	132.808	86.74818	139.7002	86.72395	148.3547	86.93635	133.5759
86.738	135.0472	86.71553	130.2556	86.82407	132.8076	86.76485	139.6986	86.74062	148.3565	86.95302	133.5737
86.75467	135.0449	86.7322	130.2571	86.84073	132.807	86.78152	139.7007	86.75728	148.3572	86.96968	133.5729
86.77133	135.0461	86.74887	130.2527	86.8574	132.8087	86.79818	139.6987	86.77395	148.3573	86.98635	133.5732
86.788	135.0461	86.76553	130.2537	86.87407	132.8058	86.81485	139.6976	86.79062	148.3594	87.00302	133.5718
86.80467	135.0437	86.7822	130.2508	86.89073	132.8048	86.83152	139.6985	86.80728	148.3595	87.01968	133.5726
86.82133	135.0408	86.79887	130.2514	86.9074	132.8046	86.84818	139.6978	86.82395	148.3581	87.03635	133.5714
86.838	135.0422	86.81553	130.2514	86.92407	132.8063	86.86485	139.6979	86.84062	148.3594	87.05302	133.5688
86.85467	135.0415	86.8322	130.2492	86.94073	132.8048	86.88152	139.6964	86.85728	148.3598	87.06968	133.5702
86.87133	135.0392	86.84887	130.2487	86.9574	132.803	86.89818	139.6958	86.87395	148.3567	87.08635	133.572
86.888	135.04	86.86553	130.2464	86.97407	132.8032	86.91485	139.6947	86.89062	148.3586	87.10302	133.5682
86.90467	135.0381	86.8822	130.2451	86.99073	132.8039	86.93152	139.6956	86.90728	148.3567	87.11968	133.5674
86.92133	135.0356	86.89887	130.2453	87.0074	132.8019	86.94818	139.6945	86.92395	148.3577	87.13635	133.5708
86.938	135.0347	86.91553	130.2438	87.02407	132.8026	86.96485	139.6948	86.94062	148.3561	87.15302	133.5685
86.95467	135.0333	86.9322	130.2418	87.04073	132.8045	86.98152	139.6946	86.95728	148.3556	87.16968	133.5697
86.97133	135.0336	86.94887	130.2429	87.0574	132.8008	86.99818	139.6916	86.97395	148.3596	87.18635	133.5686
86.988	135.0328	86.96553	130.242	87.07407	132.8008	87.01485	139.6918	86.99062	148.3572	87.20302	133.568
87.00467	135.0302	86.9822	130.2392	87.09073	132.8014	87.03152	139.6914	87.00728	148.3622	87.21968	133.5665
87.02133	135.0291	86.99887	130.2398	87.1074	132.8035	87.04818	139.6912	87.02395	148.3614	87.23635	133.5658
87.038	135.0287	87.01553	130.2414	87.12407	132.801	87.06485	139.6925	87.04062	148.3585	87.25302	133.5649
87.05467	135.0252	87.0322	130.2372	87.14073	132.7984	87.08152	139.6901	87.05728	148.3598	87.26968	133.5664
87.07133	135.0211	87.04887	130.2354	87.1574	132.7975	87.09818	139.6888	87.07395	148.3581	87.28635	133.5644
87.088	135.0225	87.06553	130.2373	87.17407	132.7987	87.11485	139.6906	87.09062	148.3603	87.30302	133.5631
87.10467	135.0238	87.0822	130.2336	87.19073	132.7953	87.13152	139.6896	87.10728	148.359	87.31968	133.566
87.12133	135.0193	87.09887	130.2353	87.2074	132.7976	87.14818	139.689	87.12395	148.363	87.33635	133.5631
87.138	135.0167	87.11553	130.2358	87.22407	132.7963	87.16485	139.6866	87.14062	148.3597	87.35302	133.5625
87.15467	135.0174	87.1322	130.2341	87.24073	132.7941	87.18152	139.6897	87.15728	148.3595	87.36968	133.5629
87.17133	135.0147	87.14887	130.2319	87.2574	132.7939	87.19818	139.6871	87.17395	148.3643	87.38635	133.5636
87.188	135.0154	87.16553	130.2319	87.27407	132.7969	87.21485	139.6877	87.19062	148.3592	87.40302	133.563
87.20467	135.0153	87.1822	130.2284	87.29073	132.7929	87.23152	139.6866	87.20728	148.3632	87.41968	133.561
87.22133	135.0128	87.19887	130.2285	87.3074	132.7947	87.24818	139.6866	87.22395	148.3608	87.43635	133.5633
87.238	135.0128	87.21553	130.2297	87.32407	132.7929	87.26485	139.6869	87.24062	148.3607	87.45302	133.562
87.25467	135.0113	87.2322	130.2294	87.34073	132.7923	87.28152	139.6862	87.25728	148.3596	87.46968	133.562
87.27133	135.007	87.24887	130.2255	87.3574	132.7925	87.29818	139.6861	87.27395	148.3646	87.48635	133.5604
87.288	135.0065	87.26553	130.2258	87.37407	132.795	87.31485	139.6839	87.29062	148.3636	87.50302	133.5624
87.30467	135.0056	87.2822	130.2255	87.39073	132.7893	87.33152	139.6901	87.30728	148.3636	87.51968	133.5613
87.32133	135.0059	87.29887	130.223	87.4074	132.7899	87.34818	139.6841	87.32395	148.3615	87.53635	133.5596
87.338	135.0048	87.31553	130.2214	87.42407	132.7922	87.36485	139.6848	87.34062	148.3617	87.55302	133.5584
87.35467	135.0023	87.3322	130.2216	87.44073	132.7914	87.38152	139.6839	87.35728	148.3622	87.56968	133.5582
87.37133	135.0032	87.34887	130.2208	87.4574	132.7899	87.39818	139.6856	87.37395	148.364	87.58635	133.5555

87.388	135.0023	87.36553	130.2203	87.47407	132.7898	87.41485	139.6841	87.39062	148.3649	87.60302	133.5524
87.40467	135.0008	87.3822	130.2231	87.49073	132.7877	87.43152	139.6837	87.40728	148.3614	87.61968	133.5514
87.42133	135.0011	87.39887	130.2219	87.5074	132.7888	87.44818	139.6833	87.42395	148.3646	87.63635	133.5519
87.438	134.9977	87.41553	130.2205	87.52407	132.7874	87.46485	139.6837	87.44062	148.362	87.65302	133.5501
87.45467	134.9964	87.4322	130.2189	87.54073	132.7869	87.48152	139.6827	87.45728	148.3624	87.66968	133.5483
87.47133	134.993	87.44887	130.2169	87.5574	132.7857	87.49818	139.6826	87.47395	148.367	87.68635	133.5482
87.488	134.993	87.46553	130.2186	87.57407	132.7858	87.51485	139.6803	87.49062	148.3626	87.70302	133.5468
87.50467	134.9912	87.4822	130.2151	87.59073	132.7837	87.53152	139.6793	87.50728	148.3655	87.71968	133.546
87.52133	134.9912	87.49887	130.2169	87.6074	132.7859	87.54818	139.6793	87.52395	148.3636	87.73635	133.5432
87.538	134.9888	87.51553	130.2149	87.62407	132.783	87.56485	139.678	87.54062	148.3643	87.75302	133.5438
87.55467	134.9962	87.5322	130.214	87.64073	132.7826	87.58152	139.6804	87.55728	148.3661	87.76968	133.5443
87.57133	134.9891	87.54887	130.2115	87.6574	132.7815	87.59818	139.6786	87.57395	148.3639	87.78635	133.5421
87.588	134.9908	87.56553	130.2137	87.67407	132.7826	87.61485	139.6796	87.59062	148.363	87.80302	133.5451
87.60467	134.9921	87.5822	130.2104	87.69073	132.7827	87.63152	139.6772	87.60728	148.3626	87.81968	133.544
87.62133	134.9871	87.59887	130.2092	87.7074	132.7798	87.64818	139.6763	87.62395	148.3641	87.83635	133.5437
87.638	134.9882	87.61553	130.2092	87.72407	132.7819	87.66485	139.6755	87.64062	148.364	87.85302	133.5414
87.65467	134.9912	87.6322	130.2083	87.74073	132.779	87.68152	139.6751	87.65728	148.3652	87.86968	133.5415
87.67133	134.9874	87.64887	130.2068	87.7574	132.7804	87.69818	139.6746	87.67395	148.364	87.88635	133.5397
87.688	134.9829	87.66553	130.2056	87.77407	132.7784	87.71485	139.6728	87.69062	148.3682	87.90302	133.5402
87.70467	134.9874	87.6822	130.2066	87.79073	132.7776	87.73152	139.6755	87.70728	148.3671	87.91968	133.5406
87.72133	134.9819	87.69887	130.2055	87.8074	132.7763	87.74818	139.6723	87.72395	148.3642	87.93635	133.5415
87.738	134.9849	87.71553	130.2051	87.82407	132.7754	87.76485	139.674	87.74062	148.3663	87.95302	133.5392
87.75467	134.9818	87.7322	130.2033	87.84073	132.7761	87.78152	139.6709	87.75728	148.3657	87.96968	133.5386
87.77133	134.9834	87.74887	130.2031	87.8574	132.7763	87.79818	139.6733	87.77395	148.3671	87.98635	133.541
87.788	134.9802	87.76553	130.201	87.87407	132.7745	87.81485	139.673	87.79062	148.3663	88.00302	133.5407
87.80467	134.9802	87.7822	130.2012	87.89073	132.7748	87.83152	139.6701	87.80728	148.3666	88.01968	133.5396
87.82133	134.9796	87.79887	130.2012	87.9074	132.7715	87.84818	139.6716	87.82395	148.3696	88.03635	133.5408
87.838	134.9803	87.81553	130.1953	87.92407	132.7766	87.86485	139.6717	87.84062	148.3689	88.05302	133.54
87.85467	134.9809	87.8322	130.1966	87.94073	132.7766	87.88152	139.6699	87.85728	148.3697	88.06968	133.5385
87.87133	134.9767	87.84887	130.1985	87.9574	132.7734	87.89818	139.6697	87.87395	148.3683	88.08635	133.5369
87.888	134.9757	87.86553	130.1957	87.97407	132.7738	87.91485	139.6702	87.89062	148.3724	88.10302	133.538
87.90467	134.9723	87.8822	130.1938	87.99073	132.7716	87.93152	139.6696	87.90728	148.3703	88.11968	133.537
87.92133	134.9747	87.89887	130.1952	88.0074	132.7721	87.94818	139.6687	87.92395	148.3715	88.13635	133.5397
87.938	134.9742	87.91553	130.1928	88.02407	132.7744	87.96485	139.6706	87.94062	148.3711	88.15302	133.5355
87.95467	134.974	87.9322	130.1938	88.04073	132.7736	87.98152	139.6678	87.95728	148.3694	88.16968	133.5374
87.97133	134.9747	87.94887	130.1919	88.0574	132.7706	87.99818	139.6678	87.97395	148.3705	88.18635	133.5345
87.988	134.9702	87.96553	130.1911	88.07407	132.7732	88.01485	139.6678	87.99062	148.3708	88.20302	133.5331
88.00467	134.967	87.9822	130.1897	88.09073	132.7708	88.03152	139.6688	88.00728	148.3719	88.21968	133.5339
88.02133	134.9668	87.99887	130.188	88.1074	132.7676	88.04818	139.6673	88.02395	148.3708	88.23635	133.5338
88.038	134.9688	88.01553	130.1863	88.12407	132.7728	88.06485	139.6659	88.04062	148.3732	88.25302	133.5336
88.05467	134.9681	88.0322	130.1883	88.14073	132.7712	88.08152	139.6692	88.05728	148.3683	88.26968	133.5317
88.07133	134.9658	88.04887	130.184	88.1574	132.7717	88.09818	139.6662	88.07395	148.3724	88.28635	133.5306
88.088	134.9665	88.06553	130.185	88.17407	132.7707	88.11485	139.6664	88.09062	148.3696	88.30302	133.5304
88.10467	134.9647	88.0822	130.185	88.19073	132.7709	88.13152	139.6692	88.10728	148.3699	88.31968	133.5324
88.12133	134.9606	88.09887	130.185	88.2074	132.7702	88.14818	139.6643	88.12395	148.3711	88.33635	133.5291
88.138	134.9629	88.11553	130.1837	88.22407	132.7703	88.16485	139.666	88.14062	148.3743	88.35302	133.5301
88.15467	134.9623	88.1322	130.182	88.24073	132.769	88.18152	139.6672	88.15728	148.371	88.36968	133.5269
88.17133	134.9591	88.14887	130.1853	88.2574	132.7712	88.19818	139.6669	88.17395	148.3713	88.38635	133.5283
88.188	134.9597	88.16553	130.1812	88.27407	132.7671	88.21485	139.667	88.19062	148.37	88.40302	133.528
88.20467	134.9597	88.1822	130.1798	88.29073	132.7672	88.23152	139.6644	88.20728	148.3708	88.41968	133.5256
88.22133	134.955	88.19887	130.1789	88.3074	132.7681	88.24818	139.6641	88.22395	148.3721	88.43635	133.5262
88.238	134.9565	88.21553	130.1801	88.32407	132.7667	88.26485	139.6655	88.24062	148.3719	88.45302	133.5249
88.25467	134.9535	88.2322	130.1779	88.34073	132.7667	88.28152	139.6645	88.25728	148.3696	88.46968	133.5252
88.27133	134.9541	88.24887	130.1816	88.3574	132.7632	88.29818	139.6662	88.27395	148.3679	88.48635	133.5232
88.288	134.9544	88.26553	130.1768	88.37407	132.7628	88.31485	139.6641	88.29062	148.3666	88.50302	133.5247
88.30467	134.9515	88.2822	130.1793	88.39073	132.7607	88.33152	139.6586	88.30728	148.3712	88.51968	133.5208
88.32133	134.9517	88.29887	130.178	88.4074	132.7633	88.34818	139.6627	88.32395	148.3695	88.53635	133.521
88.338	134.9528	88.31553	130.1788	88.42407	132.7587	88.36485	139.664	88.34062	148.3708	88.55302	133.5218
88.35467	134.9484	88.3322	130.1757	88.44073	132.7601	88.38152	139.6635	88.35728	148.3698	88.56968	133.5211
88.37133	134.9466	88.34887	130.1754	88.4574	132.7613	88.39818	139.6609	88.37395	148.3733	88.58635	133.521
88.388	134.9465	88.36553	130.172	88.47407	132.7571	88.41485	139.6615	88.39062	148.3716	88.60302	133.5198
88.40467	134.946	88.3822	130.1732	88.49073	132.7639	88.43152	139.6638	88.40728	148.3724	88.61968	133.52
88.42133	134.9447	88.39887	130.1718	88.5074	132.759	88.44818	139.6598	88.42395	148.3721	88.63635	133.518
88.438	134.9445	88.41553	130.1731	88.52407	132.7572	88.46485	139.6614	88.44062	148.3719	88.65302	133.52
88.45467	134.945	88.4322	130.169	88.54073	132.7576	88.48152	139.6589	88.45728	148.3714	88.66968	133.5188
88.47133	134.9428	88.44887	130.1706	88.5574	132.756	88.49818	139.6614	88.47395	148.3704	88.68635	133.5186
88.488	134.942	88.46553	130.1694	88.57407	132.7563	88.51485	139.6594	88.49062	148.372	88.70302	133.5172
88.50467	134.941	88.4822	130.1705	88.59073	132.757	88.53152	139.6592	88.50728	148.3704	88.71968	133.5187
88.52133	134.9411	88.49887	130.1721	88.6074	132.7563	88.54818	139.6603	88.52395	148.3706	88.73635	133.5176

88.538	134.9389	88.51553	130.1687	88.62407	132.755	88.56485	139.6574	88.54062	148.3717	88.75302	133.5162
88.55467	134.9375	88.5322	130.1689	88.64073	132.7528	88.58152	139.6576	88.55728	148.3693	88.76968	133.5175
88.57133	134.9339	88.54887	130.1663	88.6574	132.7526	88.59818	139.6582	88.57395	148.3698	88.78635	133.5187
88.588	134.9374	88.56553	130.1675	88.67407	132.7545	88.61485	139.6554	88.59062	148.3695	88.80302	133.5156
88.60467	134.9365	88.5822	130.1664	88.69073	132.7553	88.63152	139.6548	88.60728	148.3708	88.81968	133.5151
88.62133	134.9375	88.59887	130.1599	88.7074	132.7529	88.64818	139.6542	88.62395	148.3722	88.83635	133.5156
88.638	134.9372	88.61553	130.1659	88.72407	132.7516	88.66485	139.6556	88.64062	148.3712	88.85302	133.5155
88.65467	134.932	88.6322	130.164	88.74073	132.7513	88.68152	139.6524	88.65728	148.3698	88.86968	133.5118
88.67133	134.9332	88.64887	130.1619	88.7574	132.7511	88.69818	139.6547	88.67395	148.37	88.88635	133.5155
88.688	134.9331	88.66553	130.1627	88.77407	132.7514	88.71485	139.6541	88.69062	148.3694	88.90302	133.5149
88.70467	134.9307	88.6822	130.1634	88.79073	132.7499	88.73152	139.6537	88.70728	148.3704	88.91968	133.5115
88.72133	134.9277	88.69887	130.1614	88.8074	132.7518	88.74818	139.6538	88.72395	148.3695	88.93635	133.5135
88.738	134.9289	88.71553	130.159	88.82407	132.7479	88.76485	139.6525	88.74062	148.3721	88.95302	133.5131
88.75467	134.9275	88.7322	130.1576	88.84073	132.7479	88.78152	139.6503	88.75728	148.3674	88.96968	133.5121
88.77133	134.9278	88.74887	130.1578	88.8574	132.7483	88.79818	139.653	88.77395	148.3716	88.98635	133.509
88.788	134.927	88.76553	130.1575	88.87407	132.7481	88.81485	139.6513	88.79062	148.3737	89.00302	133.512
88.80467	134.9268	88.7822	130.1574	88.89073	132.7468	88.83152	139.6491	88.80728	148.3663	89.01968	133.5101
88.82133	134.9239	88.79887	130.1554	88.9074	132.7469	88.84818	139.6505	88.82395	148.371	89.03635	133.5093
88.838	134.9241	88.81553	130.156	88.92407	132.7432	88.86485	139.648	88.84062	148.3725	89.05302	133.5095
88.85467	134.9215	88.8322	130.1536	88.94073	132.7432	88.88152	139.6492	88.85728	148.3684	89.06968	133.5087
88.87133	134.921	88.84887	130.1549	88.9574	132.7468	88.89818	139.6487	88.87395	148.3699	89.08635	133.509
88.888	134.9195	88.86553	130.1539	88.97407	132.7445	88.91485	139.649	88.89062	148.3697	89.10302	133.5071
88.90467	134.9194	88.8822	130.1515	88.99073	132.7449	88.93152	139.6477	88.90728	148.3662	89.11968	133.5091
88.92133	134.919	88.89887	130.151	89.0074	132.7444	88.94818	139.6484	88.92395	148.3677	89.13635	133.5085
88.938	134.9183	88.91553	130.1505	89.02407	132.7426	88.96485	139.6455	88.94062	148.3736	89.15302	133.5077
88.95467	134.9145	88.9322	130.1505	89.04073	132.7397	88.98152	139.6472	88.95728	148.3694	89.16968	133.5064
88.97133	134.9137	88.94887	130.1476	89.0574	132.7435	88.99818	139.6447	88.97395	148.3703	89.18635	133.5057
88.988	134.9126	88.96553	130.1484	89.07407	132.7422	89.01485	139.6434	88.99062	148.3732	89.20302	133.5047
89.00467	134.916	88.9822	130.1457	89.09073	132.7402	89.03152	139.6436	89.00728	148.372	89.21968	133.5046
89.02133	134.9112	88.99887	130.145	89.1074	132.7412	89.04818	139.6408	89.02395	148.3675	89.23635	133.506
89.038	134.91	89.01553	130.1438	89.12407	132.7401	89.06485	139.6388	89.04062	148.3703	89.25302	133.5047
89.05467	134.9126	89.0322	130.1459	89.14073	132.7376	89.08152	139.6407	89.05728	148.3701	89.26968	133.5025
89.07133	134.9109	89.04887	130.1436	89.1574	132.7375	89.09818	139.6407	89.07395	148.3696	89.28635	133.5008
89.088	134.9072	89.06553	130.1408	89.17407	132.7382	89.11485	139.639	89.09062	148.3715	89.30302	133.5021
89.10467	134.9061	89.0822	130.1451	89.19073	132.7374	89.13152	139.6398	89.10728	148.3687	89.31968	133.5014
89.12133	134.9079	89.09887	130.1424	89.2074	132.737	89.14818	139.6397	89.12395	148.3704	89.33635	133.5014
89.138	134.9068	89.11553	130.1383	89.22407	132.7345	89.16485	139.6403	89.14062	148.3704	89.35302	133.5017
89.15467	134.9038	89.1322	130.14	89.24073	132.7375	89.18152	139.6405	89.15728	148.3697	89.36968	133.4996
89.17133	134.9019	89.14887	130.1443	89.2574	132.7334	89.19818	139.6363	89.17395	148.3709	89.38635	133.499
89.188	134.9043	89.16553	130.1353	89.27407	132.7315	89.21485	139.6378	89.19062	148.3692	89.40302	133.4978
89.20467	134.9007	89.1822	130.1399	89.29073	132.7321	89.23152	139.642	89.20728	148.3717	89.41968	133.4986
89.22133	134.8987	89.19887	130.1402	89.3074	132.7326	89.24818	139.6394	89.22395	148.3701	89.43635	133.4971
89.238	134.897	89.21553	130.1358	89.32407	132.7309	89.26485	139.6379	89.24062	148.3718	89.45302	133.4967
89.25467	134.9014	89.2322	130.1362	89.34073	132.7307	89.28152	139.6372	89.25728	148.3717	89.46968	133.4987
89.27133	134.9006	89.24887	130.1376	89.3574	132.7313	89.29818	139.6374	89.27395	148.3713	89.48635	133.4974
89.288	134.8965	89.26553	130.1336	89.37407	132.7325	89.31485	139.6373	89.29062	148.3718	89.50302	133.4974
89.30467	134.8995	89.2822	130.1313	89.39073	132.7288	89.33152	139.6359	89.30728	148.3688	89.51968	133.4959
89.32133	134.8942	89.29887	130.1307	89.4074	132.7315	89.34818	139.6355	89.32395	148.3707	89.53635	133.4949
89.338	134.8943	89.31553	130.1335	89.42407	132.7304	89.36485	139.6338	89.34062	148.3722	89.55302	133.4956
89.35467	134.897	89.3322	130.1329	89.44073	132.7305	89.38152	139.6343	89.35728	148.3708	89.56968	133.4957
89.37133	134.8926	89.34887	130.13	89.4574	132.7297	89.39818	139.6339	89.37395	148.3724	89.58635	133.4939
89.388	134.8927	89.36553	130.1301	89.47407	132.7277	89.41485	139.6316	89.39062	148.3729	89.60302	133.4949
89.40467	134.8959	89.3822	130.1305	89.49073	132.7278	89.43152	139.6318	89.40728	148.3738	89.61968	133.4935
89.42133	134.8921	89.39887	130.126	89.5074	132.7272	89.44818	139.6313	89.42395	148.3738	89.63635	133.4926
89.438	134.8912	89.41553	130.1292	89.52407	132.7288	89.46485	139.6349	89.44062	148.3716	89.65302	133.4928
89.45467	134.8914	89.4322	130.1299	89.54073	132.7277	89.48152	139.6303	89.45728	148.3726	89.66968	133.491
89.47133	134.8921	89.44887	130.1223	89.5574	132.7279	89.49818	139.6306	89.47395	148.3718	89.68635	133.4899
89.488	134.8899	89.46553	130.1222	89.57407	132.7261	89.51485	139.6311	89.49062	148.3712	89.70302	133.4906
89.50467	134.8873	89.4822	130.125	89.59073	132.7301	89.53152	139.6282	89.50728	148.3729	89.71968	133.4907
89.52133	134.8873	89.49887	130.1277	89.6074	132.7255	89.54818	139.6293	89.52395	148.3735	89.73635	133.4873
89.538	134.8863	89.51553	130.1206	89.62407	132.7275	89.56485	139.6278	89.54062	148.3737	89.75302	133.4884
89.55467	134.886	89.5322	130.1213	89.64073	132.7283	89.58152	139.6273	89.55728	148.3726	89.76968	133.4846
89.57133	134.8837	89.54887	130.1226	89.6574	132.7253	89.59818	139.6257	89.57395	148.3751	89.78635	133.4837
89.588	134.8811	89.56553	130.123	89.67407	132.7306	89.61485	139.6296	89.59062	148.3736	89.80302	133.4819
89.60467	134.8827	89.5822	130.1214	89.69073	132.7286	89.63152	139.6273	89.60728	148.3749	89.81968	133.4835
89.62133	134.8795	89.59887	130.1227	89.7074	132.7272	89.64818	139.6257	89.62395	148.3753	89.83635	133.4813
89.638	134.8803	89.61553	130.1189	89.72407	132.7261	89.66485	139.6273	89.64062	148.3754	89.85302	133.4807
89.65467	134.8803	89.6322	130.1197	89.74073	132.7273	89.68152	139.6262	89.65728	148.3714	89.86968	133.4801
89.67133	134.8764	89.64887	130.1146	89.7574	132.7274	89.69818	139.6268	89.67395	148.3724	89.88635	133.4797

89.688	134.8783	89.66553	130.1148	89.77407	132.729	89.71485	139.6284	89.69062	148.3733	89.90302	133.478
89.70467	134.8759	89.6822	130.116	89.79073	132.7273	89.73152	139.6258	89.70728	148.3736	89.91968	133.4785
89.72133	134.8751	89.69887	130.1149	89.8074	132.7248	89.74818	139.6244	89.72395	148.3758	89.93635	133.4766
89.738	134.8739	89.71553	130.115	89.82407	132.7268	89.76485	139.6258	89.74062	148.3708	89.95302	133.4783
89.75467	134.8742	89.7322	130.1139	89.84073	132.7254	89.78152	139.6271	89.75728	148.3719	89.96968	133.4769
89.77133	134.8748	89.74887	130.1106	89.8574	132.7243	89.79818	139.6244	89.77395	148.3721	89.98635	133.4763
89.788	134.8716	89.76553	130.1109	89.87407	132.724	89.81485	139.6254	89.79062	148.3733	90.00302	133.4775
89.80467	134.8706	89.7822	130.1101	89.89073	132.7223	89.83152	139.6246	89.80728	148.3742	90.01968	133.4766
89.82133	134.8711	89.79887	130.1098	89.9074	132.7212	89.84818	139.625	89.82395	148.3713	90.03635	133.4745
89.838	134.8709	89.81553	130.1078	89.92407	132.7212	89.86485	139.6251	89.84062	148.3718	90.05302	133.4727
89.85467	134.8677	89.8322	130.1085	89.94073	132.7201	89.88152	139.6239	89.85728	148.3725	90.06968	133.475
89.87133	134.8687	89.84887	130.1075	89.9574	132.7194	89.89818	139.6236	89.87395	148.3712	90.08635	133.4727
89.888	134.8691	89.86553	130.1067	89.97407	132.7173	89.91485	139.6223	89.89062	148.3705	90.10302	133.4721
89.90467	134.8663	89.8822	130.1039	89.99073	132.7149	89.93152	139.6233	89.90728	148.3719	90.11968	133.4714
89.92133	134.8681	89.89887	130.1035	90.0074	132.7152	89.94818	139.6215	89.92395	148.3742	90.13635	133.4744
89.938	134.864	89.91553	130.1037	90.02407	132.7122	89.96485	139.6226	89.94062	148.3739	90.15302	133.4703
89.95467	134.865	89.9322	130.1022	90.04073	132.7139	89.98152	139.6233	89.95728	148.3738	90.16968	133.4698
89.97133	134.8643	89.94887	130.1016	90.0574	132.7104	89.99818	139.6214	89.97395	148.3736	90.18635	133.4717
89.988	134.8605	89.96553	130.102	90.07407	132.7089	90.01485	139.6231	89.99062	148.3712	90.20302	133.4694
90.00467	134.8617	89.9822	130.0999	90.09073	132.7093	90.03152	139.621	90.00728	148.3714	90.21968	133.4691
90.02133	134.8612	89.99887	130.1002	90.1074	132.7091	90.04818	139.6187	90.02395	148.3706	90.23635	133.4677
90.038	134.8619	90.01553	130.0997	90.12407	132.7073	90.06485	139.6204	90.04062	148.372	90.25302	133.4677
90.05467	134.8589	90.0322	130.0984	90.14073	132.706	90.08152	139.6174	90.05728	148.3733	90.26968	133.4686
90.07133	134.8575	90.04887	130.097	90.1574	132.7063	90.09818	139.6171	90.07395	148.3737	90.28635	133.4671
90.088	134.8561	90.06553	130.0945	90.17407	132.7046	90.11485	139.6189	90.09062	148.3712	90.30302	133.4681
90.10467	134.8564	90.0822	130.0956	90.19073	132.7078	90.13152	139.6179	90.10728	148.3725	90.31968	133.4698
90.12133	134.8568	90.09887	130.0953	90.2074	132.7048	90.14818	139.6213	90.12395	148.3713	90.33635	133.4682
90.138	134.8574	90.11553	130.0962	90.22407	132.7055	90.16485	139.6192	90.14062	148.3712	90.35302	133.4659
90.15467	134.859	90.1322	130.0955	90.24073	132.7031	90.18152	139.6173	90.15728	148.3728	90.36968	133.466
90.17133	134.8584	90.14887	130.0971	90.2574	132.7035	90.19818	139.6181	90.17395	148.3744	90.38635	133.4696
90.188	134.8586	90.16553	130.0953	90.27407	132.7049	90.21485	139.6186	90.19062	148.3705	90.40302	133.4644
90.20467	134.8563	90.1822	130.0954	90.29073	132.7011	90.23152	139.6184	90.20728	148.372	90.41968	133.4672
90.22133	134.8625	90.19887	130.0923	90.3074	132.7008	90.24818	139.6169	90.22395	148.3737	90.43635	133.4672
90.238	134.8796	90.21553	130.0919	90.32407	132.7012	90.26485	139.6144	90.24062	148.3733	90.45302	133.4641
90.25467	134.9591	90.2322	130.0925	90.34073	132.6998	90.28152	139.6138	90.25728	148.3752	90.46968	133.4661
90.27133	134.975	90.24887	130.0907	90.3574	132.701	90.29818	139.6157	90.27395	148.3728	90.48635	133.4671
90.288	134.9887	90.26553	130.0885	90.37407	132.6994	90.31485	139.6149	90.29062	148.371	90.50302	133.4631
90.30467	134.991	90.2822	130.0909	90.39073	132.6999	90.33152	139.6149	90.30728	148.3727	90.51968	133.4652
90.32133	134.9878	90.29887	130.0884	90.4074	132.6991	90.34818	139.6136	90.32395	148.375	90.53635	133.4628
90.338	134.9898	90.31553	130.0877	90.42407	132.6974	90.36485	139.6131	90.34062	148.3733	90.55302	133.4629
90.35467	134.9874	90.3322	130.0872	90.44073	132.698	90.38152	139.6166	90.35728	148.3731	90.56968	133.4634
90.37133	134.9928	90.34887	130.0894	90.4574	132.6939	90.39818	139.6137	90.37395	148.3763	90.58635	133.4626
90.388	134.9817	90.36553	130.0884	90.47407	132.7016	90.41485	139.6155	90.39062	148.3719	90.60302	133.4626
90.40467	134.9862	90.3822	130.0879	90.49073	132.6952	90.43152	139.6145	90.40728	148.3722	90.61968	133.4624
90.42133	134.981	90.39887	130.0843	90.5074	132.694	90.44818	139.6128	90.42395	148.3743	90.63635	133.4606
90.438	134.9881	90.41553	130.0845	90.52407	132.6964	90.46485	139.6115	90.44062	148.3721	90.65302	133.4604
90.45467	134.9874	90.4322	130.0816	90.54073	132.6955	90.48152	139.6127	90.45728	148.373	90.66968	133.46
90.47133	134.986	90.44887	130.0824	90.5574	132.6977	90.49818	139.6121	90.47395	148.3731	90.68635	133.4604
90.488	134.9815	90.46553	130.0812	90.57407	132.6929	90.51485	139.6134	90.49062	148.3738	90.70302	133.4572
90.50467	134.9828	90.4822	130.0822	90.59073	132.6938	90.53152	139.6099	90.50728	148.3711	90.71968	133.4621
90.52133	134.9804	90.49887	130.0814	90.6074	132.6955	90.54818	139.6097	90.52395	148.37	90.73635	133.4559
90.538	134.9868	90.51553	130.0809	90.62407	132.6941	90.56485	139.6131	90.54062	148.3753	90.75302	133.4563
90.55467	134.9832	90.5322	130.0803	90.64073	132.6929	90.58152	139.61	90.55728	148.3714	90.76968	133.4557
90.57133	134.9838	90.54887	130.0764	90.6574	132.6911	90.59818	139.609	90.57395	148.3743	90.78635	133.4561
90.588	134.9751	90.56553	130.0761	90.67407	132.6905	90.61485	139.6107	90.59062	148.3723	90.80302	133.45
90.60467	134.9764	90.5822	130.0776	90.69073	132.692	90.63152	139.6087	90.60728	148.3698	90.81968	133.4559
90.62133	134.9787	90.59887	130.074	90.7074	132.689	90.64818	139.6115	90.62395	148.37	90.83635	133.4539
90.638	134.9777	90.61553	130.0702	90.72407	132.6894	90.66485	139.6069	90.64062	148.3695	90.85302	133.4521
90.65467	134.9787	90.6322	130.0697	90.74073	132.6904	90.68152	139.6077	90.65728	148.3735	90.86968	133.4534
90.67133	134.9757	90.64887	130.0719	90.7574	132.6893	90.69818	139.6068	90.67395	148.3688	90.88635	133.4528
90.688	134.9722	90.66553	130.0738	90.77407	132.6884	90.71485	139.6065	90.69062	148.375	90.90302	133.4535
90.70467	134.9766	90.6822	130.0727	90.79073	132.6861	90.73152	139.6066	90.70728	148.3715	90.91968	133.4515
90.72133	134.9688	90.69887	130.0743	90.8074	132.6838	90.74818	139.6074	90.72395	148.3723	90.93635	133.4511
90.738	134.9812	90.71553	130.073	90.82407	132.6848	90.76485	139.6056	90.74062	148.3729	90.95302	133.4503
90.75467	134.9728	90.7322	130.068	90.84073	132.6836	90.78152	139.6037	90.75728	148.3742	90.96968	133.4489
90.77133	134.9656	90.74887	130.0651	90.8574	132.6844	90.79818	139.6042	90.77395	148.3741	90.98635	133.4503
90.788	134.9733	90.76553	130.0668	90.87407	132.6887	90.81485	139.6042	90.79062	148.3764	91.00302	133.449
90.80467	134.9688	90.7822	130.067	90.89073	132.6852	90.83152	139.6043	90.80728	148.3764	91.01968	133.4494
90.82133	134.9673	90.79887	130.0694	90.9074	132.6844	90.84818	139.6045	90.82395	148.3743	91.03635	133.4479

90.838	134.9727	90.81553	130.0665	90.92407	132.6817	90.86485	139.603	90.84062	148.377	91.05302	133.4473
90.85467	134.9685	90.8322	130.0636	90.94073	132.6823	90.88152	139.6037	90.85728	148.3739	91.06968	133.4453
90.87133	134.9677	90.84887	130.0678	90.9574	132.6818	90.89818	139.6051	90.87395	148.3764	91.08635	133.4463
90.888	134.9669	90.86553	130.0638	90.97407	132.6813	90.91485	139.6038	90.89062	148.3781	91.10302	133.4431
90.90467	134.9683	90.8822	130.0601	90.99073	132.6784	90.93152	139.6013	90.90728	148.3774	91.11968	133.4454
90.92133	134.9632	90.89887	130.064	91.0074	132.6788	90.94818	139.603	90.92395	148.3749	91.13635	133.4433
90.938	134.9634	90.91553	130.0647	91.02407	132.6802	90.96485	139.6017	90.94062	148.3767	91.15302	133.4442
90.95467	134.9645	90.9322	130.0612	91.04073	132.6799	90.98152	139.5999	90.95728	148.3802	91.16968	133.4414
90.97133	134.9604	90.94887	130.0605	91.0574	132.6809	90.99818	139.5998	90.97395	148.3736	91.18635	133.4426
90.988	134.9608	90.96553	130.0593	91.07407	132.6807	91.01485	139.6003	90.99062	148.376	91.20302	133.4405
91.00467	134.9592	90.9822	130.0588	91.09073	132.6787	91.03152	139.5997	91.00728	148.3764	91.21968	133.44
91.02133	134.9607	90.99887	130.0561	91.1074	132.6739	91.04818	139.5983	91.02395	148.3768	91.23635	133.4422
91.038	134.9598	91.01553	130.0541	91.12407	132.6776	91.06485	139.5971	91.04062	148.3767	91.25302	133.4401
91.05467	134.9548	91.0322	130.0543	91.14073	132.6793	91.08152	139.5985	91.05728	148.3783	91.26968	133.4406
91.07133	134.9567	91.04887	130.0552	91.1574	132.6757	91.09818	139.5985	91.07395	148.3793	91.28635	133.4403
91.088	134.9528	91.06553	130.0516	91.17407	132.6771	91.11485	139.5975	91.09062	148.3775	91.30302	133.4392
91.10467	134.9525	91.0822	130.0523	91.19073	132.6755	91.13152	139.5981	91.10728	148.3781	91.31968	133.4391
91.12133	134.955	91.09887	130.0522	91.2074	132.6744	91.14818	139.5983	91.12395	148.3763	91.33635	133.4374
91.138	134.9486	91.11553	130.0525	91.22407	132.6773	91.16485	139.5965	91.14062	148.376	91.35302	133.4377
91.15467	134.9551	91.1322	130.0488	91.24073	132.6777	91.18152	139.5957	91.15728	148.3789	91.36968	133.4382
91.15728	135.5242	91.14887	130.047	91.2574	132.6749	91.19818	139.5979	91.17395	148.3797	91.38635	133.4368
91.15737	136.1525	91.16553	130.0496	91.27407	132.6737	91.21485	139.5944	91.19062	148.3779	91.40302	133.4365
91.15743	136.6777	91.1822	130.0494	91.29073	132.6739	91.23152	139.5941	91.20728	148.3782	91.41968	133.4369
91.1575	137.2187	91.19887	130.0463	91.3074	132.6766	91.24818	139.5958	91.22395	148.3806	91.43635	133.4337
91.15758	137.8217	91.21553	130.0485	91.32407	132.6737	91.26485	139.5937	91.24062	148.3785	91.45302	133.4328
91.15767	138.3469	91.2322	130.045	91.34073	132.6734	91.28152	139.5942	91.25728	148.3761	91.46968	133.4333
91.15777	138.8528	91.24887	130.0431	91.3574	132.6727	91.29818	139.5939	91.27395	148.3782	91.48635	133.4333
91.15793	139.386	91.26553	130.0423	91.37407	132.6722	91.31485	139.5924	91.29062	148.3795	91.50302	133.4332
91.1746	139.096	91.2822	130.0469	91.39073	132.6727	91.33152	139.5931	91.30728	148.3801	91.51968	133.4337
91.19127	139.0024	91.29887	130.0424	91.4074	132.6697	91.34818	139.5917	91.32395	148.3792	91.53635	133.432
91.20793	138.945	91.31553	130.0435	91.42407	132.6712	91.36485	139.5914	91.34062	148.3813	91.55302	133.4334
91.2246	138.909	91.3322	130.046	91.44073	132.6717	91.38152	139.5912	91.35728	148.3791	91.56968	133.4333
91.24127	138.8729	91.34887	130.0423	91.4574	132.6725	91.39818	139.5914	91.37395	148.3791	91.58635	133.4314
91.25793	138.8489	91.36553	130.0375	91.47407	132.6677	91.41485	139.5903	91.39062	148.3787	91.60302	133.4309
91.2746	138.8258	91.3822	130.0434	91.49073	132.6692	91.43152	139.5895	91.40728	148.3784	91.61968	133.4295
91.29127	138.8004	91.39887	130.04	91.5074	132.67	91.44818	139.5914	91.42395	148.3817	91.63635	133.4288
91.30793	138.7875	91.41553	130.0378	91.52407	132.668	91.46485	139.5924	91.44062	148.38	91.65302	133.4319
91.3246	138.7713	91.4322	130.0402	91.54073	132.6675	91.48152	139.5924	91.45728	148.3792	91.66968	133.4295
91.34127	138.7576	91.44887	130.0369	91.5574	132.6649	91.49818	139.591	91.47395	148.3809	91.68635	133.4283
91.35793	138.7451	91.46553	130.0363	91.57407	132.6642	91.51485	139.5924	91.49062	148.3783	91.70302	133.4264
91.3746	138.7329	91.4822	130.0364	91.59073	132.6653	91.53152	139.5908	91.50728	148.3794	91.71968	133.4285
91.39127	138.7189	91.49887	130.033	91.6074	132.6652	91.54818	139.5899	91.52395	148.3811	91.73635	133.4266
91.40793	138.7086	91.51553	130.0323	91.62407	132.6642	91.56485	139.5908	91.54062	148.3824	91.75302	133.4275
91.4246	138.6992	91.5322	130.0339	91.64073	132.6644	91.58152	139.5915	91.55728	148.3821	91.76968	133.4262
91.44127	138.6851	91.54887	130.0318	91.6574	132.6643	91.59818	139.5912	91.57395	148.3808	91.78635	133.4264
91.45793	138.6768	91.56553	130.0284	91.67407	132.6622	91.61485	139.5912	91.59062	148.3813	91.80302	133.4261
91.4746	138.6695	91.5822	130.0272	91.69073	132.6624	91.63152	139.5887	91.60728	148.3798	91.81968	133.4257
91.49127	138.6604	91.59887	130.0281	91.7074	132.6637	91.64818	139.5891	91.62395	148.3802	91.83635	133.4255
91.50793	138.6497	91.61553	130.0271	91.72407	132.6622	91.66485	139.5878	91.64062	148.383	91.85302	133.4257
91.5246	138.6401	91.6322	130.0254	91.74073	132.6604	91.68152	139.588	91.65728	148.3813	91.86968	133.4251
91.54127	138.6347	91.64887	130.0289	91.7574	132.6593	91.69818	139.5899	91.67395	148.3824	91.88635	133.425
91.55793	138.6259	91.66553	130.0233	91.77407	132.6598	91.71485	139.5888	91.69062	148.3796	91.90302	133.4246
91.5746	138.6183	91.6822	130.027	91.79073	132.6614	91.73152	139.5843	91.70728	148.3822	91.91968	133.4263
91.59127	138.6136	91.69887	130.0207	91.8074	132.6584	91.74818	139.5856	91.72395	148.3828	91.93635	133.4232
91.60793	138.6056	91.71553	130.0227	91.82407	132.6575	91.76485	139.5847	91.74062	148.3811	91.95302	133.4217
91.6246	138.6	91.7322	130.0219	91.84073	132.657	91.78152	139.5865	91.75728	148.3807	91.96968	133.4225
91.64127	138.5937	91.74887	130.0205	91.8574	132.6583	91.79818	139.5883	91.77395	148.3831	91.98635	133.4213
91.65793	138.5825	91.76553	130.0205	91.87407	132.6574	91.81485	139.5848	91.79062	148.3813	92.00302	133.4209
91.6746	138.5774	91.7822	130.0231	91.89073	132.6566	91.83152	139.5848	91.80728	148.38	92.01968	133.4194
91.69127	138.5742	91.79887	130.0177	91.9074	132.656	91.84818	139.5869	91.82395	148.3826	92.03635	133.4194
91.70793	138.5675	91.81553	130.0202	91.92407	132.6553	91.86485	139.5856	91.84062	148.382	92.05302	133.4202
91.7246	138.561	91.8322	130.0194	91.94073	132.655	91.88152	139.585	91.85728	148.3818	92.06968	133.4186
91.74127	138.5562	91.84887	130.0178	91.9574	132.6568	91.89818	139.586	91.87395	148.3848	92.08635	133.4206
91.75793	138.5535	91.86553	130.0156	91.97407	132.6552	91.91485	139.5877	91.89062	148.3832	92.10302	133.4197
91.7746	138.5442	91.8822	130.0175	91.99073	132.6553	91.93152	139.5879	91.90728	148.3815	92.11968	133.4174
91.79127	138.5384	91.89887	130.0135	92.0074	132.6543	91.94818	139.5852	91.92395	148.3836	92.13635	133.4193
91.80793	138.5355	91.91553	130.0108	92.02407	132.6552	91.96485	139.5849	91.94062	148.3838	92.15302	133.418
91.8246	138.5313	91.9322	130.0118	92.04073	132.6554	91.98152	139.5876	91.95728	148.3816	92.16968	133.4158
91.84127	138.5269	91.94887	130.0139	92.0574	132.6541	91.99818	139.5865	91.97395	148.3861	92.18635	133.4163

91.85793	138.522	91.96553	130.0126	92.07407	132.6528	92.01485	139.5862	91.99062	148.3834	92.20302	133.4159
91.8746	138.5175	91.9822	130.0113	92.09073	132.6547	92.03152	139.5835	92.00728	148.3857	92.21968	133.4174
91.89127	138.5129	91.99887	130.0104	92.1074	132.652	92.04818	139.5837	92.02395	148.3821	92.23635	133.4138
91.90793	138.5053	92.01553	130.0101	92.12407	132.6527	92.06485	139.5827	92.04062	148.382	92.25302	133.4144
91.9246	138.5036	92.0322	130.0079	92.14073	132.652	92.08152	139.5843	92.05728	148.3807	92.26968	133.4152
91.94127	138.4974	92.04887	130.0098	92.1574	132.6528	92.09818	139.5829	92.07395	148.3828	92.28635	133.4135
91.95793	138.4915	92.06553	130.0067	92.17407	132.6522	92.11485	139.583	92.09062	148.385	92.30302	133.4106
91.9746	138.4863	92.0822	130.0063	92.19073	132.6515	92.13152	139.5819	92.10728	148.3829	92.31968	133.4135
91.99127	138.4873	92.09887	130.008	92.2074	132.65	92.14818	139.5799	92.12395	148.3848	92.33635	133.4117
92.00793	138.478	92.11553	130.0058	92.22407	132.6497	92.16485	139.5813	92.14062	148.3852	92.35302	133.4094
92.0246	138.4779	92.1322	130.0052	92.24073	132.6516	92.18152	139.5799	92.15728	148.384	92.36968	133.4078
92.04127	138.4713	92.14887	130.0074	92.2574	132.6504	92.19818	139.579	92.17395	148.3837	92.38635	133.4089
92.05793	138.4653	92.16553	130.0043	92.27407	132.6509	92.21485	139.5776	92.19062	148.3849	92.40302	133.4058
92.0746	138.4585	92.1822	130.0045	92.29073	132.6497	92.23152	139.5787	92.20728	148.3831	92.41968	133.4057
92.09127	138.459	92.19887	130.0045	92.3074	132.6479	92.24818	139.5781	92.22395	148.3853	92.43635	133.4062
92.10793	138.4542	92.21553	130.0001	92.32407	132.6492	92.26485	139.5785	92.24062	148.3828	92.45302	133.4052
92.1246	138.4529	92.2322	130.0011	92.34073	132.6471	92.28152	139.5776	92.25728	148.3818	92.46968	133.4022
92.14127	138.4463	92.24887	130.0017	92.3574	132.6472	92.29818	139.5756	92.27395	148.3832	92.48635	133.4021
92.15793	138.4414	92.26553	129.9996	92.37407	132.6457	92.31485	139.5765	92.29062	148.3866	92.50302	133.403
92.1746	138.4452	92.2822	129.9988	92.39073	132.6506	92.33152	139.5783	92.30728	148.3853	92.51968	133.4025
92.19127	138.4387	92.29887	129.998	92.4074	132.6422	92.34818	139.5767	92.32395	148.387	92.53635	133.4017
92.20793	138.4372	92.31553	129.997	92.42407	132.65	92.36485	139.5756	92.34062	148.3867	92.55302	133.4018
92.2246	138.4317	92.3322	129.9999	92.44073	132.6459	92.38152	139.5759	92.35728	148.3867	92.56968	133.3982
92.24127	138.4271	92.34887	129.9981	92.4574	132.6436	92.39818	139.5736	92.37395	148.3887	92.58635	133.3978
92.25793	138.4245	92.36553	129.9975	92.47407	132.6455	92.41485	139.5728	92.39062	148.387	92.60302	133.3977
92.2746	138.4198	92.3822	129.9939	92.49073	132.645	92.43152	139.5736	92.40728	148.3872	92.61968	133.3967
92.29127	138.4185	92.39887	129.9955	92.5074	132.6423	92.44818	139.5723	92.42395	148.3854	92.63635	133.3949
92.30793	138.4135	92.41553	129.9924	92.52407	132.6416	92.46485	139.5737	92.44062	148.3879	92.65302	133.3969
92.3246	138.41	92.4322	129.9925	92.54073	132.6416	92.48152	139.5744	92.45728	148.3846	92.66968	133.3941
92.34127	138.4081	92.44887	129.9898	92.5574	132.6425	92.49818	139.5705	92.47395	148.3845	92.68635	133.3932
92.35793	138.4021	92.46553	129.9931	92.57407	132.6423	92.51485	139.5706	92.49062	148.3845	92.70302	133.3928
92.3746	138.3996	92.4822	129.9926	92.59073	132.6431	92.53152	139.5718	92.50728	148.3838	92.71968	133.3927
92.39127	138.3957	92.49887	129.9877	92.6074	132.6403	92.54818	139.5738	92.52395	148.3822	92.73635	133.3894
92.40793	138.396	92.51553	129.9925	92.62407	132.6406	92.56485	139.5714	92.54062	148.3841	92.75302	133.3916
92.4246	138.3927	92.5322	129.9852	92.64073	132.6422	92.58152	139.5719	92.55728	148.3854	92.76968	133.3899
92.44127	138.3879	92.54887	129.9856	92.6574	132.6396	92.59818	139.5695	92.57395	148.3864	92.78635	133.3892
92.45793	138.383	92.56553	129.9877	92.67407	132.639	92.61485	139.5707	92.59062	148.3826	92.80302	133.3878
92.4746	138.3793	92.5822	129.986	92.69073	132.6402	92.63152	139.5703	92.60728	148.3834	92.81968	133.3887
92.49127	138.3804	92.59887	129.9886	92.7074	132.6391	92.64818	139.569	92.62395	148.3865	92.83635	133.3845
92.50793	138.3735	92.61553	129.9875	92.72407	132.6382	92.66485	139.5686	92.64062	148.3822	92.85302	133.3868
92.5246	138.3719	92.6322	129.9793	92.74073	132.637	92.68152	139.5693	92.65728	148.3816	92.86968	133.3849
92.54127	138.3695	92.64887	129.9821	92.7574	132.6367	92.69818	139.5671	92.67395	148.3822	92.88635	133.3841
92.55793	138.3658	92.66553	129.9839	92.77407	132.6355	92.71485	139.5694	92.69062	148.3854	92.90302	133.383
92.5746	138.3641	92.6822	129.9849	92.79073	132.6377	92.73152	139.567	92.70728	148.3837	92.91968	133.3829
92.59127	138.3613	92.69887	129.9815	92.8074	132.6364	92.74818	139.5661	92.72395	148.3848	92.93635	133.3819
92.60793	138.3561	92.71553	129.9828	92.82407	132.6349	92.76485	139.5663	92.74062	148.385	92.95302	133.3829
92.6246	138.3538	92.7322	129.9824	92.84073	132.636	92.78152	139.5665	92.75728	148.3836	92.96968	133.383
92.64127	138.3528	92.74887	129.9815	92.8574	132.6326	92.79818	139.5639	92.77395	148.3854	92.98635	133.3811
92.65793	138.35	92.76553	129.9781	92.87407	132.635	92.81485	139.5673	92.79062	148.386	93.00302	133.3797
92.6746	138.3488	92.7822	129.9805	92.89073	132.637	92.83152	139.5652	92.80728	148.3844	93.01968	133.3825
92.69127	138.3444	92.79887	129.9809	92.9074	132.6347	92.84818	139.5637	92.82395	148.384	93.03635	133.3754
92.70793	138.34	92.81553	129.9817	92.92407	132.6347	92.86485	139.5624	92.84062	148.387	93.05302	133.3781
92.7246	138.3395	92.8322	129.977	92.94073	132.6327	92.88152	139.5629	92.85728	148.3855	93.06968	133.3776
92.74127	138.3384	92.84887	129.9774	92.9574	132.6304	92.89818	139.5611	92.87395	148.3813	93.08635	133.3775
92.75793	138.3355	92.86553	129.9762	92.97407	132.6323	92.91485	139.563	92.89062	148.3834	93.10302	133.375
92.7746	138.3319	92.8822	129.9763	92.99073	132.6297	92.93152	139.561	92.90728	148.3862	93.11968	133.3775
92.79127	138.3294	92.89887	129.9765	93.0074	132.633	92.94818	139.5621	92.92395	148.3842	93.13635	133.3777
92.80793	138.3235	92.91553	129.9768	93.02407	132.6298	92.96485	139.5606	92.94062	148.3852	93.15302	133.3735
92.8246	138.3237	92.9322	129.9727	93.04073	132.6308	92.98152	139.5592	92.95728	148.3819	93.16968	133.3777
92.84127	138.32	92.94887	129.9744	93.0574	132.6293	92.99818	139.5587	92.97395	148.3819	93.18635	133.3743
92.85793	138.3193	92.96553	129.9734	93.07407	132.6286	93.01485	139.5588	92.99062	148.3813	93.20302	133.3744
92.8746	138.3161	92.9822	129.9722	93.09073	132.6275	93.03152	139.5604	93.00728	148.3821	93.21968	133.3727
92.89127	138.3144	92.99887	129.9678	93.1074	132.6253	93.04818	139.5577	93.02395	148.3876	93.23635	133.3706
92.90793	138.3104	93.01553	129.9679	93.12407	132.628	93.06485	139.5588	93.04062	148.3834	93.25302	133.3736
92.9246	138.3082	93.0322	129.9709	93.14073	132.6304	93.08152	139.5596	93.05728	148.3844	93.26968	133.3698
92.94127	138.3041	93.04887	129.9709	93.1574	132.6302	93.09818	139.5578	93.07395	148.3856	93.28635	133.369
92.95793	138.3034	93.06553	129.9652	93.17407	132.628	93.11485	139.5606	93.09062	148.3814	93.30302	133.3702
92.9746	138.2992	93.0822	129.9674	93.19073	132.6281	93.13152	139.5568	93.10728	148.3826	93.31968	133.37
92.99127	138.298	93.09887	129.9669	93.2074	132.6284	93.14818	139.5576	93.12395	148.3851	93.33635	133.3672

93.00793	138.2941	93.11553	129.9685	93.22407	132.6247	93.16485	139.5562	93.14062	148.3831	93.35302	133.3694
93.0246	138.291	93.1322	129.9661	93.24073	132.6276	93.18152	139.5572	93.15728	148.3806	93.36968	133.3682
93.04127	138.2908	93.14887	129.9663	93.2574	132.6289	93.19818	139.5567	93.17395	148.3798	93.38635	133.3658
93.05793	138.2865	93.16553	129.9647	93.27407	132.6254	93.21485	139.5561	93.19062	148.3831	93.40302	133.3639
93.0746	138.2851	93.1822	129.9681	93.29073	132.6231	93.23152	139.5558	93.20728	148.3824	93.41968	133.3655
93.09127	138.2821	93.19887	129.9647	93.3074	132.6241	93.24818	139.5555	93.22395	148.3872	93.43635	133.3649
93.10793	138.2781	93.21553	129.966	93.32407	132.626	93.26485	139.5565	93.24062	148.3827	93.45302	133.3635
93.1246	138.275	93.2322	129.9641	93.34073	132.6222	93.28152	139.5566	93.25728	148.3843	93.46968	133.3633
93.14127	138.2736	93.24887	129.9626	93.3574	132.6215	93.29818	139.5549	93.27395	148.3854	93.48635	133.3629
93.15793	138.271	93.26553	129.9649	93.37407	132.6208	93.31485	139.5534	93.29062	148.3847	93.50302	133.3607
93.1746	138.2668	93.2822	129.9655	93.39073	132.6248	93.33152	139.554	93.30728	148.3875	93.51968	133.3619
93.19127	138.267	93.29887	129.9624	93.4074	132.6208	93.34818	139.5566	93.32395	148.3884	93.53635	133.3598
93.20793	138.2639	93.31553	129.9628	93.42407	132.62	93.36485	139.55	93.34062	148.3858	93.55302	133.3616
93.2246	138.2618	93.3322	129.9626	93.44073	132.622	93.38152	139.5529	93.35728	148.3876	93.56968	133.3574
93.24127	138.2603	93.34887	129.9598	93.4574	132.6194	93.39818	139.5517	93.37395	148.3859	93.58635	133.3599
93.25793	138.259	93.36553	129.9581	93.47407	132.6219	93.41485	139.5514	93.39062	148.3853	93.60302	133.3601
93.2746	138.2554	93.3822	129.9597	93.49073	132.6195	93.43152	139.5541	93.40728	148.386	93.61968	133.3558
93.29127	138.2532	93.39887	129.9634	93.5074	132.6181	93.44818	139.5532	93.42395	148.3867	93.63635	133.3579
93.30793	138.2502	93.41553	129.96	93.52407	132.6177	93.46485	139.5507	93.44062	148.3865	93.65302	133.358
93.3246	138.2479	93.4322	129.9562	93.54073	132.6172	93.48152	139.5509	93.45728	148.3867	93.66968	133.3566
93.34127	138.2461	93.44887	129.9532	93.5574	132.6205	93.49818	139.5521	93.47395	148.3871	93.68635	133.3558
93.35793	138.2434	93.46553	129.9588	93.57407	132.6176	93.51485	139.5518	93.49062	148.3867	93.70302	133.3566
93.3746	138.2423	93.4822	129.9608	93.59073	132.6183	93.53152	139.5523	93.50728	148.3844	93.71968	133.3561
93.39127	138.2405	93.49887	129.9564	93.6074	132.6189	93.54818	139.5528	93.52395	148.3859	93.73635	133.3542
93.40793	138.2383	93.51553	129.954	93.62407	132.6175	93.56485	139.5514	93.54062	148.3847	93.75302	133.3558
93.4246	138.2368	93.5322	129.9549	93.64073	132.6152	93.58152	139.5478	93.55728	148.3837	93.76968	133.3516
93.44127	138.2353	93.54887	129.9544	93.6574	132.6185	93.59818	139.5508	93.57395	148.3857	93.78635	133.353
93.45793	138.2333	93.56553	129.9519	93.67407	132.6183	93.61485	139.5482	93.59062	148.386	93.80302	133.354
93.4746	138.2292	93.5822	129.9539	93.69073	132.6172	93.63152	139.5482	93.60728	148.385	93.81968	133.3513
93.49127	138.2289	93.59887	129.9542	93.7074	132.6164	93.64818	139.5476	93.62395	148.3849	93.83635	133.3503
93.50793	138.2233	93.61553	129.9526	93.72407	132.616	93.66485	139.5475	93.64062	148.3862	93.85302	133.3504
93.5246	138.2236	93.6322	129.948	93.74073	132.6138	93.68152	139.5464	93.65728	148.3838	93.86968	133.3513
93.54127	138.2215	93.64887	129.953	93.7574	132.6166	93.69818	139.548	93.67395	148.3831	93.88635	133.3507
93.55793	138.2209	93.66553	129.9493	93.77407	132.6169	93.71485	139.5476	93.69062	148.3861	93.90302	133.3491
93.5746	138.2152	93.6822	129.9521	93.79073	132.6156	93.73152	139.5444	93.70728	148.3851	93.91968	133.3506
93.59127	138.2155	93.69887	129.9503	93.8074	132.6143	93.74818	139.5437	93.72395	148.3854	93.93635	133.3454
93.60793	138.2127	93.71553	129.9504	93.82407	132.6168	93.76485	139.5459	93.74062	148.3851	93.95302	133.3486
93.6246	138.2111	93.7322	129.9478	93.84073	132.6145	93.78152	139.5436	93.75728	148.3861	93.96968	133.3464
93.64127	138.2072	93.74887	129.9488	93.8574	132.616	93.79818	139.5437	93.77395	148.3855	93.98635	133.3473
93.65793	138.2047	93.76553	129.9492	93.87407	132.6164	93.81485	139.5453	93.79062	148.386	94.00302	133.346
93.6746	138.2051	93.7822	129.9487	93.89073	132.6187	93.83152	139.5411	93.80728	148.3837	94.01968	133.3446
93.69127	138.2008	93.79887	129.9488	93.9074	132.615	93.84818	139.5422	93.82395	148.3845	94.03635	133.3448
93.70793	138.1978	93.81553	129.9471	93.92407	132.6156	93.86485	139.5443	93.84062	148.3851	94.05302	133.3428
93.7246	138.1969	93.8322	129.9429	93.94073	132.6165	93.88152	139.5429	93.85728	148.3848	94.06968	133.3447
93.74127	138.1951	93.84887	129.9447	93.9574	132.6147	93.89818	139.5414	93.87395	148.3833	94.08635	133.3435
93.75793	138.1935	93.86553	129.9465	93.97407	132.6138	93.91485	139.5408	93.89062	148.3829	94.10302	133.3417
93.7746	138.1896	93.8822	129.944	93.99073	132.617	93.93152	139.5406	93.90728	148.3836	94.11968	133.3404
93.79127	138.1884	93.89887	129.9457	94.0074	132.6181	93.94818	139.543	93.92395	148.3857	94.13635	133.3399
93.80793	138.1883	93.91553	129.9422	94.02407	132.6152	93.96485	139.5415	93.94062	148.387	94.15302	133.3377
93.8246	138.1836	93.9322	129.9394	94.04073	132.6176	93.98152	139.5387	93.95728	148.3861	94.16968	133.3352
93.84127	138.1806	93.94887	129.9417	94.0574	132.6166	93.99818	139.5386	93.97395	148.3858	94.18635	133.3368
93.85793	138.1784	93.96553	129.9414	94.07407	132.6165	94.01485	139.5391	93.99062	148.3868	94.20302	133.3341
93.8746	138.1759	93.9822	129.9404	94.09073	132.6136	94.03152	139.5393	94.00728	148.3838	94.21968	133.3338
93.89127	138.1745	93.99887	129.9415	94.1074	132.6141	94.04818	139.5384	94.02395	148.3865	94.23635	133.3357
93.90793	138.1728	94.01553	129.941	94.12407	132.6139	94.06485	139.5383	94.04062	148.3847	94.25302	133.3358
93.9246	138.1729	94.0322	129.9379	94.14073	132.6132	94.08152	139.5405	94.05728	148.3872	94.26968	133.3341
93.94127	138.1686	94.04887	129.9399	94.1574	132.6149	94.09818	139.5348	94.07395	148.3845	94.28635	133.3341
93.95793	138.1656	94.06553	129.9392	94.17407	132.6128	94.11485	139.5383	94.09062	148.3841	94.30302	133.3338
93.9746	138.1654	94.0822	129.9399	94.19073	132.6102	94.13152	139.5346	94.10728	148.3835	94.31968	133.3336
93.99127	138.1627	94.09887	129.9356	94.2074	132.6105	94.14818	139.5343	94.12395	148.3836	94.33635	133.3321
94.00793	138.1589	94.11553	129.9383	94.22407	132.6097	94.16485	139.5347	94.14062	148.3827	94.35302	133.332
94.0246	138.1596	94.1322	129.9359	94.24073	132.6087	94.18152	139.5337	94.15728	148.383	94.36968	133.3317
94.04127	138.1598	94.14887	129.9378	94.2574	132.6089	94.19818	139.5324	94.17395	148.3843	94.38635	133.3323
94.05793	138.1558	94.16553	129.9373	94.27407	132.6073	94.21485	139.5312	94.19062	148.3826	94.40302	133.331
94.0746	138.1528	94.1822	129.9363	94.29073	132.6087	94.23152	139.5318	94.20728	148.382	94.41968	133.3305
94.09127	138.1523	94.19887	129.9355	94.3074	132.6098	94.24818	139.5309	94.22395	148.3831	94.43635	133.3303
94.10793	138.1509	94.21553	129.9307	94.32407	132.6083	94.26485	139.5335	94.24062	148.3815	94.45302	133.3288
94.1246	138.1484	94.2322	129.9333	94.34073	132.6099	94.28152	139.5295	94.25728	148.3829	94.46968	133.3281
94.14127	138.1492	94.24887	129.9343	94.3574	132.6093	94.29818	139.528	94.27395	148.3842	94.48635	133.3283

94.15793	138.1418	94.26553	129.9336	94.37407	132.6075	94.31485	139.529	94.29062	148.3832	94.50302	133.3256
94.1746	138.1419	94.2822	129.9307	94.39073	132.6091	94.33152	139.5273	94.30728	148.3841	94.51968	133.3273
94.19127	138.1408	94.29887	129.9327	94.4074	132.6069	94.34818	139.529	94.32395	148.3821	94.53635	133.3265
94.20793	138.1423	94.31553	129.933	94.42407	132.608	94.36485	139.5284	94.34062	148.3827	94.55302	133.3256
94.2246	138.1349	94.3322	129.9278	94.44073	132.6083	94.38152	139.5291	94.35728	148.3846	94.56968	133.3266
94.24127	138.1353	94.34887	129.9312	94.4574	132.6066	94.39818	139.5257	94.37395	148.3831	94.58635	133.3253
94.25793	138.1331	94.36553	129.9274	94.47407	132.6065	94.41485	139.5303	94.39062	148.3843	94.60302	133.3245
94.2746	138.1343	94.3822	129.9306	94.49073	132.6049	94.43152	139.5288	94.40728	148.3839	94.61968	133.322
94.29127	138.1308	94.39887	129.9271	94.5074	132.6052	94.44818	139.5277	94.42395	148.3856	94.63635	133.3228
94.30793	138.1266	94.41553	129.9276	94.52407	132.6074	94.46485	139.5287	94.44062	148.3833	94.65302	133.3216
94.3246	138.1273	94.4322	129.9301	94.54073	132.6046	94.48152	139.5268	94.45728	148.3867	94.66968	133.32
94.34127	138.1243	94.44887	129.9264	94.5574	132.6038	94.49818	139.5255	94.47395	148.386	94.68635	133.3211
94.35793	138.1207	94.46553	129.9249	94.57407	132.6056	94.51485	139.5265	94.49062	148.3851	94.70302	133.3191
94.3746	138.1198	94.4822	129.9246	94.59073	132.6049	94.53152	139.5276	94.50728	148.3836	94.71968	133.3189
94.39127	138.1185	94.49887	129.9207	94.6074	132.6048	94.54818	139.5264	94.52395	148.3856	94.73635	133.3187
94.40793	138.1171	94.51553	129.9226	94.62407	132.6036	94.56485	139.5244	94.54062	148.3854	94.75302	133.3207
94.4246	138.1112	94.5322	129.9226	94.64073	132.6023	94.58152	139.5257	94.55728	148.3843	94.76968	133.3188
94.44127	138.1102	94.54887	129.9227	94.6574	132.6051	94.59818	139.5247	94.57395	148.3847	94.78635	133.3177
94.45793	138.1124	94.56553	129.923	94.67407	132.6043	94.61485	139.528	94.59062	148.3857	94.80302	133.3178
94.4746	138.1059	94.5822	129.923	94.69073	132.6069	94.63152	139.5233	94.60728	148.385	94.81968	133.3181
94.49127	138.1068	94.59887	129.9185	94.7074	132.603	94.64818	139.5249	94.62395	148.384	94.83635	133.3175
94.50793	138.1007	94.61553	129.9227	94.72407	132.6038	94.66485	139.5242	94.64062	148.3857	94.85302	133.3161
94.5246	138.1	94.6322	129.9229	94.74073	132.6063	94.68152	139.5246	94.65728	148.3845	94.86968	133.3161
94.54127	138.0987	94.64887	129.921	94.7574	132.6068	94.69818	139.524	94.67395	148.3874	94.88635	133.3157
94.55793	138.0988	94.66553	129.9198	94.77407	132.607	94.71485	139.5228	94.69062	148.3876	94.90302	133.3157
94.5746	138.0957	94.6822	129.9221	94.79073	132.605	94.73152	139.5231	94.70728	148.3849	94.91968	133.3152
94.59127	138.0946	94.69887	129.9195	94.8074	132.6046	94.74818	139.5218	94.72395	148.384	94.93635	133.3176
94.60793	138.0904	94.71553	129.9193	94.82407	132.6069	94.76485	139.5239	94.74062	148.3836	94.95302	133.3162
94.6246	138.0908	94.7322	129.9216	94.84073	132.6055	94.78152	139.5207	94.75728	148.3845	94.96968	133.3146
94.64127	138.0891	94.74887	129.9195	94.8574	132.6075	94.79818	139.5227	94.77395	148.3835	94.98635	133.3141
94.65793	138.0872	94.76553	129.9169	94.87407	132.6054	94.81485	139.5233	94.79062	148.3856	95.00302	133.3142
94.6746	138.0815	94.7822	129.9187	94.89073	132.6062	94.83152	139.5218	94.80728	148.3826	95.01968	133.3144
94.69127	138.0854	94.79887	129.919	94.9074	132.6086	94.84818	139.5199	94.82395	148.3811	95.03635	133.313
94.70793	138.0784	94.81553	129.9169	94.92407	132.6072	94.86485	139.5197	94.84062	148.3829	95.05302	133.3139
94.7246	138.0788	94.8322	129.9213	94.94073	132.606	94.88152	139.5209	94.85728	148.3845	95.06968	133.3113
94.74127	138.0784	94.84887	129.919	94.9574	132.6057	94.89818	139.5205	94.87395	148.382	95.08635	133.311
94.75793	138.0763	94.86553	129.921	94.97407	132.6056	94.91485	139.5197	94.89062	148.3824	95.10302	133.3132
94.7746	138.0742	94.8822	129.9171	94.99073	132.6074	94.93152	139.5173	94.90728	148.3838	95.11968	133.3118
94.79127	138.0746	94.89887	129.9162	95.0074	132.6033	94.94818	139.5173	94.92395	148.3829	95.13635	133.3122
94.80793	138.0711	94.91553	129.9167	95.02407	132.6071	94.96485	139.5191	94.94062	148.3823	95.15302	133.3126
94.8246	138.0673	94.9322	129.918	95.04073	132.6039	94.98152	139.5177	94.95728	148.3843	95.16968	133.3099
94.84127	138.0701	94.94887	129.9168	95.0574	132.6033	94.99818	139.5196	94.97395	148.383	95.18635	133.3105
94.85793	138.0635	94.96553	129.9152	95.07407	132.6074	95.01485	139.5165	94.99062	148.3822	95.20302	133.3098
94.8746	138.0622	94.9822	129.9164	95.09073	132.604	95.03152	139.5141	95.00728	148.3817	95.21968	133.3088
94.89127	138.0604	94.99887	129.9191	95.1074	132.6053	95.04818	139.518	95.02395	148.3838	95.23635	133.3077
94.90793	138.062	95.01553	129.9167	95.12407	132.6045	95.06485	139.5171	95.04062	148.3823	95.25302	133.3085
94.9246	138.061	95.0322	129.9143	95.14073	132.6051	95.08152	139.5162	95.05728	148.3827	95.26968	133.3087
94.94127	138.0599	95.04887	129.9176	95.1574	132.6072	95.09818	139.5164	95.07395	148.3807	95.28635	133.3074
94.95793	138.0618	95.06553	129.9115	95.17407	132.6063	95.11485	139.5131	95.09062	148.3832	95.30302	133.3053
94.9746	138.0544	95.0822	129.9119	95.19073	132.6036	95.13152	139.5174	95.10728	148.381	95.31968	133.3048
94.99127	138.0531	95.09887	129.9139	95.2074	132.6054	95.14818	139.5137	95.12395	148.3808	95.33635	133.301
95.00793	138.0492	95.11553	129.9137	95.22407	132.6057	95.16485	139.5124	95.14062	148.3799	95.35302	133.3018
95.0246	138.0485	95.1322	129.9153	95.24073	132.6046	95.18152	139.5138	95.15728	148.3791	95.36968	133.3044
95.04127	138.045	95.14887	129.912	95.2574	132.6061	95.19818	139.5119	95.17395	148.38	95.38635	133.3016
95.05793	138.048	95.16553	129.9149	95.27407	132.6034	95.21485	139.5121	95.19062	148.3792	95.40302	133.3003
95.0746	138.0445	95.1822	129.9085	95.29073	132.6037	95.23152	139.5129	95.20728	148.3797	95.41968	133.3003
95.09127	138.0433	95.19887	129.9075	95.3074	132.6038	95.24818	139.5119	95.22395	148.3809	95.43635	133.2985
95.10793	138.0375	95.21553	129.9071	95.32407	132.601	95.26485	139.5125	95.24062	148.3809	95.45302	133.2977
95.1246	138.039	95.2322	129.9065	95.34073	132.6036	95.28152	139.509	95.25728	148.3821	95.46968	133.2956
95.14127	138.0357	95.24887	129.9096	95.3574	132.6033	95.29818	139.5141	95.27395	148.3814	95.48635	133.2958
95.15793	138.0347	95.26553	129.9079	95.37407	132.6024	95.31485	139.5116	95.29062	148.3829	95.50302	133.2932
95.1746	138.0304	95.2822	129.9034	95.39073	132.6	95.33152	139.5102	95.30728	148.3807	95.51968	133.2927
95.19127	138.0313	95.29887	129.9074	95.4074	132.5982	95.34818	139.5096	95.32395	148.3831	95.53635	133.2941
95.20793	138.029	95.31553	129.9085	95.42407	132.6028	95.36485	139.5102	95.34062	148.3807	95.55302	133.2918
95.2246	138.0269	95.3322	129.908	95.44073	132.5984	95.38152	139.509	95.35728	148.3816	95.56968	133.2922
95.24127	138.0235	95.34887	129.9044	95.4574	132.5998	95.39818	139.5108	95.37395	148.3808	95.58635	133.2915
95.25793	138.023	95.36553	129.901	95.47407	132.5963	95.41485	139.5089	95.39062	148.3819	95.60302	133.2912
95.2746	138.021	95.3822	129.9029	95.49073	132.5989	95.43152	139.5093	95.40728	148.3801	95.61968	133.2893
95.29127	138.025	95.39887	129.9043	95.5074	132.5978	95.44818	139.5077	95.42395	148.3807	95.63635	133.2921

95.30793	138.0198	95.41553	129.8973	95.52407	132.595	95.46485	139.5058	95.44062	148.3782	95.65302	133.2893
95.3246	138.0172	95.4322	129.8982	95.54073	132.5953	95.48152	139.5073	95.45728	148.3791	95.66968	133.2877
95.34127	138.0136	95.44887	129.8985	95.5574	132.5951	95.49818	139.5039	95.47395	148.3806	95.68635	133.2881
95.35793	138.0131	95.46553	129.9008	95.57407	132.5979	95.51485	139.5054	95.49062	148.3792	95.70302	133.2902
95.3746	138.0121	95.4822	129.9011	95.59073	132.5953	95.53152	139.5042	95.50728	148.3786	95.71968	133.2882
95.39127	138.0084	95.49887	129.8979	95.6074	132.5959	95.54818	139.5034	95.52395	148.3791	95.73635	133.285
95.40793	138.0068	95.51553	129.8922	95.62407	132.5963	95.56485	139.5048	95.54062	148.3778	95.75302	133.2868
95.4246	138.0059	95.5322	129.8938	95.64073	132.596	95.58152	139.5026	95.55728	148.3795	95.76968	133.2865
95.44127	138.0065	95.54887	129.8973	95.6574	132.5938	95.59818	139.5022	95.57395	148.3806	95.78635	133.2837
95.45793	138.0032	95.56553	129.895	95.67407	132.5944	95.61485	139.5	95.59062	148.3807	95.80302	133.284
95.4746	138.0017	95.5822	129.8916	95.69073	132.5958	95.63152	139.5022	95.60728	148.3816	95.81968	133.2843
95.49127	138.0011	95.59887	129.8915	95.7074	132.5939	95.64818	139.5009	95.62395	148.3818	95.83635	133.2822
95.50793	137.9993	95.61553	129.8934	95.72407	132.5949	95.66485	139.5012	95.64062	148.3814	95.85302	133.2803
95.5246	137.9974	95.6322	129.8903	95.74073	132.595	95.68152	139.5006	95.65728	148.3792	95.86968	133.2802
95.54127	137.9962	95.64887	129.8909	95.7574	132.5938	95.69818	139.501	95.67395	148.3791	95.88635	133.2793
95.55793	137.9951	95.66553	129.8938	95.77407	132.5942	95.71485	139.4995	95.69062	148.3814	95.90302	133.2783
95.5746	137.9934	95.6822	129.8918	95.79073	132.5981	95.73152	139.5008	95.70728	148.3818	95.91968	133.2783
95.59127	137.9888	95.69887	129.8901	95.8074	132.5948	95.74818	139.4989	95.72395	148.3829	95.93635	133.2789
95.60793	137.9917	95.71553	129.8895	95.82407	132.5945	95.76485	139.4994	95.74062	148.3841	95.95302	133.2767
95.6246	137.9874	95.7322	129.8887	95.84073	132.5951	95.78152	139.4983	95.75728	148.3802	95.96968	133.2753
95.64127	137.9872	95.74887	129.8895	95.8574	132.5926	95.79818	139.5	95.77395	148.3841	95.98635	133.2758
95.65793	137.9851	95.76553	129.8879	95.87407	132.5928	95.81485	139.4981	95.79062	148.3813	96.00302	133.2755
95.6746	137.9812	95.7822	129.8922	95.89073	132.5968	95.83152	139.4995	95.80728	148.3851	96.01968	133.2741
95.69127	137.9792	95.79887	129.8857	95.9074	132.595	95.84818	139.4982	95.82395	148.3808	96.03635	133.2725
95.70793	137.9771	95.81553	129.8892	95.92407	132.594	95.86485	139.496	95.84062	148.3832	96.05302	133.275
95.7246	137.9772	95.8322	129.8854	95.94073	132.5945	95.88152	139.4953	95.85728	148.3809	96.06968	133.2716
95.74127	137.9763	95.84887	129.8924	95.9574	132.594	95.89818	139.499	95.87395	148.3809	96.08635	133.2696
95.75793	137.9741	95.86553	129.8846	95.97407	132.5912	95.91485	139.4966	95.89062	148.3804	96.10302	133.2717
95.7746	137.9716	95.8822	129.884	95.99073	132.5906	95.93152	139.4969	95.90728	148.3816	96.11968	133.2691
95.79127	137.9713	95.89887	129.8854	96.0074	132.5927	95.94818	139.4959	95.92395	148.3785	96.13635	133.2662
95.80793	137.9698	95.91553	129.8827	96.02407	132.5892	95.96485	139.4946	95.94062	148.3802	96.15302	133.2695
95.8246	137.9671	95.9322	129.8842	96.04073	132.5908	95.98152	139.4963	95.95728	148.3822	96.16968	133.2665
95.84127	137.9658	95.94887	129.8827	96.0574	132.5895	95.99818	139.4958	95.97395	148.3786	96.18635	133.266
95.85793	137.9632	95.96553	129.8818	96.07407	132.5905	96.01485	139.4948	95.99062	148.3804	96.20302	133.2641
95.8746	137.9612	95.9822	129.8794	96.09073	132.5905	96.03152	139.4946	96.00728	148.3788	96.21968	133.2633
95.89127	137.96	95.99887	129.8777	96.1074	132.591	96.04818	139.4943	96.02395	148.381	96.23635	133.2639
95.90793	137.958	96.01553	129.8782	96.12407	132.5889	96.06485	139.495	96.04062	148.3816	96.25302	133.2623
95.9246	137.9558	96.0322	129.8786	96.14073	132.587	96.08152	139.4963	96.05728	148.3841	96.26968	133.2607
95.94127	137.9519	96.04887	129.8779	96.1574	132.5884	96.09818	139.4929	96.07395	148.3815	96.28635	133.2608
95.95793	137.952	96.06553	129.8763	96.17407	132.5887	96.11485	139.4925	96.09062	148.3816	96.30302	133.2582
95.9746	137.9489	96.0822	129.8793	96.19073	132.5877	96.13152	139.4919	96.10728	148.3845	96.31968	133.2566
95.99127	137.9478	96.09887	129.8773	96.2074	132.5869	96.14818	139.4917	96.12395	148.3822	96.33635	133.256
96.00793	137.9459	96.11553	129.8762	96.22407	132.5875	96.16485	139.494	96.14062	148.382	96.35302	133.2566
96.0246	137.9445	96.1322	129.8758	96.24073	132.5873	96.18152	139.4916	96.15728	148.3835	96.36968	133.2568
96.04127	137.9405	96.14887	129.8766	96.2574	132.5874	96.19818	139.4922	96.17395	148.3805	96.38635	133.2554
96.05793	137.9408	96.16553	129.8752	96.27407	132.5883	96.21485	139.4919	96.19062	148.3837	96.40302	133.2541
96.0746	137.9395	96.1822	129.8731	96.29073	132.5841	96.23152	139.4925	96.20728	148.3817	96.41968	133.2539
96.09127	137.9357	96.19887	129.8737	96.3074	132.5867	96.24818	139.4931	96.22395	148.384	96.43635	133.2528
96.10793	137.9334	96.21553	129.874	96.32407	132.5872	96.26485	139.491	96.24062	148.3838	96.45302	133.2547
96.1246	137.9355	96.2322	129.8708	96.34073	132.5848	96.28152	139.4902	96.25728	148.3827	96.46968	133.2518
96.14127	137.9332	96.24887	129.8703	96.3574	132.5819	96.29818	139.489	96.27395	148.3829	96.48635	133.2504
96.15793	137.9294	96.26553	129.8697	96.37407	132.5843	96.31485	139.4908	96.29062	148.3843	96.50302	133.2509
96.1746	137.9292	96.2822	129.8659	96.39073	132.5841	96.33152	139.492	96.30728	148.386	96.51968	133.2501
96.19127	137.9292	96.29887	129.8662	96.4074	132.5837	96.34818	139.488	96.32395	148.3851	96.53635	133.2477
96.20793	137.927	96.31553	129.8676	96.42407	132.5844	96.36485	139.4892	96.34062	148.3858	96.55302	133.2468
96.2246	137.9235	96.3322	129.8649	96.44073	132.5816	96.38152	139.491	96.35728	148.3861	96.56968	133.2472
96.24127	137.9224	96.34887	129.8646	96.4574	132.5832	96.39818	139.4851	96.37395	148.3842	96.58635	133.2452
96.25793	137.9225	96.36553	129.8652	96.47407	132.5819	96.41485	139.4875	96.39062	148.3843	96.60302	133.2459
96.2746	137.9212	96.3822	129.863	96.49073	132.584	96.43152	139.4871	96.40728	148.3847	96.61968	133.2447
96.29127	137.916	96.39887	129.8612	96.5074	132.5822	96.44818	139.4857	96.42395	148.3861	96.63635	133.2445
96.30793	137.9166	96.41553	129.8617	96.52407	132.5812	96.46485	139.4879	96.44062	148.3857	96.65302	133.2434
96.3246	137.9199	96.4322	129.86	96.54073	132.5827	96.48152	139.4857	96.45728	148.3852	96.66968	133.2428
96.34127	137.9147	96.44887	129.8598	96.5574	132.5805	96.49818	139.4877	96.47395	148.3838	96.68635	133.2404
96.35793	137.9121	96.46553	129.8577	96.57407	132.5821	96.51485	139.4878	96.49062	148.3834	96.70302	133.2407
96.3746	137.9121	96.4822	129.8568	96.59073	132.5818	96.53152	139.4856	96.50728	148.3841	96.71968	133.2424
96.39127	137.9102	96.49887	129.8558	96.6074	132.5799	96.54818	139.4862	96.52395	148.3844	96.73635	133.2384
96.40793	137.9094	96.51553	129.8517	96.62407	132.58	96.56485	139.4868	96.54062	148.3855	96.75302	133.2396
96.4246	137.9072	96.5322	129.8553	96.64073	132.5805	96.58152	139.4859	96.55728	148.3848	96.76968	133.2401
96.44127	137.9037	96.54887	129.8539	96.6574	132.5784	96.59818	139.4862	96.57395	148.3844	96.78635	133.239

96.45793	137.9052	96.56553	129.8513	96.67407	132.5778	96.61485	139.4853	96.59062	148.3826	96.80302	133.2394
96.4746	137.9006	96.5822	129.8503	96.69073	132.5791	96.63152	139.4866	96.60728	148.3814	96.81968	133.2398
96.49127	137.8984	96.59887	129.8558	96.7074	132.5803	96.64818	139.4822	96.62395	148.3821	96.83635	133.2389
96.50793	137.8975	96.61553	129.8506	96.72407	132.5773	96.66485	139.4839	96.64062	148.3836	96.85302	133.2396
96.5246	137.8957	96.6322	129.8472	96.74073	132.5781	96.68152	139.483	96.65728	148.3827	96.86968	133.2378
96.54127	137.8948	96.64887	129.8497	96.7574	132.5773	96.69818	139.4817	96.67395	148.3813	96.88635	133.2386
96.55793	137.8936	96.66553	129.8468	96.77407	132.5765	96.71485	139.483	96.69062	148.383	96.90302	133.234
96.5746	137.8905	96.6822	129.8382	96.79073	132.5747	96.73152	139.4821	96.70728	148.3841	96.91968	133.2394
96.59127	137.8915	96.69887	129.8485	96.8074	132.5742	96.74818	139.4804	96.72395	148.3808	96.93635	133.2373
96.60793	137.8893	96.71553	129.8434	96.82407	132.5749	96.76485	139.4808	96.74062	148.3806	96.95302	133.2332
96.6246	137.8879	96.7322	129.8302	96.84073	132.5735	96.78152	139.4803	96.75728	148.3828	96.96968	133.2345
96.64127	137.884	96.74887	129.8434	96.8574	132.5728	96.79818	139.4822	96.77395	148.3821	96.98635	133.2359
96.65793	137.8837	96.76553	129.8413	96.87407	132.5747	96.81485	139.479	96.79062	148.382	97.00302	133.2346
96.6746	137.8815	96.7822	129.844	96.89073	132.5722	96.83152	139.479	96.80728	148.381	97.01968	133.2317
96.69127	137.8793	96.79887	129.8361	96.9074	132.5738	96.84818	139.4783	96.82395	148.3829	97.03635	133.2356
96.70793	137.876	96.81553	129.8399	96.92407	132.5724	96.86485	139.4794	96.84062	148.3815	97.05302	133.2324
96.7246	137.8773	96.8322	129.8389	96.94073	132.5737	96.88152	139.4774	96.85728	148.3823	97.06968	133.2298
96.74127	137.8754	96.84887	129.8341	96.9574	132.571	96.89818	139.4779	96.87395	148.3816	97.08635	133.2317
96.75793	137.8749	96.86553	129.8374	96.97407	132.5705	96.91485	139.4761	96.89062	148.381	97.10302	133.2302
96.7746	137.8729	96.8822	129.8375	96.99073	132.5705	96.93152	139.4788	96.90728	148.3831	97.11968	133.2303
96.79127	137.8727	96.89887	129.8342	97.0074	132.5713	96.94818	139.4768	96.92395	148.3823	97.13635	133.2308
96.80793	137.8728	96.91553	129.8304	97.02407	132.5689	96.96485	139.4771	96.94062	148.3813	97.15302	133.2303
96.8246	137.8705	96.9322	129.8325	97.04073	132.5704	96.98152	139.4755	96.95728	148.3831	97.16968	133.2285
96.84127	137.8693	96.94887	129.8312	97.0574	132.5684	96.99818	139.4742	96.97395	148.3838	97.18635	133.2248
96.85793	137.8663	96.96553	129.8333	97.07407	132.5693	97.01485	139.4789	96.99062	148.3823	97.20302	133.2286
96.8746	137.8635	96.9822	129.8308	97.09073	132.5684	97.03152	139.4777	97.00728	148.3796	97.21968	133.2271
96.89127	137.8646	96.99887	129.8313	97.1074	132.5674	97.04818	139.4785	97.02395	148.3821	97.23635	133.225
96.90793	137.8613	97.01553	129.8323	97.12407	132.5675	97.06485	139.4772	97.04062	148.3802	97.25302	133.2257
96.9246	137.8614	97.0322	129.8297	97.14073	132.5658	97.08152	139.4755	97.05728	148.3808	97.26968	133.2265
96.94127	137.8575	97.04887	129.8317	97.1574	132.5655	97.09818	139.4768	97.07395	148.3808	97.28635	133.2254
96.95793	137.8583	97.06553	129.8255	97.17407	132.5658	97.11485	139.478	97.09062	148.3787	97.30302	133.2255
96.9746	137.857	97.0822	129.8259	97.19073	132.5661	97.13152	139.4764	97.10728	148.3798	97.31968	133.2249
96.99127	137.8533	97.09887	129.828	97.2074	132.5648	97.14818	139.4743	97.12395	148.38	97.33635	133.2235
97.00793	137.8513	97.11553	129.8275	97.22407	132.5632	97.16485	139.4753	97.14062	148.38	97.35302	133.2229
97.0246	137.8498	97.1322	129.828	97.24073	132.5644	97.18152	139.4746	97.15728	148.3823	97.36968	133.2244
97.04127	137.8489	97.14887	129.8258	97.2574	132.5642	97.19818	139.476	97.17395	148.3818	97.38635	133.2219
97.05793	137.8473	97.16553	129.8248	97.27407	132.5618	97.21485	139.4743	97.19062	148.3813	97.40302	133.2224
97.0746	137.8444	97.1822	129.8242	97.29073	132.5641	97.23152	139.4726	97.20728	148.3787	97.41968	133.2235
97.09127	137.8449	97.19887	129.823	97.3074	132.5616	97.24818	139.4716	97.22395	148.3805	97.43635	133.2222
97.10793	137.8433	97.21553	129.822	97.32407	132.5607	97.26485	139.4762	97.24062	148.3787	97.45302	133.2195
97.1246	137.8416	97.2322	129.825	97.34073	132.5623	97.28152	139.4736	97.25728	148.3785	97.46968	133.2222
97.14127	137.8407	97.24887	129.8221	97.3574	132.5619	97.29818	139.474	97.27395	148.378	97.48635	133.2216
97.15793	137.8394	97.26553	129.8211	97.37407	132.5609	97.31485	139.4724	97.29062	148.3792	97.50302	133.2231
97.1746	137.8372	97.2822	129.8182	97.39073	132.5603	97.33152	139.4708	97.30728	148.3794	97.51968	133.2193
97.19127	137.8359	97.29887	129.821	97.4074	132.5621	97.34818	139.4711	97.32395	148.3816	97.53635	133.219
97.20793	137.8339	97.31553	129.8214	97.42407	132.5608	97.36485	139.4732	97.34062	148.3783	97.55302	133.2188
97.2246	137.8352	97.3322	129.8154	97.44073	132.5616	97.38152	139.4696	97.35728	148.3774	97.56968	133.218
97.24127	137.8307	97.34887	129.8182	97.4574	132.5602	97.39818	139.4712	97.37395	148.3788	97.58635	133.2182
97.25793	137.8289	97.36553	129.815	97.47407	132.5619	97.41485	139.4707	97.39062	148.3787	97.60302	133.2186
97.2746	137.8291	97.3822	129.8158	97.49073	132.5652	97.43152	139.473	97.40728	148.377	97.61968	133.2168
97.29127	137.8259	97.39887	129.8147	97.5074	132.5619	97.44818	139.4698	97.42395	148.3779	97.63635	133.2169
97.30793	137.8251	97.41553	129.8154	97.52407	132.5607	97.46485	139.4695	97.44062	148.3762	97.65302	133.2148
97.3246	137.8228	97.4322	129.8161	97.54073	132.5628	97.48152	139.4707	97.45728	148.3784	97.66968	133.2142
97.34127	137.8209	97.44887	129.8139	97.5574	132.5598	97.49818	139.467	97.47395	148.3793	97.68635	133.2153
97.35793	137.8192	97.46553	129.8147	97.57407	132.5587	97.51485	139.4716	97.49062	148.377	97.70302	133.2191
97.3746	137.8177	97.4822	129.8136	97.59073	132.5633	97.53152	139.4681	97.50728	148.3779	97.71968	133.2128
97.39127	137.8166	97.49887	129.8139	97.6074	132.5604	97.54818	139.4679	97.52395	148.3777	97.73635	133.207
97.40793	137.8146	97.51553	129.8163	97.62407	132.5609	97.56485	139.469	97.54062	148.3817	97.75302	133.2146
97.4246	137.8126	97.5322	129.8162	97.64073	132.5608	97.58152	139.4668	97.55728	148.3791	97.76968	133.2128
97.44127	137.8123	97.54887	129.8111	97.6574	132.5596	97.59818	139.4683	97.57395	148.3789	97.78635	133.213
97.45793	137.81	97.56553	129.8143	97.67407	132.5658	97.61485	139.4664	97.59062	148.3793	97.80302	133.2118
97.4746	137.8078	97.5822	129.8105	97.69073	132.5572	97.63152	139.4661	97.60728	148.3773	97.81968	133.2104
97.49127	137.8078	97.59887	129.8115	97.7074	132.5617	97.64818	139.4659	97.62395	148.3783	97.83635	133.2091
97.50793	137.8074	97.61553	129.8096	97.72407	132.5573	97.66485	139.4648	97.64062	148.3765	97.85302	133.2069
97.5246	137.806	97.6322	129.81	97.74073	132.5576	97.68152	139.4661	97.65728	148.378	97.86968	133.2093
97.54127	137.8019	97.64887	129.8106	97.7574	132.5602	97.69818	139.4654	97.67395	148.3785	97.88635	133.2043
97.55793	137.8	97.66553	129.8082	97.77407	132.5585	97.71485	139.4647	97.69062	148.3764	97.90302	133.2066
97.5746	137.7992	97.6822	129.8091	97.79073	132.5586	97.73152	139.4631	97.70728	148.3786	97.91968	133.2063
97.59127	137.7992	97.69887	129.8072	97.8074	132.5602	97.74818	139.4639	97.72395	148.3774	97.93635	133.2057

97.60793	137.7996	97.71553	129.807	97.82407	132.5568	97.76485	139.4611	97.74062	148.3765	97.95302	133.2059
97.6246	137.795	97.7322	129.807	97.84073	132.5578	97.78152	139.4631	97.75728	148.3769	97.96968	133.2051
97.64127	137.7932	97.74887	129.8045	97.8574	132.555	97.79818	139.462	97.77395	148.3801	97.98635	133.2011
97.65793	137.7892	97.76553	129.8046	97.87407	132.5533	97.81485	139.4594	97.79062	148.3791	98.00302	133.2027
97.6746	137.7905	97.7822	129.8045	97.89073	132.5544	97.83152	139.4601	97.80728	148.3778	98.01968	133.2015
97.69127	137.7886	97.79887	129.8054	97.9074	132.5577	97.84818	139.4602	97.82395	148.3785	98.03635	133.2009
97.70793	137.7882	97.81553	129.8028	97.92407	132.5553	97.86485	139.4594	97.84062	148.3796	98.05302	133.1963
97.7246	137.7845	97.8322	129.8036	97.94073	132.5544	97.88152	139.4586	97.85728	148.3741	98.06968	133.2
97.74127	137.7853	97.84887	129.7974	97.9574	132.5561	97.89818	139.4567	97.87395	148.3763	98.08635	133.197
97.75793	137.783	97.86553	129.7986	97.97407	132.5551	97.91485	139.4559	97.89062	148.3829	98.10302	133.1956
97.7746	137.7805	97.8822	129.7987	97.99073	132.5534	97.93152	139.4576	97.90728	148.3818	98.11968	133.1957
97.79127	137.7819	97.89887	129.7949	98.0074	132.557	97.94818	139.4594	97.92395	148.372	98.13635	133.1944
97.80793	137.7792	97.91553	129.8001	98.02407	132.5558	97.96485	139.4564	97.94062	148.3773	98.15302	133.1935
97.8246	137.7786	97.9322	129.7977	98.04073	132.5539	97.98152	139.4577	97.95728	148.3783	98.16968	133.1943
97.84127	137.7737	97.94887	129.7951	98.0574	132.5532	97.99818	139.4571	97.97395	148.3793	98.18635	133.1951
97.85793	137.7727	97.96553	129.7939	98.07407	132.5499	98.01485	139.4566	97.99062	148.375	98.20302	133.1918
97.8746	137.7727	97.9822	129.7951	98.09073	132.5529	98.03152	139.4576	98.00728	148.3787	98.21968	133.1894
97.89127	137.77	97.99887	129.7929	98.1074	132.5532	98.04818	139.455	98.02395	148.3753	98.23635	133.1932
97.90793	137.7705	98.01553	129.7939	98.12407	132.5503	98.06485	139.4588	98.04062	148.3782	98.25302	133.1894
97.9246	137.7675	98.0322	129.7921	98.14073	132.5525	98.08152	139.4591	98.05728	148.3791	98.26968	133.1897
97.94127	137.7663	98.04887	129.7905	98.1574	132.5503	98.09818	139.4547	98.07395	148.3765	98.28635	133.1901
97.95793	137.7656	98.06553	129.7911	98.17407	132.5531	98.11485	139.4549	98.09062	148.376	98.30302	133.1891
97.9746	137.7636	98.0822	129.7879	98.19073	132.5493	98.13152	139.4546	98.10728	148.3757	98.31968	133.188
97.99127	137.7613	98.09887	129.7907	98.2074	132.5514	98.14818	139.4553	98.12395	148.3755	98.33635	133.188
98.00793	137.7638	98.11553	129.7875	98.22407	132.5493	98.16485	139.4565	98.14062	148.3742	98.35302	133.1857
98.0246	137.7602	98.1322	129.7898	98.24073	132.5506	98.18152	139.452	98.15728	148.3746	98.36968	133.1847
98.04127	137.7584	98.14887	129.7871	98.2574	132.5469	98.19818	139.454	98.17395	148.3752	98.38635	133.1839
98.05793	137.7591	98.16553	129.786	98.27407	132.5474	98.21485	139.4564	98.19062	148.3747	98.40302	133.1849
98.0746	137.7586	98.1822	129.787	98.29073	132.5472	98.23152	139.4555	98.20728	148.3757	98.41968	133.1832
98.09127	137.7548	98.19887	129.7835	98.3074	132.5492	98.24818	139.4561	98.22395	148.3771	98.43635	133.1825
98.10793	137.751	98.21553	129.7848	98.32407	132.5493	98.26485	139.4514	98.24062	148.3774	98.45302	133.1827
98.1246	137.7548	98.2322	129.7836	98.34073	132.5496	98.28152	139.4531	98.25728	148.3765	98.46968	133.1817
98.14127	137.7504	98.24887	129.7837	98.3574	132.548	98.29818	139.453	98.27395	148.3785	98.48635	133.1804
98.15793	137.7516	98.26553	129.7834	98.37407	132.5478	98.31485	139.4519	98.29062	148.3766	98.50302	133.1808
98.1746	137.7502	98.2822	129.7814	98.39073	132.5477	98.33152	139.4521	98.30728	148.3768	98.51968	133.1786
98.19127	137.7485	98.29887	129.782	98.4074	132.548	98.34818	139.4511	98.32395	148.3763	98.53635	133.1783
98.20793	137.7475	98.31553	129.7791	98.42407	132.5473	98.36485	139.4524	98.34062	148.3734	98.55302	133.1786
98.2246	137.7457	98.3322	129.7781	98.44073	132.5474	98.38152	139.4521	98.35728	148.3766	98.56968	133.1791
98.24127	137.7433	98.34887	129.78	98.4574	132.5475	98.39818	139.451	98.37395	148.3774	98.58635	133.1763
98.25793	137.7428	98.36553	129.7791	98.47407	132.5497	98.41485	139.4508	98.39062	148.3749	98.60302	133.1772
98.2746	137.7406	98.3822	129.7778	98.49073	132.5467	98.43152	139.4493	98.40728	148.3762	98.61968	133.1745
98.29127	137.7376	98.39887	129.7762	98.5074	132.5487	98.44818	139.4512	98.42395	148.3744	98.63635	133.177
98.30793	137.7405	98.41553	129.7775	98.52407	132.5488	98.46485	139.4509	98.44062	148.3776	98.65302	133.1759
98.3246	137.7363	98.4322	129.7749	98.54073	132.546	98.48152	139.4497	98.45728	148.3759	98.66968	133.1754
98.34127	137.7398	98.44887	129.773	98.5574	132.544	98.49818	139.451	98.47395	148.377	98.68635	133.1747
98.35793	137.7345	98.46553	129.7714	98.57407	132.5465	98.51485	139.4492	98.49062	148.3771	98.70302	133.1733
98.3746	137.7321	98.4822	129.7724	98.59073	132.5435	98.53152	139.4508	98.50728	148.3796	98.71968	133.1721
98.39127	137.73	98.49887	129.7732	98.6074	132.5446	98.54818	139.4479	98.52395	148.378	98.73635	133.1721
98.40793	137.7317	98.51553	129.7742	98.62407	132.5453	98.56485	139.4462	98.54062	148.3749	98.75302	133.1719
98.4246	137.7316	98.5322	129.7721	98.64073	132.5481	98.58152	139.4478	98.55728	148.375	98.76968	133.1737
98.44127	137.7299	98.54887	129.773	98.6574	132.5446	98.59818	139.4477	98.57395	148.3803	98.78635	133.1684
98.45793	137.7284	98.56553	129.7698	98.67407	132.5433	98.61485	139.4472	98.59062	148.3768	98.80302	133.1675
98.4746	137.7263	98.5822	129.771	98.69073	132.5429	98.63152	139.4471	98.60728	148.3765	98.81968	133.1667
98.49127	137.7266	98.59887	129.7685	98.7074	132.5446	98.64818	139.447	98.62395	148.3769	98.83635	133.1658
98.50793	137.7229	98.61553	129.7694	98.72407	132.5424	98.66485	139.4456	98.64062	148.3759	98.85302	133.1652
98.5246	137.725	98.6322	129.7703	98.74073	132.543	98.68152	139.4457	98.65728	148.3814	98.86968	133.166
98.54127	137.718	98.64887	129.7673	98.7574	132.5431	98.69818	139.4448	98.67395	148.3774	98.88635	133.1654
98.55793	137.7158	98.66553	129.7666	98.77407	132.5438	98.71485	139.4458	98.69062	148.3759	98.90302	133.1632
98.5746	137.717	98.6822	129.767	98.79073	132.5417	98.73152	139.4434	98.70728	148.3775	98.91968	133.1643
98.59127	137.7163	98.69887	129.7685	98.8074	132.5424	98.74818	139.4439	98.72395	148.3778	98.93635	133.1642
98.60793	137.7168	98.71553	129.7679	98.82407	132.5423	98.76485	139.4443	98.74062	148.3731	98.95302	133.1615
98.6246	137.7124	98.7322	129.765	98.84073	132.5387	98.78152	139.4431	98.75728	148.3794	98.96968	133.1641
98.64127	137.7103	98.74887	129.7621	98.8574	132.5419	98.79818	139.4425	98.77395	148.3763	98.98635	133.1616
98.65793	137.7084	98.76553	129.7608	98.87407	132.5387	98.81485	139.4416	98.79062	148.3768	99.00302	133.1616
98.6746	137.7063	98.7822	129.7606	98.89073	132.5413	98.83152	139.4451	98.80728	148.3789	99.01968	133.1586
98.69127	137.7074	98.79887	129.7641	98.9074	132.5417	98.84818	139.4417	98.82395	148.3751	99.03635	133.1612
98.70793	137.7066	98.81553	129.7603	98.92407	132.5432	98.86485	139.4427	98.84062	148.3761	99.05302	133.1592
98.7246	137.7036	98.8322	129.7601	98.94073	132.5419	98.88152	139.4422	98.85728	148.3773	99.06968	133.157
98.74127	137.7019	98.84887	129.7568	98.9574	132.5382	98.89818	139.4407	98.87395	148.3754	99.08635	133.1568

98.75793	137.6997	98.86553	129.7559	98.97407	132.5406	98.91485	139.442	98.89062	148.3762	99.10302	133.1592
98.7746	137.6986	98.8822	129.7547	98.99073	132.5409	98.93152	139.4446	98.90728	148.3761	99.11968	133.1565
98.79127	137.6956	98.89887	129.7516	99.0074	132.54	98.94818	139.4372	98.92395	148.377	99.13635	133.157
98.80793	137.6999	98.91553	129.7549	99.02407	132.5407	98.96485	139.4406	98.94062	148.3764	99.15302	133.1553
98.8246	137.6938	98.9322	129.7544	99.04073	132.5406	98.98152	139.4407	98.95728	148.3762	99.16968	133.1543
98.84127	137.6917	98.94887	129.7526	99.0574	132.5398	98.99818	139.4395	98.97395	148.3737	99.18635	133.153
98.85793	137.691	98.96553	129.7522	99.07407	132.5376	99.01485	139.4391	98.99062	148.376	99.20302	133.1548
98.8746	137.6914	98.9822	129.7505	99.09073	132.5373	99.03152	139.4396	99.00728	148.3769	99.21968	133.1516
98.89127	137.6906	98.99887	129.7493	99.1074	132.5379	99.04818	139.4386	99.02395	148.3767	99.23635	133.1526
98.90793	137.6864	99.01553	129.7512	99.12407	132.5388	99.06485	139.4377	99.04062	148.3756	99.25302	133.1533
98.9246	137.6854	99.0322	129.7523	99.14073	132.5393	99.08152	139.4373	99.05728	148.3749	99.26968	133.1527
98.94127	137.6858	99.04887	129.7522	99.1574	132.5382	99.09818	139.4383	99.07395	148.373	99.28635	133.1481
98.95793	137.6839	99.06553	129.7475	99.17407	132.5391	99.11485	139.436	99.09062	148.3767	99.30302	133.147
98.9746	137.6804	99.0822	129.7483	99.19073	132.5334	99.13152	139.4357	99.10728	148.3776	99.31968	133.1512
98.99127	137.6824	99.09887	129.7493	99.2074	132.5375	99.14818	139.4357	99.12395	148.3747	99.33635	133.1477
99.00793	137.6811	99.11553	129.7484	99.22407	132.537	99.16485	139.4345	99.14062	148.376	99.35302	133.1474
99.0246	137.6786	99.1322	129.7487	99.24073	132.5349	99.18152	139.4332	99.15728	148.374	99.36968	133.1497
99.04127	137.6767	99.14887	129.7462	99.2574	132.5366	99.19818	139.4333	99.17395	148.3733	99.38635	133.144
99.05793	137.6763	99.16553	129.747	99.27407	132.5349	99.21485	139.4322	99.19062	148.3793	99.40302	133.1473
99.0746	137.6737	99.1822	129.7464	99.29073	132.5335	99.23152	139.4329	99.20728	148.3763	99.41968	133.1444
99.09127	137.6732	99.19887	129.7455	99.3074	132.5347	99.24818	139.4319	99.22395	148.3756	99.43635	133.1464
99.10793	137.6742	99.21553	129.7424	99.32407	132.5384	99.26485	139.431	99.24062	148.3756	99.45302	133.143
99.1246	137.6702	99.2322	129.7457	99.34073	132.5332	99.28152	139.4305	99.25728	148.3788	99.46968	133.1457
99.14127	137.6677	99.24887	129.7397	99.3574	132.5363	99.29818	139.4337	99.27395	148.3721	99.48635	133.1429
99.15793	137.6672	99.26553	129.7431	99.37407	132.5329	99.31485	139.4317	99.29062	148.3748	99.50302	133.1421
99.1746	137.6678	99.2822	129.738	99.39073	132.5316	99.33152	139.433	99.30728	148.3792	99.51968	133.1398
99.19127	137.6674	99.29887	129.7392	99.4074	132.5315	99.34818	139.4314	99.32395	148.376	99.53635	133.1393
99.20793	137.6642	99.31553	129.7368	99.42407	132.5351	99.36485	139.4285	99.34062	148.3763	99.55302	133.1403
99.2246	137.6645	99.3322	129.7363	99.44073	132.5328	99.38152	139.4274	99.35728	148.3762	99.56968	133.1394
99.24127	137.6629	99.34887	129.7374	99.4574	132.5305	99.39818	139.4299	99.37395	148.3742	99.58635	133.1384
99.25793	137.6587	99.36553	129.7386	99.47407	132.5315	99.41485	139.4281	99.39062	148.3749	99.60302	133.1392
99.2746	137.6556	99.3822	129.7369	99.49073	132.5315	99.43152	139.4296	99.40728	148.3741	99.61968	133.1374
99.29127	137.657	99.39887	129.7391	99.5074	132.528	99.44818	139.4285	99.42395	148.3727	99.63635	133.137
99.30793	137.6582	99.41553	129.7364	99.52407	132.5315	99.46485	139.4263	99.44062	148.3709	99.65302	133.1345
99.3246	137.6505	99.4322	129.7382	99.54073	132.5309	99.48152	139.4289	99.45728	148.3738	99.66968	133.1379
99.34127	137.6498	99.44887	129.7359	99.5574	132.5302	99.49818	139.4292	99.47395	148.3718	99.68635	133.1349
99.35793	137.6499	99.46553	129.738	99.57407	132.5297	99.51485	139.4274	99.49062	148.3714	99.70302	133.1339
99.3746	137.649	99.4822	129.7347	99.59073	132.5299	99.53152	139.4278	99.50728	148.3739	99.71968	133.1329
99.39127	137.6472	99.49887	129.7349	99.6074	132.5285	99.54818	139.4263	99.52395	148.3729	99.73635	133.1325
99.40793	137.6448	99.51553	129.7324	99.62407	132.5257	99.56485	139.4242	99.54062	148.3732	99.75302	133.1319
99.4246	137.6458	99.5322	129.7344	99.64073	132.5269	99.58152	139.4257	99.55728	148.3734	99.76968	133.1324
99.44127	137.648	99.54887	129.7346	99.6574	132.5277	99.59818	139.4274	99.57395	148.3733	99.78635	133.1304
99.45793	137.6439	99.56553	129.7333	99.67407	132.5293	99.61485	139.4247	99.59062	148.3714	99.80302	133.1302
99.4746	137.6459	99.5822	129.7331	99.69073	132.5291	99.63152	139.4254	99.60728	148.3714	99.81968	133.1285
99.49127	137.6391	99.59887	129.7331	99.7074	132.528	99.64818	139.4248	99.62395	148.3737	99.83635	133.13
99.50793	137.6387	99.61553	129.7307	99.72407	132.5264	99.66485	139.4251	99.64062	148.3743	99.85302	133.1289
99.5246	137.6353	99.6322	129.7295	99.74073	132.5263	99.68152	139.4241	99.65728	148.3725	99.86968	133.1279
99.54127	137.6327	99.64887	129.7297	99.7574	132.5289	99.69818	139.4238	99.67395	148.3738	99.88635	133.1272
99.55793	137.6347	99.66553	129.7282	99.77407	132.528	99.71485	139.424	99.69062	148.3746	99.90302	133.1275
99.5746	137.6327	99.6822	129.7251	99.79073	132.5278	99.73152	139.4238	99.70728	148.373	99.91968	133.126
99.59127	137.6319	99.69887	129.728	99.8074	132.5287	99.74818	139.4225	99.72395	148.3754	99.93635	133.1265
99.60793	137.6306	99.71553	129.7283	99.82407	132.5239	99.76485	139.4221	99.74062	148.3753	99.95302	133.1234
99.6246	137.6269	99.7322	129.7283	99.84073	132.5259	99.78152	139.4229	99.75728	148.3728	99.96968	133.1236
99.64127	137.6264	99.74887	129.7277	99.8574	132.5251	99.79818	139.421	99.77395	148.3752	99.98635	133.1239
99.65793	137.6218	99.76553	129.7276	99.87407	132.5257	99.81485	139.4204	99.79062	148.3711	100.003	133.1238
99.6746	137.6222	99.7822	129.7254	99.89073	132.5242	99.83152	139.4224	99.80728	148.3792	100.0197	133.1236
99.69127	137.6204	99.79887	129.7277	99.9074	132.527	99.84818	139.4215	99.82395	148.3728	100.0364	133.1224
99.70793	137.6193	99.81553	129.7236	99.92407	132.5249	99.86485	139.4238	99.84062	148.376	100.053	133.1232
99.7246	137.6171	99.8322	129.7264	99.94073	132.5246	99.88152	139.4226	99.85728	148.3711	100.0697	133.1212
99.74127	137.6152	99.84887	129.7254	99.9574	132.5215	99.89818	139.4221	99.87395	148.3737	100.0864	133.1196
99.75793	137.6125	99.86553	129.7234	99.97407	132.5233	99.91485	139.4198	99.89062	148.3722	100.103	133.119
99.7746	137.6117	99.8822	129.724	99.99073	132.5233	99.93152	139.4214	99.90728	148.3725	100.1197	133.1175
99.79127	137.6104	99.89887	129.7226	100.0074	132.5236	99.94818	139.422	99.92395	148.3738	100.1364	133.1162
99.80793	137.6087	99.91553	129.7198	100.0241	132.5229	99.96485	139.4209	99.94062	148.3687	100.153	133.1164
99.8246	137.6051	99.9322	129.7218	100.0407	132.5237	99.98152	139.4216	99.95728	148.3758	100.1697	133.1174
99.84127	137.6055	99.94887	129.7227	100.0574	132.524	99.99818	139.4189	99.97395	148.3701	100.1864	133.1158
99.85793	137.606	99.96553	129.7234	100.0741	132.5244	100.0149	139.4217	99.99062	148.3739	100.203	133.1153
99.8746	137.6036	99.9822	129.7235	100.0907	132.5248	100.0315	139.4219	100.0073	148.3746	100.2197	133.1133
99.89127	137.6011	99.99887	129.7225	100.1074	132.5257	100.0482	139.4197	100.024	148.3719	100.2364	133.1138

99.90793	137.6014	100.0155	129.7213	100.1241	132.5224	100.0649	139.4152	100.0406	148.3724	100.253	133.1123
99.9246	137.5974	100.0322	129.722	100.1407	132.5253	100.0815	139.4157	100.0573	148.3707	100.2697	133.1129
99.94127	137.5994	100.0489	129.7213	100.1574	132.5221	100.0982	139.4203	100.074	148.3722	100.2864	133.1123
99.95793	137.5948	100.0655	129.7224	100.1741	132.524	100.1149	139.4162	100.0906	148.374	100.303	133.1105
99.9746	137.5941	100.0822	129.7224	100.1907	132.5234	100.1315	139.4184	100.1073	148.3749	100.3197	133.1107
99.99127	137.5913	100.0989	129.7213	100.2074	132.5187	100.1482	139.4144	100.124	148.3731	100.3364	133.1078
100.0079	137.5923	100.1155	129.72	100.2241	132.5212	100.1649	139.4185	100.1406	148.3773	100.353	133.1068
100.0246	137.5927	100.1322	129.7209	100.2407	132.5214	100.1815	139.4188	100.1573	148.3738	100.3697	133.1065
100.0413	137.5888	100.1489	129.7193	100.2574	132.5203	100.1982	139.417	100.174	148.3733	100.3864	133.1085
100.0579	137.5861	100.1655	129.7226	100.2741	132.5176	100.2149	139.4165	100.1906	148.3735	100.403	133.1057
100.0746	137.5851	100.1822	129.7193	100.2907	132.5189	100.2315	139.4154	100.2073	148.3749	100.4197	133.1044
100.0913	137.5851	100.1989	129.7178	100.3074	132.5143	100.2482	139.416	100.224	148.3732	100.4364	133.1041
100.1079	137.5834	100.2155	129.7173	100.3241	132.5187	100.2649	139.4138	100.2406	148.3716	100.453	133.1046
100.1246	137.5845	100.2322	129.7164	100.3407	132.5195	100.2815	139.4133	100.2573	148.3721	100.4697	133.1037
100.1413	137.5832	100.2489	129.7161	100.3574	132.5191	100.2982	139.4156	100.274	148.3724	100.4864	133.1016
100.1579	137.5785	100.2655	129.7154	100.3741	132.5207	100.3149	139.4126	100.2906	148.3717	100.503	133.1032
100.1746	137.5782	100.2822	129.7136	100.3907	132.5165	100.3315	139.4125	100.3073	148.3711	100.5197	133.103
100.1913	137.5758	100.2989	129.7119	100.4074	132.5187	100.3482	139.4123	100.324	148.3735	100.5364	133.0992
100.2079	137.5735	100.3155	129.713	100.4241	132.516	100.3649	139.4134	100.3406	148.3717	100.553	133.1016
100.2246	137.5732	100.3322	129.7156	100.4407	132.5177	100.3815	139.4114	100.3573	148.3704	100.5697	133.1022
100.2413	137.5734	100.3489	129.7113	100.4574	132.5201	100.3982	139.4117	100.374	148.3701	100.5864	133.0972
100.2579	137.5729	100.3655	129.7185	100.4741	132.5176	100.4149	139.4101	100.3906	148.3708	100.603	133.0977
100.2746	137.5715	100.3822	129.7103	100.4907	132.5159	100.4315	139.4101	100.4073	148.3676	100.6197	133.1018
100.2913	137.5716	100.3989	129.7109	100.5074	132.5179	100.4482	139.4109	100.424	148.3703	100.6364	133.0989
100.3079	137.5652	100.4155	129.7147	100.5241	132.5146	100.4649	139.4098	100.4406	148.3715	100.653	133.098
100.3246	137.5638	100.4322	129.7112	100.5407	132.5179	100.4815	139.4116	100.4573	148.3713	100.6697	133.0997
100.3413	137.5639	100.4489	129.7123	100.5574	132.5185	100.4982	139.4112	100.474	148.3692	100.6864	133.0984
100.3579	137.5614	100.4655	129.7149	100.5741	132.5161	100.5149	139.4099	100.4906	148.3728	100.703	133.0962
100.3746	137.5641	100.4822	129.7094	100.5907	132.5167	100.5315	139.4124	100.5073	148.3714	100.7197	133.0946
100.3913	137.5583	100.4989	129.708	100.6074	132.5153	100.5482	139.4125	100.524	148.3732	100.7364	133.1
100.4079	137.5609	100.5155	129.7094	100.6241	132.5152	100.5649	139.4085	100.5406	148.3717	100.753	133.0952
100.4246	137.5573	100.5322	129.7079	100.6407	132.5149	100.5815	139.4084	100.5573	148.3718	100.7697	133.0944
100.4413	137.5587	100.5489	129.7107	100.6574	132.5156	100.5982	139.4093	100.574	148.3715	100.7864	133.0944
100.4579	137.5568	100.5655	129.7088	100.6741	132.516	100.6149	139.4078	100.5906	148.3725	100.803	133.0953
100.4746	137.5555	100.5822	129.7105	100.6907	132.5154	100.6315	139.4126	100.6073	148.3709	100.8197	133.0935
100.4913	137.554	100.5989	129.7101	100.7074	132.5145	100.6482	139.4093	100.624	148.3712	100.8364	133.0945
100.5079	137.5517	100.6155	129.7097	100.7241	132.517	100.6649	139.4094	100.6406	148.3702	100.853	133.0909
100.5246	137.5515	100.6322	129.7081	100.7407	132.5149	100.6815	139.4082	100.6573	148.3703	100.8697	133.0918
100.5413	137.5493	100.6489	129.706	100.7574	132.5153	100.6982	139.4049	100.674	148.3694	100.8864	133.0897
100.5579	137.5488	100.6655	129.7092	100.7741	132.5122	100.7149	139.4053	100.6906	148.3708	100.903	133.0879
100.5746	137.5473	100.6822	129.7082	100.7907	132.5157	100.7315	139.4066	100.7073	148.3701	100.9197	133.0877
100.5913	137.5469	100.6989	129.7067	100.8074	132.5122	100.7482	139.4035	100.724	148.3687	100.9364	133.0879
100.6079	137.5442	100.7155	129.7081	100.8241	132.5091	100.7649	139.4056	100.7406	148.3702	100.953	133.0861
100.6246	137.5449	100.7322	129.7073	100.8407	132.5117	100.7815	139.4029	100.7573	148.3701	100.9697	133.0864
100.6413	137.5433	100.7489	129.7092	100.8574	132.5121	100.7982	139.4051	100.774	148.3703	100.9864	133.0853
100.6579	137.5405	100.7655	129.7039	100.8741	132.5153	100.8149	139.4031	100.7906	148.3724	101.003	133.0857
100.6746	137.5401	100.7822	129.7057	100.8907	132.5133	100.8315	139.4017	100.8073	148.3698	101.0197	133.0843
100.6913	137.5387	100.7989	129.7035	100.9074	132.5131	100.8482	139.4023	100.824	148.3689	101.0364	133.0851
100.7079	137.5376	100.8155	129.7026	100.9241	132.5126	100.8649	139.4016	100.8406	148.3715	101.053	133.0816
100.7246	137.5357	100.8322	129.7013	100.9407	132.5138	100.8815	139.4011	100.8573	148.3685	101.0697	133.083
100.7413	137.5365	100.8489	129.7036	100.9574	132.5138	100.8982	139.4022	100.874	148.3684	101.0864	133.0815
100.7579	137.5335	100.8655	129.7008	100.9741	132.5126	100.9149	139.4022	100.8906	148.3677	101.103	133.0829
100.7746	137.5338	100.8822	129.7023	100.9907	132.5153	100.9315	139.4033	100.9073	148.3693	101.1197	133.0797
100.7913	137.5301	100.8989	129.6991	101.0074	132.5135	100.9482	139.4022	100.924	148.3684	101.1364	133.0796
100.8079	137.5297	100.9155	129.6987	101.0241	132.5144	100.9649	139.4037	100.9406	148.3693	101.153	133.0819
100.8246	137.5281	100.9322	129.7004	101.0407	132.5117	100.9815	139.4001	100.9573	148.3672	101.1697	133.0792
100.8413	137.5278	100.9489	129.699	101.0574	132.5123	100.9982	139.4031	100.974	148.368	101.1864	133.0781
100.8579	137.5249	100.9655	129.6977	101.0741	132.5091	101.0149	139.4031	100.9906	148.3682	101.203	133.079
100.8746	137.5244	100.9822	129.696	101.0907	132.5116	101.0315	139.4005	101.0073	148.3678	101.2197	133.0776
100.8913	137.5232	100.9989	129.6954	101.1074	132.512	101.0482	139.4002	101.024	148.3671	101.2364	133.0769
100.9079	137.5213	101.0155	129.694	101.1241	132.5079	101.0649	139.3994	101.0406	148.3642	101.253	133.077
100.9246	137.519	101.0322	129.6938	101.1407	132.5102	101.0815	139.3998	101.0573	148.3704	101.2697	133.0764
100.9413	137.5194	101.0489	129.6945	101.1574	132.5083	101.0982	139.3992	101.074	148.3684	101.2864	133.0743
100.9579	137.5191	101.0655	129.6947	101.1741	132.5084	101.1149	139.3961	101.0906	148.3683	101.303	133.074
100.9746	137.5171	101.0822	129.6939	101.1907	132.5086	101.1315	139.3979	101.1073	148.3667	101.3197	133.076
100.9913	137.5146	101.0989	129.6934	101.2074	132.5088	101.1482	139.3978	101.124	148.3702	101.3364	133.0732
101.0079	137.5108	101.1155	129.6905	101.2241	132.5098	101.1649	139.3984	101.1406	148.3698	101.353	133.0706
101.0246	137.5124	101.1322	129.6912	101.2407	132.512	101.1815	139.3941	101.1573	148.37	101.3697	133.0697
101.0413	137.514	101.1489	129.694	101.2574	132.5103	101.1982	139.3958	101.174	148.3698	101.3864	133.0719

101.0579	137.5075	101.1655	129.6927	101.2741	132.507	101.2149	139.3938	101.1906	148.3711	101.403	133.0675
101.0746	137.5089	101.1822	129.6875	101.2907	132.5102	101.2315	139.3954	101.2073	148.3701	101.4197	133.0679
101.0913	137.5105	101.1989	129.6912	101.3074	132.5095	101.2482	139.3945	101.224	148.3697	101.4364	133.0671
101.1079	137.506	101.2155	129.6896	101.3241	132.5077	101.2649	139.3946	101.2406	148.3702	101.453	133.0656
101.1246	137.5052	101.2322	129.6881	101.3407	132.5086	101.2815	139.3936	101.2573	148.377	101.4697	133.0641
101.1413	137.5028	101.2489	129.6882	101.3574	132.5084	101.2982	139.3935	101.274	148.3706	101.4864	133.0666
101.1579	137.5026	101.2655	129.6915	101.3741	132.5101	101.3149	139.3942	101.2906	148.3686	101.503	133.0634
101.1746	137.5005	101.2822	129.688	101.3907	132.5077	101.3315	139.3919	101.3073	148.3699	101.5197	133.0653
101.1913	137.4982	101.2989	129.6852	101.4074	132.5068	101.3482	139.3941	101.324	148.369	101.5364	133.0616
101.2079	137.4973	101.3155	129.6868	101.4241	132.5057	101.3649	139.3922	101.3406	148.3688	101.553	133.061
101.2246	137.4974	101.3322	129.6861	101.4407	132.508	101.3815	139.3912	101.3573	148.3681	101.5697	133.064
101.2413	137.4998	101.3489	129.6847	101.4574	132.5056	101.3982	139.3923	101.374	148.371	101.5864	133.0597
101.2579	137.4964	101.3655	129.6841	101.4741	132.5066	101.4149	139.3896	101.3906	148.3687	101.603	133.056
101.2746	137.4964	101.3822	129.6853	101.4907	132.5045	101.4315	139.3892	101.4073	148.3679	101.6197	133.0583
101.2913	137.4929	101.3989	129.6837	101.5074	132.5072	101.4482	139.3899	101.424	148.3666	101.6364	133.0562
101.3079	137.4927	101.4155	129.6862	101.5241	132.5028	101.4649	139.3879	101.4406	148.3664	101.653	133.0542
101.3246	137.4913	101.4322	129.684	101.5407	132.5047	101.4815	139.3889	101.4573	148.3689	101.6697	133.0569
101.3413	137.4897	101.4489	129.6854	101.5574	132.5034	101.4982	139.3907	101.474	148.3685	101.6864	133.0529
101.3579	137.4888	101.4655	129.6829	101.5741	132.5058	101.5149	139.3898	101.4906	148.3665	101.703	133.0549
101.3746	137.4867	101.4822	129.6804	101.5907	132.5048	101.5315	139.3907	101.5073	148.3661	101.7197	133.0519
101.3913	137.4847	101.4989	129.6805	101.6074	132.5022	101.5482	139.3889	101.524	148.3659	101.7364	133.0516
101.4079	137.4858	101.5155	129.6814	101.6241	132.5016	101.5649	139.3888	101.5406	148.3663	101.753	133.0527
101.4246	137.4796	101.5322	129.6787	101.6407	132.5034	101.5815	139.3877	101.5573	148.3647	101.7697	133.0516
101.4413	137.4788	101.5489	129.678	101.6574	132.499	101.5982	139.3895	101.574	148.3672	101.7864	133.0477
101.4579	137.4801	101.5655	129.6783	101.6741	132.5034	101.6149	139.3895	101.5906	148.3654	101.803	133.05
101.4746	137.4785	101.5822	129.6795	101.6907	132.5023	101.6315	139.3885	101.6073	148.3667	101.8197	133.0491
101.4913	137.4784	101.5989	129.6771	101.7074	132.501	101.6482	139.3867	101.624	148.3675	101.8364	133.0478
101.5079	137.4775	101.6155	129.6771	101.7241	132.5012	101.6649	139.3892	101.6406	148.367	101.853	133.0452
101.5246	137.472	101.6322	129.6772	101.7407	132.4992	101.6815	139.3891	101.6573	148.3681	101.8697	133.0451
101.5413	137.4703	101.6489	129.6744	101.7574	132.4997	101.6982	139.3882	101.674	148.3651	101.8864	133.0467
101.5579	137.4716	101.6655	129.6721	101.7741	132.5017	101.7149	139.3856	101.6906	148.3645	101.903	133.0455
101.5746	137.4728	101.6822	129.6741	101.7907	132.5012	101.7315	139.3845	101.7073	148.3656	101.9197	133.0451
101.5913	137.4672	101.6989	129.6722	101.8074	132.4987	101.7482	139.3862	101.724	148.3634	101.9364	133.044
101.6079	137.4705	101.7155	129.6731	101.8241	132.4978	101.7649	139.386	101.7406	148.3652	101.953	133.0441
101.6246	137.467	101.7322	129.6753	101.8407	132.501	101.7815	139.3833	101.7573	148.3654	101.9697	133.0423
101.6413	137.4684	101.7489	129.6697	101.8574	132.4982	101.7982	139.3855	101.774	148.3692	101.9864	133.0436
101.6579	137.4664	101.7655	129.6713	101.8741	132.4989	101.8149	139.3811	101.7906	148.3654	102.003	133.0394
101.6746	137.461	101.7822	129.6703	101.8907	132.5004	101.8315	139.3825	101.8073	148.3618	102.0197	133.0425
101.6913	137.4647	101.7989	129.6692	101.9074	132.5007	101.8482	139.384	101.824	148.3675	102.0364	133.0413
101.7079	137.4601	101.8155	129.6694	101.9241	132.4984	101.8649	139.3816	101.8406	148.3647	102.053	133.0398
101.7246	137.4564	101.8322	129.668	101.9407	132.4981	101.8815	139.3803	101.8573	148.365	102.0697	133.0388
101.7413	137.4586	101.8489	129.6705	101.9574	132.4976	101.8982	139.3791	101.874	148.366	102.0864	133.0374
101.7579	137.4559	101.8655	129.6687	101.9741	132.4974	101.9149	139.3803	101.8906	148.3643	102.103	133.0378
101.7746	137.4561	101.8822	129.6701	101.9907	132.4994	101.9315	139.3821	101.9073	148.3669	102.1197	133.0387
101.7913	137.4534	101.8989	129.6712	102.0074	132.4964	101.9482	139.3806	101.924	148.3654	102.1364	133.0362
101.8079	137.4525	101.9155	129.6717	102.0241	132.4971	101.9649	139.3804	101.9406	148.3659	102.153	133.0344
101.8246	137.4488	101.9322	129.6678	102.0407	132.499	101.9815	139.3808	101.9573	148.362	102.1697	133.0355
101.8413	137.4502	101.9489	129.6659	102.0574	132.4999	101.9982	139.3784	101.974	148.3608	102.1864	133.0333
101.8579	137.444	101.9655	129.6674	102.0741	132.4972	102.0149	139.3802	101.9906	148.3628	102.203	133.0344
101.8746	137.4489	101.9822	129.6645	102.0907	132.4943	102.0315	139.3838	102.0073	148.3592	102.2197	133.0317
101.8913	137.4443	101.9989	129.6654	102.1074	132.4967	102.0482	139.3798	102.024	148.364	102.2364	133.0333
101.9079	137.4428	102.0155	129.6658	102.1241	132.4967	102.0649	139.3799	102.0406	148.3634	102.253	133.0318
101.9246	137.4413	102.0322	129.6635	102.1407	132.4976	102.0815	139.3797	102.0573	148.3603	102.2697	133.0296
101.9413	137.4421	102.0489	129.6645	102.1574	132.4969	102.0982	139.3816	102.074	148.3631	102.2864	133.0298
101.9579	137.4373	102.0655	129.6661	102.1741	132.4977	102.1149	139.3822	102.0906	148.3622	102.303	133.0299
101.9746	137.4369	102.0822	129.6646	102.1907	132.4963	102.1315	139.3796	102.1073	148.3615	102.3197	133.0289
101.9913	137.4378	102.0989	129.6629	102.2074	132.498	102.1482	139.3791	102.124	148.363	102.3364	133.0287
102.0079	137.4339	102.1155	129.6632	102.2241	132.4985	102.1649	139.3808	102.1406	148.3613	102.353	133.0275
102.0246	137.4354	102.1322	129.661	102.2407	132.4976	102.1815	139.3813	102.1573	148.3637	102.3697	133.0246
102.0413	137.4342	102.1489	129.6618	102.2574	132.4991	102.1982	139.38	102.174	148.3611	102.3864	133.0247
102.0579	137.4299	102.1655	129.6618	102.2741	132.4987	102.2149	139.3779	102.1906	148.3602	102.403	133.0285
102.0746	137.4312	102.1822	129.6614	102.2907	132.4991	102.2315	139.3824	102.2073	148.362	102.4197	133.0247
102.0913	137.4308	102.1989	129.6621	102.3074	132.5007	102.2482	139.3791	102.224	148.3605	102.4364	133.0211
102.1079	137.4289	102.2155	129.6614	102.3241	132.4997	102.2649	139.382	102.2406	148.3622	102.453	133.0222
102.1246	137.4291	102.2322	129.6605	102.3407	132.4994	102.2815	139.381	102.2573	148.359	102.4697	133.0225
102.1413	137.4259	102.2489	129.6596	102.3574	132.5001	102.2982	139.3803	102.274	148.3626	102.4864	133.0216
102.1579	137.4237	102.2655	129.6595	102.3741	132.4981	102.3149	139.3782	102.2906	148.3611	102.503	133.0209
102.1746	137.4243	102.2822	129.6587	102.3907	132.4988	102.3315	139.3773	102.3073	148.3614	102.5197	133.0182
102.1913	137.4185	102.2989	129.6596	102.4074	132.4996	102.3482	139.3787	102.324	148.3607	102.5364	133.0163

102.2079	137.4226	102.3155	129.6584	102.4241	132.4985	102.3649	139.3767	102.3406	148.3602	102.553	133.0166
102.2246	137.4163	102.3322	129.6531	102.4407	132.4988	102.3815	139.3775	102.3573	148.3593	102.5697	133.0154
102.2413	137.4156	102.3489	129.6524	102.4574	132.4988	102.3982	139.3757	102.374	148.3632	102.5864	133.0165
102.2579	137.4199	102.3655	129.6537	102.4741	132.4994	102.4149	139.3739	102.3906	148.3586	102.603	133.0118
102.2746	137.4177	102.3822	129.6555	102.4907	132.4996	102.4315	139.3763	102.4073	148.3614	102.6197	133.0085
102.2913	137.4112	102.3989	129.6532	102.5074	132.4999	102.4482	139.3747	102.424	148.3577	102.6364	133.0104
102.3079	137.4142	102.4155	129.6528	102.5241	132.4988	102.4649	139.3749	102.4406	148.3599	102.653	133.0123
102.3246	137.4121	102.4322	129.6538	102.5407	132.499	102.4815	139.3739	102.4573	148.3601	102.6697	133.009
102.3413	137.4129	102.4489	129.6526	102.5574	132.5009	102.4982	139.3723	102.474	148.3617	102.6864	133.0084
102.3579	137.4148	102.4655	129.6521	102.5741	132.4999	102.5149	139.3741	102.4906	148.3603	102.703	133.0099
102.3746	137.4121	102.4822	129.6509	102.5907	132.5015	102.5315	139.3733	102.5073	148.3609	102.7197	133.0069
102.3913	137.409	102.4989	129.6473	102.6074	132.4991	102.5482	139.373	102.524	148.3632	102.7364	133.0058
102.4079	137.4064	102.5155	129.6471	102.6241	132.501	102.5649	139.374	102.5406	148.365	102.753	133.0072
102.4246	137.4039	102.5322	129.6462	102.6407	132.5029	102.5815	139.3714	102.5573	148.3616	102.7697	133.0031
102.4413	137.4026	102.5489	129.6459	102.6574	132.5015	102.5982	139.3745	102.574	148.3634	102.7864	133.0081
102.4579	137.4027	102.5655	129.6483	102.6741	132.5012	102.6149	139.3739	102.5906	148.3634	102.803	133.0023
102.4746	137.3984	102.5822	129.6463	102.6907	132.5003	102.6315	139.3729	102.6073	148.3631	102.8197	133.0021
102.4913	137.4001	102.5989	129.6453	102.7074	132.5021	102.6482	139.3726	102.624	148.3612	102.8364	133.0037
102.5079	137.4	102.6155	129.6439	102.7241	132.5013	102.6649	139.3726	102.6406	148.3603	102.853	133.0037
102.5246	137.3964	102.6322	129.6428	102.7407	132.5034	102.6815	139.3732	102.6573	148.3586	102.8697	133.0025
102.5413	137.3986	102.6489	129.642	102.7574	132.5028	102.6982	139.3733	102.674	148.3602	102.8864	133.0026
102.5579	137.3937	102.6655	129.6417	102.7741	132.5003	102.7149	139.3732	102.6906	148.3602	102.903	133.0028
102.5746	137.3926	102.6822	129.6411	102.7907	132.5005	102.7315	139.3741	102.7073	148.3605	102.9197	133.001
102.5913	137.3931	102.6989	129.6388	102.8074	132.504	102.7482	139.3712	102.724	148.3582	102.9364	133.0001
102.6079	137.3921	102.7155	129.6377	102.8241	132.5065	102.7649	139.377	102.7406	148.3578	102.953	133.0008
102.6246	137.3916	102.7322	129.6386	102.8407	132.4969	102.7815	139.374	102.7573	148.3575	102.9697	133.0014
102.6413	137.3906	102.7489	129.6375	102.8574	132.5012	102.7982	139.3734	102.774	148.3591	102.9864	132.9995
102.6579	137.3871	102.7655	129.6377	102.8741	132.4998	102.8149	139.3736	102.7906	148.3578	103.003	132.9988
102.6746	137.3894	102.7822	129.6386	102.8907	132.5042	102.8315	139.3732	102.8073	148.3607	103.0197	132.9968
102.6913	137.387	102.7989	129.6377	102.9074	132.5002	102.8482	139.3753	102.824	148.3569	103.0364	132.9976
102.7079	137.3846	102.8155	129.6388	102.9241	132.503	102.8649	139.3725	102.8406	148.3579	103.053	132.9977
102.7246	137.3816	102.8322	129.6384	102.9407	132.4993	102.8815	139.3742	102.8573	148.3582	103.0697	132.9949
102.7413	137.3817	102.8489	129.6377	102.9574	132.5008	102.8982	139.3738	102.874	148.3603	103.0864	132.996
102.7579	137.3796	102.8655	129.637	102.9741	132.5017	102.9149	139.3738	102.8906	148.3582	103.103	132.997
102.7746	137.3796	102.8822	129.6378	102.9907	132.499	102.9315	139.3722	102.9073	148.3596	103.1197	132.9936
102.7913	137.3782	102.8989	129.6357	103.0074	132.4991	102.9482	139.3726	102.924	148.3576	103.1364	132.9915
102.8079	137.3744	102.9155	129.6331	103.0241	132.4997	102.9649	139.3728	102.9406	148.3605	103.153	132.9951
102.8246	137.3745	102.9322	129.6341	103.0407	132.4997	102.9815	139.3746	102.9573	148.3591	103.1697	132.9905
102.8413	137.3748	102.9489	129.6353	103.0574	132.4965	102.9982	139.3724	102.974	148.3572	103.1864	132.9898
102.8579	137.3729	102.9655	129.6331	103.0741	132.4982	103.0149	139.3716	102.9906	148.3572	103.203	132.9898
102.8746	137.3768	102.9822	129.6364	103.0907	132.4977	103.0315	139.3738	103.0073	148.3599	103.2197	132.9882
102.8913	137.3679	102.9989	129.6348	103.1074	132.4989	103.0482	139.3718	103.024	148.3604	103.2364	132.9887
102.9079	137.3714	103.0155	129.6337	103.1241	132.4988	103.0649	139.372	103.0406	148.3607	103.253	132.9873
102.9246	137.3677	103.0322	129.6301	103.1407	132.4983	103.0815	139.369	103.0573	148.3625	103.2697	132.9863
102.9413	137.366	103.0489	129.6324	103.1574	132.4985	103.0982	139.3708	103.074	148.3589	103.2864	132.9863
102.9579	137.3654	103.0655	129.6297	103.1741	132.4978	103.1149	139.3711	103.0906	148.3575	103.303	132.9877
102.9746	137.366	103.0822	129.6295	103.1907	132.4981	103.1315	139.3712	103.1073	148.3613	103.3197	132.985
102.9913	137.3617	103.0989	129.629	103.2074	132.4992	103.1482	139.3686	103.124	148.3581	103.3364	132.9846
103.0079	137.3604	103.1155	129.6288	103.2241	132.4982	103.1649	139.371	103.1406	148.3597	103.353	132.9893
103.0246	137.3647	103.1322	129.6246	103.2407	132.4975	103.1815	139.3668	103.1573	148.3574	103.3697	132.9838
103.0413	137.3594	103.1489	129.6251	103.2574	132.4996	103.1982	139.3708	103.174	148.3579	103.3864	132.9803
103.0579	137.3562	103.1655	129.6252	103.2741	132.5007	103.2149	139.3696	103.1906	148.3592	103.403	132.9845
103.0746	137.3589	103.1822	129.6249	103.2907	132.4963	103.2315	139.3668	103.2073	148.3592	103.4197	132.9837
103.0913	137.3546	103.1989	129.6254	103.3074	132.4985	103.2482	139.3669	103.224	148.3566	103.4364	132.9789
103.1079	137.3539	103.2155	129.6261	103.3241	132.4967	103.2649	139.3692	103.2406	148.3598	103.453	132.9797
103.1246	137.3522	103.2322	129.6227	103.3407	132.4987	103.2815	139.3679	103.2573	148.3583	103.4697	132.9802
103.1413	137.3517	103.2489	129.619	103.3574	132.4965	103.2982	139.3668	103.274	148.3591	103.4864	132.9772
103.1579	137.3516	103.2655	129.6222	103.3741	132.4981	103.3149	139.3638	103.2906	148.3566	103.503	132.9774
103.1746	137.3479	103.2822	129.6199	103.3907	132.496	103.3315	139.3664	103.3073	148.3596	103.5197	132.9777
103.1913	137.3483	103.2989	129.6212	103.4074	132.4963	103.3482	139.3665	103.324	148.3575	103.5364	132.9744
103.2079	137.3476	103.3155	129.6187	103.4241	132.4941	103.3649	139.3636	103.3406	148.3616	103.553	132.9739
103.2246	137.3435	103.3322	129.6192	103.4407	132.4955	103.3815	139.3665	103.3573	148.3587	103.5697	132.9767
103.2413	137.3453	103.3489	129.6161	103.4574	132.4945	103.3982	139.3636	103.374	148.3608	103.5864	132.9737
103.2579	137.3429	103.3655	129.6156	103.4741	132.4937	103.4149	139.3665	103.3906	148.3591	103.603	132.9729
103.2746	137.3432	103.3822	129.6197	103.4907	132.4919	103.4315	139.3639	103.4073	148.36	103.6197	132.972
103.2913	137.3423	103.3989	129.6163	103.5074	132.4938	103.4482	139.3649	103.424	148.3572	103.6364	132.9723
103.3079	137.3398	103.4155	129.6163	103.5241	132.492	103.4649	139.3655	103.4406	148.3605	103.653	132.9715
103.3246	137.3407	103.4322	129.614	103.5407	132.4897	103.4815	139.3646	103.4573	148.3603	103.6697	132.9712
103.3413	137.3382	103.4489	129.6125	103.5574	132.4907	103.4982	139.3638	103.474	148.3628	103.6864	132.9699

103.3579	137.3326	103.4655	129.6136	103.5741	132.4914	103.5149	139.365	103.4906	148.3568	103.703	132.971
103.3746	137.3344	103.4822	129.6091	103.5907	132.4918	103.5315	139.3612	103.5073	148.3603	103.7197	132.9659
103.3913	137.3353	103.4989	129.6094	103.6074	132.4906	103.5482	139.3616	103.524	148.3602	103.7364	132.966
103.4079	137.3278	103.5155	129.6109	103.6241	132.4887	103.5649	139.3626	103.5406	148.3591	103.753	132.9678
103.4246	137.3316	103.5322	129.6101	103.6407	132.4883	103.5815	139.3605	103.5573	148.3586	103.7697	132.9617
103.4413	137.3332	103.5489	129.6078	103.6574	132.4882	103.5982	139.3631	103.574	148.3581	103.7864	132.9643
103.4579	137.3299	103.5655	129.6129	103.6741	132.4893	103.6149	139.3631	103.5906	148.3592	103.803	132.9659
103.4746	137.3306	103.5822	129.6105	103.6907	132.4884	103.6315	139.3608	103.6073	148.36	103.8197	132.9623
103.4913	137.328	103.5989	129.607	103.7074	132.4877	103.6482	139.3621	103.624	148.3579	103.8364	132.9636
103.5079	137.326	103.6155	129.6051	103.7241	132.4875	103.6649	139.3629	103.6406	148.3573	103.853	132.9626
103.5246	137.3205	103.6322	129.604	103.7407	132.4843	103.6815	139.3606	103.6573	148.3571	103.8697	132.9589
103.5413	137.3263	103.6489	129.6014	103.7574	132.4854	103.6982	139.3597	103.674	148.3573	103.8864	132.9577
103.5579	137.3245	103.6655	129.6039	103.7741	132.4855	103.7149	139.3633	103.6906	148.3597	103.903	132.9587
103.5746	137.3194	103.6822	129.6033	103.7907	132.4856	103.7315	139.3616	103.7073	148.3582	103.9197	132.9597
103.5913	137.3177	103.6989	129.6022	103.8074	132.4825	103.7482	139.3625	103.724	148.358	103.9364	132.9581
103.6079	137.3158	103.7155	129.6032	103.8241	132.4819	103.7649	139.3607	103.7406	148.3573	103.953	132.9565
103.6246	137.3171	103.7322	129.6029	103.8407	132.4852	103.7815	139.3622	103.7573	148.3576	103.9697	132.958
103.6413	137.3126	103.7489	129.6032	103.8574	132.4811	103.7982	139.3596	103.774	148.3586	103.9864	132.9575
103.6579	137.311	103.7655	129.6014	103.8741	132.4814	103.8149	139.3589	103.7906	148.358	104.003	132.9553
103.6746	137.3124	103.7822	129.6013	103.8907	132.4853	103.8315	139.3607	103.8073	148.3578	104.0197	132.9563
103.6913	137.3109	103.7989	129.5985	103.9074	132.4814	103.8482	139.3594	103.824	148.3577	104.0364	132.9549
103.7079	137.311	103.8155	129.6011	103.9241	132.4808	103.8649	139.3586	103.8406	148.3557	104.053	132.9542
103.7246	137.3077	103.8322	129.6027	103.9407	132.4814	103.8815	139.361	103.8573	148.3583	104.0697	132.952
103.7413	137.3054	103.8489	129.5967	103.9574	132.4804	103.8982	139.3582	103.874	148.3577	104.0864	132.9546
103.7579	137.3055	103.8655	129.6005	103.9741	132.4818	103.9149	139.3561	103.8906	148.3574	104.103	132.9512
103.7746	137.3033	103.8822	129.5974	103.9907	132.4804	103.9315	139.3576	103.9073	148.3587	104.1197	132.9513
103.7913	137.3031	103.8989	129.5979	104.0074	132.4808	103.9482	139.3588	103.924	148.3567	104.1364	132.9511
103.8079	137.3031	103.9155	129.5968	104.0241	132.4789	103.9649	139.3569	103.9406	148.3571	104.153	132.95
103.8246	137.3002	103.9322	129.5951	104.0407	132.4792	103.9815	139.3564	103.9573	148.3567	104.1697	132.9492
103.8413	137.3022	103.9489	129.5929	104.0574	132.4809	103.9982	139.3571	103.974	148.3571	104.1864	132.9478
103.8579	137.2993	103.9655	129.5932	104.0741	132.4818	104.0149	139.3559	103.9906	148.3583	104.203	132.9505
103.8746	137.2998	103.9822	129.5929	104.0907	132.4796	104.0315	139.3579	104.0073	148.3548	104.2197	132.9467
103.8913	137.296	103.9989	129.5936	104.1074	132.4793	104.0482	139.3558	104.024	148.3573	104.2364	132.947
103.9079	137.2945	104.0155	129.5926	104.1241	132.4791	104.0649	139.3567	104.0406	148.3571	104.253	132.946
103.9246	137.2938	104.0322	129.5911	104.1407	132.4789	104.0815	139.3585	104.0573	148.3577	104.2697	132.9458
103.9413	137.2939	104.0489	129.5885	104.1574	132.477	104.0982	139.3552	104.074	148.3574	104.2864	132.9421
103.9579	137.2903	104.0655	129.5867	104.1741	132.4772	104.1149	139.3551	104.0906	148.3585	104.303	132.9433
103.9746	137.2928	104.0822	129.5882	104.1907	132.4775	104.1315	139.3528	104.1073	148.3579	104.3197	132.943
103.9913	137.2879	104.0989	129.5861	104.2074	132.4769	104.1482	139.353	104.124	148.3567	104.3364	132.9412
104.0079	137.2912	104.1155	129.5865	104.2241	132.4777	104.1649	139.3547	104.1406	148.3571	104.353	132.9412
104.0246	137.2861	104.1322	129.5863	104.2407	132.4744	104.1815	139.353	104.1573	148.3563	104.3697	132.9417
104.0413	137.2876	104.1489	129.5851	104.2574	132.4779	104.1982	139.3527	104.174	148.357	104.3864	132.9396
104.0579	137.2867	104.1655	129.5871	104.2741	132.4766	104.2149	139.3509	104.1906	148.357	104.403	132.9416
104.0746	137.2831	104.1822	129.5888	104.2907	132.4746	104.2315	139.353	104.2073	148.357	104.4197	132.9389
104.0913	137.2833	104.1989	129.5839	104.3074	132.4778	104.2482	139.3527	104.224	148.3572	104.4364	132.9384
104.1079	137.2803	104.2155	129.5831	104.3241	132.4754	104.2649	139.3517	104.2406	148.3561	104.453	132.9375
104.1246	137.2811	104.2322	129.5848	104.3407	132.474	104.2815	139.3513	104.2573	148.3552	104.4697	132.9373
104.1413	137.2781	104.2489	129.5812	104.3574	132.4758	104.2982	139.3517	104.274	148.3565	104.4864	132.9362
104.1579	137.2772	104.2655	129.5816	104.3741	132.4749	104.3149	139.3529	104.2906	148.3556	104.503	132.934
104.1746	137.273	104.2822	129.5819	104.3907	132.4743	104.3315	139.3542	104.3073	148.3606	104.5197	132.9342
104.1913	137.2745	104.2989	129.5817	104.4074	132.4758	104.3482	139.3505	104.324	148.3563	104.5364	132.9324
104.2079	137.2719	104.3155	129.5833	104.4241	132.4724	104.3649	139.3506	104.3406	148.356	104.553	132.9327
104.2246	137.2717	104.3322	129.5789	104.4407	132.4734	104.3815	139.3521	104.3573	148.3571	104.5697	132.9337
104.2413	137.2684	104.3489	129.579	104.4574	132.4733	104.3982	139.3522	104.374	148.3571	104.5864	132.9314
104.2579	137.2673	104.3655	129.5776	104.4741	132.4718	104.4149	139.3513	104.3906	148.3555	104.603	132.9315
104.2746	137.2672	104.3822	129.5753	104.4907	132.4724	104.4315	139.3493	104.4073	148.3557	104.6197	132.9289
104.2913	137.2639	104.3989	129.5761	104.5074	132.4705	104.4482	139.3502	104.424	148.3548	104.6364	132.9274
104.3079	137.264	104.4155	129.5732	104.5241	132.4716	104.4649	139.3503	104.4406	148.3554	104.653	132.9281
104.3246	137.2649	104.4322	129.5747	104.5407	132.4709	104.4815	139.3501	104.4573	148.3539	104.6697	132.9262
104.3413	137.2616	104.4489	129.5766	104.5574	132.4694	104.4982	139.35	104.474	148.3539	104.6864	132.9264
104.3579	137.2584	104.4655	129.5756	104.5741	132.4694	104.5149	139.3484	104.4906	148.3574	104.703	132.9252
104.3746	137.2582	104.4822	129.5734	104.5907	132.4687	104.5315	139.3479	104.5073	148.354	104.7197	132.9233
104.3913	137.2596	104.4989	129.5718	104.6074	132.469	104.5482	139.3484	104.524	148.3563	104.7364	132.9238
104.4079	137.256	104.5155	129.5716	104.6241	132.4682	104.5649	139.348	104.5406	148.3558	104.753	132.9213
104.4246	137.2563	104.5322	129.5688	104.6407	132.4674	104.5815	139.348	104.5573	148.3534	104.7697	132.9221
104.4413	137.2547	104.5489	129.5694	104.6574	132.4683	104.5982	139.3473	104.574	148.3539	104.7864	132.9201
104.4579	137.2531	104.5655	129.5685	104.6741	132.4674	104.6149	139.3456	104.5906	148.3558	104.803	132.9195
104.4746	137.2533	104.5822	129.573	104.6907	132.4661	104.6315	139.3465	104.6073	148.3542	104.8197	132.9191
104.4913	137.2514	104.5989	129.5701	104.7074	132.4645	104.6482	139.345	104.624	148.3556	104.8364	132.9167

104.5079	137.2492	104.6155	129.5647	104.7241	132.4646	104.6649	139.3464	104.6406	148.3546	104.853	132.9172
104.5246	137.2501	104.6322	129.5683	104.7407	132.4661	104.6815	139.3451	104.6573	148.3529	104.8697	132.915
104.5413	137.2487	104.6489	129.5667	104.7574	132.4644	104.6982	139.3441	104.674	148.3559	104.8864	132.9154
104.5579	137.2465	104.6655	129.5673	104.7741	132.4638	104.7149	139.347	104.6906	148.3542	104.903	132.9131
104.5746	137.2465	104.6822	129.5627	104.7907	132.4616	104.7315	139.3444	104.7073	148.3534	104.9197	132.912
104.5913	137.2462	104.6989	129.5644	104.8074	132.4644	104.7482	139.3411	104.724	148.3547	104.9364	132.9119
104.6079	137.2435	104.7155	129.5634	104.8241	132.4652	104.7649	139.3414	104.7406	148.3539	104.953	132.9134
104.6246	137.2426	104.7322	129.5626	104.8407	132.4624	104.7815	139.3413	104.7573	148.3506	104.9697	132.9094
104.6413	137.2402	104.7489	129.5658	104.8574	132.4619	104.7982	139.341	104.774	148.3533	104.9864	132.9087
104.6579	137.2399	104.7655	129.5596	104.8741	132.4595	104.8149	139.3383	104.7906	148.3512	105.003	132.9083
104.6746	137.2373	104.7822	129.5633	104.8907	132.4635	104.8315	139.3413	104.8073	148.3521	105.0197	132.908
104.6913	137.2392	104.7989	129.5651	104.9074	132.4631	104.8482	139.3395	104.824	148.3527	105.0364	132.9059
104.7079	137.2357	104.8155	129.5597	104.9241	132.463	104.8649	139.3379	104.8406	148.3519	105.053	132.9074
104.7246	137.2322	104.8322	129.5564	104.9407	132.46	104.8815	139.339	104.8573	148.3551	105.0697	132.9054
104.7413	137.2357	104.8489	129.5611	104.9574	132.4591	104.8982	139.337	104.874	148.3534	105.0864	132.905
104.7579	137.2302	104.8655	129.561	104.9741	132.4614	104.9149	139.3376	104.8906	148.3544	105.103	132.9009
104.7746	137.23	104.8822	129.5589	104.9907	132.4582	104.9315	139.3359	104.9073	148.3518	105.1197	132.9038
104.7913	137.2318	104.8989	129.5567	105.0074	132.4597	104.9482	139.3394	104.924	148.3519	105.1364	132.9017
104.8079	137.2306	104.9155	129.5595	105.0241	132.4573	104.9649	139.3377	104.9406	148.354	105.153	132.8997
104.8246	137.2306	104.9322	129.5561	105.0407	132.4583	104.9815	139.3346	104.9573	148.3514	105.1697	132.8991
104.8413	137.2291	104.9489	129.5563	105.0574	132.458	104.9982	139.3332	104.974	148.3531	105.1864	132.8992
104.8579	137.2288	104.9655	129.5562	105.0741	132.4595	105.0149	139.3368	104.9906	148.3525	105.203	132.8989
104.8746	137.2262	104.9822	129.5559	105.0907	132.4589	105.0315	139.3359	105.0073	148.3504	105.2197	132.8958
104.8913	137.2258	104.9989	129.5542	105.1074	132.4551	105.0482	139.3351	105.024	148.3508	105.2364	132.8986
104.9079	137.2194	105.0155	129.5557	105.1241	132.4607	105.0649	139.3353	105.0406	148.3529	105.253	132.8955
104.9246	137.2229	105.0322	129.554	105.1407	132.4573	105.0815	139.332	105.0573	148.351	105.2697	132.8947
104.9413	137.2194	105.0489	129.5513	105.1574	132.4555	105.0982	139.3315	105.074	148.3531	105.2864	132.893
104.9579	137.2168	105.0655	129.5511	105.1741	132.4579	105.1149	139.332	105.0906	148.3522	105.303	132.8936
104.9746	137.22	105.0822	129.5542	105.1907	132.4561	105.1315	139.3309	105.1073	148.3528	105.3197	132.8935
104.9913	137.2163	105.0989	129.5478	105.2074	132.4558	105.1482	139.3315	105.124	148.3513	105.3364	132.89
105.0079	137.2185	105.1155	129.5492	105.2241	132.4563	105.1649	139.3291	105.1406	148.3532	105.353	132.89
105.0246	137.214	105.1322	129.5486	105.2407	132.4549	105.1815	139.3319	105.1573	148.3517	105.3697	132.8879
105.0413	137.2132	105.1489	129.5516	105.2574	132.4547	105.1982	139.3325	105.174	148.3526	105.3864	132.8874
105.0579	137.2133	105.1655	129.5508	105.2741	132.4542	105.2149	139.3291	105.1906	148.3528	105.403	132.8825
105.0746	137.2131	105.1822	129.5504	105.2907	132.4521	105.2315	139.3303	105.2073	148.3511	105.4197	132.885
105.0913	137.2111	105.1989	129.5513	105.3074	132.4555	105.2482	139.329	105.224	148.3527	105.4364	132.8831
105.1079	137.2109	105.2155	129.5476	105.3241	132.4534	105.2649	139.3286	105.2406	148.3521	105.453	132.8808
105.1246	137.2047	105.2322	129.5486	105.3407	132.45	105.2815	139.3312	105.2573	148.3533	105.4697	132.8814
105.1413	137.205	105.2489	129.5458	105.3574	132.4506	105.2982	139.3291	105.274	148.3538	105.4864	132.8804
105.1579	137.2077	105.2655	129.5467	105.3741	132.451	105.3149	139.3313	105.2906	148.351	105.503	132.8783
105.1746	137.2046	105.2822	129.5455	105.3907	132.4526	105.3315	139.3291	105.3073	148.351	105.5197	132.8784
105.1913	137.204	105.2989	129.5469	105.4074	132.4534	105.3482	139.3308	105.324	148.3514	105.5364	132.879
105.2079	137.2028	105.3155	129.5427	105.4241	132.4508	105.3649	139.3292	105.3406	148.3495	105.553	132.8775
105.2246	137.2018	105.3322	129.5438	105.4407	132.4493	105.3815	139.3297	105.3573	148.3516	105.5697	132.8772
105.2413	137.2021	105.3489	129.5436	105.4574	132.4522	105.3982	139.33	105.374	148.3502	105.5864	132.8769
105.2579	137.1981	105.3655	129.5407	105.4741	132.4521	105.4149	139.3292	105.3906	148.3524	105.603	132.8751
105.2746	137.1991	105.3822	129.5397	105.4907	132.4518	105.4315	139.3275	105.4073	148.3501	105.6197	132.8757
105.2913	137.1985	105.3989	129.541	105.5074	132.4522	105.4482	139.3267	105.424	148.3508	105.6364	132.8745
105.3079	137.1988	105.4155	129.5401	105.5241	132.4525	105.4649	139.3246	105.4406	148.3502	105.653	132.8733
105.3246	137.1948	105.4322	129.5405	105.5407	132.4517	105.4815	139.3265	105.4573	148.3484	105.6697	132.874
105.3413	137.1943	105.4489	129.5394	105.5574	132.453	105.4982	139.3248	105.474	148.3503	105.6864	132.8745
105.3579	137.1903	105.4655	129.5397	105.5741	132.4527	105.5149	139.3246	105.4906	148.3516	105.703	132.8709
105.3746	137.1903	105.4822	129.5373	105.5907	132.45	105.5315	139.3244	105.5073	148.3489	105.7197	132.8716
105.3913	137.19	105.4989	129.5382	105.6074	132.4495	105.5482	139.326	105.524	148.3528	105.7364	132.8717
105.4079	137.1887	105.5155	129.5363	105.6241	132.448	105.5649	139.3267	105.5406	148.351	105.753	132.8695
105.4246	137.1864	105.5322	129.5365	105.6407	132.4503	105.5815	139.3262	105.5573	148.3497	105.7697	132.8702
105.4413	137.186	105.5489	129.5354	105.6574	132.4501	105.5982	139.3245	105.574	148.3498	105.7864	132.8684
105.4579	137.1864	105.5655	129.5341	105.6741	132.4495	105.6149	139.3275	105.5906	148.3506	105.803	132.8681
105.4746	137.1836	105.5822	129.535	105.6907	132.4506	105.6315	139.3233	105.6073	148.3485	105.8197	132.8675
105.4913	137.1812	105.5989	129.5348	105.7074	132.4484	105.6482	139.3251	105.624	148.3492	105.8364	132.867
105.5079	137.1814	105.6155	129.5323	105.7241	132.4499	105.6649	139.3227	105.6406	148.3485	105.853	132.8642
105.5246	137.1785	105.6322	129.5325	105.7407	132.4479	105.6815	139.3244	105.6573	148.3489	105.8697	132.8655
105.5413	137.1777	105.6489	129.5321	105.7574	132.4501	105.6982	139.322	105.674	148.3473	105.8864	132.8639
105.5579	137.1739	105.6655	129.533	105.7741	132.4486	105.7149	139.3228	105.6906	148.3492	105.903	132.865
105.5746	137.1737	105.6822	129.5318	105.7907	132.4489	105.7315	139.3231	105.7073	148.3491	105.9197	132.8639
105.5913	137.1751	105.6989	129.5285	105.8074	132.4469	105.7482	139.3234	105.724	148.3465	105.9364	132.8599
105.6079	137.1737	105.7155	129.5282	105.8241	132.4478	105.7649	139.3206	105.7406	148.3477	105.953	132.8623
105.6246	137.1699	105.7322	129.5267	105.8407	132.446	105.7815	139.3259	105.7573	148.3473	105.9697	132.8603
105.6413	137.1693	105.7489	129.5265	105.8574	132.4486	105.7982	139.3239	105.774	148.3504	105.9864	132.8583

105.6579	137.1688	105.7655	129.5237	105.8741	132.4481	105.8149	139.3218	105.7906	148.3484	106.003	132.8584
105.6746	137.1647	105.7822	129.5263	105.8907	132.4485	105.8315	139.3224	105.8073	148.3478	106.0197	132.8565
105.6913	137.1664	105.7989	129.5225	105.9074	132.4455	105.8482	139.3238	105.824	148.3473	106.0364	132.8565
105.7079	137.1662	105.8155	129.5229	105.9241	132.4451	105.8649	139.3209	105.8406	148.3482	106.053	132.859
105.7246	137.1637	105.8322	129.5233	105.9407	132.4472	105.8815	139.3226	105.8573	148.3488	106.0697	132.8574
105.7413	137.1659	105.8489	129.5235	105.9574	132.4472	105.8982	139.3213	105.874	148.3489	106.0864	132.8584
105.7579	137.1623	105.8655	129.5216	105.9741	132.4462	105.9149	139.3218	105.8906	148.3492	106.103	132.8546
105.7746	137.1597	105.8822	129.5193	105.9907	132.4451	105.9315	139.3226	105.9073	148.3482	106.1197	132.8558
105.7913	137.1575	105.8989	129.519	106.0074	132.4437	105.9482	139.3215	105.924	148.348	106.1364	132.8545
105.8079	137.1594	105.9155	129.5196	106.0241	132.4455	105.9649	139.3204	105.9406	148.3458	106.153	132.8533
105.8246	137.1562	105.9322	129.5169	106.0407	132.4449	105.9815	139.3222	105.9573	148.3418	106.1697	132.8496
105.8413	137.1565	105.9489	129.5152	106.0574	132.444	105.9982	139.3195	105.974	148.345	106.1864	132.852
105.8579	137.1532	105.9655	129.5162	106.0741	132.4436	106.0149	139.3183	105.9906	148.344	106.203	132.8507
105.8746	137.1549	105.9822	129.5169	106.0907	132.4441	106.0315	139.32	106.0073	148.3481	106.2197	132.8491
105.8913	137.1542	105.9989	129.5169	106.1074	132.4414	106.0482	139.32	106.024	148.3481	106.2364	132.8504
105.9079	137.1508	106.0155	129.5157	106.1241	132.4444	106.0649	139.3182	106.0406	148.3478	106.253	132.8472
105.9246	137.1505	106.0322	129.5142	106.1407	132.4448	106.0815	139.3201	106.0573	148.3469	106.2697	132.848
105.9413	137.1495	106.0489	129.5138	106.1574	132.4409	106.0982	139.3196	106.074	148.3482	106.2864	132.8479
105.9579	137.1497	106.0655	129.5138	106.1741	132.4407	106.1149	139.3197	106.0906	148.3496	106.303	132.8457
105.9746	137.1506	106.0822	129.5129	106.1907	132.4373	106.1315	139.3196	106.1073	148.3484	106.3197	132.8455
105.9913	137.1478	106.0989	129.5151	106.2074	132.4397	106.1482	139.3143	106.124	148.3449	106.3364	132.8439
106.0079	137.1488	106.1155	129.5126	106.2241	132.4407	106.1649	139.3184	106.1406	148.3479	106.353	132.8446
106.0246	137.1432	106.1322	129.5148	106.2407	132.4387	106.1815	139.3169	106.1573	148.3485	106.3697	132.8441
106.0413	137.1436	106.1489	129.5134	106.2574	132.4368	106.1982	139.317	106.174	148.3458	106.3864	132.844
106.0579	137.1447	106.1655	129.5123	106.2741	132.4364	106.2149	139.3165	106.1906	148.3477	106.403	132.8433
106.0746	137.143	106.1822	129.5096	106.2907	132.4355	106.2315	139.3136	106.2073	148.344	106.4197	132.845
106.0913	137.1417	106.1989	129.5048	106.3074	132.4372	106.2482	139.3193	106.224	148.347	106.4364	132.8401
106.1079	137.142	106.2155	129.5111	106.3241	132.4373	106.2649	139.3175	106.2406	148.3444	106.453	132.8403
106.1246	137.139	106.2322	129.5091	106.3407	132.4355	106.2815	139.318	106.2573	148.3468	106.4697	132.8379
106.1413	137.1375	106.2489	129.5073	106.3574	132.4356	106.2982	139.3162	106.274	148.3487	106.4864	132.8386
106.1579	137.1365	106.2655	129.5052	106.3741	132.4362	106.3149	139.3148	106.2906	148.3448	106.503	132.8377
106.1746	137.1332	106.2822	129.5056	106.3907	132.4375	106.3315	139.3172	106.3073	148.3411	106.5197	132.8385
106.1913	137.1346	106.2989	129.5062	106.4074	132.4365	106.3482	139.3149	106.324	148.3446	106.5364	132.8341
106.2079	137.1336	106.3155	129.5048	106.4241	132.4361	106.3649	139.3143	106.3406	148.3431	106.553	132.8368
106.2246	137.1318	106.3322	129.5057	106.4407	132.4337	106.3815	139.3144	106.3573	148.343	106.5697	132.8378
106.2413	137.1318	106.3489	129.5032	106.4574	132.4359	106.3982	139.3139	106.374	148.3429	106.5864	132.8332
106.2579	137.1323	106.3655	129.5038	106.4741	132.4343	106.4149	139.3137	106.3906	148.3439	106.603	132.8341
106.2746	137.1279	106.3822	129.503	106.4907	132.4369	106.4315	139.3143	106.4073	148.3406	106.6197	132.8361
106.2913	137.1291	106.3989	129.5004	106.5074	132.4339	106.4482	139.3138	106.424	148.3418	106.6364	132.8329
106.3079	137.1285	106.4155	129.5005	106.5241	132.4334	106.4649	139.312	106.4406	148.3436	106.653	132.8328
106.3246	137.1252	106.4322	129.5022	106.5407	132.4348	106.4815	139.3098	106.4573	148.3387	106.6697	132.8333
106.3413	137.1242	106.4489	129.4997	106.5574	132.4314	106.4982	139.3117	106.474	148.3402	106.6864	132.8318
106.3579	137.125	106.4655	129.4999	106.5741	132.432	106.5149	139.312	106.4906	148.3381	106.703	132.831
106.3746	137.1233	106.4822	129.4962	106.5907	132.4332	106.5315	139.3077	106.5073	148.34	106.7197	132.8314
106.3913	137.1223	106.4989	129.5008	106.6074	132.4317	106.5482	139.3117	106.524	148.3399	106.7364	132.8305
106.4079	137.12	106.5155	129.4956	106.6241	132.4322	106.5649	139.3097	106.5406	148.3395	106.753	132.8298
106.4246	137.1203	106.5322	129.4985	106.6407	132.4336	106.5815	139.3115	106.5573	148.3418	106.7697	132.8293
106.4413	137.1172	106.5489	129.498	106.6574	132.4299	106.5982	139.3104	106.574	148.3381	106.7864	132.8291
106.4579	137.1159	106.5655	129.4977	106.6741	132.4307	106.6149	139.3089	106.5906	148.3407	106.803	132.8292
106.4746	137.1174	106.5822	129.496	106.6907	132.4308	106.6315	139.3094	106.6073	148.3374	106.8197	132.8281
106.4913	137.1158	106.5989	129.5019	106.7074	132.4268	106.6482	139.3079	106.624	148.3389	106.8364	132.8279
106.5079	137.1116	106.6155	129.4938	106.7241	132.4301	106.6649	139.3099	106.6406	148.3377	106.853	132.8284
106.5246	137.1143	106.6322	129.4939	106.7407	132.4277	106.6815	139.3084	106.6573	148.3398	106.8697	132.8271
106.5413	137.1141	106.6489	129.4933	106.7574	132.4276	106.6982	139.3059	106.674	148.3364	106.8864	132.8274
106.5579	137.1114	106.6655	129.4946	106.7741	132.4263	106.7149	139.3082	106.6906	148.3385	106.903	132.8247
106.5746	137.1097	106.6822	129.4934	106.7907	132.4253	106.7315	139.3058	106.7073	148.337	106.9197	132.8261
106.5913	137.1136	106.6989	129.4908	106.8074	132.4261	106.7482	139.3072	106.724	148.3375	106.9364	132.8242
106.6079	137.1054	106.7155	129.4905	106.8241	132.4262	106.7649	139.3055	106.7406	148.3373	106.953	132.8241
106.6246	137.1022	106.7322	129.489	106.8407	132.427	106.7815	139.305	106.7573	148.3362	106.9697	132.8231
106.6413	137.1041	106.7489	129.4905	106.8574	132.4278	106.7982	139.304	106.774	148.3339	106.9864	132.8213
106.6579	137.1015	106.7655	129.4858	106.8741	132.4269	106.8149	139.307	106.7906	148.3358	107.003	132.8209
106.6746	137.1015	106.7822	129.488	106.8907	132.4284	106.8315	139.3051	106.8073	148.3361	107.0197	132.8228
106.6913	137.1	106.7989	129.4874	106.9074	132.4281	106.8482	139.3065	106.824	148.3364	107.0364	132.8194
106.7079	137.1005	106.8155	129.4872	106.9241	132.4278	106.8649	139.3045	106.8406	148.3343	107.053	132.8217
106.7246	137.1027	106.8322	129.4849	106.9407	132.4275	106.8815	139.302	106.8573	148.3341	107.0697	132.8194
106.7413	137.0986	106.8489	129.4865	106.9574	132.4271	106.8982	139.302	106.874	148.3338	107.0864	132.8186
106.7579	137.0988	106.8655	129.4852	106.9741	132.4243	106.9149	139.3036	106.8906	148.335	107.103	132.8172
106.7746	137.0993	106.8822	129.4876	106.9907	132.4261	106.9315	139.3021	106.9073	148.3353	107.1197	132.8191
106.7913	137.0907	106.8989	129.4833	107.0074	132.4273	106.9482	139.3018	106.924	148.3353	107.1364	132.8173

106.8079	137.0912	106.9155	129.4835	107.0241	132.4291	106.9649	139.3026	106.9406	148.3359	107.153	132.8173
106.8246	137.0972	106.9322	129.4837	107.0407	132.4276	106.9815	139.2998	106.9573	148.3347	107.1697	132.815
106.8413	137.0908	106.9489	129.4848	107.0574	132.4279	106.9982	139.3004	106.974	148.3357	107.1864	132.8144
106.8579	137.0926	106.9655	129.4821	107.0741	132.4272	107.0149	139.3021	106.9906	148.3351	107.203	132.8121
106.8746	137.0895	106.9822	129.4809	107.0907	132.4266	107.0315	139.2992	107.0073	148.3348	107.2197	132.8154
106.8913	137.0896	106.9989	129.4805	107.1074	132.4206	107.0482	139.2999	107.024	148.338	107.2364	132.8115
106.9079	137.0857	107.0155	129.4821	107.1241	132.4283	107.0649	139.2984	107.0406	148.3306	107.253	132.8122
106.9246	137.0839	107.0322	129.4801	107.1407	132.4258	107.0815	139.3003	107.0573	148.3341	107.2697	132.8116
106.9413	137.0822	107.0489	129.4798	107.1574	132.4254	107.0982	139.3009	107.074	148.3364	107.2864	132.812
106.9579	137.0802	107.0655	129.479	107.1741	132.4249	107.1149	139.2991	107.0906	148.3353	107.303	132.8075
106.9746	137.082	107.0822	129.4772	107.1907	132.4229	107.1315	139.2984	107.1073	148.3352	107.3197	132.8098
106.9913	137.0798	107.0989	129.4783	107.2074	132.4254	107.1482	139.3013	107.124	148.3364	107.3364	132.8088
107.0079	137.0787	107.1155	129.4784	107.2241	132.4235	107.1649	139.298	107.1406	148.3349	107.353	132.8082
107.0246	137.0781	107.1322	129.4763	107.2407	132.4258	107.1815	139.2991	107.1573	148.3376	107.3697	132.807
107.0413	137.0777	107.1489	129.4793	107.2574	132.4246	107.1982	139.2975	107.174	148.3361	107.3864	132.8068
107.0579	137.0747	107.1655	129.4751	107.2741	132.4221	107.2149	139.297	107.1906	148.3343	107.403	132.8047
107.0746	137.0724	107.1822	129.4742	107.2907	132.4198	107.2315	139.2961	107.2073	148.3368	107.4197	132.8047
107.0913	137.0733	107.1989	129.4751	107.3074	132.4204	107.2482	139.2964	107.224	148.3351	107.4364	132.8026
107.1079	137.0687	107.2155	129.4774	107.3241	132.4219	107.2649	139.2972	107.2406	148.3346	107.453	132.802
107.1246	137.0701	107.2322	129.4741	107.3407	132.423	107.2815	139.2967	107.2573	148.3338	107.4697	132.8029
107.1413	137.065	107.2489	129.472	107.3574	132.4221	107.2982	139.2959	107.274	148.3334	107.4864	132.8026
107.1579	137.0698	107.2655	129.4722	107.3741	132.42	107.3149	139.2968	107.2906	148.3354	107.503	132.8017
107.1746	137.0662	107.2822	129.471	107.3907	132.4198	107.3315	139.2966	107.3073	148.3311	107.5197	132.7992
107.1913	137.0668	107.2989	129.471	107.4074	132.4208	107.3482	139.2944	107.324	148.3362	107.5364	132.8007
107.2079	137.0631	107.3155	129.4708	107.4241	132.4214	107.3649	139.2938	107.3406	148.3317	107.553	132.7999
107.2246	137.063	107.3322	129.4679	107.4407	132.4231	107.3815	139.2945	107.3573	148.3331	107.5697	132.7977
107.2413	137.062	107.3489	129.4697	107.4574	132.421	107.3982	139.2962	107.374	148.3307	107.5864	132.7973
107.2579	137.0622	107.3655	129.4686	107.4741	132.4169	107.4149	139.2961	107.3906	148.3312	107.603	132.7978
107.2746	137.0602	107.3822	129.4695	107.4907	132.4232	107.4315	139.2938	107.4073	148.3311	107.6197	132.7983
107.2913	137.0599	107.3989	129.4663	107.5074	132.4213	107.4482	139.2947	107.424	148.3321	107.6364	132.7955
107.3079	137.057	107.4155	129.4668	107.5241	132.4222	107.4649	139.2932	107.4406	148.3316	107.653	132.794
107.3246	137.0603	107.4322	129.4669	107.5407	132.4221	107.4815	139.2939	107.4573	148.3296	107.6697	132.7954
107.3413	137.057	107.4489	129.4663	107.5574	132.4213	107.4982	139.2926	107.474	148.3302	107.6864	132.7957
107.3579	137.0549	107.4655	129.4643	107.5741	132.422	107.5149	139.2945	107.4906	148.3295	107.703	132.7932
107.3746	137.0543	107.4822	129.4645	107.5907	132.4197	107.5315	139.2921	107.5073	148.3293	107.7197	132.7901
107.3913	137.0542	107.4989	129.4596	107.6074	132.4208	107.5482	139.2917	107.524	148.331	107.7364	132.7934
107.4079	137.0481	107.5155	129.4604	107.6241	132.4204	107.5649	139.2916	107.5406	148.3286	107.753	132.7916
107.4246	137.0493	107.5322	129.459	107.6407	132.4184	107.5815	139.2937	107.5573	148.3307	107.7697	132.7906
107.4413	137.0494	107.5489	129.4602	107.6574	132.4218	107.5982	139.2917	107.574	148.3283	107.7864	132.7902
107.4579	137.0458	107.5655	129.4609	107.6741	132.42	107.6149	139.2905	107.5906	148.3292	107.803	132.7884
107.4746	137.0461	107.5822	129.4617	107.6907	132.4196	107.6315	139.2913	107.6073	148.3302	107.8197	132.7899
107.4913	137.0434	107.5989	129.4589	107.7074	132.4192	107.6482	139.2922	107.624	148.3272	107.8364	132.7877
107.5079	137.0431	107.6155	129.4572	107.7241	132.4191	107.6649	139.2911	107.6406	148.3291	107.853	132.7875
107.5246	137.0426	107.6322	129.458	107.7407	132.4197	107.6815	139.2914	107.6573	148.3299	107.8697	132.7867
107.5413	137.0409	107.6489	129.4576	107.7574	132.4174	107.6982	139.2897	107.674	148.3267	107.8864	132.7891
107.5579	137.0394	107.6655	129.4576	107.7741	132.4209	107.7149	139.2901	107.6906	148.3303	107.903	132.7856
107.5746	137.0391	107.6822	129.458	107.7907	132.4193	107.7315	139.2909	107.7073	148.3272	107.9197	132.7867
107.5913	137.0383	107.6989	129.4544	107.8074	132.4159	107.7482	139.2892	107.724	148.3276	107.9364	132.7839
107.6079	137.0348	107.7155	129.4552	107.8241	132.4199	107.7649	139.2866	107.7406	148.3271	107.953	132.785
107.6246	137.0356	107.7322	129.4542	107.8407	132.4199	107.7815	139.2862	107.7573	148.328	107.9697	132.7816
107.6413	137.0353	107.7489	129.4535	107.8574	132.4203	107.7982	139.2871	107.774	148.3262	107.9864	132.7839
107.6579	137.0338	107.7655	129.4507	107.8741	132.4168	107.8149	139.2887	107.7906	148.3277	108.003	132.7812
107.6746	137.0314	107.7822	129.4536	107.8907	132.42	107.8315	139.2852	107.8073	148.3288	108.0197	132.7817
107.6913	137.0318	107.7989	129.4521	107.9074	132.4166	107.8482	139.2874	107.824	148.3284	108.0364	132.7814
107.7079	137.0293	107.8155	129.451	107.9241	132.4164	107.8649	139.2866	107.8406	148.3267	108.053	132.7805
107.7246	137.0289	107.8322	129.4526	107.9407	132.419	107.8815	139.2874	107.8573	148.328	108.0697	132.7795
107.7413	137.029	107.8489	129.4525	107.9574	132.4174	107.8982	139.2859	107.874	148.3268	108.0864	132.7803
107.7579	137.0285	107.8655	129.4511	107.9741	132.4191	107.9149	139.2866	107.8906	148.3301	108.103	132.779
107.7746	137.0277	107.8822	129.448	107.9907	132.4188	107.9315	139.2857	107.9073	148.3293	108.1197	132.7782
107.7913	137.0222	107.8989	129.4466	108.0074	132.4191	107.9482	139.287	107.924	148.3231	108.1364	132.7783
107.8079	137.024	107.9155	129.4465	108.0241	132.4189	107.9649	139.2871	107.9406	148.3271	108.153	132.7772
107.8246	137.0236	107.9322	129.4445	108.0407	132.421	107.9815	139.2839	107.9573	148.3254	108.1697	132.7772
107.8413	137.0218	107.9489	129.4435	108.0574	132.4165	107.9982	139.2856	107.974	148.3234	108.1864	132.7767
107.8579	137.0234	107.9655	129.4452	108.0741	132.4167	108.0149	139.2868	107.9906	148.3276	108.203	132.7767
107.8746	137.0192	107.9822	129.4426	108.0907	132.4171	108.0315	139.2856	108.0073	148.3284	108.2197	132.7754
107.8913	137.0211	107.9989	129.4436	108.1074	132.4185	108.0482	139.2873	108.024	148.326	108.2364	132.7746
107.9079	137.0172	108.0155	129.4419	108.1241	132.4171	108.0649	139.2862	108.0406	148.3245	108.253	132.7736
107.9246	137.0174	108.0322	129.4408	108.1407	132.4174	108.0815	139.2874	108.0573	148.3248	108.2697	132.7748
107.9413	137.0132	108.0489	129.4398	108.1574	132.4159	108.0982	139.2867	108.074	148.3261	108.2864	132.7729

107.9579	137.0137	108.0655	129.4397	108.1741	132.4177	108.1149	139.2906	108.0906	148.3261	108.303	132.7723
107.9746	137.0094	108.0822	129.4393	108.1907	132.4143	108.1315	139.2867	108.1073	148.3262	108.3197	132.7708
107.9913	137.0096	108.0989	129.4389	108.2074	132.414	108.1482	139.2893	108.124	148.3273	108.3364	132.7702
108.0079	137.0067	108.1155	129.4391	108.2241	132.4122	108.1649	139.288	108.1406	148.3275	108.353	132.7697
108.0246	137.0077	108.1322	129.4358	108.2407	132.4125	108.1815	139.2861	108.1573	148.3279	108.3697	132.7686
108.0413	137.0058	108.1489	129.4359	108.2574	132.4135	108.1982	139.2873	108.174	148.3265	108.3864	132.7703
108.0579	137.0062	108.1655	129.4363	108.2741	132.4154	108.2149	139.2868	108.1906	148.3252	108.403	132.7674
108.0746	137.0058	108.1822	129.4355	108.2907	132.413	108.2315	139.2875	108.2073	148.3242	108.4197	132.7658
108.0913	137.0027	108.1989	129.4349	108.3074	132.4126	108.2482	139.2881	108.224	148.3246	108.4364	132.766
108.1079	137.0004	108.2155	129.4321	108.3241	132.41	108.2649	139.2846	108.2406	148.3252	108.453	132.765
108.1246	137.0006	108.2322	129.4325	108.3407	132.4099	108.2815	139.2851	108.2573	148.3262	108.4697	132.7656
108.1413	136.998	108.2489	129.4334	108.3574	132.4106	108.2982	139.2861	108.274	148.325	108.4864	132.7638
108.1579	136.9972	108.2655	129.4325	108.3741	132.4103	108.3149	139.2858	108.2906	148.3258	108.503	132.7626
108.1746	136.9938	108.2822	129.4301	108.3907	132.4096	108.3315	139.2828	108.3073	148.3228	108.5197	132.7618
108.1913	136.9951	108.2989	129.4291	108.4074	132.4086	108.3482	139.2859	108.324	148.3242	108.5364	132.7637
108.2079	136.9926	108.3155	129.4288	108.4241	132.4091	108.3649	139.2837	108.3406	148.3237	108.553	132.7607
108.2246	136.9924	108.3322	129.4271	108.4407	132.4081	108.3815	139.2834	108.3573	148.3256	108.5697	132.7616
108.2413	136.9914	108.3489	129.4276	108.4574	132.4062	108.3982	139.2833	108.374	148.3224	108.5864	132.7588
108.2579	136.9903	108.3655	129.4254	108.4741	132.4077	108.4149	139.2821	108.3906	148.3246	108.603	132.7608
108.2746	136.9907	108.3822	129.4275	108.4907	132.4065	108.4315	139.2828	108.4073	148.3211	108.6197	132.7567
108.2913	136.9891	108.3989	129.4261	108.5074	132.4055	108.4482	139.2851	108.424	148.3242	108.6364	132.757
108.3079	136.9887	108.4155	129.4262	108.5241	132.4055	108.4649	139.2832	108.4406	148.3208	108.653	132.7567
108.3246	136.9901	108.4322	129.4282	108.5407	132.4041	108.4815	139.2828	108.4573	148.3202	108.6697	132.7539
108.3413	136.9871	108.4489	129.4247	108.5574	132.4036	108.4982	139.2799	108.474	148.3226	108.6864	132.7542
108.3579	136.9836	108.4655	129.4246	108.5741	132.4052	108.5149	139.2853	108.4906	148.3211	108.703	132.7554
108.3746	136.9804	108.4822	129.4237	108.5907	132.4064	108.5315	139.2821	108.5073	148.3207	108.7197	132.7531
108.3913	136.9832	108.4989	129.4233	108.6074	132.4055	108.5482	139.2825	108.524	148.3192	108.7364	132.7523
108.4079	136.9801	108.5155	129.423	108.6241	132.4045	108.5649	139.2823	108.5406	148.3205	108.753	132.7543
108.4246	136.9806	108.5322	129.421	108.6407	132.4042	108.5815	139.2807	108.5573	148.3203	108.7697	132.7505
108.4413	136.9784	108.5489	129.4211	108.6574	132.4055	108.5982	139.2822	108.574	148.3218	108.7864	132.752
108.4579	136.979	108.5655	129.4183	108.6741	132.4044	108.6149	139.2822	108.5906	148.3204	108.803	132.7492
108.4746	136.9804	108.5822	129.4217	108.6907	132.405	108.6315	139.2817	108.6073	148.3197	108.8197	132.7492
108.4913	136.9782	108.5989	129.422	108.7074	132.4064	108.6482	139.2805	108.624	148.3199	108.8364	132.7494
108.5079	136.9771	108.6155	129.4202	108.7241	132.4049	108.6649	139.2789	108.6406	148.3186	108.853	132.7485
108.5246	136.9759	108.6322	129.4197	108.7407	132.4033	108.6815	139.2821	108.6573	148.3206	108.8697	132.7478
108.5413	136.9743	108.6489	129.4163	108.7574	132.4062	108.6982	139.2801	108.674	148.3191	108.8864	132.7482
108.5579	136.9697	108.6655	129.4176	108.7741	132.4035	108.7149	139.2787	108.6906	148.3194	108.903	132.7455
108.5746	136.9715	108.6822	129.4179	108.7907	132.4058	108.7315	139.2778	108.7073	148.3167	108.9197	132.7469
108.5913	136.9685	108.6989	129.4173	108.8074	132.4089	108.7482	139.2774	108.724	148.3175	108.9364	132.7471
108.6079	136.9676	108.7155	129.4183	108.8241	132.4048	108.7649	139.2783	108.7406	148.3162	108.953	132.746
108.6246	136.9709	108.7322	129.4155	108.8407	132.4066	108.7815	139.2769	108.7573	148.3181	108.9697	132.7454
108.6413	136.9661	108.7489	129.4171	108.8574	132.4061	108.7982	139.2769	108.774	148.3174	108.9864	132.7445
108.6579	136.9638	108.7655	129.416	108.8741	132.4061	108.8149	139.2758	108.7906	148.3169	109.003	132.7452
108.6746	136.9669	108.7822	129.4084	108.8907	132.4036	108.8315	139.274	108.8073	148.3199	109.0197	132.7437
108.6913	136.9611	108.7989	129.4122	108.9074	132.404	108.8482	139.2739	108.824	148.3186	109.0364	132.7413
108.7079	136.9586	108.8155	129.4105	108.9241	132.4012	108.8649	139.2733	108.8406	148.3162	109.053	132.7402
108.7246	136.9647	108.8322	129.4118	108.9407	132.4049	108.8815	139.2737	108.8573	148.3173	109.0697	132.743
108.7413	136.9598	108.8489	129.4105	108.9574	132.4054	108.8982	139.2711	108.874	148.316	109.0864	132.7409
108.7579	136.9574	108.8655	129.4101	108.9741	132.4021	108.9149	139.272	108.8906	148.3162	109.103	132.7386
108.7746	136.9577	108.8822	129.4102	108.9907	132.4029	108.9315	139.2693	108.9073	148.3161	109.1197	132.7401
108.7913	136.9578	108.8989	129.4092	109.0074	132.4018	108.9482	139.271	108.924	148.3151	109.1364	132.7394
108.8079	136.9549	108.9155	129.4074	109.0241	132.402	108.9649	139.2721	108.9406	148.3164	109.153	132.7403
108.8246	136.9566	108.9322	129.408	109.0407	132.4024	108.9815	139.2697	108.9573	148.3152	109.1697	132.7378
108.8413	136.9543	108.9489	129.4081	109.0574	132.4028	108.9982	139.2705	108.974	148.3149	109.1864	132.7393
108.8579	136.9541	108.9655	129.406	109.0741	132.402	109.0149	139.2698	108.9906	148.3129	109.203	132.7353
108.8746	136.9569	108.9822	129.4071	109.0907	132.4006	109.0315	139.2681	109.0073	148.3137	109.2197	132.7341
108.8913	136.9471	108.9989	129.4013	109.1074	132.4016	109.0482	139.2712	109.024	148.3143	109.2364	132.7321
108.9079	136.9509	109.0155	129.4053	109.1241	132.3997	109.0649	139.2687	109.0406	148.3132	109.253	132.7345
108.9246	136.9486	109.0322	129.4038	109.1407	132.3989	109.0815	139.2674	109.0573	148.3161	109.2697	132.7321
108.9413	136.9494	109.0489	129.4013	109.1574	132.3986	109.0982	139.2705	109.074	148.3124	109.2864	132.7321
108.9579	136.949	109.0655	129.4011	109.1741	132.3991	109.1149	139.2696	109.0906	148.3161	109.303	132.7313
108.9746	136.9435	109.0822	129.4032	109.1907	132.3993	109.1315	139.2697	109.1073	148.3132	109.3197	132.7298
108.9913	136.9454	109.0989	129.4004	109.2074	132.3968	109.1482	139.2692	109.124	148.3178	109.3364	132.7279
109.0079	136.9445	109.1155	129.4019	109.2241	132.3977	109.1649	139.2686	109.1406	148.3167	109.353	132.7294
109.0246	136.9426	109.1322	129.3979	109.2407	132.3962	109.1815	139.268	109.1573	148.3174	109.3697	132.7278
109.0413	136.9392	109.1489	129.397	109.2574	132.3951	109.1982	139.2688	109.174	148.3153	109.3864	132.726
109.0579	136.9432	109.1655	129.3953	109.2741	132.3966	109.2149	139.2668	109.1906	148.3213	109.403	132.7248
109.0746	136.9441	109.1822	129.3942	109.2907	132.3954	109.2315	139.2679	109.2073	148.317	109.4197	132.7234
109.0913	136.9374	109.1989	129.3967	109.3074	132.3964	109.2482	139.2662	109.224	148.3186	109.4364	132.7249

109.1079	136.9384	109.2155	129.395	109.3241	132.3934	109.2649	139.2641	109.2406	148.3177	109.453	132.7228
109.1246	136.9389	109.2322	129.3939	109.3407	132.3915	109.2815	139.2633	109.2573	148.3188	109.4697	132.7218
109.1413	136.9351	109.2489	129.3971	109.3574	132.3947	109.2982	139.2641	109.274	148.3177	109.4864	132.7191
109.1579	136.9359	109.2655	129.3919	109.3741	132.3932	109.3149	139.2646	109.2906	148.315	109.503	132.7224
109.1746	136.9317	109.2822	129.3942	109.3907	132.3956	109.3315	139.2628	109.3073	148.3157	109.5197	132.7207
109.1913	136.9298	109.2989	129.3951	109.4074	132.3917	109.3482	139.2637	109.324	148.3153	109.5364	132.7189
109.2079	136.9315	109.3155	129.3944	109.4241	132.3926	109.3649	139.2642	109.3406	148.3161	109.553	132.719
109.2246	136.9293	109.3322	129.3905	109.4407	132.3934	109.3815	139.2627	109.3573	148.3136	109.5697	132.7163
109.2413	136.9291	109.3489	129.3913	109.4574	132.3908	109.3982	139.2635	109.374	148.315	109.5864	132.7185
109.2579	136.9267	109.3655	129.3962	109.4741	132.3902	109.4149	139.2603	109.3906	148.3151	109.603	132.7165
109.2746	136.9247	109.3822	129.3928	109.4907	132.3887	109.4315	139.2619	109.4073	148.315	109.6197	132.7165
109.2913	136.9242	109.3989	129.3881	109.5074	132.3894	109.4482	139.2621	109.424	148.3145	109.6364	132.7176
109.3079	136.9231	109.4155	129.3926	109.5241	132.3909	109.4649	139.2623	109.4406	148.3123	109.653	132.7161
109.3246	136.924	109.4322	129.3889	109.5407	132.3918	109.4815	139.262	109.4573	148.3134	109.6697	132.7153
109.3413	136.9231	109.4489	129.3878	109.5574	132.3894	109.4982	139.2598	109.474	148.31	109.6864	132.7157
109.3579	136.9196	109.4655	129.3859	109.5741	132.3895	109.5149	139.2608	109.4906	148.3136	109.703	132.7149
109.3746	136.9163	109.4822	129.3896	109.5907	132.3902	109.5315	139.2616	109.5073	148.3124	109.7197	132.7131
109.3913	136.9152	109.4989	129.3899	109.6074	132.393	109.5482	139.2608	109.524	148.3102	109.7364	132.7138
109.4079	136.9174	109.5155	129.3877	109.6241	132.3908	109.5649	139.2579	109.5406	148.3125	109.753	132.7125
109.4246	136.9168	109.5322	129.3868	109.6407	132.3922	109.5815	139.2578	109.5573	148.3088	109.7697	132.7125
109.4413	136.912	109.5489	129.3875	109.6574	132.3901	109.5982	139.2581	109.574	148.3093	109.7864	132.7097
109.4579	136.9128	109.5655	129.3863	109.6741	132.3895	109.6149	139.2588	109.5906	148.3115	109.803	132.7098
109.4746	136.9141	109.5822	129.386	109.6907	132.3932	109.6315	139.2589	109.6073	148.3105	109.8197	132.7097
109.4913	136.9129	109.5989	129.3839	109.7074	132.3904	109.6482	139.2581	109.624	148.3092	109.8364	132.7084
109.5079	136.9124	109.6155	129.3853	109.7241	132.3912	109.6649	139.2561	109.6406	148.3116	109.853	132.7073
109.5246	136.912	109.6322	129.3851	109.7407	132.3932	109.6815	139.259	109.6573	148.3111	109.8697	132.7074
109.5413	136.91	109.6489	129.3844	109.7574	132.3902	109.6982	139.2568	109.674	148.3084	109.8864	132.7072
109.5579	136.9105	109.6655	129.3814	109.7741	132.3934	109.7149	139.2566	109.6906	148.3104	109.903	132.7068
109.5746	136.9069	109.6822	129.3807	109.7907	132.3936	109.7315	139.2541	109.7073	148.3101	109.9197	132.7043
109.5913	136.9033	109.6989	129.3798	109.8074	132.3929	109.7482	139.2566	109.724	148.3098	109.9364	132.7062
109.6079	136.9065	109.7155	129.3823	109.8241	132.3932	109.7649	139.2534	109.7406	148.3097	109.953	132.7043
109.6246	136.9023	109.7322	129.3768	109.8407	132.3928	109.7815	139.2552	109.7573	148.3099	109.9697	132.7034
109.6413	136.903	109.7489	129.3788	109.8574	132.3932	109.7982	139.2522	109.774	148.3091	109.9864	132.703
109.6579	136.9023	109.7655	129.3773	109.8741	132.3916	109.8149	139.2545	109.7906	148.3088	110.003	132.7015
109.6746	136.9035	109.7822	129.3806	109.8907	132.3895	109.8315	139.2544	109.8073	148.3067	110.0197	132.7018
109.6913	136.902	109.7989	129.3792	109.9074	132.3918	109.8482	139.2525	109.824	148.3083	110.0364	132.6972
109.7079	136.9011	109.8155	129.3784	109.9241	132.3917	109.8649	139.2551	109.8406	148.3087	110.053	132.6999
109.7246	136.8969	109.8322	129.3769	109.9407	132.3893	109.8815	139.252	109.8573	148.3084	110.0697	132.7011
109.7413	136.8981	109.8489	129.3752	109.9574	132.3908	109.8982	139.2545	109.874	148.3046	110.0864	132.6966
109.7579	136.8998	109.8655	129.3752	109.9741	132.3911	109.9149	139.2539	109.8906	148.3064	110.103	132.6978
109.7746	136.8962	109.8822	129.373	109.9907	132.3901	109.9315	139.253	109.9073	148.3054	110.1197	132.6925
109.7913	136.895	109.8989	129.374	110.0074	132.3889	109.9482	139.2513	109.924	148.3024	110.1364	132.6973
109.8079	136.8942	109.9155	129.3713	110.0241	132.3906	109.9649	139.2509	109.9406	148.3052	110.153	132.695
109.8246	136.893	109.9322	129.3699	110.0407	132.3902	109.9815	139.2545	109.9573	148.3035	110.1697	132.6932
109.8413	136.8916	109.9489	129.3707	110.0574	132.392	109.9982	139.2521	109.974	148.3033	110.1864	132.6931
109.8579	136.8922	109.9655	129.3701	110.0741	132.3912	110.0149	139.2503	109.9906	148.3045	110.203	132.6925
109.8746	136.888	109.9822	129.3693	110.0907	132.3905	110.0315	139.2545	110.0073	148.3054	110.2197	132.6916
109.8913	136.8906	109.9989	129.3679	110.1074	132.3915	110.0482	139.2493	110.024	148.3047	110.2364	132.692
109.9079	136.8867	110.0155	129.3656	110.1241	132.3899	110.0649	139.2523	110.0406	148.3042	110.253	132.6914
109.9246	136.8894	110.0322	129.3691	110.1407	132.3893	110.0815	139.2499	110.0573	148.2995	110.2697	132.6881
109.9413	136.8871	110.0489	129.3663	110.1574	132.3903	110.0982	139.25	110.074	148.3012	110.2864	132.6918
109.9579	136.8857	110.0655	129.3618	110.1741	132.3917	110.1149	139.249	110.0906	148.3029	110.303	132.6901
109.9746	136.8833	110.0822	129.3642	110.1907	132.3888	110.1315	139.2464	110.1073	148.3001	110.3197	132.6889
109.9913	136.8796	110.0989	129.3668	110.2074	132.3922	110.1482	139.247	110.124	148.3012	110.3364	132.6854
110.0079	136.8822	110.1155	129.3628	110.2241	132.3922	110.1649	139.2462	110.1406	148.3026	110.353	132.688
110.0246	136.878	110.1322	129.3566	110.2407	132.3921	110.1815	139.2456	110.1573	148.3014	110.3697	132.6866
110.0413	136.8774	110.1489	129.3571	110.2574	132.3904	110.1982	139.2439	110.174	148.3012	110.3864	132.6856
110.0579	136.8776	110.1655	129.3568	110.2741	132.3905	110.2149	139.2432	110.1906	148.3009	110.403	132.6873
110.0746	136.8762	110.1822	129.3551	110.2907	132.3893	110.2315	139.2425	110.2073	148.2978	110.4197	132.6862
110.0913	136.8763	110.1989	129.3561	110.3074	132.3892	110.2482	139.2427	110.224	148.2972	110.4364	132.6837
110.1079	136.8743	110.2155	129.3581	110.3241	132.3916	110.2649	139.241	110.2406	148.2973	110.453	132.6822
110.1246	136.8725	110.2322	129.3554	110.3407	132.3927	110.2815	139.2398	110.2573	148.2992	110.4697	132.6846
110.1413	136.8733	110.2489	129.3558	110.3574	132.3908	110.2982	139.2395	110.274	148.2978	110.4864	132.6818
110.1579	136.871	110.2655	129.356	110.3741	132.3921	110.3149	139.2407	110.2906	148.2985	110.503	132.683
110.1746	136.8709	110.2822	129.3553	110.3907	132.3911	110.3315	139.2384	110.3073	148.295	110.5197	132.6806
110.1913	136.8691	110.2989	129.3539	110.4074	132.3893	110.3482	139.2388	110.324	148.2979	110.5364	132.6794
110.2079	136.8689	110.3155	129.3522	110.4241	132.3941	110.3649	139.2388	110.3406	148.2969	110.553	132.6798
110.2246	136.868	110.3322	129.3547	110.4407	132.3928	110.3815	139.2364	110.3573	148.2985	110.5697	132.6794
110.2413	136.8648	110.3489	129.3527	110.4574	132.3908	110.3982	139.2378	110.374	148.296	110.5864	132.6808

110.2579	136.866	110.3655	129.3524	110.4741	132.3927	110.4149	139.2363	110.3906	148.2996	110.603	132.6771
110.2746	136.8613	110.3822	129.3504	110.4907	132.3914	110.4315	139.2357	110.4073	148.2938	110.6197	132.6794
110.2913	136.8605	110.3989	129.3493	110.5074	132.3936	110.4482	139.2355	110.424	148.2976	110.6364	132.6748
110.3079	136.8594	110.4155	129.3498	110.5241	132.3922	110.4649	139.2363	110.4406	148.2955	110.653	132.6767
110.3246	136.8584	110.4322	129.346	110.5407	132.3938	110.4815	139.2345	110.4573	148.2952	110.6697	132.677
110.3413	136.8569	110.4489	129.3487	110.5574	132.392	110.4982	139.2337	110.474	148.2965	110.6864	132.6748
110.3579	136.8574	110.4655	129.3479	110.5741	132.3907	110.5149	139.2321	110.4906	148.2926	110.703	132.6735
110.3746	136.8547	110.4822	129.3436	110.5907	132.3939	110.5315	139.2323	110.5073	148.2929	110.7197	132.6732
110.3913	136.8531	110.4989	129.3479	110.6074	132.3944	110.5482	139.2301	110.524	148.2936	110.7364	132.6734
110.4079	136.8498	110.5155	129.3461	110.6241	132.3921	110.5649	139.2299	110.5406	148.2968	110.753	132.6731
110.4246	136.8526	110.5322	129.3453	110.6407	132.3915	110.5815	139.227	110.5573	148.2957	110.7697	132.6715
110.4413	136.8511	110.5489	129.3466	110.6574	132.392	110.5982	139.2254	110.574	148.2945	110.7864	132.6712
110.4579	136.8483	110.5655	129.3444	110.6741	132.3938	110.6149	139.2261	110.5906	148.2956	110.803	132.6684
110.4746	136.8476	110.5822	129.3417	110.6907	132.3945	110.6315	139.225	110.6073	148.2943	110.8197	132.6699
110.4913	136.8481	110.5989	129.3427	110.7074	132.3927	110.6482	139.2268	110.624	148.2967	110.8364	132.668
110.5079	136.8448	110.6155	129.3454	110.7241	132.3917	110.6649	139.2267	110.6406	148.2909	110.853	132.6654
110.5246	136.8434	110.6322	129.3415	110.7407	132.3887	110.6815	139.2243	110.6573	148.2966	110.8697	132.6667
110.5413	136.8419	110.6489	129.3414	110.7574	132.3929	110.6982	139.2254	110.674	148.2946	110.8864	132.6662
110.5579	136.8422	110.6655	129.3407	110.7741	132.3917	110.7149	139.2256	110.6906	148.2936	110.903	132.6641
110.5746	136.8389	110.6822	129.3389	110.7907	132.3911	110.7315	139.226	110.7073	148.2971	110.9197	132.6641
110.5913	136.8404	110.6989	129.3411	110.8074	132.3908	110.7482	139.2265	110.724	148.2972	110.9364	132.6644
110.6079	136.8422	110.7155	129.339	110.8241	132.3896	110.7649	139.2229	110.7406	148.2902	110.953	132.6642
110.6246	136.8395	110.7322	129.3398	110.8407	132.3901	110.7815	139.2245	110.7573	148.2932	110.9697	132.6627
110.6413	136.8391	110.7489	129.3352	110.8574	132.3893	110.7982	139.2266	110.774	148.2902	110.9864	132.662
110.6579	136.8398	110.7655	129.3358	110.8741	132.3876	110.8149	139.2231	110.7906	148.2956	111.003	132.6622
110.6746	136.8372	110.7822	129.3327	110.8907	132.389	110.8315	139.2248	110.8073	148.2931	111.0197	132.6605
110.6913	136.8376	110.7989	129.3376	110.9074	132.3895	110.8482	139.2241	110.824	148.2918	111.0364	132.6591
110.7079	136.8349	110.8155	129.3335	110.9241	132.3891	110.8649	139.2217	110.8406	148.2911	111.053	132.6596
110.7246	136.8351	110.8322	129.3327	110.9407	132.3907	110.8815	139.2239	110.8573	148.2907	111.0697	132.6582
110.7413	136.8311	110.8489	129.3351	110.9574	132.3868	110.8982	139.2241	110.874	148.2942	111.0864	132.6585
110.7579	136.8345	110.8655	129.3358	110.9741	132.389	110.9149	139.224	110.8906	148.2918	111.103	132.6593
110.7746	136.8279	110.8822	129.3339	110.9907	132.3891	110.9315	139.2238	110.9073	148.2898	111.1197	132.6566
110.7913	136.8273	110.8989	129.3328	111.0074	132.3879	110.9482	139.2214	110.924	148.2922	111.1364	132.655
110.8079	136.8254	110.9155	129.3331	111.0241	132.3876	110.9649	139.224	110.9406	148.2923	111.153	132.6553
110.8246	136.8276	110.9322	129.3313	111.0407	132.3881	110.9815	139.2233	110.9573	148.294	111.1697	132.6558
110.8413	136.8258	110.9489	129.3323	111.0574	132.3845	110.9982	139.2229	110.974	148.2914	111.1864	132.6553
110.8579	136.8248	110.9655	129.3288	111.0741	132.3862	111.0149	139.2233	110.9906	148.2906	111.203	132.6527
110.8746	136.8242	110.9822	129.331	111.0907	132.3875	111.0315	139.222	111.0073	148.2882	111.2197	132.6546
110.8913	136.8214	110.9989	129.3292	111.1074	132.3872	111.0482	139.2222	111.024	148.2904	111.2364	132.6544
110.9079	136.8235	111.0155	129.3306	111.1241	132.3888	111.0649	139.2237	111.0406	148.2907	111.253	132.6513
110.9246	136.8208	111.0322	129.3291	111.1407	132.3878	111.0815	139.2225	111.0573	148.2898	111.2697	132.6535
110.9413	136.8206	111.0489	129.3271	111.1574	132.387	111.0982	139.2203	111.074	148.2895	111.2864	132.6525
110.9579	136.8198	111.0655	129.3271	111.1741	132.3857	111.1149	139.2222	111.0906	148.2906	111.303	132.6487
110.9746	136.8195	111.0822	129.3288	111.1907	132.3852	111.1315	139.2211	111.1073	148.2917	111.3197	132.6497
110.9913	136.8176	111.0989	129.3274	111.2074	132.3891	111.1482	139.2223	111.124	148.2887	111.3364	132.6484
111.0079	136.8183	111.1155	129.3271	111.2241	132.3889	111.1649	139.219	111.1406	148.2909	111.353	132.6479
111.0246	136.8145	111.1322	129.3271	111.2407	132.3856	111.1815	139.2203	111.1573	148.2896	111.3697	132.6459
111.0413	136.8088	111.1489	129.3239	111.2574	132.388	111.1982	139.2206	111.174	148.2891	111.3864	132.648
111.0579	136.8163	111.1655	129.3262	111.2741	132.3885	111.2149	139.2201	111.1906	148.2885	111.403	132.645
111.0746	136.8107	111.1822	129.3229	111.2907	132.3889	111.2315	139.2231	111.2073	148.2912	111.4197	132.6455
111.0913	136.8089	111.1989	129.3221	111.3074	132.3883	111.2482	139.2196	111.224	148.2891	111.4364	132.643
111.1079	136.8086	111.2155	129.32	111.3241	132.3886	111.2649	139.2203	111.2406	148.2906	111.453	132.644
111.1246	136.8048	111.2322	129.3213	111.3407	132.3874	111.2815	139.2178	111.2573	148.2881	111.4697	132.6437
111.1413	136.8066	111.2489	129.322	111.3574	132.389	111.2982	139.22	111.274	148.2909	111.4864	132.6434
111.1579	136.8056	111.2655	129.32	111.3741	132.3867	111.3149	139.222	111.2906	148.2898	111.503	132.6393
111.1746	136.8049	111.2822	129.32	111.3907	132.3879	111.3315	139.2212	111.3073	148.2876	111.5197	132.6388
111.1913	136.8039	111.2989	129.3189	111.4074	132.3893	111.3482	139.2154	111.324	148.2865	111.5364	132.6389
111.2079	136.8047	111.3155	129.318	111.4241	132.3865	111.3649	139.2194	111.3406	148.2874	111.553	132.6371
111.2246	136.8045	111.3322	129.3197	111.4407	132.3885	111.3815	139.2193	111.3573	148.287	111.5697	132.6385
111.2413	136.8012	111.3489	129.3177	111.4574	132.3869	111.3982	139.2188	111.374	148.2877	111.5864	132.6367
111.2579	136.7999	111.3655	129.3181	111.4741	132.3862	111.4149	139.2178	111.3906	148.2852	111.603	132.6372
111.2746	136.7992	111.3822	129.3162	111.4907	132.3856	111.4315	139.2172	111.4073	148.2864	111.6197	132.6356
111.2913	136.7987	111.3989	129.3137	111.5074	132.3862	111.4482	139.2172	111.424	148.2888	111.6364	132.6336
111.3079	136.7956	111.4155	129.3143	111.5241	132.3832	111.4649	139.2174	111.4406	148.2896	111.653	132.6341
111.3246	136.7951	111.4322	129.3153	111.5407	132.3862	111.4815	139.2157	111.4573	148.2883	111.6697	132.6314
111.3413	136.7922	111.4489	129.3165	111.5574	132.3847	111.4982	139.215	111.474	148.2886	111.6864	132.6325
111.3579	136.7919	111.4655	129.3152	111.5741	132.3843	111.5149	139.2168	111.4906	148.2881	111.703	132.6323
111.3746	136.796	111.4822	129.3147	111.5907	132.3854	111.5315	139.2146	111.5073	148.2894	111.7197	132.6296
111.3913	136.7909	111.4989	129.3147	111.6074	132.3832	111.5482	139.216	111.524	148.2885	111.7364	132.6318

111.4079	136.7913	111.5155	129.311	111.6241	132.3861	111.5649	139.2134	111.5406	148.2868	111.753	132.6288
111.4246	136.7887	111.5322	129.3115	111.6407	132.3865	111.5815	139.2127	111.5573	148.2867	111.7697	132.63
111.4413	136.7859	111.5489	129.3129	111.6574	132.3811	111.5982	139.2122	111.574	148.2859	111.7864	132.6289
111.4579	136.7853	111.5655	129.3051	111.6741	132.3858	111.6149	139.2112	111.5906	148.2863	111.803	132.6257
111.4746	136.7879	111.5822	129.3074	111.6907	132.3833	111.6315	139.2114	111.6073	148.287	111.8197	132.6257
111.4913	136.7847	111.5989	129.3072	111.7074	132.3827	111.6482	139.211	111.624	148.2858	111.8364	132.6262
111.5079	136.7811	111.6155	129.3045	111.7241	132.3838	111.6649	139.2118	111.6406	148.284	111.853	132.6242
111.5246	136.7818	111.6322	129.3028	111.7407	132.3821	111.6815	139.2099	111.6573	148.2876	111.8697	132.625
111.5413	136.7786	111.6489	129.3056	111.7574	132.3822	111.6982	139.2102	111.674	148.2838	111.8864	132.6248
111.5579	136.7781	111.6655	129.3053	111.7741	132.3818	111.7149	139.2095	111.6906	148.2832	111.903	132.6217
111.5746	136.7785	111.6822	129.3044	111.7907	132.3796	111.7315	139.2091	111.7073	148.2813	111.9197	132.6217
111.5913	136.776	111.6989	129.2993	111.8074	132.3818	111.7482	139.2058	111.724	148.2861	111.9364	132.6222
111.6079	136.7747	111.7155	129.3034	111.8241	132.3823	111.7649	139.2069	111.7406	148.2853	111.953	132.6205
111.6246	136.7769	111.7322	129.3045	111.8407	132.3817	111.7815	139.207	111.7573	148.2848	111.9697	132.6212
111.6413	136.772	111.7489	129.3009	111.8574	132.3782	111.7982	139.206	111.774	148.2834	111.9864	132.6218
111.6579	136.7721	111.7655	129.2977	111.8741	132.3807	111.8149	139.2053	111.7906	148.2817	112.003	132.6203
111.6746	136.7727	111.7822	129.3025	111.8907	132.3761	111.8315	139.2057	111.8073	148.2822	112.0197	132.6189
111.6913	136.77	111.7989	129.3	111.9074	132.3793	111.8482	139.2019	111.824	148.2809	112.0364	132.6171
111.7079	136.77	111.8155	129.3005	111.9241	132.3749	111.8649	139.2051	111.8406	148.2811	112.053	132.62
111.7246	136.767	111.8322	129.297	111.9407	132.3772	111.8815	139.205	111.8573	148.28	112.0697	132.6164
111.7413	136.7663	111.8489	129.2984	111.9574	132.3799	111.8982	139.2013	111.874	148.283	112.0864	132.6179
111.7579	136.7646	111.8655	129.2985	111.9741	132.3779	111.9149	139.2031	111.8906	148.2789	112.103	132.6177
111.7746	136.7652	111.8822	129.2964	111.9907	132.3772	111.9315	139.1997	111.9073	148.2801	112.1197	132.6144
111.7913	136.7631	111.8989	129.2964	112.0074	132.3769	111.9482	139.2009	111.924	148.2824	112.1364	132.617
111.8079	136.7616	111.9155	129.2941	112.0241	132.375	111.9649	139.1995	111.9406	148.2764	112.153	132.6146
111.8246	136.7585	111.9322	129.2941	112.0407	132.3752	111.9815	139.1986	111.9573	148.2757	112.1697	132.6144
111.8413	136.758	111.9489	129.2937	112.0574	132.3762	111.9982	139.1999	111.974	148.2794	112.1864	132.6142
111.8579	136.7595	111.9655	129.2919	112.0741	132.3761	112.0149	139.1961	111.9906	148.2758	112.203	132.6134
111.8746	136.7591	111.9822	129.292	112.0907	132.3754	112.0315	139.1978	112.0073	148.2781	112.2197	132.6106
111.8913	136.7586	111.9989	129.2913	112.1074	132.3746	112.0482	139.198	112.024	148.275	112.2364	132.6108
111.9079	136.7554	112.0155	129.2911	112.1241	132.3758	112.0649	139.1982	112.0406	148.2786	112.253	132.6122
111.9246	136.7566	112.0322	129.2904	112.1407	132.3743	112.0815	139.1978	112.0573	148.2744	112.2697	132.6107
111.9413	136.7556	112.0489	129.2913	112.1574	132.3731	112.0982	139.1952	112.074	148.2771	112.2864	132.6089
111.9579	136.754	112.0655	129.2928	112.1741	132.3728	112.1149	139.1931	112.0906	148.2793	112.303	132.6112
111.9746	136.7568	112.0822	129.2917	112.1907	132.3725	112.1315	139.1926	112.1073	148.2807	112.3197	132.6092
111.9913	136.7493	112.0989	129.2922	112.2074	132.3734	112.1482	139.1931	112.124	148.279	112.3364	132.6087
112.0079	136.7496	112.1155	129.2905	112.2241	132.3733	112.1649	139.1932	112.1406	148.279	112.353	132.61
112.0246	136.7496	112.1322	129.2883	112.2407	132.374	112.1815	139.1903	112.1573	148.2784	112.3697	132.6064
112.0413	136.7459	112.1489	129.2862	112.2574	132.3693	112.1982	139.1886	112.174	148.2792	112.3864	132.6074
112.0579	136.7457	112.1655	129.2889	112.2741	132.3721	112.2149	139.19	112.1906	148.2792	112.403	132.6075
112.0746	136.7453	112.1822	129.2866	112.2907	132.3711	112.2315	139.1906	112.2073	148.2787	112.4197	132.6056
112.0913	136.7463	112.1989	129.2866	112.3074	132.3704	112.2482	139.1888	112.224	148.2772	112.4364	132.6059
112.1079	136.7433	112.2155	129.2884	112.3241	132.3703	112.2649	139.1899	112.2406	148.2771	112.453	132.6053
112.1246	136.7413	112.2322	129.2861	112.3407	132.3697	112.2815	139.1887	112.2573	148.2778	112.4697	132.6054
112.1413	136.7402	112.2489	129.2839	112.3574	132.3705	112.2982	139.1893	112.274	148.2764	112.4864	132.6049
112.1579	136.7414	112.2655	129.2826	112.3741	132.3706	112.3149	139.1895	112.2906	148.2771	112.503	132.6033
112.1746	136.7404	112.2822	129.2809	112.3907	132.3671	112.3315	139.1874	112.3073	148.277	112.5197	132.602
112.1913	136.7389	112.2989	129.2808	112.4074	132.3706	112.3482	139.1896	112.324	148.2758	112.5364	132.6041
112.2079	136.7409	112.3155	129.2811	112.4241	132.3692	112.3649	139.1873	112.3406	148.2751	112.553	132.6004
112.2246	136.738	112.3322	129.2789	112.4407	132.3692	112.3815	139.1861	112.3573	148.2744	112.5697	132.6011
112.2413	136.7343	112.3489	129.2811	112.4574	132.3677	112.3982	139.1885	112.374	148.2751	112.5864	132.6017
112.2579	136.7331	112.3655	129.2825	112.4741	132.3679	112.4149	139.1859	112.3906	148.2758	112.603	132.5998
112.2746	136.7326	112.3822	129.2781	112.4907	132.3663	112.4315	139.1861	112.4073	148.2735	112.6197	132.5987
112.2913	136.7339	112.3989	129.2804	112.5074	132.3685	112.4482	139.1862	112.424	148.2736	112.6364	132.5988
112.3079	136.7303	112.4155	129.2789	112.5241	132.3683	112.4649	139.1821	112.4406	148.2711	112.653	132.5971
112.3246	136.7285	112.4322	129.2769	112.5407	132.3701	112.4815	139.1847	112.4573	148.2729	112.6697	132.5971
112.3413	136.7305	112.4489	129.2766	112.5574	132.3688	112.4982	139.1841	112.474	148.2736	112.6864	132.5956
112.3579	136.7264	112.4655	129.2768	112.5741	132.3697	112.5149	139.1823	112.4906	148.2714	112.703	132.596
112.3746	136.7251	112.4822	129.2761	112.5907	132.3684	112.5315	139.1842	112.5073	148.2705	112.7197	132.5938
112.3913	136.7326	112.4989	129.2738	112.6074	132.3673	112.5482	139.1852	112.524	148.2698	112.7364	132.5943
112.4079	136.7291	112.5155	129.2783	112.6241	132.3652	112.5649	139.184	112.5406	148.2711	112.753	132.5936
112.4246	136.7221	112.5322	129.2729	112.6407	132.3658	112.5815	139.1868	112.5573	148.2698	112.7697	132.5937
112.4413	136.7214	112.5489	129.2719	112.6574	132.3682	112.5982	139.1868	112.574	148.2709	112.7864	132.5921
112.4579	136.7234	112.5655	129.2734	112.6741	132.3643	112.6149	139.1852	112.5906	148.2691	112.803	132.5932
112.4746	136.7232	112.5822	129.2746	112.6907	132.364	112.6315	139.1836	112.6073	148.2683	112.8197	132.5926
112.4913	136.7201	112.5989	129.2729	112.7074	132.3623	112.6482	139.1835	112.624	148.2676	112.8364	132.5911
112.5079	136.7189	112.6155	129.2708	112.7241	132.3634	112.6649	139.1863	112.6406	148.2679	112.853	132.591
112.5246	136.7181	112.6322	129.2705	112.7407	132.3635	112.6815	139.183	112.6573	148.2689	112.8697	132.5903
112.5413	136.7151	112.6489	129.2728	112.7574	132.3633	112.6982	139.1822	112.674	148.2679	112.8864	132.5874

112.5579	136.7152	112.6655	129.2702	112.7741	132.3631	112.7149	139.1821	112.6906	148.2653	112.903	132.5892
112.5746	136.7125	112.6822	129.2705	112.7907	132.362	112.7315	139.1825	112.7073	148.2644	112.9197	132.5878
112.5913	136.7139	112.6989	129.2641	112.8074	132.3601	112.7482	139.1811	112.724	148.265	112.9364	132.5882
112.6079	136.7138	112.7155	129.2683	112.8241	132.3605	112.7649	139.1803	112.7406	148.2656	112.953	132.5885
112.6246	136.7094	112.7322	129.269	112.8407	132.3625	112.7815	139.1788	112.7573	148.2656	112.9697	132.5879
112.6413	136.7097	112.7489	129.2676	112.8574	132.3607	112.7982	139.1782	112.774	148.2661	112.9864	132.5862
112.6579	136.7067	112.7655	129.2679	112.8741	132.3637	112.8149	139.1794	112.7906	148.266	113.003	132.5854
112.6746	136.7065	112.7822	129.2685	112.8907	132.3584	112.8315	139.1748	112.8073	148.2681	113.0197	132.5847
112.6913	136.7041	112.7989	129.2707	112.9074	132.3602	112.8482	139.1769	112.824	148.2664	113.0364	132.5859
112.7079	136.7046	112.8155	129.2669	112.9241	132.36	112.8649	139.1754	112.8406	148.2653	113.053	132.5835
112.7246	136.7033	112.8322	129.2689	112.9407	132.3614	112.8815	139.1743	112.8573	148.2677	113.0697	132.5844
112.7413	136.7043	112.8489	129.2679	112.9574	132.3606	112.8982	139.1737	112.874	148.2666	113.0864	132.5823
112.7579	136.7021	112.8655	129.2649	112.9741	132.3597	112.9149	139.171	112.8906	148.2638	113.103	132.5851
112.7746	136.7	112.8822	129.2647	112.9907	132.3597	112.9315	139.1725	112.9073	148.2622	113.1197	132.5813
112.7913	136.6984	112.8989	129.2642	113.0074	132.3619	112.9482	139.1706	112.924	148.2642	113.1364	132.5833
112.8079	136.6977	112.9155	129.263	113.0241	132.3599	112.9649	139.1706	112.9406	148.2655	113.153	132.5818
112.8246	136.6971	112.9322	129.2626	113.0407	132.3597	112.9815	139.17	112.9573	148.2636	113.1697	132.5798
112.8413	136.6967	112.9489	129.2626	113.0574	132.3605	112.9982	139.1687	112.974	148.2608	113.1864	132.5794
112.8579	136.6951	112.9655	129.2617	113.0741	132.363	113.0149	139.1707	112.9906	148.2621	113.203	132.5814
112.8746	136.6941	112.9822	129.2581	113.0907	132.3583	113.0315	139.1702	113.0073	148.2604	113.2197	132.5781
112.8913	136.6959	112.9989	129.2575	113.1074	132.3584	113.0482	139.1674	113.024	148.2613	113.2364	132.5767
112.9079	136.6901	113.0155	129.258	113.1241	132.3591	113.0649	139.1661	113.0406	148.2629	113.253	132.5777
112.9246	136.6895	113.0322	129.2566	113.1407	132.3568	113.0815	139.1653	113.0573	148.262	113.2697	132.5776
112.9413	136.6895	113.0489	129.257	113.1574	132.355	113.0982	139.1651	113.074	148.2601	113.2864	132.5742
112.9579	136.6907	113.0655	129.257	113.1741	132.3574	113.1149	139.1653	113.0906	148.2631	113.303	132.5752
112.9746	136.6873	113.0822	129.2575	113.1907	132.3576	113.1315	139.165	113.1073	148.2626	113.3197	132.575
112.9913	136.686	113.0989	129.2584	113.2074	132.3569	113.1482	139.1623	113.124	148.2621	113.3364	132.5744
113.0079	136.6872	113.1155	129.2554	113.2241	132.3535	113.1649	139.1634	113.1406	148.2617	113.353	132.5735
113.0246	136.6856	113.1322	129.2563	113.2407	132.3566	113.1815	139.1623	113.1573	148.264	113.3697	132.5753
113.0413	136.6852	113.1489	129.255	113.2574	132.3518	113.1982	139.1616	113.174	148.2609	113.3864	132.5729
113.0579	136.6856	113.1655	129.2531	113.2741	132.3544	113.2149	139.1605	113.1906	148.2615	113.403	132.5721
113.0746	136.6822	113.1822	129.2543	113.2907	132.3532	113.2315	139.161	113.2073	148.2598	113.4197	132.5733
113.0913	136.6852	113.1989	129.2543	113.3074	132.3527	113.2482	139.157	113.224	148.2618	113.4364	132.5721
113.1079	136.6818	113.2155	129.2523	113.3241	132.3473	113.2649	139.16	113.2406	148.2586	113.453	132.5684
113.1246	136.6836	113.2322	129.2483	113.3407	132.3488	113.2815	139.1585	113.2573	148.2575	113.4697	132.5712
113.1413	136.6797	113.2489	129.249	113.3574	132.3485	113.2982	139.1584	113.274	148.2594	113.4864	132.5716
113.1579	136.6755	113.2655	129.2517	113.3741	132.3507	113.3149	139.1568	113.2906	148.2593	113.503	132.5714
113.1746	136.6806	113.2822	129.2495	113.3907	132.3473	113.3315	139.1574	113.3073	148.2586	113.5197	132.5668
113.1913	136.676	113.2989	129.2516	113.4074	132.3489	113.3482	139.1571	113.324	148.2568	113.5364	132.57
113.2079	136.6739	113.3155	129.2489	113.4241	132.3483	113.3649	139.1569	113.3406	148.2573	113.553	132.5662
113.2246	136.6739	113.3322	129.2484	113.4407	132.3418	113.3815	139.155	113.3573	148.258	113.5697	132.5659
113.2413	136.6724	113.3489	129.2463	113.4574	132.3424	113.3982	139.1522	113.374	148.2586	113.5864	132.5652
113.2579	136.6731	113.3655	129.2468	113.4741	132.341	113.4149	139.1534	113.3906	148.2596	113.603	132.5645
113.2746	136.6702	113.3822	129.2501	113.4907	132.3457	113.4315	139.1551	113.4073	148.2545	113.6197	132.5648
113.2913	136.6696	113.3989	129.2446	113.5074	132.3359	113.4482	139.1521	113.424	148.2564	113.6364	132.5634
113.3079	136.6693	113.4155	129.2447	113.5241	132.3411	113.4649	139.153	113.4406	148.2591	113.653	132.5622
113.3246	136.664	113.4322	129.2442	113.5407	132.3404	113.4815	139.1513	113.4573	148.257	113.6697	132.5621
113.3413	136.6643	113.4489	129.2411	113.5574	132.3399	113.4982	139.1507	113.474	148.2564	113.6864	132.5608
113.3579	136.6644	113.4655	129.2429	113.5741	132.3442	113.5149	139.1501	113.4906	148.2555	113.703	132.5617
113.3746	136.6624	113.4822	129.2442	113.5907	132.3387	113.5315	139.1485	113.5073	148.255	113.7197	132.559
113.3913	136.6622	113.4989	129.2403	113.6074	132.3417	113.5482	139.1466	113.524	148.2551	113.7364	132.5591
113.4079	136.6618	113.5155	129.2401	113.6241	132.3381	113.5649	139.149	113.5406	148.2597	113.753	132.5596
113.4246	136.6597	113.5322	129.2413	113.6407	132.3389	113.5815	139.1483	113.5573	148.2585	113.7697	132.5584
113.4413	136.661	113.5489	129.2382	113.6574	132.336	113.5982	139.1481	113.574	148.2563	113.7864	132.5578
113.4579	136.6567	113.5655	129.2406	113.6741	132.3383	113.6149	139.1456	113.5906	148.2546	113.803	132.5567
113.4746	136.6578	113.5822	129.2358	113.6907	132.3351	113.6315	139.1466	113.6073	148.2543	113.8197	132.5568
113.4913	136.659	113.5989	129.2353	113.7074	132.3368	113.6482	139.145	113.624	148.2542	113.8364	132.5548
113.5079	136.655	113.6155	129.2355	113.7241	132.3365	113.6649	139.1434	113.6406	148.2533	113.853	132.5551
113.5246	136.6533	113.6322	129.235	113.7407	132.3364	113.6815	139.1441	113.6573	148.2541	113.8697	132.5544
113.5413	136.6536	113.6489	129.2338	113.7574	132.3339	113.6982	139.1415	113.674	148.2519	113.8864	132.552
113.5579	136.6499	113.6655	129.2341	113.7741	132.3357	113.7149	139.144	113.6906	148.2507	113.903	132.5544
113.5746	136.6484	113.6822	129.2294	113.7907	132.3356	113.7315	139.1434	113.7073	148.2501	113.9197	132.5509
113.5913	136.6482	113.6989	129.2312	113.8074	132.3354	113.7482	139.1437	113.724	148.2509	113.9364	132.55
113.6079	136.6475	113.7155	129.2313	113.8241	132.3352	113.7649	139.1418	113.7406	148.2517	113.953	132.5509
113.6246	136.6476	113.7322	129.2295	113.8407	132.3359	113.7815	139.1435	113.7573	148.2538	113.9697	132.5501
113.6413	136.6507	113.7489	129.2281	113.8574	132.3328	113.7982	139.1409	113.774	148.2488	113.9864	132.5479
113.6579	136.6461	113.7655	129.2311	113.8741	132.3327	113.8149	139.1409	113.7906	148.2523	114.003	132.5483
113.6746	136.6445	113.7822	129.2294	113.8907	132.33	113.8315	139.1425	113.8073	148.2509	114.0197	132.5446
113.6913	136.6436	113.7989	129.2274	113.9074	132.3303	113.8482	139.1378	113.824	148.2508	114.0364	132.5486

113.7079	136.6401	113.8155	129.2268	113.9241	132.3316	113.8649	139.1421	113.8406	148.2524	114.053	132.5466
113.7246	136.6409	113.8322	129.2303	113.9407	132.3326	113.8815	139.1392	113.8573	148.2503	114.0697	132.5456
113.7413	136.649	113.8489	129.2236	113.9574	132.3319	113.8982	139.14	113.874	148.2507	114.0864	132.5415
113.7579	136.6405	113.8655	129.2224	113.9741	132.3272	113.9149	139.1396	113.8906	148.2511	114.103	132.5458
113.7746	136.6396	113.8822	129.2241	113.9907	132.3319	113.9315	139.1364	113.9073	148.2479	114.1197	132.5444
113.7913	136.6384	113.8989	129.2243	114.0074	132.3312	113.9482	139.1372	113.924	148.2486	114.1364	132.5437
113.8079	136.6366	113.9155	129.221	114.0241	132.3297	113.9649	139.1392	113.9406	148.2486	114.153	132.5422
113.8246	136.6382	113.9322	129.2154	114.0407	132.3279	113.9815	139.137	113.9573	148.2511	114.1697	132.545
113.8413	136.6359	113.9489	129.2187	114.0574	132.3298	113.9982	139.1363	113.974	148.2483	114.1864	132.5415
113.8579	136.6349	113.9655	129.2212	114.0741	132.3297	114.0149	139.1367	113.9906	148.2495	114.203	132.5432
113.8746	136.6343	113.9822	129.2201	114.0907	132.3262	114.0315	139.1346	114.0073	148.2436	114.2197	132.5402
113.8913	136.6374	113.9989	129.2162	114.1074	132.3252	114.0482	139.1354	114.024	148.2468	114.2364	132.5406
113.9079	136.6313	114.0155	129.2218	114.1241	132.3262	114.0649	139.1363	114.0406	148.252	114.253	132.5386
113.9246	136.6293	114.0322	129.2177	114.1407	132.3237	114.0815	139.1347	114.0573	148.2459	114.2697	132.5426
113.9413	136.6284	114.0489	129.2189	114.1574	132.3204	114.0982	139.1342	114.074	148.252	114.2864	132.5377
113.9579	136.6275	114.0655	129.2173	114.1741	132.3204	114.1149	139.1346	114.0906	148.2481	114.303	132.5423
113.9746	136.6269	114.0822	129.2175	114.1907	132.3233	114.1315	139.1315	114.1073	148.2469	114.3197	132.541
113.9913	136.6277	114.0989	129.2162	114.2074	132.322	114.1482	139.1295	114.124	148.2457	114.3364	132.54
114.0079	136.6257	114.1155	129.2153	114.2241	132.3192	114.1649	139.1352	114.1406	148.249	114.353	132.5378
114.0246	136.6246	114.1322	129.2147	114.2407	132.3142	114.1815	139.1326	114.1573	148.2441	114.3697	132.5374
114.0413	136.6225	114.1489	129.2123	114.2574	132.3214	114.1982	139.1325	114.174	148.2448	114.3864	132.5385
114.0579	136.6222	114.1655	129.2145	114.2741	132.3155	114.2149	139.1303	114.1906	148.2444	114.403	132.5366
114.0746	136.621	114.1822	129.2095	114.2907	132.3175	114.2315	139.1308	114.2073	148.243	114.4197	132.537
114.0913	136.6187	114.1989	129.2095	114.3074	132.3174	114.2482	139.1316	114.224	148.2418	114.4364	132.539
114.1079	136.6183	114.2155	129.2112	114.3241	132.3177	114.2649	139.1313	114.2406	148.2439	114.453	132.5333
114.1246	136.6178	114.2322	129.2126	114.3407	132.3142	114.2815	139.129	114.2573	148.2424	114.4697	132.5372
114.1413	136.6177	114.2489	129.2059	114.3574	132.3166	114.2982	139.1271	114.274	148.2419	114.4864	132.5332
114.1579	136.6154	114.2655	129.2083	114.3741	132.3157	114.3149	139.1278	114.2906	148.2432	114.503	132.5352
114.1746	136.6151	114.2822	129.2054	114.3907	132.3154	114.3315	139.1269	114.3073	148.2446	114.5197	132.5336
114.1913	136.6132	114.2989	129.2073	114.4074	132.3198	114.3482	139.1289	114.324	148.2432	114.5364	132.5344
114.2079	136.6143	114.3155	129.2059	114.4241	132.3147	114.3649	139.127	114.3406	148.2438	114.553	132.5342
114.2246	136.6115	114.3322	129.2057	114.4407	132.3126	114.3815	139.1258	114.3573	148.2437	114.5697	132.5337
114.2413	136.6088	114.3489	129.2043	114.4574	132.3153	114.3982	139.1264	114.374	148.2436	114.5864	132.5343
114.2579	136.609	114.3655	129.2043	114.4741	132.3137	114.4149	139.1234	114.3906	148.2431	114.603	132.5328
114.2746	136.606	114.3822	129.2035	114.4907	132.3151	114.4315	139.124	114.4073	148.2428	114.6197	132.5313
114.2913	136.6067	114.3989	129.202	114.5074	132.3165	114.4482	139.1208	114.424	148.2421	114.6364	132.5336
114.3079	136.6065	114.4155	129.2038	114.5241	132.316	114.4649	139.1233	114.4406	148.2412	114.653	132.5329
114.3246	136.6056	114.4322	129.203	114.5407	132.311	114.4815	139.1229	114.4573	148.2407	114.6697	132.5327
114.3413	136.6	114.4489	129.2028	114.5574	132.3119	114.4982	139.1223	114.474	148.2417	114.6864	132.5309
114.3579	136.6014	114.4655	129.201	114.5741	132.3129	114.5149	139.1183	114.4906	148.2412	114.703	132.5299
114.3746	136.6047	114.4822	129.1997	114.5907	132.3097	114.5315	139.1204	114.5073	148.24	114.7197	132.5286
114.3913	136.6019	114.4989	129.2003	114.6074	132.309	114.5482	139.1209	114.524	148.2408	114.7364	132.5304
114.4079	136.5998	114.5155	129.2001	114.6241	132.3092	114.5649	139.1182	114.5406	148.2406	114.753	132.5288
114.4246	136.5961	114.5322	129.1999	114.6407	132.3093	114.5815	139.1164	114.5573	148.2392	114.7697	132.5298
114.4413	136.5951	114.5489	129.1971	114.6574	132.3095	114.5982	139.1169	114.574	148.238	114.7864	132.5269
114.4579	136.5932	114.5655	129.1968	114.6741	132.3104	114.6149	139.1147	114.5906	148.2385	114.803	132.528
114.4746	136.5937	114.5822	129.1946	114.6907	132.3094	114.6315	139.1161	114.6073	148.2392	114.8197	132.528
114.4913	136.5912	114.5989	129.1943	114.7074	132.3072	114.6482	139.1139	114.624	148.2392	114.8364	132.5265
114.5079	136.5902	114.6155	129.1948	114.7241	132.3074	114.6649	139.1159	114.6406	148.2366	114.853	132.5259
114.5246	136.5912	114.6322	129.1971	114.7407	132.3076	114.6815	139.1141	114.6573	148.2361	114.8697	132.5256
114.5413	136.5905	114.6489	129.193	114.7574	132.3071	114.6982	139.1163	114.674	148.2366	114.8864	132.5237
114.5579	136.591	114.6655	129.1918	114.7741	132.3057	114.7149	139.1119	114.6906	148.2353	114.903	132.5231
114.5746	136.5865	114.6822	129.1912	114.7907	132.3088	114.7315	139.1155	114.7073	148.2374	114.9197	132.5222
114.5913	136.5863	114.6989	129.1919	114.8074	132.3077	114.7482	139.1133	114.724	148.2356	114.9364	132.5232
114.6079	136.5893	114.7155	129.1914	114.8241	132.3033	114.7649	139.1133	114.7406	148.2371	114.953	132.5221
114.6246	136.5822	114.7322	129.1929	114.8407	132.3052	114.7815	139.1141	114.7573	148.2357	114.9697	132.5235
114.6413	136.5824	114.7489	129.19	114.8574	132.3035	114.7982	139.1108	114.774	148.2371	114.9864	132.5216
114.6579	136.5825	114.7655	129.1893	114.8741	132.3044	114.8149	139.1117	114.7906	148.2377	115.003	132.5224
114.6746	136.5786	114.7822	129.1901	114.8907	132.3032	114.8315	139.1114	114.8073	148.2358	115.0197	132.5194
114.6913	136.5811	114.7989	129.1891	114.9074	132.3042	114.8482	139.1115	114.824	148.2361	115.0364	132.5197
114.7079	136.5814	114.8155	129.1881	114.9241	132.2998	114.8649	139.1101	114.8406	148.236	115.053	132.5172
114.7246	136.5761	114.8322	129.1859	114.9407	132.2998	114.8815	139.1088	114.8573	148.2361	115.0697	132.5187
114.7413	136.5777	114.8489	129.1862	114.9574	132.3014	114.8982	139.1102	114.874	148.2354	115.0864	132.516
114.7579	136.5784	114.8655	129.1838	114.9741	132.3006	114.9149	139.1083	114.8906	148.2355	115.103	132.5173
114.7746	136.5727	114.8822	129.1848	114.9907	132.3014	114.9315	139.1105	114.9073	148.2339	115.1197	132.5164
114.7913	136.5735	114.8989	129.1839	115.0074	132.2991	114.9482	139.1055	114.924	148.2341	115.1364	132.5162
114.8079	136.573	114.9155	129.1806	115.0241	132.2977	114.9649	139.1077	114.9406	148.2345	115.153	132.5142
114.8246	136.5712	114.9322	129.1841	115.0407	132.2975	114.9815	139.1069	114.9573	148.2338	115.1697	132.5153
114.8413	136.5691	114.9489	129.182	115.0574	132.2991	114.9982	139.1062	114.974	148.2338	115.1864	132.5142

114.8579	136.5662	114.9655	129.1779	115.0741	132.3015	115.0149	139.1083	114.9906	148.2328	115.203	132.512
114.8746	136.569	114.9822	129.18	115.0907	132.3001	115.0315	139.1048	115.0073	148.2321	115.2197	132.5113
114.8913	136.5704	114.9989	129.1768	115.1074	132.2988	115.0482	139.1072	115.024	148.2342	115.2364	132.5119
114.9079	136.5666	115.0155	129.1774	115.1241	132.2968	115.0649	139.1054	115.0406	148.2317	115.253	132.5142
114.9246	136.5671	115.0322	129.1779	115.1407	132.2986	115.0815	139.1065	115.0573	148.2327	115.2697	132.5094
114.9413	136.5675	115.0489	129.1786	115.1574	132.2984	115.0982	139.1063	115.074	148.2327	115.2864	132.5122
114.9579	136.5612	115.0655	129.1776	115.1741	132.2981	115.1149	139.1047	115.0906	148.2297	115.303	132.512
114.9746	136.5639	115.0822	129.1745	115.1907	132.2998	115.1315	139.1055	115.1073	148.2319	115.3197	132.5092
114.9913	136.56	115.0989	129.1768	115.2074	132.2977	115.1482	139.1036	115.124	148.2284	115.3364	132.5102
115.0079	136.5569	115.1155	129.1746	115.2241	132.2968	115.1649	139.1026	115.1406	148.2317	115.353	132.5079
115.0246	136.5594	115.1322	129.1766	115.2407	132.2966	115.1815	139.1031	115.1573	148.2309	115.3697	132.5077
115.0413	136.5586	115.1489	129.1742	115.2574	132.2988	115.1982	139.1052	115.174	148.2308	115.3864	132.5065
115.0579	136.5552	115.1655	129.1732	115.2741	132.2979	115.2149	139.1029	115.1906	148.233	115.403	132.5063
115.0746	136.5595	115.1822	129.1718	115.2907	132.2972	115.2315	139.1032	115.2073	148.2318	115.4197	132.5045
115.0913	136.5573	115.1989	129.1705	115.3074	132.2945	115.2482	139.1027	115.224	148.2301	115.4364	132.5034
115.1079	136.5554	115.2155	129.1712	115.3241	132.2941	115.2649	139.1027	115.2406	148.2298	115.453	132.5046
115.1246	136.5547	115.2322	129.1733	115.3407	132.2948	115.2815	139.1029	115.2573	148.2283	115.4697	132.5023
115.1413	136.5532	115.2489	129.1683	115.3574	132.2992	115.2982	139.1029	115.274	148.2292	115.4864	132.5035
115.1579	136.5535	115.2655	129.1701	115.3741	132.2948	115.3149	139.0985	115.2906	148.2292	115.503	132.5011
115.1746	136.55	115.2822	129.1683	115.3907	132.2949	115.3315	139.101	115.3073	148.2264	115.5197	132.5017
115.1913	136.5527	115.2989	129.1693	115.4074	132.2969	115.3482	139.0992	115.324	148.2262	115.5364	132.5002
115.2079	136.5478	115.3155	129.1686	115.4241	132.2946	115.3649	139.0996	115.3406	148.2284	115.553	132.5001
115.2246	136.5502	115.3322	129.1698	115.4407	132.298	115.3815	139.0963	115.3573	148.2255	115.5697	132.4987
115.2413	136.5501	115.3489	129.166	115.4574	132.296	115.3982	139.0965	115.374	148.226	115.5864	132.4955
115.2579	136.5482	115.3655	129.1648	115.4741	132.2936	115.4149	139.0988	115.3906	148.2263	115.603	132.4991
115.2746	136.5477	115.3822	129.1658	115.4907	132.2926	115.4315	139.098	115.4073	148.2252	115.6197	132.4992
115.2913	136.5462	115.3989	129.164	115.5074	132.2933	115.4482	139.0971	115.424	148.2248	115.6364	132.4971
115.3079	136.5452	115.4155	129.1644	115.5241	132.2938	115.4649	139.0937	115.4406	148.2256	115.653	132.4961
115.3246	136.5434	115.4322	129.1655	115.5407	132.2933	115.4815	139.0957	115.4573	148.2251	115.6697	132.4956
115.3413	136.5433	115.4489	129.1632	115.5574	132.2925	115.4982	139.0977	115.474	148.2259	115.6864	132.4958
115.3579	136.5454	115.4655	129.1611	115.5741	132.2941	115.5149	139.0961	115.4906	148.2248	115.703	132.4949
115.3746	136.5383	115.4822	129.1617	115.5907	132.2913	115.5315	139.0959	115.5073	148.2257	115.7197	132.4938
115.3913	136.5416	115.4989	129.1606	115.6074	132.2906	115.5482	139.0952	115.524	148.2254	115.7364	132.4942
115.4079	136.5428	115.5155	129.157	115.6241	132.2913	115.5649	139.0945	115.5406	148.2245	115.753	132.4927
115.4246	136.5355	115.5322	129.1597	115.6407	132.2898	115.5815	139.093	115.5573	148.2242	115.7697	132.493
115.4413	136.5373	115.5489	129.157	115.6574	132.2887	115.5982	139.0936	115.574	148.2251	115.7864	132.4921
115.4579	136.5351	115.5655	129.1591	115.6741	132.2886	115.6149	139.0922	115.5906	148.2232	115.803	132.4935
115.4746	136.5346	115.5822	129.1556	115.6907	132.2893	115.6315	139.0949	115.6073	148.2223	115.8197	132.4917
115.4913	136.5326	115.5989	129.1604	115.7074	132.2881	115.6482	139.0935	115.624	148.2226	115.8364	132.4909
115.5079	136.5288	115.6155	129.1573	115.7241	132.2901	115.6649	139.0924	115.6406	148.2247	115.853	132.4911
115.5246	136.5291	115.6322	129.1572	115.7407	132.2917	115.6815	139.0916	115.6573	148.2209	115.8697	132.4906
115.5413	136.5308	115.6489	129.1537	115.7574	132.2873	115.6982	139.0906	115.674	148.2202	115.8864	132.4907
115.5579	136.5282	115.6655	129.1547	115.7741	132.2878	115.7149	139.0908	115.6906	148.2206	115.903	132.4907
115.5746	136.5283	115.6822	129.1533	115.7907	132.2896	115.7315	139.0896	115.7073	148.2204	115.9197	132.4896
115.5913	136.522	115.6989	129.1543	115.8074	132.2873	115.7482	139.0886	115.724	148.2202	115.9364	132.4886
115.6079	136.5284	115.7155	129.1539	115.8241	132.2861	115.7649	139.0888	115.7406	148.2211	115.953	132.4874
115.6246	136.52	115.7322	129.1501	115.8407	132.2884	115.7815	139.0899	115.7573	148.2199	115.9697	132.4875
115.6413	136.5239	115.7489	129.151	115.8574	132.2866	115.7982	139.0895	115.774	148.2205	115.9864	132.4867
115.6579	136.5179	115.7655	129.1518	115.8741	132.2867	115.8149	139.0872	115.7906	148.2195	116.003	132.4869
115.6746	136.5159	115.7822	129.1469	115.8907	132.2856	115.8315	139.0884	115.8073	148.2218	116.0197	132.4872
115.6913	136.521	115.7989	129.1525	115.9074	132.2854	115.8482	139.0871	115.824	148.2193	116.0364	132.4861
115.7079	136.516	115.8155	129.1493	115.9241	132.282	115.8649	139.0876	115.8406	148.2214	116.053	132.4858
115.7246	136.5154	115.8322	129.1503	115.9407	132.2869	115.8815	139.0857	115.8573	148.2195	116.0697	132.4856
115.7413	136.5137	115.8489	129.1498	115.9574	132.2854	115.8982	139.0847	115.874	148.2166	116.0864	132.4836
115.7579	136.5155	115.8655	129.1445	115.9741	132.2855	115.9149	139.0842	115.8906	148.2194	116.103	132.4852
115.7746	136.5146	115.8822	129.1494	115.9907	132.2833	115.9315	139.0857	115.9073	148.2185	116.1197	132.4844
115.7913	136.5147	115.8989	129.1476	116.0074	132.2828	115.9482	139.0862	115.924	148.2207	116.1364	132.483
115.8079	136.5106	115.9155	129.1461	116.0241	132.2834	115.9649	139.0838	115.9406	148.2178	116.153	132.4836
115.8246	136.5109	115.9322	129.1439	116.0407	132.2849	115.9815	139.0832	115.9573	148.218	116.1697	132.4836
115.8413	136.5057	115.9489	129.1422	116.0574	132.2852	115.9982	139.0837	115.974	148.2143	116.1864	132.481
115.8579	136.5052	115.9655	129.1405	116.0741	132.2839	116.0149	139.0822	115.9906	148.2164	116.203	132.4832
115.8746	136.5048	115.9822	129.1434	116.0907	132.2845	116.0315	139.0848	116.0073	148.2171	116.2197	132.4823
115.8913	136.5033	115.9989	129.1397	116.1074	132.2839	116.0482	139.0837	116.024	148.2157	116.2364	132.4817
115.9079	136.5035	116.0155	129.1402	116.1241	132.2824	116.0649	139.0822	116.0406	148.2182	116.253	132.4802
115.9246	136.4991	116.0322	129.1381	116.1407	132.2821	116.0815	139.0828	116.0573	148.2162	116.2697	132.4818
115.9413	136.5019	116.0489	129.1392	116.1574	132.2833	116.0982	139.08	116.074	148.2187	116.2864	132.4805
115.9579	136.5	116.0655	129.1381	116.1741	132.2837	116.1149	139.0804	116.0906	148.2137	116.303	132.4801
115.9746	136.4963	116.0822	129.1366	116.1907	132.2829	116.1315	139.0806	116.1073	148.2134	116.3197	132.4796
115.9913	136.4927	116.0989	129.1362	116.2074	132.2825	116.1482	139.0791	116.124	148.2149	116.3364	132.4799

116.0079	136.4924	116.1155	129.1345	116.2241	132.2826	116.1649	139.082	116.1406	148.2127	116.353	132.4785
116.0246	136.488	116.1322	129.1334	116.2407	132.2836	116.1815	139.0806	116.1573	148.2134	116.3697	132.4764
116.0413	136.4884	116.1489	129.1367	116.2574	132.2826	116.1982	139.0821	116.174	148.2137	116.3864	132.4772
116.0579	136.491	116.1655	129.1353	116.2741	132.2837	116.2149	139.0799	116.1906	148.2156	116.403	132.4774
116.0746	136.4885	116.1822	129.1332	116.2907	132.2836	116.2315	139.0787	116.2073	148.2128	116.4197	132.4764
116.0913	136.485	116.1989	129.1312	116.3074	132.2837	116.2482	139.0791	116.224	148.2142	116.4364	132.4752
116.1079	136.482	116.2155	129.1304	116.3241	132.281	116.2649	139.0774	116.2406	148.216	116.453	132.4742
116.1246	136.4779	116.2322	129.1331	116.3407	132.2807	116.2815	139.0794	116.2573	148.2115	116.4697	132.4746
116.1413	136.4697	116.2489	129.1325	116.3574	132.2804	116.2982	139.0792	116.274	148.2152	116.4864	132.4703
116.1579	136.468	116.2655	129.1292	116.3741	132.2809	116.3149	139.0785	116.2906	148.214	116.503	132.4734
116.1746	136.4654	116.2822	129.1307	116.3907	132.2831	116.3315	139.08	116.3073	148.2118	116.5197	132.4699
116.1913	136.4623	116.2989	129.131	116.4074	132.2829	116.3482	139.0778	116.324	148.2133	116.5364	132.4715
116.2079	136.4636	116.3155	129.1293	116.4241	132.2766	116.3649	139.0768	116.3406	148.2115	116.553	132.4748
116.2246	136.4607	116.3322	129.1294	116.4407	132.2805	116.3815	139.0779	116.3573	148.213	116.5697	132.4721
116.2413	136.459	116.3489	129.1264	116.4574	132.2805	116.3982	139.0773	116.374	148.2125	116.5864	132.4695
116.2579	136.457	116.3655	129.1272	116.4741	132.2791	116.4149	139.0761	116.3906	148.2129	116.603	132.4706
116.2746	136.4557	116.3822	129.1284	116.4907	132.2801	116.4315	139.0765	116.4073	148.2113	116.6197	132.4703
116.2913	136.454	116.3989	129.1245	116.5074	132.2798	116.4482	139.0733	116.424	148.2101	116.6364	132.4703
116.3079	136.4524	116.4155	129.1233	116.5241	132.2786	116.4649	139.0759	116.4406	148.2084	116.653	132.4701
116.3246	136.4526	116.4322	129.1235	116.5407	132.2796	116.4815	139.0724	116.4573	148.2073	116.6697	132.4699
116.3413	136.4504	116.4489	129.1233	116.5574	132.2783	116.4982	139.074	116.474	148.2065	116.6864	132.467
116.3579	136.4508	116.4655	129.1219	116.5741	132.2786	116.5149	139.0728	116.4906	148.2087	116.703	132.4687
116.3746	136.4477	116.4822	129.1236	116.5907	132.2798	116.5315	139.0707	116.5073	148.2086	116.7197	132.4678
116.3913	136.4466	116.4989	129.1231	116.6074	132.2819	116.5482	139.0702	116.524	148.2079	116.7364	132.4653
116.4079	136.4424	116.5155	129.1194	116.6241	132.279	116.5649	139.0704	116.5406	148.2077	116.753	132.4643
116.4246	136.4436	116.5322	129.1208	116.6407	132.2788	116.5815	139.0711	116.5573	148.2081	116.7697	132.4647
116.4413	136.4437	116.5489	129.1204	116.6574	132.2778	116.5982	139.0686	116.574	148.2061	116.7864	132.465
116.4579	136.4413	116.5655	129.1203	116.6741	132.2778	116.6149	139.0677	116.5906	148.2045	116.803	132.464
116.4746	136.4401	116.5822	129.1175	116.6907	132.2789	116.6315	139.0688	116.6073	148.2073	116.8197	132.4636
116.4913	136.4377	116.5989	129.1182	116.7074	132.278	116.6482	139.0688	116.624	148.2057	116.8364	132.4618
116.5079	136.4362	116.6155	129.1159	116.7241	132.2768	116.6649	139.0686	116.6406	148.2028	116.853	132.4619
116.5246	136.4386	116.6322	129.1162	116.7407	132.2787	116.6815	139.0675	116.6573	148.2046	116.8697	132.4623
116.5413	136.4367	116.6489	129.1153	116.7574	132.2785	116.6982	139.0672	116.674	148.203	116.8864	132.4634
116.5579	136.4363	116.6655	129.1146	116.7741	132.2792	116.7149	139.0667	116.6906	148.2032	116.903	132.4638
116.5746	136.4347	116.6822	129.1148	116.7907	132.2775	116.7315	139.0679	116.7073	148.2058	116.9197	132.4603
116.5913	136.4328	116.6989	129.1112	116.8074	132.2784	116.7482	139.0668	116.724	148.2052	116.9364	132.4614
116.6079	136.4314	116.7155	129.1103	116.8241	132.2752	116.7649	139.0673	116.7406	148.1998	116.953	132.458
116.6246	136.4293	116.7322	129.1101	116.8407	132.2778	116.7815	139.0638	116.7573	148.2001	116.9697	132.4606
116.6413	136.4276	116.7489	129.1111	116.8574	132.276	116.7982	139.0653	116.774	148.2039	116.9864	132.4604
116.6579	136.4242	116.7655	129.1109	116.8741	132.2758	116.8149	139.0658	116.7906	148.2001	117.003	132.4588
116.6746	136.4236	116.7822	129.1093	116.8907	132.2766	116.8315	139.0664	116.8073	148.1998	117.0197	132.4586
116.6913	136.4207	116.7989	129.1096	116.9074	132.2761	116.8482	139.0639	116.824	148.2009	117.0364	132.4571
116.7079	136.4202	116.8155	129.1091	116.9241	132.2745	116.8649	139.0635	116.8406	148.1986	117.053	132.4549
116.7246	136.419	116.8322	129.1065	116.9407	132.2743	116.8815	139.0622	116.8573	148.1979	117.0697	132.4581
116.7413	136.4181	116.8489	129.1066	116.9574	132.2743	116.8982	139.0614	116.874	148.2008	117.0864	132.4537
116.7579	136.4147	116.8655	129.1058	116.9741	132.2723	116.9149	139.0639	116.8906	148.1997	117.103	132.4543
116.7746	136.4141	116.8822	129.1068	116.9907	132.2724	116.9315	139.0628	116.9073	148.199	117.1197	132.4566
116.7913	136.4133	116.8989	129.1061	117.0074	132.2722	116.9482	139.062	116.924	148.2013	117.1364	132.4569
116.8079	136.4121	116.9155	129.1006	117.0241	132.2724	116.9649	139.062	116.9406	148.2004	117.153	132.4549
116.8246	136.4062	116.9322	129.1033	117.0407	132.2689	116.9815	139.062	116.9573	148.1978	117.1697	132.454
116.8413	136.4091	116.9489	129.1039	117.0574	132.27	116.9982	139.06	116.974	148.1979	117.1864	132.452
116.8579	136.4064	116.9655	129.1018	117.0741	132.2691	117.0149	139.0624	116.9906	148.1976	117.203	132.4539
116.8746	136.4069	116.9822	129.1001	117.0907	132.2688	117.0315	139.0624	117.0073	148.1988	117.2197	132.4533
116.8913	136.4054	116.9989	129.1032	117.1074	132.2666	117.0482	139.0595	117.024	148.1985	117.2364	132.4539
116.9079	136.4051	117.0155	129.0986	117.1241	132.2666	117.0649	139.0586	117.0406	148.1987	117.253	132.4527
116.9246	136.4058	117.0322	129.0972	117.1407	132.2665	117.0815	139.0556	117.0573	148.1951	117.2697	132.4504
116.9413	136.401	117.0489	129.0965	117.1574	132.2668	117.0982	139.0599	117.074	148.1974	117.2864	132.4525
116.9579	136.3995	117.0655	129.101	117.1741	132.2659	117.1149	139.0586	117.0906	148.1946	117.303	132.4476
116.9746	136.3994	117.0822	129.097	117.1907	132.2649	117.1315	139.0576	117.1073	148.1949	117.3197	132.45
116.9913	136.399	117.0989	129.0969	117.2074	132.2631	117.1482	139.0573	117.124	148.1966	117.3364	132.4491
117.0079	136.3983	117.1155	129.0943	117.2241	132.2625	117.1649	139.0564	117.1406	148.1944	117.353	132.4456
117.0246	136.3959	117.1322	129.0935	117.2407	132.2646	117.1815	139.0546	117.1573	148.1934	117.3697	132.4499
117.0413	136.3956	117.1489	129.0975	117.2574	132.2633	117.1982	139.0576	117.174	148.1905	117.3864	132.4453
117.0579	136.3952	117.1655	129.0962	117.2741	132.2655	117.2149	139.0547	117.1906	148.1915	117.403	132.4464
117.0746	136.3923	117.1822	129.0934	117.2907	132.2637	117.2315	139.0544	117.2073	148.1915	117.4197	132.4458
117.0913	136.3934	117.1989	129.0936	117.3074	132.2623	117.2482	139.0551	117.224	148.1887	117.4364	132.4467
117.1079	136.3917	117.2155	129.0905	117.3241	132.2619	117.2649	139.0547	117.2406	148.1898	117.453	132.4431
117.1246	136.3916	117.2322	129.0912	117.3407	132.2626	117.2815	139.0552	117.2573	148.1933	117.4697	132.4448
117.1413	136.389	117.2489	129.0916	117.3574	132.2622	117.2982	139.0523	117.274	148.1891	117.4864	132.4463

117.1579	136.3892	117.2655	129.0934	117.3741	132.2625	117.3149	139.0502	117.2906	148.1913	117.503	132.4456
117.1746	136.3867	117.2822	129.0901	117.3907	132.2633	117.3315	139.0502	117.3073	148.194	117.5197	132.4434
117.1913	136.3845	117.2989	129.0906	117.4074	132.2625	117.3482	139.0511	117.324	148.1915	117.5364	132.4441
117.2079	136.3837	117.3155	129.0894	117.4241	132.2612	117.3649	139.0501	117.3406	148.189	117.553	132.443
117.2246	136.3819	117.3322	129.0869	117.4407	132.2598	117.3815	139.0491	117.3573	148.192	117.5697	132.4415
117.2413	136.3834	117.3489	129.0872	117.4574	132.2588	117.3982	139.0496	117.374	148.1943	117.5864	132.4434
117.2579	136.3833	117.3655	129.086	117.4741	132.2584	117.4149	139.049	117.3906	148.1867	117.603	132.4414
117.2746	136.3822	117.3822	129.0879	117.4907	132.2588	117.4315	139.0475	117.4073	148.19	117.6197	132.4398
117.2913	136.3825	117.3989	129.0884	117.5074	132.259	117.4482	139.0477	117.424	148.1871	117.6364	132.442
117.3079	136.3819	117.4155	129.0865	117.5241	132.2587	117.4649	139.0478	117.4406	148.1859	117.653	132.4398
117.3246	136.3815	117.4322	129.0839	117.5407	132.2576	117.4815	139.0482	117.4573	148.1845	117.6697	132.4375
117.3413	136.3784	117.4489	129.084	117.5574	132.2562	117.4982	139.0475	117.474	148.1849	117.6864	132.4403
117.3579	136.3772	117.4655	129.0828	117.5741	132.2567	117.5149	139.0453	117.4906	148.1833	117.703	132.4375
117.3746	136.3784	117.4822	129.0856	117.5907	132.2571	117.5315	139.0447	117.5073	148.1836	117.7197	132.4369
117.3913	136.3768	117.4989	129.0821	117.6074	132.2583	117.5482	139.0451	117.524	148.1829	117.7364	132.4371
117.4079	136.3732	117.5155	129.0822	117.6241	132.257	117.5649	139.0467	117.5406	148.18	117.753	132.4381
117.4246	136.372	117.5322	129.0823	117.6407	132.2558	117.5815	139.0439	117.5573	148.1808	117.7697	132.4382
117.4413	136.3742	117.5489	129.0823	117.6574	132.2558	117.5982	139.0432	117.574	148.1769	117.7864	132.4355
117.4579	136.373	117.5655	129.0794	117.6741	132.2544	117.6149	139.0443	117.5906	148.1784	117.803	132.4368
117.4746	136.3723	117.5822	129.0804	117.6907	132.2535	117.6315	139.0416	117.6073	148.1777	117.8197	132.4354
117.4913	136.3713	117.5989	129.0792	117.7074	132.2549	117.6482	139.0437	117.624	148.1755	117.8364	132.4355
117.5079	136.366	117.6155	129.0795	117.7241	132.2556	117.6649	139.0432	117.6406	148.1764	117.853	132.4339
117.5246	136.3694	117.6322	129.0783	117.7407	132.2536	117.6815	139.0403	117.6573	148.1761	117.8697	132.4341
117.5413	136.3686	117.6489	129.0762	117.7574	132.2516	117.6982	139.0392	117.674	148.1728	117.8864	132.4343
117.5579	136.3675	117.6655	129.0746	117.7741	132.2503	117.7149	139.0402	117.6906	148.1721	117.903	132.4338
117.5746	136.3642	117.6822	129.0771	117.7907	132.252	117.7315	139.0413	117.7073	148.1698	117.9197	132.4354
117.5913	136.3623	117.6989	129.0761	117.8074	132.2501	117.7482	139.0383	117.724	148.1716	117.9364	132.4317
117.6079	136.3631	117.7155	129.073	117.8241	132.2518	117.7649	139.0365	117.7406	148.1706	117.953	132.4324
117.6246	136.3625	117.7322	129.0719	117.8407	132.2531	117.7815	139.0394	117.7573	148.1674	117.9697	132.4316
117.6413	136.3608	117.7489	129.0743	117.8574	132.2496	117.7982	139.0388	117.774	148.1709	117.9864	132.4311
117.6579	136.3595	117.7655	129.0738	117.8741	132.2511	117.8149	139.0382	117.7906	148.1708	118.003	132.431
117.6746	136.354	117.7822	129.0687	117.8907	132.2501	117.8315	139.0378	117.8073	148.1681	118.0197	132.4293
117.6913	136.357	117.7989	129.0705	117.9074	132.2484	117.8482	139.037	117.824	148.1685	118.0364	132.4314
117.7079	136.3563	117.8155	129.073	117.9241	132.249	117.8649	139.0402	117.8406	148.1691	118.053	132.4277
117.7246	136.3578	117.8322	129.0662	117.9407	132.2504	117.8815	139.0344	117.8573	148.1672	118.0697	132.4295
117.7413	136.3549	117.8489	129.068	117.9574	132.2504	117.8982	139.0381	117.874	148.1686	118.0864	132.4289
117.7579	136.352	117.8655	129.0692	117.9741	132.2476	117.9149	139.0348	117.8906	148.1684	118.103	132.4261
117.7746	136.3549	117.8822	129.07	117.9907	132.2485	117.9315	139.0373	117.9073	148.1654	118.1197	132.4279
117.7913	136.3525	117.8989	129.0679	118.0074	132.2501	117.9482	139.0367	117.924	148.1679	118.1364	132.4256
117.8079	136.3522	117.9155	129.0651	118.0241	132.249	117.9649	139.0341	117.9406	148.1628	118.153	132.4261
117.8246	136.3509	117.9322	129.0668	118.0407	132.2457	117.9815	139.032	117.9573	148.1619	118.1697	132.4256
117.8413	136.3499	117.9489	129.0658	118.0574	132.2482	117.9982	139.0321	117.974	148.1677	118.1864	132.4279
117.8579	136.3513	117.9655	129.0648	118.0741	132.2501	118.0149	139.0324	117.9906	148.1622	118.203	132.4239
117.8746	136.3495	117.9822	129.063	118.0907	132.2468	118.0315	139.0319	118.0073	148.1613	118.2197	132.4238
117.8913	136.3477	117.9989	129.061	118.1074	132.2461	118.0482	139.0309	118.024	148.1617	118.2364	132.4255
117.9079	136.3493	118.0155	129.0615	118.1241	132.245	118.0649	139.0309	118.0406	148.1616	118.253	132.4226
117.9246	136.3453	118.0322	129.0618	118.1407	132.2451	118.0815	139.0304	118.0573	148.1571	118.2697	132.4231
117.9413	136.344	118.0489	129.0597	118.1574	132.2454	118.0982	139.0326	118.074	148.1576	118.2864	132.4238
117.9579	136.3449	118.0655	129.0607	118.1741	132.2431	118.1149	139.0324	118.0906	148.1511	118.303	132.4227
117.9746	136.3433	118.0822	129.0561	118.1907	132.2454	118.1315	139.0301	118.1073	148.1571	118.3197	132.4221
117.9913	136.339	118.0989	129.0594	118.2074	132.2446	118.1482	139.0297	118.124	148.1541	118.3364	132.4244
118.0079	136.3392	118.1155	129.0562	118.2241	132.2432	118.1649	139.0307	118.1406	148.1566	118.353	132.4215
118.0246	136.3376	118.1322	129.0586	118.2407	132.2461	118.1815	139.031	118.1573	148.1542	118.3697	132.4198
118.0413	136.3372	118.1489	129.0583	118.2574	132.2474	118.1982	139.0319	118.174	148.1527	118.3864	132.42
118.0579	136.3382	118.1655	129.0523	118.2741	132.2421	118.2149	139.0284	118.1906	148.1547	118.403	132.421
118.0746	136.336	118.1822	129.0541	118.2907	132.2431	118.2315	139.0296	118.2073	148.1582	118.4197	132.4186
118.0913	136.335	118.1989	129.055	118.3074	132.2442	118.2482	139.0278	118.224	148.1539	118.4364	132.419
118.1079	136.3355	118.2155	129.0516	118.3241	132.2461	118.2649	139.0279	118.2406	148.1561	118.453	132.4185
118.1246	136.3328	118.2322	129.0543	118.3407	132.2455	118.2815	139.0277	118.2573	148.1538	118.4697	132.4174
118.1413	136.3315	118.2489	129.053	118.3574	132.2441	118.2982	139.0262	118.274	148.1562	118.4864	132.4177
118.1579	136.3309	118.2655	129.0539	118.3741	132.2431	118.3149	139.0271	118.2906	148.1545	118.503	132.4158
118.1746	136.3294	118.2822	129.0516	118.3907	132.2432	118.3315	139.024	118.3073	148.1523	118.5197	132.415
118.1913	136.3292	118.2989	129.051	118.4074	132.2414	118.3482	139.0243	118.324	148.153	118.5364	132.4179
118.2079	136.3281	118.3155	129.0478	118.4241	132.2419	118.3649	139.0252	118.3406	148.1518	118.553	132.4161
118.2246	136.3262	118.3322	129.0488	118.4407	132.2424	118.3815	139.0239	118.3573	148.1542	118.5697	132.4138
118.2413	136.3271	118.3489	129.0465	118.4574	132.2405	118.3982	139.0249	118.374	148.1516	118.5864	132.4158
118.2579	136.3272	118.3655	129.0457	118.4741	132.2426	118.4149	139.0236	118.3906	148.1513	118.603	132.4144
118.2746	136.3236	118.3822	129.0438	118.4907	132.2416	118.4315	139.0237	118.4073	148.1536	118.6197	132.4128
118.2913	136.3235	118.3989	129.0461	118.5074	132.2422	118.4482	139.0228	118.424	148.1527	118.6364	132.4137

118.3079	136.322	118.4155	129.0452	118.5241	132.2404	118.4649	139.0223	118.4406	148.1534	118.653	132.4133
118.3246	136.3218	118.4322	129.0445	118.5407	132.2385	118.4815	139.0232	118.4573	148.1517	118.6697	132.4121
118.3413	136.3182	118.4489	129.0425	118.5574	132.2401	118.4982	139.022	118.474	148.1541	118.6864	132.4131
118.3579	136.317	118.4655	129.0429	118.5741	132.2403	118.5149	139.0211	118.4906	148.1511	118.703	132.4091
118.3746	136.317	118.4822	129.0396	118.5907	132.2385	118.5315	139.0209	118.5073	148.151	118.7197	132.4086
118.3913	136.317	118.4989	129.0399	118.6074	132.2381	118.5482	139.0213	118.524	148.1518	118.7364	132.4114
118.4079	136.3168	118.5155	129.0401	118.6241	132.2396	118.5649	139.0202	118.5406	148.1487	118.753	132.4096
118.4246	136.3139	118.5322	129.0403	118.6407	132.2393	118.5815	139.018	118.5573	148.1495	118.7697	132.4089
118.4413	136.3147	118.5489	129.0376	118.6574	132.2407	118.5982	139.0176	118.574	148.1512	118.7864	132.4096
118.4579	136.3151	118.5655	129.0395	118.6741	132.2389	118.6149	139.0177	118.5906	148.1497	118.803	132.4081
118.4746	136.3117	118.5822	129.0368	118.6907	132.2397	118.6315	139.0186	118.6073	148.1511	118.8197	132.4069
118.4913	136.3114	118.5989	129.0376	118.7074	132.2385	118.6482	139.0186	118.624	148.1516	118.8364	132.4077
118.5079	136.3094	118.6155	129.0353	118.7241	132.238	118.6649	139.0152	118.6406	148.1517	118.853	132.4063
118.5246	136.3075	118.6322	129.0334	118.7407	132.2367	118.6815	139.0157	118.6573	148.1506	118.8697	132.4055
118.5413	136.3102	118.6489	129.0351	118.7574	132.2373	118.6982	139.0142	118.674	148.1489	118.8864	132.4046
118.5579	136.3069	118.6655	129.0343	118.7741	132.2367	118.7149	139.0155	118.6906	148.1479	118.903	132.4053
118.5746	136.3061	118.6822	129.0325	118.7907	132.2392	118.7315	139.0161	118.7073	148.1485	118.9197	132.4046
118.5913	136.3028	118.6989	129.0348	118.8074	132.2356	118.7482	139.0124	118.724	148.1477	118.9364	132.4032
118.6079	136.3026	118.7155	129.0301	118.8241	132.2357	118.7649	139.0129	118.7406	148.1463	118.953	132.4051
118.6246	136.3022	118.7322	129.0328	118.8407	132.2349	118.7815	139.0159	118.7573	148.1462	118.9697	132.4014
118.6413	136.3021	118.7489	129.0305	118.8574	132.2357	118.7982	139.0145	118.774	148.1451	118.9864	132.4042
118.6579	136.3014	118.7655	129.0268	118.8741	132.2326	118.8149	139.0149	118.7906	148.1439	119.003	132.4024
118.6746	136.2988	118.7822	129.0293	118.8907	132.2319	118.8315	139.0131	118.8073	148.1473	119.0197	132.401
118.6913	136.2977	118.7989	129.0314	118.9074	132.2347	118.8482	139.0113	118.824	148.1469	119.0364	132.4009
118.7079	136.295	118.8155	129.028	118.9241	132.2335	118.8649	139.0133	118.8406	148.1431	119.053	132.4006
118.7246	136.2997	118.8322	129.0258	118.9407	132.2336	118.8815	139.0094	118.8573	148.1456	119.0697	132.3973
118.7413	136.2961	118.8489	129.0281	118.9574	132.2339	118.8982	139.0123	118.874	148.1481	119.0864	132.3984
118.7579	136.2963	118.8655	129.0285	118.9741	132.232	118.9149	139.0104	118.8906	148.1463	119.103	132.3989
118.7746	136.2954	118.8822	129.023	118.9907	132.2341	118.9315	139.0106	118.9073	148.1441	119.1197	132.3967
118.7913	136.2908	118.8989	129.0268	119.0074	132.234	118.9482	139.0112	118.924	148.1445	119.1364	132.3982
118.8079	136.294	118.9155	129.0271	119.0241	132.2339	118.9649	139.0101	118.9406	148.1448	119.153	132.3975
118.8246	136.2899	118.9322	129.0245	119.0407	132.2335	118.9815	139.0091	118.9573	148.1443	119.1697	132.3961
118.8413	136.2909	118.9489	129.0226	119.0574	132.2338	118.9982	139.0094	118.974	148.144	119.1864	132.3958
118.8579	136.2886	118.9655	129.0226	119.0741	132.2332	119.0149	139.0083	118.9906	148.1421	119.203	132.3957
118.8746	136.2898	118.9822	129.0231	119.0907	132.233	119.0315	139.0101	119.0073	148.1441	119.2197	132.396
118.8913	136.2886	118.9989	129.0232	119.1074	132.2306	119.0482	139.009	119.024	148.1453	119.2364	132.3951
118.9079	136.2864	119.0155	129.0218	119.1241	132.231	119.0649	139.0093	119.0406	148.1444	119.253	132.3945
118.9246	136.2868	119.0322	129.0226	119.1407	132.232	119.0815	139.0077	119.0573	148.1418	119.2697	132.3937
118.9413	136.2865	119.0489	129.0208	119.1574	132.2294	119.0982	139.0045	119.074	148.1433	119.2864	132.3934
118.9579	136.2873	119.0655	129.0205	119.1741	132.2298	119.1149	139.0096	119.0906	148.1414	119.303	132.3954
118.9746	136.2834	119.0822	129.0194	119.1907	132.2296	119.1315	139.0067	119.1073	148.1412	119.3197	132.3896
118.9913	136.2831	119.0989	129.0195	119.2074	132.2327	119.1482	139.0074	119.124	148.1398	119.3364	132.3901
119.0079	136.2821	119.1155	129.0183	119.2241	132.2298	119.1649	139.0086	119.1406	148.1423	119.353	132.3908
119.0246	136.2802	119.1322	129.017	119.2407	132.2265	119.1815	139.0073	119.1573	148.1396	119.3697	132.3896
119.0413	136.2782	119.1489	129.0176	119.2574	132.2273	119.1982	139.0079	119.174	148.1385	119.3864	132.3885
119.0579	136.2785	119.1655	129.016	119.2741	132.2259	119.2149	139.008	119.1906	148.1395	119.403	132.3881
119.0746	136.2751	119.1822	129.0153	119.2907	132.2274	119.2315	139.0053	119.2073	148.1405	119.4197	132.389
119.0913	136.2771	119.1989	129.0145	119.3074	132.2266	119.2482	139.0059	119.224	148.1387	119.4364	132.3874
119.1079	136.2745	119.2155	129.0171	119.3241	132.2258	119.2649	139.0049	119.2406	148.1368	119.453	132.3884
119.1246	136.2738	119.2322	129.0138	119.3407	132.224	119.2815	139.0069	119.2573	148.138	119.4697	132.386
119.1413	136.2746	119.2489	129.014	119.3574	132.2238	119.2982	139.0052	119.274	148.1388	119.4864	132.3848
119.1579	136.2742	119.2655	129.012	119.3741	132.223	119.3149	139.0049	119.2906	148.137	119.503	132.389
119.1746	136.2728	119.2822	129.0088	119.3907	132.2225	119.3315	139.0044	119.3073	148.1397	119.5197	132.3893
119.1913	136.2717	119.2989	129.0083	119.4074	132.2246	119.3482	139.0048	119.324	148.1359	119.5364	132.3862
119.2079	136.2728	119.3155	129.0126	119.4241	132.226	119.3649	139.0056	119.3406	148.1366	119.553	132.3879
119.2246	136.2679	119.3322	129.0102	119.4407	132.2207	119.3815	139.0065	119.3573	148.1342	119.5697	132.3862
119.2413	136.2719	119.3489	129.0079	119.4574	132.2213	119.3982	139.0045	119.374	148.1369	119.5864	132.3855
119.2579	136.2699	119.3655	129.0049	119.4741	132.2228	119.4149	139.0028	119.3906	148.1371	119.603	132.3869
119.2746	136.2667	119.3822	129.0071	119.4907	132.2211	119.4315	139.0029	119.4073	148.1364	119.6197	132.3856
119.2913	136.263	119.3989	129.0045	119.5074	132.219	119.4482	139.0037	119.424	148.1376	119.6364	132.3826
119.3079	136.2618	119.4155	129.0068	119.5241	132.223	119.4649	139.0037	119.4406	148.1378	119.653	132.3835
119.3246	136.2648	119.4322	129.0041	119.5407	132.2203	119.4815	139.0017	119.4573	148.1323	119.6697	132.3845
119.3413	136.2645	119.4489	129.0045	119.5574	132.221	119.4982	139.0024	119.474	148.1371	119.6864	132.384
119.3579	136.2611	119.4655	129.0034	119.5741	132.2183	119.5149	139.0022	119.4906	148.1345	119.703	132.3809
119.3746	136.262	119.4822	129.0032	119.5907	132.2205	119.5315	139.0043	119.5073	148.1324	119.7197	132.3845
119.3913	136.2628	119.4989	129.0025	119.6074	132.2206	119.5482	139.0023	119.524	148.1322	119.7364	132.3808
119.4079	136.2618	119.5155	129.0028	119.6241	132.2183	119.5649	139.0003	119.5406	148.1339	119.753	132.3812
119.4246	136.2575	119.5322	129.0031	119.6407	132.2208	119.5815	138.9993	119.5573	148.1345	119.7697	132.3808
119.4413	136.2582	119.5489	129.001	119.6574	132.2175	119.5982	139.0012	119.574	148.132	119.7864	132.3775

119.4579	136.2591	119.5655	128.9984	119.6741	132.2196	119.6149	139.0018	119.5906	148.1337	119.803	132.3781
119.4746	136.2587	119.5822	128.9991	119.6907	132.2182	119.6315	139.001	119.6073	148.1331	119.8197	132.3793
119.4913	136.2556	119.5989	128.992	119.7074	132.2152	119.6482	139.0004	119.624	148.1341	119.8364	132.3795
119.5079	136.2544	119.6155	128.9971	119.7241	132.2159	119.6649	138.9972	119.6406	148.1307	119.853	132.3763
119.5246	136.2585	119.6322	128.9954	119.7407	132.217	119.6815	138.9983	119.6573	148.1356	119.8697	132.3774
119.5413	136.2503	119.6489	128.9917	119.7574	132.2175	119.6982	138.9973	119.674	148.1355	119.8864	132.3782
119.5579	136.2507	119.6655	128.9947	119.7741	132.2169	119.7149	138.9972	119.6906	148.1315	119.903	132.3756
119.5746	136.25	119.6822	128.9936	119.7907	132.2192	119.7315	138.9975	119.7073	148.1338	119.9197	132.3771
119.5913	136.2481	119.6989	128.9925	119.8074	132.2165	119.7482	138.9979	119.724	148.1319	119.9364	132.3772
119.6079	136.2483	119.7155	128.9922	119.8241	132.2155	119.7649	138.9946	119.7406	148.1303	119.953	132.379
119.6246	136.2503	119.7322	128.992	119.8407	132.2146	119.7815	138.9942	119.7573	148.1295	119.9697	132.3739
119.6413	136.2469	119.7489	128.9906	119.8574	132.2163	119.7982	138.9942	119.774	148.1308	119.9864	132.3751
119.6579	136.2566	119.7655	128.9918	119.8741	132.216	119.8149	138.9968	119.7906	148.1315	120	132.3766
119.6746	136.2466	119.7822	128.9931	119.8907	132.2175	119.8315	138.9947	119.8073	148.1296		
119.6913	136.2477	119.7989	128.9935	119.9074	132.2146	119.8482	138.9941	119.824	148.1301		
119.7079	136.2516	119.8155	128.9914	119.9241	132.2158	119.8649	138.9942	119.8406	148.1271		
119.7246	136.2495	119.8322	128.9913	119.9407	132.2148	119.8815	138.9943	119.8573	148.1281		
119.7413	136.2444	119.8489	128.9903	119.9574	132.2133	119.8982	138.9932	119.874	148.1252		
119.7579	136.2427	119.8655	128.9904	119.9741	132.2159	119.9149	138.992	119.8906	148.1249		
119.7746	136.2406	119.8822	128.9874	119.9907	132.2138	119.9315	138.9925	119.9073	148.1257		
119.7913	136.2399	119.8989	128.984	120	132.2125	119.9482	138.9913	119.924	148.1286		
119.8079	136.2424	119.9155	128.9869			119.9649	138.9902	119.9406	148.127		
119.8246	136.238	119.9322	128.9848			119.9815	138.9917	119.9573	148.1267		
119.8413	136.2373	119.9489	128.9828			119.9982	138.9926	119.974	148.1258		
119.8579	136.2362	119.9655	128.9821			120	138.9922	119.9906	148.1242		
119.8746	136.2373	119.9822	128.982					120	148.1254		
119.8913	136.2343	119.9989	128.9842								
119.9079	136.2351	120	128.9823								
119.9246	136.2324										
119.9413	136.2319										
119.9579	136.2315										
119.9746	136.2286										
119.9913	136.2313										
120	136.2273										

TPOD	1		2		3		4		5		6	
	Time /mins	Strap Tensi	Time /mins	Strap Tensi	Time /mins	Strap Tensi	Time /mins	Strap Tensi	Time /mins	Strap Tensi	Time /mins	Strap Tension /N
	0	150.0019	0	150.0029	0	150.0045	0	150.0033	0	150.0016	0	150.0023
	0.1172	149.5016	0.035383	149.6868	0.018267	150.0751	0.124833	149.5215	0.052883	150.0035	0.184867	149.5036
	0.1313	149.0012	0.05205	149.4172	0.034933	150.0092	0.1415	149.1982	0.06955	149.5764	0.201533	149.1826
	0.147967	148.5075	0.068717	149.1852	0.0516	149.947	0.158167	148.9436	0.086217	149.2523	0.2182	148.9351
	0.164633	148.07	0.085383	148.9645	0.068267	149.889	0.174833	148.7269	0.102883	148.9866	0.234867	148.7334
	0.1813	147.6742	0.10205	148.7662	0.084933	149.8302	0.1915	148.536	0.11955	148.7534	0.251533	148.5582
	0.197967	147.305	0.118717	148.5818	0.1016	149.7782	0.208167	148.3628	0.136217	148.5431	0.2682	148.4014
	0.214633	146.9581	0.135383	148.4043	0.118267	149.7241	0.224833	148.2049	0.152883	148.348	0.284867	148.2605
	0.2313	146.6293	0.15205	148.2364	0.134933	149.674	0.2415	148.0563	0.16955	148.1735	0.301533	148.1301
	0.247967	146.3165	0.168717	148.0774	0.1516	149.6221	0.258167	147.9193	0.186217	148.0078	0.3182	148.0126
	0.264633	146.0215	0.185383	147.9243	0.168267	149.5758	0.274833	147.7888	0.202883	147.855	0.334867	147.9025
	0.2813	145.7348	0.20205	147.7808	0.184933	149.5268	0.2915	147.6647	0.21955	147.7092	0.351533	147.7983
	0.297967	145.4643	0.218717	147.6401	0.2016	149.4838	0.308167	147.5522	0.236217	147.5741	0.3682	147.6999
	0.314633	145.1993	0.235383	147.506	0.218267	149.4381	0.324833	147.44	0.252883	147.4432	0.384867	147.6103
	0.3313	144.9434	0.25205	147.3781	0.234933	149.3969	0.3415	147.338	0.26955	147.3229	0.401533	147.5226
	0.347967	144.6968	0.268717	147.2548	0.2516	149.3546	0.358167	147.2339	0.286217	147.2036	0.4182	147.4406
	0.364633	144.4601	0.285383	147.1313	0.268267	149.3124	0.374833	147.14	0.302883	147.0908	0.434867	147.3646
	0.3813	144.2298	0.30205	147.0172	0.284933	149.2701	0.3915	147.0399	0.31955	146.9842	0.451533	147.2943
	0.397967	144.0075	0.318717	146.9072	0.3016	149.2343	0.408167	146.9533	0.336217	146.8858	0.4682	147.2219
	0.414633	143.7916	0.335383	146.7969	0.318267	149.1954	0.424833	146.8659	0.352883	146.7837	0.484867	147.1544
	0.4313	143.5794	0.35205	146.6907	0.334933	149.1593	0.4415	146.7824	0.36955	146.6912	0.501533	147.0928
	0.447967	143.3758	0.368717	146.589	0.3516	149.1242	0.458167	146.6967	0.386217	146.5989	0.5182	147.0301
	0.464633	143.1784	0.385383	146.4921	0.368267	149.0867	0.474833	146.6242	0.402883	146.5128	0.534867	146.9694
	0.4813	142.986	0.40205	146.3979	0.384933	149.05	0.4915	146.5511	0.41955	146.4278	0.551533	146.9111
	0.497967	142.7976	0.418717	146.3004	0.4016	149.0174	0.508167	146.4753	0.436217	146.3453	0.5682	146.858
	0.514633	142.6147	0.435383	146.2119	0.418267	148.9832	0.524833	146.4055	0.452883	146.2653	0.584867	146.8056
	0.5313	142.436	0.45205	146.1235	0.434933	148.9458	0.5415	146.3353	0.46955	146.1929	0.601533	146.7541
	0.547967	142.2593	0.468717	146.0366	0.4516	148.9161	0.558167	146.268	0.486217	146.1199	0.6182	146.7013
	0.564633	142.0904	0.485383	145.9525	0.468267	148.8864	0.574833	146.2065	0.502883	146.0476	0.634867	146.6573
	0.5813	141.919	0.50205	145.871	0.484933	148.8525	0.5915	146.143	0.51955	145.9735	0.651533	146.6114
	0.597967	141.7579	0.518717	145.7919	0.5016	148.8201	0.608167	146.0789	0.536217	145.9091	0.6682	146.5637
	0.614633	141.597	0.535383	145.7113	0.518267	148.7926	0.624833	146.0163	0.552883	145.8462	0.684867	146.5196
	0.6313	141.443	0.55205	145.6368	0.534933	148.7604	0.6415	145.9606	0.56955	145.7834	0.701533	146.4768
	0.647967	141.2891	0.568717	145.5638	0.5516	148.7327	0.658167	145.9038	0.586217	145.7182	0.7182	146.4379
	0.664633	141.1384	0.585383	145.4899	0.568267	148.7051	0.674833	145.8479	0.602883	145.6603	0.734867	146.3981
	0.6813	140.9901	0.60205	145.4186	0.584933	148.6784	0.6915	145.7919	0.61955	145.6007	0.751533	146.3572
	0.697967	140.848	0.618717	145.3461	0.6016	148.6519	0.708167	145.7399	0.636217	145.5458	0.7682	146.3195
	0.714633	140.7034	0.635383	145.2808	0.618267	148.624	0.724833	145.689	0.652883	145.4882	0.784867	146.2801
	0.7313	140.5665	0.65205	145.2155	0.634933	148.5996	0.7415	145.6337	0.66955	145.4337	0.801533	146.2472
	0.747967	140.4296	0.668717	145.1503	0.6516	148.5709	0.758167	145.5835	0.686217	145.3835	0.8182	146.2103
	0.764633	140.2956	0.685383	145.0837	0.668267	148.5452	0.774833	145.5344	0.702883	145.3338	0.834867	146.1757
	0.7813	140.1606	0.70205	145.0233	0.684933	148.519	0.7915	145.4863	0.71955	145.283	0.851533	146.1425
	0.797967	140.0322	0.718717	144.9626	0.7016	148.4981	0.808167	145.4372	0.736217	145.2332	0.8682	146.1115
	0.814633	139.9048	0.735383	144.8999	0.718267	148.4687	0.824833	145.392	0.752883	145.1863	0.884867	146.078
	0.8313	139.7794	0.75205	144.8398	0.734933	148.4445	0.8415	145.3473	0.76955	145.1404	0.901533	146.0483
	0.847967	139.6553	0.768717	144.782	0.7516	148.4221	0.858167	145.2992	0.786217	145.0943	0.9182	146.0186
	0.864633	139.5353	0.785383	144.725	0.768267	148.3976	0.874833	145.2557	0.802883	145.0509	0.934867	145.9882
	0.8813	139.4146	0.80205	144.6692	0.784933	148.3741	0.8915	145.2108	0.81955	145.0052	0.951533	145.9574
	0.897967	139.2988	0.818717	144.6113	0.8016	148.3489	0.908167	145.1731	0.836217	144.9642	0.9682	145.9327
	0.914633	139.1829	0.835383	144.5605	0.818267	148.3272	0.924833	145.1285	0.852883	144.924	0.984867	145.9023
	0.9313	139.0679	0.85205	144.5084	0.834933	148.3038	0.9415	145.0886	0.86955	144.8842	1.001533	145.8785
	0.947967	138.9557	0.868717	144.4561	0.8516	148.2829	0.958167	145.0494	0.886217	144.8401	1.0182	145.8492
	0.964633	138.8471	0.885383	144.4038	0.868267	148.26	0.974833	145.0111	0.902883	144.8028	1.034867	145.8235
	0.9813	138.7355	0.90205	144.3545	0.884933	148.2372	0.9915	144.972	0.91955	144.7655	1.051533	145.7953
	0.997967	138.6287	0.918717	144.3018	0.9016	148.2179	1.008167	144.9334	0.936217	144.7279	1.0682	145.7689
	1.014633	138.5222	0.935383	144.2551	0.918267	148.1974	1.024833	144.8975	0.952883	144.6911	1.084867	145.7416
	1.0313	138.4174	0.95205	144.205	0.934933	148.1765	1.0415	144.8601	0.96955	144.6552	1.101533	145.7192
	1.047967	138.3154	0.968717	144.1567	0.9516	148.1562	1.058167	144.8252	0.986217	144.6201	1.1182	145.6945
	1.064633	138.2126	0.985383	144.108	0.968267	148.1349	1.074833	144.7881	1.002883	144.5849	1.134867	145.6721
	1.0813	138.1123	1.00205	144.062	0.984933	148.1158	1.0915	144.755	1.01955	144.5483	1.151533	145.6482
	1.097967	138.0133	1.018717	144.0187	1.0016	148.0964	1.108167	144.7229	1.036217	144.516	1.1682	145.6261
	1.114633	137.9141	1.035383	143.9749	1.018267	148.0758	1.124833	144.6909	1.052883	144.4828	1.184867	145.603
	1.1313	137.8185	1.05205	143.9288	1.034933	148.0566	1.1415	144.6578	1.06955	144.4515	1.201533	145.5806
	1.147967	137.725	1.068717	143.8847	1.0516	148.0398	1.158167	144.6234	1.086217	144.4173	1.2182	145.5601
	1.164633	137.6315	1.085383	143.8431	1.068267	148.0188	1.174833	144.5944	1.102883	144.3869	1.234867	145.5381
	1.1813	137.5383	1.10205	143.7987	1.084933	148.0005	1.1915	144.566	1.11955	144.356	1.251533	145.517
	1.197967	137.4479	1.118717	143.7587	1.1016	147.9827	1.208167	144.5328	1.136217	144.3266	1.2682	145.4956
	1.214633	137.3567	1.135383	143.7168	1.118267	147.9663	1.224833	144.5027	1.152883	144.2928	1.284867	145.4759
	1.2313	137.2655	1.15205	143.6738	1.134933	147.9484	1.2415	144.4761	1.16955	144.2657	1.301533	145.4565
	1.247967	137.1798	1.168717	143.6254	1.1516	147.9303	1.258167	144.446	1.186217	144.2345	1.3182	145.4367
	1.264633	137.0925	1.185383	143.5918	1.168267	147.9148	1.274833	144.4181	1.202883	144.2071	1.334867	145.4178
	1.2813	137.0058	1.20205	143.5514	1.184933	147.896	1.2915	144.3883	1.21955	144.1801	1.351533	145.3987
	1.297967	136.923	1.218717	143.512	1.2016	147.8837	1.308167	144.3593	1.236217	144.1535	1.3682	145.3809
	1.314633	136.8398	1.235383	143.4729	1.218267	147.8642	1.324833	144.3343	1.252883	144.1267	1.384867	145.3643
	1.3313	136.756	1.25205	143.4342	1.234933	147.8468	1.3415	144.3036	1.26955	144.1002	1.401533	145.3448
	1.347967	136.6769	1.268717	143.3962	1.2516	147.83	1.358167	144.2786	1.286217	144.0754	1.4182	145.3282
	1.364633	136.5963	1.285383	143.3571	1.268267	147.8137	1.374833	144.2503	1.302883	144.0475	1.434867	145.309
	1.3813	136.5139	1.30205	143.3214	1.284933	147.8034	1.3915	144.2254	1.31955	144.0222	1.451533	145.2909
	1.397967	136.4376	1.318717	143.2847	1.3016	147.786	1.408167	144.1986	1.336217	144.0001	1.4682	145.2754
	1.414633	136.3619	1.335383	143.2502	1.318267	147.7671	1.424833	144.1718	1.352883	143.974	1.484867	145.2576
	1.4313	136.2817	1.35205	143.2134	1.334933	147.7556	1.4415	144.1491	1.36955	143.9511	1.501533	145.2416
	1.447967	136.2076	1.368717	143.177	1.3516	147.7398	1.458167	144.1226	1.386217	143.9253	1.5182	145.2255
	1.464633	136.1339	1.385383	143.1427	1.368267	147.7249	1.474833	144.0983	1.402883	143.9034	1.534867	145.2086
	1.4813	136.0579	1.40205	143.1071	1.384933	147.7121	1.4915	144.0711	1.41955	143.8799	1.551533	145.1925
	1.497967	135.9835	1.418717	143.073	1.4016	147.6968	1.508167	144.0461	1.436217	143.8556	1.5682	145.1752
	1.514633	135.9089	1.435383	143.0411	1.418267	147.6855	1.524833	144.0217	1.452883	143.831	1.584867	145.1617
	1.5313	135.8373	1.45205	143.0068	1.434933	147.6685	1.5415	143.9955	1.46955	143.8094	1.601533	145.1466
	1.547967	135.7642	1.468717	142.9753	1.4516	147.6559	1.558167	143.9763	1.486217	143.7864	1.6182	145.1318
	1.564633	135.6931	1.485383	142.9418	1.468267	147.6415	1.574833	143.9496	1.502883	143.7645	1.634867	145.1169
	1.5813	135.6233	1.50205	142.9092	1.484933	147.6267	1.5915	143.9293	1.51955	143.7429	1.651533	145.1012
	1.597967	135.5546	1.518717	142.8748	1.5016	147.6126	1.608167	143.9047	1.536217	143.7229	1.6682	145.0871
	1.614633	135.4823	1.535383	142.8474	1.518267	147.5993	1.624833	143.8809	1.552883	143.7003	1.684867	145.0722
	1.6313	135.4146	1.55205	142.8143	1.534933	147.5859	1.6415	143.8581	1.56955	143.683	1.701533	145.0576
	1.647967	135.3454	1.568717	142.7854	1.5516	147.5676	1.658167	143.8378	1.586217	143.6627	1.7182	145.0416
	1.664633	135.2777	1.585383	142.753	1.568267	147.5591	1.674833	143.816	1.602883	143.6434	1.734867	145.0295

1.6813	135.2102	1.60205	142.7231	1.584933	147.5458	1.6915	143.7935	1.61955	143.6237	1.751533	145.016
1.697967	135.1482	1.618717	142.693	1.6016	147.5318	1.708167	143.7733	1.636217	143.606	1.7682	145.0019
1.714633	135.0804	1.635383	142.6621	1.618267	147.5183	1.724833	143.7498	1.652883	143.5864	1.784867	144.99
1.7313	135.0153	1.65205	142.6353	1.634933	147.51	1.7415	143.7304	1.66955	143.5648	1.801533	144.978
1.747967	134.9503	1.668717	142.6031	1.6516	147.4952	1.758167	143.7119	1.686217	143.5475	1.8182	144.9654
1.764633	134.8884	1.685383	142.574	1.668267	147.4826	1.774833	143.6889	1.702883	143.527	1.834867	144.9509
1.7813	134.8229	1.70205	142.5466	1.684933	147.4727	1.7915	143.6705	1.71955	143.5128	1.851533	144.9371
1.797967	134.7602	1.718717	142.5129	1.7016	147.4622	1.808167	143.6489	1.736217	143.4964	1.8682	144.9223
1.814633	134.7003	1.735383	142.4851	1.718267	147.4479	1.824833	143.6331	1.752883	143.4773	1.884867	144.9118
1.8313	134.6403	1.75205	142.4566	1.734933	147.4373	1.8415	143.6122	1.76955	143.4601	1.901533	144.9
1.847967	134.5758	1.768717	142.4347	1.7516	147.4256	1.858167	143.5909	1.786217	143.4429	1.9182	144.8834
1.864633	134.5155	1.785383	142.4047	1.768267	147.4126	1.874833	143.5699	1.802883	143.4251	1.934867	144.8737
1.8813	134.4559	1.80205	142.3787	1.784933	147.4011	1.8915	143.5549	1.81955	143.4062	1.951533	144.8627
1.897967	134.3982	1.818717	142.3493	1.8016	147.3899	1.908167	143.5366	1.836217	143.3897	1.9682	144.8476
1.914633	134.337	1.835383	142.3265	1.818267	147.3757	1.924833	143.5169	1.852883	143.3722	1.984867	144.8365
1.9313	134.2788	1.85205	142.3006	1.834933	147.3663	1.9415	143.4983	1.86955	143.356	2.001533	144.8251
1.947967	134.2208	1.868717	142.2733	1.8516	147.3524	1.958167	143.4784	1.886217	143.3359	2.0182	144.8124
1.964633	134.1686	1.885383	142.246	1.868267	147.3388	1.974833	143.4647	1.902883	143.3208	2.034867	144.8022
1.9813	134.1062	1.90205	142.2203	1.884933	147.3296	1.9915	143.4448	1.91955	143.3025	2.051533	144.7876
1.997967	134.0463	1.918717	142.1935	1.9016	147.3168	2.008167	143.4276	1.936217	143.2885	2.0682	144.7777
2.014633	133.9922	1.935383	142.1665	1.918267	147.3051	2.024833	143.4109	1.952883	143.2705	2.084867	144.7638
2.0313	133.9364	1.95205	142.1422	1.934933	147.2936	2.0415	143.3923	1.96955	143.2537	2.101533	144.7527
2.047967	133.8807	1.968717	142.119	1.9516	147.2822	2.058167	143.3763	1.986217	143.2378	2.1182	144.7431
2.064633	133.8249	1.985383	142.0972	1.968267	147.2713	2.074833	143.3574	2.002883	143.2242	2.134867	144.7333
2.0813	133.7704	2.00205	142.0678	1.984933	147.2622	2.0915	143.3415	2.01955	143.2088	2.151533	144.7218
2.097967	133.7172	2.018717	142.0453	2.0016	147.2506	2.108167	143.3235	2.036217	143.1914	2.1682	144.7128
2.114633	133.6645	2.035383	142.0199	2.018267	147.2396	2.124833	143.3064	2.052883	143.178	2.184867	144.7023
2.1313	133.6101	2.05205	141.9936	2.034933	147.2306	2.1415	143.2913	2.06955	143.1616	2.201533	144.6911
2.147967	133.5591	2.068717	141.9711	2.0516	147.2201	2.158167	143.2758	2.086217	143.1487	2.2182	144.6821
2.164633	133.5095	2.085383	141.9482	2.068267	147.2077	2.174833	143.2579	2.102883	143.1346	2.234867	144.6727
2.1813	133.4538	2.10205	141.9228	2.084933	147.1994	2.1915	143.2427	2.11955	143.1176	2.251533	144.6606
2.197967	133.406	2.118717	141.8995	2.1016	147.1861	2.208167	143.227	2.136217	143.1026	2.2682	144.651
2.214633	133.354	2.135383	141.8776	2.118267	147.1745	2.224833	143.2121	2.152883	143.0877	2.284867	144.6412
2.2313	133.3025	2.15205	141.854	2.134933	147.1645	2.2415	143.1947	2.16955	143.0758	2.301533	144.6288
2.247967	133.2529	2.168717	141.8323	2.1516	147.154	2.258167	143.1839	2.186217	143.0633	2.3182	144.6212
2.264633	133.2023	2.185383	141.8082	2.168267	147.1415	2.274833	143.1676	2.202883	143.0502	2.334867	144.61
2.2813	133.1538	2.20205	141.7862	2.184933	147.1305	2.2915	143.1517	2.21955	143.0309	2.351533	144.6
2.297967	133.1038	2.218717	141.7648	2.2016	147.1209	2.308167	143.1368	2.236217	143.0213	2.3682	144.5876
2.314633	133.0532	2.235383	141.7407	2.218267	147.1097	2.324833	143.124	2.252883	143.0007	2.384867	144.5799
2.3313	133.0039	2.25205	141.7212	2.234933	147.0997	2.3415	143.1067	2.26955	142.9902	2.401533	144.5711
2.347967	132.9549	2.268717	141.6977	2.2516	147.093	2.358167	143.0944	2.286217	142.9732	2.4182	144.5594
2.364633	132.9108	2.285383	141.6747	2.268267	147.0786	2.374833	143.0807	2.302883	142.9639	2.434867	144.5512
2.3813	132.8631	2.30205	141.6523	2.284933	147.0714	2.3915	143.0665	2.31955	142.9506	2.451533	144.5429
2.397967	132.8129	2.318717	141.6282	2.3016	147.0655	2.408167	143.0523	2.336217	142.9381	2.4682	144.5319
2.414633	132.7665	2.335383	141.6095	2.318267	147.0531	2.424833	143.0391	2.352883	142.9229	2.484867	144.5233
2.4313	132.7201	2.35205	141.5882	2.334933	147.0447	2.4415	143.0265	2.36955	142.9121	2.501533	144.5162
2.447967	132.6736	2.368717	141.5667	2.3516	147.0334	2.458167	143.0144	2.386217	142.8967	2.5182	144.5067
2.464633	132.6277	2.385383	141.5442	2.368267	147.0241	2.474833	142.9996	2.402883	142.8855	2.534867	144.4978
2.4813	132.5834	2.40205	141.5221	2.384933	147.0158	2.4915	142.9874	2.41955	142.873	2.551533	144.4869
2.497967	132.5357	2.418717	141.5025	2.4016	147.0067	2.508167	142.9754	2.436217	142.8583	2.5682	144.4793
2.514633	132.4934	2.435383	141.4822	2.418267	146.998	2.524833	142.9609	2.452883	142.8503	2.584867	144.4698
2.5313	132.4487	2.45205	141.4597	2.434933	146.9852	2.5415	142.9468	2.46955	142.8366	2.601533	144.463
2.547967	132.4036	2.468717	141.4404	2.4516	146.98	2.558167	142.9353	2.486217	142.8227	2.6182	144.4525
2.564633	132.3594	2.485383	141.4229	2.468267	146.9686	2.574833	142.9211	2.502883	142.8113	2.634867	144.4465
2.5813	132.3145	2.50205	141.3982	2.484933	146.9602	2.5915	142.9055	2.51955	142.7998	2.651533	144.4384
2.597967	132.2718	2.518717	141.3803	2.5016	146.9526	2.608167	142.8922	2.536217	142.7895	2.6682	144.4294
2.614633	132.2288	2.535383	141.3593	2.518267	146.9401	2.624833	142.8814	2.552883	142.7765	2.684867	144.4215
2.6313	132.1867	2.55205	141.3392	2.534933	146.9327	2.6415	142.8675	2.56955	142.7676	2.701533	144.4126
2.647967	132.1404	2.568717	141.3167	2.5516	146.9257	2.658167	142.8549	2.586217	142.7558	2.7182	144.4017
2.664633	132.0995	2.585383	141.3036	2.568267	146.9149	2.674833	142.8425	2.602883	142.7485	2.734867	144.3954
2.6813	132.0574	2.60205	141.2826	2.584933	146.9062	2.6915	142.8316	2.61955	142.7348	2.751533	144.3891
2.697967	132.0156	2.618717	141.263	2.6016	146.8967	2.708167	142.817	2.636217	142.7234	2.7682	144.3817
2.714633	131.9737	2.635383	141.242	2.618267	146.8907	2.724833	142.8027	2.652883	142.7123	2.784867	144.3698
2.7313	131.9328	2.65205	141.2227	2.634933	146.8793	2.7415	142.795	2.66955	142.7028	2.801533	144.3665
2.747967	131.8898	2.668717	141.2035	2.6516	146.8711	2.758167	142.7817	2.686217	142.6925	2.8182	144.3545
2.764633	131.8493	2.685383	141.1847	2.668267	146.8596	2.774833	142.7688	2.702883	142.6802	2.834867	144.3485
2.7813	131.8081	2.70205	141.1644	2.684933	146.8531	2.7915	142.7572	2.71955	142.667	2.851533	144.3382
2.797967	131.7684	2.718717	141.1466	2.7016	146.8441	2.808167	142.7454	2.736217	142.6588	2.8682	144.3333
2.814633	131.7255	2.735383	141.1289	2.718267	146.8346	2.824833	142.7319	2.752883	142.6496	2.884867	144.3249
2.8313	131.6913	2.75205	141.1069	2.734933	146.8268	2.8415	142.7219	2.76955	142.6391	2.901533	144.3161
2.847967	131.6478	2.768717	141.0893	2.7516	146.8197	2.858167	142.711	2.786217	142.6283	2.9182	144.3067
2.864633	131.6079	2.785383	141.0705	2.768267	146.8085	2.874833	142.6993	2.802883	142.6154	2.934867	144.3004
2.8813	131.5685	2.80205	141.0552	2.784933	146.8012	2.8915	142.6871	2.81955	142.6058	2.951533	144.2938
2.897967	131.5323	2.818717	141.0354	2.8016	146.7952	2.908167	142.676	2.836217	142.5948	2.9682	144.2855
2.914633	131.4905	2.835383	141.0158	2.818267	146.7872	2.924833	142.665	2.852883	142.5823	2.984867	144.2741
2.9313	131.454	2.85205	140.996	2.834933	146.7767	2.9415	142.6508	2.86955	142.5761	3.001533	144.2709
2.947967	131.4135	2.868717	140.9805	2.8516	146.7716	2.958167	142.6425	2.886217	142.5622	3.0182	144.2606
2.964633	131.3725	2.885383	140.9635	2.868267	146.7592	2.974833	142.6311	2.902883	142.5541	3.034867	144.2537
2.9813	131.3378	2.90205	140.943	2.884933	146.7523	2.9915	142.621	2.91955	142.5464	3.051533	144.243
2.997967	131.2978	2.918717	140.9261	2.9016	146.7464	3.008167	142.6112	2.936217	142.5298	3.0682	144.2405
3.014633	131.2603	2.935383	140.9088	2.918267	146.7368	3.024833	142.597	2.952883	142.5227	3.084867	144.2312
3.0313	131.2237	2.95205	140.8906	2.934933	146.7306	3.0415	142.5879	2.96955	142.5081	3.101533	144.2224
3.047967	131.1856	2.968717	140.8742	2.9516	146.7223	3.058167	142.5777	2.986217	142.5003	3.1182	144.2157
3.064633	131.1482	2.985383	140.8566	2.968267	146.7145	3.074833	142.5654	3.002883	142.4921	3.134867	144.2076
3.0813	131.1142	3.00205	140.8374	2.984933	146.7052	3.0915	142.5545	3.01955	142.48	3.151533	144.2025
3.097967	131.0748	3.018717	140.821	3.0016	146.6968	3.108167	142.5433	3.036217	142.4717	3.1682	144.1948
3.114633	131.039	3.035383	140.8041	3.018267	146.6891	3.124833	142.5331	3.052883	142.4611	3.184867	144.1856
3.1313	131.0029	3.05205	140.7864	3.034933	146.6834	3.1415	142.5272	3.06955	142.4504	3.201533	144.18
3.147967	130.9667	3.068717	140.7714	3.0516	146.6768	3.158167	142.5165	3.086217	142.4398	3.2182	144.1748
3.164633	130.9317	3.085383	140.7533	3.068267	146.6661	3.174833	142.5049	3.102883	142.4345	3.234867	144.1672
3.1813	130.8939	3.10205	140.7355	3.084933	146.6628	3.1915	142.4974	3.11955	142.4213	3.251533	144.1599
3.197967	130.8599	3.118717	140.7208	3.1016	146.6518	3.208167	142.4859	3.136217	142.4125	3.2682	144.1514
3.214633	130.8269	3.135383	140.7062	3.118267	146.6465	3.224833	142.4768	3.152883	142.4049	3.284867	144.1473
3.2313	130.79	3.15205	140.6873	3.134933	146.6377	3.2415	142.4669	3.16955	142.3967	3.301533	144.1395
3.247967	130.7549	3.168717	140.6738	3.1516	146.6293	3.258167	142.4569	3.186217	142.3845	3.3182	144.1336
3.264633	130.7215	3.185383	140.6532	3.168267	146.6225	3.274833	142.4462	3.202883	142.3764	3.334867	144.1265
3.2813	130.6872	3.20205	140.6404	3.184933	146.6175	3.2915	142.4393	3.21955	142.3678	3.351533	144.1198

3.297967	130.6519	3.218717	140.6243	3.2016	146.6067	3.308167	142.426	3.236217	142.3592	3.3682	144.1153
3.314633	130.6173	3.235383	140.6061	3.218267	146.5995	3.324833	142.4203	3.252883	142.3496	3.384867	144.1047
3.3313	130.5854	3.25205	140.5908	3.234933	146.5918	3.3415	142.41	3.26955	142.3421	3.401533	144.0997
3.347967	130.5528	3.268717	140.5769	3.2516	146.5893	3.358167	142.3984	3.286217	142.3302	3.4182	144.0949
3.364633	130.5149	3.285383	140.5588	3.268267	146.5807	3.374833	142.3874	3.302883	142.3241	3.434867	144.0875
3.3813	130.4859	3.30205	140.5444	3.284933	146.5747	3.3915	142.3802	3.31955	142.3139	3.451533	144.0791
3.397967	130.4512	3.318717	140.5286	3.3016	146.5641	3.408167	142.3695	3.336217	142.3068	3.4682	144.0749
3.414633	130.4187	3.335383	140.5152	3.318267	146.5588	3.424833	142.3614	3.352883	142.297	3.484867	144.0682
3.4313	130.3865	3.35205	140.4978	3.334933	146.5511	3.4415	142.3504	3.36955	142.2918	3.501533	144.0615
3.447967	130.3526	3.368717	140.4849	3.3516	146.5453	3.458167	142.3417	3.386217	142.2832	3.5182	144.0558
3.464633	130.3241	3.385383	140.4706	3.368267	146.5366	3.474833	142.3342	3.402883	142.2711	3.534867	144.0496
3.4813	130.2889	3.40205	140.4487	3.384933	146.5291	3.4915	142.3247	3.41955	142.2644	3.551533	144.0411
3.497967	130.2593	3.418717	140.4385	3.4016	146.5207	3.508167	142.3153	3.436217	142.2544	3.5682	144.038
3.514633	130.2267	3.435383	140.4192	3.418267	146.5157	3.524833	142.3059	3.452883	142.2469	3.584867	144.0324
3.5313	130.1918	3.45205	140.4053	3.434933	146.509	3.5415	142.2979	3.46955	142.2407	3.601533	144.0258
3.547967	130.1613	3.468717	140.3916	3.4516	146.502	3.558167	142.2902	3.486217	142.2297	3.6182	144.0184
3.564633	130.1302	3.485383	140.3787	3.468267	146.4956	3.574833	142.2785	3.502883	142.2201	3.634867	144.0129
3.5813	130.0981	3.50205	140.3586	3.484933	146.4912	3.5915	142.2745	3.51955	142.2145	3.651533	144.0072
3.597967	130.0643	3.518717	140.3479	3.5016	146.4853	3.608167	142.262	3.536217	142.2078	3.6682	144.0025
3.614633	130.0368	3.535383	140.3319	3.518267	146.4771	3.624833	142.2544	3.552883	142.1988	3.684867	143.9954
3.6313	130.0022	3.55205	140.3178	3.534933	146.4733	3.6415	142.2441	3.56955	142.1896	3.701533	143.9905
3.647967	129.9734	3.568717	140.3008	3.5516	146.4696	3.658167	142.236	3.586217	142.1795	3.7182	143.9827
3.664633	129.9441	3.585383	140.2884	3.568267	146.4638	3.674833	142.2279	3.602883	142.1758	3.734867	143.9784
3.6813	129.9106	3.60205	140.2739	3.584933	146.455	3.6915	142.2201	3.61955	142.1666	3.751533	143.9706
3.697967	129.8805	3.618717	140.2596	3.6016	146.4481	3.708167	142.2107	3.636217	142.1593	3.7682	143.9669
3.714633	129.8487	3.635383	140.242	3.618267	146.4435	3.724833	142.2017	3.652883	142.1483	3.784867	143.962
3.7313	129.8203	3.65205	140.2273	3.634933	146.4386	3.7415	142.1938	3.66955	142.1443	3.801533	143.9562
3.747967	129.7903	3.668717	140.214	3.6516	146.4353	3.758167	142.1866	3.686217	142.1356	3.8182	143.948
3.764633	129.7599	3.685383	140.2033	3.668267	146.4286	3.774833	142.1744	3.702883	142.1275	3.834867	143.9442
3.7813	129.7317	3.70205	140.186	3.684933	146.4194	3.7915	142.1699	3.71955	142.1188	3.851533	143.9396
3.797967	129.7007	3.718717	140.1726	3.7016	146.4144	3.808167	142.1596	3.736217	142.1126	3.8682	143.9341
3.814633	129.6685	3.735383	140.1541	3.718267	146.4116	3.824833	142.1553	3.752883	142.103	3.884867	143.9267
3.8313	129.6426	3.75205	140.1453	3.734933	146.4043	3.8415	142.1448	3.76955	142.0961	3.901533	143.9232
3.847967	129.6115	3.768717	140.1317	3.7516	146.396	3.858167	142.1308	3.786217	142.0913	3.9182	143.9181
3.864633	129.5797	3.785383	140.1228	3.768267	146.3933	3.874833	142.1263	3.802883	142.0825	3.934867	143.9138
3.8813	129.5508	3.80205	140.1045	3.784933	146.3856	3.8915	142.1176	3.81955	142.0775	3.951533	143.9056
3.897967	129.5257	3.818717	140.0926	3.8016	146.3783	3.908167	142.1118	3.836217	142.0673	3.9682	143.9002
3.914633	129.4965	3.835383	140.0796	3.818267	146.3691	3.924833	142.104	3.852883	142.0626	3.984867	143.8961
3.9313	129.4685	3.85205	140.0613	3.834933	146.3649	3.9415	142.0937	3.86955	142.0528	4.001533	143.8899
3.947967	129.4413	3.868717	140.057	3.8516	146.3585	3.958167	142.0889	3.886217	142.0465	4.0182	143.8814
3.964633	129.4106	3.885383	140.0352	3.868267	146.3484	3.974833	142.0796	3.902883	142.0382	4.034867	143.8814
3.9813	129.3809	3.90205	140.0224	3.884933	146.3464	3.9915	142.0729	3.91955	142.0308	4.051533	143.8744
3.997967	129.3559	3.918717	140.0094	3.9016	146.3395	4.008167	142.0635	3.936217	142.0247	4.0682	143.8684
4.014633	129.3276	3.935383	139.9958	3.918267	146.3347	4.024833	142.0565	3.952883	142.0191	4.084867	143.8624
4.0313	129.2981	3.95205	139.9818	3.934933	146.326	4.0415	142.0487	3.96955	142.0109	4.101533	143.8595
4.047967	129.2708	3.968717	139.9667	3.9516	146.323	4.058167	142.043	3.986217	142.0041	4.1182	143.8527
4.064633	129.2451	3.985383	139.9549	3.968267	146.3164	4.074833	142.0358	4.002883	141.9977	4.134867	143.8486
4.0813	129.2173	4.00205	139.9417	3.984933	146.3096	4.0915	142.0286	4.01955	141.9919	4.151533	143.8428
4.097967	129.1902	4.018717	139.9298	4.0016	146.3064	4.108167	142.0216	4.036217	141.9852	4.1682	143.8365
4.114633	129.1627	4.035383	139.915	4.018267	146.2977	4.124833	142.0146	4.052883	141.9796	4.184867	143.8322
4.1313	129.1356	4.05205	139.9004	4.034933	146.2929	4.1415	142.0046	4.06955	141.9721	4.201533	143.8285
4.147967	129.1113	4.068717	139.8905	4.0516	146.2863	4.158167	141.9985	4.086217	141.9672	4.2182	143.8239
4.164633	129.084	4.085383	139.8807	4.068267	146.2813	4.174833	141.9903	4.102883	141.9594	4.234867	143.8169
4.1813	129.0569	4.10205	139.8624	4.084933	146.2749	4.1915	141.9864	4.11955	141.9507	4.251533	143.8115
4.197967	129.0308	4.118717	139.8518	4.1016	146.2686	4.208167	141.9764	4.136217	141.9451	4.2682	143.807
4.214633	129.0048	4.135383	139.8423	4.118267	146.264	4.224833	141.97	4.152883	141.9375	4.284867	143.8027
4.2313	128.9785	4.15205	139.8251	4.134933	146.2596	4.2415	141.9614	4.16955	141.9323	4.301533	143.7991
4.247967	128.9514	4.168717	139.8106	4.1516	146.2538	4.258167	141.9541	4.186217	141.9255	4.3182	143.7894
4.264633	128.926	4.185383	139.8046	4.168267	146.2471	4.274833	141.9464	4.202883	141.9199	4.334867	143.7863
4.2813	128.9001	4.20205	139.79	4.184933	146.2411	4.2915	141.9416	4.21955	141.9125	4.351533	143.7824
4.297967	128.8734	4.218717	139.7748	4.2016	146.2376	4.308167	141.9328	4.236217	141.9034	4.3682	143.778
4.314633	128.8501	4.235383	139.762	4.218267	146.2294	4.324833	141.926	4.252883	141.8982	4.384867	143.7737
4.3313	128.8227	4.25205	139.7474	4.234933	146.2244	4.3415	141.9165	4.26955	141.8939	4.401533	143.7675
4.347967	128.7997	4.268717	139.7365	4.2516	146.2194	4.358167	141.9132	4.286217	141.8843	4.4182	143.7646
4.364633	128.7714	4.285383	139.7255	4.268267	146.2129	4.374833	141.9043	4.302883	141.878	4.434867	143.7594
4.3813	128.7475	4.30205	139.7136	4.284933	146.2068	4.3915	141.8957	4.31955	141.8736	4.451533	143.7539
4.397967	128.7216	4.318717	139.7021	4.3016	146.2043	4.408167	141.8874	4.336217	141.8644	4.4682	143.7505
4.414633	128.6921	4.335383	139.6904	4.318267	146.1976	4.424833	141.8809	4.352883	141.86	4.484867	143.744
4.4313	128.6696	4.35205	139.6768	4.334933	146.193	4.4415	141.8743	4.36955	141.8509	4.501533	143.7398
4.447967	128.6481	4.368717	139.6645	4.3516	146.1939	4.458167	141.8692	4.386217	141.8447	4.5182	143.7346
4.464633	128.62	4.385383	139.6538	4.368267	146.1864	4.474833	141.8591	4.402883	141.8396	4.534867	143.7309
4.4813	128.5946	4.40205	139.6392	4.384933	146.1782	4.4915	141.8529	4.41955	141.8322	4.551533	143.7246
4.497967	128.5702	4.418717	139.6291	4.4016	146.1732	4.508167	141.8444	4.436217	141.8257	4.5682	143.7225
4.514633	128.545	4.435383	139.6185	4.418267	146.1735	4.524833	141.8403	4.452883	141.8202	4.584867	143.7151
4.5313	128.5221	4.45205	139.6073	4.434933	146.1628	4.5415	141.8319	4.46955	141.8141	4.601533	143.7125
4.547967	128.4987	4.468717	139.5949	4.4516	146.1632	4.558167	141.83	4.486217	141.8055	4.6182	143.7064
4.564633	128.4744	4.485383	139.5831	4.468267	146.1513	4.574833	141.8186	4.502883	141.8003	4.634867	143.7012
4.5813	128.4498	4.50205	139.5725	4.484933	146.1476	4.5915	141.8116	4.51955	141.7949	4.651533	143.6961
4.597967	128.4239	4.518717	139.5592	4.5016	146.1435	4.608167	141.8053	4.536217	141.7863	4.6682	143.6944
4.614633	128.4009	4.535383	139.5475	4.518267	146.1415	4.624833	141.7981	4.552883	141.7829	4.684867	143.6886
4.6313	128.3786	4.55205	139.5386	4.534933	146.1279	4.6415	141.7924	4.56955	141.7741	4.701533	143.6859
4.647967	128.3551	4.568717	139.5226	4.5516	146.1302	4.658167	141.7826	4.586217	141.7688	4.7182	143.6809
4.664633	128.331	4.585383	139.5154	4.568267	146.1208	4.674833	141.7823	4.602883	141.7623	4.734867	143.6768
4.6813	128.3069	4.60205	139.4981	4.584933	146.1172	4.6915	141.7711	4.61955	141.7585	4.751533	143.6704
4.697967	128.2831	4.618717	139.491	4.6016	146.1096	4.708167	141.7643	4.636217	141.7506	4.7682	143.6678
4.714633	128.2598	4.635383	139.48	4.618267	146.1069	4.724833	141.7565	4.652883	141.7458	4.784867	143.662
4.7313	128.2349	4.65205	139.4648	4.634933	146.0988	4.7415	141.7526	4.66955	141.7409	4.801533	143.6589
4.747967	128.2158	4.668717	139.4568	4.6516	146.0946	4.758167	141.7441	4.686217	141.7312	4.8182	143.6535
4.764633	128.1879	4.685383	139.4442	4.668267	146.0893	4.774833	141.7382	4.702883	141.7268	4.834867	143.6492
4.7813	128.1638	4.70205	139.4312	4.684933	146.0845	4.7915	141.7347	4.71955	141.7228	4.851533	143.6446
4.797967	128.1435	4.718717	139.4194	4.7016	146.0755	4.808167	141.7282	4.736217	141.7152	4.8682	143.642
4.814633	128.1207	4.735383	139.4102	4.718267	146.0714	4.824833	141.72	4.752883	141.7104	4.884867	143.6344
4.8313	128.0964	4.75205	139.3991	4.734933	146.0694	4.8415	141.7137	4.76955	141.7046	4.901533	143.6329
4.847967	128.0756	4.768717	139.3883	4.7516	146.0626	4.858167	141.7085	4.786217	141.6996	4.9182	143.6277
4.864633	128.0518	4.785383	139.3759	4.768267	146.0575	4.874833	141.6995	4.802883	141.693	4.934867	143.621
4.8813	128.0314	4.80205	139.3651	4.784933	146.0554	4.8915	141.6934	4.81955	141.6871	4.951533	143.616
4.897967	128.0069	4.818717	139.3562	4.8016	146.05	4.908167	141.6886	4.836217	141.6815	4.9682	143.6149

4.914633	127.9819	4.835383	139.3467	4.818267	146.0432	4.924833	141.6849	4.852883	141.6772	4.984867	143.6101
4.9313	127.9586	4.85205	139.3353	4.834933	146.0426	4.9415	141.6749	4.86955	141.6693	5.001533	143.606
4.947967	127.938	4.868717	139.3198	4.8516	146.0293	4.958167	141.6726	4.886217	141.6667	5.0182	143.601
4.964633	127.9146	4.885383	139.3117	4.868267	146.0309	4.974833	141.6646	4.902883	141.66	5.034867	143.5988
4.9813	127.8923	4.90205	139.3014	4.884933	146.0233	4.9915	141.6588	4.91955	141.6555	5.051533	143.5952
4.997967	127.8718	4.918717	139.2929	4.9016	146.019	5.008167	141.6537	4.936217	141.6533	5.0682	143.5922
5.014633	127.8495	4.935383	139.2791	4.918267	146.0144	5.024833	141.648	4.952883	141.6436	5.084867	143.5833
5.0313	127.8251	4.95205	139.2668	4.934933	146.007	5.0415	141.6389	4.96955	141.6399	5.101533	143.5796
5.047967	127.8059	4.968717	139.2594	4.9516	146.0073	5.058167	141.6371	4.986217	141.6348	5.1182	143.5779
5.064633	127.7841	4.985383	139.2449	4.968267	145.9991	5.074833	141.6272	5.002883	141.6307	5.134867	143.5725
5.0813	127.7624	5.00205	139.2379	4.984933	145.9957	5.0915	141.6208	5.01955	141.6249	5.151533	143.5676
5.097967	127.7407	5.018717	139.2259	5.0016	145.9906	5.108167	141.6152	5.036217	141.6199	5.1682	143.5648
5.114633	127.7211	5.035383	139.216	5.018267	145.9876	5.124833	141.6097	5.052883	141.6176	5.184867	143.5621
5.1313	127.6949	5.05205	139.2065	5.034933	145.9835	5.1415	141.602	5.06955	141.609	5.201533	143.558
5.147967	127.6733	5.068717	139.1914	5.0516	145.976	5.158167	141.5961	5.086217	141.6036	5.2182	143.553
5.164633	127.6544	5.085383	139.18	5.068267	145.9733	5.174833	141.5893	5.102883	141.595	5.234867	143.549
5.1813	127.6321	5.10205	139.1713	5.084933	145.9725	5.1915	141.582	5.11955	141.5912	5.251533	143.5463
5.197967	127.6085	5.118717	139.1597	5.1016	145.9648	5.208167	141.5756	5.136217	141.5844	5.2682	143.5437
5.214633	127.5888	5.135383	139.1486	5.118267	145.9605	5.224833	141.5728	5.152883	141.5798	5.284867	143.539
5.2313	127.5687	5.15205	139.1412	5.134933	145.9567	5.2415	141.5645	5.16955	141.5733	5.301533	143.535
5.247967	127.5474	5.168717	139.13	5.1516	145.9522	5.258167	141.5586	5.186217	141.5673	5.3182	143.5322
5.264633	127.5281	5.185383	139.1186	5.168267	145.9469	5.274833	141.5536	5.202883	141.562	5.334867	143.5275
5.2813	127.5059	5.20205	139.1087	5.184933	145.9419	5.2915	141.5459	5.21955	141.5595	5.351533	143.523
5.297967	127.4843	5.218717	139.1013	5.2016	145.9361	5.308167	141.5401	5.236217	141.5537	5.3682	143.5212
5.314633	127.4631	5.235383	139.089	5.218267	145.9308	5.324833	141.5354	5.252883	141.5493	5.384867	143.5188
5.3313	127.4427	5.25205	139.0806	5.234933	145.9282	5.3415	141.5301	5.26955	141.5422	5.401533	143.5072
5.347967	127.4227	5.268717	139.0696	5.2516	145.9231	5.358167	141.5256	5.286217	141.5396	5.4182	143.5061
5.364633	127.4024	5.285383	139.0587	5.268267	145.9188	5.374833	141.5166	5.302883	141.5363	5.434867	143.501
5.3813	127.3835	5.30205	139.0473	5.284933	145.9128	5.3915	141.5129	5.31955	141.5291	5.451533	143.4982
5.397967	127.3631	5.318717	139.0408	5.3016	145.9099	5.408167	141.5043	5.336217	141.525	5.4682	143.4942
5.414633	127.34	5.335383	139.0266	5.318267	145.9005	5.424833	141.501	5.352883	141.5206	5.484867	143.4899
5.4313	127.3218	5.35205	139.018	5.334933	145.9018	5.4415	141.4967	5.36955	141.5143	5.501533	143.4886
5.447967	127.2984	5.368717	139.0103	5.3516	145.8936	5.458167	141.4917	5.386217	141.5094	5.5182	143.4822
5.464633	127.2817	5.385383	138.9961	5.368267	145.8913	5.474833	141.4844	5.402883	141.5045	5.534867	143.4796
5.4813	127.2596	5.40205	138.989	5.384933	145.889	5.4915	141.4814	5.41955	141.5011	5.551533	143.474
5.497967	127.2405	5.418717	138.9804	5.4016	145.8791	5.508167	141.4739	5.436217	141.4954	5.5682	143.4707
5.514633	127.2176	5.435383	138.9686	5.418267	145.8771	5.524833	141.4648	5.452883	141.4927	5.584867	143.468
5.5313	127.2003	5.45205	138.9594	5.434933	145.8725	5.5415	141.4629	5.46955	141.4885	5.601533	143.4653
5.547967	127.1827	5.468717	138.9484	5.4516	145.8713	5.558167	141.4562	5.486217	141.4832	5.6182	143.4587
5.564633	127.1612	5.485383	138.9368	5.468267	145.8637	5.574833	141.4493	5.502883	141.4762	5.634867	143.4575
5.5813	127.1429	5.50205	138.927	5.484933	145.861	5.5915	141.4495	5.51955	141.4732	5.651533	143.4526
5.597967	127.1216	5.518717	138.9187	5.5016	145.8558	5.608167	141.4433	5.536217	141.4672	5.6682	143.4495
5.614633	127.1046	5.535383	138.9084	5.518267	145.8529	5.624833	141.4366	5.552883	141.4639	5.684867	143.444
5.6313	127.0843	5.55205	138.8975	5.534933	145.8495	5.6415	141.43	5.56955	141.4559	5.701533	143.4411
5.647967	127.0658	5.568717	138.887	5.5516	145.847	5.658167	141.4242	5.586217	141.4518	5.7182	143.439
5.664633	127.0446	5.585383	138.8781	5.568267	145.8395	5.674833	141.4205	5.602883	141.4453	5.734867	143.4345
5.6813	127.0283	5.60205	138.8685	5.584933	145.8359	5.6915	141.4149	5.61955	141.4412	5.751533	143.4304
5.697967	127.0054	5.618717	138.8582	5.6016	145.8304	5.708167	141.4068	5.636217	141.4354	5.7682	143.4269
5.714633	126.9908	5.635383	138.8454	5.618267	145.8285	5.724833	141.4025	5.652883	141.4304	5.784867	143.4236
5.7313	126.9675	5.65205	138.8383	5.634933	145.8236	5.7415	141.3989	5.66955	141.4264	5.801533	143.4202
5.747967	126.9473	5.668717	138.8281	5.6516	145.822	5.758167	141.392	5.686217	141.4206	5.8182	143.417
5.764633	126.9312	5.685383	138.8218	5.668267	145.8163	5.774833	141.3853	5.702883	141.4173	5.834867	143.4125
5.7813	126.9135	5.70205	138.8138	5.684933	145.813	5.7915	141.3835	5.71955	141.4115	5.851533	143.4095
5.797967	126.8945	5.718717	138.8034	5.7016	145.8077	5.808167	141.3781	5.736217	141.4072	5.8682	143.4058
5.814633	126.8711	5.735383	138.7973	5.718267	145.8027	5.824833	141.3739	5.752883	141.4038	5.884867	143.4042
5.8313	126.8563	5.75205	138.7872	5.734933	145.8	5.8415	141.3663	5.76955	141.3993	5.901533	143.4003
5.847967	126.8368	5.768717	138.7751	5.7516	145.7956	5.858167	141.3626	5.786217	141.3937	5.9182	143.3966
5.864633	126.8199	5.785383	138.7663	5.768267	145.7911	5.874833	141.3572	5.802883	141.3855	5.934867	143.3928
5.8813	126.7992	5.80205	138.7534	5.784933	145.7878	5.8915	141.352	5.81955	141.3849	5.951533	143.3887
5.897967	126.7799	5.818717	138.7484	5.8016	145.7826	5.908167	141.3455	5.836217	141.3804	5.9682	143.3854
5.914633	126.7616	5.835383	138.7363	5.818267	145.7789	5.924833	141.3411	5.852883	141.3745	5.984867	143.3834
5.9313	126.7411	5.85205	138.7293	5.834933	145.7758	5.9415	141.3366	5.86955	141.3672	6.001533	143.3812
5.947967	126.7239	5.868717	138.7191	5.8516	145.771	5.958167	141.3321	5.886217	141.3629	6.0182	143.3755
5.964633	126.7041	5.885383	138.7102	5.868267	145.7669	5.974833	141.3235	5.902883	141.3595	6.034867	143.3721
5.9813	126.6857	5.90205	138.6989	5.884933	145.7604	5.9915	141.3208	5.91955	141.3543	6.051533	143.3688
5.997967	126.6701	5.918717	138.6923	5.9016	145.7606	6.008167	141.3169	5.936217	141.3499	6.0682	143.3648
6.014633	126.6513	5.935383	138.6831	5.918267	145.7555	6.024833	141.3112	5.952883	141.3434	6.084867	143.3604
6.0313	126.6332	5.95205	138.6743	5.934933	145.7514	6.0415	141.3064	5.96955	141.3413	6.101533	143.3598
6.047967	126.6101	5.968717	138.6657	5.9516	145.7496	6.058167	141.3034	5.986217	141.3373	6.1182	143.357
6.064633	126.5954	5.985383	138.6551	5.968267	145.7449	6.074833	141.2979	6.002883	141.332	6.134867	143.3517
6.0813	126.5794	6.00205	138.6462	5.984933	145.7406	6.0915	141.2947	6.01955	141.3275	6.151533	143.3482
6.097967	126.5606	6.018717	138.6363	6.0016	145.7351	6.108167	141.2877	6.036217	141.3236	6.1682	143.3451
6.114633	126.5421	6.035383	138.6264	6.018267	145.7319	6.124833	141.2825	6.052883	141.3199	6.184867	143.3421
6.1313	126.5218	6.05205	138.6204	6.034933	145.7315	6.1415	141.2775	6.06955	141.3145	6.201533	143.3403
6.147967	126.5078	6.068717	138.6109	6.0516	145.727	6.158167	141.2761	6.086217	141.3107	6.2182	143.3334
6.164633	126.487	6.085383	138.6035	6.068267	145.7221	6.174833	141.2688	6.102883	141.3083	6.234867	143.3334
6.1813	126.469	6.10205	138.5926	6.084933	145.7157	6.1915	141.2707	6.11955	141.3021	6.251533	143.3311
6.197967	126.4512	6.118717	138.5879	6.1016	145.7194	6.208167	141.2629	6.136217	141.2967	6.2682	143.3255
6.214633	126.4331	6.135383	138.5773	6.118267	145.7129	6.224833	141.2544	6.152883	141.2927	6.284867	143.3207
6.2313	126.4138	6.15205	138.5705	6.134933	145.7071	6.2415	141.2524	6.16955	141.2887	6.301533	143.3213
6.247967	126.3973	6.168717	138.5583	6.1516	145.7009	6.258167	141.2427	6.186217	141.2849	6.3182	143.3166
6.264633	126.3796	6.185383	138.5481	6.168267	145.7015	6.274833	141.2377	6.202883	141.2787	6.334867	143.3119
6.2813	126.3633	6.20205	138.5418	6.184933	145.6977	6.2915	141.2312	6.21955	141.2752	6.351533	143.3092
6.297967	126.3458	6.218717	138.5322	6.2016	145.6917	6.308167	141.2294	6.236217	141.2694	6.3682	143.3076
6.314633	126.3273	6.235383	138.5205	6.218267	145.6872	6.324833	141.2287	6.252883	141.264	6.384867	143.3039
6.3313	126.3102	6.25205	138.5148	6.234933	145.6858	6.3415	141.2184	6.26955	141.2588	6.401533	143.3021
6.347967	126.2924	6.268717	138.5049	6.2516	145.6772	6.358167	141.2167	6.286217	141.2542	6.4182	143.298
6.364633	126.2744	6.285383	138.4947	6.268267	145.6742	6.374833	141.2101	6.302883	141.2486	6.434867	143.2974
6.3813	126.256	6.30205	138.4878	6.284933	145.67	6.3915	141.2046	6.31955	141.2466	6.451533	143.2935
6.397967	126.2414	6.318717	138.4805	6.3016	145.6667	6.408167	141.1983	6.336217	141.2393	6.4682	143.2913
6.414633	126.2203	6.335383	138.4687	6.318267	145.6634	6.424833	141.1921	6.352883	141.2372	6.484867	143.2854
6.4313	126.2027	6.35205	138.4614	6.334933	145.6573	6.4415	141.1892	6.36955	141.2298	6.501533	143.285
6.447967	126.1875	6.368717	138.4508	6.3516	145.6545	6.458167	141.1848	6.386217	141.2283	6.5182	143.2808
6.464633	126.1727	6.385383	138.4454	6.368267	145.6528	6.474833	141.1773	6.402883	141.2212	6.534867	143.2782
6.4813	126.1544	6.40205	138.4334	6.384933	145.6473	6.4915	141.1734	6.41955	141.215	6.551533	143.2751
6.497967	126.1356	6.418717	138.4277	6.4016	145.6434	6.508167	141.1665	6.436217	141.211	6.5682	143.2743
6.514633	126.1172	6.435383	138.4173	6.418267	145.6388	6.524833	141.1624	6.452883	141.2107	6.584867	143.27

6.5313	126.1012	6.45205	138.4087	6.434933	145.6362	6.5415	141.159	6.46955	141.2041	6.601533	143.2664
6.547967	126.0839	6.468717	138.4009	6.4516	145.6342	6.558167	141.1566	6.486217	141.2013	6.6182	143.2646
6.564633	126.0677	6.485383	138.3925	6.468267	145.6307	6.574833	141.1484	6.502883	141.1952	6.634867	143.259
6.5813	126.052	6.50205	138.3813	6.484933	145.6262	6.5915	141.1463	6.51955	141.1938	6.651533	143.2572
6.597967	126.0333	6.518717	138.3764	6.5016	145.6228	6.608167	141.1419	6.536217	141.1885	6.6682	143.2542
6.614633	126.0178	6.535383	138.3687	6.518267	145.6183	6.624833	141.1347	6.552883	141.1849	6.684867	143.2521
6.6313	125.997	6.55205	138.3589	6.534933	145.6128	6.6415	141.1293	6.56955	141.178	6.701533	143.2472
6.647967	125.9836	6.568717	138.3487	6.5516	145.6095	6.658167	141.1273	6.586217	141.1756	6.7182	143.2466
6.664633	125.9672	6.585383	138.342	6.568267	145.6065	6.674833	141.12	6.602883	141.1718	6.734867	143.243
6.6813	125.9511	6.60205	138.3302	6.584933	145.6021	6.6915	141.1197	6.61955	141.1662	6.751533	143.2404
6.697967	125.9329	6.618717	138.3229	6.6016	145.5983	6.708167	141.1131	6.636217	141.1633	6.7682	143.2385
6.714633	125.9175	6.635383	138.3146	6.618267	145.5948	6.724833	141.1073	6.652883	141.1616	6.784867	143.2331
6.7313	125.9008	6.65205	138.3063	6.634933	145.5904	6.7415	141.1015	6.66955	141.1576	6.801533	143.2307
6.747967	125.883	6.668717	138.2971	6.6516	145.5887	6.758167	141.0987	6.686217	141.1509	6.8182	143.2275
6.764633	125.8648	6.685383	138.2903	6.668267	145.5849	6.774833	141.093	6.702883	141.1472	6.834867	143.2261
6.7813	125.8503	6.70205	138.2798	6.684933	145.582	6.7915	141.0897	6.71955	141.1435	6.851533	143.2217
6.797967	125.8332	6.718717	138.2722	6.7016	145.5783	6.808167	141.0873	6.736217	141.1414	6.8682	143.2199
6.814633	125.8217	6.735383	138.2637	6.718267	145.5742	6.824833	141.0829	6.752883	141.134	6.884867	143.2154
6.8313	125.8041	6.75205	138.2556	6.734933	145.5689	6.8415	141.0754	6.76955	141.1288	6.901533	143.2118
6.847967	125.7868	6.768717	138.2476	6.7516	145.5651	6.858167	141.0717	6.786217	141.1269	6.9182	143.2099
6.864633	125.7687	6.785383	138.2377	6.768267	145.5651	6.874833	141.0676	6.802883	141.1226	6.934867	143.2096
6.8813	125.7563	6.80205	138.2322	6.784933	145.5605	6.8915	141.0642	6.81955	141.119	6.951533	143.2047
6.897967	125.7413	6.818717	138.2234	6.8016	145.5562	6.908167	141.0614	6.836217	141.1172	6.9682	143.2026
6.914633	125.7196	6.835383	138.2133	6.818267	145.5543	6.924833	141.0536	6.852883	141.115	6.984867	143.1987
6.9313	125.7043	6.85205	138.207	6.834933	145.5482	6.9415	141.0469	6.86955	141.1091	7.001533	143.1946
6.947967	125.6914	6.868717	138.1988	6.8516	145.5463	6.958167	141.0475	6.886217	141.1088	7.0182	143.1943
6.964633	125.6765	6.885383	138.186	6.868267	145.5433	6.974833	141.0403	6.902883	141.1003	7.034867	143.1902
6.9813	125.6598	6.90205	138.1857	6.884933	145.5399	6.9915	141.0371	6.91955	141.1003	7.051533	143.1865
6.997967	125.6421	6.918717	138.1709	6.9016	145.5354	7.008167	141.0314	6.936217	141.094	7.0682	143.1856
7.014633	125.63	6.935383	138.1657	6.918267	145.5335	7.024833	141.0306	6.952883	141.0883	7.084867	143.183
7.0313	125.6118	6.95205	138.1555	6.934933	145.5305	7.0415	141.0246	6.96955	141.0863	7.101533	143.1786
7.047967	125.5944	6.968717	138.153	6.9516	145.5265	7.058167	141.0222	6.986217	141.0865	7.1182	143.1764
7.064633	125.5791	6.985383	138.1407	6.968267	145.524	7.074833	141.014	7.002883	141.0847	7.134867	143.1758
7.0813	125.565	7.00205	138.1346	6.984933	145.5216	7.0915	141.0114	7.01955	141.0769	7.151533	143.1715
7.097967	125.548	7.018717	138.1316	7.0016	145.5155	7.108167	141.0068	7.036217	141.0766	7.1682	143.1698
7.114633	125.5341	7.035383	138.118	7.018267	145.5122	7.124833	141.0024	7.052883	141.0743	7.184867	143.1668
7.1313	125.5165	7.05205	138.1157	7.034933	145.5074	7.1415	140.9962	7.06955	141.0664	7.201533	143.166
7.147967	125.5073	7.068717	138.1047	7.0516	145.5064	7.158167	140.9929	7.086217	141.0643	7.2182	143.1615
7.164633	125.4853	7.085383	138.0953	7.068267	145.5016	7.174833	140.988	7.102883	141.0613	7.234867	143.1603
7.1813	125.4738	7.10205	138.0915	7.084933	145.4988	7.1915	140.987	7.11955	141.0561	7.251533	143.1569
7.197967	125.4563	7.118717	138.0852	7.1016	145.4958	7.208167	140.9829	7.136217	141.0513	7.2682	143.1543
7.214633	125.4418	7.135383	138.0746	7.118267	145.4942	7.224833	140.98	7.152883	141.0495	7.284867	143.1531
7.2313	125.4286	7.15205	138.0692	7.134933	145.4882	7.2415	140.9762	7.16955	141.0455	7.301533	143.1523
7.247967	125.4134	7.168717	138.0587	7.1516	145.4901	7.258167	140.9721	7.186217	141.0418	7.3182	143.1479
7.264633	125.3978	7.185383	138.0492	7.168267	145.4849	7.274833	140.9691	7.202883	141.0365	7.334867	143.144
7.2813	125.384	7.20205	138.0416	7.184933	145.4775	7.2915	140.9606	7.21955	141.0338	7.351533	143.141
7.297967	125.3676	7.218717	138.0341	7.2016	145.4768	7.308167	140.9593	7.236217	141.0287	7.3682	143.1384
7.314633	125.3531	7.235383	138.0272	7.218267	145.4738	7.324833	140.9558	7.252883	141.0234	7.384867	143.1376
7.3313	125.3399	7.25205	138.0198	7.234933	145.4724	7.3415	140.9497	7.26955	141.0193	7.401533	143.1348
7.347967	125.3261	7.268717	138.013	7.2516	145.4665	7.358167	140.9494	7.286217	141.0187	7.4182	143.1297
7.364633	125.3088	7.285383	138.0029	7.268267	145.4624	7.374833	140.9412	7.302883	141.0123	7.434867	143.1284
7.3813	125.2943	7.30205	137.9954	7.284933	145.4594	7.3915	140.9388	7.31955	141.0096	7.451533	143.1279
7.397967	125.2777	7.318717	137.9863	7.3016	145.4581	7.408167	140.9348	7.336217	141.003	7.4682	143.1244
7.414633	125.2643	7.335383	137.9802	7.318267	145.454	7.424833	140.9304	7.352883	141.0034	7.484867	143.1207
7.4313	125.247	7.35205	137.9721	7.334933	145.451	7.4415	140.9263	7.36955	140.9978	7.501533	143.1191
7.447967	125.2336	7.368717	137.9667	7.3516	145.448	7.458167	140.9237	7.386217	140.9951	7.5182	143.1166
7.464633	125.2198	7.385383	137.9541	7.368267	145.4422	7.474833	140.9165	7.402883	140.9924	7.534867	143.1122
7.4813	125.2072	7.40205	137.9498	7.384933	145.4427	7.4915	140.9136	7.41955	140.9887	7.551533	143.1114
7.497967	125.1915	7.418717	137.9413	7.4016	145.4344	7.508167	140.9083	7.436217	140.9841	7.5682	143.1093
7.514633	125.1765	7.435383	137.9326	7.418267	145.432	7.524833	140.9076	7.452883	140.982	7.584867	143.1085
7.5313	125.1612	7.45205	137.9288	7.434933	145.4294	7.5415	140.9013	7.46955	140.9792	7.601533	143.1049
7.547967	125.1477	7.468717	137.9194	7.4516	145.4255	7.558167	140.8989	7.486217	140.9749	7.6182	143.1033
7.564633	125.1325	7.485383	137.9127	7.468267	145.422	7.574833	140.8943	7.502883	140.9721	7.634867	143.0977
7.5813	125.1192	7.50205	137.9045	7.484933	145.4198	7.5915	140.8892	7.51955	140.967	7.651533	143.0952
7.597967	125.1055	7.518717	137.8979	7.5016	145.4154	7.608167	140.8848	7.536217	140.9643	7.6682	143.0929
7.614633	125.0927	7.535383	137.8886	7.518267	145.4126	7.624833	140.8846	7.552883	140.962	7.684867	143.0902
7.6313	125.0765	7.55205	137.8833	7.534933	145.4101	7.6415	140.8754	7.56955	140.9562	7.701533	143.0878
7.647967	125.0619	7.568717	137.8769	7.5516	145.4031	7.658167	140.8708	7.586217	140.9544	7.7182	143.0867
7.664633	125.0462	7.585383	137.8667	7.568267	145.399	7.674833	140.8702	7.602883	140.9504	7.734867	143.0834
7.6813	125.0336	7.60205	137.8596	7.584933	145.3951	7.6915	140.8656	7.61955	140.9492	7.751533	143.082
7.697967	125.0217	7.618717	137.8547	7.6016	145.3948	7.708167	140.8611	7.636217	140.9426	7.7682	143.0801
7.714633	125.0062	7.635383	137.8464	7.618267	145.3901	7.724833	140.8565	7.652883	140.9394	7.784867	143.0758
7.7313	124.9944	7.65205	137.8389	7.634933	145.3876	7.7415	140.8519	7.66955	140.9375	7.801533	143.0739
7.747967	124.9794	7.668717	137.8355	7.6516	145.3847	7.758167	140.8506	7.686217	140.9338	7.8182	143.0717
7.764633	124.9678	7.685383	137.8262	7.668267	145.3823	7.774833	140.8459	7.702883	140.9272	7.834867	143.0712
7.7813	124.9479	7.70205	137.8191	7.684933	145.3735	7.7915	140.8397	7.71955	140.9254	7.851533	143.0672
7.797967	124.936	7.718717	137.8088	7.7016	145.3776	7.808167	140.8366	7.736217	140.9239	7.8682	143.0662
7.814633	124.9208	7.735383	137.8048	7.718267	145.368	7.824833	140.8335	7.752883	140.9206	7.884867	143.0604
7.8313	124.9071	7.75205	137.7955	7.734933	145.3705	7.8415	140.8315	7.76955	140.9168	7.901533	143.0604
7.847967	124.8941	7.768717	137.7905	7.7516	145.3643	7.858167	140.8233	7.786217	140.9108	7.9182	143.06
7.864633	124.8794	7.785383	137.7815	7.768267	145.3609	7.874833	140.8216	7.802883	140.9078	7.934867	143.0547
7.8813	124.8687	7.80205	137.772	7.784933	145.3538	7.8915	140.8168	7.81955	140.906	7.951533	143.0516
7.897967	124.8542	7.818717	137.7666	7.8016	145.3554	7.908167	140.8131	7.836217	140.9028	7.9682	143.0504
7.914633	124.8366	7.835383	137.7589	7.818267	145.3542	7.924833	140.8121	7.852883	140.8978	7.984867	143.0475
7.9313	124.8257	7.85205	137.7533	7.834933	145.3497	7.9415	140.8088	7.86955	140.8957	8.001533	143.0444
7.947967	124.8168	7.868717	137.7466	7.8516	145.3447	7.958167	140.8044	7.886217	140.8952	8.0182	143.0435
7.964633	124.7968	7.885383	137.7365	7.868267	145.3423	7.974833	140.7992	7.902883	140.8892	8.034867	143.0386
7.9813	124.7837	7.90205	137.7283	7.884933	145.3417	7.9915	140.7967	7.91955	140.8847	8.051533	143.0386
7.997967	124.7715	7.918717	137.7252	7.9016	145.3369	8.008167	140.7924	7.936217	140.8821	8.0682	143.0351
8.014633	124.7577	7.935383	137.7147	7.918267	145.3351	8.024833	140.7861	7.952883	140.8804	8.084867	143.0332
8.0313	124.7428	7.95205	137.7098	7.934933	145.329	8.0415	140.7843	7.96955	140.8761	8.101533	143.0303
8.047967	124.7296	7.968717	137.7023	7.9516	145.3257	8.058167	140.781	7.986217	140.8722	8.1182	143.0289
8.064633	124.7157	7.985383	137.6935	7.968267	145.3238	8.074833	140.7739	8.002883	140.8682	8.134867	143.0271
8.0813	124.7045	8.00205	137.6894	7.984933	145.3161	8.0915	140.7725	8.01955	140.8644	8.151533	143.0248
8.097967	124.6918	8.018717	137.6784	8.0016	145.315	8.108167	140.7662	8.036217	140.8623	8.1682	143.0243
8.114633	124.6772	8.035383	137.6732	8.018267	145.3145	8.124833	140.7641	8.052883	140.8572	8.184867	143.0199
8.1313	124.6629	8.05205	137.6661	8.034933	145.309	8.1415	140.7584	8.06955	140.8555	8.201533	143.0174

8.147967	124.6516	8.068717	137.662	8.0516	145.3047	8.158167	140.7554	8.086217	140.8526	8.2182	143.0157
8.164633	124.6389	8.085383	137.652	8.068267	145.302	8.174833	140.7523	8.102883	140.8476	8.234867	143.0129
8.1813	124.6252	8.10205	137.6482	8.084933	145.2973	8.1915	140.7485	8.11955	140.8457	8.251533	143.0093
8.197967	124.6103	8.118717	137.6367	8.1016	145.2953	8.208167	140.7427	8.136217	140.8424	8.2682	143.0089
8.214633	124.5984	8.135383	137.6327	8.118267	145.2928	8.224833	140.7398	8.152883	140.8407	8.284867	143.0058
8.2313	124.5868	8.15205	137.6276	8.134933	145.2888	8.2415	140.7351	8.16955	140.8358	8.301533	143.0044
8.247967	124.5703	8.168717	137.6194	8.1516	145.2871	8.258167	140.7299	8.186217	140.8321	8.3182	143.0026
8.264633	124.5588	8.185383	137.6134	8.168267	145.2834	8.274833	140.7248	8.202883	140.8292	8.334867	142.9994
8.2813	124.5454	8.20205	137.6069	8.184933	145.2778	8.2915	140.7233	8.21955	140.8266	8.351533	142.9974
8.297967	124.5329	8.218717	137.5982	8.2016	145.2742	8.308167	140.7191	8.236217	140.8239	8.3682	142.9955
8.314633	124.5183	8.235383	137.5933	8.218267	145.2711	8.324833	140.7149	8.252883	140.8197	8.384867	142.9941
8.3313	124.5027	8.25205	137.5884	8.234933	145.27	8.3415	140.7107	8.26955	140.8153	8.401533	142.9906
8.347967	124.4943	8.268717	137.5772	8.2516	145.2678	8.358167	140.7064	8.286217	140.813	8.4182	142.9898
8.364633	124.4776	8.285383	137.5716	8.268267	145.2628	8.374833	140.7041	8.302883	140.8101	8.434867	142.9889
8.3813	124.4654	8.30205	137.5644	8.284933	145.262	8.3915	140.7006	8.31955	140.809	8.451533	142.982
8.397967	124.4551	8.318717	137.5571	8.3016	145.258	8.408167	140.699	8.336217	140.8006	8.4682	142.9828
8.414633	124.4381	8.335383	137.5492	8.318267	145.2566	8.424833	140.6963	8.352883	140.8021	8.484867	142.9829
8.4313	124.4296	8.35205	137.5442	8.334933	145.2519	8.4415	140.6908	8.36955	140.8001	8.501533	142.98
8.447967	124.415	8.368717	137.5376	8.3516	145.2498	8.458167	140.6861	8.386217	140.7921	8.5182	142.9784
8.464633	124.4023	8.385383	137.5307	8.368267	145.2459	8.474833	140.6827	8.402883	140.7919	8.534867	142.9733
8.4813	124.3899	8.40205	137.5251	8.384933	145.2419	8.4915	140.6792	8.41955	140.7912	8.551533	142.9734
8.497967	124.3766	8.418717	137.5192	8.4016	145.2412	8.508167	140.6749	8.436217	140.7841	8.5682	142.9733
8.514633	124.3641	8.435383	137.5113	8.418267	145.2389	8.524833	140.6708	8.452883	140.7823	8.584867	142.9714
8.5313	124.3534	8.45205	137.5059	8.434933	145.234	8.5415	140.6695	8.46955	140.7821	8.601533	142.9664
8.547967	124.3369	8.468717	137.5007	8.4516	145.2324	8.558167	140.6653	8.486217	140.7775	8.6182	142.9648
8.564633	124.3232	8.485383	137.4926	8.468267	145.2268	8.574833	140.6587	8.502883	140.7742	8.634867	142.9625
8.5813	124.3145	8.50205	137.4851	8.484933	145.2297	8.5915	140.6573	8.51955	140.7708	8.651533	142.9612
8.597967	124.3	8.518717	137.4804	8.5016	145.2223	8.608167	140.6549	8.536217	140.7675	8.6682	142.9588
8.614633	124.2837	8.535383	137.4721	8.518267	145.2197	8.624833	140.6498	8.552883	140.7657	8.684867	142.9559
8.6313	124.2757	8.55205	137.4651	8.534933	145.2183	8.6415	140.6499	8.56955	140.7642	8.701533	142.9538
8.647967	124.2635	8.568717	137.4598	8.5516	145.2181	8.658167	140.6401	8.586217	140.7581	8.7182	142.9532
8.664633	124.2484	8.585383	137.4541	8.568267	145.2124	8.674833	140.6345	8.602883	140.7558	8.734867	142.9508
8.6813	124.2408	8.60205	137.4459	8.584933	145.2082	8.6915	140.6365	8.61955	140.7535	8.751533	142.9496
8.697967	124.2267	8.618717	137.4376	8.6016	145.2062	8.708167	140.6305	8.636217	140.7504	8.7682	142.9461
8.714633	124.213	8.635383	137.4352	8.618267	145.2027	8.724833	140.6252	8.652883	140.7486	8.784867	142.9453
8.7313	124.2008	8.65205	137.4258	8.634933	145.1986	8.7415	140.6231	8.66955	140.7458	8.801533	142.942
8.747967	124.1904	8.668717	137.4203	8.6516	145.1969	8.758167	140.6243	8.686217	140.7407	8.8182	142.9412
8.764633	124.1764	8.685383	137.4139	8.668267	145.1955	8.774833	140.6145	8.702883	140.7378	8.834867	142.9379
8.7813	124.1629	8.70205	137.4064	8.684933	145.1889	8.7915	140.6124	8.71955	140.7365	8.851533	142.9373
8.797967	124.1506	8.718717	137.4015	8.7016	145.1864	8.808167	140.6097	8.736217	140.7317	8.8682	142.9325
8.814633	124.1435	8.735383	137.3961	8.718267	145.1875	8.824833	140.6062	8.752883	140.7312	8.884867	142.9327
8.8313	124.1291	8.75205	137.3889	8.734933	145.182	8.8415	140.6016	8.76955	140.7265	8.901533	142.9309
8.847967	124.1185	8.768717	137.3812	8.7516	145.1801	8.858167	140.5985	8.786217	140.7247	8.9182	142.9284
8.864633	124.1061	8.785383	137.3756	8.768267	145.1753	8.874833	140.5956	8.802883	140.7205	8.934867	142.925
8.8813	124.0933	8.80205	137.3671	8.784933	145.1732	8.8915	140.5935	8.81955	140.7195	8.951533	142.9245
8.897967	124.082	8.818717	137.3637	8.8016	145.1709	8.908167	140.5879	8.836217	140.7191	8.9682	142.9224
8.914633	124.0711	8.835383	137.3575	8.818267	145.1677	8.924833	140.5864	8.852883	140.7112	8.984867	142.9205
8.9313	124.0568	8.85205	137.3505	8.834933	145.1615	8.9415	140.5795	8.86955	140.7085	9.001533	142.9169
8.947967	124.0447	8.868717	137.3452	8.8516	145.1604	8.958167	140.5761	8.886217	140.7073	9.0182	142.9155
8.964633	124.033	8.885383	137.3376	8.868267	145.1563	8.974833	140.5742	8.902883	140.7054	9.034867	142.9134
8.9813	124.0201	8.90205	137.3289	8.884933	145.1522	8.9915	140.5702	8.91955	140.7039	9.051533	142.9118
8.997967	124.0083	8.918717	137.3253	8.9016	145.1527	9.008167	140.567	8.936217	140.6985	9.0682	142.9098
9.014633	123.9982	8.935383	137.3183	8.918267	145.1491	9.024833	140.5619	8.952883	140.699	9.084867	142.9068
9.0313	123.9845	8.95205	137.3081	8.934933	145.1449	9.0415	140.5562	8.96955	140.6941	9.101533	142.9053
9.047967	123.9741	8.968717	137.3064	8.9516	145.1418	9.058167	140.558	8.986217	140.691	9.1182	142.9053
9.064633	123.9615	8.985383	137.2965	8.968267	145.1396	9.074833	140.5541	9.002883	140.6902	9.134867	142.9018
9.0813	123.95	9.00205	137.2914	8.984933	145.1382	9.0915	140.5481	9.01955	140.6865	9.151533	142.9006
9.097967	123.9377	9.018717	137.2865	9.0016	145.1359	9.108167	140.5438	9.036217	140.6856	9.1682	142.8981
9.114633	123.9249	9.035383	137.2799	9.018267	145.1305	9.124833	140.5423	9.052883	140.6825	9.184867	142.8964
9.1313	123.915	9.05205	137.2722	9.034933	145.1279	9.1415	140.5374	9.06955	140.6792	9.201533	142.8938
9.147967	123.9001	9.068717	137.2699	9.0516	145.1239	9.158167	140.5347	9.086217	140.6739	9.2182	142.8934
9.164633	123.8882	9.085383	137.2593	9.068267	145.1237	9.174833	140.5265	9.102883	140.6751	9.234867	142.8892
9.1813	123.8801	9.10205	137.2568	9.084933	145.1177	9.1915	140.5275	9.11955	140.6708	9.251533	142.8882
9.197967	123.8666	9.118717	137.249	9.1016	145.1176	9.208167	140.5225	9.136217	140.6663	9.2682	142.8869
9.214633	123.8559	9.135383	137.2444	9.118267	145.1111	9.224833	140.5191	9.152883	140.6636	9.284867	142.8853
9.2313	123.842	9.15205	137.236	9.134933	145.1122	9.2415	140.5174	9.16955	140.6628	9.301533	142.884
9.247967	123.832	9.168717	137.2261	9.1516	145.1061	9.258167	140.5139	9.186217	140.6606	9.3182	142.8831
9.264633	123.8203	9.185383	137.2255	9.168267	145.104	9.274833	140.5106	9.202883	140.6557	9.334867	142.8789
9.2813	123.805	9.20205	137.2149	9.184933	145.1	9.2915	140.503	9.21955	140.6531	9.351533	142.8799
9.297967	123.7963	9.218717	137.2085	9.2016	145.0991	9.308167	140.506	9.236217	140.6479	9.3682	142.8741
9.314633	123.7847	9.235383	137.2025	9.218267	145.0964	9.324833	140.4983	9.252883	140.6472	9.384867	142.8745
9.3313	123.7733	9.25205	137.1983	9.234933	145.0928	9.3415	140.4951	9.26955	140.6435	9.401533	142.8712
9.347967	123.7618	9.268717	137.1954	9.2516	145.0878	9.358167	140.4888	9.286217	140.642	9.4182	142.8683
9.364633	123.7464	9.285383	137.1846	9.268267	145.0868	9.374833	140.4859	9.302883	140.6371	9.434867	142.8697
9.3813	123.7389	9.30205	137.183	9.284933	145.0849	9.3915	140.4828	9.31955	140.6364	9.451533	142.867
9.397967	123.728	9.318717	137.1773	9.3016	145.0801	9.408167	140.4786	9.336217	140.6335	9.4682	142.8664
9.414633	123.7164	9.335383	137.1688	9.318267	145.0777	9.424833	140.4763	9.352883	140.6276	9.484867	142.8603
9.4313	123.7026	9.35205	137.1602	9.334933	145.0751	9.4415	140.473	9.36955	140.6254	9.501533	142.8596
9.447967	123.6936	9.368717	137.1569	9.3516	145.0716	9.458167	140.4707	9.386217	140.626	9.5182	142.8564
9.464633	123.6834	9.385383	137.1477	9.368267	145.0699	9.474833	140.4661	9.402883	140.6219	9.534867	142.8564
9.4813	123.6712	9.40205	137.1443	9.384933	145.0683	9.4915	140.4622	9.41955	140.617	9.551533	142.8554
9.497967	123.6583	9.418717	137.1403	9.4016	145.0677	9.508167	140.4597	9.436217	140.6148	9.5682	142.8487
9.514633	123.6501	9.435383	137.1316	9.418267	145.0631	9.524833	140.456	9.452883	140.6135	9.584867	142.8501
9.5313	123.6352	9.45205	137.1267	9.434933	145.0571	9.5415	140.4513	9.46955	140.6097	9.601533	142.8491
9.547967	123.6268	9.468717	137.1172	9.4516	145.0583	9.558167	140.4491	9.486217	140.6088	9.6182	142.8469
9.564633	123.6171	9.485383	137.1119	9.468267	145.0537	9.574833	140.4494	9.502883	140.6027	9.634867	142.8461
9.5813	123.6032	9.50205	137.1078	9.484933	145.0513	9.5915	140.4401	9.51955	140.6023	9.651533	142.8427
9.597967	123.5908	9.518717	137.1018	9.5016	145.0471	9.608167	140.44	9.536217	140.5973	9.6682	142.8386
9.614633	123.5812	9.535383	137.0968	9.518267	145.0456	9.624833	140.4359	9.552883	140.5974	9.684867	142.8409
9.6313	123.5701	9.55205	137.0904	9.534933	145.041	9.6415	140.435	9.56955	140.5929	9.701533	142.8388
9.647967	123.5584	9.568717	137.0865	9.5516	145.0388	9.658167	140.4301	9.586217	140.5919	9.7182	142.8343
9.664633	123.5469	9.585383	137.0796	9.568267	145.0388	9.674833	140.4232	9.602883	140.5877	9.734867	142.8324
9.6813	123.5386	9.60205	137.0699	9.584933	145.0365	9.6915	140.4194	9.61955	140.5863	9.751533	142.8335
9.697967	123.5235	9.618717	137.0671	9.6016	145.0352	9.708167	140.4197	9.636217	140.5811	9.7682	142.8309
9.714633	123.5147	9.635383	137.0625	9.618267	145.028	9.724833	140.4154	9.652883	140.5803	9.784867	142.8297
9.7313	123.5033	9.65205	137.0537	9.634933	145.0285	9.7415	140.4155	9.66955	140.5785	9.801533	142.8246
9.747967	123.4947	9.668717	137.0467	9.6516	145.0268	9.758167	140.4057	9.686217	140.5749	9.8182	142.8243

9.764633	123.4799	9.685383	137.0415	9.668267	145.0246	9.774833	140.406	9.702883	140.5728	9.834867	142.8237
9.7813	123.4731	9.70205	137.0387	9.684933	145.0163	9.7915	140.4003	9.71955	140.5707	9.851533	142.8211
9.797967	123.4598	9.718717	137.0313	9.7016	145.0159	9.808167	140.3986	9.736217	140.5684	9.8682	142.8175
9.814633	123.4504	9.735383	137.0256	9.718267	145.0169	9.824833	140.392	9.752883	140.5656	9.884867	142.8172
9.8313	123.4402	9.75205	137.0192	9.734933	145.0134	9.8415	140.3904	9.76955	140.5639	9.901533	142.8144
9.847967	123.429	9.768717	137.0153	9.7516	145.0092	9.858167	140.3842	9.786217	140.5607	9.9182	142.8134
9.864633	123.4185	9.785383	137.0101	9.768267	145.0073	9.874833	140.3778	9.802883	140.5578	9.934867	142.8097
9.8813	123.4088	9.80205	137.002	9.784933	145.0057	9.8915	140.3776	9.81955	140.5565	9.951533	142.8089
9.897967	123.3927	9.818717	136.9965	9.8016	145.0024	9.908167	140.3769	9.836217	140.5524	9.9682	142.806
9.914633	123.387	9.835383	136.9939	9.818267	144.9999	9.924833	140.3692	9.852883	140.5507	9.984867	142.805
9.9313	123.382	9.85205	136.9847	9.834933	144.9958	9.9415	140.3662	9.86955	140.5502	10.00153	142.8018
9.947967	123.3582	9.868717	136.981	9.8516	144.9864	9.958167	140.3612	9.886217	140.545	10.0182	142.8
9.964633	123.3492	9.885383	136.9755	9.868267	144.9869	9.974833	140.3628	9.902883	140.5431	10.03487	142.7986
9.9813	123.3474	9.90205	136.9715	9.884933	144.9865	9.9915	140.3559	9.91955	140.5411	10.05153	142.7974
9.997967	123.332	9.918717	136.9665	9.9016	144.9811	10.00817	140.3579	9.936217	140.5383	10.0682	142.7938
10.01463	123.3247	9.935383	136.9553	9.918267	144.9805	10.02483	140.3539	9.952883	140.5352	10.08487	142.7917
10.0313	123.3114	9.95205	136.9561	9.934933	144.9782	10.0415	140.348	9.96955	140.5335	10.10153	142.79
10.04797	123.3002	9.968717	136.951	9.9516	144.9753	10.05817	140.3471	9.986217	140.5317	10.1182	142.7889
10.06463	123.2873	9.985383	136.9446	9.968267	144.9721	10.07483	140.3397	10.00288	140.5287	10.13487	142.7845
10.0813	123.2796	10.00205	136.94	9.984933	144.9714	10.0915	140.336	10.01955	140.527	10.15153	142.7855
10.09797	123.2704	10.01872	136.9378	10.0016	144.9631	10.10817	140.3344	10.03622	140.5248	10.1682	142.7818
10.11463	123.2585	10.03538	136.9291	10.01827	144.9651	10.12483	140.3298	10.05288	140.523	10.18487	142.7812
10.1313	123.2484	10.05205	136.9245	10.03493	144.9624	10.1415	140.3263	10.06955	140.5195	10.20153	142.778
10.14797	123.2409	10.06872	136.9234	10.0516	144.9562	10.15817	140.3222	10.08622	140.5189	10.2182	142.7764
10.16463	123.2292	10.08538	136.9161	10.06827	144.9573	10.17483	140.3187	10.10288	140.5181	10.23487	142.7725
10.1813	123.2224	10.10205	136.9036	10.08493	144.951	10.1915	140.3149	10.11955	140.5184	10.25153	142.7724
10.19797	123.2041	10.11872	136.904	10.1016	144.9538	10.20817	140.3138	10.13622	140.5133	10.2682	142.7688
10.21463	123.198	10.13538	136.8981	10.11827	144.9519	10.22483	140.3108	10.15288	140.5086	10.28487	142.7672
10.2313	123.1886	10.15205	136.8929	10.13493	144.945	10.2415	140.3048	10.16955	140.5106	10.30153	142.7649
10.24797	123.176	10.16872	136.8875	10.1516	144.9439	10.25817	140.3039	10.18622	140.5067	10.3182	142.7629
10.26463	123.1695	10.18538	136.8816	10.16827	144.942	10.27483	140.3056	10.20288	140.4988	10.33487	142.7615
10.2813	123.1606	10.20205	136.8791	10.18493	144.9391	10.2915	140.2975	10.21955	140.5021	10.35153	142.7603
10.29797	123.1446	10.21872	136.872	10.2016	144.9362	10.30817	140.2991	10.23622	140.4978	10.3682	142.756
10.31463	123.1355	10.23538	136.8706	10.21827	144.9316	10.32483	140.2935	10.25288	140.4925	10.38487	142.7566
10.3313	123.1275	10.25205	136.8644	10.23493	144.9283	10.3415	140.2914	10.26955	140.4899	10.40153	142.7526
10.34797	123.117	10.26872	136.8622	10.2516	144.9256	10.35817	140.2862	10.28622	140.4887	10.4182	142.7538
10.36463	123.1055	10.28538	136.8572	10.26827	144.9241	10.37483	140.2848	10.30288	140.4844	10.43487	142.7523
10.3813	123.098	10.30205	136.8508	10.28493	144.9212	10.3915	140.2802	10.31955	140.4827	10.45153	142.7479
10.39797	123.0852	10.31872	136.8471	10.3016	144.918	10.40817	140.2795	10.33622	140.4781	10.4682	142.7484
10.41463	123.0782	10.33538	136.8415	10.31827	144.9145	10.42483	140.2743	10.35288	140.4779	10.48487	142.7455
10.4313	123.0653	10.35205	136.8358	10.33493	144.912	10.4415	140.271	10.36955	140.4733	10.50153	142.7421
10.44797	123.054	10.36872	136.8342	10.3516	144.9111	10.45817	140.2686	10.38622	140.4716	10.5182	142.7411
10.46463	123.0441	10.38538	136.8275	10.36827	144.9079	10.47483	140.2649	10.40288	140.4689	10.53487	142.741
10.4813	123.0361	10.40205	136.8207	10.38493	144.905	10.4915	140.2611	10.41955	140.4668	10.55153	142.7395
10.49797	123.0267	10.41872	136.8163	10.4016	144.9022	10.50817	140.2554	10.43622	140.4656	10.5682	142.7381
10.51463	123.0156	10.43538	136.8123	10.41827	144.9027	10.52483	140.2508	10.45288	140.4633	10.58487	142.7357
10.5313	123.0045	10.45205	136.8056	10.43493	144.8967	10.5415	140.2495	10.46955	140.459	10.60153	142.7318
10.54797	122.9989	10.46872	136.8029	10.4516	144.8914	10.55817	140.2455	10.48622	140.4582	10.6182	142.7322
10.56463	122.9879	10.48538	136.7941	10.46827	144.891	10.57483	140.2423	10.50288	140.455	10.63487	142.7298
10.5813	122.9707	10.50205	136.7942	10.48493	144.8887	10.5915	140.2374	10.51955	140.4517	10.65153	142.7266
10.59797	122.9655	10.51872	136.7843	10.5016	144.8873	10.60817	140.2354	10.53622	140.4485	10.6682	142.7271
10.61463	122.9563	10.53538	136.7798	10.51827	144.8839	10.62483	140.2339	10.55288	140.4486	10.68487	142.7246
10.6313	122.9442	10.55205	136.7754	10.53493	144.8853	10.6415	140.2316	10.56955	140.4441	10.70153	142.7247
10.64797	122.9365	10.56872	136.7721	10.5516	144.8808	10.65817	140.2251	10.58622	140.4403	10.7182	142.7198
10.66463	122.9277	10.58538	136.763	10.56827	144.8797	10.67483	140.2214	10.60288	140.4384	10.73487	142.7211
10.6813	122.9164	10.60205	136.7611	10.58493	144.88	10.6915	140.2225	10.61955	140.4373	10.75153	142.719
10.69797	122.9071	10.61872	136.7536	10.6016	144.8738	10.70817	140.2163	10.63622	140.4358	10.7682	142.7168
10.71463	122.8936	10.63538	136.7499	10.61827	144.8739	10.72483	140.2136	10.65288	140.4313	10.78487	142.7154
10.7313	122.8851	10.65205	136.746	10.63493	144.8697	10.7415	140.2103	10.66955	140.4294	10.80153	142.7172
10.74797	122.8788	10.66872	136.7428	10.6516	144.8666	10.75817	140.2082	10.68622	140.4259	10.8182	142.7134
10.76463	122.868	10.68538	136.7347	10.66827	144.8619	10.77483	140.2015	10.70288	140.4246	10.83487	142.7115
10.7813	122.859	10.70205	136.7305	10.68493	144.8613	10.7915	140.1987	10.71955	140.4217	10.85153	142.7099
10.79797	122.8481	10.71872	136.7283	10.7016	144.859	10.80817	140.1968	10.73622	140.417	10.8682	142.7069
10.81463	122.8366	10.73538	136.7236	10.71827	144.8545	10.82483	140.1949	10.75288	140.4167	10.88487	142.7031
10.8313	122.8287	10.75205	136.7178	10.73493	144.8522	10.8415	140.1904	10.76955	140.4149	10.90153	142.7031
10.84797	122.8157	10.76872	136.7113	10.7516	144.8507	10.85817	140.1859	10.78622	140.4109	10.9182	142.7034
10.86463	122.8097	10.78538	136.708	10.76827	144.8492	10.87483	140.1826	10.80288	140.4083	10.93487	142.6994
10.8813	122.7971	10.80205	136.7047	10.78493	144.8454	10.8915	140.1797	10.81955	140.4051	10.95153	142.6984
10.89797	122.7898	10.81872	136.6984	10.8016	144.8442	10.90817	140.1752	10.83622	140.4017	10.9682	142.6974
10.91463	122.7774	10.83538	136.6947	10.81827	144.8395	10.92483	140.1703	10.85288	140.4012	10.98487	142.6971
10.9313	122.7696	10.85205	136.6891	10.83493	144.839	10.9415	140.1704	10.86955	140.3993	11.00153	142.696
10.94797	122.7614	10.86872	136.6905	10.8516	144.8339	10.95817	140.1671	10.88622	140.3948	11.0182	142.6948
10.96463	122.7524	10.88538	136.6832	10.86827	144.8325	10.97483	140.1632	10.90288	140.3946	11.03487	142.6933
10.9813	122.7432	10.90205	136.678	10.88493	144.831	10.9915	140.1606	10.91955	140.3924	11.05153	142.6909
10.99797	122.7333	10.91872	136.6748	10.9016	144.8303	11.00817	140.1578	10.93622	140.3898	11.0682	142.6889
11.01463	122.7226	10.93538	136.6714	10.91827	144.8252	11.02483	140.1522	10.95288	140.3904	11.08487	142.6886
11.0313	122.7137	10.95205	136.6657	10.93493	144.8239	11.0415	140.1497	10.96955	140.3912	11.10153	142.6847
11.04797	122.7032	10.96872	136.6651	10.9516	144.8219	11.05817	140.1476	10.98622	140.3834	11.1182	142.6849
11.06463	122.6945	10.98538	136.6589	10.96827	144.8193	11.07483	140.144	11.00288	140.3835	11.13487	142.6814
11.0813	122.684	11.00205	136.655	10.98493	144.8187	11.0915	140.1417	11.01955	140.38	11.15153	142.68
11.09797	122.6709	11.01872	136.6506	11.0016	144.8145	11.10817	140.1402	11.03622	140.379	11.1682	142.6779
11.11463	122.6637	11.03538	136.6442	11.01827	144.8108	11.12483	140.1356	11.05288	140.3787	11.18487	142.6774
11.1313	122.6536	11.05205	136.6432	11.03493	144.8084	11.1415	140.1377	11.06955	140.3728	11.20153	142.6761
11.14797	122.6431	11.06872	136.6382	11.0516	144.8084	11.15817	140.1325	11.08622	140.3731	11.2182	142.6755
11.16463	122.633	11.08538	136.6333	11.06827	144.8016	11.17483	140.1283	11.10288	140.3687	11.23487	142.6726
11.1813	122.6233	11.10205	136.632	11.08493	144.8008	11.1915	140.1272	11.11955	140.3682	11.25153	142.6704
11.19797	122.6189	11.11872	136.6265	11.1016	144.7981	11.20817	140.1273	11.13622	140.3636	11.2682	142.6702
11.21463	122.6051	11.13538	136.6213	11.11827	144.7946	11.22483	140.1218	11.15288	140.3605	11.28487	142.669
11.2313	122.5938	11.15205	136.6141	11.13493	144.7935	11.2415	140.1206	11.16955	140.3609	11.30153	142.6681
11.24797	122.5869	11.16872	136.6151	11.1516	144.7894	11.25817	140.1142	11.18622	140.3566	11.3182	142.6657
11.26463	122.574	11.18538	136.6069	11.16827	144.79	11.27483	140.1098	11.20288	140.3529	11.33487	142.6638
11.2813	122.566	11.20205	136.6061	11.18493	144.7853	11.2915	140.1099	11.21955	140.3515	11.35153	142.6616
11.29797	122.555	11.21872	136.5998	11.2016	144.7871	11.30817	140.1046	11.23622	140.348	11.3682	142.6607
11.31463	122.5462	11.23538	136.5968	11.21827	144.7809	11.32483	140.0996	11.25288	140.3463	11.38487	142.6584
11.3313	122.5408	11.25205	136.5919	11.23493	144.779	11.3415	140.1029	11.26955	140.3456	11.40153	142.6582
11.34797	122.528	11.26872	136.5898	11.2516	144.779	11.35817	140.0965	11.28622	140.3411	11.4182	142.658
11.36463	122.5193	11.28538	136.5829	11.26827	144.7749	11.37483	140.0928	11.30288	140.3352	11.43487	142.6556

11.3813	122.5069	11.30205	136.5822	11.28493	144.7711	11.3915	140.0911	11.31955	140.3332	11.45153	142.6539
11.39797	122.4961	11.31872	136.5753	11.3016	144.7689	11.40817	140.0877	11.33622	140.3332	11.4682	142.6556
11.41463	122.4903	11.33538	136.5721	11.31827	144.7658	11.42483	140.0829	11.35288	140.33	11.48487	142.6538
11.4313	122.4778	11.35205	136.5689	11.33493	144.762	11.4415	140.0816	11.36955	140.3263	11.50153	142.651
11.44797	122.4719	11.36872	136.5674	11.3516	144.7593	11.45817	140.077	11.38622	140.3232	11.5182	142.6484
11.46463	122.4643	11.38538	136.5615	11.36827	144.7569	11.47483	140.0737	11.40288	140.3204	11.53487	142.6509
11.4813	122.4499	11.40205	136.5591	11.38493	144.7553	11.4915	140.069	11.41955	140.3206	11.55153	142.648
11.49797	122.4456	11.41872	136.5565	11.4016	144.7543	11.50817	140.0677	11.43622	140.3166	11.5682	142.6466
11.51463	122.4309	11.43538	136.5541	11.41827	144.7495	11.52483	140.0619	11.45288	140.3173	11.58487	142.6439
11.5313	122.426	11.45205	136.5477	11.43493	144.747	11.5415	140.0594	11.46955	140.3141	11.60153	142.6416
11.54797	122.4162	11.46872	136.5485	11.4516	144.743	11.55817	140.0564	11.48622	140.3126	11.6182	142.6431
11.56463	122.405	11.48538	136.5402	11.46827	144.7429	11.57483	140.0518	11.50288	140.3089	11.63487	142.6402
11.5813	122.4033	11.50205	136.5408	11.48493	144.7395	11.5915	140.0484	11.51955	140.309	11.65153	142.6384
11.59797	122.3885	11.51872	136.5324	11.5016	144.7412	11.60817	140.0476	11.53622	140.3077	11.6682	142.6358
11.61463	122.3794	11.53538	136.5293	11.51827	144.7354	11.62483	140.0415	11.55288	140.3046	11.68487	142.6368
11.6313	122.3683	11.55205	136.525	11.53493	144.7322	11.6415	140.0384	11.56955	140.3022	11.70153	142.6355
11.64797	122.358	11.56872	136.5249	11.5516	144.7326	11.65817	140.0385	11.58622	140.3029	11.7182	142.6337
11.66463	122.3489	11.58538	136.5239	11.56827	144.7322	11.67483	140.0345	11.60288	140.3007	11.73487	142.6291
11.6813	122.3398	11.60205	136.5161	11.58493	144.7267	11.6915	140.029	11.61955	140.298	11.75153	142.6303
11.69797	122.3305	11.61872	136.5153	11.6016	144.7254	11.70817	140.0294	11.63622	140.295	11.7682	142.6298
11.71463	122.3216	11.63538	136.51	11.61827	144.72	11.72483	140.0225	11.65288	140.2939	11.78487	142.6296
11.7313	122.3103	11.65205	136.5067	11.63493	144.7227	11.7415	140.0205	11.66955	140.2896	11.80153	142.6281
11.74797	122.3013	11.66872	136.5007	11.6516	144.72	11.75817	140.017	11.68622	140.2876	11.8182	142.6253
11.76463	122.2939	11.68538	136.4974	11.66827	144.7176	11.77483	140.0153	11.70288	140.2867	11.83487	142.6255
11.7813	122.2857	11.70205	136.4934	11.68493	144.7141	11.7915	140.0132	11.71955	140.2855	11.85153	142.6226
11.79797	122.2743	11.71872	136.4881	11.7016	144.7119	11.80817	140.0083	11.73622	140.2823	11.8682	142.6189
11.81463	122.2647	11.73538	136.4844	11.71827	144.71	11.82483	140.0058	11.75288	140.277	11.88487	142.6187
11.8313	122.2558	11.75205	136.4774	11.73493	144.7127	11.8415	140.0035	11.76955	140.2796	11.90153	142.6175
11.84797	122.2443	11.76872	136.4755	11.7516	144.708	11.85817	140.0006	11.78622	140.275	11.9182	142.6142
11.86463	122.2407	11.78538	136.4711	11.76827	144.7066	11.87483	139.9967	11.80288	140.2743	11.93487	142.6132
11.8813	122.2256	11.80205	136.4635	11.78493	144.7007	11.8915	139.9965	11.81955	140.2702	11.95153	142.6131
11.89797	122.2176	11.81872	136.4629	11.8016	144.7051	11.90817	139.9916	11.83622	140.2697	11.9682	142.6106
11.91463	122.2086	11.83538	136.4543	11.81827	144.6976	11.92483	139.9909	11.85288	140.2661	11.98487	142.6088
11.9313	122.2009	11.85205	136.4507	11.83493	144.6978	11.9415	139.9878	11.86955	140.2661	12.00153	142.6098
11.94797	122.1895	11.86872	136.4497	11.8516	144.6937	11.95817	139.9827	11.88622	140.2634	12.0182	142.6056
11.96463	122.183	11.88538	136.4409	11.86827	144.6921	11.97483	139.9828	11.90288	140.2605	12.03487	142.6038
11.9813	122.1739	11.90205	136.4394	11.88493	144.6863	11.9915	139.9782	11.91955	140.2586	12.05153	142.6022
11.99797	122.1615	11.91872	136.4332	11.9016	144.6849	12.00817	139.9786	11.93622	140.259	12.0682	142.6006
12.01463	122.1538	11.93538	136.4311	11.91827	144.685	12.02483	139.9747	11.95288	140.2527	12.08487	142.6002
12.0313	122.1474	11.95205	136.4241	11.93493	144.6823	12.0415	139.9684	11.96955	140.255	12.10153	142.5973
12.04797	122.1367	11.96872	136.4213	11.9516	144.6766	12.05817	139.968	11.98622	140.251	12.1182	142.5977
12.06463	122.1284	11.98538	136.4149	11.96827	144.675	12.07483	139.9628	12.00288	140.2484	12.13487	142.5932
12.0813	122.1177	12.00205	136.4102	11.98493	144.6673	12.0915	139.9621	12.01955	140.2468	12.15153	142.5959
12.09797	122.1127	12.01872	136.4038	12.0016	144.6689	12.10817	139.9589	12.03622	140.2452	12.1682	142.5912
12.11463	122.1019	12.03538	136.4015	12.01827	144.6662	12.12483	139.9546	12.05288	140.2445	12.18487	142.5895
12.1313	122.0907	12.05205	136.3965	12.03493	144.6616	12.1415	139.9545	12.06955	140.2405	12.20153	142.5884
12.14797	122.0857	12.06872	136.3909	12.0516	144.6652	12.15817	139.9507	12.08622	140.2404	12.2182	142.5865
12.16463	122.073	12.08538	136.3889	12.06827	144.6587	12.17483	139.9465	12.10288	140.2382	12.23487	142.5836
12.1813	122.0661	12.10205	136.3849	12.08493	144.6537	12.1915	139.9429	12.11955	140.235	12.25153	142.5834
12.19797	122.0576	12.11872	136.384	12.1016	144.6524	12.20817	139.943	12.13622	140.2332	12.2682	142.5826
12.21463	122.0437	12.13538	136.3745	12.11827	144.6493	12.22483	139.9385	12.15288	140.2307	12.28487	142.581
12.2313	122.0378	12.15205	136.3727	12.13493	144.6466	12.2415	139.9379	12.16955	140.228	12.30153	142.578
12.24797	122.0296	12.16872	136.3704	12.1516	144.643	12.25817	139.9342	12.18622	140.2226	12.3182	142.5778
12.26463	122.0188	12.18538	136.3628	12.16827	144.6413	12.27483	139.9305	12.20288	140.221	12.33487	142.5734
12.2813	122.0114	12.20205	136.3614	12.18493	144.6393	12.2915	139.9269	12.21955	140.2212	12.35153	142.5736
12.29797	122.0011	12.21872	136.355	12.2016	144.6399	12.30817	139.9274	12.23622	140.2166	12.3682	142.5725
12.31463	121.9916	12.23538	136.3512	12.21827	144.6317	12.32483	139.9231	12.25288	140.214	12.38487	142.5718
12.3313	121.986	12.25205	136.3474	12.23493	144.6309	12.3415	139.919	12.26955	140.2152	12.40153	142.5697
12.34797	121.9736	12.26872	136.3453	12.2516	144.6277	12.35817	139.9174	12.28622	140.2094	12.4182	142.568
12.36463	121.9678	12.28538	136.3414	12.26827	144.6236	12.37483	139.9137	12.30288	140.2076	12.43487	142.5666
12.3813	121.9562	12.30205	136.3382	12.28493	144.6206	12.3915	139.9109	12.31955	140.2029	12.45153	142.5667
12.39797	121.9471	12.31872	136.3376	12.3016	144.6205	12.40817	139.9105	12.33622	140.2008	12.4682	142.5653
12.41463	121.9409	12.33538	136.3352	12.31827	144.6147	12.42483	139.9073	12.35288	140.2006	12.48487	142.563
12.4313	121.9316	12.35205	136.33	12.33493	144.6133	12.4415	139.9038	12.36955	140.1966	12.50153	142.5607
12.44797	121.9228	12.36872	136.3298	12.3516	144.6103	12.45817	139.901	12.38622	140.1953	12.5182	142.5592
12.46463	121.9165	12.38538	136.3258	12.36827	144.6076	12.47483	139.8984	12.40288	140.1917	12.53487	142.5585
12.4813	121.9054	12.40205	136.324	12.38493	144.6039	12.4915	139.8931	12.41955	140.1905	12.55153	142.5561
12.49797	121.895	12.41872	136.3224	12.4016	144.6027	12.50817	139.8896	12.43622	140.1904	12.5682	142.5547
12.51463	121.8887	12.43538	136.3205	12.41827	144.6015	12.52483	139.887	12.45288	140.1849	12.58487	142.5533
12.5313	121.8817	12.45205	136.318	12.43493	144.5981	12.5415	139.8855	12.46955	140.1807	12.60153	142.5494
12.54797	121.8701	12.46872	136.3143	12.4516	144.6007	12.55817	139.8852	12.48622	140.1825	12.6182	142.5487
12.56463	121.8637	12.48538	136.3138	12.46827	144.5952	12.57483	139.8813	12.50288	140.1817	12.63487	142.5471
12.5813	121.8534	12.50205	136.3078	12.48493	144.589	12.5915	139.8776	12.51955	140.179	12.65153	142.5469
12.59797	121.8458	12.51872	136.3079	12.5016	144.5886	12.60817	139.8767	12.53622	140.1741	12.6682	142.5455
12.61463	121.8367	12.53538	136.3036	12.51827	144.5846	12.62483	139.872	12.55288	140.173	12.68487	142.5432
12.6313	121.8283	12.55205	136.2971	12.53493	144.584	12.6415	139.868	12.56955	140.1714	12.70153	142.5409
12.64797	121.8183	12.56872	136.2932	12.5516	144.5757	12.65817	139.8668	12.58622	140.1695	12.7182	142.5411
12.66463	121.8106	12.58538	136.289	12.56827	144.5795	12.67483	139.8632	12.60288	140.1681	12.73487	142.5361
12.6813	121.803	12.60205	136.2848	12.58493	144.5756	12.6915	139.8601	12.61955	140.1634	12.75153	142.5354
12.69797	121.796	12.61872	136.2799	12.6016	144.57	12.70817	139.8591	12.63622	140.164	12.7682	142.5346
12.71463	121.7841	12.63538	136.2776	12.61827	144.5717	12.72483	139.8565	12.65288	140.1605	12.78487	142.5335
12.7313	121.776	12.65205	136.2723	12.63493	144.5687	12.7415	139.853	12.66955	140.1585	12.80153	142.5322
12.74797	121.7702	12.66872	136.2699	12.6516	144.5624	12.75817	139.8516	12.68622	140.1552	12.8182	142.5303
12.76463	121.7604	12.68538	136.2672	12.66827	144.565	12.77483	139.8472	12.70288	140.1544	12.83487	142.5272
12.7813	121.7512	12.70205	136.2595	12.68493	144.5607	12.7915	139.8486	12.71955	140.1519	12.85153	142.5282
12.79797	121.7445	12.71872	136.2543	12.7016	144.5559	12.80817	139.8441	12.73622	140.1491	12.8682	142.5275
12.81463	121.7339	12.73538	136.2524	12.71827	144.5553	12.82483	139.8386	12.75288	140.1474	12.88487	142.5243
12.8313	121.7265	12.75205	136.2481	12.73493	144.5539	12.8415	139.8381	12.76955	140.1429	12.90153	142.5204
12.84797	121.7166	12.76872	136.2449	12.7516	144.5515	12.85817	139.8334	12.78622	140.1425	12.9182	142.5205
12.86463	121.7079	12.78538	136.2431	12.76827	144.5488	12.87483	139.8306	12.80288	140.142	12.93487	142.5211
12.8813	121.6999	12.80205	136.2364	12.78493	144.5489	12.8915	139.8265	12.81955	140.1371	12.95153	142.519
12.89797	121.6926	12.81872	136.2343	12.8016	144.5481	12.90817	139.8217	12.83622	140.1357	12.9682	142.5181
12.91463	121.6809	12.83538	136.2315	12.81827	144.5409	12.92483	139.8201	12.85288	140.1358	12.98487	142.5136
12.9313	121.6746	12.85205	136.2285	12.83493	144.5378	12.9415	139.8188	12.86955	140.1347	13.00153	142.5128
12.94797	121.666	12.86872	136.2217	12.8516	144.5339	12.95817	139.8126	12.88622	140.1295	13.0182	142.5137
12.96463	121.6584	12.88538	136.2194	12.86827	144.5383	12.97483	139.8106	12.90288	140.1298	13.03487	142.5091
12.9813	121.6527	12.90205	136.2171	12.88493	144.5295	12.9915	139.8086	12.91955	140.1267	13.05153	142.5098

12.99797	121.6427	12.91872	136.2109	12.9016	144.5294	13.00817	139.8074	12.93622	140.1258	13.0682	142.5102
13.01463	121.6326	12.93538	136.208	12.91827	144.5252	13.02483	139.8023	12.95288	140.1251	13.08487	142.5065
13.0313	121.6275	12.95205	136.2047	12.93493	144.5278	13.0415	139.8007	12.96955	140.1217	13.10153	142.5041
13.04797	121.6187	12.96872	136.2014	12.9516	144.5223	13.05817	139.7965	12.98622	140.1194	13.1182	142.5043
13.06463	121.6073	12.98538	136.1959	12.96827	144.5205	13.07483	139.7927	13.00288	140.1205	13.13487	142.4984
13.0813	121.5994	13.00205	136.1941	12.98493	144.519	13.0915	139.7903	13.01955	140.1156	13.15153	142.5021
13.09797	121.5918	13.01872	136.1905	13.0016	144.5156	13.10817	139.7892	13.03622	140.1147	13.1682	142.497
13.11463	121.583	13.03538	136.1844	13.01827	144.5135	13.12483	139.7869	13.05288	140.1135	13.18487	142.4988
13.1313	121.5752	13.05205	136.184	13.03493	144.5133	13.1415	139.7842	13.06955	140.1108	13.20153	142.4955
13.14797	121.5663	13.06872	136.1802	13.0516	144.5098	13.15817	139.7818	13.08622	140.1062	13.2182	142.4947
13.16463	121.5579	13.08538	136.1763	13.06827	144.5079	13.17483	139.7777	13.10288	140.1033	13.23487	142.4923
13.1813	121.5481	13.10205	136.1728	13.08493	144.5082	13.1915	139.7745	13.11955	140.1038	13.25153	142.4931
13.19797	121.5407	13.11872	136.1703	13.1016	144.5048	13.20817	139.7739	13.13622	140.1045	13.2682	142.49
13.21463	121.5315	13.13538	136.1685	13.11827	144.5014	13.22483	139.7688	13.15288	140.1015	13.28487	142.4875
13.2313	121.5264	13.15205	136.1631	13.13493	144.4998	13.2415	139.7673	13.16955	140.0975	13.30153	142.4864
13.24797	121.5173	13.16872	136.1626	13.1516	144.4954	13.25817	139.7625	13.18622	140.0983	13.3182	142.4855
13.26463	121.5105	13.18538	136.1585	13.16827	144.4966	13.27483	139.7618	13.20288	140.0921	13.33487	142.4827
13.2813	121.5017	13.20205	136.1516	13.18493	144.4914	13.2915	139.7588	13.21955	140.0912	13.35153	142.4818
13.29797	121.4941	13.21872	136.151	13.2016	144.4887	13.30817	139.7593	13.23622	140.0914	13.3682	142.4808
13.31463	121.4855	13.23538	136.1468	13.21827	144.4869	13.32483	139.7543	13.25288	140.0874	13.38487	142.4784
13.3313	121.477	13.25205	136.144	13.23493	144.4872	13.3415	139.7524	13.26955	140.0898	13.40153	142.4773
13.34797	121.4699	13.26872	136.1379	13.2516	144.4835	13.35817	139.7487	13.28622	140.0856	13.4182	142.4754
13.36463	121.4613	13.28538	136.1361	13.26827	144.4865	13.37483	139.7463	13.30288	140.0838	13.43487	142.4728
13.3813	121.4523	13.30205	136.1329	13.28493	144.4779	13.3915	139.7395	13.31955	140.0797	13.45153	142.4733
13.39797	121.4468	13.31872	136.1287	13.3016	144.477	13.40817	139.7383	13.33622	140.0807	13.4682	142.4719
13.41463	121.437	13.33538	136.127	13.31827	144.4721	13.42483	139.7354	13.35288	140.0763	13.48487	142.4701
13.4313	121.431	13.35205	136.1194	13.33493	144.4744	13.4415	139.7349	13.36955	140.0765	13.50153	142.468
13.44797	121.4203	13.36872	136.1184	13.3516	144.4686	13.45817	139.7317	13.38622	140.074	13.5182	142.4658
13.46463	121.4119	13.38538	136.1121	13.36827	144.4661	13.47483	139.7261	13.40288	140.0716	13.53487	142.466
13.4813	121.404	13.40205	136.1103	13.38493	144.4647	13.4915	139.7265	13.41955	140.0689	13.55153	142.4638
13.49797	121.3959	13.41872	136.1052	13.4016	144.4625	13.50817	139.7243	13.43622	140.0674	13.5682	142.4632
13.51463	121.3891	13.43538	136.1008	13.41827	144.462	13.52483	139.7202	13.45288	140.0653	13.58487	142.4607
13.5313	121.3805	13.45205	136.1005	13.43493	144.4578	13.5415	139.7158	13.46955	140.0645	13.60153	142.457
13.54797	121.3733	13.46872	136.0954	13.4516	144.4576	13.55817	139.7158	13.48622	140.0629	13.6182	142.4584
13.56463	121.3658	13.48538	136.089	13.46827	144.4538	13.57483	139.7077	13.50288	140.0618	13.63487	142.456
13.5813	121.3575	13.50205	136.0857	13.48493	144.4505	13.5915	139.7152	13.51955	140.0586	13.65153	142.4556
13.59797	121.3493	13.51872	136.0825	13.5016	144.4501	13.60817	139.7083	13.53622	140.0585	13.6682	142.4528
13.61463	121.3404	13.53538	136.0771	13.51827	144.4424	13.62483	139.7035	13.55288	140.0562	13.68487	142.4525
13.6313	121.3354	13.55205	136.0743	13.53493	144.4459	13.6415	139.7007	13.56955	140.0543	13.70153	142.4517
13.64797	121.3249	13.56872	136.0727	13.5516	144.4418	13.65817	139.7004	13.58622	140.0518	13.7182	142.4504
13.66463	121.3193	13.58538	136.0693	13.56827	144.4388	13.67483	139.6957	13.60288	140.0527	13.73487	142.4483
13.6813	121.3097	13.60205	136.0635	13.58493	144.4355	13.6915	139.6942	13.61955	140.0493	13.75153	142.4455
13.69797	121.3032	13.61872	136.0612	13.6016	144.4362	13.70817	139.6928	13.63622	140.0477	13.7682	142.4437
13.71463	121.2941	13.63538	136.0561	13.61827	144.4332	13.72483	139.6852	13.65288	140.0457	13.78487	142.4447
13.7313	121.2889	13.65205	136.0538	13.63493	144.4339	13.7415	139.6835	13.66955	140.0409	13.80153	142.4413
13.74797	121.278	13.66872	136.0492	13.6516	144.4271	13.75817	139.6837	13.68622	140.0382	13.8182	142.4384
13.76463	121.2731	13.68538	136.0467	13.66827	144.4266	13.77483	139.6833	13.70288	140.0416	13.83487	142.441
13.7813	121.2644	13.70205	136.0415	13.68493	144.4231	13.7915	139.6765	13.71955	140.0366	13.85153	142.4395
13.79797	121.2572	13.71872	136.0374	13.7016	144.4229	13.80817	139.6757	13.73622	140.0351	13.8682	142.4357
13.81463	121.2465	13.73538	136.0337	13.71827	144.4185	13.82483	139.6732	13.75288	140.0337	13.88487	142.4361
13.8313	121.2404	13.75205	136.033	13.73493	144.4152	13.8415	139.6712	13.76955	140.0319	13.90153	142.4338
13.84797	121.2335	13.76872	136.0238	13.7516	144.4162	13.85817	139.6678	13.78622	140.03	13.9182	142.4334
13.86463	121.2265	13.78538	136.0211	13.76827	144.4134	13.87483	139.6642	13.80288	140.0322	13.93487	142.4317
13.8813	121.2191	13.80205	136.0144	13.78493	144.411	13.8915	139.6595	13.81955	140.0259	13.95153	142.4321
13.89797	121.215	13.81872	136.0131	13.8016	144.4058	13.90817	139.6596	13.83622	140.0236	13.9682	142.4286
13.91463	121.2039	13.83538	136.0093	13.81827	144.4043	13.92483	139.6549	13.85288	140.0227	13.98487	142.4286
13.9313	121.1966	13.85205	136.0042	13.83493	144.403	13.9415	139.6525	13.86955	140.0219	14.00153	142.4277
13.94797	121.1884	13.86872	136.0018	13.8516	144.4011	13.95817	139.6529	13.88622	140.0195	14.0182	142.4262
13.96463	121.1808	13.88538	135.9963	13.86827	144.3997	13.97483	139.6475	13.90288	140.0194	14.03487	142.4257
13.9813	121.1754	13.90205	135.9963	13.88493	144.3953	13.9915	139.645	13.91955	140.0152	14.05153	142.4238
13.99797	121.1653	13.91872	135.9868	13.9016	144.3939	14.00817	139.643	13.93622	140.0118	14.0682	142.4233
14.01463	121.1586	13.93538	135.9813	13.91827	144.3906	14.02483	139.6422	13.95288	140.0114	14.08487	142.4208
14.0313	121.1518	13.95205	135.9799	13.93493	144.3877	14.0415	139.637	13.96955	140.0126	14.10153	142.42
14.04797	121.1444	13.96872	135.9749	13.9516	144.389	14.05817	139.6408	13.98622	140.0103	14.1182	142.4176
14.06463	121.1397	13.98538	135.9718	13.96827	144.3817	14.07483	139.6383	14.00288	140.0085	14.13487	142.4184
14.0813	121.1309	14.00205	135.9695	13.98493	144.3806	14.0915	139.6346	14.01955	140.0061	14.15153	142.4174
14.09797	121.1221	14.01872	135.9695	14.0016	144.3792	14.10817	139.6314	14.03622	140.005	14.1682	142.4148
14.11463	121.1155	14.03538	135.9622	14.01827	144.3766	14.12483	139.6271	14.05288	140.007	14.18487	142.4145
14.1313	121.1087	14.05205	135.9579	14.03493	144.3738	14.1415	139.6229	14.06955	140.0024	14.20153	142.413
14.14797	121.1004	14.06872	135.9551	14.0516	144.3689	14.15817	139.6239	14.08622	139.998	14.2182	142.4112
14.16463	121.093	14.08538	135.9514	14.06827	144.3675	14.17483	139.6206	14.10288	139.9988	14.23487	142.4094
14.1813	121.0848	14.10205	135.9445	14.08493	144.3615	14.1915	139.6175	14.11955	139.9971	14.25153	142.41
14.19797	121.0797	14.11872	135.9444	14.1016	144.3607	14.20817	139.6159	14.13622	139.9962	14.2682	142.4065
14.21463	121.0711	14.13538	135.9414	14.11827	144.3599	14.22483	139.6147	14.15288	139.993	14.28487	142.4043
14.2313	121.0643	14.15205	135.9363	14.13493	144.3557	14.2415	139.6102	14.16955	139.9925	14.30153	142.4025
14.24797	121.057	14.16872	135.9342	14.1516	144.354	14.25817	139.6098	14.18622	139.9893	14.3182	142.4022
14.26463	121.0492	14.18538	135.9257	14.16827	144.3486	14.27483	139.6072	14.20288	139.988	14.33487	142.3987
14.2813	121.0401	14.20205	135.9252	14.18493	144.3456	14.2915	139.6056	14.21955	139.9877	14.35153	142.3995
14.29797	121.0353	14.21872	135.9208	14.2016	144.3474	14.30817	139.606	14.23622	139.9855	14.3682	142.3991
14.31463	121.0251	14.23538	135.9165	14.21827	144.3409	14.32483	139.5993	14.25288	139.983	14.38487	142.3958
14.3313	121.02	14.25205	135.913	14.23493	144.3403	14.3415	139.5969	14.26955	139.983	14.40153	142.3948
14.34797	121.0121	14.26872	135.9087	14.2516	144.3397	14.35817	139.5912	14.28622	139.9821	14.4182	142.3946
14.36463	121.0038	14.28538	135.9056	14.26827	144.3373	14.37483	139.5936	14.30288	139.9804	14.43487	142.393
14.3813	120.9956	14.30205	135.9008	14.28493	144.3319	14.3915	139.5898	14.31955	139.9783	14.45153	142.3908
14.39797	120.9899	14.31872	135.8969	14.3016	144.3305	14.40817	139.5889	14.33622	139.9753	14.4682	142.3882
14.41463	120.982	14.33538	135.8949	14.31827	144.3283	14.42483	139.586	14.35288	139.9765	14.48487	142.3884
14.4313	120.9744	14.35205	135.8879	14.33493	144.3236	14.4415	139.5816	14.36955	139.9732	14.50153	142.3883
14.44797	120.9688	14.36872	135.8862	14.3516	144.3217	14.45817	139.5824	14.38622	139.9718	14.5182	142.3871
14.46463	120.9601	14.38538	135.8845	14.36827	144.3185	14.47483	139.579	14.40288	139.9685	14.53487	142.3831
14.4813	120.9536	14.40205	135.8781	14.38493	144.3182	14.4915	139.5744	14.41955	139.9644	14.55153	142.3833
14.49797	120.9479	14.41872	135.8752	14.4016	144.3174	14.50817	139.5709	14.43622	139.9641	14.5682	142.384
14.51463	120.9399	14.43538	135.8722	14.41827	144.311	14.52483	139.5706	14.45288	139.9623	14.58487	142.3791
14.5313	120.9333	14.45205	135.8701	14.43493	144.3135	14.5415	139.5683	14.46955	139.9624	14.60153	142.379
14.54797	120.9279	14.46872	135.8655	14.4516	144.3079	14.55817	139.5626	14.48622	139.9602	14.6182	142.3777
14.56463	120.9182	14.48538	135.8635	14.46827	144.3067	14.57483	139.5637	14.50288	139.9577	14.63487	142.3785
14.5813	120.9118	14.50205	135.8571	14.48493	144.3046	14.5915	139.5529	14.51955	139.9554	14.65153	142.3759
14.59797	120.9048	14.51872	135.8569	14.5016	144.2994	14.60817	139.5595	14.53622	139.9548	14.6682	142.3762

14.61463	120.895	14.53538	135.8536	14.51827	144.298	14.62483	139.5527	14.55288	139.9537	14.68487	142.3719
14.6313	120.8902	14.55205	135.8486	14.53493	144.2962	14.6415	139.5488	14.56955	139.9506	14.70153	142.3708
14.64797	120.8836	14.56872	135.8465	14.5516	144.2957	14.65817	139.5458	14.58622	139.9485	14.7182	142.3729
14.66463	120.874	14.58538	135.8398	14.56827	144.2935	14.67483	139.5455	14.60288	139.9467	14.73487	142.3697
14.6813	120.8647	14.60205	135.8385	14.58493	144.2906	14.6915	139.5438	14.61955	139.9456	14.75153	142.3698
14.69797	120.8618	14.61872	135.8359	14.6016	144.2886	14.70817	139.5402	14.63622	139.9453	14.7682	142.3695
14.71463	120.8547	14.63538	135.8286	14.61827	144.2832	14.72483	139.5376	14.65288	139.942	14.78487	142.3659
14.7313	120.8459	14.65205	135.8269	14.63493	144.2827	14.7415	139.5347	14.66955	139.9405	14.80153	142.3651
14.74797	120.837	14.66872	135.8218	14.6516	144.2813	14.75817	139.533	14.68622	139.9382	14.8182	142.365
14.76463	120.8284	14.68538	135.8183	14.66827	144.2792	14.77483	139.5314	14.70288	139.9361	14.83487	142.3637
14.7813	120.8225	14.70205	135.817	14.68493	144.2762	14.7915	139.5274	14.71955	139.9334	14.85153	142.3617
14.79797	120.82	14.71872	135.8112	14.7016	144.2746	14.80817	139.5261	14.73622	139.9324	14.8682	142.364
14.81463	120.8097	14.73538	135.8061	14.71827	144.27	14.82483	139.5197	14.75288	139.931	14.88487	142.358
14.8313	120.8032	14.75205	135.8038	14.73493	144.2672	14.8415	139.5228	14.76955	139.9273	14.90153	142.3549
14.84797	120.793	14.76872	135.7997	14.7516	144.2662	14.85817	139.518	14.78622	139.9257	14.9182	142.3571
14.86463	120.7868	14.78538	135.7969	14.76827	144.2635	14.87483	139.5173	14.80288	139.9292	14.93487	142.3539
14.8813	120.7782	14.80205	135.7904	14.78493	144.2632	14.8915	139.5152	14.81955	139.9245	14.95153	142.3517
14.89797	120.7716	14.81872	135.7898	14.8016	144.2599	14.90817	139.5113	14.83622	139.9243	14.9682	142.3506
14.91463	120.767	14.83538	135.7846	14.81827	144.2546	14.92483	139.5065	14.85288	139.9203	14.98487	142.351
14.9313	120.7581	14.85205	135.7785	14.83493	144.2533	14.9415	139.5061	14.86955	139.9203	15.00153	142.3496
14.94797	120.7504	14.86872	135.777	14.8516	144.251	14.95817	139.5055	14.88622	139.9177	15.0182	142.3471
14.96463	120.7443	14.88538	135.7718	14.86827	144.2476	14.97483	139.5017	14.90288	139.9156	15.03487	142.3449
14.9813	120.736	14.90205	135.767	14.88493	144.2451	14.9915	139.5029	14.91955	139.9126	15.05153	142.3425
14.99797	120.73	14.91872	135.7661	14.9016	144.2438	15.00817	139.4987	14.93622	139.9106	15.0682	142.3421
15.01463	120.7198	14.93538	135.7616	14.91827	144.2392	15.02483	139.4972	14.95288	139.9077	15.08487	142.3401
15.0313	120.7186	14.95205	135.7577	14.93493	144.2386	15.0415	139.4954	14.96955	139.9082	15.10153	142.3401
15.04797	120.7081	14.96872	135.7549	14.9516	144.2329	15.05817	139.4898	14.98622	139.9061	15.1182	142.3369
15.06463	120.7019	14.98538	135.7523	14.96827	144.2328	15.07483	139.4901	15.00288	139.9014	15.13487	142.34
15.0813	120.6937	15.00205	135.7482	14.98493	144.2282	15.0915	139.4848	15.01955	139.9	15.15153	142.3349
15.09797	120.6842	15.01872	135.7424	15.0016	144.2263	15.10817	139.4832	15.03622	139.8987	15.1682	142.3341
15.11463	120.6812	15.03538	135.7398	15.01827	144.2252	15.12483	139.4798	15.05288	139.8975	15.18487	142.3329
15.1313	120.6712	15.05205	135.7349	15.03493	144.222	15.1415	139.4788	15.06955	139.8944	15.20153	142.3313
15.14797	120.6642	15.06872	135.7337	15.0516	144.2189	15.15817	139.4747	15.08622	139.8912	15.2182	142.3313
15.16463	120.6608	15.08538	135.7285	15.06827	144.2169	15.17483	139.4732	15.10288	139.8904	15.23487	142.329
15.1813	120.6476	15.10205	135.7255	15.08493	144.2126	15.1915	139.4722	15.11955	139.888	15.25153	142.3286
15.19797	120.6428	15.11872	135.7215	15.1016	144.2144	15.20817	139.4706	15.13622	139.8876	15.2682	142.3275
15.21463	120.6385	15.13538	135.7181	15.11827	144.2105	15.22483	139.4687	15.15288	139.8839	15.28487	142.3256
15.2313	120.6311	15.15205	135.7133	15.13493	144.2076	15.2415	139.4643	15.16955	139.8853	15.30153	142.3245
15.24797	120.6229	15.16872	135.7077	15.1516	144.2059	15.25817	139.4616	15.18622	139.8784	15.3182	142.3244
15.26463	120.6146	15.18538	135.7063	15.16827	144.2015	15.27483	139.4585	15.20288	139.8796	15.33487	142.3234
15.2813	120.6094	15.20205	135.7032	15.18493	144.2016	15.2915	139.4577	15.21955	139.8759	15.35153	142.3217
15.29797	120.6037	15.21872	135.6989	15.2016	144.2004	15.30817	139.4552	15.23622	139.8746	15.3682	142.3206
15.31463	120.5985	15.23538	135.694	15.21827	144.196	15.32483	139.4519	15.25288	139.8718	15.38487	142.3202
15.3313	120.5913	15.25205	135.6893	15.23493	144.1949	15.3415	139.4489	15.26955	139.8713	15.40153	142.3184
15.34797	120.5785	15.26872	135.6879	15.2516	144.1916	15.35817	139.4474	15.28622	139.8684	15.4182	142.3191
15.36463	120.5745	15.28538	135.6876	15.26827	144.1885	15.37483	139.4464	15.30288	139.8672	15.43487	142.316
15.3813	120.5687	15.30205	135.6791	15.28493	144.1873	15.3915	139.4438	15.31955	139.8668	15.45153	142.3155
15.39797	120.56	15.31872	135.6778	15.3016	144.1838	15.40817	139.4427	15.33622	139.8653	15.4682	142.3124
15.41463	120.554	15.33538	135.6751	15.31827	144.1817	15.42483	139.4381	15.35288	139.8641	15.48487	142.312
15.4313	120.549	15.35205	135.6701	15.33493	144.1754	15.4415	139.4365	15.36955	139.861	15.50153	142.309
15.44797	120.5404	15.36872	135.6654	15.3516	144.1773	15.45817	139.4352	15.38622	139.8599	15.5182	142.3095
15.46463	120.5335	15.38538	135.6612	15.36827	144.1731	15.47483	139.4316	15.40288	139.8578	15.53487	142.3057
15.4813	120.5277	15.40205	135.6598	15.38493	144.1753	15.4915	139.4285	15.41955	139.8571	15.55153	142.3048
15.49797	120.5201	15.41872	135.6547	15.4016	144.1692	15.50817	139.4272	15.43622	139.859	15.5682	142.3039
15.51463	120.5122	15.43538	135.6524	15.41827	144.1697	15.52483	139.4252	15.45288	139.8537	15.58487	142.3031
15.5313	120.5067	15.45205	135.6492	15.43493	144.1655	15.5415	139.4231	15.46955	139.854	15.60153	142.3002
15.54797	120.4985	15.46872	135.642	15.4516	144.1633	15.55817	139.4195	15.48622	139.8513	15.6182	142.2985
15.56463	120.4926	15.48538	135.6419	15.46827	144.161	15.57483	139.4183	15.50288	139.8494	15.63487	142.2977
15.5813	120.4849	15.50205	135.6361	15.48493	144.1613	15.5915	139.417	15.51955	139.8479	15.65153	142.296
15.59797	120.4809	15.51872	135.6346	15.5016	144.1558	15.60817	139.4144	15.53622	139.8459	15.6682	142.2952
15.61463	120.4735	15.53538	135.6304	15.51827	144.157	15.62483	139.4133	15.55288	139.8435	15.68487	142.2933
15.6313	120.4674	15.55205	135.6257	15.53493	144.1563	15.6415	139.4084	15.56955	139.8426	15.70153	142.2914
15.64797	120.4566	15.56872	135.6227	15.5516	144.1522	15.65817	139.4078	15.58622	139.8428	15.7182	142.2903
15.66463	120.4534	15.58538	135.6169	15.56827	144.1508	15.67483	139.4082	15.60288	139.8413	15.73487	142.2908
15.6813	120.4457	15.60205	135.6151	15.58493	144.1473	15.6915	139.4019	15.61955	139.8382	15.75153	142.2891
15.69797	120.4401	15.61872	135.6112	15.6016	144.1459	15.70817	139.4002	15.63622	139.8387	15.7682	142.2884
15.71463	120.4325	15.63538	135.6065	15.61827	144.1412	15.72483	139.399	15.65288	139.835	15.78487	142.2866
15.7313	120.4283	15.65205	135.6027	15.63493	144.1388	15.7415	139.3998	15.66955	139.836	15.80153	142.2837
15.74797	120.4198	15.66872	135.5997	15.6516	144.1368	15.75817	139.3944	15.68622	139.8344	15.8182	142.2849
15.76463	120.4125	15.68538	135.5952	15.66827	144.1322	15.77483	139.3926	15.70288	139.8318	15.83487	142.2837
15.7813	120.4048	15.70205	135.5901	15.68493	144.1307	15.7915	139.3863	15.71955	139.8311	15.85153	142.2818
15.79797	120.3978	15.71872	135.59	15.7016	144.1294	15.80817	139.3883	15.73622	139.8306	15.8682	142.2777
15.81463	120.3903	15.73538	135.5821	15.71827	144.1251	15.82483	139.3799	15.75288	139.8291	15.88487	142.2792
15.8313	120.3847	15.75205	135.5778	15.73493	144.1228	15.8415	139.3841	15.76955	139.8272	15.90153	142.2783
15.84797	120.3775	15.76872	135.5772	15.7516	144.1204	15.85817	139.3816	15.78622	139.8249	15.9182	142.275
15.86463	120.3721	15.78538	135.5727	15.76827	144.1185	15.87483	139.3783	15.80288	139.8227	15.93487	142.2752
15.8813	120.3616	15.80205	135.5671	15.78493	144.1157	15.8915	139.3798	15.81955	139.8194	15.95153	142.2758
15.89797	120.3552	15.81872	135.5663	15.8016	144.1138	15.90817	139.3753	15.83622	139.8202	15.9682	142.2731
15.91463	120.3464	15.83538	135.5628	15.81827	144.1112	15.92483	139.3751	15.85288	139.8197	15.98487	142.2718
15.9313	120.3447	15.85205	135.5584	15.83493	144.1106	15.9415	139.3751	15.86955	139.8183	16.00153	142.2723
15.94797	120.3392	15.86872	135.5551	15.8516	144.1059	15.95817	139.3719	15.88622	139.8157	16.0182	142.2696
15.96463	120.3301	15.88538	135.5509	15.86827	144.1054	15.97483	139.3706	15.90288	139.8158	16.03487	142.2694
15.9813	120.3216	15.90205	135.5461	15.88493	144.1016	15.9915	139.3677	15.91955	139.8144	16.05153	142.2663
15.99797	120.3154	15.91872	135.5445	15.9016	144.0954	16.00817	139.3643	15.93622	139.8124	16.0682	142.2656
16.01463	120.3087	15.93538	135.5405	15.91827	144.0973	16.02483	139.3608	15.95288	139.8091	16.08487	142.2635
16.0313	120.306	15.95205	135.5368	15.93493	144.0959	16.0415	139.3587	15.96955	139.8097	16.10153	142.2613
16.04797	120.2954	15.96872	135.5308	15.9516	144.092	16.05817	139.3554	15.98622	139.8075	16.1182	142.2602
16.06463	120.2898	15.98538	135.531	15.96827	144.0918	16.07483	139.3538	16.00288	139.8081	16.13487	142.2621
16.0813	120.2816	16.00205	135.5241	15.98493	144.0885	16.0915	139.3496	16.01955	139.806	16.15153	142.2597
16.09797	120.276	16.01872	135.5199	16.0016	144.0879	16.10817	139.3445	16.03622	139.8016	16.1682	142.2567
16.11463	120.2686	16.03538	135.517	16.01827	144.0853	16.12483	139.3469	16.05288	139.8002	16.18487	142.2566
16.1313	120.2615	16.05205	135.5133	16.03493	144.0843	16.1415	139.3425	16.06955	139.8016	16.20153	142.2556
16.14797	120.2558	16.06872	135.5103	16.0516	144.0796	16.15817	139.3388	16.08622	139.8009	16.2182	142.2531
16.16463	120.2464	16.08538	135.5079	16.06827	144.0784	16.17483	139.337	16.10288	139.7983	16.23487	142.2532
16.1813	120.2414	16.10205	135.5031	16.08493	144.0742	16.1915	139.3373	16.11955	139.7954	16.25153	142.253
16.19797	120.2355	16.11872	135.4992	16.1016	144.0761	16.20817	139.3325	16.13622	139.7922	16.2682	142.2496
16.21463	120.2297	16.13538	135.4967	16.11827	144.073	16.22483	139.3309	16.15288	139.7939	16.28487	142.2487

16.2313	120.2203	16.15205	135.4933	16.13493	144.0685	16.2415	139.3261	16.16955	139.7913	16.30153	142.2489
16.24797	120.2169	16.16872	135.4873	16.1516	144.068	16.25817	139.3263	16.18622	139.7892	16.3182	142.2464
16.26463	120.2085	16.18538	135.4865	16.16827	144.0634	16.27483	139.3246	16.20288	139.7869	16.33487	142.2446
16.2813	120.202	16.20205	135.4816	16.18493	144.0647	16.2915	139.3205	16.21955	139.7881	16.35153	142.2457
16.29797	120.1934	16.21872	135.4798	16.2016	144.0607	16.30817	139.32	16.23622	139.7853	16.3682	142.2455
16.31463	120.186	16.23538	135.4769	16.21827	144.0564	16.32483	139.3175	16.25288	139.7813	16.38487	142.2432
16.3313	120.1846	16.25205	135.4707	16.23493	144.056	16.3415	139.3163	16.26955	139.7826	16.40153	142.2437
16.34797	120.1755	16.26872	135.4644	16.2516	144.0544	16.35817	139.3146	16.28622	139.7826	16.4182	142.2419
16.36463	120.1722	16.28538	135.4673	16.26827	144.0531	16.37483	139.3099	16.30288	139.7791	16.43487	142.2404
16.3813	120.1628	16.30205	135.46	16.28493	144.0487	16.3915	139.3114	16.31955	139.7768	16.45153	142.2395
16.39797	120.1549	16.31872	135.4557	16.3016	144.0473	16.40817	139.3064	16.33622	139.7764	16.4682	142.239
16.41463	120.1502	16.33538	135.4524	16.31827	144.0449	16.42483	139.3036	16.35288	139.7727	16.48487	142.2367
16.4313	120.1438	16.35205	135.4485	16.33493	144.0429	16.4415	139.3011	16.36955	139.7736	16.50153	142.2376
16.44797	120.1335	16.36872	135.445	16.3516	144.0412	16.45817	139.3008	16.38622	139.7715	16.5182	142.2353
16.46463	120.1297	16.38538	135.4425	16.36827	144.038	16.47483	139.2987	16.40288	139.7709	16.53487	142.2345
16.4813	120.1214	16.40205	135.4387	16.38493	144.0366	16.4915	139.2956	16.41955	139.7678	16.55153	142.2312
16.49797	120.115	16.41872	135.4311	16.4016	144.0317	16.50817	139.2948	16.43622	139.7689	16.5682	142.232
16.51463	120.1086	16.43538	135.4306	16.41827	144.0316	16.52483	139.2935	16.45288	139.7649	16.58487	142.228
16.5313	120.1012	16.45205	135.4258	16.43493	144.0257	16.5415	139.2883	16.46955	139.7635	16.60153	142.2298
16.54797	120.0934	16.46872	135.4223	16.4516	144.0291	16.55817	139.2874	16.48622	139.7602	16.6182	142.2278
16.56463	120.0887	16.48538	135.4196	16.46827	144.0242	16.57483	139.2851	16.50288	139.7617	16.63487	142.2262
16.5813	120.0784	16.50205	135.4154	16.48493	144.023	16.5915	139.2827	16.51955	139.7562	16.65153	142.2236
16.59797	120.0741	16.51872	135.4127	16.5016	144.0215	16.60817	139.2815	16.53622	139.7573	16.6682	142.2238
16.61463	120.0702	16.53538	135.4106	16.51827	144.0189	16.62483	139.2793	16.55288	139.7569	16.68487	142.2199
16.6313	120.0645	16.55205	135.4053	16.53493	144.0169	16.6415	139.2766	16.56955	139.7541	16.70153	142.2203
16.64797	120.0578	16.56872	135.4002	16.5516	144.0125	16.65817	139.2768	16.58622	139.7523	16.7182	142.2187
16.66463	120.0492	16.58538	135.3966	16.56827	144.0102	16.67483	139.2747	16.60288	139.7526	16.73487	142.2187
16.6813	120.0457	16.60205	135.3911	16.58493	144.0086	16.6915	139.273	16.61955	139.7484	16.75153	142.2167
16.69797	120.0402	16.61872	135.3904	16.6016	144.0069	16.70817	139.2684	16.63622	139.7481	16.7682	142.2138
16.71463	120.0333	16.63538	135.3873	16.61827	144.0045	16.72483	139.2665	16.65288	139.745	16.78487	142.2149
16.7313	120.0294	16.65205	135.3789	16.63493	144.0019	16.7415	139.2657	16.66955	139.7461	16.80153	142.2131
16.74797	120.0183	16.66872	135.3776	16.6516	144.0001	16.75817	139.2632	16.68622	139.7409	16.8182	142.2122
16.76463	120.014	16.68538	135.3752	16.66827	143.9964	16.77483	139.2607	16.70288	139.7399	16.83487	142.2115
16.7813	120.0044	16.70205	135.3676	16.68493	143.9956	16.7915	139.2589	16.71955	139.7401	16.85153	142.2104
16.79797	120.0004	16.71872	135.3669	16.7016	143.992	16.80817	139.2593	16.73622	139.736	16.8682	142.2107
16.81463	119.9933	16.73538	135.3618	16.71827	143.991	16.82483	139.255	16.75288	139.733	16.88487	142.2081
16.8313	119.988	16.75205	135.356	16.73493	143.9885	16.8415	139.2554	16.76955	139.7328	16.90153	142.2054
16.84797	119.9802	16.76872	135.352	16.7516	143.986	16.85817	139.2504	16.78622	139.7318	16.9182	142.2042
16.86463	119.9739	16.78538	135.3503	16.76827	143.9818	16.87483	139.2513	16.80288	139.7284	16.93487	142.2023
16.8813	119.9698	16.80205	135.3482	16.78493	143.9807	16.8915	139.2469	16.81955	139.7296	16.95153	142.2005
16.89797	119.9634	16.81872	135.3422	16.8016	143.9783	16.90817	139.247	16.83622	139.7273	16.9682	142.2009
16.91463	119.9538	16.83538	135.3345	16.81827	143.9752	16.92483	139.2458	16.85288	139.7261	16.98487	142.2024
16.9313	119.9485	16.85205	135.3342	16.83493	143.975	16.9415	139.242	16.86955	139.7237	17.00153	142.2
16.94797	119.9442	16.86872	135.3297	16.8516	143.9709	16.95817	139.2407	16.88622	139.7208	17.0182	142.1988
16.96463	119.938	16.88538	135.3276	16.86827	143.9691	16.97483	139.238	16.90288	139.7203	17.03487	142.1979
16.9813	119.9326	16.90205	135.3226	16.88493	143.9676	16.9915	139.2337	16.91955	139.7196	17.05153	142.197
16.99797	119.9262	16.91872	135.3159	16.9016	143.9648	17.00817	139.2347	16.93622	139.7185	17.0682	142.197
17.01463	119.9194	16.93538	135.3145	16.91827	143.9634	17.02483	139.2295	16.95288	139.7142	17.08487	142.1945
17.0313	119.9124	16.95205	135.3137	16.93493	143.9629	17.0415	139.2263	16.96955	139.7158	17.10153	142.1937
17.04797	119.9071	16.96872	135.3076	16.9516	143.9554	17.05817	139.227	16.98622	139.7134	17.1182	142.1909
17.06463	119.9005	16.98538	135.3048	16.96827	143.9568	17.07483	139.2267	17.00288	139.7122	17.13487	142.1922
17.0813	119.8957	17.00205	135.3021	16.98493	143.9556	17.0915	139.2222	17.01955	139.7094	17.15153	142.1893
17.09797	119.8914	17.01872	135.2969	17.0016	143.9536	17.10817	139.2195	17.03622	139.7093	17.1682	142.1886
17.11463	119.8816	17.03538	135.2939	17.01827	143.9485	17.12483	139.2202	17.05288	139.7065	17.18487	142.188
17.1313	119.8762	17.05205	135.2913	17.03493	143.9473	17.1415	139.2166	17.06955	139.7059	17.20153	142.1864
17.14797	119.87	17.06872	135.2861	17.0516	143.9486	17.15817	139.2137	17.08622	139.7033	17.2182	142.1854
17.16463	119.8625	17.08538	135.2829	17.06827	143.9447	17.17483	139.2122	17.10288	139.7016	17.23487	142.1867
17.1813	119.8578	17.10205	135.2786	17.08493	143.9431	17.1915	139.208	17.11955	139.7012	17.25153	142.1845
17.19797	119.8528	17.11872	135.2754	17.1016	143.9385	17.20817	139.2057	17.13622	139.702	17.2682	142.1837
17.21463	119.8459	17.13538	135.2705	17.11827	143.9363	17.22483	139.2038	17.15288	139.6999	17.28487	142.1832
17.2313	119.842	17.15205	135.2686	17.13493	143.9346	17.2415	139.2	17.16955	139.6969	17.30153	142.1818
17.24797	119.833	17.16872	135.2657	17.1516	143.9351	17.25817	139.2008	17.18622	139.6949	17.3182	142.1798
17.26463	119.8281	17.18538	135.2602	17.16827	143.931	17.27483	139.199	17.20288	139.6936	17.33487	142.1805
17.2813	119.8236	17.20205	135.2563	17.18493	143.9287	17.2915	139.1999	17.21955	139.6949	17.35153	142.1788
17.29797	119.8153	17.21872	135.2551	17.2016	143.9283	17.30817	139.1944	17.23622	139.69	17.3682	142.177
17.31463	119.8098	17.23538	135.2498	17.21827	143.926	17.32483	139.1936	17.25288	139.6893	17.38487	142.175
17.3313	119.8054	17.25205	135.2442	17.23493	143.9252	17.3415	139.1902	17.26955	139.6898	17.40153	142.1741
17.34797	119.7976	17.26872	135.2419	17.2516	143.9174	17.35817	139.1864	17.28622	139.6865	17.4182	142.1727
17.36463	119.7948	17.28538	135.2396	17.26827	143.9187	17.37483	139.1869	17.30288	139.6827	17.43487	142.1736
17.3813	119.7871	17.30205	135.2369	17.28493	143.9162	17.3915	139.1845	17.31955	139.6799	17.45153	142.1708
17.39797	119.7801	17.31872	135.2329	17.3016	143.913	17.40817	139.184	17.33622	139.6824	17.4682	142.1716
17.41463	119.775	17.33538	135.23	17.31827	143.9112	17.42483	139.1804	17.35288	139.6821	17.48487	142.1677
17.4313	119.7673	17.35205	135.2271	17.33493	143.9112	17.4415	139.1789	17.36955	139.6826	17.50153	142.1677
17.44797	119.7625	17.36872	135.2215	17.3516	143.9079	17.45817	139.1747	17.38622	139.6757	17.5182	142.1666
17.46463	119.7577	17.38538	135.2205	17.36827	143.9065	17.47483	139.1714	17.40288	139.6771	17.53487	142.1642
17.4813	119.7508	17.40205	135.2143	17.38493	143.901	17.4915	139.1713	17.41955	139.671	17.55153	142.1645
17.49797	119.7519	17.41872	135.2137	17.4016	143.9006	17.50817	139.1671	17.43622	139.671	17.5682	142.1641
17.51463	119.7416	17.43538	135.2084	17.41827	143.9015	17.52483	139.1671	17.45288	139.6679	17.58487	142.1614
17.5313	119.7362	17.45205	135.2062	17.43493	143.8978	17.5415	139.1638	17.46955	139.6683	17.60153	142.1636
17.54797	119.7246	17.46872	135.2035	17.4516	143.8967	17.55817	139.1611	17.48622	139.6692	17.6182	142.1604
17.56463	119.7261	17.48538	135.1972	17.46827	143.8941	17.57483	139.1568	17.50288	139.6651	17.63487	142.1591
17.5813	119.714	17.50205	135.1936	17.48493	143.8936	17.5915	139.1585	17.51955	139.6647	17.65153	142.1576
17.59797	119.7086	17.51872	135.1894	17.5016	143.8917	17.60817	139.1524	17.53622	139.663	17.6682	142.159
17.61463	119.7038	17.53538	135.186	17.51827	143.8865	17.62483	139.1524	17.55288	139.6595	17.68487	142.1578
17.6313	119.7004	17.55205	135.1839	17.53493	143.8842	17.6415	139.1541	17.56955	139.6596	17.70153	142.156
17.64797	119.6933	17.56872	135.1783	17.5516	143.8826	17.65817	139.1476	17.58622	139.6579	17.7182	142.1547
17.66463	119.6857	17.58538	135.177	17.56827	143.8811	17.67483	139.1455	17.60288	139.6558	17.73487	142.154
17.6813	119.678	17.60205	135.1755	17.58493	143.8828	17.6915	139.144	17.61955	139.6521	17.75153	142.1539
17.69797	119.6722	17.61872	135.1703	17.6016	143.8747	17.70817	139.139	17.63622	139.6526	17.7682	142.1532
17.71463	119.663	17.63538	135.1689	17.61827	143.8747	17.72483	139.1372	17.65288	139.649	17.78487	142.151
17.7313	119.663	17.65205	135.1641	17.63493	143.8726	17.7415	139.1406	17.66955	139.6522	17.80153	142.1499
17.74797	119.6567	17.66872	135.157	17.6516	143.872	17.75817	139.137	17.68622	139.6475	17.8182	142.1486
17.76463	119.6479	17.68538	135.1599	17.66827	143.8688	17.77483	139.1365	17.70288	139.6459	17.83487	142.1464
17.7813	119.6437	17.70205	135.1523	17.68493	143.8672	17.7915	139.1305	17.71955	139.645	17.85153	142.146
17.79797	119.6357	17.71872	135.1479	17.7016	143.8645	17.80817	139.1281	17.73622	139.6402	17.8682	142.1451
17.81463	119.6295	17.73538	135.147	17.71827	143.8633	17.82483	139.1254	17.75288	139.641	17.88487	142.1442
17.8313	119.6233	17.75205	135.1447	17.73493	143.8581	17.8415	139.1228	17.76955	139.6408	17.90153	142.1418

17.84797	119.6181	17.76872	135.136	17.7516	143.8584	17.85817	139.1224	17.78622	139.6373	17.9182	142.1393
17.86463	119.6109	17.78538	135.1347	17.76827	143.8539	17.87483	139.1201	17.80288	139.6367	17.93487	142.1411
17.8813	119.6074	17.80205	135.1287	17.78493	143.8545	17.8915	139.1159	17.81955	139.6366	17.95153	142.1387
17.89797	119.6007	17.81872	135.1233	17.8016	143.8554	17.90817	139.1145	17.83622	139.6339	17.9682	142.1383
17.91463	119.5945	17.83538	135.121	17.81827	143.8488	17.92483	139.112	17.85288	139.6311	17.98487	142.1387
17.9313	119.5896	17.85205	135.1196	17.83493	143.8471	17.9415	139.1107	17.86955	139.627	18.00153	142.1361
17.94797	119.583	17.86872	135.1153	17.8516	143.8453	17.95817	139.1077	17.88622	139.6284	18.0182	142.1363
17.96463	119.5769	17.88538	135.1061	17.86827	143.8422	17.97483	139.1052	17.90288	139.6288	18.03487	142.1334
17.9813	119.573	17.90205	135.1079	17.88493	143.8381	17.9915	139.104	17.91955	139.6275	18.05153	142.1338
17.99797	119.5635	17.91872	135.1061	17.9016	143.8368	18.00817	139.1004	17.93622	139.6267	18.0682	142.1284
18.01463	119.5623	17.93538	135.0983	17.91827	143.8379	18.02483	139.0987	17.95288	139.6218	18.08487	142.1292
18.0313	119.5513	17.95205	135.0973	17.93493	143.8328	18.0415	139.0939	17.96955	139.6222	18.10153	142.1301
18.04797	119.5457	17.96872	135.0925	17.9516	143.8322	18.05817	139.0944	17.98622	139.6224	18.1182	142.1285
18.06463	119.5403	17.98538	135.0885	17.96827	143.8278	18.07483	139.0915	18.00288	139.619	18.13487	142.1283
18.0813	119.5342	18.00205	135.0843	17.98493	143.8267	18.0915	139.0886	18.01955	139.6176	18.15153	142.1267
18.09797	119.5258	18.01872	135.0838	18.0016	143.8226	18.10817	139.0891	18.03622	139.6179	18.1682	142.1244
18.11463	119.5233	18.03538	135.0755	18.01827	143.822	18.12483	139.0859	18.05288	139.6155	18.18487	142.1255
18.1313	119.5166	18.05205	135.0719	18.03493	143.8206	18.1415	139.0813	18.06955	139.6134	18.20153	142.1233
18.14797	119.5106	18.06872	135.0709	18.0516	143.8183	18.15817	139.0828	18.08622	139.6125	18.2182	142.1206
18.16463	119.5055	18.08538	135.0657	18.06827	143.8167	18.17483	139.0792	18.10288	139.612	18.23487	142.1194
18.1813	119.4989	18.10205	135.0632	18.08493	143.8141	18.1915	139.0778	18.11955	139.6098	18.25153	142.1204
18.19797	119.4921	18.11872	135.0585	18.1016	143.8111	18.20817	139.077	18.13622	139.6097	18.2682	142.1198
18.21463	119.4886	18.13538	135.0563	18.11827	143.8093	18.22483	139.075	18.15288	139.6057	18.28487	142.1164
18.2313	119.4783	18.15205	135.0515	18.13493	143.8045	18.2415	139.0694	18.16955	139.6056	18.30153	142.1135
18.24797	119.4751	18.16872	135.0471	18.1516	143.8043	18.25817	139.0707	18.18622	139.6035	18.3182	142.1156
18.26463	119.4701	18.18538	135.0449	18.16827	143.8013	18.27483	139.0721	18.20288	139.6025	18.33487	142.1156
18.2813	119.4621	18.20205	135.0393	18.18493	143.8027	18.2915	139.0644	18.21955	139.5997	18.35153	142.1141
18.29797	119.4537	18.21872	135.0373	18.2016	143.7969	18.30817	139.0626	18.23622	139.5994	18.3682	142.1065
18.31463	119.4533	18.23538	135.0332	18.21827	143.7944	18.32483	139.0629	18.25288	139.5965	18.38487	142.11
18.3313	119.4458	18.25205	135.0313	18.23493	143.7919	18.3415	139.0579	18.26955	139.5942	18.40153	142.109
18.34797	119.4422	18.26872	135.025	18.2516	143.7917	18.35817	139.0608	18.28622	139.5928	18.4182	142.1087
18.36463	119.4341	18.28538	135.0246	18.26827	143.7867	18.37483	139.0578	18.30288	139.5906	18.43487	142.1086
18.3813	119.4307	18.30205	135.0232	18.28493	143.7857	18.3915	139.0543	18.31955	139.5889	18.45153	142.1031
18.39797	119.4236	18.31872	135.0171	18.3016	143.7792	18.40817	139.0538	18.33622	139.5886	18.4682	142.1053
18.41463	119.4161	18.33538	135.0165	18.31827	143.7837	18.42483	139.0535	18.35288	139.5869	18.48487	142.1036
18.4313	119.4115	18.35205	135.0109	18.33493	143.7782	18.4415	139.0497	18.36955	139.5825	18.50153	142.1046
18.44797	119.4015	18.36872	135.0069	18.3516	143.7763	18.45817	139.0461	18.38622	139.5817	18.5182	142.1013
18.46463	119.398	18.38538	135.004	18.36827	143.7729	18.47483	139.0443	18.40288	139.5813	18.53487	142.1012
18.4813	119.3939	18.40205	134.9995	18.38493	143.7712	18.4915	139.0435	18.41955	139.5785	18.55153	142.1022
18.49797	119.3882	18.41872	134.9976	18.4016	143.768	18.50817	139.0391	18.43622	139.5775	18.5682	142.0996
18.51463	119.3822	18.43538	134.9928	18.41827	143.7697	18.52483	139.0411	18.45288	139.5747	18.58487	142.1004
18.5313	119.3725	18.45205	134.9919	18.43493	143.7658	18.5415	139.0363	18.46955	139.571	18.60153	142.0999
18.54797	119.3698	18.46872	134.9845	18.4516	143.7635	18.55817	139.033	18.48622	139.5671	18.6182	142.0961
18.56463	119.3659	18.48538	134.9835	18.46827	143.7606	18.57483	139.0333	18.50288	139.5707	18.63487	142.0971
18.5813	119.3595	18.50205	134.9786	18.48493	143.7578	18.5915	139.0323	18.51955	139.5666	18.65153	142.0939
18.59797	119.3488	18.51872	134.9755	18.5016	143.7572	18.60817	139.029	18.53622	139.5643	18.6682	142.0954
18.61463	119.3482	18.53538	134.971	18.51827	143.7573	18.62483	139.0302	18.55288	139.563	18.68487	142.0927
18.6313	119.342	18.55205	134.9678	18.53493	143.751	18.6415	139.0238	18.56955	139.5649	18.70153	142.0944
18.64797	119.3359	18.56872	134.9664	18.5516	143.7505	18.65817	139.0249	18.58622	139.5623	18.7182	142.0896
18.66463	119.3288	18.58538	134.9623	18.56827	143.7506	18.67483	139.0248	18.60288	139.5579	18.73487	142.0905
18.6813	119.3242	18.60205	134.9587	18.58493	143.749	18.6915	139.0214	18.61955	139.5572	18.75153	142.0885
18.69797	119.3181	18.61872	134.9552	18.6016	143.7449	18.70817	139.0188	18.63622	139.5569	18.7682	142.0893
18.71463	119.3056	18.63538	134.9523	18.61827	143.7454	18.72483	139.0161	18.65288	139.5544	18.78487	142.086
18.7313	119.3129	18.65205	134.9497	18.63493	143.7409	18.7415	139.0145	18.66955	139.5536	18.80153	142.086
18.74797	119.293	18.66872	134.9449	18.6516	143.7378	18.75817	139.0132	18.68622	139.5486	18.8182	142.0857
18.76463	119.2921	18.68538	134.9451	18.66827	143.7385	18.77483	139.0131	18.70288	139.549	18.83487	142.0843
18.7813	119.2918	18.70205	134.9324	18.68493	143.736	18.7915	139.0114	18.71955	139.5509	18.85153	142.0846
18.79797	119.2808	18.71872	134.9387	18.7016	143.7323	18.80817	139.0096	18.73622	139.5472	18.8682	142.0814
18.81463	119.2776	18.73538	134.9318	18.71827	143.7326	18.82483	139.0049	18.75288	139.5465	18.88487	142.0814
18.8313	119.2721	18.75205	134.9285	18.73493	143.7284	18.8415	139.0025	18.76955	139.5445	18.90153	142.0818
18.84797	119.2647	18.76872	134.9264	18.7516	143.7267	18.85817	139.0029	18.78622	139.543	18.9182	142.0813
18.86463	119.2598	18.78538	134.9242	18.76827	143.7257	18.87483	139.0029	18.80288	139.5422	18.93487	142.0781
18.8813	119.2546	18.80205	134.9167	18.78493	143.723	18.8915	138.9986	18.81955	139.5403	18.95153	142.0791
18.89797	119.2495	18.81872	134.9158	18.8016	143.721	18.90817	138.9971	18.83622	139.5414	18.9682	142.0769
18.91463	119.2413	18.83538	134.9118	18.81827	143.7212	18.92483	138.9971	18.85288	139.5357	18.98487	142.0772
18.9313	119.2377	18.85205	134.9118	18.83493	143.7184	18.9415	138.9916	18.86955	139.5375	19.00153	142.0769
18.94797	119.2259	18.86872	134.9049	18.8516	143.7149	18.95817	138.9906	18.88622	139.5372	19.0182	142.0725
18.96463	119.2185	18.88538	134.9034	18.86827	143.7133	18.97483	138.9909	18.90288	139.5333	19.03487	142.0768
18.9813	119.2179	18.90205	134.9	18.88493	143.709	18.9915	138.989	18.91955	139.5337	19.05153	142.0763
18.99797	119.2119	18.91872	134.8986	18.9016	143.7069	19.00817	138.9856	18.93622	139.5297	19.0682	142.0741
19.01463	119.2078	18.93538	134.8907	18.91827	143.7064	19.02483	138.984	18.95288	139.5291	19.08487	142.0729
19.0313	119.2049	18.95205	134.8888	18.93493	143.7019	19.0415	138.9809	18.96955	139.5259	19.10153	142.0711
19.04797	119.1967	18.96872	134.8878	18.9516	143.6989	19.05817	138.9812	18.98622	139.5263	19.1182	142.0711
19.06463	119.1916	18.98538	134.8844	18.96827	143.699	19.07483	138.979	19.00288	139.5245	19.13487	142.0695
19.0813	119.1837	19.00205	134.8776	18.98493	143.6981	19.0915	138.98	19.01955	139.5241	19.15153	142.0675
19.09797	119.1817	19.01872	134.878	19.0016	143.6929	19.10817	138.9755	19.03622	139.5216	19.1682	142.0707
19.11463	119.1729	19.03538	134.8736	19.01827	143.6941	19.12483	138.9727	19.05288	139.5198	19.18487	142.0655
19.1313	119.1663	19.05205	134.8692	19.03493	143.6914	19.1415	138.9729	19.06955	139.5205	19.20153	142.0644
19.14797	119.1623	19.06872	134.8647	19.0516	143.688	19.15817	138.9714	19.08622	139.5182	19.2182	142.0617
19.16463	119.1561	19.08538	134.8609	19.06827	143.6861	19.17483	138.9644	19.10288	139.5153	19.23487	142.0634
19.1813	119.1507	19.10205	134.8578	19.08493	143.6858	19.1915	138.9668	19.11955	139.5145	19.25153	142.063
19.19797	119.1435	19.11872	134.8572	19.1016	143.68	19.20817	138.9658	19.13622	139.5158	19.2682	142.0613
19.21463	119.1399	19.13538	134.8496	19.11827	143.6817	19.22483	138.9633	19.15288	139.5115	19.28487	142.0581
19.2313	119.1363	19.15205	134.8457	19.13493	143.6785	19.2415	138.96	19.16955	139.5106	19.30153	142.0609
19.24797	119.1303	19.16872	134.842	19.1516	143.6742	19.25817	138.9602	19.18622	139.5118	19.3182	142.0577
19.26463	119.1228	19.18538	134.8392	19.16827	143.6743	19.27483	138.9567	19.20288	139.509	19.33487	142.0563
19.2813	119.1109	19.20205	134.8349	19.18493	143.673	19.2915	138.9565	19.21955	139.5057	19.35153	142.055
19.29797	119.1088	19.21872	134.8342	19.2016	143.6679	19.30817	138.9535	19.23622	139.5054	19.3682	142.0567
19.31463	119.1032	19.23538	134.8272	19.21827	143.6683	19.32483	138.9541	19.25288	139.5061	19.38487	142.0547
19.3313	119.0991	19.25205	134.8285	19.23493	143.6654	19.3415	138.9516	19.26955	139.5038	19.40153	142.0527
19.34797	119.0941	19.26872	134.8226	19.2516	143.6642	19.35817	138.9493	19.28622	139.5012	19.4182	142.0521
19.36463	119.0884	19.28538	134.8214	19.26827	143.6618	19.37483	138.9458	19.30288	139.5025	19.43487	142.0536
19.3813	119.083	19.30205	134.8181	19.28493	143.6603	19.3915	138.9467	19.31955	139.4982	19.45153	142.0501
19.39797	119.0759	19.31872	134.8152	19.3016	143.6571	19.40817	138.9456	19.33622	139.4949	19.4682	142.0513
19.41463	119.0693	19.33538	134.8101	19.31827	143.6571	19.42483	138.9417	19.35288	139.4982	19.48487	142.0505
19.4313	119.0656	19.35205	134.8095	19.33493	143.6542	19.4415	138.941	19.36955	139.4969	19.50153	142.0481
19.44797	119.058	19.36872	134.8032	19.3516	143.6522	19.45817	138.9393	19.38622	139.4989	19.5182	142.0487

19.46463	119.0544	19.38538	134.8029	19.36827	143.6482	19.47483	138.937	19.40288	139.4958	19.53487	142.0444
19.4813	119.048	19.40205	134.7974	19.38493	143.648	19.4915	138.9324	19.41955	139.4944	19.55153	142.0464
19.49797	119.0455	19.41872	134.7946	19.4016	143.645	19.50817	138.9351	19.43622	139.4904	19.5682	142.0458
19.51463	119.0385	19.43538	134.7909	19.41827	143.6452	19.52483	138.9342	19.45288	139.4901	19.58487	142.0473
19.5313	119.0315	19.45205	134.7878	19.43493	143.6413	19.5415	138.93	19.46955	139.4913	19.60153	142.0447
19.54797	119.0269	19.46872	134.7841	19.4516	143.6366	19.55817	138.9268	19.48622	139.4879	19.6182	142.0464
19.56463	119.0202	19.48538	134.7831	19.46827	143.6386	19.57483	138.9297	19.50288	139.487	19.63487	142.0433
19.5813	119.0136	19.50205	134.7777	19.48493	143.6337	19.5915	138.9238	19.51955	139.4847	19.65153	142.0429
19.59797	119.0115	19.51872	134.7759	19.5016	143.6309	19.60817	138.9238	19.53622	139.4832	19.6682	142.0413
19.61463	119.0046	19.53538	134.7735	19.51827	143.6298	19.62483	138.9282	19.55288	139.4829	19.68487	142.0407
19.6313	118.9969	19.55205	134.7734	19.53493	143.627	19.6415	138.9201	19.56955	139.4798	19.70153	142.039
19.64797	118.9925	19.56872	134.7654	19.5516	143.6268	19.65817	138.9178	19.58622	139.4785	19.7182	142.0396
19.66463	118.9873	19.58538	134.764	19.56827	143.6233	19.67483	138.9145	19.60288	139.4793	19.73487	142.0366
19.6813	118.9821	19.60205	134.7604	19.58493	143.6221	19.6915	138.9154	19.61955	139.4772	19.75153	142.0366
19.69797	118.9757	19.61872	134.7589	19.6016	143.6204	19.70817	138.9107	19.63622	139.4738	19.7682	142.0359
19.71463	118.9689	19.63538	134.7515	19.61827	143.617	19.72483	138.9132	19.65288	139.4738	19.78487	142.0362
19.7313	118.964	19.65205	134.7539	19.63493	143.6164	19.7415	138.9059	19.66955	139.4728	19.80153	142.0346
19.74797	118.9561	19.66872	134.7479	19.6516	143.6148	19.75817	138.9085	19.68622	139.4699	19.8182	142.035
19.76463	118.9532	19.68538	134.7452	19.66827	143.6077	19.77483	138.9061	19.70288	139.4695	19.83487	142.0344
19.7813	118.9451	19.70205	134.7412	19.68493	143.6114	19.7915	138.9029	19.71955	139.4702	19.85153	142.0332
19.79797	118.9409	19.71872	134.7391	19.7016	143.6083	19.80817	138.9016	19.73622	139.4661	19.8682	142.0314
19.81463	118.9321	19.73538	134.7356	19.71827	143.6042	19.82483	138.9005	19.75288	139.4668	19.88487	142.0323
19.8313	118.9267	19.75205	134.733	19.73493	143.6024	19.8415	138.8972	19.76955	139.4647	19.90153	142.0306
19.84797	118.9237	19.76872	134.7263	19.7516	143.6025	19.85817	138.8986	19.78622	139.4641	19.9182	142.0294
19.86463	118.9158	19.78538	134.7276	19.76827	143.5978	19.87483	138.8949	19.80288	139.4634	19.93487	142.0318
19.8813	118.9103	19.80205	134.7235	19.78493	143.5937	19.8915	138.8943	19.81955	139.4631	19.95153	142.0282
19.89797	118.903	19.81872	134.7212	19.8016	143.5925	19.90817	138.8907	19.83622	139.4589	19.9682	142.0294
19.91463	118.8979	19.83538	134.7152	19.81827	143.5933	19.92483	138.89	19.85288	139.4593	19.98487	142.0273
19.9313	118.8922	19.85205	134.7137	19.83493	143.5915	19.9415	138.8865	19.86955	139.4589	20.00153	142.0266
19.94797	118.884	19.86872	134.7106	19.8516	143.5898	19.95817	138.8874	19.88622	139.4574	20.0182	142.0247
19.96463	118.8817	19.88538	134.7073	19.86827	143.5894	19.97483	138.8865	19.90288	139.4568	20.03487	142.0242
19.9813	118.8733	19.90205	134.7015	19.88493	143.5854	19.9915	138.8834	19.91955	139.4584	20.05153	142.0267
19.99797	118.8678	19.91872	134.6979	19.9016	143.5841	20.00817	138.8831	19.93622	139.4553	20.0682	142.0234
20.01463	118.8641	19.93538	134.6976	19.91827	143.5795	20.02483	138.8801	19.95288	139.4526	20.08487	142.0228
20.0313	118.8596	19.95205	134.6955	19.93493	143.577	20.0415	138.8805	19.96955	139.4531	20.10153	142.0217
20.04797	118.8511	19.96872	134.6885	19.9516	143.5736	20.05817	138.8784	19.98622	139.4513	20.1182	142.0214
20.06463	118.8441	19.98538	134.6867	19.96827	143.5736	20.07483	138.8742	20.00288	139.4497	20.13487	142.022
20.0813	118.8419	20.00205	134.6831	19.98493	143.5702	20.0915	138.875	20.01955	139.4502	20.15153	142.0182
20.09797	118.8367	20.01872	134.6778	20.0016	143.5673	20.10817	138.8718	20.03622	139.4456	20.1682	142.018
20.11463	118.8299	20.03538	134.6781	20.01827	143.5653	20.12483	138.8717	20.05288	139.4458	20.18487	142.0183
20.1313	118.8239	20.05205	134.6736	20.03493	143.5627	20.1415	138.8704	20.06955	139.4452	20.20153	142.0182
20.14797	118.8202	20.06872	134.6736	20.0516	143.5593	20.15817	138.8649	20.08622	139.4427	20.2182	142.015
20.16463	118.8139	20.08538	134.6684	20.06827	143.5564	20.17483	138.8656	20.10288	139.4403	20.23487	142.0158
20.1813	118.8082	20.10205	134.6661	20.08493	143.5573	20.1915	138.8644	20.11955	139.4396	20.25153	142.0155
20.19797	118.8027	20.11872	134.66	20.1016	143.5521	20.20817	138.862	20.13622	139.4394	20.2682	142.0143
20.21463	118.7973	20.13538	134.6576	20.11827	143.5502	20.22483	138.8599	20.15288	139.4371	20.28487	142.0133
20.2313	118.7933	20.15205	134.6555	20.13493	143.5468	20.2415	138.8567	20.16955	139.4393	20.30153	142.0137
20.24797	118.7842	20.16872	134.6495	20.1516	143.5458	20.25817	138.8558	20.18622	139.435	20.3182	142.0134
20.26463	118.7805	20.18538	134.6485	20.16827	143.5423	20.27483	138.8564	20.20288	139.4348	20.33487	142.0112
20.2813	118.7772	20.20205	134.6464	20.18493	143.5416	20.2915	138.8585	20.21955	139.4343	20.35153	142.0122
20.29797	118.7691	20.21872	134.6447	20.2016	143.5413	20.30817	138.8527	20.23622	139.432	20.3682	142.0087
20.31463	118.7669	20.23538	134.6351	20.21827	143.538	20.32483	138.8502	20.25288	139.4333	20.38487	142.0102
20.3313	118.7603	20.25205	134.6354	20.23493	143.5381	20.3415	138.8513	20.26955	139.4279	20.40153	142.0092
20.34797	118.7543	20.26872	134.6301	20.2516	143.5334	20.35817	138.8468	20.28622	139.4272	20.4182	142.0087
20.36463	118.7491	20.28538	134.6303	20.26827	143.533	20.37483	138.8484	20.30288	139.4264	20.43487	142.0063
20.3813	118.7453	20.30205	134.6266	20.28493	143.5321	20.3915	138.8476	20.31955	139.4236	20.45153	142.0064
20.39797	118.7379	20.31872	134.6229	20.3016	143.5267	20.40817	138.8432	20.33622	139.4228	20.4682	142.0076
20.41463	118.7314	20.33538	134.6198	20.31827	143.5237	20.42483	138.8444	20.35288	139.4228	20.48487	142.0021
20.4313	118.726	20.35205	134.6188	20.33493	143.5227	20.4415	138.8395	20.36955	139.4213	20.50153	142.0021
20.44797	118.7221	20.36872	134.6143	20.3516	143.5226	20.45817	138.8398	20.38622	139.4199	20.5182	142.0005
20.46463	118.7149	20.38538	134.6097	20.36827	143.5207	20.47483	138.8364	20.40288	139.4177	20.53487	142.0012
20.4813	118.7123	20.40205	134.6079	20.38493	143.5183	20.4915	138.8338	20.41955	139.4176	20.55153	141.9971
20.49797	118.7037	20.41872	134.6043	20.4016	143.5154	20.50817	138.8304	20.43622	139.4163	20.5682	141.9986
20.51463	118.6973	20.43538	134.5994	20.41827	143.5146	20.52483	138.8332	20.45288	139.4141	20.58487	141.9956
20.5313	118.6964	20.45205	134.5969	20.43493	143.5085	20.5415	138.8287	20.46955	139.4105	20.60153	141.9995
20.54797	118.6863	20.46872	134.5924	20.4516	143.5091	20.55817	138.8283	20.48622	139.4072	20.6182	141.9962
20.56463	118.6802	20.48538	134.5889	20.46827	143.506	20.57483	138.821	20.50288	139.4114	20.63487	141.9964
20.5813	118.6779	20.50205	134.5873	20.48493	143.5039	20.5915	138.8241	20.51955	139.4109	20.65153	141.9929
20.59797	118.6696	20.51872	134.583	20.5016	143.5014	20.60817	138.8226	20.53622	139.4075	20.6682	141.9969
20.61463	118.6644	20.53538	134.5786	20.51827	143.4994	20.62483	138.8216	20.55288	139.4072	20.68487	141.9923
20.6313	118.6577	20.55205	134.5779	20.53493	143.498	20.6415	138.8207	20.56955	139.4066	20.70153	141.9915
20.64797	118.6535	20.56872	134.5741	20.5516	143.4963	20.65817	138.8152	20.58622	139.4011	20.7182	141.9922
20.66463	118.6478	20.58538	134.5716	20.56827	143.4917	20.67483	138.8143	20.60288	139.4013	20.73487	141.9913
20.6813	118.6407	20.60205	134.5695	20.58493	143.4912	20.6915	138.8139	20.61955	139.403	20.75153	141.9886
20.69797	118.6373	20.61872	134.5648	20.6016	143.4879	20.70817	138.8096	20.63622	139.398	20.7682	141.9898
20.71463	118.6323	20.63538	134.5629	20.61827	143.4886	20.72483	138.8099	20.65288	139.3971	20.78487	141.9859
20.7313	118.6239	20.65205	134.5603	20.63493	143.4858	20.7415	138.8069	20.66955	139.3951	20.80153	141.9873
20.74797	118.6188	20.66872	134.5542	20.6516	143.4849	20.75817	138.8082	20.68622	139.3956	20.8182	141.9847
20.76463	118.6177	20.68538	134.5521	20.66827	143.4797	20.77483	138.8047	20.70288	139.3938	20.83487	141.9847
20.7813	118.6094	20.70205	134.55	20.68493	143.4805	20.7915	138.8035	20.71955	139.3914	20.85153	141.9836
20.79797	118.6068	20.71872	134.5473	20.7016	143.4759	20.80817	138.8046	20.73622	139.3884	20.8682	141.9841
20.81463	118.6023	20.73538	134.5453	20.71827	143.4735	20.82483	138.8002	20.75288	139.3899	20.88487	141.9821
20.8313	118.5979	20.75205	134.5402	20.73493	143.4705	20.8415	138.7982	20.76955	139.3883	20.90153	141.9805
20.84797	118.5891	20.76872	134.5356	20.7516	143.4748	20.85817	138.7962	20.78622	139.3882	20.9182	141.9819
20.86463	118.5838	20.78538	134.5379	20.76827	143.469	20.87483	138.7948	20.80288	139.3852	20.93487	141.9806
20.8813	118.5801	20.80205	134.5323	20.78493	143.4685	20.8915	138.795	20.81955	139.3834	20.95153	141.9795
20.89797	118.5725	20.81872	134.5278	20.8016	143.4657	20.90817	138.7941	20.83622	139.3832	20.9682	141.9767
20.91463	118.5698	20.83538	134.5259	20.81827	143.4624	20.92483	138.7893	20.85288	139.3792	20.98487	141.977
20.9313	118.5645	20.85205	134.5234	20.83493	143.4622	20.9415	138.7868	20.86955	139.379	21.00153	141.9743
20.94797	118.559	20.86872	134.5176	20.8516	143.4575	20.95817	138.7879	20.88622	139.3788	21.0182	141.9759
20.96463	118.5528	20.88538	134.5169	20.86827	143.4577	20.97483	138.7812	20.90288	139.3764	21.03487	141.9741
20.9813	118.5483	20.90205	134.5141	20.88493	143.456	20.9915	138.7854	20.91955	139.3746	21.05153	141.9706
20.99797	118.5416	20.91872	134.5097	20.9016	143.4507	21.00817	138.7805	20.93622	139.3741	21.0682	141.971
21.01463	118.5377	20.93538	134.5083	20.91827	143.4496	21.02483	138.7811	20.95288	139.3737	21.08487	141.97
21.0313	118.5323	20.95205	134.504	20.93493	143.4463	21.0415	138.7777	20.96955	139.3715	21.10153	141.9663
21.04797	118.5276	20.96872	134.4984	20.9516	143.4441	21.05817	138.778	20.98622	139.3696	21.1182	141.9673
21.06463	118.519	20.98538	134.4949	20.96827	143.44	21.07483	138.7753	21.00288	139.3679	21.13487	141.9668

21.0813	118.5098	21.00205	134.4967	20.98493	143.4407	21.0915	138.7738	21.01955	139.367	21.15153	141.9662
21.09797	118.5098	21.01872	134.4915	21.0016	143.4382	21.10817	138.7722	21.03622	139.3659	21.1682	141.9645
21.11463	118.5038	21.03538	134.4859	21.01827	143.4347	21.12483	138.7712	21.05288	139.3646	21.18487	141.9629
21.1313	118.4974	21.05205	134.4874	21.03493	143.4359	21.1415	138.7692	21.06955	139.3618	21.20153	141.9612
21.14797	118.4926	21.06872	134.4818	21.0516	143.4309	21.15817	138.7654	21.08622	139.3619	21.2182	141.9619
21.16463	118.4863	21.08538	134.4799	21.06827	143.4305	21.17483	138.7663	21.10288	139.3592	21.23487	141.96
21.1813	118.4822	21.10205	134.4734	21.08493	143.4265	21.1915	138.7645	21.11955	139.3582	21.25153	141.9596
21.19797	118.477	21.11872	134.4734	21.1016	143.4239	21.20817	138.7615	21.13622	139.3549	21.2682	141.9585
21.21463	118.4722	21.13538	134.4681	21.11827	143.4276	21.22483	138.7582	21.15288	139.355	21.28487	141.9573
21.2313	118.467	21.15205	134.4668	21.13493	143.4213	21.2415	138.7558	21.16955	139.3536	21.30153	141.9562
21.24797	118.4642	21.16872	134.4627	21.1516	143.4186	21.25817	138.7576	21.18622	139.352	21.3182	141.9547
21.26463	118.4587	21.18538	134.4598	21.16827	143.4159	21.27483	138.7553	21.20288	139.3525	21.33487	141.9567
21.2813	118.4513	21.20205	134.4557	21.18493	143.4159	21.2915	138.7522	21.21955	139.3477	21.35153	141.9526
21.29797	118.448	21.21872	134.4527	21.2016	143.4142	21.30817	138.7508	21.23622	139.3496	21.3682	141.9533
21.31463	118.4441	21.23538	134.4542	21.21827	143.4114	21.32483	138.7495	21.25288	139.3495	21.38487	141.9539
21.3313	118.4364	21.25205	134.447	21.23493	143.4056	21.3415	138.7501	21.26955	139.3456	21.40153	141.9521
21.34797	118.4314	21.26872	134.4454	21.2516	143.407	21.35817	138.7449	21.28622	139.3434	21.4182	141.9521
21.36463	118.4259	21.28538	134.442	21.26827	143.4048	21.37483	138.747	21.30288	139.343	21.43487	141.9503
21.3813	118.4232	21.30205	134.4382	21.28493	143.4028	21.3915	138.7453	21.31955	139.3415	21.45153	141.9482
21.39797	118.4186	21.31872	134.4365	21.3016	143.4041	21.40817	138.743	21.33622	139.3388	21.4682	141.9479
21.41463	118.4109	21.33538	134.432	21.31827	143.3981	21.42483	138.7434	21.35288	139.3392	21.48487	141.9487
21.4313	118.405	21.35205	134.4312	21.33493	143.3934	21.4415	138.7375	21.36955	139.3361	21.50153	141.9484
21.44797	118.4018	21.36872	134.4277	21.3516	143.3935	21.45817	138.7363	21.38622	139.3354	21.5182	141.9462
21.46463	118.3955	21.38538	134.4239	21.36827	143.3922	21.47483	138.7338	21.40288	139.3329	21.53487	141.9473
21.4813	118.3904	21.40205	134.4189	21.38493	143.3883	21.4915	138.7322	21.41955	139.3323	21.55153	141.9444
21.49797	118.3843	21.41872	134.42	21.4016	143.3866	21.50817	138.7336	21.43622	139.3306	21.5682	141.9433
21.51463	118.3797	21.43538	134.4158	21.41827	143.3843	21.52483	138.7329	21.45288	139.3306	21.58487	141.9415
21.5313	118.3738	21.45205	134.4062	21.43493	143.3817	21.5415	138.7307	21.46955	139.3289	21.60153	141.9422
21.54797	118.3689	21.46872	134.4075	21.4516	143.3819	21.55817	138.7303	21.48622	139.3285	21.6182	141.9433
21.56463	118.3651	21.48538	134.4079	21.46827	143.3773	21.57483	138.728	21.50288	139.3276	21.63487	141.9419
21.5813	118.3592	21.50205	134.4	21.48493	143.3738	21.5915	138.7266	21.51955	139.3243	21.65153	141.9378
21.59797	118.3555	21.51872	134.3974	21.5016	143.3746	21.60817	138.723	21.53622	139.322	21.6682	141.94
21.61463	118.3483	21.53538	134.3992	21.51827	143.3721	21.62483	138.7238	21.55288	139.322	21.68487	141.9392
21.6313	118.344	21.55205	134.3937	21.53493	143.3667	21.6415	138.722	21.56955	139.3234	21.70153	141.9387
21.64797	118.3396	21.56872	134.3885	21.5516	143.3674	21.65817	138.7215	21.58622	139.3206	21.7182	141.9376
21.66463	118.3315	21.58538	134.3919	21.56827	143.3637	21.67483	138.7204	21.60288	139.3192	21.73487	141.9371
21.6813	118.3246	21.60205	134.3876	21.58493	143.3614	21.6915	138.7194	21.61955	139.3191	21.75153	141.9371
21.69797	118.3196	21.61872	134.3857	21.6016	143.3598	21.70817	138.7183	21.63622	139.3186	21.7682	141.9336
21.71463	118.3153	21.63538	134.3814	21.61827	143.3582	21.72483	138.7172	21.65288	139.3156	21.78487	141.9333
21.7313	118.3122	21.65205	134.3772	21.63493	143.3541	21.7415	138.7136	21.66955	139.3171	21.80153	141.9345
21.74797	118.3071	21.66872	134.3758	21.6516	143.3547	21.75817	138.715	21.68622	139.3156	21.8182	141.9316
21.76463	118.3002	21.68538	134.3733	21.66827	143.351	21.77483	138.7073	21.70288	139.3141	21.83487	141.9312
21.7813	118.2982	21.70205	134.3695	21.68493	143.3515	21.7915	138.7101	21.71955	139.3123	21.85153	141.931
21.79797	118.2884	21.71872	134.3667	21.7016	143.3469	21.80817	138.7092	21.73622	139.3111	21.8682	141.9274
21.81463	118.2851	21.73538	134.3612	21.71827	143.3457	21.82483	138.7096	21.75288	139.3097	21.88487	141.9267
21.8313	118.2786	21.75205	134.3616	21.73493	143.3459	21.8415	138.7075	21.76955	139.3105	21.90153	141.9273
21.84797	118.2734	21.76872	134.3559	21.7516	143.3408	21.85817	138.7043	21.78622	139.3087	21.9182	141.9272
21.86463	118.2668	21.78538	134.352	21.76827	143.3391	21.87483	138.7024	21.80288	139.306	21.93487	141.9244
21.8813	118.2635	21.80205	134.3466	21.78493	143.3361	21.8915	138.699	21.81955	139.3068	21.95153	141.9244
21.89797	118.2569	21.81872	134.3499	21.8016	143.3332	21.90817	138.7009	21.83622	139.3044	21.9682	141.9227
21.91463	118.2522	21.83538	134.3441	21.81827	143.3339	21.92483	138.6973	21.85288	139.3052	21.98487	141.9218
21.9313	118.2458	21.85205	134.3412	21.83493	143.3311	21.9415	138.6952	21.86955	139.3046	22.00153	141.9206
21.94797	118.242	21.86872	134.3374	21.8516	143.3328	21.95817	138.6925	21.88622	139.3052	22.0182	141.9204
21.96463	118.2353	21.88538	134.3382	21.86827	143.3251	21.97483	138.6949	21.90288	139.2999	22.03487	141.918
21.9813	118.231	21.90205	134.3358	21.88493	143.3221	21.9915	138.6947	21.91955	139.3005	22.05153	141.9189
21.99797	118.2278	21.91872	134.3285	21.9016	143.3219	22.00817	138.6887	21.93622	139.2986	22.0682	141.9178
22.01463	118.2212	21.93538	134.3276	21.91827	143.3233	22.02483	138.6895	21.95288	139.2979	22.08487	141.9161
22.0313	118.2152	21.95205	134.3165	21.93493	143.3207	22.0415	138.6877	21.96955	139.2985	22.10153	141.9144
22.04797	118.2098	21.96872	134.3189	21.9516	143.3183	22.05817	138.6891	21.98622	139.2961	22.1182	141.9128
22.06463	118.206	21.98538	134.3098	21.96827	143.3176	22.07483	138.6876	22.00288	139.2943	22.13487	141.912
22.0813	118.2015	22.00205	134.3132	21.98493	143.3151	22.0915	138.6824	22.01955	139.2936	22.15153	141.9129
22.09797	118.193	22.01872	134.3203	22.0016	143.3125	22.10817	138.6819	22.03622	139.2931	22.1682	141.9118
22.11463	118.1896	22.03538	134.3057	22.01827	143.3083	22.12483	138.6816	22.05288	139.2921	22.18487	141.908
22.1313	118.1853	22.05205	134.3043	22.03493	143.3041	22.1415	138.6783	22.06955	139.2899	22.20153	141.9103
22.14797	118.1813	22.06872	134.3016	22.0516	143.3071	22.15817	138.6798	22.08622	139.2888	22.2182	141.9086
22.16463	118.1727	22.08538	134.2972	22.06827	143.3054	22.17483	138.6757	22.10288	139.2882	22.23487	141.9078
22.1813	118.1689	22.10205	134.2908	22.08493	143.3041	22.1915	138.6764	22.11955	139.287	22.25153	141.906
22.19797	118.1632	22.11872	134.2946	22.1016	143.3016	22.20817	138.6728	22.13622	139.2872	22.2682	141.9066
22.21463	118.159	22.13538	134.2909	22.11827	143.2976	22.22483	138.6738	22.15288	139.2857	22.28487	141.9059
22.2313	118.1558	22.15205	134.2841	22.13493	143.2944	22.2415	138.6713	22.16955	139.2844	22.30153	141.9047
22.24797	118.1501	22.16872	134.2837	22.1516	143.2917	22.25817	138.6686	22.18622	139.2842	22.3182	141.9019
22.26463	118.1416	22.18538	134.2858	22.16827	143.2902	22.27483	138.6662	22.20288	139.2823	22.33487	141.9026
22.2813	118.1438	22.20205	134.2744	22.18493	143.2871	22.2915	138.6695	22.21955	139.2851	22.35153	141.9023
22.29797	118.1364	22.21872	134.2722	22.2016	143.2845	22.30817	138.6663	22.23622	139.2785	22.3682	141.9008
22.31463	118.1279	22.23538	134.2729	22.21827	143.2795	22.32483	138.6641	22.25288	139.2797	22.38487	141.8988
22.3313	118.1226	22.25205	134.2684	22.23493	143.2811	22.3415	138.6628	22.26955	139.2801	22.40153	141.8975
22.34797	118.1182	22.26872	134.262	22.2516	143.2789	22.35817	138.666	22.28622	139.2788	22.4182	141.8975
22.36463	118.1158	22.28538	134.2683	22.26827	143.2772	22.37483	138.6656	22.30288	139.2768	22.43487	141.8945
22.3813	118.1134	22.30205	134.2636	22.28493	143.2728	22.3915	138.6616	22.31955	139.2769	22.45153	141.8939
22.39797	118.107	22.31872	134.2596	22.3016	143.2722	22.40817	138.6567	22.33622	139.2734	22.4682	141.894
22.41463	118.1026	22.33538	134.2572	22.31827	143.2701	22.42483	138.6569	22.35288	139.2728	22.48487	141.8937
22.4313	118.0975	22.35205	134.2561	22.33493	143.2655	22.4415	138.6569	22.36955	139.2713	22.50153	141.8903
22.44797	118.094	22.36872	134.2504	22.3516	143.2643	22.45817	138.6553	22.38622	139.2682	22.5182	141.8931
22.46463	118.0873	22.38538	134.2489	22.36827	143.2644	22.47483	138.6545	22.40288	139.2701	22.53487	141.8908
22.4813	118.0815	22.40205	134.2471	22.38493	143.2588	22.4915	138.6526	22.41955	139.2693	22.55153	141.8892
22.49797	118.077	22.41872	134.2435	22.4016	143.2583	22.50817	138.6502	22.43622	139.2675	22.5682	141.887
22.51463	118.0719	22.43538	134.2414	22.41827	143.2563	22.52483	138.6528	22.45288	139.2664	22.58487	141.8872
22.5313	118.0666	22.45205	134.2382	22.43493	143.2527	22.5415	138.6488	22.46955	139.2638	22.60153	141.8858
22.54797	118.0641	22.46872	134.2334	22.4516	143.251	22.55817	138.6474	22.48622	139.2632	22.6182	141.8854
22.56463	118.0604	22.48538	134.2331	22.46827	143.2502	22.57483	138.6459	22.50288	139.262	22.63487	141.8821
22.5813	118.0539	22.50205	134.2239	22.48493	143.2525	22.5915	138.6464	22.51955	139.2632	22.65153	141.8835
22.59797	118.0478	22.51872	134.2255	22.5016	143.2453	22.60817	138.6466	22.53622	139.2587	22.6682	141.8845
22.61463	118.0442	22.53538	134.228	22.51827	143.2458	22.62483	138.6425	22.55288	139.2571	22.68487	141.881
22.6313	118.0413	22.55205	134.2232	22.53493	143.2445	22.6415	138.6423	22.56955	139.2582	22.70153	141.8773
22.64797	118.0353	22.56872	134.2187	22.5516	143.238	22.65817	138.6424	22.58622	139.258	22.7182	141.8771
22.66463	118.0294	22.58538	134.2205	22.56827	143.238	22.67483	138.6401	22.60288	139.2553	22.73487	141.8794
22.6813	118.0255	22.60205	134.2114	22.58493	143.2394	22.6915	138.6381	22.61955	139.2568	22.75153	141.8753

22.69797	118.0186	22.61872	134.2096	22.6016	143.2317	22.70817	138.6396	22.63622	139.2528	22.7682	141.8758
22.71463	118.0168	22.63538	134.2106	22.61827	143.2325	22.72483	138.6334	22.65288	139.2523	22.78487	141.8737
22.7313	118.0105	22.65205	134.2051	22.63493	143.2333	22.7415	138.6347	22.66955	139.2534	22.80153	141.8726
22.74797	118.0047	22.66872	134.2013	22.6516	143.2303	22.75817	138.6334	22.68622	139.25	22.8182	141.8705
22.76463	117.9978	22.68538	134.2036	22.66827	143.2296	22.77483	138.6325	22.70288	139.2494	22.83487	141.8696
22.7813	117.9944	22.70205	134.1956	22.68493	143.2256	22.7915	138.6294	22.71955	139.2503	22.85153	141.8695
22.79797	117.9902	22.71872	134.1948	22.7016	143.2227	22.80817	138.6322	22.73622	139.2479	22.8682	141.8686
22.81463	117.9852	22.73538	134.1907	22.71827	143.2205	22.82483	138.6303	22.75288	139.2496	22.88487	141.8651
22.8313	117.9819	22.75205	134.1883	22.73493	143.2213	22.8415	138.6301	22.76955	139.2455	22.90153	141.8666
22.84797	117.9767	22.76872	134.1855	22.7516	143.215	22.85817	138.6274	22.78622	139.2477	22.9182	141.8653
22.86463	117.9706	22.78538	134.1829	22.76827	143.2165	22.87483	138.6265	22.80288	139.2434	22.93487	141.8623
22.8813	117.9664	22.80205	134.1781	22.78493	143.2129	22.8915	138.6252	22.81955	139.2417	22.95153	141.8603
22.89797	117.9609	22.81872	134.1778	22.8016	143.2111	22.90817	138.623	22.83622	139.2451	22.9682	141.8608
22.91463	117.9562	22.83538	134.1715	22.81827	143.2086	22.92483	138.6222	22.85288	139.2411	22.98487	141.8607
22.9313	117.9483	22.85205	134.1686	22.83493	143.2091	22.9415	138.6205	22.86955	139.2398	23.00153	141.8589
22.94797	117.9465	22.86872	134.1681	22.8516	143.2029	22.95817	138.6189	22.88622	139.2387	23.0182	141.8567
22.96463	117.942	22.88538	134.1627	22.86827	143.2015	22.97483	138.6182	22.90288	139.2378	23.03487	141.8567
22.9813	117.936	22.90205	134.1607	22.88493	143.2018	22.9915	138.617	22.91955	139.2369	23.05153	141.8533
22.99797	117.9335	22.91872	134.1565	22.9016	143.1987	23.00817	138.6167	22.93622	139.2354	23.0682	141.8537
23.01463	117.929	22.93538	134.1544	22.91827	143.1979	23.02483	138.6139	22.95288	139.2339	23.08487	141.8533
23.0313	117.9229	22.95205	134.1489	22.93493	143.1919	23.0415	138.6147	22.96955	139.2315	23.10153	141.85
23.04797	117.9177	22.96872	134.1456	22.9516	143.1927	23.05817	138.6106	22.98622	139.2287	23.1182	141.8498
23.06463	117.9119	22.98538	134.1468	22.96827	143.1917	23.07483	138.6111	23.00288	139.2284	23.13487	141.8503
23.0813	117.9067	23.00205	134.1402	22.98493	143.1877	23.0915	138.6098	23.01955	139.2283	23.15153	141.8491
23.09797	117.9015	23.01872	134.1389	23.0016	143.1857	23.10817	138.6083	23.03622	139.2263	23.1682	141.8458
23.11463	117.8975	23.03538	134.1353	23.01827	143.1821	23.12483	138.6055	23.05288	139.2263	23.18487	141.8435
23.1313	117.8929	23.05205	134.1286	23.03493	143.1823	23.1415	138.605	23.06955	139.225	23.20153	141.8437
23.14797	117.8888	23.06872	134.1283	23.0516	143.1799	23.15817	138.605	23.08622	139.2249	23.2182	141.8421
23.16463	117.8806	23.08538	134.1254	23.06827	143.1768	23.17483	138.6021	23.10288	139.2251	23.23487	141.8441
23.1813	117.8776	23.10205	134.121	23.08493	143.1751	23.1915	138.5992	23.11955	139.2228	23.25153	141.8444
23.19797	117.8725	23.11872	134.1163	23.1016	143.1728	23.20817	138.5992	23.13622	139.2241	23.2682	141.8361
23.21463	117.8649	23.13538	134.1135	23.11827	143.1746	23.22483	138.5966	23.15288	139.2218	23.28487	141.8377
23.2313	117.8594	23.15205	134.1102	23.13493	143.1697	23.2415	138.5976	23.16955	139.2197	23.30153	141.8373
23.24797	117.8564	23.16872	134.1037	23.1516	143.1684	23.25817	138.5954	23.18622	139.2187	23.3182	141.8375
23.26463	117.8527	23.18538	134.1053	23.16827	143.1639	23.27483	138.5939	23.20288	139.2167	23.33487	141.8343
23.2813	117.85	23.20205	134.104	23.18493	143.1622	23.2915	138.5936	23.21955	139.2153	23.35153	141.8337
23.29797	117.841	23.21872	134.0965	23.2016	143.1605	23.30817	138.5887	23.23622	139.214	23.3682	141.8345
23.31463	117.8404	23.23538	134.0954	23.21827	143.1576	23.32483	138.5953	23.25288	139.2135	23.38487	141.8325
23.3313	117.8335	23.25205	134.093	23.23493	143.1569	23.3415	138.5913	23.26955	139.2098	23.40153	141.8307
23.34797	117.8271	23.26872	134.0891	23.2516	143.1531	23.35817	138.59	23.28622	139.2096	23.4182	141.83
23.36463	117.8221	23.28538	134.0855	23.26827	143.1503	23.37483	138.5891	23.30288	139.2065	23.43487	141.8289
23.3813	117.8177	23.30205	134.0843	23.28493	143.1521	23.3915	138.5898	23.31955	139.206	23.45153	141.8289
23.39797	117.8134	23.31872	134.0814	23.3016	143.1463	23.40817	138.584	23.33622	139.2035	23.4682	141.8271
23.41463	117.8102	23.33538	134.0775	23.31827	143.1453	23.42483	138.5848	23.35288	139.2061	23.48487	141.8252
23.4313	117.802	23.35205	134.0762	23.33493	143.1408	23.4415	138.5832	23.36955	139.2009	23.50153	141.8257
23.44797	117.7966	23.36872	134.0735	23.3516	143.1403	23.45817	138.5822	23.38622	139.2005	23.5182	141.8241
23.46463	117.7928	23.38538	134.0688	23.36827	143.139	23.47483	138.5809	23.40288	139.2028	23.53487	141.8236
23.4813	117.7886	23.40205	134.066	23.38493	143.1347	23.4915	138.5771	23.41955	139.1995	23.55153	141.8208
23.49797	117.7817	23.41872	134.0657	23.4016	143.1325	23.50817	138.5755	23.43622	139.1978	23.5682	141.8199
23.51463	117.7776	23.43538	134.0585	23.41827	143.1301	23.52483	138.5813	23.45288	139.1983	23.58487	141.8203
23.5313	117.7717	23.45205	134.056	23.43493	143.1288	23.5415	138.5771	23.46955	139.1976	23.60153	141.8156
23.54797	117.7663	23.46872	134.0568	23.4516	143.1256	23.55817	138.5727	23.48622	139.197	23.6182	141.817
23.56463	117.7625	23.48538	134.0491	23.46827	143.1235	23.57483	138.5697	23.50288	139.1952	23.63487	141.8149
23.5813	117.7557	23.50205	134.0477	23.48493	143.1214	23.5915	138.5746	23.51955	139.1948	23.65153	141.8181
23.59797	117.7517	23.51872	134.048	23.5016	143.1173	23.60817	138.5737	23.53622	139.1924	23.6682	141.8136
23.61463	117.7461	23.53538	134.0403	23.51827	143.1176	23.62483	138.5727	23.55288	139.1933	23.68487	141.8116
23.6313	117.7427	23.55205	134.0404	23.53493	143.1165	23.6415	138.5686	23.56955	139.1912	23.70153	141.8098
23.64797	117.7371	23.56872	134.0403	23.5516	143.116	23.65817	138.5647	23.58622	139.1883	23.7182	141.8117
23.66463	117.7308	23.58538	134.036	23.56827	143.107	23.67483	138.5675	23.60288	139.1892	23.73487	141.8114
23.6813	117.7255	23.60205	134.0346	23.58493	143.1096	23.6915	138.5608	23.61955	139.1891	23.75153	141.8083
23.69797	117.7211	23.61872	134.0285	23.6016	143.107	23.70817	138.5615	23.63622	139.1872	23.7682	141.8074
23.71463	117.7163	23.63538	134.0292	23.61827	143.1033	23.72483	138.5614	23.65288	139.1847	23.78487	141.8078
23.7313	117.7118	23.65205	134.0232	23.63493	143.1019	23.7415	138.5616	23.66955	139.1836	23.80153	141.8038
23.74797	117.705	23.66872	134.0235	23.6516	143.1002	23.75817	138.5576	23.68622	139.1857	23.8182	141.8046
23.76463	117.7015	23.68538	134.0141	23.66827	143.098	23.77483	138.558	23.70288	139.1797	23.83487	141.8036
23.7813	117.6989	23.70205	134.0181	23.68493	143.0942	23.7915	138.5575	23.71955	139.1825	23.85153	141.803
23.79797	117.6912	23.71872	134.0104	23.7016	143.0922	23.80817	138.5571	23.73622	139.1836	23.8682	141.8018
23.81463	117.689	23.73538	134.0144	23.71827	143.0885	23.82483	138.557	23.75288	139.1805	23.88487	141.8022
23.8313	117.6828	23.75205	134.0079	23.73493	143.0906	23.8415	138.5525	23.76955	139.1784	23.90153	141.8018
23.84797	117.6779	23.76872	134.0081	23.7516	143.0848	23.85817	138.5524	23.78622	139.1777	23.9182	141.7989
23.86463	117.6723	23.78538	134.0059	23.76827	143.0854	23.87483	138.5532	23.80288	139.1768	23.93487	141.7987
23.8813	117.6697	23.80205	134.0031	23.78493	143.0856	23.8915	138.5498	23.81955	139.1796	23.95153	141.7962
23.89797	117.6649	23.81872	134.0001	23.8016	143.0809	23.90817	138.5494	23.83622	139.1666	23.9682	141.7968
23.91463	117.6599	23.83538	133.9966	23.81827	143.0801	23.92483	138.5508	23.85288	139.1787	23.98487	141.7949
23.9313	117.6578	23.85205	133.9926	23.83493	143.0776	23.9415	138.5462	23.86955	139.1645	24.00153	141.7937
23.94797	117.6558	23.86872	133.9923	23.8516	143.0742	23.95817	138.5443	23.88622	139.1768	24.0182	141.7928
23.96463	117.6479	23.88538	133.9897	23.86827	143.0748	23.97483	138.5457	23.90288	139.1732	24.03487	141.7917
23.9813	117.643	23.90205	133.9854	23.88493	143.0716	23.9915	138.5446	23.91955	139.1706	24.05153	141.79
23.99797	117.6366	23.91872	133.9834	23.9016	143.0643	24.00817	138.5423	23.93622	139.1711	24.0682	141.7876
24.01463	117.6307	23.93538	133.9824	23.91827	143.0661	24.02483	138.5433	23.95288	139.1644	24.08487	141.7889
24.0313	117.6287	23.95205	133.9777	23.93493	143.0637	24.0415	138.5369	23.96955	139.1669	24.10153	141.7871
24.04797	117.6221	23.96872	133.9797	23.9516	143.0609	24.05817	138.5389	23.98622	139.1679	24.1182	141.7844
24.06463	117.6165	23.98538	133.9695	23.96827	143.0588	24.07483	138.5398	24.00288	139.1662	24.13487	141.7828
24.0813	117.6152	24.00205	133.9715	23.98493	143.0554	24.0915	138.5392	24.01955	139.1645	24.15153	141.7839
24.09797	117.6075	24.01872	133.9647	24.0016	143.0572	24.10817	138.5399	24.03622	139.1626	24.1682	141.7828
24.11463	117.6016	24.03538	133.9648	24.01827	143.0511	24.12483	138.5377	24.05288	139.1622	24.18487	141.7815
24.1313	117.5994	24.05205	133.9607	24.03493	143.0507	24.1415	138.5352	24.06955	139.1622	24.20153	141.7793
24.14797	117.5961	24.06872	133.9593	24.0516	143.0484	24.15817	138.532	24.08622	139.163	24.2182	141.7811
24.16463	117.5895	24.08538	133.9539	24.06827	143.0472	24.17483	138.532	24.10288	139.1609	24.23487	141.7778
24.1813	117.5851	24.10205	133.956	24.08493	143.0428	24.1915	138.5326	24.11955	139.1613	24.25153	141.7784
24.19797	117.58	24.11872	133.9529	24.1016	143.0384	24.20817	138.5317	24.13622	139.1607	24.2682	141.7769
24.21463	117.5761	24.13538	133.9483	24.11827	143.0367	24.22483	138.5305	24.15288	139.1573	24.28487	141.7788
24.2313	117.5713	24.15205	133.943	24.13493	143.0364	24.2415	138.5285	24.16955	139.154	24.30153	141.7762
24.24797	117.5653	24.16872	133.9423	24.1516	143.0344	24.25817	138.5267	24.18622	139.1541	24.3182	141.7754
24.26463	117.5626	24.18538	133.94	24.16827	143.0312	24.27483	138.527	24.20288	139.1525	24.33487	141.7733
24.2813	117.5576	24.20205	133.9355	24.18493	143.029	24.2915	138.5264	24.21955	139.1552	24.35153	141.7723
24.29797	117.5512	24.21872	133.9355	24.2016	143.0259	24.30817	138.5256	24.23622	139.1518	24.3682	141.7724

24.31463	117.5474	24.23538	133.932	24.21827	143.0222	24.32483	138.5238	24.25288	139.1509	24.38487	141.7691
24.3313	117.5416	24.25205	133.9304	24.23493	143.0236	24.3415	138.5234	24.26955	139.1502	24.40153	141.7725
24.34797	117.5355	24.26872	133.9276	24.2516	143.0205	24.35817	138.5238	24.28622	139.1463	24.4182	141.7684
24.36463	117.5329	24.28538	133.9248	24.26827	143.0184	24.37483	138.5202	24.30288	139.145	24.43487	141.7671
24.3813	117.5263	24.30205	133.9233	24.28493	143.0171	24.3915	138.5223	24.31955	139.1472	24.45153	141.7685
24.39797	117.5222	24.31872	133.9216	24.3016	143.0138	24.40817	138.5194	24.33622	139.1423	24.4682	141.7653
24.41463	117.5183	24.33538	133.9171	24.31827	143.0112	24.42483	138.5164	24.35288	139.1423	24.48487	141.7665
24.4313	117.5126	24.35205	133.9143	24.33493	143.0086	24.4415	138.5175	24.36955	139.1404	24.50153	141.7657
24.44797	117.5087	24.36872	133.9118	24.3516	143.0069	24.45817	138.5182	24.38622	139.1372	24.5182	141.7675
24.46463	117.5026	24.38538	133.9108	24.36827	143.0031	24.47483	138.5147	24.40288	139.1341	24.53487	141.7628
24.4813	117.5002	24.40205	133.9085	24.38493	142.9993	24.4915	138.5142	24.41955	139.1351	24.55153	141.7644
24.49797	117.4942	24.41872	133.906	24.4016	143.0004	24.50817	138.5145	24.43622	139.1333	24.5682	141.7647
24.51463	117.4898	24.43538	133.9033	24.41827	142.9956	24.52483	138.5145	24.45288	139.1307	24.58487	141.7612
24.5313	117.4846	24.45205	133.9001	24.43493	142.9969	24.5415	138.5132	24.46955	139.1299	24.60153	141.7616
24.54797	117.4799	24.46872	133.9001	24.4516	142.9922	24.55817	138.5116	24.48622	139.129	24.6182	141.7618
24.56463	117.4768	24.48538	133.8936	24.46827	142.9878	24.57483	138.507	24.50288	139.127	24.63487	141.7594
24.5813	117.4725	24.50205	133.8911	24.48493	142.9866	24.5915	138.5082	24.51955	139.1273	24.65153	141.7594
24.59797	117.4663	24.51872	133.89	24.5016	142.9868	24.60817	138.508	24.53622	139.125	24.6682	141.7552
24.61463	117.4628	24.53538	133.8859	24.51827	142.9815	24.62483	138.5072	24.55288	139.1244	24.68487	141.7574
24.6313	117.4567	24.55205	133.884	24.53493	142.9791	24.6415	138.5079	24.56955	139.1199	24.70153	141.7534
24.64797	117.4519	24.56872	133.881	24.5516	142.9796	24.65817	138.5068	24.58622	139.1207	24.7182	141.7546
24.66463	117.4444	24.58538	133.8786	24.56827	142.9773	24.67483	138.5061	24.60288	139.1193	24.73487	141.7516
24.6813	117.4436	24.60205	133.8798	24.58493	142.9747	24.6915	138.5042	24.61955	139.1176	24.75153	141.7539
24.69797	117.4357	24.61872	133.8751	24.6016	142.9716	24.70817	138.5032	24.63622	139.1175	24.7682	141.7513
24.71463	117.4306	24.63538	133.8738	24.61827	142.9695	24.72483	138.503	24.65288	139.1155	24.78487	141.7507
24.7313	117.4299	24.65205	133.8727	24.63493	142.9695	24.7415	138.4999	24.66955	139.1145	24.80153	141.7473
24.74797	117.4221	24.66872	133.8714	24.6516	142.9687	24.75817	138.4999	24.68622	139.1099	24.8182	141.7492
24.76463	117.4173	24.68538	133.8689	24.66827	142.9629	24.77483	138.5002	24.70288	139.1083	24.83487	141.7471
24.7813	117.412	24.70205	133.8666	24.68493	142.9577	24.7915	138.4959	24.71955	139.109	24.85153	141.7467
24.79797	117.4073	24.71872	133.8646	24.7016	142.9642	24.80817	138.4964	24.73622	139.1076	24.8682	141.744
24.81463	117.3996	24.73538	133.8621	24.71827	142.9589	24.82483	138.4944	24.75288	139.1059	24.88487	141.7425
24.8313	117.4002	24.75205	133.8594	24.73493	142.9542	24.8415	138.4954	24.76955	139.1028	24.90153	141.741
24.84797	117.3942	24.76872	133.8602	24.7516	142.9558	24.85817	138.4913	24.78622	139.1038	24.9182	141.7419
24.86463	117.3932	24.78538	133.8591	24.76827	142.9532	24.87483	138.4934	24.80288	139.0995	24.93487	141.7374
24.8813	117.3861	24.80205	133.8519	24.78493	142.949	24.8915	138.4877	24.81955	139.0992	24.95153	141.7349
24.89797	117.3793	24.81872	133.8518	24.8016	142.9499	24.90817	138.4889	24.83622	139.0981	24.9682	141.7329
24.91463	117.3745	24.83538	133.8474	24.81827	142.948	24.92483	138.4875	24.85288	139.0998	24.98487	141.7318
24.9313	117.3703	24.85205	133.8453	24.83493	142.9468	24.9415	138.4881	24.86955	139.0991	25.00153	141.7306
24.94797	117.3682	24.86872	133.8449	24.8516	142.9431	24.95817	138.4828	24.88622	139.0971	25.0182	141.73
24.96463	117.3621	24.88538	133.8428	24.86827	142.9425	24.97483	138.4849	24.90288	139.0946	25.03487	141.7306
24.9813	117.3572	24.90205	133.8392	24.88493	142.9378	24.9915	138.481	24.91955	139.0939	25.05153	141.7269
24.99797	117.3533	24.91872	133.8368	24.9016	142.9393	25.00817	138.4821	24.93622	139.0943	25.0682	141.7226
25.01463	117.3478	24.93538	133.8361	24.91827	142.9339	25.02483	138.4805	24.95288	139.0921	25.08487	141.7219
25.0313	117.3425	24.95205	133.835	24.93493	142.9337	25.0415	138.4834	24.96955	139.0901	25.10153	141.7228
25.04797	117.3389	24.96872	133.8299	24.9516	142.9307	25.05817	138.4791	24.98622	139.0907	25.1182	141.7203
25.06463	117.3377	24.98538	133.8278	24.96827	142.9287	25.07483	138.4761	25.00288	139.0891	25.13487	141.7182
25.0813	117.3302	25.00205	133.8269	24.98493	142.9258	25.0915	138.4785	25.01955	139.0871	25.15153	141.7193
25.09797	117.3255	25.01872	133.8245	25.0016	142.924	25.10817	138.4755	25.03622	139.0854	25.1682	141.7174
25.11463	117.3242	25.03538	133.8214	25.01827	142.9235	25.12483	138.4744	25.05288	139.0847	25.18487	141.7153
25.1313	117.3157	25.05205	133.8207	25.03493	142.9199	25.1415	138.4725	25.06955	139.0799	25.20153	141.7132
25.14797	117.3115	25.06872	133.8186	25.0516	142.9192	25.15817	138.4718	25.08622	139.0806	25.2182	141.7129
25.16463	117.3105	25.08538	133.816	25.06827	142.9139	25.17483	138.4706	25.10288	139.0813	25.23487	141.7133
25.1813	117.3009	25.10205	133.8133	25.08493	142.9107	25.1915	138.4695	25.11955	139.0797	25.25153	141.7123
25.19797	117.2993	25.11872	133.8108	25.1016	142.9123	25.20817	138.4673	25.13622	139.0776	25.2682	141.7077
25.21463	117.297	25.13538	133.8109	25.11827	142.9103	25.22483	138.4669	25.15288	139.0759	25.28487	141.7087
25.2313	117.2934	25.15205	133.8047	25.13493	142.9087	25.2415	138.466	25.16955	139.0754	25.30153	141.7065
25.24797	117.2886	25.16872	133.8067	25.1516	142.9045	25.25817	138.4636	25.18622	139.0731	25.3182	141.7078
25.26463	117.2839	25.18538	133.8058	25.16827	142.9021	25.27483	138.4649	25.20288	139.0727	25.33487	141.7043
25.2813	117.2808	25.20205	133.8032	25.18493	142.9007	25.2915	138.459	25.21955	139.0716	25.35153	141.705
25.29797	117.2746	25.21872	133.7978	25.2016	142.9007	25.30817	138.4616	25.23622	139.0719	25.3682	141.7017
25.31463	117.2719	25.23538	133.7973	25.21827	142.8998	25.32483	138.4598	25.25288	139.0706	25.38487	141.7018
25.3313	117.2675	25.25205	133.7959	25.23493	142.8979	25.3415	138.4606	25.26955	139.0687	25.40153	141.701
25.34797	117.2628	25.26872	133.7914	25.2516	142.8928	25.35817	138.4549	25.28622	139.0689	25.4182	141.7027
25.36463	117.2574	25.28538	133.7918	25.26827	142.8947	25.37483	138.4564	25.30288	139.0651	25.43487	141.6981
25.3813	117.2547	25.30205	133.7884	25.28493	142.8919	25.3915	138.4574	25.31955	139.0646	25.45153	141.7006
25.39797	117.2502	25.31872	133.7839	25.3016	142.8864	25.40817	138.4551	25.33622	139.0625	25.4682	141.6962
25.41463	117.2458	25.33538	133.7824	25.31827	142.8855	25.42483	138.4512	25.35288	139.0637	25.48487	141.6977
25.4313	117.2429	25.35205	133.7829	25.33493	142.8831	25.4415	138.4514	25.36955	139.0599	25.50153	141.6948
25.44797	117.2383	25.36872	133.7788	25.3516	142.8798	25.45817	138.4497	25.38622	139.0585	25.5182	141.6907
25.46463	117.2319	25.38538	133.7751	25.36827	142.8777	25.47483	138.4518	25.40288	139.0583	25.53487	141.6933
25.4813	117.2278	25.40205	133.7755	25.38493	142.874	25.4915	138.4457	25.41955	139.0571	25.55153	141.6914
25.49797	117.2258	25.41872	133.7736	25.4016	142.875	25.50817	138.4501	25.43622	139.0563	25.5682	141.6885
25.51463	117.2191	25.43538	133.7732	25.41827	142.8734	25.52483	138.4442	25.45288	139.0551	25.58487	141.6895
25.5313	117.2164	25.45205	133.767	25.43493	142.8689	25.5415	138.4459	25.46955	139.0526	25.60153	141.6869
25.54797	117.2105	25.46872	133.7666	25.4516	142.8651	25.55817	138.4456	25.48622	139.0551	25.6182	141.686
25.56463	117.208	25.48538	133.7666	25.46827	142.8638	25.57483	138.4438	25.50288	139.0503	25.63487	141.6851
25.5813	117.2011	25.50205	133.7608	25.48493	142.8623	25.5915	138.4383	25.51955	139.0503	25.65153	141.6838
25.59797	117.1985	25.51872	133.7605	25.5016	142.8621	25.60817	138.4386	25.53622	139.0499	25.6682	141.6823
25.61463	117.1919	25.53538	133.7617	25.51827	142.8581	25.62483	138.4393	25.55288	139.0494	25.68487	141.6799
25.6313	117.1869	25.55205	133.7549	25.53493	142.858	25.6415	138.4377	25.56955	139.0468	25.70153	141.6793
25.64797	117.1826	25.56872	133.7532	25.5516	142.8537	25.65817	138.4352	25.58622	139.0478	25.7182	141.6774
25.66463	117.1812	25.58538	133.7526	25.56827	142.8542	25.67483	138.4337	25.60288	139.0444	25.73487	141.6762
25.6813	117.1733	25.60205	133.752	25.58493	142.8496	25.6915	138.4311	25.61955	139.045	25.75153	141.6752
25.69797	117.1696	25.61872	133.7487	25.6016	142.85	25.70817	138.4318	25.63622	139.0427	25.7682	141.6749
25.71463	117.1663	25.63538	133.7478	25.61827	142.8461	25.72483	138.4296	25.65288	139.0401	25.78487	141.6734
25.7313	117.159	25.65205	133.7458	25.63493	142.8423	25.7415	138.4298	25.66955	139.0427	25.80153	141.6716
25.74797	117.1564	25.66872	133.7422	25.6516	142.8404	25.75817	138.4284	25.68622	139.0396	25.8182	141.6679
25.76463	117.1533	25.68538	133.7392	25.66827	142.841	25.77483	138.427	25.70288	139.0399	25.83487	141.6702
25.7813	117.1489	25.70205	133.7381	25.68493	142.837	25.7915	138.426	25.71955	139.037	25.85153	141.6688
25.79797	117.1436	25.71872	133.7385	25.7016	142.8365	25.80817	138.4235	25.73622	139.0355	25.8682	141.6662
25.81463	117.1414	25.73538	133.7346	25.71827	142.8342	25.82483	138.4259	25.75288	139.0346	25.88487	141.6635
25.8313	117.1383	25.75205	133.731	25.73493	142.8313	25.8415	138.421	25.76955	139.0364	25.90153	141.6642
25.84797	117.1318	25.76872	133.7312	25.7516	142.8296	25.85817	138.4243	25.78622	139.0318	25.9182	141.6629
25.86463	117.1298	25.78538	133.7276	25.76827	142.8328	25.87483	138.4219	25.80288	139.0317	25.93487	141.6612
25.8813	117.1247	25.80205	133.726	25.78493	142.8228	25.8915	138.4176	25.81955	139.0328	25.95153	141.6601
25.89797	117.1193	25.81872	133.7237	25.8016	142.8261	25.90817	138.42	25.83622	139.0293	25.9682	141.6578
25.91463	117.1144	25.83538	133.7232	25.81827	142.8206	25.92483	138.4181	25.85288	139.0328	25.98487	141.6592

25.9313	117.1104	25.85205	133.7152	25.83493	142.8216	25.9415	138.4172	25.86955	139.03	26.00153	141.6554
25.94797	117.1049	25.86872	133.7185	25.8516	142.8164	25.95817	138.416	25.88622	139.0272	26.0182	141.6536
25.96463	117.1072	25.88538	133.7183	25.86827	142.8165	25.97483	138.4172	25.90288	139.0275	26.03487	141.654
25.9813	117.0975	25.90205	133.7153	25.88493	142.8135	25.9915	138.415	25.91955	139.027	26.05153	141.6529
25.99797	117.0927	25.91872	133.7117	25.9016	142.8119	26.00817	138.414	25.93622	139.0277	26.0682	141.6567
26.01463	117.0897	25.93538	133.7115	25.91827	142.8114	26.02483	138.4103	25.95288	139.0244	26.08487	141.6472
26.0313	117.0891	25.95205	133.7057	25.93493	142.8076	26.0415	138.4079	25.96955	139.0231	26.10153	141.6479
26.04797	117.0823	25.96872	133.7083	25.9516	142.8041	26.05817	138.4089	25.98622	139.0231	26.1182	141.6436
26.06463	117.079	25.98538	133.7048	25.96827	142.8032	26.07483	138.4095	26.00288	139.0187	26.13487	141.6458
26.0813	117.0729	26.00205	133.705	25.98493	142.8029	26.0915	138.4052	26.01955	139.0198	26.15153	141.6424
26.09797	117.0706	26.01872	133.7003	26.0016	142.8011	26.10817	138.4074	26.03622	139.0213	26.1682	141.6455
26.11463	117.0656	26.03538	133.6988	26.01827	142.7969	26.12483	138.4042	26.05288	139.0185	26.18487	141.6447
26.1313	117.0623	26.05205	133.6959	26.03493	142.796	26.1415	138.406	26.06955	139.0185	26.20153	141.6402
26.14797	117.0575	26.06872	133.6976	26.0516	142.7924	26.15817	138.402	26.08622	139.0169	26.2182	141.64
26.16463	117.0551	26.08538	133.693	26.06827	142.7932	26.17483	138.4026	26.10288	139.0143	26.23487	141.6386
26.1813	117.0499	26.10205	133.6913	26.08493	142.7868	26.1915	138.4	26.11955	139.0178	26.25153	141.643
26.19797	117.0435	26.11872	133.6905	26.1016	142.7897	26.20817	138.3964	26.13622	139.0156	26.2682	141.6387
26.21463	117.0423	26.13538	133.6878	26.11827	142.7867	26.22483	138.3955	26.15288	139.012	26.28487	141.6391
26.2313	117.0357	26.15205	133.6854	26.13493	142.7862	26.2415	138.396	26.16955	139.0094	26.30153	141.6359
26.24797	117.0337	26.16872	133.6848	26.1516	142.7792	26.25817	138.3918	26.18622	139.0085	26.3182	141.6343
26.26463	117.0292	26.18538	133.683	26.16827	142.7794	26.27483	138.3921	26.20288	139.0084	26.33487	141.6351
26.2813	117.0213	26.20205	133.68	26.18493	142.774	26.2915	138.3909	26.21955	139.0059	26.35153	141.6345
26.29797	117.0164	26.21872	133.676	26.2016	142.7779	26.30817	138.3892	26.23622	139.0075	26.3682	141.6305
26.31463	117.0141	26.23538	133.6782	26.21827	142.7738	26.32483	138.3899	26.25288	139.0048	26.38487	141.6315
26.3313	117.0089	26.25205	133.6773	26.23493	142.7749	26.3415	138.3887	26.26955	139.0025	26.40153	141.629
26.34797	117.0039	26.26872	133.6736	26.2516	142.7682	26.35817	138.3853	26.28622	139.0019	26.4182	141.6285
26.36463	117.0016	26.28538	133.6712	26.26827	142.7668	26.37483	138.3844	26.30288	139.0006	26.43487	141.6267
26.3813	116.995	26.30205	133.6701	26.28493	142.7656	26.3915	138.3849	26.31955	139.0039	26.45153	141.6295
26.39797	116.996	26.31872	133.6691	26.3016	142.764	26.40817	138.3841	26.33622	139.0001	26.4682	141.6239
26.41463	116.9865	26.33538	133.6664	26.31827	142.7621	26.42483	138.3835	26.35288	138.9971	26.48487	141.6245
26.4313	116.9839	26.35205	133.6642	26.33493	142.7583	26.4415	138.3824	26.36955	138.9964	26.50153	141.6232
26.44797	116.9811	26.36872	133.6625	26.3516	142.7555	26.45817	138.3824	26.38622	138.9947	26.5182	141.6224
26.46463	116.976	26.38538	133.6626	26.36827	142.7551	26.47483	138.3816	26.40288	138.9927	26.53487	141.6206
26.4813	116.9724	26.40205	133.6614	26.38493	142.748	26.4915	138.3807	26.41955	138.9943	26.55153	141.6202
26.49797	116.9652	26.41872	133.6573	26.4016	142.7479	26.50817	138.3779	26.43622	138.9917	26.5682	141.6204
26.51463	116.9625	26.43538	133.6551	26.41827	142.7467	26.52483	138.3768	26.45288	138.992	26.58487	141.6172
26.5313	116.9595	26.45205	133.6535	26.43493	142.7465	26.5415	138.3796	26.46955	138.9919	26.60153	141.6179
26.54797	116.9543	26.46872	133.6498	26.4516	142.7389	26.55817	138.3749	26.48622	138.9897	26.6182	141.6162
26.56463	116.9504	26.48538	133.6476	26.46827	142.7409	26.57483	138.3748	26.50288	138.9868	26.63487	141.6164
26.5813	116.9447	26.50205	133.6455	26.48493	142.7362	26.5915	138.3735	26.51955	138.9861	26.65153	141.6139
26.59797	116.9438	26.51872	133.6465	26.5016	142.7352	26.60817	138.3753	26.53622	138.9858	26.6682	141.6118
26.61463	116.9367	26.53538	133.644	26.51827	142.7311	26.62483	138.3706	26.55288	138.9825	26.68487	141.6125
26.6313	116.9344	26.55205	133.6384	26.53493	142.733	26.6415	138.3707	26.56955	138.9827	26.70153	141.6114
26.64797	116.9287	26.56872	133.6383	26.5516	142.7287	26.65817	138.3693	26.58622	138.9806	26.7182	141.6105
26.66463	116.9262	26.58538	133.6385	26.56827	142.7253	26.67483	138.3698	26.60288	138.9787	26.73487	141.6076
26.6813	116.9208	26.60205	133.6336	26.58493	142.722	26.6915	138.3679	26.61955	138.9806	26.75153	141.6078
26.69797	116.9168	26.61872	133.6326	26.6016	142.7222	26.70817	138.367	26.63622	138.9771	26.7682	141.6049
26.71463	116.9107	26.63538	133.63	26.61827	142.7158	26.72483	138.3654	26.65288	138.9763	26.78487	141.604
26.7313	116.9082	26.65205	133.6291	26.63493	142.7153	26.7415	138.3656	26.66955	138.9757	26.80153	141.6015
26.74797	116.9033	26.66872	133.6279	26.6516	142.7107	26.75817	138.3639	26.68622	138.9731	26.8182	141.6032
26.76463	116.8989	26.68538	133.6248	26.66827	142.7137	26.77483	138.3637	26.70288	138.9711	26.83487	141.5993
26.7813	116.8955	26.70205	133.6173	26.68493	142.7126	26.7915	138.3625	26.71955	138.9711	26.85153	141.5979
26.79797	116.8893	26.71872	133.621	26.7016	142.7084	26.80817	138.3624	26.73622	138.9729	26.8682	141.5964
26.81463	116.8877	26.73538	133.6194	26.71827	142.7044	26.82483	138.3591	26.75288	138.9717	26.88487	141.5978
26.8313	116.8838	26.75205	133.6171	26.73493	142.7036	26.8415	138.3602	26.76955	138.9694	26.90153	141.5949
26.84797	116.8781	26.76872	133.613	26.7516	142.7019	26.85817	138.3571	26.78622	138.9682	26.9182	141.5955
26.86463	116.8743	26.78538	133.6097	26.76827	142.6996	26.87483	138.3588	26.80288	138.9685	26.93487	141.5912
26.8813	116.8692	26.80205	133.6114	26.78493	142.7028	26.8915	138.359	26.81955	138.9678	26.95153	141.591
26.89797	116.8635	26.81872	133.6094	26.8016	142.7008	26.90817	138.3574	26.83622	138.9635	26.9682	141.5905
26.91463	116.8605	26.83538	133.6076	26.81827	142.6947	26.92483	138.3567	26.85288	138.9636	26.98487	141.5887
26.9313	116.8568	26.85205	133.6035	26.83493	142.6948	26.9415	138.3561	26.86955	138.9637	27.00153	141.5857
26.94797	116.8511	26.86872	133.6028	26.8516	142.6915	26.95817	138.3546	26.88622	138.963	27.0182	141.5872
26.96463	116.8477	26.88538	133.6032	26.86827	142.6876	26.97483	138.3542	26.90288	138.9613	27.03487	141.5841
26.9813	116.8428	26.90205	133.5984	26.88493	142.6864	26.9915	138.3489	26.91955	138.9607	27.05153	141.5812
26.99797	116.8386	26.91872	133.6017	26.9016	142.6844	27.00817	138.3488	26.93622	138.9589	27.0682	141.58
27.01463	116.8353	26.93538	133.5997	26.91827	142.6816	27.02483	138.3465	26.95288	138.9569	27.08487	141.5821
27.0313	116.8306	26.95205	133.5924	26.93493	142.6796	27.0415	138.3458	26.96955	138.955	27.10153	141.5778
27.04797	116.8272	26.96872	133.5948	26.9516	142.6759	27.05817	138.3457	26.98622	138.9535	27.1182	141.5766
27.06463	116.8192	26.98538	133.5944	26.96827	142.6746	27.07483	138.3486	27.00288	138.9558	27.13487	141.5741
27.0813	116.8172	27.00205	133.5889	26.98493	142.6728	27.0915	138.3457	27.01955	138.9531	27.15153	141.5762
27.09797	116.8135	27.01872	133.5894	27.0016	142.6727	27.10817	138.3467	27.03622	138.9525	27.1682	141.5734
27.11463	116.8085	27.03538	133.5888	27.01827	142.6689	27.12483	138.3446	27.05288	138.9475	27.18487	141.5733
27.1313	116.8037	27.05205	133.5859	27.03493	142.6715	27.1415	138.3458	27.06955	138.9468	27.20153	141.5679
27.14797	116.8017	27.06872	133.5825	27.0516	142.667	27.15817	138.3469	27.08622	138.9456	27.2182	141.569
27.16463	116.7967	27.08538	133.5828	27.06827	142.6659	27.17483	138.3467	27.10288	138.9479	27.23487	141.569
27.1813	116.7897	27.10205	133.5798	27.08493	142.6622	27.1915	138.3446	27.11955	138.9461	27.25153	141.566
27.19797	116.7875	27.11872	133.5811	27.1016	142.6612	27.20817	138.3455	27.13622	138.9439	27.2682	141.5647
27.21463	116.7812	27.13538	133.5803	27.11827	142.6571	27.22483	138.3448	27.15288	138.9449	27.28487	141.5648
27.2313	116.7804	27.15205	133.577	27.13493	142.6562	27.2415	138.3445	27.16955	138.9427	27.30153	141.5646
27.24797	116.7732	27.16872	133.5754	27.1516	142.6513	27.25817	138.3433	27.18622	138.94	27.3182	141.5611
27.26463	116.7713	27.18538	133.5716	27.16827	142.6523	27.27483	138.3461	27.20288	138.942	27.33487	141.5587
27.2813	116.7713	27.20205	133.5699	27.18493	142.6505	27.2915	138.3399	27.21955	138.9404	27.35153	141.5601
27.29797	116.7598	27.21872	133.5683	27.2016	142.6485	27.30817	138.3404	27.23622	138.9389	27.3682	141.5576
27.31463	116.754	27.23538	133.5671	27.21827	142.6463	27.32483	138.3402	27.25288	138.9365	27.38487	141.5576
27.3313	116.7573	27.25205	133.5641	27.23493	142.6463	27.3415	138.344	27.26955	138.9342	27.40153	141.5551
27.34797	116.7476	27.26872	133.5647	27.2516	142.6443	27.35817	138.3366	27.28622	138.9346	27.4182	141.5534
27.36463	116.7447	27.28538	133.56	27.26827	142.6407	27.37483	138.3376	27.30288	138.9342	27.43487	141.5532
27.3813	116.7413	27.30205	133.5599	27.28493	142.6383	27.3915	138.3377	27.31955	138.9333	27.45153	141.5531
27.39797	116.7394	27.31872	133.5583	27.3016	142.6377	27.40817	138.3366	27.33622	138.9319	27.4682	141.5504
27.41463	116.7305	27.33538	133.5546	27.31827	142.6349	27.42483	138.3358	27.35288	138.9285	27.48487	141.5504
27.4313	116.7264	27.35205	133.5542	27.33493	142.6347	27.4415	138.331	27.36955	138.9297	27.50153	141.5474
27.44797	116.7251	27.36872	133.5509	27.3516	142.6308	27.45817	138.3303	27.38622	138.9309	27.5182	141.5467
27.46463	116.7163	27.38538	133.5502	27.36827	142.6278	27.47483	138.3305	27.40288	138.9266	27.53487	141.5468
27.4813	116.7126	27.40205	133.5498	27.38493	142.624	27.4915	138.3303	27.41955	138.9251	27.55153	141.5451
27.49797	116.7117	27.41872	133.5473	27.4016	142.6269	27.50817	138.3244	27.43622	138.9225	27.5682	141.5413
27.51463	116.7081	27.43538	133.5445	27.41827	142.6212	27.52483	138.3241	27.45288	138.9244	27.58487	141.5431
27.5313	116.7022	27.45205	133.5438	27.43493	142.6197	27.5415	138.3244	27.46955	138.9237	27.60153	141.5403

27.54797	116.6986	27.46872	133.5409	27.4516	142.6205	27.55817	138.3216	27.48622	138.9226	27.6182	141.5406
27.56463	116.6947	27.48538	133.5419	27.46827	142.6141	27.57483	138.3211	27.50288	138.9217	27.63487	141.5375
27.5813	116.6902	27.50205	133.5396	27.48493	142.614	27.5915	138.3174	27.51955	138.9186	27.65153	141.5373
27.59797	116.6857	27.51872	133.5333	27.5016	142.6125	27.60817	138.3143	27.53622	138.9195	27.6682	141.5337
27.61463	116.6815	27.53538	133.5401	27.51827	142.6107	27.62483	138.3172	27.55288	138.9165	27.68487	141.5343
27.6313	116.6783	27.55205	133.5325	27.53493	142.6096	27.6415	138.3179	27.56955	138.9168	27.70153	141.5342
27.64797	116.6746	27.56872	133.5328	27.5516	142.604	27.65817	138.3149	27.58622	138.9156	27.7182	141.5313
27.66463	116.6688	27.58538	133.5303	27.56827	142.6065	27.67483	138.3108	27.60288	138.9159	27.73487	141.5296
27.6813	116.6656	27.60205	133.5289	27.58493	142.6061	27.6915	138.3143	27.61955	138.9125	27.75153	141.5311
27.69797	116.664	27.61872	133.5278	27.6016	142.6035	27.70817	138.3092	27.63622	138.9094	27.7682	141.5301
27.71463	116.6557	27.63538	133.5243	27.61827	142.5986	27.72483	138.3066	27.65288	138.9101	27.78487	141.5287
27.7313	116.6546	27.65205	133.5215	27.63493	142.5981	27.7415	138.3051	27.66955	138.9083	27.80153	141.5241
27.74797	116.6495	27.66872	133.5191	27.6516	142.6004	27.75817	138.3061	27.68622	138.9058	27.8182	141.5264
27.76463	116.6438	27.68538	133.5157	27.66827	142.5968	27.77483	138.3066	27.70288	138.9044	27.83487	141.5216
27.7813	116.6444	27.70205	133.517	27.68493	142.5941	27.7915	138.3069	27.71955	138.9056	27.85153	141.5215
27.79797	116.6392	27.71872	133.5117	27.7016	142.5904	27.80817	138.3016	27.73622	138.9041	27.8682	141.5217
27.81463	116.6347	27.73538	133.5128	27.71827	142.588	27.82483	138.3026	27.75288	138.902	27.88487	141.5212
27.8313	116.6258	27.75205	133.5072	27.73493	142.5913	27.8415	138.3006	27.76955	138.9032	27.90153	141.517
27.84797	116.6302	27.76872	133.5038	27.7516	142.5892	27.85817	138.3015	27.78622	138.9011	27.9182	141.5174
27.86463	116.6232	27.78538	133.5007	27.76827	142.5852	27.87483	138.296	27.80288	138.8983	27.93487	141.5172
27.8813	116.6178	27.80205	133.5001	27.78493	142.5842	27.8915	138.2974	27.81955	138.8983	27.95153	141.5152
27.89797	116.6196	27.81872	133.498	27.8016	142.5857	27.90817	138.2984	27.83622	138.8971	27.9682	141.5144
27.91463	116.6117	27.83538	133.5023	27.81827	142.5793	27.92483	138.297	27.85288	138.894	27.98487	141.5102
27.9313	116.6077	27.85205	133.4959	27.83493	142.5778	27.9415	138.2958	27.86955	138.896	28.00153	141.5099
27.94797	116.6066	27.86872	133.4926	27.8516	142.5777	27.95817	138.2918	27.88622	138.8945	28.0182	141.5101
27.96463	116.6004	27.88538	133.4931	27.86827	142.5741	27.97483	138.2927	27.90288	138.8912	28.03487	141.5069
27.9813	116.5995	27.90205	133.4899	27.88493	142.5715	27.9915	138.2924	27.91955	138.8905	28.05153	141.5064
27.99797	116.5944	27.91872	133.4894	27.9016	142.5716	28.00817	138.2897	27.93622	138.8908	28.0682	141.5062
28.01463	116.5898	27.93538	133.4875	27.91827	142.5659	28.02483	138.2885	27.95288	138.8888	28.08487	141.5061
28.0313	116.5879	27.95205	133.4833	27.93493	142.5684	28.0415	138.2867	27.96955	138.8866	28.10153	141.5035
28.04797	116.5816	27.96872	133.4861	27.9516	142.5656	28.05817	138.2865	27.98622	138.886	28.1182	141.502
28.06463	116.5783	27.98538	133.4849	27.96827	142.5632	28.07483	138.286	28.00288	138.8841	28.13487	141.5019
28.0813	116.5763	28.00205	133.4838	27.98493	142.5596	28.0915	138.2825	28.01955	138.8825	28.15153	141.5006
28.09797	116.5698	28.01872	133.4774	28.0016	142.5574	28.10817	138.285	28.03622	138.8829	28.1682	141.4989
28.11463	116.5651	28.03538	133.4793	28.01827	142.558	28.12483	138.2802	28.05288	138.8827	28.18487	141.498
28.1313	116.5648	28.05205	133.4757	28.03493	142.5554	28.1415	138.2789	28.06955	138.8799	28.20153	141.4961
28.14797	116.5595	28.06872	133.4777	28.0516	142.5529	28.15817	138.2791	28.08622	138.8784	28.2182	141.4949
28.16463	116.5557	28.08538	133.4728	28.06827	142.5512	28.17483	138.279	28.10288	138.8782	28.23487	141.4927
28.1813	116.5528	28.10205	133.4707	28.08493	142.5501	28.1915	138.2783	28.11955	138.8755	28.25153	141.4911
28.19797	116.5501	28.11872	133.4625	28.1016	142.547	28.20817	138.2786	28.13622	138.8764	28.2682	141.4927
28.21463	116.5427	28.13538	133.4688	28.11827	142.5457	28.22483	138.2762	28.15288	138.875	28.28487	141.4904
28.2313	116.5417	28.15205	133.466	28.13493	142.5435	28.2415	138.2776	28.16955	138.8742	28.30153	141.4893
28.24797	116.5361	28.16872	133.4615	28.1516	142.5422	28.25817	138.2755	28.18622	138.8747	28.3182	141.4862
28.26463	116.5336	28.18538	133.4563	28.16827	142.5418	28.27483	138.2753	28.20288	138.8727	28.33487	141.4863
28.2813	116.5272	28.20205	133.4584	28.18493	142.5381	28.2915	138.2726	28.21955	138.8716	28.35153	141.4841
28.29797	116.5229	28.21872	133.4546	28.2016	142.5377	28.30817	138.2739	28.23622	138.8684	28.3682	141.4821
28.31463	116.5204	28.23538	133.4509	28.21827	142.5368	28.32483	138.2698	28.25288	138.8685	28.38487	141.4825
28.3313	116.5183	28.25205	133.4502	28.23493	142.534	28.3415	138.2727	28.26955	138.8689	28.40153	141.4812
28.34797	116.5115	28.26872	133.4486	28.2516	142.532	28.35817	138.2678	28.28622	138.868	28.4182	141.4795
28.36463	116.5061	28.28538	133.4453	28.26827	142.5325	28.37483	138.2663	28.30288	138.8676	28.43487	141.48
28.3813	116.5023	28.30205	133.4465	28.28493	142.5274	28.3915	138.2649	28.31955	138.8658	28.45153	141.4771
28.39797	116.5025	28.31872	133.4409	28.3016	142.5257	28.40817	138.2659	28.33622	138.8653	28.4682	141.4769
28.41463	116.4979	28.33538	133.4425	28.31827	142.5217	28.42483	138.2634	28.35288	138.8625	28.48487	141.4757
28.4313	116.4934	28.35205	133.44	28.33493	142.5211	28.4415	138.2641	28.36955	138.8612	28.50153	141.4745
28.44797	116.49	28.36872	133.4383	28.3516	142.52	28.45817	138.2613	28.38622	138.8599	28.5182	141.4732
28.46463	116.4874	28.38538	133.4364	28.36827	142.52	28.47483	138.2591	28.40288	138.8612	28.53487	141.4714
28.4813	116.4815	28.40205	133.4327	28.38493	142.5168	28.4915	138.259	28.41955	138.8594	28.55153	141.469
28.49797	116.4788	28.41872	133.4361	28.4016	142.5113	28.50817	138.2583	28.43622	138.8584	28.5682	141.4688
28.51463	116.4752	28.43538	133.4296	28.41827	142.5108	28.52483	138.2591	28.45288	138.856	28.58487	141.4686
28.5313	116.468	28.45205	133.427	28.43493	142.5128	28.5415	138.2551	28.46955	138.855	28.60153	141.4671
28.54797	116.4662	28.46872	133.4264	28.4516	142.5084	28.55817	138.2545	28.48622	138.8504	28.6182	141.4663
28.56463	116.4587	28.48538	133.4227	28.46827	142.5054	28.57483	138.2525	28.50288	138.8496	28.63487	141.464
28.5813	116.4574	28.50205	133.4208	28.48493	142.504	28.5915	138.2496	28.51955	138.8472	28.65153	141.4648
28.59797	116.4549	28.51872	133.4177	28.5016	142.5029	28.60817	138.2519	28.53622	138.8496	28.6682	141.4611
28.61463	116.4487	28.53538	133.4211	28.51827	142.505	28.62483	138.2513	28.55288	138.8505	28.68487	141.461
28.6313	116.4446	28.55205	133.4175	28.53493	142.4985	28.6415	138.2478	28.56955	138.8495	28.70153	141.4589
28.64797	116.4435	28.56872	133.4147	28.5516	142.4989	28.65817	138.2509	28.58622	138.8446	28.7182	141.4578
28.66463	116.4379	28.58538	133.4135	28.56827	142.494	28.67483	138.2454	28.60288	138.843	28.73487	141.4567
28.6813	116.4334	28.60205	133.4125	28.58493	142.4962	28.6915	138.2472	28.61955	138.8445	28.75153	141.4549
28.69797	116.429	28.61872	133.409	28.6016	142.4939	28.70817	138.2452	28.63622	138.8427	28.7682	141.4553
28.71463	116.427	28.63538	133.4087	28.61827	142.4917	28.72483	138.2437	28.65288	138.841	28.78487	141.4534
28.7313	116.4239	28.65205	133.4039	28.63493	142.4913	28.7415	138.2421	28.66955	138.8409	28.80153	141.4501
28.74797	116.4182	28.66872	133.4033	28.6516	142.4859	28.75817	138.2392	28.68622	138.8403	28.8182	141.4522
28.76463	116.4145	28.68538	133.4028	28.66827	142.4872	28.77483	138.2402	28.70288	138.8393	28.83487	141.4486
28.7813	116.4095	28.70205	133.4001	28.68493	142.4846	28.7915	138.2374	28.71955	138.8394	28.85153	141.4496
28.79797	116.4097	28.71872	133.3962	28.7016	142.4821	28.80817	138.2378	28.73622	138.8389	28.8682	141.4479
28.81463	116.4038	28.73538	133.4001	28.71827	142.4822	28.82483	138.2367	28.75288	138.8345	28.88487	141.4486
28.8313	116.3987	28.75205	133.3948	28.73493	142.4792	28.8415	138.2349	28.76955	138.8352	28.90153	141.4465
28.84797	116.3959	28.76872	133.3912	28.7516	142.4779	28.85817	138.229	28.78622	138.8344	28.9182	141.447
28.86463	116.3918	28.78538	133.3898	28.76827	142.4802	28.87483	138.2322	28.80288	138.8341	28.93487	141.4451
28.8813	116.3875	28.80205	133.3896	28.78493	142.4741	28.8915	138.2298	28.81955	138.8341	28.95153	141.4421
28.89797	116.3858	28.81872	133.3857	28.8016	142.4699	28.90817	138.2317	28.83622	138.8324	28.9682	141.4425
28.91463	116.3795	28.83538	133.3837	28.81827	142.4713	28.92483	138.2286	28.85288	138.8304	28.98487	141.4413
28.9313	116.376	28.85205	133.3771	28.83493	142.4696	28.9415	138.2271	28.86955	138.8302	29.00153	141.4405
28.94797	116.3731	28.86872	133.3785	28.8516	142.468	28.95817	138.2229	28.88622	138.8279	29.0182	141.4381
28.96463	116.371	28.88538	133.3775	28.86827	142.4645	28.97483	138.225	28.90288	138.8287	29.03487	141.4368
28.9813	116.3639	28.90205	133.3723	28.88493	142.4615	28.9915	138.2234	28.91955	138.8265	29.05153	141.4362
28.99797	116.3599	28.91872	133.3695	28.9016	142.4589	29.00817	138.2202	28.93622	138.8284	29.0682	141.4346
29.01463	116.3559	28.93538	133.3703	28.91827	142.4615	29.02483	138.2203	28.95288	138.8273	29.08487	141.4333
29.0313	116.3513	28.95205	133.3688	28.93493	142.4576	29.0415	138.2206	28.96955	138.8244	29.10153	141.432
29.04797	116.3469	28.96872	133.363	28.9516	142.4544	29.05817	138.22	28.98622	138.8237	29.1182	141.4291
29.06463	116.3454	28.98538	133.3655	28.96827	142.454	29.07483	138.2172	29.00288	138.8228	29.13487	141.4289
29.0813	116.3385	29.00205	133.3653	28.98493	142.4485	29.0915	138.2157	29.01955	138.8209	29.15153	141.4279
29.09797	116.335	29.01872	133.363	29.0016	142.4496	29.10817	138.2188	29.03622	138.8218	29.1682	141.4259
29.11463	116.3325	29.03538	133.3607	29.01827	142.4448	29.12483	138.2152	29.05288	138.8213	29.18487	141.4245
29.1313	116.3292	29.05205	133.3595	29.03493	142.4438	29.1415	138.212	29.06955	138.8193	29.20153	141.423
29.14797	116.3243	29.06872	133.3567	29.0516	142.4471	29.15817	138.2141	29.08622	138.8173	29.2182	141.4235

29.16463	116.3204	29.08538	133.3564	29.06827	142.4448	29.17483	138.2132	29.10288	138.8162	29.23487	141.4227
29.1813	116.3142	29.10205	133.3504	29.08493	142.4377	29.1915	138.2091	29.11955	138.8175	29.25153	141.4194
29.19797	116.3133	29.11872	133.3562	29.1016	142.4452	29.20817	138.2102	29.13622	138.8142	29.2682	141.4156
29.21463	116.309	29.13538	133.3505	29.11827	142.4356	29.22483	138.2105	29.15288	138.8114	29.28487	141.4176
29.2313	116.305	29.15205	133.3488	29.13493	142.4325	29.2415	138.2099	29.16955	138.8111	29.30153	141.4149
29.24797	116.298	29.16872	133.3486	29.1516	142.4316	29.25817	138.2061	29.18622	138.8095	29.3182	141.4115
29.26463	116.3006	29.18538	133.3449	29.16827	142.431	29.27483	138.2049	29.20288	138.8097	29.33487	141.4105
29.2813	116.2939	29.20205	133.342	29.18493	142.4322	29.2915	138.2044	29.21955	138.8098	29.35153	141.4109
29.29797	116.29	29.21872	133.3407	29.2016	142.4276	29.30817	138.2043	29.23622	138.806	29.3682	141.4076
29.31463	116.2839	29.23538	133.3408	29.21827	142.4269	29.32483	138.2011	29.25288	138.8053	29.38487	141.4067
29.3313	116.2832	29.25205	133.3383	29.23493	142.4259	29.3415	138.2024	29.26955	138.8034	29.40153	141.4046
29.34797	116.278	29.26872	133.3367	29.2516	142.4215	29.35817	138.2008	29.28622	138.8035	29.4182	141.4042
29.36463	116.2713	29.28538	133.3342	29.26827	142.4208	29.37483	138.1994	29.30288	138.8045	29.43487	141.4023
29.3813	116.2712	29.30205	133.3368	29.28493	142.4219	29.3915	138.1957	29.31955	138.8037	29.45153	141.4009
29.39797	116.2672	29.31872	133.3293	29.3016	142.4183	29.40817	138.1974	29.33622	138.8014	29.4682	141.3989
29.41463	116.2601	29.33538	133.3317	29.31827	142.4161	29.42483	138.1996	29.35288	138.8012	29.48487	141.3997
29.4313	116.2618	29.35205	133.3292	29.33493	142.4168	29.4415	138.1961	29.36955	138.8026	29.50153	141.4
29.44797	116.2544	29.36872	133.3204	29.3516	142.4138	29.45817	138.1926	29.38622	138.7986	29.5182	141.3981
29.46463	116.252	29.38538	133.3242	29.36827	142.4152	29.47483	138.1938	29.40288	138.7962	29.53487	141.3952
29.4813	116.2484	29.40205	133.3237	29.38493	142.4123	29.4915	138.1928	29.41955	138.7989	29.55153	141.3955
29.49797	116.2454	29.41872	133.3203	29.4016	142.4068	29.50817	138.1918	29.43622	138.7935	29.5682	141.3938
29.51463	116.2423	29.43538	133.3185	29.41827	142.4063	29.52483	138.1905	29.45288	138.7995	29.58487	141.3918
29.5313	116.239	29.45205	133.3167	29.43493	142.4084	29.5415	138.1898	29.46955	138.7957	29.60153	141.3909
29.54797	116.2329	29.46872	133.3136	29.4516	142.4033	29.55817	138.1901	29.48622	138.7929	29.6182	141.3901
29.56463	116.2304	29.48538	133.3136	29.46827	142.4029	29.57483	138.188	29.50288	138.7944	29.63487	141.3887
29.5813	116.226	29.50205	133.3112	29.48493	142.3994	29.5915	138.1852	29.51955	138.7905	29.65153	141.3883
29.59797	116.2214	29.51872	133.307	29.5016	142.3989	29.60817	138.1879	29.53622	138.7926	29.6682	141.386
29.61463	116.2187	29.53538	133.3062	29.51827	142.3966	29.62483	138.1863	29.55288	138.79	29.68487	141.3864
29.6313	116.2171	29.55205	133.3036	29.53493	142.3947	29.6415	138.1863	29.56955	138.7885	29.70153	141.383
29.64797	116.208	29.56872	133.3062	29.5516	142.3939	29.65817	138.1833	29.58622	138.7872	29.7182	141.383
29.66463	116.2098	29.58538	133.3009	29.56827	142.3911	29.67483	138.1827	29.60288	138.7859	29.73487	141.3799
29.6813	116.2025	29.60205	133.3018	29.58493	142.3913	29.6915	138.1793	29.61955	138.7853	29.75153	141.3796
29.69797	116.2006	29.61872	133.2965	29.6016	142.3872	29.70817	138.1782	29.63622	138.7848	29.7682	141.3783
29.71463	116.1953	29.63538	133.2941	29.61827	142.3865	29.72483	138.1799	29.65288	138.7841	29.78487	141.3769
29.7313	116.1926	29.65205	133.2937	29.63493	142.387	29.7415	138.1801	29.66955	138.7815	29.80153	141.3768
29.74797	116.1886	29.66872	133.2905	29.6516	142.3845	29.75817	138.1776	29.68622	138.7806	29.8182	141.375
29.76463	116.1852	29.68538	133.292	29.66827	142.383	29.77483	138.1769	29.70288	138.778	29.83487	141.3737
29.7813	116.1818	29.70205	133.2871	29.68493	142.374	29.7915	138.1758	29.71955	138.776	29.85153	141.3712
29.79797	116.1784	29.71872	133.284	29.7016	142.3769	29.80817	138.1729	29.73622	138.7768	29.8682	141.3721
29.81463	116.1736	29.73538	133.2845	29.71827	142.377	29.82483	138.1729	29.75288	138.7746	29.88487	141.3707
29.8313	116.1719	29.75205	133.2819	29.73493	142.3734	29.8415	138.1703	29.76955	138.7732	29.90153	141.3713
29.84797	116.1678	29.76872	133.2787	29.7516	142.3728	29.85817	138.1711	29.78622	138.771	29.9182	141.3689
29.86463	116.1634	29.78538	133.278	29.76827	142.3716	29.87483	138.1727	29.80288	138.768	29.93487	141.3693
29.8813	116.1582	29.80205	133.2776	29.78493	142.3687	29.8915	138.1675	29.81955	138.7693	29.95153	141.3657
29.89797	116.1572	29.81872	133.2739	29.8016	142.3676	29.90817	138.169	29.83622	138.7691	29.9682	141.3639
29.91463	116.1539	29.83538	133.2752	29.81827	142.3647	29.92483	138.1668	29.85288	138.7639	29.98487	141.3623
29.9313	116.1487	29.85205	133.2713	29.83493	142.3641	29.9415	138.1654	29.86955	138.7653	30.00153	141.3617
29.94797	116.1444	29.86872	133.2698	29.8516	142.3613	29.95817	138.1658	29.88622	138.7635	30.0182	141.3599
29.96463	116.1408	29.88538	133.2667	29.86827	142.3585	29.97483	138.164	29.90288	138.7591	30.03487	141.3602
29.9813	116.1386	29.90205	133.264	29.88493	142.3575	29.9915	138.1609	29.91955	138.7598	30.05153	141.3587
29.99797	116.1355	29.91872	133.2639	29.9016	142.3585	30.00817	138.1601	29.93622	138.7599	30.0682	141.3576
30.01463	116.1328	29.93538	133.26	29.91827	142.3532	30.02483	138.1608	29.95288	138.7588	30.08487	141.3569
30.0313	116.1309	29.95205	133.2598	29.93493	142.35	30.0415	138.1591	29.96955	138.7573	30.10153	141.3569
30.04797	116.1252	29.96872	133.2575	29.9516	142.35	30.05817	138.1553	29.98622	138.7576	30.1182	141.3526
30.06463	116.121	29.98538	133.2557	29.96827	142.3465	30.07483	138.1562	30.00288	138.7569	30.13487	141.3551
30.0813	116.1157	30.00205	133.2541	29.98493	142.3448	30.0915	138.156	30.01955	138.7546	30.15153	141.3527
30.09797	116.1151	30.01872	133.2519	30.0016	142.3473	30.10817	138.1515	30.03622	138.7545	30.1682	141.3518
30.11463	116.1139	30.03538	133.2503	30.01827	142.3446	30.12483	138.1538	30.05288	138.7501	30.18487	141.3515
30.1313	116.1071	30.05205	133.2513	30.03493	142.3405	30.1415	138.151	30.06955	138.7502	30.20153	141.3493
30.14797	116.1064	30.06872	133.2483	30.0516	142.3389	30.15817	138.1495	30.08622	138.7493	30.2182	141.3476
30.16463	116.0989	30.08538	133.2483	30.06827	142.3377	30.17483	138.1479	30.10288	138.7494	30.23487	141.3485
30.1813	116.0959	30.10205	133.2431	30.08493	142.3349	30.1915	138.1468	30.11955	138.7462	30.25153	141.3443
30.19797	116.0955	30.11872	133.246	30.1016	142.3344	30.20817	138.1453	30.13622	138.7462	30.2682	141.3459
30.21463	116.0903	30.13538	133.2417	30.11827	142.3337	30.22483	138.1427	30.15288	138.7429	30.28487	141.3444
30.2313	116.0862	30.15205	133.2336	30.13493	142.3309	30.2415	138.1447	30.16955	138.7438	30.30153	141.3433
30.24797	116.0858	30.16872	133.2362	30.1516	142.3282	30.25817	138.1415	30.18622	138.743	30.3182	141.3425
30.26463	116.0775	30.18538	133.233	30.16827	142.3271	30.27483	138.1408	30.20288	138.741	30.33487	141.3406
30.2813	116.0772	30.20205	133.2325	30.18493	142.3261	30.2915	138.14	30.21955	138.7402	30.35153	141.3379
30.29797	116.0713	30.21872	133.2308	30.2016	142.3234	30.30817	138.1387	30.23622	138.7387	30.3682	141.3364
30.31463	116.0676	30.23538	133.2282	30.21827	142.3223	30.32483	138.1354	30.25288	138.7397	30.38487	141.3375
30.3313	116.0666	30.25205	133.2264	30.23493	142.3199	30.3415	138.1346	30.26955	138.7374	30.40153	141.3364
30.34797	116.0583	30.26872	133.226	30.2516	142.321	30.35817	138.1339	30.28622	138.7357	30.4182	141.3332
30.36463	116.0562	30.28538	133.2212	30.26827	142.318	30.37483	138.1342	30.30288	138.7363	30.43487	141.3319
30.3813	116.0558	30.30205	133.2215	30.28493	142.3169	30.3915	138.1333	30.31955	138.7321	30.45153	141.3295
30.39797	116.0464	30.31872	133.2187	30.3016	142.3177	30.40817	138.1343	30.33622	138.7301	30.4682	141.3299
30.41463	116.0453	30.33538	133.2178	30.31827	142.3136	30.42483	138.131	30.35288	138.7281	30.48487	141.3303
30.4313	116.0415	30.35205	133.214	30.33493	142.308	30.4415	138.1284	30.36955	138.7283	30.50153	141.3282
30.44797	116.0355	30.36872	133.2117	30.3516	142.31	30.45817	138.1268	30.38622	138.7278	30.5182	141.3271
30.46463	116.035	30.38538	133.2106	30.36827	142.3102	30.47483	138.1254	30.40288	138.7268	30.53487	141.3254
30.4813	116.0283	30.40205	133.2069	30.38493	142.3078	30.4915	138.1266	30.41955	138.7245	30.55153	141.326
30.49797	116.0252	30.41872	133.207	30.4016	142.3057	30.50817	138.124	30.43622	138.7244	30.5682	141.3241
30.51463	116.0214	30.43538	133.2037	30.41827	142.3056	30.52483	138.1202	30.45288	138.7244	30.58487	141.3235
30.5313	116.018	30.45205	133.2013	30.43493	142.3059	30.5415	138.1209	30.46955	138.7215	30.60153	141.3208
30.54797	116.0149	30.46872	133.2005	30.4516	142.3003	30.55817	138.1163	30.48622	138.7206	30.6182	141.3197
30.56463	116.0137	30.48538	133.1986	30.46827	142.3008	30.57483	138.1159	30.50288	138.7194	30.63487	141.3192
30.5813	116.0073	30.50205	133.1946	30.48493	142.297	30.5915	138.1153	30.51955	138.7195	30.65153	141.3193
30.59797	116.0031	30.51872	133.1934	30.5016	142.2968	30.60817	138.1153	30.53622	138.7181	30.6682	141.3179
30.61463	115.9991	30.53538	133.1904	30.51827	142.2958	30.62483	138.1153	30.55288	138.7163	30.68487	141.3155
30.6313	115.9961	30.55205	133.1898	30.53493	142.2918	30.6415	138.1131	30.56955	138.7122	30.70153	141.3161
30.64797	115.9926	30.56872	133.1855	30.5516	142.2934	30.65817	138.1131	30.58622	138.7127	30.7182	141.3158
30.66463	115.987	30.58538	133.1855	30.56827	142.2885	30.67483	138.1139	30.60288	138.714	30.73487	141.3155
30.6813	115.9845	30.60205	133.1832	30.58493	142.2899	30.6915	138.1101	30.61955	138.7109	30.75153	141.3127
30.69797	115.9808	30.61872	133.1822	30.6016	142.2868	30.70817	138.1076	30.63622	138.7091	30.7682	141.3123
30.71463	115.9756	30.63538	133.1783	30.61827	142.2876	30.72483	138.11	30.65288	138.7082	30.78487	141.3116
30.7313	115.9735	30.65205	133.1768	30.63493	142.2827	30.7415	138.1066	30.66955	138.7086	30.80153	141.3106
30.74797	115.9685	30.66872	133.1759	30.6516	142.2821	30.75817	138.1058	30.68622	138.7065	30.8182	141.3076
30.76463	115.9641	30.68538	133.1735	30.66827	142.2815	30.77483	138.104	30.70288	138.7049	30.83487	141.3069

30.7813	115.9592	30.70205	133.173	30.68493	142.2814	30.7915	138.1002	30.71955	138.7024	30.85153	141.3068
30.79797	115.955	30.71872	133.1697	30.7016	142.2788	30.80817	138.0991	30.73622	138.7039	30.8682	141.3048
30.81463	115.9537	30.73538	133.1687	30.71827	142.2763	30.82483	138.0974	30.75288	138.7012	30.88487	141.3031
30.8313	115.9496	30.75205	133.1667	30.73493	142.2737	30.8415	138.0989	30.76955	138.7029	30.90153	141.304
30.84797	115.9461	30.76872	133.165	30.7516	142.2744	30.85817	138.0966	30.78622	138.6986	30.9182	141.303
30.86463	115.9411	30.78538	133.1617	30.76827	142.2733	30.87483	138.0964	30.80288	138.6987	30.93487	141.301
30.8813	115.9376	30.80205	133.1618	30.78493	142.2671	30.8915	138.0943	30.81955	138.697	30.95153	141.2993
30.89797	115.9362	30.81872	133.1612	30.8016	142.2679	30.90817	138.0981	30.83622	138.6959	30.9682	141.2976
30.91463	115.9334	30.83538	133.1577	30.81827	142.2663	30.92483	138.0932	30.85288	138.6934	30.98487	141.2981
30.9313	115.9291	30.85205	133.1569	30.83493	142.263	30.9415	138.0895	30.86955	138.6936	31.00153	141.2963
30.94797	115.9282	30.86872	133.1546	30.8516	142.2614	30.95817	138.0881	30.88622	138.69	31.0182	141.2943
30.96463	115.9241	30.88538	133.1508	30.86827	142.2586	30.97483	138.0908	30.90288	138.6914	31.03487	141.2949
30.9813	115.9219	30.90205	133.1504	30.88493	142.2583	30.9915	138.0851	30.91955	138.6891	31.05153	141.2943
30.99797	115.9185	30.91872	133.1497	30.9016	142.2589	31.00817	138.0869	30.93622	138.6865	31.0682	141.2932
31.01463	115.9133	30.93538	133.1469	30.91827	142.2533	31.02483	138.0863	30.95288	138.684	31.08487	141.291
31.0313	115.9108	30.95205	133.1443	30.93493	142.2512	31.0415	138.0845	30.96955	138.6865	31.10153	141.2896
31.04797	115.9075	30.96872	133.1404	30.9516	142.2502	31.05817	138.0823	30.98622	138.682	31.1182	141.2883
31.06463	115.9036	30.98538	133.1421	30.96827	142.2477	31.07483	138.083	31.00288	138.6837	31.13487	141.2893
31.0813	115.8996	31.00205	133.1401	30.98493	142.2461	31.0915	138.083	31.01955	138.68	31.15153	141.2875
31.09797	115.8939	31.01872	133.1402	31.0016	142.2451	31.10817	138.0806	31.03622	138.6801	31.1682	141.2859
31.11463	115.8909	31.03538	133.1361	31.01827	142.2448	31.12483	138.0797	31.05288	138.6798	31.18487	141.2845
31.1313	115.8864	31.05205	133.1362	31.03493	142.2418	31.1415	138.0794	31.06955	138.6772	31.20153	141.2845
31.14797	115.8843	31.06872	133.131	31.0516	142.2369	31.15817	138.0787	31.08622	138.6775	31.2182	141.2813
31.16463	115.8823	31.08538	133.1318	31.06827	142.2362	31.17483	138.0763	31.10288	138.6756	31.23487	141.2812
31.1813	115.8753	31.10205	133.1305	31.08493	142.2357	31.1915	138.0772	31.11955	138.6737	31.25153	141.2808
31.19797	115.8719	31.11872	133.1275	31.1016	142.2325	31.20817	138.0748	31.13622	138.6732	31.2682	141.2788
31.21463	115.8673	31.13538	133.125	31.11827	142.2309	31.22483	138.0738	31.15288	138.6732	31.28487	141.2764
31.2313	115.8629	31.15205	133.1251	31.13493	142.2273	31.2415	138.0744	31.16955	138.6733	31.30153	141.2764
31.24797	115.861	31.16872	133.1231	31.1516	142.2285	31.25817	138.0716	31.18622	138.6726	31.3182	141.2713
31.26463	115.8565	31.18538	133.1199	31.16827	142.2263	31.27483	138.0706	31.20288	138.6727	31.33487	141.2741
31.2813	115.8524	31.20205	133.1197	31.18493	142.2249	31.2915	138.0713	31.21955	138.6696	31.35153	141.2729
31.29797	115.848	31.21872	133.119	31.2016	142.2212	31.30817	138.0689	31.23622	138.6717	31.3682	141.2714
31.31463	115.8417	31.23538	133.1179	31.21827	142.2172	31.32483	138.0682	31.25288	138.6694	31.38487	141.2682
31.3313	115.8408	31.25205	133.1142	31.23493	142.2183	31.3415	138.0688	31.26955	138.6681	31.40153	141.2696
31.34797	115.8357	31.26872	133.1112	31.2516	142.213	31.35817	138.0652	31.28622	138.6675	31.4182	141.2682
31.36463	115.8325	31.28538	133.1116	31.26827	142.2136	31.37483	138.0648	31.30288	138.6663	31.43487	141.2658
31.3813	115.8293	31.30205	133.1082	31.28493	142.2114	31.3915	138.0654	31.31955	138.663	31.45153	141.2645
31.39797	115.8239	31.31872	133.106	31.3016	142.209	31.40817	138.0632	31.33622	138.6645	31.4682	141.2622
31.41463	115.8219	31.33538	133.1049	31.31827	142.2071	31.42483	138.0623	31.35288	138.6641	31.48487	141.2605
31.4313	115.8172	31.35205	133.1041	31.33493	142.2051	31.4415	138.0606	31.36955	138.6608	31.50153	141.2626
31.44797	115.8117	31.36872	133.1034	31.3516	142.203	31.45817	138.0608	31.38622	138.6595	31.5182	141.2622
31.46463	115.8084	31.38538	133.1011	31.36827	142.2018	31.47483	138.057	31.40288	138.6581	31.53487	141.2585
31.4813	115.8062	31.40205	133.0975	31.38493	142.2001	31.4915	138.0595	31.41955	138.6548	31.55153	141.258
31.49797	115.803	31.41872	133.0973	31.4016	142.1986	31.50817	138.0586	31.43622	138.6573	31.5682	141.2582
31.51463	115.7991	31.43538	133.0961	31.41827	142.1972	31.52483	138.0534	31.45288	138.6536	31.58487	141.2552
31.5313	115.7964	31.45205	133.0935	31.43493	142.1955	31.5415	138.0521	31.46955	138.6567	31.60153	141.256
31.54797	115.7904	31.46872	133.0903	31.4516	142.1917	31.55817	138.0541	31.48622	138.6514	31.6182	141.255
31.56463	115.7851	31.48538	133.0925	31.46827	142.1933	31.57483	138.0509	31.50288	138.6524	31.63487	141.2517
31.5813	115.7819	31.50205	133.09	31.48493	142.187	31.5915	138.0509	31.51955	138.6515	31.65153	141.2504
31.59797	115.7792	31.51872	133.0856	31.5016	142.1884	31.60817	138.0486	31.53622	138.6491	31.6682	141.2516
31.61463	115.7731	31.53538	133.0853	31.51827	142.1858	31.62483	138.0531	31.55288	138.6478	31.68487	141.2513
31.6313	115.7722	31.55205	133.0822	31.53493	142.1844	31.6415	138.0514	31.56955	138.6475	31.70153	141.2491
31.64797	115.768	31.56872	133.0789	31.5516	142.184	31.65817	138.0517	31.58622	138.647	31.7182	141.2466
31.66463	115.7657	31.58538	133.0781	31.56827	142.1799	31.67483	138.0448	31.60288	138.6472	31.73487	141.2472
31.6813	115.7626	31.60205	133.0778	31.58493	142.1791	31.6915	138.0445	31.61955	138.6416	31.75153	141.245
31.69797	115.7567	31.61872	133.0708	31.6016	142.1795	31.70817	138.0455	31.63622	138.6437	31.7682	141.2478
31.71463	115.7543	31.63538	133.0747	31.61827	142.1762	31.72483	138.0475	31.65288	138.6409	31.78487	141.2453
31.7313	115.7519	31.65205	133.0725	31.63493	142.174	31.7415	138.0431	31.66955	138.6411	31.80153	141.2435
31.74797	115.744	31.66872	133.0702	31.6516	142.1711	31.75817	138.0424	31.68622	138.6373	31.8182	141.2434
31.76463	115.7452	31.68538	133.0691	31.66827	142.1723	31.77483	138.0435	31.70288	138.6394	31.83487	141.2422
31.7813	115.7389	31.70205	133.0695	31.68493	142.1705	31.7915	138.0424	31.71955	138.6346	31.85153	141.2388
31.79797	115.7343	31.71872	133.0641	31.7016	142.1664	31.80817	138.0425	31.73622	138.6358	31.8682	141.2382
31.81463	115.733	31.73538	133.0638	31.71827	142.1669	31.82483	138.0415	31.75288	138.6318	31.88487	141.2388
31.8313	115.7289	31.75205	133.0612	31.73493	142.163	31.8415	138.036	31.76955	138.6302	31.90153	141.2355
31.84797	115.7229	31.76872	133.0585	31.7516	142.1604	31.85817	138.0399	31.78622	138.6306	31.9182	141.2356
31.86463	115.7235	31.78538	133.0591	31.76827	142.1624	31.87483	138.0376	31.80288	138.6315	31.93487	141.2362
31.8813	115.7199	31.80205	133.057	31.78493	142.1578	31.8915	138.034	31.81955	138.6277	31.95153	141.2325
31.89797	115.7106	31.81872	133.0543	31.8016	142.1563	31.90817	138.0358	31.83622	138.6261	31.9682	141.2332
31.91463	115.712	31.83538	133.0535	31.81827	142.1539	31.92483	138.0335	31.85288	138.6259	31.98487	141.2308
31.9313	115.7082	31.85205	133.0518	31.83493	142.1535	31.9415	138.0324	31.86955	138.6246	32.00153	141.2315
31.94797	115.7076	31.86872	133.0497	31.8516	142.152	31.95817	138.035	31.88622	138.6246	32.0182	141.2293
31.96463	115.7024	31.88538	133.051	31.86827	142.1495	31.97483	138.0265	31.90288	138.622	32.03487	141.2297
31.9813	115.6969	31.90205	133.0455	31.88493	142.1469	31.9915	138.0293	31.91955	138.6209	32.05153	141.2267
31.99797	115.6939	31.91872	133.0473	31.9016	142.1428	32.00817	138.0301	31.93622	138.6197	32.0682	141.2273
32.01463	115.6935	31.93538	133.0436	31.91827	142.1465	32.02483	138.0267	31.95288	138.6194	32.08487	141.2266
32.0313	115.6876	31.95205	133.0451	31.93493	142.1419	32.0415	138.0222	31.96955	138.6201	32.10153	141.2262
32.04797	115.6863	31.96872	133.0391	31.9516	142.1399	32.05817	138.0237	31.98622	138.6175	32.1182	141.2236
32.06463	115.6816	31.98538	133.0435	31.96827	142.1395	32.07483	138.0195	32.00288	138.6139	32.13487	141.2241
32.0813	115.6737	32.00205	133.0361	31.98493	142.1388	32.0915	138.0203	32.01955	138.6124	32.15153	141.2226
32.09797	115.6741	32.01872	133.0373	32.0016	142.1342	32.10817	138.0194	32.03622	138.613	32.1682	141.2227
32.11463	115.6689	32.03538	133.0335	32.01827	142.1341	32.12483	138.0153	32.05288	138.6122	32.18487	141.2208
32.1313	115.6646	32.05205	133.0322	32.03493	142.1308	32.1415	138.0174	32.06955	138.6132	32.20153	141.2204
32.14797	115.6621	32.06872	133.0265	32.0516	142.1359	32.15817	138.0127	32.08622	138.6114	32.2182	141.2195
32.16463	115.6585	32.08538	133.0341	32.06827	142.1282	32.17483	138.0139	32.10288	138.6078	32.23487	141.2192
32.1813	115.6531	32.10205	133.0254	32.08493	142.1364	32.1915	138.011	32.11955	138.608	32.25153	141.2175
32.19797	115.6521	32.11872	133.0228	32.1016	142.1258	32.20817	138.0116	32.13622	138.6081	32.2682	141.2155
32.21463	115.6494	32.13538	133.0205	32.11827	142.1259	32.22483	138.0099	32.15288	138.6037	32.28487	141.2137
32.2313	115.6438	32.15205	133.0201	32.13493	142.1235	32.2415	138.0054	32.16955	138.6039	32.30153	141.2139
32.24797	115.6407	32.16872	133.0196	32.1516	142.1191	32.25817	138.0066	32.18622	138.6033	32.3182	141.2127
32.26463	115.6372	32.18538	133.0201	32.16827	142.1194	32.27483	138.0049	32.20288	138.6033	32.33487	141.2104
32.2813	115.6305	32.20205	133.0173	32.18493	142.1158	32.2915	138.004	32.21955	138.6027	32.35153	141.2091
32.29797	115.6269	32.21872	133.0136	32.2016	142.1172	32.30817	138.0027	32.23622	138.5999	32.3682	141.2084
32.31463	115.6237	32.23538	133.0115	32.21827	142.1145	32.32483	137.9988	32.25288	138.5976	32.38487	141.209
32.3313	115.6191	32.25205	133.0159	32.23493	142.1126	32.3415	138.0012	32.26955	138.5987	32.40153	141.206
32.34797	115.618	32.26872	133.0126	32.2516	142.1106	32.35817	137.9989	32.28622	138.5963	32.4182	141.2032
32.36463	115.6137	32.28538	133.0093	32.26827	142.1113	32.37483	137.9972	32.30288	138.5968	32.43487	141.2048
32.3813	115.6074	32.30205	133.0046	32.28493	142.1079	32.3915	137.9972	32.31955	138.5953	32.45153	141.2022

32.39797	115.6076	32.31872	133.0068	32.3016	142.1067	32.40817	137.9953	32.33622	138.5976	32.4682	141.2011
32.41463	115.6033	32.33538	133.0034	32.31827	142.1078	32.42483	137.9956	32.35288	138.5913	32.48487	141.1988
32.4313	115.5975	32.35205	133.0017	32.33493	142.1057	32.4415	137.9953	32.36955	138.5911	32.50153	141.1993
32.44797	115.5953	32.36872	132.9994	32.3516	142.1055	32.45817	137.9938	32.38622	138.5905	32.5182	141.1978
32.46463	115.5904	32.38538	132.9995	32.36827	142.1001	32.47483	137.9899	32.40288	138.5906	32.53487	141.196
32.4813	115.585	32.40205	132.9972	32.38493	142.1006	32.4915	137.9915	32.41955	138.5897	32.55153	141.1938
32.49797	115.5819	32.41872	132.9921	32.4016	142.0974	32.50817	137.9889	32.43622	138.5895	32.5682	141.1952
32.51463	115.5773	32.43538	132.9937	32.41827	142.0955	32.52483	137.9875	32.45288	138.5849	32.58487	141.1932
32.5313	115.5758	32.45205	132.9921	32.43493	142.0931	32.5415	137.9887	32.46955	138.587	32.60153	141.1924
32.54797	115.5729	32.46872	132.9899	32.4516	142.0923	32.55817	137.9849	32.48622	138.5855	32.6182	141.189
32.56463	115.5697	32.48538	132.9884	32.46827	142.0901	32.57483	137.9844	32.50288	138.5857	32.63487	141.1912
32.5813	115.565	32.50205	132.9904	32.48493	142.0876	32.5915	137.9847	32.51955	138.5817	32.65153	141.1874
32.59797	115.5647	32.51872	132.9829	32.5016	142.0865	32.60817	137.9817	32.53622	138.5824	32.6682	141.1865
32.61463	115.5621	32.53538	132.9796	32.51827	142.0829	32.62483	137.9818	32.55288	138.5791	32.68487	141.1845
32.6313	115.5559	32.55205	132.9824	32.53493	142.0834	32.6415	137.9791	32.56955	138.5791	32.70153	141.1866
32.64797	115.5486	32.56872	132.9773	32.5516	142.0819	32.65817	137.9786	32.58622	138.5798	32.7182	141.1831
32.66463	115.5493	32.58538	132.9763	32.56827	142.0812	32.67483	137.9783	32.60288	138.579	32.73487	141.1838
32.6813	115.5437	32.60205	132.9776	32.58493	142.0776	32.6915	137.9758	32.61955	138.5777	32.75153	141.1816
32.69797	115.5397	32.61872	132.9706	32.6016	142.0757	32.70817	137.9739	32.63622	138.5769	32.7682	141.1824
32.71463	115.5393	32.63538	132.9695	32.61827	142.0768	32.72483	137.9744	32.65288	138.5762	32.78487	141.1794
32.7313	115.5342	32.65205	132.9705	32.63493	142.0722	32.7415	137.9702	32.66955	138.5723	32.80153	141.1786
32.74797	115.5268	32.66872	132.9675	32.6516	142.0741	32.75817	137.9708	32.68622	138.5717	32.8182	141.1762
32.76463	115.5263	32.68538	132.969	32.66827	142.07	32.77483	137.9714	32.70288	138.5724	32.83487	141.1762
32.7813	115.5192	32.70205	132.9658	32.68493	142.0696	32.7915	137.969	32.71955	138.5706	32.85153	141.1741
32.79797	115.5181	32.71872	132.9636	32.7016	142.0663	32.80817	137.9674	32.73622	138.5703	32.8682	141.1728
32.81463	115.5164	32.73538	132.9598	32.71827	142.0674	32.82483	137.9666	32.75288	138.5686	32.88487	141.1711
32.8313	115.5099	32.75205	132.9608	32.73493	142.0647	32.8415	137.9683	32.76955	138.5672	32.90153	141.1705
32.84797	115.5075	32.76872	132.9575	32.7516	142.0637	32.85817	137.9672	32.78622	138.5647	32.9182	141.1697
32.86463	115.5048	32.78538	132.9559	32.76827	142.0609	32.87483	137.9656	32.80288	138.5648	32.93487	141.1673
32.8813	115.5019	32.80205	132.9548	32.78493	142.0607	32.8915	137.9638	32.81955	138.5632	32.95153	141.1659
32.89797	115.4991	32.81872	132.9537	32.8016	142.0568	32.90817	137.9617	32.83622	138.5618	32.9682	141.1663
32.91463	115.4941	32.83538	132.9508	32.81827	142.0581	32.92483	137.962	32.85288	138.5602	32.98487	141.162
32.9313	115.4913	32.85205	132.9476	32.83493	142.056	32.9415	137.9615	32.86955	138.5587	33.00153	141.1651
32.94797	115.488	32.86872	132.9491	32.8516	142.0535	32.95817	137.9618	32.88622	138.559	33.0182	141.1637
32.96463	115.4843	32.88538	132.9448	32.86827	142.0511	32.97483	137.96	32.90288	138.5584	33.03487	141.1622
32.9813	115.4802	32.90205	132.9425	32.88493	142.0488	32.9915	137.9574	32.91955	138.5564	33.05153	141.1573
32.99797	115.4772	32.91872	132.942	32.9016	142.0456	33.00817	137.9568	32.93622	138.555	33.0682	141.162
33.01463	115.4732	32.93538	132.9405	32.91827	142.0498	33.02483	137.9575	32.95288	138.553	33.08487	141.16
33.0313	115.4714	32.95205	132.9389	32.93493	142.0472	33.0415	137.9564	32.96955	138.5531	33.10153	141.1536
33.04797	115.468	32.96872	132.9353	32.9516	142.0456	33.05817	137.9557	32.98622	138.5511	33.1182	141.1568
33.06463	115.4639	32.98538	132.9347	32.96827	142.0445	33.07483	137.9519	33.00288	138.5509	33.13487	141.1535
33.0813	115.4584	33.00205	132.9313	32.98493	142.0424	33.0915	137.9511	33.01955	138.5511	33.15153	141.1517
33.09797	115.4565	33.01872	132.9341	33.0016	142.0392	33.10817	137.9531	33.03622	138.5489	33.1682	141.1521
33.11463	115.4539	33.03538	132.9304	33.01827	142.0409	33.12483	137.951	33.05288	138.5475	33.18487	141.1518
33.1313	115.4497	33.05205	132.9297	33.03493	142.0365	33.1415	137.9479	33.06955	138.5475	33.20153	141.1474
33.14797	115.4468	33.06872	132.9282	33.0516	142.0361	33.15817	137.9476	33.08622	138.5443	33.2182	141.1473
33.16463	115.4451	33.08538	132.9239	33.06827	142.0329	33.17483	137.9475	33.10288	138.5444	33.23487	141.1455
33.1813	115.4391	33.10205	132.9241	33.08493	142.0347	33.1915	137.9477	33.11955	138.5424	33.25153	141.1452
33.19797	115.4375	33.11872	132.9267	33.1016	142.0299	33.20817	137.9443	33.13622	138.5407	33.2682	141.147
33.21463	115.4362	33.13538	132.9197	33.11827	142.0327	33.22483	137.942	33.15288	138.5392	33.28487	141.1434
33.2313	115.4316	33.15205	132.9164	33.13493	142.0273	33.2415	137.9421	33.16955	138.5401	33.30153	141.142
33.24797	115.4257	33.16872	132.9157	33.1516	142.0276	33.25817	137.9437	33.18622	138.5394	33.3182	141.1404
33.26463	115.4236	33.18538	132.9126	33.16827	142.0252	33.27483	137.9416	33.20288	138.536	33.33487	141.1389
33.2813	115.4189	33.20205	132.911	33.18493	142.0241	33.2915	137.9411	33.21955	138.5353	33.35153	141.1389
33.29797	115.4162	33.21872	132.9121	33.2016	142.0212	33.30817	137.9376	33.23622	138.5356	33.3682	141.1371
33.31463	115.4119	33.23538	132.91	33.21827	142.0196	33.32483	137.9368	33.25288	138.5344	33.38487	141.1352
33.3313	115.4105	33.25205	132.9069	33.23493	142.0183	33.3415	137.9347	33.26955	138.5318	33.40153	141.1356
33.34797	115.4059	33.26872	132.9083	33.2516	142.0184	33.35817	137.9371	33.28622	138.5303	33.4182	141.1345
33.36463	115.4017	33.28538	132.9031	33.26827	142.0131	33.37483	137.9349	33.30288	138.5305	33.43487	141.132
33.3813	115.3988	33.30205	132.9024	33.28493	142.0137	33.3915	137.9345	33.31955	138.5286	33.45153	141.1298
33.39797	115.3967	33.31872	132.9018	33.3016	142.011	33.40817	137.9325	33.33622	138.5272	33.4682	141.1313
33.41463	115.3915	33.33538	132.8982	33.31827	142.0101	33.42483	137.9293	33.35288	138.5253	33.48487	141.1286
33.4313	115.3881	33.35205	132.8975	33.33493	142.0077	33.4415	137.929	33.36955	138.524	33.50153	141.1283
33.44797	115.3842	33.36872	132.895	33.3516	142.0066	33.45817	137.9286	33.38622	138.5244	33.5182	141.127
33.46463	115.3808	33.38538	132.8938	33.36827	142.009	33.47483	137.9266	33.40288	138.5225	33.53487	141.1257
33.4813	115.3786	33.40205	132.89	33.38493	142.0057	33.4915	137.927	33.41955	138.5221	33.55153	141.1233
33.49797	115.3734	33.41872	132.8893	33.4016	141.9987	33.50817	137.9271	33.43622	138.5213	33.5682	141.1216
33.51463	115.3687	33.43538	132.8881	33.41827	142	33.52483	137.9258	33.45288	138.5189	33.58487	141.1227
33.5313	115.3679	33.45205	132.8854	33.43493	141.9993	33.5415	137.9224	33.46955	138.5185	33.60153	141.1208
33.54797	115.362	33.46872	132.8854	33.4516	141.9974	33.55817	137.9248	33.48622	138.5152	33.6182	141.1192
33.56463	115.3566	33.48538	132.8843	33.46827	141.9943	33.57483	137.9224	33.50288	138.5131	33.63487	141.1154
33.5813	115.3614	33.50205	132.8802	33.48493	141.9931	33.5915	137.9204	33.51955	138.5117	33.65153	141.1162
33.59797	115.354	33.51872	132.8774	33.5016	141.9927	33.60817	137.9204	33.53622	138.5124	33.6682	141.1168
33.61463	115.3484	33.53538	132.8767	33.51827	141.9874	33.62483	137.9198	33.55288	138.5108	33.68487	141.1146
33.6313	115.3455	33.55205	132.8777	33.53493	141.987	33.6415	137.9188	33.56955	138.5086	33.70153	141.113
33.64797	115.343	33.56872	132.8731	33.5516	141.9858	33.65817	137.9197	33.58622	138.5082	33.7182	141.1138
33.66463	115.336	33.58538	132.8743	33.56827	141.9857	33.67483	137.9162	33.60288	138.5068	33.73487	141.1114
33.6813	115.3362	33.60205	132.8711	33.58493	141.9771	33.6915	137.9171	33.61955	138.5032	33.75153	141.1096
33.69797	115.3306	33.61872	132.8682	33.6016	141.9821	33.70817	137.9149	33.63622	138.5032	33.7682	141.1095
33.71463	115.3274	33.63538	132.8664	33.61827	141.9817	33.72483	137.9148	33.65288	138.5004	33.78487	141.1089
33.7313	115.3233	33.65205	132.8652	33.63493	141.9766	33.7415	137.9125	33.66955	138.5011	33.80153	141.1077
33.74797	115.3242	33.66872	132.8641	33.6516	141.9753	33.75817	137.9144	33.68622	138.5015	33.8182	141.1067
33.76463	115.3152	33.68538	132.8626	33.66827	141.9746	33.77483	137.9135	33.70288	138.5002	33.83487	141.1077
33.7813	115.3151	33.70205	132.8618	33.68493	141.9732	33.7915	137.9155	33.71955	138.4981	33.85153	141.104
33.79797	115.3117	33.71872	132.8604	33.7016	141.9692	33.80817	137.9112	33.73622	138.4982	33.8682	141.1062
33.81463	115.3066	33.73538	132.8561	33.71827	141.9698	33.82483	137.9087	33.75288	138.4966	33.88487	141.1035
33.8313	115.3027	33.75205	132.8561	33.73493	141.9693	33.8415	137.9097	33.76955	138.4935	33.90153	141.1008
33.84797	115.3033	33.76872	132.8541	33.7516	141.9668	33.85817	137.9097	33.78622	138.4947	33.9182	141.1012
33.86463	115.294	33.78538	132.8516	33.76827	141.9663	33.87483	137.9096	33.80288	138.4933	33.93487	141.0996
33.8813	115.2931	33.80205	132.8515	33.78493	141.9615	33.8915	137.9071	33.81955	138.4914	33.95153	141.0982
33.89797	115.2879	33.81872	132.8507	33.8016	141.9607	33.90817	137.9047	33.83622	138.4897	33.9682	141.0968
33.91463	115.283	33.83538	132.8469	33.81827	141.9609	33.92483	137.9058	33.85288	138.4885	33.98487	141.0981
33.9313	115.2831	33.85205	132.8461	33.83493	141.9539	33.9415	137.9042	33.86955	138.4856	34.00153	141.0939
33.94797	115.2777	33.86872	132.845	33.8516	141.9558	33.95817	137.901	33.88622	138.4857	34.0182	141.0937
33.96463	115.2711	33.88538	132.8434	33.86827	141.9543	33.97483	137.9017	33.90288	138.4856	34.03487	141.0944
33.9813	115.2701	33.90205	132.8417	33.88493	141.9519	33.9915	137.9036	33.91955	138.4832	34.05153	141.0924
33.99797	115.2648	33.91872	132.8401	33.9016	141.9516	34.00817	137.902	33.93622	138.4814	34.0682	141.0899

34.01463	115.2639	33.93538	132.8373	33.91827	141.9512	34.02483	137.9019	33.95288	138.4802	34.08487	141.0903
34.0313	115.26	33.95205	132.8368	33.93493	141.9467	34.0415	137.8988	33.96955	138.4788	34.10153	141.0886
34.04797	115.2557	33.96872	132.8355	33.9516	141.9441	34.05817	137.8996	33.98622	138.476	34.1182	141.0864
34.06463	115.2539	33.98538	132.8344	33.96827	141.9457	34.07483	137.9005	34.00288	138.4777	34.13487	141.0868
34.0813	115.2513	34.00205	132.8314	33.98493	141.9408	34.0915	137.8974	34.01955	138.4762	34.15153	141.0855
34.09797	115.2476	34.01872	132.8296	34.0016	141.9385	34.10817	137.8957	34.03622	138.4756	34.1682	141.0833
34.11463	115.2429	34.03538	132.8296	34.01827	141.9397	34.12483	137.8971	34.05288	138.4737	34.18487	141.0819
34.1313	115.2389	34.05205	132.8271	34.03493	141.9364	34.1415	137.8952	34.06955	138.4729	34.20153	141.0832
34.14797	115.2353	34.06872	132.8251	34.0516	141.9372	34.15817	137.8955	34.08622	138.4694	34.2182	141.08
34.16463	115.2326	34.08538	132.8241	34.06827	141.9324	34.17483	137.8947	34.10288	138.4694	34.23487	141.0801
34.1813	115.2242	34.10205	132.8195	34.08493	141.9332	34.1915	137.8926	34.11955	138.4685	34.25153	141.0779
34.19797	115.2251	34.11872	132.8208	34.1016	141.9326	34.20817	137.8928	34.13622	138.4681	34.2682	141.0753
34.21463	115.2203	34.13538	132.8179	34.11827	141.9291	34.22483	137.8921	34.15288	138.4624	34.28487	141.0762
34.2313	115.2183	34.15205	132.8195	34.13493	141.9312	34.2415	137.8881	34.16955	138.4624	34.30153	141.0749
34.24797	115.2146	34.16872	132.814	34.1516	141.929	34.25817	137.8878	34.18622	138.4612	34.3182	141.0717
34.26463	115.2119	34.18538	132.8141	34.16827	141.9284	34.27483	137.8884	34.20288	138.4604	34.33487	141.0731
34.2813	115.2072	34.20205	132.8088	34.18493	141.9269	34.2915	137.8866	34.21955	138.4591	34.35153	141.0698
34.29797	115.2039	34.21872	132.8109	34.2016	141.9242	34.30817	137.8861	34.23622	138.4564	34.3682	141.07
34.31463	115.2006	34.23538	132.8071	34.21827	141.9245	34.32483	137.8846	34.25288	138.4575	34.38487	141.0697
34.3313	115.1972	34.25205	132.8067	34.23493	141.9223	34.3415	137.8849	34.26955	138.4548	34.40153	141.0691
34.34797	115.194	34.26872	132.8045	34.2516	141.9245	34.35817	137.8814	34.28622	138.4535	34.4182	141.0654
34.36463	115.1916	34.28538	132.8035	34.26827	141.9203	34.37483	137.8804	34.30288	138.4527	34.43487	141.0648
34.3813	115.1884	34.30205	132.7999	34.28493	141.9206	34.3915	137.882	34.31955	138.4538	34.45153	141.065
34.39797	115.1844	34.31872	132.8005	34.3016	141.914	34.40817	137.8766	34.33622	138.4501	34.4682	141.0624
34.41463	115.181	34.33538	132.7996	34.31827	141.9154	34.42483	137.8771	34.35288	138.448	34.48487	141.0615
34.4313	115.1773	34.35205	132.7971	34.33493	141.9128	34.4415	137.8755	34.36955	138.4467	34.50153	141.063
34.44797	115.1785	34.36872	132.7955	34.3516	141.9114	34.45817	137.8762	34.38622	138.4454	34.5182	141.0591
34.46463	115.1726	34.38538	132.7966	34.36827	141.9107	34.47483	137.8722	34.40288	138.4433	34.53487	141.0574
34.4813	115.1691	34.40205	132.793	34.38493	141.908	34.4915	137.8723	34.41955	138.4445	34.55153	141.0579
34.49797	115.1649	34.41872	132.7901	34.4016	141.9072	34.50817	137.8713	34.43622	138.4414	34.5682	141.0568
34.51463	115.163	34.43538	132.7888	34.41827	141.9054	34.52483	137.8701	34.45288	138.4402	34.58487	141.0551
34.5313	115.1608	34.45205	132.7917	34.43493	141.9026	34.5415	137.8704	34.46955	138.4381	34.60153	141.0543
34.54797	115.156	34.46872	132.7863	34.4516	141.9012	34.55817	137.8687	34.48622	138.4383	34.6182	141.0533
34.56463	115.1536	34.48538	132.7862	34.46827	141.8993	34.57483	137.8671	34.50288	138.438	34.63487	141.0519
34.5813	115.1507	34.50205	132.7813	34.48493	141.8949	34.5915	137.8676	34.51955	138.4347	34.65153	141.0497
34.59797	115.1462	34.51872	132.7829	34.5016	141.8982	34.60817	137.8651	34.53622	138.4314	34.6682	141.0502
34.61463	115.1467	34.53538	132.7782	34.51827	141.8959	34.62483	137.8666	34.55288	138.4307	34.68487	141.0494
34.6313	115.1416	34.55205	132.7757	34.53493	141.8929	34.6415	137.8641	34.56955	138.4297	34.70153	141.0479
34.64797	115.1374	34.56872	132.7769	34.5516	141.8894	34.65817	137.8625	34.58622	138.4264	34.7182	141.0486
34.66463	115.135	34.58538	132.7731	34.56827	141.8915	34.67483	137.8625	34.60288	138.4255	34.73487	141.047
34.6813	115.1302	34.60205	132.7734	34.58493	141.8886	34.6915	137.8623	34.61955	138.4252	34.75153	141.0451
34.69797	115.1253	34.61872	132.7712	34.6016	141.8859	34.70817	137.858	34.63622	138.4257	34.7682	141.0458
34.71463	115.1241	34.63538	132.7688	34.61827	141.8838	34.72483	137.8579	34.65288	138.4236	34.78487	141.0458
34.7313	115.1191	34.65205	132.7667	34.63493	141.8843	34.7415	137.8531	34.66955	138.4213	34.80153	141.0418
34.74797	115.1151	34.66872	132.7623	34.6516	141.8794	34.75817	137.8559	34.68622	138.4206	34.8182	141.0413
34.76463	115.1142	34.68538	132.764	34.66827	141.8778	34.77483	137.8556	34.70288	138.4186	34.83487	141.0438
34.7813	115.1119	34.70205	132.7602	34.68493	141.8767	34.7915	137.8541	34.71955	138.4184	34.85153	141.0385
34.79797	115.1076	34.71872	132.7582	34.7016	141.8766	34.80817	137.8497	34.73622	138.4189	34.8682	141.0386
34.81463	115.1054	34.73538	132.7572	34.71827	141.8744	34.82483	137.8509	34.75288	138.4157	34.88487	141.0376
34.8313	115.1005	34.75205	132.7556	34.73493	141.8711	34.8415	137.8475	34.76955	138.4167	34.90153	141.0378
34.84797	115.0979	34.76872	132.7538	34.7516	141.8735	34.85817	137.8496	34.78622	138.4169	34.9182	141.0338
34.86463	115.0948	34.78538	132.7539	34.76827	141.8683	34.87483	137.8473	34.80288	138.4104	34.93487	141.0339
34.8813	115.0943	34.80205	132.7502	34.78493	141.8673	34.8915	137.8487	34.81955	138.4113	34.95153	141.0345
34.89797	115.0897	34.81872	132.7499	34.8016	141.8646	34.90817	137.8453	34.83622	138.4204	34.9682	141.0308
34.91463	115.0884	34.83538	132.7478	34.81827	141.8634	34.92483	137.8451	34.85288	138.4115	34.98487	141.0313
34.9313	115.0816	34.85205	132.7465	34.83493	141.8583	34.9415	137.8413	34.86955	138.4079	35.00153	141.0289
34.94797	115.082	34.86872	132.7459	34.8516	141.8596	34.95817	137.8412	34.88622	138.4042	35.0182	141.0288
34.96463	115.0784	34.88538	132.7426	34.86827	141.8569	34.97483	137.8396	34.90288	138.4099	35.03487	141.025
34.9813	115.0732	34.90205	132.7414	34.88493	141.8552	34.9915	137.8402	34.91955	138.4033	35.05153	141.027
34.99797	115.0713	34.91872	132.7394	34.9016	141.8529	35.00817	137.8401	34.93622	138.4036	35.0682	141.0251
35.01463	115.0693	34.93538	132.7376	34.91827	141.8528	35.02483	137.84	34.95288	138.406	35.08487	141.0225
35.0313	115.0637	34.95205	132.7364	34.93493	141.8529	35.0415	137.8356	34.96955	138.4067	35.10153	141.0247
35.04797	115.0614	34.96872	132.7351	34.9516	141.8502	35.05817	137.8358	34.98622	138.4025	35.1182	141.0209
35.06463	115.0595	34.98538	132.733	34.96827	141.8476	35.07483	137.8351	35.00288	138.398	35.13487	141.0209
35.0813	115.0547	35.00205	132.7317	34.98493	141.8458	35.0915	137.8362	35.01955	138.401	35.15153	141.0178
35.09797	115.0519	35.01872	132.7326	35.0016	141.847	35.10817	137.8343	35.03622	138.3973	35.1682	141.0184
35.11463	115.0521	35.03538	132.7301	35.01827	141.8416	35.12483	137.8312	35.05288	138.3971	35.18487	141.0141
35.1313	115.0446	35.05205	132.7294	35.03493	141.8407	35.1415	137.8313	35.06955	138.3967	35.20153	141.0159
35.14797	115.042	35.06872	132.7246	35.0516	141.8405	35.15817	137.8305	35.08622	138.395	35.2182	141.0153
35.16463	115.0436	35.08538	132.723	35.06827	141.8388	35.17483	137.8303	35.10288	138.3893	35.23487	141.0125
35.1813	115.0381	35.10205	132.7234	35.08493	141.8369	35.1915	137.8303	35.11955	138.3911	35.25153	141.0112
35.19797	115.0349	35.11872	132.7241	35.1016	141.8342	35.20817	137.8306	35.13622	138.3891	35.2682	141.0116
35.21463	115.0326	35.13538	132.7176	35.11827	141.8327	35.22483	137.8264	35.15288	138.3893	35.28487	141.0097
35.2313	115.0292	35.15205	132.7162	35.13493	141.8327	35.2415	137.8253	35.16955	138.3881	35.30153	141.0111
35.24797	115.0244	35.16872	132.7188	35.1516	141.8309	35.25817	137.8256	35.18622	138.3892	35.3182	141.0061
35.26463	115.021	35.18538	132.7165	35.16827	141.8321	35.27483	137.824	35.20288	138.3849	35.33487	141.0066
35.2813	115.0181	35.20205	132.7132	35.18493	141.8276	35.2915	137.8221	35.21955	138.3851	35.35153	141.0057
35.29797	115.0157	35.21872	132.7114	35.2016	141.8267	35.30817	137.8204	35.23622	138.3831	35.3682	141.0069
35.31463	115.0117	35.23538	132.7103	35.21827	141.8269	35.32483	137.821	35.25288	138.3809	35.38487	141.0039
35.3313	115.0067	35.25205	132.7096	35.23493	141.826	35.3415	137.8182	35.26955	138.3795	35.40153	141.0016
35.34797	115.0066	35.26872	132.7048	35.2516	141.8246	35.35817	137.8192	35.28622	138.3795	35.4182	141.0029
35.36463	115.0056	35.28538	132.7034	35.26827	141.8247	35.37483	137.8188	35.30288	138.379	35.43487	141.0001
35.3813	114.9998	35.30205	132.7024	35.28493	141.8224	35.3915	137.8157	35.31955	138.3761	35.45153	140.9982
35.39797	114.9949	35.31872	132.7067	35.3016	141.8226	35.40817	137.811	35.33622	138.3744	35.4682	140.9993
35.41463	114.9926	35.33538	132.7034	35.31827	141.8201	35.42483	137.8139	35.35288	138.3723	35.48487	140.9981
35.4313	114.9886	35.35205	132.6959	35.33493	141.8196	35.4415	137.8125	35.36955	138.3716	35.50153	140.9965
35.44797	114.9848	35.36872	132.6944	35.3516	141.8168	35.45817	137.8099	35.38622	138.3703	35.5182	140.9952
35.46463	114.9844	35.38538	132.6959	35.36827	141.8133	35.47483	137.8077	35.40288	138.3677	35.53487	140.9959
35.4813	114.9795	35.40205	132.6952	35.38493	141.813	35.4915	137.8088	35.41955	138.3681	35.55153	140.9903
35.49797	114.9793	35.41872	132.6897	35.4016	141.8123	35.50817	137.8066	35.43622	138.3672	35.5682	140.9949
35.51463	114.9716	35.43538	132.6927	35.41827	141.808	35.52483	137.8058	35.45288	138.3646	35.58487	140.9894
35.5313	114.9685	35.45205	132.6862	35.43493	141.8068	35.5415	137.8024	35.46955	138.366	35.60153	140.9914
35.54797	114.9664	35.46872	132.6885	35.4516	141.807	35.55817	137.8043	35.48622	138.3622	35.6182	140.9873
35.56463	114.9613	35.48538	132.6865	35.46827	141.8036	35.57483	137.803	35.50288	138.3634	35.63487	140.9875
35.5813	114.9638	35.50205	132.6816	35.48493	141.8023	35.5915	137.8038	35.51955	138.3633	35.65153	140.9888
35.59797	114.9547	35.51872	132.6841	35.5016	141.7996	35.60817	137.8004	35.53622	138.3617	35.6682	140.9859
35.61463	114.9532	35.53538	132.6802	35.51827	141.8001	35.62483	137.7988	35.55288	138.3582	35.68487	140.9863

35.6313	114.9501	35.55205	132.6805	35.53493	141.7952	35.6415	137.7996	35.56955	138.3566	35.70153	140.9834
35.64797	114.9437	35.56872	132.6816	35.5516	141.7963	35.65817	137.797	35.58622	138.3593	35.7182	140.9825
35.66463	114.9414	35.58538	132.6777	35.56827	141.794	35.67483	137.7971	35.60288	138.3561	35.73487	140.9822
35.6813	114.94	35.60205	132.6726	35.58493	141.7937	35.6915	137.7959	35.61955	138.3562	35.75153	140.9821
35.69797	114.9373	35.61872	132.6759	35.6016	141.7908	35.70817	137.7951	35.63622	138.3525	35.7682	140.9804
35.71463	114.9337	35.63538	132.6736	35.61827	141.7901	35.72483	137.7966	35.65288	138.3522	35.78487	140.9804
35.7313	114.9291	35.65205	132.6679	35.63493	141.7884	35.7415	137.794	35.66955	138.351	35.80153	140.9807
35.74797	114.9265	35.66872	132.6698	35.6516	141.7889	35.75817	137.7935	35.68622	138.3515	35.8182	140.9775
35.76463	114.9231	35.68538	132.6676	35.66827	141.7853	35.77483	137.7916	35.70288	138.3498	35.83487	140.976
35.7813	114.9206	35.70205	132.6633	35.68493	141.7825	35.7915	137.7929	35.71955	138.3469	35.85153	140.9768
35.79797	114.9187	35.71872	132.6643	35.7016	141.7803	35.80817	137.7913	35.73622	138.348	35.8682	140.9746
35.81463	114.9153	35.73538	132.6611	35.71827	141.7803	35.82483	137.7901	35.75288	138.3471	35.88487	140.974
35.8313	114.9096	35.75205	132.6605	35.73493	141.7783	35.8415	137.7885	35.76955	138.3418	35.90153	140.9731
35.84797	114.9089	35.76872	132.6597	35.7516	141.7786	35.85817	137.7896	35.78622	138.344	35.9182	140.9725
35.86463	114.9026	35.78538	132.6603	35.76827	141.7743	35.87483	137.7867	35.80288	138.3431	35.93487	140.9697
35.8813	114.8997	35.80205	132.6586	35.78493	141.7736	35.8915	137.7865	35.81955	138.34	35.95153	140.9695
35.89797	114.8972	35.81872	132.6577	35.8016	141.7725	35.90817	137.7864	35.83622	138.3393	35.9682	140.9691
35.91463	114.8952	35.83538	132.6542	35.81827	141.7704	35.92483	137.7827	35.85288	138.339	35.98487	140.966
35.9313	114.8901	35.85205	132.6565	35.83493	141.768	35.9415	137.7815	35.86955	138.3358	36.00153	140.9682
35.94797	114.8869	35.86872	132.6549	35.8516	141.7691	35.95817	137.7834	35.88622	138.3357	36.0182	140.9665
35.96463	114.8837	35.88538	132.6525	35.86827	141.7678	35.97483	137.7785	35.90288	138.3349	36.03487	140.9676
35.9813	114.8805	35.90205	132.6536	35.88493	141.7659	35.9915	137.778	35.91955	138.3325	36.05153	140.9642
35.99797	114.876	35.91872	132.6496	35.9016	141.7632	36.00817	137.7796	35.93622	138.3325	36.0682	140.9641
36.01463	114.8747	35.93538	132.6477	35.91827	141.7629	36.02483	137.7789	35.95288	138.3331	36.08487	140.9621
36.0313	114.8709	35.95205	132.6459	35.93493	141.7621	36.0415	137.7766	35.96955	138.331	36.10153	140.9613
36.04797	114.8677	35.96872	132.6464	35.9516	141.7623	36.05817	137.7774	35.98622	138.3244	36.1182	140.961
36.06463	114.8616	35.98538	132.6464	35.96827	141.7577	36.07483	137.7765	36.00288	138.3253	36.13487	140.9592
36.0813	114.8606	36.00205	132.6416	35.98493	141.7575	36.0915	137.7745	36.01955	138.3276	36.15153	140.9598
36.09797	114.8588	36.01872	132.641	36.0016	141.7558	36.10817	137.7746	36.03622	138.3228	36.1682	140.9578
36.11463	114.8576	36.03538	132.6402	36.01827	141.7538	36.12483	137.7723	36.05288	138.3238	36.18487	140.9565
36.1313	114.8508	36.05205	132.6386	36.03493	141.7534	36.1415	137.7706	36.06955	138.3222	36.20153	140.9559
36.14797	114.8475	36.06872	132.635	36.0516	141.7519	36.15817	137.7685	36.08622	138.3219	36.2182	140.9546
36.16463	114.8445	36.08538	132.6382	36.06827	141.7488	36.17483	137.7697	36.10288	138.3206	36.23487	140.9521
36.1813	114.8393	36.10205	132.6348	36.08493	141.7501	36.1915	137.7709	36.11955	138.3224	36.25153	140.9514
36.19797	114.8384	36.11872	132.634	36.1016	141.7488	36.20817	137.7672	36.13622	138.3174	36.2682	140.9491
36.21463	114.8376	36.13538	132.6318	36.11827	141.7433	36.22483	137.7679	36.15288	138.3205	36.28487	140.9511
36.2313	114.8289	36.15205	132.6296	36.13493	141.7448	36.2415	137.7675	36.16955	138.3202	36.30153	140.9481
36.24797	114.83	36.16872	132.6257	36.1516	141.744	36.25817	137.7642	36.18622	138.3178	36.3182	140.9464
36.26463	114.8242	36.18538	132.6251	36.16827	141.7429	36.27483	137.7642	36.20288	138.3173	36.33487	140.9477
36.2813	114.8199	36.20205	132.6262	36.18493	141.7395	36.2915	137.7637	36.21955	138.3173	36.35153	140.9449
36.29797	114.8208	36.21872	132.6228	36.2016	141.7445	36.30817	137.7627	36.23622	138.3153	36.3682	140.9442
36.31463	114.8131	36.23538	132.6209	36.21827	141.7415	36.32483	137.7601	36.25288	138.3159	36.38487	140.9433
36.3313	114.8136	36.25205	132.6224	36.23493	141.734	36.3415	137.7598	36.26955	138.3141	36.40153	140.9414
36.34797	114.8055	36.26872	132.6188	36.2516	141.7375	36.35817	137.7581	36.28622	138.3131	36.4182	140.9395
36.36463	114.8055	36.28538	132.6212	36.26827	141.7378	36.37483	137.7581	36.30288	138.3133	36.43487	140.9404
36.3813	114.8058	36.30205	132.6156	36.28493	141.7294	36.3915	137.7582	36.31955	138.3122	36.45153	140.9397
36.39797	114.7986	36.31872	132.6148	36.3016	141.733	36.40817	137.7549	36.33622	138.313	36.4682	140.9377
36.41463	114.7953	36.33538	132.6168	36.31827	141.7299	36.42483	137.7532	36.35288	138.3113	36.48487	140.9376
36.4313	114.7942	36.35205	132.6121	36.33493	141.7331	36.4415	137.7521	36.36955	138.3099	36.50153	140.9368
36.44797	114.7892	36.36872	132.6129	36.3516	141.7306	36.45817	137.7541	36.38622	138.3094	36.5182	140.9348
36.46463	114.789	36.38538	132.6103	36.36827	141.7276	36.47483	137.7528	36.40288	138.3081	36.53487	140.9341
36.4813	114.7824	36.40205	132.6095	36.38493	141.7238	36.4915	137.7511	36.41955	138.3073	36.55153	140.9341
36.49797	114.7792	36.41872	132.6081	36.4016	141.725	36.50817	137.7505	36.43622	138.3044	36.5682	140.9329
36.51463	114.7763	36.43538	132.6049	36.41827	141.7238	36.52483	137.7497	36.45288	138.3051	36.58487	140.9318
36.5313	114.7764	36.45205	132.6047	36.43493	141.7196	36.5415	137.7473	36.46955	138.3024	36.60153	140.9321
36.54797	114.7714	36.46872	132.6024	36.4516	141.7204	36.55817	137.7487	36.48622	138.3018	36.6182	140.9307
36.56463	114.7697	36.48538	132.6032	36.46827	141.7167	36.57483	137.7463	36.50288	138.3005	36.63487	140.9287
36.5813	114.7683	36.50205	132.5988	36.48493	141.7174	36.5915	137.7441	36.51955	138.3002	36.65153	140.9296
36.59797	114.762	36.51872	132.6016	36.5016	141.7133	36.60817	137.7455	36.53622	138.2986	36.6682	140.9284
36.61463	114.7629	36.53538	132.5973	36.51827	141.716	36.62483	137.7457	36.55288	138.297	36.68487	140.9279
36.6313	114.7585	36.55205	132.5959	36.53493	141.7115	36.6415	137.741	36.56955	138.2932	36.70153	140.925
36.64797	114.7539	36.56872	132.598	36.5516	141.7112	36.65817	137.7436	36.58622	138.2951	36.7182	140.9272
36.66463	114.7525	36.58538	132.5915	36.56827	141.7106	36.67483	137.7422	36.60288	138.2916	36.73487	140.9242
36.6813	114.7528	36.60205	132.5929	36.58493	141.7091	36.6915	137.7381	36.61955	138.2913	36.75153	140.9256
36.69797	114.7481	36.61872	132.5894	36.6016	141.7059	36.70817	137.7385	36.63622	138.2919	36.7682	140.9239
36.71463	114.7433	36.63538	132.5889	36.61827	141.7065	36.72483	137.7386	36.65288	138.2891	36.78487	140.9221
36.7313	114.7444	36.65205	132.5888	36.63493	141.7028	36.7415	137.7364	36.66955	138.2848	36.80153	140.9228
36.74797	114.7377	36.66872	132.5889	36.6516	141.7039	36.75817	137.7363	36.68622	138.284	36.8182	140.9224
36.76463	114.7336	36.68538	132.5841	36.66827	141.7007	36.77483	137.7336	36.70288	138.2818	36.83487	140.9219
36.7813	114.7344	36.70205	132.583	36.68493	141.6996	36.7915	137.7329	36.71955	138.2837	36.85153	140.9201
36.79797	114.7291	36.71872	132.5856	36.7016	141.6975	36.80817	137.7308	36.73622	138.283	36.8682	140.9206
36.81463	114.7273	36.73538	132.5852	36.71827	141.6944	36.82483	137.7302	36.75288	138.2779	36.88487	140.921
36.8313	114.7224	36.75205	132.5817	36.73493	141.6941	36.8415	137.7292	36.76955	138.2751	36.90153	140.9172
36.84797	114.7212	36.76872	132.5814	36.7516	141.6937	36.85817	137.7258	36.78622	138.2766	36.9182	140.9177
36.86463	114.7179	36.78538	132.5792	36.76827	141.6909	36.87483	137.7271	36.80288	138.2742	36.93487	140.9158
36.8813	114.7172	36.80205	132.5809	36.78493	141.6916	36.8915	137.724	36.81955	138.2728	36.95153	140.9165
36.89797	114.7127	36.81872	132.5794	36.8016	141.6891	36.90817	137.7243	36.83622	138.2698	36.9682	140.9152
36.91463	114.7086	36.83538	132.5772	36.81827	141.6884	36.92483	137.7227	36.85288	138.2698	36.98487	140.9137
36.9313	114.709	36.85205	132.5765	36.83493	141.6848	36.9415	137.7205	36.86955	138.2666	37.00153	140.9138
36.94797	114.7043	36.86872	132.5743	36.8516	141.6864	36.95817	137.7203	36.88622	138.2656	37.0182	140.9112
36.96463	114.7003	36.88538	132.5744	36.86827	141.6823	36.97483	137.7195	36.90288	138.2646	37.03487	140.9112
36.9813	114.698	36.90205	132.5734	36.88493	141.6829	36.9915	137.7189	36.91955	138.262	37.05153	140.9087
36.99797	114.6956	36.91872	132.5728	36.9016	141.6801	37.00817	137.7157	36.93622	138.2611	37.0682	140.9095
37.01463	114.6914	36.93538	132.5718	36.91827	141.6805	37.02483	137.7137	36.95288	138.2629	37.08487	140.9065
37.0313	114.6854	36.95205	132.5711	36.93493	141.6797	37.0415	137.7115	36.96955	138.2574	37.10153	140.906
37.04797	114.6855	36.96872	132.5679	36.9516	141.6773	37.05817	137.7129	36.98622	138.2559	37.1182	140.9044
37.06463	114.6808	36.98538	132.5676	36.96827	141.6749	37.07483	137.7084	37.00288	138.2566	37.13487	140.9028
37.0813	114.6801	37.00205	132.5663	36.98493	141.6718	37.0915	137.7068	37.01955	138.2532	37.15153	140.9028
37.09797	114.6772	37.01872	132.5627	37.0016	141.6731	37.10817	137.7066	37.03622	138.2539	37.1682	140.9008
37.11463	114.6745	37.03538	132.5645	37.01827	141.6705	37.12483	137.7063	37.05288	138.2537	37.18487	140.9013
37.1313	114.672	37.05205	132.5618	37.03493	141.6683	37.1415	137.7065	37.06955	138.2525	37.20153	140.898
37.14797	114.6716	37.06872	132.5583	37.0516	141.6688	37.15817	137.7024	37.08622	138.2504	37.2182	140.8984
37.16463	114.6658	37.08538	132.5605	37.06827	141.6654	37.17483	137.7033	37.10288	138.2529	37.23487	140.8983
37.1813	114.6632	37.10205	132.5597	37.08493	141.6653	37.1915	137.7034	37.11955	138.246	37.25153	140.8932
37.19797	114.6612	37.11872	132.5544	37.1016	141.6609	37.20817	137.7015	37.13622	138.2461	37.2682	140.8965
37.21463	114.6553	37.13538	132.5558	37.11827	141.6621	37.22483	137.6993	37.15288	138.2435	37.28487	140.8948
37.2313	114.6549	37.15205	132.5553	37.13493	141.6602	37.2415	137.6967	37.16955	138.2405	37.30153	140.8923

37.24797	114.6545	37.16872	132.5554	37.1516	141.6565	37.25817	137.6953	37.18622	138.2414	37.3182	140.8925
37.26463	114.647	37.18538	132.5508	37.16827	141.6574	37.27483	137.6969	37.20288	138.239	37.33487	140.8901
37.2813	114.6467	37.20205	132.5508	37.18493	141.657	37.2915	137.6986	37.21955	138.2375	37.35153	140.8868
37.29797	114.6451	37.21872	132.5499	37.2016	141.6537	37.30817	137.6943	37.23622	138.2352	37.3682	140.8838
37.31463	114.6401	37.23538	132.5497	37.21827	141.6522	37.32483	137.694	37.25288	138.2347	37.38487	140.888
37.3313	114.6375	37.25205	132.548	37.23493	141.6529	37.3415	137.6927	37.26955	138.2343	37.40153	140.8836
37.34797	114.6346	37.26872	132.547	37.2516	141.6493	37.35817	137.6915	37.28622	138.233	37.4182	140.8855
37.36463	114.6368	37.28538	132.5504	37.26827	141.6496	37.37483	137.6906	37.30288	138.2315	37.43487	140.8858
37.3813	114.6284	37.30205	132.5481	37.28493	141.6478	37.3915	137.6936	37.31955	138.2294	37.45153	140.8845
37.39797	114.6258	37.31872	132.5459	37.3016	141.6453	37.40817	137.6897	37.33622	138.2278	37.4682	140.8785
37.41463	114.6265	37.33538	132.546	37.31827	141.645	37.42483	137.6887	37.35288	138.2277	37.48487	140.878
37.4313	114.6201	37.35205	132.5428	37.33493	141.6451	37.4415	137.6867	37.36955	138.2244	37.50153	140.8788
37.44797	114.6158	37.36872	132.5437	37.3516	141.6414	37.45817	137.6864	37.38622	138.2242	37.5182	140.8771
37.46463	114.614	37.38538	132.5406	37.36827	141.639	37.47483	137.6856	37.40288	138.2218	37.53487	140.876
37.4813	114.6128	37.40205	132.5456	37.38493	141.6379	37.4915	137.6804	37.41955	138.2208	37.55153	140.874
37.49797	114.6067	37.41872	132.5415	37.4016	141.6395	37.50817	137.6813	37.43622	138.2182	37.5682	140.8724
37.51463	114.6041	37.43538	132.5402	37.41827	141.636	37.52483	137.6832	37.45288	138.216	37.58487	140.8729
37.5313	114.6018	37.45205	132.5391	37.43493	141.6347	37.5415	137.6805	37.46955	138.2153	37.60153	140.8702
37.54797	114.5994	37.46872	132.5364	37.4516	141.6344	37.55817	137.6791	37.48622	138.2152	37.6182	140.8698
37.56463	114.597	37.48538	132.5382	37.46827	141.6333	37.57483	137.6812	37.50288	138.2131	37.63487	140.8706
37.5813	114.5936	37.50205	132.534	37.48493	141.6293	37.5915	137.6774	37.51955	138.2106	37.65153	140.8712
37.59797	114.5862	37.51872	132.5306	37.5016	141.6292	37.60817	137.679	37.53622	138.21	37.6682	140.8666
37.61463	114.5859	37.53538	132.5344	37.51827	141.6237	37.62483	137.6731	37.55288	138.2093	37.68487	140.8684
37.6313	114.5817	37.55205	132.5363	37.53493	141.6253	37.6415	137.6719	37.56955	138.2077	37.70153	140.8659
37.64797	114.583	37.56872	132.5283	37.5516	141.6238	37.65817	137.6736	37.58622	138.2063	37.7182	140.8645
37.66463	114.5755	37.58538	132.5287	37.56827	141.6212	37.67483	137.6745	37.60288	138.2034	37.73487	140.8614
37.6813	114.575	37.60205	132.5295	37.58493	141.6191	37.6915	137.6703	37.61955	138.2037	37.75153	140.8641
37.69797	114.5716	37.61872	132.5274	37.6016	141.6167	37.70817	137.6699	37.63622	138.2012	37.7682	140.8626
37.71463	114.5664	37.63538	132.5256	37.61827	141.6156	37.72483	137.6655	37.65288	138.1985	37.78487	140.8621
37.7313	114.5642	37.65205	132.525	37.63493	141.6149	37.7415	137.6635	37.66955	138.2004	37.80153	140.8587
37.74797	114.5627	37.66872	132.521	37.6516	141.6125	37.75817	137.665	37.68622	138.1999	37.8182	140.8604
37.76463	114.5587	37.68538	132.5239	37.66827	141.61	37.77483	137.6623	37.70288	138.1973	37.83487	140.8586
37.7813	114.5561	37.70205	132.5218	37.68493	141.6091	37.7915	137.6585	37.71955	138.1972	37.85153	140.8549
37.79797	114.5525	37.71872	132.5232	37.7016	141.6101	37.80817	137.6579	37.73622	138.1946	37.8682	140.8551
37.81463	114.5526	37.73538	132.5195	37.71827	141.6076	37.82483	137.6587	37.75288	138.1939	37.88487	140.8555
37.8313	114.5491	37.75205	132.5177	37.73493	141.6053	37.8415	137.657	37.76955	138.1931	37.90153	140.8533
37.84797	114.5458	37.76872	132.517	37.7516	141.6036	37.85817	137.6569	37.78622	138.1935	37.9182	140.8524
37.86463	114.5428	37.78538	132.5174	37.76827	141.6034	37.87483	137.6578	37.80288	138.1906	37.93487	140.85
37.8813	114.5404	37.80205	132.5173	37.78493	141.6024	37.8915	137.6559	37.81955	138.1886	37.95153	140.8517
37.89797	114.5347	37.81872	132.5165	37.8016	141.5996	37.90817	137.6537	37.83622	138.1854	37.9682	140.8494
37.91463	114.5318	37.83538	132.5112	37.81827	141.5981	37.92483	137.6535	37.85288	138.1838	37.98487	140.8476
37.9313	114.53	37.85205	132.5128	37.83493	141.5989	37.9415	137.6521	37.86955	138.1855	38.00153	140.8451
37.94797	114.5268	37.86872	132.5096	37.8516	141.5964	37.95817	137.6548	37.88622	138.1853	38.0182	140.8458
37.96463	114.5244	37.88538	132.5115	37.86827	141.5938	37.97483	137.6517	37.90288	138.183	38.03487	140.8445
37.9813	114.5189	37.90205	132.513	37.88493	141.5899	37.9915	137.6495	37.91955	138.1798	38.05153	140.8422
37.99797	114.5224	37.91872	132.5101	37.9016	141.593	38.00817	137.6494	37.93622	138.1797	38.0682	140.8409
38.01463	114.5137	37.93538	132.5106	37.91827	141.5936	38.02483	137.6489	37.95288	138.178	38.08487	140.8443
38.0313	114.5108	37.95205	132.51	37.93493	141.5905	38.0415	137.648	37.96955	138.1769	38.10153	140.8392
38.04797	114.5077	37.96872	132.5082	37.9516	141.5874	38.05817	137.6488	37.98622	138.176	38.1182	140.838
38.06463	114.5065	37.98538	132.5113	37.96827	141.5856	38.07483	137.6474	38.00288	138.1754	38.13487	140.8375
38.0813	114.5031	38.00205	132.5099	37.98493	141.5848	38.0915	137.6447	38.01955	138.1742	38.15153	140.836
38.09797	114.501	38.01872	132.5079	38.0016	141.5862	38.10817	137.6428	38.03622	138.1738	38.1682	140.8346
38.11463	114.4992	38.03538	132.5075	38.01827	141.5846	38.12483	137.6429	38.05288	138.1701	38.18487	140.8327
38.1313	114.494	38.05205	132.5061	38.03493	141.5804	38.1415	137.6406	38.06955	138.1679	38.20153	140.8311
38.14797	114.4921	38.06872	132.5075	38.0516	141.5798	38.15817	137.6399	38.08622	138.1676	38.2182	140.8296
38.16463	114.4879	38.08538	132.5034	38.06827	141.5778	38.17483	137.6384	38.10288	138.1696	38.23487	140.8294
38.1813	114.4865	38.10205	132.5039	38.08493	141.576	38.1915	137.6386	38.11955	138.1697	38.25153	140.8283
38.19797	114.4824	38.11872	132.501	38.1016	141.5768	38.20817	137.6365	38.13622	138.1665	38.2682	140.8283
38.21463	114.478	38.13538	132.5033	38.11827	141.5749	38.22483	137.6357	38.15288	138.1642	38.28487	140.8273
38.2313	114.4769	38.15205	132.5022	38.13493	141.571	38.2415	137.6352	38.16955	138.1645	38.30153	140.828
38.24797	114.473	38.16872	132.5024	38.1516	141.5705	38.25817	137.6347	38.18622	138.1623	38.3182	140.8229
38.26463	114.4724	38.18538	132.5016	38.16827	141.5682	38.27483	137.6319	38.20288	138.1649	38.33487	140.8242
38.2813	114.4687	38.20205	132.5035	38.18493	141.5684	38.2915	137.6323	38.21955	138.1609	38.35153	140.8237
38.29797	114.4645	38.21872	132.5022	38.2016	141.5651	38.30817	137.6333	38.23622	138.1617	38.3682	140.8182
38.31463	114.4616	38.23538	132.4974	38.21827	141.5645	38.32483	137.6279	38.25288	138.1602	38.38487	140.8198
38.3313	114.4599	38.25205	132.4969	38.23493	141.5624	38.3415	137.6283	38.26955	138.1579	38.40153	140.8184
38.34797	114.4582	38.26872	132.4978	38.2516	141.5581	38.35817	137.6283	38.28622	138.159	38.4182	140.8168
38.36463	114.4525	38.28538	132.4985	38.26827	141.5574	38.37483	137.6302	38.30288	138.1559	38.43487	140.815
38.3813	114.4509	38.30205	132.4966	38.28493	141.556	38.3915	137.6277	38.31955	138.1569	38.45153	140.8138
38.39797	114.4473	38.31872	132.4918	38.3016	141.5573	38.40817	137.6254	38.33622	138.1539	38.4682	140.8143
38.41463	114.4481	38.33538	132.4931	38.31827	141.556	38.42483	137.6258	38.35288	138.1534	38.48487	140.8134
38.4313	114.4435	38.35205	132.4921	38.33493	141.5551	38.4415	137.6252	38.36955	138.1526	38.50153	140.8112
38.44797	114.4398	38.36872	132.4905	38.3516	141.553	38.45817	137.6248	38.38622	138.1544	38.5182	140.8104
38.46463	114.4346	38.38538	132.491	38.36827	141.5488	38.47483	137.6267	38.40288	138.1512	38.53487	140.8097
38.4813	114.4316	38.40205	132.4897	38.38493	141.5498	38.4915	137.6265	38.41955	138.1515	38.55153	140.8078
38.49797	114.4346	38.41872	132.4886	38.4016	141.5485	38.50817	137.6254	38.43622	138.1475	38.5682	140.808
38.51463	114.4326	38.43538	132.4922	38.41827	141.5468	38.52483	137.6219	38.45288	138.1437	38.58487	140.805
38.5313	114.4255	38.45205	132.4874	38.43493	141.5443	38.5415	137.621	38.46955	138.1447	38.60153	140.8076
38.54797	114.4251	38.46872	132.4853	38.4516	141.5451	38.55817	137.6228	38.48622	138.1445	38.6182	140.804
38.56463	114.4188	38.48538	132.4857	38.46827	141.5451	38.57483	137.619	38.50288	138.1399	38.63487	140.8045
38.5813	114.4166	38.50205	132.4864	38.48493	141.5439	38.5915	137.6162	38.51955	138.1412	38.65153	140.803
38.59797	114.4177	38.51872	132.4871	38.5016	141.5401	38.60817	137.6146	38.53622	138.1397	38.6682	140.8018
38.61463	114.4143	38.53538	132.4857	38.51827	141.5392	38.62483	137.6137	38.55288	138.1395	38.68487	140.7988
38.6313	114.4087	38.55205	132.482	38.53493	141.5396	38.6415	137.6131	38.56955	138.1357	38.70153	140.7989
38.64797	114.4072	38.56872	132.481	38.5516	141.5376	38.65817	137.6097	38.58622	138.1352	38.7182	140.8003
38.66463	114.4037	38.58538	132.4786	38.56827	141.5375	38.67483	137.6112	38.60288	138.1342	38.73487	140.7987
38.6813	114.3981	38.60205	132.4833	38.58493	141.535	38.6915	137.6064	38.61955	138.1358	38.75153	140.7962
38.69797	114.3985	38.61872	132.4805	38.6016	141.5336	38.70817	137.6064	38.63622	138.1287	38.7682	140.7969
38.71463	114.3956	38.63538	132.4783	38.61827	141.5329	38.72483	137.6044	38.65288	138.1299	38.78487	140.7963
38.7313	114.3939	38.65205	132.4789	38.63493	141.5289	38.7415	137.6019	38.66955	138.1293	38.80153	140.7932
38.74797	114.3878	38.66872	132.479	38.6516	141.53	38.75817	137.6001	38.68622	138.129	38.8182	140.7922
38.76463	114.3869	38.68538	132.4786	38.66827	141.5262	38.77483	137.5986	38.70288	138.1271	38.83487	140.7932
38.7813	114.3821	38.70205	132.4761	38.68493	141.5267	38.7915	137.5947	38.71955	138.1259	38.85153	140.7915
38.79797	114.3792	38.71872	132.4765	38.7016	141.5248	38.80817	137.5978	38.73622	138.1237	38.8682	140.7899
38.81463	114.3762	38.73538	132.4729	38.71827	141.5215	38.82483	137.5925	38.75288	138.1228	38.88487	140.7889
38.8313	114.3693	38.75205	132.4744	38.73493	141.5212	38.8415	137.5957	38.76955	138.1204	38.90153	140.787
38.84797	114.3745	38.76872	132.474	38.7516	141.5215	38.85817	137.5902	38.78622	138.1211	38.9182	140.7859

38.86463	114.3671	38.78538	132.4665	38.76827	141.5221	38.87483	137.5924	38.80288	138.1195	38.93487	140.7832
38.8813	114.3657	38.80205	132.4667	38.78493	141.5195	38.8915	137.5902	38.81955	138.1169	38.95153	140.7829
38.89797	114.3625	38.81872	132.467	38.8016	141.5205	38.90817	137.5892	38.83622	138.1139	38.9682	140.7846
38.91463	114.3601	38.83538	132.4638	38.81827	141.5167	38.92483	137.5858	38.85288	138.1166	38.98487	140.7822
38.9313	114.3547	38.85205	132.4643	38.83493	141.519	38.9415	137.5853	38.86955	138.1129	39.00153	140.7786
38.94797	114.3557	38.86872	132.4631	38.8516	141.5158	38.95817	137.5844	38.88622	138.1122	39.0182	140.7815
38.96463	114.3494	38.88538	132.4618	38.86827	141.517	38.97483	137.5807	38.90288	138.1114	39.03487	140.78
38.9813	114.3432	38.90205	132.4632	38.88493	141.5139	38.9915	137.5794	38.91955	138.109	39.05153	140.7779
38.99797	114.3462	38.91872	132.4568	38.9016	141.5133	39.00817	137.5794	38.93622	138.1093	39.0682	140.777
39.01463	114.3439	38.93538	132.4605	38.91827	141.512	39.02483	137.5799	38.95288	138.1082	39.08487	140.7767
39.0313	114.3401	38.95205	132.4601	38.93493	141.5109	39.0415	137.5806	38.96955	138.106	39.10153	140.7744
39.04797	114.3366	38.96872	132.4603	38.9516	141.5099	39.05817	137.5792	38.98622	138.1059	39.1182	140.7733
39.06463	114.3331	38.98538	132.4547	38.96827	141.5091	39.07483	137.5757	39.00288	138.1033	39.13487	140.7722
39.0813	114.3332	39.00205	132.4567	38.98493	141.5075	39.0915	137.5741	39.01955	138.1004	39.15153	140.7723
39.09797	114.3298	39.01872	132.4572	39.0016	141.5038	39.10817	137.5725	39.03622	138.1018	39.1682	140.7701
39.11463	114.3245	39.03538	132.455	39.01827	141.4978	39.12483	137.5722	39.05288	138.1001	39.18487	140.7708
39.1313	114.3234	39.05205	132.4544	39.03493	141.5012	39.1415	137.5704	39.06955	138.0992	39.20153	140.7691
39.14797	114.3189	39.06872	132.456	39.0516	141.4968	39.15817	137.5681	39.08622	138.099	39.2182	140.7676
39.16463	114.3177	39.08538	132.4547	39.06827	141.4949	39.17483	137.5684	39.10288	138.094	39.23487	140.7666
39.1813	114.3136	39.10205	132.4543	39.08493	141.4932	39.1915	137.5695	39.11955	138.0927	39.25153	140.7685
39.19797	114.3101	39.11872	132.4508	39.1016	141.4979	39.20817	137.566	39.13622	138.0937	39.2682	140.763
39.21463	114.3089	39.13538	132.4521	39.11827	141.4921	39.22483	137.5644	39.15288	138.0928	39.28487	140.7647
39.2313	114.3041	39.15205	132.4518	39.13493	141.49	39.2415	137.565	39.16955	138.091	39.30153	140.7638
39.24797	114.3019	39.16872	132.4516	39.1516	141.4882	39.25817	137.5631	39.18622	138.0917	39.3182	140.762
39.26463	114.3009	39.18538	132.4527	39.16827	141.488	39.27483	137.5633	39.20288	138.0889	39.33487	140.7628
39.2813	114.2954	39.20205	132.4501	39.18493	141.4853	39.2915	137.5611	39.21955	138.0869	39.35153	140.7602
39.29797	114.2913	39.21872	132.4465	39.2016	141.4831	39.30817	137.5617	39.23622	138.0846	39.3682	140.7597
39.31463	114.2897	39.23538	132.449	39.21827	141.48	39.32483	137.5596	39.25288	138.0846	39.38487	140.7585
39.3313	114.2875	39.25205	132.4485	39.23493	141.4792	39.3415	137.5612	39.26955	138.0824	39.40153	140.7568
39.34797	114.2816	39.26872	132.4486	39.2516	141.4756	39.35817	137.5595	39.28622	138.0818	39.4182	140.7579
39.36463	114.2835	39.28538	132.4458	39.26827	141.4763	39.37483	137.5612	39.30288	138.0799	39.43487	140.7533
39.3813	114.2792	39.30205	132.4481	39.28493	141.4765	39.3915	137.5577	39.31955	138.0821	39.45153	140.755
39.39797	114.2774	39.31872	132.4445	39.3016	141.4735	39.40817	137.5577	39.33622	138.08	39.4682	140.7543
39.41463	114.2734	39.33538	132.4459	39.31827	141.4726	39.42483	137.5526	39.35288	138.0778	39.48487	140.754
39.4313	114.2733	39.35205	132.442	39.33493	141.4704	39.4415	137.5529	39.36955	138.076	39.50153	140.753
39.44797	114.2719	39.36872	132.4416	39.3516	141.4686	39.45817	137.5545	39.38622	138.0741	39.5182	140.7536
39.46463	114.2636	39.38538	132.4412	39.36827	141.468	39.47483	137.5505	39.40288	138.0741	39.53487	140.7518
39.4813	114.2655	39.40205	132.4407	39.38493	141.4637	39.4915	137.547	39.41955	138.072	39.55153	140.7499
39.49797	114.2612	39.41872	132.4405	39.4016	141.4662	39.50817	137.5505	39.43622	138.0703	39.5682	140.7467
39.51463	114.2571	39.43538	132.4417	39.41827	141.4669	39.52483	137.55	39.45288	138.0695	39.58487	140.7475
39.5313	114.254	39.45205	132.435	39.43493	141.4624	39.5415	137.5496	39.46955	138.0667	39.60153	140.7459
39.54797	114.2543	39.46872	132.4369	39.4516	141.4609	39.55817	137.547	39.48622	138.0677	39.6182	140.7462
39.56463	114.2489	39.48538	132.4359	39.46827	141.4588	39.57483	137.5475	39.50288	138.0648	39.63487	140.7431
39.5813	114.2449	39.50205	132.4339	39.48493	141.4587	39.5915	137.546	39.51955	138.0668	39.65153	140.7433
39.59797	114.2438	39.51872	132.4296	39.5016	141.4548	39.60817	137.5459	39.53622	138.0652	39.6682	140.7429
39.61463	114.2417	39.53538	132.4331	39.51827	141.4588	39.62483	137.5468	39.55288	138.0623	39.68487	140.745
39.6313	114.2355	39.55205	132.4359	39.53493	141.4529	39.6415	137.5407	39.56955	138.0629	39.70153	140.7441
39.64797	114.2357	39.56872	132.4349	39.5516	141.4511	39.65817	137.5413	39.58622	138.0609	39.7182	140.7394
39.66463	114.2334	39.58538	132.4325	39.56827	141.4497	39.67483	137.5416	39.60288	138.0602	39.73487	140.7396
39.6813	114.2295	39.60205	132.4281	39.58493	141.4488	39.6915	137.5358	39.61955	138.0598	39.75153	140.7399
39.69797	114.2252	39.61872	132.4279	39.6016	141.4459	39.70817	137.5378	39.63622	138.0573	39.7682	140.7372
39.71463	114.2261	39.63538	132.4294	39.61827	141.4428	39.72483	137.5356	39.65288	138.0591	39.78487	140.7382
39.7313	114.2239	39.65205	132.4278	39.63493	141.4436	39.7415	137.5345	39.66955	138.0564	39.80153	140.7378
39.74797	114.2173	39.66872	132.4288	39.6516	141.4408	39.75817	137.5339	39.68622	138.0539	39.8182	140.7385
39.76463	114.2179	39.68538	132.4239	39.66827	141.4407	39.77483	137.5338	39.70288	138.053	39.83487	140.7347
39.7813	114.217	39.70205	132.4232	39.68493	141.4413	39.7915	137.5276	39.71955	138.0535	39.85153	140.7342
39.79797	114.2126	39.71872	132.4246	39.7016	141.4369	39.80817	137.5308	39.73622	138.0492	39.8682	140.7308
39.81463	114.208	39.73538	132.4216	39.71827	141.4387	39.82483	137.5303	39.75288	138.0504	39.88487	140.7319
39.8313	114.2079	39.75205	132.4214	39.73493	141.4354	39.8415	137.5273	39.76955	138.0486	39.90153	140.7311
39.84797	114.2052	39.76872	132.4206	39.7516	141.4315	39.85817	137.5273	39.78622	138.0466	39.9182	140.7307
39.86463	114.202	39.78538	132.4223	39.76827	141.4352	39.87483	137.5262	39.80288	138.0471	39.93487	140.7295
39.8813	114.1976	39.80205	132.419	39.78493	141.4313	39.8915	137.5247	39.81955	138.0447	39.95153	140.7294
39.89797	114.1979	39.81872	132.4187	39.8016	141.4309	39.90817	137.5263	39.83622	138.0436	39.9682	140.7298
39.91463	114.1928	39.83538	132.4193	39.81827	141.4304	39.92483	137.523	39.85288	138.0441	39.98487	140.7283
39.9313	114.1892	39.85205	132.4145	39.83493	141.4289	39.9415	137.5227	39.86955	138.0433	40.00153	140.7276
39.94797	114.1862	39.86872	132.4157	39.8516	141.4289	39.95817	137.5213	39.88622	138.0407	40.0182	140.7267
39.96463	114.1852	39.88538	132.4153	39.86827	141.4278	39.97483	137.5199	39.90288	138.0386	40.03487	140.7254
39.9813	114.1815	39.90205	132.4156	39.88493	141.4237	39.9915	137.5174	39.91955	138.0367	40.05153	140.7258
39.99797	114.1792	39.91872	132.4155	39.9016	141.4243	40.00817	137.5167	39.93622	138.0369	40.0682	140.7223
40.01463	114.1755	39.93538	132.412	39.91827	141.4231	40.02483	137.515	39.95288	138.0366	40.08487	140.7229
40.0313	114.1724	39.95205	132.4119	39.93493	141.4223	40.0415	137.5136	39.96955	138.0326	40.10153	140.7223
40.04797	114.1716	39.96872	132.41	39.9516	141.418	40.05817	137.5139	39.98622	138.0336	40.1182	140.7221
40.06463	114.1681	39.98538	132.4092	39.96827	141.4217	40.07483	137.51	40.00288	138.0289	40.13487	140.7198
40.0813	114.1665	40.00205	132.4042	39.98493	141.4168	40.0915	137.5116	40.01955	138.0283	40.15153	140.7202
40.09797	114.1621	40.01872	132.4065	40.0016	141.4158	40.10817	137.5091	40.03622	138.0252	40.1682	140.7166
40.11463	114.1597	40.03538	132.4079	40.01827	141.414	40.12483	137.5072	40.05288	138.0258	40.18487	140.7189
40.1313	114.1563	40.05205	132.4047	40.03493	141.4129	40.1415	137.5069	40.06955	138.0241	40.20153	140.7161
40.14797	114.1548	40.06872	132.4048	40.0516	141.4096	40.15817	137.5063	40.08622	138.023	40.2182	140.714
40.16463	114.1513	40.08538	132.402	40.06827	141.4137	40.17483	137.5049	40.10288	138.0224	40.23487	140.712
40.1813	114.1486	40.10205	132.4012	40.08493	141.4056	40.1915	137.5039	40.11955	138.0211	40.25153	140.7145
40.19797	114.1479	40.11872	132.4021	40.1016	141.4098	40.20817	137.5037	40.13622	138.0214	40.2682	140.7095
40.21463	114.1448	40.13538	132.402	40.11827	141.4058	40.22483	137.5023	40.15288	138.0203	40.28487	140.7128
40.2313	114.1426	40.15205	132.3995	40.13493	141.4113	40.2415	137.5011	40.16955	138.0181	40.30153	140.7111
40.24797	114.1407	40.16872	132.3989	40.1516	141.4029	40.25817	137.5006	40.18622	138.0165	40.3182	140.7113
40.26463	114.1397	40.18538	132.3943	40.16827	141.4059	40.27483	137.4978	40.20288	138.0142	40.33487	140.7074
40.2813	114.1352	40.20205	132.395	40.18493	141.4003	40.2915	137.4963	40.21955	138.0153	40.35153	140.7089
40.29797	114.132	40.21872	132.3958	40.2016	141.4023	40.30817	137.4987	40.23622	138.0142	40.3682	140.7065
40.31463	114.1313	40.23538	132.3948	40.21827	141.4009	40.32483	137.4963	40.25288	138.0124	40.38487	140.7082
40.3313	114.129	40.25205	132.391	40.23493	141.3989	40.3415	137.4938	40.26955	138.009	40.40153	140.705
40.34797	114.1236	40.26872	132.3937	40.2516	141.3954	40.35817	137.4906	40.28622	138.0103	40.4182	140.7047
40.36463	114.1217	40.28538	132.3892	40.26827	141.3984	40.37483	137.4901	40.30288	138.0075	40.43487	140.7016
40.3813	114.1219	40.30205	132.391	40.28493	141.3951	40.3915	137.4895	40.31955	138.0059	40.45153	140.7034
40.39797	114.1184	40.31872	132.3874	40.3016	141.3912	40.40817	137.4876	40.33622	138.0078	40.4682	140.7015
40.41463	114.1137	40.33538	132.3891	40.31827	141.3898	40.42483	137.4859	40.35288	138.0063	40.48487	140.7037
40.4313	114.1108	40.35205	132.3855	40.33493	141.3899	40.4415	137.4851	40.36955	138.0059	40.50153	140.698
40.44797	114.1055	40.36872	132.3843	40.3516	141.3916	40.45817	137.4855	40.38622	138.0057	40.5182	140.6985
40.46463	114.1045	40.38538	132.3841	40.36827	141.3887	40.47483	137.4835	40.40288	138.0016	40.53487	140.6965

40.4813	114.1016	40.40205	132.3833	40.38493	141.3864	40.4915	137.484	40.41955	138.0013	40.55153	140.6973
40.49797	114.0997	40.41872	132.3822	40.4016	141.3818	40.50817	137.4806	40.43622	138.0016	40.5682	140.6956
40.51463	114.0961	40.43538	132.3794	40.41827	141.3845	40.52483	137.4783	40.45288	137.9972	40.58487	140.6962
40.5313	114.0949	40.45205	132.3775	40.43493	141.3898	40.5415	137.4789	40.46955	137.9976	40.60153	140.6921
40.54797	114.0898	40.46872	132.3789	40.4516	141.3825	40.55817	137.4769	40.48622	137.9943	40.6182	140.6941
40.56463	114.0889	40.48538	132.3769	40.46827	141.3812	40.57483	137.4774	40.50288	137.994	40.63487	140.6934
40.5813	114.0842	40.50205	132.3748	40.48493	141.3801	40.5915	137.4754	40.51955	137.9949	40.65153	140.6907
40.59797	114.0846	40.51872	132.372	40.5016	141.3797	40.60817	137.4712	40.53622	137.9945	40.6682	140.6908
40.61463	114.0797	40.53538	132.3745	40.51827	141.3767	40.62483	137.4707	40.55288	137.9946	40.68487	140.6911
40.6313	114.0776	40.55205	132.374	40.53493	141.3771	40.6415	137.4733	40.56955	137.9922	40.70153	140.6875
40.64797	114.0751	40.56872	132.3731	40.5516	141.3705	40.65817	137.4711	40.58622	137.9911	40.7182	140.6837
40.66463	114.0696	40.58538	132.3721	40.56827	141.3753	40.67483	137.4689	40.60288	137.9881	40.73487	140.6871
40.6813	114.0687	40.60205	132.3685	40.58493	141.3703	40.6915	137.4678	40.61955	137.9851	40.75153	140.6845
40.69797	114.067	40.61872	132.3687	40.6016	141.3687	40.70817	137.4653	40.63622	137.9888	40.7682	140.6832
40.71463	114.0634	40.63538	132.3697	40.61827	141.3682	40.72483	137.4668	40.65288	137.9862	40.78487	140.6834
40.7313	114.0621	40.65205	132.3682	40.63493	141.3683	40.7415	137.4638	40.66955	137.9849	40.80153	140.6793
40.74797	114.0579	40.66872	132.3691	40.6516	141.3666	40.75817	137.462	40.68622	137.9838	40.8182	140.6812
40.76463	114.0544	40.68538	132.3622	40.66827	141.3635	40.77483	137.464	40.70288	137.9831	40.83487	140.6809
40.7813	114.0514	40.70205	132.3622	40.68493	141.3646	40.7915	137.4578	40.71955	137.9809	40.85153	140.6794
40.79797	114.0516	40.71872	132.3622	40.7016	141.3625	40.80817	137.4579	40.73622	137.9796	40.8682	140.6776
40.81463	114.0466	40.73538	132.3624	40.71827	141.3602	40.82483	137.4607	40.75288	137.979	40.88487	140.6781
40.8313	114.0443	40.75205	132.3629	40.73493	141.36	40.8415	137.4568	40.76955	137.9779	40.90153	140.6766
40.84797	114.0418	40.76872	132.3571	40.7516	141.3594	40.85817	137.4553	40.78622	137.9786	40.9182	140.6744
40.86463	114.0403	40.78538	132.3586	40.76827	141.3561	40.87483	137.4538	40.80288	137.9767	40.93487	140.674
40.8813	114.0361	40.80205	132.359	40.78493	141.3563	40.8915	137.4521	40.81955	137.9766	40.95153	140.6752
40.89797	114.0354	40.81872	132.3523	40.8016	141.353	40.90817	137.4544	40.83622	137.9751	40.9682	140.6716
40.91463	114.032	40.83538	132.3567	40.81827	141.3512	40.92483	137.4482	40.85288	137.9737	40.98487	140.6705
40.9313	114.029	40.85205	132.3544	40.83493	141.352	40.9415	137.4485	40.86955	137.972	41.00153	140.6694
40.94797	114.0272	40.86872	132.3537	40.8516	141.3496	40.95817	137.4487	40.88622	137.972	41.0182	140.6695
40.96463	114.0217	40.88538	132.3527	40.86827	141.3482	40.97483	137.4467	40.90288	137.9679	41.03487	140.6676
40.9813	114.0193	40.90205	132.3507	40.88493	141.346	40.9915	137.4464	40.91955	137.9673	41.05153	140.6676
40.99797	114.0192	40.91872	132.3511	40.9016	141.3455	41.00817	137.4441	40.93622	137.9672	41.0682	140.6657
41.01463	114.0133	40.93538	132.3502	40.91827	141.3436	41.02483	137.4411	40.95288	137.9668	41.08487	140.6659
41.0313	114.0135	40.95205	132.3479	40.93493	141.3414	41.0415	137.4414	40.96955	137.9665	41.10153	140.6652
41.04797	114.0107	40.96872	132.3478	40.9516	141.3404	41.05817	137.4425	40.98622	137.9641	41.1182	140.6615
41.06463	114.008	40.98538	132.3465	40.96827	141.3418	41.07483	137.4412	41.00288	137.9629	41.13487	140.6623
41.0813	114.0045	41.00205	132.3433	40.98493	141.341	41.0915	137.4389	41.01955	137.9623	41.15153	140.6602
41.09797	114.0023	41.01872	132.3419	41.0016	141.3369	41.10817	137.4395	41.03622	137.9607	41.1682	140.6596
41.11463	114.0005	41.03538	132.3488	41.01827	141.3368	41.12483	137.4379	41.05288	137.9613	41.18487	140.6576
41.1313	113.9962	41.05205	132.342	41.03493	141.3372	41.1415	137.435	41.06955	137.9596	41.20153	140.6584
41.14797	113.9964	41.06872	132.3383	41.0516	141.3353	41.15817	137.4349	41.08622	137.9584	41.2182	140.6578
41.16463	113.9928	41.08538	132.3389	41.06827	141.3327	41.17483	137.4354	41.10288	137.9567	41.23487	140.6541
41.1813	113.9875	41.10205	132.3378	41.08493	141.33	41.1915	137.4355	41.11955	137.9543	41.25153	140.6557
41.19797	113.9857	41.11872	132.3381	41.1016	141.3307	41.20817	137.4366	41.13622	137.957	41.2682	140.6535
41.21463	113.9831	41.13538	132.3368	41.11827	141.3267	41.22483	137.4306	41.15288	137.955	41.28487	140.6523
41.2313	113.9776	41.15205	132.3362	41.13493	141.3275	41.2415	137.4323	41.16955	137.9531	41.30153	140.6505
41.24797	113.9774	41.16872	132.3376	41.1516	141.3254	41.25817	137.4303	41.18622	137.9543	41.3182	140.6499
41.26463	113.9771	41.18538	132.335	41.16827	141.3236	41.27483	137.4299	41.20288	137.9557	41.33487	140.6474
41.2813	113.9703	41.20205	132.3361	41.18493	141.3209	41.2915	137.426	41.21955	137.9512	41.35153	140.6488
41.29797	113.9688	41.21872	132.3334	41.2016	141.32	41.30817	137.4273	41.23622	137.9511	41.3682	140.647
41.31463	113.9668	41.23538	132.3361	41.21827	141.3191	41.32483	137.4283	41.25288	137.9527	41.38487	140.6458
41.3313	113.9648	41.25205	132.3323	41.23493	141.3161	41.3415	137.4248	41.26955	137.9494	41.40153	140.6439
41.34797	113.9622	41.26872	132.3336	41.2516	141.3168	41.35817	137.4252	41.28622	137.9493	41.4182	140.6458
41.36463	113.9599	41.28538	132.3305	41.26827	141.313	41.37483	137.424	41.30288	137.9495	41.43487	140.6432
41.3813	113.956	41.30205	132.3332	41.28493	141.3135	41.3915	137.42	41.31955	137.9438	41.45153	140.6434
41.39797	113.9556	41.31872	132.3322	41.3016	141.3106	41.40817	137.4206	41.33622	137.9435	41.4682	140.6412
41.41463	113.9505	41.33538	132.3311	41.31827	141.3115	41.42483	137.421	41.35288	137.9449	41.48487	140.6403
41.4313	113.9488	41.35205	132.3282	41.33493	141.3112	41.4415	137.4195	41.36955	137.9447	41.50153	140.6384
41.44797	113.9487	41.36872	132.3276	41.3516	141.3051	41.45817	137.4153	41.38622	137.9439	41.5182	140.639
41.46463	113.9469	41.38538	132.328	41.36827	141.3083	41.47483	137.4197	41.40288	137.9401	41.53487	140.6363
41.4813	113.9406	41.40205	132.3272	41.38493	141.3058	41.4915	137.415	41.41955	137.939	41.55153	140.6375
41.49797	113.938	41.41872	132.3273	41.4016	141.3035	41.50817	137.4156	41.43622	137.9344	41.5682	140.6337
41.51463	113.9351	41.43538	132.3261	41.41827	141.2982	41.52483	137.4141	41.45288	137.9356	41.58487	140.6347
41.5313	113.9342	41.45205	132.3258	41.43493	141.304	41.5415	137.4115	41.46955	137.935	41.60153	140.6313
41.54797	113.9333	41.46872	132.3267	41.4516	141.2983	41.55817	137.4111	41.48622	137.9337	41.6182	140.6338
41.56463	113.929	41.48538	132.3261	41.46827	141.2955	41.57483	137.4113	41.50288	137.9323	41.63487	140.6301
41.5813	113.9274	41.50205	132.325	41.48493	141.2986	41.5915	137.4095	41.51955	137.9306	41.65153	140.6315
41.59797	113.9203	41.51872	132.3226	41.5016	141.2968	41.60817	137.4103	41.53622	137.9265	41.6682	140.6303
41.61463	113.9211	41.53538	132.324	41.51827	141.2927	41.62483	137.4083	41.55288	137.9283	41.68487	140.6291
41.6313	113.9198	41.55205	132.3235	41.53493	141.2934	41.6415	137.4064	41.56955	137.9288	41.70153	140.6256
41.64797	113.9136	41.56872	132.3231	41.5516	141.2956	41.65817	137.4049	41.58622	137.9259	41.7182	140.6257
41.66463	113.9156	41.58538	132.3212	41.56827	141.2911	41.67483	137.4051	41.60288	137.9231	41.73487	140.6247
41.6813	113.9109	41.60205	132.3202	41.58493	141.2891	41.6915	137.4031	41.61955	137.9213	41.75153	140.6242
41.69797	113.9086	41.61872	132.3209	41.6016	141.291	41.70817	137.4017	41.63622	137.922	41.7682	140.6228
41.71463	113.9031	41.63538	132.3236	41.61827	141.289	41.72483	137.4017	41.65288	137.9211	41.78487	140.6218
41.7313	113.9018	41.65205	132.3206	41.63493	141.2856	41.7415	137.399	41.66955	137.921	41.80153	140.6209
41.74797	113.8995	41.66872	132.3225	41.6516	141.2845	41.75817	137.3981	41.68622	137.9181	41.8182	140.6195
41.76463	113.8969	41.68538	132.3189	41.66827	141.2849	41.77483	137.399	41.70288	137.918	41.83487	140.6186
41.7813	113.8948	41.70205	132.3215	41.68493	141.2807	41.7915	137.3945	41.71955	137.9182	41.85153	140.6183
41.79797	113.891	41.71872	132.325	41.7016	141.2778	41.80817	137.3972	41.73622	137.9153	41.8682	140.617
41.81463	113.8916	41.73538	132.3219	41.71827	141.2802	41.82483	137.3958	41.75288	137.9166	41.88487	140.6133
41.8313	113.8886	41.75205	132.3215	41.73493	141.2766	41.8415	137.3925	41.76955	137.9149	41.90153	140.6142
41.84797	113.8827	41.76872	132.3194	41.7516	141.2748	41.85817	137.3923	41.78622	137.9121	41.9182	140.6142
41.86463	113.8843	41.78538	132.3192	41.76827	141.2719	41.87483	137.3911	41.80288	137.9118	41.93487	140.6137
41.8813	113.8814	41.80205	132.3211	41.78493	141.2722	41.8915	137.3899	41.81955	137.9115	41.95153	140.6103
41.89797	113.8803	41.81872	132.3203	41.8016	141.2711	41.90817	137.3891	41.83622	137.9106	41.9682	140.6103
41.91463	113.876	41.83538	132.3201	41.81827	141.2689	41.92483	137.386	41.85288	137.9097	41.98487	140.6097
41.9313	113.8743	41.85205	132.3192	41.83493	141.2694	41.9415	137.3866	41.86955	137.9057	42.00153	140.608
41.94797	113.8717	41.86872	132.3185	41.8516	141.2641	41.95817	137.3844	41.88622	137.9056	42.0182	140.6058
41.96463	113.8698	41.88538	132.3172	41.86827	141.2663	41.97483	137.3849	41.90288	137.9037	42.03487	140.6056
41.9813	113.8677	41.90205	132.3169	41.88493	141.266	41.9915	137.381	41.91955	137.9003	42.05153	140.6067
41.99797	113.8654	41.91872	132.3169	41.9016	141.2657	42.00817	137.3837	41.93622	137.9011	42.0682	140.6046
42.01463	113.8615	41.93538	132.317	41.91827	141.2593	42.02483	137.3818	41.95288	137.8997	42.08487	140.6029
42.0313	113.8617	41.95205	132.3169	41.93493	141.2615	42.0415	137.3816	41.96955	137.899	42.10153	140.6013
42.04797	113.8586	41.96872	132.316	41.9516	141.2585	42.05817	137.3774	41.98622	137.898	42.1182	140.602
42.06463	113.8557	41.98538	132.3152	41.96827	141.2555	42.07483	137.3808	42.00288	137.8957	42.13487	140.6005
42.0813	113.8497	42.00205	132.3136	41.98493	141.2566	42.0915	137.3772	42.01955	137.8948	42.15153	140.5995

42.09797	113.8481	42.01872	132.312	42.0016	141.2548	42.10817	137.3758	42.03622	137.8946	42.1682	140.5979
42.11463	113.8466	42.03538	132.3136	42.01827	141.2514	42.12483	137.3723	42.05288	137.8948	42.18487	140.5973
42.1313	113.8448	42.05205	132.3139	42.03493	141.2496	42.1415	137.3743	42.06955	137.891	42.20153	140.5952
42.14797	113.8414	42.06872	132.3126	42.0516	141.2527	42.15817	137.3736	42.08622	137.8923	42.2182	140.5948
42.16463	113.8416	42.08538	132.3123	42.06827	141.2518	42.17483	137.3718	42.10288	137.8888	42.23487	140.5921
42.1813	113.8378	42.10205	132.3142	42.08493	141.248	42.1915	137.3707	42.11955	137.889	42.25153	140.5928
42.19797	113.8344	42.11872	132.3122	42.1016	141.2487	42.20817	137.3707	42.13622	137.8865	42.2682	140.5915
42.21463	113.8302	42.13538	132.31	42.11827	141.2434	42.22483	137.3703	42.15288	137.8838	42.28487	140.5892
42.2313	113.8275	42.15205	132.3116	42.13493	141.2447	42.2415	137.3671	42.16955	137.8849	42.30153	140.5875
42.24797	113.8276	42.16872	132.3108	42.1516	141.2435	42.25817	137.3647	42.18622	137.8826	42.3182	140.5898
42.26463	113.8241	42.18538	132.3085	42.16827	141.2401	42.27483	137.3659	42.20288	137.882	42.33487	140.588
42.2813	113.8219	42.20205	132.3086	42.18493	141.2406	42.2915	137.3626	42.21955	137.8814	42.35153	140.5851
42.29797	113.8174	42.21872	132.3069	42.2016	141.2397	42.30817	137.3618	42.23622	137.8789	42.3682	140.5853
42.31463	113.8131	42.23538	132.308	42.21827	141.2366	42.32483	137.3617	42.25288	137.8786	42.38487	140.5843
42.3313	113.8137	42.25205	132.3066	42.23493	141.2371	42.3415	137.3608	42.26955	137.8791	42.40153	140.5854
42.34797	113.8095	42.26872	132.3061	42.2516	141.232	42.35817	137.359	42.28622	137.8744	42.4182	140.5831
42.36463	113.8047	42.28538	132.3029	42.26827	141.2353	42.37483	137.3587	42.30288	137.8758	42.43487	140.5816
42.3813	113.8062	42.30205	132.3025	42.28493	141.2336	42.3915	137.3568	42.31955	137.876	42.45153	140.5809
42.39797	113.803	42.31872	132.2998	42.3016	141.2293	42.40817	137.3568	42.33622	137.874	42.4682	140.5831
42.41463	113.7971	42.33538	132.3016	42.31827	141.2281	42.42483	137.3524	42.35288	137.8737	42.48487	140.5795
42.4313	113.7971	42.35205	132.301	42.33493	141.2294	42.4415	137.3525	42.36955	137.8746	42.50153	140.5785
42.44797	113.7961	42.36872	132.3007	42.3516	141.2265	42.45817	137.3526	42.38622	137.871	42.5182	140.5779
42.46463	113.7908	42.38538	132.2988	42.36827	141.2227	42.47483	137.3512	42.40288	137.8708	42.53487	140.5791
42.4813	113.7877	42.40205	132.2991	42.38493	141.2271	42.4915	137.3489	42.41955	137.8703	42.55153	140.5744
42.49797	113.7888	42.41872	132.2949	42.4016	141.2244	42.50817	137.3478	42.43622	137.869	42.5682	140.5767
42.51463	113.7797	42.43538	132.2983	42.41827	141.2236	42.52483	137.3457	42.45288	137.8661	42.58487	140.5746
42.5313	113.7775	42.45205	132.2978	42.43493	141.2227	42.5415	137.3451	42.46955	137.8677	42.60153	140.5751
42.54797	113.7772	42.46872	132.296	42.4516	141.2202	42.55817	137.3426	42.48622	137.865	42.6182	140.5729
42.56463	113.7739	42.48538	132.2962	42.46827	141.2209	42.57483	137.3417	42.50288	137.8646	42.63487	140.5719
42.5813	113.7717	42.50205	132.2936	42.48493	141.2161	42.5915	137.3382	42.51955	137.8657	42.65153	140.5701
42.59797	113.7685	42.51872	132.295	42.5016	141.2183	42.60817	137.3382	42.53622	137.8631	42.6682	140.5693
42.61463	113.7669	42.53538	132.2957	42.51827	141.2155	42.62483	137.3358	42.55288	137.8619	42.68487	140.567
42.6313	113.7644	42.55205	132.2923	42.53493	141.2112	42.6415	137.3345	42.56955	137.8612	42.70153	140.5675
42.64797	113.7614	42.56872	132.293	42.5516	141.2128	42.65817	137.3345	42.58622	137.8595	42.7182	140.5633
42.66463	113.759	42.58538	132.2911	42.56827	141.211	42.67483	137.3341	42.60288	137.859	42.73487	140.5662
42.6813	113.7552	42.60205	132.293	42.58493	141.2094	42.6915	137.3322	42.61955	137.8599	42.75153	140.5632
42.69797	113.7535	42.61872	132.2912	42.6016	141.2072	42.70817	137.3308	42.63622	137.857	42.7682	140.5649
42.71463	113.7499	42.63538	132.2918	42.61827	141.2058	42.72483	137.3318	42.65288	137.8551	42.78487	140.5629
42.7313	113.7469	42.65205	132.2891	42.63493	141.2037	42.7415	137.3267	42.66955	137.8538	42.80153	140.5615
42.74797	113.7437	42.66872	132.2861	42.6516	141.201	42.75817	137.3279	42.68622	137.856	42.8182	140.5602
42.76463	113.7435	42.68538	132.2911	42.66827	141.205	42.77483	137.3268	42.70288	137.8541	42.83487	140.5598
42.7813	113.7382	42.70205	132.2871	42.68493	141.198	42.7915	137.3258	42.71955	137.8516	42.85153	140.5571
42.79797	113.738	42.71872	132.2864	42.7016	141.1978	42.80817	137.322	42.73622	137.8527	42.8682	140.5566
42.81463	113.7354	42.73538	132.2876	42.71827	141.1936	42.82483	137.3202	42.75288	137.8503	42.88487	140.5568
42.8313	113.7354	42.75205	132.2875	42.73493	141.1961	42.8415	137.3221	42.76955	137.8512	42.90153	140.5576
42.84797	113.7341	42.76872	132.2877	42.7516	141.1947	42.85817	137.3168	42.78622	137.8497	42.9182	140.5527
42.86463	113.7297	42.78538	132.2838	42.76827	141.191	42.87483	137.317	42.80288	137.8487	42.93487	140.5553
42.8813	113.7286	42.80205	132.2934	42.78493	141.1899	42.8915	137.3185	42.81955	137.8496	42.95153	140.5541
42.89797	113.7249	42.81872	132.2818	42.8016	141.1881	42.90817	137.3162	42.83622	137.8468	42.9682	140.5521
42.91463	113.7213	42.83538	132.2874	42.81827	141.1855	42.92483	137.313	42.85288	137.8491	42.98487	140.5499
42.9313	113.7192	42.85205	132.2834	42.83493	141.1881	42.9415	137.3107	42.86955	137.8477	43.00153	140.5489
42.94797	113.7161	42.86872	132.2838	42.8516	141.1837	42.95817	137.3132	42.88622	137.8433	43.0182	140.5474
42.96463	113.7121	42.88538	132.2827	42.86827	141.1835	42.97483	137.3124	42.90288	137.8456	43.03487	140.549
42.9813	113.7115	42.90205	132.2824	42.88493	141.1813	42.9915	137.306	42.91955	137.8423	43.05153	140.547
42.99797	113.7141	42.91872	132.2856	42.9016	141.1826	43.00817	137.3094	42.93622	137.8414	43.0682	140.5445
43.01463	113.7069	42.93538	132.2798	42.91827	141.1784	43.02483	137.3068	42.95288	137.8421	43.08487	140.5478
43.0313	113.7031	42.95205	132.2778	42.93493	141.1779	43.0415	137.3067	42.96955	137.8427	43.10153	140.5454
43.04797	113.7026	42.96872	132.2788	42.9516	141.1764	43.05817	137.302	42.98622	137.8408	43.1182	140.5444
43.06463	113.6985	42.98538	132.2769	42.96827	141.1759	43.07483	137.3055	43.00288	137.8387	43.13487	140.5414
43.0813	113.6963	43.00205	132.2777	42.98493	141.1774	43.0915	137.3026	43.01955	137.838	43.15153	140.5429
43.09797	113.698	43.01872	132.275	43.0016	141.1721	43.10817	137.2997	43.03622	137.8359	43.1682	140.5407
43.11463	113.6913	43.03538	132.2739	43.01827	141.1729	43.12483	137.2995	43.05288	137.8359	43.18487	140.54
43.1313	113.6902	43.05205	132.2766	43.03493	141.1711	43.1415	137.2979	43.06955	137.8358	43.20153	140.5382
43.14797	113.6858	43.06872	132.2752	43.0516	141.1708	43.15817	137.2982	43.08622	137.8359	43.2182	140.5384
43.16463	113.6851	43.08538	132.2761	43.06827	141.1691	43.17483	137.2985	43.10288	137.8326	43.23487	140.5372
43.1813	113.6822	43.10205	132.2757	43.08493	141.1648	43.1915	137.2925	43.11955	137.8326	43.25153	140.536
43.19797	113.6804	43.11872	132.2721	43.1016	141.166	43.20817	137.2961	43.13622	137.8324	43.2682	140.5351
43.21463	113.6766	43.13538	132.2737	43.11827	141.1616	43.22483	137.2914	43.15288	137.832	43.28487	140.5378
43.2313	113.6715	43.15205	132.2739	43.13493	141.1636	43.2415	137.2898	43.16955	137.83	43.30153	140.5345
43.24797	113.6736	43.16872	132.2748	43.1516	141.1606	43.25817	137.2922	43.18622	137.8308	43.3182	140.5338
43.26463	113.6692	43.18538	132.2716	43.16827	141.1594	43.27483	137.2895	43.20288	137.8292	43.33487	140.5329
43.2813	113.6669	43.20205	132.2712	43.18493	141.1597	43.2915	137.2845	43.21955	137.8263	43.35153	140.5333
43.29797	113.665	43.21872	132.2704	43.2016	141.1542	43.30817	137.2877	43.23622	137.8249	43.3682	140.5293
43.31463	113.6623	43.23538	132.2716	43.21827	141.1537	43.32483	137.2845	43.25288	137.8257	43.38487	140.5314
43.3313	113.6624	43.25205	132.2697	43.23493	141.1535	43.3415	137.287	43.26955	137.8269	43.40153	140.5295
43.34797	113.6554	43.26872	132.2695	43.2516	141.1517	43.35817	137.2834	43.28622	137.8225	43.4182	140.5289
43.36463	113.6524	43.28538	132.271	43.26827	141.1503	43.37483	137.2822	43.30288	137.8235	43.43487	140.5268
43.3813	113.6506	43.30205	132.2695	43.28493	141.1485	43.3915	137.2789	43.31955	137.8229	43.45153	140.5271
43.39797	113.651	43.31872	132.2688	43.3016	141.1491	43.40817	137.28	43.33622	137.8224	43.4682	140.5241
43.41463	113.6438	43.33538	132.27	43.31827	141.1475	43.42483	137.2763	43.35288	137.8208	43.48487	140.5219
43.4313	113.6439	43.35205	132.2675	43.33493	141.1463	43.4415	137.2766	43.36955	137.8221	43.50153	140.5236
43.44797	113.6406	43.36872	132.2667	43.3516	141.1448	43.45817	137.2786	43.38622	137.8209	43.5182	140.521
43.46463	113.6407	43.38538	132.2667	43.36827	141.1424	43.47483	137.2768	43.40288	137.8188	43.53487	140.5211
43.4813	113.6351	43.40205	132.2675	43.38493	141.1418	43.4915	137.277	43.41955	137.8177	43.55153	140.5232
43.49797	113.6329	43.41872	132.2693	43.4016	141.1407	43.50817	137.2747	43.43622	137.8183	43.5682	140.519
43.51463	113.6319	43.43538	132.2682	43.41827	141.1397	43.52483	137.2707	43.45288	137.8161	43.58487	140.5179
43.5313	113.6308	43.45205	132.2671	43.43493	141.1395	43.5415	137.269	43.46955	137.8155	43.60153	140.5191
43.54797	113.6259	43.46872	132.2669	43.4516	141.1399	43.55817	137.2705	43.48622	137.8138	43.6182	140.5182
43.56463	113.6255	43.48538	132.2696	43.46827	141.1377	43.57483	137.2688	43.50288	137.8157	43.63487	140.5177
43.5813	113.6211	43.50205	132.2691	43.48493	141.1387	43.5915	137.2671	43.51955	137.8133	43.65153	140.5157
43.59797	113.6192	43.51872	132.2704	43.5016	141.1366	43.60817	137.2623	43.53622	137.8114	43.6682	140.516
43.61463	113.6155	43.53538	132.2645	43.51827	141.1326	43.62483	137.2624	43.55288	137.8087	43.68487	140.5115
43.6313	113.615	43.55205	132.2673	43.53493	141.1336	43.6415	137.2637	43.56955	137.8107	43.70153	140.5122
43.64797	113.6095	43.56872	132.2691	43.5516	141.1309	43.65817	137.2633	43.58622	137.808	43.7182	140.5125
43.66463	113.6095	43.58538	132.2642	43.56827	141.1308	43.67483	137.261	43.60288	137.8078	43.73487	140.5098
43.6813	113.6063	43.60205	132.2691	43.58493	141.1298	43.6915	137.2603	43.61955	137.8056	43.75153	140.5077
43.69797	113.6079	43.61872	132.2646	43.6016	141.1272	43.70817	137.2596	43.63622	137.8052	43.7682	140.5087

43.71463	113.6023	43.63538	132.2621	43.61827	141.1262	43.72483	137.2557	43.65288	137.8056	43.78487	140.5097
43.7313	113.5985	43.65205	132.2659	43.63493	141.1235	43.7415	137.2565	43.66955	137.8038	43.80153	140.5057
43.74797	113.5975	43.66872	132.264	43.6516	141.1212	43.75817	137.2524	43.68622	137.8047	43.8182	140.5048
43.76463	113.5929	43.68538	132.2667	43.66827	141.1216	43.77483	137.2544	43.70288	137.8045	43.83487	140.5032
43.7813	113.5914	43.70205	132.2642	43.68493	141.1166	43.7915	137.2514	43.71955	137.802	43.85153	140.5009
43.79797	113.588	43.71872	132.2649	43.7016	141.1178	43.80817	137.2501	43.73622	137.8014	43.8682	140.5015
43.81463	113.5852	43.73538	132.2644	43.71827	141.1171	43.82483	137.2489	43.75288	137.8005	43.88487	140.5016
43.8313	113.5839	43.75205	132.2633	43.73493	141.1164	43.8415	137.2489	43.76955	137.7971	43.90153	140.4989
43.84797	113.5814	43.76872	132.261	43.7516	141.1124	43.85817	137.2491	43.78622	137.7978	43.9182	140.4994
43.86463	113.5771	43.78538	132.2629	43.76827	141.1148	43.87483	137.2457	43.80288	137.7985	43.93487	140.4979
43.8813	113.5758	43.80205	132.264	43.78493	141.1119	43.8915	137.2458	43.81955	137.7965	43.95153	140.4969
43.89797	113.5742	43.81872	132.2624	43.8016	141.1097	43.90817	137.246	43.83622	137.7956	43.9682	140.4962
43.91463	113.5709	43.83538	132.2609	43.81827	141.111	43.92483	137.2425	43.85288	137.7943	43.98487	140.4963
43.9313	113.5678	43.85205	132.2614	43.83493	141.1097	43.9415	137.2447	43.86955	137.7926	44.00153	140.4915
43.94797	113.5675	43.86872	132.26	43.8516	141.1064	43.95817	137.2439	43.88622	137.7903	44.0182	140.4944
43.96463	113.5657	43.88538	132.2605	43.86827	141.1043	43.97483	137.2411	43.90288	137.7921	44.03487	140.4924
43.9813	113.5605	43.90205	132.2591	43.88493	141.107	43.9915	137.2387	43.91955	137.7899	44.05153	140.4909
43.99797	113.5594	43.91872	132.2586	43.9016	141.1058	44.00817	137.2395	43.93622	137.7912	44.0682	140.4911
44.01463	113.5576	43.93538	132.2597	43.91827	141.1004	44.02483	137.2378	43.95288	137.7879	44.08487	140.4895
44.0313	113.5546	43.95205	132.2588	43.93493	141.1031	44.0415	137.2354	43.96955	137.7895	44.10153	140.4886
44.04797	113.5523	43.96872	132.2568	43.9516	141.0993	44.05817	137.2358	43.98622	137.7867	44.1182	140.487
44.06463	113.5505	43.98538	132.2561	43.96827	141.099	44.07483	137.2353	44.00288	137.7869	44.13487	140.4869
44.0813	113.5466	44.00205	132.2569	43.98493	141.0963	44.0915	137.2329	44.01955	137.7857	44.15153	140.4824
44.09797	113.5434	44.01872	132.2565	44.0016	141.0987	44.10817	137.2313	44.03622	137.7866	44.1682	140.4844
44.11463	113.5412	44.03538	132.2519	44.01827	141.0939	44.12483	137.2316	44.05288	137.7839	44.18487	140.484
44.1313	113.54	44.05205	132.2553	44.03493	141.0938	44.1415	137.2283	44.06955	137.7824	44.20153	140.4832
44.14797	113.5364	44.06872	132.2522	44.0516	141.0932	44.15817	137.2284	44.08622	137.7824	44.2182	140.4796
44.16463	113.5345	44.08538	132.2551	44.06827	141.0929	44.17483	137.2256	44.10288	137.7816	44.23487	140.4814
44.1813	113.5333	44.10205	132.2534	44.08493	141.0906	44.1915	137.2246	44.11955	137.782	44.25153	140.4784
44.19797	113.5298	44.11872	132.2531	44.1016	141.0893	44.20817	137.2237	44.13622	137.7817	44.2682	140.4765
44.21463	113.5263	44.13538	132.2506	44.11827	141.0875	44.22483	137.2222	44.15288	137.7808	44.28487	140.4775
44.2313	113.5254	44.15205	132.2499	44.13493	141.0868	44.2415	137.2209	44.16955	137.781	44.30153	140.479
44.24797	113.5229	44.16872	132.2502	44.1516	141.0843	44.25817	137.2192	44.18622	137.7784	44.3182	140.4751
44.26463	113.5212	44.18538	132.2487	44.16827	141.0831	44.27483	137.2178	44.20288	137.7805	44.33487	140.4753
44.2813	113.5187	44.20205	132.2525	44.18493	141.0824	44.2915	137.2171	44.21955	137.7787	44.35153	140.474
44.29797	113.5158	44.21872	132.2505	44.2016	141.0827	44.30817	137.2171	44.23622	137.7796	44.3682	140.4761
44.31463	113.5127	44.23538	132.2484	44.21827	141.0807	44.32483	137.2157	44.25288	137.778	44.38487	140.4735
44.3313	113.5117	44.25205	132.2484	44.23493	141.0812	44.3415	137.2157	44.26955	137.777	44.40153	140.4722
44.34797	113.5064	44.26872	132.247	44.2516	141.0797	44.35817	137.2131	44.28622	137.7753	44.4182	140.4707
44.36463	113.5071	44.28538	132.2452	44.26827	141.0765	44.37483	137.2167	44.30288	137.7732	44.43487	140.4701
44.3813	113.5026	44.30205	132.2464	44.28493	141.0761	44.3915	137.2121	44.31955	137.7726	44.45153	140.4723
44.39797	113.5019	44.31872	132.2482	44.3016	141.0721	44.40817	137.2127	44.33622	137.7736	44.4682	140.4694
44.41463	113.4971	44.33538	132.2483	44.31827	141.0748	44.42483	137.2117	44.35288	137.772	44.48487	140.4701
44.4313	113.4977	44.35205	132.2488	44.33493	141.0708	44.4415	137.2128	44.36955	137.7705	44.50153	140.4694
44.44797	113.4947	44.36872	132.2486	44.3516	141.0707	44.45817	137.2097	44.38622	137.77	44.5182	140.4683
44.46463	113.492	44.38538	132.2462	44.36827	141.0709	44.47483	137.208	44.40288	137.7702	44.53487	140.4651
44.4813	113.4892	44.40205	132.2468	44.38493	141.0686	44.4915	137.2078	44.41955	137.7695	44.55153	140.4646
44.49797	113.4869	44.41872	132.2465	44.4016	141.0647	44.50817	137.2089	44.43622	137.7674	44.5682	140.4638
44.51463	113.4838	44.43538	132.2464	44.41827	141.0695	44.52483	137.2077	44.45288	137.7668	44.58487	140.462
44.5313	113.4835	44.45205	132.2479	44.43493	141.0674	44.5415	137.2034	44.46955	137.7674	44.60153	140.462
44.54797	113.4813	44.46872	132.2478	44.4516	141.0664	44.55817	137.2062	44.48622	137.7648	44.6182	140.461
44.56463	113.4765	44.48538	132.246	44.46827	141.0647	44.57483	137.204	44.50288	137.7657	44.63487	140.4601
44.5813	113.4739	44.50205	132.2431	44.48493	141.0643	44.5915	137.2002	44.51955	137.7652	44.65153	140.4591
44.59797	113.4731	44.51872	132.2434	44.5016	141.062	44.60817	137.2009	44.53622	137.7648	44.6682	140.4579
44.61463	113.471	44.53538	132.2465	44.51827	141.0615	44.62483	137.2005	44.55288	137.7618	44.68487	140.4587
44.6313	113.4677	44.55205	132.2453	44.53493	141.0602	44.6415	137.1971	44.56955	137.7619	44.70153	140.4571
44.64797	113.4656	44.56872	132.2474	44.5516	141.0592	44.65817	137.1935	44.58622	137.762	44.7182	140.4558
44.66463	113.465	44.58538	132.2449	44.56827	141.0577	44.67483	137.1951	44.60288	137.7611	44.73487	140.4546
44.6813	113.4582	44.60205	132.243	44.58493	141.0535	44.6915	137.1938	44.61955	137.7605	44.75153	140.4537
44.69797	113.4576	44.61872	132.2456	44.6016	141.0568	44.70817	137.1899	44.63622	137.7628	44.7682	140.453
44.71463	113.4553	44.63538	132.2465	44.61827	141.0543	44.72483	137.1888	44.65288	137.7587	44.78487	140.4505
44.7313	113.4519	44.65205	132.2438	44.63493	141.0522	44.7415	137.1896	44.66955	137.758	44.80153	140.4511
44.74797	113.4492	44.66872	132.2487	44.6516	141.0501	44.75817	137.1874	44.68622	137.7548	44.8182	140.4494
44.76463	113.4491	44.68538	132.2448	44.66827	141.0501	44.77483	137.1861	44.70288	137.7579	44.83487	140.449
44.7813	113.4445	44.70205	132.2465	44.68493	141.0503	44.7915	137.1871	44.71955	137.7541	44.85153	140.4471
44.79797	113.4429	44.71872	132.2451	44.7016	141.05	44.80817	137.1822	44.73622	137.7541	44.8682	140.4486
44.81463	113.44	44.73538	132.2467	44.71827	141.0467	44.82483	137.1826	44.75288	137.7524	44.88487	140.4457
44.8313	113.4402	44.75205	132.2451	44.73493	141.0474	44.8415	137.184	44.76955	137.7519	44.90153	140.4448
44.84797	113.4354	44.76872	132.2431	44.7516	141.0452	44.85817	137.1803	44.78622	137.7501	44.9182	140.4438
44.86463	113.433	44.78538	132.2463	44.76827	141.0444	44.87483	137.178	44.80288	137.7498	44.93487	140.4436
44.8813	113.4314	44.80205	132.2446	44.78493	141.0439	44.8915	137.1785	44.81955	137.7457	44.95153	140.4425
44.89797	113.4274	44.81872	132.2426	44.8016	141.0424	44.90817	137.1796	44.83622	137.7478	44.9682	140.4397
44.91463	113.4252	44.83538	132.2441	44.81827	141.0418	44.92483	137.1747	44.85288	137.7445	44.98487	140.4399
44.9313	113.4251	44.85205	132.2455	44.83493	141.0396	44.9415	137.1744	44.86955	137.7455	45.00153	140.4401
44.94797	113.421	44.86872	132.2463	44.8516	141.0412	44.95817	137.1722	44.88622	137.742	45.0182	140.4372
44.96463	113.4199	44.88538	132.2451	44.86827	141.039	44.97483	137.1699	44.90288	137.7431	45.03487	140.4374
44.9813	113.4163	44.90205	132.2476	44.88493	141.0347	44.9915	137.1696	44.91955	137.7395	45.05153	140.4359
44.99797	113.4141	44.91872	132.2474	44.9016	141.0356	45.00817	137.168	44.93622	137.7393	45.0682	140.4362
45.01463	113.4079	44.93538	132.2449	44.91827	141.0342	45.02483	137.1669	44.95288	137.739	45.08487	140.4338
45.0313	113.4102	44.95205	132.2472	44.93493	141.0355	45.0415	137.167	44.96955	137.7369	45.10153	140.4342
45.04797	113.4032	44.96872	132.2485	44.9516	141.0333	45.05817	137.1648	44.98622	137.7369	45.1182	140.4324
45.06463	113.4049	44.98538	132.2485	44.96827	141.0292	45.07483	137.163	45.00288	137.7358	45.13487	140.4332
45.0813	113.403	45.00205	132.247	44.98493	141.0316	45.0915	137.163	45.01955	137.7348	45.15153	140.4297
45.09797	113.3984	45.01872	132.249	45.0016	141.0293	45.10817	137.1618	45.03622	137.7329	45.1682	140.4306
45.11463	113.3962	45.03538	132.2474	45.01827	141.0251	45.12483	137.1622	45.05288	137.7315	45.18487	140.4287
45.1313	113.3974	45.05205	132.2499	45.03493	141.0262	45.1415	137.1618	45.06955	137.73	45.20153	140.4288
45.14797	113.3954	45.06872	132.2502	45.0516	141.0248	45.15817	137.1614	45.08622	137.7298	45.2182	140.4255
45.16463	113.3919	45.08538	132.2498	45.06827	141.022	45.17483	137.1589	45.10288	137.7308	45.23487	140.4271
45.1813	113.3849	45.10205	132.2496	45.08493	141.022	45.1915	137.1584	45.11955	137.726	45.25153	140.4266
45.19797	113.3861	45.11872	132.2483	45.1016	141.0238	45.20817	137.1584	45.13622	137.7276	45.2682	140.4264
45.21463	113.3812	45.13538	132.2499	45.11827	141.0205	45.22483	137.1578	45.15288	137.7294	45.28487	140.4224
45.2313	113.3825	45.15205	132.2499	45.13493	141.0203	45.2415	137.1579	45.16955	137.728	45.30153	140.4237
45.24797	113.3777	45.16872	132.2497	45.1516	141.0202	45.25817	137.1555	45.18622	137.7256	45.3182	140.4231
45.26463	113.3741	45.18538	132.2465	45.16827	141.0212	45.27483	137.1569	45.20288	137.7241	45.33487	140.4227
45.2813	113.3702	45.20205	132.2476	45.18493	141.015	45.2915	137.1562	45.21955	137.7234	45.35153	140.4212
45.29797	113.373	45.21872	132.2493	45.2016	141.0173	45.30817	137.1541	45.23622	137.7218	45.3682	140.4199
45.31463	113.3689	45.23538	132.249	45.21827	141.017	45.32483	137.1505	45.25288	137.7192	45.38487	140.4186

45.3313	113.3678	45.25205	132.2516	45.23493	141.0153	45.3415	137.1496	45.26955	137.7216	45.40153	140.4185
45.34797	113.3657	45.26872	132.2507	45.2516	141.0116	45.35817	137.1498	45.28622	137.7188	45.4182	140.4155
45.36463	113.3643	45.28538	132.2504	45.26827	141.014	45.37483	137.1495	45.30288	137.7201	45.43487	140.4143
45.3813	113.3591	45.30205	132.2515	45.28493	141.0094	45.3915	137.144	45.31955	137.7172	45.45153	140.4162
45.39797	113.3589	45.31872	132.247	45.3016	141.0111	45.40817	137.1444	45.33622	137.7145	45.4682	140.4129
45.41463	113.3562	45.33538	132.2495	45.31827	141.007	45.42483	137.1424	45.35288	137.7163	45.48487	140.4135
45.4313	113.3563	45.35205	132.2503	45.33493	141.0049	45.4415	137.141	45.36955	137.7155	45.50153	140.4125
45.44797	113.3496	45.36872	132.252	45.3516	141.005	45.45817	137.1385	45.38622	137.7117	45.5182	140.4118
45.46463	113.3481	45.38538	132.2485	45.36827	141.0041	45.47483	137.1411	45.40288	137.7115	45.53487	140.4096
45.4813	113.3485	45.40205	132.2509	45.38493	140.9998	45.4915	137.1376	45.41955	137.712	45.55153	140.4082
45.49797	113.3444	45.41872	132.2477	45.4016	141.0014	45.50817	137.1356	45.43622	137.7113	45.5682	140.4053
45.51463	113.3424	45.43538	132.254	45.41827	140.9978	45.52483	137.136	45.45288	137.7092	45.58487	140.4063
45.5313	113.3405	45.45205	132.2524	45.43493	140.9979	45.5415	137.1359	45.46955	137.7093	45.60153	140.404
45.54797	113.3375	45.46872	132.253	45.4516	140.994	45.55817	137.1338	45.48622	137.7072	45.6182	140.4054
45.56463	113.336	45.48538	132.2525	45.46827	140.9957	45.57483	137.1327	45.50288	137.7054	45.63487	140.4048
45.5813	113.3332	45.50205	132.2526	45.48493	140.993	45.5915	137.1271	45.51955	137.7041	45.65153	140.4041
45.59797	113.3291	45.51872	132.2508	45.5016	140.9925	45.60817	137.1289	45.53622	137.7071	45.6682	140.4015
45.61463	113.3289	45.53538	132.2499	45.51827	140.9909	45.62483	137.1282	45.55288	137.7034	45.68487	140.4017
45.6313	113.3272	45.55205	132.2494	45.53493	140.99	45.6415	137.1244	45.56955	137.7052	45.70153	140.3993
45.64797	113.3233	45.56872	132.2506	45.5516	140.9855	45.65817	137.1257	45.58622	137.7037	45.7182	140.4022
45.66463	113.323	45.58538	132.2499	45.56827	140.9879	45.67483	137.1234	45.60288	137.7019	45.73487	140.3997
45.6813	113.3172	45.60205	132.2504	45.58493	140.9834	45.6915	137.122	45.61955	137.699	45.75153	140.3975
45.69797	113.3171	45.61872	132.248	45.6016	140.9852	45.70817	137.1191	45.63622	137.7016	45.7682	140.3969
45.71463	113.3131	45.63538	132.2496	45.61827	140.9813	45.72483	137.1189	45.65288	137.6981	45.78487	140.3979
45.7313	113.3128	45.65205	132.2507	45.63493	140.9819	45.7415	137.1188	45.66955	137.6966	45.80153	140.396
45.74797	113.3087	45.66872	132.2489	45.6516	140.9806	45.75817	137.1149	45.68622	137.696	45.8182	140.392
45.76463	113.3071	45.68538	132.2501	45.66827	140.9788	45.77483	137.1143	45.70288	137.6953	45.83487	140.3931
45.7813	113.3051	45.70205	132.2458	45.68493	140.9776	45.7915	137.1142	45.71955	137.6955	45.85153	140.3917
45.79797	113.3012	45.71872	132.2477	45.7016	140.9776	45.80817	137.1158	45.73622	137.6944	45.8682	140.3919
45.81463	113.298	45.73538	132.2501	45.71827	140.9761	45.82483	137.1116	45.75288	137.6928	45.88487	140.3912
45.8313	113.2979	45.75205	132.251	45.73493	140.9751	45.8415	137.1121	45.76955	137.6923	45.90153	140.3895
45.84797	113.2926	45.76872	132.2494	45.7516	140.9756	45.85817	137.1094	45.78622	137.6911	45.9182	140.3889
45.86463	113.2913	45.78538	132.2486	45.76827	140.9737	45.87483	137.1115	45.80288	137.6911	45.93487	140.3877
45.8813	113.2908	45.80205	132.2503	45.78493	140.9716	45.8915	137.1098	45.81955	137.6885	45.95153	140.3878
45.89797	113.2873	45.81872	132.2503	45.8016	140.9717	45.90817	137.108	45.83622	137.6882	45.9682	140.3864
45.91463	113.2845	45.83538	132.2501	45.81827	140.97	45.92483	137.1072	45.85288	137.6921	45.98487	140.3855
45.9313	113.2795	45.85205	132.248	45.83493	140.9678	45.9415	137.1072	45.86955	137.6878	46.00153	140.384
45.94797	113.2778	45.86872	132.2513	45.8516	140.9658	45.95817	137.1039	45.88622	137.6858	46.0182	140.3824
45.96463	113.2768	45.88538	132.2512	45.86827	140.9667	45.97483	137.103	45.90288	137.6867	46.03487	140.381
45.9813	113.2721	45.90205	132.2516	45.88493	140.9629	45.9915	137.103	45.91955	137.683	46.05153	140.3831
45.99797	113.2709	45.91872	132.2483	45.9016	140.9625	46.00817	137.102	45.93622	137.6794	46.0682	140.3785
46.01463	113.2698	45.93538	132.2483	45.91827	140.9609	46.02483	137.0994	45.95288	137.6816	46.08487	140.3789
46.0313	113.2658	45.95205	132.2478	45.93493	140.9591	46.0415	137.0993	45.96955	137.6823	46.10153	140.3764
46.04797	113.2635	45.96872	132.2471	45.9516	140.9602	46.05817	137.0965	45.98622	137.6817	46.1182	140.3782
46.06463	113.2628	45.98538	132.2489	45.96827	140.957	46.07483	137.0973	46.00288	137.6802	46.13487	140.376
46.0813	113.2565	46.00205	132.2465	45.98493	140.9564	46.0915	137.0936	46.01955	137.6795	46.15153	140.3737
46.09797	113.2586	46.01872	132.2474	46.0016	140.9561	46.10817	137.0942	46.03622	137.6795	46.1682	140.3724
46.11463	113.2535	46.03538	132.2497	46.01827	140.9546	46.12483	137.094	46.05288	137.6773	46.18487	140.373
46.1313	113.2535	46.05205	132.2478	46.03493	140.9519	46.1415	137.0933	46.06955	137.6782	46.20153	140.3723
46.14797	113.2469	46.06872	132.2489	46.0516	140.9489	46.15817	137.0915	46.08622	137.6753	46.2182	140.3704
46.16463	113.249	46.08538	132.2479	46.06827	140.9516	46.17483	137.0889	46.10288	137.6736	46.23487	140.3705
46.1813	113.2442	46.10205	132.2491	46.08493	140.9517	46.1915	137.0877	46.11955	137.671	46.25153	140.3715
46.19797	113.2443	46.11872	132.2521	46.1016	140.9481	46.20817	137.0877	46.13622	137.6718	46.2682	140.3723
46.21463	113.2402	46.13538	132.249	46.11827	140.9449	46.22483	137.0868	46.15288	137.6742	46.28487	140.3683
46.2313	113.2385	46.15205	132.2483	46.13493	140.9466	46.2415	137.0855	46.16955	137.6715	46.30153	140.3699
46.24797	113.2347	46.16872	132.2488	46.1516	140.9437	46.25817	137.0837	46.18622	137.6704	46.3182	140.3697
46.26463	113.2328	46.18538	132.2476	46.16827	140.942	46.27483	137.0821	46.20288	137.6676	46.33487	140.3671
46.2813	113.2299	46.20205	132.2497	46.18493	140.9375	46.2915	137.0824	46.21955	137.6643	46.35153	140.3657
46.29797	113.2304	46.21872	132.2505	46.2016	140.935	46.30817	137.0801	46.23622	137.6643	46.3682	140.3664
46.31463	113.2243	46.23538	132.252	46.21827	140.9432	46.32483	137.0783	46.25288	137.6634	46.38487	140.3664
46.3313	113.2209	46.25205	132.2527	46.23493	140.938	46.3415	137.0779	46.26955	137.665	46.40153	140.3623
46.34797	113.2234	46.26872	132.2492	46.2516	140.9356	46.35817	137.0776	46.28622	137.6661	46.4182	140.363
46.36463	113.2177	46.28538	132.2495	46.26827	140.9344	46.37483	137.0766	46.30288	137.6658	46.43487	140.3638
46.3813	113.2143	46.30205	132.2498	46.28493	140.9333	46.3915	137.0762	46.31955	137.6656	46.45153	140.3609
46.39797	113.2154	46.31872	132.2505	46.3016	140.9326	46.40817	137.0772	46.33622	137.6625	46.4682	140.3577
46.41463	113.2113	46.33538	132.2478	46.31827	140.9338	46.42483	137.0712	46.35288	137.6606	46.48487	140.3576
46.4313	113.21	46.35205	132.2505	46.33493	140.932	46.4415	137.0722	46.36955	137.6623	46.50153	140.3574
46.44797	113.2068	46.36872	132.2512	46.3516	140.9286	46.45817	137.0737	46.38622	137.6612	46.5182	140.3575
46.46463	113.206	46.38538	132.2504	46.36827	140.9295	46.47483	137.0726	46.40288	137.659	46.53487	140.3557
46.4813	113.2023	46.40205	132.2494	46.38493	140.932	46.4915	137.0707	46.41955	137.6587	46.55153	140.3564
46.49797	113.1994	46.41872	132.2523	46.4016	140.9257	46.50817	137.07	46.43622	137.6589	46.5682	140.3547
46.51463	113.1978	46.43538	132.2507	46.41827	140.9278	46.52483	137.0688	46.45288	137.6557	46.58487	140.3531
46.5313	113.1977	46.45205	132.2509	46.43493	140.9232	46.5415	137.0658	46.46955	137.6571	46.60153	140.3524
46.54797	113.1967	46.46872	132.2518	46.4516	140.9228	46.55817	137.0641	46.48622	137.6565	46.6182	140.3539
46.56463	113.1932	46.48538	132.2501	46.46827	140.9225	46.57483	137.0654	46.50288	137.655	46.63487	140.3506
46.5813	113.1895	46.50205	132.2508	46.48493	140.9211	46.5915	137.0628	46.51955	137.6534	46.65153	140.3503
46.59797	113.1893	46.51872	132.251	46.5016	140.9196	46.60817	137.061	46.53622	137.6548	46.6682	140.3493
46.61463	113.1852	46.53538	132.2507	46.51827	140.9194	46.62483	137.0611	46.55288	137.6519	46.68487	140.3503
46.6313	113.1843	46.55205	132.2529	46.53493	140.917	46.6415	137.0598	46.56955	137.6495	46.70153	140.3473
46.64797	113.1814	46.56872	132.2514	46.5516	140.9171	46.65817	137.0599	46.58622	137.6508	46.7182	140.3462
46.66463	113.1781	46.58538	132.2541	46.56827	140.9183	46.67483	137.0565	46.60288	137.6469	46.73487	140.3453
46.6813	113.1747	46.60205	132.2547	46.58493	140.913	46.6915	137.0556	46.61955	137.6466	46.75153	140.3464
46.69797	113.1764	46.61872	132.2546	46.6016	140.9114	46.70817	137.0536	46.63622	137.6489	46.7682	140.3433
46.71463	113.1698	46.63538	132.2586	46.61827	140.9117	46.72483	137.0528	46.65288	137.6466	46.78487	140.3424
46.7313	113.1713	46.65205	132.2557	46.63493	140.912	46.7415	137.0519	46.66955	137.6449	46.80153	140.3424
46.74797	113.1676	46.66872	132.2552	46.6516	140.9098	46.75817	137.0521	46.68622	137.6421	46.8182	140.3409
46.76463	113.1625	46.68538	132.2566	46.66827	140.907	46.77483	137.0498	46.70288	137.6443	46.83487	140.3414
46.7813	113.1629	46.70205	132.2526	46.68493	140.9076	46.7915	137.051	46.71955	137.6403	46.85153	140.3393
46.79797	113.1612	46.71872	132.2546	46.7016	140.904	46.80817	137.0515	46.73622	137.6403	46.8682	140.3409
46.81463	113.1573	46.73538	132.256	46.71827	140.9044	46.82483	137.0501	46.75288	137.6406	46.88487	140.3379
46.8313	113.1567	46.75205	132.2542	46.73493	140.9026	46.8415	137.0468	46.76955	137.6382	46.90153	140.3401
46.84797	113.1573	46.76872	132.2592	46.7516	140.9003	46.85817	137.0467	46.78622	137.6364	46.9182	140.3386
46.86463	113.1527	46.78538	132.2582	46.76827	140.8998	46.87483	137.0486	46.80288	137.637	46.93487	140.3374
46.8813	113.1502	46.80205	132.2563	46.78493	140.8993	46.8915	137.0471	46.81955	137.6359	46.95153	140.3362
46.89797	113.1485	46.81872	132.2569	46.8016	140.8969	46.90817	137.0456	46.83622	137.6339	46.9682	140.3356
46.91463	113.146	46.83538	132.258	46.81827	140.8988	46.92483	137.0433	46.85288	137.6345	46.98487	140.3358
46.9313	113.145	46.85205	132.2595	46.83493	140.8985	46.9415	137.043	46.86955	137.6338	47.00153	140.3337

46.94797	113.1434	46.86872	132.2592	46.8516	140.8945	46.95817	137.045	46.88622	137.6314	47.0182	140.3333
46.96463	113.1415	46.88538	132.2573	46.86827	140.894	46.97483	137.041	46.90288	137.6335	47.03487	140.3326
46.9813	113.1391	46.90205	132.259	46.88493	140.8925	46.9915	137.0383	46.91955	137.631	47.05153	140.3334
46.99797	113.1362	46.91872	132.2597	46.9016	140.8934	47.00817	137.0392	46.93622	137.6264	47.0682	140.3302
47.01463	113.1342	46.93538	132.2569	46.91827	140.8887	47.02483	137.0414	46.95288	137.6286	47.08487	140.3285
47.0313	113.133	46.95205	132.2577	46.93493	140.8917	47.0415	137.0373	46.96955	137.6269	47.10153	140.3297
47.04797	113.13	46.96872	132.2575	46.9516	140.888	47.05817	137.0389	46.98622	137.6257	47.1182	140.3289
47.06463	113.1304	46.98538	132.2565	46.96827	140.8838	47.07483	137.0379	47.00288	137.6262	47.13487	140.3284
47.0813	113.1269	47.00205	132.2586	46.98493	140.8881	47.0915	137.037	47.01955	137.6238	47.15153	140.3263
47.09797	113.1235	47.01872	132.2588	47.0016	140.8818	47.10817	137.0363	47.03622	137.6216	47.1682	140.3266
47.11463	113.1239	47.03538	132.2551	47.01827	140.8813	47.12483	137.0364	47.05288	137.6232	47.18487	140.3264
47.1313	113.1203	47.05205	132.257	47.03493	140.8805	47.1415	137.0336	47.06955	137.6213	47.20153	140.3241
47.14797	113.1209	47.06872	132.256	47.0516	140.8796	47.15817	137.036	47.08622	137.6189	47.2182	140.3233
47.16463	113.1181	47.08538	132.2574	47.06827	140.8755	47.17483	137.0319	47.10288	137.62	47.23487	140.3236
47.1813	113.1132	47.10205	132.2581	47.08493	140.8763	47.1915	137.0357	47.11955	137.6166	47.25153	140.3225
47.19797	113.1133	47.11872	132.2577	47.1016	140.8722	47.20817	137.0319	47.13622	137.6188	47.2682	140.3244
47.21463	113.1102	47.13538	132.2549	47.11827	140.8712	47.22483	137.0314	47.15288	137.6167	47.28487	140.3219
47.2313	113.1066	47.15205	132.2561	47.13493	140.8737	47.2415	137.0281	47.16955	137.6154	47.30153	140.3201
47.24797	113.106	47.16872	132.2559	47.1516	140.8728	47.25817	137.0297	47.18622	137.6126	47.3182	140.3206
47.26463	113.1038	47.18538	132.2548	47.16827	140.8687	47.27483	137.0303	47.20288	137.6127	47.33487	140.3204
47.2813	113.0971	47.20205	132.2539	47.18493	140.8669	47.2915	137.0305	47.21955	137.6133	47.35153	140.3181
47.29797	113.098	47.21872	132.2541	47.2016	140.8698	47.30817	137.0253	47.23622	137.6095	47.3682	140.3173
47.31463	113.0945	47.23538	132.2534	47.21827	140.8668	47.32483	137.0288	47.25288	137.6112	47.38487	140.3139
47.3313	113.0904	47.25205	132.2538	47.23493	140.8677	47.3415	137.0248	47.26955	137.6087	47.40153	140.317
47.34797	113.089	47.26872	132.2525	47.2516	140.8632	47.35817	137.028	47.28622	137.6099	47.4182	140.3139
47.36463	113.0886	47.28538	132.2516	47.26827	140.8617	47.37483	137.0241	47.30288	137.6118	47.43487	140.3125
47.3813	113.0835	47.30205	132.2496	47.28493	140.8642	47.3915	137.0253	47.31955	137.6067	47.45153	140.3121
47.39797	113.0831	47.31872	132.2506	47.3016	140.8611	47.40817	137.0229	47.33622	137.6067	47.4682	140.3105
47.41463	113.0792	47.33538	132.2539	47.31827	140.8542	47.42483	137.0232	47.35288	137.6044	47.48487	140.3082
47.4313	113.0733	47.35205	132.2492	47.33493	140.8578	47.4415	137.0193	47.36955	137.6079	47.50153	140.3099
47.44797	113.0767	47.36872	132.2483	47.3516	140.8547	47.45817	137.0197	47.38622	137.6054	47.5182	140.305
47.46463	113.075	47.38538	132.2522	47.36827	140.8574	47.47483	137.0164	47.40288	137.6059	47.53487	140.3068
47.4813	113.0684	47.40205	132.2492	47.38493	140.8545	47.4915	137.0173	47.41955	137.6034	47.55153	140.3061
47.49797	113.0698	47.41872	132.2502	47.4016	140.8531	47.50817	137.017	47.43622	137.6023	47.5682	140.3068
47.51463	113.0654	47.43538	132.2473	47.41827	140.8492	47.52483	137.0189	47.45288	137.6019	47.58487	140.3044
47.5313	113.0653	47.45205	132.2492	47.43493	140.8517	47.5415	137.0143	47.46955	137.6004	47.60153	140.3027
47.54797	113.0592	47.46872	132.2476	47.4516	140.8539	47.55817	137.0162	47.48622	137.5969	47.6182	140.2993
47.56463	113.0586	47.48538	132.2464	47.46827	140.851	47.57483	137.0142	47.50288	137.5958	47.63487	140.3009
47.5813	113.0568	47.50205	132.2483	47.48493	140.8494	47.5915	137.0154	47.51955	137.5971	47.65153	140.3007
47.59797	113.0515	47.51872	132.2475	47.5016	140.8481	47.60817	137.012	47.53622	137.5941	47.6682	140.3021
47.61463	113.0498	47.53538	132.2458	47.51827	140.8465	47.62483	137.0146	47.55288	137.5935	47.68487	140.2981
47.6313	113.0472	47.55205	132.2471	47.53493	140.8456	47.6415	137.0103	47.56955	137.5911	47.70153	140.2985
47.64797	113.0439	47.56872	132.2469	47.5516	140.845	47.65817	137.0108	47.58622	137.5924	47.7182	140.2981
47.66463	113.0411	47.58538	132.2466	47.56827	140.8438	47.67483	137.0089	47.60288	137.5893	47.73487	140.2972
47.6813	113.0386	47.60205	132.2452	47.58493	140.844	47.6915	137.0112	47.61955	137.5888	47.75153	140.2938
47.69797	113.0345	47.61872	132.2485	47.6016	140.8439	47.70817	137.007	47.63622	137.5857	47.7682	140.2953
47.71463	113.0354	47.63538	132.2461	47.61827	140.842	47.72483	137.0086	47.65288	137.5831	47.78487	140.2943
47.7313	113.0319	47.65205	132.246	47.63493	140.8385	47.7415	137.0052	47.66955	137.5853	47.80153	140.295
47.74797	113.028	47.66872	132.2449	47.6516	140.842	47.75817	137.002	47.68622	137.5839	47.8182	140.2909
47.76463	113.0273	47.68538	132.2475	47.66827	140.8378	47.77483	137.0022	47.70288	137.5834	47.83487	140.2916
47.7813	113.0258	47.70205	132.2482	47.68493	140.8395	47.7915	137.0031	47.71955	137.5821	47.85153	140.2915
47.79797	113.0227	47.71872	132.2488	47.7016	140.8358	47.80817	137.0021	47.73622	137.5818	47.8682	140.2912
47.81463	113.0241	47.73538	132.2479	47.71827	140.8356	47.82483	136.9992	47.75288	137.5802	47.88487	140.2901
47.8313	113.0194	47.75205	132.2526	47.73493	140.8339	47.8415	137.0026	47.76955	137.5794	47.90153	140.2873
47.84797	113.0167	47.76872	132.2527	47.7516	140.834	47.85817	137.0009	47.78622	137.5766	47.9182	140.2888
47.86463	113.0163	47.78538	132.2508	47.76827	140.8308	47.87483	136.9998	47.80288	137.5783	47.93487	140.2859
47.8813	113.0131	47.80205	132.2509	47.78493	140.8322	47.8915	136.998	47.81955	137.5758	47.95153	140.2866
47.89797	113.0086	47.81872	132.2508	47.8016	140.8293	47.90817	136.997	47.83622	137.5745	47.9682	140.288
47.91463	113.0087	47.83538	132.2542	47.81827	140.8278	47.92483	136.9992	47.85288	137.5737	47.98487	140.2821
47.9313	113.0049	47.85205	132.2514	47.83493	140.826	47.9415	136.9968	47.86955	137.5734	48.00153	140.2869
47.94797	113.0022	47.86872	132.2517	47.8516	140.8274	47.95817	136.9961	47.88622	137.5729	48.0182	140.2824
47.96463	113.0001	47.88538	132.2532	47.86827	140.8259	47.97483	136.9927	47.90288	137.5711	48.03487	140.2828
47.9813	112.9968	47.90205	132.2535	47.88493	140.8222	47.9915	136.9959	47.91955	137.5702	48.05153	140.2838
47.99797	112.9969	47.91872	132.2537	47.9016	140.8217	48.00817	136.9945	47.93622	137.5701	48.0682	140.283
48.01463	112.992	47.93538	132.254	47.91827	140.8195	48.02483	136.9927	47.95288	137.5688	48.08487	140.2819
48.0313	112.9889	47.95205	132.2525	47.93493	140.8237	48.0415	136.9913	47.96955	137.5701	48.10153	140.2812
48.04797	112.9902	47.96872	132.2519	47.9516	140.8181	48.05817	136.9931	47.98622	137.5673	48.1182	140.2794
48.06463	112.987	47.98538	132.2521	47.96827	140.8155	48.07483	136.9903	48.00288	137.5676	48.13487	140.2788
48.0813	112.9825	48.00205	132.2516	47.98493	140.8164	48.0915	136.9886	48.01955	137.566	48.15153	140.2805
48.09797	112.9819	48.01872	132.252	48.0016	140.8129	48.10817	136.9868	48.03622	137.5659	48.1682	140.2781
48.11463	112.979	48.03538	132.2523	48.01827	140.8163	48.12483	136.9885	48.05288	137.564	48.18487	140.2776
48.1313	112.9781	48.05205	132.2521	48.03493	140.8135	48.1415	136.9867	48.06955	137.5634	48.20153	140.2776
48.14797	112.9752	48.06872	132.2532	48.0516	140.8103	48.15817	136.9857	48.08622	137.5625	48.2182	140.273
48.16463	112.9729	48.08538	132.2567	48.06827	140.8075	48.17483	136.9836	48.10288	137.5608	48.23487	140.2722
48.1813	112.9691	48.10205	132.2547	48.08493	140.8079	48.1915	136.9841	48.11955	137.5599	48.25153	140.2723
48.19797	112.9683	48.11872	132.2572	48.1016	140.8073	48.20817	136.9858	48.13622	137.5587	48.2682	140.2719
48.21463	112.9674	48.13538	132.2581	48.11827	140.8044	48.22483	136.9808	48.15288	137.5591	48.28487	140.2681
48.2313	112.9636	48.15205	132.259	48.13493	140.8057	48.2415	136.9813	48.16955	137.559	48.30153	140.2694
48.24797	112.9621	48.16872	132.2585	48.1516	140.8057	48.25817	136.979	48.18622	137.5567	48.3182	140.2671
48.26463	112.9599	48.18538	132.2611	48.16827	140.803	48.27483	136.9779	48.20288	137.5552	48.33487	140.2663
48.2813	112.9594	48.20205	132.2586	48.18493	140.8014	48.2915	136.9772	48.21955	137.554	48.35153	140.2649
48.29797	112.954	48.21872	132.2632	48.2016	140.7996	48.30817	136.9779	48.23622	137.5548	48.3682	140.2637
48.31463	112.9541	48.23538	132.2638	48.21827	140.8009	48.32483	136.9777	48.25288	137.5514	48.38487	140.2636
48.3313	112.9513	48.25205	132.2642	48.23493	140.8002	48.3415	136.9762	48.26955	137.5515	48.40153	140.2636
48.34797	112.9487	48.26872	132.2624	48.2516	140.7955	48.35817	136.9749	48.28622	137.5504	48.4182	140.2629
48.36463	112.9453	48.28538	132.263	48.26827	140.793	48.37483	136.9742	48.30288	137.5493	48.43487	140.2614
48.3813	112.9446	48.30205	132.2633	48.28493	140.7938	48.3915	136.9709	48.31955	137.5506	48.45153	140.2601
48.39797	112.9441	48.31872	132.2679	48.3016	140.7946	48.40817	136.974	48.33622	137.5496	48.4682	140.2607
48.41463	112.9391	48.33538	132.2668	48.31827	140.7939	48.42483	136.9703	48.35288	137.5468	48.48487	140.2568
48.4313	112.9391	48.35205	132.2659	48.33493	140.7897	48.4415	136.9697	48.36955	137.5458	48.50153	140.2557
48.44797	112.9363	48.36872	132.267	48.3516	140.7907	48.45817	136.9685	48.38622	137.5482	48.5182	140.2574
48.46463	112.933	48.38538	132.2713	48.36827	140.7872	48.47483	136.9647	48.40288	137.5437	48.53487	140.255
48.4813	112.9305	48.40205	132.2691	48.38493	140.7877	48.4915	136.9678	48.41955	137.5433	48.55153	140.2527
48.49797	112.927	48.41872	132.2702	48.4016	140.783	48.50817	136.9673	48.43622	137.5418	48.5682	140.2525
48.51463	112.9232	48.43538	132.2722	48.41827	140.781	48.52483	136.968	48.45288	137.5437	48.58487	140.251
48.5313	112.925	48.45205	132.2719	48.43493	140.7823	48.5415	136.9653	48.46955	137.5417	48.60153	140.2527
48.54797	112.9213	48.46872	132.2713	48.4516	140.7798	48.55817	136.9643	48.48622	137.5403	48.6182	140.2508

48.56463	112.9201	48.48538	132.275	48.46827	140.7818	48.57483	136.9635	48.50288	137.5384	48.63487	140.2472
48.5813	112.9172	48.50205	132.2758	48.48493	140.777	48.5915	136.9626	48.51955	137.5371	48.65153	140.245
48.59797	112.9149	48.51872	132.2765	48.5016	140.7755	48.60817	136.9616	48.53622	137.5388	48.6682	140.2451
48.61463	112.9135	48.53538	132.2777	48.51827	140.7767	48.62483	136.9642	48.55288	137.5369	48.68487	140.2464
48.6313	112.9115	48.55205	132.2779	48.53493	140.7756	48.6415	136.9604	48.56955	137.5327	48.70153	140.2425
48.64797	112.9079	48.56872	132.2754	48.5516	140.774	48.65817	136.9623	48.58622	137.5338	48.7182	140.2431
48.66463	112.9073	48.58538	132.2812	48.56827	140.7707	48.67483	136.9603	48.60288	137.5352	48.73487	140.2418
48.6813	112.9039	48.60205	132.2782	48.58493	140.7722	48.6915	136.9596	48.61955	137.532	48.75153	140.2385
48.69797	112.9033	48.61872	132.2788	48.6016	140.77	48.70817	136.9591	48.63622	137.5317	48.7682	140.2402
48.71463	112.9011	48.63538	132.2792	48.61827	140.7702	48.72483	136.9601	48.65288	137.5312	48.78487	140.2391
48.7313	112.8983	48.65205	132.2799	48.63493	140.7691	48.7415	136.9554	48.66955	137.5293	48.80153	140.2397
48.74797	112.8952	48.66872	132.2774	48.6516	140.7669	48.75817	136.9574	48.68622	137.5296	48.8182	140.2375
48.76463	112.8942	48.68538	132.2764	48.66827	140.7657	48.77483	136.9556	48.70288	137.5256	48.83487	140.2373
48.7813	112.8926	48.70205	132.2803	48.68493	140.7656	48.7915	136.9562	48.71955	137.5271	48.85153	140.2353
48.79797	112.8904	48.71872	132.2762	48.7016	140.7612	48.80817	136.9576	48.73622	137.5264	48.8682	140.2362
48.81463	112.8878	48.73538	132.2775	48.71827	140.7612	48.82483	136.9554	48.75288	137.5251	48.88487	140.2322
48.8313	112.8853	48.75205	132.2771	48.73493	140.7591	48.8415	136.9536	48.76955	137.5225	48.90153	140.2324
48.84797	112.887	48.76872	132.2745	48.7516	140.7601	48.85817	136.9542	48.78622	137.5221	48.9182	140.2318
48.86463	112.8801	48.78538	132.2745	48.76827	140.7573	48.87483	136.9542	48.80288	137.5225	48.93487	140.2321
48.8813	112.8796	48.80205	132.2751	48.78493	140.7551	48.8915	136.9515	48.81955	137.5222	48.95153	140.2296
48.89797	112.8775	48.81872	132.2744	48.8016	140.7545	48.90817	136.9555	48.83622	137.52	48.9682	140.23
48.91463	112.8739	48.83538	132.2773	48.81827	140.7546	48.92483	136.9537	48.85288	137.5205	48.98487	140.2301
48.9313	112.8727	48.85205	132.2759	48.83493	140.751	48.9415	136.9516	48.86955	137.5193	49.00153	140.2258
48.94797	112.8714	48.86872	132.2737	48.8516	140.7498	48.95817	136.9515	48.88622	137.5173	49.0182	140.2272
48.96463	112.8694	48.88538	132.2738	48.86827	140.7481	48.97483	136.9513	48.90288	137.5176	49.03487	140.2254
48.9813	112.8666	48.90205	132.2726	48.88493	140.7461	48.9915	136.9487	48.91955	137.5169	49.05153	140.2234
48.99797	112.8636	48.91872	132.2724	48.9016	140.7432	49.00817	136.9457	48.93622	137.5155	49.0682	140.2243
49.01463	112.8605	48.93538	132.2705	48.91827	140.7427	49.02483	136.9476	48.95288	137.5168	49.08487	140.2227
49.0313	112.8594	48.95205	132.2725	48.93493	140.7386	49.0415	136.945	48.96955	137.5139	49.10153	140.2201
49.04797	112.8565	48.96872	132.2731	48.9516	140.7433	49.05817	136.9457	48.98622	137.516	49.1182	140.2195
49.06463	112.8564	48.98538	132.2724	48.96827	140.738	49.07483	136.943	49.00288	137.5156	49.13487	140.2201
49.0813	112.8538	49.00205	132.2699	48.98493	140.7382	49.0915	136.9422	49.01955	137.5152	49.15153	140.2172
49.09797	112.8514	49.01872	132.2699	49.0016	140.7374	49.10817	136.943	49.03622	137.5134	49.1682	140.2169
49.11463	112.8487	49.03538	132.2711	49.01827	140.7366	49.12483	136.9428	49.05288	137.5124	49.18487	140.215
49.1313	112.8478	49.05205	132.2695	49.03493	140.7335	49.1415	136.9403	49.06955	137.5105	49.20153	140.2162
49.14797	112.8469	49.06872	132.268	49.0516	140.7344	49.15817	136.9399	49.08622	137.5109	49.2182	140.2127
49.16463	112.8412	49.08538	132.2713	49.06827	140.7279	49.17483	136.938	49.10288	137.511	49.23487	140.2128
49.1813	112.8395	49.10205	132.2674	49.08493	140.7314	49.1915	136.943	49.11955	137.509	49.25153	140.2113
49.19797	112.8384	49.11872	132.2719	49.1016	140.7274	49.20817	136.9387	49.13622	137.5074	49.2682	140.2107
49.21463	112.8347	49.13538	132.2705	49.11827	140.7291	49.22483	136.938	49.15288	137.506	49.28487	140.2083
49.2313	112.8316	49.15205	132.2707	49.13493	140.7267	49.2415	136.9393	49.16955	137.5062	49.30153	140.2101
49.24797	112.8288	49.16872	132.27	49.1516	140.7277	49.25817	136.9408	49.18622	137.5047	49.3182	140.2068
49.26463	112.8296	49.18538	132.2689	49.16827	140.7246	49.27483	136.9386	49.20288	137.5043	49.33487	140.2072
49.2813	112.8248	49.20205	132.2675	49.18493	140.7231	49.2915	136.9358	49.21955	137.5048	49.35153	140.2059
49.29797	112.8248	49.21872	132.2699	49.2016	140.721	49.30817	136.9377	49.23622	137.5023	49.3682	140.2072
49.31463	112.8225	49.23538	132.268	49.21827	140.7208	49.32483	136.9383	49.25288	137.5023	49.38487	140.2048
49.3313	112.8168	49.25205	132.27	49.23493	140.7214	49.3415	136.9364	49.26955	137.4995	49.40153	140.2032
49.34797	112.8188	49.26872	132.2693	49.2516	140.7179	49.35817	136.9358	49.28622	137.4999	49.4182	140.2043
49.36463	112.8163	49.28538	132.2695	49.26827	140.7189	49.37483	136.9379	49.30288	137.5004	49.43487	140.2037
49.3813	112.8109	49.30205	132.2696	49.28493	140.7135	49.3915	136.9352	49.31955	137.5001	49.45153	140.2006
49.39797	112.8128	49.31872	132.2708	49.3016	140.7156	49.40817	136.9343	49.33622	137.4979	49.4682	140.2009
49.41463	112.8093	49.33538	132.2663	49.31827	140.7125	49.42483	136.9349	49.35288	137.4976	49.48487	140.1984
49.4313	112.8063	49.35205	132.2714	49.33493	140.7101	49.4415	136.933	49.36955	137.497	49.50153	140.1998
49.44797	112.8077	49.36872	132.2687	49.3516	140.7097	49.45817	136.9331	49.38622	137.4967	49.5182	140.1968
49.46463	112.8047	49.38538	132.2698	49.36827	140.7082	49.47483	136.9305	49.40288	137.4933	49.53487	140.1964
49.4813	112.8	49.40205	132.2682	49.38493	140.7061	49.4915	136.9328	49.41955	137.4925	49.55153	140.1946
49.49797	112.7982	49.41872	132.2722	49.4016	140.7026	49.50817	136.9305	49.43622	137.4922	49.5682	140.1939
49.51463	112.7983	49.43538	132.2708	49.41827	140.7036	49.52483	136.9297	49.45288	137.4931	49.58487	140.1942
49.5313	112.7941	49.45205	132.2714	49.43493	140.7037	49.5415	136.9289	49.46955	137.491	49.60153	140.1937
49.54797	112.7934	49.46872	132.2715	49.4516	140.7002	49.55817	136.9289	49.48622	137.4911	49.6182	140.1911
49.56463	112.792	49.48538	132.2689	49.46827	140.7004	49.57483	136.9289	49.50288	137.4924	49.63487	140.1924
49.5813	112.7876	49.50205	132.2721	49.48493	140.6983	49.5915	136.9311	49.51955	137.4901	49.65153	140.1915
49.59797	112.7919	49.51872	132.272	49.5016	140.6969	49.60817	136.9278	49.53622	137.488	49.6682	140.1898
49.61463	112.7887	49.53538	132.2713	49.51827	140.698	49.62483	136.9303	49.55288	137.4863	49.68487	140.186
49.6313	112.7847	49.55205	132.2695	49.53493	140.6955	49.6415	136.9269	49.56955	137.4901	49.70153	140.1884
49.64797	112.7855	49.56872	132.2703	49.5516	140.6964	49.65817	136.9252	49.58622	137.4876	49.7182	140.1866
49.66463	112.7807	49.58538	132.2739	49.56827	140.6925	49.67483	136.9276	49.60288	137.4834	49.73487	140.1831
49.6813	112.7806	49.60205	132.2716	49.58493	140.6919	49.6915	136.9258	49.61955	137.4841	49.75153	140.1854
49.69797	112.779	49.61872	132.2737	49.6016	140.6906	49.70817	136.924	49.63622	137.4825	49.7682	140.1838
49.71463	112.7757	49.63538	132.2725	49.61827	140.6908	49.72483	136.9237	49.65288	137.4803	49.78487	140.1807
49.7313	112.7736	49.65205	132.2749	49.63493	140.6887	49.7415	136.9265	49.66955	137.4808	49.80153	140.181
49.74797	112.7713	49.66872	132.2733	49.6516	140.6834	49.75817	136.9219	49.68622	137.4799	49.8182	140.1802
49.76463	112.7677	49.68538	132.2742	49.66827	140.6844	49.77483	136.9239	49.70288	137.4827	49.83487	140.1778
49.7813	112.7648	49.70205	132.2761	49.68493	140.6845	49.7915	136.9209	49.71955	137.4801	49.85153	140.177
49.79797	112.7643	49.71872	132.2752	49.7016	140.6792	49.80817	136.9212	49.73622	137.4788	49.8682	140.1757
49.81463	112.7634	49.73538	132.2788	49.71827	140.6816	49.82483	136.9232	49.75288	137.477	49.88487	140.1757
49.8313	112.7588	49.75205	132.2777	49.73493	140.6789	49.8415	136.9224	49.76955	137.4792	49.90153	140.1743
49.84797	112.7584	49.76872	132.2802	49.7516	140.6787	49.85817	136.9213	49.78622	137.4772	49.9182	140.1721
49.86463	112.756	49.78538	132.2762	49.76827	140.6747	49.87483	136.9213	49.80288	137.4754	49.93487	140.1708
49.8813	112.7528	49.80205	132.28	49.78493	140.676	49.8915	136.92	49.81955	137.4753	49.95153	140.1681
49.89797	112.7497	49.81872	132.2817	49.8016	140.6714	49.90817	136.9206	49.83622	137.4754	49.9682	140.1678
49.91463	112.7511	49.83538	132.2803	49.81827	140.6745	49.92483	136.9204	49.85288	137.4755	49.98487	140.1661
49.9313	112.7471	49.85205	132.2834	49.83493	140.6714	49.9415	136.9184	49.86955	137.4745	50.00153	140.1643
49.94797	112.7462	49.86872	132.2813	49.8516	140.6698	49.95817	136.921	49.88622	137.4712	50.0182	140.1622
49.96463	112.7425	49.88538	132.2848	49.86827	140.6663	49.97483	136.9136	49.90288	137.4734	50.03487	140.1629
49.9813	112.7393	49.90205	132.2845	49.88493	140.6648	49.9915	136.9167	49.91955	137.4702	50.05153	140.1607
49.99797	112.7358	49.91872	132.2832	49.9016	140.6682	50.00817	136.9187	49.93622	137.469	50.0682	140.1616
50.01463	112.7363	49.93538	132.2841	49.91827	140.6659	50.02483	136.9165	49.95288	137.4706	50.08487	140.16
50.0313	112.7306	49.95205	132.2862	49.93493	140.6646	50.0415	136.9157	49.96955	137.4692	50.10153	140.1588
50.04797	112.7301	49.96872	132.2862	49.9516	140.6623	50.05817	136.9161	49.98622	137.4675	50.1182	140.1593
50.06463	112.7272	49.98538	132.2882	49.96827	140.6622	50.07483	136.9141	50.00288	137.4664	50.13487	140.1565
50.0813	112.7272	50.00205	132.2883	49.98493	140.6601	50.0915	136.9172	50.01955	137.4661	50.15153	140.1568
50.09797	112.7189	50.01872	132.2882	50.0016	140.66	50.10817	136.9128	50.03622	137.464	50.1682	140.1582
50.11463	112.7212	50.03538	132.2884	50.01827	140.6598	50.12483	136.9127	50.05288	137.4636	50.18487	140.1535
50.1313	112.7206	50.05205	132.29	50.03493	140.6595	50.1415	136.9135	50.06955	137.4632	50.20153	140.1539
50.14797	112.716	50.06872	132.2909	50.0516	140.659	50.15817	136.9118	50.08622	137.4624	50.2182	140.1539
50.16463	112.7139	50.08538	132.29	50.06827	140.6521	50.17483	136.9131	50.10288	137.4616	50.23487	140.1526

50.1813	112.7111	50.10205	132.2888	50.08493	140.6565	50.1915	136.9125	50.11955	137.4617	50.25153	140.1504
50.19797	112.7107	50.11872	132.2892	50.1016	140.6511	50.20817	136.913	50.13622	137.459	50.2682	140.1517
50.21463	112.7052	50.13538	132.2894	50.11827	140.6525	50.22483	136.9091	50.15288	137.4592	50.28487	140.1483
50.2313	112.7055	50.15205	132.2924	50.13493	140.6501	50.2415	136.9089	50.16955	137.4589	50.30153	140.1492
50.24797	112.7024	50.16872	132.2886	50.1516	140.6528	50.25817	136.9094	50.18622	137.4598	50.3182	140.1482
50.26463	112.6982	50.18538	132.2883	50.16827	140.6483	50.27483	136.9089	50.20288	137.4584	50.33487	140.1479
50.2813	112.6957	50.20205	132.2925	50.18493	140.6497	50.2915	136.9065	50.21955	137.4562	50.35153	140.1442
50.29797	112.6964	50.21872	132.2892	50.2016	140.6442	50.30817	136.9053	50.23622	137.4558	50.3682	140.1465
50.31463	112.693	50.23538	132.291	50.21827	140.6465	50.32483	136.9053	50.25288	137.4548	50.38487	140.1433
50.3313	112.6903	50.25205	132.2922	50.23493	140.643	50.3415	136.9048	50.26955	137.452	50.40153	140.1425
50.34797	112.6874	50.26872	132.2973	50.2516	140.645	50.35817	136.9042	50.28622	137.4539	50.4182	140.1415
50.36463	112.6836	50.28538	132.2924	50.26827	140.6412	50.37483	136.9048	50.30288	137.4503	50.43487	140.1398
50.3813	112.6834	50.30205	132.288	50.28493	140.6402	50.3915	136.9039	50.31955	137.4508	50.45153	140.1368
50.39797	112.6831	50.31872	132.2906	50.3016	140.6419	50.40817	136.9001	50.33622	137.4504	50.4682	140.1396
50.41463	112.6829	50.33538	132.294	50.31827	140.6406	50.42483	136.8999	50.35288	137.449	50.48487	140.1358
50.4313	112.6731	50.35205	132.2905	50.33493	140.6364	50.4415	136.9	50.36955	137.4489	50.50153	140.1405
50.44797	112.6763	50.36872	132.2907	50.3516	140.6361	50.45817	136.8989	50.38622	137.4493	50.5182	140.1362
50.46463	112.6716	50.38538	132.2905	50.36827	140.633	50.47483	136.8978	50.40288	137.4441	50.53487	140.1325
50.4813	112.6713	50.40205	132.2894	50.38493	140.6321	50.4915	136.8971	50.41955	137.4463	50.55153	140.1325
50.49797	112.6683	50.41872	132.2901	50.4016	140.6321	50.50817	136.896	50.43622	137.4437	50.5682	140.134
50.51463	112.6675	50.43538	132.2885	50.41827	140.6304	50.52483	136.8943	50.45288	137.4438	50.58487	140.1322
50.5313	112.6628	50.45205	132.2926	50.43493	140.6287	50.5415	136.8948	50.46955	137.4412	50.60153	140.1334
50.54797	112.6603	50.46872	132.2901	50.4516	140.6348	50.55817	136.8939	50.48622	137.4422	50.6182	140.1306
50.56463	112.6606	50.48538	132.2875	50.46827	140.6277	50.57483	136.8917	50.50288	137.4445	50.63487	140.1264
50.5813	112.6561	50.50205	132.2864	50.48493	140.6285	50.5915	136.893	50.51955	137.442	50.65153	140.129
50.59797	112.6538	50.51872	132.2868	50.5016	140.6226	50.60817	136.8917	50.53622	137.4402	50.6682	140.1257
50.61463	112.6506	50.53538	132.2877	50.51827	140.6234	50.62483	136.8936	50.55288	137.4375	50.68487	140.1274
50.6313	112.6476	50.55205	132.2881	50.53493	140.6241	50.6415	136.8872	50.56955	137.4377	50.70153	140.1258
50.64797	112.6477	50.56872	132.2854	50.5516	140.6218	50.65817	136.8894	50.58622	137.4397	50.7182	140.1231
50.66463	112.6448	50.58538	132.2859	50.56827	140.6197	50.67483	136.8891	50.60288	137.4384	50.73487	140.1267
50.6813	112.6444	50.60205	132.2848	50.58493	140.6208	50.6915	136.8858	50.61955	137.4359	50.75153	140.1206
50.69797	112.641	50.61872	132.2822	50.6016	140.6202	50.70817	136.8836	50.63622	137.4354	50.7682	140.1214
50.71463	112.6409	50.63538	132.2846	50.61827	140.6168	50.72483	136.8865	50.65288	137.4339	50.78487	140.1191
50.7313	112.6362	50.65205	132.2844	50.63493	140.6143	50.7415	136.8824	50.66955	137.4353	50.80153	140.1207
50.74797	112.6368	50.66872	132.2829	50.6516	140.6139	50.75817	136.8844	50.68622	137.4354	50.8182	140.1198
50.76463	112.6334	50.68538	132.2837	50.66827	140.6132	50.77483	136.8846	50.70288	137.4314	50.83487	140.1171
50.7813	112.6311	50.70205	132.2844	50.68493	140.6104	50.7915	136.8832	50.71955	137.4317	50.85153	140.1174
50.79797	112.6295	50.71872	132.2822	50.7016	140.6117	50.80817	136.8826	50.73622	137.4306	50.8682	140.1171
50.81463	112.6271	50.73538	132.2782	50.71827	140.611	50.82483	136.884	50.75288	137.4282	50.88487	140.115
50.8313	112.6284	50.75205	132.2828	50.73493	140.6068	50.8415	136.8804	50.76955	137.4289	50.90153	140.1142
50.84797	112.6244	50.76872	132.2799	50.7516	140.607	50.85817	136.8812	50.78622	137.4293	50.9182	140.1132
50.86463	112.6202	50.78538	132.2825	50.76827	140.6068	50.87483	136.8809	50.80288	137.4263	50.93487	140.1121
50.8813	112.6201	50.80205	132.2813	50.78493	140.6047	50.8915	136.8805	50.81955	137.4273	50.95153	140.1106
50.89797	112.6163	50.81872	132.2803	50.8016	140.6007	50.90817	136.8774	50.83622	137.4245	50.9682	140.1105
50.91463	112.615	50.83538	132.2804	50.81827	140.6034	50.92483	136.8775	50.85288	137.4247	50.98487	140.111
50.9313	112.6142	50.85205	132.2774	50.83493	140.5999	50.9415	136.8785	50.86955	137.4261	51.00153	140.1101
50.94797	112.6099	50.86872	132.2784	50.8516	140.5979	50.95817	136.8754	50.88622	137.4238	51.0182	140.1083
50.96463	112.6074	50.88538	132.2815	50.86827	140.5973	50.97483	136.8769	50.90288	137.4237	51.03487	140.1074
50.9813	112.6058	50.90205	132.2807	50.88493	140.5976	50.9915	136.8758	50.91955	137.4244	51.05153	140.1076
50.99797	112.6033	50.91872	132.2818	50.9016	140.5927	51.00817	136.8729	50.93622	137.4243	51.0682	140.1082
51.01463	112.6026	50.93538	132.2795	50.91827	140.5943	51.02483	136.874	50.95288	137.4224	51.08487	140.1065
51.0313	112.5989	50.95205	132.281	50.93493	140.5928	51.0415	136.8732	50.96955	137.4217	51.10153	140.1057
51.04797	112.5977	50.96872	132.2836	50.9516	140.592	51.05817	136.872	50.98622	137.422	51.1182	140.1021
51.06463	112.5957	50.98538	132.2828	50.96827	140.5918	51.07483	136.8701	51.00288	137.42	51.13487	140.1028
51.0813	112.5942	51.00205	132.2861	50.98493	140.5906	51.0915	136.8686	51.01955	137.4198	51.15153	140.1003
51.09797	112.5923	51.01872	132.287	51.0016	140.5862	51.10817	136.8695	51.03622	137.419	51.1682	140.1011
51.11463	112.5913	51.03538	132.2865	51.01827	140.5862	51.12483	136.869	51.05288	137.4152	51.18487	140.0997
51.1313	112.589	51.05205	132.2854	51.03493	140.584	51.1415	136.8695	51.06955	137.4161	51.20153	140.0987
51.14797	112.584	51.06872	132.288	51.0516	140.5829	51.15817	136.8658	51.08622	137.4152	51.2182	140.096
51.16463	112.5846	51.08538	132.2908	51.06827	140.5796	51.17483	136.8657	51.10288	137.4138	51.23487	140.0963
51.1813	112.5814	51.10205	132.2911	51.08493	140.5781	51.1915	136.8673	51.11955	137.4138	51.25153	140.0955
51.19797	112.5794	51.11872	132.2898	51.1016	140.5772	51.20817	136.8653	51.13622	137.4138	51.2682	140.0949
51.21463	112.579	51.13538	132.292	51.11827	140.5773	51.22483	136.8629	51.15288	137.4124	51.28487	140.0949
51.2313	112.574	51.15205	132.2932	51.13493	140.5765	51.2415	136.8613	51.16955	137.4107	51.30153	140.0934
51.24797	112.5744	51.16872	132.2953	51.1516	140.5756	51.25817	136.8616	51.18622	137.4119	51.3182	140.0925
51.26463	112.5695	51.18538	132.2942	51.16827	140.5734	51.27483	136.8617	51.20288	137.4088	51.33487	140.0924
51.2813	112.5699	51.20205	132.2957	51.18493	140.5736	51.2915	136.8621	51.21955	137.4089	51.35153	140.0905
51.29797	112.5663	51.21872	132.2971	51.2016	140.5742	51.30817	136.858	51.23622	137.4083	51.3682	140.091
51.31463	112.5666	51.23538	132.2984	51.21827	140.5744	51.32483	136.8596	51.25288	137.4083	51.38487	140.0902
51.3313	112.5633	51.25205	132.2981	51.23493	140.5672	51.3415	136.8612	51.26955	137.4063	51.40153	140.0899
51.34797	112.563	51.26872	132.2989	51.2516	140.5681	51.35817	136.8575	51.28622	137.4067	51.4182	140.0878
51.36463	112.5603	51.28538	132.2993	51.26827	140.5654	51.37483	136.8571	51.30288	137.4052	51.43487	140.0869
51.3813	112.557	51.30205	132.2983	51.28493	140.5667	51.3915	136.8576	51.31955	137.4046	51.45153	140.0871
51.39797	112.5545	51.31872	132.3013	51.3016	140.5652	51.40817	136.8557	51.33622	137.4051	51.4682	140.0859
51.41463	112.5568	51.33538	132.2988	51.31827	140.563	51.42483	136.8576	51.35288	137.4038	51.48487	140.0849
51.4313	112.552	51.35205	132.3028	51.33493	140.561	51.4415	136.8553	51.36955	137.4	51.50153	140.0844
51.44797	112.5482	51.36872	132.3031	51.3516	140.5623	51.45817	136.8534	51.38622	137.4036	51.5182	140.0805
51.46463	112.5456	51.38538	132.3025	51.36827	140.5562	51.47483	136.8523	51.40288	137.402	51.53487	140.0815
51.4813	112.545	51.40205	132.3044	51.38493	140.5571	51.4915	136.8529	51.41955	137.4018	51.55153	140.0822
51.49797	112.5442	51.41872	132.3056	51.4016	140.5573	51.50817	136.8524	51.43622	137.4005	51.5682	140.0817
51.51463	112.54	51.43538	132.3063	51.41827	140.5552	51.52483	136.8497	51.45288	137.3999	51.58487	140.0803
51.5313	112.5389	51.45205	132.3059	51.43493	140.5548	51.5415	136.8509	51.46955	137.3981	51.60153	140.0793
51.54797	112.5388	51.46872	132.3084	51.4516	140.5554	51.55817	136.8468	51.48622	137.3964	51.6182	140.0746
51.56463	112.5335	51.48538	132.3069	51.46827	140.5497	51.57483	136.8489	51.50288	137.3978	51.63487	140.0763
51.5813	112.5316	51.50205	132.305	51.48493	140.552	51.5915	136.8459	51.51955	137.3972	51.65153	140.0753
51.59797	112.5313	51.51872	132.3063	51.5016	140.5492	51.60817	136.8458	51.53622	137.3941	51.6682	140.0756
51.61463	112.5306	51.53538	132.3054	51.51827	140.5468	51.62483	136.8488	51.55288	137.3943	51.68487	140.0704
51.6313	112.5263	51.55205	132.3074	51.53493	140.546	51.6415	136.8448	51.56955	137.3948	51.70153	140.0718
51.64797	112.5271	51.56872	132.3069	51.5516	140.5448	51.65817	136.8454	51.58622	137.3919	51.7182	140.072
51.66463	112.524	51.58538	132.3074	51.56827	140.5439	51.67483	136.8433	51.60288	137.3901	51.73487	140.0688
51.6813	112.5216	51.60205	132.3047	51.58493	140.5411	51.6915	136.8437	51.61955	137.3908	51.75153	140.0684
51.69797	112.5159	51.61872	132.3059	51.6016	140.5394	51.70817	136.8438	51.63622	137.3896	51.7682	140.0672
51.71463	112.5155	51.63538	132.3058	51.61827	140.5378	51.72483	136.8403	51.65288	137.388	51.78487	140.0657
51.7313	112.5137	51.65205	132.3073	51.63493	140.538	51.7415	136.8409	51.66955	137.3901	51.80153	140.0668
51.74797	112.5141	51.66872	132.3063	51.6516	140.535	51.75817	136.8392	51.68622	137.3889	51.8182	140.0636
51.76463	112.5107	51.68538	132.3091	51.66827	140.5321	51.77483	136.8405	51.70288	137.3883	51.83487	140.062
51.7813	112.5081	51.70205	132.3083	51.68493	140.5317	51.7915	136.8386	51.71955	137.3893	51.85153	140.0614

51.79797	112.5037	51.71872	132.3082	51.7016	140.5307	51.80817	136.837	51.73622	137.3851	51.8682	140.0613
51.81463	112.5042	51.73538	132.3096	51.71827	140.53	51.82483	136.8342	51.75288	137.386	51.88487	140.0609
51.8313	112.5022	51.75205	132.3084	51.73493	140.5262	51.8415	136.8336	51.76955	137.3859	51.90153	140.0603
51.84797	112.4979	51.76872	132.309	51.7516	140.5265	51.85817	136.8359	51.78622	137.387	51.9182	140.0581
51.86463	112.498	51.78538	132.3068	51.76827	140.5231	51.87483	136.8308	51.80288	137.385	51.93487	140.0583
51.8813	112.4972	51.80205	132.3079	51.78493	140.5217	51.8915	136.8331	51.81955	137.3836	51.95153	140.0557
51.89797	112.492	51.81872	132.3099	51.8016	140.5186	51.90817	136.831	51.83622	137.3796	51.9682	140.0552
51.91463	112.4898	51.83538	132.3089	51.81827	140.5185	51.92483	136.8323	51.85288	137.3838	51.98487	140.0538
51.9313	112.4875	51.85205	132.3096	51.83493	140.5157	51.9415	136.8284	51.86955	137.3835	52.00153	140.0526
51.94797	112.4848	51.86872	132.3108	51.8516	140.5163	51.95817	136.8279	51.88622	137.3781	52.0182	140.0534
51.96463	112.4811	51.88538	132.3097	51.86827	140.5154	51.97483	136.8283	51.90288	137.3781	52.03487	140.0519
51.9813	112.4792	51.90205	132.3104	51.88493	140.5136	51.9915	136.8264	51.91955	137.3793	52.05153	140.0493
51.99797	112.4758	51.91872	132.3108	51.9016	140.513	52.00817	136.8245	51.93622	137.3773	52.0682	140.0486
52.01463	112.4747	51.93538	132.3125	51.91827	140.5113	52.02483	136.8252	51.95288	137.377	52.08487	140.0469
52.0313	112.4714	51.95205	132.3152	51.93493	140.5057	52.0415	136.8261	51.96955	137.3793	52.10153	140.0478
52.04797	112.4707	51.96872	132.3149	51.9516	140.5077	52.05817	136.8268	51.98622	137.3749	52.1182	140.0447
52.06463	112.4668	51.98538	132.3131	51.96827	140.5052	52.07483	136.8229	52.00288	137.3742	52.13487	140.0436
52.0813	112.4644	52.00205	132.3135	51.98493	140.5052	52.0915	136.8222	52.01955	137.3746	52.15153	140.043
52.09797	112.4617	52.01872	132.3159	52.0016	140.4999	52.10817	136.8218	52.03622	137.3739	52.1682	140.041
52.11463	112.4607	52.03538	132.3163	52.01827	140.4995	52.12483	136.8233	52.05288	137.373	52.18487	140.0419
52.1313	112.4568	52.05205	132.3171	52.03493	140.4988	52.1415	136.8212	52.06955	137.3705	52.20153	140.0382
52.14797	112.4574	52.06872	132.3186	52.0516	140.4987	52.15817	136.8191	52.08622	137.3699	52.2182	140.0373
52.16463	112.453	52.08538	132.3213	52.06827	140.4958	52.17483	136.8202	52.10288	137.37	52.23487	140.0386
52.1813	112.4491	52.10205	132.321	52.08493	140.4984	52.1915	136.8162	52.11955	137.371	52.25153	140.0383
52.19797	112.4476	52.11872	132.3242	52.1016	140.4945	52.20817	136.819	52.13622	137.3703	52.2682	140.0369
52.21463	112.4465	52.13538	132.3238	52.11827	140.4949	52.22483	136.8191	52.15288	137.3685	52.28487	140.0353
52.2313	112.4455	52.15205	132.3249	52.13493	140.492	52.2415	136.8159	52.16955	137.3667	52.30153	140.0337
52.24797	112.442	52.16872	132.3272	52.1516	140.4953	52.25817	136.819	52.18622	137.369	52.3182	140.037
52.26463	112.4403	52.18538	132.327	52.16827	140.4879	52.27483	136.8166	52.20288	137.3694	52.33487	140.034
52.2813	112.4391	52.20205	132.3311	52.18493	140.4894	52.2915	136.8155	52.21955	137.3669	52.35153	140.0308
52.29797	112.4349	52.21872	132.3292	52.2016	140.4895	52.30817	136.8158	52.23622	137.3648	52.3682	140.0313
52.31463	112.4347	52.23538	132.3285	52.21827	140.488	52.32483	136.8178	52.25288	137.3658	52.38487	140.0326
52.3313	112.4327	52.25205	132.33	52.23493	140.4849	52.3415	136.8157	52.26955	137.3628	52.40153	140.0292
52.34797	112.4305	52.26872	132.3337	52.2516	140.483	52.35817	136.8135	52.28622	137.3627	52.4182	140.0264
52.36463	112.4293	52.28538	132.3306	52.26827	140.4827	52.37483	136.8129	52.30288	137.3626	52.43487	140.0264
52.3813	112.4273	52.30205	132.333	52.28493	140.4801	52.3915	136.8142	52.31955	137.3605	52.45153	140.0265
52.39797	112.4219	52.31872	132.3348	52.3016	140.4787	52.40817	136.8118	52.33622	137.3605	52.4682	140.0269
52.41463	112.4208	52.33538	132.3353	52.31827	140.4792	52.42483	136.8114	52.35288	137.3597	52.48487	140.0249
52.4313	112.4174	52.35205	132.3389	52.33493	140.4792	52.4415	136.8103	52.36955	137.3581	52.50153	140.0239
52.44797	112.4168	52.36872	132.3382	52.3516	140.4785	52.45817	136.8108	52.38622	137.3593	52.5182	140.0245
52.46463	112.4145	52.38538	132.34	52.36827	140.4752	52.47483	136.8074	52.40288	137.3577	52.53487	140.0265
52.4813	112.414	52.40205	132.3419	52.38493	140.473	52.4915	136.8074	52.41955	137.3565	52.55153	140.0221
52.49797	112.4098	52.41872	132.3448	52.4016	140.4721	52.50817	136.8062	52.43622	137.3554	52.5682	140.022
52.51463	112.4097	52.43538	132.3428	52.41827	140.4715	52.52483	136.8056	52.45288	137.3537	52.58487	140.022
52.5313	112.4026	52.45205	132.3439	52.43493	140.47	52.5415	136.8071	52.46955	137.3509	52.60153	140.0189
52.54797	112.404	52.46872	132.3447	52.4516	140.4683	52.55817	136.8056	52.48622	137.352	52.6182	140.0182
52.56463	112.4033	52.48538	132.3474	52.46827	140.4679	52.57483	136.8036	52.50288	137.35	52.63487	140.0172
52.5813	112.4022	52.50205	132.3519	52.48493	140.4685	52.5915	136.8002	52.51955	137.3496	52.65153	140.0171
52.59797	112.3993	52.51872	132.3515	52.5016	140.4644	52.60817	136.7989	52.53622	137.3464	52.6682	140.0137
52.61463	112.3963	52.53538	132.3533	52.51827	140.4631	52.62483	136.8011	52.55288	137.3502	52.68487	140.0123
52.6313	112.3917	52.55205	132.3511	52.53493	140.4594	52.6415	136.7966	52.56955	137.3468	52.70153	140.0133
52.64797	112.3937	52.56872	132.3536	52.5516	140.4588	52.65817	136.7973	52.58622	137.3481	52.7182	140.0129
52.66463	112.3894	52.58538	132.3517	52.56827	140.4563	52.67483	136.7973	52.60288	137.3453	52.73487	140.0106
52.6813	112.3878	52.60205	132.3514	52.58493	140.4569	52.6915	136.7951	52.61955	137.3441	52.75153	140.0117
52.69797	112.3853	52.61872	132.3542	52.6016	140.4539	52.70817	136.7958	52.63622	137.3433	52.7682	140.0086
52.71463	112.3822	52.63538	132.3575	52.61827	140.4535	52.72483	136.7945	52.65288	137.346	52.78487	140.0092
52.7313	112.3804	52.65205	132.3565	52.63493	140.4528	52.7415	136.7929	52.66955	137.3444	52.80153	140.0064
52.74797	112.3785	52.66872	132.3561	52.6516	140.4512	52.75817	136.7889	52.68622	137.345	52.8182	140.0067
52.76463	112.3728	52.68538	132.3591	52.66827	140.4511	52.77483	136.7914	52.70288	137.343	52.83487	140.0048
52.7813	112.3744	52.70205	132.3586	52.68493	140.4471	52.7915	136.7921	52.71955	137.3428	52.85153	140.0062
52.79797	112.3732	52.71872	132.3624	52.7016	140.4461	52.80817	136.7884	52.73622	137.34	52.8682	140.0037
52.81463	112.3679	52.73538	132.3571	52.71827	140.4463	52.82483	136.7899	52.75288	137.3408	52.88487	140.0032
52.8313	112.3659	52.75205	132.3624	52.73493	140.4441	52.8415	136.7885	52.76955	137.3399	52.90153	139.9989
52.84797	112.3657	52.76872	132.3622	52.7516	140.4434	52.85817	136.7859	52.78622	137.339	52.9182	139.9999
52.86463	112.3615	52.78538	132.3636	52.76827	140.4387	52.87483	136.7851	52.80288	137.3365	52.93487	140.0008
52.8813	112.362	52.80205	132.3683	52.78493	140.439	52.8915	136.7832	52.81955	137.3383	52.95153	140.0003
52.89797	112.359	52.81872	132.3663	52.8016	140.4339	52.90817	136.783	52.83622	137.3367	52.9682	139.9979
52.91463	112.355	52.83538	132.3678	52.81827	140.4352	52.92483	136.7845	52.85288	137.338	52.98487	139.9988
52.9313	112.3543	52.85205	132.3678	52.83493	140.4346	52.9415	136.7792	52.86955	137.3354	53.00153	139.9965
52.94797	112.3533	52.86872	132.369	52.8516	140.4324	52.95817	136.7791	52.88622	137.3355	53.0182	139.9974
52.96463	112.3511	52.88538	132.3694	52.86827	140.4286	52.97483	136.7801	52.90288	137.333	53.03487	139.9945
52.9813	112.3473	52.90205	132.3717	52.88493	140.428	52.9915	136.7792	52.91955	137.3341	53.05153	139.9943
52.99797	112.3481	52.91872	132.3764	52.9016	140.4271	53.00817	136.7762	52.93622	137.3314	53.0682	139.9935
53.01463	112.3439	52.93538	132.3785	52.91827	140.4253	53.02483	136.7767	52.95288	137.3305	53.08487	139.9926
53.0313	112.3418	52.95205	132.3755	52.93493	140.4255	53.0415	136.7729	52.96955	137.3299	53.10153	139.9903
53.04797	112.3392	52.96872	132.377	52.9516	140.4222	53.05817	136.7729	52.98622	137.33	53.1182	139.9902
53.06463	112.3366	52.98538	132.3835	52.96827	140.4197	53.07483	136.7731	53.00288	137.3285	53.13487	139.9874
53.0813	112.3345	53.00205	132.3787	52.98493	140.4192	53.0915	136.7713	53.01955	137.3263	53.15153	139.9871
53.09797	112.3328	53.01872	132.3808	53.0016	140.4162	53.10817	136.7701	53.03622	137.3272	53.1682	139.9863
53.11463	112.3312	53.03538	132.3823	53.01827	140.4192	53.12483	136.7684	53.05288	137.3246	53.18487	139.986
53.1313	112.3287	53.05205	132.3794	53.03493	140.4142	53.1415	136.768	53.06955	137.3241	53.20153	139.9863
53.14797	112.3283	53.06872	132.3815	53.0516	140.411	53.15817	136.7671	53.08622	137.3254	53.2182	139.9851
53.16463	112.3267	53.08538	132.381	53.06827	140.4117	53.17483	136.7701	53.10288	137.3217	53.23487	139.984
53.1813	112.3224	53.10205	132.3789	53.08493	140.407	53.1915	136.7656	53.11955	137.3206	53.25153	139.984
53.19797	112.3185	53.11872	132.3854	53.1016	140.4062	53.20817	136.7629	53.13622	137.3215	53.2682	139.9833
53.21463	112.3196	53.13538	132.3838	53.11827	140.4057	53.22483	136.764	53.15288	137.3217	53.28487	139.9823
53.2313	112.3181	53.15205	132.3864	53.13493	140.4033	53.2415	136.7618	53.16955	137.321	53.30153	139.9807
53.24797	112.316	53.16872	132.3847	53.1516	140.3998	53.25817	136.7602	53.18622	137.3186	53.3182	139.9803
53.26463	112.3115	53.18538	132.3865	53.16827	140.4002	53.27483	136.7564	53.20288	137.3177	53.33487	139.9799
53.2813	112.3119	53.20205	132.3889	53.18493	140.3979	53.2915	136.7573	53.21955	137.3157	53.35153	139.9791
53.29797	112.3103	53.21872	132.3899	53.2016	140.3941	53.30817	136.7566	53.23622	137.3147	53.3682	139.9793
53.31463	112.3059	53.23538	132.3868	53.21827	140.3944	53.32483	136.7547	53.25288	137.3161	53.38487	139.9764
53.3313	112.3057	53.25205	132.3916	53.23493	140.3953	53.3415	136.7542	53.26955	137.315	53.40153	139.9785
53.34797	112.3018	53.26872	132.3914	53.2516	140.3921	53.35817	136.7538	53.28622	137.3139	53.4182	139.9756
53.36463	112.3001	53.28538	132.3928	53.26827	140.3899	53.37483	136.7502	53.30288	137.3141	53.43487	139.9762
53.3813	112.2991	53.30205	132.3962	53.28493	140.3874	53.3915	136.7492	53.31955	137.3135	53.45153	139.9737
53.39797	112.2967	53.31872	132.3985	53.3016	140.3877	53.40817	136.7502	53.33622	137.3113	53.4682	139.9726

53.41463	112.2913	53.33538	132.3965	53.31827	140.3851	53.42483	136.7461	53.35288	137.3121	53.48487	139.975
53.4313	112.2897	53.35205	132.3997	53.33493	140.3835	53.4415	136.7461	53.36955	137.3111	53.50153	139.9716
53.44797	112.2878	53.36872	132.4018	53.3516	140.382	53.45817	136.7458	53.38622	137.3111	53.5182	139.9712
53.46463	112.2867	53.38538	132.4038	53.36827	140.3818	53.47483	136.7425	53.40288	137.3103	53.53487	139.9694
53.4813	112.2859	53.40205	132.4058	53.38493	140.3782	53.4915	136.7442	53.41955	137.3101	53.55153	139.9704
53.49797	112.2817	53.41872	132.4053	53.4016	140.375	53.50817	136.7419	53.43622	137.3069	53.5682	139.9688
53.51463	112.2807	53.43538	132.4071	53.41827	140.3756	53.52483	136.7396	53.45288	137.3085	53.58487	139.9688
53.5313	112.2751	53.45205	132.4098	53.43493	140.3712	53.5415	136.7384	53.46955	137.3076	53.60153	139.9687
53.54797	112.2782	53.46872	132.4096	53.4516	140.3718	53.55817	136.7395	53.48622	137.3085	53.6182	139.9674
53.56463	112.2763	53.48538	132.4132	53.46827	140.3732	53.57483	136.7386	53.50288	137.3083	53.63487	139.9662
53.5813	112.271	53.50205	132.4154	53.48493	140.3693	53.5915	136.7363	53.51955	137.3053	53.65153	139.9662
53.59797	112.2709	53.51872	132.4187	53.5016	140.369	53.60817	136.7379	53.53622	137.3039	53.6682	139.9661
53.61463	112.2693	53.53538	132.4205	53.51827	140.3661	53.62483	136.7368	53.55288	137.3071	53.68487	139.965
53.6313	112.2689	53.55205	132.4189	53.53493	140.3663	53.6415	136.7341	53.56955	137.3056	53.70153	139.9636
53.64797	112.2651	53.56872	132.4221	53.5516	140.363	53.65817	136.7309	53.58622	137.3022	53.7182	139.9649
53.66463	112.2641	53.58538	132.4264	53.56827	140.3644	53.67483	136.7328	53.60288	137.3032	53.73487	139.9617
53.6813	112.2544	53.60205	132.4279	53.58493	140.3611	53.6915	136.7323	53.61955	137.3037	53.75153	139.9616
53.69797	112.2622	53.61872	132.4291	53.6016	140.3636	53.70817	136.732	53.63622	137.2997	53.7682	139.9622
53.71463	112.2614	53.63538	132.43	53.61827	140.3585	53.72483	136.7291	53.65288	137.302	53.78487	139.9585
53.7313	112.2535	53.65205	132.4301	53.63493	140.3582	53.7415	136.7319	53.66955	137.2977	53.80153	139.9583
53.74797	112.255	53.66872	132.4333	53.6516	140.3572	53.75817	136.7292	53.68622	137.2996	53.8182	139.9584
53.76463	112.2501	53.68538	132.4346	53.66827	140.3562	53.77483	136.7273	53.70288	137.2998	53.83487	139.9552
53.7813	112.2536	53.70205	132.4361	53.68493	140.354	53.7915	136.7289	53.71955	137.3004	53.85153	139.9568
53.79797	112.247	53.71872	132.4367	53.7016	140.3535	53.80817	136.7259	53.73622	137.2959	53.8682	139.9527
53.81463	112.2442	53.73538	132.4382	53.71827	140.3593	53.82483	136.725	53.75288	137.2966	53.88487	139.9526
53.8313	112.2448	53.75205	132.4389	53.73493	140.3504	53.8415	136.7245	53.76955	137.2956	53.90153	139.951
53.84797	112.2424	53.76872	132.4414	53.7516	140.3489	53.85817	136.7226	53.78622	137.2924	53.9182	139.9521
53.86463	112.241	53.78538	132.4412	53.76827	140.348	53.87483	136.7221	53.80288	137.2916	53.93487	139.9517
53.8813	112.2357	53.80205	132.4418	53.78493	140.349	53.8915	136.7196	53.81955	137.292	53.95153	139.9505
53.89797	112.2367	53.81872	132.4451	53.8016	140.3493	53.90817	136.721	53.83622	137.2901	53.9682	139.9473
53.91463	112.2337	53.83538	132.4466	53.81827	140.3477	53.92483	136.7198	53.85288	137.2916	53.98487	139.9473
53.9313	112.2325	53.85205	132.4476	53.83493	140.3458	53.9415	136.7182	53.86955	137.2895	54.00153	139.9475
53.94797	112.2286	53.86872	132.4512	53.8516	140.3438	53.95817	136.7179	53.88622	137.2896	54.0182	139.9463
53.96463	112.2274	53.88538	132.4504	53.86827	140.342	53.97483	136.72	53.90288	137.2859	54.03487	139.9434
53.9813	112.2272	53.90205	132.4523	53.88493	140.3401	53.9915	136.7168	53.91955	137.2875	54.05153	139.9449
53.99797	112.2202	53.91872	132.4543	53.9016	140.3427	54.00817	136.7156	53.93622	137.2861	54.0682	139.9427
54.01463	112.2204	53.93538	132.455	53.91827	140.3389	54.02483	136.7182	53.95288	137.2847	54.08487	139.9435
54.0313	112.2198	53.95205	132.451	53.93493	140.3408	54.0415	136.7154	53.96955	137.2855	54.10153	139.9394
54.04797	112.2168	53.96872	132.4597	53.9516	140.3356	54.05817	136.7141	53.98622	137.2833	54.1182	139.9385
54.06463	112.2149	53.98538	132.4549	53.96827	140.3347	54.07483	136.7117	54.00288	137.2825	54.13487	139.936
54.0813	112.2128	54.00205	132.4614	53.98493	140.3314	54.0915	136.7154	54.01955	137.2831	54.15153	139.935
54.09797	112.2101	54.01872	132.4606	54.0016	140.3325	54.10817	136.7142	54.03622	137.2814	54.1682	139.9382
54.11463	112.2089	54.03538	132.461	54.01827	140.3344	54.12483	136.7104	54.05288	137.2795	54.18487	139.9354
54.1313	112.2062	54.05205	132.466	54.03493	140.3307	54.1415	136.7115	54.06955	137.278	54.20153	139.9325
54.14797	112.2044	54.06872	132.4657	54.0516	140.3292	54.15817	136.7094	54.08622	137.2798	54.2182	139.9351
54.16463	112.2015	54.08538	132.4689	54.06827	140.3312	54.17483	136.7085	54.10288	137.2792	54.23487	139.9305
54.1813	112.2006	54.10205	132.4653	54.08493	140.3265	54.1915	136.7096	54.11955	137.278	54.25153	139.9328
54.19797	112.1996	54.11872	132.4689	54.1016	140.3259	54.20817	136.7084	54.13622	137.277	54.2682	139.9292
54.21463	112.1915	54.13538	132.4737	54.11827	140.326	54.22483	136.7042	54.15288	137.2752	54.28487	139.9284
54.2313	112.192	54.15205	132.4682	54.13493	140.3216	54.2415	136.7049	54.16955	137.2748	54.30153	139.9267
54.24797	112.1889	54.16872	132.4718	54.1516	140.3224	54.25817	136.7056	54.18622	137.2747	54.3182	139.9279
54.26463	112.1857	54.18538	132.4721	54.16827	140.3218	54.27483	136.7035	54.20288	137.2734	54.33487	139.9275
54.2813	112.1893	54.20205	132.4798	54.18493	140.3203	54.2915	136.7013	54.21955	137.2705	54.35153	139.9254
54.29797	112.1853	54.21872	132.4745	54.2016	140.3187	54.30817	136.699	54.23622	137.2723	54.3682	139.925
54.31463	112.1825	54.23538	132.4775	54.21827	140.3202	54.32483	136.7012	54.25288	137.2726	54.38487	139.9252
54.3313	112.1801	54.25205	132.4781	54.23493	140.316	54.3415	136.698	54.26955	137.268	54.40153	139.9236
54.34797	112.1781	54.26872	132.4806	54.2516	140.3148	54.35817	136.6988	54.28622	137.2685	54.4182	139.9196
54.36463	112.1763	54.28538	132.4812	54.26827	140.3153	54.37483	136.7005	54.30288	137.2665	54.43487	139.9235
54.3813	112.1745	54.30205	132.4834	54.28493	140.3113	54.3915	136.6949	54.31955	137.269	54.45153	139.92
54.39797	112.1765	54.31872	132.4851	54.3016	140.3107	54.40817	136.6961	54.33622	137.2664	54.4682	139.9185
54.41463	112.1725	54.33538	132.4878	54.31827	140.3095	54.42483	136.6923	54.35288	137.2679	54.48487	139.9177
54.4313	112.1714	54.35205	132.488	54.33493	140.3077	54.4415	136.6935	54.36955	137.2653	54.50153	139.9183
54.44797	112.1667	54.36872	132.4908	54.3516	140.3041	54.45817	136.6925	54.38622	137.2661	54.5182	139.9166
54.46463	112.1665	54.38538	132.4923	54.36827	140.3067	54.47483	136.6905	54.40288	137.2648	54.53487	139.9161
54.4813	112.1652	54.40205	132.4939	54.38493	140.305	54.4915	136.6901	54.41955	137.2635	54.55153	139.9164
54.49797	112.1615	54.41872	132.4955	54.4016	140.3006	54.50817	136.6891	54.43622	137.2641	54.5682	139.9125
54.51463	112.1584	54.43538	132.4988	54.41827	140.3006	54.52483	136.6886	54.45288	137.2646	54.58487	139.9151
54.5313	112.1581	54.45205	132.4985	54.43493	140.2995	54.5415	136.6858	54.46955	137.264	54.60153	139.9143
54.54797	112.1551	54.46872	132.5004	54.4516	140.2947	54.55817	136.685	54.48622	137.2604	54.6182	139.9134
54.56463	112.1567	54.48538	132.502	54.46827	140.2982	54.57483	136.6829	54.50288	137.2621	54.63487	139.9124
54.5813	112.1513	54.50205	132.5043	54.48493	140.2946	54.5915	136.6831	54.51955	137.2616	54.65153	139.9117
54.59797	112.1526	54.51872	132.5053	54.5016	140.2947	54.60817	136.6816	54.53622	137.2614	54.6682	139.9084
54.61463	112.1492	54.53538	132.5044	54.51827	140.2923	54.62483	136.6839	54.55288	137.2605	54.68487	139.904
54.6313	112.148	54.55205	132.5101	54.53493	140.2938	54.6415	136.6823	54.56955	137.258	54.70153	139.9061
54.64797	112.1437	54.56872	132.5099	54.5516	140.2886	54.65817	136.6774	54.58622	137.2597	54.7182	139.9075
54.66463	112.1414	54.58538	132.5122	54.56827	140.2867	54.67483	136.677	54.60288	137.2566	54.73487	139.9043
54.6813	112.1405	54.60205	132.5134	54.58493	140.2873	54.6915	136.6752	54.61955	137.2575	54.75153	139.9038
54.69797	112.1371	54.61872	132.5136	54.6016	140.2858	54.70817	136.6722	54.63622	137.2568	54.7682	139.9049
54.71463	112.1369	54.63538	132.5146	54.61827	140.2844	54.72483	136.6756	54.65288	137.2555	54.78487	139.9019
54.7313	112.1357	54.65205	132.5184	54.63493	140.2841	54.7415	136.6727	54.66955	137.2543	54.80153	139.9004
54.74797	112.1327	54.66872	132.5196	54.6516	140.2797	54.75817	136.6707	54.68622	137.2535	54.8182	139.9029
54.76463	112.13	54.68538	132.5179	54.66827	140.2797	54.77483	136.6701	54.70288	137.2522	54.83487	139.9018
54.7813	112.1281	54.70205	132.5196	54.68493	140.28	54.7915	136.6667	54.71955	137.2528	54.85153	139.8993
54.79797	112.1244	54.71872	132.5208	54.7016	140.2792	54.80817	136.6682	54.73622	137.2503	54.8682	139.8977
54.81463	112.1254	54.73538	132.5238	54.71827	140.2776	54.82483	136.6676	54.75288	137.25	54.88487	139.8978
54.8313	112.1238	54.75205	132.5263	54.73493	140.2754	54.8415	136.6666	54.76955	137.2494	54.90153	139.8991
54.84797	112.1188	54.76872	132.5302	54.7516	140.275	54.85817	136.6644	54.78622	137.2481	54.9182	139.8954
54.86463	112.1166	54.78538	132.5247	54.76827	140.273	54.87483	136.6669	54.80288	137.2472	54.93487	139.8945
54.8813	112.1171	54.80205	132.5285	54.78493	140.2713	54.8915	136.6642	54.81955	137.2442	54.95153	139.8943
54.89797	112.1148	54.81872	132.5305	54.8016	140.2732	54.90817	136.6615	54.83622	137.247	54.9682	139.8938
54.91463	112.1105	54.83538	132.5312	54.81827	140.2683	54.92483	136.6604	54.85288	137.246	54.98487	139.8922
54.9313	112.1109	54.85205	132.5345	54.83493	140.268	54.9415	136.6639	54.86955	137.2456	55.00153	139.8912
54.94797	112.1052	54.86872	132.5344	54.8516	140.2656	54.95817	136.6589	54.88622	137.2436	55.0182	139.8902
54.96463	112.108	54.88538	132.5365	54.86827	140.2668	54.97483	136.6602	54.90288	137.2446	55.03487	139.8892
54.9813	112.105	54.90205	132.5369	54.88493	140.2645	54.9915	136.6602	54.91955	137.2432	55.05153	139.8904
54.99797	112.1034	54.91872	132.5367	54.9016	140.2626	55.00817	136.6558	54.93622	137.2422	55.0682	139.8879
55.01463	112.1007	54.93538	132.5403	54.91827	140.2649	55.02483	136.6561	54.95288	137.2401	55.08487	139.8878

55.0313	112.0996	54.95205	132.5391	54.93493	140.2642	55.0415	136.6568	54.96955	137.2406	55.10153	139.886
55.04797	112.096	54.96872	132.5397	54.9516	140.2608	55.05817	136.6552	54.98622	137.241	55.1182	139.8877
55.06463	112.0957	54.98538	132.5404	54.96827	140.2605	55.07483	136.652	55.00288	137.2385	55.13487	139.8841
55.0813	112.0918	55.00205	132.5413	54.98493	140.2566	55.0915	136.653	55.01955	137.2369	55.15153	139.8831
55.09797	112.0893	55.01872	132.5456	55.0016	140.2557	55.10817	136.6525	55.03622	137.2369	55.1682	139.8832
55.11463	112.0897	55.03538	132.5465	55.01827	140.2554	55.12483	136.6515	55.05288	137.2375	55.18487	139.884
55.1313	112.085	55.05205	132.5466	55.03493	140.2555	55.1415	136.6508	55.06955	137.2348	55.20153	139.8812
55.14797	112.0839	55.06872	132.5474	55.0516	140.2512	55.15817	136.6451	55.08622	137.2331	55.2182	139.8814
55.16463	112.0823	55.08538	132.5474	55.06827	140.2541	55.17483	136.6471	55.10288	137.2324	55.23487	139.8816
55.1813	112.079	55.10205	132.5523	55.08493	140.2511	55.1915	136.6465	55.11955	137.2311	55.25153	139.8795
55.19797	112.0771	55.11872	132.5513	55.1016	140.2495	55.20817	136.6429	55.13622	137.2279	55.2682	139.8799
55.21463	112.076	55.13538	132.5518	55.11827	140.2466	55.22483	136.6435	55.15288	137.2303	55.28487	139.8806
55.2313	112.0735	55.15205	132.5535	55.13493	140.2471	55.2415	136.6428	55.16955	137.2294	55.30153	139.8787
55.24797	112.0733	55.16872	132.5556	55.1516	140.2433	55.25817	136.6408	55.18622	137.2278	55.3182	139.8777
55.26463	112.069	55.18538	132.5588	55.16827	140.2467	55.27483	136.6432	55.20288	137.2267	55.33487	139.8789
55.2813	112.0688	55.20205	132.5555	55.18493	140.2448	55.2915	136.6396	55.21955	137.2277	55.35153	139.8767
55.29797	112.0661	55.21872	132.5568	55.2016	140.2435	55.30817	136.6415	55.23622	137.2237	55.3682	139.8775
55.31463	112.0646	55.23538	132.5566	55.21827	140.2396	55.32483	136.6385	55.25288	137.2254	55.38487	139.8759
55.3313	112.0607	55.25205	132.5613	55.23493	140.2411	55.3415	136.6364	55.26955	137.2234	55.40153	139.8711
55.34797	112.0616	55.26872	132.5633	55.2516	140.2414	55.35817	136.6357	55.28622	137.2244	55.4182	139.8726
55.36463	112.0574	55.28538	132.5648	55.26827	140.2376	55.37483	136.6364	55.30288	137.2215	55.43487	139.8726
55.3813	112.0545	55.30205	132.565	55.28493	140.2364	55.3915	136.6349	55.31955	137.2221	55.45153	139.8706
55.39797	112.0513	55.31872	132.5696	55.3016	140.2358	55.40817	136.6363	55.33622	137.2199	55.4682	139.8698
55.41463	112.0523	55.33538	132.5709	55.31827	140.2358	55.42483	136.6323	55.35288	137.2192	55.48487	139.8676
55.4313	112.0501	55.35205	132.5718	55.33493	140.2344	55.4415	136.6358	55.36955	137.2185	55.50153	139.8677
55.44797	112.0471	55.36872	132.5746	55.3516	140.2332	55.45817	136.632	55.38622	137.2161	55.5182	139.8664
55.46463	112.0462	55.38538	132.5719	55.36827	140.2331	55.47483	136.6309	55.40288	137.2192	55.53487	139.8656
55.4813	112.0439	55.40205	132.5805	55.38493	140.2307	55.4915	136.6294	55.41955	137.2181	55.55153	139.8663
55.49797	112.0407	55.41872	132.5803	55.4016	140.2281	55.50817	136.6276	55.43622	137.2156	55.5682	139.8639
55.51463	112.0388	55.43538	132.5807	55.41827	140.2279	55.52483	136.6251	55.45288	137.2131	55.58487	139.8627
55.5313	112.0376	55.45205	132.5807	55.43493	140.2261	55.5415	136.625	55.46955	137.2153	55.60153	139.8619
55.54797	112.0335	55.46872	132.5861	55.4516	140.2244	55.55817	136.6196	55.48622	137.2124	55.6182	139.8587
55.56463	112.033	55.48538	132.5855	55.46827	140.2254	55.57483	136.6229	55.50288	137.2113	55.63487	139.8589
55.5813	112.0295	55.50205	132.5879	55.48493	140.2262	55.5915	136.6263	55.51955	137.2136	55.65153	139.8614
55.59797	112.0269	55.51872	132.587	55.5016	140.2207	55.60817	136.6235	55.53622	137.2069	55.6682	139.8576
55.61463	112.026	55.53538	132.5916	55.51827	140.2212	55.62483	136.6214	55.55288	137.2081	55.68487	139.8565
55.6313	112.0235	55.55205	132.595	55.53493	140.2217	55.6415	136.6221	55.56955	137.2068	55.70153	139.8566
55.64797	112.0215	55.56872	132.594	55.5516	140.2169	55.65817	136.6226	55.58622	137.2096	55.7182	139.8554
55.66463	112.0216	55.58538	132.5969	55.56827	140.2176	55.67483	136.6197	55.60288	137.2042	55.73487	139.855
55.6813	112.0169	55.60205	132.5985	55.58493	140.2147	55.6915	136.6167	55.61955	137.209	55.75153	139.8549
55.69797	112.0159	55.61872	132.6018	55.6016	140.2146	55.70817	136.6172	55.63622	137.205	55.7682	139.8516
55.71463	112.0145	55.63538	132.6018	55.61827	140.2091	55.72483	136.6148	55.65288	137.2041	55.78487	139.8529
55.7313	112.014	55.65205	132.6044	55.63493	140.2098	55.7415	136.6127	55.66955	137.2029	55.80153	139.8518
55.74797	112.0114	55.66872	132.6056	55.6516	140.2117	55.75817	136.611	55.68622	137.1998	55.8182	139.8498
55.76463	112.008	55.68538	132.6058	55.66827	140.2086	55.77483	136.6141	55.70288	137.2028	55.83487	139.8473
55.7813	112.008	55.70205	132.6104	55.68493	140.2063	55.7915	136.6118	55.71955	137.2003	55.85153	139.8475
55.79797	112.0068	55.71872	132.613	55.7016	140.2064	55.80817	136.6119	55.73622	137.2018	55.8682	139.8447
55.81463	112.0049	55.73538	132.6116	55.71827	140.2069	55.82483	136.6085	55.75288	137.1986	55.88487	139.8449
55.8313	112.001	55.75205	132.6126	55.73493	140.2041	55.8415	136.6089	55.76955	137.1988	55.90153	139.8448
55.84797	111.9984	55.76872	132.6126	55.7516	140.204	55.85817	136.6075	55.78622	137.1974	55.9182	139.8418
55.86463	111.9966	55.78538	132.612	55.76827	140.202	55.87483	136.6083	55.80288	137.196	55.93487	139.8401
55.8813	111.9973	55.80205	132.615	55.78493	140.202	55.8915	136.6054	55.81955	137.198	55.95153	139.8416
55.89797	111.9925	55.81872	132.6117	55.8016	140.1999	55.90817	136.6051	55.83622	137.1983	55.9682	139.8368
55.91463	111.9889	55.83538	132.6119	55.81827	140.1998	55.92483	136.603	55.85288	137.1994	55.98487	139.8375
55.9313	111.9887	55.85205	132.6118	55.83493	140.1995	55.9415	136.6036	55.86955	137.195	56.00153	139.8375
55.94797	111.9883	55.86872	132.6128	55.8516	140.1976	55.95817	136.6016	55.88622	137.1972	56.0182	139.8357
55.96463	111.9878	55.88538	132.6111	55.86827	140.1968	55.97483	136.6005	55.90288	137.1928	56.03487	139.8326
55.9813	111.9853	55.90205	132.616	55.88493	140.1992	55.9915	136.6011	55.91955	137.195	56.05153	139.8333
55.99797	111.9815	55.91872	132.6163	55.9016	140.1966	56.00817	136.6002	55.93622	137.1914	56.0682	139.8314
56.01463	111.9779	55.93538	132.6149	55.91827	140.1943	56.02483	136.599	55.95288	137.1891	56.08487	139.8306
56.0313	111.978	55.95205	132.6197	55.93493	140.1945	56.0415	136.6001	55.96955	137.1887	56.10153	139.828
56.04797	111.9738	55.96872	132.6186	55.9516	140.1957	56.05817	136.5987	55.98622	137.1861	56.1182	139.8294
56.06463	111.9718	55.98538	132.6196	55.96827	140.1909	56.07483	136.5974	56.00288	137.183	56.13487	139.8274
56.0813	111.9731	56.00205	132.6247	55.98493	140.1924	56.0915	136.5967	56.01955	137.1852	56.15153	139.8269
56.09797	111.9692	56.01872	132.6247	56.0016	140.1902	56.10817	136.5973	56.03622	137.1834	56.1682	139.825
56.11463	111.9701	56.03538	132.6234	56.01827	140.1918	56.12483	136.5966	56.05288	137.1804	56.18487	139.8245
56.1313	111.9651	56.05205	132.625	56.03493	140.1883	56.1415	136.5998	56.06955	137.1802	56.20153	139.8233
56.14797	111.9633	56.06872	132.6279	56.0516	140.1889	56.15817	136.5961	56.08622	137.182	56.2182	139.8233
56.16463	111.9593	56.08538	132.6296	56.06827	140.1878	56.17483	136.5955	56.10288	137.1789	56.23487	139.8219
56.1813	111.9583	56.10205	132.6312	56.08493	140.1832	56.1915	136.596	56.11955	137.1759	56.25153	139.8204
56.19797	111.9554	56.11872	132.633	56.1016	140.1864	56.20817	136.5957	56.13622	137.1794	56.2682	139.8197
56.21463	111.9561	56.13538	132.6274	56.11827	140.1821	56.22483	136.5959	56.15288	137.1762	56.28487	139.8217
56.2313	111.9534	56.15205	132.6354	56.13493	140.1829	56.2415	136.5938	56.16955	137.174	56.30153	139.8175
56.24797	111.9528	56.16872	132.6342	56.1516	140.1801	56.25817	136.5924	56.18622	137.1738	56.3182	139.8176
56.26463	111.9479	56.18538	132.6391	56.16827	140.1788	56.27483	136.5907	56.20288	137.1759	56.33487	139.8167
56.2813	111.945	56.20205	132.6447	56.18493	140.1785	56.2915	136.5901	56.21955	137.1721	56.35153	139.815
56.29797	111.9441	56.21872	132.6426	56.2016	140.1795	56.30817	136.5898	56.23622	137.1689	56.3682	139.8158
56.31463	111.9464	56.23538	132.6446	56.21827	140.1777	56.32483	136.5887	56.25288	137.1705	56.38487	139.8156
56.3313	111.9433	56.25205	132.6435	56.23493	140.1739	56.3415	136.5864	56.26955	137.1696	56.40153	139.8116
56.34797	111.9407	56.26872	132.6489	56.2516	140.1734	56.35817	136.5845	56.28622	137.1683	56.4182	139.8134
56.36463	111.9426	56.28538	132.6499	56.26827	140.1718	56.37483	136.5836	56.30288	137.1655	56.43487	139.8118
56.3813	111.9333	56.30205	132.6488	56.28493	140.1722	56.3915	136.5822	56.31955	137.1661	56.45153	139.8111
56.39797	111.9306	56.31872	132.6547	56.3016	140.1707	56.40817	136.5823	56.33622	137.1629	56.4682	139.8104
56.41463	111.9324	56.33538	132.6511	56.31827	140.1657	56.42483	136.5799	56.35288	137.164	56.48487	139.8095
56.4313	111.9288	56.35205	132.6516	56.33493	140.1664	56.4415	136.5793	56.36955	137.163	56.50153	139.81
56.44797	111.9285	56.36872	132.6577	56.3516	140.1661	56.45817	136.5764	56.38622	137.1639	56.5182	139.8083
56.46463	111.9285	56.38538	132.6547	56.36827	140.1643	56.47483	136.5744	56.40288	137.1625	56.53487	139.8064
56.4813	111.9203	56.40205	132.6578	56.38493	140.1609	56.4915	136.575	56.41955	137.161	56.55153	139.8049
56.49797	111.9218	56.41872	132.6591	56.4016	140.1627	56.50817	136.572	56.43622	137.1606	56.5682	139.8039
56.51463	111.9194	56.43538	132.664	56.41827	140.161	56.52483	136.5733	56.45288	137.1596	56.58487	139.8047
56.5313	111.9142	56.45205	132.6629	56.43493	140.159	56.5415	136.5721	56.46955	137.1585	56.60153	139.8031
56.54797	111.9141	56.46872	132.6682	56.4516	140.1576	56.55817	136.5703	56.48622	137.1565	56.6182	139.802
56.56463	111.9122	56.48538	132.6652	56.46827	140.1574	56.57483	136.5695	56.50288	137.1563	56.63487	139.802
56.5813	111.9099	56.50205	132.6655	56.48493	140.1532	56.5915	136.5675	56.51955	137.1566	56.65153	139.802
56.59797	111.9069	56.51872	132.667	56.5016	140.1528	56.60817	136.5683	56.53622	137.1545	56.6682	139.799
56.61463	111.9065	56.53538	132.6646	56.51827	140.1503	56.62483	136.5648	56.55288	137.1549	56.68487	139.7989
56.6313	111.9048	56.55205	132.6742	56.53493	140.1529	56.6415	136.5636	56.56955	137.1537	56.70153	139.7983

56.64797	111.9049	56.56872	132.6691	56.5516	140.1479	56.65817	136.5618	56.58622	137.1532	56.7182	139.7981
56.66463	111.901	56.58538	132.6738	56.56827	140.1499	56.67483	136.5634	56.60288	137.1519	56.73487	139.7966
56.6813	111.9004	56.60205	132.6782	56.58493	140.1455	56.6915	136.561	56.61955	137.1531	56.75153	139.7979
56.69797	111.8993	56.61872	132.6781	56.6016	140.1446	56.70817	136.56	56.63622	137.1518	56.7682	139.7958
56.71463	111.8929	56.63538	132.6822	56.61827	140.1436	56.72483	136.5569	56.65288	137.1508	56.78487	139.7937
56.7313	111.8952	56.65205	132.6831	56.63493	140.1432	56.7415	136.5591	56.66955	137.1495	56.80153	139.7942
56.74797	111.8932	56.66872	132.6842	56.6516	140.139	56.75817	136.5562	56.68622	137.1515	56.8182	139.7931
56.76463	111.8893	56.68538	132.6857	56.66827	140.1424	56.77483	136.5573	56.70288	137.1487	56.83487	139.7909
56.7813	111.8873	56.70205	132.6892	56.68493	140.1397	56.7915	136.5551	56.71955	137.1475	56.85153	139.7908
56.79797	111.888	56.71872	132.6885	56.7016	140.1401	56.80817	136.5543	56.73622	137.1467	56.8682	139.787
56.81463	111.884	56.73538	132.6918	56.71827	140.1382	56.82483	136.5532	56.75288	137.1447	56.88487	139.7866
56.8313	111.8812	56.75205	132.6971	56.73493	140.1394	56.8415	136.5533	56.76955	137.1453	56.90153	139.7861
56.84797	111.8806	56.76872	132.695	56.7516	140.1348	56.85817	136.5499	56.78622	137.1459	56.9182	139.7838
56.86463	111.8784	56.78538	132.6943	56.76827	140.1349	56.87483	136.5529	56.80288	137.1422	56.93487	139.7814
56.8813	111.8767	56.80205	132.6989	56.78493	140.135	56.8915	136.5487	56.81955	137.1437	56.95153	139.783
56.89797	111.8746	56.81872	132.6982	56.8016	140.1342	56.90817	136.5494	56.83622	137.1419	56.9682	139.7806
56.91463	111.8714	56.83538	132.7007	56.81827	140.1303	56.92483	136.5487	56.85288	137.1416	56.98487	139.7797
56.9313	111.8688	56.85205	132.6978	56.83493	140.1322	56.9415	136.5474	56.86955	137.1404	57.00153	139.7789
56.94797	111.8663	56.86872	132.7008	56.8516	140.1291	56.95817	136.5443	56.88622	137.14	57.0182	139.7771
56.96463	111.8655	56.88538	132.6996	56.86827	140.129	56.97483	136.5475	56.90288	137.1392	57.03487	139.7756
56.9813	111.8621	56.90205	132.7031	56.88493	140.1274	56.9915	136.5424	56.91955	137.1378	57.05153	139.7751
56.99797	111.8605	56.91872	132.7005	56.9016	140.1293	57.00817	136.5426	56.93622	137.1392	57.0682	139.7742
57.01463	111.8601	56.93538	132.7033	56.91827	140.125	57.02483	136.5405	56.95288	137.1386	57.08487	139.7729
57.0313	111.8568	56.95205	132.7012	56.93493	140.1271	57.0415	136.5424	56.96955	137.1349	57.10153	139.7711
57.04797	111.8537	56.96872	132.7044	56.9516	140.1234	57.05817	136.5369	56.98622	137.1363	57.1182	139.772
57.06463	111.8526	56.98538	132.7059	56.96827	140.1237	57.07483	136.5404	57.00288	137.1353	57.13487	139.7694
57.0813	111.8515	57.00205	132.7051	56.98493	140.1218	57.0915	136.5385	57.01955	137.1344	57.15153	139.7703
57.09797	111.8478	57.01872	132.7096	57.0016	140.1213	57.10817	136.5356	57.03622	137.1329	57.1682	139.7693
57.11463	111.8483	57.03538	132.7086	57.01827	140.1199	57.12483	136.5348	57.05288	137.1343	57.18487	139.7682
57.1313	111.8457	57.05205	132.7094	57.03493	140.1169	57.1415	136.5348	57.06955	137.1323	57.20153	139.7662
57.14797	111.8438	57.06872	132.7114	57.0516	140.1156	57.15817	136.5298	57.08622	137.1304	57.2182	139.767
57.16463	111.8387	57.08538	132.7128	57.06827	140.1161	57.17483	136.5325	57.10288	137.1301	57.23487	139.7655
57.1813	111.8394	57.10205	132.714	57.08493	140.1136	57.1915	136.5315	57.11955	137.1296	57.25153	139.7632
57.19797	111.8365	57.11872	132.7158	57.1016	140.1129	57.20817	136.5318	57.13622	137.13	57.2682	139.7637
57.21463	111.8334	57.13538	132.7198	57.11827	140.1124	57.22483	136.5288	57.15288	137.1295	57.28487	139.7629
57.2313	111.8326	57.15205	132.7183	57.13493	140.1121	57.2415	136.5286	57.16955	137.1273	57.30153	139.7612
57.24797	111.8298	57.16872	132.7252	57.1516	140.109	57.25817	136.5259	57.18622	137.1268	57.3182	139.7611
57.26463	111.8302	57.18538	132.7226	57.16827	140.1098	57.27483	136.5268	57.20288	137.1267	57.33487	139.7597
57.2813	111.8261	57.20205	132.7265	57.18493	140.1091	57.2915	136.5241	57.21955	137.1246	57.35153	139.7588
57.29797	111.8246	57.21872	132.728	57.2016	140.1089	57.30817	136.5239	57.23622	137.1256	57.3682	139.7593
57.31463	111.8234	57.23538	132.7304	57.21827	140.1075	57.32483	136.5219	57.25288	137.1244	57.38487	139.7593
57.3313	111.8204	57.25205	132.7345	57.23493	140.1053	57.3415	136.5245	57.26955	137.1247	57.40153	139.7566
57.34797	111.8203	57.26872	132.7341	57.2516	140.1039	57.35817	136.5199	57.28622	137.1207	57.4182	139.7568
57.36463	111.8202	57.28538	132.7356	57.26827	140.1029	57.37483	136.5209	57.30288	137.122	57.43487	139.7559
57.3813	111.8154	57.30205	132.7347	57.28493	140.1008	57.3915	136.5185	57.31955	137.1221	57.45153	139.7535
57.39797	111.8157	57.31872	132.739	57.3016	140.1003	57.40817	136.518	57.33622	137.12	57.4682	139.7528
57.41463	111.8137	57.33538	132.7379	57.31827	140.098	57.42483	136.517	57.35288	137.1181	57.48487	139.7535
57.4313	111.8095	57.35205	132.7404	57.33493	140.0962	57.4415	136.5163	57.36955	137.1199	57.50153	139.7512
57.44797	111.8093	57.36872	132.7422	57.3516	140.0964	57.45817	136.5126	57.38622	137.118	57.5182	139.7504
57.46463	111.8067	57.38538	132.7443	57.36827	140.0959	57.47483	136.5115	57.40288	137.1179	57.53487	139.7494
57.4813	111.8067	57.40205	132.7435	57.38493	140.0919	57.4915	136.5121	57.41955	137.1159	57.55153	139.7497
57.49797	111.8041	57.41872	132.744	57.4016	140.093	57.50817	136.5107	57.43622	137.1164	57.5682	139.7485
57.51463	111.8019	57.43538	132.7446	57.41827	140.0904	57.52483	136.5069	57.45288	137.1153	57.58487	139.7475
57.5313	111.7991	57.45205	132.7468	57.43493	140.0881	57.5415	136.5058	57.46955	137.1136	57.60153	139.7444
57.54797	111.7964	57.46872	132.748	57.4516	140.0888	57.55817	136.5063	57.48622	137.1115	57.6182	139.7456
57.56463	111.7949	57.48538	132.7488	57.46827	140.0885	57.57483	136.5074	57.50288	137.1123	57.63487	139.7456
57.5813	111.7905	57.50205	132.7506	57.48493	140.0881	57.5915	136.505	57.51955	137.1108	57.65153	139.7458
57.59797	111.7923	57.51872	132.753	57.5016	140.0857	57.60817	136.5056	57.53622	137.1091	57.6682	139.7419
57.61463	111.7912	57.53538	132.7553	57.51827	140.0832	57.62483	136.5047	57.55288	137.1079	57.68487	139.7429
57.6313	111.7869	57.55205	132.7548	57.53493	140.0837	57.6415	136.5026	57.56955	137.1085	57.70153	139.7416
57.64797	111.7817	57.56872	132.7573	57.5516	140.0827	57.65817	136.5003	57.58622	137.1074	57.7182	139.7419
57.66463	111.7841	57.58538	132.7576	57.56827	140.0843	57.67483	136.4983	57.60288	137.1056	57.73487	139.7399
57.6813	111.7817	57.60205	132.762	57.58493	140.0806	57.6915	136.4988	57.61955	137.1062	57.75153	139.7392
57.69797	111.7797	57.61872	132.7642	57.6016	140.0812	57.70817	136.4974	57.63622	137.105	57.7682	139.7363
57.71463	111.7755	57.63538	132.7664	57.61827	140.079	57.72483	136.4939	57.65288	137.1049	57.78487	139.7358
57.7313	111.7782	57.65205	132.7685	57.63493	140.0788	57.7415	136.4936	57.66955	137.1018	57.80153	139.7358
57.74797	111.7715	57.66872	132.7727	57.6516	140.0767	57.75817	136.4949	57.68622	137.1012	57.8182	139.7364
57.76463	111.7699	57.68538	132.7736	57.66827	140.078	57.77483	136.4965	57.70288	137.0993	57.83487	139.7325
57.7813	111.7662	57.70205	132.7735	57.68493	140.073	57.7915	136.4915	57.71955	137.1013	57.85153	139.7321
57.79797	111.7683	57.71872	132.7772	57.7016	140.074	57.80817	136.4919	57.73622	137.1008	57.8682	139.7313
57.81463	111.7655	57.73538	132.7771	57.71827	140.0701	57.82483	136.4919	57.75288	137.0987	57.88487	139.7307
57.8313	111.7648	57.75205	132.7766	57.73493	140.0679	57.8415	136.4896	57.76955	137.0977	57.90153	139.7276
57.84797	111.7623	57.76872	132.7822	57.7516	140.0666	57.85817	136.4888	57.78622	137.0981	57.9182	139.7293
57.86463	111.7621	57.78538	132.7851	57.76827	140.0663	57.87483	136.4886	57.80288	137.0978	57.93487	139.7265
57.8813	111.7561	57.80205	132.7843	57.78493	140.0662	57.8915	136.4869	57.81955	137.0937	57.95153	139.724
57.89797	111.7512	57.81872	132.7839	57.8016	140.0643	57.90817	136.4873	57.83622	137.0969	57.9682	139.7246
57.91463	111.7557	57.83538	132.7868	57.81827	140.0637	57.92483	136.486	57.85288	137.094	57.98487	139.7246
57.9313	111.7519	57.85205	132.7863	57.83493	140.0641	57.9415	136.4848	57.86955	137.0933	58.00153	139.725
57.94797	111.7486	57.86872	132.788	57.8516	140.0594	57.95817	136.4813	57.88622	137.0935	58.0182	139.7228
57.96463	111.748	57.88538	132.7904	57.86827	140.0598	57.97483	136.4842	57.90288	137.0913	58.03487	139.7243
57.9813	111.7476	57.90205	132.7916	57.88493	140.0577	57.9915	136.4803	57.91955	137.0916	58.05153	139.7199
57.99797	111.7451	57.91872	132.7944	57.9016	140.0563	58.00817	136.4789	57.93622	137.0898	58.0682	139.7189
58.01463	111.738	57.93538	132.7959	57.91827	140.0533	58.02483	136.4802	57.95288	137.0869	58.08487	139.7213
58.0313	111.7426	57.95205	132.7962	57.93493	140.0567	58.0415	136.481	57.96955	137.0883	58.10153	139.7175
58.04797	111.7379	57.96872	132.8002	57.9516	140.0541	58.05817	136.4746	57.98622	137.0856	58.1182	139.7188
58.06463	111.7399	57.98538	132.8029	57.96827	140.0537	58.07483	136.4765	58.00288	137.0838	58.13487	139.7151
58.0813	111.7314	58.00205	132.8038	57.98493	140.0532	58.0915	136.4748	58.01955	137.0848	58.15153	139.7185
58.09797	111.7314	58.01872	132.8024	58.0016	140.0514	58.10817	136.4768	58.03622	137.0813	58.1682	139.7161
58.11463	111.7299	58.03538	132.8062	58.01827	140.0501	58.12483	136.4727	58.05288	137.0834	58.18487	139.715
58.1313	111.729	58.05205	132.8073	58.03493	140.0531	58.1415	136.471	58.06955	137.0793	58.20153	139.7129
58.14797	111.7255	58.06872	132.8103	58.0516	140.0457	58.15817	136.4707	58.08622	137.0782	58.2182	139.7125
58.16463	111.7243	58.08538	132.8107	58.06827	140.0489	58.17483	136.4705	58.10288	137.0796	58.23487	139.7097
58.1813	111.7233	58.10205	132.8141	58.08493	140.0461	58.1915	136.4683	58.11955	137.0757	58.25153	139.7111
58.19797	111.7179	58.11872	132.8153	58.1016	140.0457	58.20817	136.4678	58.13622	137.0744	58.2682	139.7078
58.21463	111.7219	58.13538	132.8177	58.11827	140.0441	58.22483	136.4645	58.15288	137.073	58.28487	139.7084
58.2313	111.7153	58.15205	132.817	58.13493	140.0444	58.2415	136.4647	58.16955	137.0754	58.30153	139.7083
58.24797	111.7137	58.16872	132.8162	58.1516	140.042	58.25817	136.4647	58.18622	137.0729	58.3182	139.7053

58.26463	111.7108	58.18538	132.8188	58.16827	140.0397	58.27483	136.4641	58.20288	137.0717	58.33487	139.7045
58.2813	111.7129	58.20205	132.8207	58.18493	140.0409	58.2915	136.4657	58.21955	137.0697	58.35153	139.7033
58.29797	111.7087	58.21872	132.8229	58.2016	140.0378	58.30817	136.4621	58.23622	137.0716	58.3682	139.7033
58.31463	111.7084	58.23538	132.8243	58.21827	140.0351	58.32483	136.4632	58.25288	137.0687	58.38487	139.7043
58.3313	111.705	58.25205	132.8245	58.23493	140.0352	58.3415	136.4607	58.26955	137.067	58.40153	139.7018
58.34797	111.7022	58.26872	132.8274	58.2516	140.0336	58.35817	136.459	58.28622	137.0664	58.4182	139.7017
58.36463	111.7022	58.28538	132.8282	58.26827	140.0325	58.37483	136.4595	58.30288	137.0675	58.43487	139.6999
58.3813	111.7002	58.30205	132.8283	58.28493	140.0303	58.3915	136.4596	58.31955	137.0667	58.45153	139.7008
58.39797	111.6991	58.31872	132.8269	58.3016	140.0264	58.40817	136.4603	58.33622	137.0654	58.4682	139.6975
58.41463	111.6955	58.33538	132.8279	58.31827	140.0261	58.42483	136.4572	58.35288	137.0631	58.48487	139.6979
58.4313	111.6944	58.35205	132.8316	58.33493	140.0284	58.4415	136.4572	58.36955	137.0618	58.50153	139.6972
58.44797	111.6926	58.36872	132.8352	58.3516	140.0223	58.45817	136.4562	58.38622	137.0614	58.5182	139.6981
58.46463	111.6918	58.38538	132.8364	58.36827	140.0245	58.47483	136.4563	58.40288	137.0611	58.53487	139.6962
58.4813	111.6886	58.40205	132.8359	58.38493	140.0216	58.4915	136.4543	58.41955	137.0612	58.55153	139.6977
58.49797	111.6884	58.41872	132.8366	58.4016	140.0209	58.50817	136.4538	58.43622	137.0606	58.5682	139.6957
58.51463	111.6861	58.43538	132.8417	58.41827	140.0207	58.52483	136.4519	58.45288	137.0565	58.58487	139.6957
58.5313	111.6848	58.45205	132.8439	58.43493	140.0224	58.5415	136.4508	58.46955	137.0566	58.60153	139.6939
58.54797	111.6829	58.46872	132.8407	58.4516	140.0189	58.55817	136.4502	58.48622	137.0569	58.6182	139.6944
58.56463	111.6815	58.48538	132.8499	58.46827	140.0166	58.57483	136.4491	58.50288	137.0546	58.63487	139.6938
58.5813	111.6784	58.50205	132.8522	58.48493	140.0164	58.5915	136.45	58.51955	137.053	58.65153	139.6928
58.59797	111.6777	58.51872	132.8494	58.5016	140.0151	58.60817	136.4495	58.53622	137.0527	58.6682	139.6909
58.61463	111.6763	58.53538	132.8567	58.51827	140.0142	58.62483	136.4458	58.55288	137.0511	58.68487	139.6926
58.6313	111.6743	58.55205	132.8589	58.53493	140.0126	58.6415	136.4467	58.56955	137.0512	58.70153	139.6892
58.64797	111.6673	58.56872	132.8562	58.5516	140.0124	58.65817	136.4428	58.58622	137.0508	58.7182	139.6868
58.66463	111.6691	58.58538	132.8588	58.56827	140.0096	58.67483	136.4465	58.60288	137.0469	58.73487	139.6874
58.6813	111.6683	58.60205	132.8611	58.58493	140.0083	58.6915	136.445	58.61955	137.0479	58.75153	139.6857
58.69797	111.666	58.61872	132.8625	58.6016	140.0098	58.70817	136.4423	58.63622	137.0472	58.7682	139.6852
58.71463	111.6679	58.63538	132.8639	58.61827	140.0059	58.72483	136.441	58.65288	137.046	58.78487	139.685
58.7313	111.664	58.65205	132.8713	58.63493	140.0061	58.7415	136.4384	58.66955	137.0443	58.80153	139.6816
58.74797	111.6598	58.66872	132.8725	58.6516	140.0034	58.75817	136.4389	58.68622	137.0451	58.8182	139.6819
58.76463	111.6583	58.68538	132.8733	58.66827	140.0031	58.77483	136.4374	58.70288	137.044	58.83487	139.6802
58.7813	111.6567	58.70205	132.8715	58.68493	139.9999	58.7915	136.4354	58.71955	137.0415	58.85153	139.6781
58.79797	111.659	58.71872	132.8765	58.7016	140.0004	58.80817	136.4338	58.73622	137.0411	58.8682	139.6778
58.81463	111.6521	58.73538	132.8808	58.71827	140.0003	58.82483	136.4329	58.75288	137.0414	58.88487	139.677
58.8313	111.6535	58.75205	132.882	58.73493	139.9956	58.8415	136.4334	58.76955	137.0379	58.90153	139.6751
58.84797	111.647	58.76872	132.8824	58.7516	139.9914	58.85817	136.4303	58.78622	137.0378	58.9182	139.6743
58.86463	111.6496	58.78538	132.8844	58.76827	139.9888	58.87483	136.4321	58.80288	137.0366	58.93487	139.6742
58.8813	111.6451	58.80205	132.887	58.78493	139.9906	58.8915	136.4293	58.81955	137.0372	58.95153	139.6702
58.89797	111.6482	58.81872	132.8887	58.8016	139.9947	58.90817	136.4302	58.83622	137.0371	58.9682	139.6706
58.91463	111.6463	58.83538	132.89	58.81827	139.984	58.92483	136.4285	58.85288	137.0377	58.98487	139.6712
58.9313	111.6406	58.85205	132.8896	58.83493	139.9884	58.9415	136.4257	58.86955	137.0366	59.00153	139.6671
58.94797	111.6374	58.86872	132.8975	58.8516	139.9875	58.95817	136.4246	58.88622	137.0345	59.0182	139.6691
58.96463	111.6348	58.88538	132.8966	58.86827	139.9844	58.97483	136.4255	58.90288	137.0324	59.03487	139.6679
58.9813	111.6344	58.90205	132.8974	58.88493	139.9858	58.9915	136.4229	58.91955	137.0306	59.05153	139.6662
58.99797	111.6364	58.91872	132.8975	58.9016	139.9859	59.00817	136.4215	58.93622	137.0332	59.0682	139.6645
59.01463	111.631	58.93538	132.9	58.91827	139.9831	59.02483	136.4191	58.95288	137.0293	59.08487	139.6636
59.0313	111.6312	58.95205	132.9029	58.93493	139.9772	59.0415	136.4201	58.96955	137.0295	59.10153	139.6633
59.04797	111.629	58.96872	132.9036	58.9516	139.9779	59.05817	136.4202	58.98622	137.0298	59.1182	139.6628
59.06463	111.629	58.98538	132.9037	58.96827	139.978	59.07483	136.4168	59.00288	137.0289	59.13487	139.6623
59.0813	111.6233	59.00205	132.9081	58.98493	139.9764	59.0915	136.4167	59.01955	137.0257	59.15153	139.6604
59.09797	111.6223	59.01872	132.9084	59.0016	139.9742	59.10817	136.4161	59.03622	137.0262	59.1682	139.6589
59.11463	111.6208	59.03538	132.9116	59.01827	139.9723	59.12483	136.4136	59.05288	137.0254	59.18487	139.6584
59.1313	111.6214	59.05205	132.9111	59.03493	139.968	59.1415	136.4139	59.06955	137.0232	59.20153	139.6573
59.14797	111.6155	59.06872	132.9155	59.0516	139.969	59.15817	136.4141	59.08622	137.0231	59.2182	139.6564
59.16463	111.6154	59.08538	132.9164	59.06827	139.9671	59.17483	136.4102	59.10288	137.0224	59.23487	139.655
59.1813	111.6154	59.10205	132.9187	59.08493	139.9625	59.1915	136.4109	59.11955	137.0197	59.25153	139.6545
59.19797	111.6152	59.11872	132.9214	59.1016	139.963	59.20817	136.4095	59.13622	137.018	59.2682	139.6544
59.21463	111.61	59.13538	132.9205	59.11827	139.9603	59.22483	136.4108	59.15288	137.019	59.28487	139.6532
59.2313	111.6087	59.15205	132.9214	59.13493	139.9611	59.2415	136.4103	59.16955	137.0214	59.30153	139.6527
59.24797	111.6084	59.16872	132.9244	59.1516	139.9589	59.25817	136.4079	59.18622	137.0183	59.3182	139.65
59.26463	111.6033	59.18538	132.9232	59.16827	139.9603	59.27483	136.4068	59.20288	137.0184	59.33487	139.6493
59.2813	111.6014	59.20205	132.926	59.18493	139.9548	59.2915	136.405	59.21955	137.0184	59.35153	139.6492
59.29797	111.601	59.21872	132.9245	59.2016	139.9538	59.30817	136.404	59.23622	137.0166	59.3682	139.6499
59.31463	111.5994	59.23538	132.9241	59.21827	139.9514	59.32483	136.4029	59.25288	137.017	59.38487	139.65
59.3313	111.5957	59.25205	132.9244	59.23493	139.9549	59.3415	136.4016	59.26955	137.0154	59.40153	139.6447
59.34797	111.5969	59.26872	132.9265	59.2516	139.9531	59.35817	136.4009	59.28622	137.0127	59.4182	139.6447
59.36463	111.5938	59.28538	132.9255	59.26827	139.9524	59.37483	136.3976	59.30288	137.0122	59.43487	139.6451
59.3813	111.5918	59.30205	132.9248	59.28493	139.9501	59.3915	136.3982	59.31955	137.0114	59.45153	139.6416
59.39797	111.5908	59.31872	132.9242	59.3016	139.9477	59.40817	136.3977	59.33622	137.0101	59.4682	139.6418
59.41463	111.5928	59.33538	132.9241	59.31827	139.9488	59.42483	136.3987	59.35288	137.0102	59.48487	139.6403
59.4313	111.5879	59.35205	132.9258	59.33493	139.9476	59.4415	136.3952	59.36955	137.0077	59.50153	139.6385
59.44797	111.5863	59.36872	132.9281	59.3516	139.9443	59.45817	136.3931	59.38622	137.0035	59.5182	139.6405
59.46463	111.5859	59.38538	132.9266	59.36827	139.9471	59.47483	136.3956	59.40288	137.0093	59.53487	139.6393
59.4813	111.5802	59.40205	132.9269	59.38493	139.9453	59.4915	136.3914	59.41955	137.0054	59.55153	139.6387
59.49797	111.5787	59.41872	132.9284	59.4016	139.9443	59.50817	136.3917	59.43622	137.004	59.5682	139.6386
59.51463	111.5759	59.43538	132.9279	59.41827	139.9397	59.52483	136.3891	59.45288	137.0061	59.58487	139.6351
59.5313	111.5787	59.45205	132.9288	59.43493	139.942	59.5415	136.3897	59.46955	137.0039	59.60153	139.6369
59.54797	111.5673	59.46872	132.9301	59.4516	139.9393	59.55817	136.3877	59.48622	137.0006	59.6182	139.6356
59.56463	111.5724	59.48538	132.9323	59.46827	139.9375	59.57483	136.3865	59.50288	137.0002	59.63487	139.6346
59.5813	111.5734	59.50205	132.9307	59.48493	139.9375	59.5915	136.3835	59.51955	136.9995	59.65153	139.6347
59.59797	111.5662	59.51872	132.9339	59.5016	139.9374	59.60817	136.383	59.53622	137.0004	59.6682	139.6315
59.61463	111.5672	59.53538	132.9369	59.51827	139.9362	59.62483	136.3812	59.55288	136.9969	59.68487	139.6309
59.6313	111.5629	59.55205	132.9394	59.53493	139.934	59.6415	136.3808	59.56955	136.9967	59.70153	139.6302
59.64797	111.562	59.56872	132.9379	59.5516	139.9321	59.65817	136.3789	59.58622	136.9964	59.7182	139.6293
59.66463	111.5614	59.58538	132.9414	59.56827	139.9335	59.67483	136.3785	59.60288	136.9959	59.73487	139.6279
59.6813	111.5601	59.60205	132.9418	59.58493	139.9311	59.6915	136.3769	59.61955	136.9933	59.75153	139.6272
59.69797	111.5585	59.61872	132.9411	59.6016	139.9284	59.70817	136.3745	59.63622	136.9951	59.7682	139.6246
59.71463	111.5559	59.63538	132.9472	59.61827	139.9275	59.72483	136.3729	59.65288	136.9921	59.78487	139.6263
59.7313	111.5579	59.65205	132.9439	59.63493	139.9271	59.7415	136.3699	59.66955	136.992	59.80153	139.6265
59.74797	111.5525	59.66872	132.9444	59.6516	139.9248	59.75817	136.3728	59.68622	136.99	59.8182	139.6242
59.76463	111.5519	59.68538	132.9482	59.66827	139.9234	59.77483	136.3701	59.70288	136.9893	59.83487	139.624
59.7813	111.554	59.70205	132.9489	59.68493	139.9231	59.7915	136.3689	59.71955	136.9886	59.85153	139.6222
59.79797	111.5434	59.71872	132.9528	59.7016	139.9239	59.80817	136.3668	59.73622	136.9891	59.8682	139.6203
59.81463	111.5443	59.73538	132.9566	59.71827	139.9239	59.82483	136.3663	59.75288	136.9882	59.88487	139.6221
59.8313	111.5461	59.75205	132.956	59.73493	139.9213	59.8415	136.3653	59.76955	136.9858	59.90153	139.6208
59.84797	111.5419	59.76872	132.9589	59.7516	139.9231	59.85817	136.3618	59.78622	136.9849	59.9182	139.62
59.86463	111.541	59.78538	132.9617	59.76827	139.9202	59.87483	136.3626	59.80288	136.9845	59.93487	139.6193

59.8813	111.5414	59.80205	132.9642	59.78493	139.9163	59.8915	136.3601	59.81955	136.981	59.95153	139.618
59.89797	111.5373	59.81872	132.9643	59.8016	139.9196	59.90817	136.3591	59.83622	136.9817	59.9682	139.6183
59.91463	111.5352	59.83538	132.963	59.81827	139.9162	59.92483	136.3586	59.85288	136.9808	59.98487	139.6182
59.9313	111.537	59.85205	132.9656	59.83493	139.9126	59.9415	136.3586	59.86955	136.9818	60.00153	139.6178
59.94797	111.5308	59.86872	132.965	59.8516	139.9116	59.95817	136.3585	59.88622	136.979	60.0182	139.6156
59.96463	111.5308	59.88538	132.9686	59.86827	139.9073	59.97483	136.3553	59.90288	136.9787	60.03487	139.6133
59.9813	111.5265	59.90205	132.9725	59.88493	139.9086	59.9915	136.3542	59.91955	136.9763	60.05153	139.6132
59.99797	111.5251	59.91872	132.9675	59.9016	139.9076	60.00817	136.3532	59.93622	136.9784	60.0682	139.6128
60.01463	111.5246	59.93538	132.9683	59.91827	139.9023	60.02483	136.3525	59.95288	136.9763	60.08487	139.6113
60.0313	111.5235	59.95205	132.9741	59.93493	139.9036	60.0415	136.3508	59.96955	136.9775	60.10153	139.6122
60.04797	111.5175	59.96872	132.9659	59.9516	139.9054	60.05817	136.3504	59.98622	136.9746	60.1182	139.6109
60.06463	111.5191	59.98538	132.9719	59.96827	139.8997	60.07483	136.3486	60.00288	136.9734	60.13487	139.6097
60.0813	111.5145	60.00205	132.9722	59.98493	139.898	60.0915	136.3463	60.01955	136.9691	60.15153	139.6094
60.09797	111.5148	60.01872	132.9719	60.0016	139.8994	60.10817	136.3477	60.03622	136.9719	60.1682	139.6101
60.11463	111.5164	60.03538	132.9745	60.01827	139.8953	60.12483	136.3454	60.05288	136.9685	60.18487	139.6092
60.1313	111.5126	60.05205	132.9766	60.03493	139.8961	60.1415	136.3427	60.06955	136.9702	60.20153	139.6081
60.14797	111.5066	60.06872	132.9773	60.0516	139.8919	60.15817	136.3418	60.08622	136.9679	60.2182	139.607
60.16463	111.5104	60.08538	132.9748	60.06827	139.8925	60.17483	136.3407	60.10288	136.9699	60.23487	139.6078
60.1813	111.5083	60.10205	132.976	60.08493	139.8907	60.1915	136.3379	60.11955	136.9667	60.25153	139.606
60.19797	111.5067	60.11872	132.9801	60.1016	139.8925	60.20817	136.3395	60.13622	136.9681	60.2682	139.606
60.21463	111.5045	60.13538	132.978	60.11827	139.8884	60.22483	136.3369	60.15288	136.9655	60.28487	139.6022
60.2313	111.5006	60.15205	132.9795	60.13493	139.8885	60.2415	136.3358	60.16955	136.9649	60.30153	139.603
60.24797	111.501	60.16872	132.9796	60.1516	139.8857	60.25817	136.3348	60.18622	136.9616	60.3182	139.6016
60.26463	111.4993	60.18538	132.979	60.16827	139.8859	60.27483	136.3343	60.20288	136.9628	60.33487	139.6003
60.2813	111.4954	60.20205	132.9783	60.18493	139.8836	60.2915	136.3325	60.21955	136.959	60.35153	139.6007
60.29797	111.4932	60.21872	132.9806	60.2016	139.883	60.30817	136.3333	60.23622	136.9586	60.3682	139.6002
60.31463	111.4951	60.23538	132.9806	60.21827	139.8802	60.32483	136.3288	60.25288	136.96	60.38487	139.6007
60.3313	111.4921	60.25205	132.9803	60.23493	139.8802	60.3415	136.3294	60.26955	136.9559	60.40153	139.5996
60.34797	111.4876	60.26872	132.9797	60.2516	139.8804	60.35817	136.3291	60.28622	136.9547	60.4182	139.5973
60.36463	111.4894	60.28538	132.98	60.26827	139.878	60.37483	136.3286	60.30288	136.9564	60.43487	139.597
60.3813	111.4848	60.30205	132.9824	60.28493	139.8751	60.3915	136.3238	60.31955	136.9562	60.45153	139.5961
60.39797	111.4852	60.31872	132.9859	60.3016	139.8751	60.40817	136.3253	60.33622	136.9531	60.4682	139.5942
60.41463	111.4854	60.33538	132.984	60.31827	139.8742	60.42483	136.3231	60.35288	136.9519	60.48487	139.5974
60.4313	111.4807	60.35205	132.9873	60.33493	139.8715	60.4415	136.3226	60.36955	136.9549	60.50153	139.5912
60.44797	111.4777	60.36872	132.986	60.3516	139.8701	60.45817	136.3204	60.38622	136.9505	60.5182	139.5924
60.46463	111.4797	60.38538	132.9874	60.36827	139.8711	60.47483	136.3185	60.40288	136.9513	60.53487	139.5922
60.4813	111.4767	60.40205	132.992	60.38493	139.8671	60.4915	136.3165	60.41955	136.9465	60.55153	139.5918
60.49797	111.4735	60.41872	132.992	60.4016	139.8655	60.50817	136.3156	60.43622	136.9483	60.5682	139.5878
60.51463	111.4734	60.43538	132.9894	60.41827	139.8635	60.52483	136.3159	60.45288	136.9509	60.58487	139.5879
60.5313	111.4715	60.45205	132.9936	60.43493	139.8611	60.5415	136.3152	60.46955	136.9486	60.60153	139.5883
60.54797	111.4687	60.46872	132.9934	60.4516	139.86	60.55817	136.31	60.48622	136.9456	60.6182	139.5862
60.56463	111.4703	60.48538	132.9918	60.46827	139.8567	60.57483	136.3112	60.50288	136.9447	60.63487	139.5832
60.5813	111.4659	60.50205	132.9961	60.48493	139.8565	60.5915	136.309	60.51955	136.9442	60.65153	139.5836
60.59797	111.4665	60.51872	132.9934	60.5016	139.8554	60.60817	136.3098	60.53622	136.9451	60.6682	139.5827
60.61463	111.4651	60.53538	132.9981	60.51827	139.8544	60.62483	136.3082	60.55288	136.9419	60.68487	139.5793
60.6313	111.4617	60.55205	132.9996	60.53493	139.8534	60.6415	136.307	60.56955	136.9402	60.70153	139.5818
60.64797	111.4614	60.56872	133.0021	60.5516	139.8491	60.65817	136.3054	60.58622	136.9425	60.7182	139.5811
60.66463	111.4575	60.58538	133.0034	60.56827	139.8492	60.67483	136.3043	60.60288	136.9428	60.73487	139.5798
60.6813	111.4567	60.60205	133.0032	60.58493	139.8488	60.6915	136.3042	60.61955	136.9393	60.75153	139.5792
60.69797	111.4562	60.61872	133.0045	60.6016	139.8462	60.70817	136.3017	60.63622	136.9376	60.7682	139.5805
60.71463	111.4534	60.63538	133.0077	60.61827	139.845	60.72483	136.3023	60.65288	136.9379	60.78487	139.5761
60.7313	111.4525	60.65205	133.007	60.63493	139.8439	60.7415	136.3006	60.66955	136.9378	60.80153	139.5772
60.74797	111.4475	60.66872	133.01	60.6516	139.8433	60.75817	136.303	60.68622	136.9382	60.8182	139.5749
60.76463	111.4508	60.68538	133.0116	60.66827	139.8416	60.77483	136.3019	60.70288	136.9362	60.83487	139.5742
60.7813	111.4475	60.70205	133.0122	60.68493	139.8383	60.7915	136.299	60.71955	136.934	60.85153	139.5737
60.79797	111.4433	60.71872	133.0129	60.7016	139.8367	60.80817	136.3	60.73622	136.9367	60.8682	139.5705
60.81463	111.4429	60.73538	133.0135	60.71827	139.835	60.82483	136.301	60.75288	136.9373	60.88487	139.5709
60.8313	111.4433	60.75205	133.0178	60.73493	139.8354	60.8415	136.2965	60.76955	136.933	60.90153	139.571
60.84797	111.4385	60.76872	133.0213	60.7516	139.8334	60.85817	136.2948	60.78622	136.934	60.9182	139.5694
60.86463	111.4363	60.78538	133.0213	60.76827	139.834	60.87483	136.2963	60.80288	136.9324	60.93487	139.5691
60.8813	111.4366	60.80205	133.0225	60.78493	139.8285	60.8915	136.2966	60.81955	136.9324	60.95153	139.5645
60.89797	111.4355	60.81872	133.0245	60.8016	139.8292	60.90817	136.2929	60.83622	136.9299	60.9682	139.566
60.91463	111.4329	60.83538	133.0263	60.81827	139.8291	60.92483	136.2948	60.85288	136.9301	60.98487	139.5616
60.9313	111.4305	60.85205	133.0263	60.83493	139.8237	60.9415	136.292	60.86955	136.9295	61.00153	139.5613
60.94797	111.4272	60.86872	133.0286	60.8516	139.8229	60.95817	136.2917	60.88622	136.9257	61.0182	139.564
60.96463	111.4293	60.88538	133.0347	60.86827	139.8216	60.97483	136.2925	60.90288	136.9245	61.03487	139.5629
60.9813	111.425	60.90205	133.0335	60.88493	139.8219	60.9915	136.2886	60.91955	136.9246	61.05153	139.5595
60.99797	111.4241	60.91872	133.035	60.9016	139.8222	61.00817	136.2875	60.93622	136.9241	61.0682	139.5609
61.01463	111.4242	60.93538	133.0368	60.91827	139.8193	61.02483	136.2855	60.95288	136.9227	61.08487	139.5577
61.0313	111.4234	60.95205	133.0375	60.93493	139.8175	61.0415	136.2855	60.96955	136.9231	61.10153	139.5573
61.04797	111.42	60.96872	133.0395	60.9516	139.8151	61.05817	136.2848	60.98622	136.9214	61.1182	139.5567
61.06463	111.4164	60.98538	133.0413	60.96827	139.8146	61.07483	136.2837	61.00288	136.9239	61.13487	139.5539
61.0813	111.4145	61.00205	133.0432	60.98493	139.812	61.0915	136.2829	61.01955	136.9196	61.15153	139.5521
61.09797	111.4183	61.01872	133.0443	61.0016	139.8113	61.10817	136.2796	61.03622	136.9201	61.1682	139.5547
61.11463	111.411	61.03538	133.0467	61.01827	139.8099	61.12483	136.2817	61.05288	136.9172	61.18487	139.5513
61.1313	111.4101	61.05205	133.0493	61.03493	139.8087	61.1415	136.2806	61.06955	136.9166	61.20153	139.5517
61.14797	111.4102	61.06872	133.0473	61.0516	139.8072	61.15817	136.277	61.08622	136.917	61.2182	139.5505
61.16463	111.4108	61.08538	133.048	61.06827	139.8067	61.17483	136.2779	61.10288	136.9154	61.23487	139.5524
61.1813	111.4072	61.10205	133.0526	61.08493	139.8052	61.1915	136.2766	61.11955	136.9158	61.25153	139.5514
61.19797	111.407	61.11872	133.0509	61.1016	139.8007	61.20817	136.274	61.13622	136.9138	61.2682	139.5514
61.21463	111.4022	61.13538	133.052	61.11827	139.8019	61.22483	136.2735	61.15288	136.9134	61.28487	139.5486
61.2313	111.4012	61.15205	133.0521	61.13493	139.8023	61.2415	136.2706	61.16955	136.913	61.30153	139.5483
61.24797	111.3988	61.16872	133.0549	61.1516	139.7982	61.25817	136.2722	61.18622	136.9103	61.3182	139.5473
61.26463	111.4006	61.18538	133.0546	61.16827	139.7982	61.27483	136.2687	61.20288	136.9093	61.33487	139.5481
61.2813	111.398	61.20205	133.0554	61.18493	139.796	61.2915	136.2679	61.21955	136.9065	61.35153	139.5456
61.29797	111.3974	61.21872	133.056	61.2016	139.7932	61.30817	136.2666	61.23622	136.9085	61.3682	139.5466
61.31463	111.3938	61.23538	133.0557	61.21827	139.7935	61.32483	136.2652	61.25288	136.9058	61.38487	139.5445
61.3313	111.3911	61.25205	133.0585	61.23493	139.7899	61.3415	136.2613	61.26955	136.9086	61.40153	139.5449
61.34797	111.3896	61.26872	133.0608	61.2516	139.7877	61.35817	136.2613	61.28622	136.9041	61.4182	139.5407
61.36463	111.3901	61.28538	133.0629	61.26827	139.7863	61.37483	136.2601	61.30288	136.9057	61.43487	139.5414
61.3813	111.3883	61.30205	133.0614	61.28493	139.7854	61.3915	136.2617	61.31955	136.9035	61.45153	139.5378
61.39797	111.3854	61.31872	133.0665	61.3016	139.7842	61.40817	136.2586	61.33622	136.9046	61.4682	139.539
61.41463	111.3851	61.33538	133.0682	61.31827	139.782	61.42483	136.2549	61.35288	136.9016	61.48487	139.5386
61.4313	111.3812	61.35205	133.069	61.33493	139.7819	61.4415	136.2554	61.36955	136.9022	61.50153	139.5349
61.44797	111.38	61.36872	133.0692	61.3516	139.7811	61.45817	136.2525	61.38622	136.9022	61.5182	139.5332
61.46463	111.382	61.38538	133.0704	61.36827	139.7793	61.47483	136.2534	61.40288	136.9013	61.53487	139.5355
61.4813	111.3773	61.40205	133.0746	61.38493	139.7765	61.4915	136.2553	61.41955	136.8989	61.55153	139.5335

61.49797	111.3766	61.41872	133.0736	61.4016	139.7767	61.50817	136.2516	61.43622	136.8988	61.5682	139.5307
61.51463	111.3729	61.43538	133.0777	61.41827	139.7737	61.52483	136.2499	61.45288	136.8974	61.58487	139.5301
61.5313	111.3751	61.45205	133.0767	61.43493	139.7748	61.5415	136.2481	61.46955	136.8987	61.60153	139.5295
61.54797	111.3688	61.46872	133.0792	61.4516	139.7758	61.55817	136.2489	61.48622	136.8971	61.6182	139.5274
61.56463	111.3675	61.48538	133.0771	61.46827	139.7712	61.57483	136.2453	61.50288	136.897	61.63487	139.5276
61.5813	111.3701	61.50205	133.081	61.48493	139.7693	61.5915	136.2442	61.51955	136.8922	61.65153	139.5261
61.59797	111.3634	61.51872	133.0786	61.5016	139.7696	61.60817	136.2447	61.53622	136.8954	61.6682	139.5234
61.61463	111.3623	61.53538	133.0807	61.51827	139.7698	61.62483	136.2439	61.55288	136.8925	61.68487	139.5236
61.6313	111.3642	61.55205	133.083	61.53493	139.7675	61.6415	136.2425	61.56955	136.8939	61.70153	139.5245
61.64797	111.3628	61.56872	133.0837	61.5516	139.7657	61.65817	136.2392	61.58622	136.8916	61.7182	139.5212
61.66463	111.3594	61.58538	133.0866	61.56827	139.7624	61.67483	136.2401	61.60288	136.8915	61.73487	139.5208
61.6813	111.3569	61.60205	133.0903	61.58493	139.7603	61.6915	136.2402	61.61955	136.8895	61.75153	139.5182
61.69797	111.355	61.61872	133.0906	61.6016	139.7619	61.70817	136.2388	61.63622	136.887	61.7682	139.5173
61.71463	111.3538	61.63538	133.0918	61.61827	139.7593	61.72483	136.2387	61.65288	136.8869	61.78487	139.5163
61.7313	111.35	61.65205	133.0912	61.63493	139.759	61.7415	136.2377	61.66955	136.8877	61.80153	139.5149
61.74797	111.3495	61.66872	133.0936	61.6516	139.7569	61.75817	136.2349	61.68622	136.8869	61.8182	139.5108
61.76463	111.3484	61.68538	133.0974	61.66827	139.7542	61.77483	136.2348	61.70288	136.8851	61.83487	139.5108
61.7813	111.3456	61.70205	133.0986	61.68493	139.7526	61.7915	136.234	61.71955	136.8827	61.85153	139.5114
61.79797	111.3429	61.71872	133.1021	61.7016	139.7532	61.80817	136.2306	61.73622	136.8816	61.8682	139.5091
61.81463	111.3415	61.73538	133.1013	61.71827	139.7506	61.82483	136.2321	61.75288	136.8813	61.88487	139.5096
61.8313	111.3415	61.75205	133.105	61.73493	139.7455	61.8415	136.2306	61.76955	136.8795	61.90153	139.5075
61.84797	111.3363	61.76872	133.1045	61.7516	139.7474	61.85817	136.2317	61.78622	136.8798	61.9182	139.5064
61.86463	111.3355	61.78538	133.1088	61.76827	139.7449	61.87483	136.229	61.80288	136.878	61.93487	139.5071
61.8813	111.3344	61.80205	133.1117	61.78493	139.7459	61.8915	136.2308	61.81955	136.8781	61.95153	139.5047
61.89797	111.328	61.81872	133.1124	61.8016	139.7411	61.90817	136.2267	61.83622	136.8755	61.9682	139.5048
61.91463	111.3274	61.83538	133.1138	61.81827	139.7402	61.92483	136.227	61.85288	136.8748	61.98487	139.5002
61.9313	111.339	61.85205	133.1157	61.83493	139.7402	61.9415	136.2267	61.86955	136.8746	62.00153	139.5012
61.94797	111.3336	61.86872	133.1167	61.8516	139.7372	61.95817	136.2226	61.88622	136.8732	62.0182	139.4995
61.96463	111.3265	61.88538	133.1191	61.86827	139.734	61.97483	136.2235	61.90288	136.874	62.03487	139.4988
61.9813	111.3271	61.90205	133.1219	61.88493	139.7355	61.9915	136.219	61.91955	136.8697	62.05153	139.4957
61.99797	111.324	61.91872	133.122	61.9016	139.7321	62.00817	136.2227	61.93622	136.874	62.0682	139.4938
62.01463	111.3253	61.93538	133.1217	61.91827	139.7305	62.02483	136.22	61.95288	136.8699	62.08487	139.4923
62.0313	111.3209	61.95205	133.1213	61.93493	139.7321	62.0415	136.218	61.96955	136.8735	62.10153	139.4931
62.04797	111.3184	61.96872	133.123	61.9516	139.7268	62.05817	136.2172	61.98622	136.8656	62.1182	139.4894
62.06463	111.3195	61.98538	133.1255	61.96827	139.7286	62.07483	136.215	62.00288	136.8671	62.13487	139.4912
62.0813	111.316	62.00205	133.1284	61.98493	139.7271	62.0915	136.2164	62.01955	136.8685	62.15153	139.4877
62.09797	111.3166	62.01872	133.1277	62.0016	139.7243	62.10817	136.213	62.03622	136.8678	62.1682	139.4853
62.11463	111.3129	62.03538	133.1256	62.01827	139.7238	62.12483	136.2119	62.05288	136.8659	62.18487	139.4848
62.1313	111.3121	62.05205	133.1357	62.03493	139.7225	62.1415	136.2093	62.06955	136.8645	62.20153	139.4847
62.14797	111.3116	62.06872	133.134	62.0516	139.719	62.15817	136.2102	62.08622	136.8634	62.2182	139.4835
62.16463	111.3096	62.08538	133.1351	62.06827	139.7175	62.17483	136.21	62.10288	136.8633	62.23487	139.4822
62.1813	111.3064	62.10205	133.1369	62.08493	139.7188	62.1915	136.2058	62.11955	136.8634	62.25153	139.4798
62.19797	111.3031	62.11872	133.1375	62.1016	139.7161	62.20817	136.2032	62.13622	136.8633	62.2682	139.4823
62.21463	111.3053	62.13538	133.1397	62.11827	139.7109	62.22483	136.205	62.15288	136.8593	62.28487	139.4792
62.2313	111.3014	62.15205	133.1428	62.13493	139.7125	62.2415	136.2038	62.16955	136.8602	62.30153	139.4794
62.24797	111.3009	62.16872	133.1423	62.1516	139.7109	62.25817	136.2019	62.18622	136.8585	62.3182	139.4754
62.26463	111.3021	62.18538	133.1402	62.16827	139.7091	62.27483	136.2008	62.20288	136.8588	62.33487	139.4743
62.2813	111.3004	62.20205	133.1426	62.18493	139.7077	62.2915	136.1988	62.21955	136.858	62.35153	139.4738
62.29797	111.2971	62.21872	133.1415	62.2016	139.7068	62.30817	136.1996	62.23622	136.8563	62.3682	139.4729
62.31463	111.2963	62.23538	133.1405	62.21827	139.7057	62.32483	136.1975	62.25288	136.8532	62.38487	139.4732
62.3313	111.2943	62.25205	133.1371	62.23493	139.7046	62.3415	136.1957	62.26955	136.855	62.40153	139.4689
62.34797	111.2926	62.26872	133.1412	62.2516	139.6999	62.35817	136.1968	62.28622	136.8513	62.4182	139.4687
62.36463	111.289	62.28538	133.1405	62.26827	139.7003	62.37483	136.1956	62.30288	136.853	62.43487	139.4691
62.3813	111.2903	62.30205	133.1411	62.28493	139.7005	62.3915	136.1936	62.31955	136.8496	62.45153	139.4684
62.39797	111.2866	62.31872	133.1451	62.3016	139.6958	62.40817	136.193	62.33622	136.8469	62.4682	139.4663
62.41463	111.2836	62.33538	133.1434	62.31827	139.695	62.42483	136.1928	62.35288	136.8455	62.48487	139.4639
62.4313	111.2835	62.35205	133.1441	62.33493	139.6939	62.4415	136.1903	62.36955	136.8444	62.50153	139.4641
62.44797	111.2837	62.36872	133.1436	62.3516	139.6946	62.45817	136.1915	62.38622	136.8433	62.5182	139.4643
62.46463	111.2776	62.38538	133.1484	62.36827	139.6902	62.47483	136.1884	62.40288	136.8416	62.53487	139.4613
62.4813	111.2772	62.40205	133.1474	62.38493	139.6913	62.4915	136.1884	62.41955	136.8392	62.55153	139.46
62.49797	111.2776	62.41872	133.1509	62.4016	139.6882	62.50817	136.1882	62.43622	136.8379	62.5682	139.4602
62.51463	111.2727	62.43538	133.1499	62.41827	139.6853	62.52483	136.1868	62.45288	136.8381	62.58487	139.4571
62.5313	111.2743	62.45205	133.1502	62.43493	139.6832	62.5415	136.1858	62.46955	136.8369	62.60153	139.4577
62.54797	111.2732	62.46872	133.1559	62.4516	139.6822	62.55817	136.1853	62.48622	136.8336	62.6182	139.4556
62.56463	111.269	62.48538	133.1527	62.46827	139.681	62.57483	136.184	62.50288	136.8341	62.63487	139.4552
62.5813	111.2696	62.50205	133.1547	62.48493	139.6812	62.5915	136.1814	62.51955	136.8343	62.65153	139.4546
62.59797	111.2689	62.51872	133.1593	62.5016	139.6761	62.60817	136.1824	62.53622	136.8336	62.6682	139.4538
62.61463	111.2642	62.53538	133.1553	62.51827	139.6753	62.62483	136.1806	62.55288	136.8338	62.68487	139.4494
62.6313	111.2662	62.55205	133.1587	62.53493	139.6755	62.6415	136.1808	62.56955	136.8292	62.70153	139.45
62.64797	111.2622	62.56872	133.1609	62.5516	139.6757	62.65817	136.18	62.58622	136.8303	62.7182	139.4522
62.66463	111.2613	62.58538	133.1618	62.56827	139.6731	62.67483	136.1799	62.60288	136.8312	62.73487	139.4501
62.6813	111.2594	62.60205	133.1629	62.58493	139.672	62.6915	136.1774	62.61955	136.8305	62.75153	139.4448
62.69797	111.2576	62.61872	133.1632	62.6016	139.6698	62.70817	136.1743	62.63622	136.8304	62.7682	139.4472
62.71463	111.2552	62.63538	133.1639	62.61827	139.6678	62.72483	136.1735	62.65288	136.828	62.78487	139.4433
62.7313	111.2524	62.65205	133.1648	62.63493	139.6654	62.7415	136.1759	62.66955	136.8284	62.80153	139.4451
62.74797	111.2541	62.66872	133.1672	62.6516	139.6677	62.75817	136.1737	62.68622	136.8257	62.8182	139.4446
62.76463	111.2503	62.68538	133.1703	62.66827	139.6634	62.77483	136.1702	62.70288	136.8251	62.83487	139.4434
62.7813	111.2522	62.70205	133.1626	62.68493	139.665	62.7915	136.1729	62.71955	136.8243	62.85153	139.4432
62.79797	111.2493	62.71872	133.1693	62.7016	139.6616	62.80817	136.1702	62.73622	136.8278	62.8682	139.4416
62.81463	111.2471	62.73538	133.1661	62.71827	139.6601	62.82483	136.169	62.75288	136.8226	62.88487	139.4375
62.8313	111.2527	62.75205	133.167	62.73493	139.6595	62.8415	136.1671	62.76955	136.8209	62.90153	139.439
62.84797	111.2442	62.76872	133.1689	62.7516	139.6586	62.85817	136.1664	62.78622	136.8212	62.9182	139.4364
62.86463	111.2336	62.78538	133.168	62.76827	139.6549	62.87483	136.1649	62.80288	136.8214	62.93487	139.4366
62.8813	111.2352	62.80205	133.1684	62.78493	139.6533	62.8915	136.1626	62.81955	136.822	62.95153	139.4339
62.89797	111.2368	62.81872	133.1689	62.8016	139.65	62.90817	136.1636	62.83622	136.8198	62.9682	139.4342
62.91463	111.2346	62.83538	133.1677	62.81827	139.6495	62.92483	136.1607	62.85288	136.8171	62.98487	139.4342
62.9313	111.2321	62.85205	133.1704	62.83493	139.6469	62.9415	136.1601	62.86955	136.8152	63.00153	139.4318
62.94797	111.234	62.86872	133.1667	62.8516	139.647	62.95817	136.1605	62.88622	136.8149	63.0182	139.428
62.96463	111.2299	62.88538	133.1708	62.86827	139.6462	62.97483	136.1599	62.90288	136.8161	63.03487	139.4305
62.9813	111.2278	62.90205	133.1651	62.88493	139.6439	62.9915	136.1603	62.91955	136.8134	63.05153	139.428
62.99797	111.2302	62.91872	133.1683	62.9016	139.6423	63.00817	136.1576	62.93622	136.8119	63.0682	139.4271
63.01463	111.2264	62.93538	133.1681	62.91827	139.6394	63.02483	136.1562	62.95288	136.8116	63.08487	139.4246
63.0313	111.2236	62.95205	133.1703	62.93493	139.6377	63.0415	136.1535	62.96955	136.8114	63.10153	139.4252
63.04797	111.2189	62.96872	133.175	62.9516	139.6375	63.05817	136.1556	62.98622	136.8104	63.1182	139.4219
63.06463	111.2165	62.98538	133.175	62.96827	139.6364	63.07483	136.1519	63.00288	136.8109	63.13487	139.4206
63.0813	111.2206	63.00205	133.1756	62.98493	139.6344	63.0915	136.1513	63.01955	136.8086	63.15153	139.4189
63.09797	111.2203	63.01872	133.1813	63.0016	139.6309	63.10817	136.1508	63.03622	136.8066	63.1682	139.4185

63.11463	111.2175	63.03538	133.1802	63.01827	139.63	63.12483	136.1494	63.05288	136.8077	63.18487	139.4194
63.1313	111.213	63.05205	133.1839	63.03493	139.6272	63.1415	136.145	63.06955	136.8074	63.20153	139.417
63.14797	111.2143	63.06872	133.1856	63.0516	139.6278	63.15817	136.1472	63.08622	136.8043	63.2182	139.4142
63.16463	111.2125	63.08538	133.1897	63.06827	139.6254	63.17483	136.145	63.10288	136.8073	63.23487	139.4147
63.1813	111.2104	63.10205	133.1893	63.08493	139.6229	63.1915	136.1425	63.11955	136.8048	63.25153	139.414
63.19797	111.2099	63.11872	133.1907	63.1016	139.6213	63.20817	136.1419	63.13622	136.8033	63.2682	139.4109
63.21463	111.2065	63.13538	133.1939	63.11827	139.6202	63.22483	136.1417	63.15288	136.8037	63.28487	139.4099
63.2313	111.2045	63.15205	133.1975	63.13493	139.6177	63.2415	136.1402	63.16955	136.8014	63.30153	139.4092
63.24797	111.2055	63.16872	133.2019	63.1516	139.6178	63.25817	136.1389	63.18622	136.8002	63.3182	139.4081
63.26463	111.2006	63.18538	133.1995	63.16827	139.6171	63.27483	136.1365	63.20288	136.8017	63.33487	139.4048
63.2813	111.2007	63.20205	133.2019	63.18493	139.6135	63.2915	136.1387	63.21955	136.7991	63.35153	139.4045
63.29797	111.1985	63.21872	133.204	63.2016	139.6116	63.30817	136.1367	63.23622	136.7965	63.3682	139.4028
63.31463	111.1976	63.23538	133.2054	63.21827	139.6125	63.32483	136.1342	63.25288	136.7967	63.38487	139.4035
63.3313	111.1952	63.25205	133.2069	63.23493	139.6085	63.3415	136.1342	63.26955	136.7982	63.40153	139.4015
63.34797	111.1934	63.26872	133.2065	63.2516	139.609	63.35817	136.1336	63.28622	136.7955	63.4182	139.4003
63.36463	111.1892	63.28538	133.2108	63.26827	139.608	63.37483	136.1316	63.30288	136.7946	63.43487	139.4006
63.3813	111.189	63.30205	133.2106	63.28493	139.6043	63.3915	136.1303	63.31955	136.7942	63.45153	139.3971
63.39797	111.1865	63.31872	133.2152	63.3016	139.6036	63.40817	136.128	63.33622	136.7925	63.4682	139.3956
63.41463	111.1877	63.33538	133.217	63.31827	139.6032	63.42483	136.1283	63.35288	136.7919	63.48487	139.3947
63.4313	111.1836	63.35205	133.2193	63.33493	139.6007	63.4415	136.1261	63.36955	136.793	63.50153	139.3949
63.44797	111.1815	63.36872	133.2194	63.3516	139.6	63.45817	136.1251	63.38622	136.79	63.5182	139.3911
63.46463	111.1847	63.38538	133.2179	63.36827	139.5984	63.47483	136.1222	63.40288	136.7894	63.53487	139.3907
63.4813	111.177	63.40205	133.2223	63.38493	139.5985	63.4915	136.1236	63.41955	136.789	63.55153	139.391
63.49797	111.1732	63.41872	133.2255	63.4016	139.5957	63.50817	136.1211	63.43622	136.7875	63.5682	139.3898
63.51463	111.1761	63.43538	133.2281	63.41827	139.592	63.52483	136.1211	63.45288	136.7885	63.58487	139.3871
63.5313	111.1749	63.45205	133.2279	63.43493	139.5909	63.5415	136.119	63.46955	136.7879	63.60153	139.3884
63.54797	111.1726	63.46872	133.229	63.4516	139.5888	63.55817	136.1183	63.48622	136.7857	63.6182	139.3863
63.56463	111.1732	63.48538	133.233	63.46827	139.5899	63.57483	136.1164	63.50288	136.7869	63.63487	139.3863
63.5813	111.1689	63.50205	133.2294	63.48493	139.5892	63.5915	136.1186	63.51955	136.783	63.65153	139.3839
63.59797	111.1669	63.51872	133.2338	63.5016	139.5841	63.60817	136.1153	63.53622	136.7836	63.6682	139.3837
63.61463	111.1681	63.53538	133.2288	63.51827	139.5843	63.62483	136.1134	63.55288	136.7816	63.68487	139.3819
63.6313	111.1635	63.55205	133.2319	63.53493	139.5826	63.6415	136.114	63.56955	136.7815	63.70153	139.3809
63.64797	111.1662	63.56872	133.2308	63.5516	139.5817	63.65817	136.1113	63.58622	136.781	63.7182	139.3784
63.66463	111.1622	63.58538	133.2311	63.56827	139.5787	63.67483	136.1113	63.60288	136.778	63.73487	139.377
63.6813	111.1615	63.60205	133.226	63.58493	139.58	63.6915	136.1111	63.61955	136.7772	63.75153	139.3772
63.69797	111.16	63.61872	133.2304	63.6016	139.5759	63.70817	136.1092	63.63622	136.7765	63.7682	139.3777
63.71463	111.1606	63.63538	133.2309	63.61827	139.5752	63.72483	136.1103	63.65288	136.7747	63.78487	139.3752
63.7313	111.152	63.65205	133.2302	63.63493	139.5765	63.7415	136.1049	63.66955	136.7777	63.80153	139.376
63.74797	111.1566	63.66872	133.2308	63.6516	139.5737	63.75817	136.1055	63.68622	136.7762	63.8182	139.375
63.76463	111.1539	63.68538	133.2344	63.66827	139.5731	63.77483	136.104	63.70288	136.7724	63.83487	139.3765
63.7813	111.1477	63.70205	133.2334	63.68493	139.571	63.7915	136.1049	63.71955	136.774	63.85153	139.3723
63.79797	111.1465	63.71872	133.2371	63.7016	139.5692	63.80817	136.1013	63.73622	136.7741	63.8682	139.3736
63.81463	111.1474	63.73538	133.2369	63.71827	139.5661	63.82483	136.0997	63.75288	136.774	63.88487	139.3711
63.8313	111.1449	63.75205	133.2366	63.73493	139.5667	63.8415	136.0975	63.76955	136.7716	63.90153	139.3698
63.84797	111.1414	63.76872	133.2364	63.7516	139.5664	63.85817	136.0994	63.78622	136.771	63.9182	139.3686
63.86463	111.1407	63.78538	133.2411	63.76827	139.5636	63.87483	136.0997	63.80288	136.7707	63.93487	139.3691
63.8813	111.1386	63.80205	133.2412	63.78493	139.5636	63.8915	136.0954	63.81955	136.7673	63.95153	139.3679
63.89797	111.1397	63.81872	133.2425	63.8016	139.5599	63.90817	136.094	63.83622	136.7686	63.9682	139.3677
63.91463	111.1361	63.83538	133.2421	63.81827	139.5601	63.92483	136.0952	63.85288	136.7683	63.98487	139.366
63.9313	111.135	63.85205	133.244	63.83493	139.5561	63.9415	136.0937	63.86955	136.7656	64.00153	139.3628
63.94797	111.1329	63.86872	133.2451	63.8516	139.5537	63.95817	136.0932	63.88622	136.768	64.0182	139.3617
63.96463	111.133	63.88538	133.2451	63.86827	139.5535	63.97483	136.092	63.90288	136.766	64.03487	139.3618
63.9813	111.126	63.90205	133.2485	63.88493	139.5538	63.9915	136.0904	63.91955	136.7627	64.05153	139.3593
63.99797	111.1327	63.91872	133.2484	63.9016	139.5524	64.00817	136.0907	63.93622	136.765	64.0682	139.3619
64.01463	111.1279	63.93538	133.246	63.91827	139.5521	64.02483	136.0911	63.95288	136.766	64.08487	139.3601
64.0313	111.1229	63.95205	133.2444	63.93493	139.5489	64.0415	136.087	63.96955	136.7629	64.10153	139.3585
64.04797	111.1244	63.96872	133.2465	63.9516	139.5476	64.05817	136.0886	63.98622	136.7626	64.1182	139.3573
64.06463	111.1213	63.98538	133.2457	63.96827	139.5441	64.07483	136.0863	64.00288	136.7642	64.13487	139.357
64.0813	111.1185	64.00205	133.245	63.98493	139.5426	64.0915	136.0871	64.01955	136.76	64.15153	139.3566
64.09797	111.1192	64.01872	133.2462	64.0016	139.5435	64.10817	136.0831	64.03622	136.7618	64.1682	139.3547
64.11463	111.1172	64.03538	133.2499	64.01827	139.5425	64.12483	136.0829	64.05288	136.7593	64.18487	139.3528
64.1313	111.1166	64.05205	133.249	64.03493	139.5422	64.1415	136.0817	64.06955	136.7581	64.20153	139.352
64.14797	111.116	64.06872	133.2518	64.0516	139.5379	64.15817	136.0818	64.08622	136.7569	64.2182	139.3525
64.16463	111.1117	64.08538	133.2499	64.06827	139.5385	64.17483	136.0786	64.10288	136.7567	64.23487	139.3501
64.1813	111.1126	64.10205	133.2499	64.08493	139.5369	64.1915	136.0798	64.11955	136.755	64.25153	139.3493
64.19797	111.1059	64.11872	133.2495	64.1016	139.5309	64.20817	136.0789	64.13622	136.7543	64.2682	139.3492
64.21463	111.1069	64.13538	133.2512	64.11827	139.5314	64.22483	136.0774	64.15288	136.7544	64.28487	139.3459
64.2313	111.1063	64.15205	133.2522	64.13493	139.5321	64.2415	136.077	64.16955	136.7506	64.30153	139.3472
64.24797	111.102	64.16872	133.2526	64.1516	139.5303	64.25817	136.0719	64.18622	136.7523	64.3182	139.343
64.26463	111.0996	64.18538	133.2551	64.16827	139.5233	64.27483	136.0731	64.20288	136.7494	64.33487	139.3444
64.2813	111.1031	64.20205	133.2571	64.18493	139.5254	64.2915	136.0743	64.21955	136.7495	64.35153	139.3429
64.29797	111.0976	64.21872	133.261	64.2016	139.5221	64.30817	136.0711	64.23622	136.7522	64.3682	139.3429
64.31463	111.1003	64.23538	133.2564	64.21827	139.5192	64.32483	136.0724	64.25288	136.7504	64.38487	139.3419
64.3313	111.0946	64.25205	133.2598	64.23493	139.519	64.3415	136.0684	64.26955	136.7455	64.40153	139.341
64.34797	111.0946	64.26872	133.2624	64.2516	139.5142	64.35817	136.0686	64.28622	136.7482	64.4182	139.3401
64.36463	111.0941	64.28538	133.2616	64.26827	139.516	64.37483	136.0674	64.30288	136.7469	64.43487	139.3381
64.3813	111.0924	64.30205	133.2635	64.28493	139.516	64.3915	136.0649	64.31955	136.7446	64.45153	139.3376
64.39797	111.0916	64.31872	133.2629	64.3016	139.5136	64.40817	136.0641	64.33622	136.7458	64.4682	139.3353
64.41463	111.0896	64.33538	133.2667	64.31827	139.5148	64.42483	136.0628	64.35288	136.7464	64.48487	139.3327
64.4313	111.0893	64.35205	133.2643	64.33493	139.5094	64.4415	136.0632	64.36955	136.7433	64.50153	139.3339
64.44797	111.086	64.36872	133.2693	64.3516	139.5086	64.45817	136.0602	64.38622	136.7454	64.5182	139.334
64.46463	111.0817	64.38538	133.2707	64.36827	139.5076	64.47483	136.0566	64.40288	136.7439	64.53487	139.3318
64.4813	111.0861	64.40205	133.2627	64.38493	139.5069	64.4915	136.0565	64.41955	136.7452	64.55153	139.3287
64.49797	111.0788	64.41872	133.2675	64.4016	139.5059	64.50817	136.055	64.43622	136.7415	64.5682	139.3309
64.51463	111.0793	64.43538	133.2663	64.41827	139.5044	64.52483	136.0583	64.45288	136.7452	64.58487	139.3272
64.5313	111.0805	64.45205	133.2659	64.43493	139.501	64.5415	136.0535	64.46955	136.7434	64.60153	139.3261
64.54797	111.0764	64.46872	133.2707	64.4516	139.4985	64.55817	136.0531	64.48622	136.7414	64.6182	139.3263
64.56463	111.077	64.48538	133.2682	64.46827	139.4985	64.57483	136.051	64.50288	136.7436	64.63487	139.3262
64.5813	111.0715	64.50205	133.2664	64.48493	139.4972	64.5915	136.0515	64.51955	136.7418	64.65153	139.3236
64.59797	111.0713	64.51872	133.2653	64.5016	139.4951	64.60817	136.0495	64.53622	136.7385	64.6682	139.3223
64.61463	111.0729	64.53538	133.2628	64.51827	139.4925	64.62483	136.0493	64.55288	136.7399	64.68487	139.3238
64.6313	111.0709	64.55205	133.2652	64.53493	139.4911	64.6415	136.044	64.56955	136.7401	64.70153	139.3205
64.64797	111.0665	64.56872	133.264	64.5516	139.4903	64.65817	136.0461	64.58622	136.7382	64.7182	139.3193
64.66463	111.0682	64.58538	133.2604	64.56827	139.4895	64.67483	136.0423	64.60288	136.738	64.73487	139.3196
64.6813	111.0624	64.60205	133.2608	64.58493	139.4867	64.6915	136.0418	64.61955	136.7377	64.75153	139.3175
64.69797	111.0603	64.61872	133.2568	64.6016	139.4867	64.70817	136.046	64.63622	136.7386	64.7682	139.3145
64.71463	111.0605	64.63538	133.2574	64.61827	139.4841	64.72483	136.0406	64.65288	136.7401	64.78487	139.3148

64.7313	111.0603	64.65205	133.2556	64.63493	139.4818	64.7415	136.0395	64.66955	136.7381	64.80153	139.313
64.74797	111.0594	64.66872	133.2531	64.6516	139.4815	64.75817	136.0375	64.68622	136.7355	64.8182	139.3117
64.76463	111.0541	64.68538	133.2563	64.66827	139.4785	64.77483	136.0371	64.70288	136.733	64.83487	139.312
64.7813	111.0557	64.70205	133.2549	64.68493	139.4781	64.7915	136.0355	64.71955	136.7327	64.85153	139.3083
64.79797	111.0526	64.71872	133.2568	64.7016	139.4775	64.80817	136.0326	64.73622	136.7368	64.8682	139.3085
64.81463	111.0509	64.73538	133.2554	64.71827	139.4756	64.82483	136.0327	64.75288	136.7351	64.88487	139.3084
64.8313	111.0467	64.75205	133.2551	64.73493	139.4724	64.8415	136.0315	64.76955	136.7367	64.90153	139.3063
64.84797	111.0488	64.76872	133.2553	64.7516	139.4768	64.85817	136.0329	64.78622	136.7354	64.9182	139.3041
64.86463	111.0459	64.78538	133.2587	64.76827	139.4741	64.87483	136.0285	64.80288	136.7328	64.93487	139.3067
64.8813	111.0442	64.80205	133.2554	64.78493	139.4729	64.8915	136.0287	64.81955	136.7329	64.95153	139.3041
64.89797	111.0404	64.81872	133.2571	64.8016	139.4701	64.90817	136.029	64.83622	136.7351	64.9682	139.3025
64.91463	111.0407	64.83538	133.2545	64.81827	139.4643	64.92483	136.0268	64.85288	136.7327	64.98487	139.3007
64.9313	111.0419	64.85205	133.2551	64.83493	139.4692	64.9415	136.0256	64.86955	136.7322	65.00153	139.2995
64.94797	111.0378	64.86872	133.2538	64.8516	139.462	64.95817	136.0239	64.88622	136.7307	65.0182	139.298
64.96463	111.034	64.88538	133.2548	64.86827	139.4643	64.97483	136.0214	64.90288	136.7303	65.03487	139.2987
64.9813	111.04	64.90205	133.2534	64.88493	139.4626	64.9915	136.021	64.91955	136.7327	65.05153	139.2953
64.99797	111.0327	64.91872	133.2505	64.9016	139.4595	65.00817	136.0171	64.93622	136.7304	65.0682	139.2958
65.01463	111.0297	64.93538	133.2578	64.91827	139.4573	65.02483	136.016	64.95288	136.7285	65.08487	139.2951
65.0313	111.0319	64.95205	133.2532	64.93493	139.4528	65.0415	136.0152	64.96955	136.7281	65.10153	139.2948
65.04797	111.0316	64.96872	133.2533	64.9516	139.4536	65.05817	136.0141	64.98622	136.7252	65.1182	139.2912
65.06463	111.0228	64.98538	133.2556	64.96827	139.4494	65.07483	136.0123	65.00288	136.7257	65.13487	139.2911
65.0813	111.0278	65.00205	133.2553	64.98493	139.4467	65.0915	136.0112	65.01955	136.7267	65.15153	139.2906
65.09797	111.024	65.01872	133.2558	65.0016	139.4464	65.10817	136.0095	65.03622	136.7257	65.1682	139.2886
65.11463	111.0211	65.03538	133.2554	65.01827	139.4465	65.12483	136.0078	65.05288	136.7242	65.18487	139.2894
65.1313	111.0216	65.05205	133.2516	65.03493	139.4434	65.1415	136.0057	65.06955	136.7246	65.20153	139.2893
65.14797	111.0201	65.06872	133.2575	65.0516	139.4416	65.15817	136.0046	65.08622	136.723	65.2182	139.285
65.16463	111.0174	65.08538	133.2587	65.06827	139.44	65.17483	136.0042	65.10288	136.7224	65.23487	139.2855
65.1813	111.0158	65.10205	133.2593	65.08493	139.4382	65.1915	136.0037	65.11955	136.7222	65.25153	139.2841
65.19797	111.0144	65.11872	133.2613	65.1016	139.4343	65.20817	136.0008	65.13622	136.7206	65.2682	139.2838
65.21463	111.0107	65.13538	133.2582	65.11827	139.4328	65.22483	135.9995	65.15288	136.7193	65.28487	139.2809
65.2313	111.011	65.15205	133.2611	65.13493	139.4328	65.2415	135.9975	65.16955	136.7209	65.30153	139.282
65.24797	111.0099	65.16872	133.2639	65.1516	139.4355	65.25817	136.0006	65.18622	136.7189	65.3182	139.279
65.26463	111.0067	65.18538	133.2597	65.16827	139.4306	65.27483	135.997	65.20288	136.7173	65.33487	139.2791
65.2813	111.0069	65.20205	133.264	65.18493	139.4306	65.2915	135.9942	65.21955	136.7193	65.35153	139.2779
65.29797	111.0008	65.21872	133.2628	65.2016	139.4273	65.30817	135.9913	65.23622	136.7148	65.3682	139.2764
65.31463	111.0021	65.23538	133.2675	65.21827	139.4251	65.32483	135.9915	65.25288	136.7161	65.38487	139.2756
65.3313	111.0013	65.25205	133.2672	65.23493	139.4246	65.3415	135.9907	65.26955	136.7141	65.40153	139.2741
65.34797	110.9991	65.26872	133.2682	65.2516	139.4221	65.35817	135.9884	65.28622	136.7151	65.4182	139.274
65.36463	110.9959	65.28538	133.2696	65.26827	139.4203	65.37483	135.985	65.30288	136.7139	65.43487	139.272
65.3813	110.9961	65.30205	133.2697	65.28493	139.4208	65.3915	135.9816	65.31955	136.7148	65.45153	139.2719
65.39797	110.9908	65.31872	133.2715	65.3016	139.4185	65.40817	135.9833	65.33622	136.7133	65.4682	139.2687
65.41463	110.9925	65.33538	133.2695	65.31827	139.4178	65.42483	135.9818	65.35288	136.7103	65.48487	139.269
65.4313	110.9913	65.35205	133.2684	65.33493	139.4144	65.4415	135.9807	65.36955	136.7119	65.50153	139.2658
65.44797	110.9905	65.36872	133.2724	65.3516	139.4131	65.45817	135.9781	65.38622	136.7108	65.5182	139.2654
65.46463	110.9867	65.38538	133.276	65.36827	139.4135	65.47483	135.9759	65.40288	136.7104	65.53487	139.2648
65.4813	110.9859	65.40205	133.2706	65.38493	139.4107	65.4915	135.9779	65.41955	136.7103	65.55153	139.2638
65.49797	110.9834	65.41872	133.2749	65.4016	139.4095	65.50817	135.9742	65.43622	136.7083	65.5682	139.2638
65.51463	110.981	65.43538	133.2717	65.41827	139.4076	65.52483	135.9734	65.45288	136.7094	65.58487	139.2621
65.5313	110.9779	65.45205	133.2719	65.43493	139.4054	65.5415	135.9701	65.46955	136.7081	65.60153	139.2618
65.54797	110.9782	65.46872	133.2785	65.4516	139.4054	65.55817	135.9701	65.48622	136.71	65.6182	139.2586
65.56463	110.9756	65.48538	133.2768	65.46827	139.406	65.57483	135.9683	65.50288	136.7081	65.63487	139.2573
65.5813	110.9749	65.50205	133.2765	65.48493	139.403	65.5915	135.9666	65.51955	136.7065	65.65153	139.258
65.59797	110.9728	65.51872	133.2788	65.5016	139.3984	65.60817	135.9661	65.53622	136.7055	65.6682	139.2552
65.61463	110.9704	65.53538	133.277	65.51827	139.402	65.62483	135.965	65.55288	136.7049	65.68487	139.2577
65.6313	110.9691	65.55205	133.2818	65.53493	139.3951	65.6415	135.9625	65.56955	136.7055	65.70153	139.2514
65.64797	110.9703	65.56872	133.2793	65.5516	139.3972	65.65817	135.9627	65.58622	136.7051	65.7182	139.2525
65.66463	110.9665	65.58538	133.2805	65.56827	139.3932	65.67483	135.9593	65.60288	136.7041	65.73487	139.2517
65.6813	110.9659	65.60205	133.2818	65.58493	139.3935	65.6915	135.9597	65.61955	136.7073	65.75153	139.2516
65.69797	110.9613	65.61872	133.2816	65.6016	139.3906	65.70817	135.9566	65.63622	136.7037	65.7682	139.2507
65.71463	110.9612	65.63538	133.2806	65.61827	139.3886	65.72483	135.9589	65.65288	136.7064	65.78487	139.2467
65.7313	110.9595	65.65205	133.2812	65.63493	139.3875	65.7415	135.9569	65.66955	136.7056	65.80153	139.2499
65.74797	110.9587	65.66872	133.2836	65.6516	139.3845	65.75817	135.9537	65.68622	136.7052	65.8182	139.2471
65.76463	110.9572	65.68538	133.2843	65.66827	139.3816	65.77483	135.9512	65.70288	136.7028	65.83487	139.2477
65.7813	110.9549	65.70205	133.2838	65.68493	139.3818	65.7915	135.95	65.71955	136.7038	65.85153	139.2438
65.79797	110.9528	65.71872	133.286	65.7016	139.38	65.80817	135.9522	65.73622	136.7038	65.8682	139.2448
65.81463	110.9535	65.73538	133.2886	65.71827	139.3773	65.82483	135.9506	65.75288	136.703	65.88487	139.2417
65.8313	110.9502	65.75205	133.2904	65.73493	139.377	65.8415	135.9481	65.76955	136.7013	65.90153	139.2404
65.84797	110.9485	65.76872	133.2889	65.7516	139.3782	65.85817	135.9472	65.78622	136.6997	65.9182	139.2406
65.86463	110.9466	65.78538	133.2911	65.76827	139.3735	65.87483	135.9445	65.80288	136.7008	65.93487	139.2405
65.8813	110.9452	65.80205	133.2946	65.78493	139.3735	65.8915	135.9465	65.81955	136.7006	65.95153	139.2387
65.89797	110.9422	65.81872	133.2971	65.8016	139.3718	65.90817	135.943	65.83622	136.7019	65.9682	139.2363
65.91463	110.9443	65.83538	133.2982	65.81827	139.3677	65.92483	135.9441	65.85288	136.6974	65.98487	139.2346
65.9313	110.9428	65.85205	133.2987	65.83493	139.3693	65.9415	135.9377	65.86955	136.6973	66.00153	139.2355
65.94797	110.941	65.86872	133.2985	65.8516	139.3726	65.95817	135.9405	65.88622	136.6995	66.0182	139.2342
65.96463	110.9384	65.88538	133.3017	65.86827	139.3649	65.97483	135.938	65.90288	136.6961	66.03487	139.2328
65.9813	110.9403	65.90205	133.3032	65.88493	139.3624	65.9915	135.9376	65.91955	136.6986	66.05153	139.2298
65.99797	110.935	65.91872	133.3038	65.9016	139.3637	66.00817	135.9357	65.93622	136.6948	66.0682	139.2318
66.01463	110.9339	65.93538	133.3046	65.91827	139.3602	66.02483	135.9371	65.95288	136.6973	66.08487	139.2311
66.0313	110.9332	65.95205	133.3042	65.93493	139.3573	66.0415	135.9336	65.96955	136.6957	66.10153	139.2298
66.04797	110.9364	65.96872	133.3069	65.9516	139.3584	66.05817	135.9308	65.98622	136.6961	66.1182	139.2279
66.06463	110.9297	65.98538	133.3031	65.96827	139.3575	66.07483	135.9303	66.00288	136.6936	66.13487	139.229
66.0813	110.9295	66.00205	133.3088	65.98493	139.3538	66.0915	135.9289	66.01955	136.6934	66.15153	139.2276
66.09797	110.926	66.01872	133.3117	66.0016	139.3542	66.10817	135.9308	66.03622	136.6969	66.1682	139.2266
66.11463	110.9266	66.03538	133.3071	66.01827	139.352	66.12483	135.9295	66.05288	136.6923	66.18487	139.2243
66.1313	110.9261	66.05205	133.3103	66.03493	139.3532	66.1415	135.9268	66.06955	136.6912	66.20153	139.2239
66.14797	110.9204	66.06872	133.3122	66.0516	139.35	66.15817	135.9261	66.08622	136.6924	66.2182	139.2228
66.16463	110.9215	66.08538	133.3102	66.06827	139.3477	66.17483	135.922	66.10288	136.6912	66.23487	139.2215
66.1813	110.9197	66.10205	133.3114	66.08493	139.346	66.1915	135.9216	66.11955	136.6924	66.25153	139.2203
66.19797	110.919	66.11872	133.3142	66.1016	139.3443	66.20817	135.9226	66.13622	136.6904	66.2682	139.2189
66.21463	110.9203	66.13538	133.3148	66.11827	139.3436	66.22483	135.9181	66.15288	136.6895	66.28487	139.2176
66.2313	110.9167	66.15205	133.3122	66.13493	139.3432	66.2415	135.914	66.16955	136.6882	66.30153	139.2181
66.24797	110.9145	66.16872	133.3151	66.1516	139.3417	66.25817	135.9144	66.18622	136.6893	66.3182	139.2175
66.26463	110.9136	66.18538	133.3157	66.16827	139.3392	66.27483	135.9124	66.20288	136.6891	66.33487	139.2168
66.2813	110.9125	66.20205	133.318	66.18493	139.3385	66.2915	135.9122	66.21955	136.6835	66.35153	139.2155
66.29797	110.9072	66.21872	133.3174	66.2016	139.3348	66.30817	135.9112	66.23622	136.6875	66.3682	139.214
66.31463	110.9126	66.23538	133.3184	66.21827	139.3343	66.32483	135.9101	66.25288	136.686	66.38487	139.2168
66.3313	110.9067	66.25205	133.3215	66.23493	139.3341	66.3415	135.9082	66.26955	136.6882	66.40153	139.2135

66.34797	110.9033	66.26872	133.3209	66.2516	139.3335	66.35817	135.9087	66.28622	136.6856	66.4182	139.2109
66.36463	110.9031	66.28538	133.3212	66.26827	139.3325	66.37483	135.9056	66.30288	136.686	66.43487	139.212
66.3813	110.9022	66.30205	133.3199	66.28493	139.3313	66.3915	135.9047	66.31955	136.6854	66.45153	139.2101
66.39797	110.898	66.31872	133.3224	66.3016	139.3298	66.40817	135.8984	66.33622	136.6848	66.4682	139.2083
66.41463	110.8977	66.33538	133.3237	66.31827	139.3289	66.42483	135.9031	66.35288	136.6842	66.48487	139.2091
66.4313	110.8966	66.35205	133.323	66.33493	139.3258	66.4415	135.9006	66.36955	136.6851	66.50153	139.2068
66.44797	110.8885	66.36872	133.3216	66.3516	139.3242	66.45817	135.8995	66.38622	136.6822	66.5182	139.2062
66.46463	110.8942	66.38538	133.3281	66.36827	139.3253	66.47483	135.8989	66.40288	136.6819	66.53487	139.2063
66.4813	110.8902	66.40205	133.3263	66.38493	139.3228	66.4915	135.8975	66.41955	136.682	66.55153	139.2043
66.49797	110.8898	66.41872	133.3281	66.4016	139.3245	66.50817	135.8968	66.43622	136.6796	66.5682	139.1998
66.51463	110.8875	66.43538	133.3251	66.41827	139.3179	66.52483	135.8929	66.45288	136.6786	66.58487	139.203
66.5313	110.8873	66.45205	133.327	66.43493	139.3169	66.5415	135.8935	66.46955	136.6785	66.60153	139.2029
66.54797	110.8836	66.46872	133.3282	66.4516	139.3184	66.55817	135.8911	66.48622	136.6793	66.6182	139.2008
66.56463	110.8831	66.48538	133.3286	66.46827	139.3174	66.57483	135.8904	66.50288	136.6773	66.63487	139.2006
66.5813	110.8804	66.50205	133.3233	66.48493	139.3186	66.5915	135.8896	66.51955	136.6782	66.65153	139.1977
66.59797	110.8791	66.51872	133.327	66.5016	139.3156	66.60817	135.8885	66.53622	136.6774	66.6682	139.1961
66.61463	110.874	66.53538	133.3251	66.51827	139.3169	66.62483	135.8853	66.55288	136.676	66.68487	139.1986
66.6313	110.876	66.55205	133.3256	66.53493	139.3125	66.6415	135.8863	66.56955	136.6755	66.70153	139.1949
66.64797	110.8745	66.56872	133.3291	66.5516	139.3093	66.65817	135.8883	66.58622	136.6732	66.7182	139.1929
66.66463	110.8717	66.58538	133.328	66.56827	139.3109	66.67483	135.8826	66.60288	136.6732	66.73487	139.1931
66.6813	110.87	66.60205	133.3332	66.58493	139.3095	66.6915	135.8837	66.61955	136.6725	66.75153	139.1927
66.69797	110.8675	66.61872	133.3294	66.6016	139.307	66.70817	135.8812	66.63622	136.6717	66.7682	139.191
66.71463	110.8655	66.63538	133.3307	66.61827	139.3062	66.72483	135.8799	66.65288	136.6727	66.78487	139.1895
66.7313	110.8655	66.65205	133.3337	66.63493	139.3023	66.7415	135.8773	66.66955	136.6678	66.80153	139.1896
66.74797	110.8641	66.66872	133.331	66.6516	139.3029	66.75817	135.8781	66.68622	136.6701	66.8182	139.1884
66.76463	110.8616	66.68538	133.3329	66.66827	139.301	66.77483	135.8781	66.70288	136.6695	66.83487	139.1889
66.7813	110.8612	66.70205	133.3361	66.68493	139.298	66.7915	135.8767	66.71955	136.6701	66.85153	139.1857
66.79797	110.8574	66.71872	133.3354	66.7016	139.296	66.80817	135.8739	66.73622	136.6654	66.8682	139.1846
66.81463	110.8564	66.73538	133.3373	66.71827	139.2948	66.82483	135.8737	66.75288	136.6663	66.88487	139.184
66.8313	110.8534	66.75205	133.3386	66.73493	139.2933	66.8415	135.8707	66.76955	136.6671	66.90153	139.1848
66.84797	110.8535	66.76872	133.3384	66.7516	139.2899	66.85817	135.8711	66.78622	136.6665	66.9182	139.1847
66.86463	110.852	66.78538	133.3407	66.76827	139.2891	66.87483	135.8697	66.80288	136.6659	66.93487	139.1837
66.8813	110.8488	66.80205	133.3401	66.78493	139.2869	66.8915	135.8687	66.81955	136.6637	66.95153	139.1827
66.89797	110.8434	66.81872	133.3407	66.8016	139.2865	66.90817	135.8667	66.83622	136.6644	66.9682	139.1801
66.91463	110.8462	66.83538	133.3425	66.81827	139.2848	66.92483	135.8667	66.85288	136.6676	66.98487	139.1779
66.9313	110.8463	66.85205	133.3378	66.83493	139.2835	66.9415	135.8619	66.86955	136.6606	67.00153	139.177
66.94797	110.8446	66.86872	133.3404	66.8516	139.2813	66.95817	135.8625	66.88622	136.6668	67.0182	139.1775
66.96463	110.8416	66.88538	133.3431	66.86827	139.2769	66.97483	135.8617	66.90288	136.6603	67.03487	139.1758
66.9813	110.8403	66.90205	133.341	66.88493	139.2771	66.9915	135.862	66.91955	136.6571	67.05153	139.1742
66.99797	110.839	66.91872	133.3383	66.9016	139.2746	67.00817	135.8593	66.93622	136.6613	67.0682	139.1726
67.01463	110.8353	66.93538	133.3454	66.91827	139.2753	67.02483	135.8593	66.95288	136.6624	67.08487	139.1718
67.0313	110.8325	66.95205	133.3459	66.93493	139.2707	67.0415	135.8555	66.96955	136.6538	67.10153	139.1715
67.04797	110.8317	66.96872	133.3408	66.9516	139.2726	67.05817	135.8558	66.98622	136.6567	67.1182	139.1716
67.06463	110.8314	66.98538	133.3445	66.96827	139.2706	67.07483	135.8528	67.00288	136.6583	67.13487	139.168
67.0813	110.8279	67.00205	133.3464	66.98493	139.2671	67.0915	135.8535	67.01955	136.6566	67.15153	139.1681
67.09797	110.8237	67.01872	133.3437	67.0016	139.265	67.10817	135.8501	67.03622	136.6562	67.1682	139.1663
67.11463	110.8243	67.03538	133.3432	67.01827	139.2666	67.12483	135.8497	67.05288	136.6558	67.18487	139.1641
67.1313	110.8233	67.05205	133.3455	67.03493	139.2648	67.1415	135.8476	67.06955	136.654	67.20153	139.1645
67.14797	110.8218	67.06872	133.3456	67.0516	139.2628	67.15817	135.8458	67.08622	136.654	67.2182	139.1634
67.16463	110.8206	67.08538	133.3463	67.06827	139.261	67.17483	135.8452	67.10288	136.6542	67.23487	139.1626
67.1813	110.8194	67.10205	133.347	67.08493	139.2587	67.1915	135.8449	67.11955	136.653	67.25153	139.1616
67.19797	110.816	67.11872	133.3475	67.1016	139.2575	67.20817	135.8432	67.13622	136.6513	67.2682	139.1597
67.21463	110.8166	67.13538	133.3487	67.11827	139.2572	67.22483	135.8416	67.15288	136.6518	67.28487	139.1595
67.2313	110.8149	67.15205	133.3486	67.13493	139.2559	67.2415	135.8415	67.16955	136.6509	67.30153	139.159
67.24797	110.8103	67.16872	133.3446	67.1516	139.2542	67.25817	135.84	67.18622	136.6494	67.3182	139.1592
67.26463	110.8101	67.18538	133.3484	67.16827	139.2527	67.27483	135.8387	67.20288	136.6484	67.33487	139.1585
67.2813	110.809	67.20205	133.3497	67.18493	139.2484	67.2915	135.8362	67.21955	136.6465	67.35153	139.157
67.29797	110.8051	67.21872	133.3491	67.2016	139.2471	67.30817	135.8376	67.23622	136.6468	67.3682	139.1582
67.31463	110.8064	67.23538	133.3504	67.21827	139.2496	67.32483	135.8343	67.25288	136.6494	67.38487	139.1555
67.3313	110.8031	67.25205	133.3493	67.23493	139.2456	67.3415	135.8334	67.26955	136.6459	67.40153	139.1542
67.34797	110.8004	67.26872	133.3496	67.2516	139.243	67.35817	135.8313	67.28622	136.6466	67.4182	139.1555
67.36463	110.7992	67.28538	133.352	67.26827	139.2395	67.37483	135.8304	67.30288	136.6441	67.43487	139.1517
67.3813	110.7952	67.30205	133.3527	67.28493	139.2403	67.3915	135.8312	67.31955	136.644	67.45153	139.1513
67.39797	110.7948	67.31872	133.3519	67.3016	139.2367	67.40817	135.8278	67.33622	136.6465	67.4682	139.1501
67.41463	110.7936	67.33538	133.3534	67.31827	139.2368	67.42483	135.8276	67.35288	136.6426	67.48487	139.1515
67.4313	110.7911	67.35205	133.3513	67.33493	139.2335	67.4415	135.8251	67.36955	136.6421	67.50153	139.1488
67.44797	110.7937	67.36872	133.3561	67.3516	139.2309	67.45817	135.8256	67.38622	136.642	67.5182	139.1488
67.46463	110.7895	67.38538	133.3538	67.36827	139.2279	67.47483	135.8246	67.40288	136.6398	67.53487	139.1486
67.4813	110.7915	67.40205	133.3561	67.38493	139.2281	67.4915	135.8222	67.41955	136.6416	67.55153	139.1476
67.49797	110.7875	67.41872	133.3534	67.4016	139.2242	67.50817	135.822	67.43622	136.6412	67.5682	139.1455
67.51463	110.7863	67.43538	133.3569	67.41827	139.2232	67.52483	135.8212	67.45288	136.6397	67.58487	139.1458
67.5313	110.7844	67.45205	133.3572	67.43493	139.2248	67.5415	135.8186	67.46955	136.6395	67.60153	139.1437
67.54797	110.7874	67.46872	133.3588	67.4516	139.2201	67.55817	135.8191	67.48622	136.6413	67.6182	139.1423
67.56463	110.7812	67.48538	133.3566	67.46827	139.2195	67.57483	135.8167	67.50288	136.6363	67.63487	139.1433
67.5813	110.7836	67.50205	133.3599	67.48493	139.2193	67.5915	135.8139	67.51955	136.6383	67.65153	139.1401
67.59797	110.7797	67.51872	133.3578	67.5016	139.2165	67.60817	135.8128	67.53622	136.6383	67.6682	139.1416
67.61463	110.7807	67.53538	133.3609	67.51827	139.215	67.62483	135.8096	67.55288	136.6354	67.68487	139.1406
67.6313	110.7797	67.55205	133.3605	67.53493	139.2125	67.6415	135.8107	67.56955	136.6364	67.70153	139.1389
67.64797	110.778	67.56872	133.3594	67.5516	139.2098	67.65817	135.8102	67.58622	136.6349	67.7182	139.1395
67.66463	110.7754	67.58538	133.3613	67.56827	139.2073	67.67483	135.8077	67.60288	136.634	67.73487	139.136
67.6813	110.7735	67.60205	133.3685	67.58493	139.208	67.6915	135.8047	67.61955	136.6329	67.75153	139.1366
67.69797	110.77	67.61872	133.3628	67.6016	139.2058	67.70817	135.8067	67.63622	136.632	67.7682	139.1338
67.71463	110.7684	67.63538	133.3645	67.61827	139.2035	67.72483	135.8034	67.65288	136.6314	67.78487	139.1341
67.7313	110.7647	67.65205	133.3696	67.63493	139.2008	67.7415	135.8018	67.66955	136.6295	67.80153	139.1319
67.74797	110.7701	67.66872	133.3672	67.6516	139.1995	67.75817	135.8018	67.68622	136.6304	67.8182	139.1322
67.76463	110.7642	67.68538	133.3679	67.66827	139.2	67.77483	135.7999	67.70288	136.6301	67.83487	139.1297
67.7813	110.7627	67.70205	133.3725	67.68493	139.1966	67.7915	135.7966	67.71955	136.6296	67.85153	139.1264
67.79797	110.7606	67.71872	133.3685	67.7016	139.1939	67.80817	135.7937	67.73622	136.6268	67.8682	139.1289
67.81463	110.7619	67.73538	133.3724	67.71827	139.1948	67.82483	135.7969	67.75288	136.627	67.88487	139.1254
67.8313	110.7579	67.75205	133.3733	67.73493	139.1921	67.8415	135.7943	67.76955	136.6267	67.90153	139.126
67.84797	110.7585	67.76872	133.377	67.7516	139.1914	67.85817	135.7919	67.78622	136.6277	67.9182	139.1251
67.86463	110.7555	67.78538	133.3765	67.76827	139.1932	67.87483	135.7922	67.80288	136.6244	67.93487	139.1223
67.8813	110.7526	67.80205	133.3771	67.78493	139.1883	67.8915	135.7908	67.81955	136.6235	67.95153	139.1239
67.89797	110.7529	67.81872	133.38	67.8016	139.187	67.90817	135.7892	67.83622	136.6247	67.9682	139.1213
67.91463	110.7509	67.83538	133.3815	67.81827	139.1829	67.92483	135.7869	67.85288	136.6234	67.98487	139.1198
67.9313	110.7483	67.85205	133.3836	67.83493	139.1816	67.9415	135.7863	67.86955	136.621	68.00153	139.1198
67.94797	110.7492	67.86872	133.3836	67.8516	139.1814	67.95817	135.7845	67.88622	136.6196	68.0182	139.1191

67.96463	110.7462	67.88538	133.3823	67.86827	139.1795	67.97483	135.786	67.90288	136.6182	68.03487	139.117
67.9813	110.7465	67.90205	133.3819	67.88493	139.1783	67.9915	135.782	67.91955	136.6208	68.05153	139.1181
67.99797	110.7406	67.91872	133.3855	67.9016	139.1758	68.00817	135.7796	67.93622	136.6164	68.0682	139.1166
68.01463	110.74	67.93538	133.3812	67.91827	139.1749	68.02483	135.7808	67.95288	136.6183	68.08487	139.1153
68.0313	110.7338	67.95205	133.3859	67.93493	139.1721	68.0415	135.7782	67.96955	136.6176	68.10153	139.114
68.04797	110.7377	67.96872	133.3848	67.9516	139.1701	68.05817	135.7787	67.98622	136.6159	68.1182	139.1134
68.06463	110.733	67.98538	133.3861	67.96827	139.1679	68.07483	135.7766	68.00288	136.6113	68.13487	139.1129
68.0813	110.7344	68.00205	133.3884	67.98493	139.1667	68.0915	135.7745	68.01955	136.6143	68.15153	139.1122
68.09797	110.7288	68.01872	133.3874	68.0016	139.1636	68.10817	135.7758	68.03622	136.6135	68.1682	139.112
68.11463	110.7283	68.03538	133.3902	68.01827	139.1606	68.12483	135.7725	68.05288	136.6113	68.18487	139.1131
68.1313	110.7209	68.05205	133.3887	68.03493	139.1621	68.1415	135.7717	68.06955	136.6099	68.20153	139.1101
68.14797	110.7256	68.06872	133.3885	68.0516	139.1582	68.15817	135.7705	68.08622	136.6088	68.2182	139.1099
68.16463	110.723	68.08538	133.3901	68.06827	139.1591	68.17483	135.7697	68.10288	136.6085	68.23487	139.1104
68.1813	110.722	68.10205	133.3882	68.08493	139.1577	68.1915	135.7677	68.11955	136.6088	68.25153	139.107
68.19797	110.7219	68.11872	133.3905	68.1016	139.1546	68.20817	135.7662	68.13622	136.6049	68.2682	139.1074
68.21463	110.7197	68.13538	133.393	68.11827	139.1512	68.22483	135.7641	68.15288	136.6067	68.28487	139.1043
68.2313	110.7145	68.15205	133.3938	68.13493	139.1512	68.2415	135.7626	68.16955	136.6041	68.30153	139.1037
68.24797	110.7167	68.16872	133.3945	68.1516	139.1478	68.25817	135.7615	68.18622	136.6043	68.3182	139.1045
68.26463	110.7135	68.18538	133.3919	68.16827	139.147	68.27483	135.7625	68.20288	136.6043	68.33487	139.102
68.2813	110.7112	68.20205	133.3927	68.18493	139.1438	68.2915	135.7596	68.21955	136.6013	68.35153	139.1034
68.29797	110.7084	68.21872	133.3957	68.2016	139.139	68.30817	135.7596	68.23622	136.5977	68.3682	139.1002
68.31463	110.7106	68.23538	133.4014	68.21827	139.1417	68.32483	135.7579	68.25288	136.5971	68.38487	139.1012
68.3313	110.707	68.25205	133.3989	68.23493	139.1411	68.3415	135.7525	68.26955	136.5968	68.40153	139.0973
68.34797	110.7062	68.26872	133.399	68.2516	139.1388	68.35817	135.7541	68.28622	136.5939	68.4182	139.0986
68.36463	110.7062	68.28538	133.4003	68.26827	139.1336	68.37483	135.7525	68.30288	136.5945	68.43487	139.0966
68.3813	110.7041	68.30205	133.4016	68.28493	139.1291	68.3915	135.7511	68.31955	136.5949	68.45153	139.0961
68.39797	110.6992	68.31872	133.4044	68.3016	139.1295	68.40817	135.7504	68.33622	136.5923	68.4682	139.0959
68.41463	110.7029	68.33538	133.4011	68.31827	139.1268	68.42483	135.7479	68.35288	136.5934	68.48487	139.0932
68.4313	110.6978	68.35205	133.3996	68.33493	139.1285	68.4415	135.7477	68.36955	136.5932	68.50153	139.0938
68.44797	110.6948	68.36872	133.4046	68.3516	139.1261	68.45817	135.7476	68.38622	136.5933	68.5182	139.0923
68.46463	110.6962	68.38538	133.4035	68.36827	139.1213	68.47483	135.7445	68.40288	136.5921	68.53487	139.0924
68.4813	110.6941	68.40205	133.406	68.38493	139.1177	68.4915	135.7433	68.41955	136.5894	68.55153	139.0912
68.49797	110.6908	68.41872	133.4071	68.4016	139.1188	68.50817	135.74	68.43622	136.5912	68.5682	139.091
68.51463	110.6877	68.43538	133.408	68.41827	139.1176	68.52483	135.741	68.45288	136.5904	68.58487	139.0892
68.5313	110.686	68.45205	133.4084	68.43493	139.1116	68.5415	135.7395	68.46955	136.588	68.60153	139.0891
68.54797	110.6886	68.46872	133.4086	68.4516	139.1139	68.55817	135.7395	68.48622	136.5862	68.6182	139.0888
68.56463	110.6852	68.48538	133.4125	68.46827	139.1095	68.57483	135.7346	68.50288	136.5854	68.63487	139.0882
68.5813	110.6839	68.50205	133.4128	68.48493	139.1083	68.5915	135.7363	68.51955	136.5871	68.65153	139.087
68.59797	110.6849	68.51872	133.4137	68.5016	139.1066	68.60817	135.7377	68.53622	136.5857	68.6682	139.0872
68.61463	110.679	68.53538	133.4098	68.51827	139.1034	68.62483	135.7326	68.55288	136.5826	68.68487	139.0858
68.6313	110.6783	68.55205	133.4165	68.53493	139.1039	68.6415	135.7283	68.56955	136.5838	68.70153	139.0836
68.64797	110.6773	68.56872	133.4155	68.5516	139.0992	68.65817	135.7306	68.58622	136.5809	68.7182	139.0839
68.66463	110.6744	68.58538	133.4095	68.56827	139.0952	68.67483	135.7283	68.60288	136.5786	68.73487	139.0848
68.6813	110.6717	68.60205	133.4158	68.58493	139.095	68.6915	135.7257	68.61955	136.578	68.75153	139.083
68.69797	110.6707	68.61872	133.4172	68.6016	139.093	68.70817	135.7259	68.63622	136.5781	68.7682	139.0818
68.71463	110.6684	68.63538	133.417	68.61827	139.0946	68.72483	135.724	68.65288	136.5771	68.78487	139.0827
68.7313	110.6684	68.65205	133.4182	68.63493	139.0899	68.7415	135.7224	68.66955	136.5746	68.80153	139.0791
68.74797	110.6672	68.66872	133.4196	68.6516	139.087	68.75817	135.7203	68.68622	136.5734	68.8182	139.0792
68.76463	110.6658	68.68538	133.4188	68.66827	139.0874	68.77483	135.7212	68.70288	136.5745	68.83487	139.0769
68.7813	110.6611	68.70205	133.4215	68.68493	139.085	68.7915	135.7192	68.71955	136.573	68.85153	139.0775
68.79797	110.6596	68.71872	133.4181	68.7016	139.0844	68.80817	135.7205	68.73622	136.5735	68.8682	139.0761
68.81463	110.6603	68.73538	133.4208	68.71827	139.0829	68.82483	135.7171	68.75288	136.5713	68.88487	139.0768
68.8313	110.6558	68.75205	133.4181	68.73493	139.0797	68.8415	135.719	68.76955	136.571	68.90153	139.0742
68.84797	110.6576	68.76872	133.4211	68.7516	139.08	68.85817	135.7164	68.78622	136.571	68.9182	139.0738
68.86463	110.6548	68.78538	133.4201	68.76827	139.0804	68.87483	135.7165	68.80288	136.5722	68.93487	139.0721
68.8813	110.6539	68.80205	133.4234	68.78493	139.0778	68.8915	135.7137	68.81955	136.5711	68.95153	139.0742
68.89797	110.6537	68.81872	133.4229	68.8016	139.0769	68.90817	135.7115	68.83622	136.5697	68.9682	139.0723
68.91463	110.6512	68.83538	133.4235	68.81827	139.0733	68.92483	135.7119	68.85288	136.571	68.98487	139.0711
68.9313	110.6481	68.85205	133.4232	68.83493	139.0706	68.9415	135.7122	68.86955	136.5694	69.00153	139.0695
68.94797	110.6455	68.86872	133.4273	68.8516	139.071	68.95817	135.7115	68.88622	136.5674	69.0182	139.0688
68.96463	110.6474	68.88538	133.4263	68.86827	139.067	68.97483	135.7102	68.90288	136.5649	69.03487	139.0673
68.9813	110.6441	68.90205	133.4277	68.88493	139.0663	68.9915	135.7086	68.91955	136.5668	69.05153	139.0666
68.99797	110.6407	68.91872	133.4244	68.9016	139.0655	69.00817	135.7081	68.93622	136.5652	69.0682	139.0668
69.01463	110.6376	68.93538	133.4274	68.91827	139.0629	69.02483	135.7063	68.95288	136.5645	69.08487	139.0668
69.0313	110.6412	68.95205	133.4284	68.93493	139.0618	69.0415	135.705	68.96955	136.5628	69.10153	139.0661
69.04797	110.6377	68.96872	133.4326	68.9516	139.0593	69.05817	135.7056	68.98622	136.5616	69.1182	139.0624
69.06463	110.6356	68.98538	133.4281	68.96827	139.0556	69.07483	135.7041	69.00288	136.56	69.13487	139.0626
69.0813	110.6319	69.00205	133.4299	68.98493	139.0533	69.0915	135.702	69.01955	136.5611	69.15153	139.0624
69.09797	110.6336	69.01872	133.4324	69.0016	139.055	69.10817	135.703	69.03622	136.5595	69.1682	139.0616
69.11463	110.6316	69.03538	133.4325	69.01827	139.0487	69.12483	135.701	69.05288	136.5596	69.18487	139.0604
69.1313	110.6284	69.05205	133.4321	69.03493	139.0502	69.1415	135.6988	69.06955	136.561	69.20153	139.0582
69.14797	110.6266	69.06872	133.433	69.0516	139.0482	69.15817	135.697	69.08622	136.5581	69.2182	139.0594
69.16463	110.6216	69.08538	133.4318	69.06827	139.046	69.17483	135.695	69.10288	136.5556	69.23487	139.0574
69.1813	110.6248	69.10205	133.4336	69.08493	139.0444	69.1915	135.6923	69.11955	136.5581	69.25153	139.0589
69.19797	110.6205	69.11872	133.4332	69.1016	139.0412	69.20817	135.6891	69.13622	136.5554	69.2682	139.0581
69.21463	110.6187	69.13538	133.4326	69.11827	139.0428	69.22483	135.6869	69.15288	136.5531	69.28487	139.0561
69.2313	110.6194	69.15205	133.4333	69.13493	139.0401	69.2415	135.6867	69.16955	136.5519	69.30153	139.0543
69.24797	110.6146	69.16872	133.4335	69.1516	139.0374	69.25817	135.6826	69.18622	136.5532	69.3182	139.0531
69.26463	110.6153	69.18538	133.4326	69.16827	139.0378	69.27483	135.6817	69.20288	136.5483	69.33487	139.0542
69.2813	110.6126	69.20205	133.4358	69.18493	139.036	69.2915	135.681	69.21955	136.549	69.35153	139.052
69.29797	110.6107	69.21872	133.4344	69.2016	139.0367	69.30817	135.6788	69.23622	136.5487	69.3682	139.0516
69.31463	110.6117	69.23538	133.4347	69.21827	139.0316	69.32483	135.6769	69.25288	136.5492	69.38487	139.0517
69.3313	110.6049	69.25205	133.4365	69.23493	139.0307	69.3415	135.6746	69.26955	136.5465	69.40153	139.05
69.34797	110.6087	69.26872	133.4418	69.2516	139.029	69.35817	135.6731	69.28622	136.5451	69.4182	139.0501
69.36463	110.6053	69.28538	133.4388	69.26827	139.0278	69.37483	135.6698	69.30288	136.5447	69.43487	139.0496
69.3813	110.6023	69.30205	133.4398	69.28493	139.0236	69.3915	135.6704	69.31955	136.5433	69.45153	139.0488
69.39797	110.6032	69.31872	133.4425	69.3016	139.0255	69.40817	135.6639	69.33622	136.5411	69.4682	139.049
69.41463	110.6014	69.33538	133.444	69.31827	139.0192	69.42483	135.6643	69.35288	136.5404	69.48487	139.0477
69.4313	110.5992	69.35205	133.4433	69.33493	139.0195	69.4415	135.6627	69.36955	136.5384	69.50153	139.0461
69.44797	110.5962	69.36872	133.4449	69.3516	139.0209	69.45817	135.6631	69.38622	136.5387	69.5182	139.0482
69.46463	110.5963	69.38538	133.4444	69.36827	139.0217	69.47483	135.6592	69.40288	136.537	69.53487	139.0457
69.4813	110.5948	69.40205	133.4454	69.38493	139.0187	69.4915	135.6585	69.41955	136.5361	69.55153	139.0454
69.49797	110.5933	69.41872	133.4462	69.4016	139.0147	69.50817	135.6592	69.43622	136.5352	69.5682	139.0452
69.51463	110.5925	69.43538	133.449	69.41827	139.0138	69.52483	135.6565	69.45288	136.5362	69.58487	139.0465
69.5313	110.5895	69.45205	133.445	69.43493	139.0109	69.5415	135.6543	69.46955	136.5317	69.60153	139.0415
69.54797	110.5895	69.46872	133.4469	69.4516	139.0096	69.55817	135.6535	69.48622	136.533	69.6182	139.0421
69.56463	110.589	69.48538	133.4462	69.46827	139.0075	69.57483	135.651	69.50288	136.5317	69.63487	139.041

69.5813	110.5861	69.50205	133.4496	69.48493	139.006	69.5915	135.652	69.51955	136.5319	69.65153	139.0402
69.59797	110.5874	69.51872	133.4467	69.5016	139.0018	69.60817	135.6486	69.53622	136.5259	69.6682	139.0382
69.61463	110.5867	69.53538	133.4488	69.51827	139.0025	69.62483	135.6454	69.55288	136.5265	69.68487	139.0382
69.6313	110.5866	69.55205	133.4523	69.53493	138.9985	69.6415	135.6446	69.56955	136.5256	69.70153	139.0356
69.64797	110.5835	69.56872	133.4529	69.5516	139.0011	69.65817	135.6433	69.58622	136.5264	69.7182	139.0373
69.66463	110.5818	69.58538	133.4487	69.56827	138.9954	69.67483	135.6415	69.60288	136.5254	69.73487	139.0355
69.6813	110.5782	69.60205	133.4553	69.58493	138.9934	69.6915	135.6402	69.61955	136.5233	69.75153	139.0338
69.69797	110.5796	69.61872	133.4546	69.6016	138.9934	69.70817	135.6429	69.63622	136.5231	69.7682	139.0323
69.71463	110.5789	69.63538	133.4519	69.61827	138.9888	69.72483	135.6399	69.65288	136.5233	69.78487	139.0325
69.7313	110.5744	69.65205	133.4575	69.63493	138.9919	69.7415	135.639	69.66955	136.5211	69.80153	139.0302
69.74797	110.5723	69.66872	133.4562	69.6516	138.9876	69.75817	135.6372	69.68622	136.5204	69.8182	139.0282
69.76463	110.5733	69.68538	133.4569	69.66827	138.9867	69.77483	135.634	69.70288	136.5218	69.83487	139.0299
69.7813	110.571	69.70205	133.4573	69.68493	138.9845	69.7915	135.634	69.71955	136.5198	69.85153	139.0291
69.79797	110.5705	69.71872	133.4556	69.7016	138.9826	69.80817	135.6335	69.73622	136.5181	69.8682	139.0262
69.81463	110.5666	69.73538	133.4575	69.71827	138.9816	69.82483	135.631	69.75288	136.5186	69.88487	139.028
69.8313	110.5628	69.75205	133.4571	69.73493	138.9796	69.8415	135.6291	69.76955	136.5165	69.90153	139.0249
69.84797	110.5647	69.76872	133.4604	69.7516	138.9782	69.85817	135.6271	69.78622	136.5149	69.9182	139.0246
69.86463	110.5634	69.78538	133.4594	69.76827	138.9795	69.87483	135.6277	69.80288	136.5144	69.93487	139.0244
69.8813	110.5622	69.80205	133.4589	69.78493	138.9743	69.8915	135.6286	69.81955	136.5146	69.95153	139.0235
69.89797	110.5594	69.81872	133.4599	69.8016	138.9758	69.90817	135.6271	69.83622	136.5127	69.9682	139.022
69.91463	110.5585	69.83538	133.4594	69.81827	138.9725	69.92483	135.6258	69.85288	136.5119	69.98487	139.0244
69.9313	110.5564	69.85205	133.4624	69.83493	138.968	69.9415	135.6233	69.86955	136.5095	70.00153	139.0228
69.94797	110.5555	69.86872	133.4587	69.8516	138.9664	69.95817	135.622	69.88622	136.5098	70.0182	139.0207
69.96463	110.5535	69.88538	133.4597	69.86827	138.9635	69.97483	135.6248	69.90288	136.5078	70.03487	139.0183
69.9813	110.5526	69.90205	133.4584	69.88493	138.9639	69.9915	135.6205	69.91955	136.5076	70.05153	139.0165
69.99797	110.5491	69.91872	133.4615	69.9016	138.9596	70.00817	135.6204	69.93622	136.5061	70.0682	139.0162
70.01463	110.5487	69.93538	133.4619	69.91827	138.9594	70.02483	135.6202	69.95288	136.5062	70.08487	139.0145
70.0313	110.5448	69.95205	133.4616	69.93493	138.9564	70.0415	135.6191	69.96955	136.5058	70.10153	139.0156
70.04797	110.5459	69.96872	133.4624	69.9516	138.9549	70.05817	135.6185	69.98622	136.5042	70.1182	139.0143
70.06463	110.5428	69.98538	133.4602	69.96827	138.9531	70.07483	135.6165	70.00288	136.5029	70.13487	139.0137
70.0813	110.5384	70.00205	133.4596	69.98493	138.948	70.0915	135.6154	70.01955	136.5047	70.15153	139.0129
70.09797	110.5412	70.01872	133.4626	70.0016	138.9489	70.10817	135.6141	70.03622	136.5023	70.1682	139.0124
70.11463	110.5386	70.03538	133.4659	70.01827	138.9471	70.12483	135.6129	70.05288	136.5019	70.18487	139.0129
70.1313	110.5345	70.05205	133.4613	70.03493	138.9443	70.1415	135.6116	70.06955	136.4987	70.20153	139.0109
70.14797	110.5308	70.06872	133.4639	70.0516	138.9423	70.15817	135.6091	70.08622	136.499	70.2182	139.0099
70.16463	110.5312	70.08538	133.4621	70.06827	138.9422	70.17483	135.6088	70.10288	136.4994	70.23487	139.007
70.1813	110.5314	70.10205	133.4654	70.08493	138.94	70.1915	135.6083	70.11955	136.4991	70.25153	139.0065
70.19797	110.5271	70.11872	133.4655	70.1016	138.9365	70.20817	135.6045	70.13622	136.4978	70.2682	139.0074
70.21463	110.5283	70.13538	133.4657	70.11827	138.9356	70.22483	135.6033	70.15288	136.4985	70.28487	139.0079
70.2313	110.5259	70.15205	133.4668	70.13493	138.9342	70.2415	135.601	70.16955	136.4945	70.30153	139.0059
70.24797	110.5207	70.16872	133.4681	70.1516	138.9319	70.25817	135.6	70.18622	136.4956	70.3182	139.0038
70.26463	110.5227	70.18538	133.4671	70.16827	138.9308	70.27483	135.5962	70.20288	136.4985	70.33487	139.0036
70.2813	110.5204	70.20205	133.4668	70.18493	138.9268	70.2915	135.5944	70.21955	136.4961	70.35153	139.0064
70.29797	110.5177	70.21872	133.4648	70.2016	138.9307	70.30817	135.5935	70.23622	136.4977	70.3682	139.0013
70.31463	110.5183	70.23538	133.4665	70.21827	138.9227	70.32483	135.5921	70.25288	136.4924	70.38487	139.0015
70.3313	110.5158	70.25205	133.466	70.23493	138.9229	70.3415	135.5897	70.26955	136.4905	70.40153	139.0008
70.34797	110.5143	70.26872	133.467	70.2516	138.9202	70.35817	135.5893	70.28622	136.4911	70.4182	139.0003
70.36463	110.5108	70.28538	133.4676	70.26827	138.9196	70.37483	135.5855	70.30288	136.4899	70.43487	138.9972
70.3813	110.5104	70.30205	133.4679	70.28493	138.919	70.3915	135.5843	70.31955	136.4895	70.45153	138.9971
70.39797	110.5081	70.31872	133.4662	70.3016	138.9175	70.40817	135.5847	70.33622	136.4865	70.4682	138.9975
70.41463	110.5064	70.33538	133.4679	70.31827	138.9177	70.42483	135.5811	70.35288	136.489	70.48487	138.9957
70.4313	110.5041	70.35205	133.4663	70.33493	138.9118	70.4415	135.5821	70.36955	136.4837	70.50153	138.9914
70.44797	110.5026	70.36872	133.4676	70.3516	138.9109	70.45817	135.5782	70.38622	136.4807	70.5182	138.9945
70.46463	110.5037	70.38538	133.4639	70.36827	138.9099	70.47483	135.5773	70.40288	136.4832	70.53487	138.9922
70.4813	110.4975	70.40205	133.4651	70.38493	138.9046	70.4915	135.5753	70.41955	136.4843	70.55153	138.9948
70.49797	110.4996	70.41872	133.4649	70.4016	138.9038	70.50817	135.5732	70.43622	136.479	70.5682	138.9925
70.51463	110.4963	70.43538	133.4662	70.41827	138.899	70.52483	135.5722	70.45288	136.4786	70.58487	138.9906
70.5313	110.4955	70.45205	133.4674	70.43493	138.8985	70.5415	135.5728	70.46955	136.4751	70.60153	138.9878
70.54797	110.4937	70.46872	133.4672	70.4516	138.8986	70.55817	135.5693	70.48622	136.4747	70.6182	138.9883
70.56463	110.4941	70.48538	133.4659	70.46827	138.8966	70.57483	135.5654	70.50288	136.4721	70.63487	138.987
70.5813	110.4904	70.50205	133.4694	70.48493	138.8923	70.5915	135.5663	70.51955	136.473	70.65153	138.9878
70.59797	110.4903	70.51872	133.4684	70.5016	138.8928	70.60817	135.5633	70.53622	136.4719	70.6682	138.9858
70.61463	110.4871	70.53538	133.4691	70.51827	138.8897	70.62483	135.5637	70.55288	136.4728	70.68487	138.9842
70.6313	110.4869	70.55205	133.4699	70.53493	138.8898	70.6415	135.5613	70.56955	136.4731	70.70153	138.9836
70.64797	110.4834	70.56872	133.4702	70.5516	138.8867	70.65817	135.56	70.58622	136.4693	70.7182	138.9817
70.66463	110.4852	70.58538	133.4692	70.56827	138.8839	70.67483	135.5574	70.60288	136.4689	70.73487	138.9791
70.6813	110.4803	70.60205	133.4722	70.58493	138.8849	70.6915	135.5534	70.61955	136.4704	70.75153	138.979
70.69797	110.4805	70.61872	133.4712	70.6016	138.8858	70.70817	135.5511	70.63622	136.4656	70.7682	138.9778
70.71463	110.4769	70.63538	133.4752	70.61827	138.8826	70.72483	135.5543	70.65288	136.4654	70.78487	138.9764
70.7313	110.4772	70.65205	133.4736	70.63493	138.8798	70.7415	135.5486	70.66955	136.4663	70.80153	138.9748
70.74797	110.4739	70.66872	133.4773	70.6516	138.8758	70.75817	135.5496	70.68622	136.4644	70.8182	138.9776
70.76463	110.4732	70.68538	133.4772	70.66827	138.8764	70.77483	135.5468	70.70288	136.463	70.83487	138.9723
70.7813	110.4715	70.70205	133.4769	70.68493	138.8754	70.7915	135.5443	70.71955	136.4601	70.85153	138.9708
70.79797	110.4698	70.71872	133.4795	70.7016	138.8715	70.80817	135.5432	70.73622	136.4598	70.8682	138.9705
70.81463	110.469	70.73538	133.4793	70.71827	138.8697	70.82483	135.5417	70.75288	136.4605	70.88487	138.9716
70.8313	110.4663	70.75205	133.4787	70.73493	138.8696	70.8415	135.539	70.76955	136.4588	70.90153	138.9685
70.84797	110.468	70.76872	133.4797	70.7516	138.8651	70.85817	135.5385	70.78622	136.4584	70.9182	138.9664
70.86463	110.4649	70.78538	133.4771	70.76827	138.8639	70.87483	135.5379	70.80288	136.4576	70.93487	138.9651
70.8813	110.4627	70.80205	133.4804	70.78493	138.8602	70.8915	135.5345	70.81955	136.456	70.95153	138.966
70.89797	110.4617	70.81872	133.482	70.8016	138.8608	70.90817	135.5322	70.83622	136.4547	70.9682	138.9631
70.91463	110.4607	70.83538	133.4822	70.81827	138.8555	70.92483	135.5312	70.85288	136.4526	70.98487	138.9614
70.9313	110.4591	70.85205	133.4824	70.83493	138.8538	70.9415	135.5283	70.86955	136.4526	71.00153	138.9612
70.94797	110.456	70.86872	133.4821	70.8516	138.8533	70.95817	135.5258	70.88622	136.4527	71.0182	138.9609
70.96463	110.4558	70.88538	133.484	70.86827	138.8511	70.97483	135.5276	70.90288	136.4497	71.03487	138.9589
70.9813	110.455	70.90205	133.4839	70.88493	138.8482	70.9915	135.5238	70.91955	136.451	71.05153	138.9573
70.99797	110.4546	70.91872	133.4829	70.9016	138.8449	71.00817	135.5229	70.93622	136.4507	71.0682	138.9577
71.01463	110.4507	70.93538	133.484	70.91827	138.8432	71.02483	135.5195	70.95288	136.4454	71.08487	138.9579
71.0313	110.4508	70.95205	133.4835	70.93493	138.8431	71.0415	135.5178	70.96955	136.4454	71.10153	138.9547
71.04797	110.4475	70.96872	133.4836	70.9516	138.8405	71.05817	135.5148	70.98622	136.4441	71.1182	138.9551
71.06463	110.4483	70.98538	133.4822	70.96827	138.8398	71.07483	135.5166	71.00288	136.4445	71.13487	138.9534
71.0813	110.4459	71.00205	133.4855	70.98493	138.8368	71.0915	135.516	71.01955	136.4429	71.15153	138.954
71.09797	110.4445	71.01872	133.4817	71.0016	138.8369	71.10817	135.5126	71.03622	136.4422	71.1682	138.9493
71.11463	110.4434	71.03538	133.4847	71.01827	138.8346	71.12483	135.5102	71.05288	136.4409	71.18487	138.9491
71.1313	110.4401	71.05205	133.4869	71.03493	138.8313	71.1415	135.5103	71.06955	136.4372	71.20153	138.9506
71.14797	110.4405	71.06872	133.4872	71.0516	138.8311	71.15817	135.5086	71.08622	136.4378	71.2182	138.9486
71.16463	110.4376	71.08538	133.4868	71.06827	138.8292	71.17483	135.5066	71.10288	136.4385	71.23487	138.9463
71.1813	110.4364	71.10205	133.484	71.08493	138.8282	71.1915	135.5037	71.11955	136.4376	71.25153	138.946

71.19797	110.4366	71.11872	133.488	71.1016	138.8274	71.20817	135.5039	71.13622	136.4349	71.2682	138.945
71.21463	110.4331	71.13538	133.4848	71.11827	138.8264	71.22483	135.5039	71.15288	136.4351	71.28487	138.9428
71.2313	110.4337	71.15205	133.4867	71.13493	138.8236	71.2415	135.5011	71.16955	136.4327	71.30153	138.9424
71.24797	110.4313	71.16872	133.4847	71.1516	138.8207	71.25817	135.4988	71.18622	136.4312	71.3182	138.9423
71.26463	110.4311	71.18538	133.488	71.16827	138.8219	71.27483	135.4983	71.20288	136.432	71.33487	138.9416
71.2813	110.4298	71.20205	133.4891	71.18493	138.819	71.2915	135.4969	71.21955	136.4299	71.35153	138.9409
71.29797	110.4255	71.21872	133.4885	71.2016	138.818	71.30817	135.496	71.23622	136.4292	71.3682	138.94
71.31463	110.426	71.23538	133.4916	71.21827	138.8133	71.32483	135.4946	71.25288	136.4293	71.38487	138.9391
71.3313	110.425	71.25205	133.4871	71.23493	138.8131	71.3415	135.4928	71.26955	136.4283	71.40153	138.9357
71.34797	110.4235	71.26872	133.4894	71.2516	138.8107	71.35817	135.489	71.28622	136.4243	71.4182	138.9391
71.36463	110.4202	71.28538	133.4906	71.26827	138.8102	71.37483	135.4892	71.30288	136.426	71.43487	138.9363
71.3813	110.4192	71.30205	133.4913	71.28493	138.8085	71.3915	135.4875	71.31955	136.4255	71.45153	138.9365
71.39797	110.4178	71.31872	133.4923	71.3016	138.8052	71.40817	135.4882	71.33622	136.424	71.4682	138.9354
71.41463	110.4157	71.33538	133.4944	71.31827	138.8025	71.42483	135.4848	71.35288	136.4229	71.48487	138.9326
71.4313	110.4139	71.35205	133.4901	71.33493	138.8032	71.4415	135.4825	71.36955	136.4217	71.50153	138.9298
71.44797	110.4137	71.36872	133.4936	71.3516	138.8015	71.45817	135.4802	71.38622	136.4194	71.5182	138.931
71.46463	110.4135	71.38538	133.4948	71.36827	138.8003	71.47483	135.4812	71.40288	136.419	71.53487	138.9317
71.4813	110.408	71.40205	133.4945	71.38493	138.7976	71.4915	135.4818	71.41955	136.421	71.55153	138.9292
71.49797	110.407	71.41872	133.4961	71.4016	138.7973	71.50817	135.4802	71.43622	136.419	71.5682	138.9279
71.51463	110.4043	71.43538	133.4976	71.41827	138.7963	71.52483	135.4785	71.45288	136.4178	71.58487	138.927
71.5313	110.4074	71.45205	133.495	71.43493	138.794	71.5415	135.4766	71.46955	136.4154	71.60153	138.924
71.54797	110.3989	71.46872	133.497	71.4516	138.7913	71.55817	135.475	71.48622	136.4158	71.6182	138.9263
71.56463	110.4031	71.48538	133.4969	71.46827	138.7902	71.57483	135.4755	71.50288	136.4151	71.63487	138.9235
71.5813	110.397	71.50205	133.5012	71.48493	138.7891	71.5915	135.4726	71.51955	136.4151	71.65153	138.9231
71.59797	110.3978	71.51872	133.4972	71.5016	138.7861	71.60817	135.4709	71.53622	136.4159	71.6682	138.9226
71.61463	110.3957	71.53538	133.4996	71.51827	138.7836	71.62483	135.4679	71.55288	136.4134	71.68487	138.9236
71.6313	110.397	71.55205	133.4943	71.53493	138.7857	71.6415	135.4675	71.56955	136.4121	71.70153	138.9201
71.64797	110.3927	71.56872	133.4992	71.5516	138.7832	71.65817	135.4644	71.58622	136.4116	71.7182	138.9218
71.66463	110.3882	71.58538	133.4938	71.56827	138.7801	71.67483	135.4653	71.60288	136.413	71.73487	138.9176
71.6813	110.3884	71.60205	133.5002	71.58493	138.7804	71.6915	135.4634	71.61955	136.4088	71.75153	138.9183
71.69797	110.388	71.61872	133.4976	71.6016	138.7785	71.70817	135.4634	71.63622	136.4076	71.7682	138.9174
71.71463	110.384	71.63538	133.4957	71.61827	138.7758	71.72483	135.4609	71.65288	136.4077	71.78487	138.9164
71.7313	110.3866	71.65205	133.4948	71.63493	138.7743	71.7415	135.4574	71.66955	136.4087	71.80153	138.9153
71.74797	110.3815	71.66872	133.4976	71.6516	138.7713	71.75817	135.4574	71.68622	136.4074	71.8182	138.9167
71.76463	110.3808	71.68538	133.4972	71.66827	138.7702	71.77483	135.4556	71.70288	136.4059	71.83487	138.9127
71.7813	110.3785	71.70205	133.4996	71.68493	138.7665	71.7915	135.4514	71.71955	136.4076	71.85153	138.9156
71.79797	110.379	71.71872	133.4947	71.7016	138.7675	71.80817	135.4511	71.73622	136.4048	71.8682	138.9123
71.81463	110.3785	71.73538	133.4972	71.71827	138.763	71.82483	135.4521	71.75288	136.402	71.88487	138.9122
71.8313	110.3731	71.75205	133.4971	71.73493	138.7625	71.8415	135.4493	71.76955	136.4021	71.90153	138.9095
71.84797	110.3724	71.76872	133.5	71.7516	138.7564	71.85817	135.4462	71.78622	136.4007	71.9182	138.914
71.86463	110.3695	71.78538	133.5002	71.76827	138.7586	71.87483	135.4484	71.80288	136.399	71.93487	138.908
71.8813	110.3714	71.80205	133.4998	71.78493	138.7581	71.8915	135.4452	71.81955	136.4004	71.95153	138.91
71.89797	110.3695	71.81872	133.501	71.8016	138.7534	71.90817	135.4442	71.83622	136.3989	71.9682	138.908
71.91463	110.367	71.83538	133.5037	71.81827	138.7512	71.92483	135.4419	71.85288	136.3983	71.98487	138.9083
71.9313	110.3687	71.85205	133.5046	71.83493	138.7541	71.9415	135.4447	71.86955	136.3964	72.00153	138.9067
71.94797	110.3638	71.86872	133.5035	71.8516	138.7494	71.95817	135.4415	71.88622	136.3988	72.0182	138.9086
71.96463	110.3641	71.88538	133.506	71.86827	138.7481	71.97483	135.4395	71.90288	136.3949	72.03487	138.9064
71.9813	110.3596	71.90205	133.5015	71.88493	138.7477	71.9915	135.4362	71.91955	136.394	72.05153	138.9046
71.99797	110.3608	71.91872	133.5095	71.9016	138.7475	72.00817	135.4371	71.93622	136.3955	72.0682	138.9021
72.01463	110.3571	71.93538	133.5101	71.91827	138.742	72.02483	135.4366	71.95288	136.3932	72.08487	138.903
72.0313	110.3552	71.95205	133.5052	71.93493	138.7411	72.0415	135.4357	71.96955	136.3925	72.10153	138.9021
72.04797	110.3544	71.96872	133.5108	71.9516	138.7397	72.05817	135.43	71.98622	136.3909	72.1182	138.9006
72.06463	110.3513	71.98538	133.51	71.96827	138.7388	72.07483	135.4326	72.00288	136.3932	72.13487	138.8998
72.0813	110.3505	72.00205	133.5108	71.98493	138.7352	72.0915	135.4298	72.01955	136.3885	72.15153	138.8983
72.09797	110.3514	72.01872	133.5148	72.0016	138.735	72.10817	135.4284	72.03622	136.3855	72.1682	138.8969
72.11463	110.348	72.03538	133.5156	72.01827	138.7297	72.12483	135.4262	72.05288	136.3872	72.18487	138.8969
72.1313	110.3465	72.05205	133.5141	72.03493	138.7245	72.1415	135.4275	72.06955	136.3835	72.20153	138.897
72.14797	110.3445	72.06872	133.5166	72.0516	138.7269	72.15817	135.4228	72.08622	136.386	72.2182	138.8934
72.16463	110.3445	72.08538	133.5163	72.06827	138.7244	72.17483	135.4205	72.10288	136.3841	72.23487	138.8937
72.1813	110.3419	72.10205	133.5174	72.08493	138.7224	72.1915	135.4209	72.11955	136.3823	72.25153	138.8916
72.19797	110.3405	72.11872	133.517	72.1016	138.7169	72.20817	135.42	72.13622	136.3825	72.2682	138.8919
72.21463	110.3395	72.13538	133.5188	72.11827	138.7187	72.22483	135.4176	72.15288	136.3841	72.28487	138.891
72.2313	110.3385	72.15205	133.5173	72.13493	138.7153	72.2415	135.415	72.16955	136.3802	72.30153	138.8902
72.24797	110.3342	72.16872	133.5207	72.1516	138.7135	72.25817	135.415	72.18622	136.3809	72.3182	138.8887
72.26463	110.3356	72.18538	133.5198	72.16827	138.7127	72.27483	135.4145	72.20288	136.3804	72.33487	138.8866
72.2813	110.337	72.20205	133.5245	72.18493	138.7108	72.2915	135.4121	72.21955	136.379	72.35153	138.8879
72.29797	110.3346	72.21872	133.5235	72.2016	138.7077	72.30817	135.4132	72.23622	136.3755	72.3682	138.8866
72.31463	110.3293	72.23538	133.5238	72.21827	138.7084	72.32483	135.4103	72.25288	136.3775	72.38487	138.8867
72.3313	110.3302	72.25205	133.5229	72.23493	138.7022	72.3415	135.409	72.26955	136.3765	72.40153	138.8856
72.34797	110.3299	72.26872	133.5254	72.2516	138.7034	72.35817	135.408	72.28622	136.3762	72.4182	138.8842
72.36463	110.328	72.28538	133.5246	72.26827	138.6993	72.37483	135.4091	72.30288	136.3714	72.43487	138.8835
72.3813	110.3247	72.30205	133.5252	72.28493	138.6988	72.3915	135.406	72.31955	136.3733	72.45153	138.8819
72.39797	110.3262	72.31872	133.5261	72.3016	138.6949	72.40817	135.4052	72.33622	136.3706	72.4682	138.8821
72.41463	110.3204	72.33538	133.5288	72.31827	138.6963	72.42483	135.4014	72.35288	136.3712	72.48487	138.8797
72.4313	110.3206	72.35205	133.5244	72.33493	138.6932	72.4415	135.4034	72.36955	136.3723	72.50153	138.8797
72.44797	110.3227	72.36872	133.5294	72.3516	138.6957	72.45817	135.4012	72.38622	136.3703	72.5182	138.8791
72.46463	110.3186	72.38538	133.5298	72.36827	138.6924	72.47483	135.4024	72.40288	136.3689	72.53487	138.8798
72.4813	110.3167	72.40205	133.5296	72.38493	138.6897	72.4915	135.3961	72.41955	136.3673	72.55153	138.878
72.49797	110.3192	72.41872	133.5316	72.4016	138.6881	72.50817	135.3986	72.43622	136.3685	72.5682	138.8756
72.51463	110.3173	72.43538	133.5324	72.41827	138.6833	72.52483	135.3921	72.45288	136.3656	72.58487	138.8744
72.5313	110.3102	72.45205	133.5372	72.43493	138.6819	72.5415	135.3932	72.46955	136.3637	72.60153	138.8723
72.54797	110.3121	72.46872	133.5356	72.4516	138.6791	72.55817	135.3918	72.48622	136.3621	72.6182	138.874
72.56463	110.3095	72.48538	133.5335	72.46827	138.6776	72.57483	135.3887	72.50288	136.3632	72.63487	138.8715
72.5813	110.3097	72.50205	133.5385	72.48493	138.6763	72.5915	135.3878	72.51955	136.363	72.65153	138.8734
72.59797	110.3098	72.51872	133.5373	72.5016	138.6736	72.60817	135.3848	72.53622	136.3602	72.6682	138.8692
72.61463	110.3097	72.53538	133.5374	72.51827	138.6721	72.62483	135.386	72.55288	136.3611	72.68487	138.8697
72.6313	110.3039	72.55205	133.5373	72.53493	138.6719	72.6415	135.384	72.56955	136.3574	72.70153	138.8685
72.64797	110.3042	72.56872	133.5399	72.5516	138.6699	72.65817	135.3814	72.58622	136.3554	72.7182	138.8695
72.66463	110.2966	72.58538	133.5371	72.56827	138.6693	72.67483	135.3802	72.60288	136.3561	72.73487	138.8658
72.6813	110.3002	72.60205	133.5382	72.58493	138.6653	72.6915	135.377	72.61955	136.3573	72.75153	138.866
72.69797	110.3023	72.61872	133.5409	72.6016	138.665	72.70817	135.3779	72.63622	136.3552	72.7682	138.8641
72.71463	110.296	72.63538	133.5398	72.61827	138.6621	72.72483	135.3733	72.65288	136.3543	72.78487	138.8647
72.7313	110.2954	72.65205	133.5397	72.63493	138.6606	72.7415	135.3756	72.66955	136.3534	72.80153	138.8633
72.74797	110.2911	72.66872	133.5399	72.6516	138.6582	72.75817	135.3703	72.68622	136.3537	72.8182	138.8651
72.76463	110.2946	72.68538	133.5399	72.66827	138.657	72.77483	135.3714	72.70288	136.3509	72.83487	138.86
72.7813	110.2924	72.70205	133.5411	72.68493	138.6534	72.7915	135.3703	72.71955	136.349	72.85153	138.86
72.79797	110.2906	72.71872	133.54	72.7016	138.6537	72.80817	135.3668	72.73622	136.3487	72.8682	138.8588

72.81463	110.2873	72.73538	133.5426	72.71827	138.6511	72.82483	135.3669	72.75288	136.3473	72.88487	138.8587
72.8313	110.2887	72.75205	133.5393	72.73493	138.6462	72.8415	135.3652	72.76955	136.3483	72.90153	138.8577
72.84797	110.288	72.76872	133.5408	72.7516	138.6444	72.85817	135.3592	72.78622	136.3427	72.9182	138.855
72.86463	110.2828	72.78538	133.5428	72.76827	138.6431	72.87483	135.3611	72.80288	136.3443	72.93487	138.856
72.8813	110.2833	72.80205	133.5435	72.78493	138.6386	72.8915	135.3603	72.81955	136.3416	72.95153	138.8538
72.89797	110.2808	72.81872	133.5436	72.8016	138.636	72.90817	135.3584	72.83622	136.3432	72.9682	138.8523
72.91463	110.2783	72.83538	133.5431	72.81827	138.6363	72.92483	135.3566	72.85288	136.3402	72.98487	138.8529
72.9313	110.2776	72.85205	133.5428	72.83493	138.633	72.9415	135.3538	72.86955	136.3388	73.00153	138.8499
72.94797	110.2759	72.86872	133.544	72.8516	138.6309	72.95817	135.3518	72.88622	136.3383	73.0182	138.8492
72.96463	110.2757	72.88538	133.5458	72.86827	138.6275	72.97483	135.3521	72.90288	136.3378	73.03487	138.8493
72.9813	110.2737	72.90205	133.5453	72.88493	138.6286	72.9915	135.3497	72.91955	136.3355	73.05153	138.8478
72.99797	110.2719	72.91872	133.5433	72.9016	138.6273	73.00817	135.348	72.93622	136.3359	73.0682	138.8474
73.01463	110.2705	72.93538	133.5435	72.91827	138.6216	73.02483	135.3452	72.95288	136.336	73.08487	138.8474
73.0313	110.2704	72.95205	133.5469	72.93493	138.6195	73.0415	135.3477	72.96955	136.3326	73.10153	138.8444
73.04797	110.2659	72.96872	133.5502	72.9516	138.6193	73.05817	135.3439	72.98622	136.3335	73.1182	138.8459
73.06463	110.2658	72.98538	133.5471	72.96827	138.6173	73.07483	135.3419	73.00288	136.3303	73.13487	138.845
73.0813	110.2639	73.00205	133.5468	72.98493	138.615	73.0915	135.3399	73.01955	136.3287	73.15153	138.8395
73.09797	110.2605	73.01872	133.549	73.0016	138.6133	73.10817	135.3388	73.03622	136.3285	73.1682	138.8417
73.11463	110.2614	73.03538	133.5517	73.01827	138.6108	73.12483	135.3357	73.05288	136.3271	73.18487	138.8414
73.1313	110.2609	73.05205	133.5504	73.03493	138.6094	73.1415	135.3373	73.06955	136.3266	73.20153	138.8404
73.14797	110.2549	73.06872	133.554	73.0516	138.6069	73.15817	135.3324	73.08622	136.3242	73.2182	138.8416
73.16463	110.2569	73.08538	133.5528	73.06827	138.6057	73.17483	135.3334	73.10288	136.3264	73.23487	138.84
73.1813	110.2551	73.10205	133.5551	73.08493	138.6058	73.1915	135.3289	73.11955	136.3236	73.25153	138.838
73.19797	110.2546	73.11872	133.5571	73.1016	138.6004	73.20817	135.331	73.13622	136.3238	73.2682	138.8373
73.21463	110.2511	73.13538	133.5575	73.11827	138.5984	73.22483	135.3285	73.15288	136.3224	73.28487	138.8386
73.2313	110.2496	73.15205	133.5577	73.13493	138.5958	73.2415	135.3286	73.16955	136.3218	73.30153	138.8354
73.24797	110.2475	73.16872	133.5592	73.1516	138.596	73.25817	135.3248	73.18622	136.3208	73.3182	138.835
73.26463	110.2446	73.18538	133.5585	73.16827	138.5927	73.27483	135.3243	73.20288	136.3183	73.33487	138.8331
73.2813	110.2455	73.20205	133.559	73.18493	138.5897	73.2915	135.3213	73.21955	136.318	73.35153	138.833
73.29797	110.2441	73.21872	133.5572	73.2016	138.5898	73.30817	135.3211	73.23622	136.3178	73.3682	138.8338
73.31463	110.2415	73.23538	133.5619	73.21827	138.5883	73.32483	135.3213	73.25288	136.3156	73.38487	138.8333
73.3313	110.2407	73.25205	133.5589	73.23493	138.5863	73.3415	135.3165	73.26955	136.3155	73.40153	138.8312
73.34797	110.2398	73.26872	133.5629	73.2516	138.5837	73.35817	135.3185	73.28622	136.3119	73.4182	138.829
73.36463	110.2379	73.28538	133.5628	73.26827	138.5812	73.37483	135.3165	73.30288	136.3114	73.43487	138.8277
73.3813	110.2357	73.30205	133.5629	73.28493	138.5789	73.3915	135.3146	73.31955	136.314	73.45153	138.8282
73.39797	110.2342	73.31872	133.5613	73.3016	138.577	73.40817	135.3123	73.33622	136.3122	73.4682	138.8255
73.41463	110.2318	73.33538	133.5633	73.31827	138.5746	73.42483	135.3127	73.35288	136.3085	73.48487	138.8288
73.4313	110.2315	73.35205	133.5591	73.33493	138.5725	73.4415	135.3111	73.36955	136.3093	73.50153	138.8281
73.44797	110.2281	73.36872	133.5608	73.3516	138.57	73.45817	135.3117	73.38622	136.3082	73.5182	138.8248
73.46463	110.2295	73.38538	133.5622	73.36827	138.5715	73.47483	135.3105	73.40288	136.3076	73.53487	138.8249
73.4813	110.2269	73.40205	133.5595	73.38493	138.5676	73.4915	135.3053	73.41955	136.3063	73.55153	138.8238
73.49797	110.225	73.41872	133.5604	73.4016	138.567	73.50817	135.3044	73.43622	136.3039	73.5682	138.8237
73.51463	110.2263	73.43538	133.563	73.41827	138.5641	73.52483	135.3021	73.45288	136.3022	73.58487	138.8248
73.5313	110.221	73.45205	133.564	73.43493	138.5617	73.5415	135.3023	73.46955	136.3033	73.60153	138.8189
73.54797	110.2187	73.46872	133.5582	73.4516	138.5604	73.55817	135.2998	73.48622	136.3017	73.6182	138.8212
73.56463	110.2209	73.48538	133.562	73.46827	138.5586	73.57483	135.2984	73.50288	136.3017	73.63487	138.8199
73.5813	110.2176	73.50205	133.5661	73.48493	138.5581	73.5915	135.2983	73.51955	136.3017	73.65153	138.8177
73.59797	110.2164	73.51872	133.5612	73.5016	138.5547	73.60817	135.2962	73.53622	136.3003	73.6682	138.8149
73.61463	110.2151	73.53538	133.5641	73.51827	138.5504	73.62483	135.2946	73.55288	136.2996	73.68487	138.8195
73.6313	110.2153	73.55205	133.5632	73.53493	138.5497	73.6415	135.2965	73.56955	136.3003	73.70153	138.8168
73.64797	110.2136	73.56872	133.5632	73.5516	138.5501	73.65817	135.2904	73.58622	136.2975	73.7182	138.8162
73.66463	110.2097	73.58538	133.5625	73.56827	138.5462	73.67483	135.2924	73.60288	136.2979	73.73487	138.813
73.6813	110.2093	73.60205	133.5628	73.58493	138.5428	73.6915	135.289	73.61955	136.2953	73.75153	138.8143
73.69797	110.2097	73.61872	133.5652	73.6016	138.5391	73.70817	135.2877	73.63622	136.2946	73.7682	138.8132
73.71463	110.2056	73.63538	133.5647	73.61827	138.5401	73.72483	135.286	73.65288	136.2985	73.78487	138.8119
73.7313	110.2043	73.65205	133.5678	73.63493	138.5385	73.7415	135.2865	73.66955	136.2931	73.80153	138.8133
73.74797	110.2007	73.66872	133.567	73.6516	138.5372	73.75817	135.285	73.68622	136.2925	73.8182	138.8105
73.76463	110.2008	73.68538	133.5659	73.66827	138.5367	73.77483	135.2835	73.70288	136.2929	73.83487	138.8074
73.7813	110.1989	73.70205	133.5681	73.68493	138.5317	73.7915	135.2788	73.71955	136.289	73.85153	138.8088
73.79797	110.1971	73.71872	133.5723	73.7016	138.532	73.80817	135.2788	73.73622	136.2912	73.8682	138.8082
73.81463	110.196	73.73538	133.5719	73.71827	138.5271	73.82483	135.2762	73.75288	136.2896	73.88487	138.8085
73.8313	110.1912	73.75205	133.5744	73.73493	138.5294	73.8415	135.2756	73.76955	136.2888	73.90153	138.8059
73.84797	110.1918	73.76872	133.5742	73.7516	138.5273	73.85817	135.2761	73.78622	136.2886	73.9182	138.8043
73.86463	110.1899	73.78538	133.5747	73.76827	138.5233	73.87483	135.2741	73.80288	136.289	73.93487	138.8051
73.8813	110.1868	73.80205	133.5796	73.78493	138.5195	73.8915	135.274	73.81955	136.2886	73.95153	138.8047
73.89797	110.1861	73.81872	133.5746	73.8016	138.5221	73.90817	135.2744	73.83622	136.2875	73.9682	138.8014
73.91463	110.1854	73.83538	133.5778	73.81827	138.5186	73.92483	135.2726	73.85288	136.2862	73.98487	138.802
73.9313	110.1833	73.85205	133.5802	73.83493	138.5169	73.9415	135.2711	73.86955	136.2839	74.00153	138.7996
73.94797	110.1807	73.86872	133.5784	73.8516	138.5153	73.95817	135.2673	73.88622	136.2821	74.0182	138.8014
73.96463	110.1832	73.88538	133.582	73.86827	138.5142	73.97483	135.2684	73.90288	136.2829	74.03487	138.7995
73.9813	110.179	73.90205	133.5828	73.88493	138.5135	73.9915	135.2687	73.91955	136.2801	74.05153	138.7973
73.99797	110.1769	73.91872	133.5789	73.9016	138.5095	74.00817	135.2664	73.93622	136.2797	74.0682	138.7981
74.01463	110.1737	73.93538	133.5845	73.91827	138.5084	74.02483	135.2651	73.95288	136.2801	74.08487	138.7951
74.0313	110.177	73.95205	133.5859	73.93493	138.5029	74.0415	135.2614	73.96955	136.2775	74.10153	138.7943
74.04797	110.1718	73.96872	133.5853	73.9516	138.504	74.05817	135.2612	73.98622	136.277	74.1182	138.7945
74.06463	110.1714	73.98538	133.5868	73.96827	138.5029	74.07483	135.2629	74.00288	136.2755	74.13487	138.7948
74.0813	110.1686	74.00205	133.5899	73.98493	138.498	74.0915	135.2584	74.01955	136.2732	74.15153	138.7923
74.09797	110.1677	74.01872	133.5912	74.0016	138.4979	74.10817	135.2562	74.03622	136.275	74.1682	138.7923
74.11463	110.1654	74.03538	133.5921	74.01827	138.4986	74.12483	135.2546	74.05288	136.272	74.18487	138.7923
74.1313	110.1638	74.05205	133.5929	74.03493	138.496	74.1415	135.2542	74.06955	136.2722	74.20153	138.7895
74.14797	110.1635	74.06872	133.5869	74.0516	138.4924	74.15817	135.254	74.08622	136.2699	74.2182	138.7885
74.16463	110.1622	74.08538	133.5904	74.06827	138.4882	74.17483	135.2514	74.10288	136.2722	74.23487	138.7873
74.1813	110.1607	74.10205	133.592	74.08493	138.4888	74.1915	135.2496	74.11955	136.2693	74.25153	138.7881
74.19797	110.1607	74.11872	133.5929	74.1016	138.4875	74.20817	135.2487	74.13622	136.2676	74.2682	138.785
74.21463	110.1562	74.13538	133.5935	74.11827	138.4843	74.22483	135.2503	74.15288	136.2642	74.28487	138.7854
74.2313	110.1558	74.15205	133.5918	74.13493	138.4842	74.2415	135.247	74.16955	136.267	74.30153	138.7827
74.24797	110.155	74.16872	133.5958	74.1516	138.4813	74.25817	135.2456	74.18622	136.2639	74.3182	138.7817
74.26463	110.1533	74.18538	133.5961	74.16827	138.4794	74.27483	135.2434	74.20288	136.2657	74.33487	138.7843
74.2813	110.1506	74.20205	133.5979	74.18493	138.4756	74.2915	135.2443	74.21955	136.2638	74.35153	138.7826
74.29797	110.1512	74.21872	133.598	74.2016	138.4772	74.30817	135.2424	74.23622	136.2616	74.3682	138.7793
74.31463	110.1495	74.23538	133.5947	74.21827	138.4733	74.32483	135.2387	74.25288	136.2613	74.38487	138.778
74.3313	110.1463	74.25205	133.5995	74.23493	138.4699	74.3415	135.2395	74.26955	136.2618	74.40153	138.7791
74.34797	110.1441	74.26872	133.6014	74.2516	138.4669	74.35817	135.2352	74.28622	136.2613	74.4182	138.7784
74.36463	110.144	74.28538	133.6001	74.26827	138.4685	74.37483	135.2373	74.30288	136.2607	74.43487	138.776
74.3813	110.1407	74.30205	133.5974	74.28493	138.464	74.3915	135.2326	74.31955	136.2586	74.45153	138.7772
74.39797	110.1426	74.31872	133.6004	74.3016	138.4634	74.40817	135.2316	74.33622	136.2571	74.4682	138.7758
74.41463	110.1329	74.33538	133.6003	74.31827	138.4595	74.42483	135.2292	74.35288	136.2574	74.48487	138.7753

74.4313	110.1362	74.35205	133.6011	74.33493	138.4595	74.4415	135.2314	74.36955	136.2576	74.50153	138.7747
74.44797	110.1366	74.36872	133.6027	74.3516	138.4573	74.45817	135.2257	74.38622	136.2549	74.5182	138.7701
74.46463	110.1325	74.38538	133.6027	74.36827	138.4565	74.47483	135.2243	74.40288	136.2529	74.53487	138.7697
74.4813	110.1327	74.40205	133.6021	74.38493	138.4559	74.4915	135.2252	74.41955	136.2544	74.55153	138.7723
74.49797	110.1332	74.41872	133.6017	74.4016	138.4514	74.50817	135.2254	74.43622	136.2531	74.5682	138.7708
74.51463	110.1282	74.43538	133.603	74.41827	138.4489	74.52483	135.2206	74.45288	136.2523	74.58487	138.7715
74.5313	110.1291	74.45205	133.6026	74.43493	138.4466	74.5415	135.2227	74.46955	136.2469	74.60153	138.7705
74.54797	110.1267	74.46872	133.6019	74.4516	138.4458	74.55817	135.2211	74.48622	136.2498	74.6182	138.7678
74.56463	110.1239	74.48538	133.6055	74.46827	138.4416	74.57483	135.2192	74.50288	136.2486	74.63487	138.7676
74.5813	110.1266	74.50205	133.6013	74.48493	138.4429	74.5915	135.2176	74.51955	136.2458	74.65153	138.7659
74.59797	110.1248	74.51872	133.603	74.5016	138.4412	74.60817	135.2154	74.53622	136.2484	74.6682	138.7659
74.61463	110.123	74.53538	133.6044	74.51827	138.4402	74.62483	135.2131	74.55288	136.2477	74.68487	138.7648
74.6313	110.1206	74.55205	133.6031	74.53493	138.4357	74.6415	135.2111	74.56955	136.2442	74.70153	138.7631
74.64797	110.1179	74.56872	133.6048	74.5516	138.4334	74.65817	135.2082	74.58622	136.2443	74.7182	138.7623
74.66463	110.1171	74.58538	133.6047	74.56827	138.4332	74.67483	135.2066	74.60288	136.2416	74.73487	138.7625
74.6813	110.1166	74.60205	133.6041	74.58493	138.4319	74.6915	135.2073	74.61955	136.2421	74.75153	138.761
74.69797	110.1146	74.61872	133.6058	74.6016	138.4313	74.70817	135.2067	74.63622	136.2421	74.7682	138.7608
74.71463	110.1146	74.63538	133.6069	74.61827	138.4295	74.72483	135.2052	74.65288	136.2417	74.78487	138.762
74.7313	110.1117	74.65205	133.6052	74.63493	138.4285	74.7415	135.2055	74.66955	136.2396	74.80153	138.7594
74.74797	110.1104	74.66872	133.6048	74.6516	138.4246	74.75817	135.2035	74.68622	136.2373	74.8182	138.7593
74.76463	110.1082	74.68538	133.608	74.66827	138.4271	74.77483	135.2026	74.70288	136.236	74.83487	138.7607
74.7813	110.1079	74.70205	133.6081	74.68493	138.4227	74.7915	135.1982	74.71955	136.2357	74.85153	138.757
74.79797	110.106	74.71872	133.6104	74.7016	138.4224	74.80817	135.1987	74.73622	136.2373	74.8682	138.7577
74.81463	110.1048	74.73538	133.6068	74.71827	138.4223	74.82483	135.1966	74.75288	136.2369	74.88487	138.7549
74.8313	110.1033	74.75205	133.6097	74.73493	138.4177	74.8415	135.1951	74.76955	136.2322	74.90153	138.7559
74.84797	110.0997	74.76872	133.6097	74.7516	138.4135	74.85817	135.1945	74.78622	136.2336	74.9182	138.7527
74.86463	110.0999	74.78538	133.6088	74.76827	138.4132	74.87483	135.1948	74.80288	136.2333	74.93487	138.7538
74.8813	110.0981	74.80205	133.6115	74.78493	138.4125	74.8915	135.1922	74.81955	136.2309	74.95153	138.7538
74.89797	110.0971	74.81872	133.6098	74.8016	138.4086	74.90817	135.1924	74.83622	136.2314	74.9682	138.7532
74.91463	110.0965	74.83538	133.6101	74.81827	138.4078	74.92483	135.1875	74.85288	136.2305	74.98487	138.7513
74.9313	110.0936	74.85205	133.61	74.83493	138.4029	74.9415	135.1875	74.86955	136.2299	75.00153	138.7499
74.94797	110.0918	74.86872	133.6114	74.8516	138.4012	74.95817	135.1846	74.88622	136.2285	75.0182	138.7504
74.96463	110.0932	74.88538	133.6117	74.86827	138.3974	74.97483	135.1864	74.90288	136.2287	75.03487	138.7475
74.9813	110.0903	74.90205	133.6132	74.88493	138.3971	74.9915	135.1807	74.91955	136.2287	75.05153	138.7459
74.99797	110.0908	74.91872	133.6123	74.9016	138.3968	75.00817	135.181	74.93622	136.2261	75.0682	138.7466
75.01463	110.0886	74.93538	133.6146	74.91827	138.3915	75.02483	135.1802	74.95288	136.2238	75.08487	138.7472
75.0313	110.0885	74.95205	133.6153	74.93493	138.3894	75.0415	135.1807	74.96955	136.2247	75.10153	138.7452
75.04797	110.0839	74.96872	133.6148	74.9516	138.3881	75.05817	135.1759	74.98622	136.2237	75.1182	138.7444
75.06463	110.0845	74.98538	133.6152	74.96827	138.3876	75.07483	135.1753	75.00288	136.2224	75.13487	138.7417
75.0813	110.0831	75.00205	133.6182	74.98493	138.3813	75.0915	135.174	75.01955	136.2227	75.15153	138.7428
75.09797	110.0867	75.01872	133.6184	75.0016	138.3856	75.10817	135.1729	75.03622	136.22	75.1682	138.7396
75.11463	110.0786	75.03538	133.6169	75.01827	138.3807	75.12483	135.1703	75.05288	136.2223	75.18487	138.7372
75.1313	110.0772	75.05205	133.6183	75.03493	138.3772	75.1415	135.1705	75.06955	136.218	75.20153	138.739
75.14797	110.0763	75.06872	133.6168	75.0516	138.3736	75.15817	135.1686	75.08622	136.2197	75.2182	138.7388
75.16463	110.077	75.08538	133.6172	75.06827	138.3767	75.17483	135.1696	75.10288	136.2197	75.23487	138.7371
75.1813	110.0725	75.10205	133.6185	75.08493	138.3732	75.1915	135.1646	75.11955	136.218	75.25153	138.7359
75.19797	110.0737	75.11872	133.617	75.1016	138.3749	75.20817	135.1658	75.13622	136.2164	75.2682	138.7333
75.21463	110.0707	75.13538	133.6169	75.11827	138.3679	75.22483	135.164	75.15288	136.2146	75.28487	138.7336
75.2313	110.0705	75.15205	133.618	75.13493	138.369	75.2415	135.1636	75.16955	136.2154	75.30153	138.732
75.24797	110.0661	75.16872	133.6179	75.1516	138.3664	75.25817	135.1585	75.18622	136.2124	75.3182	138.7324
75.26463	110.0659	75.18538	133.6171	75.16827	138.363	75.27483	135.1616	75.20288	136.214	75.33487	138.7314
75.2813	110.0662	75.20205	133.6137	75.18493	138.3574	75.2915	135.1594	75.21955	136.2103	75.35153	138.7324
75.29797	110.0592	75.21872	133.6151	75.2016	138.3614	75.30817	135.1601	75.23622	136.2115	75.3682	138.7303
75.31463	110.0616	75.23538	133.6173	75.21827	138.3572	75.32483	135.1592	75.25288	136.2087	75.38487	138.7274
75.3313	110.0588	75.25205	133.6165	75.23493	138.3556	75.3415	135.1577	75.26955	136.2098	75.40153	138.7241
75.34797	110.0576	75.26872	133.6158	75.2516	138.3558	75.35817	135.1544	75.28622	136.208	75.4182	138.7249
75.36463	110.057	75.28538	133.6141	75.26827	138.3503	75.37483	135.1533	75.30288	136.2057	75.43487	138.7248
75.3813	110.0526	75.30205	133.6178	75.28493	138.3521	75.3915	135.1498	75.31955	136.206	75.45153	138.7252
75.39797	110.0537	75.31872	133.6156	75.3016	138.3478	75.40817	135.1524	75.33622	136.2065	75.4682	138.725
75.41463	110.0466	75.33538	133.6149	75.31827	138.3425	75.42483	135.1485	75.35288	136.2051	75.48487	138.7196
75.4313	110.0506	75.35205	133.6152	75.33493	138.3456	75.4415	135.1474	75.36955	136.2034	75.50153	138.7222
75.44797	110.0503	75.36872	133.6163	75.3516	138.3399	75.45817	135.1439	75.38622	136.2031	75.5182	138.7198
75.46463	110.0437	75.38538	133.6157	75.36827	138.3387	75.47483	135.146	75.40288	136.2021	75.53487	138.7183
75.4813	110.0442	75.40205	133.6158	75.38493	138.3346	75.4915	135.1429	75.41955	136.2001	75.55153	138.717
75.49797	110.0448	75.41872	133.6153	75.4016	138.3383	75.50817	135.1422	75.43622	136.2013	75.5682	138.7172
75.51463	110.0411	75.43538	133.6122	75.41827	138.3313	75.52483	135.1421	75.45288	136.1989	75.58487	138.7145
75.5313	110.0415	75.45205	133.6127	75.43493	138.3288	75.5415	135.1427	75.46955	136.197	75.60153	138.716
75.54797	110.039	75.46872	133.6152	75.4516	138.3309	75.55817	135.1387	75.48622	136.1969	75.6182	138.7161
75.56463	110.0374	75.48538	133.6163	75.46827	138.3302	75.57483	135.1382	75.50288	136.1951	75.63487	138.7131
75.5813	110.0358	75.50205	133.6133	75.48493	138.3233	75.5915	135.1371	75.51955	136.1951	75.65153	138.709
75.59797	110.0334	75.51872	133.6158	75.5016	138.3254	75.60817	135.1364	75.53622	136.196	75.6682	138.7095
75.61463	110.0318	75.53538	133.6154	75.51827	138.325	75.62483	135.1363	75.55288	136.1944	75.68487	138.708
75.6313	110.0336	75.55205	133.6145	75.53493	138.3216	75.6415	135.1323	75.56955	136.1924	75.70153	138.7078
75.64797	110.0302	75.56872	133.6165	75.5516	138.3217	75.65817	135.1313	75.58622	136.193	75.7182	138.7061
75.66463	110.0297	75.58538	133.6115	75.56827	138.3191	75.67483	135.1285	75.60288	136.197	75.73487	138.705
75.6813	110.0299	75.60205	133.6127	75.58493	138.3169	75.6915	135.1293	75.61955	136.1895	75.75153	138.7044
75.69797	110.0248	75.61872	133.6148	75.6016	138.3166	75.70817	135.125	75.63622	136.191	75.7682	138.7016
75.71463	110.0246	75.63538	133.6121	75.61827	138.3136	75.72483	135.1251	75.65288	136.1889	75.78487	138.7016
75.7313	110.027	75.65205	133.6129	75.63493	138.3125	75.7415	135.1239	75.66955	136.1909	75.80153	138.7006
75.74797	110.0213	75.66872	133.6142	75.6516	138.3126	75.75817	135.1247	75.68622	136.1914	75.8182	138.6969
75.76463	110.0213	75.68538	133.615	75.66827	138.3095	75.77483	135.1207	75.70288	136.1894	75.83487	138.6986
75.7813	110.0167	75.70205	133.6126	75.68493	138.3077	75.7915	135.1199	75.71955	136.1886	75.85153	138.6974
75.79797	110.0157	75.71872	133.6184	75.7016	138.305	75.80817	135.1185	75.73622	136.1864	75.8682	138.6995
75.81463	110.0152	75.73538	133.6146	75.71827	138.3015	75.82483	135.1195	75.75288	136.1881	75.88487	138.6952
75.8313	110.0126	75.75205	133.6176	75.73493	138.2994	75.8415	135.1179	75.76955	136.1848	75.90153	138.6962
75.84797	110.0121	75.76872	133.6166	75.7516	138.298	75.85817	135.1151	75.78622	136.1821	75.9182	138.6969
75.86463	110.0134	75.78538	133.6176	75.76827	138.2973	75.87483	135.1142	75.80288	136.1825	75.93487	138.6962
75.8813	110.0084	75.80205	133.6204	75.78493	138.2937	75.8915	135.1124	75.81955	136.1808	75.95153	138.6956
75.89797	110.0026	75.81872	133.6184	75.8016	138.2927	75.90817	135.11	75.83622	136.1783	75.9682	138.6944
75.91463	110.0059	75.83538	133.6157	75.81827	138.2917	75.92483	135.1104	75.85288	136.1798	75.98487	138.693
75.9313	110.0056	75.85205	133.6179	75.83493	138.2877	75.9415	135.1084	75.86955	136.1796	76.00153	138.6929
75.94797	110.0025	75.86872	133.6163	75.8516	138.2855	75.95817	135.1072	75.88622	136.1778	76.0182	138.6934
75.96463	110.0039	75.88538	133.6172	75.86827	138.2831	75.97483	135.1062	75.90288	136.1798	76.03487	138.694
75.9813	109.9975	75.90205	133.6169	75.88493	138.2801	75.9915	135.1061	75.91955	136.1755	76.05153	138.6907
75.99797	109.997	75.91872	133.6184	75.9016	138.279	76.00817	135.1049	75.93622	136.1754	76.0682	138.6927
76.01463	109.9935	75.93538	133.6168	75.91827	138.2781	76.02483	135.1032	75.95288	136.1738	76.08487	138.6881
76.0313	109.9934	75.95205	133.6189	75.93493	138.2731	76.0415	135.1029	75.96955	136.1745	76.10153	138.6879

76.04797	109.9921	75.96872	133.615	75.9516	138.272	76.05817	135.1011	75.98622	136.1729	76.1182	138.6891
76.06463	109.9905	75.98538	133.6165	75.96827	138.2706	76.07483	135.0979	76.00288	136.1735	76.13487	138.6887
76.0813	109.9905	76.00205	133.6161	75.98493	138.2695	76.0915	135.0989	76.01955	136.1703	76.15153	138.6869
76.09797	109.9886	76.01872	133.6146	76.0016	138.2651	76.10817	135.0967	76.03622	136.1698	76.1682	138.6871
76.11463	109.9916	76.03538	133.6121	76.01827	138.2652	76.12483	135.0942	76.05288	136.168	76.18487	138.6853
76.1313	109.9885	76.05205	133.6115	76.03493	138.2626	76.1415	135.0936	76.06955	136.1676	76.20153	138.6854
76.14797	109.9814	76.06872	133.6097	76.0516	138.2618	76.15817	135.0928	76.08622	136.1672	76.2182	138.6818
76.16463	109.9825	76.08538	133.6089	76.06827	138.2599	76.17483	135.0912	76.10288	136.1686	76.23487	138.6831
76.1813	109.9806	76.10205	133.6087	76.08493	138.2528	76.1915	135.0891	76.11955	136.1666	76.25153	138.6807
76.19797	109.9836	76.11872	133.6073	76.1016	138.2529	76.20817	135.0887	76.13622	136.1656	76.2682	138.6841
76.21463	109.979	76.13538	133.6048	76.11827	138.2505	76.22483	135.0877	76.15288	136.164	76.28487	138.6817
76.2313	109.978	76.15205	133.6055	76.13493	138.2491	76.2415	135.0844	76.16955	136.1653	76.30153	138.6821
76.24797	109.9795	76.16872	133.6051	76.1516	138.2498	76.25817	135.0828	76.18622	136.1638	76.3182	138.678
76.26463	109.9756	76.18538	133.6049	76.16827	138.2421	76.27483	135.0817	76.20288	136.164	76.33487	138.6782
76.2813	109.9744	76.20205	133.6039	76.18493	138.2454	76.2915	135.0804	76.21955	136.1633	76.35153	138.6774
76.29797	109.975	76.21872	133.6043	76.2016	138.2402	76.30817	135.0807	76.23622	136.161	76.3682	138.6763
76.31463	109.9729	76.23538	133.6039	76.21827	138.2381	76.32483	135.0796	76.25288	136.1605	76.38487	138.6753
76.3313	109.9715	76.25205	133.6037	76.23493	138.2373	76.3415	135.0767	76.26955	136.1609	76.40153	138.6737
76.34797	109.9687	76.26872	133.6061	76.2516	138.231	76.35817	135.0746	76.28622	136.1585	76.4182	138.6725
76.36463	109.9693	76.28538	133.6063	76.26827	138.2339	76.37483	135.0743	76.30288	136.158	76.43487	138.6699
76.3813	109.9695	76.30205	133.6062	76.28493	138.2301	76.3915	135.0731	76.31955	136.1576	76.45153	138.6705
76.39797	109.9674	76.31872	133.6054	76.3016	138.228	76.40817	135.0692	76.33622	136.1564	76.4682	138.6697
76.41463	109.9656	76.33538	133.6047	76.31827	138.2254	76.42483	135.0714	76.35288	136.1551	76.48487	138.669
76.4313	109.9675	76.35205	133.6033	76.33493	138.2236	76.4415	135.0695	76.36955	136.1576	76.50153	138.6664
76.44797	109.9651	76.36872	133.6058	76.3516	138.2254	76.45817	135.0642	76.38622	136.1536	76.5182	138.6663
76.46463	109.9635	76.38538	133.6043	76.36827	138.2204	76.47483	135.0659	76.40288	136.1545	76.53487	138.6662
76.4813	109.9588	76.40205	133.6016	76.38493	138.2169	76.4915	135.0648	76.41955	136.1515	76.55153	138.6639
76.49797	109.9639	76.41872	133.6039	76.4016	138.2138	76.50817	135.0645	76.43622	136.1526	76.5682	138.6642
76.51463	109.9604	76.43538	133.6013	76.41827	138.2129	76.52483	135.064	76.45288	136.1508	76.58487	138.662
76.5313	109.9573	76.45205	133.6019	76.43493	138.2118	76.5415	135.062	76.46955	136.15	76.60153	138.6599
76.54797	109.9578	76.46872	133.603	76.4516	138.208	76.55817	135.0628	76.48622	136.1494	76.6182	138.6592
76.56463	109.9553	76.48538	133.6067	76.46827	138.2107	76.57483	135.0624	76.50288	136.149	76.63487	138.6589
76.5813	109.9543	76.50205	133.6008	76.48493	138.2008	76.5915	135.0623	76.51955	136.1496	76.65153	138.6578
76.59797	109.9492	76.51872	133.6012	76.5016	138.2013	76.60817	135.0587	76.53622	136.1453	76.6682	138.6562
76.61463	109.9525	76.53538	133.6025	76.51827	138.2011	76.62483	135.0578	76.55288	136.146	76.68487	138.6555
76.6313	109.9504	76.55205	133.6024	76.53493	138.1995	76.6415	135.0573	76.56955	136.1459	76.70153	138.6562
76.64797	109.9481	76.56872	133.6023	76.5516	138.1981	76.65817	135.0562	76.58622	136.144	76.7182	138.6526
76.66463	109.9444	76.58538	133.6028	76.56827	138.1955	76.67483	135.0562	76.60288	136.1443	76.73487	138.6559
76.6813	109.9422	76.60205	133.6047	76.58493	138.1938	76.6915	135.0548	76.61955	136.1437	76.75153	138.6536
76.69797	109.9413	76.61872	133.6023	76.6016	138.1902	76.70817	135.0524	76.63622	136.1434	76.7682	138.651
76.71463	109.9391	76.63538	133.602	76.61827	138.1914	76.72483	135.0521	76.65288	136.1416	76.78487	138.6512
76.7313	109.9346	76.65205	133.5995	76.63493	138.1903	76.7415	135.0513	76.66955	136.1413	76.80153	138.6525
76.74797	109.936	76.66872	133.6003	76.6516	138.1857	76.75817	135.0482	76.68622	136.141	76.8182	138.6499
76.76463	109.9367	76.68538	133.5996	76.66827	138.1848	76.77483	135.0473	76.70288	136.1416	76.83487	138.6506
76.7813	109.9315	76.70205	133.6012	76.68493	138.1822	76.7915	135.0462	76.71955	136.137	76.85153	138.6469
76.79797	109.9289	76.71872	133.6016	76.7016	138.1791	76.80817	135.045	76.73622	136.1381	76.8682	138.6475
76.81463	109.9284	76.73538	133.6028	76.71827	138.1757	76.82483	135.0443	76.75288	136.1372	76.88487	138.6454
76.8313	109.931	76.75205	133.6028	76.73493	138.1717	76.8415	135.0449	76.76955	136.1362	76.90153	138.6444
76.84797	109.9272	76.76872	133.5997	76.7516	138.1712	76.85817	135.0422	76.78622	136.1348	76.9182	138.6437
76.86463	109.928	76.78538	133.599	76.76827	138.1729	76.87483	135.0394	76.80288	136.136	76.93487	138.6444
76.8813	109.9226	76.80205	133.6016	76.78493	138.17	76.8915	135.0402	76.81955	136.1336	76.95153	138.6425
76.89797	109.9242	76.81872	133.6048	76.8016	138.1698	76.90817	135.0394	76.83622	136.1317	76.9682	138.6408
76.91463	109.9233	76.83538	133.6052	76.81827	138.165	76.92483	135.0359	76.85288	136.132	76.98487	138.641
76.9313	109.9227	76.85205	133.6022	76.83493	138.1636	76.9415	135.0346	76.86955	136.1318	77.00153	138.641
76.94797	109.9195	76.86872	133.6036	76.8516	138.1641	76.95817	135.0348	76.88622	136.1321	77.0182	138.6391
76.96463	109.9199	76.88538	133.6033	76.86827	138.1581	76.97483	135.0316	76.90288	136.1308	77.03487	138.6364
76.9813	109.9176	76.90205	133.6026	76.88493	138.156	76.9915	135.0308	76.91955	136.1306	77.05153	138.6358
76.99797	109.9182	76.91872	133.6015	76.9016	138.1544	77.00817	135.0283	76.93622	136.1298	77.0682	138.6368
77.01463	109.9157	76.93538	133.6016	76.91827	138.153	77.02483	135.0255	76.95288	136.1267	77.08487	138.6359
77.0313	109.9162	76.95205	133.6017	76.93493	138.1494	77.0415	135.0255	76.96955	136.1272	77.10153	138.6334
77.04797	109.9097	76.96872	133.6021	76.9516	138.1494	77.05817	135.0255	76.98622	136.127	77.1182	138.6318
77.06463	109.9102	76.98538	133.6052	76.96827	138.1441	77.07483	135.0238	77.00288	136.1275	77.13487	138.6311
77.0813	109.908	77.00205	133.603	76.98493	138.1454	77.0915	135.024	77.01955	136.1237	77.15153	138.6314
77.09797	109.9085	77.01872	133.6068	77.0016	138.1435	77.10817	135.0201	77.03622	136.1242	77.1682	138.6309
77.11463	109.9066	77.03538	133.6049	77.01827	138.1403	77.12483	135.0189	77.05288	136.1241	77.18487	138.6304
77.1313	109.9079	77.05205	133.6069	77.03493	138.1388	77.1415	135.0177	77.06955	136.1255	77.20153	138.6304
77.14797	109.9048	77.06872	133.6041	77.0516	138.1363	77.15817	135.0186	77.08622	136.1227	77.2182	138.6284
77.16463	109.9055	77.08538	133.6044	77.06827	138.134	77.17483	135.0161	77.10288	136.1218	77.23487	138.6288
77.1813	109.901	77.10205	133.6066	77.08493	138.1304	77.1915	135.0151	77.11955	136.1198	77.25153	138.6274
77.19797	109.9007	77.11872	133.6064	77.1016	138.1288	77.20817	135.0142	77.13622	136.1215	77.2682	138.6257
77.21463	109.8984	77.13538	133.6059	77.11827	138.1256	77.22483	135.0128	77.15288	136.1191	77.28487	138.6254
77.2313	109.8975	77.15205	133.6044	77.13493	138.1253	77.2415	135.0107	77.16955	136.1185	77.30153	138.6263
77.24797	109.8965	77.16872	133.606	77.1516	138.1229	77.25817	135.0078	77.18622	136.1164	77.3182	138.6238
77.26463	109.8926	77.18538	133.6039	77.16827	138.1229	77.27483	135.0076	77.20288	136.1176	77.33487	138.6212
77.2813	109.8924	77.20205	133.6042	77.18493	138.1179	77.2915	135.0079	77.21955	136.1159	77.35153	138.6222
77.29797	109.89	77.21872	133.6052	77.2016	138.1162	77.30817	135.0067	77.23622	136.1137	77.3682	138.6195
77.31463	109.8894	77.23538	133.6027	77.21827	138.1173	77.32483	135.0077	77.25288	136.1137	77.38487	138.6187
77.3313	109.8886	77.25205	133.6028	77.23493	138.1108	77.3415	135.0019	77.26955	136.1122	77.40153	138.618
77.34797	109.8848	77.26872	133.6037	77.2516	138.1091	77.35817	135.0007	77.28622	136.11	77.4182	138.6163
77.36463	109.8856	77.28538	133.6026	77.26827	138.1074	77.37483	135.0011	77.30288	136.1106	77.43487	138.6141
77.3813	109.8804	77.30205	133.6028	77.28493	138.1102	77.3915	134.9973	77.31955	136.1088	77.45153	138.6139
77.39797	109.8806	77.31872	133.6033	77.3016	138.1083	77.40817	134.997	77.33622	136.1075	77.4682	138.6149
77.41463	109.8792	77.33538	133.6024	77.31827	138.1058	77.42483	134.9958	77.35288	136.1053	77.48487	138.61
77.4313	109.8794	77.35205	133.6	77.33493	138.1056	77.4415	134.9931	77.36955	136.1058	77.50153	138.611
77.44797	109.8772	77.36872	133.6009	77.3516	138.1049	77.45817	134.9922	77.38622	136.1043	77.5182	138.6122
77.46463	109.8742	77.38538	133.6026	77.36827	138.1016	77.47483	134.9909	77.40288	136.1043	77.53487	138.6098
77.4813	109.873	77.40205	133.6021	77.38493	138.1031	77.4915	134.9892	77.41955	136.1011	77.55153	138.6071
77.49797	109.8722	77.41872	133.6026	77.4016	138.0954	77.50817	134.9864	77.43622	136.1033	77.5682	138.6082
77.51463	109.8696	77.43538	133.6022	77.41827	138.0983	77.52483	134.9863	77.45288	136.1019	77.58487	138.6032
77.5313	109.87	77.45205	133.6026	77.43493	138.0956	77.5415	134.9851	77.46955	136.101	77.60153	138.6067
77.54797	109.8688	77.46872	133.6052	77.4516	138.0923	77.55817	134.9855	77.48622	136.0988	77.6182	138.6047
77.56463	109.8672	77.48538	133.6038	77.46827	138.093	77.57483	134.9839	77.50288	136.098	77.63487	138.6028
77.5813	109.8668	77.50205	133.604	77.48493	138.0887	77.5915	134.9799	77.51955	136.0975	77.65153	138.6019
77.59797	109.865	77.51872	133.6014	77.5016	138.0873	77.60817	134.9777	77.53622	136.0971	77.6682	138.6028
77.61463	109.8631	77.53538	133.6051	77.51827	138.0879	77.62483	134.9793	77.55288	136.0984	77.68487	138.5993
77.6313	109.8593	77.55205	133.6062	77.53493	138.0835	77.6415	134.9748	77.56955	136.0987	77.70153	138.5997
77.64797	109.8585	77.56872	133.6071	77.5516	138.0782	77.65817	134.9735	77.58622	136.0966	77.7182	138.597

77.66463	109.8582	77.58538	133.6061	77.56827	138.0772	77.67483	134.9752	77.60288	136.0939	77.73487	138.5958
77.6813	109.8563	77.60205	133.6053	77.58493	138.0798	77.6915	134.9738	77.61955	136.0942	77.75153	138.5952
77.69797	109.8547	77.61872	133.6071	77.6016	138.0758	77.70817	134.9713	77.63622	136.0944	77.7682	138.5948
77.71463	109.8558	77.63538	133.6078	77.61827	138.0711	77.72483	134.9706	77.65288	136.0913	77.78487	138.593
77.7313	109.853	77.65205	133.6113	77.63493	138.0711	77.7415	134.9707	77.66955	136.0906	77.80153	138.591
77.74797	109.8523	77.66872	133.6092	77.6516	138.0687	77.75817	134.9674	77.68622	136.0915	77.8182	138.5902
77.76463	109.8493	77.68538	133.6097	77.66827	138.0637	77.77483	134.9668	77.70288	136.089	77.83487	138.5917
77.7813	109.8501	77.70205	133.6079	77.68493	138.0627	77.7915	134.9633	77.71955	136.0875	77.85153	138.5859
77.79797	109.8468	77.71872	133.609	77.7016	138.0611	77.80817	134.9634	77.73622	136.0859	77.8682	138.5872
77.81463	109.8478	77.73538	133.6073	77.71827	138.0603	77.82483	134.9613	77.75288	136.0873	77.88487	138.585
77.8313	109.8455	77.75205	133.6093	77.73493	138.0582	77.8415	134.9612	77.76955	136.0853	77.90153	138.5851
77.84797	109.841	77.76872	133.6104	77.7516	138.0577	77.85817	134.9587	77.78622	136.0854	77.9182	138.5853
77.86463	109.8418	77.78538	133.6131	77.76827	138.0528	77.87483	134.9602	77.80288	136.0842	77.93487	138.5837
77.8813	109.8394	77.80205	133.6093	77.78493	138.0526	77.8915	134.9579	77.81955	136.0838	77.95153	138.5816
77.89797	109.8374	77.81872	133.6112	77.8016	138.0487	77.90817	134.9558	77.83622	136.0855	77.9682	138.5808
77.91463	109.8396	77.83538	133.6102	77.81827	138.0462	77.92483	134.9557	77.85288	136.0804	77.98487	138.5815
77.9313	109.8352	77.85205	133.6109	77.83493	138.0471	77.9415	134.9534	77.86955	136.0815	78.00153	138.5804
77.94797	109.8352	77.86872	133.6107	77.8516	138.0436	77.95817	134.9504	77.88622	136.0772	78.0182	138.5788
77.96463	109.8343	77.88538	133.6108	77.86827	138.0405	77.97483	134.9519	77.90288	136.0813	78.03487	138.5784
77.9813	109.8308	77.90205	133.6107	77.88493	138.0394	77.9915	134.9481	77.91955	136.0779	78.05153	138.5759
77.99797	109.8288	77.91872	133.6124	77.9016	138.035	78.00817	134.9465	77.93622	136.078	78.0682	138.5774
78.01463	109.8281	77.93538	133.6115	77.91827	138.0332	78.02483	134.9491	77.95288	136.0753	78.08487	138.5753
78.0313	109.8246	77.95205	133.6146	77.93493	138.0326	78.0415	134.9432	77.96955	136.0757	78.10153	138.574
78.04797	109.825	77.96872	133.6126	77.9516	138.0281	78.05817	134.9441	77.98622	136.0774	78.1182	138.5727
78.06463	109.8245	77.98538	133.6101	77.96827	138.0247	78.07483	134.9431	78.00288	136.0733	78.13487	138.5724
78.0813	109.8221	78.00205	133.6108	77.98493	138.0254	78.0915	134.9406	78.01955	136.0715	78.15153	138.5704
78.09797	109.8234	78.01872	133.6139	78.0016	138.0225	78.10817	134.9414	78.03622	136.0714	78.1682	138.5717
78.11463	109.8211	78.03538	133.6105	78.01827	138.0213	78.12483	134.9393	78.05288	136.0733	78.18487	138.5693
78.1313	109.8172	78.05205	133.6119	78.03493	138.0191	78.1415	134.9358	78.06955	136.0728	78.20153	138.568
78.14797	109.8153	78.06872	133.6127	78.0516	138.0153	78.15817	134.9362	78.08622	136.0709	78.2182	138.5678
78.16463	109.8157	78.08538	133.6123	78.06827	138.012	78.17483	134.9345	78.10288	136.0687	78.23487	138.5677
78.1813	109.8147	78.10205	133.6073	78.08493	138.0125	78.1915	134.9332	78.11955	136.0687	78.25153	138.5649
78.19797	109.8124	78.11872	133.6104	78.1016	138.0098	78.20817	134.9309	78.13622	136.0694	78.2682	138.5655
78.21463	109.8125	78.13538	133.6086	78.11827	138.0068	78.22483	134.9308	78.15288	136.0657	78.28487	138.5651
78.2313	109.8109	78.15205	133.6086	78.13493	138.0058	78.2415	134.9274	78.16955	136.0647	78.30153	138.5642
78.24797	109.8067	78.16872	133.6095	78.1516	138.0053	78.25817	134.9278	78.18622	136.0651	78.3182	138.5624
78.26463	109.8054	78.18538	133.6092	78.16827	138.002	78.27483	134.9274	78.20288	136.0665	78.33487	138.5644
78.2813	109.804	78.20205	133.6083	78.18493	138.0001	78.2915	134.9258	78.21955	136.066	78.35153	138.5641
78.29797	109.8068	78.21872	133.6059	78.2016	138.0017	78.30817	134.9235	78.23622	136.0628	78.3682	138.561
78.31463	109.8018	78.23538	133.6086	78.21827	137.9994	78.32483	134.9237	78.25288	136.063	78.38487	138.5624
78.3313	109.7994	78.25205	133.6053	78.23493	137.9951	78.3415	134.9213	78.26955	136.0604	78.40153	138.5637
78.34797	109.7991	78.26872	133.6048	78.2516	137.992	78.35817	134.9198	78.28622	136.0612	78.4182	138.5607
78.36463	109.7965	78.28538	133.6062	78.26827	137.9906	78.37483	134.9187	78.30288	136.0572	78.43487	138.561
78.3813	109.7978	78.30205	133.6061	78.28493	137.9898	78.3915	134.9177	78.31955	136.0585	78.45153	138.5591
78.39797	109.7965	78.31872	133.6061	78.3016	137.9888	78.40817	134.9156	78.33622	136.0563	78.4682	138.558
78.41463	109.7924	78.33538	133.6048	78.31827	137.984	78.42483	134.9155	78.35288	136.0577	78.48487	138.5577
78.4313	109.7908	78.35205	133.6031	78.33493	137.9832	78.4415	134.9144	78.36955	136.0554	78.50153	138.5572
78.44797	109.7881	78.36872	133.6015	78.3516	137.9821	78.45817	134.9114	78.38622	136.0533	78.5182	138.5556
78.46463	109.7874	78.38538	133.6027	78.36827	137.979	78.47483	134.91	78.40288	136.0534	78.53487	138.556
78.4813	109.7879	78.40205	133.6023	78.38493	137.9764	78.4915	134.9085	78.41955	136.0514	78.55153	138.5554
78.49797	109.7838	78.41872	133.6015	78.4016	137.9737	78.50817	134.9067	78.43622	136.0515	78.5682	138.5541
78.51463	109.7839	78.43538	133.5977	78.41827	137.9726	78.52483	134.9054	78.45288	136.0501	78.58487	138.5536
78.5313	109.7831	78.45205	133.598	78.43493	137.9691	78.5415	134.9064	78.46955	136.0501	78.60153	138.5506
78.54797	109.7797	78.46872	133.6017	78.4516	137.969	78.55817	134.9057	78.48622	136.0459	78.6182	138.5499
78.56463	109.777	78.48538	133.601	78.46827	137.9645	78.57483	134.9018	78.50288	136.0481	78.63487	138.5501
78.5813	109.7797	78.50205	133.6004	78.48493	137.9647	78.5915	134.9014	78.51955	136.0477	78.65153	138.5488
78.59797	109.7765	78.51872	133.5988	78.5016	137.962	78.60817	134.8995	78.53622	136.0491	78.6682	138.5504
78.61463	109.7777	78.53538	133.6021	78.51827	137.9622	78.62483	134.8981	78.55288	136.0458	78.68487	138.5486
78.6313	109.7728	78.55205	133.6002	78.53493	137.9603	78.6415	134.8962	78.56955	136.048	78.70153	138.5491
78.64797	109.7702	78.56872	133.5987	78.5516	137.9573	78.65817	134.8957	78.58622	136.0452	78.7182	138.5473
78.66463	109.7719	78.58538	133.6018	78.56827	137.9558	78.67483	134.8952	78.60288	136.044	78.73487	138.547
78.6813	109.7696	78.60205	133.5959	78.58493	137.9538	78.6915	134.8921	78.61955	136.0451	78.75153	138.5468
78.69797	109.7667	78.61872	133.6017	78.6016	137.956	78.70817	134.89	78.63622	136.0427	78.7682	138.5462
78.71463	109.7671	78.63538	133.6029	78.61827	137.9526	78.72483	134.8883	78.65288	136.0424	78.78487	138.5455
78.7313	109.7672	78.65205	133.6007	78.63493	137.9491	78.7415	134.8893	78.66955	136.0428	78.80153	138.5455
78.74797	109.7656	78.66872	133.6008	78.6516	137.9502	78.75817	134.8885	78.68622	136.0417	78.8182	138.5442
78.76463	109.7628	78.68538	133.6013	78.66827	137.9441	78.77483	134.886	78.70288	136.0403	78.83487	138.544
78.7813	109.7639	78.70205	133.5977	78.68493	137.9435	78.7915	134.8855	78.71955	136.0392	78.85153	138.5428
78.79797	109.7584	78.71872	133.5991	78.7016	137.94	78.80817	134.882	78.73622	136.0379	78.8682	138.5454
78.81463	109.7618	78.73538	133.6004	78.71827	137.9429	78.82483	134.8822	78.75288	136.0389	78.88487	138.5406
78.8313	109.7585	78.75205	133.6018	78.73493	137.9371	78.8415	134.8778	78.76955	136.0395	78.90153	138.5425
78.84797	109.757	78.76872	133.6018	78.7516	137.9378	78.85817	134.8785	78.78622	136.0373	78.9182	138.5407
78.86463	109.7563	78.78538	133.6012	78.76827	137.9306	78.87483	134.8766	78.80288	136.0372	78.93487	138.5401
78.8813	109.7537	78.80205	133.6013	78.78493	137.9317	78.8915	134.8772	78.81955	136.0341	78.95153	138.5393
78.89797	109.7524	78.81872	133.6007	78.8016	137.9283	78.90817	134.8746	78.83622	136.0369	78.9682	138.5399
78.91463	109.7537	78.83538	133.6024	78.81827	137.9244	78.92483	134.8755	78.85288	136.0359	78.98487	138.5381
78.9313	109.7515	78.85205	133.6043	78.83493	137.9214	78.9415	134.8732	78.86955	136.0346	79.00153	138.5401
78.94797	109.7446	78.86872	133.6016	78.8516	137.9224	78.95817	134.8711	78.88622	136.0351	79.0182	138.5395
78.96463	109.7509	78.88538	133.6032	78.86827	137.9175	78.97483	134.8704	78.90288	136.0334	79.03487	138.5381
78.9813	109.7451	78.90205	133.602	78.88493	137.9173	78.9915	134.8684	78.91955	136.0295	79.05153	138.5387
78.99797	109.7423	78.91872	133.6025	78.9016	137.9142	79.00817	134.8677	78.93622	136.0299	79.0682	138.5391
79.01463	109.7412	78.93538	133.6035	78.91827	137.912	79.02483	134.8636	78.95288	136.0301	79.08487	138.5343
79.0313	109.7424	78.95205	133.6064	78.93493	137.9111	79.0415	134.8628	78.96955	136.0305	79.10153	138.5386
79.04797	109.7387	78.96872	133.6036	78.9516	137.9081	79.05817	134.8617	78.98622	136.0294	79.1182	138.5351
79.06463	109.7375	78.98538	133.6047	78.96827	137.9056	79.07483	134.8627	79.00288	136.0268	79.13487	138.5341
79.0813	109.7377	79.00205	133.6051	78.98493	137.9068	79.0915	134.8602	79.01955	136.0262	79.15153	138.5326
79.09797	109.7351	79.01872	133.6037	79.0016	137.9019	79.10817	134.858	79.03622	136.0266	79.1682	138.5324
79.11463	109.7339	79.03538	133.6055	79.01827	137.9	79.12483	134.8575	79.05288	136.027	79.18487	138.5323
79.1313	109.7337	79.05205	133.6085	79.03493	137.9002	79.1415	134.8563	79.06955	136.025	79.20153	138.5339
79.14797	109.7291	79.06872	133.6081	79.0516	137.8979	79.15817	134.8514	79.08622	136.0242	79.2182	138.5319
79.16463	109.7324	79.08538	133.6082	79.06827	137.8987	79.17483	134.8518	79.10288	136.023	79.23487	138.5304
79.1813	109.7288	79.10205	133.6081	79.08493	137.8962	79.1915	134.8497	79.11955	136.0236	79.25153	138.5295
79.19797	109.7278	79.11872	133.6068	79.1016	137.8936	79.20817	134.8491	79.13622	136.024	79.2682	138.5306
79.21463	109.7264	79.13538	133.6078	79.11827	137.8907	79.22483	134.8482	79.15288	136.0217	79.28487	138.5276
79.2313	109.7256	79.15205	133.6095	79.13493	137.8921	79.2415	134.8466	79.16955	136.0183	79.30153	138.5267
79.24797	109.7245	79.16872	133.6076	79.1516	137.8862	79.25817	134.8469	79.18622	136.0175	79.3182	138.5278
79.26463	109.7208	79.18538	133.6111	79.16827	137.8839	79.27483	134.8471	79.20288	136.0167	79.33487	138.5265

79.2813	109.7222	79.20205	133.6103	79.18493	137.8841	79.2915	134.8467	79.21955	136.0174	79.35153	138.524
79.29797	109.7201	79.21872	133.6081	79.2016	137.8836	79.30817	134.843	79.23622	136.0159	79.3682	138.5238
79.31463	109.7164	79.23538	133.6081	79.21827	137.8801	79.32483	134.8437	79.25288	136.0152	79.38487	138.5204
79.3313	109.7173	79.25205	133.6107	79.23493	137.8756	79.3415	134.8429	79.26955	136.0155	79.40153	138.5217
79.34797	109.7164	79.26872	133.6079	79.2516	137.8774	79.35817	134.8421	79.28622	136.0142	79.4182	138.5211
79.36463	109.711	79.28538	133.6104	79.26827	137.8742	79.37483	134.838	79.30288	136.0144	79.43487	138.5205
79.3813	109.7116	79.30205	133.6068	79.28493	137.8722	79.3915	134.838	79.31955	136.0129	79.45153	138.5199
79.39797	109.7092	79.31872	133.6059	79.3016	137.8699	79.40817	134.8379	79.33622	136.011	79.4682	138.5189
79.41463	109.7078	79.33538	133.6088	79.31827	137.8681	79.42483	134.8358	79.35288	136.011	79.48487	138.5194
79.4313	109.7073	79.35205	133.6082	79.33493	137.8634	79.4415	134.837	79.36955	136.0093	79.50153	138.5176
79.44797	109.7059	79.36872	133.6111	79.3516	137.8635	79.45817	134.8353	79.38622	136.0096	79.5182	138.5164
79.46463	109.7057	79.38538	133.6113	79.36827	137.8621	79.47483	134.8323	79.40288	136.0103	79.53487	138.5158
79.4813	109.7045	79.40205	133.6077	79.38493	137.8605	79.4915	134.8352	79.41955	136.0087	79.55153	138.5132
79.49797	109.7026	79.41872	133.6107	79.4016	137.8578	79.50817	134.8331	79.43622	136.0073	79.5682	138.5138
79.51463	109.7008	79.43538	133.6096	79.41827	137.8565	79.52483	134.8297	79.45288	136.0066	79.58487	138.5127
79.5313	109.6989	79.45205	133.6114	79.43493	137.8527	79.5415	134.8298	79.46955	136.0044	79.60153	138.5134
79.54797	109.6988	79.46872	133.6115	79.4516	137.8514	79.55817	134.8303	79.48622	136.0052	79.6182	138.5116
79.56463	109.6972	79.48538	133.6105	79.46827	137.8502	79.57483	134.8288	79.50288	136.0057	79.63487	138.5122
79.5813	109.6984	79.50205	133.6117	79.48493	137.8474	79.5915	134.8268	79.51955	135.9994	79.65153	138.5096
79.59797	109.6946	79.51872	133.6106	79.5016	137.8431	79.60817	134.8267	79.53622	136.0037	79.6682	138.5104
79.61463	109.693	79.53538	133.6078	79.51827	137.8457	79.62483	134.8259	79.55288	136.0027	79.68487	138.5091
79.6313	109.6914	79.55205	133.6098	79.53493	137.8401	79.6415	134.8219	79.56955	136.0003	79.70153	138.5088
79.64797	109.6913	79.56872	133.6107	79.5516	137.837	79.65817	134.8205	79.58622	136.0024	79.7182	138.5065
79.66463	109.6888	79.58538	133.6134	79.56827	137.834	79.67483	134.8187	79.60288	136.0022	79.73487	138.5073
79.6813	109.6882	79.60205	133.6084	79.58493	137.8375	79.6915	134.8197	79.61955	135.9951	79.75153	138.5081
79.69797	109.6851	79.61872	133.6099	79.6016	137.8322	79.70817	134.8188	79.63622	135.9965	79.7682	138.5072
79.71463	109.6839	79.63538	133.6085	79.61827	137.8329	79.72483	134.8168	79.65288	135.9988	79.78487	138.506
79.7313	109.683	79.65205	133.6107	79.63493	137.8268	79.7415	134.8135	79.66955	135.9956	79.80153	138.5064
79.74797	109.6821	79.66872	133.6076	79.6516	137.8254	79.75817	134.8139	79.68622	135.9974	79.8182	138.5041
79.76463	109.6783	79.68538	133.6082	79.66827	137.8247	79.77483	134.8141	79.70288	135.9954	79.83487	138.5055
79.7813	109.6779	79.70205	133.6093	79.68493	137.8226	79.7915	134.812	79.71955	135.9935	79.85153	138.503
79.79797	109.6779	79.71872	133.6121	79.7016	137.8162	79.80817	134.8085	79.73622	135.9952	79.8682	138.5037
79.81463	109.677	79.73538	133.611	79.71827	137.8169	79.82483	134.8104	79.75288	135.9928	79.88487	138.5029
79.8313	109.6738	79.75205	133.6122	79.73493	137.8143	79.8415	134.8074	79.76955	135.9933	79.90153	138.502
79.84797	109.6741	79.76872	133.6104	79.7516	137.8151	79.85817	134.8082	79.78622	135.9931	79.9182	138.5021
79.86463	109.6703	79.78538	133.6094	79.76827	137.8092	79.87483	134.8046	79.80288	135.9927	79.93487	138.5023
79.8813	109.6659	79.80205	133.6095	79.78493	137.8095	79.8915	134.8024	79.81955	135.9905	79.95153	138.4994
79.89797	109.6681	79.81872	133.6108	79.8016	137.8072	79.90817	134.8035	79.83622	135.9897	79.9682	138.499
79.91463	109.665	79.83538	133.61	79.81827	137.8051	79.92483	134.8025	79.85288	135.9879	79.98487	138.499
79.9313	109.6643	79.85205	133.6101	79.83493	137.8014	79.9415	134.7993	79.86955	135.9885	80.00153	138.4972
79.94797	109.6657	79.86872	133.6089	79.8516	137.8017	79.95817	134.799	79.88622	135.9852	80.0182	138.4942
79.96463	109.6627	79.88538	133.6082	79.86827	137.8003	79.97483	134.8014	79.90288	135.9856	80.03487	138.4957
79.9813	109.6583	79.90205	133.6095	79.88493	137.7988	79.9915	134.7983	79.91955	135.9861	80.05153	138.4968
79.99797	109.6584	79.91872	133.6079	79.9016	137.7929	80.00817	134.7942	79.93622	135.9828	80.0682	138.4961
80.01463	109.6583	79.93538	133.6099	79.91827	137.7948	80.02483	134.7956	79.95288	135.9818	80.08487	138.4945
80.0313	109.6555	79.95205	133.6067	79.93493	137.7945	80.0415	134.7962	79.96955	135.9832	80.10153	138.496
80.04797	109.657	79.96872	133.6086	79.9516	137.7919	80.05817	134.7934	79.98622	135.9824	80.1182	138.4926
80.06463	109.6533	79.98538	133.6087	79.96827	137.7872	80.07483	134.7934	80.00288	135.9811	80.13487	138.4925
80.0813	109.6537	80.00205	133.6065	79.98493	137.7872	80.0915	134.7927	80.01955	135.9801	80.15153	138.4934
80.09797	109.65	80.01872	133.6072	80.0016	137.7843	80.10817	134.7901	80.03622	135.9801	80.1682	138.4931
80.11463	109.6477	80.03538	133.6062	80.01827	137.7823	80.12483	134.792	80.05288	135.9782	80.18487	138.4936
80.1313	109.6482	80.05205	133.6084	80.03493	137.7825	80.1415	134.7876	80.06955	135.9769	80.20153	138.4904
80.14797	109.6475	80.06872	133.6089	80.0516	137.779	80.15817	134.7886	80.08622	135.9781	80.2182	138.4897
80.16463	109.6455	80.08538	133.6077	80.06827	137.7774	80.17483	134.7867	80.10288	135.978	80.23487	138.4908
80.1813	109.6448	80.10205	133.6065	80.08493	137.7749	80.1915	134.7879	80.11955	135.9778	80.25153	138.4913
80.19797	109.6421	80.11872	133.6099	80.1016	137.7728	80.20817	134.7874	80.13622	135.9734	80.2682	138.4907
80.21463	109.64	80.13538	133.6081	80.11827	137.772	80.22483	134.7846	80.15288	135.9725	80.28487	138.4886
80.2313	109.6382	80.15205	133.6076	80.13493	137.7657	80.2415	134.7836	80.16955	135.9754	80.30153	138.4904
80.24797	109.6414	80.16872	133.6058	80.1516	137.7663	80.25817	134.7858	80.18622	135.9742	80.3182	138.4865
80.26463	109.6362	80.18538	133.6062	80.16827	137.7613	80.27483	134.7837	80.20288	135.9713	80.33487	138.4888
80.2813	109.636	80.20205	133.6057	80.18493	137.7614	80.2915	134.7816	80.21955	135.9719	80.35153	138.4872
80.29797	109.6368	80.21872	133.6061	80.2016	137.7588	80.30817	134.783	80.23622	135.9699	80.3682	138.4888
80.31463	109.6319	80.23538	133.6057	80.21827	137.7575	80.32483	134.7819	80.25288	135.9723	80.38487	138.4871
80.3313	109.631	80.25205	133.6066	80.23493	137.7571	80.3415	134.7779	80.26955	135.9706	80.40153	138.4854
80.34797	109.6284	80.26872	133.6072	80.2516	137.7571	80.35817	134.7802	80.28622	135.9687	80.4182	138.4864
80.36463	109.6289	80.28538	133.6085	80.26827	137.7539	80.37483	134.7761	80.30288	135.9697	80.43487	138.4877
80.3813	109.6272	80.30205	133.6056	80.28493	137.754	80.3915	134.7756	80.31955	135.9681	80.45153	138.4881
80.39797	109.6232	80.31872	133.6065	80.3016	137.7495	80.40817	134.7777	80.33622	135.967	80.4682	138.4863
80.41463	109.6282	80.33538	133.6064	80.31827	137.7518	80.42483	134.7745	80.35288	135.967	80.48487	138.4859
80.4313	109.622	80.35205	133.6073	80.33493	137.7496	80.4415	134.7734	80.36955	135.9669	80.50153	138.4849
80.44797	109.6203	80.36872	133.6066	80.3516	137.7467	80.45817	134.7742	80.38622	135.9656	80.5182	138.4877
80.46463	109.6199	80.38538	133.6064	80.36827	137.7437	80.47483	134.7736	80.40288	135.963	80.53487	138.4866
80.4813	109.6197	80.40205	133.606	80.38493	137.7414	80.4915	134.7725	80.41955	135.9662	80.55153	138.4853
80.49797	109.6171	80.41872	133.605	80.4016	137.7389	80.50817	134.7661	80.43622	135.9639	80.5682	138.4871
80.51463	109.6151	80.43538	133.6085	80.41827	137.7391	80.52483	134.7696	80.45288	135.9633	80.58487	138.484
80.5313	109.6161	80.45205	133.6064	80.43493	137.7375	80.5415	134.7691	80.46955	135.9616	80.60153	138.4855
80.54797	109.6147	80.46872	133.6063	80.4516	137.733	80.55817	134.7644	80.48622	135.9625	80.6182	138.4872
80.56463	109.6125	80.48538	133.6067	80.46827	137.7299	80.57483	134.7656	80.50288	135.9593	80.63487	138.4853
80.5813	109.6099	80.50205	133.6061	80.48493	137.7291	80.5915	134.7651	80.51955	135.9613	80.65153	138.4805
80.59797	109.61	80.51872	133.6076	80.5016	137.7273	80.60817	134.7633	80.53622	135.9605	80.6682	138.4856
80.61463	109.6096	80.53538	133.608	80.51827	137.725	80.62483	134.7641	80.55288	135.9562	80.68487	138.4823
80.6313	109.6064	80.55205	133.61	80.53493	137.7229	80.6415	134.7614	80.56955	135.9566	80.70153	138.483
80.64797	109.6055	80.56872	133.6079	80.5516	137.7204	80.65817	134.7576	80.58622	135.9572	80.7182	138.4834
80.66463	109.6066	80.58538	133.6063	80.56827	137.7172	80.67483	134.76	80.60288	135.9537	80.73487	138.4834
80.6813	109.6005	80.60205	133.6085	80.58493	137.7145	80.6915	134.7587	80.61955	135.9572	80.75153	138.4807
80.69797	109.6007	80.61872	133.6093	80.6016	137.7138	80.70817	134.7576	80.63622	135.9538	80.7682	138.4816
80.71463	109.6014	80.63538	133.6064	80.61827	137.7127	80.72483	134.76	80.65288	135.9544	80.78487	138.4825
80.7313	109.5997	80.65205	133.6067	80.63493	137.7088	80.7415	134.7545	80.66955	135.955	80.80153	138.4815
80.74797	109.598	80.66872	133.6127	80.6516	137.7061	80.75817	134.7513	80.68622	135.9555	80.8182	138.4827
80.76463	109.5971	80.68538	133.6106	80.66827	137.7047	80.77483	134.7515	80.70288	135.9522	80.83487	138.4806
80.7813	109.5962	80.70205	133.6016	80.68493	137.703	80.7915	134.7531	80.71955	135.9529	80.85153	138.4795
80.79797	109.5951	80.71872	133.6058	80.7016	137.7031	80.80817	134.7515	80.73622	135.9516	80.8682	138.4792
80.81463	109.5914	80.73538	133.616	80.71827	137.6979	80.82483	134.7512	80.75288	135.9512	80.88487	138.4785
80.8313	109.5908	80.75205	133.6096	80.73493	137.6975	80.8415	134.7487	80.76955	135.949	80.90153	138.479
80.84797	109.5898	80.76872	133.604	80.7516	137.695	80.85817	134.748	80.78622	135.9484	80.9182	138.4768
80.86463	109.5872	80.78538	133.6094	80.76827	137.6934	80.87483	134.7448	80.80288	135.9477	80.93487	138.4751
80.8813	109.5868	80.80205	133.6089	80.78493	137.6908	80.8915	134.7462	80.81955	135.9442	80.95153	138.4763

80.89797	109.5857	80.81872	133.6093	80.8016	137.6903	80.90817	134.744	80.83622	135.9444	80.9682	138.4753
80.91463	109.5905	80.83538	133.6057	80.81827	137.6908	80.92483	134.7462	80.85288	135.9458	80.98487	138.4746
80.9313	109.5836	80.85205	133.61	80.83493	137.6849	80.9415	134.7434	80.86955	135.941	81.00153	138.4738
80.94797	109.5825	80.86872	133.6055	80.8516	137.6852	80.95817	134.7422	80.88622	135.9438	81.0182	138.474
80.96463	109.5793	80.88538	133.6127	80.86827	137.6797	80.97483	134.7403	80.90288	135.9429	81.03487	138.472
80.9813	109.5839	80.90205	133.6104	80.88493	137.6793	80.9915	134.7403	80.91955	135.9432	81.05153	138.4713
80.99797	109.5755	80.91872	133.6061	80.9016	137.6764	81.00817	134.74	80.93622	135.9412	81.0682	138.4738
81.01463	109.5772	80.93538	133.6093	80.91827	137.6719	81.02483	134.7384	80.95288	135.9412	81.08487	138.4692
81.0313	109.5772	80.95205	133.6074	80.93493	137.6708	81.0415	134.7375	80.96955	135.939	81.10153	138.4699
81.04797	109.5761	80.96872	133.6064	80.9516	137.6716	81.05817	134.7367	80.98622	135.9412	81.1182	138.4689
81.06463	109.5745	80.98538	133.605	80.96827	137.6688	81.07483	134.7324	81.00288	135.9389	81.13487	138.4689
81.0813	109.5712	81.00205	133.6089	80.98493	137.6653	81.0915	134.7347	81.01955	135.9363	81.15153	138.4646
81.09797	109.57	81.01872	133.6081	81.0016	137.663	81.10817	134.7325	81.03622	135.9389	81.1682	138.4674
81.11463	109.569	81.03538	133.6074	81.01827	137.6621	81.12483	134.7308	81.05288	135.9372	81.18487	138.4655
81.1313	109.5661	81.05205	133.6067	81.03493	137.6608	81.1415	134.7319	81.06955	135.9363	81.20153	138.4649
81.14797	109.5667	81.06872	133.611	81.0516	137.6619	81.15817	134.73	81.08622	135.9367	81.2182	138.4637
81.16463	109.5658	81.08538	133.6078	81.06827	137.6565	81.17483	134.7287	81.10288	135.9366	81.23487	138.4624
81.1813	109.5664	81.10205	133.6097	81.08493	137.658	81.1915	134.732	81.11955	135.9346	81.25153	138.4632
81.19797	109.5625	81.11872	133.6031	81.1016	137.6547	81.20817	134.729	81.13622	135.933	81.2682	138.463
81.21463	109.5612	81.13538	133.6091	81.11827	137.6528	81.22483	134.7251	81.15288	135.9352	81.28487	138.4623
81.2313	109.5605	81.15205	133.6079	81.13493	137.6507	81.2415	134.7257	81.16955	135.9321	81.30153	138.4635
81.24797	109.5556	81.16872	133.6066	81.1516	137.6493	81.25817	134.7238	81.18622	135.9338	81.3182	138.4603
81.26463	109.5583	81.18538	133.608	81.16827	137.6467	81.27483	134.7255	81.20288	135.931	81.33487	138.4611
81.2813	109.5582	81.20205	133.6086	81.18493	137.6462	81.2915	134.7226	81.21955	135.9323	81.35153	138.4609
81.29797	109.5549	81.21872	133.6077	81.2016	137.6433	81.30817	134.722	81.23622	135.9315	81.3682	138.4586
81.31463	109.5543	81.23538	133.6041	81.21827	137.6403	81.32483	134.7243	81.25288	135.9306	81.38487	138.4589
81.3313	109.5509	81.25205	133.606	81.23493	137.6379	81.3415	134.7195	81.26955	135.9276	81.40153	138.4602
81.34797	109.5511	81.26872	133.6076	81.2516	137.6389	81.35817	134.7229	81.28622	135.9273	81.4182	138.4581
81.36463	109.5484	81.28538	133.6049	81.26827	137.6355	81.37483	134.7232	81.30288	135.9291	81.43487	138.4597
81.3813	109.548	81.30205	133.6058	81.28493	137.6344	81.3915	134.7203	81.31955	135.9279	81.45153	138.4613
81.39797	109.5464	81.31872	133.6022	81.3016	137.6314	81.40817	134.7172	81.33622	135.9238	81.4682	138.4595
81.41463	109.545	81.33538	133.6015	81.31827	137.6339	81.42483	134.7164	81.35288	135.9235	81.48487	138.4594
81.4313	109.5446	81.35205	133.603	81.33493	137.6276	81.4415	134.7147	81.36955	135.9243	81.50153	138.4591
81.44797	109.5411	81.36872	133.5983	81.3516	137.6292	81.45817	134.7137	81.38622	135.922	81.5182	138.4582
81.46463	109.5416	81.38538	133.5997	81.36827	137.6249	81.47483	134.7119	81.40288	135.9206	81.53487	138.4584
81.4813	109.5399	81.40205	133.5987	81.38493	137.6242	81.4915	134.7115	81.41955	135.9206	81.55153	138.4566
81.49797	109.539	81.41872	133.6034	81.4016	137.6234	81.50817	134.7096	81.43622	135.9189	81.5682	138.4576
81.51463	109.542	81.43538	133.5975	81.41827	137.6194	81.52483	134.7103	81.45288	135.9192	81.58487	138.457
81.5313	109.5347	81.45205	133.599	81.43493	137.6194	81.5415	134.7084	81.46955	135.9163	81.60153	138.4572
81.54797	109.5364	81.46872	133.5971	81.4516	137.6179	81.55817	134.7099	81.48622	135.9172	81.6182	138.4567
81.56463	109.5346	81.48538	133.5981	81.46827	137.6157	81.57483	134.708	81.50288	135.9159	81.63487	138.4558
81.5813	109.5345	81.50205	133.5981	81.48493	137.6138	81.5915	134.7072	81.51955	135.9156	81.65153	138.4565
81.59797	109.5293	81.51872	133.6015	81.5016	137.6108	81.60817	134.7062	81.53622	135.9149	81.6682	138.4547
81.61463	109.533	81.53538	133.5975	81.51827	137.6118	81.62483	134.7046	81.55288	135.9129	81.68487	138.4543
81.6313	109.5272	81.55205	133.6044	81.53493	137.609	81.6415	134.7036	81.56955	135.9136	81.70153	138.4534
81.64797	109.5279	81.56872	133.5967	81.5516	137.6086	81.65817	134.7038	81.58622	135.9111	81.7182	138.4529
81.66463	109.5272	81.58538	133.601	81.56827	137.5993	81.67483	134.7012	81.60288	135.9113	81.73487	138.4516
81.6813	109.5245	81.60205	133.5977	81.58493	137.6027	81.6915	134.7018	81.61955	135.9112	81.75153	138.4512
81.69797	109.5213	81.61872	133.6007	81.6016	137.5966	81.70817	134.6999	81.63622	135.9084	81.7682	138.4516
81.71463	109.5192	81.63538	133.6006	81.61827	137.5989	81.72483	134.6996	81.65288	135.9096	81.78487	138.4499
81.7313	109.5209	81.65205	133.5983	81.63493	137.5958	81.7415	134.6978	81.66955	135.9076	81.80153	138.4489
81.74797	109.5189	81.66872	133.5993	81.6516	137.5938	81.75817	134.6967	81.68622	135.9079	81.8182	138.4495
81.76463	109.5176	81.68538	133.5985	81.66827	137.5917	81.77483	134.6964	81.70288	135.9059	81.83487	138.4496
81.7813	109.5185	81.70205	133.6003	81.68493	137.5885	81.7915	134.6968	81.71955	135.9052	81.85153	138.447
81.79797	109.5158	81.71872	133.5999	81.7016	137.5881	81.80817	134.6922	81.73622	135.904	81.8682	138.4455
81.81463	109.5162	81.73538	133.5999	81.71827	137.5841	81.82483	134.6945	81.75288	135.9036	81.88487	138.4476
81.8313	109.5122	81.75205	133.5975	81.73493	137.5818	81.8415	134.692	81.76955	135.9022	81.90153	138.4462
81.84797	109.5119	81.76872	133.5968	81.7516	137.582	81.85817	134.6893	81.78622	135.9028	81.9182	138.4462
81.86463	109.5086	81.78538	133.5983	81.76827	137.5793	81.87483	134.6892	81.80288	135.901	81.93487	138.4454
81.8813	109.5086	81.80205	133.5989	81.78493	137.5757	81.8915	134.6892	81.81955	135.9006	81.95153	138.4456
81.89797	109.5077	81.81872	133.5959	81.8016	137.5749	81.90817	134.6864	81.83622	135.8987	81.9682	138.4426
81.91463	109.5068	81.83538	133.5949	81.81827	137.5734	81.92483	134.685	81.85288	135.9005	81.98487	138.4445
81.9313	109.5055	81.85205	133.5973	81.83493	137.5707	81.9415	134.6874	81.86955	135.8984	82.00153	138.4418
81.94797	109.507	81.86872	133.5944	81.8516	137.568	81.95817	134.6835	81.88622	135.9002	82.0182	138.4426
81.96463	109.5031	81.88538	133.5967	81.86827	137.5658	81.97483	134.6814	81.90288	135.8968	82.03487	138.4432
81.9813	109.5022	81.90205	133.5979	81.88493	137.5679	81.9915	134.6829	81.91955	135.8943	82.05153	138.4424
81.99797	109.5011	81.91872	133.5954	81.9016	137.5647	82.00817	134.681	81.93622	135.8961	82.0682	138.4432
82.01463	109.5016	81.93538	133.5975	81.91827	137.5623	82.02483	134.6797	81.95288	135.8951	82.08487	138.442
82.0313	109.4991	81.95205	133.5951	81.93493	137.5583	82.0415	134.6779	81.96955	135.8948	82.10153	138.4428
82.04797	109.4967	81.96872	133.5937	81.9516	137.5602	82.05817	134.6773	81.98622	135.8958	82.1182	138.441
82.06463	109.4951	81.98538	133.5965	81.96827	137.5576	82.07483	134.6764	82.00288	135.8938	82.13487	138.4412
82.0813	109.496	82.00205	133.5989	81.98493	137.5536	82.0915	134.677	82.01955	135.8913	82.15153	138.4417
82.09797	109.4962	82.01872	133.597	82.0016	137.5513	82.10817	134.6744	82.03622	135.8935	82.1682	138.4383
82.11463	109.4907	82.03538	133.5953	82.01827	137.5526	82.12483	134.674	82.05288	135.8891	82.18487	138.439
82.1313	109.4909	82.05205	133.6027	82.03493	137.5476	82.1415	134.6713	82.06955	135.8891	82.20153	138.4397
82.14797	109.4948	82.06872	133.5966	82.0516	137.5494	82.15817	134.6706	82.08622	135.8908	82.2182	138.44
82.16463	109.4871	82.08538	133.5976	82.06827	137.545	82.17483	134.6699	82.10288	135.8892	82.23487	138.4392
82.1813	109.4852	82.10205	133.5998	82.08493	137.543	82.1915	134.6702	82.11955	135.8873	82.25153	138.4391
82.19797	109.4878	82.11872	133.5987	82.1016	137.5418	82.20817	134.6696	82.13622	135.8852	82.2682	138.436
82.21463	109.4832	82.13538	133.601	82.11827	137.5405	82.22483	134.6674	82.15288	135.8868	82.28487	138.435
82.2313	109.486	82.15205	133.6003	82.13493	137.5388	82.2415	134.667	82.16955	135.883	82.30153	138.4352
82.24797	109.4789	82.16872	133.6035	82.1516	137.5405	82.25817	134.6669	82.18622	135.8845	82.3182	138.4366
82.26463	109.4805	82.18538	133.6022	82.16827	137.538	82.27483	134.6685	82.20288	135.8834	82.33487	138.4362
82.2813	109.4808	82.20205	133.6035	82.18493	137.5353	82.2915	134.6663	82.21955	135.8826	82.35153	138.4348
82.29797	109.4801	82.21872	133.6056	82.2016	137.5322	82.30817	134.6639	82.23622	135.8816	82.3682	138.435
82.31463	109.48	82.23538	133.6042	82.21827	137.5315	82.32483	134.6654	82.25288	135.8815	82.38487	138.4349
82.3313	109.4772	82.25205	133.6035	82.23493	137.5313	82.3415	134.6628	82.26955	135.8764	82.40153	138.4324
82.34797	109.4752	82.26872	133.606	82.2516	137.5295	82.35817	134.6646	82.28622	135.8826	82.4182	138.4302
82.36463	109.4728	82.28538	133.6039	82.26827	137.522	82.37483	134.6623	82.30288	135.8785	82.43487	138.4314
82.3813	109.4739	82.30205	133.6063	82.28493	137.5241	82.3915	134.6599	82.31955	135.8761	82.45153	138.4319
82.39797	109.4778	82.31872	133.6042	82.3016	137.5246	82.40817	134.6593	82.33622	135.8744	82.4682	138.4293
82.41463	109.4713	82.33538	133.605	82.31827	137.5198	82.42483	134.6611	82.35288	135.8755	82.48487	138.4313
82.4313	109.4712	82.35205	133.6056	82.33493	137.5192	82.4415	134.6597	82.36955	135.8747	82.50153	138.4302
82.44797	109.4726	82.36872	133.6043	82.3516	137.5162	82.45817	134.6604	82.38622	135.8726	82.5182	138.4295
82.46463	109.4665	82.38538	133.6019	82.36827	137.5159	82.47483	134.6604	82.40288	135.8696	82.53487	138.4263
82.4813	109.4691	82.40205	133.6043	82.38493	137.5144	82.4915	134.6585	82.41955	135.8737	82.55153	138.4259
82.49797	109.4634	82.41872	133.6042	82.4016	137.5126	82.50817	134.6598	82.43622	135.869	82.5682	138.4246

82.51463	109.4667	82.43538	133.603	82.41827	137.5078	82.52483	134.6587	82.45288	135.8711	82.58487	138.4245
82.5313	109.4652	82.45205	133.6027	82.43493	137.5087	82.5415	134.6556	82.46955	135.8699	82.60153	138.4253
82.54797	109.4659	82.46872	133.6015	82.4516	137.5071	82.55817	134.6554	82.48622	135.8694	82.6182	138.4264
82.56463	109.46	82.48538	133.6026	82.46827	137.5024	82.57483	134.6558	82.50288	135.8659	82.63487	138.4243
82.5813	109.4619	82.50205	133.6019	82.48493	137.5028	82.5915	134.6555	82.51955	135.8666	82.65153	138.4222
82.59797	109.4603	82.51872	133.6009	82.5016	137.4997	82.60817	134.6518	82.53622	135.8649	82.6682	138.4226
82.61463	109.4588	82.53538	133.6016	82.51827	137.4986	82.62483	134.6516	82.55288	135.8656	82.68487	138.4236
82.6313	109.4578	82.55205	133.602	82.53493	137.4966	82.6415	134.6521	82.56955	135.8642	82.70153	138.4203
82.64797	109.4572	82.56872	133.6037	82.5516	137.4925	82.65817	134.648	82.58622	135.8628	82.7182	138.4185
82.66463	109.4549	82.58538	133.6033	82.56827	137.4939	82.67483	134.6464	82.60288	135.864	82.73487	138.4229
82.6813	109.4551	82.60205	133.605	82.58493	137.4934	82.6915	134.6471	82.61955	135.8615	82.75153	138.4195
82.69797	109.4531	82.61872	133.6047	82.6016	137.4871	82.70817	134.6451	82.63622	135.8607	82.7682	138.4223
82.71463	109.4523	82.63538	133.6072	82.61827	137.4862	82.72483	134.6464	82.65288	135.8615	82.78487	138.4211
82.7313	109.4533	82.65205	133.6074	82.63493	137.4848	82.7415	134.6443	82.66955	135.8608	82.80153	138.4181
82.74797	109.4493	82.66872	133.6087	82.6516	137.4828	82.75817	134.645	82.68622	135.8605	82.8182	138.4191
82.76463	109.4484	82.68538	133.6062	82.66827	137.4806	82.77483	134.6437	82.70288	135.8564	82.83487	138.4188
82.7813	109.4498	82.70205	133.6067	82.68493	137.4774	82.7915	134.6408	82.71955	135.8616	82.85153	138.4176
82.79797	109.4444	82.71872	133.6075	82.7016	137.4772	82.80817	134.6398	82.73622	135.8557	82.8682	138.4164
82.81463	109.4487	82.73538	133.607	82.71827	137.474	82.82483	134.6394	82.75288	135.8582	82.88487	138.4159
82.8313	109.4443	82.75205	133.6044	82.73493	137.472	82.8415	134.6383	82.76955	135.856	82.90153	138.4153
82.84797	109.4429	82.76872	133.6055	82.7516	137.4715	82.85817	134.6387	82.78622	135.8557	82.9182	138.4158
82.86463	109.4418	82.78538	133.6085	82.76827	137.4684	82.87483	134.635	82.80288	135.8537	82.93487	138.4144
82.8813	109.4413	82.80205	133.6062	82.78493	137.4678	82.8915	134.6369	82.81955	135.8531	82.95153	138.4135
82.89797	109.4378	82.81872	133.6094	82.8016	137.4659	82.90817	134.6322	82.83622	135.8524	82.9682	138.4139
82.91463	109.4383	82.83538	133.6066	82.81827	137.4651	82.92483	134.6321	82.85288	135.8514	82.98487	138.414
82.9313	109.4362	82.85205	133.6076	82.83493	137.4612	82.9415	134.6302	82.86955	135.8515	83.00153	138.411
82.94797	109.4365	82.86872	133.6057	82.8516	137.4598	82.95817	134.6321	82.88622	135.8495	83.0182	138.4123
82.96463	109.4342	82.88538	133.6105	82.86827	137.4592	82.97483	134.6271	82.90288	135.8496	83.03487	138.409
82.9813	109.4329	82.90205	133.6085	82.88493	137.4549	82.9915	134.6278	82.91955	135.8486	83.05153	138.4112
82.99797	109.4317	82.91872	133.6089	82.9016	137.4549	83.00817	134.6273	82.93622	135.8468	83.0682	138.4111
83.01463	109.4324	82.93538	133.6106	82.91827	137.451	83.02483	134.628	82.95288	135.8465	83.08487	138.4098
83.0313	109.4289	82.95205	133.6121	82.93493	137.4492	83.0415	134.626	82.96955	135.8429	83.10153	138.4092
83.04797	109.4278	82.96872	133.616	82.9516	137.4521	83.05817	134.6252	82.98622	135.8464	83.1182	138.4079
83.06463	109.4278	82.98538	133.61	82.96827	137.4484	83.07483	134.6228	83.00288	135.8432	83.13487	138.408
83.0813	109.4256	83.00205	133.6137	82.98493	137.4462	83.0915	134.6222	83.01955	135.8419	83.15153	138.4052
83.09797	109.4234	83.01872	133.6123	83.0016	137.4439	83.10817	134.619	83.03622	135.8412	83.1682	138.4068
83.11463	109.4226	83.03538	133.6102	83.01827	137.4408	83.12483	134.618	83.05288	135.8418	83.18487	138.4065
83.1313	109.4201	83.05205	133.6147	83.03493	137.4398	83.1415	134.6202	83.06955	135.8398	83.20153	138.4038
83.14797	109.4197	83.06872	133.6109	83.0516	137.4373	83.15817	134.6207	83.08622	135.8391	83.2182	138.4018
83.16463	109.415	83.08538	133.6131	83.06827	137.4348	83.17483	134.6164	83.10288	135.8385	83.23487	138.4027
83.1813	109.4182	83.10205	133.6112	83.08493	137.432	83.1915	134.6167	83.11955	135.8359	83.25153	138.4014
83.19797	109.4174	83.11872	133.6105	83.1016	137.4323	83.20817	134.6177	83.13622	135.8367	83.2682	138.3993
83.21463	109.4157	83.13538	133.6094	83.11827	137.4253	83.22483	134.6138	83.15288	135.8371	83.28487	138.4019
83.2313	109.4116	83.15205	133.6135	83.13493	137.4267	83.2415	134.6155	83.16955	135.8332	83.30153	138.4001
83.24797	109.4089	83.16872	133.6134	83.1516	137.421	83.25817	134.6152	83.18622	135.8336	83.3182	138.4001
83.26463	109.4088	83.18538	133.6107	83.16827	137.4208	83.27483	134.6092	83.20288	135.8316	83.33487	138.3975
83.2813	109.4091	83.20205	133.6119	83.18493	137.4201	83.2915	134.6125	83.21955	135.8319	83.35153	138.3978
83.29797	109.4069	83.21872	133.6115	83.2016	137.4174	83.30817	134.6113	83.23622	135.8317	83.3682	138.3955
83.31463	109.4047	83.23538	133.6126	83.21827	137.416	83.32483	134.6116	83.25288	135.8316	83.38487	138.3986
83.3313	109.4042	83.25205	133.614	83.23493	137.4127	83.3415	134.6075	83.26955	135.8296	83.40153	138.3964
83.34797	109.402	83.26872	133.6135	83.2516	137.4111	83.35817	134.6075	83.28622	135.8304	83.4182	138.3953
83.36463	109.4036	83.28538	133.6142	83.26827	137.4125	83.37483	134.6097	83.30288	135.8284	83.43487	138.3928
83.3813	109.4015	83.30205	133.6159	83.28493	137.4104	83.3915	134.6082	83.31955	135.8277	83.45153	138.3942
83.39797	109.4011	83.31872	133.6134	83.3016	137.4054	83.40817	134.606	83.33622	135.8284	83.4682	138.3929
83.41463	109.3938	83.33538	133.6148	83.31827	137.4044	83.42483	134.6057	83.35288	135.8256	83.48487	138.3941
83.4313	109.3934	83.35205	133.6147	83.33493	137.4016	83.4415	134.6039	83.36955	135.8267	83.50153	138.3915
83.44797	109.3934	83.36872	133.6149	83.3516	137.3974	83.45817	134.6051	83.38622	135.8235	83.5182	138.3916
83.46463	109.3941	83.38538	133.6172	83.36827	137.4006	83.47483	134.6044	83.40288	135.8249	83.53487	138.3894
83.4813	109.3923	83.40205	133.6138	83.38493	137.3969	83.4915	134.6002	83.41955	135.8239	83.55153	138.3902
83.49797	109.3883	83.41872	133.6164	83.4016	137.3956	83.50817	134.5997	83.43622	135.825	83.5682	138.3903
83.51463	109.3876	83.43538	133.619	83.41827	137.3942	83.52483	134.5987	83.45288	135.822	83.58487	138.3894
83.5313	109.3891	83.45205	133.6139	83.43493	137.3903	83.5415	134.6012	83.46955	135.8221	83.60153	138.3892
83.54797	109.3873	83.46872	133.6157	83.4516	137.3893	83.55817	134.5997	83.48622	135.8236	83.6182	138.387
83.56463	109.3813	83.48538	133.6157	83.46827	137.3896	83.57483	134.5989	83.50288	135.8201	83.63487	138.387
83.5813	109.3853	83.50205	133.6169	83.48493	137.3851	83.5915	134.5985	83.51955	135.8204	83.65153	138.3884
83.59797	109.3833	83.51872	133.6159	83.5016	137.3851	83.60817	134.5963	83.53622	135.8187	83.6682	138.3859
83.61463	109.3785	83.53538	133.6131	83.51827	137.386	83.62483	134.5955	83.55288	135.821	83.68487	138.3843
83.6313	109.3774	83.55205	133.6139	83.53493	137.3834	83.6415	134.5976	83.56955	135.8194	83.70153	138.3859
83.64797	109.3783	83.56872	133.6163	83.5516	137.3793	83.65817	134.5947	83.58622	135.8163	83.7182	138.3863
83.66463	109.3777	83.58538	133.6165	83.56827	137.379	83.67483	134.592	83.60288	135.8178	83.73487	138.3823
83.6813	109.3742	83.60205	133.6141	83.58493	137.3797	83.6915	134.5933	83.61955	135.8161	83.75153	138.3855
83.69797	109.3723	83.61872	133.6143	83.6016	137.3751	83.70817	134.5896	83.63622	135.8151	83.7682	138.384
83.71463	109.3736	83.63538	133.6137	83.61827	137.3715	83.72483	134.5908	83.65288	135.8131	83.78487	138.3836
83.7313	109.3716	83.65205	133.6161	83.63493	137.3716	83.7415	134.5882	83.66955	135.8122	83.80153	138.3826
83.74797	109.3697	83.66872	133.6169	83.6516	137.3667	83.75817	134.587	83.68622	135.8114	83.8182	138.383
83.76463	109.3701	83.68538	133.6143	83.66827	137.3669	83.77483	134.586	83.70288	135.8109	83.83487	138.3823
83.7813	109.3675	83.70205	133.6147	83.68493	137.3649	83.7915	134.584	83.71955	135.8116	83.85153	138.3809
83.79797	109.3662	83.71872	133.6177	83.7016	137.3649	83.80817	134.5843	83.73622	135.8112	83.8682	138.3802
83.81463	109.3654	83.73538	133.6182	83.71827	137.3637	83.82483	134.5827	83.75288	135.809	83.88487	138.3805
83.8313	109.3628	83.75205	133.6146	83.73493	137.3583	83.8415	134.5802	83.76955	135.808	83.90153	138.3794
83.84797	109.3642	83.76872	133.6176	83.7516	137.3595	83.85817	134.5808	83.78622	135.8107	83.9182	138.3779
83.86463	109.3605	83.78538	133.6153	83.76827	137.3544	83.87483	134.5795	83.80288	135.8104	83.93487	138.3775
83.8813	109.3607	83.80205	133.6182	83.78493	137.3541	83.8915	134.5788	83.81955	135.8082	83.95153	138.3737
83.89797	109.3581	83.81872	133.617	83.8016	137.3527	83.90817	134.575	83.83622	135.8083	83.9682	138.3759
83.91463	109.3557	83.83538	133.6144	83.81827	137.3514	83.92483	134.576	83.85288	135.8057	83.98487	138.373
83.9313	109.3551	83.85205	133.6193	83.83493	137.3512	83.9415	134.5753	83.86955	135.8061	84.00153	138.3725
83.94797	109.3552	83.86872	133.6177	83.8516	137.3504	83.95817	134.5727	83.88622	135.8056	84.0182	138.3733
83.96463	109.3525	83.88538	133.6122	83.86827	137.3448	83.97483	134.5719	83.90288	135.8052	84.03487	138.3729
83.9813	109.3533	83.90205	133.6187	83.88493	137.3419	83.9915	134.5687	83.91955	135.8058	84.05153	138.3731
83.99797	109.3515	83.91872	133.6181	83.9016	137.3451	84.00817	134.5706	83.93622	135.8058	84.0682	138.3714
84.01463	109.35	83.93538	133.6188	83.91827	137.3407	84.02483	134.5659	83.95288	135.8054	84.08487	138.3711
84.0313	109.3469	83.95205	133.6172	83.93493	137.338	84.0415	134.5648	83.96955	135.8002	84.10153	138.3702
84.04797	109.3493	83.96872	133.6205	83.9516	137.3342	84.05817	134.5649	83.98622	135.8019	84.1182	138.3704
84.06463	109.3468	83.98538	133.6176	83.96827	137.3366	84.07483	134.5628	84.00288	135.8011	84.13487	138.3681
84.0813	109.3422	84.00205	133.6188	83.98493	137.3362	84.0915	134.5617	84.01955	135.8011	84.15153	138.3683
84.09797	109.3438	84.01872	133.6164	84.0016	137.3312	84.10817	134.5606	84.03622	135.8003	84.1682	138.3679
84.11463	109.3425	84.03538	133.6168	84.01827	137.3292	84.12483	134.5598	84.05288	135.7989	84.18487	138.3655

84.1313	109.3422	84.05205	133.6166	84.03493	137.3262	84.1415	134.56	84.06955	135.7967	84.20153	138.366
84.14797	109.3402	84.06872	133.6193	84.0516	137.3256	84.15817	134.5586	84.08622	135.7982	84.2182	138.3646
84.16463	109.3416	84.08538	133.6174	84.06827	137.3245	84.17483	134.5579	84.10288	135.7959	84.23487	138.3618
84.1813	109.3377	84.10205	133.62	84.08493	137.3202	84.1915	134.5558	84.11955	135.797	84.25153	138.3633
84.19797	109.3358	84.11872	133.6184	84.1016	137.3158	84.20817	134.5549	84.13622	135.794	84.2682	138.3614
84.21463	109.3351	84.13538	133.6194	84.11827	137.3179	84.22483	134.5547	84.15288	135.7965	84.28487	138.3641
84.2313	109.3324	84.15205	133.622	84.13493	137.3234	84.2415	134.554	84.16955	135.7946	84.30153	138.3616
84.24797	109.3318	84.16872	133.6222	84.1516	137.3179	84.25817	134.5488	84.18622	135.7947	84.3182	138.3606
84.26463	109.3319	84.18538	133.6207	84.16827	137.3093	84.27483	134.5507	84.20288	135.7914	84.33487	138.3596
84.2813	109.3351	84.20205	133.6184	84.18493	137.3109	84.2915	134.5502	84.21955	135.7933	84.35153	138.3592
84.29797	109.3278	84.21872	133.6216	84.2016	137.3085	84.30817	134.5485	84.23622	135.7897	84.3682	138.3586
84.31463	109.3277	84.23538	133.6187	84.21827	137.3063	84.32483	134.5468	84.25288	135.7915	84.38487	138.3561
84.3313	109.3278	84.25205	133.6185	84.23493	137.3055	84.3415	134.5461	84.26955	135.7891	84.40153	138.3553
84.34797	109.3242	84.26872	133.6185	84.2516	137.2998	84.35817	134.5459	84.28622	135.7892	84.4182	138.3556
84.36463	109.3229	84.28538	133.62	84.26827	137.302	84.37483	134.5462	84.30288	135.7871	84.43487	138.3533
84.3813	109.3196	84.30205	133.6246	84.28493	137.2965	84.3915	134.5428	84.31955	135.7878	84.45153	138.3544
84.39797	109.3229	84.31872	133.6226	84.3016	137.3047	84.40817	134.5414	84.33622	135.7871	84.4682	138.3539
84.41463	109.3204	84.33538	133.6261	84.31827	137.2933	84.42483	134.5414	84.35288	135.7875	84.48487	138.3505
84.4313	109.3191	84.35205	133.6233	84.33493	137.291	84.4415	134.541	84.36955	135.7849	84.50153	138.3512
84.44797	109.3209	84.36872	133.6212	84.3516	137.294	84.45817	134.5394	84.38622	135.7851	84.5182	138.3511
84.46463	109.3151	84.38538	133.6299	84.36827	137.2855	84.47483	134.5384	84.40288	135.786	84.53487	138.3507
84.4813	109.3134	84.40205	133.626	84.38493	137.2899	84.4915	134.5363	84.41955	135.7843	84.55153	138.3495
84.49797	109.3143	84.41872	133.6245	84.4016	137.2873	84.50817	134.5377	84.43622	135.7812	84.5682	138.348
84.51463	109.3139	84.43538	133.6278	84.41827	137.2853	84.52483	134.5341	84.45288	135.7828	84.58487	138.3482
84.5313	109.3088	84.45205	133.6319	84.43493	137.2855	84.5415	134.534	84.46955	135.7843	84.60153	138.3477
84.54797	109.3099	84.46872	133.6247	84.4516	137.2828	84.55817	134.5318	84.48622	135.7807	84.6182	138.3479
84.56463	109.3117	84.48538	133.6259	84.46827	137.2801	84.57483	134.5311	84.50288	135.78	84.63487	138.346
84.5813	109.3077	84.50205	133.6287	84.48493	137.2817	84.5915	134.5325	84.51955	135.7788	84.65153	138.3471
84.59797	109.3063	84.51872	133.6297	84.5016	137.2792	84.60817	134.5301	84.53622	135.7778	84.6682	138.3474
84.61463	109.3033	84.53538	133.6262	84.51827	137.2787	84.62483	134.528	84.55288	135.7788	84.68487	138.3444
84.6313	109.3053	84.55205	133.6283	84.53493	137.2779	84.6415	134.5268	84.56955	135.7768	84.70153	138.3434
84.64797	109.2992	84.56872	133.6242	84.5516	137.2725	84.65817	134.5271	84.58622	135.7755	84.7182	138.3435
84.66463	109.3027	84.58538	133.6281	84.56827	137.2685	84.67483	134.5263	84.60288	135.7748	84.73487	138.3423
84.6813	109.2979	84.60205	133.6258	84.58493	137.2685	84.6915	134.5239	84.61955	135.7752	84.75153	138.3424
84.69797	109.2954	84.61872	133.6267	84.6016	137.2697	84.70817	134.5214	84.63622	135.7718	84.7682	138.3417
84.71463	109.2963	84.63538	133.6245	84.61827	137.2633	84.72483	134.5213	84.65288	135.7727	84.78487	138.3408
84.7313	109.297	84.65205	133.6286	84.63493	137.2645	84.7415	134.5196	84.66955	135.7702	84.80153	138.3395
84.74797	109.2932	84.66872	133.6271	84.6516	137.2635	84.75817	134.5204	84.68622	135.7718	84.8182	138.3414
84.76463	109.2918	84.68538	133.624	84.66827	137.2639	84.77483	134.5171	84.70288	135.7678	84.83487	138.34
84.7813	109.2922	84.70205	133.6282	84.68493	137.2599	84.7915	134.5155	84.71955	135.7686	84.85153	138.3386
84.79797	109.2909	84.71872	133.6252	84.7016	137.2625	84.80817	134.5151	84.73622	135.768	84.8682	138.3374
84.81463	109.2868	84.73538	133.6284	84.71827	137.2512	84.82483	134.5167	84.75288	135.7683	84.88487	138.3385
84.8313	109.2863	84.75205	133.6268	84.73493	137.2503	84.8415	134.5144	84.76955	135.7679	84.90153	138.335
84.84797	109.2867	84.76872	133.6271	84.7516	137.2506	84.85817	134.5138	84.78622	135.7652	84.9182	138.3356
84.86463	109.2832	84.78538	133.6279	84.76827	137.2524	84.87483	134.512	84.80288	135.7646	84.93487	138.3364
84.8813	109.2845	84.80205	133.6269	84.78493	137.2514	84.8915	134.5092	84.81955	135.7625	84.95153	138.3351
84.89797	109.282	84.81872	133.629	84.8016	137.2465	84.90817	134.5094	84.83622	135.7632	84.9682	138.3311
84.91463	109.2792	84.83538	133.6309	84.81827	137.2472	84.92483	134.5082	84.85288	135.7632	84.98487	138.3341
84.9313	109.278	84.85205	133.6314	84.83493	137.2435	84.9415	134.5063	84.86955	135.7611	85.00153	138.3316
84.94797	109.2776	84.86872	133.6307	84.8516	137.2456	84.95817	134.506	84.88622	135.7604	85.0182	138.3322
84.96463	109.2762	84.88538	133.63	84.86827	137.2416	84.97483	134.5053	84.90288	135.7595	85.03487	138.3321
84.9813	109.276	84.90205	133.6361	84.88493	137.2404	84.9915	134.502	84.91955	135.762	85.05153	138.3301
84.99797	109.2733	84.91872	133.6334	84.9016	137.2372	85.00817	134.5041	84.93622	135.7603	85.0682	138.3285
85.01463	109.2718	84.93538	133.633	84.91827	137.2357	85.02483	134.5017	84.95288	135.7588	85.08487	138.3289
85.0313	109.2722	84.95205	133.6354	84.93493	137.2347	85.0415	134.5	84.96955	135.7573	85.10153	138.3287
85.04797	109.2684	84.96872	133.6348	84.9516	137.2313	85.05817	134.4981	84.98622	135.7585	85.1182	138.329
85.06463	109.2686	84.98538	133.6333	84.96827	137.2304	85.07483	134.4986	85.00288	135.7559	85.13487	138.3279
85.0813	109.2685	85.00205	133.6372	84.98493	137.2307	85.0915	134.4963	85.01955	135.7556	85.15153	138.3273
85.09797	109.2655	85.01872	133.6361	85.0016	137.2295	85.10817	134.4928	85.03622	135.7553	85.1682	138.3252
85.11463	109.2674	85.03538	133.6359	85.01827	137.2237	85.12483	134.4927	85.05288	135.7555	85.18487	138.3252
85.1313	109.2624	85.05205	133.6379	85.03493	137.2243	85.1415	134.4907	85.06955	135.7544	85.20153	138.3251
85.14797	109.2643	85.06872	133.637	85.0516	137.2225	85.15817	134.4909	85.08622	135.7531	85.2182	138.3244
85.16463	109.2623	85.08538	133.637	85.06827	137.2178	85.17483	134.488	85.10288	135.7498	85.23487	138.3233
85.1813	109.2595	85.10205	133.633	85.08493	137.2172	85.1915	134.4883	85.11955	135.7515	85.25153	138.322
85.19797	109.2594	85.11872	133.6363	85.1016	137.2165	85.20817	134.4859	85.13622	135.752	85.2682	138.3209
85.21463	109.2596	85.13538	133.6366	85.11827	137.2151	85.22483	134.4866	85.15288	135.7489	85.28487	138.3224
85.2313	109.2578	85.15205	133.6366	85.13493	137.2102	85.2415	134.4827	85.16955	135.7488	85.30153	138.32
85.24797	109.2551	85.16872	133.6341	85.1516	137.2099	85.25817	134.4815	85.18622	135.7477	85.3182	138.318
85.26463	109.2534	85.18538	133.6367	85.16827	137.2055	85.27483	134.4802	85.20288	135.7478	85.33487	138.3184
85.2813	109.2543	85.20205	133.637	85.18493	137.2071	85.2915	134.4808	85.21955	135.7459	85.35153	138.317
85.29797	109.2491	85.21872	133.637	85.2016	137.2051	85.30817	134.477	85.23622	135.7451	85.3682	138.3163
85.31463	109.2512	85.23538	133.6335	85.21827	137.2023	85.32483	134.4769	85.25288	135.745	85.38487	138.3187
85.3313	109.2475	85.25205	133.6378	85.23493	137.2027	85.3415	134.4785	85.26955	135.7443	85.40153	138.3152
85.34797	109.2475	85.26872	133.6403	85.2516	137.1986	85.35817	134.4752	85.28622	135.7431	85.4182	138.3136
85.36463	109.2476	85.28538	133.6381	85.26827	137.1978	85.37483	134.4726	85.30288	135.7438	85.43487	138.3122
85.3813	109.2479	85.30205	133.638	85.28493	137.1957	85.3915	134.472	85.31955	135.7399	85.45153	138.3136
85.39797	109.2458	85.31872	133.6402	85.3016	137.1928	85.40817	134.471	85.33622	135.7416	85.4682	138.3097
85.41463	109.2458	85.33538	133.6425	85.31827	137.1929	85.42483	134.4694	85.35288	135.7416	85.48487	138.3106
85.4313	109.2417	85.35205	133.6449	85.33493	137.1903	85.4415	134.4706	85.36955	135.739	85.50153	138.3114
85.44797	109.2446	85.36872	133.6435	85.3516	137.1875	85.45817	134.4633	85.38622	135.7395	85.5182	138.3097
85.46463	109.2409	85.38538	133.6447	85.36827	137.1849	85.47483	134.4664	85.40288	135.7391	85.53487	138.3093
85.4813	109.2399	85.40205	133.6465	85.38493	137.1835	85.4915	134.4669	85.41955	135.7378	85.55153	138.31
85.49797	109.2363	85.41872	133.6448	85.4016	137.1847	85.50817	134.4655	85.43622	135.7374	85.5682	138.3086
85.51463	109.2377	85.43538	133.6486	85.41827	137.1812	85.52483	134.4627	85.45288	135.7379	85.58487	138.3063
85.5313	109.2416	85.45205	133.6442	85.43493	137.1808	85.5415	134.4607	85.46955	135.7366	85.60153	138.3056
85.54797	109.2346	85.46872	133.6516	85.4516	137.1759	85.55817	134.4611	85.48622	135.7361	85.6182	138.3057
85.56463	109.233	85.48538	133.6505	85.46827	137.1748	85.57483	134.4602	85.50288	135.7353	85.63487	138.3055
85.5813	109.2361	85.50205	133.6497	85.48493	137.1756	85.5915	134.4578	85.51955	135.7361	85.65153	138.3063
85.59797	109.2356	85.51872	133.6497	85.5016	137.173	85.60817	134.4551	85.53622	135.7333	85.6682	138.3042
85.61463	109.2273	85.53538	133.6515	85.51827	137.1703	85.62483	134.4553	85.55288	135.7344	85.68487	138.3036
85.6313	109.2313	85.55205	133.6489	85.53493	137.1688	85.6415	134.4545	85.56955	135.7336	85.70153	138.3031
85.64797	109.2263	85.56872	133.6544	85.5516	137.1695	85.65817	134.4522	85.58622	135.7292	85.7182	138.3001
85.66463	109.2276	85.58538	133.6515	85.56827	137.1619	85.67483	134.4511	85.60288	135.7312	85.73487	138.2994
85.6813	109.2267	85.60205	133.6515	85.58493	137.1611	85.6915	134.4505	85.61955	135.7331	85.75153	138.3005
85.69797	109.2278	85.61872	133.651	85.6016	137.1618	85.70817	134.4509	85.63622	135.7311	85.7682	138.2987
85.71463	109.2219	85.63538	133.6499	85.61827	137.1598	85.72483	134.4486	85.65288	135.7322	85.78487	138.2961
85.7313	109.2252	85.65205	133.651	85.63493	137.1609	85.7415	134.4452	85.66955	135.7298	85.80153	138.2978

85.74797	109.2225	85.66872	133.6547	85.6516	137.1564	85.75817	134.4458	85.68622	135.7303	85.8182	138.2989
85.76463	109.2164	85.68538	133.6535	85.66827	137.1528	85.77483	134.4453	85.70288	135.7316	85.83487	138.2943
85.7813	109.2209	85.70205	133.6553	85.68493	137.153	85.7915	134.4425	85.71955	135.7277	85.85153	138.2955
85.79797	109.2176	85.71872	133.6539	85.7016	137.1523	85.80817	134.4411	85.73622	135.7261	85.8682	138.2943
85.81463	109.2165	85.73538	133.654	85.71827	137.1448	85.82483	134.439	85.75288	135.7312	85.88487	138.2942
85.8313	109.2153	85.75205	133.6529	85.73493	137.1457	85.8415	134.4378	85.76955	135.7295	85.90153	138.2933
85.84797	109.2145	85.76872	133.6562	85.7516	137.148	85.85817	134.4379	85.78622	135.7279	85.9182	138.2927
85.86463	109.2107	85.78538	133.6546	85.76827	137.1424	85.87483	134.4365	85.80288	135.7285	85.93487	138.291
85.8813	109.2121	85.80205	133.6539	85.78493	137.1401	85.8915	134.4332	85.81955	135.727	85.95153	138.2903
85.89797	109.2106	85.81872	133.656	85.8016	137.1409	85.90817	134.433	85.83622	135.7257	85.9682	138.2909
85.91463	109.2082	85.83538	133.6559	85.81827	137.1412	85.92483	134.4342	85.85288	135.7243	85.98487	138.2893
85.9313	109.2087	85.85205	133.6542	85.83493	137.1368	85.9415	134.4296	85.86955	135.7235	86.00153	138.2861
85.94797	109.2052	85.86872	133.6593	85.8516	137.1347	85.95817	134.4285	85.88622	135.7249	86.0182	138.2866
85.96463	109.2076	85.88538	133.6539	85.86827	137.1295	85.97483	134.4284	85.90288	135.7256	86.03487	138.2859
85.9813	109.2027	85.90205	133.6544	85.88493	137.1272	85.9915	134.427	85.91955	135.7207	86.05153	138.2862
85.99797	109.2047	85.91872	133.654	85.9016	137.1266	86.00817	134.4239	85.93622	135.7203	86.0682	138.2855
86.01463	109.2023	85.93538	133.6539	85.91827	137.1268	86.02483	134.4231	85.95288	135.7177	86.08487	138.2859
86.0313	109.2028	85.95205	133.6558	85.93493	137.1249	86.0415	134.4228	85.96955	135.7178	86.10153	138.2835
86.04797	109.2014	85.96872	133.6579	85.9516	137.1301	86.05817	134.4206	85.98622	135.7159	86.1182	138.2837
86.06463	109.1978	85.98538	133.6544	85.96827	137.1224	86.07483	134.4199	86.00288	135.7151	86.13487	138.2828
86.0813	109.1976	86.00205	133.6535	85.98493	137.1204	86.0915	134.4187	86.01955	135.714	86.15153	138.2821
86.09797	109.1973	86.01872	133.6566	86.0016	137.1204	86.10817	134.4155	86.03622	135.7138	86.1682	138.2799
86.11463	109.1953	86.03538	133.6567	86.01827	137.1203	86.12483	134.4145	86.05288	135.7112	86.18487	138.2797
86.1313	109.1946	86.05205	133.6538	86.03493	137.1157	86.1415	134.4145	86.06955	135.7102	86.20153	138.2777
86.14797	109.1933	86.06872	133.6565	86.0516	137.1141	86.15817	134.4124	86.08622	135.7098	86.2182	138.2776
86.16463	109.1952	86.08538	133.6579	86.06827	137.1109	86.17483	134.4125	86.10288	135.7066	86.23487	138.2771
86.1813	109.1907	86.10205	133.6587	86.08493	137.1109	86.1915	134.4118	86.11955	135.7068	86.25153	138.2774
86.19797	109.1935	86.11872	133.6574	86.1016	137.1083	86.20817	134.4096	86.13622	135.7046	86.2682	138.2756
86.21463	109.1916	86.13538	133.6571	86.11827	137.108	86.22483	134.4077	86.15288	135.7043	86.28487	138.2757
86.2313	109.19	86.15205	133.6568	86.13493	137.1052	86.2415	134.406	86.16955	135.7046	86.30153	138.2719
86.24797	109.1871	86.16872	133.6605	86.1516	137.1024	86.25817	134.4034	86.18622	135.7021	86.3182	138.2735
86.26463	109.1829	86.18538	133.6603	86.16827	137.0987	86.27483	134.4023	86.20288	135.7021	86.33487	138.2717
86.2813	109.1855	86.20205	133.6582	86.18493	137.0993	86.2915	134.4019	86.21955	135.6988	86.35153	138.2706
86.29797	109.1814	86.21872	133.6575	86.2016	137.0973	86.30817	134.3988	86.23622	135.6994	86.3682	138.2693
86.31463	109.182	86.23538	133.6571	86.21827	137.0927	86.32483	134.398	86.25288	135.6964	86.38487	138.2708
86.3313	109.1789	86.25205	133.6608	86.23493	137.0934	86.3415	134.3973	86.26955	135.6948	86.40153	138.2694
86.34797	109.1841	86.26872	133.659	86.2516	137.09	86.35817	134.3959	86.28622	135.6965	86.4182	138.2685
86.36463	109.1806	86.28538	133.6597	86.26827	137.0882	86.37483	134.3916	86.30288	135.6951	86.43487	138.2671
86.3813	109.1788	86.30205	133.6599	86.28493	137.0846	86.3915	134.3932	86.31955	135.694	86.45153	138.2671
86.39797	109.1786	86.31872	133.6589	86.3016	137.0845	86.40817	134.3918	86.33622	135.6903	86.4682	138.2658
86.41463	109.1761	86.33538	133.6604	86.31827	137.0806	86.42483	134.3892	86.35288	135.6917	86.48487	138.2625
86.4313	109.174	86.35205	133.6567	86.33493	137.0796	86.4415	134.3899	86.36955	135.6888	86.50153	138.2634
86.44797	109.1772	86.36872	133.6606	86.3516	137.0785	86.45817	134.3875	86.38622	135.6889	86.5182	138.261
86.46463	109.1761	86.38538	133.6585	86.36827	137.076	86.47483	134.3873	86.40288	135.6871	86.53487	138.2639
86.4813	109.17	86.40205	133.6605	86.38493	137.0704	86.4915	134.3879	86.41955	135.6879	86.55153	138.263
86.49797	109.1728	86.41872	133.6605	86.4016	137.0719	86.50817	134.3872	86.43622	135.6853	86.5682	138.2591
86.51463	109.1673	86.43538	133.6595	86.41827	137.0726	86.52483	134.3847	86.45288	135.6858	86.58487	138.26
86.5313	109.1694	86.45205	133.6625	86.43493	137.0674	86.5415	134.3861	86.46955	135.6815	86.60153	138.2547
86.54797	109.1705	86.46872	133.6615	86.4516	137.066	86.55817	134.3833	86.48622	135.6793	86.6182	138.2574
86.56463	109.1642	86.48538	133.6641	86.46827	137.0649	86.57483	134.383	86.50288	135.6817	86.63487	138.2554
86.5813	109.1638	86.50205	133.6639	86.48493	137.0634	86.5915	134.382	86.51955	135.6831	86.65153	138.2558
86.59797	109.1684	86.51872	133.6636	86.5016	137.0642	86.60817	134.3791	86.53622	135.6802	86.6682	138.2566
86.61463	109.1631	86.53538	133.6659	86.51827	137.0621	86.62483	134.3787	86.55288	135.6777	86.68487	138.2544
86.6313	109.1615	86.55205	133.6641	86.53493	137.0583	86.6415	134.3804	86.56955	135.681	86.70153	138.253
86.64797	109.1609	86.56872	133.6651	86.5516	137.0598	86.65817	134.3761	86.58622	135.6777	86.7182	138.2511
86.66463	109.1585	86.58538	133.6656	86.56827	137.0582	86.67483	134.3757	86.60288	135.6755	86.73487	138.2506
86.6813	109.1541	86.60205	133.6661	86.58493	137.0553	86.6915	134.3751	86.61955	135.674	86.75153	138.2479
86.69797	109.1546	86.61872	133.6665	86.6016	137.0564	86.70817	134.3744	86.63622	135.6749	86.7682	138.2495
86.71463	109.1549	86.63538	133.6657	86.61827	137.054	86.72483	134.3726	86.65288	135.6717	86.78487	138.2494
86.7313	109.1504	86.65205	133.6644	86.63493	137.0516	86.7415	134.3686	86.66955	135.6739	86.80153	138.2478
86.74797	109.1498	86.66872	133.6691	86.6516	137.0501	86.75817	134.3712	86.68622	135.6713	86.8182	138.2471
86.76463	109.1488	86.68538	133.6675	86.66827	137.0533	86.77483	134.3677	86.70288	135.6703	86.83487	138.2446
86.7813	109.1471	86.70205	133.6719	86.68493	137.0493	86.7915	134.3675	86.71955	135.6697	86.85153	138.2445
86.79797	109.1509	86.71872	133.6687	86.7016	137.0489	86.80817	134.3676	86.73622	135.6679	86.8682	138.2437
86.81463	109.1482	86.73538	133.6732	86.71827	137.0466	86.82483	134.363	86.75288	135.6679	86.88487	138.2448
86.8313	109.1442	86.75205	133.6714	86.73493	137.045	86.8415	134.3656	86.76955	135.666	86.90153	138.2438
86.84797	109.1471	86.76872	133.6711	86.7516	137.0441	86.85817	134.3641	86.78622	135.6669	86.9182	138.2414
86.86463	109.1453	86.78538	133.6721	86.76827	137.0435	86.87483	134.3614	86.80288	135.6657	86.93487	138.2419
86.8813	109.14	86.80205	133.6714	86.78493	137.04	86.8915	134.3621	86.81955	135.6681	86.95153	138.2408
86.89797	109.1428	86.81872	133.6718	86.8016	137.0398	86.90817	134.3607	86.83622	135.6643	86.9682	138.2381
86.91463	109.1385	86.83538	133.6712	86.81827	137.0372	86.92483	134.3588	86.85288	135.6627	86.98487	138.2383
86.9313	109.1387	86.85205	133.6698	86.83493	137.0317	86.9415	134.3565	86.86955	135.663	87.00153	138.2374
86.94797	109.1363	86.86872	133.6705	86.8516	137.0321	86.95817	134.3552	86.88622	135.6617	87.0182	138.2376
86.96463	109.1356	86.88538	133.6746	86.86827	137.0326	86.97483	134.353	86.90288	135.6578	87.03487	138.2348
86.9813	109.1345	86.90205	133.6734	86.88493	137.0301	86.9915	134.3523	86.91955	135.6583	87.05153	138.2361
86.99797	109.1313	86.91872	133.6723	86.9016	137.0285	87.00817	134.3516	86.93622	135.6583	87.0682	138.2344
87.01463	109.1309	86.93538	133.6765	86.91827	137.0257	87.02483	134.3501	86.95288	135.6556	87.08487	138.2338
87.0313	109.1324	86.95205	133.6721	86.93493	137.0239	87.0415	134.3485	86.96955	135.6575	87.10153	138.2331
87.04797	109.1307	86.96872	133.6775	86.9516	137.0212	87.05817	134.3464	86.98622	135.6556	87.1182	138.2322
87.06463	109.1319	86.98538	133.6779	86.96827	137.02	87.07483	134.3472	87.00288	135.6545	87.13487	138.2303
87.0813	109.1273	87.00205	133.6744	86.98493	137.0168	87.0915	134.3459	87.01955	135.6544	87.15153	138.2307
87.09797	109.1274	87.01872	133.6765	87.0016	137.014	87.10817	134.3445	87.03622	135.6545	87.1682	138.2295
87.11463	109.1251	87.03538	133.68	87.01827	137.0157	87.12483	134.3423	87.05288	135.6508	87.18487	138.2269
87.1313	109.1254	87.05205	133.6823	87.03493	137.0113	87.1415	134.342	87.06955	135.6516	87.20153	138.2287
87.14797	109.1213	87.06872	133.681	87.0516	137.0085	87.15817	134.3415	87.08622	135.6513	87.2182	138.2285
87.16463	109.1224	87.08538	133.6806	87.06827	137.0058	87.17483	134.3385	87.10288	135.6491	87.23487	138.2252
87.1813	109.1193	87.10205	133.6815	87.08493	137.006	87.1915	134.3398	87.11955	135.6481	87.25153	138.2268
87.19797	109.1184	87.11872	133.6812	87.1016	137.0042	87.20817	134.3368	87.13622	135.6469	87.2682	138.2279
87.21463	109.1197	87.13538	133.6844	87.11827	137.0012	87.22483	134.3371	87.15288	135.644	87.28487	138.2257
87.2313	109.1168	87.15205	133.6814	87.13493	136.9998	87.2415	134.3355	87.16955	135.6425	87.30153	138.2233
87.24797	109.1157	87.16872	133.6831	87.1516	136.997	87.25817	134.3312	87.18622	135.6402	87.3182	138.2236
87.26463	109.1176	87.18538	133.6841	87.16827	136.9974	87.27483	134.3321	87.20288	135.6393	87.33487	138.2202
87.2813	109.1161	87.20205	133.6826	87.18493	136.9962	87.2915	134.3301	87.21955	135.6372	87.35153	138.2209
87.29797	109.115	87.21872	133.6827	87.2016	136.994	87.30817	134.3308	87.23622	135.6378	87.3682	138.2212
87.31463	109.1124	87.23538	133.6825	87.21827	136.9909	87.32483	134.3291	87.25288	135.6356	87.38487	138.2203
87.3313	109.1137	87.25205	133.6822	87.23493	136.9888	87.3415	134.328	87.26955	135.6361	87.40153	138.2173
87.34797	109.1101	87.26872	133.6838	87.2516	136.986	87.35817	134.3268	87.28622	135.634	87.4182	138.2167

87.36463	109.1093	87.28538	133.6835	87.26827	136.9861	87.37483	134.3249	87.30288	135.6329	87.43487	138.2153
87.3813	109.108	87.30205	133.6835	87.28493	136.9842	87.3915	134.3234	87.31955	135.6317	87.45153	138.2157
87.39797	109.1098	87.31872	133.6822	87.3016	136.9822	87.40817	134.3256	87.33622	135.6318	87.4682	138.2166
87.41463	109.1064	87.33538	133.6848	87.31827	136.9808	87.42483	134.323	87.35288	135.6334	87.48487	138.2146
87.4313	109.106	87.35205	133.6849	87.33493	136.9805	87.4415	134.322	87.36955	135.6298	87.50153	138.2118
87.44797	109.1059	87.36872	133.6867	87.3516	136.9775	87.45817	134.3193	87.38622	135.6283	87.5182	138.2113
87.46463	109.1035	87.38538	133.684	87.36827	136.9762	87.47483	134.3202	87.40288	135.6287	87.53487	138.2082
87.4813	109.1027	87.40205	133.6861	87.38493	136.9733	87.4915	134.3167	87.41955	135.6282	87.55153	138.2104
87.49797	109.1031	87.41872	133.6873	87.4016	136.9742	87.50817	134.318	87.43622	135.6256	87.5682	138.2094
87.51463	109.0994	87.43538	133.6881	87.41827	136.9694	87.52483	134.314	87.45288	135.6269	87.58487	138.2082
87.5313	109.0987	87.45205	133.6884	87.43493	136.9684	87.5415	134.3137	87.46955	135.623	87.60153	138.2093
87.54797	109.0991	87.46872	133.6891	87.4516	136.9667	87.55817	134.3146	87.48622	135.6247	87.6182	138.2068
87.56463	109.0967	87.48538	133.6899	87.46827	136.9639	87.57483	134.3123	87.50288	135.6232	87.63487	138.2048
87.5813	109.0964	87.50205	133.6914	87.48493	136.9607	87.5915	134.3117	87.51955	135.6218	87.65153	138.2034
87.59797	109.0953	87.51872	133.6863	87.5016	136.9615	87.60817	134.3099	87.53622	135.6213	87.6682	138.2045
87.61463	109.0947	87.53538	133.691	87.51827	136.9578	87.62483	134.3073	87.55288	135.6235	87.68487	138.2026
87.6313	109.0943	87.55205	133.6891	87.53493	136.9575	87.6415	134.3086	87.56955	135.6182	87.70153	138.2022
87.64797	109.0939	87.56872	133.6899	87.5516	136.9552	87.65817	134.307	87.58622	135.6174	87.7182	138.1997
87.66463	109.0926	87.58538	133.6873	87.56827	136.9562	87.67483	134.3058	87.60288	135.6181	87.73487	138.2013
87.6813	109.0901	87.60205	133.6888	87.58493	136.9459	87.6915	134.3039	87.61955	135.6178	87.75153	138.2014
87.69797	109.0894	87.61872	133.6894	87.6016	136.9461	87.70817	134.3029	87.63622	135.6179	87.7682	138.2011
87.71463	109.0877	87.63538	133.6914	87.61827	136.9472	87.72483	134.302	87.65288	135.6156	87.78487	138.2016
87.7313	109.0852	87.65205	133.6901	87.63493	136.9398	87.7415	134.302	87.66955	135.6139	87.80153	138.2
87.74797	109.0833	87.66872	133.6886	87.6516	136.9418	87.75817	134.2975	87.68622	135.614	87.8182	138.1993
87.76463	109.0824	87.68538	133.6914	87.66827	136.9416	87.77483	134.2988	87.70288	135.6132	87.83487	138.1993
87.7813	109.0848	87.70205	133.6913	87.68493	136.9359	87.7915	134.2984	87.71955	135.6124	87.85153	138.1992
87.79797	109.0815	87.71872	133.6899	87.7016	136.9363	87.80817	134.2961	87.73622	135.61	87.8682	138.1967
87.81463	109.0819	87.73538	133.6903	87.71827	136.9357	87.82483	134.2933	87.75288	135.6099	87.88487	138.197
87.8313	109.0805	87.75205	133.6924	87.73493	136.9355	87.8415	134.2929	87.76955	135.6074	87.90153	138.1981
87.84797	109.0777	87.76872	133.6907	87.7516	136.9316	87.85817	134.2928	87.78622	135.6084	87.9182	138.1951
87.86463	109.0763	87.78538	133.6938	87.76827	136.9302	87.87483	134.2931	87.80288	135.6079	87.93487	138.1954
87.8813	109.0748	87.80205	133.6927	87.78493	136.9287	87.8915	134.2902	87.81955	135.6088	87.95153	138.1958
87.89797	109.0727	87.81872	133.6937	87.8016	136.9279	87.90817	134.2882	87.83622	135.6072	87.9682	138.193
87.91463	109.072	87.83538	133.6956	87.81827	136.9247	87.92483	134.2868	87.85288	135.6049	87.98487	138.1928
87.9313	109.0707	87.85205	133.6953	87.83493	136.9237	87.9415	134.2854	87.86955	135.6049	88.00153	138.1916
87.94797	109.0735	87.86872	133.6936	87.8516	136.9221	87.95817	134.2851	87.88622	135.604	88.0182	138.1919
87.96463	109.0717	87.88538	133.6949	87.86827	136.9188	87.97483	134.2825	87.90288	135.6013	88.03487	138.1904
87.9813	109.0671	87.90205	133.6966	87.88493	136.9188	87.9915	134.2841	87.91955	135.6013	88.05153	138.1908
87.99797	109.0643	87.91872	133.6964	87.9016	136.9162	88.00817	134.2848	87.93622	135.6007	88.0682	138.1911
88.01463	109.0605	87.93538	133.6969	87.91827	136.9148	88.02483	134.2813	87.95288	135.6024	88.08487	138.189
88.0313	109.0648	87.95205	133.6978	87.93493	136.912	88.0415	134.2785	87.96955	135.5993	88.10153	138.1881
88.04797	109.0651	87.96872	133.7006	87.9516	136.9113	88.05817	134.2808	87.98622	135.5981	88.1182	138.19
88.06463	109.0594	87.98538	133.7007	87.96827	136.9102	88.07483	134.2801	88.00288	135.598	88.13487	138.1878
88.0813	109.0599	88.00205	133.7026	87.98493	136.9069	88.0915	134.276	88.01955	135.5989	88.15153	138.1864
88.09797	109.0612	88.01872	133.6974	88.0016	136.9049	88.10817	134.2735	88.03622	135.5963	88.1682	138.1842
88.11463	109.0544	88.03538	133.6998	88.01827	136.9054	88.12483	134.2736	88.05288	135.5946	88.18487	138.1835
88.1313	109.0577	88.05205	133.7011	88.03493	136.9024	88.1415	134.2721	88.06955	135.5937	88.20153	138.1827
88.14797	109.053	88.06872	133.7039	88.0516	136.899	88.15817	134.271	88.08622	135.5918	88.2182	138.1823
88.16463	109.0551	88.08538	133.7029	88.06827	136.9003	88.17483	134.2716	88.10288	135.5938	88.23487	138.183
88.1813	109.0563	88.10205	133.7004	88.08493	136.8972	88.1915	134.2688	88.11955	135.5905	88.25153	138.181
88.19797	109.0512	88.11872	133.7056	88.1016	136.8962	88.20817	134.2685	88.13622	135.5919	88.2682	138.1817
88.21463	109.0492	88.13538	133.7042	88.11827	136.8936	88.22483	134.2675	88.15288	135.5898	88.28487	138.1814
88.2313	109.0523	88.15205	133.7041	88.13493	136.8941	88.2415	134.2663	88.16955	135.5892	88.30153	138.1797
88.24797	109.0532	88.16872	133.7057	88.1516	136.8941	88.25817	134.2633	88.18622	135.5878	88.3182	138.1797
88.26463	109.0527	88.18538	133.706	88.16827	136.8892	88.27483	134.2637	88.20288	135.5865	88.33487	138.1796
88.2813	109.0462	88.20205	133.7072	88.18493	136.8876	88.2915	134.2616	88.21955	135.5865	88.35153	138.1777
88.29797	109.0462	88.21872	133.7047	88.2016	136.8882	88.30817	134.262	88.23622	135.5854	88.3682	138.1774
88.31463	109.0423	88.23538	133.7051	88.21827	136.8805	88.32483	134.2595	88.25288	135.5855	88.38487	138.1775
88.3313	109.0449	88.25205	133.706	88.23493	136.8838	88.3415	134.2614	88.26955	135.5817	88.40153	138.1766
88.34797	109.0432	88.26872	133.7066	88.2516	136.8819	88.35817	134.2588	88.28622	135.5804	88.4182	138.1768
88.36463	109.0449	88.28538	133.7056	88.26827	136.8771	88.37483	134.2595	88.30288	135.5826	88.43487	138.1772
88.3813	109.0453	88.30205	133.7046	88.28493	136.881	88.3915	134.2565	88.31955	135.5807	88.45153	138.1743
88.39797	109.0401	88.31872	133.7063	88.3016	136.8773	88.40817	134.2574	88.33622	135.5787	88.4682	138.1748
88.41463	109.0356	88.33538	133.7048	88.31827	136.8751	88.42483	134.2549	88.35288	135.5777	88.48487	138.1725
88.4313	109.0372	88.35205	133.7053	88.33493	136.8763	88.4415	134.2555	88.36955	135.5789	88.50153	138.1748
88.44797	109.0354	88.36872	133.7076	88.3516	136.8749	88.45817	134.2516	88.38622	135.5766	88.5182	138.1719
88.46463	109.0377	88.38538	133.7064	88.36827	136.871	88.47483	134.2509	88.40288	135.5775	88.53487	138.1736
88.4813	109.0335	88.40205	133.708	88.38493	136.8693	88.4915	134.2499	88.41955	135.5746	88.55153	138.1708
88.49797	109.0375	88.41872	133.7088	88.4016	136.8648	88.50817	134.2496	88.43622	135.5766	88.5682	138.1706
88.51463	109.0312	88.43538	133.7097	88.41827	136.8681	88.52483	134.2462	88.45288	135.5754	88.58487	138.1708
88.5313	109.0307	88.45205	133.7108	88.43493	136.8659	88.5415	134.2469	88.46955	135.5742	88.60153	138.169
88.54797	109.0298	88.46872	133.7099	88.4516	136.8648	88.55817	134.245	88.48622	135.5722	88.6182	138.1668
88.56463	109.0328	88.48538	133.7131	88.46827	136.8654	88.57483	134.2434	88.50288	135.573	88.63487	138.1672
88.5813	109.0294	88.50205	133.7122	88.48493	136.8615	88.5915	134.2419	88.51955	135.5736	88.65153	138.1652
88.59797	109.0328	88.51872	133.7102	88.5016	136.8571	88.60817	134.2424	88.53622	135.5717	88.6682	138.1647
88.61463	109.0254	88.53538	133.7153	88.51827	136.8586	88.62483	134.2391	88.55288	135.5714	88.68487	138.165
88.6313	109.0246	88.55205	133.7165	88.53493	136.8537	88.6415	134.2418	88.56955	135.5718	88.70153	138.1658
88.64797	109.0226	88.56872	133.7178	88.5516	136.8565	88.65817	134.2414	88.58622	135.5709	88.7182	138.1615
88.66463	109.0256	88.58538	133.7117	88.56827	136.8522	88.67483	134.2392	88.60288	135.5722	88.73487	138.1617
88.6813	109.0231	88.60205	133.7185	88.58493	136.8528	88.6915	134.2372	88.61955	135.5705	88.75153	138.1609
88.69797	109.021	88.61872	133.716	88.6016	136.8518	88.70817	134.2376	88.63622	135.5695	88.7682	138.1594
88.71463	109.0197	88.63538	133.7158	88.61827	136.8501	88.72483	134.2364	88.65288	135.569	88.78487	138.1603
88.7313	109.0184	88.65205	133.7146	88.63493	136.8508	88.7415	134.2357	88.66955	135.5691	88.80153	138.1612
88.74797	109.0184	88.66872	133.714	88.6516	136.8481	88.75817	134.2336	88.68622	135.5689	88.8182	138.1592
88.76463	109.0129	88.68538	133.7154	88.66827	136.8466	88.77483	134.235	88.70288	135.5679	88.83487	138.1589
88.7813	109.0152	88.70205	133.7155	88.68493	136.8443	88.7915	134.2328	88.71955	135.5672	88.85153	138.1581
88.79797	109.017	88.71872	133.7172	88.7016	136.8448	88.80817	134.2323	88.73622	135.5688	88.8682	138.1576
88.81463	109.0141	88.73538	133.7149	88.71827	136.8426	88.82483	134.2287	88.75288	135.5652	88.88487	138.155
88.8313	109.0129	88.75205	133.7145	88.73493	136.8425	88.8415	134.2262	88.76955	135.5634	88.90153	138.1565
88.84797	109.0151	88.76872	133.7155	88.7516	136.8386	88.85817	134.2275	88.78622	135.562	88.9182	138.1529
88.86463	109.011	88.78538	133.7136	88.76827	136.8395	88.87483	134.2275	88.80288	135.5643	88.93487	138.1529
88.8813	109.0065	88.80205	133.714	88.78493	136.8372	88.8915	134.2249	88.81955	135.5622	88.95153	138.1518
88.89797	109.0056	88.81872	133.7158	88.8016	136.8367	88.90817	134.2227	88.83622	135.5616	88.9682	138.1553
88.91463	109.0118	88.83538	133.7141	88.81827	136.8324	88.92483	134.2225	88.85288	135.562	88.98487	138.1509
88.9313	109.0035	88.85205	133.716	88.83493	136.8318	88.9415	134.2232	88.86955	135.5608	89.00153	138.1495
88.94797	109.0033	88.86872	133.713	88.8516	136.8305	88.95817	134.2209	88.88622	135.5608	89.0182	138.1489
88.96463	109.0057	88.88538	133.7139	88.86827	136.8284	88.97483	134.2184	88.90288	135.5625	89.03487	138.1509

88.9813	109.0063	88.90205	133.714	88.88493	136.8264	88.9915	134.2192	88.91955	135.5569	89.05153	138.1487
88.99797	109.0034	88.91872	133.7151	88.9016	136.8265	89.00817	134.2175	88.93622	135.5573	89.0682	138.1472
89.01463	109.004	88.93538	133.7127	88.91827	136.8224	89.02483	134.2137	88.95288	135.556	89.08487	138.1464
89.0313	108.9991	88.95205	133.714	88.93493	136.8195	89.0415	134.2115	88.96955	135.5558	89.10153	138.1451
89.04797	108.9985	88.96872	133.7136	88.9516	136.8178	89.05817	134.211	88.98622	135.5529	89.1182	138.1467
89.06463	108.9988	88.98538	133.7138	88.96827	136.8167	89.07483	134.2118	89.00288	135.5517	89.13487	138.142
89.0813	108.9999	89.00205	133.7125	88.98493	136.8124	89.0915	134.2066	89.01955	135.5482	89.15153	138.1439
89.09797	108.9979	89.01872	133.7124	89.0016	136.814	89.10817	134.2086	89.03622	135.5499	89.1682	138.1426
89.11463	108.9962	89.03538	133.714	89.01827	136.8113	89.12483	134.2071	89.05288	135.5501	89.18487	138.1401
89.1313	108.9995	89.05205	133.7139	89.03493	136.8075	89.1415	134.2065	89.06955	135.5446	89.20153	138.1401
89.14797	108.9931	89.06872	133.7119	89.0516	136.8094	89.15817	134.2037	89.08622	135.5468	89.2182	138.1421
89.16463	108.9931	89.08538	133.7128	89.06827	136.8059	89.17483	134.2014	89.10288	135.5451	89.23487	138.1403
89.1813	108.9944	89.10205	133.713	89.08493	136.8042	89.1915	134.1998	89.11955	135.5433	89.25153	138.1376
89.19797	108.9943	89.11872	133.7132	89.1016	136.8026	89.20817	134.1994	89.13622	135.544	89.2682	138.14
89.21463	108.9912	89.13538	133.7126	89.11827	136.8029	89.22483	134.1995	89.15288	135.5415	89.28487	138.138
89.2313	108.9889	89.15205	133.711	89.13493	136.8012	89.2415	134.1958	89.16955	135.5403	89.30153	138.1386
89.24797	108.9901	89.16872	133.713	89.1516	136.7973	89.25817	134.1949	89.18622	135.5386	89.3182	138.1388
89.26463	108.9876	89.18538	133.7111	89.16827	136.7966	89.27483	134.1962	89.20288	135.5418	89.33487	138.1359
89.2813	108.9844	89.20205	133.7129	89.18493	136.7969	89.2915	134.1917	89.21955	135.5376	89.35153	138.1356
89.29797	108.9878	89.21872	133.7106	89.2016	136.7938	89.30817	134.1926	89.23622	135.5373	89.3682	138.1354
89.31463	108.9854	89.23538	133.713	89.21827	136.7918	89.32483	134.1917	89.25288	135.5362	89.38487	138.1343
89.3313	108.9867	89.25205	133.7119	89.23493	136.7938	89.3415	134.1906	89.26955	135.5366	89.40153	138.134
89.34797	108.9812	89.26872	133.7115	89.2516	136.7888	89.35817	134.1889	89.28622	135.5332	89.4182	138.132
89.36463	108.9836	89.28538	133.7091	89.26827	136.7888	89.37483	134.1874	89.30288	135.5342	89.43487	138.1329
89.3813	108.98	89.30205	133.7088	89.28493	136.787	89.3915	134.1878	89.31955	135.5327	89.45153	138.1302
89.39797	108.978	89.31872	133.71	89.3016	136.7865	89.40817	134.1858	89.33622	135.5302	89.4682	138.13
89.41463	108.978	89.33538	133.7094	89.31827	136.7825	89.42483	134.1823	89.35288	135.53	89.48487	138.1294
89.4313	108.9806	89.35205	133.7089	89.33493	136.7817	89.4415	134.1836	89.36955	135.5296	89.50153	138.1292
89.44797	108.9748	89.36872	133.71	89.3516	136.7792	89.45817	134.1789	89.38622	135.5266	89.5182	138.1272
89.46463	108.9765	89.38538	133.7085	89.36827	136.7797	89.47483	134.18	89.40288	135.5261	89.53487	138.1266
89.4813	108.977	89.40205	133.7065	89.38493	136.7787	89.4915	134.1784	89.41955	135.5283	89.55153	138.1273
89.49797	108.9721	89.41872	133.7097	89.4016	136.7744	89.50817	134.177	89.43622	135.526	89.5682	138.1267
89.51463	108.9731	89.43538	133.7077	89.41827	136.7752	89.52483	134.1765	89.45288	135.5265	89.58487	138.1258
89.5313	108.972	89.45205	133.7059	89.43493	136.7761	89.5415	134.1724	89.46955	135.5242	89.60153	138.1263
89.54797	108.9709	89.46872	133.7059	89.4516	136.7738	89.55817	134.1734	89.48622	135.5228	89.6182	138.1262
89.56463	108.9687	89.48538	133.707	89.46827	136.7697	89.57483	134.1721	89.50288	135.5265	89.63487	138.1252
89.5813	108.9676	89.50205	133.7057	89.48493	136.768	89.5915	134.1698	89.51955	135.5208	89.65153	138.1235
89.59797	108.9687	89.51872	133.7083	89.5016	136.768	89.60817	134.1708	89.53622	135.5254	89.6682	138.1217
89.61463	108.9658	89.53538	133.7082	89.51827	136.7651	89.62483	134.1682	89.55288	135.519	89.68487	138.121
89.6313	108.9666	89.55205	133.7068	89.53493	136.7642	89.6415	134.1663	89.56955	135.5204	89.70153	138.1207
89.64797	108.9635	89.56872	133.7059	89.5516	136.762	89.65817	134.1663	89.58622	135.5184	89.7182	138.1202
89.66463	108.9627	89.58538	133.708	89.56827	136.7623	89.67483	134.1645	89.60288	135.5216	89.73487	138.1205
89.6813	108.9606	89.60205	133.7073	89.58493	136.7576	89.6915	134.1621	89.61955	135.5191	89.75153	138.1195
89.69797	108.9599	89.61872	133.7102	89.6016	136.7565	89.70817	134.1627	89.63622	135.5177	89.7682	138.1184
89.71463	108.9595	89.63538	133.7085	89.61827	136.7555	89.72483	134.162	89.65288	135.5193	89.78487	138.1166
89.7313	108.9585	89.65205	133.7084	89.63493	136.7546	89.7415	134.1598	89.66955	135.5183	89.80153	138.1175
89.74797	108.9573	89.66872	133.7087	89.6516	136.7532	89.75817	134.1585	89.68622	135.5182	89.8182	138.1159
89.76463	108.9542	89.68538	133.71	89.66827	136.7518	89.77483	134.1587	89.70288	135.5161	89.83487	138.115
89.7813	108.9544	89.70205	133.708	89.68493	136.7501	89.7915	134.1573	89.71955	135.5166	89.85153	138.1141
89.79797	108.9537	89.71872	133.7101	89.7016	136.7475	89.80817	134.1549	89.73622	135.5158	89.8682	138.1145
89.81463	108.9517	89.73538	133.7086	89.71827	136.7446	89.82483	134.1529	89.75288	135.5136	89.88487	138.1125
89.8313	108.9499	89.75205	133.709	89.73493	136.749	89.8415	134.153	89.76955	135.5142	89.90153	138.1118
89.84797	108.9503	89.76872	133.71	89.7516	136.7445	89.85817	134.1523	89.78622	135.5121	89.9182	138.1101
89.86463	108.949	89.78538	133.7116	89.76827	136.7436	89.87483	134.1516	89.80288	135.5146	89.93487	138.1109
89.8813	108.9495	89.80205	133.712	89.78493	136.7407	89.8915	134.1484	89.81955	135.5132	89.95153	138.1099
89.89797	108.9456	89.81872	133.7122	89.8016	136.7422	89.90817	134.1491	89.83622	135.514	89.9682	138.1094
89.91463	108.9474	89.83538	133.7114	89.81827	136.7371	89.92483	134.149	89.85288	135.5118	89.98487	138.1062
89.9313	108.9444	89.85205	133.7125	89.83493	136.7369	89.9415	134.1462	89.86955	135.5107	90.00153	138.1049
89.94797	108.9415	89.86872	133.7127	89.8516	136.7359	89.95817	134.1458	89.88622	135.5095	90.0182	138.105
89.96463	108.9399	89.88538	133.7146	89.86827	136.7342	89.97483	134.145	89.90288	135.5119	90.03487	138.1033
89.9813	108.9404	89.90205	133.715	89.88493	136.7326	89.9915	134.1426	89.91955	135.5107	90.05153	138.1018
89.99797	108.9432	89.91872	133.717	89.9016	136.7326	90.00817	134.1404	89.93622	135.5094	90.0682	138.1018
90.01463	108.9388	89.93538	133.7177	89.91827	136.7276	90.02483	134.1417	89.95288	135.5102	90.08487	138.1013
90.0313	108.9392	89.95205	133.7172	89.93493	136.7268	90.0415	134.1391	89.96955	135.5099	90.10153	138.0991
90.04797	108.9352	89.96872	133.7198	89.9516	136.7262	90.05817	134.1374	89.98622	135.508	90.1182	138.0992
90.06463	108.9364	89.98538	133.7167	89.96827	136.7252	90.07483	134.1361	90.00288	135.5095	90.13487	138.1005
90.0813	108.9353	90.00205	133.7188	89.98493	136.7225	90.0915	134.1347	90.01955	135.507	90.15153	138.0959
90.09797	108.9372	90.01872	133.7168	90.0016	136.7194	90.10817	134.1351	90.03622	135.5085	90.1682	138.0956
90.11463	108.9364	90.03538	133.719	90.01827	136.7192	90.12483	134.1323	90.05288	135.5066	90.18487	138.0962
90.1313	108.931	90.05205	133.718	90.03493	136.7159	90.1415	134.1323	90.06955	135.5071	90.20153	138.0966
90.14797	108.9312	90.06872	133.7184	90.0516	136.7162	90.15817	134.1302	90.08622	135.5059	90.2182	138.0923
90.16463	108.932	90.08538	133.7195	90.06827	136.7137	90.17483	134.1311	90.10288	135.5056	90.23487	138.0944
90.1813	108.9319	90.10205	133.7184	90.08493	136.7162	90.1915	134.1287	90.11955	135.5018	90.25153	138.0924
90.19797	108.9264	90.11872	133.7191	90.1016	136.7112	90.20817	134.1285	90.13622	135.5018	90.2682	138.0925
90.21463	108.9294	90.13538	133.7187	90.11827	136.7097	90.22483	134.129	90.15288	135.5035	90.28487	138.0912
90.2313	108.9298	90.15205	133.722	90.13493	136.7078	90.2415	134.1272	90.16955	135.5006	90.30153	138.091
90.24797	108.9237	90.16872	133.719	90.1516	136.7047	90.25817	134.1249	90.18622	135.5008	90.3182	138.089
90.26463	108.9249	90.18538	133.7217	90.16827	136.7045	90.27483	134.1235	90.20288	135.5004	90.33487	138.0889
90.2813	108.9238	90.20205	133.7193	90.18493	136.7038	90.2915	134.123	90.21955	135.4974	90.35153	138.0879
90.29797	108.9211	90.21872	133.7237	90.2016	136.7021	90.30817	134.1207	90.23622	135.4975	90.3682	138.0863
90.31463	108.9189	90.23538	133.7215	90.21827	136.702	90.32483	134.1188	90.25288	135.4954	90.38487	138.0868
90.3313	108.921	90.25205	133.7231	90.23493	136.6994	90.3415	134.1208	90.26955	135.497	90.40153	138.0852
90.34797	108.919	90.26872	133.7211	90.2516	136.6966	90.35817	134.1169	90.28622	135.4937	90.4182	138.0848
90.36463	108.9155	90.28538	133.721	90.26827	136.6963	90.37483	134.1158	90.30288	135.496	90.43487	138.0865
90.3813	108.9153	90.30205	133.7222	90.28493	136.6931	90.3915	134.1147	90.31955	135.492	90.45153	138.0831
90.39797	108.9162	90.31872	133.7239	90.3016	136.6901	90.40817	134.1148	90.33622	135.4912	90.4682	138.0826
90.41463	108.9113	90.33538	133.7239	90.31827	136.6917	90.42483	134.1116	90.35288	135.4922	90.48487	138.0839
90.4313	108.9141	90.35205	133.7237	90.33493	136.6905	90.4415	134.1147	90.36955	135.4914	90.50153	138.0808
90.44797	108.9099	90.36872	133.7242	90.3516	136.6869	90.45817	134.1087	90.38622	135.4911	90.5182	138.0809
90.46463	108.9103	90.38538	133.7255	90.36827	136.6862	90.47483	134.1091	90.40288	135.4875	90.53487	138.0815
90.4813	108.9077	90.40205	133.7244	90.38493	136.6871	90.4915	134.1078	90.41955	135.4892	90.55153	138.0789
90.49797	108.9082	90.41872	133.7239	90.4016	136.6839	90.50817	134.1073	90.43622	135.4868	90.5682	138.0786
90.51463	108.9058	90.43538	133.7238	90.41827	136.6813	90.52483	134.1046	90.45288	135.4879	90.58487	138.0778
90.5313	108.9052	90.45205	133.7268	90.43493	136.6805	90.5415	134.1061	90.46955	135.4865	90.60153	138.0779
90.54797	108.9068	90.46872	133.7264	90.4516	136.677	90.55817	134.104	90.48622	135.4856	90.6182	138.0775
90.56463	108.905	90.48538	133.7251	90.46827	136.6808	90.57483	134.1018	90.50288	135.4831	90.63487	138.0776
90.5813	108.9019	90.50205	133.7258	90.48493	136.6753	90.5915	134.1007	90.51955	135.4816	90.65153	138.0766

90.59797	108.9035	90.51872	133.7257	90.5016	136.6735	90.60817	134.1016	90.53622	135.4845	90.6682	138.0728
90.61463	108.9007	90.53538	133.7248	90.51827	136.6732	90.62483	134.099	90.55288	135.48	90.68487	138.0734
90.6313	108.9005	90.55205	133.7294	90.53493	136.6697	90.6415	134.0994	90.56955	135.4819	90.70153	138.0727
90.64797	108.9006	90.56872	133.7279	90.5516	136.6683	90.65817	134.0973	90.58622	135.4789	90.7182	138.0712
90.66463	108.898	90.58538	133.7281	90.56827	136.6654	90.67483	134.0959	90.60288	135.4799	90.73487	138.0723
90.6813	108.8971	90.60205	133.7276	90.58493	136.6675	90.6915	134.0944	90.61955	135.4784	90.75153	138.072
90.69797	108.8974	90.61872	133.7292	90.6016	136.6644	90.70817	134.0943	90.63622	135.4787	90.7682	138.0713
90.71463	108.8966	90.63538	133.7284	90.61827	136.6627	90.72483	134.0915	90.65288	135.4745	90.78487	138.0703
90.7313	108.8944	90.65205	133.7321	90.63493	136.6611	90.7415	134.0907	90.66955	135.4775	90.80153	138.0709
90.74797	108.8951	90.66872	133.729	90.6516	136.6609	90.75817	134.0884	90.68622	135.4765	90.8182	138.0689
90.76463	108.896	90.68538	133.7321	90.66827	136.6559	90.77483	134.0897	90.70288	135.4745	90.83487	138.068
90.7813	108.8916	90.70205	133.7303	90.68493	136.657	90.7915	134.0854	90.71955	135.4741	90.85153	138.0692
90.79797	108.8928	90.71872	133.7296	90.7016	136.6545	90.80817	134.0868	90.73622	135.4711	90.8682	138.0682
90.81463	108.8927	90.73538	133.7289	90.71827	136.6542	90.82483	134.0843	90.75288	135.4724	90.88487	138.066
90.8313	108.8908	90.75205	133.7309	90.73493	136.6517	90.8415	134.0819	90.76955	135.4696	90.90153	138.065
90.84797	108.8916	90.76872	133.7301	90.7516	136.6496	90.85817	134.0815	90.78622	135.4707	90.9182	138.0648
90.86463	108.8904	90.78538	133.7294	90.76827	136.6483	90.87483	134.0848	90.80288	135.4698	90.93487	138.0643
90.8813	108.891	90.80205	133.7277	90.78493	136.6486	90.8915	134.0827	90.81955	135.469	90.95153	138.0634
90.89797	108.8898	90.81872	133.7285	90.8016	136.646	90.90817	134.0785	90.83622	135.4698	90.9682	138.0609
90.91463	108.8873	90.83538	133.7299	90.81827	136.6446	90.92483	134.0796	90.85288	135.4669	90.98487	138.0605
90.9313	108.8863	90.85205	133.7284	90.83493	136.6428	90.9415	134.0768	90.86955	135.4658	91.00153	138.0597
90.94797	108.8894	90.86872	133.7287	90.8516	136.6417	90.95817	134.0768	90.88622	135.4634	91.0182	138.0568
90.96463	108.8879	90.88538	133.7336	90.86827	136.6404	90.97483	134.077	90.90288	135.4642	91.03487	138.0592
90.9813	108.884	90.90205	133.7273	90.88493	136.6397	90.9915	134.0745	90.91955	135.4626	91.05153	138.0558
90.99797	108.8865	90.91872	133.7319	90.9016	136.6382	91.00817	134.0705	90.93622	135.4616	91.0682	138.0561
91.01463	108.8826	90.93538	133.7295	90.91827	136.6339	91.02483	134.0691	90.95288	135.4603	91.08487	138.0568
91.0313	108.8845	90.95205	133.7326	90.93493	136.634	91.0415	134.0678	90.96955	135.4582	91.10153	138.0532
91.04797	108.8846	90.96872	133.7282	90.9516	136.6333	91.05817	134.0675	90.98622	135.4591	91.1182	138.054
91.06463	108.8849	90.98538	133.7327	90.96827	136.6301	91.07483	134.0651	91.00288	135.4577	91.13487	138.0523
91.0813	108.8817	91.00205	133.7296	90.98493	136.6323	91.0915	134.0634	91.01955	135.4573	91.15153	138.0511
91.09797	108.8824	91.01872	133.7303	91.0016	136.6308	91.10817	134.0628	91.03622	135.457	91.1682	138.0513
91.11463	108.8788	91.03538	133.7321	91.01827	136.6266	91.12483	134.0646	91.05288	135.4578	91.18487	138.0482
91.1313	108.8809	91.05205	133.732	91.03493	136.6249	91.1415	134.0591	91.06955	135.4558	91.20153	138.0464
91.14797	108.8789	91.06872	133.7336	91.0516	136.626	91.15817	134.0593	91.08622	135.456	91.2182	138.0479
91.16463	108.8782	91.08538	133.7284	91.06827	136.6249	91.17483	134.0575	91.10288	135.4555	91.23487	138.0472
91.1813	108.8757	91.10205	133.7289	91.08493	136.6228	91.1915	134.0555	91.11955	135.4535	91.25153	138.0442
91.19797	108.8784	91.11872	133.731	91.1016	136.6207	91.20817	134.0544	91.13622	135.4552	91.2682	138.0442
91.21463	108.8754	91.13538	133.7309	91.11827	136.6195	91.22483	134.0537	91.15288	135.4527	91.28487	138.0414
91.2313	108.875	91.15205	133.7316	91.13493	136.6173	91.2415	134.0534	91.16955	135.4513	91.30153	138.0406
91.24797	108.877	91.16872	133.7294	91.1516	136.6167	91.25817	134.05	91.18622	135.4509	91.3182	138.0399
91.26463	108.8757	91.18538	133.7287	91.16827	136.6127	91.27483	134.0474	91.20288	135.4502	91.33487	138.0413
91.2813	108.8751	91.20205	133.7298	91.18493	136.6149	91.2915	134.0483	91.21955	135.4503	91.35153	138.039
91.29797	108.8713	91.21872	133.729	91.2016	136.6137	91.30817	134.0458	91.23622	135.4465	91.3682	138.0373
91.31463	108.8722	91.23538	133.7307	91.21827	136.6102	91.32483	134.0429	91.25288	135.4462	91.38487	138.0402
91.3313	108.8738	91.25205	133.7298	91.23493	136.6084	91.3415	134.043	91.26955	135.4454	91.40153	138.0385
91.34797	108.8686	91.26872	133.729	91.2516	136.6052	91.35817	134.04	91.28622	135.4445	91.4182	138.0362
91.36463	108.8692	91.28538	133.7331	91.26827	136.6084	91.37483	134.0392	91.30288	135.4439	91.43487	138.0354
91.3813	108.867	91.30205	133.7303	91.28493	136.6043	91.3915	134.0412	91.31955	135.4432	91.45153	138.0331
91.39797	108.8669	91.31872	133.7307	91.3016	136.6	91.40817	134.039	91.33622	135.4427	91.4682	138.0324
91.41463	108.866	91.33538	133.7259	91.31827	136.6001	91.42483	134.0364	91.35288	135.4429	91.48487	138.033
91.4313	108.8613	91.35205	133.7291	91.33493	136.5986	91.4415	134.0358	91.36955	135.4414	91.50153	138.033
91.44797	108.863	91.36872	133.7288	91.3516	136.5974	91.45817	134.0359	91.38622	135.4403	91.5182	138.0305
91.46463	108.8646	91.38538	133.7298	91.36827	136.5939	91.47483	134.0316	91.40288	135.4395	91.53487	138.0324
91.4813	108.8617	91.40205	133.7281	91.38493	136.5941	91.4915	134.0314	91.41955	135.4389	91.55153	138.0303
91.49797	108.8666	91.41872	133.728	91.4016	136.5919	91.50817	134.0292	91.43622	135.4384	91.5682	138.0306
91.51463	108.862	91.43538	133.7293	91.41827	136.591	91.52483	134.0275	91.45288	135.4373	91.58487	138.0286
91.5313	108.8591	91.45205	133.7288	91.43493	136.5888	91.5415	134.0258	91.46955	135.4369	91.60153	138.0264
91.54797	108.8573	91.46872	133.7266	91.4516	136.5857	91.55817	134.0257	91.48622	135.4384	91.6182	138.0255
91.56463	108.8609	91.48538	133.7303	91.46827	136.5866	91.57483	134.0238	91.50288	135.4363	91.63487	138.0259
91.5813	108.8552	91.50205	133.7257	91.48493	136.5851	91.5915	134.0219	91.51955	135.4349	91.65153	138.0243
91.59797	108.8549	91.51872	133.7296	91.5016	136.5841	91.60817	134.0217	91.53622	135.4344	91.6682	138.0242
91.61463	108.8538	91.53538	133.7287	91.51827	136.5797	91.62483	134.0197	91.55288	135.4341	91.68487	138.0216
91.6313	108.8503	91.55205	133.7268	91.53493	136.579	91.6415	134.0183	91.56955	135.4366	91.70153	138.0225
91.64797	108.8521	91.56872	133.7283	91.5516	136.5795	91.65817	134.0176	91.58622	135.4341	91.7182	138.0191
91.66463	108.8536	91.58538	133.7305	91.56827	136.5772	91.67483	134.016	91.60288	135.4322	91.73487	138.0187
91.6813	108.851	91.60205	133.727	91.58493	136.5747	91.6915	134.0139	91.61955	135.4305	91.75153	138.0184
91.69797	108.8488	91.61872	133.7299	91.6016	136.5751	91.70817	134.0122	91.63622	135.4338	91.7682	138.0191
91.71463	108.8483	91.63538	133.7293	91.61827	136.5744	91.72483	134.0122	91.65288	135.4331	91.78487	138.0168
91.7313	108.8486	91.65205	133.7309	91.63493	136.5693	91.7415	134.011	91.66955	135.4298	91.80153	138.0167
91.74797	108.8456	91.66872	133.7302	91.6516	136.5681	91.75817	134.0088	91.68622	135.4291	91.8182	138.0143
91.76463	108.8477	91.68538	133.7339	91.66827	136.5671	91.77483	134.008	91.70288	135.4288	91.83487	138.0161
91.7813	108.8445	91.70205	133.7313	91.68493	136.5646	91.7915	134.0075	91.71955	135.4273	91.85153	138.014
91.79797	108.8438	91.71872	133.7321	91.7016	136.5642	91.80817	134.0062	91.73622	135.4258	91.8682	138.014
91.81463	108.8441	91.73538	133.7314	91.71827	136.5639	91.82483	134.006	91.75288	135.4268	91.88487	138.0121
91.8313	108.8436	91.75205	133.7325	91.73493	136.5608	91.8415	134.0019	91.76955	135.4253	91.90153	138.0129
91.84797	108.8397	91.76872	133.7336	91.7516	136.5608	91.85817	134.0038	91.78622	135.4237	91.9182	138.0101
91.86463	108.84	91.78538	133.7336	91.76827	136.5621	91.87483	133.9996	91.80288	135.4261	91.93487	138.0083
91.8813	108.8385	91.80205	133.7338	91.78493	136.557	91.8915	134.0003	91.81955	135.4233	91.95153	138.0097
91.89797	108.8371	91.81872	133.7365	91.8016	136.5555	91.90817	133.9972	91.83622	135.4238	91.9682	138.0108
91.91463	108.8401	91.83538	133.7335	91.81827	136.5551	91.92483	133.9967	91.85288	135.4239	91.98487	138.007
91.9313	108.8374	91.85205	133.7351	91.83493	136.5544	91.9415	133.9943	91.86955	135.4199	92.00153	138.0089
91.94797	108.8363	91.86872	133.7348	91.8516	136.5557	91.95817	133.9945	91.88622	135.421	92.0182	138.004
91.96463	108.8361	91.88538	133.7369	91.86827	136.5517	91.97483	133.9939	91.90288	135.4216	92.03487	138.0052
91.9813	108.834	91.90205	133.7354	91.88493	136.5489	91.9915	133.9925	91.91955	135.422	92.05153	138.0038
91.99797	108.8342	91.91872	133.7384	91.9016	136.5469	92.00817	133.9901	91.93622	135.4208	92.0682	138.0062
92.01463	108.833	91.93538	133.7369	91.91827	136.5448	92.02483	133.9908	91.95288	135.4198	92.08487	138.0027
92.0313	108.8313	91.95205	133.74	91.93493	136.5439	92.0415	133.9873	91.96955	135.4209	92.10153	138.0026
92.04797	108.8299	91.96872	133.7427	91.9516	136.5463	92.05817	133.9846	91.98622	135.4193	92.1182	138.0024
92.06463	108.8318	91.98538	133.7417	91.96827	136.5391	92.07483	133.9884	92.00288	135.4183	92.13487	137.9989
92.0813	108.8293	92.00205	133.7398	91.98493	136.5374	92.0915	133.9838	92.01955	135.4157	92.15153	137.9994
92.09797	108.8268	92.01872	133.7431	92.0016	136.5369	92.10817	133.9836	92.03622	135.4187	92.1682	137.9985
92.11463	108.8274	92.03538	133.7455	92.01827	136.5366	92.12483	133.9826	92.05288	135.4175	92.18487	137.9993
92.1313	108.8267	92.05205	133.746	92.03493	136.5334	92.1415	133.9814	92.06955	135.4173	92.20153	137.9975
92.14797	108.8266	92.06872	133.7424	92.0516	136.5336	92.15817	133.9802	92.08622	135.4144	92.2182	137.997
92.16463	108.825	92.08538	133.7475	92.06827	136.5345	92.17483	133.9802	92.10288	135.4154	92.23487	137.9981
92.1813	108.8233	92.10205	133.7465	92.08493	136.5286	92.1915	133.9766	92.11955	135.417	92.25153	137.9947
92.19797	108.8217	92.11872	133.7465	92.1016	136.5319	92.20817	133.9783	92.13622	135.4154	92.2682	137.9955

92.21463	108.8206	92.13538	133.7476	92.11827	136.5269	92.22483	133.9775	92.15288	135.4143	92.28487	137.9938
92.2313	108.8206	92.15205	133.7481	92.13493	136.5287	92.2415	133.9754	92.16955	135.4132	92.30153	137.9934
92.24797	108.8166	92.16872	133.7492	92.1516	136.5255	92.25817	133.9738	92.18622	135.4132	92.3182	137.9941
92.26463	108.8168	92.18538	133.7512	92.16827	136.5258	92.27483	133.9731	92.20288	135.4137	92.33487	137.9928
92.2813	108.8169	92.20205	133.7491	92.18493	136.5228	92.2915	133.9745	92.21955	135.4121	92.35153	137.9899
92.29797	108.8141	92.21872	133.7486	92.2016	136.5205	92.30817	133.9712	92.23622	135.4114	92.3682	137.9908
92.31463	108.8118	92.23538	133.7514	92.21827	136.5208	92.32483	133.9712	92.25288	135.4114	92.38487	137.9909
92.3313	108.8146	92.25205	133.7529	92.23493	136.5196	92.3415	133.9711	92.26955	135.4114	92.40153	137.9882
92.34797	108.8108	92.26872	133.7515	92.2516	136.5189	92.35817	133.9712	92.28622	135.409	92.4182	137.9877
92.36463	108.8113	92.28538	133.7508	92.26827	136.5182	92.37483	133.9689	92.30288	135.4077	92.43487	137.9866
92.3813	108.8097	92.30205	133.7534	92.28493	136.5186	92.3915	133.9672	92.31955	135.4093	92.45153	137.9853
92.39797	108.8089	92.31872	133.7578	92.3016	136.5128	92.40817	133.969	92.33622	135.4085	92.4682	137.9846
92.41463	108.8084	92.33538	133.7546	92.31827	136.5147	92.42483	133.967	92.35288	135.4056	92.48487	137.9838
92.4313	108.8084	92.35205	133.7551	92.33493	136.5132	92.4415	133.9656	92.36955	135.4049	92.50153	137.9836
92.44797	108.8085	92.36872	133.7567	92.3516	136.5094	92.45817	133.9641	92.38622	135.4069	92.5182	137.9822
92.46463	108.8068	92.38538	133.7572	92.36827	136.5084	92.47483	133.9624	92.40288	135.4054	92.53487	137.9845
92.4813	108.8058	92.40205	133.7575	92.38493	136.5115	92.4915	133.9623	92.41955	135.4068	92.55153	137.9795
92.49797	108.8057	92.41872	133.7538	92.4016	136.5071	92.50817	133.9607	92.43622	135.4044	92.5682	137.9809
92.51463	108.8057	92.43538	133.7525	92.41827	136.5062	92.52483	133.96	92.45288	135.4034	92.58487	137.9774
92.5313	108.8053	92.45205	133.7575	92.43493	136.5035	92.5415	133.958	92.46955	135.4035	92.60153	137.9795
92.54797	108.8041	92.46872	133.7555	92.4516	136.5038	92.55817	133.9582	92.48622	135.4023	92.6182	137.9759
92.56463	108.803	92.48538	133.755	92.46827	136.5028	92.57483	133.9552	92.50288	135.4024	92.63487	137.9763
92.5813	108.8017	92.50205	133.7567	92.48493	136.5	92.5915	133.9533	92.51955	135.4011	92.65153	137.9768
92.59797	108.8	92.51872	133.7585	92.5016	136.4999	92.60817	133.9531	92.53622	135.4018	92.6682	137.9765
92.61463	108.8012	92.53538	133.7576	92.51827	136.5035	92.62483	133.9517	92.55288	135.3988	92.68487	137.9735
92.6313	108.8003	92.55205	133.7553	92.53493	136.498	92.6415	133.9479	92.56955	135.3977	92.70153	137.9727
92.64797	108.7986	92.56872	133.7563	92.5516	136.4969	92.65817	133.9479	92.58622	135.3982	92.7182	137.9728
92.66463	108.8002	92.58538	133.7582	92.56827	136.4947	92.67483	133.9455	92.60288	135.3961	92.73487	137.9746
92.6813	108.7981	92.60205	133.7558	92.58493	136.494	92.6915	133.9421	92.61955	135.3948	92.75153	137.9716
92.69797	108.7936	92.61872	133.7596	92.6016	136.4951	92.70817	133.9415	92.63622	135.3941	92.7682	137.9714
92.71463	108.7977	92.63538	133.7582	92.61827	136.491	92.72483	133.9384	92.65288	135.3941	92.78487	137.9694
92.7313	108.7965	92.65205	133.7604	92.63493	136.4892	92.7415	133.939	92.66955	135.3933	92.80153	137.9707
92.74797	108.7922	92.66872	133.76	92.6516	136.491	92.75817	133.9365	92.68622	135.3926	92.8182	137.9675
92.76463	108.7933	92.68538	133.7623	92.66827	136.4878	92.77483	133.9353	92.70288	135.3918	92.83487	137.9684
92.7813	108.794	92.70205	133.7595	92.68493	136.4835	92.7915	133.9345	92.71955	135.3911	92.85153	137.9667
92.79797	108.7908	92.71872	133.7626	92.7016	136.4804	92.80817	133.9331	92.73622	135.3876	92.8682	137.9675
92.81463	108.7914	92.73538	133.7607	92.71827	136.484	92.82483	133.9325	92.75288	135.3913	92.88487	137.9644
92.8313	108.7882	92.75205	133.7579	92.73493	136.4798	92.8415	133.9307	92.76955	135.3872	92.90153	137.9667
92.84797	108.7878	92.76872	133.7636	92.7516	136.4797	92.85817	133.9285	92.78622	135.3899	92.9182	137.9635
92.86463	108.7882	92.78538	133.7636	92.76827	136.4743	92.87483	133.9234	92.80288	135.3887	92.93487	137.9622
92.8813	108.7855	92.80205	133.7639	92.78493	136.4736	92.8915	133.925	92.81955	135.3862	92.95153	137.9611
92.89797	108.7873	92.81872	133.7601	92.8016	136.4743	92.90817	133.9224	92.83622	135.3856	92.9682	137.9632
92.91463	108.7847	92.83538	133.7578	92.81827	136.4726	92.92483	133.9205	92.85288	135.3872	92.98487	137.9609
92.9313	108.7849	92.85205	133.7648	92.83493	136.4727	92.9415	133.9208	92.86955	135.3867	93.00153	137.9603
92.94797	108.7814	92.86872	133.7635	92.8516	136.4702	92.95817	133.917	92.88622	135.3866	93.0182	137.9595
92.96463	108.782	92.88538	133.7656	92.86827	136.4678	92.97483	133.9159	92.90288	135.3845	93.03487	137.9604
92.9813	108.7813	92.90205	133.7632	92.88493	136.468	92.9915	133.9171	92.91955	135.3849	93.05153	137.9582
92.99797	108.7781	92.91872	133.7613	92.9016	136.4644	93.00817	133.9142	92.93622	135.3852	93.0682	137.9587
93.01463	108.7801	92.93538	133.7638	92.91827	136.4652	93.02483	133.9137	92.95288	135.3865	93.08487	137.9582
93.0313	108.7794	92.95205	133.7668	92.93493	136.4607	93.0415	133.9136	92.96955	135.3848	93.10153	137.9576
93.04797	108.7762	92.96872	133.7637	92.9516	136.4659	93.05817	133.9086	92.98622	135.3831	93.1182	137.9561
93.06463	108.7759	92.98538	133.7657	92.96827	136.4619	93.07483	133.9069	93.00288	135.3827	93.13487	137.9552
93.0813	108.778	93.00205	133.7665	92.98493	136.4571	93.0915	133.9086	93.01955	135.3846	93.15153	137.9533
93.09797	108.7753	93.01872	133.7653	93.0016	136.459	93.10817	133.9078	93.03622	135.3839	93.1682	137.9548
93.11463	108.7764	93.03538	133.765	93.01827	136.4568	93.12483	133.9021	93.05288	135.3824	93.18487	137.9528
93.1313	108.7754	93.05205	133.7653	93.03493	136.4542	93.1415	133.9049	93.06955	135.3825	93.20153	137.9525
93.14797	108.7722	93.06872	133.7667	93.0516	136.4534	93.15817	133.9019	93.08622	135.3808	93.2182	137.9501
93.16463	108.7728	93.08538	133.7688	93.06827	136.451	93.17483	133.8997	93.10288	135.3838	93.23487	137.9507
93.1813	108.7717	93.10205	133.768	93.08493	136.4504	93.1915	133.8981	93.11955	135.3811	93.25153	137.9487
93.19797	108.7706	93.11872	133.7692	93.1016	136.4504	93.20817	133.8988	93.13622	135.3811	93.2682	137.948
93.21463	108.7684	93.13538	133.7706	93.11827	136.4509	93.22483	133.8971	93.15288	135.376	93.28487	137.9468
93.2313	108.77	93.15205	133.7723	93.13493	136.445	93.2415	133.8969	93.16955	135.381	93.30153	137.9489
93.24797	108.7689	93.16872	133.773	93.1516	136.4429	93.25817	133.8932	93.18622	135.3789	93.3182	137.9481
93.26463	108.7637	93.18538	133.7737	93.16827	136.4425	93.27483	133.8918	93.20288	135.3796	93.33487	137.9504
93.2813	108.7618	93.20205	133.7731	93.18493	136.4426	93.2915	133.8928	93.21955	135.3767	93.35153	137.9445
93.29797	108.7657	93.21872	133.7749	93.2016	136.4408	93.30817	133.8902	93.23622	135.3789	93.3682	137.9468
93.31463	108.7649	93.23538	133.7749	93.21827	136.4406	93.32483	133.8883	93.25288	135.377	93.38487	137.945
93.3313	108.7606	93.25205	133.7731	93.23493	136.4376	93.3415	133.8877	93.26955	135.3761	93.40153	137.9475
93.34797	108.7646	93.26872	133.7704	93.2516	136.4367	93.35817	133.8872	93.28622	135.375	93.4182	137.9438
93.36463	108.7618	93.28538	133.7746	93.26827	136.4355	93.37483	133.8832	93.30288	135.3739	93.43487	137.9422
93.3813	108.7601	93.30205	133.7746	93.28493	136.4338	93.3915	133.8834	93.31955	135.3739	93.45153	137.9423
93.39797	108.7585	93.31872	133.7747	93.3016	136.4329	93.40817	133.8825	93.33622	135.3733	93.4682	137.94
93.41463	108.7568	93.33538	133.7712	93.31827	136.4302	93.42483	133.8806	93.35288	135.3712	93.48487	137.9371
93.4313	108.7563	93.35205	133.7688	93.33493	136.4306	93.4415	133.8793	93.36955	135.3695	93.50153	137.9402
93.44797	108.7561	93.36872	133.7696	93.3516	136.4286	93.45817	133.8828	93.38622	135.3691	93.5182	137.9399
93.46463	108.7554	93.38538	133.7682	93.36827	136.4261	93.47483	133.8765	93.40288	135.3677	93.53487	137.9414
93.4813	108.7538	93.40205	133.7709	93.38493	136.4257	93.4915	133.8754	93.41955	135.3667	93.55153	137.9383
93.49797	108.7518	93.41872	133.7722	93.4016	136.4228	93.50817	133.8746	93.43622	135.3663	93.5682	137.9361
93.51463	108.7521	93.43538	133.7737	93.41827	136.4233	93.52483	133.8742	93.45288	135.3665	93.58487	137.9359
93.5313	108.7503	93.45205	133.771	93.43493	136.4193	93.5415	133.8715	93.46955	135.3644	93.60153	137.9361
93.54797	108.7507	93.46872	133.7687	93.4516	136.4175	93.55817	133.8717	93.48622	135.3632	93.6182	137.9351
93.56463	108.7501	93.48538	133.7706	93.46827	136.4168	93.57483	133.8717	93.50288	135.3646	93.63487	137.9365
93.5813	108.7444	93.50205	133.7711	93.48493	136.4134	93.5915	133.8687	93.51955	135.3607	93.65153	137.9343
93.59797	108.7479	93.51872	133.7724	93.5016	136.4135	93.60817	133.8659	93.53622	135.3605	93.6682	137.9327
93.61463	108.7443	93.53538	133.7723	93.51827	136.4105	93.62483	133.8665	93.55288	135.3627	93.68487	137.9307
93.6313	108.7422	93.55205	133.7748	93.53493	136.4094	93.6415	133.8666	93.56955	135.3585	93.70153	137.9301
93.64797	108.7462	93.56872	133.776	93.5516	136.4077	93.65817	133.8631	93.58622	135.359	93.7182	137.9298
93.66463	108.7431	93.58538	133.7743	93.56827	136.4076	93.67483	133.8612	93.60288	135.358	93.73487	137.9311
93.6813	108.7406	93.60205	133.7746	93.58493	136.4071	93.6915	133.8595	93.61955	135.356	93.75153	137.9284
93.69797	108.7419	93.61872	133.7723	93.6016	136.405	93.70817	133.862	93.63622	135.3582	93.7682	137.9275
93.71463	108.7415	93.63538	133.7731	93.61827	136.4046	93.72483	133.8597	93.65288	135.3565	93.78487	137.9251
93.7313	108.739	93.65205	133.7737	93.63493	136.4014	93.7415	133.8572	93.66955	135.3564	93.80153	137.9261
93.74797	108.7401	93.66872	133.77	93.6516	136.402	93.75817	133.858	93.68622	135.3537	93.8182	137.9259
93.76463	108.7376	93.68538	133.771	93.66827	136.3986	93.77483	133.8543	93.70288	135.3534	93.83487	137.925
93.7813	108.7363	93.70205	133.7716	93.68493	136.3975	93.7915	133.854	93.71955	135.3528	93.85153	137.923
93.79797	108.7376	93.71872	133.7736	93.7016	136.3961	93.80817	133.854	93.73622	135.3519	93.8682	137.9248
93.81463	108.7381	93.73538	133.7729	93.71827	136.3962	93.82483	133.8523	93.75288	135.3496	93.88487	137.9209

93.8313	108.7334	93.75205	133.7752	93.73493	136.3931	93.8415	133.8511	93.76955	135.3505	93.90153	137.9212
93.84797	108.7317	93.76872	133.7729	93.7516	136.3928	93.85817	133.8505	93.78622	135.3465	93.9182	137.9193
93.86463	108.7336	93.78538	133.7741	93.76827	136.3892	93.87483	133.8496	93.80288	135.3465	93.93487	137.9211
93.8813	108.7311	93.80205	133.7746	93.78493	136.3878	93.8915	133.8474	93.81955	135.3476	93.95153	137.9179
93.89797	108.729	93.81872	133.775	93.8016	136.3887	93.90817	133.8473	93.83622	135.3463	93.9682	137.9202
93.91463	108.7317	93.83538	133.7739	93.81827	136.3865	93.92483	133.8458	93.85288	135.3449	93.98487	137.9174
93.9313	108.7302	93.85205	133.7748	93.83493	136.3876	93.9415	133.8447	93.86955	135.3448	94.00153	137.9147
93.94797	108.7279	93.86872	133.7721	93.8516	136.3863	93.95817	133.8445	93.88622	135.3427	94.0182	137.9177
93.96463	108.7269	93.88538	133.7729	93.86827	136.3843	93.97483	133.8395	93.90288	135.3437	94.03487	137.9125
93.9813	108.7252	93.90205	133.7752	93.88493	136.3842	93.9915	133.8412	93.91955	135.3415	94.05153	137.9123
93.99797	108.7237	93.91872	133.7741	93.9016	136.3796	94.00817	133.8378	93.93622	135.3407	94.0682	137.915
94.01463	108.7266	93.93538	133.7736	93.91827	136.3809	94.02483	133.8375	93.95288	135.3413	94.08487	137.9123
94.0313	108.7273	93.95205	133.7779	93.93493	136.3791	94.0415	133.8364	93.96955	135.3417	94.10153	137.9125
94.04797	108.7224	93.96872	133.7762	93.9516	136.378	94.05817	133.8345	93.98622	135.3418	94.1182	137.9099
94.06463	108.7218	93.98538	133.7747	93.96827	136.3764	94.07483	133.8343	94.00288	135.3399	94.13487	137.9104
94.0813	108.7224	94.00205	133.7749	93.98493	136.3753	94.0915	133.8336	94.01955	135.3388	94.15153	137.9087
94.09797	108.7188	94.01872	133.7747	94.0016	136.3736	94.10817	133.8338	94.03622	135.3397	94.1682	137.9067
94.11463	108.7214	94.03538	133.7731	94.01827	136.3734	94.12483	133.831	94.05288	135.3383	94.18487	137.9059
94.1313	108.7194	94.05205	133.7738	94.03493	136.3729	94.1415	133.8324	94.06955	135.3375	94.20153	137.9056
94.14797	108.7176	94.06872	133.7741	94.0516	136.3712	94.15817	133.8295	94.08622	135.3357	94.2182	137.905
94.16463	108.7188	94.08538	133.7771	94.06827	136.3677	94.17483	133.8277	94.10288	135.3357	94.23487	137.9041
94.1813	108.7181	94.10205	133.7768	94.08493	136.3698	94.1915	133.8288	94.11955	135.3348	94.25153	137.9036
94.19797	108.7171	94.11872	133.7752	94.1016	136.3705	94.20817	133.8253	94.13622	135.3346	94.2682	137.9031
94.21463	108.7149	94.13538	133.7767	94.11827	136.3664	94.22483	133.8256	94.15288	135.3339	94.28487	137.903
94.2313	108.7142	94.15205	133.7776	94.13493	136.3688	94.2415	133.8235	94.16955	135.3327	94.30153	137.9014
94.24797	108.7132	94.16872	133.779	94.1516	136.3636	94.25817	133.8229	94.18622	135.3325	94.3182	137.9023
94.26463	108.714	94.18538	133.779	94.16827	136.3631	94.27483	133.8202	94.20288	135.3311	94.33487	137.9032
94.2813	108.7097	94.20205	133.7803	94.18493	136.3596	94.2915	133.819	94.21955	135.3318	94.35153	137.8976
94.29797	108.7123	94.21872	133.7803	94.2016	136.3611	94.30817	133.8178	94.23622	135.3297	94.3682	137.9008
94.31463	108.7099	94.23538	133.7804	94.21827	136.3625	94.32483	133.8151	94.25288	135.3285	94.38487	137.897
94.3313	108.7097	94.25205	133.7817	94.23493	136.3585	94.3415	133.818	94.26955	135.329	94.40153	137.8978
94.34797	108.7062	94.26872	133.783	94.2516	136.3596	94.35817	133.8154	94.28622	135.3272	94.4182	137.8959
94.36463	108.7069	94.28538	133.786	94.26827	136.3554	94.37483	133.8129	94.30288	135.3277	94.43487	137.8982
94.3813	108.7077	94.30205	133.7848	94.28493	136.3541	94.3915	133.8138	94.31955	135.3263	94.45153	137.896
94.39797	108.7035	94.31872	133.7871	94.3016	136.3533	94.40817	133.8117	94.33622	135.3258	94.4682	137.8971
94.41463	108.7049	94.33538	133.785	94.31827	136.3537	94.42483	133.8114	94.35288	135.3289	94.48487	137.8917
94.4313	108.7016	94.35205	133.7892	94.33493	136.3501	94.4415	133.8071	94.36955	135.3234	94.50153	137.8959
94.44797	108.7025	94.36872	133.7876	94.3516	136.3516	94.45817	133.8081	94.38622	135.3253	94.5182	137.8909
94.46463	108.7003	94.38538	133.7884	94.36827	136.3488	94.47483	133.8085	94.40288	135.3258	94.53487	137.8907
94.4813	108.6985	94.40205	133.7881	94.38493	136.3491	94.4915	133.807	94.41955	135.3217	94.55153	137.8931
94.49797	108.6992	94.41872	133.7907	94.4016	136.3459	94.50817	133.8032	94.43622	135.324	94.5682	137.8926
94.51463	108.697	94.43538	133.7911	94.41827	136.347	94.52483	133.8026	94.45288	135.3244	94.58487	137.8921
94.5313	108.6961	94.45205	133.7921	94.43493	136.3428	94.5415	133.8012	94.46955	135.3206	94.60153	137.8902
94.54797	108.6958	94.46872	133.7905	94.4516	136.3447	94.55817	133.803	94.48622	135.3195	94.6182	137.8892
94.56463	108.6962	94.48538	133.7932	94.46827	136.3434	94.57483	133.8005	94.50288	135.3207	94.63487	137.887
94.5813	108.6937	94.50205	133.7953	94.48493	136.3414	94.5915	133.7988	94.51955	135.3184	94.65153	137.8891
94.59797	108.6938	94.51872	133.7937	94.5016	136.339	94.60817	133.7926	94.53622	135.3202	94.6682	137.8878
94.61463	108.6925	94.53538	133.7947	94.51827	136.3389	94.62483	133.7963	94.55288	135.3179	94.68487	137.8869
94.6313	108.6903	94.55205	133.7963	94.53493	136.3361	94.6415	133.7947	94.56955	135.3164	94.70153	137.887
94.64797	108.6923	94.56872	133.7967	94.5516	136.3347	94.65817	133.7923	94.58622	135.3158	94.7182	137.8862
94.66463	108.6901	94.58538	133.7978	94.56827	136.3339	94.67483	133.7909	94.60288	135.3156	94.73487	137.8855
94.6813	108.6903	94.60205	133.7965	94.58493	136.3307	94.6915	133.7895	94.61955	135.3164	94.75153	137.8846
94.69797	108.6887	94.61872	133.7989	94.6016	136.3315	94.70817	133.7875	94.63622	135.3158	94.7682	137.8849
94.71463	108.6864	94.63538	133.8006	94.61827	136.3288	94.72483	133.7882	94.65288	135.3117	94.78487	137.8837
94.7313	108.6853	94.65205	133.801	94.63493	136.3259	94.7415	133.7878	94.66955	135.3134	94.80153	137.8843
94.74797	108.6851	94.66872	133.801	94.6516	136.327	94.75817	133.7862	94.68622	135.3115	94.8182	137.8822
94.76463	108.6821	94.68538	133.8017	94.66827	136.3236	94.77483	133.7851	94.70288	135.3144	94.83487	137.8821
94.7813	108.6837	94.70205	133.8016	94.68493	136.3235	94.7915	133.7835	94.71955	135.313	94.85153	137.8809
94.79797	108.6829	94.71872	133.8036	94.7016	136.3223	94.80817	133.7835	94.73622	135.3118	94.8682	137.8802
94.81463	108.6777	94.73538	133.8021	94.71827	136.32	94.82483	133.7835	94.75288	135.3114	94.88487	137.8797
94.8313	108.6796	94.75205	133.8012	94.73493	136.319	94.8415	133.7805	94.76955	135.31	94.90153	137.8798
94.84797	108.6798	94.76872	133.8027	94.7516	136.3181	94.85817	133.7802	94.78622	135.3097	94.9182	137.8805
94.86463	108.6752	94.78538	133.8029	94.76827	136.3182	94.87483	133.7785	94.80288	135.3088	94.93487	137.8776
94.8813	108.6763	94.80205	133.8019	94.78493	136.3145	94.8915	133.7816	94.81955	135.3086	94.95153	137.8773
94.89797	108.6758	94.81872	133.8042	94.8016	136.315	94.90817	133.7759	94.83622	135.3079	94.9682	137.8767
94.91463	108.6735	94.83538	133.802	94.81827	136.3132	94.92483	133.7764	94.85288	135.309	94.98487	137.8766
94.9313	108.6731	94.85205	133.8031	94.83493	136.3103	94.9415	133.7727	94.86955	135.3083	95.00153	137.8747
94.94797	108.6743	94.86872	133.8034	94.8516	136.31	94.95817	133.7734	94.88622	135.3076	95.0182	137.8744
94.96463	108.6682	94.88538	133.8053	94.86827	136.3095	94.97483	133.7725	94.90288	135.3066	95.03487	137.8731
94.9813	108.6716	94.90205	133.8012	94.88493	136.3102	94.9915	133.7747	94.91955	135.3048	95.05153	137.8711
94.99797	108.67	94.91872	133.8042	94.9016	136.3075	95.00817	133.769	94.93622	135.3053	95.0682	137.8705
95.01463	108.6681	94.93538	133.8011	94.91827	136.3067	95.02483	133.7684	94.95288	135.3043	95.08487	137.8707
95.0313	108.6672	94.95205	133.8027	94.93493	136.3057	95.0415	133.7673	94.96955	135.3029	95.10153	137.8709
95.04797	108.6679	94.96872	133.8033	94.9516	136.3065	95.05817	133.7652	94.98622	135.3044	95.1182	137.8709
95.06463	108.6652	94.98538	133.8016	94.96827	136.3044	95.07483	133.7618	95.00288	135.3019	95.13487	137.8694
95.0813	108.6657	95.00205	133.804	94.98493	136.3012	95.0915	133.7623	95.01955	135.3032	95.15153	137.8675
95.09797	108.6651	95.01872	133.8029	95.0016	136.3016	95.10817	133.7593	95.03622	135.3017	95.1682	137.8666
95.11463	108.6654	95.03538	133.8043	95.01827	136.3004	95.12483	133.7598	95.05288	135.3007	95.18487	137.8664
95.1313	108.6648	95.05205	133.8041	95.03493	136.2964	95.1415	133.756	95.06955	135.3018	95.20153	137.8643
95.14797	108.6648	95.06872	133.8052	95.0516	136.2978	95.15817	133.7548	95.08622	135.3009	95.2182	137.8648
95.16463	108.6621	95.08538	133.8086	95.06827	136.2934	95.17483	133.7503	95.10288	135.2998	95.23487	137.8661
95.1813	108.6623	95.10205	133.8075	95.08493	136.2931	95.1915	133.7499	95.11955	135.2976	95.25153	137.8632
95.19797	108.6624	95.11872	133.8102	95.1016	136.2903	95.20817	133.7519	95.13622	135.2968	95.2682	137.8637
95.21463	108.6607	95.13538	133.806	95.11827	136.2897	95.22483	133.7525	95.15288	135.2988	95.28487	137.8619
95.2313	108.6591	95.15205	133.805	95.13493	136.2895	95.2415	133.7494	95.16955	135.297	95.30153	137.862
95.24797	108.6627	95.16872	133.8076	95.1516	136.2866	95.25817	133.7496	95.18622	135.2984	95.3182	137.8591
95.26463	108.6598	95.18538	133.8051	95.16827	136.2861	95.27483	133.7457	95.20288	135.298	95.33487	137.8598
95.2813	108.6583	95.20205	133.8048	95.18493	136.2836	95.2915	133.7444	95.21955	135.2937	95.35153	137.8584
95.29797	108.6559	95.21872	133.805	95.2016	136.2856	95.30817	133.7461	95.23622	135.2934	95.3682	137.8591
95.31463	108.656	95.23538	133.8022	95.21827	136.2846	95.32483	133.7433	95.25288	135.2956	95.38487	137.8579
95.3313	108.6548	95.25205	133.802	95.23493	136.2838	95.3415	133.7424	95.26955	135.294	95.40153	137.8585
95.34797	108.6539	95.26872	133.8039	95.2516	136.2828	95.35817	133.7399	95.28622	135.2952	95.4182	137.854
95.36463	108.6534	95.28538	133.8033	95.26827	136.2838	95.37483	133.7374	95.30288	135.293	95.43487	137.8544
95.3813	108.6519	95.30205	133.8064	95.28493	136.283	95.3915	133.7403	95.31955	135.2919	95.45153	137.8554
95.39797	108.649	95.31872	133.8043	95.3016	136.2795	95.40817	133.7393	95.33622	135.2929	95.4682	137.8539
95.41463	108.6511	95.33538	133.8039	95.31827	136.2778	95.42483	133.7376	95.35288	135.2944	95.48487	137.8513
95.4313	108.6506	95.35205	133.8041	95.33493	136.2764	95.4415	133.7347	95.36955	135.2906	95.50153	137.8508

95.44797	108.6477	95.36872	133.8044	95.3516	136.2779	95.45817	133.735	95.38622	135.2891	95.5182	137.8485
95.46463	108.65	95.38538	133.8034	95.36827	136.273	95.47483	133.7339	95.40288	135.29	95.53487	137.8517
95.4813	108.6481	95.40205	133.8037	95.38493	136.2723	95.4915	133.7328	95.41955	135.2914	95.55153	137.8512
95.49797	108.6467	95.41872	133.8049	95.4016	136.2706	95.50817	133.7303	95.43622	135.2909	95.5682	137.8491
95.51463	108.6485	95.43538	133.8086	95.41827	136.2711	95.52483	133.7308	95.45288	135.2903	95.58487	137.8497
95.5313	108.6438	95.45205	133.8064	95.43493	136.2677	95.5415	133.7275	95.46955	135.2909	95.60153	137.8489
95.54797	108.6453	95.46872	133.8063	95.4516	136.2651	95.55817	133.7309	95.48622	135.2851	95.6182	137.8463
95.56463	108.6455	95.48538	133.8101	95.46827	136.2645	95.57483	133.7271	95.50288	135.2902	95.63487	137.8468
95.5813	108.6414	95.50205	133.806	95.48493	136.2666	95.5915	133.7265	95.51955	135.2911	95.65153	137.8479
95.59797	108.644	95.51872	133.8137	95.5016	136.2582	95.60817	133.7246	95.53622	135.2892	95.6682	137.8451
95.61463	108.6419	95.53538	133.8122	95.51827	136.2629	95.62483	133.7228	95.55288	135.2899	95.68487	137.8451
95.6313	108.6388	95.55205	133.805	95.53493	136.2619	95.6415	133.7223	95.56955	135.2872	95.70153	137.8446
95.64797	108.6413	95.56872	133.8108	95.5516	136.2601	95.65817	133.7203	95.58622	135.287	95.7182	137.8431
95.66463	108.6387	95.58538	133.8164	95.56827	136.2548	95.67483	133.7182	95.60288	135.2867	95.73487	137.8424
95.6813	108.6387	95.60205	133.813	95.58493	136.2584	95.6915	133.7172	95.61955	135.2868	95.75153	137.8412
95.69797	108.6388	95.61872	133.8112	95.6016	136.2547	95.70817	133.7145	95.63622	135.2838	95.7682	137.8401
95.71463	108.6385	95.63538	133.8129	95.61827	136.2556	95.72483	133.7169	95.65288	135.2842	95.78487	137.8377
95.7313	108.6374	95.65205	133.8163	95.63493	136.2535	95.7415	133.7112	95.66955	135.2839	95.80153	137.8377
95.74797	108.636	95.66872	133.8171	95.6516	136.2512	95.75817	133.7107	95.68622	135.2843	95.8182	137.8374
95.76463	108.6337	95.68538	133.8179	95.66827	136.2542	95.77483	133.7081	95.70288	135.2806	95.83487	137.8368
95.7813	108.6344	95.70205	133.8192	95.68493	136.2534	95.7915	133.7096	95.71955	135.2813	95.85153	137.8372
95.79797	108.6328	95.71872	133.8185	95.7016	136.2477	95.80817	133.707	95.73622	135.282	95.8682	137.8366
95.81463	108.6313	95.73538	133.8128	95.71827	136.2456	95.82483	133.7068	95.75288	135.2798	95.88487	137.8344
95.8313	108.6311	95.75205	133.8177	95.73493	136.2507	95.8415	133.7031	95.76955	135.2803	95.90153	137.8352
95.84797	108.6295	95.76872	133.8182	95.7516	136.2486	95.85817	133.7017	95.78622	135.2785	95.9182	137.8356
95.86463	108.6289	95.78538	133.8195	95.76827	136.2391	95.87483	133.7032	95.80288	135.2784	95.93487	137.8332
95.8813	108.6272	95.80205	133.8183	95.78493	136.2429	95.8915	133.7009	95.81955	135.2798	95.95153	137.8323
95.89797	108.6271	95.81872	133.8194	95.8016	136.2437	95.90817	133.6994	95.83622	135.2771	95.9682	137.8313
95.91463	108.6275	95.83538	133.8199	95.81827	136.2421	95.92483	133.6995	95.85288	135.2772	95.98487	137.8334
95.9313	108.6265	95.85205	133.82	95.83493	136.24	95.9415	133.6978	95.86955	135.2754	96.00153	137.8313
95.94797	108.6266	95.86872	133.8199	95.8516	136.2426	95.95817	133.6965	95.88622	135.2763	96.0182	137.8336
95.96463	108.6257	95.88538	133.8204	95.86827	136.2378	95.97483	133.6948	95.90288	135.2735	96.03487	137.8312
95.9813	108.6215	95.90205	133.8211	95.88493	136.2349	95.9915	133.6941	95.91955	135.2744	96.05153	137.8289
95.99797	108.6213	95.91872	133.8211	95.9016	136.2348	96.00817	133.6921	95.93622	135.2736	96.0682	137.8261
96.01463	108.6243	95.93538	133.8219	95.91827	136.2352	96.02483	133.692	95.95288	135.2749	96.08487	137.8278
96.0313	108.619	95.95205	133.8238	95.93493	136.2299	96.0415	133.6906	95.96955	135.2722	96.10153	137.8251
96.04797	108.6215	95.96872	133.8233	95.9516	136.2302	96.05817	133.6893	95.98622	135.2735	96.1182	137.8263
96.06463	108.6176	95.98538	133.8229	95.96827	136.2297	96.07483	133.6873	96.00288	135.274	96.13487	137.8268
96.0813	108.617	96.00205	133.8256	95.98493	136.2291	96.0915	133.6902	96.01955	135.2723	96.15153	137.8238
96.09797	108.617	96.01872	133.8241	96.0016	136.2275	96.10817	133.6852	96.03622	135.2739	96.1682	137.8241
96.11463	108.62	96.03538	133.8268	96.01827	136.2247	96.12483	133.6856	96.05288	135.2725	96.18487	137.8242
96.1313	108.6155	96.05205	133.8263	96.03493	136.2257	96.1415	133.6836	96.06955	135.2725	96.20153	137.8239
96.14797	108.6165	96.06872	133.8246	96.0516	136.2259	96.15817	133.6833	96.08622	135.2722	96.2182	137.8214
96.16463	108.6153	96.08538	133.8288	96.06827	136.2224	96.17483	133.6819	96.10288	135.2709	96.23487	137.8202
96.1813	108.6131	96.10205	133.8277	96.08493	136.2235	96.1915	133.6809	96.11955	135.2699	96.25153	137.8211
96.19797	108.6152	96.11872	133.8289	96.1016	136.2221	96.20817	133.6793	96.13622	135.2694	96.2682	137.8202
96.21463	108.6129	96.13538	133.8283	96.11827	136.2204	96.22483	133.6766	96.15288	135.2706	96.28487	137.8199
96.2313	108.6126	96.15205	133.8286	96.13493	136.218	96.2415	133.6781	96.16955	135.2695	96.30153	137.8179
96.24797	108.6093	96.16872	133.8307	96.1516	136.2196	96.25817	133.6773	96.18622	135.2697	96.3182	137.8168
96.26463	108.6107	96.18538	133.8267	96.16827	136.2153	96.27483	133.6759	96.20288	135.2698	96.33487	137.8172
96.2813	108.6111	96.20205	133.8286	96.18493	136.2186	96.2915	133.6772	96.21955	135.2684	96.35153	137.8147
96.29797	108.6085	96.21872	133.8292	96.2016	136.2154	96.30817	133.6729	96.23622	135.266	96.3682	137.8153
96.31463	108.6087	96.23538	133.8239	96.21827	136.2133	96.32483	133.673	96.25288	135.2669	96.38487	137.8134
96.3313	108.6083	96.25205	133.8245	96.23493	136.2126	96.3415	133.6737	96.26955	135.2656	96.40153	137.8124
96.34797	108.6054	96.26872	133.8264	96.2516	136.2129	96.35817	133.6707	96.28622	135.2649	96.4182	137.8128
96.36463	108.605	96.28538	133.8259	96.26827	136.2098	96.37483	133.6703	96.30288	135.2644	96.43487	137.8105
96.3813	108.6054	96.30205	133.825	96.28493	136.2072	96.3915	133.6686	96.31955	135.2616	96.45153	137.8113
96.39797	108.6055	96.31872	133.8258	96.3016	136.2066	96.40817	133.6676	96.33622	135.2637	96.4682	137.8108
96.41463	108.6035	96.33538	133.8287	96.31827	136.207	96.42483	133.669	96.35288	135.2632	96.48487	137.8088
96.4313	108.601	96.35205	133.826	96.33493	136.2055	96.4415	133.6636	96.36955	135.2595	96.50153	137.808
96.44797	108.603	96.36872	133.8291	96.3516	136.207	96.45817	133.6644	96.38622	135.2587	96.5182	137.8082
96.46463	108.5991	96.38538	133.8314	96.36827	136.2009	96.47483	133.6615	96.40288	135.2582	96.53487	137.8072
96.4813	108.5983	96.40205	133.8288	96.38493	136.2016	96.4915	133.6642	96.41955	135.2574	96.55153	137.8063
96.49797	108.5973	96.41872	133.8294	96.4016	136.2013	96.50817	133.6623	96.43622	135.2558	96.5682	137.8054
96.51463	108.5987	96.43538	133.8281	96.41827	136.2009	96.52483	133.6598	96.45288	135.2566	96.58487	137.8055
96.5313	108.5973	96.45205	133.8293	96.43493	136.1997	96.5415	133.6558	96.46955	135.2536	96.60153	137.8048
96.54797	108.5962	96.46872	133.8291	96.4516	136.1972	96.55817	133.6555	96.48622	135.2566	96.6182	137.8035
96.56463	108.5966	96.48538	133.8285	96.46827	136.1941	96.57483	133.6565	96.50288	135.2537	96.63487	137.8047
96.5813	108.5967	96.50205	133.8279	96.48493	136.1956	96.5915	133.654	96.51955	135.2512	96.65153	137.8018
96.59797	108.5962	96.51872	133.83	96.5016	136.1973	96.60817	133.6525	96.53622	135.2527	96.6682	137.8013
96.61463	108.598	96.53538	133.8277	96.51827	136.1943	96.62483	133.6511	96.55288	135.2506	96.68487	137.8021
96.6313	108.5933	96.55205	133.8315	96.53493	136.1929	96.6415	133.6498	96.56955	135.253	96.70153	137.7994
96.64797	108.5912	96.56872	133.8329	96.5516	136.1958	96.65817	133.6489	96.58622	135.2529	96.7182	137.8008
96.66463	108.5926	96.58538	133.8314	96.56827	136.1929	96.67483	133.6476	96.60288	135.2481	96.73487	137.8014
96.6813	108.5912	96.60205	133.8314	96.58493	136.1916	96.6915	133.6459	96.61955	135.2479	96.75153	137.7995
96.69797	108.5912	96.61872	133.8296	96.6016	136.1899	96.70817	133.6463	96.63622	135.2472	96.7682	137.7975
96.71463	108.5875	96.63538	133.8333	96.61827	136.1919	96.72483	133.6451	96.65288	135.2493	96.78487	137.7976
96.7313	108.5893	96.65205	133.8333	96.63493	136.192	96.7415	133.6419	96.66955	135.2474	96.80153	137.7972
96.74797	108.5861	96.66872	133.833	96.6516	136.1882	96.75817	133.6411	96.68622	135.2505	96.8182	137.7956
96.76463	108.5864	96.68538	133.8308	96.66827	136.1866	96.77483	133.6399	96.70288	135.2456	96.83487	137.7969
96.7813	108.5866	96.70205	133.8342	96.68493	136.1874	96.7915	133.6395	96.71955	135.244	96.85153	137.7963
96.79797	108.5833	96.71872	133.8388	96.7016	136.1834	96.80817	133.6371	96.73622	135.2489	96.8682	137.7945
96.81463	108.5833	96.73538	133.8343	96.71827	136.1886	96.82483	133.6364	96.75288	135.2479	96.88487	137.7929
96.8313	108.5814	96.75205	133.8329	96.73493	136.1827	96.8415	133.6358	96.76955	135.2403	96.90153	137.7923
96.84797	108.5851	96.76872	133.8365	96.7516	136.1839	96.85817	133.6355	96.78622	135.2472	96.9182	137.7914
96.86463	108.5817	96.78538	133.8391	96.76827	136.1815	96.87483	133.6336	96.80288	135.2434	96.93487	137.7904
96.8813	108.5825	96.80205	133.8375	96.78493	136.1803	96.8915	133.6321	96.81955	135.241	96.95153	137.7912
96.89797	108.5811	96.81872	133.8365	96.8016	136.1802	96.90817	133.6298	96.83622	135.2415	96.9682	137.7903
96.91463	108.5806	96.83538	133.8372	96.81827	136.1803	96.92483	133.6326	96.85288	135.2415	96.98487	137.7889
96.9313	108.5774	96.85205	133.8374	96.83493	136.1754	96.9415	133.6305	96.86955	135.2375	97.00153	137.7891
96.94797	108.5783	96.86872	133.8381	96.8516	136.1804	96.95817	133.6304	96.88622	135.2388	97.0182	137.788
96.96463	108.5799	96.88538	133.8396	96.86827	136.177	96.97483	133.6301	96.90288	135.2371	97.03487	137.7863
96.9813	108.5769	96.90205	133.8391	96.88493	136.177	96.9915	133.6296	96.91955	135.2373	97.05153	137.786
96.99797	108.5758	96.91872	133.8379	96.9016	136.1759	97.00817	133.6262	96.93622	135.235	97.0682	137.7857
97.01463	108.5763	96.93538	133.8389	96.91827	136.1733	97.02483	133.6249	96.95288	135.2353	97.08487	137.7872
97.0313	108.5757	96.95205	133.8393	96.93493	136.1741	97.0415	133.6262	96.96955	135.2343	97.10153	137.7849
97.04797	108.5758	96.96872	133.8419	96.9516	136.1747	97.05817	133.6224	96.98622	135.2335	97.1182	137.7866

97.06463	108.5754	96.98538	133.8411	96.96827	136.1726	97.07483	133.6231	97.00288	135.2325	97.13487	137.7852
97.0813	108.5733	97.00205	133.8426	96.98493	136.1721	97.0915	133.6205	97.01955	135.2344	97.15153	137.784
97.09797	108.5724	97.01872	133.8449	97.0016	136.1685	97.10817	133.6191	97.03622	135.2336	97.1682	137.7852
97.11463	108.5723	97.03538	133.8412	97.01827	136.1697	97.12483	133.6174	97.05288	135.2331	97.18487	137.783
97.1313	108.5688	97.05205	133.8429	97.03493	136.1674	97.1415	133.6175	97.06955	135.2319	97.20153	137.7849
97.14797	108.5709	97.06872	133.8438	97.0516	136.1692	97.15817	133.6147	97.08622	135.233	97.2182	137.7842
97.16463	108.5694	97.08538	133.8447	97.06827	136.1682	97.17483	133.6136	97.10288	135.2322	97.23487	137.7807
97.1813	108.5693	97.10205	133.8463	97.08493	136.1686	97.1915	133.6112	97.11955	135.2304	97.25153	137.7803
97.19797	108.57	97.11872	133.8446	97.1016	136.1661	97.20817	133.6114	97.13622	135.2302	97.2682	137.7819
97.21463	108.5687	97.13538	133.8437	97.11827	136.1658	97.22483	133.6082	97.15288	135.23	97.28487	137.7818
97.2313	108.5646	97.15205	133.8439	97.13493	136.1681	97.2415	133.6074	97.16955	135.2283	97.30153	137.781
97.24797	108.569	97.16872	133.8443	97.1516	136.166	97.25817	133.6052	97.18622	135.2285	97.3182	137.7758
97.26463	108.5688	97.18538	133.8433	97.16827	136.1694	97.27483	133.6044	97.20288	135.2297	97.33487	137.7784
97.2813	108.5682	97.20205	133.8448	97.18493	136.1619	97.2915	133.6022	97.21955	135.228	97.35153	137.7772
97.29797	108.5649	97.21872	133.845	97.2016	136.1641	97.30817	133.6003	97.23622	135.2263	97.3682	137.7746
97.31463	108.5659	97.23538	133.8462	97.21827	136.1633	97.32483	133.5999	97.25288	135.2256	97.38487	137.7752
97.3313	108.5637	97.25205	133.8461	97.23493	136.1612	97.3415	133.5986	97.26955	135.2253	97.40153	137.7701
97.34797	108.5627	97.26872	133.8439	97.2516	136.1597	97.35817	133.5995	97.28622	135.2245	97.4182	137.7745
97.36463	108.5626	97.28538	133.8471	97.26827	136.1606	97.37483	133.5942	97.30288	135.2254	97.43487	137.7726
97.3813	108.5633	97.30205	133.8447	97.28493	136.1613	97.3915	133.594	97.31955	135.2245	97.45153	137.7715
97.39797	108.5643	97.31872	133.8491	97.3016	136.1583	97.40817	133.5929	97.33622	135.2232	97.4682	137.7679
97.41463	108.5589	97.33538	133.8474	97.31827	136.1606	97.42483	133.5912	97.35288	135.2221	97.48487	137.7698
97.4313	108.5608	97.35205	133.8474	97.33493	136.1582	97.4415	133.5882	97.36955	135.2217	97.50153	137.766
97.44797	108.5619	97.36872	133.8484	97.3516	136.1594	97.45817	133.5865	97.38622	135.2218	97.5182	137.7678
97.46463	108.5596	97.38538	133.8513	97.36827	136.1562	97.47483	133.5853	97.40288	135.2229	97.53487	137.7657
97.4813	108.558	97.40205	133.8492	97.38493	136.1562	97.4915	133.5853	97.41955	135.2198	97.55153	137.7634
97.49797	108.5574	97.41872	133.8499	97.4016	136.156	97.50817	133.5839	97.43622	135.2196	97.5682	137.7642
97.51463	108.5574	97.43538	133.8494	97.41827	136.1533	97.52483	133.5813	97.45288	135.2203	97.58487	137.7655
97.5313	108.5527	97.45205	133.8542	97.43493	136.154	97.5415	133.5795	97.46955	135.2178	97.60153	137.7615
97.54797	108.5549	97.46872	133.8482	97.4516	136.1521	97.55817	133.5798	97.48622	135.2171	97.6182	137.762
97.56463	108.554	97.48538	133.8502	97.46827	136.1518	97.57483	133.5777	97.50288	135.2144	97.63487	137.7633
97.5813	108.5525	97.50205	133.8498	97.48493	136.1524	97.5915	133.5778	97.51955	135.2161	97.65153	137.763
97.59797	108.5513	97.51872	133.8492	97.5016	136.149	97.60817	133.5746	97.53622	135.2166	97.6682	137.7612
97.61463	108.55	97.53538	133.8502	97.51827	136.1499	97.62483	133.5767	97.55288	135.2141	97.68487	137.763
97.6313	108.5466	97.55205	133.8507	97.53493	136.1495	97.6415	133.5755	97.56955	135.2125	97.70153	137.7601
97.64797	108.5468	97.56872	133.8508	97.5516	136.1505	97.65817	133.5717	97.58622	135.2127	97.7182	137.7581
97.66463	108.5475	97.58538	133.8531	97.56827	136.1468	97.67483	133.5707	97.60288	135.2121	97.73487	137.7577
97.6813	108.5474	97.60205	133.851	97.58493	136.1491	97.6915	133.5736	97.61955	135.2129	97.75153	137.7574
97.69797	108.5456	97.61872	133.8493	97.6016	136.146	97.70817	133.572	97.63622	135.2089	97.7682	137.7543
97.71463	108.545	97.63538	133.8522	97.61827	136.1474	97.72483	133.5716	97.65288	135.2102	97.78487	137.7575
97.7313	108.5457	97.65205	133.8512	97.63493	136.1473	97.7415	133.5673	97.66955	135.2089	97.80153	137.7559
97.74797	108.5447	97.66872	133.8562	97.6516	136.1481	97.75817	133.5682	97.68622	135.2093	97.8182	137.7531
97.76463	108.5401	97.68538	133.8561	97.66827	136.1438	97.77483	133.5671	97.70288	135.206	97.83487	137.7525
97.7813	108.541	97.70205	133.8562	97.68493	136.1444	97.7915	133.5668	97.71955	135.2088	97.85153	137.7529
97.79797	108.5391	97.71872	133.8579	97.7016	136.1453	97.80817	133.5617	97.73622	135.207	97.8682	137.7529
97.81463	108.5414	97.73538	133.8553	97.71827	136.1425	97.82483	133.5604	97.75288	135.2073	97.88487	137.7499
97.8313	108.5396	97.75205	133.8578	97.73493	136.1414	97.8415	133.5604	97.76955	135.2031	97.90153	137.7494
97.84797	108.5373	97.76872	133.8605	97.7516	136.1439	97.85817	133.556	97.78622	135.2051	97.9182	137.7498
97.86463	108.5362	97.78538	133.8588	97.76827	136.1411	97.87483	133.5548	97.80288	135.206	97.93487	137.7461
97.8813	108.5406	97.80205	133.8585	97.78493	136.1407	97.8915	133.5541	97.81955	135.2059	97.95153	137.7459
97.89797	108.5338	97.81872	133.8611	97.8016	136.1406	97.90817	133.5536	97.83622	135.2029	97.9682	137.7457
97.91463	108.5354	97.83538	133.8598	97.81827	136.1376	97.92483	133.5512	97.85288	135.2045	97.98487	137.7445
97.9313	108.5323	97.85205	133.8615	97.83493	136.1382	97.9415	133.5476	97.86955	135.2043	98.00153	137.745
97.94797	108.5318	97.86872	133.8626	97.8516	136.139	97.95817	133.5453	97.88622	135.2035	98.0182	137.7454
97.96463	108.5333	97.88538	133.8631	97.86827	136.1375	97.97483	133.5456	97.90288	135.2021	98.03487	137.7445
97.9813	108.5289	97.90205	133.8628	97.88493	136.1384	97.9915	133.5451	97.91955	135.2046	98.05153	137.7404
97.99797	108.5297	97.91872	133.867	97.9016	136.1381	98.00817	133.5416	97.93622	135.203	98.0682	137.7397
98.01463	108.5288	97.93538	133.8659	97.91827	136.1393	98.02483	133.5416	97.95288	135.2029	98.08487	137.7373
98.0313	108.5267	97.95205	133.8664	97.93493	136.1367	98.0415	133.5405	97.96955	135.2018	98.10153	137.7389
98.04797	108.526	97.96872	133.8645	97.9516	136.1359	98.05817	133.5376	97.98622	135.2001	98.1182	137.7372
98.06463	108.5266	97.98538	133.8639	97.96827	136.1344	98.07483	133.5365	98.00288	135.199	98.13487	137.7374
98.0813	108.5255	98.00205	133.8703	97.98493	136.134	98.0915	133.5357	98.01955	135.2004	98.15153	137.736
98.09797	108.524	98.01872	133.8648	98.0016	136.131	98.10817	133.5335	98.03622	135.1985	98.1682	137.735
98.11463	108.5228	98.03538	133.8683	98.01827	136.1324	98.12483	133.533	98.05288	135.2	98.18487	137.7325
98.1313	108.5235	98.05205	133.8699	98.03493	136.1303	98.1415	133.5305	98.06955	135.198	98.20153	137.731
98.14797	108.5215	98.06872	133.8702	98.0516	136.1312	98.15817	133.5316	98.08622	135.1972	98.2182	137.7307
98.16463	108.5176	98.08538	133.8726	98.06827	136.1258	98.17483	133.5336	98.10288	135.198	98.23487	137.7286
98.1813	108.5203	98.10205	133.8701	98.08493	136.1287	98.1915	133.5269	98.11955	135.1966	98.25153	137.7302
98.19797	108.5168	98.11872	133.8716	98.1016	136.129	98.20817	133.5302	98.13622	135.1964	98.2682	137.7298
98.21463	108.5173	98.13538	133.8682	98.11827	136.1315	98.22483	133.5268	98.15288	135.1968	98.28487	137.7271
98.2313	108.5155	98.15205	133.8712	98.13493	136.1306	98.2415	133.5247	98.16955	135.1952	98.30153	137.7261
98.24797	108.5183	98.16872	133.8689	98.1516	136.1274	98.25817	133.5205	98.18622	135.1954	98.3182	137.7268
98.26463	108.514	98.18538	133.8677	98.16827	136.1293	98.27483	133.5242	98.20288	135.1929	98.33487	137.7258
98.2813	108.5126	98.20205	133.8663	98.18493	136.1273	98.2915	133.522	98.21955	135.1944	98.35153	137.7245
98.29797	108.5136	98.21872	133.865	98.2016	136.1267	98.30817	133.5202	98.23622	135.1918	98.3682	137.7234
98.31463	108.5108	98.23538	133.8677	98.21827	136.1259	98.32483	133.5217	98.25288	135.1938	98.38487	137.7235
98.3313	108.513	98.25205	133.8678	98.23493	136.1194	98.3415	133.518	98.26955	135.1913	98.40153	137.7223
98.34797	108.5103	98.26872	133.8698	98.2516	136.1178	98.35817	133.5165	98.28622	135.1913	98.4182	137.7212
98.36463	108.5076	98.28538	133.8697	98.26827	136.1224	98.37483	133.5151	98.30288	135.1903	98.43487	137.7196
98.3813	108.5071	98.30205	133.8693	98.28493	136.1203	98.3915	133.5124	98.31955	135.1913	98.45153	137.7196
98.39797	108.509	98.31872	133.8691	98.3016	136.1205	98.40817	133.5134	98.33622	135.1886	98.4682	137.7165
98.41463	108.5072	98.33538	133.8691	98.31827	136.1193	98.42483	133.5117	98.35288	135.1877	98.48487	137.7153
98.4313	108.5048	98.35205	133.8691	98.33493	136.118	98.4415	133.5109	98.36955	135.1872	98.50153	137.7151
98.44797	108.5061	98.36872	133.8728	98.3516	136.1181	98.45817	133.5063	98.38622	135.1878	98.5182	137.718
98.46463	108.505	98.38538	133.8686	98.36827	136.1152	98.47483	133.5076	98.40288	135.186	98.53487	137.7161
98.4813	108.5041	98.40205	133.8701	98.38493	136.1146	98.4915	133.5067	98.41955	135.1863	98.55153	137.7167
98.49797	108.5038	98.41872	133.87	98.4016	136.1156	98.50817	133.5036	98.43622	135.1842	98.5682	137.7137
98.51463	108.5035	98.43538	133.8711	98.41827	136.1126	98.52483	133.5028	98.45288	135.1863	98.58487	137.7143
98.5313	108.5001	98.45205	133.8728	98.43493	136.1134	98.5415	133.5011	98.46955	135.1828	98.60153	137.7134
98.54797	108.4999	98.46872	133.8696	98.4516	136.1124	98.55817	133.5031	98.48622	135.1825	98.6182	137.7104
98.56463	108.4996	98.48538	133.8728	98.46827	136.1127	98.57483	133.5001	98.50288	135.1822	98.63487	137.7104
98.5813	108.5005	98.50205	133.8714	98.48493	136.1104	98.5915	133.4993	98.51955	135.1831	98.65153	137.7096
98.59797	108.4975	98.51872	133.8762	98.5016	136.1138	98.60817	133.5013	98.53622	135.1833	98.6682	137.7094
98.61463	108.4966	98.53538	133.8717	98.51827	136.1105	98.62483	133.4982	98.55288	135.1803	98.68487	137.7078
98.6313	108.4965	98.55205	133.8732	98.53493	136.1124	98.6415	133.4964	98.56955	135.1811	98.70153	137.7061
98.64797	108.4946	98.56872	133.8739	98.5516	136.1077	98.65817	133.4965	98.58622	135.1851	98.7182	137.707
98.66463	108.4931	98.58538	133.8763	98.56827	136.1068	98.67483	133.4965	98.60288	135.1807	98.73487	137.7051

98.6813	108.4942	98.60205	133.8749	98.58493	136.1058	98.6915	133.4941	98.61955	135.1804	98.75153	137.7046
98.69797	108.493	98.61872	133.8737	98.6016	136.1074	98.70817	133.4915	98.63622	135.1777	98.7682	137.704
98.71463	108.4921	98.63538	133.8789	98.61827	136.1086	98.72483	133.4887	98.65288	135.1779	98.78487	137.7021
98.7313	108.4926	98.65205	133.8799	98.63493	136.1005	98.7415	133.4883	98.66955	135.1799	98.80153	137.701
98.74797	108.4912	98.66872	133.8762	98.6516	136.1048	98.75817	133.4858	98.68622	135.1774	98.8182	137.7017
98.76463	108.4882	98.68538	133.8778	98.66827	136.1035	98.77483	133.484	98.70288	135.1778	98.83487	137.698
98.7813	108.4916	98.70205	133.882	98.68493	136.1034	98.7915	133.4819	98.71955	135.1745	98.85153	137.6991
98.79797	108.4885	98.71872	133.8766	98.7016	136.1002	98.80817	133.481	98.73622	135.1776	98.8682	137.6959
98.81463	108.4865	98.73538	133.8793	98.71827	136.105	98.82483	133.4797	98.75288	135.1779	98.88487	137.6948
98.8313	108.4849	98.75205	133.8811	98.73493	136.0992	98.8415	133.4782	98.76955	135.1772	98.90153	137.6958
98.84797	108.486	98.76872	133.8809	98.7516	136.1002	98.85817	133.4795	98.78622	135.1712	98.9182	137.6942
98.86463	108.4842	98.78538	133.881	98.76827	136.0996	98.87483	133.4783	98.80288	135.1723	98.93487	137.6934
98.8813	108.482	98.80205	133.8796	98.78493	136.1005	98.8915	133.4771	98.81955	135.1749	98.95153	137.693
98.89797	108.4808	98.81872	133.8798	98.8016	136.0949	98.90817	133.4734	98.83622	135.1715	98.9682	137.6929
98.91463	108.4804	98.83538	133.8807	98.81827	136.095	98.92483	133.4773	98.85288	135.173	98.98487	137.6914
98.9313	108.4784	98.85205	133.8804	98.83493	136.0974	98.9415	133.4727	98.86955	135.1714	99.00153	137.6898
98.94797	108.4796	98.86872	133.879	98.8516	136.0946	98.95817	133.4712	98.88622	135.1737	99.0182	137.6894
98.96463	108.478	98.88538	133.8847	98.86827	136.0938	98.97483	133.4706	98.90288	135.1758	99.03487	137.6872
98.9813	108.4775	98.90205	133.8803	98.88493	136.0951	98.9915	133.4713	98.91955	135.1708	99.05153	137.6874
98.99797	108.4752	98.91872	133.8797	98.9016	136.0934	99.00817	133.4677	98.93622	135.1708	99.0682	137.6853
99.01463	108.4751	98.93538	133.8827	98.91827	136.0945	99.02483	133.466	98.95288	135.1708	99.08487	137.6857
99.0313	108.4761	98.95205	133.8852	98.93493	136.0922	99.0415	133.4647	98.96955	135.1694	99.10153	137.6826
99.04797	108.4737	98.96872	133.884	98.9516	136.092	99.05817	133.4633	98.98622	135.1708	99.1182	137.6856
99.06463	108.4687	98.98538	133.8827	98.96827	136.0921	99.07483	133.4613	99.00288	135.1654	99.13487	137.6827
99.0813	108.4711	99.00205	133.8839	98.98493	136.0938	99.0915	133.4574	99.01955	135.1681	99.15153	137.6829
99.09797	108.4708	99.01872	133.8865	99.0016	136.0911	99.10817	133.4564	99.03622	135.1671	99.1682	137.6805
99.11463	108.4682	99.03538	133.8861	99.01827	136.0924	99.12483	133.4577	99.05288	135.1656	99.18487	137.6795
99.1313	108.4682	99.05205	133.8866	99.03493	136.0886	99.1415	133.4533	99.06955	135.1652	99.20153	137.6799
99.14797	108.4644	99.06872	133.8871	99.0516	136.091	99.15817	133.4513	99.08622	135.1633	99.2182	137.6778
99.16463	108.4661	99.08538	133.8874	99.06827	136.0928	99.17483	133.4521	99.10288	135.1624	99.23487	137.6774
99.1813	108.4663	99.10205	133.8875	99.08493	136.0904	99.1915	133.451	99.11955	135.1631	99.25153	137.677
99.19797	108.4628	99.11872	133.8853	99.1016	136.0905	99.20817	133.449	99.13622	135.1589	99.2682	137.673
99.21463	108.4637	99.13538	133.891	99.11827	136.0913	99.22483	133.4457	99.15288	135.1629	99.28487	137.6747
99.2313	108.4611	99.15205	133.8886	99.13493	136.091	99.2415	133.447	99.16955	135.1581	99.30153	137.6741
99.24797	108.4592	99.16872	133.8887	99.1516	136.0895	99.25817	133.4408	99.18622	135.1596	99.3182	137.669
99.26463	108.4632	99.18538	133.8884	99.16827	136.0871	99.27483	133.4431	99.20288	135.1602	99.33487	137.6693
99.2813	108.4612	99.20205	133.8932	99.18493	136.0879	99.2915	133.4401	99.21955	135.1578	99.35153	137.6697
99.29797	108.459	99.21872	133.8896	99.2016	136.0867	99.30817	133.439	99.23622	135.1563	99.3682	137.6696
99.31463	108.4576	99.23538	133.8913	99.21827	136.089	99.32483	133.4375	99.25288	135.1562	99.38487	137.6683
99.3313	108.4587	99.25205	133.8932	99.23493	136.0855	99.3415	133.4388	99.26955	135.1567	99.40153	137.6661
99.34797	108.4563	99.26872	133.8941	99.2516	136.0897	99.35817	133.4344	99.28622	135.155	99.4182	137.6674
99.36463	108.4542	99.28538	133.89	99.26827	136.0879	99.37483	133.4354	99.30288	135.1523	99.43487	137.6656
99.3813	108.4558	99.30205	133.8901	99.28493	136.0866	99.3915	133.4341	99.31955	135.1553	99.45153	137.6643
99.39797	108.4509	99.31872	133.8898	99.3016	136.087	99.40817	133.4318	99.33622	135.1556	99.4682	137.6664
99.41463	108.4501	99.33538	133.8889	99.31827	136.0854	99.42483	133.4302	99.35288	135.1524	99.48487	137.6621
99.4313	108.4501	99.35205	133.8894	99.33493	136.0854	99.4415	133.4311	99.36955	135.1521	99.50153	137.6601
99.44797	108.4514	99.36872	133.8885	99.3516	136.0846	99.45817	133.4284	99.38622	135.1566	99.5182	137.6628
99.46463	108.4507	99.38538	133.8873	99.36827	136.0857	99.47483	133.4276	99.40288	135.1492	99.53487	137.6594
99.4813	108.4473	99.40205	133.8866	99.38493	136.0828	99.4915	133.4254	99.41955	135.151	99.55153	137.6598
99.49797	108.4496	99.41872	133.8867	99.4016	136.0861	99.50817	133.4267	99.43622	135.1537	99.5682	137.6593
99.51463	108.4466	99.43538	133.8859	99.41827	136.0844	99.52483	133.4203	99.45288	135.1491	99.58487	137.6577
99.5313	108.4473	99.45205	133.8914	99.43493	136.085	99.5415	133.4239	99.46955	135.147	99.60153	137.6569
99.54797	108.4463	99.46872	133.8873	99.4516	136.0831	99.55817	133.4211	99.48622	135.1487	99.6182	137.6552
99.56463	108.4438	99.48538	133.8883	99.46827	136.0834	99.57483	133.4198	99.50288	135.1473	99.63487	137.6546
99.5813	108.4448	99.50205	133.889	99.48493	136.0825	99.5915	133.4194	99.51955	135.1487	99.65153	137.6529
99.59797	108.4406	99.51872	133.8873	99.5016	136.0822	99.60817	133.4155	99.53622	135.1453	99.6682	137.6517
99.61463	108.4412	99.53538	133.8889	99.51827	136.0816	99.62483	133.4151	99.55288	135.1463	99.68487	137.6499
99.6313	108.4407	99.55205	133.8882	99.53493	136.0827	99.6415	133.4149	99.56955	135.1444	99.70153	137.6501
99.64797	108.4415	99.56872	133.8908	99.5516	136.0824	99.65817	133.4143	99.58622	135.1442	99.7182	137.6493
99.66463	108.4394	99.58538	133.888	99.56827	136.0787	99.67483	133.4109	99.60288	135.1422	99.73487	137.648
99.6813	108.4391	99.60205	133.8894	99.58493	136.0782	99.6915	133.4118	99.61955	135.1431	99.75153	137.6491
99.69797	108.4384	99.61872	133.8872	99.6016	136.0818	99.70817	133.4106	99.63622	135.1431	99.7682	137.6447
99.71463	108.4383	99.63538	133.8888	99.61827	136.0798	99.72483	133.4089	99.65288	135.1438	99.78487	137.645
99.7313	108.4341	99.65205	133.8881	99.63493	136.0799	99.7415	133.4069	99.66955	135.1403	99.80153	137.6421
99.74797	108.4356	99.66872	133.8898	99.6516	136.0777	99.75817	133.4091	99.68622	135.1402	99.8182	137.6418
99.76463	108.4351	99.68538	133.8887	99.66827	136.0764	99.77483	133.4065	99.70288	135.1399	99.83487	137.6404
99.7813	108.4307	99.70205	133.8898	99.68493	136.0774	99.7915	133.4023	99.71955	135.1403	99.85153	137.6408
99.79797	108.4333	99.71872	133.8881	99.7016	136.0797	99.80817	133.4047	99.73622	135.1409	99.8682	137.639
99.81463	108.4316	99.73538	133.888	99.71827	136.0778	99.82483	133.4038	99.75288	135.1364	99.88487	137.6395
99.8313	108.4307	99.75205	133.8879	99.73493	136.0778	99.8415	133.4009	99.76955	135.1387	99.90153	137.6369
99.84797	108.4314	99.76872	133.8875	99.7516	136.0748	99.85817	133.3987	99.78622	135.1389	99.9182	137.6373
99.86463	108.4293	99.78538	133.8878	99.76827	136.075	99.87483	133.3999	99.80288	135.1383	99.93487	137.6357
99.8813	108.4273	99.80205	133.8874	99.78493	136.0733	99.8915	133.3973	99.81955	135.137	99.95153	137.6357
99.89797	108.4289	99.81872	133.8876	99.8016	136.0776	99.90817	133.3941	99.83622	135.1369	99.9682	137.635
99.91463	108.4307	99.83538	133.8855	99.81827	136.0707	99.92483	133.3941	99.85288	135.1364	99.98487	137.6354
99.9313	108.428	99.85205	133.883	99.83493	136.0759	99.9415	133.3938	99.86955	135.1348	100.0015	137.632
99.94797	108.4272	99.86872	133.889	99.8516	136.0724	99.95817	133.3897	99.88622	135.1353	100.0182	137.633
99.96463	108.4215	99.88538	133.8867	99.86827	136.0695	99.97483	133.3884	99.90288	135.1334	100.0349	137.6311
99.9813	108.4248	99.90205	133.8865	99.88493	136.07	99.9915	133.3906	99.91955	135.1334	100.0515	137.6334
99.99797	108.4236	99.91872	133.8851	99.9016	136.0726	100.0082	133.3866	99.93622	135.1323	100.0682	137.6305
100.0146	108.424	99.93538	133.888	99.91827	136.0698	100.0248	133.3862	99.95288	135.1311	100.0849	137.6291
100.0313	108.4253	99.95205	133.8898	99.93493	136.0699	100.0415	133.3836	99.96955	135.1311	100.1015	137.6264
100.048	108.4164	99.96872	133.8873	99.9516	136.0682	100.0582	133.3852	99.98622	135.1316	100.1182	137.6263
100.0646	108.4185	99.98538	133.8878	99.96827	136.0666	100.0748	133.3822	100.0029	135.1288	100.1349	137.6252
100.0813	108.4192	100.0021	133.8894	99.98493	136.0644	100.0915	133.3782	100.0196	135.1294	100.1515	137.6244
100.098	108.4211	100.0187	133.8875	100.0016	136.0638	100.1082	133.3814	100.0362	135.1284	100.1682	137.6235
100.1146	108.4145	100.0354	133.887	100.0183	136.0639	100.1248	133.3784	100.0529	135.129	100.1849	137.6228
100.1313	108.4151	100.0521	133.8862	100.0349	136.0656	100.1415	133.3778	100.0696	135.1273	100.2015	137.6228
100.148	108.4133	100.0687	133.8888	100.0516	136.061	100.1582	133.3745	100.0862	135.1257	100.2182	137.619
100.1646	108.4164	100.0854	133.8881	100.0683	136.0623	100.1748	133.3766	100.1029	135.1268	100.2349	137.6182
100.1813	108.4106	100.1021	133.8918	100.0849	136.0617	100.1915	133.3708	100.1196	135.1265	100.2515	137.6189
100.198	108.4118	100.1187	133.8882	100.1016	136.0628	100.2082	133.3727	100.1362	135.1239	100.2682	137.6161
100.2146	108.4087	100.1354	133.8908	100.1183	136.0641	100.2248	133.3709	100.1529	135.1227	100.2849	137.615
100.2313	108.4069	100.1521	133.8912	100.1349	136.0637	100.2415	133.3698	100.1696	135.124	100.3015	137.6154
100.248	108.4093	100.1687	133.8897	100.1516	136.0644	100.2582	133.3672	100.1862	135.1233	100.3182	137.615
100.2646	108.406	100.1854	133.8908	100.1683	136.0625	100.2748	133.3674	100.2029	135.124	100.3349	137.6151
100.2813	108.4053	100.2021	133.892	100.1849	136.0609	100.2915	133.368	100.2196	135.1209	100.3515	137.6141

100.298	108.4025	100.2187	133.8937	100.2016	136.0595	100.3082	133.3614	100.2362	135.1201	100.3682	137.6122
100.3146	108.4025	100.2354	133.893	100.2183	136.0584	100.3248	133.3625	100.2529	135.1184	100.3849	137.6115
100.3313	108.4033	100.2521	133.8941	100.2349	136.0591	100.3415	133.3626	100.2696	135.1183	100.4015	137.6103
100.348	108.4018	100.2687	133.892	100.2516	136.0599	100.3582	133.3598	100.2862	135.12	100.4182	137.6107
100.3646	108.3992	100.2854	133.8943	100.2683	136.0612	100.3748	133.3586	100.3029	135.1185	100.4349	137.6095
100.3813	108.403	100.3021	133.8936	100.2849	136.061	100.3915	133.36	100.3196	135.1185	100.4515	137.6088
100.398	108.3997	100.3187	133.8905	100.3016	136.0589	100.4082	133.3574	100.3362	135.1168	100.4682	137.6068
100.4146	108.3988	100.3354	133.89	100.3183	136.0569	100.4248	133.3556	100.3529	135.1187	100.4849	137.6067
100.4313	108.3979	100.3521	133.8886	100.3349	136.0573	100.4415	133.3558	100.3696	135.1179	100.5015	137.6053
100.448	108.3982	100.3687	133.89	100.3516	136.0572	100.4582	133.3537	100.3862	135.1167	100.5182	137.6062
100.4646	108.3945	100.3854	133.8887	100.3683	136.0547	100.4748	133.3535	100.4029	135.1146	100.5349	137.6029
100.4813	108.3966	100.4021	133.8892	100.3849	136.052	100.4915	133.3501	100.4196	135.1163	100.5515	137.6049
100.498	108.3951	100.4187	133.8863	100.4016	136.0551	100.5082	133.3517	100.4362	135.1143	100.5682	137.6036
100.5146	108.3918	100.4354	133.885	100.4183	136.0554	100.5248	133.3493	100.4529	135.115	100.5849	137.6005
100.5313	108.3936	100.4521	133.8843	100.4349	136.0564	100.5415	133.3495	100.4696	135.1144	100.6015	137.602
100.548	108.3911	100.4687	133.8836	100.4516	136.0536	100.5582	133.347	100.4862	135.1127	100.6182	137.6015
100.5646	108.3902	100.4854	133.8811	100.4683	136.0514	100.5748	133.3466	100.5029	135.113	100.6349	137.6
100.5813	108.3911	100.5021	133.8843	100.4849	136.0538	100.5915	133.3433	100.5196	135.1118	100.6515	137.5992
100.598	108.3894	100.5187	133.8821	100.5016	136.0544	100.6082	133.3423	100.5362	135.1104	100.6682	137.5973
100.6146	108.3892	100.5354	133.8818	100.5183	136.0521	100.6248	133.3428	100.5529	135.1096	100.6849	137.5974
100.6313	108.3861	100.5521	133.8807	100.5349	136.0533	100.6415	133.3414	100.5696	135.1109	100.7015	137.5961
100.648	108.3833	100.5687	133.8845	100.5516	136.0531	100.6582	133.3412	100.5862	135.1095	100.7182	137.5958
100.6646	108.3833	100.5854	133.8802	100.5683	136.05	100.6748	133.3385	100.6029	135.1056	100.7349	137.5964
100.6813	108.384	100.6021	133.882	100.5849	136.0518	100.6915	133.3386	100.6196	135.1102	100.7515	137.5969
100.698	108.3816	100.6187	133.8827	100.6016	136.0505	100.7082	133.3354	100.6362	135.1108	100.7682	137.5922
100.7146	108.381	100.6354	133.8831	100.6183	136.0493	100.7248	133.3337	100.6529	135.1061	100.7849	137.594
100.7313	108.3831	100.6521	133.8852	100.6349	136.0471	100.7415	133.3342	100.6696	135.1069	100.8015	137.5924
100.748	108.3777	100.6687	133.8835	100.6516	136.0471	100.7582	133.33	100.6862	135.1066	100.8182	137.5932
100.7646	108.3774	100.6854	133.8831	100.6683	136.0462	100.7748	133.3315	100.7029	135.1052	100.8349	137.5903
100.7813	108.3775	100.7021	133.8822	100.6849	136.0461	100.7915	133.3292	100.7196	135.1044	100.8515	137.5883
100.798	108.3749	100.7187	133.8825	100.7016	136.0448	100.8082	133.329	100.7362	135.1042	100.8682	137.5893
100.8146	108.3763	100.7354	133.8839	100.7183	136.0444	100.8248	133.3272	100.7529	135.104	100.8849	137.5872
100.8313	108.374	100.7521	133.8825	100.7349	136.0449	100.8415	133.3259	100.7696	135.104	100.9015	137.5871
100.848	108.3718	100.7687	133.8839	100.7516	136.0437	100.8582	133.3267	100.7862	135.1048	100.9182	137.5851
100.8646	108.3718	100.7854	133.8799	100.7683	136.0437	100.8748	133.3237	100.8029	135.1053	100.9349	137.5847
100.8813	108.3686	100.8021	133.8838	100.7849	136.0403	100.8915	133.3234	100.8196	135.1015	100.9515	137.5848
100.898	108.3698	100.8187	133.8828	100.8016	136.042	100.9082	133.3219	100.8362	135.1021	100.9682	137.5818
100.9146	108.3719	100.8354	133.8826	100.8183	136.0425	100.9248	133.3211	100.8529	135.1002	100.9849	137.5835
100.9313	108.3643	100.8521	133.8814	100.8349	136.0415	100.9415	133.3209	100.8696	135.1003	101.0015	137.5812
100.948	108.3645	100.8687	133.8826	100.8516	136.0393	100.9582	133.3191	100.8862	135.0991	101.0182	137.5822
100.9646	108.3658	100.8854	133.8811	100.8683	136.0402	100.9748	133.318	100.9029	135.0995	101.0349	137.5814
100.9813	108.3665	100.9021	133.8836	100.8849	136.0409	100.9915	133.3146	100.9196	135.0979	101.0515	137.5788
100.998	108.3612	100.9187	133.8821	100.9016	136.0418	101.0082	133.3153	100.9362	135.0966	101.0682	137.5815
101.0146	108.3608	100.9354	133.8824	100.9183	136.0374	101.0248	133.3138	100.9529	135.0975	101.0849	137.5798
101.0313	108.3583	100.9521	133.8837	100.9349	136.0389	101.0415	133.3126	100.9696	135.0981	101.1015	137.5789
101.048	108.3567	100.9687	133.883	100.9516	136.0391	101.0582	133.3104	100.9862	135.0966	101.1182	137.5791
101.0646	108.3574	100.9854	133.8809	100.9683	136.039	101.0748	133.3143	101.0029	135.095	101.1349	137.5782
101.0813	108.3592	101.0021	133.8814	100.9849	136.0369	101.0915	133.3106	101.0196	135.0921	101.1515	137.5789
101.098	108.3548	101.0187	133.882	101.0016	136.0349	101.1082	133.3093	101.0362	135.0966	101.1682	137.5763
101.1146	108.3585	101.0354	133.882	101.0183	136.0339	101.1248	133.3079	101.0529	135.0938	101.1849	137.5769
101.1313	108.3528	101.0521	133.8813	101.0349	136.0346	101.1415	133.3083	101.0696	135.0922	101.2015	137.5758
101.148	108.3519	101.0687	133.8808	101.0516	136.0371	101.1582	133.3058	101.0862	135.0921	101.2182	137.5724
101.1646	108.3554	101.0854	133.8801	101.0683	136.0365	101.1748	133.3064	101.1029	135.0903	101.2349	137.5747
101.1813	108.3476	101.1021	133.8815	101.0849	136.035	101.1915	133.3083	101.1196	135.0916	101.2515	137.5745
101.198	108.351	101.1187	133.8813	101.1016	136.0365	101.2082	133.3054	101.1362	135.0897	101.2682	137.5711
101.2146	108.347	101.1354	133.8815	101.1183	136.0367	101.2248	133.3039	101.1529	135.0903	101.2849	137.5728
101.2313	108.3473	101.1521	133.8818	101.1349	136.0354	101.2415	133.3043	101.1696	135.0882	101.3015	137.5706
101.248	108.3468	101.1687	133.8825	101.1516	136.0362	101.2582	133.304	101.1862	135.0887	101.3182	137.5704
101.2646	108.3459	101.1854	133.8817	101.1683	136.0369	101.2748	133.2993	101.2029	135.0879	101.3349	137.5676
101.2813	108.3461	101.2021	133.8816	101.1849	136.0333	101.2915	133.2992	101.2196	135.086	101.3515	137.5691
101.298	108.3442	101.2187	133.8821	101.2016	136.0357	101.3082	133.2976	101.2362	135.0834	101.3682	137.5676
101.3146	108.3427	101.2354	133.8847	101.2183	136.0333	101.3248	133.2959	101.2529	135.0847	101.3849	137.5666
101.3313	108.3418	101.2521	133.8819	101.2349	136.036	101.3415	133.2927	101.2696	135.0838	101.4015	137.5641
101.348	108.3409	101.2687	133.8821	101.2516	136.035	101.3582	133.2906	101.2862	135.0818	101.4182	137.5654
101.3646	108.3416	101.2854	133.8813	101.2683	136.0382	101.3748	133.2883	101.3029	135.0808	101.4349	137.5637
101.3813	108.3381	101.3021	133.8832	101.2849	136.0355	101.3915	133.2879	101.3196	135.0799	101.4515	137.5628
101.398	108.3388	101.3187	133.8832	101.3016	136.0359	101.4082	133.285	101.3362	135.0797	101.4682	137.5617
101.4146	108.3391	101.3354	133.8822	101.3183	136.0353	101.4248	133.2844	101.3529	135.0793	101.4849	137.5622
101.4313	108.3342	101.3521	133.8812	101.3349	136.0342	101.4415	133.2838	101.3696	135.0806	101.5015	137.5616
101.448	108.3376	101.3687	133.8837	101.3516	136.0338	101.4582	133.2817	101.3862	135.0775	101.5182	137.5589
101.4646	108.3355	101.3854	133.8814	101.3683	136.035	101.4748	133.2796	101.4029	135.077	101.5349	137.5565
101.4813	108.3334	101.4021	133.8861	101.3849	136.0324	101.4915	133.2771	101.4196	135.0759	101.5515	137.554
101.498	108.3331	101.4187	133.881	101.4016	136.0324	101.5082	133.2772	101.4362	135.0742	101.5682	137.5531
101.5146	108.3335	101.4354	133.8844	101.4183	136.0302	101.5248	133.2759	101.4529	135.0733	101.5849	137.5536
101.5313	108.3331	101.4521	133.8814	101.4349	136.0297	101.5415	133.2761	101.4696	135.0727	101.6015	137.555
101.548	108.3298	101.4687	133.8825	101.4516	136.0297	101.5582	133.2714	101.4862	135.0733	101.6182	137.5532
101.5646	108.3316	101.4854	133.8832	101.4683	136.0314	101.5748	133.2735	101.5029	135.0723	101.6349	137.552
101.5813	108.3315	101.5021	133.8838	101.4849	136.0271	101.5915	133.2705	101.5196	135.0715	101.6515	137.5499
101.598	108.3253	101.5187	133.8824	101.5016	136.0276	101.6082	133.2707	101.5362	135.0722	101.6682	137.5512
101.6146	108.3292	101.5354	133.8853	101.5183	136.0284	101.6248	133.2694	101.5529	135.0704	101.6849	137.5493
101.6313	108.3284	101.5521	133.8843	101.5349	136.0282	101.6415	133.2688	101.5696	135.0686	101.7015	137.5461
101.648	108.3236	101.5687	133.8852	101.5516	136.0261	101.6582	133.2672	101.5862	135.0711	101.7182	137.5464
101.6646	108.3244	101.5854	133.8845	101.5683	136.0262	101.6748	133.2673	101.6029	135.0678	101.7349	137.5465
101.6813	108.3232	101.6021	133.8864	101.5849	136.0249	101.6915	133.2636	101.6196	135.0678	101.7515	137.546
101.698	108.3218	101.6187	133.8848	101.6016	136.025	101.7082	133.2641	101.6362	135.0655	101.7682	137.5431
101.7146	108.3237	101.6354	133.8831	101.6183	136.0235	101.7248	133.2634	101.6529	135.0654	101.7849	137.542
101.7313	108.3209	101.6521	133.8824	101.6349	136.0232	101.7415	133.2632	101.6696	135.0653	101.8015	137.5412
101.748	108.3191	101.6687	133.8844	101.6516	136.0191	101.7582	133.2612	101.6862	135.0651	101.8182	137.5413
101.7646	108.3198	101.6854	133.8829	101.6683	136.024	101.7748	133.2597	101.7029	135.0645	101.8349	137.538
101.7813	108.3161	101.7021	133.881	101.6849	136.0202	101.7915	133.2579	101.7196	135.0628	101.8515	137.5386
101.798	108.3172	101.7187	133.8817	101.7016	136.0225	101.8082	133.2556	101.7362	135.0645	101.8682	137.535
101.8146	108.3151	101.7354	133.8836	101.7183	136.0198	101.8248	133.2534	101.7529	135.0622	101.8849	137.5366
101.8313	108.3134	101.7521	133.879	101.7349	136.0207	101.8415	133.2529	101.7696	135.0633	101.9015	137.5344
101.848	108.3135	101.7687	133.8804	101.7516	136.02	101.8582	133.2511	101.7862	135.063	101.9182	137.5349
101.8646	108.3149	101.7854	133.8792	101.7683	136.02	101.8748	133.2498	101.8029	135.0626	101.9349	137.5315
101.8813	108.3121	101.8021	133.8762	101.7849	136.0182	101.8915	133.25	101.8196	135.0611	101.9515	137.5336
101.898	108.3134	101.8187	133.8767	101.8016	136.0172	101.9082	133.2484	101.8362	135.0616	101.9682	137.5293

101.9146	108.3113	101.8354	133.8766	101.8183	136.0199	101.9248	133.2458	101.8529	135.0617	101.9849	137.5317
101.9313	108.3114	101.8521	133.8724	101.8349	136.0171	101.9415	133.2434	101.8696	135.0606	102.0015	137.5291
101.948	108.3107	101.8687	133.8757	101.8516	136.0187	101.9582	133.2435	101.8862	135.0586	102.0182	137.5289
101.9646	108.3092	101.8854	133.874	101.8683	136.0186	101.9748	133.2425	101.9029	135.0585	102.0349	137.5276
101.9813	108.3117	101.9021	133.8744	101.8849	136.0179	101.9915	133.2415	101.9196	135.0573	102.0515	137.5284
101.998	108.3051	101.9187	133.8738	101.9016	136.0196	102.0082	133.2397	101.9362	135.0571	102.0682	137.5254
102.0146	108.3053	101.9354	133.8747	101.9183	136.0183	102.0248	133.2396	101.9529	135.0582	102.0849	137.5235
102.0313	108.3069	101.9521	133.8721	101.9349	136.0191	102.0415	133.2389	101.9696	135.0565	102.1015	137.5226
102.048	108.3063	101.9687	133.8754	101.9516	136.0158	102.0582	133.2361	101.9862	135.0552	102.1182	137.5215
102.0646	108.3057	101.9854	133.8745	101.9683	136.019	102.0748	133.2346	102.0029	135.0557	102.1349	137.5204
102.0813	108.3017	102.0021	133.8742	101.9849	136.0172	102.0915	133.2354	102.0196	135.0549	102.1515	137.5195
102.098	108.3016	102.0187	133.8753	102.0016	136.0167	102.1082	133.2322	102.0362	135.0527	102.1682	137.5171
102.1146	108.3038	102.0354	133.8755	102.0183	136.0174	102.1248	133.2317	102.0529	135.051	102.1849	137.5178
102.1313	108.2996	102.0521	133.8745	102.0349	136.0157	102.1415	133.234	102.0696	135.0527	102.2015	137.5173
102.148	108.299	102.0687	133.8745	102.0516	136.0162	102.1582	133.2323	102.0862	135.0505	102.2182	137.5139
102.1646	108.2999	102.0854	133.878	102.0683	136.016	102.1748	133.2297	102.1029	135.05	102.2349	137.5147
102.1813	108.2992	102.1021	133.8765	102.0849	136.0155	102.1915	133.2278	102.1196	135.0484	102.2515	137.5134
102.198	108.2975	102.1187	133.8759	102.1016	136.0169	102.2082	133.2288	102.1362	135.0482	102.2682	137.5099
102.2146	108.2997	102.1354	133.8771	102.1183	136.0139	102.2248	133.2237	102.1529	135.0484	102.2849	137.5105
102.2313	108.2942	102.1521	133.8798	102.1349	136.0125	102.2415	133.2237	102.1696	135.0477	102.3015	137.5119
102.248	108.2969	102.1687	133.8786	102.1516	136.0097	102.2582	133.2201	102.1862	135.0443	102.3182	137.511
102.2646	108.2919	102.1854	133.8768	102.1683	136.0101	102.2748	133.2229	102.2029	135.044	102.3349	137.5085
102.2813	108.292	102.2021	133.8759	102.1849	136.0143	102.2915	133.22	102.2196	135.0455	102.3515	137.5071
102.298	108.2926	102.2187	133.88	102.2016	136.0129	102.3082	133.2187	102.2362	135.0432	102.3682	137.5064
102.3146	108.2926	102.2354	133.8801	102.2183	136.0128	102.3248	133.2202	102.2529	135.0432	102.3849	137.5054
102.3313	108.2914	102.2521	133.8796	102.2349	136.0129	102.3415	133.2203	102.2696	135.0435	102.4015	137.5044
102.348	108.2909	102.2687	133.8803	102.2516	136.0091	102.3582	133.2203	102.2862	135.0397	102.4182	137.5046
102.3646	108.2895	102.2854	133.88	102.2683	136.0107	102.3748	133.219	102.3029	135.0411	102.4349	137.5005
102.3813	108.2869	102.3021	133.8797	102.2849	136.0096	102.3915	133.2185	102.3196	135.0413	102.4515	137.5013
102.398	108.2882	102.3187	133.8805	102.3016	136.01	102.4082	133.214	102.3362	135.039	102.4682	137.4987
102.4146	108.2892	102.3354	133.8792	102.3183	136.0089	102.4248	133.2142	102.3529	135.036	102.4849	137.5002
102.4313	108.2846	102.3521	133.8787	102.3349	136.0082	102.4415	133.2162	102.3696	135.0379	102.5015	137.4973
102.448	108.285	102.3687	133.879	102.3516	136.0104	102.4582	133.2155	102.3862	135.0345	102.5182	137.4994
102.4646	108.2854	102.3854	133.8794	102.3683	136.009	102.4748	133.2154	102.4029	135.0351	102.5349	137.4958
102.4813	108.2814	102.4021	133.8792	102.3849	136.0068	102.4915	133.2139	102.4196	135.0347	102.5515	137.4954
102.498	108.2845	102.4187	133.8789	102.4016	136.0082	102.5082	133.2128	102.4362	135.034	102.5682	137.4958
102.5146	108.2842	102.4354	133.8807	102.4183	136.0063	102.5248	133.2092	102.4529	135.0326	102.5849	137.4939
102.5313	108.2793	102.4521	133.8806	102.4349	136.008	102.5415	133.2116	102.4696	135.0306	102.6015	137.4929
102.548	108.2802	102.4687	133.8816	102.4516	136.0074	102.5582	133.2082	102.4862	135.0287	102.6182	137.4926
102.5646	108.2813	102.4854	133.8825	102.4683	136.0078	102.5748	133.2072	102.5029	135.0264	102.6349	137.4936
102.5813	108.2777	102.5021	133.8811	102.4849	136.0083	102.5915	133.2057	102.5196	135.0272	102.6515	137.4922
102.598	108.2807	102.5187	133.8785	102.5016	136.0076	102.6082	133.2064	102.5362	135.0266	102.6682	137.4929
102.6146	108.2781	102.5354	133.8801	102.5183	136.0071	102.6248	133.2049	102.5529	135.0259	102.6849	137.4916
102.6313	108.2787	102.5521	133.8789	102.5349	136.0085	102.6415	133.2046	102.5696	135.0258	102.7015	137.4887
102.648	108.275	102.5687	133.8825	102.5516	136.0063	102.6582	133.2027	102.5862	135.0256	102.7182	137.4902
102.6646	108.2739	102.5854	133.8807	102.5683	136.0058	102.6748	133.2004	102.6029	135.0229	102.7349	137.4895
102.6813	108.2718	102.6021	133.8775	102.5849	136.0065	102.6915	133.1983	102.6196	135.0228	102.7515	137.4883
102.698	108.2747	102.6187	133.8811	102.6016	136.0056	102.7082	133.1954	102.6362	135.021	102.7682	137.4871
102.7146	108.2778	102.6354	133.8799	102.6183	136.0049	102.7248	133.1954	102.6529	135.0234	102.7849	137.4881
102.7313	108.2708	102.6521	133.8766	102.6349	136.0062	102.7415	133.1926	102.6696	135.021	102.8015	137.485
102.748	108.2719	102.6687	133.8768	102.6516	136.0052	102.7582	133.191	102.6862	135.0221	102.8182	137.4858
102.7646	108.2699	102.6854	133.8829	102.6683	136.0066	102.7748	133.19	102.7029	135.0214	102.8349	137.4846
102.7813	108.2732	102.7021	133.8809	102.6849	136.0058	102.7915	133.1865	102.7196	135.0202	102.8515	137.4831
102.798	108.2676	102.7187	133.8768	102.7016	136.003	102.8082	133.1876	102.7362	135.0193	102.8682	137.4818
102.8146	108.2673	102.7354	133.8798	102.7183	135.9994	102.8248	133.1854	102.7529	135.0197	102.8849	137.4839
102.8313	108.2708	102.7521	133.8774	102.7349	136.0004	102.8415	133.1826	102.7696	135.0195	102.9015	137.481
102.848	108.267	102.7687	133.8817	102.7516	135.9966	102.8582	133.182	102.7862	135.0203	102.9182	137.4804
102.8646	108.264	102.7854	133.8781	102.7683	135.9976	102.8748	133.1806	102.8029	135.0173	102.9349	137.4801
102.8813	108.2662	102.8021	133.8791	102.7849	135.9952	102.8915	133.1788	102.8196	135.017	102.9515	137.4768
102.898	108.2677	102.8187	133.8779	102.8016	135.9954	102.9082	133.1768	102.8362	135.0184	102.9682	137.4775
102.9146	108.2631	102.8354	133.8792	102.8183	135.9904	102.9248	133.1773	102.8529	135.0161	102.9849	137.4773
102.9313	108.2651	102.8521	133.8781	102.8349	135.992	102.9415	133.1755	102.8696	135.0164	103.0015	137.4757
102.948	108.2596	102.8687	133.8784	102.8516	135.9918	102.9582	133.1744	102.8862	135.0149	103.0182	137.476
102.9646	108.2608	102.8854	133.8756	102.8683	135.9879	102.9748	133.1727	102.9029	135.0142	103.0349	137.4746
102.9813	108.2581	102.9021	133.8763	102.8849	135.9929	102.9915	133.1717	102.9196	135.0123	103.0515	137.4756
102.998	108.2587	102.9187	133.876	102.9016	135.9894	103.0082	133.1716	102.9362	135.0133	103.0682	137.4734
103.0146	108.257	102.9354	133.8779	102.9183	135.9889	103.0248	133.1684	102.9529	135.0117	103.0849	137.4744
103.0313	108.2558	102.9521	133.8783	102.9349	135.99	103.0415	133.1678	102.9696	135.0093	103.1015	137.4726
103.048	108.2559	102.9687	133.8728	102.9516	135.9863	103.0582	133.1672	102.9862	135.0081	103.1182	137.4739
103.0646	108.2564	102.9854	133.8757	102.9683	135.9883	103.0748	133.1677	103.0029	135.0087	103.1349	137.4729
103.0813	108.253	103.0021	133.8766	102.9849	135.985	103.0915	133.1667	103.0196	135.0056	103.1515	137.4731
103.098	108.2526	103.0187	133.8764	103.0016	135.9859	103.1082	133.1654	103.0362	135.0082	103.1682	137.4705
103.1146	108.2518	103.0354	133.8736	103.0183	135.9838	103.1248	133.164	103.0529	135.0067	103.1849	137.4697
103.1313	108.2492	103.0521	133.8769	103.0349	135.9865	103.1415	133.1632	103.0696	135.0053	103.2015	137.47
103.148	108.253	103.0687	133.8749	103.0516	135.9859	103.1582	133.1594	103.0862	135.0069	103.2182	137.4668
103.1646	108.2484	103.0854	133.874	103.0683	135.9845	103.1748	133.1609	103.1029	135.0064	103.2349	137.468
103.1813	108.2483	103.1021	133.8757	103.0849	135.986	103.1915	133.159	103.1196	135.0038	103.2515	137.4669
103.198	108.2464	103.1187	133.8768	103.1016	135.9871	103.2082	133.158	103.1362	135.0035	103.2682	137.4651
103.2146	108.2452	103.1354	133.8754	103.1183	135.9833	103.2248	133.1567	103.1529	135.005	103.2849	137.4654
103.2313	108.2477	103.1521	133.8726	103.1349	135.9868	103.2415	133.1578	103.1696	135.0028	103.3015	137.4639
103.248	108.2448	103.1687	133.8728	103.1516	135.9842	103.2582	133.1534	103.1862	135	103.3182	137.4622
103.2646	108.2463	103.1854	133.8768	103.1683	135.9835	103.2748	133.1549	103.2029	135.0022	103.3349	137.4632
103.2813	108.2414	103.2021	133.8763	103.1849	135.9835	103.2915	133.1538	103.2196	135.0008	103.3515	137.4607
103.298	108.2425	103.2187	133.8739	103.2016	135.981	103.3082	133.1545	103.2362	134.9989	103.3682	137.4593
103.3146	108.2433	103.2354	133.8762	103.2183	135.9813	103.3248	133.151	103.2529	134.9986	103.3849	137.4606
103.3313	108.2395	103.2521	133.8721	103.2349	135.9778	103.3415	133.1503	103.2696	135.0011	103.4015	137.4611
103.348	108.2394	103.2687	133.8729	103.2516	135.9774	103.3582	133.1481	103.2862	134.9996	103.4182	137.4569
103.3646	108.239	103.2854	133.8755	103.2683	135.9772	103.3748	133.1477	103.3029	135.0003	103.4349	137.4555
103.3813	108.236	103.3021	133.876	103.2849	135.9772	103.3915	133.1453	103.3196	134.9974	103.4515	137.4564
103.398	108.2358	103.3187	133.8731	103.3016	135.9761	103.4082	133.145	103.3362	134.9982	103.4682	137.4536
103.4146	108.2376	103.3354	133.8744	103.3183	135.9733	103.4248	133.1435	103.3529	134.9979	103.4849	137.4531
103.4313	108.2355	103.3521	133.8723	103.3349	135.9736	103.4415	133.1451	103.3696	134.9974	103.5015	137.4557
103.448	108.2358	103.3687	133.8759	103.3516	135.974	103.4582	133.1434	103.3862	134.9945	103.5182	137.4532
103.4646	108.2348	103.3854	133.8743	103.3683	135.9722	103.4748	133.1424	103.4029	134.9978	103.5349	137.4491
103.4813	108.2326	103.4021	133.8753	103.3849	135.9719	103.4915	133.1424	103.4196	134.9948	103.5515	137.4524
103.498	108.2331	103.4187	133.8742	103.4016	135.9719	103.5082	133.1412	103.4362	134.9935	103.5682	137.451
103.5146	108.233	103.4354	133.874	103.4183	135.9705	103.5248	133.1386	103.4529	134.9923	103.5849	137.4478

103.5313	108.2303	103.4521	133.8716	103.4349	135.9696	103.5415	133.1383	103.4696	134.9937	103.6015	137.4466
103.548	108.2318	103.4687	133.8749	103.4516	135.9703	103.5582	133.1364	103.4862	134.9922	103.6182	137.4453
103.5646	108.2305	103.4854	133.872	103.4683	135.9673	103.5748	133.1349	103.5029	134.9904	103.6349	137.4452
103.5813	108.2273	103.5021	133.873	103.4849	135.9668	103.5915	133.1345	103.5196	134.991	103.6515	137.4445
103.598	108.2328	103.5187	133.8702	103.5016	135.9696	103.6082	133.1354	103.5362	134.99	103.6682	137.445
103.6146	108.2244	103.5354	133.8706	103.5183	135.966	103.6248	133.1311	103.5529	134.9869	103.6849	137.4395
103.6313	108.2268	103.5521	133.869	103.5349	135.9671	103.6415	133.1321	103.5696	134.9895	103.7015	137.4394
103.648	108.2266	103.5687	133.867	103.5516	135.9648	103.6582	133.1313	103.5862	134.9888	103.7182	137.4389
103.6646	108.2247	103.5854	133.8663	103.5683	135.9656	103.6748	133.1298	103.6029	134.9877	103.7349	137.4373
103.6813	108.2246	103.6021	133.8683	103.5849	135.9637	103.6915	133.1284	103.6196	134.9872	103.7515	137.4375
103.698	108.2219	103.6187	133.8684	103.6016	135.9639	103.7082	133.1267	103.6362	134.9867	103.7682	137.4353
103.7146	108.22	103.6354	133.8683	103.6183	135.962	103.7248	133.1257	103.6529	134.9855	103.7849	137.4355
103.7313	108.2204	103.6521	133.8658	103.6349	135.9611	103.7415	133.124	103.6696	134.9842	103.8015	137.4321
103.748	108.22	103.6687	133.8657	103.6516	135.9655	103.7582	133.1215	103.6862	134.981	103.8182	137.4343
103.7646	108.2206	103.6854	133.8668	103.6683	135.9636	103.7748	133.1211	103.7029	134.9844	103.8349	137.4328
103.7813	108.2158	103.7021	133.8717	103.6849	135.9619	103.7915	133.1182	103.7196	134.981	103.8515	137.4324
103.798	108.2145	103.7187	133.8699	103.7016	135.9629	103.8082	133.1157	103.7362	134.9823	103.8682	137.4305
103.8146	108.2153	103.7354	133.8661	103.7183	135.9608	103.8248	133.1149	103.7529	134.9807	103.8849	137.4327
103.8313	108.2156	103.7521	133.8677	103.7349	135.9624	103.8415	133.1113	103.7696	134.9816	103.9015	137.4318
103.848	108.2168	103.7687	133.8671	103.7516	135.9597	103.8582	133.1118	103.7862	134.979	103.9182	137.4286
103.8646	108.2142	103.7854	133.8662	103.7683	135.9619	103.8748	133.11	103.8029	134.9782	103.9349	137.4296
103.8813	108.2138	103.8021	133.8662	103.7849	135.9599	103.8915	133.1088	103.8196	134.9798	103.9515	137.4277
103.898	108.2122	103.8187	133.8645	103.8016	135.9611	103.9082	133.1058	103.8362	134.9763	103.9682	137.427
103.9146	108.215	103.8354	133.8646	103.8183	135.9598	103.9248	133.1057	103.8529	134.9767	103.9849	137.4268
103.9313	108.2118	103.8521	133.8616	103.8349	135.9578	103.9415	133.1042	103.8696	134.9762	104.0015	137.4255
103.948	108.2119	103.8687	133.8641	103.8516	135.9608	103.9582	133.1038	103.8862	134.9762	104.0182	137.4229
103.9646	108.2093	103.8854	133.8602	103.8683	135.9601	103.9748	133.1013	103.9029	134.9768	104.0349	137.4226
103.9813	108.2069	103.9021	133.8631	103.8849	135.9608	103.9915	133.1003	103.9196	134.9765	104.0515	137.421
103.998	108.209	103.9187	133.8639	103.9016	135.9577	104.0082	133.0984	103.9362	134.9751	104.0682	137.4224
104.0146	108.2058	103.9354	133.8563	103.9183	135.959	104.0248	133.0983	103.9529	134.9745	104.0849	137.4219
104.0313	108.2068	103.9521	133.8582	103.9349	135.9541	104.0415	133.0959	103.9696	134.9744	104.1015	137.4216
104.048	108.2028	103.9687	133.8577	103.9516	135.9583	104.0582	133.0934	103.9862	134.9724	104.1182	137.4183
104.0646	108.2041	103.9854	133.857	103.9683	135.96	104.0748	133.0958	104.0029	134.9743	104.1349	137.42
104.0813	108.2027	104.0021	133.8575	103.9849	135.9566	104.0915	133.0944	104.0196	134.9723	104.1515	137.4194
104.098	108.2017	104.0187	133.8559	104.0016	135.9535	104.1082	133.0922	104.0362	134.9722	104.1682	137.4175
104.1146	108.2009	104.0354	133.8557	104.0183	135.9549	104.1248	133.0914	104.0529	134.9702	104.1849	137.4173
104.1313	108.1991	104.0521	133.8561	104.0349	135.9578	104.1415	133.0924	104.0696	134.9709	104.2015	137.4176
104.148	108.1978	104.0687	133.8573	104.0516	135.9546	104.1582	133.0885	104.0862	134.9689	104.2182	137.4172
104.1646	108.2006	104.0854	133.8543	104.0683	135.9534	104.1748	133.0888	104.1029	134.9688	104.2349	137.4149
104.1813	108.1942	104.1021	133.855	104.0849	135.9542	104.1915	133.0846	104.1196	134.9694	104.2515	137.4149
104.198	108.1966	104.1187	133.8567	104.1016	135.9518	104.2082	133.0844	104.1362	134.9662	104.2682	137.4134
104.2146	108.1947	104.1354	133.8574	104.1183	135.9511	104.2248	133.0856	104.1529	134.9652	104.2849	137.4131
104.2313	108.1934	104.1521	133.8571	104.1349	135.9518	104.2415	133.0848	104.1696	134.968	104.3015	137.4111
104.248	108.1907	104.1687	133.8555	104.1516	135.9494	104.2582	133.0826	104.1862	134.9632	104.3182	137.4117
104.2646	108.1951	104.1854	133.8549	104.1683	135.9495	104.2748	133.0814	104.2029	134.9654	104.3349	137.4096
104.2813	108.1928	104.2021	133.8563	104.1849	135.9506	104.2915	133.0818	104.2196	134.9629	104.3515	137.409
104.298	108.1886	104.2187	133.8563	104.2016	135.9491	104.3082	133.0799	104.2362	134.9608	104.3682	137.4069
104.3146	108.1891	104.2354	133.8569	104.2183	135.9461	104.3248	133.0783	104.2529	134.9621	104.3849	137.4061
104.3313	108.1913	104.2521	133.853	104.2349	135.9503	104.3415	133.0765	104.2696	134.9623	104.4015	137.4089
104.348	108.1854	104.2687	133.8571	104.2516	135.9475	104.3582	133.0775	104.2862	134.9597	104.4182	137.4064
104.3646	108.186	104.2854	133.8556	104.2683	135.9456	104.3748	133.0769	104.3029	134.96	104.4349	137.4077
104.3813	108.1881	104.3021	133.8589	104.2849	135.947	104.3915	133.0745	104.3196	134.96	104.4515	137.4051
104.398	108.1809	104.3187	133.8559	104.3016	135.9501	104.4082	133.0749	104.3362	134.9589	104.4682	137.4044
104.4146	108.1823	104.3354	133.8589	104.3183	135.9411	104.4248	133.0698	104.3529	134.9591	104.4849	137.4053
104.4313	108.1876	104.3521	133.8585	104.3349	135.9482	104.4415	133.0735	104.3696	134.9567	104.5015	137.4037
104.448	108.1816	104.3687	133.8583	104.3516	135.9472	104.4582	133.0712	104.3862	134.9575	104.5182	137.4027
104.4646	108.1763	104.3854	133.8563	104.3683	135.942	104.4748	133.0686	104.4029	134.9577	104.5349	137.4013
104.4813	108.182	104.4021	133.8574	104.3849	135.946	104.4915	133.0685	104.4196	134.9549	104.5515	137.4009
104.498	108.1797	104.4187	133.8554	104.4016	135.9445	104.5082	133.0681	104.4362	134.9535	104.5682	137.4013
104.5146	108.1785	104.4354	133.857	104.4183	135.944	104.5248	133.0671	104.4529	134.9545	104.5849	137.3984
104.5313	108.1748	104.4521	133.8525	104.4349	135.9432	104.5415	133.0686	104.4696	134.9548	104.6015	137.3988
104.548	108.1754	104.4687	133.8551	104.4516	135.9412	104.5582	133.0654	104.4862	134.9523	104.6182	137.3975
104.5646	108.1779	104.4854	133.8534	104.4683	135.9415	104.5748	133.0636	104.5029	134.95	104.6349	137.397
104.5813	108.1704	104.5021	133.8525	104.4849	135.9448	104.5915	133.0632	104.5196	134.9517	104.6515	137.3981
104.598	108.1731	104.5187	133.8545	104.5016	135.9447	104.6082	133.0633	104.5362	134.9502	104.6682	137.3975
104.6146	108.1712	104.5354	133.8523	104.5183	135.9383	104.6248	133.0631	104.5529	134.9483	104.6849	137.3972
104.6313	108.1742	104.5521	133.8563	104.5349	135.9398	104.6415	133.0605	104.5696	134.9498	104.7015	137.3963
104.648	108.1685	104.5687	133.8551	104.5516	135.9428	104.6582	133.0594	104.5862	134.9486	104.7182	137.3905
104.6646	108.1695	104.5854	133.852	104.5683	135.9388	104.6748	133.0561	104.6029	134.9491	104.7349	137.3934
104.6813	108.1673	104.6021	133.8493	104.5849	135.9408	104.6915	133.0573	104.6196	134.9478	104.7515	137.394
104.698	108.1685	104.6187	133.8506	104.6016	135.9372	104.7082	133.0566	104.6362	134.9452	104.7682	137.393
104.7146	108.1669	104.6354	133.8498	104.6183	135.9365	104.7248	133.0534	104.6529	134.9441	104.7849	137.3905
104.7313	108.1642	104.6521	133.8516	104.6349	135.9404	104.7415	133.0523	104.6696	134.9457	104.8015	137.3903
104.748	108.1622	104.6687	133.8492	104.6516	135.9367	104.7582	133.0505	104.6862	134.9441	104.8182	137.3906
104.7646	108.1625	104.6854	133.8502	104.6683	135.9365	104.7748	133.0509	104.7029	134.9434	104.8349	137.3896
104.7813	108.1648	104.7021	133.8491	104.6849	135.9261	104.7915	133.0477	104.7196	134.9459	104.8515	137.3878
104.798	108.1624	104.7187	133.8473	104.7016	135.9338	104.8082	133.0472	104.7362	134.9419	104.8682	137.3865
104.8146	108.1604	104.7354	133.8476	104.7183	135.9299	104.8248	133.0459	104.7529	134.9426	104.8849	137.3854
104.8313	108.1598	104.7521	133.8495	104.7349	135.9307	104.8415	133.0451	104.7696	134.9425	104.9015	137.3826
104.848	108.1584	104.7687	133.844	104.7516	135.9302	104.8582	133.0446	104.7862	134.9425	104.9182	137.3845
104.8646	108.1568	104.7854	133.8454	104.7683	135.9286	104.8748	133.046	104.8029	134.9413	104.9349	137.3827
104.8813	108.1563	104.8021	133.8415	104.7849	135.9293	104.8915	133.044	104.8196	134.9386	104.9515	137.3784
104.898	108.1571	104.8187	133.8431	104.8016	135.9331	104.9082	133.0406	104.8362	134.9414	104.9682	137.3822
104.9146	108.1539	104.8354	133.8418	104.8183	135.9247	104.9248	133.0417	104.8529	134.9386	104.9849	137.38
104.9313	108.1533	104.8521	133.8411	104.8349	135.9283	104.9415	133.0372	104.8696	134.9388	105.0015	137.3794
104.948	108.1552	104.8687	133.8382	104.8516	135.9306	104.9582	133.0394	104.8862	134.938	105.0182	137.3767
104.9646	108.1507	104.8854	133.8388	104.8683	135.9248	104.9748	133.0366	104.9029	134.9379	105.0349	137.3769
104.9813	108.1543	104.9021	133.8365	104.8849	135.9269	104.9915	133.0335	104.9196	134.9367	105.0515	137.3751
104.998	108.1491	104.9187	133.8362	104.9016	135.9268	105.0082	133.035	104.9362	134.9387	105.0682	137.3746
105.0146	108.1491	104.9354	133.8353	104.9183	135.9276	105.0248	133.0355	104.9529	134.935	105.0849	137.3748
105.0313	108.1518	104.9521	133.837	104.9349	135.9234	105.0415	133.032	104.9696	134.9357	105.1015	137.3743
105.048	108.1468	104.9687	133.8335	104.9516	135.9229	105.0582	133.0306	104.9862	134.9349	105.1182	137.3735
105.0646	108.1476	104.9854	133.8328	104.9683	135.922	105.0748	133.0324	105.0029	134.9359	105.1349	137.3729
105.0813	108.1462	105.0021	133.8318	104.9849	135.9232	105.0915	133.0311	105.0196	134.9313	105.1515	137.3687
105.098	108.1462	105.0187	133.8326	105.0016	135.9201	105.1082	133.029	105.0362	134.9345	105.1682	137.3712
105.1146	108.1472	105.0354	133.8321	105.0183	135.9172	105.1248	133.0295	105.0529	134.9319	105.1849	137.3689
105.1313	108.1448	105.0521	133.8298	105.0349	135.9182	105.1415	133.0297	105.0696	134.9322	105.2015	137.368

105.148	108.1423	105.0687	133.83	105.0516	135.9187	105.1582	133.0265	105.0862	134.932	105.2182	137.3678
105.1646	108.1427	105.0854	133.8287	105.0683	135.9168	105.1748	133.0256	105.1029	134.9318	105.2349	137.3663
105.1813	108.1403	105.1021	133.8312	105.0849	135.9154	105.1915	133.0231	105.1196	134.9308	105.2515	137.3665
105.198	108.1435	105.1187	133.828	105.1016	135.9151	105.2082	133.0212	105.1362	134.9312	105.2682	137.364
105.2146	108.1414	105.1354	133.8271	105.1183	135.9157	105.2248	133.0226	105.1529	134.9305	105.2849	137.3657
105.2313	108.1405	105.1521	133.8269	105.1349	135.9094	105.2415	133.0214	105.1696	134.9312	105.3015	137.3631
105.248	108.1401	105.1687	133.8275	105.1516	135.91	105.2582	133.0175	105.1862	134.9278	105.3182	137.3599
105.2646	108.1381	105.1854	133.8238	105.1683	135.9073	105.2748	133.0201	105.2029	134.9282	105.3349	137.3623
105.2813	108.14	105.2021	133.8257	105.1849	135.9098	105.2915	133.0138	105.2196	134.928	105.3515	137.361
105.298	108.137	105.2187	133.8204	105.2016	135.9072	105.3082	133.013	105.2362	134.928	105.3682	137.3593
105.3146	108.1356	105.2354	133.8238	105.2183	135.9082	105.3248	133.0142	105.2529	134.9249	105.3849	137.3595
105.3313	108.1361	105.2521	133.8231	105.2349	135.906	105.3415	133.0155	105.2696	134.9249	105.4015	137.3587
105.348	108.1389	105.2687	133.8224	105.2516	135.9072	105.3582	133.0121	105.2862	134.9251	105.4182	137.3561
105.3646	108.1353	105.2854	133.8224	105.2683	135.9048	105.3748	133.0092	105.3029	134.9248	105.4349	137.3577
105.3813	108.1357	105.3021	133.8226	105.2849	135.9053	105.3915	133.0097	105.3196	134.9243	105.4515	137.3574
105.398	108.1314	105.3187	133.8233	105.3016	135.905	105.4082	133.0076	105.3362	134.922	105.4682	137.3534
105.4146	108.1331	105.3354	133.8196	105.3183	135.9046	105.4248	133.005	105.3529	134.9203	105.4849	137.3547
105.4313	108.1313	105.3521	133.8187	105.3349	135.9017	105.4415	133.0068	105.3696	134.9222	105.5015	137.3539
105.448	108.1319	105.3687	133.8209	105.3516	135.9037	105.4582	133.0034	105.3862	134.9219	105.5182	137.3541
105.4646	108.1311	105.3854	133.8207	105.3683	135.9051	105.4748	133.0038	105.4029	134.9199	105.5349	137.3519
105.4813	108.1297	105.4021	133.8204	105.3849	135.9011	105.4915	133.0015	105.4196	134.9201	105.5515	137.3522
105.498	108.1301	105.4187	133.8191	105.4016	135.9028	105.5082	132.9987	105.4362	134.9189	105.5682	137.3514
105.5146	108.1295	105.4354	133.8195	105.4183	135.9021	105.5248	132.998	105.4529	134.9177	105.5849	137.3509
105.5313	108.1253	105.4521	133.8198	105.4349	135.9024	105.5415	132.9974	105.4696	134.9179	105.6015	137.3492
105.548	108.1269	105.4687	133.8185	105.4516	135.9026	105.5582	132.9984	105.4862	134.9166	105.6182	137.3486
105.5646	108.1272	105.4854	133.8187	105.4683	135.8995	105.5748	132.9964	105.5029	134.9185	105.6349	137.3488
105.5813	108.1242	105.5021	133.8199	105.4849	135.9015	105.5915	132.9951	105.5196	134.9157	105.6515	137.3456
105.598	108.1254	105.5187	133.8186	105.5016	135.9012	105.6082	132.9931	105.5362	134.916	105.6682	137.3478
105.6146	108.1246	105.5354	133.8203	105.5183	135.897	105.6248	132.9912	105.5529	134.9152	105.6849	137.3445
105.6313	108.122	105.5521	133.8174	105.5349	135.8986	105.6415	132.9919	105.5696	134.9165	105.7015	137.3452
105.648	108.1212	105.5687	133.8166	105.5516	135.8982	105.6582	132.9916	105.5862	134.9155	105.7182	137.3426
105.6646	108.1209	105.5854	133.8182	105.5683	135.899	105.6748	132.9898	105.6029	134.9142	105.7349	137.3432
105.6813	108.1225	105.6021	133.8174	105.5849	135.8975	105.6915	132.991	105.6196	134.9134	105.7515	137.343
105.698	108.1189	105.6187	133.8188	105.6016	135.8959	105.7082	132.9876	105.6362	134.9155	105.7682	137.3425
105.7146	108.1167	105.6354	133.8168	105.6183	135.8968	105.7248	132.9854	105.6529	134.9116	105.7849	137.3396
105.7313	108.1184	105.6521	133.8222	105.6349	135.8968	105.7415	132.9848	105.6696	134.9126	105.8015	137.3403
105.748	108.1175	105.6687	133.8168	105.6516	135.8951	105.7582	132.9833	105.6862	134.9133	105.8182	137.3398
105.7646	108.1156	105.6854	133.8137	105.6683	135.8967	105.7748	132.9826	105.7029	134.9129	105.8349	137.3406
105.7813	108.1168	105.7021	133.8162	105.6849	135.8962	105.7915	132.9823	105.7196	134.91	105.8515	137.3388
105.798	108.1163	105.7187	133.8163	105.7016	135.8944	105.8082	132.9833	105.7362	134.9113	105.8682	137.336
105.8146	108.1128	105.7354	133.8143	105.7183	135.8947	105.8248	132.9814	105.7529	134.9107	105.8849	137.3347
105.8313	108.1136	105.7521	133.8147	105.7349	135.8947	105.8415	132.9825	105.7696	134.9096	105.9015	137.3358
105.848	108.1132	105.7687	133.8097	105.7516	135.8951	105.8582	132.9809	105.7862	134.9098	105.9182	137.3338
105.8646	108.1138	105.7854	133.8147	105.7683	135.8932	105.8748	132.9775	105.8029	134.9087	105.9349	137.3326
105.8813	108.1107	105.8021	133.8138	105.7849	135.89	105.8915	132.9784	105.8196	134.907	105.9515	137.3294
105.898	108.1126	105.8187	133.812	105.8016	135.8936	105.9082	132.9776	105.8362	134.9093	105.9682	137.3333
105.9146	108.1111	105.8354	133.8109	105.8183	135.8915	105.9248	132.9752	105.8529	134.9049	105.9849	137.33
105.9313	108.1073	105.8521	133.8095	105.8349	135.8932	105.9415	132.977	105.8696	134.9062	106.0015	137.331
105.948	108.1078	105.8687	133.8125	105.8516	135.8876	105.9582	132.9771	105.8862	134.9057	106.0182	137.3315
105.9646	108.1071	105.8854	133.8114	105.8683	135.8901	105.9748	132.9726	105.9029	134.905	106.0349	137.3269
105.9813	108.1069	105.9021	133.8115	105.8849	135.8905	105.9915	132.9712	105.9196	134.9024	106.0515	137.328
105.998	108.1054	105.9187	133.8098	105.9016	135.8898	106.0082	132.9705	105.9362	134.9045	106.0682	137.3268
106.0146	108.1069	105.9354	133.8094	105.9183	135.89	106.0248	132.9704	105.9529	134.9053	106.0849	137.3259
106.0313	108.1041	105.9521	133.8082	105.9349	135.8898	106.0415	132.9701	105.9696	134.9028	106.1015	137.3251
106.048	108.1043	105.9687	133.8077	105.9516	135.8869	106.0582	132.9693	105.9862	134.9014	106.1182	137.3249
106.0646	108.1031	105.9854	133.8074	105.9683	135.8898	106.0748	132.9671	106.0029	134.9032	106.1349	137.3241
106.0813	108.103	106.0021	133.8107	105.9849	135.8865	106.0915	132.9638	106.0196	134.8992	106.1515	137.3234
106.098	108.1002	106.0187	133.8052	106.0016	135.8851	106.1082	132.9641	106.0362	134.8987	106.1682	137.3262
106.1146	108.1021	106.0354	133.815	106.0183	135.8852	106.1248	132.9641	106.0529	134.8988	106.1849	137.3242
106.1313	108.0999	106.0521	133.807	106.0349	135.8885	106.1415	132.9634	106.0696	134.8984	106.2015	137.3222
106.148	108.1011	106.0687	133.8076	106.0516	135.884	106.1582	132.9622	106.0862	134.8987	106.2182	137.3234
106.1646	108.1027	106.0854	133.8115	106.0683	135.8833	106.1748	132.9617	106.1029	134.8994	106.2349	137.3213
106.1813	108.0979	106.1021	133.8061	106.0849	135.8856	106.1915	132.9576	106.1196	134.8969	106.2515	137.3191
106.198	108.0962	106.1187	133.8057	106.1016	135.8853	106.2082	132.9592	106.1362	134.8961	106.2682	137.3208
106.2146	108.0963	106.1354	133.8075	106.1183	135.8882	106.2248	132.9604	106.1529	134.8987	106.2849	137.3189
106.2313	108.0963	106.1521	133.8059	106.1349	135.8872	106.2415	132.9586	106.1696	134.8968	106.3015	137.3197
106.248	108.0964	106.1687	133.8054	106.1516	135.8861	106.2582	132.9598	106.1862	134.8937	106.3182	137.3184
106.2646	108.0966	106.1854	133.8057	106.1683	135.887	106.2748	132.956	106.2029	134.8946	106.3349	137.3164
106.2813	108.0939	106.2021	133.8058	106.1849	135.8858	106.2915	132.9538	106.2196	134.8946	106.3515	137.3137
106.298	108.0923	106.2187	133.8036	106.2016	135.8884	106.3082	132.9551	106.2362	134.8957	106.3682	137.3142
106.3146	108.0908	106.2354	133.8042	106.2183	135.8861	106.3248	132.9535	106.2529	134.894	106.3849	137.314
106.3313	108.0939	106.2521	133.8044	106.2349	135.889	106.3415	132.9516	106.2696	134.8932	106.4015	137.3135
106.348	108.0899	106.2687	133.8034	106.2516	135.8864	106.3582	132.9507	106.2862	134.8919	106.4182	137.3137
106.3646	108.0879	106.2854	133.8027	106.2683	135.8861	106.3748	132.9483	106.3029	134.8925	106.4349	137.314
106.3813	108.0908	106.3021	133.8019	106.2849	135.8863	106.3915	132.9461	106.3196	134.8917	106.4515	137.3091
106.398	108.0891	106.3187	133.8064	106.3016	135.885	106.4082	132.9479	106.3362	134.8918	106.4682	137.3112
106.4146	108.0871	106.3354	133.8013	106.3183	135.885	106.4248	132.9456	106.3529	134.8878	106.4849	137.3087
106.4313	108.0878	106.3521	133.8028	106.3349	135.8831	106.4415	132.9456	106.3696	134.89	106.5015	137.3066
106.448	108.0876	106.3687	133.8037	106.3516	135.8843	106.4582	132.9432	106.3862	134.8893	106.5182	137.3094
106.4646	108.0867	106.3854	133.8027	106.3683	135.8844	106.4748	132.9412	106.4029	134.8885	106.5349	137.3065
106.4813	108.0875	106.4021	133.8013	106.3849	135.8825	106.4915	132.942	106.4196	134.8874	106.5515	137.3085
106.498	108.0901	106.4187	133.8015	106.4016	135.8836	106.5082	132.94	106.4362	134.8853	106.5682	137.3053
106.5146	108.0832	106.4354	133.8006	106.4183	135.8803	106.5248	132.9387	106.4529	134.8858	106.5849	137.3074
106.5313	108.0841	106.4521	133.8005	106.4349	135.8819	106.5415	132.9386	106.4696	134.8875	106.6015	137.3032
106.548	108.0839	106.4687	133.7977	106.4516	135.8791	106.5582	132.9378	106.4862	134.8854	106.6182	137.3021
106.5646	108.0823	106.4854	133.8013	106.4683	135.8774	106.5748	132.9358	106.5029	134.8823	106.6349	137.3027
106.5813	108.0802	106.5021	133.8	106.4849	135.8789	106.5915	132.936	106.5196	134.8843	106.6515	137.3011
106.598	108.0811	106.5187	133.8003	106.5016	135.881	106.6082	132.9364	106.5362	134.8857	106.6682	137.3021
106.6146	108.0811	106.5354	133.8007	106.5183	135.8797	106.6248	132.9336	106.5529	134.882	106.6849	137.3001
106.6313	108.0779	106.5521	133.7992	106.5349	135.8764	106.6415	132.9336	106.5696	134.8824	106.7015	137.3033
106.648	108.0799	106.5687	133.7974	106.5516	135.8785	106.6582	132.9334	106.5862	134.8819	106.7182	137.2992
106.6646	108.0798	106.5854	133.7988	106.5683	135.8771	106.6748	132.9326	106.6029	134.8825	106.7349	137.2985
106.6813	108.077	106.6021	133.7976	106.5849	135.8778	106.6915	132.9348	106.6196	134.8844	106.7515	137.2964
106.698	108.0743	106.6187	133.7958	106.6016	135.8786	106.7082	132.93	106.6362	134.8836	106.7682	137.2967
106.7146	108.0761	106.6354	133.7972	106.6183	135.8769	106.7248	132.9309	106.6529	134.8821	106.7849	137.2958
106.7313	108.0752	106.6521	133.7999	106.6349	135.8771	106.7415	132.928	106.6696	134.8804	106.8015	137.296
106.748	108.074	106.6687	133.7954	106.6516	135.8735	106.7582	132.9288	106.6862	134.8829	106.8182	137.2951

106.7646	108.0739	106.6854	133.7974	106.6683	135.8781	106.7748	132.9266	106.7029	134.8808	106.8349	137.2943
106.7813	108.0735	106.7021	133.795	106.6849	135.8776	106.7915	132.9276	106.7196	134.8782	106.8515	137.2949
106.798	108.0736	106.7187	133.7949	106.7016	135.8748	106.8082	132.9254	106.7362	134.8792	106.8682	137.2927
106.8146	108.0704	106.7354	133.794	106.7183	135.877	106.8248	132.9248	106.7529	134.8772	106.8849	137.2929
106.8313	108.0729	106.7521	133.7963	106.7349	135.8747	106.8415	132.9245	106.7696	134.8794	106.9015	137.2933
106.848	108.0721	106.7687	133.7981	106.7516	135.8768	106.8582	132.9244	106.7862	134.8757	106.9182	137.2914
106.8646	108.0722	106.7854	133.7958	106.7683	135.8744	106.8748	132.9213	106.8029	134.8783	106.9349	137.2904
106.8813	108.0666	106.8021	133.7948	106.7849	135.8743	106.8915	132.9179	106.8196	134.8747	106.9515	137.2896
106.898	108.0704	106.8187	133.7976	106.8016	135.8723	106.9082	132.9211	106.8362	134.8765	106.9682	137.2887
106.9146	108.0693	106.8354	133.7956	106.8183	135.8722	106.9248	132.9189	106.8529	134.8755	106.9849	137.2902
106.9313	108.0663	106.8521	133.7926	106.8349	135.8681	106.9415	132.9209	106.8696	134.8752	107.0015	137.2906
106.948	108.0658	106.8687	133.7971	106.8516	135.8712	106.9582	132.9197	106.8862	134.8734	107.0182	137.2859
106.9646	108.067	106.8854	133.7973	106.8683	135.8696	106.9748	132.9184	106.9029	134.8745	107.0349	137.2867
106.9813	108.0638	106.9021	133.794	106.8849	135.8675	106.9915	132.9203	106.9196	134.8729	107.0515	137.287
106.998	108.0655	106.9187	133.7965	106.9016	135.8675	107.0082	132.9183	106.9362	134.873	107.0682	137.2868
107.0146	108.0613	106.9354	133.7976	106.9183	135.8674	107.0248	132.9182	106.9529	134.8714	107.0849	137.2856
107.0313	108.0643	106.9521	133.7983	106.9349	135.8677	107.0415	132.9165	106.9696	134.8716	107.1015	137.2853
107.048	108.0609	106.9687	133.7982	106.9516	135.8672	107.0582	132.9152	106.9862	134.8716	107.1182	137.2855
107.0646	108.0583	106.9854	133.7944	106.9683	135.8652	107.0748	132.9132	107.0029	134.8692	107.1349	137.2842
107.0813	108.0586	107.0021	133.7957	106.9849	135.8663	107.0915	132.9114	107.0196	134.8703	107.1515	137.2851
107.098	108.0551	107.0187	133.7976	107.0016	135.863	107.1082	132.913	107.0362	134.8681	107.1682	137.2845
107.1146	108.0563	107.0354	133.7963	107.0183	135.8638	107.1248	132.9112	107.0529	134.8683	107.1849	137.2811
107.1313	108.0558	107.0521	133.7944	107.0349	135.8647	107.1415	132.9108	107.0696	134.8652	107.2015	137.2825
107.148	108.0508	107.0687	133.7956	107.0516	135.8663	107.1582	132.911	107.0862	134.8652	107.2182	137.2792
107.1646	108.0548	107.0854	133.7943	107.0683	135.8639	107.1748	132.9091	107.1029	134.8683	107.2349	137.2814
107.1813	108.0505	107.1021	133.7954	107.0849	135.8636	107.1915	132.9081	107.1196	134.8651	107.2515	137.2815
107.198	108.0525	107.1187	133.7937	107.1016	135.862	107.2082	132.905	107.1362	134.8663	107.2682	137.2798
107.2146	108.0449	107.1354	133.7963	107.1183	135.8638	107.2248	132.9055	107.1529	134.8646	107.2849	137.2788
107.2313	108.0464	107.1521	133.7958	107.1349	135.8611	107.2415	132.9049	107.1696	134.8646	107.3015	137.2784
107.248	108.036	107.1687	133.797	107.1516	135.8586	107.2582	132.9026	107.1862	134.8612	107.3182	137.2767
107.2646	108.0492	107.1854	133.7971	107.1683	135.8596	107.2748	132.904	107.2029	134.863	107.3349	137.2766
107.2813	108.0455	107.2021	133.7946	107.1849	135.8609	107.2915	132.9027	107.2196	134.8584	107.3515	137.2767
107.298	108.0361	107.2187	133.7955	107.2016	135.8598	107.3082	132.9021	107.2362	134.8617	107.3682	137.2784
107.3146	108.0488	107.2354	133.7923	107.2183	135.8581	107.3248	132.9016	107.2529	134.8604	107.3849	137.275
107.3313	108.0442	107.2521	133.7953	107.2349	135.8558	107.3415	132.8996	107.2696	134.8587	107.4015	137.2743
107.348	108.0388	107.2687	133.7945	107.2516	135.8584	107.3582	132.9022	107.2862	134.8558	107.4182	137.2738
107.3646	108.0376	107.2854	133.7961	107.2683	135.8545	107.3748	132.8989	107.3029	134.8579	107.4349	137.2727
107.3813	108.0419	107.3021	133.7919	107.2849	135.8563	107.3915	132.897	107.3196	134.8569	107.4515	137.2727
107.398	108.0385	107.3187	133.7955	107.3016	135.8539	107.4082	132.8966	107.3362	134.8555	107.4682	137.2705
107.4146	108.0373	107.3354	133.7904	107.3183	135.8569	107.4248	132.8937	107.3529	134.8561	107.4849	137.2688
107.4313	108.0311	107.3521	133.7928	107.3349	135.8543	107.4415	132.8986	107.3696	134.854	107.5015	137.2679
107.448	108.0331	107.3687	133.7931	107.3516	135.8564	107.4582	132.8968	107.3862	134.856	107.5182	137.2698
107.4646	108.0337	107.3854	133.7936	107.3683	135.8541	107.4748	132.8958	107.4029	134.854	107.5349	137.2681
107.4813	108.0341	107.4021	133.7909	107.3849	135.8538	107.4915	132.896	107.4196	134.8531	107.5515	137.266
107.498	108.0276	107.4187	133.7971	107.4016	135.8538	107.5082	132.89	107.4362	134.853	107.5682	137.2664
107.5146	108.0308	107.4354	133.7905	107.4183	135.8517	107.5248	132.8923	107.4529	134.8534	107.5849	137.2639
107.5313	108.0254	107.4521	133.7932	107.4349	135.8535	107.5415	132.8931	107.4696	134.8527	107.6015	137.2645
107.548	108.0343	107.4687	133.7914	107.4516	135.8533	107.5582	132.8905	107.4862	134.8505	107.6182	137.2643
107.5646	108.0215	107.4854	133.7928	107.4683	135.8499	107.5748	132.8891	107.5029	134.8515	107.6349	137.2641
107.5813	108.0303	107.5021	133.7919	107.4849	135.849	107.5915	132.8886	107.5196	134.8496	107.6515	137.2625
107.598	108.0247	107.5187	133.79	107.5016	135.8479	107.6082	132.8871	107.5362	134.8504	107.6682	137.2631
107.6146	108.0252	107.5354	133.7888	107.5183	135.8471	107.6248	132.887	107.5529	134.8489	107.6849	137.2624
107.6313	108.0244	107.5521	133.789	107.5349	135.8464	107.6415	132.8874	107.5696	134.8499	107.7015	137.2602
107.648	108.0186	107.5687	133.7909	107.5516	135.8467	107.6582	132.8852	107.5862	134.8491	107.7182	137.2595
107.6646	108.0207	107.5854	133.7893	107.5683	135.8412	107.6748	132.884	107.6029	134.849	107.7349	137.262
107.6813	108.0272	107.6021	133.7927	107.5849	135.8446	107.6915	132.884	107.6196	134.8483	107.7515	137.2574
107.698	108.0192	107.6187	133.7854	107.6016	135.8451	107.7082	132.8838	107.6362	134.8477	107.7682	137.2567
107.7146	108.0217	107.6354	133.788	107.6183	135.8403	107.7248	132.883	107.6529	134.8482	107.7849	137.2568
107.7313	108.0181	107.6521	133.789	107.6349	135.8404	107.7415	132.8798	107.6696	134.8468	107.8015	137.2554
107.748	108.0146	107.6687	133.79	107.6516	135.8418	107.7582	132.8826	107.6862	134.8466	107.8182	137.2538
107.7646	108.0167	107.6854	133.7864	107.6683	135.8399	107.7748	132.8807	107.7029	134.8444	107.8349	137.2551
107.7813	108.0184	107.7021	133.7892	107.6849	135.837	107.7915	132.8813	107.7196	134.844	107.8515	137.2515
107.798	108.0152	107.7187	133.787	107.7016	135.8402	107.8082	132.8796	107.7362	134.8429	107.8682	137.2545
107.8146	108.0153	107.7354	133.7852	107.7183	135.8359	107.8248	132.8781	107.7529	134.844	107.8849	137.2507
107.8313	108.013	107.7521	133.7875	107.7349	135.8339	107.8415	132.8788	107.7696	134.8422	107.9015	137.2506
107.848	108.0134	107.7687	133.7849	107.7516	135.8356	107.8582	132.8794	107.7862	134.8406	107.9182	137.2517
107.8646	108.0097	107.7854	133.7856	107.7683	135.8366	107.8748	132.8777	107.8029	134.8429	107.9349	137.2481
107.8813	108.0104	107.8021	133.7859	107.7849	135.8354	107.8915	132.8769	107.8196	134.8417	107.9515	137.2468
107.898	108.009	107.8187	133.7867	107.8016	135.8352	107.9082	132.8772	107.8362	134.8396	107.9682	137.2466
107.9146	108.0114	107.8354	133.7818	107.8183	135.8345	107.9248	132.8734	107.8529	134.8382	107.9849	137.2463
107.9313	108.0086	107.8521	133.7833	107.8349	135.8333	107.9415	132.8756	107.8696	134.8377	108.0015	137.2472
107.948	108.0067	107.8687	133.784	107.8516	135.8326	107.9582	132.8737	107.8862	134.8382	108.0182	137.246
107.9646	108.0056	107.8854	133.7837	107.8683	135.8343	107.9748	132.8753	107.9029	134.8382	108.0349	137.243
107.9813	108.0068	107.9021	133.7814	107.8849	135.8348	107.9915	132.8762	107.9196	134.8355	108.0515	137.2419
107.998	108.0041	107.9187	133.7836	107.9016	135.8351	108.0082	132.8745	107.9362	134.8363	108.0682	137.2415
108.0146	108.0017	107.9354	133.7805	107.9183	135.8321	108.0248	132.8741	107.9529	134.8383	108.0849	137.2405
108.0313	108.0022	107.9521	133.7821	107.9349	135.8319	108.0415	132.8756	107.9696	134.8359	108.1015	137.2444
108.048	108.0012	107.9687	133.7799	107.9516	135.8323	108.0582	132.876	107.9862	134.8321	108.1182	137.2394
108.0646	108	107.9854	133.7825	107.9683	135.8293	108.0748	132.8742	108.0029	134.8359	108.1349	137.2383
108.0813	108.0019	108.0021	133.7817	107.9849	135.8313	108.0915	132.8743	108.0196	134.8318	108.1515	137.2417
108.098	108.0024	108.0187	133.7829	108.0016	135.8297	108.1082	132.8727	108.0362	134.8341	108.1682	137.239
108.1146	107.9961	108.0354	133.7781	108.0183	135.8293	108.1248	132.8665	108.0529	134.8328	108.1849	137.2381
108.1313	108.0002	108.0521	133.7784	108.0349	135.8261	108.1415	132.8732	108.0696	134.8322	108.2015	137.2381
108.148	107.9993	108.0687	133.7799	108.0516	135.8302	108.1582	132.8682	108.0862	134.8311	108.2182	137.2366
108.1646	107.9972	108.0854	133.7806	108.0683	135.8299	108.1748	132.8668	108.1029	134.8319	108.2349	137.2379
108.1813	107.9981	108.1021	133.7776	108.0849	135.8279	108.1915	132.8681	108.1196	134.8309	108.2515	137.2356
108.198	107.9964	108.1187	133.777	108.1016	135.8245	108.2082	132.8667	108.1362	134.8302	108.2682	137.2349
108.2146	107.9925	108.1354	133.7774	108.1183	135.8271	108.2248	132.8656	108.1529	134.8311	108.2849	137.2324
108.2313	107.9937	108.1521	133.7779	108.1349	135.8239	108.2415	132.8628	108.1696	134.8308	108.3015	137.2336
108.248	107.9938	108.1687	133.775	108.1516	135.8273	108.2582	132.8627	108.1862	134.8296	108.3182	137.2315
108.2646	107.9922	108.1854	133.7756	108.1683	135.8239	108.2748	132.8619	108.2029	134.8278	108.3349	137.2327
108.2813	107.9976	108.2021	133.7759	108.1849	135.8233	108.2915	132.8609	108.2196	134.8275	108.3515	137.2301
108.298	107.9899	108.2187	133.7746	108.2016	135.8241	108.3082	132.8623	108.2362	134.8275	108.3682	137.2333
108.3146	107.9914	108.2354	133.7718	108.2183	135.8201	108.3248	132.8615	108.2529	134.8265	108.3849	137.2293
108.3313	107.9874	108.2521	133.7732	108.2349	135.8211	108.3415	132.8587	108.2696	134.8273	108.4015	137.232
108.348	107.989	108.2687	133.7726	108.2516	135.8217	108.3582	132.8569	108.2862	134.8261	108.4182	137.2286
108.3646	107.9871	108.2854	133.7751	108.2683	135.8221	108.3748	132.8586	108.3029	134.8229	108.4349	137.2299

108.3813	107.9851	108.3021	133.7701	108.2849	135.8192	108.3915	132.8573	108.3196	134.8242	108.4515	137.229
108.398	107.9862	108.3187	133.7736	108.3016	135.8194	108.4082	132.8555	108.3362	134.8223	108.4682	137.2293
108.4146	107.9836	108.3354	133.7751	108.3183	135.8226	108.4248	132.8567	108.3529	134.822	108.4849	137.2283
108.4313	107.984	108.3521	133.7719	108.3349	135.8193	108.4415	132.8533	108.3696	134.822	108.5015	137.2272
108.448	107.9819	108.3687	133.7723	108.3516	135.8163	108.4582	132.8535	108.3862	134.8213	108.5182	137.2258
108.4646	107.9833	108.3854	133.7734	108.3683	135.8191	108.4748	132.854	108.4029	134.8199	108.5349	137.2277
108.4813	107.9818	108.4021	133.7702	108.3849	135.8172	108.4915	132.8529	108.4196	134.8198	108.5515	137.2246
108.498	107.9786	108.4187	133.7706	108.4016	135.8185	108.5082	132.8531	108.4362	134.8201	108.5682	137.227
108.5146	107.9805	108.4354	133.7679	108.4183	135.8189	108.5248	132.855	108.4529	134.8181	108.5849	137.225
108.5313	107.9802	108.4521	133.77	108.4349	135.8199	108.5415	132.8508	108.4696	134.8181	108.6015	137.2251
108.548	107.9796	108.4687	133.7695	108.4516	135.8169	108.5582	132.8504	108.4862	134.8191	108.6182	137.2226
108.5646	107.9774	108.4854	133.7693	108.4683	135.818	108.5748	132.8513	108.5029	134.8176	108.6349	137.225
108.5813	107.9785	108.5021	133.7677	108.4849	135.8152	108.5915	132.8504	108.5196	134.8161	108.6515	137.2224
108.598	107.9789	108.5187	133.7678	108.5016	135.8148	108.6082	132.8497	108.5362	134.8168	108.6682	137.2218
108.6146	107.978	108.5354	133.7662	108.5183	135.8174	108.6248	132.848	108.5529	134.8157	108.6849	137.2206
108.6313	107.973	108.5521	133.7668	108.5349	135.812	108.6415	132.8465	108.5696	134.8155	108.7015	137.2225
108.648	107.9737	108.5687	133.765	108.5516	135.8125	108.6582	132.8477	108.5862	134.814	108.7182	137.2205
108.6646	107.9747	108.5854	133.7679	108.5683	135.8174	108.6748	132.8462	108.6029	134.8153	108.7349	137.2198
108.6813	107.9715	108.6021	133.767	108.5849	135.8134	108.6915	132.8466	108.6196	134.8134	108.7515	137.2194
108.698	107.9735	108.6187	133.766	108.6016	135.812	108.7082	132.8442	108.6362	134.8135	108.7682	137.2182
108.7146	107.9716	108.6354	133.7667	108.6183	135.8124	108.7248	132.8436	108.6529	134.812	108.7849	137.217
108.7313	107.9715	108.6521	133.7654	108.6349	135.8098	108.7415	132.8449	108.6696	134.8115	108.8015	137.2175
108.748	107.9664	108.6687	133.7635	108.6516	135.8095	108.7582	132.8435	108.6862	134.8112	108.8182	137.216
108.7646	107.9672	108.6854	133.764	108.6683	135.8096	108.7748	132.8421	108.7029	134.8117	108.8349	137.2153
108.7813	107.9668	108.7021	133.7632	108.6849	135.8073	108.7915	132.8421	108.7196	134.8089	108.8515	137.2154
108.798	107.964	108.7187	133.764	108.7016	135.8044	108.8082	132.8411	108.7362	134.809	108.8682	137.2145
108.8146	107.9626	108.7354	133.7632	108.7183	135.8028	108.8248	132.8401	108.7529	134.8114	108.8849	137.2119
108.8313	107.9637	108.7521	133.7632	108.7349	135.8052	108.8415	132.8413	108.7696	134.8079	108.9015	137.2137
108.848	107.9616	108.7687	133.7635	108.7516	135.798	108.8582	132.8395	108.7862	134.808	108.9182	137.2104
108.8646	107.963	108.7854	133.7627	108.7683	135.8021	108.8748	132.8379	108.8029	134.8086	108.9349	137.2098
108.8813	107.9626	108.8021	133.7599	108.7849	135.7998	108.8915	132.8401	108.8196	134.8083	108.9515	137.211
108.898	107.9594	108.8187	133.7614	108.8016	135.7989	108.9082	132.837	108.8362	134.807	108.9682	137.2109
108.9146	107.9594	108.8354	133.7596	108.8183	135.8021	108.9248	132.8388	108.8529	134.8054	108.9849	137.2102
108.9313	107.9595	108.8521	133.759	108.8349	135.8005	108.9415	132.8352	108.8696	134.8056	109.0015	137.2098
108.948	107.9594	108.8687	133.7592	108.8516	135.7976	108.9582	132.8354	108.8862	134.8058	109.0182	137.2076
108.9646	107.9562	108.8854	133.7598	108.8683	135.7968	108.9748	132.8337	108.9029	134.8047	109.0349	137.2077
108.9813	107.9581	108.9021	133.7581	108.8849	135.7978	108.9915	132.835	108.9196	134.8033	109.0515	137.2059
108.998	107.9583	108.9187	133.7609	108.9016	135.7965	109.0082	132.835	108.9362	134.8019	109.0682	137.2042
109.0146	107.9564	108.9354	133.7582	108.9183	135.7976	109.0248	132.8329	108.9529	134.8065	109.0849	137.2028
109.0313	107.9581	108.9521	133.7582	108.9349	135.7946	109.0415	132.835	108.9696	134.8021	109.1015	137.205
109.048	107.9549	108.9687	133.758	108.9516	135.7926	109.0582	132.8333	108.9862	134.7981	109.1182	137.2044
109.0646	107.9528	108.9854	133.7568	108.9683	135.7936	109.0748	132.8333	109.0029	134.8034	109.1349	137.2
109.0813	107.9535	109.0021	133.7562	108.9849	135.7922	109.0915	132.8327	109.0196	134.8021	109.1515	137.2021
109.098	107.9526	109.0187	133.756	109.0016	135.7919	109.1082	132.8322	109.0362	134.7963	109.1682	137.2014
109.1146	107.952	109.0354	133.7551	109.0183	135.7912	109.1248	132.8322	109.0529	134.7986	109.1849	137.1979
109.1313	107.9498	109.0521	133.7553	109.0349	135.792	109.1415	132.8309	109.0696	134.7986	109.2015	137.199
109.148	107.95	109.0687	133.7554	109.0516	135.788	109.1582	132.8311	109.0862	134.7977	109.2182	137.1996
109.1646	107.9491	109.0854	133.7585	109.0683	135.79	109.1748	132.8269	109.1029	134.7976	109.2349	137.2003
109.1813	107.9495	109.1021	133.7525	109.0849	135.7909	109.1915	132.8303	109.1196	134.7967	109.2515	137.1972
109.198	107.9478	109.1187	133.7528	109.1016	135.7862	109.2082	132.8293	109.1362	134.797	109.2682	137.1963
109.2146	107.9476	109.1354	133.7509	109.1183	135.7869	109.2248	132.8271	109.1529	134.7958	109.2849	137.1953
109.2313	107.9438	109.1521	133.7524	109.1349	135.7881	109.2415	132.8267	109.1696	134.7972	109.3015	137.1981
109.248	107.9459	109.1687	133.7526	109.1516	135.7881	109.2582	132.8293	109.1862	134.7955	109.3182	137.1964
109.2646	107.9454	109.1854	133.7528	109.1683	135.7861	109.2748	132.8248	109.2029	134.7946	109.3349	137.1939
109.2813	107.9419	109.2021	133.7519	109.1849	135.786	109.2915	132.8261	109.2196	134.7936	109.3515	137.1933
109.298	107.9431	109.2187	133.75	109.2016	135.786	109.3082	132.8229	109.2362	134.794	109.3682	137.1921
109.3146	107.9451	109.2354	133.75	109.2183	135.7863	109.3248	132.8262	109.2529	134.792	109.3849	137.1923
109.3313	107.9415	109.2521	133.7504	109.2349	135.784	109.3415	132.8235	109.2696	134.7921	109.4015	137.1902
109.348	107.9406	109.2687	133.7489	109.2516	135.7816	109.3582	132.8262	109.2862	134.7898	109.4182	137.1907
109.3646	107.9381	109.2854	133.7501	109.2683	135.7826	109.3748	132.8249	109.3029	134.7923	109.4349	137.1887
109.3813	107.939	109.3021	133.7496	109.2849	135.7808	109.3915	132.8239	109.3196	134.7901	109.4515	137.1876
109.398	107.9381	109.3187	133.7495	109.3016	135.7847	109.4082	132.8237	109.3362	134.7886	109.4682	137.1889
109.4146	107.9368	109.3354	133.7499	109.3183	135.7829	109.4248	132.8238	109.3529	134.7887	109.4849	137.1878
109.4313	107.9387	109.3521	133.7508	109.3349	135.7801	109.4415	132.823	109.3696	134.787	109.5015	137.187
109.448	107.9334	109.3687	133.7483	109.3516	135.7806	109.4582	132.8239	109.3862	134.7876	109.5182	137.1858
109.4646	107.9385	109.3854	133.7466	109.3683	135.7785	109.4748	132.8216	109.4029	134.7863	109.5349	137.1864
109.4813	107.9319	109.4021	133.7477	109.3849	135.7813	109.4915	132.8223	109.4196	134.7859	109.5515	137.1851
109.498	107.9333	109.4187	133.7505	109.4016	135.779	109.5082	132.822	109.4362	134.7859	109.5682	137.1838
109.5146	107.9366	109.4354	133.747	109.4183	135.781	109.5248	132.8237	109.4529	134.7822	109.5849	137.1815
109.5313	107.9309	109.4521	133.7471	109.4349	135.7796	109.5415	132.8213	109.4696	134.7837	109.6015	137.1804
109.548	107.9332	109.4687	133.7462	109.4516	135.7785	109.5582	132.821	109.4862	134.784	109.6182	137.181
109.5646	107.9302	109.4854	133.7447	109.4683	135.7773	109.5748	132.8219	109.5029	134.7824	109.6349	137.1803
109.5813	107.9288	109.5021	133.7437	109.4849	135.7758	109.5915	132.8189	109.5196	134.7828	109.6515	137.1801
109.598	107.9306	109.5187	133.743	109.5016	135.7759	109.6082	132.8166	109.5362	134.781	109.6682	137.178
109.6146	107.9305	109.5354	133.7442	109.5183	135.7755	109.6248	132.8178	109.5529	134.7806	109.6849	137.1762
109.6313	107.9265	109.5521	133.7447	109.5349	135.7743	109.6415	132.8155	109.5696	134.7803	109.7015	137.178
109.648	107.9288	109.5687	133.7448	109.5516	135.7724	109.6582	132.8157	109.5862	134.7791	109.7182	137.1764
109.6646	107.9264	109.5854	133.741	109.5683	135.7732	109.6748	132.8153	109.6029	134.7778	109.7349	137.178
109.6813	107.9243	109.6021	133.7421	109.5849	135.7758	109.6915	132.815	109.6196	134.7788	109.7515	137.1742
109.698	107.9224	109.6187	133.7405	109.6016	135.7706	109.7082	132.8158	109.6362	134.7776	109.7682	137.1759
109.7146	107.9235	109.6354	133.7404	109.6183	135.7716	109.7248	132.8134	109.6529	134.7771	109.7849	137.1737
109.7313	107.9214	109.6521	133.7421	109.6349	135.7716	109.7415	132.8122	109.6696	134.7755	109.8015	137.1752
109.748	107.9251	109.6687	133.7391	109.6516	135.7693	109.7582	132.814	109.6862	134.7777	109.8182	137.1714
109.7646	107.9266	109.6854	133.738	109.6683	135.7679	109.7748	132.8126	109.7029	134.7771	109.8349	137.173
109.7813	107.9219	109.7021	133.7404	109.6849	135.7687	109.7915	132.8119	109.7196	134.775	109.8515	137.1712
109.798	107.9227	109.7187	133.7363	109.7016	135.7691	109.8082	132.8113	109.7362	134.7725	109.8682	137.1701
109.8146	107.9183	109.7354	133.7377	109.7183	135.7687	109.8248	132.813	109.7529	134.772	109.8849	137.1709
109.8313	107.9167	109.7521	133.7366	109.7349	135.7669	109.8415	132.8139	109.7696	134.7731	109.9015	137.1707
109.848	107.9167	109.7687	133.7381	109.7516	135.7662	109.8582	132.81	109.7862	134.7729	109.9182	137.1668
109.8646	107.9126	109.7854	133.7363	109.7683	135.7676	109.8748	132.8114	109.8029	134.7731	109.9349	137.1691
109.8813	107.9174	109.8021	133.7349	109.7849	135.7676	109.8915	132.8122	109.8196	134.7691	109.9515	137.1674
109.898	107.9136	109.8187	133.7324	109.8016	135.7653	109.9082	132.809	109.8362	134.7719	109.9682	137.1652
109.9146	107.9146	109.8354	133.7308	109.8183	135.7653	109.9248	132.8083	109.8529	134.7706	109.9849	137.1657
109.9313	107.91	109.8521	133.7338	109.8349	135.7683	109.9415	132.8092	109.8696	134.7719	110.0015	137.166
109.948	107.9099	109.8687	133.7307	109.8516	135.7652	109.9582	132.8054	109.8862	134.769	110.0182	137.165
109.9646	107.9086	109.8854	133.7339	109.8683	135.7641	109.9748	132.8061	109.9029	134.7717	110.0349	137.1641
109.9813	107.9123	109.9021	133.7284	109.8849	135.7662	109.9915	132.8077	109.9196	134.7681	110.0515	137.1615

109.998	107.9053	109.9187	133.7327	109.9016	135.7644	110.0082	132.8085	109.9362	134.7672	110.0682	137.1605
110.0146	107.9073	109.9354	133.7285	109.9183	135.7639	110.0248	132.8045	109.9529	134.7655	110.0849	137.1612
110.0313	107.9056	109.9521	133.7314	109.9349	135.764	110.0415	132.805	109.9696	134.7669	110.1015	137.1606
110.048	107.9039	109.9687	133.7284	109.9516	135.7638	110.0582	132.8074	109.9862	134.7667	110.1182	137.1576
110.0646	107.9053	109.9854	133.7297	109.9683	135.7634	110.0748	132.806	110.0029	134.7653	110.1349	137.1586
110.0813	107.9062	110.0021	133.7284	109.9849	135.7626	110.0915	132.8043	110.0196	134.7658	110.1515	137.1579
110.098	107.9058	110.0187	133.729	110.0016	135.7613	110.1082	132.8033	110.0362	134.7632	110.1682	137.1572
110.1146	107.9008	110.0354	133.7282	110.0183	135.7589	110.1248	132.8055	110.0529	134.7643	110.1849	137.1566
110.1313	107.9038	110.0521	133.7286	110.0349	135.7623	110.1415	132.8017	110.0696	134.7626	110.2015	137.1576
110.148	107.902	110.0687	133.7279	110.0516	135.7606	110.1582	132.8021	110.0862	134.7598	110.2182	137.1565
110.1646	107.8995	110.0854	133.725	110.0683	135.7584	110.1748	132.8017	110.1029	134.7617	110.2349	137.1573
110.1813	107.9004	110.1021	133.7244	110.0849	135.7609	110.1915	132.802	110.1196	134.7637	110.2515	137.1555
110.198	107.897	110.1187	133.7251	110.1016	135.7587	110.2082	132.8	110.1362	134.7597	110.2682	137.1545
110.2146	107.8977	110.1354	133.7239	110.1183	135.7586	110.2248	132.7991	110.1529	134.7602	110.2849	137.1533
110.2313	107.898	110.1521	133.7234	110.1349	135.7593	110.2415	132.799	110.1696	134.7606	110.3015	137.1553
110.248	107.8957	110.1687	133.7242	110.1516	135.759	110.2582	132.7994	110.1862	134.7576	110.3182	137.153
110.2646	107.8943	110.1854	133.7236	110.1683	135.7562	110.2748	132.7985	110.2029	134.7586	110.3349	137.1516
110.2813	107.8927	110.2021	133.7215	110.1849	135.7601	110.2915	132.7995	110.2196	134.7576	110.3515	137.1523
110.298	107.8928	110.2187	133.7208	110.2016	135.7579	110.3082	132.7981	110.2362	134.7564	110.3682	137.1529
110.3146	107.8939	110.2354	133.7191	110.2183	135.7578	110.3248	132.7957	110.2529	134.7577	110.3849	137.153
110.3313	107.8885	110.2521	133.7211	110.2349	135.7576	110.3415	132.7985	110.2696	134.7566	110.4015	137.1518
110.348	107.8925	110.2687	133.7173	110.2516	135.7581	110.3582	132.798	110.2862	134.7566	110.4182	137.1514
110.3646	107.8909	110.2854	133.7195	110.2683	135.7579	110.3748	132.7953	110.3029	134.7556	110.4349	137.15
110.3813	107.8913	110.3021	133.72	110.2849	135.7569	110.3915	132.7958	110.3196	134.7555	110.4515	137.1509
110.398	107.8907	110.3187	133.7196	110.3016	135.7553	110.4082	132.7944	110.3362	134.7543	110.4682	137.1501
110.4146	107.8897	110.3354	133.7175	110.3183	135.7582	110.4248	132.7946	110.3529	134.7534	110.4849	137.1478
110.4313	107.8845	110.3521	133.7212	110.3349	135.7563	110.4415	132.7924	110.3696	134.7552	110.5015	137.1481
110.448	107.8851	110.3687	133.7198	110.3516	135.7561	110.4582	132.7957	110.3862	134.7519	110.5182	137.1471
110.4646	107.8833	110.3854	133.72	110.3683	135.7558	110.4748	132.7922	110.4029	134.7531	110.5349	137.1478
110.4813	107.8865	110.4021	133.7204	110.3849	135.7546	110.4915	132.7915	110.4196	134.7531	110.5515	137.1467
110.498	107.8829	110.4187	133.719	110.4016	135.7517	110.5082	132.7912	110.4362	134.7507	110.5682	137.148
110.5146	107.8846	110.4354	133.7166	110.4183	135.7521	110.5248	132.7923	110.4529	134.7544	110.5849	137.1465
110.5313	107.8818	110.4521	133.7201	110.4349	135.7508	110.5415	132.7878	110.4696	134.751	110.6015	137.1445
110.548	107.883	110.4687	133.7196	110.4516	135.7513	110.5582	132.7912	110.4862	134.7522	110.6182	137.1433
110.5646	107.8819	110.4854	133.718	110.4683	135.7486	110.5748	132.7888	110.5029	134.752	110.6349	137.1462
110.5813	107.8767	110.5021	133.7169	110.4849	135.7525	110.5915	132.7891	110.5196	134.7493	110.6515	137.1441
110.598	107.8765	110.5187	133.7177	110.5016	135.7506	110.6082	132.7882	110.5362	134.7496	110.6682	137.1449
110.6146	107.8768	110.5354	133.7152	110.5183	135.7507	110.6248	132.7886	110.5529	134.7511	110.6849	137.1434
110.6313	107.8756	110.5521	133.7172	110.5349	135.7498	110.6415	132.7883	110.5696	134.7491	110.7015	137.1455
110.648	107.874	110.5687	133.7163	110.5516	135.7512	110.6582	132.7867	110.5862	134.7496	110.7182	137.1423
110.6646	107.8753	110.5854	133.7156	110.5683	135.7543	110.6748	132.7843	110.6029	134.7492	110.7349	137.1444
110.6813	107.8741	110.6021	133.7153	110.5849	135.7515	110.6915	132.7842	110.6196	134.7476	110.7515	137.1432
110.698	107.8753	110.6187	133.7155	110.6016	135.7489	110.7082	132.783	110.6362	134.7449	110.7682	137.1443
110.7146	107.868	110.6354	133.7145	110.6183	135.749	110.7248	132.7842	110.6529	134.7472	110.7849	137.1425
110.7313	107.8683	110.6521	133.7141	110.6349	135.7472	110.7415	132.7808	110.6696	134.7472	110.8015	137.1408
110.748	107.8698	110.6687	133.7145	110.6516	135.7519	110.7582	132.7805	110.6862	134.7444	110.8182	137.1419
110.7646	107.868	110.6854	133.7138	110.6683	135.746	110.7748	132.7797	110.7029	134.7447	110.8349	137.1436
110.7813	107.8673	110.7021	133.7116	110.6849	135.7491	110.7915	132.7782	110.7196	134.7437	110.8515	137.1416
110.798	107.8658	110.7187	133.7124	110.7016	135.746	110.8082	132.7776	110.7362	134.7416	110.8682	137.1418
110.8146	107.864	110.7354	133.7104	110.7183	135.746	110.8248	132.7781	110.7529	134.7403	110.8849	137.1415
110.8313	107.8628	110.7521	133.7137	110.7349	135.7458	110.8415	132.7769	110.7696	134.7411	110.9015	137.1421
110.848	107.8603	110.7687	133.7118	110.7516	135.7454	110.8582	132.7773	110.7862	134.74	110.9182	137.1408
110.8646	107.8613	110.7854	133.7088	110.7683	135.742	110.8748	132.7734	110.8029	134.7389	110.9349	137.1414
110.8813	107.8602	110.8021	133.7087	110.7849	135.7437	110.8915	132.7755	110.8196	134.7402	110.9515	137.1412
110.898	107.8611	110.8187	133.7116	110.8016	135.7402	110.9082	132.7722	110.8362	134.7364	110.9682	137.1398
110.9146	107.8577	110.8354	133.709	110.8183	135.7403	110.9248	132.773	110.8529	134.7386	110.9849	137.1387
110.9313	107.8566	110.8521	133.7075	110.8349	135.7372	110.9415	132.7727	110.8696	134.7388	111.0015	137.1401
110.948	107.8548	110.8687	133.7093	110.8516	135.7347	110.9582	132.771	110.8862	134.7352	111.0182	137.1391
110.9646	107.8563	110.8854	133.7063	110.8683	135.7352	110.9748	132.7737	110.9029	134.7366	111.0349	137.1393
110.9813	107.8542	110.9021	133.7048	110.8849	135.7326	110.9915	132.7709	110.9196	134.7348	111.0515	137.1356
110.998	107.8548	110.9187	133.7066	110.9016	135.7335	111.0082	132.7712	110.9362	134.734	111.0682	137.1367
111.0146	107.8556	110.9354	133.7049	110.9183	135.7291	111.0248	132.7712	110.9529	134.7347	111.0849	137.1342
111.0313	107.8527	110.9521	133.7033	110.9349	135.727	111.0415	132.773	110.9696	134.7315	111.1015	137.1366
111.048	107.8509	110.9687	133.7035	110.9516	135.7288	111.0582	132.7691	110.9862	134.7317	111.1182	137.1349
111.0646	107.8505	110.9854	133.7041	110.9683	135.724	111.0748	132.7699	111.0029	134.7305	111.1349	137.1341
111.0813	107.8525	111.0021	133.7009	110.9849	135.7231	111.0915	132.7705	111.0196	134.7312	111.1515	137.1343
111.098	107.8483	111.0187	133.7065	111.0016	135.7246	111.1082	132.7698	111.0362	134.7318	111.1682	137.1329
111.1146	107.85	111.0354	133.7004	111.0183	135.7208	111.1248	132.7669	111.0529	134.728	111.1849	137.1331
111.1313	107.848	111.0521	133.702	111.0349	135.7227	111.1415	132.7681	111.0696	134.7277	111.2015	137.1326
111.148	107.8466	111.0687	133.7053	111.0516	135.7216	111.1582	132.7682	111.0862	134.7278	111.2182	137.1308
111.1646	107.8436	111.0854	133.7002	111.0683	135.7219	111.1748	132.7669	111.1029	134.7279	111.2349	137.1325
111.1813	107.8468	111.1021	133.6989	111.0849	135.7192	111.1915	132.7688	111.1196	134.7281	111.2515	137.1309
111.198	107.8429	111.1187	133.7015	111.1016	135.7163	111.2082	132.7655	111.1362	134.7259	111.2682	137.1315
111.2146	107.8415	111.1354	133.6999	111.1183	135.7179	111.2248	132.764	111.1529	134.7255	111.2849	137.1298
111.2313	107.8452	111.1521	133.6969	111.1349	135.7164	111.2415	132.7634	111.1696	134.726	111.3015	137.1314
111.248	107.8412	111.1687	133.6994	111.1516	135.7174	111.2582	132.7664	111.1862	134.7264	111.3182	137.1287
111.2646	107.8382	111.1854	133.6987	111.1683	135.7166	111.2748	132.7616	111.2029	134.7245	111.3349	137.1286
111.2813	107.8428	111.2021	133.6962	111.1849	135.7141	111.2915	132.7613	111.2196	134.7248	111.3515	137.1279
111.298	107.8361	111.2187	133.6999	111.2016	135.7157	111.3082	132.7638	111.2362	134.7217	111.3682	137.1279
111.3146	107.8414	111.2354	133.6985	111.2183	135.7151	111.3248	132.7635	111.2529	134.7218	111.3849	137.1282
111.3313	107.8377	111.2521	133.6965	111.2349	135.7129	111.3415	132.7608	111.2696	134.7219	111.4015	137.1278
111.348	107.8399	111.2687	133.6956	111.2516	135.7143	111.3582	132.7593	111.2862	134.723	111.4182	137.1276
111.3646	107.831	111.2854	133.6971	111.2683	135.7131	111.3748	132.7572	111.3029	134.7213	111.4349	137.1259
111.3813	107.8378	111.3021	133.6947	111.2849	135.7139	111.3915	132.7595	111.3196	134.7208	111.4515	137.1257
111.398	107.8339	111.3187	133.6951	111.3016	135.7131	111.4082	132.7616	111.3362	134.7198	111.4682	137.1267
111.4146	107.8318	111.3354	133.6967	111.3183	135.7122	111.4248	132.7567	111.3529	134.7192	111.4849	137.1231
111.4313	107.8317	111.3521	133.6953	111.3349	135.7119	111.4415	132.7582	111.3696	134.7182	111.5015	137.1249
111.448	107.8301	111.3687	133.6985	111.3516	135.7152	111.4582	132.7593	111.3862	134.7219	111.5182	137.1241
111.4646	107.8285	111.3854	133.6925	111.3683	135.7118	111.4748	132.7559	111.4029	134.7174	111.5349	137.1214
111.4813	107.8283	111.4021	133.6942	111.3849	135.7094	111.4915	132.7573	111.4196	134.7169	111.5515	137.1224
111.498	107.8279	111.4187	133.6995	111.4016	135.7065	111.5082	132.7554	111.4362	134.7159	111.5682	137.1229
111.5146	107.8262	111.4354	133.6966	111.4183	135.7075	111.5248	132.7554	111.4529	134.7176	111.5849	137.1222
111.5313	107.8252	111.4521	133.6952	111.4349	135.7073	111.5415	132.7541	111.4696	134.7166	111.6015	137.1217
111.548	107.8295	111.4687	133.6952	111.4516	135.7067	111.5582	132.7535	111.4862	134.7139	111.6182	137.1205
111.5646	107.8233	111.4854	133.6953	111.4683	135.7068	111.5748	132.7541	111.5029	134.7136	111.6349	137.12
111.5813	107.8241	111.5021	133.6905	111.4849	135.7068	111.5915	132.7523	111.5196	134.715	111.6515	137.1222
111.598	107.8239	111.5187	133.6955	111.5016	135.7043	111.6082	132.7516	111.5362	134.7113	111.6682	137.1202

111.6146	107.8211	111.5354	133.6971	111.5183	135.7022	111.6248	132.7525	111.5529	134.7127	111.6849	137.1194
111.6313	107.8201	111.5521	133.6949	111.5349	135.7019	111.6415	132.7525	111.5696	134.7128	111.7015	137.1189
111.648	107.8217	111.5687	133.6925	111.5516	135.7041	111.6582	132.7523	111.5862	134.7134	111.7182	137.1199
111.6646	107.8212	111.5854	133.6901	111.5683	135.703	111.6748	132.7526	111.6029	134.7109	111.7349	137.1193
111.6813	107.8187	111.6021	133.6896	111.5849	135.7004	111.6915	132.7505	111.6196	134.7126	111.7515	137.1183
111.698	107.8194	111.6187	133.6904	111.6016	135.6997	111.7082	132.7538	111.6362	134.7089	111.7682	137.1183
111.7146	107.8158	111.6354	133.6922	111.6183	135.7008	111.7248	132.75	111.6529	134.7101	111.7849	137.1191
111.7313	107.8146	111.6521	133.6911	111.6349	135.6965	111.7415	132.7472	111.6696	134.7091	111.8015	137.119
111.748	107.8158	111.6687	133.6933	111.6516	135.6974	111.7582	132.7512	111.6862	134.7088	111.8182	137.1176
111.7646	107.8136	111.6854	133.6923	111.6683	135.6977	111.7748	132.7485	111.7029	134.7081	111.8349	137.118
111.7813	107.8112	111.7021	133.6897	111.6849	135.6971	111.7915	132.7511	111.7196	134.7083	111.8515	137.1165
111.798	107.8125	111.7187	133.6928	111.7016	135.6962	111.8082	132.7506	111.7362	134.7058	111.8682	137.1193
111.8146	107.8131	111.7354	133.6922	111.7183	135.6958	111.8248	132.7514	111.7529	134.7048	111.8849	137.1156
111.8313	107.8124	111.7521	133.6932	111.7349	135.6941	111.8415	132.7476	111.7696	134.7072	111.9015	137.1161
111.848	107.8063	111.7687	133.691	111.7516	135.6954	111.8582	132.7488	111.7862	134.7057	111.9182	137.1158
111.8646	107.8097	111.7854	133.6926	111.7683	135.6998	111.8748	132.7495	111.8029	134.7033	111.9349	137.1173
111.8813	107.8097	111.8021	133.6909	111.7849	135.6936	111.8915	132.7495	111.8196	134.7024	111.9515	137.117
111.898	107.8092	111.8187	133.6896	111.8016	135.6942	111.9082	132.7463	111.8362	134.7038	111.9682	137.1148
111.9146	107.8104	111.8354	133.6904	111.8183	135.6935	111.9248	132.7493	111.8529	134.7035	111.9849	137.116
111.9313	107.8061	111.8521	133.6906	111.8349	135.6918	111.9415	132.7467	111.8696	134.701	112.0015	137.1139
111.948	107.8047	111.8687	133.69	111.8516	135.6944	111.9582	132.7467	111.8862	134.6996	112.0182	137.113
111.9646	107.8003	111.8854	133.6905	111.8683	135.6928	111.9748	132.7468	111.9029	134.7	112.0349	137.113
111.9813	107.8065	111.9021	133.6879	111.8849	135.6907	111.9915	132.745	111.9196	134.7017	112.0515	137.1113
111.998	107.8032	111.9187	133.689	111.9016	135.6879	112.0082	132.7438	111.9362	134.6991	112.0682	137.1098
112.0146	107.8028	111.9354	133.6889	111.9183	135.6903	112.0248	132.7453	111.9529	134.6991	112.0849	137.1094
112.0313	107.8028	111.9521	133.6894	111.9349	135.6884	112.0415	132.7449	111.9696	134.6986	112.1015	137.1105
112.048	107.8017	111.9687	133.6894	111.9516	135.6891	112.0582	132.7426	111.9862	134.6981	112.1182	137.1096
112.0646	107.7976	111.9854	133.688	111.9683	135.687	112.0748	132.7449	112.0029	134.6976	112.1349	137.1094
112.0813	107.7949	112.0021	133.6885	111.9849	135.6859	112.0915	132.7436	112.0196	134.6958	112.1515	137.1082
112.098	107.7972	112.0187	133.6861	112.0016	135.6864	112.1082	132.7411	112.0362	134.6954	112.1682	137.107
112.1146	107.7978	112.0354	133.6868	112.0183	135.6887	112.1248	132.7411	112.0529	134.6958	112.1849	137.1047
112.1313	107.8011	112.0521	133.6864	112.0349	135.6853	112.1415	132.7407	112.0696	134.693	112.2015	137.104
112.148	107.792	112.0687	133.6861	112.0516	135.6841	112.1582	132.7392	112.0862	134.6935	112.2182	137.1032
112.1646	107.7925	112.0854	133.6861	112.0683	135.6883	112.1748	132.7403	112.1029	134.6916	112.2349	137.1021
112.1813	107.7942	112.1021	133.6865	112.0849	135.6858	112.1915	132.74	112.1196	134.692	112.2515	137.1006
112.198	107.7929	112.1187	133.6854	112.1016	135.6874	112.2082	132.7365	112.1362	134.6916	112.2682	137.0999
112.2146	107.7916	112.1354	133.6859	112.1183	135.6852	112.2248	132.7399	112.1529	134.6903	112.2849	137.1014
112.2313	107.7897	112.1521	133.6852	112.1349	135.6841	112.2415	132.738	112.1696	134.6894	112.3015	137.1007
112.248	107.791	112.1687	133.6825	112.1516	135.6875	112.2582	132.7394	112.1862	134.6876	112.3182	137.1008
112.2646	107.7888	112.1854	133.6858	112.1683	135.6829	112.2748	132.7375	112.2029	134.6882	112.3349	137.097
112.2813	107.7876	112.2021	133.6847	112.1849	135.6846	112.2915	132.7377	112.2196	134.6911	112.3515	137.0958
112.298	107.7886	112.2187	133.6836	112.2016	135.6797	112.3082	132.7367	112.2362	134.6868	112.3682	137.0953
112.3146	107.7863	112.2354	133.6837	112.2183	135.6837	112.3248	132.7362	112.2529	134.6856	112.3849	137.0943
112.3313	107.7873	112.2521	133.6824	112.2349	135.6831	112.3415	132.7361	112.2696	134.686	112.4015	137.0933
112.348	107.7827	112.2687	133.684	112.2516	135.6826	112.3582	132.7362	112.2862	134.6863	112.4182	137.0935
112.3646	107.7825	112.2854	133.6826	112.2683	135.6821	112.3748	132.7355	112.3029	134.6842	112.4349	137.0909
112.3813	107.7836	112.3021	133.682	112.2849	135.679	112.3915	132.7341	112.3196	134.6846	112.4515	137.0936
112.398	107.7806	112.3187	133.6823	112.3016	135.6824	112.4082	132.7334	112.3362	134.6837	112.4682	137.0925
112.4146	107.7832	112.3354	133.6824	112.3183	135.6805	112.4248	132.7332	112.3529	134.684	112.4849	137.0905
112.4313	107.7833	112.3521	133.6836	112.3349	135.6818	112.4415	132.734	112.3696	134.6817	112.5015	137.0892
112.448	107.7782	112.3687	133.6809	112.3516	135.677	112.4582	132.7328	112.3862	134.6813	112.5182	137.0896
112.4646	107.7792	112.3854	133.6805	112.3683	135.6803	112.4748	132.7311	112.4029	134.6806	112.5349	137.0862
112.4813	107.7756	112.4021	133.6778	112.3849	135.6771	112.4915	132.7315	112.4196	134.6806	112.5515	137.0878
112.498	107.7745	112.4187	133.6817	112.4016	135.6787	112.5082	132.7306	112.4362	134.6815	112.5682	137.0861
112.5146	107.777	112.4354	133.6818	112.4183	135.677	112.5248	132.7308	112.4529	134.6784	112.5849	137.0866
112.5313	107.7734	112.4521	133.6804	112.4349	135.6775	112.5415	132.7294	112.4696	134.6792	112.6015	137.084
112.548	107.7742	112.4687	133.6767	112.4516	135.6774	112.5582	132.7307	112.4862	134.6777	112.6182	137.0849
112.5646	107.7733	112.4854	133.6803	112.4683	135.677	112.5748	132.7303	112.5029	134.6771	112.6349	137.0831
112.5813	107.7714	112.5021	133.6793	112.4849	135.6766	112.5915	132.728	112.5196	134.6754	112.6515	137.0799
112.598	107.7698	112.5187	133.6778	112.5016	135.6757	112.6082	132.7285	112.5362	134.6749	112.6682	137.0805
112.6146	107.7687	112.5354	133.6791	112.5183	135.672	112.6248	132.7269	112.5529	134.6751	112.6849	137.0828
112.6313	107.7666	112.5521	133.676	112.5349	135.6716	112.6415	132.7249	112.5696	134.6735	112.7015	137.0799
112.648	107.7671	112.5687	133.6768	112.5516	135.6684	112.6582	132.7255	112.5862	134.6751	112.7182	137.0789
112.6646	107.7638	112.5854	133.6771	112.5683	135.6708	112.6748	132.7241	112.6029	134.6725	112.7349	137.0782
112.6813	107.7663	112.6021	133.6776	112.5849	135.669	112.6915	132.7246	112.6196	134.674	112.7515	137.0781
112.698	107.7658	112.6187	133.675	112.6016	135.6669	112.7082	132.7239	112.6362	134.6707	112.7682	137.0769
112.7146	107.7625	112.6354	133.6767	112.6183	135.665	112.7248	132.723	112.6529	134.6689	112.7849	137.0746
112.7313	107.7592	112.6521	133.6764	112.6349	135.6678	112.7415	132.7209	112.6696	134.6692	112.8015	137.0756
112.748	107.7588	112.6687	133.6769	112.6516	135.6635	112.7582	132.7218	112.6862	134.6688	112.8182	137.0746
112.7646	107.7619	112.6854	133.6751	112.6683	135.6623	112.7748	132.7209	112.7029	134.6681	112.8349	137.0728
112.7813	107.7617	112.7021	133.6762	112.6849	135.6656	112.7915	132.7187	112.7196	134.6674	112.8515	137.0714
112.798	107.7561	112.7187	133.6726	112.7016	135.6651	112.8082	132.7192	112.7362	134.6662	112.8682	137.0703
112.8146	107.7591	112.7354	133.6769	112.7183	135.6602	112.8248	132.7186	112.7529	134.6656	112.8849	137.0705
112.8313	107.7577	112.7521	133.6743	112.7349	135.6591	112.8415	132.7164	112.7696	134.6642	112.9015	137.0679
112.848	107.7539	112.7687	133.6712	112.7516	135.6596	112.8582	132.7181	112.7862	134.6649	112.9182	137.0687
112.8646	107.7514	112.7854	133.6701	112.7683	135.6564	112.8748	132.7169	112.8029	134.6621	112.9349	137.0667
112.8813	107.7508	112.8021	133.6719	112.7849	135.6582	112.8915	132.7138	112.8196	134.6623	112.9515	137.0668
112.898	107.7515	112.8187	133.6713	112.8016	135.6569	112.9082	132.7143	112.8362	134.6598	112.9682	137.0659
112.9146	107.7494	112.8354	133.67	112.8183	135.6529	112.9248	132.7149	112.8529	134.6599	112.9849	137.0667
112.9313	107.748	112.8521	133.671	112.8349	135.6567	112.9415	132.7114	112.8696	134.6561	113.0015	137.0631
112.948	107.7495	112.8687	133.6685	112.8516	135.6567	112.9582	132.7127	112.8862	134.66	113.0182	137.0634
112.9646	107.7468	112.8854	133.6715	112.8683	135.6551	112.9748	132.7102	112.9029	134.6573	113.0349	137.0633
112.9813	107.7452	112.9021	133.6671	112.8849	135.6554	112.9915	132.7095	112.9196	134.6565	113.0515	137.0617
112.998	107.744	112.9187	133.668	112.9016	135.6526	113.0082	132.7105	112.9362	134.6546	113.0682	137.0589
113.0146	107.7445	112.9354	133.6682	112.9183	135.6536	113.0248	132.7092	112.9529	134.6561	113.0849	137.0568
113.0313	107.7431	112.9521	133.6662	112.9349	135.6545	113.0415	132.7093	112.9696	134.6525	113.1015	137.0556
113.048	107.7419	112.9687	133.6644	112.9516	135.6536	113.0582	132.708	112.9862	134.6523	113.1182	137.0541
113.0646	107.7429	112.9854	133.6648	112.9683	135.6513	113.0748	132.7081	113.0029	134.6519	113.1349	137.0537
113.0813	107.7372	113.0021	133.6665	112.9849	135.65	113.0915	132.7043	113.0196	134.6509	113.1515	137.0511
113.098	107.7395	113.0187	133.6663	113.0016	135.6518	113.1082	132.7035	113.0362	134.6523	113.1682	137.0498
113.1146	107.7398	113.0354	133.6663	113.0183	135.6536	113.1248	132.7054	113.0529	134.6515	113.1849	137.0526
113.1313	107.7375	113.0521	133.6662	113.0349	135.6497	113.1415	132.7028	113.0696	134.649	113.2015	137.0531
113.148	107.7389	113.0687	133.662	113.0516	135.6514	113.1582	132.7029	113.0862	134.6487	113.2182	137.0516
113.1646	107.7362	113.0854	133.6651	113.0683	135.649	113.1748	132.7011	113.1029	134.6487	113.2349	137.0509
113.1813	107.7361	113.1021	133.6622	113.0849	135.6483	113.1915	132.6991	113.1196	134.6489	113.2515	137.0494
113.198	107.7321	113.1187	133.6653	113.1016	135.6497	113.2082	132.7002	113.1362	134.646	113.2682	137.0478
113.2146	107.7341	113.1354	133.6644	113.1183	135.6508	113.2248	132.7004	113.1529	134.6467	113.2849	137.047

113.2313	107.7334	113.1521	133.6656	113.1349	135.6483	113.2415	132.6992	113.1696	134.6431	113.3015	137.0486
113.248	107.7315	113.1687	133.6642	113.1516	135.6461	113.2582	132.6967	113.1862	134.6467	113.3182	137.0445
113.2646	107.7348	113.1854	133.6656	113.1683	135.6438	113.2748	132.6947	113.2029	134.6449	113.3349	137.045
113.2813	107.7332	113.2021	133.6631	113.1849	135.6462	113.2915	132.6959	113.2196	134.6435	113.3515	137.045
113.298	107.7303	113.2187	133.6615	113.2016	135.6477	113.3082	132.6968	113.2362	134.6416	113.3682	137.0416
113.3146	107.7332	113.2354	133.6615	113.2183	135.6431	113.3248	132.6938	113.2529	134.6426	113.3849	137.0437
113.3313	107.7349	113.2521	133.6628	113.2349	135.645	113.3415	132.6946	113.2696	134.6411	113.4015	137.0405
113.348	107.7336	113.2687	133.662	113.2516	135.6432	113.3582	132.6922	113.2862	134.6402	113.4182	137.0391
113.3646	107.7302	113.2854	133.6604	113.2683	135.6433	113.3748	132.6952	113.3029	134.6395	113.4349	137.038
113.3813	107.7315	113.3021	133.6623	113.2849	135.6441	113.3915	132.6934	113.3196	134.6389	113.4515	137.0363
113.398	107.7324	113.3187	133.6617	113.3016	135.6432	113.4082	132.6938	113.3362	134.6378	113.4682	137.0348
113.4146	107.7294	113.3354	133.6608	113.3183	135.6442	113.4248	132.6937	113.3529	134.6404	113.4849	137.0364
113.4313	107.7289	113.3521	133.6606	113.3349	135.6401	113.4415	132.6943	113.3696	134.6364	113.5015	137.0348
113.448	107.7292	113.3687	133.6615	113.3516	135.6407	113.4582	132.694	113.3862	134.6381	113.5182	137.0318
113.4646	107.7287	113.3854	133.66	113.3683	135.6413	113.4748	132.6936	113.4029	134.6358	113.5349	137.0301
113.4813	107.7266	113.4021	133.6606	113.3849	135.6387	113.4915	132.6909	113.4196	134.6364	113.5515	137.0289
113.498	107.7235	113.4187	133.6603	113.4016	135.6382	113.5082	132.6909	113.4362	134.6352	113.5682	137.0252
113.5146	107.724	113.4354	133.6594	113.4183	135.6395	113.5248	132.6915	113.4529	134.6359	113.5849	137.0254
113.5313	107.726	113.4521	133.661	113.4349	135.64	113.5415	132.6887	113.4696	134.6337	113.6015	137.0243
113.548	107.7219	113.4687	133.6606	113.4516	135.6378	113.5582	132.6898	113.4862	134.6334	113.6182	137.0218
113.5646	107.7208	113.4854	133.6578	113.4683	135.6379	113.5748	132.6889	113.5029	134.6331	113.6349	137.019
113.5813	107.7217	113.5021	133.6601	113.4849	135.6375	113.5915	132.6889	113.5196	134.631	113.6515	137.0181
113.598	107.721	113.5187	133.6602	113.5016	135.6363	113.6082	132.6872	113.5362	134.632	113.6682	137.0161
113.6146	107.7186	113.5354	133.6591	113.5183	135.6354	113.6248	132.685	113.5529	134.6307	113.6849	137.0154
113.6313	107.717	113.5521	133.6598	113.5349	135.6358	113.6415	132.6843	113.5696	134.6293	113.7015	137.0122
113.648	107.7179	113.5687	133.6605	113.5516	135.6357	113.6582	132.6847	113.5862	134.6285	113.7182	137.0119
113.6646	107.7169	113.5854	133.658	113.5683	135.6319	113.6748	132.6831	113.6029	134.6297	113.7349	137.0108
113.6813	107.7135	113.6021	133.6582	113.5849	135.634	113.6915	132.6823	113.6196	134.6291	113.7515	137.0084
113.698	107.7125	113.6187	133.6581	113.6016	135.6343	113.7082	132.6845	113.6362	134.6267	113.7682	137.0081
113.7146	107.7111	113.6354	133.6571	113.6183	135.6331	113.7248	132.6806	113.6529	134.6274	113.7849	137.0061
113.7313	107.7124	113.6521	133.6553	113.6349	135.6317	113.7415	132.6811	113.6696	134.6279	113.8015	137.0056
113.748	107.7119	113.6687	133.6554	113.6516	135.6311	113.7582	132.6814	113.6862	134.6252	113.8182	137.0034
113.7646	107.7087	113.6854	133.6549	113.6683	135.6295	113.7748	132.6803	113.7029	134.6251	113.8349	137.0001
113.7813	107.7099	113.7021	133.6562	113.6849	135.6302	113.7915	132.6816	113.7196	134.6267	113.8515	136.9991
113.798	107.7066	113.7187	133.656	113.7016	135.6311	113.8082	132.6796	113.7362	134.6234	113.8682	137.001
113.8146	107.7055	113.7354	133.6571	113.7183	135.6311	113.8248	132.6813	113.7529	134.6211	113.8849	137.0006
113.8313	107.7052	113.7521	133.6533	113.7349	135.6308	113.8415	132.679	113.7696	134.6224	113.9015	136.9963
113.848	107.7043	113.7687	133.6549	113.7516	135.6297	113.8582	132.6791	113.7862	134.6221	113.9182	136.9968
113.8646	107.7026	113.7854	133.6517	113.7683	135.6304	113.8748	132.6788	113.8029	134.6227	113.9349	136.9958
113.8813	107.7017	113.8021	133.6529	113.7849	135.6295	113.8915	132.677	113.8196	134.6225	113.9515	136.9939
113.898	107.697	113.8187	133.6535	113.8016	135.6308	113.9082	132.679	113.8362	134.6181	113.9682	136.9944
113.9146	107.6994	113.8354	133.6515	113.8183	135.6302	113.9248	132.6765	113.8529	134.6201	113.9849	136.9921
113.9313	107.6971	113.8521	133.6511	113.8349	135.6272	113.9415	132.6739	113.8696	134.6179	114.0015	136.9894
113.948	107.6967	113.8687	133.6542	113.8516	135.6267	113.9582	132.6777	113.8862	134.62	114.0182	136.9875
113.9646	107.6974	113.8854	133.6533	113.8683	135.6285	113.9748	132.6763	113.9029	134.616	114.0349	136.9878
113.9813	107.6946	113.9021	133.6511	113.8849	135.6282	113.9915	132.6736	113.9196	134.6165	114.0515	136.9873
113.998	107.6942	113.9187	133.6503	113.9016	135.6275	114.0082	132.6728	113.9362	134.6157	114.0682	136.9833
114.0146	107.6945	113.9354	133.6521	113.9183	135.6283	114.0248	132.6721	113.9529	134.6169	114.0849	136.9862
114.0313	107.6897	113.9521	133.6523	113.9349	135.6247	114.0415	132.673	113.9696	134.6157	114.1015	136.981
114.048	107.6922	113.9687	133.6497	113.9516	135.6249	114.0582	132.6717	113.9862	134.6146	114.1182	136.9807
114.0646	107.6898	113.9854	133.6479	113.9683	135.6267	114.0748	132.6733	114.0029	134.6155	114.1349	136.98
114.0813	107.6886	114.0021	133.6522	113.9849	135.6281	114.0915	132.6715	114.0196	134.6157	114.1515	136.9798
114.098	107.6852	114.0187	133.6513	114.0016	135.6228	114.1082	132.6705	114.0362	134.614	114.1682	136.979
114.1146	107.6858	114.0354	133.6474	114.0183	135.6221	114.1248	132.6723	114.0529	134.6123	114.1849	136.9774
114.1313	107.6847	114.0521	133.6484	114.0349	135.6209	114.1415	132.6685	114.0696	134.6125	114.2015	136.9754
114.148	107.6843	114.0687	133.6516	114.0516	135.6206	114.1582	132.6666	114.0862	134.6128	114.2182	136.9761
114.1646	107.6837	114.0854	133.6475	114.0683	135.6214	114.1748	132.6672	114.1029	134.6116	114.2349	136.9729
114.1813	107.6815	114.1021	133.6482	114.0849	135.619	114.1915	132.6679	114.1196	134.6118	114.2515	136.9718
114.198	107.6835	114.1187	133.6467	114.1016	135.6204	114.2082	132.666	114.1362	134.6093	114.2682	136.9694
114.2146	107.6781	114.1354	133.6468	114.1183	135.6181	114.2248	132.6658	114.1529	134.612	114.2849	136.9698
114.2313	107.6807	114.1521	133.6432	114.1349	135.6146	114.2415	132.6643	114.1696	134.6091	114.3015	136.969
114.248	107.6762	114.1687	133.6446	114.1516	135.6148	114.2582	132.6647	114.1862	134.6099	114.3182	136.9662
114.2646	107.6769	114.1854	133.6453	114.1683	135.6132	114.2748	132.6623	114.2029	134.6075	114.3349	136.9661
114.2813	107.6748	114.2021	133.6451	114.1849	135.6135	114.2915	132.6587	114.2196	134.6078	114.3515	136.9642
114.298	107.6739	114.2187	133.6419	114.2016	135.6117	114.3082	132.6621	114.2362	134.6074	114.3682	136.9611
114.3146	107.6698	114.2354	133.6429	114.2183	135.6135	114.3248	132.6633	114.2529	134.6055	114.3849	136.9603
114.3313	107.6699	114.2521	133.6436	114.2349	135.6098	114.3415	132.6629	114.2696	134.6052	114.4015	136.9579
114.348	107.6725	114.2687	133.6455	114.2516	135.6108	114.3582	132.6625	114.2862	134.6047	114.4182	136.9577
114.3646	107.6702	114.2854	133.6399	114.2683	135.6115	114.3748	132.6577	114.3029	134.6074	114.4349	136.956
114.3813	107.6683	114.3021	133.6392	114.2849	135.608	114.3915	132.6604	114.3196	134.6041	114.4515	136.9543
114.398	107.6691	114.3187	133.6428	114.3016	135.6125	114.4082	132.6597	114.3362	134.6038	114.4682	136.9531
114.4146	107.6662	114.3354	133.6411	114.3183	135.6079	114.4248	132.6588	114.3529	134.604	114.4849	136.9518
114.4313	107.6638	114.3521	133.6379	114.3349	135.6056	114.4415	132.6565	114.3696	134.6017	114.5015	136.9497
114.448	107.6635	114.3687	133.6393	114.3516	135.6079	114.4582	132.6582	114.3862	134.6035	114.5182	136.9494
114.4646	107.6624	114.3854	133.6366	114.3683	135.6069	114.4748	132.6565	114.4029	134.602	114.5349	136.9466
114.4813	107.6642	114.4021	133.6388	114.3849	135.6054	114.4915	132.6529	114.4196	134.6028	114.5515	136.9476
114.498	107.6582	114.4187	133.6356	114.4016	135.6041	114.5082	132.6509	114.4362	134.5997	114.5682	136.9435
114.5146	107.6572	114.4354	133.6371	114.4183	135.6035	114.5248	132.651	114.4529	134.6015	114.5849	136.9434
114.5313	107.658	114.4521	133.6327	114.4349	135.6042	114.5415	132.6525	114.4696	134.6	114.6015	136.9398
114.548	107.6571	114.4687	133.634	114.4516	135.6045	114.5582	132.6496	114.4862	134.5996	114.6182	136.938
114.5646	107.6558	114.4854	133.6325	114.4683	135.6021	114.5748	132.6479	114.5029	134.5984	114.6349	136.9351
114.5813	107.6598	114.5021	133.6316	114.4849	135.602	114.5915	132.6495	114.5196	134.6005	114.6515	136.9371
114.598	107.655	114.5187	133.6332	114.5016	135.6022	114.6082	132.6483	114.5362	134.5965	114.6682	136.9329
114.6146	107.6538	114.5354	133.6302	114.5183	135.6028	114.6248	132.6481	114.5529	134.5962	114.6849	136.9305
114.6313	107.6511	114.5521	133.6339	114.5349	135.6024	114.6415	132.6472	114.5696	134.5962	114.7015	136.9304
114.648	107.6504	114.5687	133.63	114.5516	135.6026	114.6582	132.6465	114.5862	134.5943	114.7182	136.9265
114.6646	107.6509	114.5854	133.6279	114.5683	135.598	114.6748	132.6456	114.6029	134.595	114.7349	136.9252
114.6813	107.6482	114.6021	133.6294	114.5849	135.6008	114.6915	132.6458	114.6196	134.5958	114.7515	136.9243
114.698	107.6467	114.6187	133.6299	114.6016	135.601	114.7082	132.6449	114.6362	134.5949	114.7682	136.922
114.7146	107.6473	114.6354	133.6281	114.6183	135.598	114.7248	132.6434	114.6529	134.5894	114.7849	136.9208
114.7313	107.6452	114.6521	133.627	114.6349	135.5971	114.7415	132.6418	114.6696	134.5943	114.8015	136.9181
114.748	107.6466	114.6687	133.6274	114.6516	135.5974	114.7582	132.6412	114.6862	134.5921	114.8182	136.9183
114.7646	107.6406	114.6854	133.6253	114.6683	135.5984	114.7748	132.6418	114.7029	134.5892	114.8349	136.9137
114.7813	107.6443	114.7021	133.6289	114.6849	135.5966	114.7915	132.6386	114.7196	134.5874	114.8515	136.9119
114.798	107.6439	114.7187	133.6258	114.7016	135.5962	114.8082	132.6397	114.7362	134.5928	114.8682	136.9102
114.8146	107.6405	114.7354	133.6292	114.7183	135.5945	114.8248	132.6363	114.7529	134.5855	114.8849	136.9097
114.8313	107.6375	114.7521	133.6266	114.7349	135.5945	114.8415	132.6384	114.7696	134.5881	114.9015	136.9058

114.848	107.6413	114.7687	133.6264	114.7516	135.593	114.8582	132.6352	114.7862	134.5888	114.9182	136.9056
114.8646	107.6368	114.7854	133.628	114.7683	135.5936	114.8748	132.6336	114.8029	134.5858	114.9349	136.9012
114.8813	107.6369	114.8021	133.6269	114.7849	135.5928	114.8915	132.6325	114.8196	134.5872	114.9515	136.9001
114.898	107.6364	114.8187	133.6291	114.8016	135.5922	114.9082	132.6335	114.8362	134.5845	114.9682	136.8975
114.9146	107.6348	114.8354	133.6268	114.8183	135.5927	114.9248	132.6316	114.8529	134.5869	114.9849	136.8963
114.9313	107.6331	114.8521	133.6258	114.8349	135.5897	114.9415	132.6298	114.8696	134.5842	115.0015	136.8943
114.948	107.6332	114.8687	133.6285	114.8516	135.5913	114.9582	132.6313	114.8862	134.5871	115.0182	136.8913
114.9646	107.6325	114.8854	133.6244	114.8683	135.5914	114.9748	132.6296	114.9029	134.5851	115.0349	136.8872
114.9813	107.6319	114.9021	133.6249	114.8849	135.5912	114.9915	132.629	114.9196	134.5848	115.0515	136.8862
114.998	107.6293	114.9187	133.6278	114.9016	135.5914	115.0082	132.6277	114.9362	134.5859	115.0682	136.8835
115.0146	107.6312	114.9354	133.6296	114.9183	135.5862	115.0248	132.6271	114.9529	134.5827	115.0849	136.8823
115.0313	107.6296	114.9521	133.6257	114.9349	135.5862	115.0415	132.6291	114.9696	134.5835	115.1015	136.8819
115.048	107.6241	114.9687	133.6243	114.9516	135.5872	115.0582	132.6268	114.9862	134.5841	115.1182	136.8796
115.0646	107.6254	114.9854	133.6255	114.9683	135.5858	115.0748	132.6289	115.0029	134.5845	115.1349	136.8767
115.0813	107.6265	115.0021	133.6213	114.9849	135.5849	115.0915	132.6243	115.0196	134.5822	115.1515	136.8744
115.098	107.6234	115.0187	133.624	115.0016	135.5855	115.1082	132.6267	115.0362	134.5832	115.1682	136.8718
115.1146	107.6251	115.0354	133.6261	115.0183	135.5843	115.1248	132.6252	115.0529	134.5824	115.1849	136.871
115.1313	107.6218	115.0521	133.6229	115.0349	135.5863	115.1415	132.6224	115.0696	134.5833	115.2015	136.8657
115.148	107.6189	115.0687	133.621	115.0516	135.5846	115.1582	132.6245	115.0862	134.5825	115.2182	136.8651
115.1646	107.6213	115.0854	133.6215	115.0683	135.5843	115.1748	132.621	115.1029	134.582	115.2349	136.8597
115.1813	107.6204	115.1021	133.6219	115.0849	135.5816	115.1915	132.6207	115.1196	134.5803	115.2515	136.858
115.198	107.6188	115.1187	133.6218	115.1016	135.5805	115.2082	132.6192	115.1362	134.5802	115.2682	136.8569
115.2146	107.6182	115.1354	133.6217	115.1183	135.5819	115.2248	132.6201	115.1529	134.5802	115.2849	136.8547
115.2313	107.6122	115.1521	133.6195	115.1349	135.5834	115.2415	132.6189	115.1696	134.5806	115.3015	136.8509
115.248	107.6148	115.1687	133.6183	115.1516	135.579	115.2582	132.618	115.1862	134.5816	115.3182	136.85
115.2646	107.6141	115.1854	133.6198	115.1683	135.5767	115.2748	132.6182	115.2029	134.5772	115.3349	136.846
115.2813	107.6119	115.2021	133.6207	115.1849	135.5762	115.2915	132.6178	115.2196	134.5771	115.3515	136.844
115.298	107.6126	115.2187	133.6201	115.2016	135.5769	115.3082	132.617	115.2362	134.576	115.3682	136.8423
115.3146	107.6119	115.2354	133.6191	115.2183	135.5756	115.3248	132.615	115.2529	134.5795	115.3849	136.8415
115.3313	107.608	115.2521	133.6185	115.2349	135.5774	115.3415	132.6139	115.2696	134.5786	115.4015	136.8397
115.348	107.608	115.2687	133.617	115.2516	135.5753	115.3582	132.6143	115.2862	134.577	115.4182	136.8373
115.3646	107.6056	115.2854	133.6173	115.2683	135.5735	115.3748	132.6108	115.3029	134.5765	115.4349	136.8356
115.3813	107.6084	115.3021	133.619	115.2849	135.5719	115.3915	132.6091	115.3196	134.5765	115.4515	136.8318
115.398	107.606	115.3187	133.6166	115.3016	135.571	115.4082	132.609	115.3362	134.5774	115.4682	136.8296
115.4146	107.6059	115.3354	133.6174	115.3183	135.5724	115.4248	132.6092	115.3529	134.575	115.4849	136.8273
115.4313	107.6034	115.3521	133.6168	115.3349	135.5712	115.4415	132.6065	115.3696	134.5749	115.5015	136.8269
115.448	107.6039	115.3687	133.6168	115.3516	135.5714	115.4582	132.6053	115.3862	134.5717	115.5182	136.8211
115.4646	107.6028	115.3854	133.6169	115.3683	135.5695	115.4748	132.6062	115.4029	134.5727	115.5349	136.8159
115.4813	107.6016	115.4021	133.6182	115.3849	135.5674	115.4915	132.6053	115.4196	134.5712	115.5515	136.8152
115.498	107.5996	115.4187	133.6165	115.4016	135.5655	115.5082	132.6034	115.4362	134.5669	115.5682	136.812
115.5146	107.5975	115.4354	133.6146	115.4183	135.5657	115.5248	132.6024	115.4529	134.5675	115.5849	136.8096
115.5313	107.5951	115.4521	133.6135	115.4349	135.5673	115.5415	132.6017	115.4696	134.5659	115.6015	136.8072
115.548	107.5966	115.4687	133.6134	115.4516	135.5648	115.5582	132.6003	115.4862	134.5663	115.6182	136.8044
115.5646	107.5939	115.4854	133.6115	115.4683	135.5648	115.5748	132.5998	115.5029	134.5626	115.6349	136.8017
115.5813	107.5942	115.5021	133.6149	115.4849	135.5611	115.5915	132.5972	115.5196	134.5627	115.6515	136.7994
115.598	107.5925	115.5187	133.6111	115.5016	135.5634	115.6082	132.5978	115.5362	134.5608	115.6682	136.7949
115.6146	107.5918	115.5354	133.6108	115.5183	135.5621	115.6248	132.5982	115.5529	134.5612	115.6849	136.7941
115.6313	107.5895	115.5521	133.6111	115.5349	135.5624	115.6415	132.5973	115.5696	134.5586	115.7015	136.7908
115.648	107.5895	115.5687	133.6124	115.5516	135.5599	115.6582	132.5929	115.5862	134.5579	115.7182	136.7898
115.6646	107.5879	115.5854	133.6099	115.5683	135.5605	115.6748	132.5956	115.6029	134.5568	115.7349	136.7858
115.6813	107.5876	115.6021	133.6118	115.5849	135.5605	115.6915	132.5923	115.6196	134.5563	115.7515	136.7844
115.698	107.5882	115.6187	133.6083	115.6016	135.5585	115.7082	132.5912	115.6362	134.5558	115.7682	136.7826
115.7146	107.5865	115.6354	133.6096	115.6183	135.5603	115.7248	132.5912	115.6529	134.5544	115.7849	136.7802
115.7313	107.5838	115.6521	133.6098	115.6349	135.5589	115.7415	132.588	115.6696	134.5529	115.8015	136.776
115.748	107.5841	115.6687	133.6075	115.6516	135.5556	115.7582	132.5912	115.6862	134.5562	115.8182	136.7784
115.7646	107.5841	115.6854	133.6081	115.6683	135.5571	115.7748	132.5864	115.7029	134.5529	115.8349	136.7759
115.7813	107.5832	115.7021	133.6089	115.6849	135.5566	115.7915	132.5891	115.7196	134.5524	115.8515	136.7727
115.798	107.584	115.7187	133.6043	115.7016	135.5569	115.8082	132.5859	115.7362	134.5538	115.8682	136.7692
115.8146	107.58	115.7354	133.6073	115.7183	135.5541	115.8248	132.5866	115.7529	134.5558	115.8849	136.7681
115.8313	107.5753	115.7521	133.6058	115.7349	135.5548	115.8415	132.5854	115.7696	134.553	115.9015	136.7674
115.848	107.5823	115.7687	133.6059	115.7516	135.5545	115.8582	132.5836	115.7862	134.553	115.9182	136.7654
115.8646	107.5757	115.7854	133.6062	115.7683	135.5563	115.8748	132.5846	115.8029	134.5544	115.9349	136.7603
115.8813	107.5769	115.8021	133.6052	115.7849	135.5533	115.8915	132.583	115.8196	134.5532	115.9515	136.7623
115.898	107.5769	115.8187	133.6027	115.8016	135.5517	115.9082	132.5841	115.8362	134.5548	115.9682	136.7595
115.9146	107.5734	115.8354	133.6082	115.8183	135.5531	115.9248	132.5825	115.8529	134.5533	115.9849	136.7547
115.9313	107.5741	115.8521	133.6055	115.8349	135.5503	115.9415	132.5813	115.8696	134.5542	116.0015	136.7507
115.948	107.5694	115.8687	133.6046	115.8516	135.5516	115.9582	132.5818	115.8862	134.5532	116.0182	136.7486
115.9646	107.5716	115.8854	133.6028	115.8683	135.5534	115.9748	132.5803	115.9029	134.5531	116.0349	136.7462
115.9813	107.5682	115.9021	133.6031	115.8849	135.5527	115.9915	132.5783	115.9196	134.5531	116.0515	136.7436
115.998	107.5702	115.9187	133.6046	115.9016	135.5513	116.0082	132.5798	115.9362	134.5514	116.0682	136.7418
116.0146	107.5672	115.9354	133.6045	115.9183	135.5482	116.0248	132.5771	115.9529	134.5526	116.0849	136.7343
116.0313	107.5697	115.9521	133.6044	115.9349	135.5496	116.0415	132.5755	115.9696	134.5518	116.1015	136.7331
116.048	107.5671	115.9687	133.6029	115.9516	135.5479	116.0582	132.5776	115.9862	134.5515	116.1182	136.7301
116.0646	107.5627	115.9854	133.6035	115.9683	135.5476	116.0748	132.5755	116.0029	134.5488	116.1349	136.7248
116.0813	107.5639	116.0021	133.604	115.9849	135.5479	116.0915	132.5747	116.0196	134.55	116.1515	136.7246
116.098	107.5636	116.0187	133.6005	116.0016	135.5474	116.1082	132.5747	116.0362	134.5495	116.1682	136.7242
116.1146	107.5608	116.0354	133.603	116.0183	135.5428	116.1248	132.5715	116.0529	134.5506	116.1849	136.7177
116.1313	107.5617	116.0521	133.6025	116.0349	135.542	116.1415	132.5733	116.0696	134.5492	116.2015	136.7161
116.148	107.5597	116.0687	133.6018	116.0516	135.5534	116.1582	132.5726	116.0862	134.5487	116.2182	136.716
116.1646	107.5612	116.0854	133.5994	116.0683	135.5454	116.1748	132.572	116.1029	134.5494	116.2349	136.7125
116.1813	107.563	116.1021	133.6034	116.0849	135.5451	116.1915	132.5726	116.1196	134.5485	116.2515	136.7104
116.198	107.558	116.1187	133.6007	116.1016	135.5458	116.2082	132.5716	116.1362	134.5453	116.2682	136.7107
116.2146	107.558	116.1354	133.5996	116.1183	135.5444	116.2248	132.571	116.1529	134.5474	116.2849	136.7084
116.2313	107.5567	116.1521	133.5997	116.1349	135.5414	116.2415	132.5703	116.1696	134.5469	116.3015	136.704
116.248	107.552	116.1687	133.5978	116.1516	135.5403	116.2582	132.567	116.1862	134.5469	116.3182	136.7006
116.2646	107.5565	116.1854	133.5982	116.1683	135.541	116.2748	132.5693	116.2029	134.5468	116.3349	136.6985
116.2813	107.553	116.2021	133.5993	116.1849	135.5414	116.2915	132.5671	116.2196	134.5462	116.3515	136.6956
116.298	107.5556	116.2187	133.5986	116.2016	135.5374	116.3082	132.5657	116.2362	134.5457	116.3682	136.6907
116.3146	107.5535	116.2354	133.5955	116.2183	135.5372	116.3248	132.5647	116.2529	134.5468	116.3849	136.6906
116.3313	107.5528	116.2521	133.5934	116.2349	135.5387	116.3415	132.5663	116.2696	134.5453	116.4015	136.6862
116.348	107.5488	116.2687	133.5966	116.2516	135.5364	116.3582	132.5644	116.2862	134.5418	116.4182	136.6835
116.3646	107.5499	116.2854	133.5967	116.2683	135.5366	116.3748	132.563	116.3029	134.5447	116.4349	136.681
116.3813	107.5458	116.3021	133.5931	116.2849	135.5363	116.3915	132.5604	116.3196	134.5457	116.4515	136.6784
116.398	107.5467	116.3187	133.5915	116.3016	135.5347	116.4082	132.5603	116.3362	134.5425	116.4682	136.6729
116.4146	107.5457	116.3354	133.5839	116.3183	135.5328	116.4248	132.5606	116.3529	134.5414	116.4849	136.6715
116.4313	107.5464	116.3521	133.5953	116.3349	135.5335	116.4415	132.5594	116.3696	134.5433	116.5015	136.6677
116.448	107.5432	116.3687	133.5945	116.3516	135.5315	116.4582	132.5575	116.3862	134.5422	116.5182	136.6649

116.4646	107.546	116.3854	133.5895	116.3683	135.5333	116.4748	132.5554	116.4029	134.541	116.5349	136.6632
116.4813	107.5439	116.4021	133.5897	116.3849	135.5317	116.4915	132.555	116.4196	134.5423	116.5515	136.6618
116.498	107.5436	116.4187	133.5914	116.4016	135.5279	116.5082	132.556	116.4362	134.5405	116.5682	136.6569
116.5146	107.5411	116.4354	133.5871	116.4183	135.5268	116.5248	132.5536	116.4529	134.5416	116.5849	136.6547
116.5313	107.542	116.4521	133.5919	116.4349	135.5281	116.5415	132.5534	116.4696	134.5432	116.6015	136.653
116.548	107.5399	116.4687	133.5879	116.4516	135.5291	116.5582	132.5499	116.4862	134.5403	116.6182	136.6496
116.5646	107.5396	116.4854	133.5883	116.4683	135.5285	116.5748	132.5529	116.5029	134.5402	116.6349	136.6499
116.5813	107.5364	116.5021	133.5879	116.4849	135.524	116.5915	132.5507	116.5196	134.5404	116.6515	136.6467
116.598	107.5357	116.5187	133.5888	116.5016	135.5246	116.6082	132.5493	116.5362	134.5412	116.6682	136.6417
116.6146	107.5344	116.5354	133.5852	116.5183	135.5252	116.6248	132.5495	116.5529	134.5404	116.6849	136.641
116.6313	107.5334	116.5521	133.5852	116.5349	135.5267	116.6415	132.5476	116.5696	134.5398	116.7015	136.6371
116.648	107.5335	116.5687	133.5857	116.5516	135.5244	116.6582	132.5475	116.5862	134.5401	116.7182	136.6348
116.6646	107.535	116.5854	133.5834	116.5683	135.5229	116.6748	132.5486	116.6029	134.5393	116.7349	136.6344
116.6813	107.5329	116.6021	133.5858	116.5849	135.5221	116.6915	132.5472	116.6196	134.5396	116.7515	136.6326
116.698	107.5323	116.6187	133.5855	116.6016	135.5216	116.7082	132.5469	116.6362	134.54	116.7682	136.629
116.7146	107.5339	116.6354	133.5802	116.6183	135.521	116.7248	132.5443	116.6529	134.5392	116.7849	136.627
116.7313	107.532	116.6521	133.5835	116.6349	135.5211	116.7415	132.5426	116.6696	134.5357	116.8015	136.6251
116.748	107.5307	116.6687	133.5828	116.6516	135.5187	116.7582	132.5439	116.6862	134.5385	116.8182	136.6246
116.7646	107.5283	116.6854	133.5811	116.6683	135.5188	116.7748	132.5437	116.7029	134.5377	116.8349	136.6219
116.7813	107.5286	116.7021	133.5817	116.6849	135.5184	116.7915	132.5426	116.7196	134.5354	116.8515	136.6197
116.798	107.5281	116.7187	133.5809	116.7016	135.5185	116.8082	132.5426	116.7362	134.5366	116.8682	136.617
116.8146	107.527	116.7354	133.583	116.7183	135.5159	116.8248	132.5406	116.7529	134.5353	116.8849	136.6139
116.8313	107.5285	116.7521	133.5816	116.7349	135.5181	116.8415	132.5399	116.7696	134.5339	116.9015	136.613
116.848	107.5256	116.7687	133.582	116.7516	135.5158	116.8582	132.5379	116.7862	134.5341	116.9182	136.6098
116.8646	107.5248	116.7854	133.5815	116.7683	135.5127	116.8748	132.5392	116.8029	134.5341	116.9349	136.6081
116.8813	107.5251	116.8021	133.5843	116.7849	135.5148	116.8915	132.5391	116.8196	134.5369	116.9515	136.6054
116.898	107.5246	116.8187	133.5814	116.8016	135.5124	116.9082	132.537	116.8362	134.5323	116.9682	136.6041
116.9146	107.523	116.8354	133.5811	116.8183	135.5111	116.9248	132.5359	116.8529	134.5328	116.9849	136.6015
116.9313	107.5204	116.8521	133.581	116.8349	135.5122	116.9415	132.535	116.8696	134.5325	117.0015	136.5964
116.948	107.5181	116.8687	133.5812	116.8516	135.512	116.9582	132.5347	116.8862	134.5326	117.0182	136.5959
116.9646	107.5192	116.8854	133.5798	116.8683	135.5113	116.9748	132.5332	116.9029	134.531	117.0349	136.5913
116.9813	107.5173	116.9021	133.5807	116.8849	135.5097	116.9915	132.5336	116.9196	134.533	117.0515	136.593
116.998	107.5187	116.9187	133.5781	116.9016	135.5079	117.0082	132.532	116.9362	134.5321	117.0682	136.5898
117.0146	107.5142	116.9354	133.5818	116.9183	135.5083	117.0248	132.5313	116.9529	134.5295	117.0849	136.587
117.0313	107.5145	116.9521	133.5754	116.9349	135.5105	117.0415	132.5303	116.9696	134.5296	117.1015	136.5834
117.048	107.5125	116.9687	133.5798	116.9516	135.5081	117.0582	132.5295	116.9862	134.5307	117.1182	136.5818
117.0646	107.5139	116.9854	133.5761	116.9683	135.5063	117.0748	132.5299	117.0029	134.5308	117.1349	136.5803
117.0813	107.5091	117.0021	133.5803	116.9849	135.5053	117.0915	132.5268	117.0196	134.5306	117.1515	136.5791
117.098	107.5099	117.0187	133.5715	117.0016	135.503	117.1082	132.5263	117.0362	134.5301	117.1682	136.5772
117.1146	107.5101	117.0354	133.5727	117.0183	135.5064	117.1248	132.528	117.0529	134.5311	117.1849	136.5744
117.1313	107.5114	117.0521	133.5712	117.0349	135.5059	117.1415	132.5248	117.0696	134.5287	117.2015	136.5713
117.148	107.5079	117.0687	133.5732	117.0516	135.5048	117.1582	132.5229	117.0862	134.5259	117.2182	136.567
117.1646	107.5079	117.0854	133.5731	117.0683	135.5057	117.1748	132.5251	117.1029	134.5274	117.2349	136.5625
117.1813	107.505	117.1021	133.5706	117.0849	135.5014	117.1915	132.5209	117.1196	134.5268	117.2515	136.5609
117.198	107.5053	117.1187	133.5714	117.1016	135.5019	117.2082	132.5207	117.1362	134.5256	117.2682	136.5565
117.2146	107.5022	117.1354	133.5714	117.1183	135.5012	117.2248	132.5196	117.1529	134.5255	117.2849	136.5544
117.2313	107.5041	117.1521	133.569	117.1349	135.5001	117.2415	132.5186	117.1696	134.5251	117.3015	136.551
117.248	107.5021	117.1687	133.5696	117.1516	135.498	117.2582	132.5181	117.1862	134.5251	117.3182	136.5511
117.2646	107.4996	117.1854	133.5679	117.1683	135.5008	117.2748	132.5184	117.2029	134.5269	117.3349	136.5485
117.2813	107.5027	117.2021	133.5659	117.1849	135.4995	117.2915	132.5183	117.2196	134.5234	117.3515	136.5457
117.298	107.501	117.2187	133.5669	117.2016	135.4997	117.3082	132.5169	117.2362	134.5241	117.3682	136.5434
117.3146	107.4963	117.2354	133.5642	117.2183	135.498	117.3248	132.5147	117.2529	134.5228	117.3849	136.5401
117.3313	107.4949	117.2521	133.5646	117.2349	135.4949	117.3415	132.515	117.2696	134.5239	117.4015	136.535
117.348	107.4984	117.2687	133.5659	117.2516	135.4982	117.3582	132.5134	117.2862	134.5205	117.4182	136.5321
117.3646	107.4976	117.2854	133.562	117.2683	135.4984	117.3748	132.5128	117.3029	134.5228	117.4349	136.5292
117.3813	107.4932	117.3021	133.5642	117.2849	135.4954	117.3915	132.5118	117.3196	134.5225	117.4515	136.5252
117.398	107.4934	117.3187	133.5618	117.3016	135.4986	117.4082	132.5117	117.3362	134.5223	117.4682	136.5212
117.4146	107.4929	117.3354	133.5624	117.3183	135.4945	117.4248	132.5106	117.3529	134.5194	117.4849	136.5207
117.4313	107.492	117.3521	133.5593	117.3349	135.4959	117.4415	132.5093	117.3696	134.5206	117.5015	136.5176
117.448	107.49	117.3687	133.5575	117.3516	135.4889	117.4582	132.5081	117.3862	134.519	117.5182	136.5148
117.4646	107.4901	117.3854	133.5619	117.3683	135.4866	117.4748	132.5077	117.4029	134.5194	117.5349	136.512
117.4813	107.4902	117.4021	133.5601	117.3849	135.4904	117.4915	132.507	117.4196	134.5196	117.5515	136.5079
117.498	107.4873	117.4187	133.5626	117.4016	135.4911	117.5082	132.5098	117.4362	134.5214	117.5682	136.5039
117.5146	107.4874	117.4354	133.56	117.4183	135.4917	117.5248	132.5067	117.4529	134.5203	117.5849	136.5029
117.5313	107.4877	117.4521	133.5557	117.4349	135.4918	117.5415	132.5066	117.4696	134.518	117.6015	136.4985
117.548	107.4857	117.4687	133.5602	117.4516	135.4885	117.5582	132.5052	117.4862	134.5185	117.6182	136.4967
117.5646	107.4834	117.4854	133.5589	117.4683	135.4918	117.5748	132.507	117.5029	134.5177	117.6349	136.4953
117.5813	107.4866	117.5021	133.5559	117.4849	135.4863	117.5915	132.5045	117.5196	134.5189	117.6515	136.4927
117.598	107.4813	117.5187	133.5621	117.5016	135.4863	117.6082	132.5022	117.5362	134.5191	117.6682	136.4908
117.6146	107.4817	117.5354	133.5573	117.5183	135.4866	117.6248	132.5028	117.5529	134.5178	117.6849	136.4881
117.6313	107.4815	117.5521	133.5514	117.5349	135.4873	117.6415	132.5043	117.5696	134.5148	117.7015	136.4831
117.648	107.4811	117.5687	133.5561	117.5516	135.4844	117.6582	132.5036	117.5862	134.5153	117.7182	136.4811
117.6646	107.4806	117.5854	133.554	117.5683	135.488	117.6748	132.5034	117.6029	134.5178	117.7349	136.4791
117.6813	107.4788	117.6021	133.5567	117.5849	135.4839	117.6915	132.4994	117.6196	134.5133	117.7515	136.4778
117.698	107.481	117.6187	133.5513	117.6016	135.4834	117.7082	132.5019	117.6362	134.5134	117.7682	136.4759
117.7146	107.476	117.6354	133.5537	117.6183	135.4833	117.7248	132.499	117.6529	134.5139	117.7849	136.4737
117.7313	107.477	117.6521	133.5533	117.6349	135.4824	117.7415	132.499	117.6696	134.5148	117.8015	136.47
117.748	107.4763	117.6687	133.5519	117.6516	135.482	117.7582	132.4979	117.6862	134.513	117.8182	136.466
117.7646	107.4766	117.6854	133.5541	117.6683	135.4829	117.7748	132.4948	117.7029	134.5124	117.8349	136.4636
117.7813	107.4734	117.7021	133.5518	117.6849	135.4788	117.7915	132.4971	117.7196	134.5096	117.8515	136.4601
117.798	107.4746	117.7187	133.5535	117.7016	135.4798	117.8082	132.4972	117.7362	134.511	117.8682	136.4575
117.8146	107.4745	117.7354	133.5509	117.7183	135.4787	117.8248	132.4954	117.7529	134.5117	117.8849	136.4547
117.8313	107.4704	117.7521	133.553	117.7349	135.4781	117.8415	132.495	117.7696	134.5106	117.9015	136.4521
117.848	107.4712	117.7687	133.5502	117.7516	135.479	117.8582	132.4958	117.7862	134.5102	117.9182	136.4501
117.8646	107.4713	117.7854	133.5485	117.7683	135.4766	117.8748	132.4936	117.8029	134.5096	117.9349	136.4472
117.8813	107.4665	117.8021	133.5495	117.7849	135.4761	117.8915	132.4928	117.8196	134.5082	117.9515	136.4442
117.898	107.4676	117.8187	133.5445	117.8016	135.475	117.9082	132.4915	117.8362	134.5071	117.9682	136.442
117.9146	107.4679	117.8354	133.5521	117.8183	135.4748	117.9248	132.49	117.8529	134.5077	117.9849	136.4387
117.9313	107.4668	117.8521	133.5473	117.8349	135.4761	117.9415	132.4923	117.8696	134.5075	118.0015	136.4349
117.948	107.4669	117.8687	133.5499	117.8516	135.4757	117.9582	132.4884	117.8862	134.5066	118.0182	136.4333
117.9646	107.4651	117.8854	133.5472	117.8683	135.4721	117.9748	132.49	117.9029	134.5067	118.0349	136.4306
117.9813	107.464	117.9021	133.5484	117.8849	135.4732	117.9915	132.487	117.9196	134.5074	118.0515	136.4268
117.998	107.4631	117.9187	133.5459	117.9016	135.4722	118.0082	132.4915	117.9362	134.5062	118.0682	136.423
118.0146	107.4611	117.9354	133.5428	117.9183	135.4743	118.0248	132.4874	117.9529	134.5063	118.0849	136.4209
118.0313	107.4643	117.9521	133.5505	117.9349	135.4719	118.0415	132.4889	117.9696	134.506	118.1015	136.4179
118.048	107.46	117.9687	133.5453	117.9516	135.4693	118.0582	132.4867	117.9862	134.5046	118.1182	136.4146
118.0646	107.4635	117.9854	133.5461	117.9683	135.4713	118.0748	132.4855	118.0029	134.5052	118.1349	136.4133

118.0813	107.4567	118.0021	133.5442	117.9849	135.4694	118.0915	132.4874	118.0196	134.5038	118.1515	136.408
118.098	107.4612	118.0187	133.546	118.0016	135.4699	118.1082	132.4853	118.0362	134.5023	118.1682	136.4028
118.1146	107.4566	118.0354	133.5408	118.0183	135.4706	118.1248	132.482	118.0529	134.503	118.1849	136.3986
118.1313	107.4599	118.0521	133.5434	118.0349	135.4691	118.1415	132.4816	118.0696	134.5039	118.2015	136.3963
118.148	107.4574	118.0687	133.5424	118.0516	135.4664	118.1582	132.4829	118.0862	134.5034	118.2182	136.3937
118.1646	107.4556	118.0854	133.5395	118.0683	135.4683	118.1748	132.4833	118.1029	134.5035	118.2349	136.391
118.1813	107.4558	118.1021	133.5406	118.0849	135.4663	118.1915	132.4769	118.1196	134.5036	118.2515	136.3895
118.198	107.4526	118.1187	133.5447	118.1016	135.4663	118.2082	132.4786	118.1362	134.5009	118.2682	136.386
118.2146	107.4537	118.1354	133.5366	118.1183	135.4659	118.2248	132.4774	118.1529	134.5017	118.2849	136.3859
118.2313	107.4517	118.1521	133.5386	118.1349	135.4662	118.2415	132.4781	118.1696	134.5013	118.3015	136.3812
118.248	107.4521	118.1687	133.5382	118.1516	135.4671	118.2582	132.4738	118.1862	134.4997	118.3182	136.3788
118.2646	107.4545	118.1854	133.5385	118.1683	135.4645	118.2748	132.4738	118.2029	134.4993	118.3349	136.3776
118.2813	107.449	118.2021	133.5368	118.1849	135.4641	118.2915	132.4753	118.2196	134.5018	118.3515	136.3768
118.298	107.45	118.2187	133.5389	118.2016	135.4647	118.3082	132.4744	118.2362	134.4994	118.3682	136.3736
118.3146	107.4481	118.2354	133.5377	118.2183	135.4637	118.3248	132.4726	118.2529	134.4992	118.3849	136.3702
118.3313	107.4505	118.2521	133.5357	118.2349	135.4621	118.3415	132.4732	118.2696	134.4977	118.4015	136.3686
118.348	107.4465	118.2687	133.5368	118.2516	135.4606	118.3582	132.4718	118.2862	134.4972	118.4182	136.3685
118.3646	107.4479	118.2854	133.5374	118.2683	135.4605	118.3748	132.4714	118.3029	134.4981	118.4349	136.3647
118.3813	107.447	118.3021	133.5365	118.2849	135.4612	118.3915	132.4702	118.3196	134.498	118.4515	136.3626
118.398	107.4444	118.3187	133.5333	118.3016	135.4616	118.4082	132.4692	118.3362	134.4971	118.4682	136.361
118.4146	107.4432	118.3354	133.5334	118.3183	135.4578	118.4248	132.4698	118.3529	134.4958	118.4849	136.3593
118.4313	107.4444	118.3521	133.533	118.3349	135.4604	118.4415	132.4682	118.3696	134.4965	118.5015	136.3578
118.448	107.4435	118.3687	133.533	118.3516	135.4589	118.4582	132.4682	118.3862	134.4962	118.5182	136.3542
118.4646	107.4422	118.3854	133.533	118.3683	135.4577	118.4748	132.4694	118.4029	134.4917	118.5349	136.3513
118.4813	107.4414	118.4021	133.534	118.3849	135.4565	118.4915	132.4688	118.4196	134.492	118.5515	136.3501
118.498	107.4423	118.4187	133.5318	118.4016	135.4582	118.5082	132.467	118.4362	134.4954	118.5682	136.3471
118.5146	107.4375	118.4354	133.5322	118.4183	135.4555	118.5248	132.4671	118.4529	134.4925	118.5849	136.3446
118.5313	107.4381	118.4521	133.5311	118.4349	135.4524	118.5415	132.4651	118.4696	134.4901	118.6015	136.3424
118.548	107.4375	118.4687	133.5328	118.4516	135.4504	118.5582	132.466	118.4862	134.4905	118.6182	136.3411
118.5646	107.4389	118.4854	133.5294	118.4683	135.4552	118.5748	132.4638	118.5029	134.4929	118.6349	136.3378
118.5813	107.435	118.5021	133.5308	118.4849	135.4528	118.5915	132.4639	118.5196	134.4911	118.6515	136.3361
118.598	107.4345	118.5187	133.5311	118.5016	135.4535	118.6082	132.4634	118.5362	134.4895	118.6682	136.3326
118.6146	107.4329	118.5354	133.5315	118.5183	135.4497	118.6248	132.4632	118.5529	134.4891	118.6849	136.332
118.6313	107.4332	118.5521	133.5279	118.5349	135.4503	118.6415	132.463	118.5696	134.4914	118.7015	136.3268
118.648	107.4298	118.5687	133.5305	118.5516	135.4532	118.6582	132.462	118.5862	134.4899	118.7182	136.3249
118.6646	107.4315	118.5854	133.5297	118.5683	135.4522	118.6748	132.4608	118.6029	134.4877	118.7349	136.3206
118.6813	107.4253	118.6021	133.5273	118.5849	135.4521	118.6915	132.461	118.6196	134.4904	118.7515	136.3185
118.698	107.432	118.6187	133.5291	118.6016	135.451	118.7082	132.4576	118.6362	134.4889	118.7682	136.3136
118.7146	107.4309	118.6354	133.5255	118.6183	135.4515	118.7248	132.4572	118.6529	134.4887	118.7849	136.3105
118.7313	107.4303	118.6521	133.5281	118.6349	135.4499	118.7415	132.4582	118.6696	134.4874	118.8015	136.3067
118.748	107.4261	118.6687	133.5279	118.6516	135.4498	118.7582	132.4575	118.6862	134.4886	118.8182	136.3002
118.7646	107.4261	118.6854	133.5257	118.6683	135.4485	118.7748	132.4577	118.7029	134.4881	118.8349	136.2996
118.7813	107.4201	118.7021	133.5256	118.6849	135.4455	118.7915	132.4555	118.7196	134.4881	118.8515	136.2944
118.798	107.4235	118.7187	133.5252	118.7016	135.4461	118.8082	132.4579	118.7362	134.4855	118.8682	136.2917
118.8146	107.4226	118.7354	133.5249	118.7183	135.4457	118.8248	132.4551	118.7529	134.4853	118.8849	136.2887
118.8313	107.421	118.7521	133.5236	118.7349	135.4424	118.8415	132.4524	118.7696	134.4877	118.9015	136.286
118.848	107.4208	118.7687	133.5239	118.7516	135.4416	118.8582	132.4561	118.7862	134.4853	118.9182	136.281
118.8646	107.419	118.7854	133.5246	118.7683	135.4428	118.8748	132.4542	118.8029	134.482	118.9349	136.2807
118.8813	107.4171	118.8021	133.5234	118.7849	135.4429	118.8915	132.4543	118.8196	134.4836	118.9515	136.2781
118.898	107.4169	118.8187	133.5235	118.8016	135.4416	118.9082	132.4514	118.8362	134.484	118.9682	136.2748
118.9146	107.4174	118.8354	133.5216	118.8183	135.4396	118.9248	132.4519	118.8529	134.4827	118.9849	136.2717
118.9313	107.4159	118.8521	133.5214	118.8349	135.4385	118.9415	132.4521	118.8696	134.4803	119.0015	136.2653
118.948	107.4172	118.8687	133.5218	118.8516	135.4403	118.9582	132.4507	118.8862	134.4831	119.0182	136.2656
118.9646	107.416	118.8854	133.5218	118.8683	135.4377	118.9748	132.4484	118.9029	134.4813	119.0349	136.2629
118.9813	107.4107	118.9021	133.5205	118.8849	135.4349	118.9915	132.4499	118.9196	134.4797	119.0515	136.2585
118.998	107.4113	118.9187	133.5203	118.9016	135.435	119.0082	132.4495	118.9362	134.4807	119.0682	136.2554
119.0146	107.41	118.9354	133.5198	118.9183	135.4348	119.0248	132.4459	118.9529	134.4809	119.0849	136.2498
119.0313	107.4097	118.9521	133.519	118.9349	135.4363	119.0415	132.4459	118.9696	134.484	119.1015	136.2511
119.048	107.4096	118.9687	133.5187	118.9516	135.4334	119.0582	132.4469	118.9862	134.4782	119.1182	136.2423
119.0646	107.4076	118.9854	133.522	118.9683	135.4337	119.0748	132.4447	119.0029	134.4811	119.1349	136.2389
119.0813	107.4043	119.0021	133.5156	118.9849	135.4313	119.0915	132.4436	119.0196	134.4797	119.1515	136.2384
119.098	107.4072	119.0187	133.5187	119.0016	135.4318	119.1082	132.4436	119.0362	134.4764	119.1682	136.2365
119.1146	107.4037	119.0354	133.519	119.0183	135.4315	119.1248	132.4451	119.0529	134.4801	119.1849	136.2334
119.1313	107.4048	119.0521	133.5137	119.0349	135.4306	119.1415	132.4408	119.0696	134.4792	119.2015	136.2311
119.148	107.4015	119.0687	133.515	119.0516	135.4301	119.1582	132.439	119.0862	134.4775	119.2182	136.2289
119.1646	107.4013	119.0854	133.5156	119.0683	135.4312	119.1748	132.4397	119.1029	134.4752	119.2349	136.2265
119.1813	107.3995	119.1021	133.5147	119.0849	135.4298	119.1915	132.4422	119.1196	134.4737	119.2515	136.2234
119.198	107.4009	119.1187	133.5153	119.1016	135.4273	119.2082	132.4385	119.1362	134.4755	119.2682	136.22
119.2146	107.3948	119.1354	133.5151	119.1183	135.4253	119.2248	132.4379	119.1529	134.4729	119.2849	136.2138
119.2313	107.3991	119.1521	133.5144	119.1349	135.425	119.2415	132.4367	119.1696	134.4751	119.3015	136.2133
119.248	107.3953	119.1687	133.5112	119.1516	135.421	119.2582	132.4369	119.1862	134.4729	119.3182	136.2124
119.2646	107.3979	119.1854	133.5143	119.1683	135.422	119.2748	132.438	119.2029	134.4721	119.3349	136.2091
119.2813	107.3944	119.2021	133.5143	119.1849	135.4272	119.2915	132.4346	119.2196	134.4718	119.3515	136.2066
119.298	107.3916	119.2187	133.5091	119.2016	135.4248	119.3082	132.4327	119.2362	134.4722	119.3682	136.2036
119.3146	107.3927	119.2354	133.5125	119.2183	135.4239	119.3248	132.4339	119.2529	134.4704	119.3849	136.2025
119.3313	107.3915	119.2521	133.5111	119.2349	135.4228	119.3415	132.4352	119.2696	134.4677	119.4015	136.1991
119.348	107.3909	119.2687	133.5109	119.2516	135.4217	119.3582	132.4314	119.2862	134.47	119.4182	136.1982
119.3646	107.3892	119.2854	133.5072	119.2683	135.4205	119.3748	132.4315	119.3029	134.4684	119.4349	136.1954
119.3813	107.3903	119.3021	133.5092	119.2849	135.4181	119.3915	132.4312	119.3196	134.4698	119.4515	136.1915
119.398	107.3891	119.3187	133.5062	119.3016	135.4176	119.4082	132.4332	119.3362	134.4688	119.4682	136.1898
119.4146	107.3861	119.3354	133.5078	119.3183	135.421	119.4248	132.4325	119.3529	134.4674	119.4849	136.1872
119.4313	107.3826	119.3521	133.5088	119.3349	135.4195	119.4415	132.4308	119.3696	134.47	119.5015	136.1846
119.448	107.384	119.3687	133.5067	119.3516	135.4157	119.4582	132.4309	119.3862	134.4676	119.5182	136.181
119.4646	107.3844	119.3854	133.5064	119.3683	135.4146	119.4748	132.4322	119.4029	134.4675	119.5349	136.1783
119.4813	107.3817	119.4021	133.5059	119.3849	135.418	119.4915	132.4312	119.4196	134.4644	119.5515	136.1736
119.498	107.3806	119.4187	133.5021	119.4016	135.4155	119.5082	132.4293	119.4362	134.4673	119.5682	136.1688
119.5146	107.3788	119.4354	133.5057	119.4183	135.4128	119.5248	132.4273	119.4529	134.4657	119.5849	136.1676
119.5313	107.3827	119.4521	133.5049	119.4349	135.4125	119.5415	132.4284	119.4696	134.4659	119.6015	136.1631
119.548	107.3782	119.4687	133.5039	119.4516	135.4142	119.5582	132.428	119.4862	134.4669	119.6182	136.1606
119.5646	107.3785	119.4854	133.5034	119.4683	135.415	119.5748	132.4262	119.5029	134.4658	119.6349	136.1582
119.5813	107.3753	119.5021	133.5044	119.4849	135.4115	119.5915	132.4266	119.5196	134.465	119.6515	136.156
119.598	107.3748	119.5187	133.5042	119.5016	135.4115	119.6082	132.4259	119.5362	134.4631	119.6682	136.1517
119.6146	107.3753	119.5354	133.5058	119.5183	135.4128	119.6248	132.4242	119.5529	134.4681	119.6849	136.1516
119.6313	107.3745	119.5521	133.5034	119.5349	135.4115	119.6415	132.4257	119.5696	134.4632	119.7015	136.1482
119.648	107.3729	119.5687	133.5028	119.5516	135.4113	119.6582	132.4228	119.5862	134.4623	119.7182	136.1488
119.6646	107.3746	119.5854	133.5045	119.5683	135.4085	119.6748	132.4235	119.6029	134.4655	119.7349	136.1435
119.6813	107.3721	119.6021	133.5056	119.5849	135.412	119.6915	132.4235	119.6196	134.463	119.7515	136.1395

119.698	107.3702	119.6187	133.5003	119.6016	135.4093	119.7082	132.4264	119.6362	134.4629	119.7682	136.138		
119.7146	107.3683	119.6354	133.5028	119.6183	135.4094	119.7248	132.4241	119.6529	134.4626	119.7849	136.1356		
119.7313	107.369	119.6521	133.5015	119.6349	135.4063	119.7415	132.422	119.6696	134.4645	119.8015	136.1323		
119.748	107.3667	119.6687	133.5	119.6516	135.4085	119.7582	132.4238	119.6862	134.4606	119.8182	136.1265		
119.7646	107.3642	119.6854	133.5007	119.6683	135.4076	119.7748	132.4238	119.7029	134.4633	119.8349	136.1236		
119.7813	107.3684	119.7021	133.5004	119.6849	135.4073	119.7915	132.4232	119.7196	134.4613	119.8515	136.12		
119.798	107.3653	119.7187	133.4994	119.7016	135.4064	119.8082	132.42	119.7362	134.4608	119.8682	136.1175		
119.8146	107.3656	119.7354	133.4995	119.7183	135.4073	119.8248	132.42	119.7529	134.4619	119.8849	136.1132		
119.8313	107.3609	119.7521	133.5002	119.7349	135.4073	119.8415	132.4218	119.7696	134.4582	119.9015	136.1123		
119.848	107.3623	119.7687	133.4997	119.7516	135.4054	119.8582	132.4165	119.7862	134.4571	119.9182	136.1085		
119.8646	107.3643	119.7854	133.5012	119.7683	135.4071	119.8748	132.4231	119.8029	134.4598	119.9349	136.1072		
119.8813	107.3621	119.8021	133.4998	119.7849	135.4095	119.8915	132.4188	119.8196	134.4571	119.9515	136.1035		
119.898	107.3598	119.8187	133.4989	119.8016	135.4064	119.9082	132.4178	119.8362	134.4583	119.9682	136.1003		
119.9146	107.357	119.8354	133.4977	119.8183	135.4028	119.9248	132.4169	119.8529	134.4576	119.9849	136.0965		
119.9313	107.3587	119.8521	133.4995	119.8349	135.4063	119.9415	132.4136	119.8696	134.4577	120	136.0957		
119.948	107.3558	119.8687	133.4989	119.8516	135.4097	119.9582	132.4152	119.8862	134.4561			119.9025	128.6271
119.9646	107.3566	119.8854	133.4993	119.8683	135.4073	119.9748	132.414	119.9029	134.4573			119.9192	128.6269
119.9813	107.3535	119.9021	133.4976	119.8849	135.4024	119.9915	132.4126	119.9196	134.456			119.9359	128.6244
119.998	107.3564	119.9187	133.5	119.9016	135.4033	120	132.4109	119.9362	134.4538			119.9509	128.6249
120	107.3515	119.9354	133.4986	119.9183	135.4069			119.9529	134.4546				
		119.9521	133.5008	119.9349	135.4017			119.9696	134.4551				
		119.9687	133.4966	119.9516	135.4024			119.9862	134.4533				
		119.9854	133.4972	119.9683	135.4018			120	134.4521				
		120	133.4965	119.9849	135.401								
				120	135.4005								

PROMETHE	1		2		3		4		5		6	
	Time /mins	Strap Tensi	Time /mins	Strap Tensi	Time /mins	Strap Tensi	Time /mins	Strap Tensi	Time /mins	Strap Tensi	Time /mins	Strap Tension /N
	0	150.029	0	150.067	0	150.0277	0	150.0171	0	150.0015	0	150.0047
	0.0179	149.1534	0.034533	149.6139	0.017667	150.0715	0.020283	148.1042	1.67E-05	150.0072	1.67E-05	150.011
	0.034567	148.2874	0.0512	149.2128	0.034333	149.6092	0.03695	147.3102	0.016683	145.5351	0.016683	145.4687
	0.051233	147.5876	0.067867	148.8397	0.051	149.2034	0.053617	146.7334	0.03335	143.4795	0.03335	143.4712
	0.0679	146.9757	0.084533	148.4836	0.067667	148.8262	0.070283	146.2692	0.050017	142.0274	0.050017	142.1084
	0.084567	146.424	0.1012	148.1531	0.084333	148.488	0.08695	145.863	0.066683	140.8647	0.066683	141.0324
	0.101233	145.9237	0.117867	147.8401	0.101	148.1623	0.103617	145.4948	0.08335	139.8884	0.08335	140.1312
	0.1179	145.4446	0.134533	147.5423	0.117667	147.854	0.120283	145.1666	0.100017	139.0406	0.100017	139.367
	0.134567	144.9965	0.1512	147.2561	0.134333	147.5615	0.13695	144.865	0.116683	138.3115	0.116683	138.697
	0.151233	144.5907	0.167867	146.9837	0.151	147.2852	0.153617	144.5797	0.13335	137.6586	0.13335	138.094
	0.1679	144.2105	0.184533	146.7215	0.167667	147.0228	0.170283	144.3084	0.150017	137.0766	0.150017	137.5539
	0.184567	143.8307	0.2012	146.4699	0.184333	146.7501	0.18695	144.0521	0.166683	136.5417	0.166683	137.0617
	0.201233	143.4864	0.217867	146.2272	0.201	146.5032	0.203617	143.8177	0.18335	136.0602	0.18335	136.6059
	0.2179	143.1615	0.234533	145.9931	0.217667	146.2645	0.220283	143.5867	0.200017	135.6096	0.200017	136.1838
	0.234567	142.8498	0.2512	145.7692	0.234333	146.0305	0.23695	143.3645	0.216683	135.1936	0.216683	135.7735
	0.251233	142.5427	0.267867	145.5512	0.251	145.8057	0.253617	143.154	0.23335	134.7947	0.23335	135.4072
	0.2679	142.2608	0.284533	145.34	0.267667	145.588	0.270283	142.9503	0.250017	134.4296	0.250017	135.0667
	0.284567	141.9864	0.3012	145.1362	0.284333	145.3788	0.28695	142.7544	0.266683	134.0839	0.266683	134.7428
	0.301233	141.7216	0.317867	144.9415	0.301	145.1691	0.303617	142.5658	0.28335	133.7536	0.28335	134.4327
	0.3179	141.4749	0.334533	144.7493	0.317667	144.9665	0.320283	142.3842	0.300017	133.4382	0.300017	134.1424
	0.334567	141.2133	0.3512	144.5632	0.334333	144.7686	0.33695	142.2057	0.316683	133.1386	0.316683	133.8674
	0.351233	140.9808	0.367867	144.3815	0.351	144.5801	0.353617	142.0336	0.33335	132.8534	0.33335	133.6054
	0.3679	140.7574	0.384533	144.2083	0.367667	144.3949	0.370283	141.8607	0.350017	132.5772	0.350017	133.3494
	0.384567	140.5406	0.4012	144.0306	0.384333	144.2077	0.38695	141.6972	0.366683	132.3137	0.366683	133.1069
	0.401233	140.3236	0.417867	143.8686	0.401	144.0307	0.403617	141.5393	0.38335	132.0627	0.38335	132.8751
	0.4179	140.1272	0.434533	143.7037	0.417667	143.8594	0.420283	141.3858	0.400017	131.8152	0.400017	132.6494
	0.434567	139.9302	0.4512	143.5447	0.434333	143.6921	0.43695	141.233	0.416683	131.5808	0.416683	132.4342
	0.451233	139.7376	0.467867	143.3918	0.451	143.5272	0.453617	141.0887	0.43335	131.3506	0.43335	132.225
	0.4679	139.5537	0.484533	143.2387	0.467667	143.3682	0.470283	140.9452	0.450017	131.1298	0.450017	132.0254
	0.484567	139.3777	0.5012	143.0919	0.484333	143.2112	0.48695	140.8064	0.466683	130.9189	0.466683	131.8328
	0.501233	139.1857	0.517867	142.9472	0.501	143.055	0.503617	140.6632	0.48335	130.7105	0.48335	131.6473
	0.5179	139.0155	0.534533	142.8074	0.517667	142.9031	0.520283	140.5313	0.500017	130.5095	0.500017	131.4612
	0.534567	138.8484	0.5512	142.6671	0.534333	142.7566	0.53695	140.3964	0.516683	130.3126	0.516683	131.2851
	0.551233	138.6885	0.567867	142.5329	0.551	142.6141	0.553617	140.2707	0.53335	130.1234	0.53335	131.1149
	0.5679	138.5263	0.584533	142.397	0.567667	142.4712	0.570283	140.1415	0.550017	129.9373	0.550017	130.9448
	0.584567	138.3773	0.6012	142.2644	0.584333	142.327	0.58695	140.0185	0.566683	129.758	0.566683	130.7808
	0.601233	138.2227	0.617867	142.1379	0.601	142.1952	0.603617	139.8952	0.58335	129.5785	0.58335	130.6232
	0.6179	138.0716	0.634533	142.0122	0.617667	142.0594	0.620283	139.7726	0.600017	129.4114	0.600017	130.4654
	0.634567	137.9317	0.6512	141.8928	0.634333	141.9276	0.63695	139.6529	0.616683	129.2379	0.616683	130.3164
	0.651233	137.7885	0.667867	141.768	0.651	141.7964	0.653617	139.5371	0.63335	129.0723	0.63335	130.1712
	0.6679	137.6518	0.684533	141.6554	0.667667	141.6715	0.670283	139.4253	0.650017	128.9116	0.650017	130.0262
	0.684567	137.5168	0.7012	141.5377	0.684333	141.5476	0.68695	139.3105	0.666683	128.7553	0.666683	129.8877
	0.701233	137.3797	0.717867	141.425	0.701	141.4235	0.703617	139.1997	0.68335	128.5995	0.68335	129.7503
	0.7179	137.2521	0.734533	141.3114	0.717667	141.2982	0.720283	139.0928	0.700017	128.4515	0.700017	129.6168
	0.734567	137.1201	0.7512	141.2032	0.734333	141.1782	0.73695	138.9858	0.716683	128.3063	0.716683	129.4843
	0.751233	136.9955	0.767867	141.0962	0.751	141.0622	0.753617	138.8758	0.73335	128.1619	0.73335	129.3584
	0.7679	136.8688	0.784533	140.9893	0.767667	140.9438	0.770283	138.7723	0.750017	128.021	0.750017	129.2308
	0.784567	136.7512	0.8012	140.8863	0.784333	140.8308	0.78695	138.6708	0.766683	127.8796	0.766683	129.1031
	0.801233	136.6314	0.817867	140.7821	0.801	140.7201	0.803617	138.5676	0.78335	127.7444	0.78335	128.9835
	0.8179	136.5154	0.834533	140.6808	0.817667	140.6111	0.820283	138.4719	0.800017	127.6105	0.800017	128.8667
	0.834567	136.4032	0.8512	140.5808	0.834333	140.4952	0.83695	138.3766	0.816683	127.4784	0.816683	128.752
	0.851233	136.29	0.867867	140.4887	0.851	140.3842	0.853617	138.2808	0.83335	127.3442	0.83335	128.6367
	0.8679	136.1774	0.884533	140.3927	0.867667	140.2797	0.870283	138.1879	0.850017	127.2181	0.850017	128.5263
	0.884567	136.0715	0.9012	140.2953	0.884333	140.1731	0.88695	138.0936	0.866683	127.0873	0.866683	128.4177
	0.901233	135.9664	0.917867	140.2064	0.901	140.0698	0.903617	138.0052	0.88335	126.9595	0.88335	128.3102
	0.9179	135.8589	0.934533	140.112	0.917667	139.9737	0.920283	137.911	0.900017	126.83	0.900017	128.1984
	0.934567	135.7585	0.9512	140.0215	0.934333	139.8685	0.93695	137.8232	0.916683	126.6939	0.916683	128.095
	0.951233	135.6461	0.967867	139.9338	0.951	139.7709	0.953617	137.7325	0.93335	126.5466	0.93335	127.9905
	0.9679	135.5354	0.984533	139.8447	0.967667	139.6736	0.970283	137.6454	0.950017	126.3815	0.950017	127.8925
	0.984567	135.4307	1.0012	139.7593	0.984333	139.575	0.98695	137.555	0.966683	126.2149	0.966683	127.7955
	1.001233	135.3268	1.017867	139.6747	1.001	139.4793	1.003617	137.4709	0.98335	126.069	0.98335	127.7006
	1.0179	135.2286	1.034533	139.5874	1.017667	139.3872	1.020283	137.3838	1.000017	125.9417	1.000017	127.6049
	1.034567	135.1321	1.0512	139.505	1.034333	139.2908	1.03695	137.3015	1.016683	125.8253	1.016683	127.5128
	1.051233	135.0389	1.067867	139.4191	1.051	139.2075	1.053617	137.2197	1.03335	125.7148	1.03335	127.4247
	1.0679	134.9453	1.084533	139.3378	1.067667	139.1183	1.070283	137.1387	1.050017	125.6096	1.050017	127.3325
	1.084567	134.8509	1.1012	139.259	1.084333	139.0277	1.08695	137.0608	1.066683	125.5072	1.066683	127.2464
	1.101233	134.7621	1.117867	139.1806	1.101	138.9392	1.103617	136.9796	1.08335	125.4074	1.08335	127.1601
	1.1179	134.6751	1.134533	139.0998	1.117667	138.8532	1.120283	136.9006	1.100017	125.3121	1.100017	127.0712
	1.134567	134.5772	1.1512	139.0225	1.134333	138.7685	1.13695	136.8213	1.116683	125.2146	1.116683	126.9867
	1.151233	134.4922	1.167867	138.9486	1.151	138.6833	1.153617	136.7448	1.13335	125.1199	1.13335	126.9008
	1.1679	134.4015	1.184533	138.8678	1.167667	138.5961	1.170283	136.6668	1.150017	125.0301	1.150017	126.8195
	1.184567	134.3135	1.2012	138.7984	1.184333	138.513	1.18695	136.5903	1.166683	124.9359	1.166683	126.7338
	1.201233	134.2311	1.217867	138.7204	1.201	138.4302	1.203617	136.5131	1.18335	124.848	1.18335	126.6588
	1.2179	134.1499	1.234533	138.646	1.217667	138.3492	1.220283	136.4365	1.200017	124.7584	1.200017	126.5766
	1.234567	134.0671	1.2512	138.5764	1.234333	138.2716	1.23695	136.3626	1.216683	124.6711	1.216683	126.4982
	1.251233	133.9848	1.267867	138.5035	1.251	138.1885	1.253617	136.2936	1.23335	124.5845	1.23335	126.4213
	1.2679	133.9085	1.284533	138.4322	1.267667	138.1077	1.270283	136.2192	1.250017	124.5028	1.250017	126.3457

1.284567	133.8298	1.3012	138.3584	1.284333	138.03	1.28695	136.144	1.266683	124.4144	1.266683	126.2741
1.301233	133.7523	1.317867	138.2936	1.301	137.9554	1.303617	136.0773	1.28335	124.332	1.28335	126.1959
1.3179	133.6779	1.334533	138.2243	1.317667	137.8774	1.320283	136.0085	1.300017	124.2484	1.300017	126.1228
1.334567	133.6034	1.3512	138.153	1.334333	137.7991	1.33695	135.937	1.316683	124.1668	1.316683	126.0515
1.351233	133.5282	1.367867	138.086	1.351	137.7286	1.353617	135.8731	1.33335	124.0866	1.33335	125.9777
1.3679	133.4563	1.384533	138.023	1.367667	137.651	1.370283	135.8076	1.350017	124.0069	1.350017	125.9087
1.384567	133.3857	1.4012	137.9548	1.384333	137.5771	1.38695	135.7465	1.366683	123.9276	1.366683	125.8386
1.401233	133.3116	1.417867	137.8938	1.401	137.5041	1.403617	135.6753	1.38335	123.8481	1.38335	125.7685
1.4179	133.2397	1.434533	137.8249	1.417667	137.4349	1.420283	135.6123	1.400017	123.7719	1.400017	125.7029
1.434567	133.1701	1.4512	137.7646	1.434333	137.3608	1.43695	135.5481	1.416683	123.6963	1.416683	125.6352
1.451233	133.1014	1.467867	137.7034	1.451	137.2956	1.453617	135.4872	1.43335	123.6233	1.43335	125.5673
1.4679	133.0304	1.484533	137.6377	1.467667	137.2224	1.470283	135.4205	1.450017	123.5484	1.450017	125.5041
1.484567	132.9625	1.5012	137.5712	1.484333	137.1481	1.48695	135.3585	1.466683	123.471	1.466683	125.4391
1.501233	132.8981	1.517867	137.5111	1.501	137.0865	1.503617	135.2971	1.48335	123.3991	1.48335	125.3741
1.5179	132.8283	1.534533	137.4524	1.517667	137.0153	1.520283	135.2361	1.500017	123.3255	1.500017	125.3087
1.534567	132.7623	1.5512	137.3898	1.534333	136.9497	1.53695	135.1769	1.516683	123.2546	1.516683	125.2487
1.551233	132.6966	1.567867	137.329	1.551	136.8751	1.553617	135.1175	1.53335	123.1844	1.53335	125.1858
1.5679	132.634	1.584533	137.2704	1.567667	136.8172	1.570283	135.053	1.550017	123.1101	1.550017	125.1233
1.584567	132.5704	1.6012	137.2111	1.584333	136.7532	1.58695	134.9982	1.566683	123.0403	1.566683	125.0644
1.601233	132.5078	1.617867	137.1519	1.601	136.6874	1.603617	134.9396	1.58335	122.9733	1.58335	125.0025
1.6179	132.439	1.634533	137.098	1.617667	136.6229	1.620283	134.8804	1.600017	122.9065	1.600017	124.9435
1.634567	132.381	1.6512	137.0426	1.634333	136.5632	1.63695	134.8217	1.616683	122.839	1.616683	124.8844
1.651233	132.317	1.667867	136.9841	1.651	136.4988	1.653617	134.7664	1.63335	122.7731	1.63335	124.8266
1.6679	132.254	1.684533	136.9264	1.667667	136.4337	1.670283	134.7082	1.650017	122.7067	1.650017	124.7725
1.684567	132.1952	1.7012	136.8698	1.684333	136.3757	1.68695	134.6484	1.666683	122.6433	1.666683	124.7172
1.701233	132.1323	1.717867	136.8157	1.701	136.3163	1.703617	134.5942	1.68335	122.5777	1.68335	124.6603
1.7179	132.0755	1.734533	136.7644	1.717667	136.2601	1.720283	134.5385	1.700017	122.5147	1.700017	124.6036
1.734567	132.0171	1.7512	136.7079	1.734333	136.1941	1.73695	134.4813	1.716683	122.4514	1.716683	124.5477
1.751233	131.957	1.767867	136.6539	1.751	136.1356	1.753617	134.4301	1.73335	122.3904	1.73335	124.4929
1.7679	131.9034	1.784533	136.6029	1.767667	136.0771	1.770283	134.3766	1.750017	122.3304	1.750017	124.437
1.784567	131.8427	1.8012	136.548	1.784333	136.0227	1.78695	134.3207	1.766683	122.2653	1.766683	124.3836
1.801233	131.7882	1.817867	136.4962	1.801	135.9573	1.803617	134.2669	1.78335	122.2041	1.78335	124.3319
1.8179	131.732	1.834533	136.4441	1.817667	135.9021	1.820283	134.2078	1.800017	122.1432	1.800017	124.2787
1.834567	131.6795	1.8512	136.3894	1.834333	135.8467	1.83695	134.1542	1.816683	122.0829	1.816683	124.2262
1.851233	131.6246	1.867867	136.3442	1.851	135.7879	1.853617	134.1035	1.83335	122.0255	1.83335	124.1752
1.8679	131.5717	1.884533	136.2916	1.867667	135.7296	1.870283	134.0501	1.850017	121.9656	1.850017	124.1238
1.884567	131.5175	1.9012	136.2386	1.884333	135.6756	1.88695	134.0014	1.866683	121.9071	1.866683	124.0723
1.901233	131.4641	1.917867	136.1883	1.901	135.6187	1.903617	133.9461	1.88335	121.8515	1.88335	124.0241
1.9179	131.412	1.934533	136.1402	1.917667	135.5619	1.920283	133.8929	1.900017	121.7928	1.900017	123.973
1.934567	131.3575	1.9512	136.0895	1.934333	135.5059	1.93695	133.8381	1.916683	121.7327	1.916683	123.9241
1.951233	131.304	1.967867	136.0431	1.951	135.4503	1.953617	133.79	1.93335	121.6782	1.93335	123.8748
1.9679	131.2594	1.984533	135.9933	1.967667	135.3957	1.970283	133.7348	1.950017	121.6232	1.950017	123.8237
1.984567	131.2041	2.0012	135.9441	1.984333	135.3402	1.98695	133.6873	1.966683	121.5679	1.966683	123.7782
2.001233	131.1555	2.017867	135.897	2.001	135.2871	2.003617	133.6394	1.98335	121.5123	1.98335	123.7302
2.0179	131.102	2.034533	135.8497	2.017667	135.2341	2.020283	133.5903	2.000017	121.4595	2.000017	123.682
2.034567	131.0534	2.0512	135.8037	2.034333	135.1806	2.03695	133.5408	2.016683	121.4027	2.016683	123.6342
2.051233	131.0058	2.067867	135.7564	2.051	135.1262	2.053617	133.4925	2.03335	121.347	2.03335	123.5874
2.0679	130.9545	2.084533	135.7087	2.067667	135.0752	2.070283	133.4428	2.050017	121.2952	2.050017	123.5398
2.084567	130.9059	2.1012	135.6617	2.084333	135.0256	2.08695	133.3967	2.066683	121.2407	2.066683	123.4942
2.101233	130.8575	2.117867	135.617	2.101	134.9726	2.103617	133.349	2.08335	121.1892	2.08335	123.4479
2.1179	130.8133	2.134533	135.5693	2.117667	134.9207	2.120283	133.3042	2.100017	121.1406	2.100017	123.4018
2.134567	130.7601	2.1512	135.5253	2.134333	134.868	2.13695	133.253	2.116683	121.087	2.116683	123.3549
2.151233	130.7123	2.167867	135.4805	2.151	134.8198	2.153617	133.2108	2.13335	121.0357	2.13335	123.3125
2.1679	130.6678	2.184533	135.4329	2.167667	134.7716	2.170283	133.1647	2.150017	120.9819	2.150017	123.2695
2.184567	130.6198	2.2012	135.3916	2.184333	134.7217	2.18695	133.1208	2.166683	120.9339	2.166683	123.2268
2.201233	130.5685	2.217867	135.346	2.201	134.6723	2.203617	133.074	2.18335	120.8825	2.18335	123.1803
2.2179	130.5216	2.234533	135.3082	2.217667	134.62	2.220283	133.0269	2.200017	120.834	2.200017	123.1391
2.234567	130.4768	2.2512	135.2623	2.234333	134.5686	2.23695	132.9814	2.216683	120.7853	2.216683	123.0938
2.251233	130.4297	2.267867	135.2235	2.251	134.5272	2.253617	132.9402	2.23335	120.7322	2.23335	123.0545
2.2679	130.3852	2.284533	135.1761	2.267667	134.4732	2.270283	132.8955	2.250017	120.6824	2.250017	123.0117
2.284567	130.3373	2.3012	135.1358	2.284333	134.4287	2.28695	132.854	2.266683	120.6334	2.266683	122.968
2.301233	130.2906	2.317867	135.0928	2.301	134.3755	2.303617	132.8084	2.28335	120.5859	2.28335	122.9264
2.3179	130.2477	2.334533	135.0492	2.317667	134.3341	2.320283	132.7657	2.300017	120.5385	2.300017	122.8838
2.334567	130.2037	2.3512	135.0096	2.334333	134.2866	2.33695	132.7228	2.316683	120.4938	2.316683	122.844
2.351233	130.1568	2.367867	134.9672	2.351	134.2414	2.353617	132.6791	2.33335	120.4442	2.33335	122.801
2.3679	130.1147	2.384533	134.9224	2.367667	134.192	2.370283	132.6385	2.350017	120.3962	2.350017	122.7628
2.384567	130.0727	2.4012	134.8869	2.384333	134.15	2.38695	132.5993	2.366683	120.3489	2.366683	122.7211
2.401233	130.0263	2.417867	134.8411	2.401	134.1045	2.403617	132.5526	2.38335	120.304	2.38335	122.6814
2.4179	129.9829	2.434533	134.8022	2.417667	134.0614	2.420283	132.5101	2.400017	120.2566	2.400017	122.6426
2.434567	129.9303	2.4512	134.7637	2.434333	134.0156	2.43695	132.4696	2.416683	120.213	2.416683	122.6032
2.451233	129.8861	2.467867	134.7211	2.451	133.9719	2.453617	132.4264	2.43335	120.169	2.43335	122.5635
2.4679	129.8427	2.484533	134.6818	2.467667	133.9252	2.470283	132.3877	2.450017	120.122	2.450017	122.5268
2.484567	129.8007	2.5012	134.6428	2.484333	133.8856	2.48695	132.3485	2.466683	120.0786	2.466683	122.4873
2.501233	129.7599	2.517867	134.6027	2.501	133.8372	2.503617	132.3078	2.48335	120.032	2.48335	122.4471
2.5179	129.717	2.534533	134.5668	2.517667	133.7915	2.520283	132.2673	2.500017	119.987	2.500017	122.4076
2.534567	129.6768	2.5512	134.5286	2.534333	133.749	2.53695	132.2264	2.516683	119.943	2.516683	122.372
2.551233	129.6291	2.567867	134.4889	2.551	133.7095	2.553617	132.1877	2.53335	119.9013	2.53335	122.3336
2.5679	129.5883	2.584533	134.4499	2.567667	133.6642	2.570283	132.148	2.550017	119.8556	2.550017	122.2976
2.584567	129.5451	2.6012	134.4107	2.584333	133.6197	2.58695	132.1127	2.566683	119.8122	2.566683	122.2584

2.601233	129.503	2.617867	134.3748	2.601	133.5783	2.603617	132.0716	2.58335	119.7717	2.58335	122.218
2.6179	129.4667	2.634533	134.3361	2.617667	133.5368	2.620283	132.0322	2.600017	119.7259	2.600017	122.1811
2.634567	129.4236	2.6512	134.3002	2.634333	133.495	2.63695	131.9933	2.616683	119.6862	2.616683	122.1464
2.651233	129.3865	2.667867	134.2635	2.651	133.4506	2.653617	131.953	2.63335	119.6423	2.63335	122.106
2.6679	129.3457	2.684533	134.2267	2.667667	133.4088	2.670283	131.9162	2.650017	119.6018	2.650017	122.0734
2.684567	129.3036	2.7012	134.1909	2.684333	133.3667	2.68695	131.8816	2.666683	119.5596	2.666683	122.0354
2.701233	129.2637	2.717867	134.1545	2.701	133.3281	2.703617	131.844	2.68335	119.5158	2.68335	122.001
2.7179	129.2285	2.734533	134.1201	2.717667	133.2867	2.720283	131.8042	2.700017	119.4769	2.700017	121.9661
2.734567	129.1884	2.7512	134.0834	2.734333	133.2446	2.73695	131.7653	2.716683	119.4386	2.716683	121.9246
2.751233	129.1528	2.767867	134.0494	2.751	133.2036	2.753617	131.7253	2.73335	119.3943	2.73335	121.891
2.7679	129.1111	2.784533	134.0118	2.767667	133.1649	2.770283	131.6905	2.750017	119.3526	2.750017	121.8567
2.784567	129.0751	2.8012	133.975	2.784333	133.1226	2.78695	131.6538	2.766683	119.3143	2.766683	121.8211
2.801233	129.041	2.817867	133.9384	2.801	133.0807	2.803617	131.6182	2.78335	119.2733	2.78335	121.7911
2.8179	128.9982	2.834533	133.904	2.817667	133.0431	2.820283	131.5809	2.800017	119.2318	2.800017	121.7545
2.834567	128.9648	2.8512	133.8686	2.834333	133.0029	2.83695	131.5447	2.816683	119.1938	2.816683	121.7224
2.851233	128.9269	2.867867	133.8344	2.851	132.9612	2.853617	131.5099	2.83335	119.155	2.83335	121.6891
2.8679	128.8896	2.884533	133.7987	2.867667	132.9197	2.870283	131.4731	2.850017	119.1198	2.850017	121.6552
2.884567	128.8496	2.9012	133.7634	2.884333	132.8833	2.88695	131.4412	2.866683	119.0793	2.866683	121.6224
2.901233	128.8138	2.917867	133.7305	2.901	132.8459	2.903617	131.3999	2.88335	119.0421	2.88335	121.5899
2.9179	128.7767	2.934533	133.6961	2.917667	132.8089	2.920283	131.3684	2.900017	119.0007	2.900017	121.5561
2.934567	128.7413	2.9512	133.6601	2.934333	132.7674	2.93695	131.3342	2.916683	118.9602	2.916683	121.529
2.951233	128.7056	2.967867	133.6283	2.951	132.7302	2.953617	131.299	2.93335	118.922	2.93335	121.4957
2.9679	128.6671	2.984533	133.597	2.967667	132.6937	2.970283	131.2634	2.950017	118.8868	2.950017	121.4652
2.984567	128.6346	3.0012	133.5624	2.984333	132.6576	2.98695	131.2299	2.966683	118.8498	2.966683	121.4324
3.001233	128.6007	3.017867	133.5302	3.001	132.6178	3.003617	131.1973	2.98335	118.8132	2.98335	121.4015
3.0179	128.5624	3.034533	133.4963	3.017667	132.5821	3.020283	131.1613	3.000017	118.7738	3.000017	121.3706
3.034567	128.5293	3.0512	133.463	3.034333	132.5473	3.03695	131.1249	3.016683	118.7368	3.016683	121.3401
3.051233	128.4961	3.067867	133.4317	3.051	132.5131	3.053617	131.0931	3.03335	118.7002	3.03335	121.3071
3.0679	128.4616	3.084533	133.3961	3.067667	132.4754	3.070283	131.0578	3.050017	118.6632	3.050017	121.2785
3.084567	128.43	3.1012	133.3646	3.084333	132.4377	3.08695	131.0233	3.066683	118.6287	3.066683	121.2483
3.101233	128.3961	3.117867	133.3322	3.101	132.4034	3.103617	130.9915	3.08335	118.5918	3.08335	121.2148
3.1179	128.363	3.134533	133.2981	3.117667	132.3694	3.120283	130.9597	3.100017	118.5554	3.100017	121.1854
3.134567	128.3268	3.1512	133.2658	3.134333	132.3322	3.13695	130.9269	3.116683	118.5206	3.116683	121.157
3.151233	128.2888	3.167867	133.2321	3.151	132.2958	3.153617	130.8958	3.13335	118.4849	3.13335	121.1246
3.1679	128.2581	3.184533	133.1981	3.167667	132.2596	3.170283	130.8637	3.150017	118.4483	3.150017	121.0933
3.184567	128.2274	3.2012	133.1681	3.184333	132.2282	3.18695	130.8291	3.166683	118.4123	3.166683	121.0671
3.201233	128.1924	3.217867	133.1359	3.201	132.1932	3.203617	130.7982	3.18335	118.3771	3.18335	121.0365
3.2179	128.1601	3.234533	133.1055	3.217667	132.1557	3.220283	130.7649	3.200017	118.3439	3.200017	121.006
3.234567	128.1292	3.2512	133.075	3.234333	132.1215	3.23695	130.7315	3.216683	118.309	3.216683	120.978
3.251233	128.0925	3.267867	133.0432	3.251	132.0856	3.253617	130.7017	3.23335	118.2767	3.23335	120.9479
3.2679	128.0624	3.284533	133.0152	3.267667	132.0517	3.270283	130.6698	3.250017	118.243	3.250017	120.9203
3.284567	128.0254	3.3012	132.9839	3.284333	132.0189	3.28695	130.6387	3.266683	118.2064	3.266683	120.8915
3.301233	127.9944	3.317867	132.9499	3.301	131.9826	3.303617	130.6054	3.28335	118.1733	3.28335	120.8653
3.3179	127.9625	3.334533	132.9215	3.317667	131.9521	3.320283	130.5724	3.300017	118.14	3.300017	120.8352
3.334567	127.9353	3.3512	132.8914	3.334333	131.9177	3.33695	130.5409	3.316683	118.1054	3.316683	120.8078
3.351233	127.9	3.367867	132.8573	3.351	131.882	3.353617	130.51	3.33335	118.0715	3.33335	120.7833
3.3679	127.8684	3.384533	132.8302	3.367667	131.8519	3.370283	130.477	3.350017	118.0375	3.350017	120.7545
3.384567	127.8361	3.4012	132.7965	3.384333	131.8163	3.38695	130.4491	3.366683	118.0045	3.366683	120.7254
3.401233	127.8043	3.417867	132.7704	3.401	131.7827	3.403617	130.4169	3.38335	117.9742	3.38335	120.7011
3.4179	127.7732	3.434533	132.7386	3.417667	131.7484	3.420283	130.385	3.400017	117.9409	3.400017	120.6731
3.434567	127.7389	3.4512	132.7097	3.434333	131.714	3.43695	130.3546	3.416683	117.9097	3.416683	120.6457
3.451233	127.7033	3.467867	132.6814	3.451	131.6801	3.453617	130.3247	3.43335	117.8788	3.43335	120.6187
3.4679	127.6755	3.484533	132.6469	3.467667	131.6476	3.470283	130.2944	3.450017	117.8454	3.450017	120.5921
3.484567	127.6452	3.5012	132.6216	3.484333	131.6195	3.48695	130.2627	3.466683	117.8148	3.466683	120.5661
3.501233	127.6137	3.517867	132.5946	3.501	131.585	3.503617	130.232	3.48335	117.7839	3.48335	120.5394
3.5179	127.584	3.534533	132.5614	3.517667	131.5518	3.520283	130.2012	3.500017	117.7504	3.500017	120.5101
3.534567	127.5533	3.5512	132.5352	3.534333	131.5236	3.53695	130.17	3.516683	117.7193	3.516683	120.4851
3.551233	127.52	3.567867	132.5021	3.551	131.4878	3.553617	130.1453	3.53335	117.6898	3.53335	120.4578
3.5679	127.4924	3.584533	132.4768	3.567667	131.456	3.570283	130.1146	3.550017	117.6547	3.550017	120.4318
3.584567	127.4631	3.6012	132.4467	3.584333	131.4266	3.58695	130.0863	3.566683	117.6257	3.566683	120.4065
3.601233	127.4308	3.617867	132.4182	3.601	131.3941	3.603617	130.0505	3.58335	117.595	3.58335	120.3767
3.6179	127.4054	3.634533	132.3947	3.617667	131.3643	3.620283	130.0254	3.600017	117.5624	3.600017	120.3544
3.634567	127.3726	3.6512	132.3606	3.634333	131.334	3.63695	129.9945	3.616683	117.5343	3.616683	120.3276
3.651233	127.3454	3.667867	132.3359	3.651	131.3035	3.653617	129.9652	3.63335	117.4991	3.63335	120.3021
3.6679	127.3174	3.684533	132.3093	3.667667	131.2724	3.670283	129.9379	3.650017	117.472	3.650017	120.279
3.684567	127.285	3.7012	132.2816	3.684333	131.2372	3.68695	129.9097	3.666683	117.4377	3.666683	120.2528
3.701233	127.26	3.717867	132.2527	3.701	131.2112	3.703617	129.8808	3.68335	117.4052	3.68335	120.2261
3.7179	127.2292	3.734533	132.2264	3.717667	131.1797	3.720283	129.8502	3.700017	117.3759	3.700017	120.2004
3.734567	127.2016	3.7512	132.1987	3.734333	131.1503	3.73695	129.8233	3.716683	117.3454	3.716683	120.1775
3.751233	127.1752	3.767867	132.1741	3.751	131.1205	3.753617	129.7931	3.73335	117.3154	3.73335	120.1533
3.7679	127.1443	3.784533	132.1437	3.767667	131.0899	3.770283	129.7687	3.750017	117.2853	3.750017	120.1267
3.784567	127.1173	3.8012	132.12	3.784333	131.0589	3.78695	129.7375	3.766683	117.256	3.766683	120.1054
3.801233	127.0866	3.817867	132.0914	3.801	131.0308	3.803617	129.7102	3.78335	117.2256	3.78335	120.077
3.8179	127.0585	3.834533	132.0638	3.817667	130.9994	3.820283	129.6824	3.800017	117.1991	3.800017	120.0544
3.834567	127.0372	3.8512	132.0343	3.834333	130.9687	3.83695	129.654	3.816683	117.1682	3.816683	120.0312
3.851233	127.0036	3.867867	132.004	3.851	130.9367	3.853617	129.6281	3.83335	117.1392	3.83335	120.0068
3.8679	126.9807	3.884533	131.9778	3.867667	130.9098	3.870283	129.6002	3.850017	117.111	3.850017	119.9833
3.884567	126.9548	3.9012	131.9496	3.884333	130.8778	3.88695	129.5734	3.866683	117.0829	3.866683	119.9589
3.901233	126.9277	3.917867	131.9224	3.901	130.8482	3.903617	129.5424	3.88335	117.0524	3.88335	119.9367

3.9179	126.8983	3.934533	131.899	3.917667	130.8231	3.920283	129.5161	3.900017	117.0242	3.900017	119.9131
3.934567	126.8748	3.9512	131.8714	3.934333	130.7893	3.93695	129.4861	3.916683	116.9955	3.916683	119.8884
3.951233	126.8466	3.967867	131.8457	3.951	130.7619	3.953617	129.4597	3.93335	116.9671	3.93335	119.8651
3.9679	126.8184	3.984533	131.8155	3.967667	130.7315	3.970283	129.4322	3.950017	116.9425	3.950017	119.8413
3.984567	126.7949	4.0012	131.7884	3.984333	130.7023	3.98695	129.4054	3.966683	116.9144	3.966683	119.8179
4.001233	126.7684	4.017867	131.7625	4.001	130.6794	4.003617	129.379	3.98335	116.8838	3.98335	119.7947
4.0179	126.7366	4.034533	131.7365	4.017667	130.6501	4.020283	129.3505	4.000017	116.856	4.000017	119.7714
4.034567	126.7135	4.0512	131.7135	4.034333	130.6247	4.03695	129.3267	4.016683	116.8315	4.016683	119.7468
4.051233	126.6888	4.067867	131.6862	4.051	130.596	4.053617	129.2977	4.03335	116.7989	4.03335	119.7239
4.0679	126.661	4.084533	131.6634	4.067667	130.568	4.070283	129.2722	4.050017	116.7729	4.050017	119.7012
4.084567	126.6381	4.1012	131.6382	4.084333	130.5399	4.08695	129.246	4.066683	116.7469	4.066683	119.6801
4.101233	126.6111	4.117867	131.612	4.101	130.5113	4.103617	129.2197	4.08335	116.7178	4.08335	119.6569
4.1179	126.583	4.134533	131.5891	4.117667	130.4837	4.120283	129.1906	4.100017	116.6907	4.100017	119.6345
4.134567	126.5574	4.1512	131.5548	4.134333	130.4598	4.13695	129.1684	4.116683	116.6604	4.116683	119.6158
4.151233	126.5309	4.167867	131.5405	4.151	130.4317	4.153617	129.1413	4.13335	116.6353	4.13335	119.5933
4.1679	126.5063	4.184533	131.5069	4.167667	130.4039	4.170283	129.1148	4.150017	116.6112	4.150017	119.5684
4.184567	126.4782	4.2012	131.4846	4.184333	130.3785	4.18695	129.0893	4.166683	116.5838	4.166683	119.551
4.201233	126.456	4.217867	131.4625	4.201	130.3513	4.203617	129.0636	4.18335	116.5578	4.18335	119.5258
4.2179	126.4273	4.234533	131.4398	4.217667	130.3224	4.220283	129.0399	4.200017	116.5307	4.200017	119.5044
4.234567	126.4027	4.2512	131.4093	4.234333	130.2968	4.23695	129.0153	4.216683	116.503	4.216683	119.4807
4.251233	126.3791	4.267867	131.3903	4.251	130.2702	4.253617	128.9915	4.23335	116.4747	4.23335	119.4582
4.2679	126.3555	4.284533	131.3584	4.267667	130.2421	4.270283	128.9637	4.250017	116.4491	4.250017	119.4393
4.284567	126.3251	4.3012	131.343	4.284333	130.2165	4.28695	128.9404	4.266683	116.4289	4.266683	119.4186
4.301233	126.3017	4.317867	131.3156	4.301	130.1889	4.303617	128.9175	4.28335	116.3971	4.28335	119.3941
4.3179	126.2775	4.334533	131.289	4.317667	130.1655	4.320283	128.8924	4.300017	116.3756	4.300017	119.3746
4.334567	126.2546	4.3512	131.2672	4.334333	130.1367	4.33695	128.8664	4.316683	116.3459	4.316683	119.3511
4.351233	126.2296	4.367867	131.2425	4.351	130.1111	4.353617	128.845	4.33335	116.3218	4.33335	119.3328
4.3679	126.2056	4.384533	131.2185	4.367667	130.0847	4.370283	128.8182	4.350017	116.2909	4.350017	119.3079
4.384567	126.1795	4.4012	131.1937	4.384333	130.0593	4.38695	128.7928	4.366683	116.2676	4.366683	119.2893
4.401233	126.1592	4.417867	131.172	4.401	130.0311	4.403617	128.7684	4.38335	116.242	4.38335	119.2685
4.4179	126.1317	4.434533	131.1451	4.417667	130.0077	4.420283	128.7455	4.400017	116.215	4.400017	119.2451
4.434567	126.1115	4.4512	131.1229	4.434333	129.9823	4.43695	128.7188	4.416683	116.1886	4.416683	119.2234
4.451233	126.0883	4.467867	131.1035	4.451	129.9554	4.453617	128.695	4.43335	116.1645	4.43335	119.2037
4.4679	126.0647	4.484533	131.0792	4.467667	129.93	4.470283	128.6701	4.450017	116.1387	4.450017	119.1844
4.484567	126.0399	4.5012	131.0573	4.484333	129.9039	4.48695	128.6478	4.466683	116.1159	4.466683	119.1651
4.501233	126.0186	4.517867	131.0355	4.501	129.8797	4.503617	128.622	4.48335	116.0907	4.48335	119.1423
4.5179	125.9946	4.534533	131.0116	4.517667	129.8558	4.520283	128.6	4.500017	116.0674	4.500017	119.1217
4.534567	125.9721	4.5512	130.9882	4.534333	129.8319	4.53695	128.576	4.516683	116.0386	4.516683	119.1036
4.551233	125.9497	4.567867	130.9682	4.551	129.8091	4.553617	128.554	4.53335	116.0128	4.53335	119.0832
4.5679	125.9265	4.584533	130.9452	4.567667	129.7789	4.570283	128.5294	4.550017	115.9896	4.550017	119.064
4.584567	125.9039	4.6012	130.921	4.584333	129.7531	4.58695	128.508	4.566683	115.9705	4.566683	119.0443
4.601233	125.8817	4.617867	130.898	4.601	129.7318	4.603617	128.482	4.58335	115.9397	4.58335	119.0245
4.6179	125.8556	4.634533	130.8778	4.617667	129.7059	4.620283	128.4626	4.600017	115.9199	4.600017	119.0032
4.634567	125.8359	4.6512	130.856	4.634333	129.6856	4.63695	128.44	4.616683	115.8934	4.616683	118.9806
4.651233	125.8133	4.667867	130.8329	4.651	129.6618	4.653617	128.4181	4.63335	115.8691	4.63335	118.9623
4.6679	125.7917	4.684533	130.809	4.667667	129.6323	4.670283	128.3951	4.650017	115.8484	4.650017	118.9448
4.684567	125.7686	4.7012	130.7842	4.684333	129.6116	4.68695	128.3685	4.666683	115.8221	4.666683	118.9275
4.701233	125.7453	4.717867	130.7641	4.701	129.5882	4.703617	128.3454	4.68335	115.7961	4.68335	118.9069
4.7179	125.7237	4.734533	130.7391	4.717667	129.5603	4.720283	128.3239	4.700017	115.7719	4.700017	118.8852
4.734567	125.6977	4.7512	130.7173	4.734333	129.5354	4.73695	128.3002	4.716683	115.747	4.716683	118.8702
4.751233	125.6746	4.767867	130.6958	4.751	129.5166	4.753617	128.2792	4.73335	115.7253	4.73335	118.8489
4.7679	125.6529	4.784533	130.6722	4.767667	129.4852	4.770283	128.255	4.750017	115.702	4.750017	118.8305
4.784567	125.6302	4.8012	130.6534	4.784333	129.4632	4.78695	128.2308	4.766683	115.6774	4.766683	118.8128
4.801233	125.6083	4.817867	130.6341	4.801	129.4343	4.803617	128.2104	4.78335	115.653	4.78335	118.7929
4.8179	125.5836	4.834533	130.6115	4.817667	129.4156	4.820283	128.1889	4.800017	115.6307	4.800017	118.7756
4.834567	125.5598	4.8512	130.5874	4.834333	129.3896	4.83695	128.1671	4.816683	115.6125	4.816683	118.7546
4.851233	125.539	4.867867	130.566	4.851	129.3662	4.853617	128.1444	4.83335	115.5894	4.83335	118.7339
4.8679	125.5176	4.884533	130.5427	4.867667	129.3424	4.870283	128.1219	4.850017	115.5628	4.850017	118.7184
4.884567	125.4953	4.9012	130.5224	4.884333	129.3186	4.88695	128.1004	4.866683	115.5429	4.866683	118.6992
4.901233	125.4736	4.917867	130.5014	4.901	129.2947	4.903617	128.0768	4.88335	115.5133	4.88335	118.6805
4.9179	125.453	4.934533	130.4786	4.917667	129.2719	4.920283	128.0549	4.900017	115.4918	4.900017	118.6619
4.934567	125.4307	4.9512	130.4567	4.934333	129.2456	4.93695	128.0349	4.916683	115.4704	4.916683	118.6426
4.951233	125.4091	4.967867	130.439	4.951	129.2265	4.953617	128.0123	4.93335	115.4472	4.93335	118.6255
4.9679	125.3872	4.984533	130.417	4.967667	129.2053	4.970283	127.9887	4.950017	115.4248	4.950017	118.6078
4.984567	125.366	5.0012	130.397	4.984333	129.1798	4.98695	127.9695	4.966683	115.4015	4.966683	118.587
5.001233	125.3434	5.017867	130.3762	5.001	129.1579	5.003617	127.9472	4.98335	115.3784	4.98335	118.5706
5.0179	125.3216	5.034533	130.3535	5.017667	129.1363	5.020283	127.926	5.000017	115.3582	5.000017	118.5513
5.034567	125.3013	5.0512	130.3315	5.034333	129.1123	5.03695	127.9027	5.016683	115.3322	5.016683	118.5313
5.051233	125.2793	5.067867	130.3129	5.051	129.0886	5.053617	127.8793	5.03335	115.3093	5.03335	118.5157
5.0679	125.2602	5.084533	130.2909	5.067667	129.066	5.070283	127.8606	5.050017	115.2871	5.050017	118.4974
5.084567	125.2391	5.1012	130.2722	5.084333	129.0408	5.08695	127.8373	5.066683	115.2662	5.066683	118.478
5.101233	125.2192	5.117867	130.2499	5.101	129.0192	5.103617	127.8164	5.08335	115.2446	5.08335	118.4585
5.1179	125.1977	5.134533	130.2286	5.117667	128.9993	5.120283	127.7929	5.100017	115.2219	5.100017	118.4443
5.134567	125.1774	5.1512	130.2096	5.134333	128.973	5.13695	127.7726	5.116683	115.1985	5.116683	118.4256
5.151233	125.1553	5.167867	130.1922	5.151	128.9504	5.153617	127.7499	5.13335	115.1772	5.13335	118.4063
5.1679	125.1358	5.184533	130.1688	5.167667	128.9302	5.170283	127.7323	5.150017	115.1551	5.150017	118.3899
5.184567	125.1166	5.2012	130.1496	5.184333	128.9059	5.18695	127.7121	5.166683	115.1344	5.166683	118.3723
5.201233	125.0904	5.217867	130.1299	5.201	128.8827	5.203617	127.6892	5.18335	115.1116	5.18335	118.3558
5.2179	125.0678	5.234533	130.1078	5.217667	128.864	5.220283	127.6692	5.200017	115.0901	5.200017	118.34

5.234567	125.0456	5.2512	130.0892	5.234333	128.8403	5.23695	127.6476	5.216683	115.0691	5.216683	118.3215
5.251233	125.0253	5.267867	130.0682	5.251	128.8222	5.253617	127.6229	5.23335	115.0478	5.23335	118.3065
5.2679	125.0058	5.284533	130.045	5.267667	128.7978	5.270283	127.6072	5.250017	115.0276	5.250017	118.2903
5.284567	124.9862	5.3012	130.0211	5.284333	128.7716	5.28695	127.5838	5.266683	115.0049	5.266683	118.2709
5.301233	124.9686	5.317867	130.0059	5.301	128.7512	5.303617	127.5628	5.28335	114.9844	5.28335	118.2534
5.3179	124.9471	5.334533	129.982	5.317667	128.7319	5.320283	127.5437	5.300017	114.9611	5.300017	118.2375
5.334567	124.9281	5.3512	129.9644	5.334333	128.7071	5.33695	127.5233	5.316683	114.9405	5.316683	118.2206
5.351233	124.9079	5.367867	129.9422	5.351	128.6889	5.353617	127.5033	5.33335	114.9185	5.33335	118.2064
5.3679	124.8879	5.384533	129.9257	5.367667	128.6657	5.370283	127.482	5.350017	114.8979	5.350017	118.1873
5.384567	124.8688	5.4012	129.9045	5.384333	128.6453	5.38695	127.4632	5.366683	114.8783	5.366683	118.1691
5.401233	124.8521	5.417867	129.8823	5.401	128.6291	5.403617	127.4426	5.38335	114.8579	5.38335	118.1525
5.4179	124.8301	5.434533	129.864	5.417667	128.6073	5.420283	127.4248	5.400017	114.8377	5.400017	118.1367
5.434567	124.8119	5.4512	129.8467	5.434333	128.5816	5.43695	127.4028	5.416683	114.8154	5.416683	118.1186
5.451233	124.7924	5.467867	129.8254	5.451	128.5633	5.453617	127.3828	5.43335	114.7954	5.43335	118.1023
5.4679	124.7732	5.484533	129.8045	5.467667	128.539	5.470283	127.3605	5.450017	114.7739	5.450017	118.0868
5.484567	124.7531	5.5012	129.7855	5.484333	128.5209	5.48695	127.3389	5.466683	114.7543	5.466683	118.0682
5.501233	124.7385	5.517867	129.7676	5.501	128.499	5.503617	127.3191	5.48335	114.7339	5.48335	118.0516
5.5179	124.71	5.534533	129.747	5.517667	128.481	5.520283	127.2986	5.500017	114.7166	5.500017	118.0355
5.534567	124.6966	5.5512	129.7305	5.534333	128.4601	5.53695	127.2807	5.516683	114.693	5.516683	118.0182
5.551233	124.6758	5.567867	129.7109	5.551	128.4369	5.553617	127.2625	5.53335	114.6722	5.53335	118.005
5.5679	124.6521	5.584533	129.692	5.567667	128.4176	5.570283	127.2421	5.550017	114.6518	5.550017	117.9883
5.584567	124.6383	5.6012	129.6762	5.584333	128.3973	5.58695	127.2209	5.566683	114.6293	5.566683	117.973
5.601233	124.6162	5.617867	129.6571	5.601	128.3758	5.603617	127.2028	5.58335	114.6117	5.58335	117.9558
5.6179	124.5925	5.634533	129.6382	5.617667	128.356	5.620283	127.183	5.600017	114.5898	5.600017	117.9385
5.634567	124.5773	5.6512	129.6202	5.634333	128.3354	5.63695	127.1618	5.616683	114.5729	5.616683	117.9234
5.651233	124.5578	5.667867	129.6016	5.651	128.3174	5.653617	127.1421	5.63335	114.5509	5.63335	117.9082
5.6679	124.5418	5.684533	129.5799	5.667667	128.2966	5.670283	127.1224	5.650017	114.531	5.650017	117.8932
5.684567	124.5187	5.7012	129.5612	5.684333	128.2753	5.68695	127.1025	5.666683	114.5129	5.666683	117.8775
5.701233	124.5045	5.717867	129.5423	5.701	128.2545	5.703617	127.0831	5.68335	114.4889	5.68335	117.8618
5.7179	124.4845	5.734533	129.5234	5.717667	128.2328	5.720283	127.0641	5.700017	114.4723	5.700017	117.8476
5.734567	124.4646	5.7512	129.5075	5.734333	128.2148	5.73695	127.0477	5.716683	114.4529	5.716683	117.8287
5.751233	124.4499	5.767867	129.4878	5.751	128.1927	5.753617	127.0288	5.73335	114.4327	5.73335	117.8155
5.7679	124.4321	5.784533	129.4718	5.767667	128.174	5.770283	127.0092	5.750017	114.4135	5.750017	117.7994
5.784567	124.4113	5.8012	129.4534	5.784333	128.156	5.78695	126.9861	5.766683	114.3947	5.766683	117.7829
5.801233	124.3932	5.817867	129.4338	5.801	128.1399	5.803617	126.9666	5.78335	114.3735	5.78335	117.768
5.8179	124.3748	5.834533	129.415	5.817667	128.1186	5.820283	126.9532	5.800017	114.3522	5.800017	117.7522
5.834567	124.359	5.8512	129.3979	5.834333	128.0969	5.83695	126.9308	5.816683	114.3361	5.816683	117.7358
5.851233	124.334	5.867867	129.3769	5.851	128.078	5.853617	126.9132	5.83335	114.3135	5.83335	117.7221
5.8679	124.32	5.884533	129.36	5.867667	128.0572	5.870283	126.8962	5.850017	114.2951	5.850017	117.7048
5.884567	124.3036	5.9012	129.341	5.884333	128.0378	5.88695	126.8745	5.866683	114.2776	5.866683	117.692
5.901233	124.2861	5.917867	129.3228	5.901	128.0196	5.903617	126.8601	5.88335	114.2539	5.88335	117.6773
5.9179	124.2681	5.934533	129.3052	5.917667	128.0028	5.920283	126.8392	5.900017	114.2386	5.900017	117.6614
5.934567	124.2506	5.9512	129.2862	5.934333	127.9818	5.93695	126.8209	5.916683	114.2193	5.916683	117.6461
5.951233	124.2287	5.967867	129.2687	5.951	127.9642	5.953617	126.8015	5.93335	114.1999	5.93335	117.6308
5.9679	124.214	5.984533	129.2512	5.967667	127.9457	5.970283	126.7843	5.950017	114.1807	5.950017	117.6162
5.984567	124.1948	6.0012	129.2338	5.984333	127.9265	5.98695	126.7682	5.966683	114.162	5.966683	117.6038
6.001233	124.1752	6.017867	129.2148	6.001	127.9067	6.003617	126.7504	5.98335	114.1472	5.98335	117.5876
6.0179	124.1606	6.034533	129.1988	6.017667	127.8872	6.020283	126.7285	6.000017	114.1249	6.000017	117.5722
6.034567	124.1413	6.0512	129.1802	6.034333	127.8652	6.03695	126.7124	6.016683	114.1056	6.016683	117.5589
6.051233	124.1232	6.067867	129.162	6.051	127.8472	6.053617	126.6934	6.03335	114.0869	6.03335	117.5431
6.0679	124.1091	6.084533	129.1453	6.067667	127.8261	6.070283	126.6751	6.050017	114.0671	6.050017	117.5289
6.084567	124.0885	6.1012	129.1277	6.084333	127.8077	6.08695	126.6604	6.066683	114.049	6.066683	117.5148
6.101233	124.0717	6.117867	129.1077	6.101	127.7904	6.103617	126.641	6.08335	114.0318	6.08335	117.4998
6.1179	124.0543	6.134533	129.092	6.117667	127.7719	6.120283	126.6217	6.100017	114.014	6.100017	117.4849
6.134567	124.0383	6.1512	129.0734	6.134333	127.7535	6.13695	126.602	6.116683	113.9947	6.116683	117.4716
6.151233	124.0223	6.167867	129.0566	6.151	127.7335	6.153617	126.5858	6.13335	113.9765	6.13335	117.4567
6.1679	124.002	6.184533	129.0364	6.167667	127.7169	6.170283	126.5663	6.150017	113.9577	6.150017	117.4395
6.184567	123.9882	6.2012	129.0214	6.184333	127.7003	6.18695	126.5549	6.166683	113.9375	6.166683	117.4264
6.201233	123.9709	6.217867	129.0073	6.201	127.6771	6.203617	126.5338	6.18335	113.9232	6.18335	117.4135
6.2179	123.9557	6.234533	128.9887	6.217667	127.6631	6.220283	126.5161	6.200017	113.9024	6.200017	117.3987
6.234567	123.9392	6.2512	128.9734	6.234333	127.6444	6.23695	126.4997	6.216683	113.8843	6.216683	117.3858
6.251233	123.919	6.267867	128.9569	6.251	127.6261	6.253617	126.4813	6.23335	113.8657	6.23335	117.3733
6.2679	123.9039	6.284533	128.939	6.267667	127.6061	6.270283	126.4658	6.250017	113.8486	6.250017	117.3584
6.284567	123.8834	6.3012	128.9228	6.284333	127.5868	6.28695	126.4453	6.266683	113.8289	6.266683	117.3438
6.301233	123.8643	6.317867	128.9052	6.301	127.5698	6.303617	126.4265	6.28335	113.8104	6.28335	117.3304
6.3179	123.8494	6.334533	128.8887	6.317667	127.5547	6.320283	126.4091	6.300017	113.792	6.300017	117.3151
6.334567	123.8319	6.3512	128.8728	6.334333	127.5355	6.33695	126.3922	6.316683	113.7753	6.316683	117.2986
6.351233	123.8118	6.367867	128.8587	6.351	127.5197	6.353617	126.3749	6.33335	113.757	6.33335	117.2864
6.3679	123.7984	6.384533	128.8389	6.367667	127.4965	6.370283	126.357	6.350017	113.7377	6.350017	117.2709
6.384567	123.7799	6.4012	128.8222	6.384333	127.4829	6.38695	126.3394	6.366683	113.7184	6.366683	117.2563
6.401233	123.7627	6.417867	128.8061	6.401	127.4628	6.403617	126.3227	6.38335	113.6978	6.38335	117.2431
6.4179	123.747	6.434533	128.7886	6.417667	127.4435	6.420283	126.3047	6.400017	113.681	6.400017	117.228
6.434567	123.73	6.4512	128.7653	6.434333	127.4264	6.43695	126.2894	6.416683	113.6631	6.416683	117.2135
6.451233	123.7133	6.467867	128.7541	6.451	127.4094	6.453617	126.2718	6.43335	113.6456	6.43335	117.2015
6.4679	123.7026	6.484533	128.7365	6.467667	127.3937	6.470283	126.2531	6.450017	113.6237	6.450017	117.1869
6.484567	123.6787	6.5012	128.7203	6.484333	127.377	6.48695	126.2362	6.466683	113.6117	6.466683	117.1748
6.501233	123.6661	6.517867	128.7024	6.501	127.3589	6.503617	126.217	6.48335	113.5917	6.48335	117.162
6.5179	123.6475	6.534533	128.6872	6.517667	127.3397	6.520283	126.2038	6.500017	113.5735	6.500017	117.1471
6.534567	123.6275	6.5512	128.6669	6.534333	127.3138	6.53695	126.1844	6.516683	113.5535	6.516683	117.1315

6.551233	123.6129	6.567867	128.6542	6.551	127.3098	6.553617	126.167	6.53335	113.538	6.53335	117.1196
6.5679	123.5983	6.584533	128.6386	6.567667	127.2806	6.570283	126.1506	6.550017	113.5205	6.550017	117.1088
6.584567	123.5798	6.6012	128.6239	6.584333	127.2656	6.58695	126.1337	6.566683	113.5027	6.566683	117.0933
6.601233	123.5675	6.617867	128.607	6.601	127.2538	6.603617	126.1169	6.58335	113.4849	6.58335	117.0806
6.6179	123.5487	6.634533	128.5935	6.617667	127.2328	6.620283	126.0978	6.600017	113.4678	6.600017	117.0687
6.634567	123.5336	6.6512	128.575	6.634333	127.2184	6.63695	126.0822	6.616683	113.4504	6.616683	117.0532
6.651233	123.5169	6.667867	128.5603	6.651	127.2008	6.653617	126.0648	6.63335	113.4377	6.63335	117.0417
6.6679	123.5054	6.684533	128.5437	6.667667	127.1792	6.670283	126.0465	6.650017	113.4177	6.650017	117.0293
6.684567	123.4863	6.7012	128.5274	6.684333	127.1657	6.68695	126.0309	6.666683	113.4002	6.666683	117.0138
6.701233	123.4713	6.717867	128.5099	6.701	127.1483	6.703617	126.0147	6.68335	113.3824	6.68335	117.0005
6.7179	123.4577	6.734533	128.494	6.717667	127.1311	6.720283	125.9989	6.700017	113.3654	6.700017	116.9877
6.734567	123.4417	6.7512	128.4793	6.734333	127.1125	6.73695	125.9839	6.716683	113.3486	6.716683	116.9744
6.751233	123.4257	6.767867	128.4646	6.751	127.0959	6.753617	125.9648	6.73335	113.3326	6.73335	116.9603
6.7679	123.4083	6.784533	128.4505	6.767667	127.0793	6.770283	125.949	6.750017	113.3144	6.750017	116.9488
6.784567	123.3937	6.8012	128.432	6.784333	127.0639	6.78695	125.932	6.766683	113.2965	6.766683	116.9348
6.801233	123.3766	6.817867	128.4163	6.801	127.0445	6.803617	125.9157	6.78335	113.285	6.78335	116.9185
6.8179	123.3594	6.834533	128.4062	6.817667	127.0283	6.820283	125.8994	6.800017	113.2637	6.800017	116.9079
6.834567	123.3487	6.8512	128.3845	6.834333	127.0128	6.83695	125.8847	6.816683	113.2513	6.816683	116.8964
6.851233	123.3312	6.867867	128.3653	6.851	126.9962	6.853617	125.8647	6.83335	113.233	6.83335	116.882
6.8679	123.3158	6.884533	128.3525	6.867667	126.9774	6.870283	125.8491	6.850017	113.2174	6.850017	116.8683
6.884567	123.2956	6.9012	128.3361	6.884333	126.9659	6.88695	125.8364	6.866683	113.2042	6.866683	116.857
6.901233	123.2792	6.917867	128.3232	6.901	126.9469	6.903617	125.819	6.88335	113.1855	6.88335	116.8449
6.9179	123.2591	6.934533	128.307	6.917667	126.9318	6.920283	125.8049	6.900017	113.1707	6.900017	116.8323
6.934567	123.2412	6.9512	128.2937	6.934333	126.9161	6.93695	125.7867	6.916683	113.1531	6.916683	116.8214
6.951233	123.2249	6.967867	128.2785	6.951	126.8972	6.953617	125.7714	6.93335	113.138	6.93335	116.8102
6.9679	123.209	6.984533	128.264	6.967667	126.8818	6.970283	125.7555	6.950017	113.1223	6.950017	116.798
6.984567	123.1898	7.0012	128.2489	6.984333	126.8638	6.98695	125.7401	6.966683	113.1057	6.966683	116.7853
7.001233	123.1758	7.017867	128.234	7.001	126.8481	7.003617	125.7239	6.98335	113.0896	6.98335	116.7706
7.0179	123.1578	7.034533	128.2182	7.017667	126.8312	7.020283	125.71	7.000017	113.0729	7.000017	116.7602
7.034567	123.1421	7.0512	128.2036	7.034333	126.814	7.03695	125.6913	7.016683	113.0542	7.016683	116.7473
7.051233	123.1247	7.067867	128.1882	7.051	126.7996	7.053617	125.6762	7.03335	113.0407	7.03335	116.7373
7.0679	123.1121	7.084533	128.1739	7.067667	126.7815	7.070283	125.6654	7.050017	113.0274	7.050017	116.7222
7.084567	123.0955	7.1012	128.1591	7.084333	126.7669	7.08695	125.6478	7.066683	113.0091	7.066683	116.7092
7.101233	123.0793	7.117867	128.1419	7.101	126.7497	7.103617	125.6294	7.08335	112.9935	7.08335	116.6947
7.1179	123.0642	7.134533	128.1242	7.117667	126.7325	7.120283	125.6152	7.100017	112.9776	7.100017	116.6823
7.134567	123.0493	7.1512	128.1132	7.134333	126.7187	7.13695	125.6004	7.116683	112.9606	7.116683	116.6727
7.151233	123.033	7.167867	128.0965	7.151	126.7004	7.153617	125.585	7.13335	112.9445	7.13335	116.6596
7.1679	123.0181	7.184533	128.0768	7.167667	126.684	7.170283	125.5677	7.150017	112.9289	7.150017	116.6498
7.184567	123.0015	7.2012	128.0636	7.184333	126.6688	7.18695	125.5527	7.166683	112.9094	7.166683	116.6341
7.201233	122.988	7.217867	128.0473	7.201	126.6534	7.203617	125.5361	7.18335	112.8947	7.18335	116.6204
7.2179	122.9733	7.234533	128.035	7.217667	126.6364	7.220283	125.5206	7.200017	112.8796	7.200017	116.6088
7.234567	122.9591	7.2512	128.0187	7.234333	126.6199	7.23695	125.5069	7.216683	112.865	7.216683	116.5992
7.251233	122.9452	7.267867	128.006	7.251	126.6037	7.253617	125.4949	7.23335	112.8459	7.23335	116.5852
7.2679	122.9299	7.284533	127.9895	7.267667	126.586	7.270283	125.4735	7.250017	112.8359	7.250017	116.5722
7.284567	122.9113	7.3012	127.9786	7.284333	126.5707	7.28695	125.4624	7.266683	112.8174	7.266683	116.5601
7.301233	122.8975	7.317867	127.9621	7.301	126.5583	7.303617	125.4464	7.28335	112.8032	7.28335	116.547
7.3179	122.8838	7.334533	127.9471	7.317667	126.5409	7.320283	125.4292	7.300017	112.7884	7.300017	116.5386
7.334567	122.8686	7.3512	127.9334	7.334333	126.5263	7.33695	125.4151	7.316683	112.7735	7.316683	116.5231
7.351233	122.8512	7.367867	127.919	7.351	126.508	7.353617	125.4013	7.33335	112.7579	7.33335	116.514
7.3679	122.8384	7.384533	127.9045	7.367667	126.4861	7.370283	125.3822	7.350017	112.7415	7.350017	116.5008
7.384567	122.8246	7.4012	127.889	7.384333	126.473	7.38695	125.367	7.366683	112.7272	7.366683	116.4876
7.401233	122.8094	7.417867	127.8731	7.401	126.4602	7.403617	125.3527	7.38335	112.7096	7.38335	116.4764
7.4179	122.7931	7.434533	127.8564	7.417667	126.438	7.420283	125.3374	7.400017	112.693	7.400017	116.4662
7.434567	122.78	7.4512	127.8451	7.434333	126.4242	7.43695	125.3235	7.416683	112.6831	7.416683	116.4535
7.451233	122.7671	7.467867	127.828	7.451	126.4063	7.453617	125.3079	7.43335	112.6678	7.43335	116.4401
7.4679	122.7512	7.484533	127.8133	7.467667	126.3901	7.470283	125.2951	7.450017	112.6517	7.450017	116.4292
7.484567	122.7368	7.5012	127.8039	7.484333	126.3749	7.48695	125.2774	7.466683	112.6387	7.466683	116.4169
7.501233	122.7219	7.517867	127.7885	7.501	126.355	7.503617	125.2638	7.48335	112.6227	7.48335	116.4043
7.5179	122.7116	7.534533	127.7736	7.517667	126.3426	7.520283	125.2508	7.500017	112.6046	7.500017	116.3925
7.534567	122.6967	7.5512	127.7602	7.534333	126.3248	7.53695	125.2363	7.516683	112.5903	7.516683	116.3812
7.551233	122.6871	7.567867	127.7455	7.551	126.3095	7.553617	125.2202	7.53335	112.5753	7.53335	116.367
7.5679	122.6661	7.584533	127.7315	7.567667	126.2902	7.570283	125.2059	7.550017	112.5587	7.550017	116.3567
7.584567	122.6515	7.6012	127.7151	7.584333	126.2774	7.58695	125.1917	7.566683	112.5431	7.566683	116.3467
7.601233	122.6334	7.617867	127.7023	7.601	126.2622	7.603617	125.1758	7.58335	112.5267	7.58335	116.3322
7.6179	122.6262	7.634533	127.6886	7.617667	126.2456	7.620283	125.1599	7.600017	112.5142	7.600017	116.3206
7.634567	122.6128	7.6512	127.6751	7.634333	126.2301	7.63695	125.1455	7.616683	112.4999	7.616683	116.3108
7.651233	122.5955	7.667867	127.661	7.651	126.2142	7.653617	125.1325	7.63335	112.4839	7.63335	116.2989
7.6679	122.5845	7.684533	127.6467	7.667667	126.1992	7.670283	125.1153	7.650017	112.4713	7.650017	116.2887
7.684567	122.5669	7.7012	127.6339	7.684333	126.1833	7.68695	125.102	7.666683	112.4536	7.666683	116.2781
7.701233	122.5558	7.717867	127.6169	7.701	126.167	7.703617	125.088	7.68335	112.4379	7.68335	116.2632
7.7179	122.5399	7.734533	127.6026	7.717667	126.1543	7.720283	125.0756	7.700017	112.4237	7.700017	116.2509
7.734567	122.5294	7.7512	127.5901	7.734333	126.1345	7.73695	125.0589	7.716683	112.4098	7.716683	116.2422
7.751233	122.5165	7.767867	127.5764	7.751	126.1202	7.753617	125.0452	7.73335	112.3916	7.73335	116.2315
7.7679	122.5026	7.784533	127.5604	7.767667	126.1048	7.770283	125.0304	7.750017	112.377	7.750017	116.2177
7.784567	122.4913	7.8012	127.5472	7.784333	126.0912	7.78695	125.0158	7.766683	112.3652	7.766683	116.2097
7.801233	122.4756	7.817867	127.5342	7.801	126.0779	7.803617	125.0016	7.78335	112.3518	7.78335	116.1968
7.8179	122.4602	7.834533	127.5219	7.817667	126.0585	7.820283	124.9897	7.800017	112.3384	7.800017	116.1835
7.834567	122.4445	7.8512	127.5072	7.834333	126.0439	7.83695	124.9728	7.816683	112.3217	7.816683	116.1734
7.851233	122.4321	7.867867	127.4939	7.851	126.0258	7.853617	124.9579	7.83335	112.3101	7.83335	116.1586

7.8679	122.4246	7.884533	127.4779	7.867667	126.0053	7.870283	124.9428	7.850017	112.2942	7.850017	116.149
7.884567	122.4099	7.9012	127.4652	7.884333	125.9882	7.88695	124.9299	7.866683	112.2801	7.866683	116.1355
7.901233	122.3924	7.917867	127.4502	7.901	125.9716	7.903617	124.9172	7.88335	112.2652	7.88335	116.1262
7.9179	122.3785	7.934533	127.4365	7.917667	125.9558	7.920283	124.9029	7.900017	112.2538	7.900017	116.1155
7.934567	122.3667	7.9512	127.4271	7.934333	125.9417	7.93695	124.8898	7.916683	112.2407	7.916683	116.1032
7.951233	122.3518	7.967867	127.4114	7.951	125.9249	7.953617	124.8714	7.93335	112.2239	7.93335	116.0901
7.9679	122.3372	7.984533	127.3985	7.967667	125.9055	7.970283	124.8618	7.950017	112.2126	7.950017	116.0812
7.984567	122.3263	8.0012	127.3801	7.984333	125.8894	7.98695	124.8462	7.966683	112.1973	7.966683	116.0702
8.001233	122.3121	8.017867	127.3683	8.001	125.8742	8.003617	124.8324	7.98335	112.1815	7.98335	116.0586
8.0179	122.3005	8.034533	127.3563	8.017667	125.857	8.020283	124.8221	8.000017	112.1658	8.000017	116.0478
8.034567	122.2795	8.0512	127.3417	8.034333	125.8419	8.03695	124.8068	8.016683	112.1533	8.016683	116.0368
8.051233	122.2701	8.067867	127.3301	8.051	125.8268	8.053617	124.7917	8.03335	112.1367	8.03335	116.0254
8.0679	122.2586	8.084533	127.3172	8.067667	125.8114	8.070283	124.7783	8.050017	112.1259	8.050017	116.0151
8.084567	122.2405	8.1012	127.3	8.084333	125.7973	8.08695	124.7649	8.066683	112.1117	8.066683	116.0014
8.101233	122.2283	8.117867	127.2866	8.101	125.7836	8.103617	124.7504	8.08335	112.0976	8.08335	115.9887
8.1179	122.2142	8.134533	127.2735	8.117667	125.767	8.120283	124.7381	8.100017	112.0861	8.100017	115.9785
8.134567	122.2008	8.1512	127.2625	8.134333	125.7542	8.13695	124.7243	8.116683	112.069	8.116683	115.9669
8.151233	122.1848	8.167867	127.2458	8.151	125.7392	8.153617	124.7096	8.13335	112.0587	8.13335	115.9557
8.1679	122.1747	8.184533	127.2362	8.167667	125.7224	8.170283	124.6972	8.150017	112.0449	8.150017	115.9449
8.184567	122.1608	8.2012	127.2215	8.184333	125.7086	8.18695	124.6842	8.166683	112.0315	8.166683	115.9359
8.201233	122.1466	8.217867	127.2067	8.201	125.6954	8.203617	124.67	8.18335	112.0159	8.18335	115.9244
8.2179	122.1332	8.234533	127.1955	8.217667	125.6834	8.220283	124.6551	8.200017	112.0022	8.200017	115.9119
8.234567	122.1186	8.2512	127.1824	8.234333	125.6685	8.23695	124.6431	8.216683	111.9905	8.216683	115.9013
8.251233	122.1085	8.267867	127.1676	8.251	125.6551	8.253617	124.6297	8.23335	111.9765	8.23335	115.8898
8.2679	122.0895	8.284533	127.1535	8.267667	125.641	8.270283	124.6141	8.250017	111.9631	8.250017	115.8801
8.284567	122.0738	8.3012	127.1429	8.284333	125.626	8.28695	124.6016	8.266683	111.9489	8.266683	115.8675
8.301233	122.0626	8.317867	127.128	8.301	125.6127	8.303617	124.5894	8.28335	111.9328	8.28335	115.8573
8.3179	122.0505	8.334533	127.1149	8.317667	125.5975	8.320283	124.5751	8.300017	111.9243	8.300017	115.8491
8.334567	122.0366	8.3512	127.1002	8.334333	125.5844	8.33695	124.5649	8.316683	111.9075	8.316683	115.8379
8.351233	122.0243	8.367867	127.0859	8.351	125.5713	8.353617	124.552	8.33335	111.8955	8.33335	115.8261
8.3679	122.0097	8.384533	127.0749	8.367667	125.5556	8.370283	124.5372	8.350017	111.8808	8.350017	115.8154
8.384567	121.9987	8.4012	127.0665	8.384333	125.5471	8.38695	124.5242	8.366683	111.8692	8.366683	115.8056
8.401233	121.9854	8.417867	127.0503	8.401	125.5296	8.403617	124.512	8.38335	111.8523	8.38335	115.7953
8.4179	121.9733	8.434533	127.0382	8.417667	125.5154	8.420283	124.5006	8.400017	111.8403	8.400017	115.7822
8.434567	121.959	8.4512	127.0218	8.434333	125.5021	8.43695	124.4876	8.416683	111.8246	8.416683	115.7723
8.451233	121.9463	8.467867	127.0095	8.451	125.4916	8.453617	124.4738	8.43335	111.8119	8.43335	115.7632
8.4679	121.9299	8.484533	126.9998	8.467667	125.4772	8.470283	124.4618	8.450017	111.7982	8.450017	115.7524
8.484567	121.9164	8.5012	126.9869	8.484333	125.4623	8.48695	124.448	8.466683	111.7841	8.466683	115.7404
8.501233	121.9055	8.517867	126.9746	8.501	125.4488	8.503617	124.4336	8.48335	111.7718	8.48335	115.7295
8.5179	121.8912	8.534533	126.9597	8.517667	125.4356	8.520283	124.4217	8.500017	111.7598	8.500017	115.7184
8.534567	121.8784	8.5512	126.9505	8.534333	125.4241	8.53695	124.407	8.516683	111.7461	8.516683	115.7076
8.551233	121.8677	8.567867	126.9366	8.551	125.4108	8.553617	124.3955	8.53335	111.7329	8.53335	115.697
8.5679	121.8562	8.584533	126.924	8.567667	125.3964	8.570283	124.3816	8.550017	111.7176	8.550017	115.689
8.584567	121.8399	8.6012	126.9101	8.584333	125.3823	8.58695	124.3718	8.566683	111.7041	8.566683	115.6789
8.601233	121.829	8.617867	126.8966	8.601	125.3676	8.603617	124.3589	8.58335	111.6915	8.58335	115.6711
8.6179	121.8197	8.634533	126.881	8.617667	125.355	8.620283	124.3418	8.600017	111.6786	8.600017	115.6592
8.634567	121.8067	8.6512	126.8695	8.634333	125.3439	8.63695	124.3293	8.616683	111.6626	8.616683	115.6491
8.651233	121.7927	8.667867	126.8578	8.651	125.3289	8.653617	124.317	8.63335	111.6515	8.63335	115.6416
8.6679	121.7818	8.684533	126.8446	8.667667	125.3178	8.670283	124.3034	8.650017	111.6401	8.650017	115.6303
8.684567	121.7667	8.7012	126.834	8.684333	125.3011	8.68695	124.2921	8.666683	111.6244	8.666683	115.6195
8.701233	121.7544	8.717867	126.8212	8.701	125.2863	8.703617	124.2805	8.68335	111.6111	8.68335	115.6086
8.7179	121.7377	8.734533	126.8082	8.717667	125.2746	8.720283	124.2657	8.700017	111.6002	8.700017	115.6004
8.734567	121.7268	8.7512	126.7998	8.734333	125.2625	8.73695	124.2521	8.716683	111.5849	8.716683	115.5894
8.751233	121.7166	8.767867	126.7875	8.751	125.2456	8.753617	124.2411	8.73335	111.5728	8.73335	115.5783
8.7679	121.7042	8.784533	126.7753	8.767667	125.2364	8.770283	124.2265	8.750017	111.5569	8.750017	115.5681
8.784567	121.6897	8.8012	126.7598	8.784333	125.22	8.78695	124.214	8.766683	111.5458	8.766683	115.5582
8.801233	121.6784	8.817867	126.7487	8.801	125.2111	8.803617	124.2026	8.78335	111.5328	8.78335	115.5491
8.8179	121.6676	8.834533	126.7366	8.817667	125.1985	8.820283	124.1908	8.800017	111.5232	8.800017	115.5391
8.834567	121.6524	8.8512	126.7232	8.834333	125.1907	8.83695	124.1771	8.816683	111.509	8.816683	115.5285
8.851233	121.6396	8.867867	126.7133	8.851	125.175	8.853617	124.1651	8.83335	111.4928	8.83335	115.5156
8.8679	121.6261	8.884533	126.6977	8.867667	125.1636	8.870283	124.1516	8.850017	111.482	8.850017	115.5086
8.884567	121.6117	8.9012	126.688	8.884333	125.1509	8.88695	124.1366	8.866683	111.4681	8.866683	115.4976
8.901233	121.603	8.917867	126.675	8.901	125.137	8.903617	124.1291	8.88335	111.4546	8.88335	115.4873
8.9179	121.5894	8.934533	126.66	8.917667	125.122	8.920283	124.1141	8.900017	111.4416	8.900017	115.4783
8.934567	121.5796	8.9512	126.6524	8.934333	125.1098	8.93695	124.1006	8.916683	111.4327	8.916683	115.4667
8.951233	121.5634	8.967867	126.6387	8.951	125.0942	8.953617	124.0917	8.93335	111.4187	8.93335	115.4581
8.9679	121.5499	8.984533	126.6243	8.967667	125.0819	8.970283	124.0751	8.950017	111.4065	8.950017	115.4494
8.984567	121.5416	9.0012	126.614	8.984333	125.0754	8.98695	124.0636	8.966683	111.3936	8.966683	115.4409
9.001233	121.5298	9.017867	126.6	9.001	125.0621	9.003617	124.0509	8.98335	111.3803	8.98335	115.4292
9.0179	121.5148	9.034533	126.5871	9.017667	125.0451	9.020283	124.0401	9.000017	111.3663	9.000017	115.4196
9.034567	121.5036	9.0512	126.5751	9.034333	125.0376	9.03695	124.0262	9.016683	111.3588	9.016683	115.41
9.051233	121.4891	9.067867	126.5655	9.051	125.0215	9.053617	124.014	9.03335	111.3411	9.03335	115.3982
9.0679	121.4765	9.084533	126.5511	9.067667	125.0152	9.070283	124.006	9.050017	111.3262	9.050017	115.3881
9.084567	121.4644	9.1012	126.5385	9.084333	124.9985	9.08695	123.9895	9.066683	111.3174	9.066683	115.3801
9.101233	121.4493	9.117867	126.5267	9.101	124.9845	9.103617	123.9783	9.08335	111.304	9.08335	115.3705
9.1179	121.4357	9.134533	126.5155	9.117667	124.9775	9.120283	123.9622	9.100017	111.2921	9.100017	115.3608
9.134567	121.4228	9.1512	126.5039	9.134333	124.9622	9.13695	123.9514	9.116683	111.279	9.116683	115.3487
9.151233	121.4091	9.167867	126.4936	9.151	124.9522	9.153617	123.9401	9.13335	111.2705	9.13335	115.3414
9.1679	121.3958	9.184533	126.4769	9.167667	124.941	9.170283	123.9261	9.150017	111.2555	9.150017	115.3312

9.184567	121.3864	9.2012	126.4669	9.184333	124.9265	9.18695	123.9133	9.166683	111.2434	9.166683	115.3175
9.201233	121.3719	9.217867	126.4553	9.201	124.9167	9.203617	123.9021	9.18335	111.2321	9.18335	115.3118
9.2179	121.3548	9.234533	126.4413	9.217667	124.9046	9.220283	123.8906	9.200017	111.2206	9.200017	115.301
9.234567	121.3466	9.2512	126.4336	9.234333	124.8916	9.23695	123.876	9.216683	111.2078	9.216683	115.2881
9.251233	121.3332	9.267867	126.4178	9.251	124.8805	9.253617	123.864	9.23335	111.1961	9.23335	115.2794
9.2679	121.3188	9.284533	126.4073	9.267667	124.8648	9.270283	123.8561	9.250017	111.1827	9.250017	115.2673
9.284567	121.3069	9.3012	126.3971	9.284333	124.8538	9.28695	123.8439	9.266683	111.1702	9.266683	115.261
9.301233	121.2958	9.317867	126.3828	9.301	124.8441	9.303617	123.8326	9.28335	111.1581	9.28335	115.2526
9.3179	121.2828	9.334533	126.3725	9.317667	124.8276	9.320283	123.819	9.300017	111.1442	9.300017	115.2385
9.334567	121.268	9.3512	126.3606	9.334333	124.8199	9.33695	123.8092	9.316683	111.1311	9.316683	115.2311
9.351233	121.2555	9.367867	126.3514	9.351	124.806	9.353617	123.7968	9.33335	111.1215	9.33335	115.2237
9.3679	121.2452	9.384533	126.3373	9.367667	124.7947	9.370283	123.7834	9.350017	111.1101	9.350017	115.2138
9.384567	121.2342	9.4012	126.3279	9.384333	124.7834	9.38695	123.7731	9.366683	111.0989	9.366683	115.2006
9.401233	121.218	9.417867	126.3163	9.401	124.7705	9.403617	123.759	9.38335	111.0857	9.38335	115.1918
9.4179	121.2089	9.434533	126.3017	9.417667	124.7585	9.420283	123.7477	9.400017	111.0747	9.400017	115.1822
9.434567	121.1935	9.4512	126.2931	9.434333	124.7472	9.43695	123.7349	9.416683	111.0635	9.416683	115.1731
9.451233	121.1827	9.467867	126.283	9.451	124.7365	9.453617	123.7245	9.43335	111.0496	9.43335	115.1657
9.4679	121.1698	9.484533	126.2684	9.467667	124.7252	9.470283	123.7113	9.450017	111.0392	9.450017	115.1543
9.484567	121.1612	9.5012	126.2571	9.484333	124.7123	9.48695	123.6997	9.466683	111.0262	9.466683	115.1445
9.501233	121.1482	9.517867	126.2476	9.501	124.6996	9.503617	123.6873	9.48335	111.013	9.48335	115.1335
9.5179	121.1365	9.534533	126.2361	9.517667	124.6865	9.520283	123.6746	9.500017	111.001	9.500017	115.1274
9.534567	121.1229	9.5512	126.2251	9.534333	124.6771	9.53695	123.6643	9.516683	110.9888	9.516683	115.1174
9.551233	121.1125	9.567867	126.2159	9.551	124.6676	9.553617	123.6492	9.53335	110.9759	9.53335	115.1094
9.5679	121.1017	9.584533	126.2023	9.567667	124.6535	9.570283	123.6375	9.550017	110.9634	9.550017	115.0969
9.584567	121.09	9.6012	126.1906	9.584333	124.6387	9.58695	123.6267	9.566683	110.9534	9.566683	115.0869
9.601233	121.0782	9.617867	126.1787	9.601	124.6299	9.603617	123.6147	9.58335	110.9428	9.58335	115.0799
9.6179	121.0663	9.634533	126.1692	9.617667	124.619	9.620283	123.6042	9.600017	110.9291	9.600017	115.07
9.634567	121.0469	9.6512	126.1558	9.634333	124.604	9.63695	123.5946	9.616683	110.9181	9.616683	115.0603
9.651233	121.032	9.667867	126.1465	9.651	124.5941	9.653617	123.5813	9.63335	110.9118	9.63335	115.0535
9.6679	121.0224	9.684533	126.1329	9.667667	124.5807	9.670283	123.5697	9.650017	110.8977	9.650017	115.044
9.684567	121.0087	9.7012	126.1218	9.684333	124.5675	9.68695	123.5577	9.666683	110.8861	9.666683	115.0346
9.701233	120.9995	9.717867	126.1088	9.701	124.5569	9.703617	123.5459	9.68335	110.8763	9.68335	115.0243
9.7179	120.9894	9.734533	126.1005	9.717667	124.5456	9.720283	123.5348	9.700017	110.8623	9.700017	115.0162
9.734567	120.9776	9.7512	126.0888	9.734333	124.5332	9.73695	123.522	9.716683	110.8559	9.716683	115.007
9.751233	120.9654	9.767867	126.0796	9.751	124.5224	9.753617	123.5137	9.73335	110.8415	9.73335	114.9975
9.7679	120.9545	9.784533	126.0679	9.767667	124.5121	9.770283	123.5021	9.750017	110.8333	9.750017	114.9885
9.784567	120.9434	9.8012	126.0555	9.784333	124.501	9.78695	123.4892	9.766683	110.824	9.766683	114.98
9.801233	120.9328	9.817867	126.0441	9.801	124.4905	9.803617	123.4784	9.78335	110.8068	9.78335	114.9717
9.8179	120.9195	9.834533	126.0329	9.817667	124.4796	9.820283	123.4691	9.800017	110.798	9.800017	114.9629
9.834567	120.9083	9.8512	126.0217	9.834333	124.47	9.83695	123.4546	9.816683	110.783	9.816683	114.9553
9.851233	120.9003	9.867867	126.0108	9.851	124.4569	9.853617	123.4441	9.83335	110.7736	9.83335	114.9457
9.8679	120.8862	9.884533	126.0013	9.867667	124.4458	9.870283	123.4298	9.850017	110.762	9.850017	114.9351
9.884567	120.8762	9.9012	125.9911	9.884333	124.4339	9.88695	123.421	9.866683	110.7524	9.866683	114.9264
9.901233	120.8656	9.917867	125.9806	9.901	124.4213	9.903617	123.4095	9.88335	110.7382	9.88335	114.9168
9.9179	120.8534	9.934533	125.97	9.917667	124.4101	9.920283	123.4	9.900017	110.7302	9.900017	114.9054
9.934567	120.8439	9.9512	125.9577	9.934333	124.3983	9.93695	123.3878	9.916683	110.7189	9.916683	114.896
9.951233	120.8338	9.967867	125.9484	9.951	124.3866	9.953617	123.3778	9.93335	110.7063	9.93335	114.8865
9.9679	120.8235	9.984533	125.9385	9.967667	124.3748	9.970283	123.3629	9.950017	110.6955	9.950017	114.8771
9.984567	120.8102	10.0012	125.9275	9.984333	124.3638	9.98695	123.3561	9.966683	110.6819	9.966683	114.8697
10.00123	120.8	10.01787	125.9135	10.001	124.3514	10.00362	123.344	9.98335	110.671	9.98335	114.8599
10.0179	120.7872	10.03453	125.903	10.01767	124.3407	10.02028	123.3334	10.00002	110.6588	10.00002	114.85
10.03457	120.7746	10.0512	125.8943	10.03433	124.3286	10.03695	123.3239	10.01668	110.6483	10.01668	114.8432
10.05123	120.7634	10.06787	125.8798	10.051	124.3175	10.05362	123.3103	10.03335	110.6417	10.03335	114.8336
10.0679	120.7549	10.08453	125.872	10.06767	124.3063	10.07028	123.3014	10.05002	110.6279	10.05002	114.8229
10.08457	120.7437	10.1012	125.8608	10.08433	124.2938	10.08695	123.2902	10.06668	110.6186	10.06668	114.8118
10.10123	120.7321	10.11787	125.8484	10.101	124.2847	10.10362	123.2763	10.08335	110.6054	10.08335	114.805
10.1179	120.7199	10.13453	125.8388	10.11767	124.2712	10.12028	123.268	10.10002	110.5962	10.10002	114.7985
10.13457	120.7084	10.1512	125.8291	10.13433	124.2603	10.13695	123.2548	10.11668	110.5837	10.11668	114.7867
10.15123	120.6996	10.16787	125.8182	10.151	124.2503	10.15362	123.2472	10.13335	110.577	10.13335	114.7803
10.1679	120.6867	10.18453	125.808	10.16767	124.2378	10.17028	123.2345	10.15002	110.5628	10.15002	114.7691
10.18457	120.6778	10.2012	125.7985	10.18433	124.2282	10.18695	123.2225	10.16668	110.5517	10.16668	114.7609
10.20123	120.6642	10.21787	125.7837	10.201	124.217	10.20362	123.2135	10.18335	110.5389	10.18335	114.7555
10.2179	120.6535	10.23453	125.7753	10.21767	124.2039	10.22028	123.2023	10.20002	110.532	10.20002	114.747
10.23457	120.6402	10.2512	125.7652	10.23433	124.198	10.23695	123.1902	10.21668	110.5183	10.21668	114.7376
10.25123	120.6325	10.26787	125.7541	10.251	124.1832	10.25362	123.1807	10.23335	110.5089	10.23335	114.7282
10.2679	120.6222	10.28453	125.7459	10.26767	124.1742	10.27028	123.1662	10.25002	110.4956	10.25002	114.7231
10.28457	120.6081	10.3012	125.7323	10.28433	124.1648	10.28695	123.1578	10.26668	110.4844	10.26668	114.7125
10.30123	120.5974	10.31787	125.7218	10.301	124.152	10.30362	123.1461	10.28335	110.4737	10.28335	114.7025
10.3179	120.588	10.33453	125.7129	10.31767	124.1413	10.32028	123.1352	10.30002	110.4589	10.30002	114.6942
10.33457	120.5766	10.3512	125.7026	10.33433	124.1291	10.33695	123.1246	10.31668	110.4517	10.31668	114.6856
10.35123	120.5682	10.36787	125.6919	10.351	124.1205	10.35362	123.1129	10.33335	110.4382	10.33335	114.6762
10.3679	120.5544	10.38453	125.6824	10.36767	124.11	10.37028	123.1021	10.35002	110.428	10.35002	114.6646
10.38457	120.5452	10.4012	125.6683	10.38433	124.0977	10.38695	123.0917	10.36668	110.4171	10.36668	114.6584
10.40123	120.5336	10.41787	125.6568	10.401	124.087	10.40362	123.0828	10.38335	110.4075	10.38335	114.6485
10.4179	120.5242	10.43453	125.646	10.41767	124.078	10.42028	123.0716	10.40002	110.3959	10.40002	114.638
10.43457	120.5125	10.4512	125.637	10.43433	124.0677	10.43695	123.0586	10.41668	110.3857	10.41668	114.6306
10.45123	120.5027	10.46787	125.6257	10.451	124.0566	10.45362	123.0475	10.43335	110.3788	10.43335	114.6218
10.4679	120.4921	10.48453	125.6176	10.46767	124.0463	10.47028	123.0352	10.45002	110.3681	10.45002	114.6134
10.48457	120.4807	10.5012	125.6062	10.48433	124.0324	10.48695	123.0279	10.46668	110.3584	10.46668	114.6045

10.50123	120.4701	10.51787	125.5949	10.501	124.0232	10.50362	123.0162	10.48335	110.3442	10.48335	114.5965
10.5179	120.4617	10.53453	125.5853	10.51767	124.0129	10.52028	123.0047	10.50002	110.3365	10.50002	114.5881
[truncated: 2,777,527 more chars]
